# NHC-Ni catalyzed 1,3- and 1,4-diastereodivergent heterocycle synthesis from hetero-substituted enyne

**DOI:** 10.1038/s42004-020-0299-9

**Published:** 2020-04-30

**Authors:** Xuefeng Yong, Weiwei Gao, Xiulian Lin, Chun-Yu Ho

**Affiliations:** 1 School of Chemistry and Chemical Engineering, Harbin Institute of Technology, Harbin, (150001) Heilongjiang China; 2 Shenzhen Grubbs Institute, Guangdong Provincial Key Laboratory of Catalysis, Department of Chemistry, Southern University of Science and Technology (SUSTech), Shenzhen, (518055) Guangdong China

**Keywords:** Asymmetric catalysis, Synthetic chemistry methodology

## Abstract

Diastereodivergent heterocycle synthesis has been recognized as an important tool for drug discovery in recent years, yet strategies based on nickelacycle formation have not been established. Here, we report a NHC-Ni catalyzed highly 1,3- and 1,4-diastereodivergent heterocycle synthesis from enyne, which is achieved by manipulating the enyne N-substituent (allowing switching of selectivity from up to 2:98 to 98:2). The key to success is the efficient diastereodivergent formation of a nickelacyclopentene, with broad enyne scope at mild conditions, which subsequently provides reductive hydroalkenylation, acylation and silylation products on demand. Diastereoisomers which are sterically hard to distinguish or difficult to access by conventional routes are now accessible easily, including those with very similar 4°, contiguous and skipped stereocenters.

## Abstract (web summary)

Diastereoselective cyclisation of heteroatom-substituted enynes offers a valuable route to bioactive heterocycles. Here, a diastereodivergent nickel-catalysed cyclisation of heteroenynes offers access to diastereomers of 1,3- and 1,4-hydroalkenylation products, controlled by the choice of substituents.

## Introduction

**Fig. 1:**
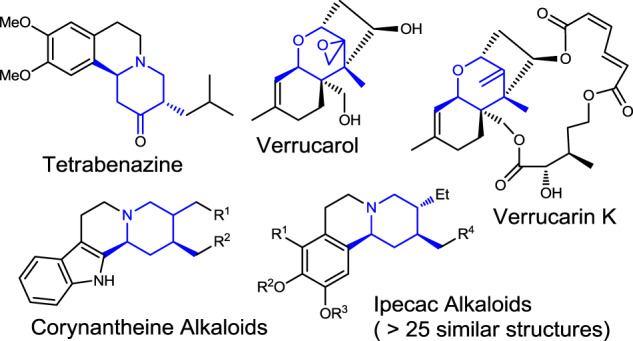
Bioactive targets. Examples of bioactive multi-substituted heterocycles involved exocyclic olefin precursors.

**Fig. 2:**
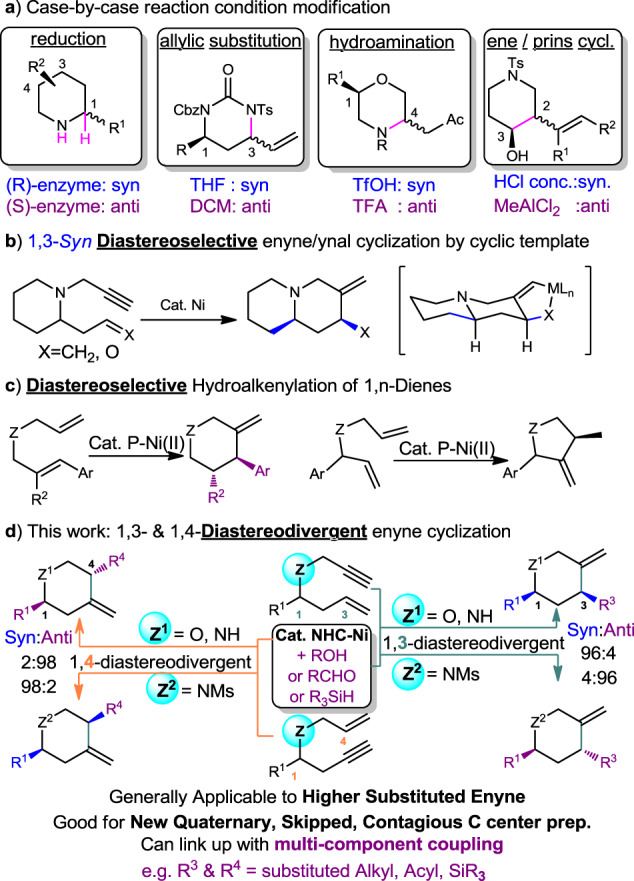
Strategy for catalytic diastereo-divergent/-selective heterocycle synthesis from π-systems. **a** By condition modification. **b** By cyclic template. **c** By steric substituents. **d** By hetero-substituted enynes.

Heterocycles bearing multiple stereocenters are common structural motifs in many biologically active molecules^1^. Given that different diastereomers may exhibit diverse bioactivities, synthesis of individual diastereomers is of great importance. Multi-substituted hydropyran and piperidine skeletons with exocyclic olefin are key core structures or precursors in many biologically active natural products and drug molecules (Fig. 1)^2–7^. Over the years, various innovative strategies and elegant synthetic methods have been developed for (dia)stereodivergent heterocycle synthesis^8–13^. Substituents in 1,2-, 1,3- or 1,4-diastereodivergent relationships could be built by tailor-made ring closure methods efficiently, which are controlled often by selective facial attack either on prochiral sp^2^ center or on radical acceptors (Fig. 2a)^14,15^. Some were controlled efficiently by solvent assistance and steric repulsion among substituent Rs on ring closure, or by redesign of reaction pathways or catalysts^16–19^. Yet, some are limited severely by the choice of nucleophiles and polarized tether structures. Undesired steric repulsion among substituents (mismatch) as well as the high demands of functional groups often limited the scope and the efficiency of the process.

Transition metal-mediated metallacyclopentene formation by using hetero-substituted enyne or related variant is a very powerful technique in catalytic heterocycle synthesis and is a common intermediate for various multi-component coupling^20–24^. Highly 1,3-syn-diastereoselective synthesis has been achieved in a few examples according to the steric demands of enyne substituents and metallacycle formation (Fig. 2b)^25–27^, however, other possible combinations were explored rarely (e.g., 1,3- 1,4-diastereodivergent, and in higher substituted cases). Moreover, unlike a few examples noted in intramolecular hydroalkenylation of diene (Fig. 2c)^28,29^, the scope is mostly limited to those equipped with cyclic template, some requires Thorpe-Ingold effect assistance, and no efficient diastereodivergent example has been developed. Developing a novel and general diastereodivergent strategy which is not relied primarily on minimizing undesired steric repulsion among Rs is therefore useful, especially for those sterically less accessible and higher substituted products.

Here, a NHC-Ni catalyzed highly diastereodivergent heterocycle synthetic method is thus developed (Fig. 2d). That is based on conformational cooperation in the nickelacyclopentene formation step, directed by the choice of N-substituents on the heteroenynes, and promoted by NHC-Ni catalyst π-electronic effect. By trapping the NHC-nickelacyclopentenes with alcohol, silane and aldehyde, 1,3- and 1,4-diastereodivergent reductive hydroalkenylation^30–32^, silylation and acylation products are obtained^26,33^.

## Results

### Optimization for N-substituents directed diastereodivergent reductive hydroalkenylation

Our study commenced with a set of simple 1,7-heteroenynes **1a–f** having a racemic stereocenter and terminal propargyl-Z for a study of N-substituents on nickelacyclopentene selective formation (Table 1, entry 1–6, Eq. (1), Z = O, NH or NMs). The condition employed is as simple as an NHC-Ni(0) catalyzed enyne cycloaddition reported in the literature with shorter tethers^24^, except 1-phenylethanol is used as terminal reductant to complete the desired catalytic cycle by reductive hydroalkenylation under mild condition (Supplementary Table 1). To our delight, a NHC-Ni(0) catalyzed reductive hydroalkenylation of enyne **1** was observed. High yield and high reactivity were obtained by using a catalytic amount of IPr-Ni(0) in toluene. This is useful because selective nickelacyclopentene formation is less efficient for those terminal enynes due to highly competitive and undesired [2 + 2 + 2] oligomerization or dimerization. Yet, the most remarkable result is that a highly diastereodivergent piperidine synthesis was achieved by this rarely employed strategy. Also, the role of R^1^ size in 1,3-*syn-*:*anti-*selectivity was found not so crucial (R^1^ = Ph c.f. Me). A high 1,3-syn-:anti-selectivity (**3**:**3′**) was observed by using **1c**-**d** (with propargyl-Z = NH). On the contrary, a high 1,3-anti-selectivity (**4′**) was noted in **1e-f** (with propargyl-Z = NMs). Oxa-enyne **1a-b** followed the same reactivity and selectivity pattern as **1c-d** did. Moreover, a highly efficient diastereodivergent 1,4-stereotransfer was observed by using enyne **2** with different allyl-Z as substrate (Table 2, Eq. (2), Z = O, NH or NMs, R^1^ = Ph or Me). Heterocycles **5′** and **6** were obtained as major products (Table 2, entry 1–6). Similarly, the preferred product stereo-configuration (syn*-*:anti*-*selectivity) was found correlated to the selected choice of Z. Yet, the preferred stereo-configuration directed by allyl-Z was found different from the propargyl-Z cases (Tables 1 and 2, e.g., allyl-NMs favored 1,4-syn*-*, propargyl-NMs favored 1,3-anti*-*).

**Table 1:** Substituent effects on NHC-Ni catalyzed diastereodivergent reductive hydroalkenylation of enyne with propargyl Z ^a^.


Entry	1	Z	R^1^	Ligand	Product	*Syn:Anti* 3:3′	*Syn:Anti* 4:4′	Yield (%)
1	**1a**	O	Ph	IPr SIPr	**3a**	**>95**:5 **92**:8	*n.a*.	74 43
2	**1b**		Me	IPr	**3b**	**>95**:5		78
3	**1c**	NH	Ph	IPr	**3c**	**96**:4^c^	*n.a*.	81
4	**1d**		Me	IPr	**3d**	**>95**:5		62
5	**1e**	NMs	Ph	IPr	**4′e**	*n.a*.	6:**94**^c^	84
				SIPr			18:**82**^c^	48
				IMes			*n.a*.	0
				PCy_3_^b^			19:**81**^c^	11
6	**1** **f**	NMs	Me	IPr	**4′d**	*n.a*.	12:**88**	58
7	**1** **g**	NTs	Ph	IPr	**4′g**	*n.a*.	40:**60**	30
8	**1** **h**	NBn		IPr	**3i**	**88**:12	*n.a*.	48

^a^ See Methods section for procedure. Homo-dimerization and oligomerization were obtained in some ineffective cases. Products were shown in relative configuration only.

^b^ 20 mol% Ni, Ni:PCy_3_ = 1:2.

^c^ By GCMS.

**Table 2:** Substituent effects on NHC-Ni catalyzed diastereodivergent reductive hydroalkenylation of enyne with allyl Z^a^.


Entry	2	Z	R^1^	Ligand	Product	*Syn:Anti* 5:5′	*Syn:Anti* 6:6′	Yield (%)
1	**2a**	O	Ph	IPr SIPr	**5′a**	8:**92** 21:**79**	*n.a*.	78 32
2	**2b**		Me	IPr	**5′b**	11:**89**		64
3	**2c**	NH	Ph	IPr	**5′c**	3:**97**^b^	*n.a*.	51
4	**2d**		Me	IPr	**5′d**	7:**93**		76
5	**2e**	NMs	Ph	IPr	**6e**	*n.a*.	**93**:7^b^	73
				SIPr			**85**:15^b^	21
6	**2f**		Me	IPr	**6f**		**94**:6	72

^a^ See Methods section for procedure. Homo-dimerization and oligomerization were obtained in some ineffective cases. Products were shown in relative configuration only.

^b^ By GCMS.

At first, one might consider the steric difference in Z is sufficient to explain the above diastereodivergent synthesis. Yet, very similar efficiency among enynes having different sizes of R^1^ in both Eqs. (1) and (2) caught our attention. That made us suspected it was not just directed by simple steric interaction, so Z with different steric and electronic property was evaluated next (Supplementary Table 2). First, using sterically bulkier Z than NMs were found not helpful in favoring anti-selectivity further but led to a drop in selectivity (Table 1, N-Ms vs -Ts and -Bn, entry 5, 7 and 8). This is unusual since a larger Z like NTs or NBn is expected to favor a stronger steric repulsion among R^1^ and Z for more anti-product. Second, using a bulky N-Bn could favor the syn-product as N-H did (Table 1, entry 3, 8). These 2 sets of results are in sharp contrast to the former rationale that based on steric effect increment on Z from NH to NMs alone. Furthermore, Z = NTf did not provide the desired reactivity. Altogether, the diastereodivergent synthesis as noted above was attributed mainly to the use of Z with different electronic property. Yet, the size of Z is still critical in terms of diastereodivergent efficiency, in which using small Z could avoid competing steric interactions. Overall, the diastereodivergent synthesis efficiency and preference did not follow the order of the Z sizes to change gradually (Size: N-H < Ms < Bn~Ts, while the Syn-:Anti-ratio ranged from 96:4 to 6:94 in order of N-H > Bn > Ts > Ms). Similar phenomenon was noted in Table 2, Eq. (2), entry 3 and 5.

### Ligand effect on diastereoselectivity

The above results prompted us to screen ligands with different steric and electronic properties by using **1a**, **1c** and **1e** as well as **2a**, **2c** and **2e** (Tables 1, 2, Supplementary Table 3, 4). In general, using bulky NHC is one of the keys to obtain desired reactivity. Indeed, non-selective oligomerization and dimerization was noted in IMes and PCy_3_ (Table 1, entry 5). More interestingly, the NHC electronic effect has a strong impact on the diastereodivergent efficiency in both equations and IPr performed much better than SIPr in general (Table 1, entry 1, 5; Table 2, entry 1, 5), in which dramatic drops were observed when using Z = NMs and SIPr. This result suggested that the use of NHC with lower π-accepting ability is highly desirable for higher diastereodivergent efficiency, given that IPr and SIPr are very similar in size and σ-donating power^34,35^. Also, it showed that the above is not entirely a Z controlled process.

### Substrate scope of diastereodivergent reductive hydroalkenylation

**Fig. 3:**
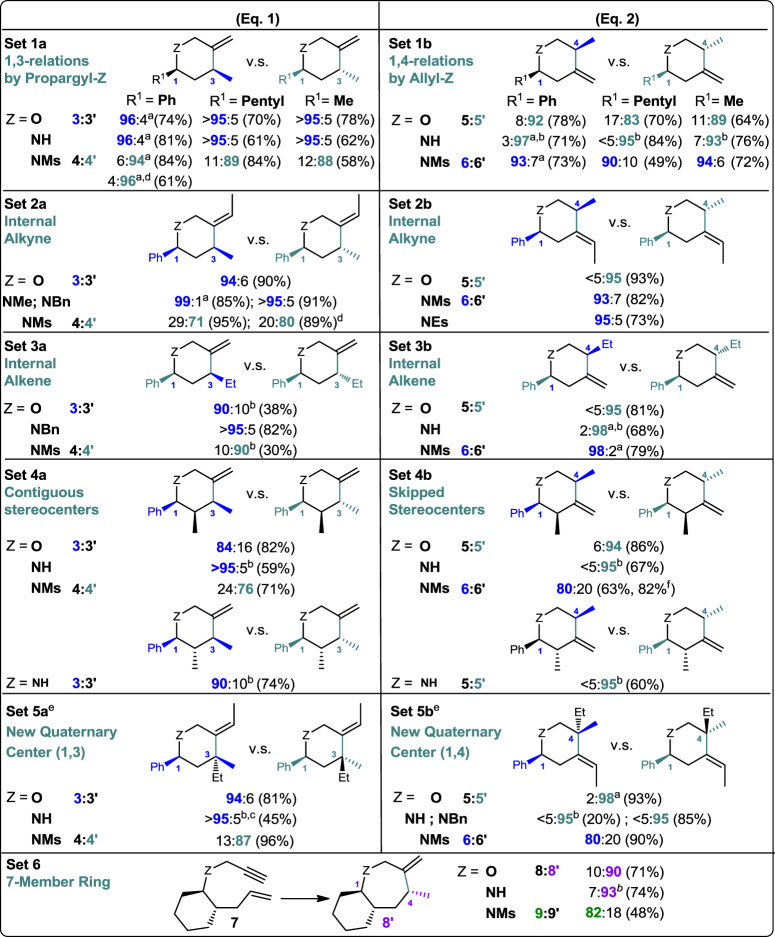
Scope of the diastereodivergent reductive hydroalkenylation of enyne by NHC-Ni(0) catalyst and 1-phenylethanol. See Methods section for hydroalkenylation procedure; see Tables 1 and 2 for Eqs. (1) and (2). Product syn:anti-selectivity was determined by NMR, yield of desired product in relative configuration is in parenthesis. **a** By GCMS. **b** 2 h addition, to suppress oligomerization. **c** 20 mol% catalyst. **d** IPr^Me^ was used instead of IPr. **e** enyne: alcohol = 1:1.5, to avoid undesired enyne direct reduction. **f** 3 equiv. of NaBH_4_, to avoid acetophenone insertion (from the 1-phenylethanol).

The fine-tuned reductive hydroalkenylation reactivity, which was brought by propargyl-/allyl-Z, 2° alcohol and NHC-Ni cooperation, also came with a broad scope of **1** and **2** (Fig. 3). It provided a general access to functionalized hydroalkenylation products (3 and 6, 4′ and 5′) in one step by simply using an in situ generated catalyst from over-the-counter sources. Enynes with aryl and alkyl substitutions (Set 1), with internal alkenes and alkynes (Set 2 and 3), with nearby stereocenter interference at 2-position (Set 4), with gem-disubstituted alkenes (Set 5) and with a longer chain length (Set 6) are all good substrates for this reductive hydroalkenylation (Eqs. (1) and (2)). Only a few cases required a slow addition of enyne, protected NH and employed IPr^Me^ as NHC. Those changes were used to compete with the undesired oligomerization. Overall, this method allows us to synthesis heterocycle derivatives with higher substituted olefins, longer side chains, 1,2,3-contagious and 1,2,4-skipped stereocenters, new quaternary centers, and 7-member rings.

The robust reactivity also came along with moderate to excellent 1,3- and 1,4-diastereodivergent efficiency (e.g., from up to 2:98 to 98:2). Several notable results deserve further comment. First, the diastereodivergent synthesis efficiency is excellent even in cases with substituents as small as Me. It highlighted that the basis of this switching is not primarily on steric repulsion (Set 1, Eqs. (1) and (2)). Second, the method can still perform even when interfered by an extra 2-substituent at syn/anti-relative configuration (Set 4). This indicated that such gauche interactions are not competitive enough to the desired preference directed by our strategy (e.g., Z = NH, 95:5 vs 90:10 in Eq. (1); <5:95 in Eq. (2)). Hence, assortments of densely substituted stereoisomers are now easily accessible by this reductive hydroalkenylation. Third, stereo-defined 4° centers only having marginal steric differences are hard to make selectively (Set 5, e.g., Me vs Et). Our approach offers a possible entry to build those very similar products as well as isomers. Overall, it complements to a few shortcomings by the traditional steric approaches, the cyclic templates, the rearrangement of side chains or the stoichiometric organometallic reagents^19,36–39^.

## Discussion

**Fig. 4:**
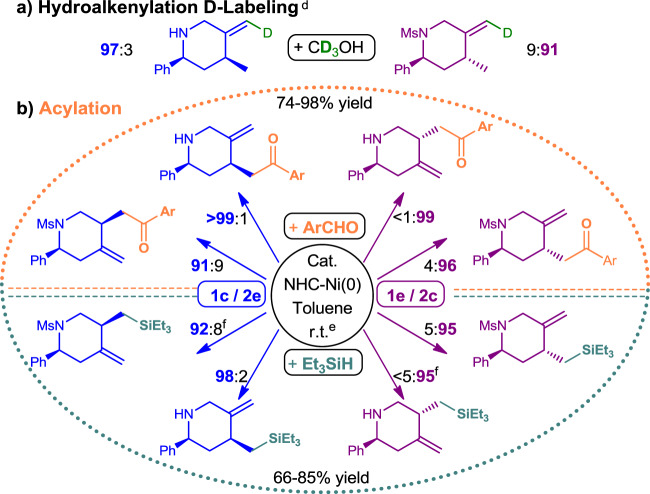
Labeling experiment and diastereodivergent applications. See Supplementary Methods. **a** D-Labeling experiment of reductive hydroalkenylaition. **b** Diastereodivergent acylation; NHC = IPr, Enyne: (*p*-anisyl)CHO = 1:1.5. **c** Diastereodivergent silylation; NHC = IPr^Cl^, Enyne: TESH = 1:8. **d** 50 mol% catalyst, NHC = IPr, 91–94% D-labeled in the products. **e** 10 mol% catalyst, Syn-:Anti-ratio by GCMS (Supplementary Method E). **f** by NMR.

**Fig. 5:**
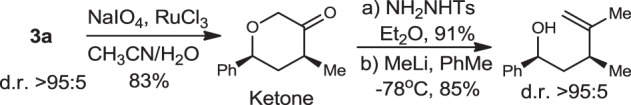
Synthetic applications. (**i**) Heterocyclic ketone, and (**ii**) Acyclic alkenol (Supplementary Methods).

The precise mechanism for our desired reactivity and the diastereodivergent strategy are still under investigation, yet some insights were obtained from the studies that used CD_3_OH, ArCHO and TESH instead of 1-phenylethanol (Fig. 4). First, using CD_3_OH in the reductive hydroalkenylation of **1c** and **1e** yielded the corresponding D_1_-**3c** and D_1_-**4′e** (Fig. 4a). The stereo-defined D_1_-olefin obtained and high D-incorporation suggest a nickela-cyclopentene mechanism rather than Ni-H insertion to enyne at this stage^40^. Second, by using ArCHO or TESH as enyne reaction partners in place of alcohol, several sets of highly efficient diastereodivergent acylations or silylations were achieved, respectively (Fig. 4b, c)^24,26^. The excellent diastereodivergent synthesis efficiency and trend noted at here are comparable with the above reductive hydroalkenylation (Tables 1, 2, Fig. 3). This strongly suggested the strategy is related to a common diastereodivergent nickelacyclopentene formation step, and not by an in situ generated Ni(II)H from NHC-Ni(0) and 2° alcohol. Thus, the diastereodivergent synthesis could be a Z directed NHC-nickelacyclopentene formation with a suitable choice of NHC, not on choices of partners^41–43^. Synthetically, the exocyclic-olefin also links up many useful organic transformations (Fig. 5), which broaden the impacts and the applications of this hydroalkenylation.

In summary, an efficient 1,3- and 1,4-diastereodivergent heterocycle synthesis strategy was established by tuning property of Z on enynes under NHC-Ni catalysis (switch from up to 2:98 to 98:2). It was demonstrated by a reductive hydroalkenylation of enynes using 2° alcohol as reductant. Highly competing side reactions were suppressed and broad scope was achieved simultaneously. Unlike a number of former diastereodivergent approaches that based on facial selection at the latter stage of the catalytic cycle, here the diastereodivergent efficiency is governed by Z directed nickelacyclopentene forming step at the start (a 3 x bonds formation event, including 1 x C–C and 2 x Ni–C bond). Moreover, that can be promoted by choice of NHC ligand (IPr vs SIPr). That feature makes the preferred diastereodivergent outcome highly predicable and tunable, which in turn makes it less dependent on steric interactions among external reaction partners and substituents on the tether. Products packed densely with diastereocenters which is difficult to be made by conventional steric approaches (either sterically unfavorable or hardly distinguishable, e.g., preparation of 1,2,3-contagious and skipped stereocenters, and new 4° center with Me vs Et) are now easily accessible expediently, rather than just the sterically favorable ones. Finally, the diastereodivergent results obtained in acylations and silylations imply a great potential of this work in joining many other transformations and cross-coupling reactions that based on nickelacyclopentenes and related species for broader scope. Exploration along this line is now underway.

## Methods

Preparation of hetero-substituted enynes: See Supplementary Methods.

Determination of diastereoselectivity: See Supplementary Methods, Supplementary Figs. 4–10.

Acylation procedure: See Supplementary Methods.

Sylilation procedure: See Supplementary Methods.

Products characterization: See Supplementary Data 1.

Standard reductive hydroalkenylation procedure: Ni(cod)_2_ and IPr (0.05 mmol each) were dissolved in toluene (2 mL) and stirred at 30 °C in glovebox for 1 hr. An enyne and 1-phenylethanol (0.5: 1.5 mmol) toluene solution (1 mL) were added dropwise to the above catalyst in 0.5 h, and was stirred at 30 °C for 3 h. After work up, the yield, structure and selectivity were determined by ^1^H NMR, NOESY, isolation and GCMS (average of two runs). Mesylation of NH products were carried out for direct comparison of the isomers when necessary (Supplementary Method A).

## Supplementary information

Supplementary Information

Description of Additional Supplementary Files

Supplementary Data 1

## Data Availability

The authors declare that all the other data supporting the findings of this study are available within this paper, its Supplementary Information file and Supplementary Data 1.
