# Supplementary material for: NHC-Ni catalyzed 1,3- and 1,4-diastereodivergent heterocycle synthesis from hetero-substituted enyne
Source: Commun Chem. 2020 Apr 30;3:50. doi: 10.1038/s42004-020-0299-9 (PMC9814851; doi:10.1038/s42004-020-0299-9)
Supplement: Supplementary file 1 — Supplementary Information [file 42004_2020_299_MOESM1_ESM.pdf]

NHC-Ni Catalyzed 1,3- and 1,4-Diastereodivergent Heterocycle Synthesis from Hetero-substituted Enyne

Xuefeng Yong *et al.*

Supplementary Methods

General aspect. Unless otherwise indicated, all reactions were performed in glove box from which oxygen and moisture were excluded from reagents and glassware. $Ni(cod)_2$ [Bis(1,5-cyclooctadiene)nickel(0)], IPr [1,3-Bis(2,6-diisopropylphenyl)-imidazol-2-ylidene], IMes [1,3-Bis(2,4,6-trimethylphenyl)imidazol-2-ylidene] were purchased from Acros or Aldrich, stored in a glovebox and used without further purification. PCy_3 [Tricyclohexyl phosphine] was purchased from Energy, stored in a glove box before use. 1-Phenethylalcohol was purchased from Aldrich, and was filtered through a short plug of silica gel before use. Toluene was distilled over sodium and CaH_2 before use.

Most of the enynes are unknown compounds and could be prepared from readily available materials accordingly or analogously. Analytical thin layer chromatography (TLC) was performed using EM Science silica gel 60 F254 plates. The developed chromatogram was analyzed by UV lamp (254 nm), ethanolic phosphomolybdic acid (PMA) or basic potassium permanganate ($KMnO_4$). Purification of product was performed by using Silica Gel (230–400 mesh, 0.040–0.063 mm) coarse fritted glass column. Desired reductive hydroalkenylation products and cycloaddition products were isolated by column chromatography on silica gel. ^{1}H and ^{13}C NMR spectra were recorded on Bruker spectrometers in $CDCl_3$ (400 or 500 MHz for ^{1}H and 101 or 126 MHz for ^{13}C). Chemical shifts in ^{1}H NMR spectra are reported in ppm on the δ scale from an internal standard of TMS. Data are reported as follows: chemical shift, multiplicity (s = singlet, d = doublet, t = triplet, q = quartet, m = multiplet, br = broad), coupling constant in hertz (Hz), and integration. Chemical shifts of ^{13}C NMR spectra are reported in ppm from the central peak of $CDCl_3$ (77.16 ppm) on the δ scale. Yield and selectivity was determined by integration of areas of selected peaks in crude ^{1}H NMR with relaxation time d1 = 10 seconds and benzaldehyde as internal standard. Relative configuration of the reductive hydroalkenylation product was determined by 2D NMRs.

High resolution mass spectra (HRMS) were obtained on a Finnigan MAT 95XL GC Mass Spectrometer of the Southern University of Science and Technology (SUSTech), China.

Method A: Reductive Hydroalkenylation of Enyne with 1-Phenethylalcohol

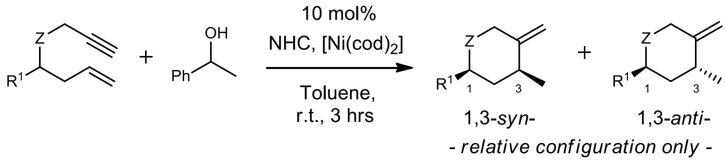

<u>Catalyst preparation and reductive hydroalkenylation:</u> In a glove box, the $Ni(cod)_2$ and IPr (in 1:1 mol ratio, 0.05 mmol each) catalyst were dissolved in toluene (2 mL) in an oven dried scintillation vial equipped with a magnetic stir bar, and this catalyst solution was stirred for at least 1 hr at r.t. before use. To the above, a solution of hetero-enyne (**1**, **2** or **7**) and 1-phenylethanol (0.5:1.5 mmol, in 1 mL toluene) was added drop wise over 0.5 hr (or indicated amount and time) at r.t..

<u>General work up procedure:</u> After 3 hrs, the crude mixture was diluted with hexane (4 mL), and stirred in open air for an additional 1 hr. Then it was filtered through a short plug of silica gel and rinsed by ethyl acetate (75 mL). The filtrate was collected, solvent was removed under reduced pressure, and the crude reaction mixture was subjected to ^{1}H NMR analysis using benzaldehyde as NMR standard. Product ratio (**3:3′**, **4:4′**, **5:5′**, **6:6′**, **8:8′**, **9:9′**) were determined by NMR or GC-MS (see later section) and isolation, the relative configuration was determined by 2D NMR analysis and compared with related derivatives (See later section). Products were isolated by silica gel chromatography using hexane/ethyl acetate or DCM.

<u>Representative example using 1e and 1-phenylethanol:</u>

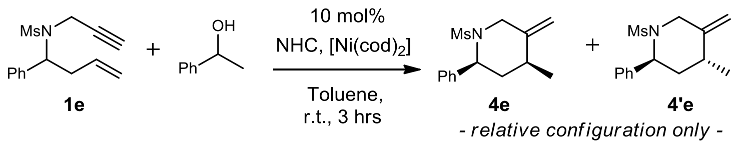

In a glove box, $Ni(cod)_2$ (13.8 mg, 0.05 mmol) and IPr (19.4 mg, 0.05 mmol) were dissolved in an oven dried scintillation vial using 2 mL toluene equipped with magnetic stir

bar. The catalyst solution was stirred at r.t. for at least 1 hr. To the above, a solution of 0.5 mmol enyne **1e** and 1.5 mmol 1-phenylethanol in 1 mL toluene was added drop wise in 0.5 hr. After 3 hrs, it was worked up and analyzed by following the general work up procedure. 84% yield, **4e**:**4′e** = 6:94 (1,3-syn:anti, by GC-MS).

Method B: Reductive Hydroalkenylation of Enyne with CD_3OH

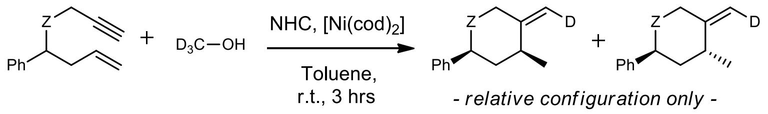

In a glove box, $Ni(cod)_2$ and IPr (in 1:1 mol ratio, 0.1 mmol each) were dissolved in 2 mL toluene and was stirred for at least 1 hr before use. To the above catalyst solution, a solution of enyne and CD_3OH (0.2:1.0 mmol) in 2 mL toluene was added drop wise in 0.5 hr.

After 3 hrs, it was worked up and analyzed by following the general work up procedure.

As above procedure was followed, the following products were obtained when enynes **1c** and **1e** were used respectively (Fig. 3a).

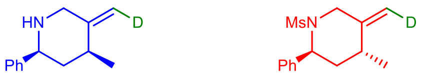

**Product**	D_1-**3c**	D_1-**4'e**
**1,3-*syn*:anti**	**97**:3	9:**91**
**Yield**	41%	37%

Method C: Acylation of Enyne with Aldehyde

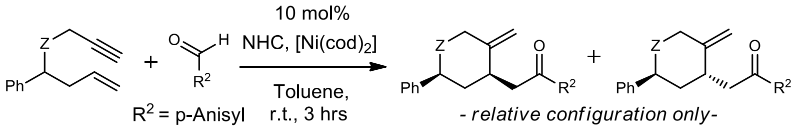

In a glove box, $Ni(cod)_2$ and IPr (1:1 mol ratio, 0.05 mmol each) were dissolved in 2 mL toluene and was stirred for at least 1 h at r.t. before use. To the catalyst, a solution of 0.5 mmol hetero-enyne and 0.75 mmol p-anisaldehyde in 1 mL toluene was added drop wise in 0.5 hr.

After 3 hrs, it was worked up and analyzed by following the general work up procedure.

As above procedure was followed, the following products were obtained when enynes **1c**, **1e**, **2c** and **2e** were used respectively (Fig. 3b).

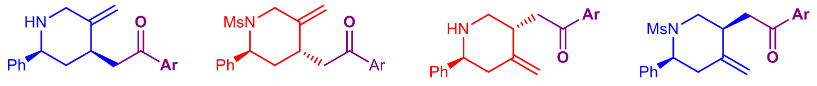

Product	Acyl-3c	Acyl-4'e	Acyl-5'c	Acyl-6e
Syn:*Anti*	>**99**:1	4:**96**	<1:**99**	**91**:9
Yield	62%	98%	81%	74%

Method D: Silylation of Enyne with Silane

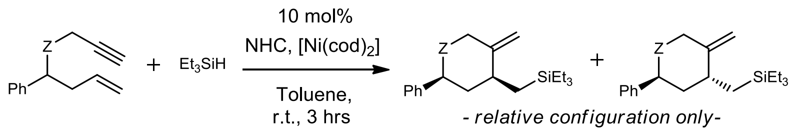

In a glove box, $Ni(cod)_2$ and IPr^{Cl} (1:1 mol ratio, 0.1 mmol each) were dissolved in 2 mL toluene and was stirred for at least 1 h at r.t. before use. To the catalyst, a solution of 0.5 mmol hetero-enyne and 4 mmol Et_3SiH in 1 mL toluene was added drop wise in 0.5 hr.

After 3 hrs, it was worked up and analyzed by following the general work up procedure.

As above procedure was followed, the following products were obtained when enynes **1c**, **1e**, **2c** and **2e** were used respectively (Fig. 3c).

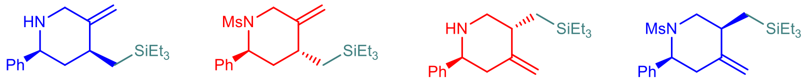

Product	Si-3c	Si-4'e	Si-5'c	Si-6e
Syn:*Anti*	**98**:2	5:**95**	<5:**95**	**92**:8
Yield	74%	85%	66%	67%

Diastereoselectivity determination by NMR

Most of the product ratio and structural assignment were determined by using NMR (^{1}H, ^{13}C, 135 DEPT, COSY, HSQC and NOESY).

The above data analysis is actually quite straight forward since the authentic minor isomers could be obtained by the following post-modification (mesylation). It also provided the authentic minor isomers for GC-MS analysis in some cases. The oxacycles were assigned and compared with the corresponding azacylces.

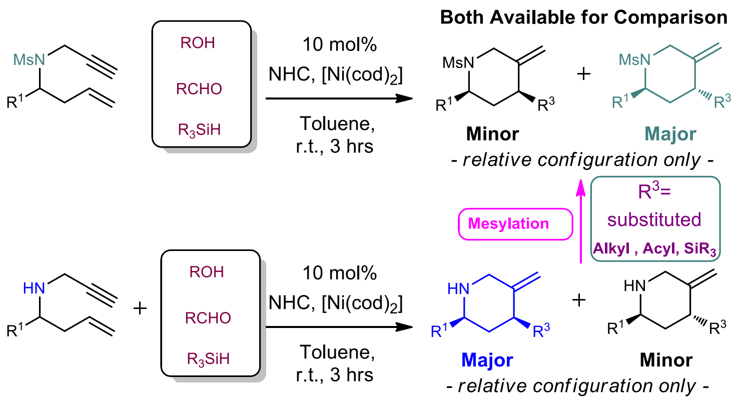

Supplementary Fig. 1 Access to both diastereomers of NMs azacycles

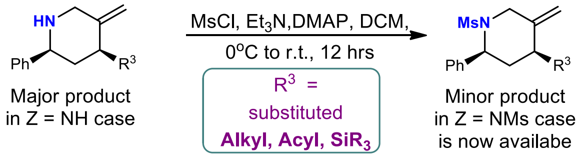

Supplementary Fig. 2 Mesylation of NH azacycles to its NMs derivatize

The product (0.5 mmol) was mesylated under buffered condition at 0 °C (1.5 mmol Et_3N and 10 mg DMAP in 10 mL DCM) by dropwise addition of MsCl (1 mmol in 2 mL DCM). After stirred overnight at r.t., saturated $NaHCO_3$ (aq) (10 mL) was added. Organic layer was washed with brine, then dried over $MgSO_4$. Solvent was removed and product was subjected to NMR or GC-MS analysis. Product was isolated by silica gel chromatography.

The mesylation of crude reaction mixture could be done by using the same method as shown above when necessary (e.g. obtain the NMR for a comparison with the crude enyne reductive hydroalkenylation in Z = NMs case).

Diastereoselectivity determination by GCMS

In some cases where NMR analysis is not good enough to tell the high product diastereoselectivity, those were determined by GC-MS. The products retention time were assigned by MS as well as the authentic samples obtained by mesylation in above section.

GC-MS were obtained on Agilent Technologies 7890B GC system and 5977A MSD of Southern University of Science and Technology, China. Column: Agilent Technologies HP-5MS UI, 30 m * 0.25 mm, 0.25 micron. GC-MS temperature programming method is listed below:

	Rate (℃/min)	Value (℃)	Hold time (min)	Run time (min)
Initial		50	2	2
Ramp 1	30	200	1	8
Ramp 2	40	280	15	25

The NMs derivatives allowed reasonable separation and therefore the NH product was converted to NMs for a comparison:

In the following, the spectrum on the right was obtained from reductive hydroalkenylation product using enyne with Z = NMs directly, while the spectrum on the left was obtained from product derived from mesylation of NH products.

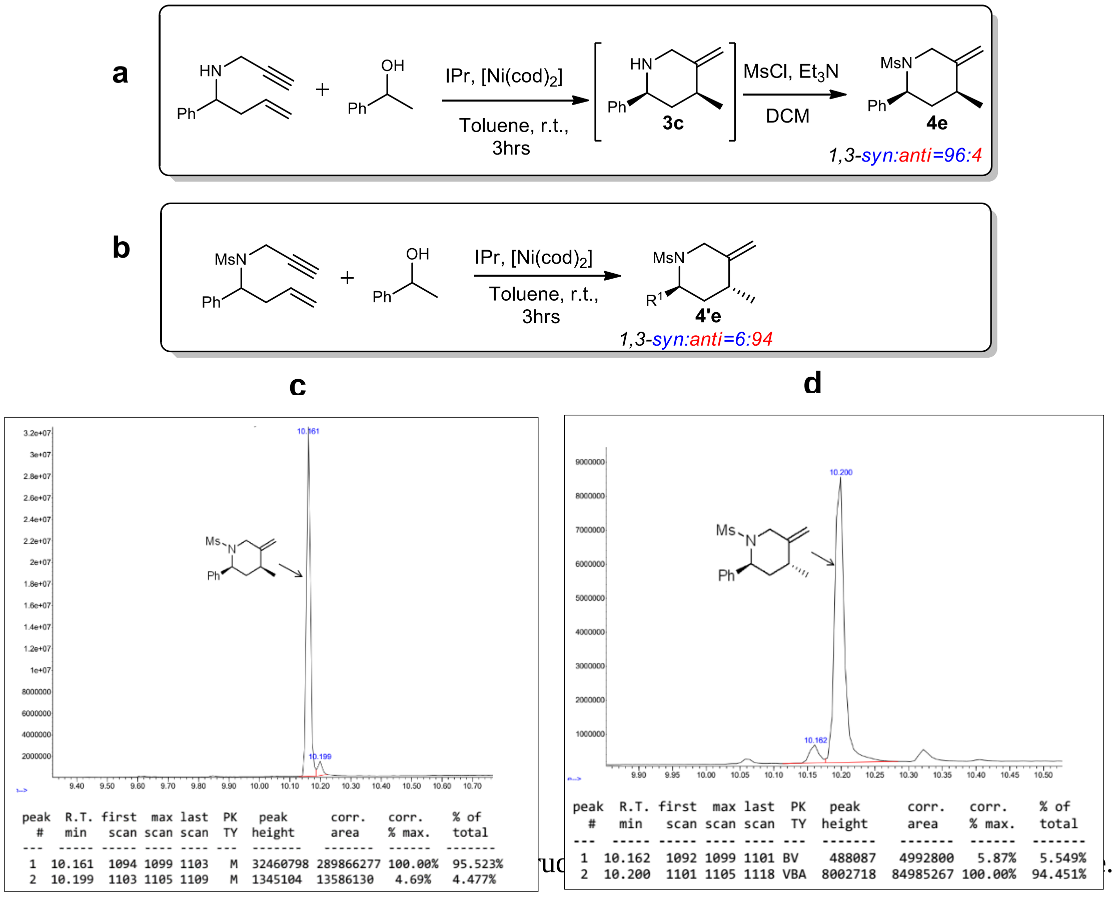

peak #	R.T. min	first scan	max scan	last scan	PK TY	peak height	corr. area	corr. % max.	% of total
1	10.161	1094	1099	1103	M	32460798	289866277	100.00%	95.523%
2	10.199	1103	1105	1109	M	1345104	13586130	4.69%	4.477%

peak #	R.T. min	first scan	max scan	last scan	PK TY	peak height	corr. area	corr. % max.	% of total
1	10.162	1092	1099	1101	BV	488087	4992800	5.87%	5.549%
2	10.200	1101	1105	1118	VBA	8002718	84985267	100.00%	94.451%

b **4'e** prepared from reductive hydroalkenylation. **c** Diastereoselectivity determination of **3c** by GCMS after mesylation. **d** Diastereoselectivity determination of **4'e** by GCMS.

a

b

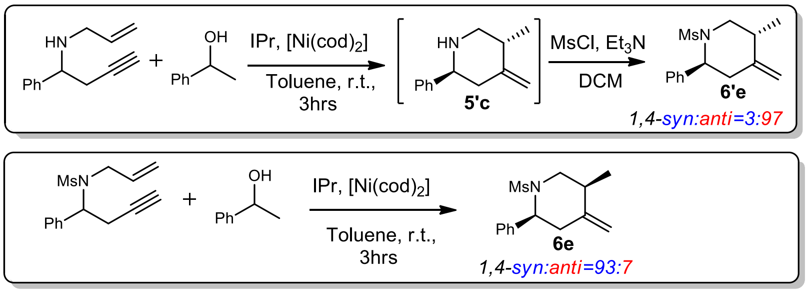

c

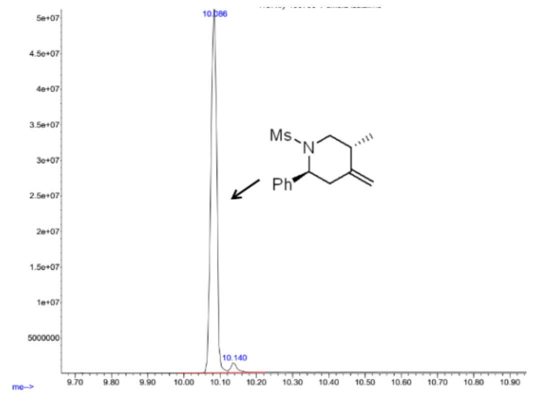

peak #	R.T. min	first scan	max scan	last scan	PK TY	peak height	corr. area	corr. % max.	% of total
1	10.086	1072	1087	1093	BV	51416965	581555088	100.00%	96.991%
2	10.140	1093	1096	1109	VV	1326917	18042091	3.10%	3.009%

d

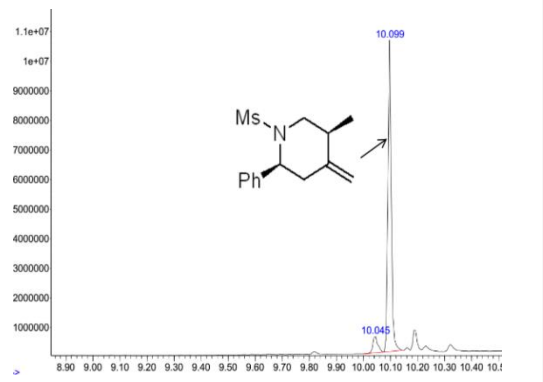

peak #	R.T. min	first scan	max scan	last scan	PK TY	peak height	corr. area	corr. % max.	% of total
1	10.045	1074	1081	1086	BV 2	543415	6844618	7.66%	7.113%
2	10.099	1086	1089	1097	PBA	9107525	89376480	100.00%	92.887%

Supplementary Fig. 5 **a** Mesylation of crude **5’c**.from reductive hydroalkenylation to **6’e**. **b** **6e** prepared from reductive hydroalkenylation. **c** Diastereoselectivity determination of **5’c** by GCMS after mesylation. **d** Diastereoselectivity determination of **6e** by GCMS

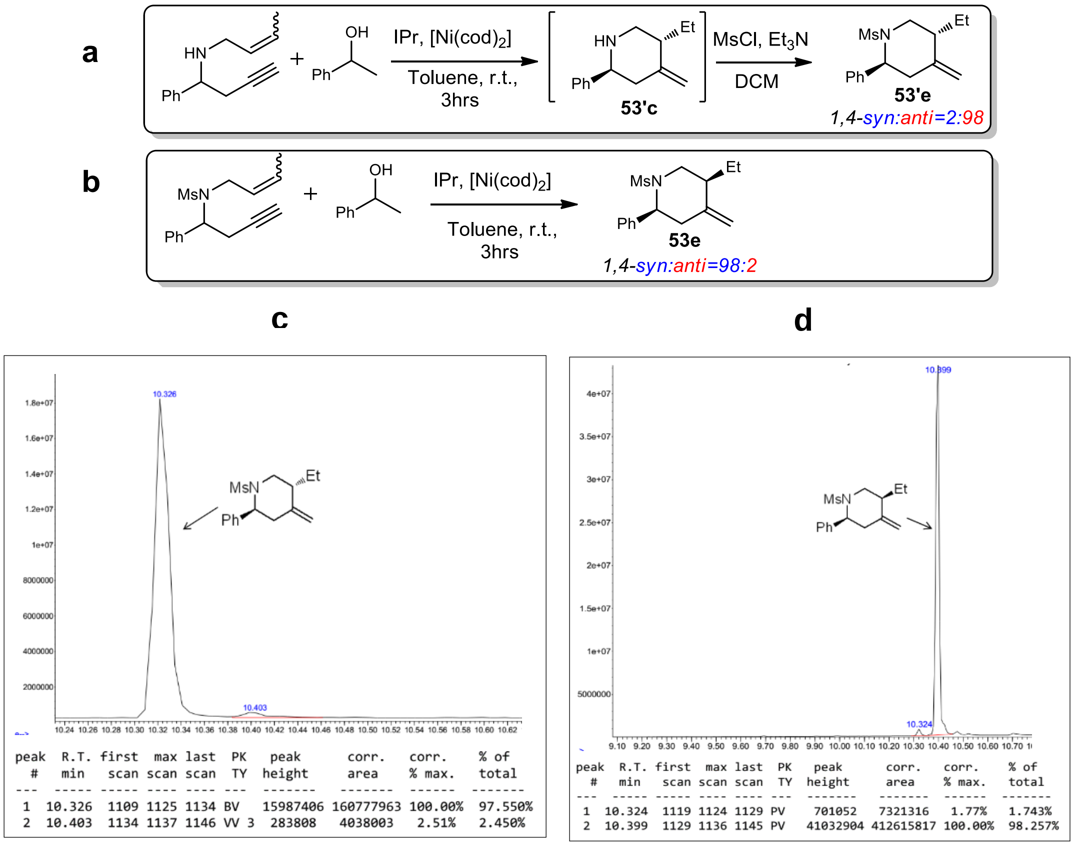

Supplementary Fig. 6 **a** Mesylation of crude **53'c**.from reductive hydroalkenylation to **53'e**. **b** **53e** prepared from reductive hydroalkenylation. **c** Diastereoselectivity determination of **53'c** by GCMS after mesylation. **d** Diastereoselectivity determination of **53e** by GCMS

a

b

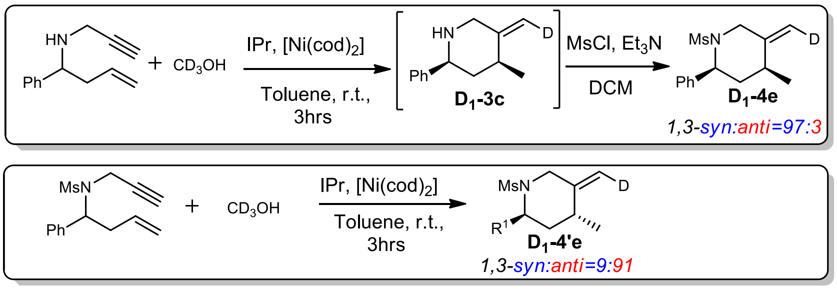

c

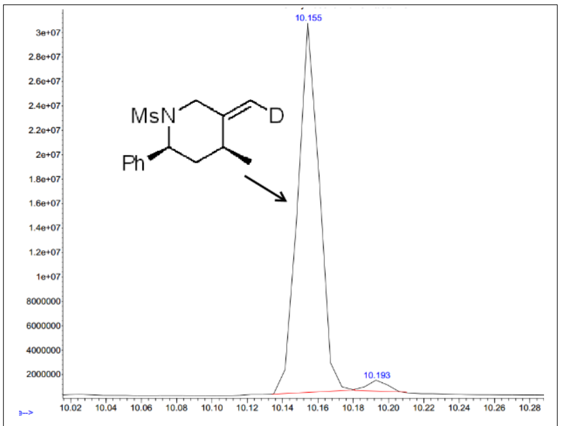

peak #	R.T. min	first scan	max scan	last scan	PK TY	peak height	corr. area	corr. % max.	% of total
1	10.155	1095	1098	1102	M	30303171	257284344	100.00%	97.455%
2	10.193	1102	1104	1107	M	904812	6719171	2.61%	2.545%

d

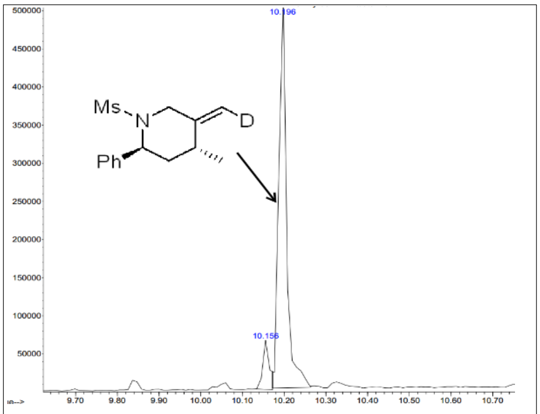

peak #	R.T. min	first scan	max scan	last scan	PK TY	peak height	corr. area	corr. % max.	% of total
1	10.156	616	618	620	M	64502	646485	9.69%	8.836%
2	10.196	620	622	628	M	519829	6670124	100.00%	91.164%

Supplementary Fig. 7 **a** Mesylation of crude **D_1-3c**.from reductive hydroalkenylation to **D_{1}-4e**. **b** **D_{1}-4'e** prepared from reductive hydroalkenylation. **c** Diastereoselectivity determination of **D_1-3c** by GCMS after mesylation. **d** Diastereoselectivity determination of **D_{1}-4'e** by GCMS

a

b

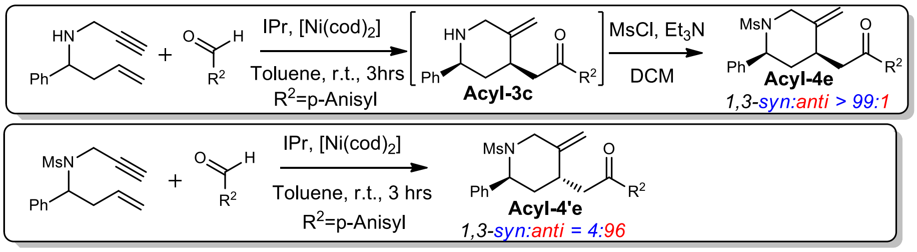

c

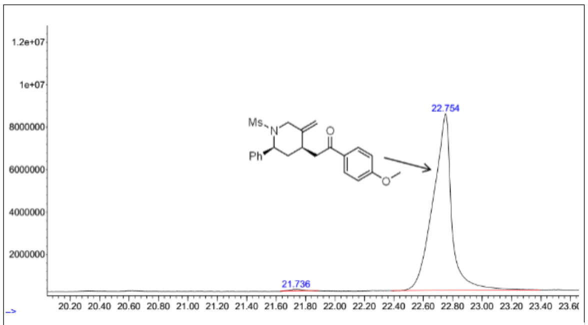

peak #	R.T. min	first scan	max scan	last scan	PK TY	peak height	corr. area	corr. % max.	% of total
1	21.736	2881	2897	2921	M5	73108	4489991	0.61%	0.605%
2	22.754	2998	3055	3155	BB	8301216	737845758	100.00%	99.395%

d

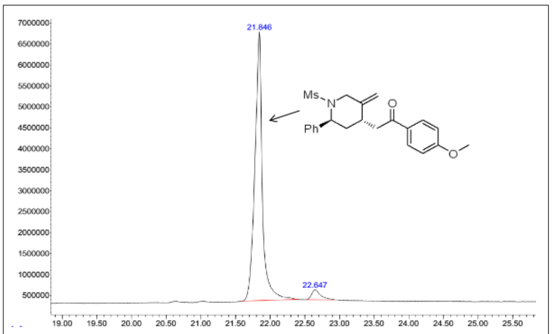

peak #	R.T. min	first scan	max scan	last scan	PK TY	peak height	corr. area	corr. % max.	% of total
1	21.846	2875	2914	3003	BB	6387342	484635943	100.00%	95.720%
2	22.647	3019	3039	3087	BB 5	240940	21671398	4.47%	4.280%

Supplementary Fig. 8 **a** Mesylation of crude **Acyl-3c**.from reductive hydroalkenylation to **Acyl-4e**. **b** **Acyl-4’e** prepared from reductive hydroalkenylation. **c** Diastereoselectivity determination of **Acyl-3c** by GCMS after mesylation. **d** Diastereoselectivity determination of **Acyl-4’e** by GCMS

a

b

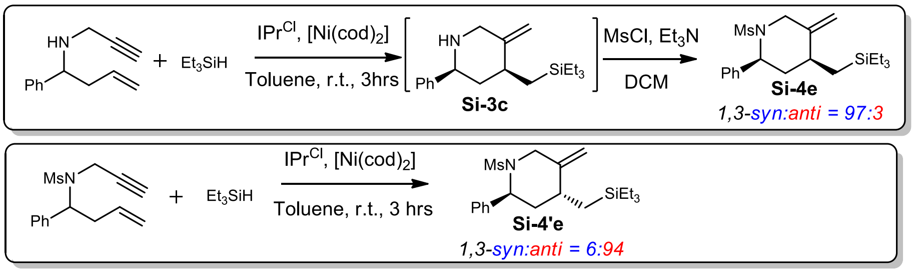

c

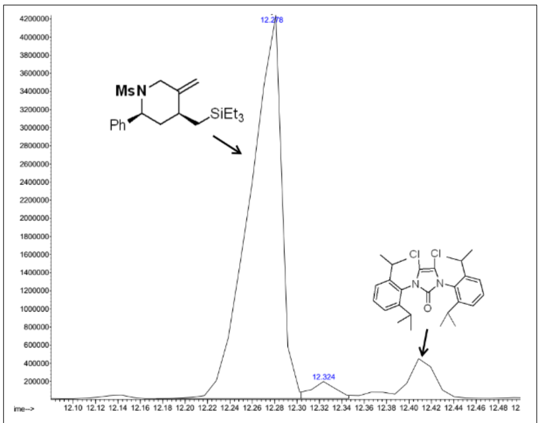

peak #	R.T. min	first scan	max scan	last scan	PK TY	peak height	corr. area	corr. % max.	% of total
1	12.278	808	818	820	M	4464548	84017795	100.00%	96.630%
2	12.324	820	822	824	M	188980	2930466	3.49%	3.370%

d

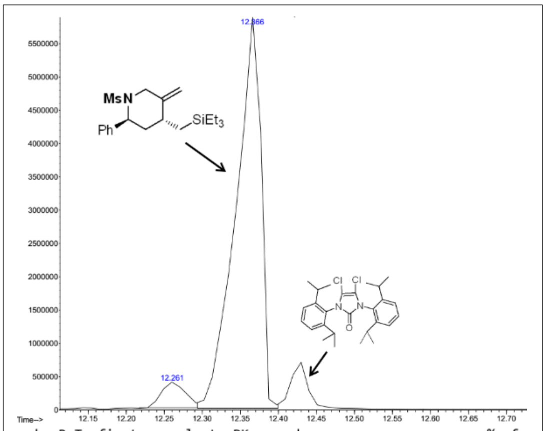

peak #	R.T. min	first scan	max scan	last scan	PK TY	peak height	corr. area	corr. % max.	% of total
1	12.261	812	816	819	M	386563	8894629	6.51%	6.112%
2	12.366	819	826	829	M	5884625	136630661	100.00%	93.888%

Supplementary Fig. 9 **a** Mesylation of crude **Si-3c**.from reductive hydroalkenylation to **Si-4e**. **b** **Si-4'e** prepared from reductive hydroalkenylation. **c** Diastereoselectivity determination of **Si-3c** by GCMS after mesylation. **d** Diastereoselectivity determination of **Si-4'e** by GCMS

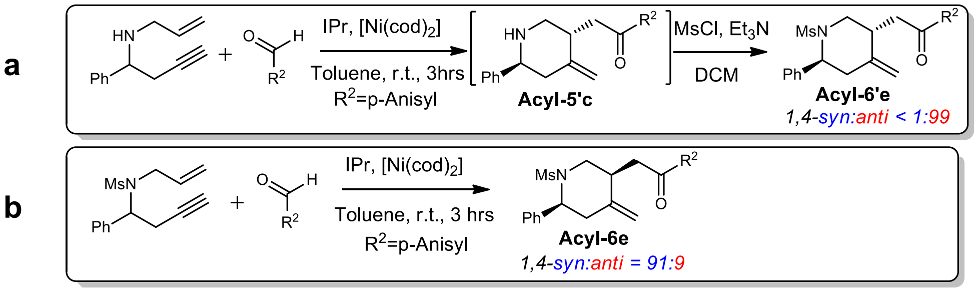

c

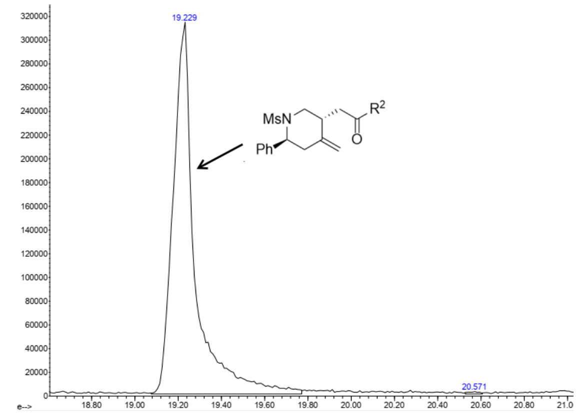

peak #	R.T. min	first scan	max scan	last scan	PK TY	peak height	corr. area	corr. % max.	% of total
1	19.229	1457	1472	1523	M	315981	22149489	100.00%	99.662%
2	20.571	1594	1598	1601	M4	2116	75230	0.34%	0.338%

d

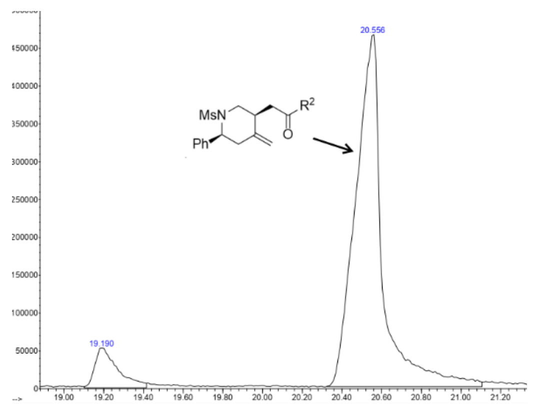

peak #	R.T. min	first scan	max scan	last scan	PK TY	peak height	corr. area	corr. % max.	% of total
1	19.190	1460	1468	1489	M3	52483	4249010	9.56%	8.726%
2	20.556	1575	1597	1648	M	469057	44445362	100.00%	91.274%

Supplementary Fig. 10 **a** Mesylation of crude **Acyl-5'c**.from reductive hydroalkenylation to **Acyl-5'e**. **b** **Acyl-6e** prepared from reductive hydroalkenylation. **c** Diastereoselectivity determination of **Acyl-5'c** by GCMS after mesylation. **d** Diastereoselectivity determination of **Acyl-6e** by GCMS.

Supplementary Table 1 Screening of alcohol for reductive hydroalkenylation of **1c** [a]

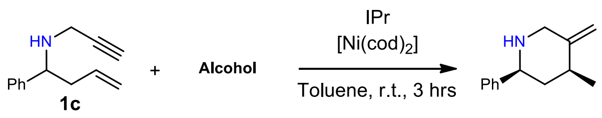

Entry	Alcohol	Catalyst loading (mol %)	Enyne/Alcohol (mol/mol)	Yield (%)
1	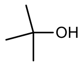	20	1:5	0
2	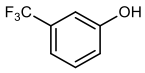	20	1:5	0
3	MeOH	50	1:5	41
4		20	1:5	16
5	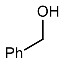	20	1:3	30
6	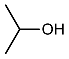	20	1:3	18
7	Cy-OH	20	1:3	28
8		20	1:3	87
9	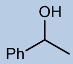	10	1:3	81
10		10	1:1.5	68

[a] Standard hydroalkenylation procedure was followed. 1,3-syn:anti ratio was > 95:5 in all cases by GC-Ms except entry 1-2. Yield was determined by crude NMR.

The above showed that the alcohol structure is very important for the desired reductive hydroalkenylation reactivity, only the bulky acyclic secondary alcohol gave us good to high yield when used in excess (entry 8-10, 1-phenylethanol). It should be noted that the desired reactivity was also observed when other 1° or 2° alcohol was used (entry 1-2 vs 3-10), but no correlation among alcohol structure and stereoselectivity was found (syn:anti > 95:5). Condition employed in Entry 9 was selected as the standard condition for the later on screening and study.

Supplementary Table 2 Effect of N-substitution on reductive hydroalkenylation [a]

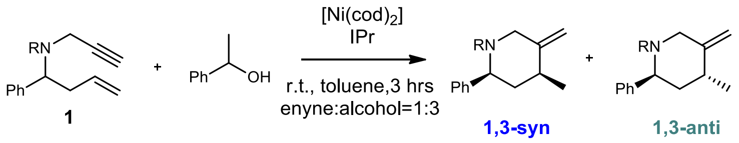

Entry	R	Yield (%)	1,3-syn: anti
1	H	81	**96**:4[b]
2	Bn	48	**88**:12[c]
3	Ts	30	40:**60**[c]
4	Ms	84	6:**94**[d]

[a] Conversion of enyne was 100% in all above cases. [b] Determined by GC-MS after mesylation of rude product; [c] Determined by NMR; [d] Determined by GC-Ms.

Unlike some of the steric controlled transformations, the gradual increase in N-substituent R steric effect did not increase/decrease the selectivity gradually (entry 1-4). Smaller R gave us higher selectivity in both syn- and anti-product preparation, while larger R gave us lower selectivity in both cases.

Interestingly, the preferred product stereoselectivity was correlated mainly to the electronic property of the R choice (syn in H, Bn vs anti in Ts, Ms).

All of the above indicated that the change in stereoselectivity preference from R = H to Ms may not be a direct result of optimal steric effect alone and the electronic property of N-substitution plays a significant role in the stereoselectivity preference change, which prompted us to study the ligand effect below.

Supplementary Table 3 Ligand effect on reductive hydroalkenylation of **1**

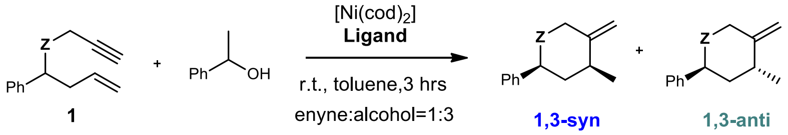

Entry	Z	Ligand	Yield (%)	1,3-Syn:Anti
1	NH	SIPr	27	**90**:10[a]
2		IPr	81	**96**:4[a]
3	NMs	SIPr	48	18:**82**[b]
4		IPr	84	6:**94**[b]
5	O	SIPr	43	**92**:8[c]
6		IPr	74	**96**:4[b]

Note: Conversion of enyne was 100% in all cases. [a] Determined by GC-MS after mesylation of the rude product; [b] Determined by GC-MS; [c] Determined by NMR.

In general, the desired reductive hydroalkenylation reactivity was low when small ligand (e.g. PCy_3 give <10% yield, IMes gave 0% yield) or dppe was used (0% yield). Only bulky NHC gave result for an effective comparison.

Here, bulky unsaturated NHC (IPr) always performed better than the corresponding saturated NHC (SIPr) in all cases examined (Z = NH, NMs and O, entry 1-6). The effect on yield is more significant when Z = O and NH (2.5-3 times better, entry 1, 2, 5, 6), while the effect on syn:anti ratio is more significant when Z = NMs (entry 3 and 4).

Supplementary Table 4 Ligand Effect on reductive hydroalkenylation of **2** [a]

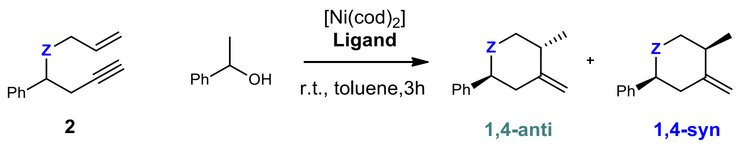

Entry	Z	Ligand	Yield (%)	1,4-Syn:Anti
1	NH	IPr	51	3:**97**[b]
2		SIPr	19	6:**94**[b]
3	NMs	IPr	73	**93**:7[c]
4		SIPr	21	**85**:15[c]
5	O	IPr	78	8:**92**[d]
6		SIPr	32	21:**79**[d]

[a] Conversion of enyne was 100% in all cases above. [b] Determined by GC-MS after mesylation of rude product; [c] Determined by GC-MS; [d] Determined by NMR.

The above indicated that the NHC electronic property is very important in both desired reactivity and stereoselectivity.

NHCs Ligands employed in this paper were listed below. IPr and SIPr were purchased from TCI and used without further purification. IPr^{Me} and IPr^{Cl} were prepared according to literature procedure.[1,2]

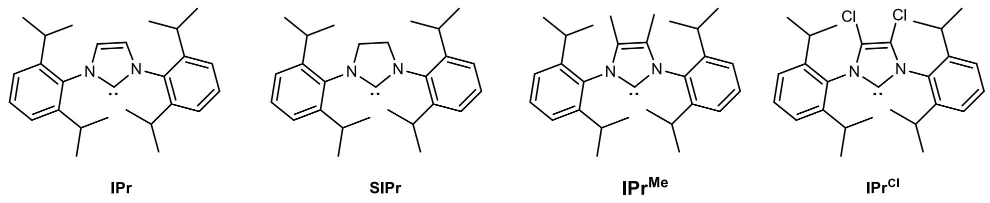

Supplementary Fig. 3 Structure of NHC ligands employed

General synthesis of heteroenynes

Most of the enynes were prepared in 1-3 steps from the corresponding aldehyde and aldimine based on the relevant methods reported in the literature (Supplementary Figure 11).[3]

The first step was Zinc-mediated Barbier-type allylation or propargylation of aldehyde [4] or aldimine,[5] and the second step was alkylation of the alcohol or amine.

A few enynes which could be not obtained efficiently by above method were prepared with Mitsunobu reaction as key step (see later).

All the aldimine were prepared according to literature procedure. [6-9]

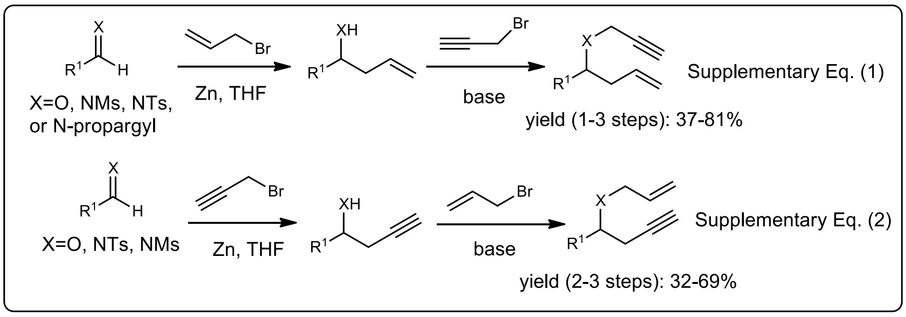

Supplementary Fig. 11

a) <u>Enynes for Set 1a, Table 3</u>

The oxa-enynes were prepared similar to literature procedure. The aza-enynes were prepared by Barbier type allylic addition, followed by treatment with propargyl bromide under basic conditions (typically Cs_2CO_3/DMF).

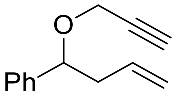

1a

Prepared according to literature procedure.

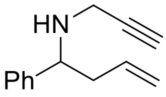

1c

Started from corresponding N-propargyl aldimine,[8] the enyne was obtained as a colorless to bright yellow oil with 81% yield by allylation in 1 step (Supplementary Eq. (1)).

^{1}H NMR (400 MHz, $CDCl_3$) : δ 7.37 – 7.22 (m, 5H), 5.81 – 5.66 (m, 1H), 5.17 – 5.05 (m, 2H), 3.92 (dd, J = 8.2, 5.6 Hz, 1H), 3.36 (dd, J = 17.2, 2.4 Hz, 1H), 3.09 (dd, J = 17.2, 2.4 Hz, 1H), 2.51 – 2.33 (m, 2H), 2.20 (t, J = 2.4 Hz, 1H).

^{13}C NMR (101 MHz, $CDCl_3$) : δ 142.8, 135.3, 128.6, 127.6, 127.5, 118.0, 82.4, 71.4, 60.5, 42.8, 35.9.

HRMS ESI (m/z): $[M+H]^+$ calcd for $C_{13}H_{16}N$, 186.1277; found, 186.1272.

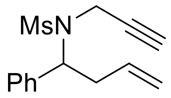

1e

Started from N-Ms aldimine,[9] the enyne was obtained as a colorless to bright yellow oil, 68% yield for 2 steps (Supplementary Eq. (1)).

^{1}H NMR (400 MHz, $CDCl_3$) : δ 7.49 – 7.31 (m, 5H), 5.88 (ddt, *J* = 17.0, 10.2, 6.8 Hz, 1H), 5.27 – 5.11 (m, 3H), 4.09 (dd, *J* = 18.8, 2.5 Hz, 1H), 3.73 (dd, *J* = 18.8, 2.5 Hz, 1H), 3.02 (s, 3H), 2.95 – 2.84 (m, 2H), 2.31 (t, *J* = 2.5 Hz, 1H).

^{13}C NMR (126 MHz, $CDCl_3$) : δ 138.0, 134.7, 128.8, 128.5, 128.4, 118.7, 79.8, 73.3, 61.3, 42.1, 36.0, 33.0.

HRMS ESI (m/z): $[M+NH_4]^+$ calcd for $C_{14}H_{21}N_2O_2S$, 281.1318; found, 281.1309.

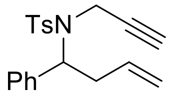

1g

Started from N-Ts aldimine,[6] the enyne was obtained as bright sticky yellow oil, 72% yield for 2 steps (Supplementary Eq. (1)).

^{1}H NMR (400 MHz, $CDCl_3$) : δ 7.80 (d, *J* = 7.8 Hz, 2H), 7.39 – 7.20 (m, 7H), 5.59 (ddt, *J* = 17.0, 10.0, 6.7 Hz, 1H), 5.11 (dd, *J* = 9.3, 6.5 Hz, 1H), 5.01 (d, *J* = 17.1 Hz, 1H), 4.92 (d, *J* = 10.2 Hz, 1H), 4.18 (dd, *J* = 18.6, 2.4 Hz, 1H), 3.59 (d, *J* = 18.7 Hz, 1H), 2.93 (dt, *J* = 15.5, 8.0 Hz, 1H), 2.62 (dt, *J* = 13.4, 6.5 Hz, 1H), 2.43 (s, 3H), 2.14 – 2.08 (m, 1H).

^{13}C NMR (101 MHz, $CDCl_3$) : δ 143.5, 138.1, 137.3, 134.7, 129.5, 128.6, 128.6, 128.2, 127.8, 117.7, 79.9, 72.7, 61.0, 35.6, 32.9, 21.7.

HRMS ESI (m/z): $[M+H]^+$ calcd for $C_{20}H_{22}NO_2S$, 340.1371; found, 340.1376.

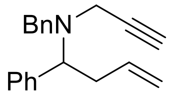

1h

Started from N-Bn aldimine, the enyne was obtained as colorless oil, 74% yield for 2 steps (Supplementary Eq. (1)).

^{1}H NMR (400 MHz, $CDCl_3$) : δ 7.49 – 7.22 (m, 10H), 5.65 (ddt, *J* = 14.2, 10.2, 6.9 Hz, 1H), 5.06 – 4.91 (m, 2H), 3.85 (dd, *J* = 8.8, 4.7 Hz, 1H), 3.72 (d, *J* = 13.4 Hz, 1H), 3.55 – 3.43 (m, 2H), 3.20 (dd, *J* = 17.7, 2.1 Hz, 1H), 2.85 – 2.74 (m, 1H), 2.58 (dt, *J* = 8.4, 7.9 Hz, 1H), 2.28 (t, *J* = 1.9 Hz, 1H).

^{13}C NMR (101 MHz, $CDCl_3$) : δ 141.8, 139.4, 135.6, 129.0, 128.7, 128.4, 128.3, 127.3, 127.1, 116.8, 79.1, 73.2, 65.6, 54.4, 38.6, 38.4.

HRMS ESI (m/z): $[M+H]^+$ calcd for $C_{20}H_{22}N$, 276.1747, found, 276.1742.

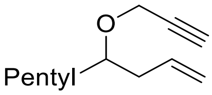

Started from the hexanal, enyne was obtained as yellow oil, 72% for 2 steps (Supplementary Eq. (1)).

^{1}H NMR (400 MHz, $CDCl_3$) : δ 5.90 – 5.76 (m, 1H), 5.20 – 5.09 (m, 2H), 3.70 – 3.60 (m, 1H), 2.36 – 2.26 (m, 1H), 2.19 – 2.09 (m, 1H), 1.58 – 1.23 (m, 11H), 0.89 (t, J = 6.8 Hz, 3H).

^{13}C NMR (126 MHz, $CDCl_3$) : δ 134.8, 117.2, 80.6, 78.3, 73.8, 56.3, 38.1, 33.6, 32.1, 25.0, 22.7, 14.2.

HRMS ESI (m/z): $[M+H]^+$ calcd for $C_{12}H_{21}O$, 181.1592, found, 181.1588.

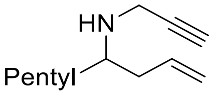

Started from the N-propargyl aldimine, the enyne was obtained as a bright yellow oil, 76% yield for 1 step (Supplementary Eq. (1)).

^{1}H NMR (400 MHz, $CDCl_3$) : δ 5.90 – 5.66 (m, 1H), 5.14 – 5.00 (m, 2H), 3.48 – 3.31 (m, 2H), 2.82 – 2.66 (m, 1H), 2.26 – 2.18 (m, 1H), 2.16 (t, J = 2.3 Hz, 1H), 2.12 – 2.03 (m, 1H), 1.48 – 1.19 (m, 8H), 0.84 (t, J = 6.7 Hz, 3H).

^{13}C NMR (101 MHz, $CDCl_3$) : δ 135.5, 117.4, 82.4, 71.0, 55.0, 38.1, 35.6, 33.4, 32.0, 25.2, 22.6, 14.0.

HRMS ESI (m/z): $[M+H]^+$ calcd for $C_{12}H_{22}N$, 180.1752, found, 180.1747.

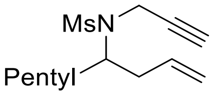

Prepared by treating corresponding N-H enyne with MsCl/Et_3N in DCM to afford the enyne as a yellow oil, 78% yield for 1 step (Supplementary Eq. (1)).

^{1}H NMR (500 MHz, $CDCl_3$) : δ 5.81 – 5.70 (m, 1H), 5.10 – 5.01 (m, 2H), 3.95 (dd, J = 18.7, 2.4 Hz, 1H), 3.88 (dd, J = 10.9, 2.5 Hz, 1H), 3.88 – 3.79 (m, 1H), 2.97 (s, 3H), 2.36 – 2.22 (m, 3H), 1.50 (q, J = 7.3 Hz, 2H), 1.39 – 1.31 (m, 1H), 1.29 – 1.20 (m, 5H), 0.83 (t, J = 6.9 Hz, 3H).

^{13}C NMR (126 MHz, $CDCl_3$) : δ 135.0, 117.8, 79.8, 72.8, 58.8, 41.6, 38.2, 32.7, 31.5, 31.3, 26.3, 22.5, 14.0.

HRMS ESI (m/z): $[M+H]^+$ calcd for $C_{13}H_{24}NO_2S$, 258.1582, found, 258.1523.

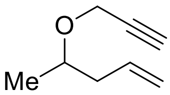

1b

Started from pent-4-en-2-ol, the enyne was obtained as colorless oil with 52% yield for 1 step (Supplementary Eq. (1)), purified by vacuum distillation (46 ^{o}C at 50 mbar).

^{1}H NMR (400 MHz, $CDCl_3$) : δ 5.82 (ddt, J = 17.2, 10.3, 7.0 Hz, 1H), 5.26 – 5.00 (m, 2H), 4.18 (dd, J = 3.6, 2.5 Hz, 2H), 3.72 (h, J = 6.1 Hz, 1H), 2.40 (t, J = 2.4 Hz, 1H), 2.34 (dt, J = 12.5, 6.2 Hz, 1H), 2.21 (dt, J = 14.0, 6.8 Hz, 1H), 1.17 (d, J = 6.2 Hz, 3H).

^{13}C NMR (101 MHz, $CDCl_3$) : δ 134.7, 117.2, 80.5, 74.2, 73.9, 55.7, 40.7, 19.2.

HRMS ESI (m/z): $[M+H]^+$ calcd for $C_8H_{13}O$, 125.0961; found, 125.0959.

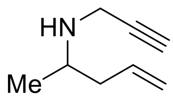

1d

Started from the N-propargyl aldimine, the enyne was obtained as a yellow oil with 62% yield for 1 step (Supplementary Eq. (1)).

^{1}H NMR (400 MHz, $CDCl_3$) : δ 5.78 (ddt, *J* = 17.3, 10.2, 7.1 Hz, 1H), 5.15 – 5.06 (m, 2H), 3.49 (dd, *J* = 17.2, 2.4 Hz, 1H), 3.41 (dd, *J* = 17.2, 2.4 Hz, 1H), 2.95 (h, *J* = 6.3 Hz, 1H), 2.20 (t, *J* = 2.4 Hz, 1H), 2.15 (ddd, *J* = 7.5, 5.9, 1.4 Hz, 2H), 1.05 (d, *J* = 6.3 Hz, 3H).

^{13}C NMR (101 MHz, $CDCl_3$) : δ 135.5, 117.6, 82.3, 71.3, 50.6, 41.4, 35.6, 19.7.

HRMS ESI (m/z): $[M+H]^+$ calcd for $C_8H_{14}N$, 124.1126; found, 124.1120.

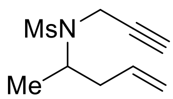

1f

Prepared by treating corresponding N-H enyne with MsCl/Et_3N in DCM to afford the enyne as yellow oil with 82% yield.

^{1}H NMR (500 MHz, $CDCl_3$) : δ 5.79 (m, 1H), 5.19 – 5.07 (m, 2H), 4.05 – 3.95 (m, 3H), 3.02 (s, 3H), 2.42 (ddd, *J* = 14.3, 7.1, 1.2 Hz, 1H), 2.34 (t, *J* = 2.5 Hz, 1H), 2.29 (ddd, *J* = 14.3, 7.1, 1.2 Hz, 1H), 1.31 (dd, *J* = 6.8, 1.0 Hz, 3H).

^{13}C NMR (126 MHz, $CDCl_3$) : δ 134.7, 118.0, 80.1, 73.0, 54.2, 41.3, 39.8, 31.8, 19.1.

HRMS ESI (m/z): $[M+H]^+$ calcd for $C_9H_{16}NO_2S$, 202.0902; found, 202.0896.

b) Enynes for Set 1b, Table 3

Preparation of allylic terminal enynes is similar to that of the propargyl terminal enyne. Due to that allylic amine is not available, preparation of N-H enyne was achieved by deprotection of N-Ts enyne under Mg/MeOH condition.[10]

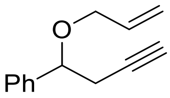

2a

Prepared according to the literature procedure.

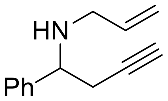

2c

Started from the N-Ts aldimine, the enyne was obtained as yellow oil with 52% for 3 steps (Supplementary Eq. (2)).

^{1}H NMR (400 MHz, $CDCl_3$) : δ 7.41 –7.27 (m, 5H), 5.98 – 5.85 (m, 1H), 5.23 – 5.08 (m, 2H), 3.90 (dd, J = 7.3, 6.0 Hz, 1H), 3.15 (ddt, J = 14.2, 5.4, 1.7 Hz, 1H), 3.06 (ddt, J = 14.2, 6.6, 1.4 Hz, 1H)., 2.59 – 2.55 (m, 2H), 2.06 (t, J = 2.6 Hz, 1H), 1.87 (br, 1H).

^{13}C NMR (101 MHz, $CDCl_3$) : δ 142.5, 136.7, 128.6, 127.6, 127.2, 116.0, 81.6, 70.6, 60.8, 50.0, 28.1.

HRMS ESI (m/z): $[M+H]^+$ calcd for $C_{13}H_{16}N$, 186.1277; found, 186.1273.

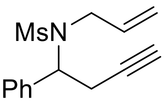

2e

Started from N-Ms aldimine, the enyne was obtained as yellow oil with 63% for 2 steps (Supplementary Eq. (2)).

^{1}H NMR (500 MHz, $CDCl_3$) : δ 7.55–7.32 (m, 5H), 5.93–5.72 (m, 1H), 5.34–5.15 (m, 3H), 3.87 (ddt, J = 16.3, 6.9, 1.4 Hz, 1H), 3.61 (ddt, J = 16.3, 6.4, 1.4 Hz, 1H), 3.04–2.98 (m, 2H), 2.96 (s, 3H), 2.08 (t, J = 2.6 Hz, 1H).

^{13}C NMR (126 MHz, $CDCl_3$) : δ 137.6, 134.7, 128.8, 128.4, 128.0, 118.8, 81.3, 71.7, 59.9, 47.5, 42.1, 22.9.

HRMS ESI (m/z): $[M+H]^+$ calcd for $C_{14}H_{18}NSO_2$, 264.1053; found, 264.1046.

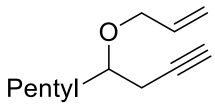

Started from hexanal, the enyne was obtained as a yellow oil with 69% yield for 2 steps (Supplementary Eq. (2)).

^{1}H NMR (400 MHz, $CDCl_3$) : δ 5.99 – 5.86 (m, 1H), 5.28 (d, *J* = 17.2, 1H), 5.16 (d, *J* = 10.3 Hz, 1H), 4.11 (ddd, *J* = 12.7, 5.6, 1.6 Hz, 1H), 3.99 (ddd, *J* = 12.7, 5.8, 1.6 Hz, 1H), 3.46 (td, *J* = 6.0, 5.2, 1.6 Hz, 1H), 2.47 – 2.32 (m, 2H), 2.01 – 1.95 (m, 1H), 1.68 – 1.54 (m, 2H), 1.49 – 1.23 (m, 6H), 0.89 (t, *J* = 6.7 Hz, 3H).

^{13}C NMR (101 MHz, $CDCl_3$) : δ 135.1, 116.8, 81.3, 77.3, 70.4, 69.8, 33.9, 31.8, 24.9, 23.8, 22.6, 14.0.

HRMS ESI (m/z): $[M+H]^+$ calcd for $C_{12}H_{21}O$, 181.1592; found, 181.1587.

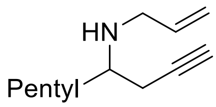

Started from N-Ts aldimine, the enyne was obtained as a yellow oil with 35% yield for 3 steps (Supplementary Eq. (2)).

^{1}H NMR (400 MHz, $CDCl_3$) : δ 5.89 (ddt, *J* = 16.6, 10.3, 6.1 Hz, 1H), 5.18 (dd, *J* = 17.2, 1.8 Hz, 1H), 5.07 (dd, *J* = 10.1, 1.6 Hz, 1H), 3.29 (dd, *J* = 14.0, 6.0 Hz, 1H), 3.21 (dd, *J* = 14.0, 6.0 Hz, 1H), 2.69 (p, *J* = 5.7 Hz, 1H), 2.40 (ddd, *J* = 17.0, 5.5, 2.8 Hz, 1H), 2.27 (ddd, *J* = 17.0, 5.5, 2.8 Hz, 1H), 1.98 (t, *J* = 2.6 Hz, 1H), 1.55 – 1.41 (m, 3H), 1.37 – 1.20 (m, 6H), 0.87 (t, *J* = 6.9 Hz, 3H).

^{13}C NMR (101 MHz, $CDCl_3$) : δ 137.0, 115.9, 81.6, 70.1, 55.2, 49.6, 33.9, 32.0, 25.6, 23.2, 22.6, 14.1.

HRMS ESI (m/z): $[M+H]^+$ calcd for $C_{12}H_{22}N$, 180.1752; found, 180.1743.

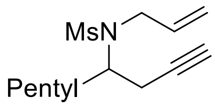

Prepared by treating corresponding N-H enyne with $MsCl/Et_3N$ in DCM to afford the enyne as yellow oil with 77% yield.

^{1}H NMR (500 MHz, $CDCl_3$) : δ 5.97 – 5.85 (m, 1H), 5.29 (d, *J* = 17.2 Hz, 1H), 5.19 (d, *J* = 10.1 Hz, 1H), 4.00 – 3.86 (m, 2H), 3.80 (dd, *J* = 16.2, 6.1 Hz, 1H), 2.96 (s, 3H), 2.52 – 2.39 (m, 2H), 2.08 – 2.02 (m, 1H), 1.63 – 1.55 (m, 2H), 1.39 – 1.23 (m, 6H), 0.88 (t, *J* = 6.2 Hz, 3H).

^{13}C NMR (126 MHz, $CDCl_3$) : δ 135.4, 118.5, 81.7, 71.1, 57.8, 46.5, 41.8, 33.4, 31.6, 26.3, 24.1, 22.6, 14.2.

HRMS ESI (m/z): $[M+H]^+$ calcd for $C_{13}H_{24}NO_2S$, 258.1528; found, 258.1519.

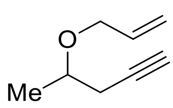

2b

Started from pent-4-yn-2-ol, the enyne was obtained as a colorless oil with 45% yield for 1 step (Supplementary Eq. (2)), which was purified by vacuum distillation (45 °C at 50 mbar).

^{1}H NMR (500 MHz, $CDCl_3$) : δ 5.92 (ddt, *J* = 17.2, 10.4, 5.6 Hz, 1H), 5.29 (dq, *J* = 17.1, 1.6 Hz, 1H), 5.17 (dq, *J* = 10.4, 1.5 Hz, 1H), 4.03 (d, *J* = 5.6 Hz, 2H), 3.69 – 3.59 (m, 1H), 2.47 (ddd, *J* = 16.6, 4.9, 2.7 Hz, 1H), 2.32 (ddd, *J* = 16.6, 7.1, 2.7 Hz, 1H), 2.01 (t, *J* = 2.7 Hz, 1H), 1.28 (d, *J* = 6.1 Hz, 3H).

^{13}C NMR (126 MHz, $CDCl_3$) : δ 135.1, 117.0, 81.3, 73.3, 70.0, 69.9, 26.1, 19.6.

HRMS ESI (m/z): $[M+H]^+$ calcd for $C_8H_{13}O$, 125.0961; found, 125.0960.

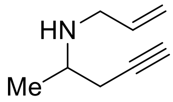

2d

Started from N-Ts aldimine,[7] the enyne was obtained with 32% yield for 3 steps as colorless oil (Supplementary Eq. (2)), which was purified by silica gel chromatography with hexane/EA=8:1 as eluent.

^{1}H NMR (400 MHz, $CDCl_3$) : δ 5.91 (ddt, *J* = 16.3, 10.3, 6.0 Hz, 1H), 5.26 –5.06 (m, 2H), 3.26 (dq, *J* = 6.0, 1.4 Hz, 2H), 2.90 (h, *J* = 6.1 Hz, 1H), 2.40 – 2.23 (m, 2H), 2.02 (t, *J* = 2.6 Hz, 1H), 1.16 (d, *J* = 6.3 Hz, 3H).

^{13}C NMR (101 MHz, $CDCl_3$) : δ 136.9, 116.0, 81.6, 70.3, 51.1, 49.7, 26.1, 20.2.

HRMS ESI (m/z): $[M+H]^+$ calcd for $C_8H_{14}N$,124.1121; found, 124.1121.

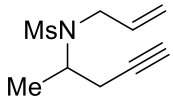

2f

Prepared by treating corresponding N-H enyne with $MsCl/Et_3N$ in DCM to afford the enyne as yellow oil, with 72% yield for 1 step.

^{1}H NMR (500 MHz, $CDCl_3$) : δ 5.88 (ddt, *J* = 16.7, 10.1, 6.3 Hz, 1H), 5.27 (dd, *J* = 17.1, 1.4 Hz, 1H), 5.17 (dd, *J* = 10.1, 1.3 Hz, 1H), 4.06 (dq, *J* = 13.6, 7.0 Hz, 1H), 3.86 (qd, *J* = 16.4, 6.4 Hz, 2H), 2.91 (s, 3H), 2.51 (ddd, *J* = 17.0, 8.0, 2.7 Hz, 1H), 2.40 (ddd, *J* = 17.0, 6.6, 2.6 Hz, 1H), 2.05 (t, *J* = 2.7 Hz, 1H), 1.31 (d, *J* = 6.8 Hz, 3H).

^{13}C NMR (126 MHz, $CDCl_3$) : δ 135.4, 118.2, 81.3, 71.0, 53.4, 46.5, 41.3, 25.5, 19.7.

HRMS ESI (m/z): $[M+H]^+$ calcd for $C_9H_{16}NSO_2$, 202.0896; found, 202.0893.

c) Enynes for Set 2a/b, Table 3

Preparation of internal propargyl enyne was similar to corresponding terminal enyne, except that 1-bromobut-2-yne was used instead of propargyl bromide in last step. In contrast, preparation of the internal alkyne allylic enyne was based on methylation of the corresponding terminal enyne, Supplementary Figure 5.

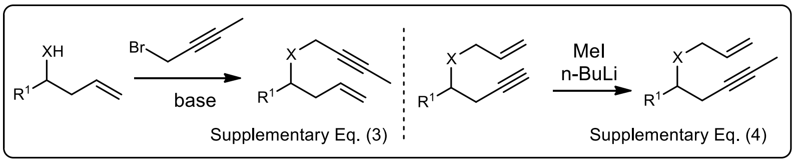

Supplementary Fig. 12

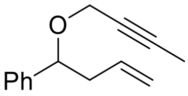

Prepared according to Supplementary Eq. (3): started from benzaldehyde, the enyne was obtained as a bright yellow oil with 78% yield for 2 steps.

^{1}H NMR (500 MHz, $CDCl_3$) : δ 7.39 – 7.26 (m, 5H), 5.76 (ddt, *J* = 17.2, 10.2, 7.0 Hz, 1H), 5.05 (d, *J* = 17.2 Hz, 1H), 5.01 (d, *J* = 10.2 Hz, 1H), 4.49 (t, *J* = 6.8 Hz, 1H), 4.05 (d, *J* = 15.1 Hz, 1H), 3.83 (d, *J* = 15.1 Hz, 1H), 2.68 – 2.58 (m, 1H), 2.50 – 2.39 (m, 1H), 1.85 (s, 3H).

^{13}C NMR (101 MHz, $CDCl_3$) : δ 141.0, 134.7, 128.4, 127.8, 127.0, 117.0, 82.2, 80.4, 75.2, 56.3, 42.2, 3.7.

HRMS-ESI (m/z): $[M+H]^+$ calcd for $C_{14}H_{16}O$, 200.1202; found, 200.1212.

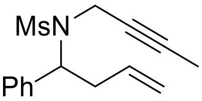

Prepared according to Supplementary Eq. (3): started from N-Ms aldimine, the enyne was obtained as a yellow oil with 64% yield for 2 steps.

^{1}H NMR (400 MHz, $CDCl_3$) : δ 7.45 (d, *J* = 7.8 Hz, 2H), 7.36 (t, *J* = 7.4 Hz, 2H), 7.30 (t, *J* = 7.2 Hz, 1H), 5.98–5.71 (m, 1H), 5.22 (d, *J* = 17.2 Hz, 1H), 5.14 (dd, *J* = 14.2, 7.0 Hz, 1H), 4.02 (d, *J* = 18.6 Hz, 1H), 3.68 (d, *J* = 18.6 Hz, 1H), 2.96 (s, 3H), 2.92 – 2.85 (m, 2H), 1.78 (s, 3H).

^{13}C NMR (126 MHz, $CDCl_3$) : δ 138.2, 134.9, 128.5, 128.4, 128.1, 118.3, 80.9, 74.9, 61.0, 41.8, 36.0, 33.4, 3.5.

HRMS-ESI (m/z): $[M+Na]^+$ calcd for $C_{15}H_{19}NNaO_2S$, 300.1034; found, 300.1031.

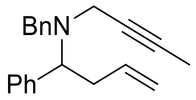

Prepared according to Supplementary Eq. (3): started from N-Bn aldimine, the enyne was obtained as a yellow oil with 69% yield for 2 steps.

^{1}H NMR (400 MHz, $CDCl_3$) : δ 7.60 – 7.14 (m, 10H), 5.71 – 5.59 (m, 1H), 4.97 (dd, *J* = 19.6, 13.7 Hz, 2H), 3.84 (dd, *J* = 8.9, 4.8 Hz, 1H), 3.69 (d, *J* = 13.4 Hz, 1H), 3.48 – 3.36 (m, 2H), 3.11 (d, *J* = 17.2 Hz, 1H), 2.84 – 2.74 (m, 1H), 2.63 – 2.52 (m, 1H), 1.91 (s, 3H).

^{13}C NMR (126 MHz, $CDCl_3$) : δ 141.8, 139.8, 135.8, 129.0, 128.8, 128.3, 128.2, 127.2, 127.0, 116.6, 80.7, 74.2, 65.4, 54.4, 39.0, 38.3, 3.7.

HRMS-ESI (m/z): $[M+H]^+$ calcd for $C_{21}H_{24}N$, 290.1909; found, 290.1900.

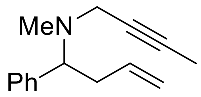

Prepared similar to Supplementary Eq. (3): started from the N-Ts aldimine, the N-H enyne was prepared firstly, then treated with dimethyl sulfate to yield the enyne as a yellow oil with 37% yield for 4 steps.

^{1}H NMR (500 MHz, $CDCl_3$) : δ 7.36 – 7.24 (m, 5H), 5.61 – 5.52 (m, 1H), 4.98 (d, *J* = 17.1 Hz, 1H), 4.92 (d, *J* = 10.2 Hz, 1H), 3.55 (dd, *J* = 9.2, 4.8 Hz, 1H), 3.37 (dt, *J* = 16.6, 2.1 Hz, 1H), 3.12 (dt, *J* = 16.6, 2.2 Hz, 1H), 2.72 – 2.64 (m, 1H), 2.56 – 2.48 (m, 1H), 2.30 (s, 3H), 1.88 (t, *J* = 2.2 Hz, 3H).

^{13}C NMR (126 MHz, $CDCl_3$) : δ 140.9, 135.6, 128.7, 128.2, 127.3, 116.7, 80.8, 74.3, 67.2, 44.4, 39.6, 38.4, 3.7.

HRMS ESI (m/z): $[M+H]^+$ calcd for $C_{15}H_{20}N$, 214.1596; found, 214.1588.

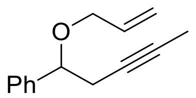

Prepared according to Supplementary Eq. (4): the terminal enyne was prepared firstly according to Supplementary Eq. (2), then treated with n-BuLi and MeI to yield the enyne as a yellow oil (45% yield for 3 steps).

^{1}H NMR (500 MHz, $CDCl_3$) : δ 7.43 – 7.27 (m, 5H), 5.91 (dddd, *J* = 17.2, 10.4, 6.1, 5.0 Hz, 1H), 5.27 (dq, *J* = 17.2, 1.7 Hz, 1H), 5.17 (dq, *J* = 10.4, 1.4 Hz, 1H), 4.44 (t, *J* = 6.6 Hz, 1H), 3.98 (ddt, *J* = 12.8, 5.0, 1.4 Hz, 1H), 3.82 (ddt, *J* = 13.0, 6.1, 1.4 Hz, 1H), 2.65 (ddq, *J* = 16.6, 6.9, 2.6 Hz, 1H), 2.49 (ddq, *J* = 16.5, 6.4, 2.6 Hz, 1H), 1.75 (t, *J* = 2.6 Hz, 3H).

^{13}C NMR (126 MHz, $CDCl_3$) : δ 141.3, 134.9, 128.4, 128.0, 126.9, 117.1, 80.0, 77.5, 75.7, 69.8, 28.6, 3.7.

HRMS ESI (m/z): $[M+H]^+$ calcd for $C_{14}H_{17}O$, 201.1279; found, 201.1274.

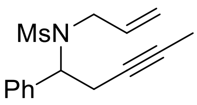

Prepared according to Supplementary Eq. (4): started from the N-Ms aldimine, the terminal enyne was prepared firstly according to Supplementary Eq. (2), then treated with n-BuLi (1.3 equiv.) and MeI (1.4 equiv.) to yield the enyne as a yellow oil (37% yield for 3 steps).

^{1}H NMR (500 MHz, $CDCl_3$) : δ 7.45 – 7.29 (m, 5H), 5.84 – 5.73 (m, 1H), 5.26 – 5.11 (m, 3H), 3.88 (dd, *J* = 16.3, 7.2 Hz, 1H), 3.61 (ddd, *J* = 16.3, 6.1, 1.3 Hz, 1H), 2.96 (s, 3H), 2.93 – 2.87 (m, 2H), 1.76 (t, *J* = 2.5 Hz, 3H).

^{13}C NMR (101 MHz, $CDCl_3$) : δ 138.3, 134.8, 128.7, 128.1, 127.9, 118.5, 78.8, 76.3, 60.3, 47.3, 42.1, 22.9, 3.6.

HRMS ESI (m/z): $[M+Na]^+$ calcd for $C_{15}H_{19}NNaO_2S$, 300.1034; found, 300.1028.

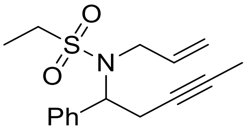

Prepared according to Supplementary Eq. (4): started from the N-Ms aldimine, the terminal enyne was prepared firstly according to Supplementary Eq. (2), then treated with n-BuLi (2.6 equiv.) and MeI (3 equiv.) to yield the enyne as a yellow oil (35% yield for 3 steps).

^{1}H NMR (500 MHz, $CDCl_3$) : δ 7.47 – 7.28 (m, 5H), 5.74 (ddt, *J* = 16.8, 10.1, 6.6 Hz, 1H), 5.22 – 5.05 (m, 3H), 3.86 (dd, *J* = 16.3, 6.7 Hz, 1H), 3.59 (dd, *J* = 16.3, 6.4 Hz, 1H), 3.07 (ddt, *J* = 25.9, 13.9, 7.2 Hz, 2H), 2.97 – 2.84 (m, 2H), 1.74 (t, *J* = 2.6 Hz, 3H), 1.38 (t, *J* = 7.4 Hz, 3H).

^{13}C NMR (126 MHz, $CDCl_3$): δ 138.2, 135.4, 128.5, 128.1, 128.0, 118.0, 78.8, 76.1, 60.1, 48.9, 47.4, 23.1, 8.3, 3.6.

HRMS ESI (m/z): $[M+H]^+$ calcd for $C_{16}H_{22}NO_2S$, 292.1371; found, 292.1365.

d) Enyne for Set 3a/b, Table 3

As shown in **Supplementary Fig. 13**, the internal alkene propargyl enyne prepared via Mitsunobu reaction, while the allylic enyne was prepared similarly to Supplementary Figure 5. The set of enynes were generally a mixture of EZ isomers, of which olefin geometry were not assigned. The mixture was sent to the reductive hydroalkenylation reaction without further purification.

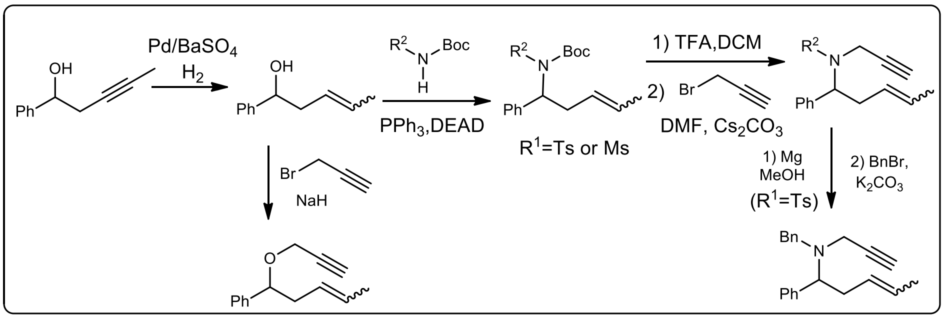

Supplementary Fig. 13

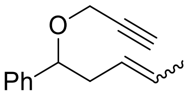

Prepared according to Supplementary Figure 13: the homoallylic alcohol was prepared by Lindlar reduction, and then treated with NaH and propargyl bromide to afford the enyne as a >10:1 mixture of EZ isomer with 72% yield for 2 steps.

^{1}H NMR (400 MHz, $CDCl_3$) : δ 7.41 – 7.27 (m, 5H), 5.57 – 5.45 (m, 1H), 5.44 – 5.34 (m, 1H), 4.50 (t, *J* = 6.8 Hz, 1H), 4.12 (d, *J* = 15.7 Hz, 1H), 3.87 (d, *J* = 15.8 Hz, 1H), 2.63 (dt, *J* = 14.5, 7.1 Hz, 1H), 2.46 (dt, *J* = 13.6, 6.7 Hz, 1H), 2.43 (t, *J* = 2.3 Hz, 1H), 1.51 (d, *J* = 6.7 Hz, 3H).

^{13}C NMR (101 MHz, $CDCl_3$) : δ 141.0, 128.6, 128.0, 127.2, 126.3, 125.9, 80.7, 80.1, 74.2, 55.8, 35.5, 13.0.

HRMS ESI (m/z): $[M+H]^+$ calcd for $C_{14}H_{17}O$, 201.1279; found, 201.1273.

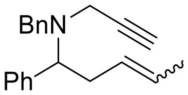

Prepared according to Supplementary Fig.13. The enyne was obtained as a 1.2:1 mixture of EZ isomer with 41% yield for 5 steps.

^{1}H NMR (400 MHz, $CDCl_3$) : δ 7.48 – 7.22 (m, 10H), 5.50 – 5.36 (m, 1H), 5.33 – 5.18 (m, 1H), 3.84 – 3.75 (m, 1H), 3.70 (dd, *J* = 13.4, 5.9 Hz, 1H), 3.55 – 3.42 (m, 2H), 3.26 – 3.13 (m, 1H), 2.88 – 2.77 (m, 0.45H), 2.76 – 2.66 (m, 0.56H), 2.56 – 2.45 (m, 1H), 2.31 – 2.24 (m, 1H), 1.59 (d, *J* = 7.4 Hz, 1.75H), 1.53 (d, *J* = 6.8 Hz, 1.25H).

^{13}C NMR (101 MHz, $CDCl_3$) : δ 142.3, 142.2, 139.5, 139.5, 129.0, 128.8, 128.7, 128.4, 128.4, 128.3, 128.2, 127.8, 127.3, 127.2, 127.2, 127.1, 127.1, 126.9, 125.6, 79.2, 79.2, 73.2, 73.1, 66.0, 65.6, 54.6, 54.4, 38.6, 37.1, 31.4, 18.1, 13.0.

HRMS ESI (m/z): $[M+H]^+$ calcd for $C_{21}H_{24}N$, 290.1909; found, 290.1900.

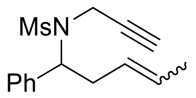

Prepared according to Supplementary Fig. 13: The N-Ms amino group was introduced by Mitsunobu condition. The enyne was obtained as a 7:1 mixture of EZ isomer with 37% yield for 4 steps.

^{1}H NMR (500 MHz, $CDCl_3$) : δ 7.50 – 7.28 (m, 6H), 5.69 – 5.54 (m, 1.14H), 5.50 – 5.37 (m, 1.14H), 5.15 – 5.04 (m, 1.14H), 4.10 – 4.03 (m, 1H), 3.72 (dd, *J* = 18.8, 2.5 Hz, 1H), 3.66 (dd, *J* = 18.8, 2.4 Hz, 0.14H), 3.00 (s, 3H), 2.99 (s, 0.42H), 2.95 – 2.78 (m, 2.42H), 2.54 (t, *J* = 2.4 Hz, 0.14H), 2.29 (t, *J* = 2.5 Hz, 1H), 1.70 (d, *J* = 6.9 Hz, 3H), 1.66 (d, *J* = 6.4 Hz, 0.42H).

^{13}C NMR (126 MHz, $CDCl_3$) : δ 138.2, 128.8, 128.5, 128.3, 127.2, 126.5, 79.8, 73.2, 61.4, 41.9, 33.0, 29.5, 13.4.

HRMS ESI (m/z): $[M+H]^+$ calcd for $C_{15}H_{20}NO_2S$, 278.1215; found, 278.1210.

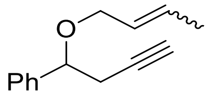

Prepared analogy to Supplementary Eq. (2): crotyl bromide was used instead of allylic bromide in last step to afford the enyne as a 3.6:1mixture of EZ isomer with 68% yield for 2 steps.

^{1}H NMR (400 MHz, $CDCl_3$) : δ 7.46 – 7.28 (m, 5H), 5.80 – 5.51 (m, 2H), 4.47 (td, *J* = 6.6, 2.0 Hz, 1H), 4.05 – 3.69 (m, 2H), 2.76 – 2.66 (m, 1H), 2.61 – 2.52 (m, 1H), 1.96 (t, *J* = 2.4 Hz, 1H), 1.70 (d, *J* = 6.0 Hz, 2.45H), 1.55 (d, *J* = 6.4 Hz, 0.67H).

^{13}C NMR (126 MHz, $CDCl_3$) : δ 141.0, 129.8, 128.5, 128.5, 128.4, 128.1, 128.1, 127.5, 126.9, 126.9, 126.7, 81.1, 81.0, 79.4, 79.2, 70.1, 70.1, 69.7, 64.2, 28.3, 28.2, 17.9, 13.3.

HRMS-ESI (m/z): $[M+H]^+$ calcd for $C_{14}H_{17}O$, 201.1279; found, 201.1272.

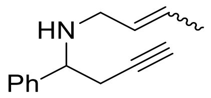

Prepared analogy to Supplementary Eq. (2): started from the N-Ts imine, and crotyl bromide was used to afford the N-Ts enyne firstly, followed by deprotection of N-Ts under Na/naphthalene condition[11] to afford the N-H enyne as a 6:1mixture of EZ isomer with 54% yield for 3 steps.

^{1}H NMR (500 MHz, $CDCl_3$) : δ 7.40 – 7.26 (m, 5H), 5.60 – 5.47 (m, 2H), 3.88 – 3.82 (m, 1H), 3.17 – 2.97 (m, 2H), 2.59 – 2.48 (m, 2H), 2.02 (t, *J* = 2.7 Hz, 1H), 1.67 (d, *J* = 5.0 Hz, 2.55H), 1.52 (d, *J* = 6.0 Hz, 0.45H).

^{13}C NMR (126 MHz, $CDCl_3$) : δ 142.7, 142.6, 129.8, 129.4, 128.7, 128.6, 127.6, 127.6, 127.6, 127.3, 127.2, 126.6, 81.7, 70.6, 61.1, 60.9, 49.4, 43.8, 28.2, 28.1, 17.9, 13.1.

HRMS-ESI (m/z): $[M+H]^+$ calcd for $C_{14}H_{18}N$,200.1434; found, 200.1431.

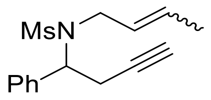

Prepared analogy to Supplementary Eq. (2): started from the N-Ms imine, the homopropargyl amine was prepared firstly, followed by alkylation with crotyl bromide to afford the enyne as a 3.5:1mixture of EZ isomer with 52% yield for 2 steps.

^{1}H NMR (500 MHz, $CDCl_3$) : δ 7.47 – 7.31 (m, 5H), 5.69 – 5.58 (m, 1H), 5.47 – 5.38 (m, 1H), 5.34 – 5.24 (m, 1H), 3.98 (dd, *J* = 16.6, 7.1 Hz, 0.22H), 3.82 (dd, *J* = 15.9, 7.2, 0.78H), 3.66 – 3.55 (m, 1H), 3.07 – 2.97 (m, 2.7H), 2.92 (s, 2.4H), 2.07 – 2.12 (m, 1H), 1.68 (d, *J* = 6.4 Hz, 2.3H), 1.63 (d, *J* = 6.9 Hz, 0.7H).

^{13}C NMR (126 MHz, $CDCl_3$) : δ 137.9, 137.8, 130.3, 128.8, 128.7, 128.4, 128.3, 128.0, 128.0, 127.9, 127.2, 126.8, 81.5, 81.4, 71.6, 71.5, 59.8, 59.7, 47.0, 42.2, 42.1, 41.4, 22.9, 22.8, 17.8, 12.9.

HRMS-ESI (m/z): $[M+H]^+$ calcd for $C_{15}H_{20}NO_2S$, 278.1215; found, 278.1206.

e) Enyne for Set 5a/b, Table 3

The di-substituted propargyl enynes were prepared according to literature procedure.[12] Addition of crotyl bromide to aldehyde or aldimine under Barbier condition afforded a pair of branched diastereomers, followed by alkylation (**Supplementary Fig. 14**). In case of oxa-enyne and N-H enyne, both of the diastereomers could be separated by silica gel column chromatography. The relative configuration was determined by backstepping the relative configuration of their reductive hydroalkenylation product.

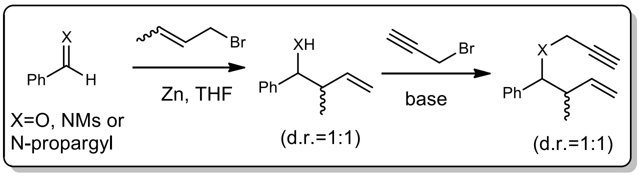

Supplementary Fig. 14

Started from benzaldehyde, the following two enynes were obtained as a 1:1 mixture of diastereomer with 74% yield for 2 steps (**Supplementary Fig. 14**). The 1,2-syn isomer was separated by column chromatography with 1% EtOAc in hexane as eluent.

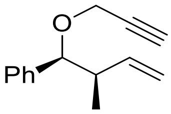

relative configuration

^{1}H NMR (400 MHz, $CDCl_3$) : δ 7.36 – 7.23 (m, 5H), 5.72 – 5.58 (m, 1H), 4.92 (dt, *J* = 6.3, 1.6 Hz, 1H), 4.91 (d, *J* = 1.2 Hz, 1H),4.31 (d, *J* = 7.0 Hz, 1H), 4.13 (dd, *J* = 15.7, 2.4 Hz, 1H), 3.84 (dd, *J* = 15.8, 2.3 Hz, 1H), 2.56 (hept, *J* = 6.8 Hz, 1H), 2.39 (t, *J* = 2.4 Hz, 1H), 1.11 (d, *J* = 6.8 Hz, 3H).

13C NMR (126 MHz, $CDCl_3$) : δ 140.3, 139.6, 128.2, 127.9, 127.8, 114.9, 84.6, 80.2, 74.1, 55.9, 44.2, 15.9.

HRMS ESI (m/z): $[M+H]^+$ calcd for $C_{14}H_{17}O$, 201.1274; found, 201.1271.

Started from N-propargyl imine, the following two enynes were obtain a mixture of 1.2:1 diastereomer with 71% yield for 1 step (**Supplementary Fig. 14**). Both of the diastereomers could be separated by column chromatography with hexane as eluent.

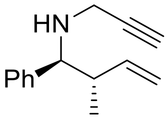

relative configuration

^{1}H NMR (400 MHz, $CDCl_3$) : δ 7.43 – 7.14 (m, 5H), 5.79 – 5.54 (m, 1H), 5.30 – 5.02 (m, 2H), 3.54 (dd, *J* = 9.2, 2.8 Hz, 1H), 3.31 (dt, *J* = 17.3, 2.8 Hz, 1H), 2.95 (dt, *J* = 17.3, 2.7 Hz, 1H), 2.44 – 2.31 (m, 1H), 2.18 (t, *J* = 2.7 Hz, 1H), 1.89 (s, 1H), 0.77 (dd, *J* = 6.8, 2.8 Hz, 3H).

^{13}C NMR (101 MHz, $CDCl_3$) : δ 142.2, 141.3, 128.6, 128.4, 127.5, 116.6, 82.5, 71.3, 65.6, 45.6, 35.8, 18.1.

HRMS ESI (m/z): $[M+H]^+$ calcd for $C_{14}H_{18}N$, 200.1434; found, 200.1430.

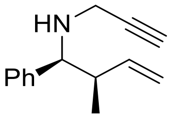

relative configuration

^{1}H NMR (500 MHz, $CDCl_3$) : δ 7.39 – 7.21 (m, 5H), 5.86 – 5.66 (m, 1H), 5.06 – 4.94 (m, 2H), 3.85 (d, *J* = 5.3 Hz, 1H), 3.38 (dd, *J* = 17.1, 2.5 Hz, 1H), 3.08 (dd, *J* = 17.1, 2.4 Hz, 1H), 2.58 – 2.46 (m, 1H), 2.18 (t, *J* = 2.4 Hz, 1H), 1.64 (s, 1H), 0.97 (d, *J* = 6.9 Hz, 3H).

^{13}C NMR (126 MHz, $CDCl_3$) : δ 140.9, 140.8, 128.4, 128.1, 127.2, 115.2, 82.4, 71.3, 65.2, 43.5, 36.0, 15.3.

HRMS ESI (m/z): $[M+H]^+$ calcd for $C_{14}H_{18}N$, 200.1434; found, 200.1429.

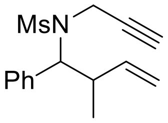

Started from N-Ms aldimine, the enyne was obtained as a 1.2:1 mixture of diastereomer with 67% yield for 2 steps (Supplementary Fig. 14).

^{1}H NMR (500 MHz, $CDCl_3$) : δ 7.48 – 7.28 (m, 12.5H), 5.94 (ddd, *J* = 17.2, 10.2, 8.4 Hz, 1.5H), 5.50 (ddd, *J* = 17.6, 10.4, 7.6 Hz, 1H), 5.23 (d, *J* = 16.9 Hz, 1.5H), 5.14 (d, *J* = 9.5 Hz, 1.5H), 5.00 (d, *J* = 17.2 Hz, 1H), 4.89 (d, *J* = 10.5 Hz, 1H), 4.75 (d, *J* = 11.3 Hz, 2.5H), 4.10 – 3.96 (m, 5H), 3.21 – 3.12 (m, 1.0H), 3.12 – 3.03 (m, 1.5H), 2.83 (s, 3H), 2.78 (s, 4.5H), 2.20 (t, *J* = 2.5 Hz, 1H), 2.16 (t, *J* = 2.5 Hz, 1.5H), 1.29 (d, *J* = 6.6 Hz, 3H), 0.88 (d, *J* = 6.6 Hz, 4.5H).

^{13}C NMR (126 MHz, $CDCl_3$) : δ 141.3, 140.3, 137.1, 129.5, 129.2, 128.8, 128.6, 128.5, 128.4, 116.1, 116.0, 79.4, 79.1, 73.4, 73.4, 66.9, 66.6, 41.7, 41.4, 39.6, 38.9, 33.7, 33.6, 18.9, 18.8.

HRMS ESI (m/z): $[M+NH_4]^+$ calcd for $C_{15}H_{23}N_2O_2S$, 295.1480; found, 295.1472.

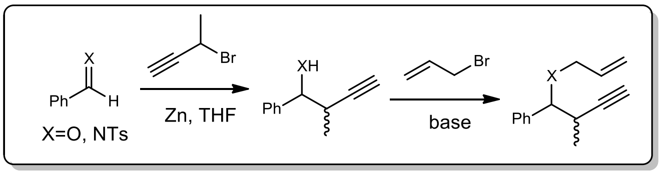

Supplementary Fig. 15

Started from benzaldehyde, the following two enynes were obtained as a 1:1 mixture of diastereomer with 72% yield for 2 steps (**Supplementary Fig. 15**). The 1,2-syn isomer was separated by silica gel chromatography with 1% EtOAc in hexane as eluent.

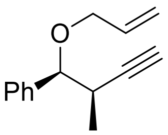

relative configuration

^{1}H NMR (500 MHz, $CDCl_3$) : δ 7.38 – 7.26 (m, 5H), 5.96 – 5.86 (m, 1H), 5.24 (dd, *J* = 17.2, 1.7 Hz, 1H), 5.16 (dd, *J* = 10.4, 1.6 Hz, 1H), 4.21 (d, *J* = 7.1 Hz, 1H), 3.96 (dd, *J* = 13.0, 5.0 Hz, 1H), 3.78 (dd, *J* = 13.0, 6.1 Hz, 1H), 2.82 (pd, *J* = 6.9, 2.3 Hz, 1H), 1.98 (d, *J* = 2.4 Hz, 1H), 1.27 (d, *J* = 6.9 Hz, 3H).
^{13}C NMR (101 MHz, $CDCl_3$) : δ 139.4, 134.8, 128.2, 128.0, 127.6, 117.0, 86.3, 83.4, 70.0, 69.8, 33.2, 17.2.
HRMS ESI (m/z): $[M+H]^+$ calcd for $C_{14}H_{17}O$, 201.1274; found, 201.1267.

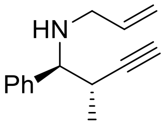

relative configuration

Started from N-Ts aldimine, the enyne was obtained as a single diastereomer (d.r.>19:1 by NMR), with 35% yield for 3 steps (Supplementary Figure 15).
^{1}H NMR (500 MHz, $CDCl_3$) : δ 7.36 – 7.24 (m, 5H), 5.93 – 5.77 (m, 1H), 5.18 – 4.97 (m, 2H), 3.53 (d, *J* = 8.6 Hz, 1H), 3.09 (dd, *J* = 14.3, 5.1 Hz, 1H), 2.96 (dd, *J* = 14.3, 6.9 Hz, 1H), 2.74 – 2.62 (m, 1H), 2.20 (d, *J* = 2.4 Hz, 1H), 2.05 – 1.92 (br, 1H), 1.00 (d, *J* = 7.0 Hz, 3H).
^{13}C NMR (126 MHz, $CDCl_3$) : δ 141.4, 137.0, 128.5, 128.2, 127.7, 115.9, 86.9, 70.8, 66.9, 50.0, 33.7, 18.3.
HRMS-ESI (m/z): $[M+H]^+$ calcd for $C_{14}H_{18}N$, 200.1434; found, 200.1432.

To obtain another diastereomer of allylic aza-enyne, method based on Mitsunobu reaction was developed, as shown in **Supplementary Fig. 16**. The enyne was obtained as a diastereomer mixture, and could be separated by silica gel column chromatography.

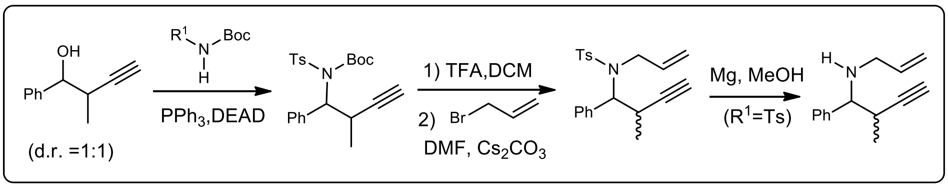

Supplementary Fig. 16

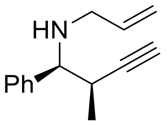

Prepared according to Supplementary Fig. 16: started from the 1:1 diastereomers mixture of homo-propargyl alcohol, the enyne was obtained as a mixture of diastereomer with 40% yield for 4 steps. The 1,2-syn diastereomer was obtained by column chromatography with 10% EtOAc/hexane as eluent.

^{1}H NMR (500 MHz, $CDCl_3$) : δ 7.37 – 7.27 (m, 5H), 5.88 (ddt, *J* = 16.5, 10.2, 6.0 Hz, 1H), 5.12(dd, *J* = 17.2, 1.8 Hz, 1H), 5.07 (dd, *J* = 10.2, 1.6 Hz, 1H), 3.71 (d, *J* = 5.2 Hz, 1H), 3.17 (dd, *J* = 14.2, 5.5 Hz, 1H), 3.04 (dd, *J* = 14.1, 6.5 Hz, 1H), 2.95 – 2.89 (m, 1H), 2.11 (d, *J* = 2.5 Hz, 1H), 1.06 (d, *J* = 7.1 Hz, 3H).

^{13}C NMR (126 MHz, $CDCl_3$) : δ 140.5, 136.9, 128.3, 128.2, 127.5, 116.1, 86.4, 71.0, 64.9, 50.1, 32.7, 17.3.

HRMS-ESI (m/z): $[M+H]^+$ calcd for $C_{14}H_{18}N$, 200.1434; found, 200.1429.

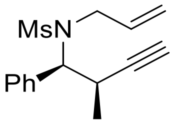

Prepared according to Supplementary Fig. 16: started from the 1:1 diastereomers mixture of homo-propargyl alcohol, the enyne was obtained as a mixture of diastereomer with 37% yield for 4 steps. The 1,2-syn diastereomer could be obtained by column chromatography with 2% EtOAc in hexane as eluent.

^{1}H NMR (400 MHz, $CDCl_3$) : δ 7.49 – 7.33 (m, 5H), 5.65 – 5.53 (m, 1H), 5.27 – 5.20 (dd, *J* = 17.1, 1.3 Hz, 1H), 5.18 – 5.13 (dd, *J* =10.1, 1.3Hz, 1H), 4.83 – 4.79 (d, *J* = 10.7 Hz, 1H), 3.81 – 3.76 (d, *J* = 6.6Hz, 2H), 3.48 – 3.38 (m, 1H), 2.65 – 2.61 (s, 3H), 1.96 – 1.94 (d, *J* = 2.4 Hz, 1H), 1.45 – 1.40 (d, *J* = 6.8 Hz, 3H).

^{13}C NMR (500 MHz, $CDCl_3$) : δ 137.1, 134.4, 129.2, 128.8, 128.6, 119.4, 85.8, 71.6, 65.9, 48.4, 40.9, 28.2, 19.0.

HRMS-ESI(m/z):$[M+NH_4]^+$ calcd for $C_{15}H_{23}O_2N_2S$, 295.1480; found, 295.1468.

a) Enynes for Set 5a/b, Table 3

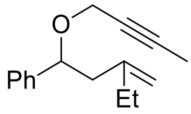

Prepared analogously to Supplementary Eq. (1): started from benzaldehyde, the 2-(bromomethyl)but-1-ene and 1-bromobut-2-yne were employed in first and second step respectively. The enyne was obtained as a yellow oil with 64% yield for 2 steps.

^{1}H NMR (500 MHz, $CDCl_3$) : δ 7.37 – 7.26 (m, 5H), 4.78 (d, *J* = 4.0 Hz, 2H), 4.63 (dd, *J* = 8.0, 5.7 Hz, 1H), 4.05 (dq, *J* = 15.3, 2.4 Hz, 1H), 3.81 (dq, *J* = 15.1, 2.4 Hz, 1H), 2.62 (dd, *J* = 14.5, 8.1 Hz, 1H), 2.36 (dd, *J* = 14.6, 5.8 Hz, 1H), 2.03 (q, *J* = 7.5 Hz, 2H), 1.84 (t, *J* = 2.4 Hz, 3H), 0.99 (t, *J* = 7.4 Hz, 3H).

^{13}C NMR (126 MHz, $CDCl_3$) : δ 147.6, 141.5, 128.4, 127.7, 127.0, 110.3, 82.1, 79.2, 75.3, 56.2, 44.7, 29.0, 12.2, 3.7.

HRMS ESI (m/z): $[M+H]^+$ calcd for $C_{16}H_{21}O$, 229.15869; found, 229.15782.

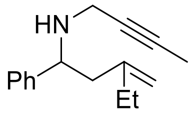

Prepared analogously to Supplementary Eq. (1): started from N-Ts aldimine, 2-(bromomethyl)but-1-ene and 1-bromobut-2-yne were employed in first and second step respectively. After deprotection of N-Ts group under Mg/MeOH condition, the enyne was obtained as yellow oil with 53% yield for 3 steps.

^{1}H NMR (400 MHz, $CDCl_3$) : δ 7.50 – 7.31 (m, 5H), 4.98 – 4.84 (m, 2H), 3.98 (dd, *J* = 8.4, 5.8 Hz, 1H) 3.31 (dq, *J* = 16.8, 2.4 Hz, 1H), 3.03 (dq, *J* = 16.6, 2.4 Hz, 1H), 2.46 – 2.33 (m, 2H), 2.21 – 2.02 (m, 2H), 1.84 (t, *J* = 2.4 Hz, 3H), 1.73 (d, *J* = 7.2 Hz, 1H), 1.09 (t, *J* = 7.4 Hz, 3H).

^{13}C NMR (126 MHz, $CDCl_3$) : δ 148.1, 143.7, 128.5, 127.5, 127.2, 111.4, 78.9, 76.9, 58.6, 45.8, 36.4, 28.3, 12.4, 3.6.

HRMS ESI (m/z): $[M+H]^+$ calcd for $C_{16}H_{22}N$, 228.1752; found, 228.1746..

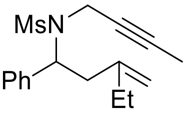

Prepared analogously to Supplementary Eq. (1): started from N-Ms aldimine, 2-(bromomethyl)but-1-ene and 1-bromobut-2-yne were employed in first and second step respectively to afford the enyne as a yellow oil with 54% yield for 2 steps.

^{1}H NMR (500 MHz, $CDCl_3$) : δ 7.50 – 7.28 (m, 5H), 5.33 (t, *J* = 7.7 Hz, 1H), 4.93 – 4.86 (m, 2H), 3.99 (dq, *J* = 18.6, 2.6 Hz, 1H), 3.70 (dq, *J* = 18.6, 2.5 Hz, 1H), 2.90 (s, 3H), 2.92 – 2.78 (m, 2H), 2.22–2.05 (m, 2H), 1.77 (t, *J* = 2.5 Hz, 3H), 1.06 (t, *J* = 7.4 Hz, 3H).

^{13}C NMR (126 MHz, $CDCl_3$) : δ 147.2, 138.6, 128.6, 128.6, 128.2, 112.1, 81.0, 75.2, 59.3, 41.9, 38.5, 33.5, 28.5, 12.3, 3.6.

HRMS ESI (m/z): $[M+Na]^+$ calcd for $C_{17}H_{23}NNaO_2S$, 328.1347; found, 328.1339.

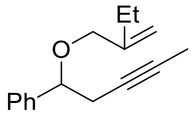

Prepared analogously to Supplementary Eq. (2): the internal homo propargyl alcohol was prepared firstly, and then treated with NaH and 2-(bromomethyl)but-1-ene, to afford the enyne as a yellow oil (47% yield for 3 steps).

^{1}H NMR (500 MHz, $CDCl_3$) : δ 7.47 – 7.30 (m, 5H), 5.01 (s, 1H), 4.92 (s, 1H), 4.42 (t, *J* = 6.7 Hz, 1H), 3.95 (d, *J* = 12.5 Hz, 1H), 3.75 (d, *J* = 12.7 Hz, 1H), 2.72 – 2.62 (m, 1H), 2.55 – 2.43 (m, 1H), 2.13 (dp, *J* = 23.2, 7.6 Hz, 2H), 1.77 (t, *J* = 2.6 Hz, 3H), 1.06 (t, *J* = 7.5 Hz, 3H).

^{13}C NMR (126 MHz, CD_2Cl_2) : δ 148.2, 141.5, 128.3, 127.8, 126.9, 109.9, 79.7, 77.1, 75.7, 71.6, 28.3, 25.9, 11.9, 3.2.

HRMS ESI (m/z): $[M+H]^+$ calcd for $C_{16}H_{21}O$, 229.1592; found, 229.1586.

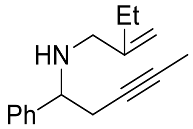

Prepared by deprotection of N-Ts group of corresponding N-Ts enyne to afford the enyne as a yellow oil with 81% yield for 1 step.

^{1}H NMR (500 MHz, $CDCl_3$) : δ 7.46 – 7.25 (m, 5H), 4.92 (s, 1H), 4.86 (s, 1H), 3.78 (dd, *J* = 8.1, 5.3 Hz, 1H), 3.11 (d, *J* = 14.2 Hz, 1H), 3.02 (d, *J* = 14.3 Hz, 1H), 2.59 – 2.41 (m, 2H), 2.18 – 2.01 (m, 2H), 1.82 (t, *J* = 2.6 Hz, 3H), 1.04 (t, *J* = 7.5 Hz, 3H).

^{13}C NMR (126 MHz, $CDCl_3$) : δ 149.7, 143.4, 128.5, 127.4, 127.3, 108.8, 77.9, 76.6, 61.1, 52.2, 28.8, 27.2, 12.3, 3.7.

HRMS ESI (m/z): $[M+H]^+$ calcd for $C_{16}H_{22}N$, 228.1752; found, 228.1747.

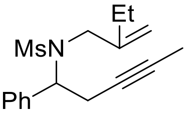

Prepared by treating corresponding N-H enyne with $MsCl/Et_3N$ in DCM to afford the enyne as yellow oil with 78% yield.

^{1}H NMR (400 MHz, $CDCl_3$) : δ 7.50 – 7.31 (m, 5H), 5.16 – 5.04 (m, 2H), 4.95 (s, 1H), 3.77 (d, *J* = 15.9 Hz, 1H), 3.65 (d, *J* = 15.8 Hz, 1H), 3.06 – 2.89 (m, 2H), 2.85 (s, 3H), 2.13 – 1.95 (m, 2H), 1.75 (s, 3H), 1.00 (t, *J* = 7.4 Hz, 3H).

^{13}C NMR (126 MHz, $CDCl_3$) : δ 147.2, 137.8, 128.6, 128.5, 128.3, 112.6, 78.5, 76.6, 61.0, 50.8, 40.9, 25.9, 22.9, 11.9, 3.7.

HRMS ESI (m/z): $[M+H]^+$ calcd for $C_{17}H_{24}NO_2S$, 306.1528; found, 306.1522.

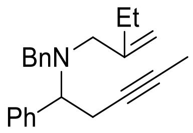

Prepared by treating corresponding N-H enyne with BnBr/K_2CO_3 in DMF to afford the enyne as a yellow oil with 83% yield for 1 step.

^{1}H NMR (500 MHz, $CDCl_3$) : δ 7.41 – 7.26 (m, 9H), 7.25 – 7.21 (m, 1H), 4.99 (s, 1H), 4.87 (s, 1H), 3.98 (t, *J* = 7.1 Hz, 1H), 3.72 (d, *J* = 13.9 Hz, 1H), 3.24 (d, *J* = 13.9 Hz, 1H), 3.10 (d, *J* = 13.6 Hz, 1H), 2.86 – 2.78 (m, 1H), 2.76 (d, *J* = 13.4 Hz, 1H), 2.65 – 2.57 (m, 1H), 2.19 – 2.08 (m, 2H), 1.73 (td, *J* = 2.6, 1.2 Hz, 3H), 0.92 (td, *J* = 7.4, 1.3 Hz, 3H).

^{13}C NMR (126 MHz, $CDCl_3$) : δ 149.6, 140.4, 139.1, 129.0, 128.8, 128.3, 128.0, 127.1, 126.9, 111.4, 77.5, 77.4, 61.1, 55.1, 53.6, 26.6, 20.2, 12.2, 3.7.

HRMS ESI (m/z): $[M+H]^+$ calcd for $C_{23}H_{28}N$, 318.2221; found, 318.2216.

b) Enyne for Set 6, Table 2

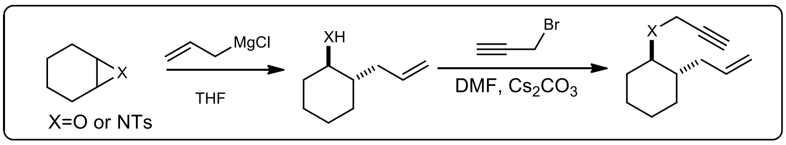

Supplementary Fig. 17

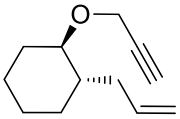

2-allylcyclohexanol was prepared according to literature procedure firstly,[13] then treated with 2equiv. NaH and propargyl bromide. The enyne was obtained as a yellow oil with 71% yield for 2 steps.

^{1}H NMR (500 MHz, $CDCl_3$) : δ 5.80 (dddd, J = 16.8, 10.2, 8.2, 6.3 Hz, 1H), 5.07 – 4.95 (m, 2H), 4.22 (dd, J = 15.8, 2.4 Hz, 1H), 4.14 (dd, J = 15.8, 2.4 Hz, 1H), 3.14 – 3.04 (m, 1H), 2.58 – 2.49 (m, 1H), 2.39 (t, J = 2.4 Hz, 1H), 2.18 – 2.05 (m, 1H), 1.96 – 1.87 (m, 1H), 1.86 – 1.72 (m, 2H), 1.66 – 1.60 (m, 1H), 1.46 – 1.37 (m, 1H), 1.28 – 1.11 (m, 3H), 1.00 – 0.90 (m, 1H).

^{13}C NMR (126 MHz, $CDCl_3$) : δ 137.4, 116.0, 80.9, 80.8, 73.7, 55.8, 42.8, 36.8, 30.9, 30.3, 25.4, 24.8.

HRMS-ESI (m/z): $[M+H]^+$ calcd for $C_{12}H_{19}O$, 179.1430; found, 179.1428.

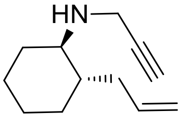

Prepared according to **Supplementary Fig. 17**: started from 7-tosyl-7-azabicyclo-[4.1.0] heptane, the N-Ts enyne was prepared firstly, then N-Ts was deprotected under Mg/MeOH conditions. The total yield was 54% after 3 steps to give the enyne as a yellow oil.

^{1}H NMR (500 MHz, $CDCl_3$) : δ 5.92 – 5.73 (m, 1H), 5.09 – 5.01 (m, 2H), 3.50 (dd, J = 17.1, 2.4 Hz, 1H), 3.42 (dd, J = 17.1, 2.4 Hz, 1H), 2.43 – 2.35 (m, 2H), 2.21 (t, J = 2.4 Hz, 1H), 2.05 – 1.94 (m, 2H), 1.82 – 1.60 (m, 4H), 1.37 – 1.19 (m, 3H), 1.11 – 1.00 (m, 2H).

^{13}C NMR (126 MHz, $CDCl_3$) : δ 137.2, 116.2, 82.8, 71.0, 59.1, 42.4, 37.5, 35.6, 31.7, 30.9, 25.7, 25.1.

HRMS-ESI (m/z): $[M+H]^+$ calcd for $C_{12}H_{20}N$,178.1596; found, 178.1589.

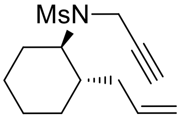

The enyne was obtained by treating the corresponding NH enyne with $MsCl/Et_3N$ in DCM with 73% yield in 1 step.

^{1}H NMR (500 MHz, $CDCl_3$) : δ 5.75 (dddd, J = 16.8, 10.4, 8.0, 6.4 Hz, 1H), 5.10 – 4.94 (m, 2H), 4.15 (dd, J = 18.7, 2.5 Hz, 1H), 3.88 (d, J = 18.1 Hz, 1H), 3.53-3.38 (m, 1H), 3.04 (s, 3H), 2.51 – 2.41 (m, 1H), 2.34 (t, J = 2.5 Hz, 1H), 2.00 – 1.88 (m, 2H), 1.87 – 1.76 (m, 2H), 1.74 – 1.63 (m, 2H), 1.57 – 1.42 (m, 1H), 1.38 – 1.25 (m, 1H), 1.22 – 1.09 (m, 1H), 1.05 – 0.93 (m, 1H).

^{13}C NMR (126 MHz, $CDCl_3$) : δ 136.6, 116.5, 80.1, 73.0, 62.4, 41.5, 40.2, 37.4, 31.9, 31.6, 26.1, 25.6.

HRMS-ESI (m/z): $[M+H]^+$ calcd for $C_{13}H_{22}NO_2S$, 256.1366; found, 256.1359.

Product analysis for reductive hydroalkenylation

Table 1

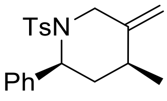

relative configuration

4'g

Supplementary Method A was followed.

^{1}H NMR (500 MHz, $CDCl_3$) : δ 7.66 – 7.54 (m, 2H), 7.32 – 7.16 (m, 7H), 4.91 (d, *J* = 2.0 Hz, 1H), 4.78 (d, *J* = 2.0 Hz, 1H), 4.71 (dd, *J* = 10.5, 6.4 Hz, 1H), 4.31 (d, *J* = 15.1 Hz, 1H), 4.11 (d, *J* = 15.1 Hz, 1H), 2.40 (s, 3H), 1.97 (ddd, *J* = 13.9, 6.6, 4.0 Hz, 1H), 1.91 – 1.80 (m, 1H), 1.57 (dd, *J* = 24.3, 11.9 Hz, 1H), 0.97 (d, *J* = 6.7 Hz, 3H).

^{13}C NMR (126 MHz, $CDCl_3$) : δ 146.9, 143.1, 142.6, 136.7, 129.4, 128.5, 127.7, 127.2, 126.2, 108.6, 58.8, 48.4, 39.5, 32.6, 21.7, 18.7.

HRMS ESI (m/z): $[M+H]^+$ calcd for $C_{20}H_{24}NO_2S$, 342.1528; found, 342.1519.

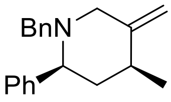

relative configuration

3i

Supplementary Method A was followed.

^{1}H NMR (400 MHz, $CDCl_3$) : δ 7.53 – 7.44 (m, 2H), 7.41 – 7.18 (m, 8H), 4.76 (s, 1H), 4.73 (s, 1H), 3.82 (dd, *J* = 13.5, 2.8 Hz, 1H), 3.40 (td, *J* = 12.0, 3.1 Hz, 2H), 2.90 (dd, *J* = 13.5, 2.9 Hz, 1H), 2.65 (d, *J* = 12.5 Hz, 1H), 2.35 – 2.21 (m, 1H), 1.97 – 1.84 (m, 1H), 1.47 (q, *J* = 12.2, 11.6 Hz, 1H), 1.10 (d, *J* = 6.5 Hz, 3H).

^{13}C NMR (101 MHz, $CDCl_3$) : δ 149.0, 144.5, 139.3, 128.8, 128.7, 128.2, 127.6, 127.2, 126.8, 106.5, 69.1, 60.1, 59.6, 45.7, 36.1, 17.5.

HRMS ESI (m/z): $[M+H]^+$ calcd for $C_{20}H_{24}N$, 278.1909; found, 278.1900.

Table 3, Set 1a

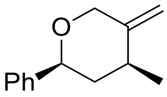

relative configuration

3a

Supplementary Method A was followed.

^{1}H NMR (500 MHz, $CDCl_3$) : δ 7.38 – 7.26 (m, 5H), 4.95 (d, J = 2.0 Hz, 1H), 4.85 (t, J = 1.6 Hz, 1H), 4.55 (dd, J = 11.4, 2.2 Hz, 1H), 4.37 (d, J = 12.2 Hz, 1H), 4.15 (d, J = 12.3 Hz, 1H), 2.52 – 2.44 (m, 1H), 2.00 (ddd, J = 13.1, 4.6, 2.2 Hz, 1H), 1.43 (q, J = 12.3 Hz, 1H), 1.15 (d, J = 6.5 Hz, 3H).

^{13}C NMR (126 MHz, $CDCl_3$) : δ 148.3, 142.4, 128.5, 127.6, 126.0, 107.6, 80.3, 73.9, 44.3, 35.2, 17.7.

HRMS ESI (m/z): $[M+H]^+$ calcd for $C_{13}H_{17}O$, 189.1274; found, 189.1270.

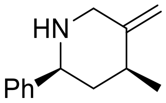

relative configuration

3c

Supplementary Method A was followed. The 1,3-syn:anti ratio was determined by GC-MS after mesylation of crude product.

^{1}H NMR (500 MHz, $CDCl_3$) : δ 7.38 –7.22 (m, 5H), 4.89 (d, J = 1.7 Hz, 1H), 4.77 (d, J = 1.9 Hz, 1H), 3.85 (dd, J = 11.4, 2.6 Hz, 1H), 3.61 (d, J = 12.4 Hz, 1H), 3.43 (d, J = 12.5 Hz, 1H), 2.34 (dqd, J = 8.7, 6.5, 4.6 Hz, 1H), 1.98 (ddd, J = 12.7, 4.5, 2.7 Hz, 1H), 1.31 (q, J = 12.2 Hz, 1H), 1.12 (d, J = 6.5 Hz, 3H).

^{13}C NMR (126 MHz, $CDCl_3$) : δ 149.8, 144.3, 128.5, 127.3, 126.7, 106.4, 62.1, 54.9, 45.1, 36.3, 18.1.

HRMS ESI (m/z): $[M+H]^+$ calcd for $C_{13}H_{18}N$, 188.1434; found, 188.1433.

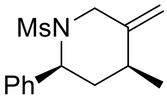

relative configuration

4e

The typical mesylation procedure was followed.

^{1}H NMR (500 MHz, $CDCl_3$) : δ 7.39 –7.25 (m, 5H), 5.02 (d, J = 2.2 Hz, 1H), 4.97 (d, J = 1.9 Hz, 1H), 4.83 (dd, J = 10.7, 6.3 Hz, 1H), 4.26 (d, J = 16.0 Hz, 1H), 4.11 (m, 1H), 2.65 (s, 3H), 2.53 – 2.45 (m, 1H), 2.18 – 2.10 (m, 1H), 1.79 – 1.69 (m, 1H), 1.14 (d, J = 6.8 Hz, 3H).

^{13}C NMR (126 MHz, $CDCl_3$) : δ 147.0, 142.4, 128.8, 127.6, 126.3, 109.3, 58.9, 47.7, 39.5, 39.2, 33.0, 19.1.

HRMS ESI (m/z): $[M+H]^+$ calcd for $C_{14}H_{20}NSO_2$, 226.1209; found, 226.1207.

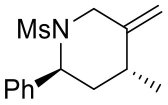 relative configuration

4'e

Supplementary Method A was followed. The 1,3-syn:anti ratio was determined by GC-MS.

^{1}H NMR (500 MHz, $CDCl_3$) : δ 7.51 –7.24 (m, 5H), 5.27 (d, *J* = 5.5 Hz, 1H), 4.98 (d, *J* = 1.6 Hz, 1H), 4.83 (d, *J* = 1.3 Hz, 1H), 4.19 (dd, *J* = 15.2, 1.3 Hz, 1H), 3.62 (d, *J* = 15.0 Hz, 1H), 2.92 (s, 3H), 2.49 (ddd, *J* = 13.9, 4.1, 2.0 Hz, 1H), 2.41 – 2.32 (m, 1H), 1.73 (ddd, *J* = 13.8, 12.6, 5.6 Hz, 1H), 1.14 (d, *J* = 6.4 Hz, 3H).

^{13}C NMR (126 MHz, $CDCl_3$) : δ 146.6, 138.5, 129.0, 127.4, 126.9, 108.8, 55.6, 48.7, 41.1, 37.1, 30.9, 18.0.

HRMS ESI (m/z): $[M+H]^+$ calcd for $C_{14}H_{20}NSO_2$, 226.1209; found, 226.1206.

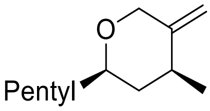 relative configuration

Supplementary Method A was followed.

^{1}H NMR (400 MHz, $CDCl_3$) : δ 4.85 (d, *J* = 1.7 Hz, 1H), 4.77 (t, *J* = 1.8 Hz, 1H), 4.20 (d, *J* = 12.1 Hz, 1H), 3.94 (d, *J* = 12.1 Hz, 1H), 3.50 – 3.41 (m, 1H), 2.32 – 2.22 (m, 1H), 1.75 (ddd, *J* = 12.9, 4.6, 2.0 Hz, 1H), 1.58 – 1.22 (m, 8H), 1.12 – 1.00 (m, 4H), 0.88 (t, *J* = 6.9 Hz, 3H).

^{13}C NMR (126 MHz, $CDCl_3$) : δ 149.0, 107.0, 78.0, 73.5, 42.4, 36.2, 34.7, 32.1, 25.5, 22.8, 17.8, 14.2.

HRMS ESI (m/z): $[M+H]^+$ calcd for $C_{12}H_{23}O$, 183.1749; found, 183.1742.

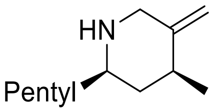 relative configuration

Supplementary Method A was followed.

^{1}H NMR (500 MHz, $CDCl_3$) : δ 4.83 (s, 1H), 4.80 (s, 1H), 3.60 (d, *J* = 13.0 Hz, 1H), 3.32 (d, *J* = 13.0 Hz, 1H), 2.89 – 2.79 (m, 1H), 2.18 (dt, *J* = 13.4, 6.6 Hz, 1H), 1.90 (ddd, *J* = 13.2, 4.4, 2.7 Hz, 1H), 1.63 – 1.54 (m, 1H), 1.50 – 1.23 (m, 7H), 1.10 (d, *J* = 6.5 Hz, 3H), 1.01 (q, *J* = 12.3 Hz, 1H), 0.89 (t, *J* = 6.9 Hz, 3H).

^{13}C NMR (126 MHz, $CDCl_3$) : δ 147.6, 108.5, 57.1, 53.0, 41.6, 35.5, 35.2, 31.9, 25.7, 22.7, 17.9, 14.2.

HRMS ESI (m/z): $[M+H]^+$ calcd for $C_{12}H_{23}N$, 182.1908; found, 182.1901.

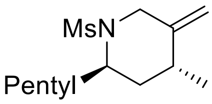

relative configuration

Supplementary Method A was followed.

^{1}H NMR (500 MHz, $CDCl_3$) : δ 4.96 (s, 1H), 4.83 (s, 1H), 4.13 (d, *J* = 15.3 Hz, 1H), 4.02 – 3.95 (m, 1H), 3.69 (d, *J* = 15.2 Hz, 1H), 2.80 (s, 3H), 2.42 (dt, *J* = 12.6, 6.1 Hz, 1H), 1.86 (ddt, *J* = 14.1, 9.5, 4.7 Hz, 1H), 1.71 (dd, *J* = 13.4, 4.5 Hz, 1H), 1.56 – 1.28 (m, 8H), 1.09 (d, *J* = 6.5 Hz, 3H), 0.90 (t, *J* = 6.8 Hz, 3H).

^{13}C NMR (126 MHz, $CDCl_3$) : δ 146.8, 108.8, 53.7, 47.7, 41.0, 38.5, 31.7, 30.8, 30.5, 26.3, 22.7, 18.0, 14.2.

HRMS ESI (m/z): $[M+H]^+$ calcd for $C_{13}H_{26}NSO_2$, 260.1684; found, 260.1675.

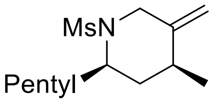

relative configuration

The typical mesylation procedure was followed.

^{1}H NMR (500 MHz, $CDCl_3$) : δ 4.95 (s, 1H), 4.91 (s, 1H), 4.09 (d, *J* = 17.1 Hz, 1H), 3.86 (dq, *J* = 16.5, 1.8 Hz, 1H), 3.73 (p, *J* = 7.2, 6.7 Hz, 1H), 2.82 (s, 3H), 2.36 – 2.24 (m, 1H), 1.95 (ddd, *J* = 13.9, 6.9, 4.6 Hz, 1H), 1.77 – 1.69 (m, 1H), 1.38 – 1.24 (m, 8H), 1.16 (d, *J* = 6.9 Hz, 3H), 0.92 – 0.85 (m, 3H).

^{13}C NMR (126 MHz, $CDCl_3$) : δ 147.3, 108.9, 55.4, 46.3, 38.9, 36.4, 35.8, 32.3, 31.8, 25.4, 22.7, 19.4, 14.2.

HRMS ESI (m/z): $[M+H]^+$ calcd for $C_{13}H_{26}NSO_2$, 260.1684; found, 260.1677.

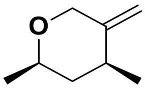

relative configuration

3b

Supplementary Method A was followed, except that the crude reaction mixture was directly sent to analysis and silica gel chromatography without remove of toluene under vacuum.

^{1}H NMR (500 MHz, $CDCl_3$) : δ 4.86 (d, *J* = 1.5 Hz, 1H), 4.77 (t, *J* = 1.8 Hz, 1H), 4.17 (d, *J* = 12.2 Hz, 1H), 3.95 (dt, *J* = 12.1, 1.2 Hz, 1H), 3.63 (dqd, *J* = 12.4, 6.2, 2.1 Hz, 1H), 2.33 – 2.24 (m, 1H), 1.75 (ddd, *J* = 13.0, 4.7, 2.1 Hz, 1H), 1.18 (d, *J* = 6.3 Hz, 3H), 1.11 – 1.03 (m, 4H).

^{13}C NMR (126 MHz, $CDCl_3$) : δ 148.6, 107.2, 74.0, 73.4, 44.1, 34.7, 21.7, 17.8.

HRMS ESI (m/z): $[M+H]^+$ calcd for $C_8H_{15}O$, 127.1123; found, 127.1118.

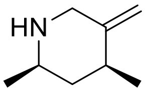

relative configuration

3d

Supplementary Method A was followed. The yield and 1,3-syn:anti ratio was determined by NMR after mesylation of the crude product.

^{1}H NMR (500 MHz, $CDCl_3$) : δ 4.77 (d, *J* = 1.8 Hz, 1H), 4.66 (s, 1H), 3.42 (d, *J* = 13.0 Hz, 1H), 3.23 (d, *J* = 13.0 Hz, 1H), 2.86 – 2.79 (m, 1H), 2.22 – 2.13 (m, 1H), 1.80 (ddd, *J* = 12.8, 4.6, 2.6 Hz, 1H), 1.77 – 1.72 (br,1H), 1.05 (dd, *J* = 6.4, 4.4 Hz, 6H), 0.89 – 0.78 (m, 1H).

^{13}C NMR (126 MHz, $CDCl_3$) : δ 150.8, 105.7, 54.4, 52.6, 45.9, 35.7, 22.5, 18.2.

HRMS ESI (m/z): $[M+H]^+$ calcd for $C_8H_{16}N$, 126.1283; found, 126.1276.

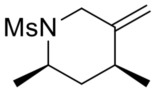

relative configuration

4d

The typical mesylation procedure was followed.

^{1}H NMR (400 MHz, $CDCl_3$) : δ 4.97 (s, 1H), 4.91 (s, 1H), 3.99 – 3.93 (m, 2H), 3.81 – 3.71 (m, 1H), 2.81 (s, 3H), 2.34 – 2.23 (m, 1H), 1.89 (ddd, *J* = 13.8, 6.1, 4.5 Hz, 1H), 1.33 (dd, *J* = 6.4, 1.3 Hz, 3H), 1.28 – 1.25 (m, 1H), 1.15 (dd, *J* = 6.7, 1.2 Hz, 3H).

^{13}C NMR (101 MHz, $CDCl_3$) : δ 147.0, 109.2, 52.1, 47.1, 39.0, 38.6, 32.9, 22.3, 19.2.

HRMS ESI (m/z): $[M+H]^+$ calcd for $C_9H_{18}NSO_2$, 204.1058; found, 204.1051.

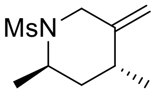

relative configuration

4'd

Supplementary Method A was followed.

^{1}H NMR (500 MHz, $CDCl_3$) : δ 4.96 (d, *J* = 1.5 Hz, 1H), 4.81 (d, *J* = 1.5 Hz, 1H), 4.29 – 4.22 (m, 1H), 4.09 (d, *J* = 13.9 Hz, 1H), 3.73 (d, *J* = 14.8 Hz, 1H), 2.81 (s, 3H), 2.50 – 2.41 (m, 1H), 1.64 (ddd, *J* = 13.3, 4.4, 2.0 Hz, 1H), 1.51 – 1.47 (m, 1H), 1.37 (d, *J* = 7.0 Hz, 3H), 1.09 (d, *J* = 6.6 Hz, 3H).

^{13}C NMR (126 MHz, $CDCl_3$) : δ 146.6, 108.7, 49.1, 47.4, 40.5, 40.1, 30.0, 17.9, 17.5.

HRMS ESI (m/z): $[M+H]^+$ calcd for $C_9H_{18}NSO_2$, 204.1058; found, 204.1050.

Table 3, Set 1b

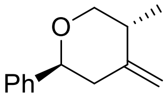

relative configuration

5'a

Supplementary Method A was followed.

^{1}H NMR (400 MHz, $CDCl_3$) : δ 7.46 – 7.22 (m, 5H), 4.85 (d, *J* = 1.8 Hz, 1H), 4.76 (d, *J* = 1.7 Hz, 1H), 4.31 (dd, *J* = 11.4, 2.5 Hz, 1H), 4.11 (dd, *J* = 10.7, 5.2 Hz, 1H), 3.15 (t, *J* = 11.0 Hz, 1H), 2.55 – 2.46 (m, 1H), 2.47 – 2.40 (m, 1H), 2.36 (t, *J* = 12.3 Hz, 1H), 1.02 (d, *J* = 6.7 Hz, 3H).

^{13}C NMR (101 MHz, $CDCl_3$) : δ 149.2, 142.4, 128.5, 127.7, 126.0, 106.4, 81.6, 75.3, 44.3, 36.9, 12.7.

HRMS ESI (m/z): $[M+H]^+$ calcd for $C_{13}H_{17}O$, 189.1279; found, 189.1270.

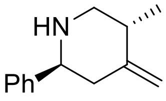

relative configuration

5'c

Supplementary Method A was followed, except 2 hrs addition time of reactant. The 1,4-syn:anti ratio was determined by GC-MS after mesylation of the crude product.

^{1}H NMR (600 MHz, $CDCl_3$) : δ 7.42 – 7.31 (m, 5H), 4.79 (s, 1H), 4.71 (s, 1H), 3.62 (d, *J* = 11.5 Hz, 1H), 3.24 (dd, *J* = 11.5, 4.6 Hz, 1H), 2.49 – 2.39 (m, 2H), 2.33 – 2.25 (m, 2H), 1.07 (d, *J* = 6.5 Hz, 3H).

^{13}C NMR (101 MHz, $CDCl_3$) : δ 150.9, 144.2, 128.5, 127.3, 126.6, 105.9, 63.5, 55.6, 44.9, 37.1, 15.0.

HRMS ESI (m/z): $[M+H]^+$ calcd for $C_{13}H_{18}N$, 188.1434; found, 188.1432.

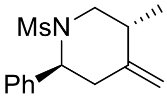

relative configuration

6e

The typical mesylation procedure was followed.

^{1}H NMR (500 MHz, $CDCl_3$) : δ 7.51 – 7.45 (m, 2H), 7.37 – 7.28 (m, 3H), 5.14 (dd, *J* = 6.9, 2.7 Hz, 1H), 4.94 (d, *J* = 2.0 Hz, 1H), 4.83 (d, *J* = 2.0 Hz, 1H), 3.46 (dd, *J* = 12.6, 2.7 Hz, 1H), 3.35 (dd, *J* = 12.7, 3.7 Hz, 1H), 3.02 (ddt, *J* = 14.5, 6.9, 2.1 Hz, 1H), 2.63 (tt, *J* = 7.2, 4.0 Hz, 1H), 2.58 (dd, *J* = 14.5, 2.6 Hz, 1H), 2.52 (s, 3H), 1.29 (d, *J* = 7.0 Hz, 3H).

^{13}C NMR (101 MHz, $CDCl_3$) : δ 145.7, 139.3, 128.6, 128.1, 127.9, 111.4, 56.8, 47.0, 39.0, 37.0, 35.0, 18.6.

HRMS ESI (m/z): $[M+H]^+$ calcd for $C_{14}H_{20}NSO_2$, 266.1215; found, 266.1207.

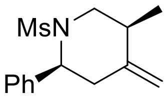

6'e

relative configuration

Supplementary Method A was followed. The 1,4-syn:anti ratio was determined by GC-MS.

^{1}H NMR (500 MHz, $CDCl_3$) : δ 7.47 (d, *J* = 7.5 Hz, 2H), 7.40 – 7.28 (m, 3H), 5.24 (s, 1H), 4.93 (d, *J* = 13.1 Hz, 2H), 3.77 (dd, *J* = 12.9, 5.2 Hz, 1H), 2.94 – 2.78 (m, 2H), 2.76 – 2.61 (m, 4H), 2.57 – 2.48 (m, 1H), 1.12 (d, *J* = 6.5 Hz, 3H).

^{13}C NMR (126 MHz, $CDCl_3$) : δ 145.4, 139.3, 128.6, 127.9, 127.7, 110.3, 56.4, 47.9, 39.5, 38.8, 36.2, 14.8.

HRMS ESI (m/z): $[M+H]^+$ calcd for $C_{14}H_{20}NSO_2$, 266.1215; found, 266.1207.

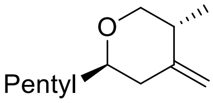

relative configuration

Supplementary Method A was followed.

^{1}H NMR (500 MHz, $CDCl_3$) : δ 4.74 (s, 1H), 4.66 (s, 1H), 3.984 (dd, *J* = 10.7, 5.2 Hz, 1H), 3.26 – 3.19 (m, 1H), 2.96 (t, *J* = 10.9 Hz, 1H), 2.36 – 2.22 (m, 2H), 1.96 – 2.06 (m, 1H), 1.58 – 1.51 (m, 1H)， 1.45 – 1.39 (m, 2H)， 1.29 – 1.22 (m, 5H), 0.97 – 0.94 (d, *J* = 6.6 Hz, 3H), 0.91 – 0.86 (t, *J* = 6.9 Hz, 3H).

^{13}C NMR (101 MHz, $CDCl_3$) : δ 149.7, 105.6, 79.6, 75.0, 42.2, 37.1, 36.4, 32.1, 25.3, 22.8, 14.2, 12.7.

HRMS ESI (m/z): $[M+H]^+$ calcd for $C_{12}H_{23}O$, 183.1749; found, 183.1743.

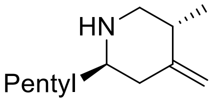

relative configuration

Supplementary Method A was followed, except 2 hrs addition time of reactant.

^{1}H NMR (400 MHz, $CDCl_3$) : δ 4.71 (d, *J* = 1.8 Hz, 1H), 4.62 (d, *J* = 1.7 Hz, 1H), 3.12 (dd, *J* = 11.6, 4.6 Hz, 1H), 2.52 – 2.44 (m, 1H), 2.34 (dd, *J* = 12.9, 2.8 Hz, 1H), 2.25 (t, *J* = 11.4 Hz, 1H), 2.18 – 2.10 (m, 1H), 1.84 – 1.75 (m, 2H), 1.44 – 1.25 (m, 8H), 1.01 (d, *J* = 6.5 Hz, 3H), 0.89 (t, *J* = 7.0 Hz, 3H).

^{13}C NMR (126 MHz, $CDCl_3$) : δ 151.2, 105.1, 58.7, 55.3, 43.3, 37.9, 37.0, 32.0, 25.7, 22.6, 14.9, 14.1.

HRMS ESI (m/z): $[M+H]^+$ calcd for $C_{12}H_{24}N$, 182.1908; found, 182.1901.

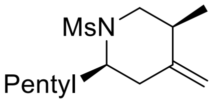

relative configuration

Supplementary Method A was followed.

^{1}H NMR (500 MHz, $CDCl_3$) : δ 4.83 (d, *J* = 1.6 Hz, 1H), 4.81 (d, *J* = 1.7 Hz, 1H), 4.03 (q, *J* = 7.2 Hz, 1H), 3.70 (dd, *J* = 13.3, 5.0 Hz, 1H), 2.90 (s, 3H), 2.58 (dd, *J* = 13.5, 11.9 Hz, 1H), 2.46 (dd, *J* = 13.5, 5.2 Hz, 1H), 2.32 – 2.21 (m, 2H), 1.52 – 1.38 (m, 2H), 1.34 – 1.22 (m, 6H), 1.04 (d, *J* = 6.6 Hz, 3H), 0.88 (t, *J* = 6.9 Hz, 3H).

^{13}C NMR (126 MHz, $CDCl_3$) : δ 145.6, 109.5, 54.1, 47.6, 40.7, 39.2, 36.3, 31.6, 29.8, 25.8, 22.6, 14.5, 14.0.

HRMS ESI (m/z): $[M+H]^+$ calcd for $C_{13}H_{26}NSO_2$, 260.1684; found, 260.1674.

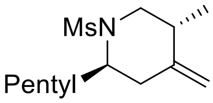

relative configuration

The typical mesylation procedure was followed.

^{1}H NMR (400 MHz, $CDCl_3$) : δ 4.87 (s, 1H), 4.69 (s, 1H), 3.97 (q, *J* = 6.5 Hz, 1H), 3.47 (d, *J* = 13.1 Hz, 1H), 3.17 (dd, *J* = 13.1, 3.9 Hz, 1H), 2.87 (s, 3H), 2.63 (dd, *J* = 13.8, 5.8 Hz, 1H), 2.50 – 2.41 (m, 1H), 2.04 (d, *J* = 13.7 Hz, 1H), 1.62 – 1.52 (m, 1H), 1.49 – 1.38 (m, 1H), 1.33 – 1.23 (m, 6H), 1.18 (dd, *J* = 7.0, 1.2 Hz, 3H), 0.88 (t, *J* = 7.0 Hz, 3H).

^{13}C NMR (101 MHz, $CDCl_3$) : δ 145.6, 111.4, 54.2, 46.5, 40.5, 37.4, 33.6, 31.7, 29.6, 26.2, 22.6, 18.5, 14.1.

HRMS ESI (m/z): $[M+H]^+$ calcd for $C_{13}H_{26}NSO_2$, 260.1684; found, 260.1677.

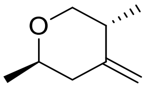

relative configuration

5'b

Supplementary Method A was followed, except that the crude reaction mixture was directly sent to analysis and silica gel chromatography without remove of toluene under vacuum.

^{1}H NMR (400 MHz, $CDCl_3$) : δ 4.74 (d, *J* = 1.8 Hz, 1H), 4.67 (d, *J* = 1.8 Hz, 1H), 3.92 (dd, *J* = 10.5, 5.0 Hz, 1H), 3.47 – 3.34 (m, 1H), 2.98 (t, *J* = 10.9 Hz, 1H), 2.40 – 2.22 (m, 2H), 2.01 (t, *J* = 13.1 Hz, 1H), 1.21 (d, *J* = 6.1 Hz, 3H), 0.95 (d, *J* = 6.7 Hz, 3H).

^{13}C NMR (101 MHz, $CDCl_3$) : δ 149.5, 105.7, 75.6, 75.0, 43.9, 36.8, 21.9, 12.6.

HRMS ESI (m/z): $[M+H]^+$ calcd for $C_8H_{15}O$, 127.1123; found, 127.1120.

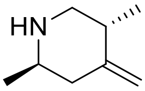

relative configuration

5'd

Supplementary Method A was followed, except 2 hrs addition time of reactant. The yield and 1,4-syn:anti ratio was determined by NMR after mesylation of crude product.

^{1}H NMR (500 MHz, $CDCl_3$) : δ 4.69 (d, *J* = 1.7 Hz, 1H), 4.60 (d, *J* = 1.7 Hz, 1H), 3.07 (dd, *J* = 11.7, 4.7 Hz, 1H), 2.65 – 2.55 (m, 1H), 2.32 – 2.26 (m, 1H), 2.24 (dd, *J* = 11.5, 1.9 Hz, 1H), 2.13 – 2.04 (m, 1H), 1.83 – 1.73 (m, 2H), 1.09 (d, *J* = 6.3 Hz, 3H), 0.98 (d, *J* = 6.6 Hz, 3H).

^{13}C NMR (126 MHz, $CDCl_3$) : δ 151.3, 105.2, 55.4, 54.3, 45.2, 37.7, 22.7, 14.9.

HRMS ESI (m/z): $[M+H]^+$ calcd for $C_8H_{16}N$, 126.1283; found, 126.1277.

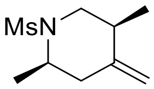

relative configuration

6'f

Supplementary Method A was followed.

^{1}H NMR (500 MHz, $CDCl_3$) : δ 4.85 (d, *J* = 1.7 Hz, 1H), 4.83 (d, *J* = 1.7 Hz, 1H), 4.33 – 4.26 (m, 1H), 3.67 (dd, *J* = 12.7, 5.3 Hz, 1H), 2.86 (s, 3H), 2.64 – 2.55 (m, 1H), 2.53 (dd, *J* = 13.1, 5.8 Hz, 1H), 2.32 – 2.24 (m, 1H), 2.13 (dd, *J* = 13.2, 1.9 Hz, 1H), 1.12 (dd, *J* = 6.8, 1.4 Hz, 3H), 1.06 (dd, *J* = 6.6, 1.4 Hz, 3H).

^{13}C NMR (126 MHz, $CDCl_3$) : δ 145.2, 109.9, 49.7, 47.4, 41.5, 40.1, 36.5, 16.1, 14.7.

HRMS ESI (m/z): $[M+H]^+$ calcd for $C_9H_{18}NO_2S$, 204.1058; found, 204.1050.

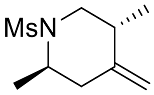

relative configuration

6f

The typical mesylation procedure was followed.

^{1}H NMR (400 MHz, $CDCl_3$) : δ 4.90 (s, 1H), 4.72 (s, 1H), 4.26 – 4.20 (m, 1H), 3.40 (d, *J* = 12.6 Hz, 1H), 3.20 (dd, *J* = 12.7, 3.8 Hz, 1H), 2.84 (s, 3H), 2.73 (dd, *J* = 13.5, 6.3 Hz, 1H), 2.53 – 2.45 (m, 1H), 1.91 (d, *J* = 13.6 Hz, 1H), 1.21 – 1.12 (m, 6H).

^{13}C NMR (101 MHz, $CDCl_3$) : δ 145.3, 111.5, 49.8, 46.3, 39.7, 37.5, 36.5, 18.0, 15.8.

HRMS ESI (m/z): $[M+H]^+$ calcd for $C_9H_{18}NO_2S$, 204.1058; found, 204.1052.

Table 3, Set 2

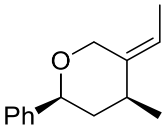

relative configuration

Supplementary Method A was followed.

^{1}H NMR (400 MHz, $CDCl_3$) : δ 7.38 – 7.20 (m, 5H), 5.30 (dd, *J* = 6.6, 2.0 Hz, 1H), 4.85 (d, *J* = 12.5 Hz, 1H), 4.53 (dd, *J* = 11.4, 2.2 Hz, 1H), 3.87 (dd, *J* = 12.5, 1.1 Hz, 1H), 2.43 (s, 1H), 1.96 (ddd, *J* = 12.9, 4.3, 2.3 Hz, 1H), 1.71 (ddd, *J* = 6.9, 2.0, 1.1 Hz, 1H), 1.48 – 1.34 (m, 1H), 1.08 (d, *J* = 6.5 Hz, 3H).

^{13}C NMR (101 MHz, $CDCl_3$) : δ 142.6, 138.4, 128.3, 127.4, 125.9, 116.0, 80.1, 67.0, 44.5, 35.5, 17.7, 12.7.

HRMS ESI (m/z): $[M+H]^+$ calcd for $C_{14}H_{19}O$, 203.1436; found, 203.1429.

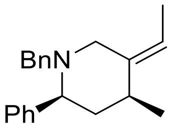

relative configuration

Supplementary Method A was followed.

^{1}H NMR (400 MHz, $CDCl_3$) : δ 7.48 – 7.14 (m, 10H), 5.20 (q, *J* = 6.4 Hz, 1H), 3.80 (dd, *J* = 12.9, 4.4 Hz, 2H), 3.36 (dd, *J* = 11.3, 2.9 Hz, 1H), 2.90 (d, *J* = 13.7 Hz, 1H), 2.29 – 2.16 (m, 2H), 1.86 (ddd, *J* = 13.0, 4.3, 3.0 Hz, 1H), 1.48 – 1.34 (m, 4H), 1.03 (d, *J* = 6.5 Hz, 3H).

^{13}C NMR (101 MHz, $CDCl_3$) : δ 144.7, 139.8, 139.4, 128.5, 128.4, 128.1, 127.5, 127.0, 126.6, 114.5, 69.3, 59.4, 52.8, 46.3, 36.7, 17.4, 12.7.

HRMS ESI (m/z): $[M+H]^+$ calcd for $C_{12}H_{26}N$, 292.2065; found, 292.2054.

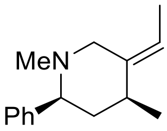

relative configuration

Supplementary Method A was followed. The1,3-syn:anti ratio was determined by GC-Ms.

^{1}H NMR (500 MHz, $CDCl_3$) : δ 7.36 – 7.19 (m, 5H), 5.28 (qt, *J* = 6.9, 1.8 Hz, 1H), 3.86 (d, *J* = 12.2 Hz, 1H), 2.99 (dd, *J* = 11.4, 2.7 Hz, 1H), 2.45 (dq, *J* = 12.2, 1.2 Hz, 1H), 2.23 – 2.14 (m, 1H), 2.05 (s, 3H), 1.77 (ddd, *J* = 13.0, 4.3, 2.7 Hz, 1H), 1.72 (dt, *J* = 6.7, 1.7 Hz, 3H), 1.38 (td, *J* = 12.8, 11.4 Hz, 1H), 1.04 (d, *J* = 6.6 Hz, 3H).

^{13}C NMR (126 MHz, $CDCl_3$) : δ 144.2, 139.2, 128.5, 127.7, 127.2, 115.0, 71.3, 57.2, 45.3, 44.4, 36.6, 17.5, 13.2.

HRMS ESI (m/z): $[M+H]^+$ calcd for $C_{15}H_{22}N$, 216.1752; found, 216.1745.

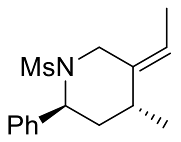

relative configuration

Supplementary Method A was followed. When IPr as ligand, 95% yield and 1,3-syn:anti = 29:71 was obtained; when IPr^{Me} as ligand, 89% yield and 1,3-syn:anti = 20:80 was obtained.

^{1}H NMR (500 MHz, $CDCl_3$) : δ 7.49 (d, *J* = 7.3 Hz, 2H), 7.39 (t, *J* = 7.9 Hz, 2H), 7.28 (t, *J* = 7.2 Hz, 1H), 5.34 (q, *J* = 7.2, 6.7 Hz, 1H), 5.22 (d, *J* = 6.6 Hz, 1H), 4.61 (d, *J* = 15.3 Hz, 1H), 3.35 (d, *J* = 15.3 Hz, 1H), 2.87 (s, 3H), 2.44 (ddd, *J* = 13.7, 3.9, 2.2 Hz, 1H), 2.38 – 2.29 (m, 1H), 1.76 – 1.68 (m, 4H), 1.10 (d, *J* = 6.6 Hz, 3H).

^{13}C NMR (101 MHz, $CDCl_3$) : δ 138.9, 137.1, 128.9, 127.3, 126.9, 117.1, 55.6, 42.1, 40.7, 37.5, 31.5, 18.2, 13.0.

HRMS ESI (m/z): $[M+H]^+$ calcd for $C_{15}H_{22}NSO_2$, 280.1371; found, 280.1365.

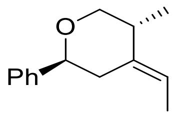

relative configuration

Supplementary Method A was followed.

^{1}H NMR (500 MHz, $CDCl_3$) : δ 7.51 – 7.25 (m, 5H), 5.33 (q, *J* = 6.8 Hz, 1H), 4.29 (dd, *J* = 11.6, 2.6 Hz, 1H), 4.10 (dd, *J* = 10.7, 4.9 Hz, 1H), 3.18 (t, *J* = 10.9 Hz, 1H), 2.86 (dd, *J* = 13.7, 2.6 Hz, 1H), 2.52 – 2.38 (m, 1H), 2.13 – 2.03 (m, 1H), 1.70 (d, *J* = 6.7 Hz, 3H), 1.01 (d, *J* = 6.7 Hz, 3H).

^{13}C NMR (126 MHz, $CDCl_3$) : δ 142.7, 139.2, 128.5, 127.7, 126.1, 114.7, 80.8, 75.7, 37.1 (37.1), 12.8 (12.8).

^{13}C NMR (126 MHz, C_6D_6) : δ 143.6, 139.6, 128.5, 127.6, 126.3, 114.4, 80.8, 75.6, 37.9, 37.3, 12.7, 12.7.

HRMS ESI (m/z): $[M+H]^+$ calcd for $C_{14}H_{19}O$, 203.1436; found, 203.1430.

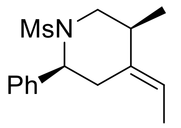

relative configuration

Supplementary Method A was followed.

^{1}H NMR (500 MHz, $CDCl_3$) : δ 7.44 – 7.39 (m, 2H), 7.35 – 7.30 (m, 2H), 7.30 – 7.25 (m, 1H), 5.41 (q, *J* = 6.9 Hz, 1H), 5.23 (d, *J* = 4.9 Hz, 1H), 3.73 (dd, *J* = 12.8, 5.0 Hz, 1H), 3.07 (dd, *J* = 14.3, 2.2 Hz, 1H), 2.71 (t, *J* = 12.3 Hz, 1H), 2.59 (s, 3H), 2.55 – 2.49 (m, 2H), 1.61 (dd, *J* = 6.9, 1.8 Hz, 3H), 1.07 (d, *J* = 6.6 Hz, 3H).

^{13}C NMR (126 MHz, $CDCl_3$) : δ 139.9, 136.0, 128.7, 127.8, 127.7, 117.9, 56.7, 48.6, 39.4, 36.8, 32.1, 15.1, 13.1.

HRMS ESI (m/z): $[M+H]^+$ calcd for $C_{15}H_{22}NO_2S$, 280.1371; found, 280.1364.

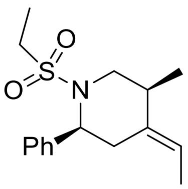

Supplementary Method A was followed.

^{1}H NMR (500 MHz, $CDCl_3$) : δ 7.42 – 7.24 (m, 5H), 5.36 (qt, *J* = 6.7, 2.1 Hz, 1H), 5.23 (dd, *J* = 6.5, 3.0 Hz, 1H), 3.71 (ddd, *J* = 13.1, 5.0, 1.3 Hz, 1H), 3.11 (dd, *J* = 14.2, 2.7 Hz, 1H), 2.91 – 2.71 (m, 3H), 2.51 (ddt, *J* = 15.7, 7.9, 1.4 Hz, 1H), 2.47 – 2.40 (m, 1H), 1.61 (s, 3H), 1.29 (t, *J* = 7.4 Hz, 3H), 1.03 (d, *J* = 6.6 Hz, 3H).

^{13}C NMR (126 MHz, $CDCl_3$) : δ 140.4, 136.1, 128.6, 127.5, 127.4, 117.7, 56.7, 49.1, 47.4, 37.2, 32.2, 15.1, 13.1, 8.2.

HRMS ESI (m/z): $[M+H]^+$ calcd for $C_{16}H_{24}NO_2S$, 294.1528; found, 294.1519.

Table 3, Set 3a/b

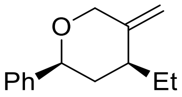

relative configuration

Supplementary Method A was followed except 2 hrs addition time of reactant. The 1,3-syn:anti ratio was determined by GC-MS. The NMR data was abstracted from a 1:1 mixture of desired product and its exo-cyclic olefin reduced byproduct. For further structure confirmation, the mixture was oxidized to corresponding ketone (see below) analogously to the literature procedure. [14]

^{1}H NMR (500 MHz, $CDCl_3$) : δ 7.40 – 7.24 (m, 5H), 4.97 (s, 1H), 4.86 (s, 1H), 4.55 (dd, *J* = 11.4, 2.4 Hz, 1H), 4.35 (d, *J* = 12.1 Hz, 1H), 4.12 (d, *J* = 11.9 Hz, 1H), 2.29 – 2.21 (m, 1H), 2.10 (ddd, *J* = 13.0, 4.6, 2.4 Hz, 1H), 1.88 – 1.78 (m, 1H), 1.38 – 1.29 (m, 2H), 0.97 (t, *J* = 7.5 Hz, 3H).

^{13}C NMR (126 MHz, $CDCl_3$) : δ 147.4, 142.6, 128.5, 127.6, 126.1, 107.8, 80.5, 74.2, 41.9, 41.4, 24.4, 11.4.

HRMS ESI (m/z): $[M+H]^+$ calcd for $C_{14}H_{19}O$, 203.1436; found, 203.1430.

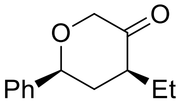

relative configuration

^{1}H NMR (500 MHz, $CDCl_3$) : δ 7.44 – 7.29 (m, 5H), 4.86 (dd, *J* = 11.3, 2.4 Hz, 1H), 4.25 (d, *J* = 15.4 Hz, 1H), 4.15 (d, *J* = 15.4 Hz, 1H), 2.59 – 2.51 (m, 1H), 2.42 (ddd, *J* = 13.6, 6.3, 2.4 Hz, 1H), 2.02 – 1.94 (m, 1H), 1.95 – 1.84 (m, 1H), 1.48 – 1.38 (m, 1H), 0.94 (t, *J* = 7.6 Hz, 3H).

^{13}C NMR (126 MHz, $CDCl_3$) : δ 208.3, 141.2, 128.7, 128.1, 125.9, 78.9, 74.7, 49.0, 39.0, 21.8, 11.1.

HRMS ESI (m/z): $[M+H]^+$ calcd for $C_{13}H_{17}O_2$, 205.1229; found, 205.1225.

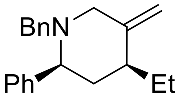

relative configuration

Supplementary Method A was followed.

^{1}H NMR (500 MHz, $CDCl_3$) : δ 7.48 – 7.45 (m, 2H), 7.33 (t, *J* = 7.6 Hz, 2H), 7.29 – 7.25 (m, 4H), 7.23 – 7.18 (m, 2H), 4.75 (d, *J* = 1.7 Hz, 1H), 4.70 (t, *J* = 1.9 Hz, 1H), 3.79 (d, *J* = 13.4 Hz, 1H), 3.37 (d, *J* = 11.7 Hz, 1H), 3.33 (dd, *J* = 11.3, 2.7 Hz, 1H), 2.88 (d, *J* = 13.4 Hz, 1H), 2.60 (d, *J* = 11.4 Hz, 1H), 2.03 – 1.96 (m, 2H), 1.79 – 1.69 (m, 1H), 1.37 – 1.24 (m, 2H), 0.92 (t, *J* = 7.5 Hz, 3H).

^{13}C NMR (101 MHz, $CDCl_3$) : δ 148.2, 144.7, 139.3, 128.8, 128.7, 128.2, 127.7, 127.2, 126.8, 106.7, 69.2, 60.6, 59.6, 43.0, 42.8, 24.3, 11.7.

HRMS ESI (m/z): $[M+H]^+$ calcd for $C_{21}H_{26}N$, 292.2065; found, 292.2057.

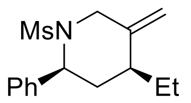 relative configuration

The typical mesylation procedure was followed.

^{1}H NMR (500 MHz, $CDCl_3$) : δ 7.43 – 7.26 (m, 5H), 5.07 (s, 1H), 4.96 (s, 1H), 4.82 (dd, *J* = 9.6, 6.1 Hz, 1H), 4.24 (d, *J* = 15.7 Hz, 1H), 4.04 (d, *J* = 15.7 Hz, 1H), 2.71 (s, 3H), 2.37 – 2.28 (m, 1H), 2.18 (ddd, *J* = 14.2, 6.3, 4.3 Hz, 1H), 1.83 (dt, *J* = 14.0, 10.1 Hz, 1H), 1.59 – 1.53 (m, 1H), 1.41 – 1.33 (m, 1H), 0.86 (t, *J* = 7.4 Hz, 3H).

^{13}C NMR (126 MHz, $CDCl_3$) : δ 145.1, 142.5, 128.8, 127.5, 126.2, 110.5, 58.4, 47.6, 40.0, 39.6, 35.8, 26.1, 11.2.

HRMS ESI (m/z): $[M+H]^+$ calcd for $C_{15}H_{22}NO_2S$, 280.1371; found, 280.1363.

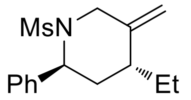 relative configuration

Supplementary Method A was followed except 2 hrs addition time of reactant. The NMR data was abstracted from a mixture of desired product and its exo-olefin reduced byproduct.

^{1}H NMR (400 MHz, $CDCl_3$) : δ 7.51 – 7.27 (m, 5H), 5.27 (d, *J* = 5.9 Hz, 1H), 5.00 (s, 1H), 4.83 (s, 1H), 4.17 (d, *J* = 14.9 Hz, 1H), 3.62 (d, *J* = 14.7 Hz, 1H), 2.91 (s, 3H), 2.60 (ddd, *J* = 11.7, 4.2, 2.2 Hz, 1H), 2.16 – 2.07 (m, 1H), 1.78 – 1.68 (m, 1H), 1.68 – 1.60 (m, 1H), 1.43 – 1.31 (m, 1H), 1.00 (t, *J* = 6.5 Hz, 3H).

^{13}C NMR (101 MHz, $CDCl_3$) : δ 145.9, 138.7, 129.0, 127.4, 126.8, 109.2, 55.6, 49.1, 41.0, 37.7, 34.3, 24.7, 11.4.

HRMS ESI (m/z): $[M+H]^+$ calcd for $C_{15}H_{22}NO_2S$, 280.1371; found, 280.1369.

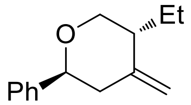 relative configuration

Supplementary Method A was followed.

^{1}H NMR (400 MHz, $CDCl_3$) : δ 7.44 – 7.23 (m, 5H), 4.86 (s, 1H), 4.76 (s, 1H), 4.32 (dt, *J* = 11.1, 2.5 Hz, 1H), 4.23 (dd, *J* = 10.7, 5.1 Hz, 1H), 3.16 (t, *J* = 10.9 Hz, 1H), 2.49 (d, *J* = 13.1 Hz, 1H), 2.34 (t, *J* = 12.2 Hz, 1H), 2.27 – 2.15 (m, 1H), 1.78 – 1.65 (m, 1H), 1.33 – 1.24 (m, 1H), 1.00 (t, *J* = 7.5 Hz, 3H).

^{13}C NMR (101 MHz, $CDCl_3$) : δ 148.4, 142.4, 128.5, 127.7, 126.0, 106.5, 81.8, 73.7, 44.7, 43.9, 20.7, 11.8.

HRMS ESI (m/z): $[M+H]^+$ calcd for $C_{14}H_{19}O$, 203.1436; found, 203.1430.

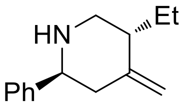

relative configuration

Supplementary Method A was followed, except 2 hrs addition time of reactant. The 1,4-syn:anti ratio was determined by GC-MS after mesylation of crude product.

^{1}H NMR (500 MHz, $CDCl_3$) : δ 7.42 – 7.25 (m, 5H), 4.80 (d, *J* = 1.7 Hz, 1H), 4.70 (d, *J* = 1.5 Hz, 1H), 3.63 (dd, *J* = 11.4, 2.9 Hz, 1H), 3.38 (dd, *J* = 11.1, 4.7 Hz, 1H), 2.45 (dd, *J* = 12.9, 3.0 Hz, 1H), 2.37 (t, *J* = 11.1 Hz, 1H), 2.26 (t, *J* = 12.1 Hz, 1H), 2.12 – 2.04 (m, 1H), 1.79 – 1.70 (m, 2H), 1.35 – 1.26 (m, 1H), 1.00 (t, *J* = 7.5 Hz, 3H).

^{13}C NMR (126 MHz, $CDCl_3$) : δ 150.4, 144.4, 128.6, 127.4, 126.7, 106.0, 64.0, 53.5, 45.7, 44.4, 22.4, 12.0.

HRMS ESI (m/z): $[M+H]^+$ calcd for $C_{14}H_{20}N$, 202.1596; found, 202.1589.

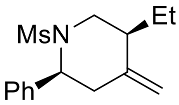

relative configuration

Supplementary Method A was followed. The 1,4-syn:anti ratio was determined by GC-MS.

^{1}H NMR (400 MHz, $CDCl_3$) : δ 7.47 – 7.28 (m, 5H), 5.23 – 5.15 (m, 1H), 4.97 (s, 1H), 4.93 (s, 1H), 3.97 – 3.86 (m, 1H), 2.83 – 2.74 (m, 3H), 2.67 (s, 3H), 2.40 – 2.29 (m, 1H), 1.78 – 1.67 (m, 1H), 1.44 – 1.35 (m, 1H), 1.02 (td, *J* = 7.5, 2.6 Hz, 3H).

^{13}C NMR (126 MHz, $CDCl_3$) : δ 144.6, 139.9, 128.7, 127.7, 127.7, 111.0, 57.3, 46.5, 43.2, 39.7, 39.4, 22.8, 11.6.

HRMS ESI (m/z): $[M+H]^+$ calcd for $C_{15}H_{22}NO_2S$, 280.1371; found, 280.1363.

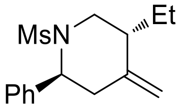

relative configuration

The typical mesylation procedure was followed.

^{1}H NMR (500 MHz, $CDCl_3$) : δ 7.58 – 7.20 (m, 5H), 5.22 – 5.17 (m, 1H), 4.96 – 4.90 (m, 1H), 4.92 – 4.86 (m, 1H), 3.63 (d, *J* = 12.8 Hz, 1H), 3.28 (dt, *J* = 12.8, 3.8 Hz, 1H), 2.98 – 2.90 (m, 1H), 2.55 (d, *J* = 16.5 Hz, 1H), 2.50 (s, 3H), 2.30 – 2.23 (m, 1H), 1.74 – 1.65 (m, 2H), 0.93 (t, *J* = 7.5 Hz, 3H).

^{13}C NMR (126 MHz, $CDCl_3$) : δ 143.7, 139.3, 128.7, 128.3, 128.0, 113.0, 56.4, 45.0, 44.7, 39.0, 34.9, 24.2, 12.0.

HRMS ESI (m/z): $[M+H]^+$ calcd for $C_{15}H_{22}NO_2S$, 280.1371; found, 280.1363.

Table 3, Set 4a/b

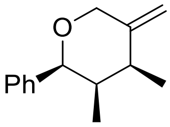

relative configuration

Supplementary Method A was followed.

^{1}H NMR (400 MHz, $CDCl_3$) : δ 7.36 – 7.20 (m, 5H), 4.94 (s, 1H), 4.88 (s, 1H), 4.33 (d, *J* = 12.0 Hz, 1H), 4.14 (d, *J* = 12.0 Hz, 1H), 4.07 (d, *J* = 9.9 Hz, 1H), 2.12 – 2.03 (m, 1H), 1.57 – 1.46 (m, 1H), 1.18 (d, *J* = 6.4 Hz, 3H), 0.73 (d, *J* = 6.6 Hz, 3H).

^{13}C NMR (101 MHz, $CDCl_3$) : δ 148.2, 141.2, 128.3, 127.9, 127.6, 107.6, 87.0, 74.0, 45.1, 41.3, 15.4, 14.5.

HRMS ESI (m/z): $[M+H]^+$ calcd for $C_{14}H_{19}O$, 203.1436; found, 203.1430.

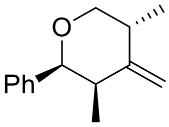

relative configuration

Supplementary Method A was followed.

^{1}H NMR (400 MHz, $CDCl_3$) : δ 7.38 – 7.27 (m, 5H), 4.85 (dd, *J* = 3.2, 1.6 Hz, 2H), 4.09 (dd, *J* = 10.6, 5.0 Hz, 1H), 3.83 (d, *J* = 10.0 Hz, 1H), 3.15 (t, *J* = 10.9 Hz, 1H), 2.60 – 2.49 (m, 1H), 2.43 – 2.33 (m, 1H), 1.04 (d, *J* = 6.6 Hz, 3H), 0.78 (d, *J* = 6.7 Hz, 3H).

^{13}C NMR (101 MHz, $CDCl_3$) : δ 153.7, 141.3, 128.4, 128.0, 127.3, 103.6, 88.0, 75.6, 43.4, 38.0, 13.4, 13.1.

HRMS ESI (m/z): $[M+H]^+$ calcd for $C_{14}H_{19}O$, 203.1436; found, 203.1429.

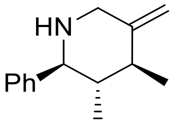

relative configuration

Supplementary Method A was followed except 2 hrs addition time of reactant.

^{1}H NMR (500 MHz, $CDCl_3$) : δ 7.32 – 7.28 (m, 4H), 7.26 – 7.22 (m, 1H), 4.88 (d, *J* = 1.7 Hz, 1H), 4.80 (t, *J* = 1.9 Hz, 1H), 3.56 (d, *J* = 12.2 Hz, 1H), 3.42 (d, *J* = 12.1 Hz, 1H), 3.39 (d, *J* = 10.1 Hz, 1H), 2.02 – 1.87 (m, 1H), 1.47 – 1.38 (m, 1H), 1.16 (d, *J* = 6.6 Hz, 3H), 0.73 (d, *J* = 6.6 Hz, 3H).

^{13}C NMR (126 MHz, $CDCl_3$) : δ 150.1, 143.5, 128.5, 128.0, 127.4, 106.5, 69.5, 55.2, 45.6, 42.5, 16.7, 15.0.

HRMS ESI (m/z): $[M+H]^+$ calcd for $C_{14}H_{20}N$, 202.1596; found, 202.1589.

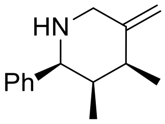

relative configuration

Supplementary Method A was followed except 2 hrs addition time of reactant.

^{1}H NMR (500 MHz, $CDCl_3$) : δ 7.50 – 7.21 (m, 5H), 4.98 (s, 1H), 4.73 (s, 1H), 4.14 (d, J = 2.9 Hz, 1H), 3.65 (dd, J = 12.7, 2.1 Hz, 1H), 3.44 (d, J = 12.7 Hz, 1H), 2.69 – 2.61 (m, 1H), 2.08 – 2.00 (m, 1H), 1.10 (d, J = 6.7 Hz, 3H), 0.50 (d, J = 6.9 Hz, 3H).

^{13}C NMR (126 MHz, $CDCl_3$) : δ 150.1, 143.6, 128.5, 128.0, 127.5, 106.5, 69.6, 55.3, 45.6, 42.6, 16.7, 15.0.

HRMS ESI (m/z): $[M+H]^+$ calcd for $C_{14}H_{20}N$, 202.1596; found, 202.1589.

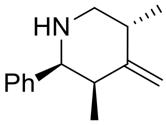

relative configuration

Supplementary Method A was followed except 2 hrs addition time of reactant.

^{1}H NMR (500 MHz, $CDCl_3$) : δ 7.35 – 7.30 (m, 4H), 7.25 – 7.19 (m, 1H), 4.86 (t, J = 1.8 Hz, 1H), 4.66 (t, J = 1.9 Hz, 1H), 3.92 (d, J = 3.4 Hz, 1H), 3.26 (dd, J = 11.1, 4.8 Hz, 1H), 2.66 – 2.59 (m, 1H), 2.57 – 2.46 (m, 1H), 2.38 (t, J = 11.2 Hz, 1H), 1.85 – 1.82 (m, 3H), 1.04 (d, J = 6.6 Hz, 3H), 0.84 (d, J = 7.0 Hz, 3H).

^{13}C NMR (126 MHz, $CDCl_3$) : δ156.3, 142.7, 128.2, 126.7, 126.5, 105.0, 65.3, 55.8, 45.6, 32.1, 15.0, 13.5.

HRMS ESI (m/z): $[M+H]^+$ calcd for $C_{14}H_{20}N$, 202.1596; found, 202.1587.

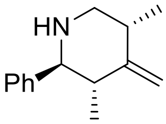

relative configuration

Supplementary Method A was followed, except 2 hrs addition time of reactant.

^{1}H NMR (500 MHz, $CDCl_3$) : δ 7.43 – 7.29 (m, 5H), 4.82 (d, J = 1.5 Hz, 2H), 3.23 (d, J = 6.6 Hz, 1H), 3.18 (d, J = 10.1 Hz, 1H), 2.49 – 2.39 (m, 2H), 2.36 – 2.28 (m, 1H), 1.10 (d, J = 4.9 Hz, 3H), 0.79 (d, J = 6.7, 3H).

^{13}C NMR (126 MHz, $CDCl_3$) : δ 155.8, 143.8, 128.5, 127.8, 127.6, 103.2, 70.7, 56.3, 43.9, 38.7, 15.6, 14.5.

HRMS ESI (m/z): $[M+H]^+$ calcd for $C_{14}H_{20}N$, 202.1596; found, 202.1588.

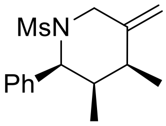

relative configuration

The typical mesylation procedure was followed.

^{1}H NMR (500 MHz, $CDCl_3$) : δ 7.36 – 7.32 (m, 2H), 7.26 – 7.22 (m, 3H), 5.15 (d, *J* = 7.5 Hz, 1H), 5.02 (d, *J* = 1.8 Hz, 1H), 4.93 (d, *J* = 1.2 Hz, 1H), 4.55 (d, *J* = 17.4 Hz, 1H), 4.06 (dq, *J* = 17.4, 2.1 Hz, 1H), 2.76 (s, 3H), 2.63 – 2.56 (m, 1H), 2.39 – 2.33 (m, 1H), 1.15 (d, *J* = 6.9 Hz, 3H), 0.53 (d, *J* = 7.3 Hz, 3H).

^{13}C NMR (126 MHz, $CDCl_3$) : δ 145.6, 140.9, 128.5, 127.1, 126.3, 109.2, 62.3, 47.3, 39.1, 37.9, 36.8, 15.6, 11.0.

HRMS ESI (m/z): $[M+H]^+$ calcd for $C_{15}H_{22}NO_2S$, 280.1371; found, 280.1361.

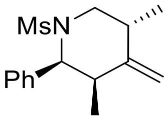

relative configuration

The typical mesylation procedure was followed.

^{1}H NMR (500 MHz, $CDCl_3$) : δ 7.46 – 7.39 (m, 2H), 7.35 – 7.28 (m, 3H), 5.09 (s, 1H), 5.02 (d, *J* = 6.3 Hz, 1H), 4.79 (s, 1H), 3.49 – 3.44 (m, 1H), 3.37 (dd, *J* = 12.1, 3.7 Hz, 1H), 3.17 – 3.07 (m, 1H), 2.87 – 2.79 (m, 1H), 2.19 (s, 3H), 1.36 (d, *J* = 7.0 Hz, 3H), 0.85 (d, *J* = 6.9 Hz, 3H).

^{13}C NMR (126 MHz, $CDCl_3$) : δ 150.4, 136.3, 129.7, 128.6, 128.5, 109.7, 62.3, 46.3, 38.4, 37.6, 34.5, 19.8, 14.9.

HRMS ESI (m/z): $[M+H]^+$ calcd for $C_{15}H_{22}NO_2S$, 280.1371; found, 280.1361.

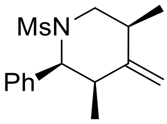

relative configuration

Supplementary Method A was followed, except that 3 equiv. of $NaBH_4$ was added to the catalyst before addition reactant, to suppress the side reaction of acetophenone insertion.

^{1}H NMR (500 MHz, $CDCl_3$) : δ 7.40 – 7.27 (m, 5H), 5.07 (s, 1H), 5.01 (d, *J* = 6.1 Hz, 1H), 4.89 (s, 1H), 3.73 (dd, *J* = 11.7, 5.4 Hz, 1H), 2.90 (p, *J* = 6.9 Hz, 1H), 2.81 (t, *J* = 11.7 Hz, 1H), 2.62 (dq, *J* = 12.1, 5.9 Hz, 1H), 2.14 (s, 3H) , 1.23 (d, *J* = 6.6 Hz, 3H), 0.88 (d, *J* = 6.9 Hz, 3H).

^{13}C NMR (126 MHz, $CDCl_3$) : δ 149.8, 136.9, 129.8, 128.5, 128.4, 107.7, 62.6, 48.0, 40.1, 37.5, 36.9, 15.2, 15.2.

HRMS ESI (m/z): $[M+H]^+$ calcd for $C_{15}H_{22}NO_2S$, 280.1371; found, 280.1365.

Table 3, Set 5a/b

In this set, Supplementary Method A was modified as: enyne to alcohol ratio was reduced from 1:3 to 1:1.5 (in mol), to suppress the undesired reduction of enyne to corresponding diene.

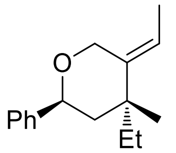

relative configuration

Supplementary Method A was followed.

^{1}H NMR (500 MHz, $CDCl_3$) : δ 7.42 – 7.20 (m, 5H), 5.38 (q, *J* = 6.9 Hz, 1H), 4.81 – 4.63 (m, 2H), 4.03 (d, *J* = 13.1 Hz, 1H), 1.93 (dq, *J* = 14.6, 7.5 Hz, 1H), 1.70 (d, *J* = 6.9 Hz, 3H), 1.69 – 1.55 (m, 2H), 1.43 (dq, *J* = 14.6, 7.4 Hz, 1H), 1.03 (s, 3H), 0.77 (t, *J* = 7.5 Hz, 3H).

^{13}C NMR (126 MHz, $CDCl_3$) : δ 143.0, 139.4, 128.4, 127.4, 126.0, 118.5, 75.5, 63.4, 48.2, 38.5, 29.6, 25.0, 13.0, 8.6.

HRMS ESI (m/z): $[M+NH_4]^+$ calcd for $C_{16}H_{26}ON$, 248.2014; found, 248.2000.

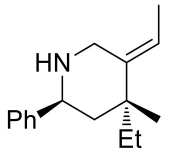

relative configuration

Supplementary Method A was followed, except 2 hrs addition of reactant and 20% catalyst loading.

^{1}H NMR (500 MHz, $CDCl_3$) : δ 7.37 – 7.21 (m, 5H), 5.32 (q, *J* = 6.5 Hz, 1H), 4.07 (dd, *J* = 12.1, 2.7 Hz, 1H), 3.89 (d, *J* = 13.4 Hz, 1H), 3.24 (d, *J* = 13.4 Hz, 1H), 2.08 – 1.97 (m, 1H), 1.88 (dd, *J* = 13.7, 7.5 Hz, 1H), 1.73 – 1.66 (m, 4H), 1.52 (dd, *J* = 13.4, 12.0 Hz, 1H), 1.36 (dt, *J* = 13.6, 7.4 Hz, 1H), 1.01 (s, 3H), 0.74 (t, *J* = 7.5 Hz, 3H).

^{13}C NMR (126 MHz, $CDCl_3$) : δ 144.6, 140.6, 128.5, 127.2, 126.7, 117.3, 57.1, 48.9, 43.5, 39.3, 29.8, 25.2, 13.2, 8.4.

HRMS ESI (m/z): $[M+H]^+$ calcd for $C_{16}H_{24}N$, 230.1909; found, 230.1903.

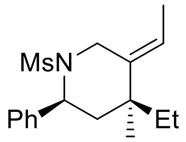

relative configuration

Supplementary Method A was followed. When IPr as ligand, 96% yield and 1,3-syn:anti = 13:87 was obtained; when IPr^{Me} as ligand, 89% yield and 1,3-syn:anti = 20:80 was obtained.

^{1}H NMR (500 MHz, $CDCl_3$) : δ 7.42 – 7.21 (m, 5H), 5.44 (q, *J* = 6.9 Hz, 1H), 4.83 (dd, *J* = 10.8, 5.5 Hz, 1H), 4.71 (d, *J* = 15.7 Hz, 1H), 3.73 (dt, *J* = 15.7, 1.3 Hz, 1H), 2.62 (s, 3H), 2.05 (dd, *J* = 14.3, 10.8 Hz, 1H), 1.75 (dd, *J* = 6.9, 1.2 Hz, 3H), 1.66 (dd, *J* = 14.3, 5.5 Hz, 1H), 1.36 (tq, *J* = 13.9, 7.0 Hz, 2H), 1.20 (s, 3H), 0.72 (t, *J* = 7.4 Hz, 3H).

^{13}C NMR (126 MHz, $CDCl_3$) : δ 143.5, 139.7, 128.8, 127.4, 126.1, 119.4, 55.9, 42.6, 41.5, 39.9, 38.1, 35.4, 26.7, 13.3, 8.5.

HRMS ESI (m/z): $[M+H]^+$ calcd for $C_{17}H_{26}O_2NS$, 308.1684; found, 308.1676.

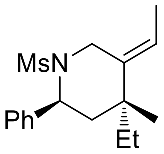

relative configuration

The typical mesylation procedure was followed.

^{1}H NMR (500 MHz, $CDCl_3$) : δ 7.41 – 7.26 (m, 5H), 5.42 (q, *J* = 6.9 Hz, 1H), 4.85 (dd, *J* = 11.1, 6.1 Hz, 1H), 4.56 (d, *J* = 15.7 Hz, 1H), 3.98 (d, *J* = 15.7 Hz, 1H), 2.49 (s, 3H), 1.93 (dd, *J* = 14.4, 6.2 Hz, 1H), 1.88 – 1.79 (m, 1H), 1.69 (d, *J* = 6.9 Hz, 3H), 1.68 – 1.58 (m, 2H), 0.98 (s, 3H), 0.91 (t, *J* = 7.5 Hz, 3H).

^{13}C NMR (126 MHz, $CDCl_3$) : δ 143.0, 140.7, 128.9, 127.7, 126.6, 118.5, 55.9, 42.2, 41.8, 40.2, 37.8, 32.1, 26.3, 13.1, 8.9.

HRMS ESI (m/z): $[M+H]^+$ calcd for $C_{17}H_{26}O_2NS$, 308.1684; found, 308.1676.

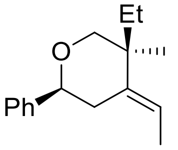

relative configuration

Supplementary Method A was followed. The 1,4-syn:anti ratio was determined by GC-Ms.

^{1}H NMR (400 MHz, $CDCl_3$) : δ 7.47 – 7.27 (m, 5H), 5.33 (q, *J* = 6.8 Hz, 1H), 4.27 (dd, *J* = 11.9, 2.9 Hz, 1H), 3.73 (dd, *J* = 10.9, 3.4 Hz, 1H), 3.35 (dd, *J* = 10.9, 3.5 Hz, 1H), 2.68 (dd, *J* = 14.2, 2.9 Hz, 1H), 2.18 – 2.04 (m, 1H), 1.68 (d, *J* = 6.7 Hz, 3H), 1.41 – 1.28 (m, 2H), 0.92 (s, 3H), 0.75 (t, *J* = 7.5 Hz, 3H).

^{13}C NMR (101 MHz, $CDCl_3$) : δ 143.0, 139.9, 128.5, 127.7, 126.1, 116.9, 81.0, 78.7, 40.4, 33.4, 28.8, 19.4, 12.9, 8.4.

HRMS ESI (m/z): $[M+H]^+$ calcd for $C_{16}H_{23}O$, 231.1749; found, 231.1743.

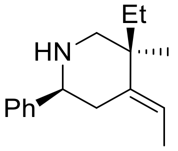

relative configuration

Supplementary Method A was followed except 2hrs addition time of reactant. The 1,4-syn:anti ratio was determined by NMR after mesylation of crude product.

^{1}H NMR (500 MHz, $CDCl_3$) : δ 7.49 – 7.28 (m, 5H), 5.27 (qd, *J* = 6.7, 1.9 Hz, 1H), 3.55 (dd, *J* = 11.9, 3.2 Hz, 1H), 2.83 (d, *J* = 11.7 Hz, 1H), 2.67 – 2.60 (m, 2H), 2.10 (dq, *J* = 15.1, 7.6 Hz, 1H), 2.04 – 1.95 (m, 1H), 1.62 (d, *J* = 6.6 Hz, 3H), 1.32 – 1.26 (m, 1H), 0.94 (s, 3H), 0.71 (t, *J* = 7.6 Hz, 3H).

^{13}C NMR (126 MHz, $CDCl_3$) : δ 141.5, 132.3, 128.6, 127.4, 126.9, 116.5, 62.8, 59.4, 39.8, 33.4, 28.9, 22.0, 13.0, 8.4.

HRMS ESI (m/z): $[M+H]^+$ calcd for $C_{16}H_{24}N$, 230.1909; found, 230.1902.

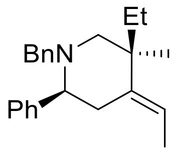

Supplementary Method A was followed.

^{1}H NMR (500 MHz, $CDCl_3$) : δ 7.51 (d, *J* = 7.5 Hz, 2H), 7.38 – 7.17 (m, 8H), 5.20 (qd, *J* = 6.7, 1.9 Hz, 1H), 3.76 (d, *J* = 13.8 Hz, 1H), 3.07 (dd, *J* = 11.6, 3.5 Hz, 1H), 2.74 (d, *J* = 13.8 Hz, 1H), 2.65 – 2.57 (m, 2H), 2.23 (dq, *J* = 15.2, 7.7 Hz, 1H), 2.19 – 2.12 (m, 1H), 1.77 (d, *J* = 11.3 Hz, 1H), 1.60 (dd, *J* = 6.6, 1.5 Hz, 3H), 1.33 – 1.27 (m, 1H), 0.84 (s, 3H), 0.63 (t, *J* = 7.5 Hz, 3H).

^{13}C NMR (126 MHz, $CDCl_3$) : δ 145.4, 141.7, 140.3, 128.7, 128.4, 128.1, 127.5, 127.2, 126.6, 115.5, 70.0, 65.2, 59.1, 40.5, 35.6, 29.7, 21.9, 13.0, 8.4.

HRMS ESI (m/z): $[M+H]^+$ calcd for $C_{23}H_{30}N$, 320.2378; found, 320.2366.

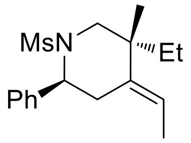

Supplementary Method A was followed.

^{1}H NMR (400 MHz, $CDCl_3$) : δ 7.48 – 7.41(m, 2H), 7.38 – 7.28 (m, 3H), 5.45 (q, *J* = 7.0 Hz, 1H), 5.20 (d, *J* = 6.6 Hz, 1H), 3.35 (d, *J* = 12.3 Hz, 1H), 2.92 (d, *J* = 12.3 Hz, 1H), 2.87 (d, *J* = 14.7 Hz, 1H), 2.64 – 2.52 (m, 1H), 2.41 (s, 3H), 1.99 (dt, *J* = 14.8, 7.2 Hz, 1H), 1.57 (d, *J* = 1.5 Hz, 3H), 1.36 (dq, *J* = 14.2, 7.3 Hz, 1H), 1.04 (s, 3H), 0.73 (t, *J* = 7.5 Hz, 3H).

^{13}C NMR (101 MHz, $CDCl_3$) : δ 140.0, 137.4, 128.8, 128.1, 128.0, 119.5, 56.6, 51.7, 40.2, 38.7, 29.0, 29.0, 21.7, 13.2, 8.3.

HRMS ESI (m/z): $[M+H]^+$ calcd for $C_{17}H_{26}NO_2S$, 308.1684; found, 308.1677.

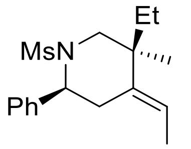

The typical mesylation procedure was followed.

^{1}H NMR (400 MHz, $CDCl_3$) : δ 7.52 – 7.26 (m, 5H), 5.46 (q, *J* = 7.2, 6.7 Hz, 1H), 4.96 (t, *J* = 6.5 Hz, 1H), 3.37 (d, *J* = 12.8 Hz, 1H), 3.19 (d, *J* = 12.7 Hz, 1H), 2.81 (dd, *J* = 14.4, 5.9 Hz, 1H), 2.65 (dd, *J* = 14.4, 6.4 Hz, 1H), 2.38 (s, 3H), 1.61 (d, *J* = 6.5 Hz, 3H), 1.61 – 1.51 (m, 2H), 1.21 (s, 3H), 0.90 (t, *J* = 8.2 Hz, 3H).

^{13}C NMR (101 MHz, $CDCl_3$) : δ 140.8, 139.5, 128.8, 128.1, 127.7, 118.5, 58.2, 51.5, 40.3, 39.8, 31.5, 30.8, 24.2, 13.4, 8.4.

HRMS ESI (m/z): $[M+H]^+$ calcd for $C_{17}H_{26}NO_2S$, 308.1684; found, 308.1677.

Table 3, Set 6

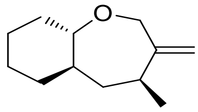

relative configuration

Supplementary Method A was followed.

^{1}H NMR (500 MHz, $CDCl_3$) : δ 4.85 (s, 1H), 4.83 (s, 1H), 4.39 (dt, J = 14.0, 2.1 Hz, 1H), 4.18 (d, J = 14.0 Hz, 1H), 2.94 (td, J = 10.2, 4.5 Hz, 1H), 2.63 – 2.56 (m, 1H), 1.97 – 1.92 (m, 1H), 1.78 – 1.73 (m, 2H), 1.71 – 1.65 (m, 2H), 1.42 – 1.36 (m, 1H), 1.25 – 1.22 (m, 1H), 1.18 (ddd, J = 7.8, 5.1, 2.4 Hz, 2H), 1.12 (d, J = 6.9 Hz, 3H), 1.08 – 1.00 (m, 1H) , 0.88 – 0.93 (m, 1H).

^{13}C NMR (126 MHz, $CDCl_3$) : δ 155.3, 107.5, 82.6, 74.6, 46.4, 45.2, 34.9, 34.1, 33.2, 25.6, 25.3, 21.3.

HRMS ESI (m/z): $[M+H]^+$ calcd for $C_{12}H_{21}O$, 181.1592; found, 181.1587.

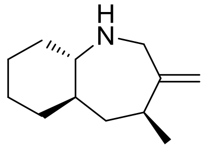

relative configuration

Supplementary Method A was followed except 2 hrs addition time of reactant. Due to serious ^{1}H NMR signal overlap, the 1,4-syn:anti ratio and relative configuration was determined after mesylation.

^{1}H NMR (400 MHz, $CDCl_3$) : δ 4.81 (s, 1H), 4.76 (s, 1H), 3.53 (d, J = 14.8 Hz, 1H), 3.44 (d, J = 14.8 Hz, 1H), 2.58 (ddt, J = 14.3, 10.8, 5.3 Hz, 1H), 2.09 (ddd, J = 11.6, 9.1, 3.8 Hz, 1H), 1.87 – 1.49 (m, 6H), 1.29 – 1.12 (m, 5H), 1.09 (d, J = 6.8 Hz, 3H), 1.03 – 0.95 (m, 1H).

^{13}C NMR (101 MHz, $CDCl_3$) : δ 156.6, 108.8, 63.7, 53.4, 47.3, 45.3, 36.8, 35.5, 34.4, 26.1, 26.0, 23.0.

HRMS ESI (m/z): $[M+H]^+$ calcd for $C_{12}H_{22}N$, 180.1752; found, 180.1746.

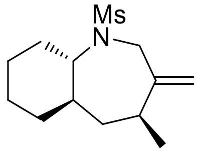

relative configuration

The typical mesylation procedure was followed.

^{1}H NMR (400 MHz, $CDCl_3$) : δ 5.08 (d, J = 2.1 Hz, 1H), 4.96 (t, J = 2.0 Hz, 1H), 4.24 (d, J = 15.5 Hz, 1H), 3.66 (d, J = 15.4 Hz, 1H), 3.02 (td, J = 10.8, 3.5 Hz, 1H), 2.80 (s, 3H), 2.24 – 2.13 (m, 1H), 2.11 – 2.04 (m, 1H), 1.83 – 1.67 (m, 3H), 1.62 – 1.57 (m, 1H), 1.54 (dd, J = 13.9, 3.8 Hz, 1H), 1.42 – 1.33 (m, 2H), 1.23 (ddd, J = 13.9, 10.5, 3.9 Hz, 3H), 1.12 (d, J = 6.6 Hz, 3H).

^{13}C NMR (101 MHz, $CDCl_3$) : δ 148.3, 113.7, 64.4, 49.1, 44.4, 43.1, 40.1, 39.0, 36.1, 34.6, 26.3, 25.7, 19.6.

HRMS ESI (m/z): $[M+H]^+$ calcd for $C_{13}H_{24}NO_2S$, 258.1528; found, 258.1522.

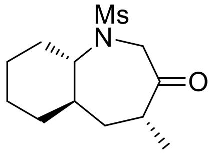

relative configuration

Supplementary Method A was followed. The 1,4-syn:anti ratio was determined by comparison of the crude NMR and mesylated NH product. Due to the desired product was hard to isolate from other byproducts, it was oxidized to corresponding ketone for structure determination.

^{1}H NMR (500 MHz, $CDCl_3$) : δ 4.12 (d, *J* = 18.8 Hz, 1H), 3.74 (td, *J* = 11.1, 3.8 Hz, 1H), 3.53 (d, *J* = 18.9 Hz, 1H), 3.51 – 3.44 (m,1H), 2.94 (s, 3H), 2.03 – 1.92 (m, 1H), 1.91 – 1.85 (m, 1H), 1.83 – 1.77 (m, 1H), 1.71 – 1.66 (m, 2H), 1.36 (t, *J* = 9.5 Hz, 3H), 1.19 – 1.08 (m, 3H), 1.01 (d, *J* = 6.6 Hz, 3H).

^{13}C NMR (126 MHz, $CDCl_3$) : δ 213.2, 61.3, 52.5, 42.0, 41.1, 39.7, 38.3, 34.2, 30.6, 25.4, 25.1, 15.9.

HRMS ESI (m/z): $[M+H]^+$ calcd for $C_{12}H_{22}NO_3S$, 260.1320; found, 260.1314.

Product analysis for D-labeling reductive hydroalkenylation

Figure 3a

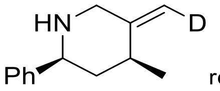

relative configuration

D$_1$-**3c**

Supplementary Method B was followed. The 1,3-syn:anti ratio was determined by GC-MS after mesylation of the crude product.

^{1}H NMR (500 MHz, $CDCl_3$) : δ 7.41 – 7.25 (m, 5H), 4.88 (d, *J* = 1.8 Hz, 1H), 3.86 (dd, *J* = 11.4, 2.6 Hz, 1H), 3.61 (d, *J* = 12.5 Hz, 1H), 3.43 (dt, *J* = 12.4, 1.3 Hz, 1H), 2.38 – 2.30 (m, 1H), 1.98 (ddd, *J* = 12.8, 4.6, 2.6 Hz, 1H), 1.82 (s, 1H), 1.32 (td, *J* = 12.5, 11.4 Hz, 1H), 1.12 (d, *J* = 6.6 Hz, 3H).

^{13}C NMR (126 MHz, $CDCl_3$) : δ 149.5, 144.2, 128.6, 127.3, 126.7, 106.3 (t, *J* = 23.9 Hz), 62.1, 54.8, 45.0, 36.2, 18.0.

HRMS ESI (m/z): $[M+H]^+$ calcd for $C_{13}H_{17}DN$, 189.1502; found, 189.1495.

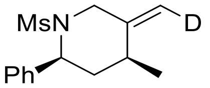

relative configuration

D$_1$-**4e**

The typical mesylation procedure was followed.

^{1}H NMR (400 MHz, $CDCl_3$) : δ 7.45 – 7.28 (m, 5H), 5.03 (d, *J* = 3.4 Hz, 1H), 4.85 (dd, *J* = 10.7, 6.4 Hz, 1H), 4.28 (d, *J* = 16.0 Hz, 1H), 4.13 (dt, *J* = 16.0, 1.8 Hz, 1H), 2.67 (s, 3H), 2.57 – 2.46 (m, 1H), 2.16 (ddd, *J* = 14.1, 6.4, 4.1 Hz, 1H), 1.76 (ddd, *J* = 14.1, 12.1, 10.6 Hz, 1H), 1.16 (d, *J* = 6.7 Hz, 3H).

^{13}C NMR (101 MHz, $CDCl_3$) : δ 147.0, 142.4, 128.8, 127.7, 126.3, 108.9 (t, *J* = 24.0 Hz), 59.0, 47.7, 39.5, 39.3, 33.0, 19.1.

HRMS ESI (m/z): $[M+H]^+$ calcd for $C_{14}H_{19}DNO_2S$, 267.1278; found, 267.1270.

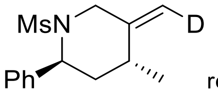

relative configuration

D$_1$-**4'e**

Supplementary Method B was followed. The 1,3-syn:anti ratio was determined by GC-MS.

^{1}H NMR (400 MHz, $CDCl_3$) : δ 7.48 (d, *J* = 8.3 Hz, 2H), 7.44 – 7.34 (m, 2H), 7.30 (d, *J* = 7.7 Hz, 1H), 5.26 (d, *J* = 5.7 Hz, 1H), 4.96 (s, 1H), 4.19 (d, *J* = 15.0 Hz, 1H), 3.62 (d, *J* = 15.0 Hz, 1H), 2.92 (s, 3H), 2.48 (ddd, *J* = 13.9, 4.2, 2.2 Hz, 1H), 2.42 – 2.30 (m, 1H), 1.73 (td, *J* = 13.3, 5.6 Hz, 1H), 1.14 (d, *J* = 6.5 Hz, 3H).

^{13}C NMR (126 MHz, $CDCl_3$) : δ 146.5, 138.5, 129.0, 127.4, 126.9, 108.5 (t, *J* = 23.6 Hz), 55.6, 48.7, 41.2, 37.1, 30.9, 18.0.

HRMS ESI (m/z): $[M+H]^+$ calcd for $C_{14}H_{19}DNO_2S$, 267.1278; found, 267.1270.

Product analysis for Acylation

Figure 3b

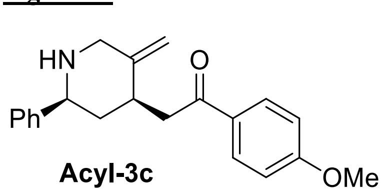

Acyl-3c relative configuration

Supplementary Method C was followed. The 1,3-syn:anti ratio was determined by GC-Ms after mesylation.

^{1}H NMR (400 MHz, $CDCl_3$) : δ 7.97 (d, *J* = 8.8 Hz, 2H), 7.35– 7.20 (m, 5H), 6.94 (d, *J* = 8.9 Hz, 2H), 4.94 (s, 1H), 4.69 (d, *J* = 1.8 Hz, 1H), 3.94 (dd, *J* = 11.3, 2.6 Hz, 1H), 3.87 (s, 3H), 3.64 (d, *J* = 12.5 Hz, 1H), 3.53 (d, *J* = 12.4 Hz, 1H), 3.32 (dd, *J* = 16.3, 5.7 Hz, 1H), 3.12 – 3.01 (m, 1H), 2.89 (dd, *J* = 16.3, 7.5 Hz, 1H), 2.07 (ddd, *J* = 12.7, 4.4, 2.7 Hz, 1H), 1.39 (q, *J* = 12.1 Hz, 1H).

^{13}C NMR (101 MHz, $CDCl_3$) : δ 197.7, 163.7, 147.6, 143.7, 130.5, 130.4, 128.6, 127.4, 126.8, 113.9, 107.1, 61.9, 55.6, 54.8, 42.6, 40.7, 37.7.

HRMS ESI (m/z): $[M+H]^+$ calcd for $C_{21}H_{24}NO_2$, 322.1802; found, 322.1799.

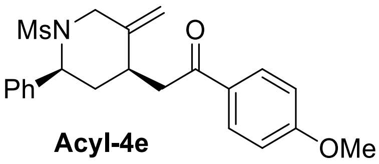

Acyl-4e relative configuration

The typical mesylation procedure was followed.

^{1}H NMR (400 MHz, $CDCl_3$) : δ 7.82 (d, *J* = 8.9 Hz, 2H), 7.37 – 7.20 (m, 5H), 6.90 (d, *J* = 8.9 Hz, 2H), 5.08 (s, 1H), 4.98 (s, 1H), 4.90 (dd, *J* = 9.4, 6.1 Hz, 1H), 4.30 (d, *J* = 15.8 Hz, 1H), 4.18 (d, *J* = 15.8 Hz, 1H), 3.86 (s, 3H), 3.20 – 3.14 (m, 1H), 3.09 (dd, *J* = 17.1, 4.7 Hz, 1H), 2.97 (dd, *J* = 17.1, 8.4 Hz, 1H), 2.75 (s, 3H), 2.30 – 2.22 (m, 1H) , 2.06 – 1.93 (m, 1H).

^{13}C NMR (101 MHz, $CDCl_3$) : δ 196.9, 163.8, 145.0, 142.0, 130.4, 130.0, 128.9, 127.6, 126.2, 113.9, 110.5, 58.2, 55.6, 47.7, 41.9, 39.6, 36.1, 34.4.

HRMS ESI (m/z): $[M+H]^+$ calcd for $C_{22}H_{26}NO_4S$, 400.1577; found, 400.1567.

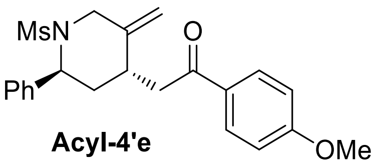

Acyl-4'e relative configuration

Supplementary Method C was followed. The 1,3-syn:anti ratio was determined by GC-Ms.

^{1}H NMR (400 MHz, $CDCl_3$) : δ 8.01 (d, *J* = 7.3 Hz, 2H), 7.60 (d, *J* = 7.6 Hz, 2H), 7.47 – 7.40 (m, 2H), 7.32 (t, *J* = 7.4 Hz, 1H), 6.99 (d, *J* = 7.3 Hz, 2H), 5.28 (d, *J* = 5.1 Hz, 1H), 5.05 (s, 1H), 4.83 (s, 1H), 4.24 (d, *J* = 15.1 Hz, 1H), 3.91 (s, 3H), 3.67 (d, *J* = 15.1 Hz, 1H), 3.32 (dd, *J* = 16.6, 3.7 Hz, 1H), 3.17 – 3.08 (m, 1H), 3.08 – 2.97 (m, 1H), 2.96 (s, 3H), 2.71 (dt, *J* = 14.6, 2.7 Hz, 1H), 1.82 – 2.97 (m, 1H).

^{13}C NMR (101 MHz, $CDCl_3$) : δ 196.9, 163.8, 145.0, 138.0, 130.4, 130.1, 128.9, 127.5, 127.1, 114.0, 109.0, 55.6, 55.5, 48.7, 41.2, 40.5, 34.4, 32.5.

HRMS ESI (m/z): $[M+H]^+$ calcd for $C_{22}H_{26}NO_4S$, 400.1577; found, 400.1580.

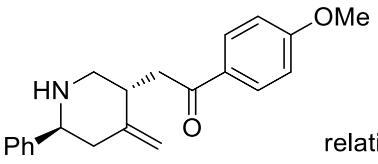

relative configuration

Acyl-5'c

Supplementary Method C was followed. The 1,4-syn:anti ratio was determined by GC-Ms after mesylation.

^{1}H NMR (500 MHz, $CDCl_3$) : δ 7.99 (d, *J* = 8.6 Hz, 2H), 7.38 (d, *J* = 7.6 Hz, 2H), 7.33 (t, *J* = 7.5 Hz, 2H), 7.30 – 7.22 (m, 1H), 6.95 (d, *J* = 8.5 Hz, 2H), 4.83 (s, 1H), 4.65 (s, 1H), 3.87 (s, 3H), 3.66 (dd, *J* = 11.5, 2.7 Hz, 1H), 3.39 – 3.26 (m, 2H), 3.02 – 2.90 (m, 1H), 2.81 (dd, *J* = 16.0, 7.8 Hz, 1H), 2.51 (ddd, *J* = 11.2, 6.1, 3.3 Hz, 2H), 2.35 (t, *J* = 12.2 Hz, 1H), 2.09 (s, 1H).

^{13}C NMR (126 MHz, $CDCl_3$) : δ 197.6, 163.6, 149.3, 144.0, 130.5, 130.2, 128.6, 127.5, 126.7, 113.9, 106.3, 63.6, 55.6, 53.7, 45.3, 39.2, 38.6.

HRMS ESI (m/z): $[M+H]^+$ calcd for $C_{21}H_{24}NO_2$, 322.18016; found, 322.17929.

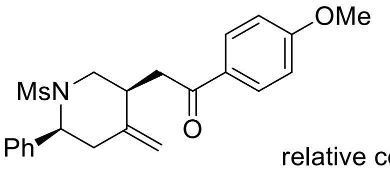

relative configuration

Acyl-6e

Supplementary Method C was followed. The 1,3-syn:anti ratio was determined by GC-Ms.

^{1}H NMR (500 MHz, $CDCl_3$) : δ 7.94 (d, *J* = 8.9 Hz, 2H), 7.49 – 7.42 (m, 2H), 7.35 (t, *J* = 7.6 Hz, 2H), 7.27 (t, *J* = 7.2 Hz, 1H), 6.94 (d, *J* = 8.9 Hz, 2H), 5.33 – 5.24 (m, 1H), 4.96 (s, 1H), 4.81 (s, 1H), 3.93 – 3.89 (m, 1H), 3.87 (s, 3H), 3.26 (dd, *J* = 17.0, 5.6 Hz, 1H), 3.15 (dq, *J* = 11.7, 5.7 Hz, 2H), 2.95 (dd, *J* = 14.1, 2.8 Hz, 1H), 2.89 (s, 3H), 2.86 – 2.80 (m, 2H).

^{13}C NMR (126 MHz, $CDCl_3$) : δ 196.2, 163.9, 143.7, 139.2, 130.5, 129.9, 128.7, 127.7, 127.6, 114.0, 111.0, 56.6, 55.7, 46.5, 40.4, 38.7, 38.0, 37.5.

HRMS ESI (m/z): $[M+H]^+$ calcd for $C_{22}H_{26}NO_4S$, 400.1577; found, 400.1576.

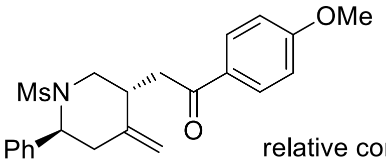

relative configuration

Acyl-6'e

The typical mesylation procedure was followed.

^{1}H NMR (500 MHz, $CDCl_3$) : δ 7.97 (d, J = 8.7 Hz, 2H), 7.50 (d, J = 7.5 Hz, 2H), 7.40 – 7.29 (m, 3H), 6.93 (d, J = 8.7 Hz, 2H), 5.27 (d, J = 7.0 Hz, 1H), 5.06 (d, J = 2.1 Hz, 1H), 4.92 (d, J = 1.9 Hz, 1H), 3.87 (s, 3H), 3.69 (d, J = 12.9 Hz, 1H), 3.59 (dd, J = 17.3, 8.4 Hz, 1H), 3.36 – 3.30 (m, 1H), 3.30 – 3.26 (m, 1H), 3.07 (dd, J = 17.4, 4.9 Hz, 1H), 3.04 – 3.00 (m, 1H), 2.68 – 2.63 (m, 1H), 2.50 (s, 3H).

^{13}C NMR (126 MHz, $CDCl_3$) : δ 197.2, 163.6, 143.8, 139.2, 130.5, 130.3, 128.8, 128.2, 128.2, 113.9, 113.5, 56.2, 55.6, 44.5, 39.8, 39.2, 37.9, 35.6.

HRMS ESI (m/z): $[M+H]^+$ calcd for $C_{22}H_{26}NO_4S$, 400.1577; found, 400.1575.

Product analysis for Silylation

Figure 3c

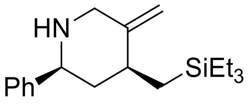
relative configuration

Si-3c

Supplementary Method D was followed. The 1,3-syn:anti ratio was determined by GC-Ms after mesylation.

^{1}H NMR (500 MHz, $CDCl_3$) : δ 7.41 – 7.22 (m, 5H), 4.92 (s, 1H), 4.87 (s, 1H), 3.87 (dd, J = 11.4, 2.9 Hz, 1H), 3.61 (d, J = 12.4 Hz, 1H), 3.41 (d, J = 12.2 Hz, 1H), 2.38 – 2.31 (m, 1H), 2.05 (dt, J = 12.7, 3.6 Hz, 1H), 1.35 (q, J = 12.1 Hz, 1H), 1.04 – 0.90 (m, 10H), 0.61 – 0.51 (m, 7H).

^{13}C NMR (126 MHz, $CDCl_3$) : δ 150.6, 143.8, 128.6, 127.4, 126.8, 107.1, 62.3, 55.0, 45.5, 38.1, 14.3, 7.7, 4.3.

HRMS-ESI (m/z): $[M+H]^+$ calcd for $C_{19}H_{31}NSi$, 302.2304 ; found, 302.2296.

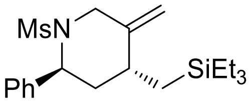
relative configuration

Si-4'e

Supplementary Method D was followed. The 1,3-syn:anti ratio was determined by GC-Ms.

^{1}H NMR (500 MHz, $CDCl_3$) : δ 7.47 (d, *J* = 8.2 Hz, 2H), 7.40 (t, *J* = 7.8 Hz, 2H), 7.29 (t, *J* = 7.2 Hz, 1H), 5.24 (d, *J* = 5.8 Hz, 1H), 5.01 (s, 1H), 4.93 (s, 1H), 4.22 (d, *J* = 15.0 Hz, 1H), 3.65 (d, *J* = 14.8 Hz, 1H), 2.91 (s, 3H), 2.55 – 2.49 (m, 1H), 2.36 – 2.27 (m, 1H), 1.77 – 1.69 (m, 1H), 0.99 – 0.88 (m, 10H), 0.62 – 0.50 (m, 7H).

^{13}C NMR (126 MHz, $CDCl_3$) : δ 147.7, 138.5, 128.8, 127.3, 126.7, 109.1, 55.7, 49.0, 41.0, 38.2, 32.6, 14.2, 7.5, 4.1.

HRMS-ESI (m/z): $[M+H]^+$ calcd for $C_{20}H_{34}NSi$, 380.2076 ; found, 380.2068.

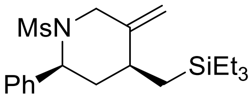
relative configuration

Si-4e

The typical mesylation procedure was followed.

^{1}H NMR (500 MHz, $CDCl_3$) : δ 7.42 – 7.26 (m, 5H), 5.01 (d, *J* = 8.2 Hz, 2H), 4.82 (dd, *J* = 9.6, 6.3 Hz, 1H), 4.24 (d, *J* = 15.6 Hz, 1H), 4.12 (dq, *J* = 15.6, 1.5 Hz, 1H), 2.63 (s, 3H), 2.55 – 2.46 (m, 1H), 2.19 (ddd, *J* = 14.0, 6.4, 4.3 Hz, 1H), 1.80 (ddd, *J* = 14.0, 11.1, 9.7 Hz, 1H), 0.91 (t, *J* = 7.9 Hz, 10H), 0.62 – 0.47 (m, 7H).

^{13}C NMR (126 MHz, $CDCl_3$) : δ 148.3, 142.1, 128.9, 127.6, 126.5, 109.4, 58.7, 47.7, 39.7, 39.6, 34.9, 15.9, 7.6, 4.1.

HRMS-ESI (m/z): $[M+H]^+$ calcd for $C_{20}H_{34}NSi$, 380.2076 ; found, 380.2071.

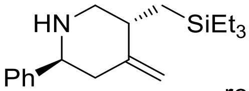

relative configuration

Si-5'c

Supplementary Method D was followed. The 1,4-syn:anti ratio was determined by NMR after mesylation.

^{1}H NMR (400 MHz, $CDCl_3$) : δ 7.42 – 7.30 (m, 4H), 7.29 – 7.23 (m, 1H), 4.81 (d, *J* = 1.7 Hz, 1H), 4.80 (s, 1H), 3.62 (dd, *J* = 11.4, 2.9 Hz, 1H), 3.28 (dd, *J* = 10.9, 4.5 Hz, 1H), 2.49 (dd, *J* = 12.7, 2.9 Hz, 1H), 2.41 (t, *J* = 10.9 Hz, 1H), 2.28 (t, *J* = 11.8 Hz, 2H), 1.79 (s, 1H), 0.95 (t, *J* = 7.9 Hz, 9H), 0.93 – 0.86 (m, 1H), 0.63 – 0.52 (m, 6H), 0.48 (dd, *J* = 14.9, 8.2 Hz, 1H).

^{13}C NMR (101 MHz, $CDCl_3$) : δ 152.3, 144.4, 128.6, 127.4, 126.8, 106.2, 63.8, 56.7, 45.5, 39.3, 11.4, 7.7, 4.2.

HRMS-ESI (m/z): $[M+H]^+$ calcd for $C_{19}H_{32}NSi$, 302.2304 ; found, 302.2296.

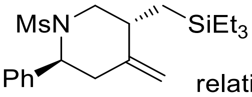

relative configuration

Si-6'e

The typical mesylation procedure was followed.

^{1}H NMR (400 MHz, $CDCl_3$) : δ 7.52 – 7.47 (m, 2H), 7.37 – 7.28 (m, 3H), 5.19 (d, *J* = 6.7 Hz, 1H), 4.95 (s, 1H), 4.79 (s, 1H), 3.48 (d, *J* = 13.6 Hz, 1H), 3.33 (dd, *J* = 12.5, 3.3 Hz, 1H), 3.08 (ddt, *J* = 14.2, 7.1, 2.1 Hz, 1H), 2.68 – 2.61 (m, 1H), 2.49 (d, *J* = 14.1 Hz, 1H), 2.45 (s, 3H), 1.02 (dd, *J* = 11.6, 6.9 Hz, 2H), 0.95 (t, *J* = 7.9 Hz, 9H), 0.57 (qd, *J* = 7.9, 1.7 Hz, 6H).

^{13}C NMR (101 MHz, $CDCl_3$) : δ 146.3, 139.3, 128.7, 128.3, 128.0, 111.6, 56.7, 48.5, 38.8, 38.6, 34.7, 14.7, 7.6, 3.6.

^{13}C NMR (101 MHz, $CDCl_3$) : δ 146.3, 139.3, 128.7, 128.3, 128.0, 111.6, 56.7, 48.5, 38.8, 38.6, 34.7, 14.7, 7.6, 3.6.

HRMS-ESI (m/z): $[M+H]^+$ calcd for $C_{20}H_{34}NO_2SSi$, 380.2080 ; found, 380.2071.

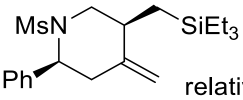

relative configuration

Si-6e

Supplementary Method D was followed. The 1,4-syn:anti ratio was determined by NMR.

^{1}H NMR (400 MHz, $CDCl_3$) : δ 7.47 – 7.27 (m, 5H), 5.19 (t, *J* = 4.6 Hz, 1H), 4.97 (s, 1H), 4.92 (s, 1H), 3.81 (dd, *J* = 12.0, 5.3 Hz, 1H), 2.83 (d, *J* = 4.2 Hz, 2H), 2.74 – 2.68 (m, 1H), 2.66 (s, 3H), 2.52 – 2.46 (m, 1H), 0.95 (t, *J* = 7.9 Hz, 9H), 0.98 – 0.89 (m, 1H), 0.63 – 0.55 (m, 6H), 0.46 (dd, *J* = 15.0, 8.3 Hz, 1H).

^{13}C NMR (101 MHz, $CDCl_3$) : δ 146.6, 139.6, 128.7, 127.8, 127.7, 110.7, 56.8, 49.1, 39.9, 39.3, 38.5, 11.8, 7.6, 4.1.

HRMS-ESI (m/z): $[M+H]^+$ calcd for $C_{20}H_{34}NO_2SSi$, 380.2080 ; found, 380.2072.

Product analysis for synthetic application

Figure 4

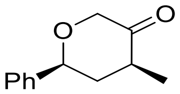

relative configuration

The ketone was prepared analogously to the literature procedure. [14] 0.94g **3a** was treated with 3.2g $NaIO_4$ and 50 mg $RuCl_3 \cdot 2H_2O$. The ketone was purified by silica gel chromatography, eluting with EA/hexane=1:8, as a light-yellow oil (0.79 g, 83% yield).

^{1}H NMR (400 MHz, $CDCl_3$) : δ 7.54 – 7.30 (m, 5H), 4.90 (dt, *J* = 11.2, 1.7 Hz, 1H), 4.28 (d, *J* = 15.3 Hz, 1H), 4.19 (d, *J* = 15.3 Hz, 1H), 2.76 (dp, *J* = 13.0, 6.6 Hz, 1H), 2.46 – 2.39 (m, 1H), 2.03 – 1.88 (m, 1H), 1.20 (d, *J* = 6.7 Hz, 3H).

^{13}C NMR (101 MHz, $CDCl_3$) : δ 208.6, 141.0, 128.7, 128.0, 125.9, 78.8, 74.5, 42.8, 42.2, 14.2.

HRMS ESI (m/z): $[M+H]^+$ calcd for $C_{12}H_{15}O_2$, 191.1072; found, 191.1064.

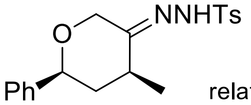

relative configuration

The hydrazide was prepared according to the literature procedure.[15] 380 mg Ketone was dissolved in 15 mL diethyl ether. To the solution, 372 mg of 4-Methylbenzenesulfonhydrazide was added. The mixture was stirred at r.t. overnight, then placed at -40 ^{o}C. The desired hydrazide was obtained by filtration (640 mg, 91% yield).

^{1}H NMR (500 MHz, $CDCl_3$): δ 7.86 – 7.82 (m, 2H), 7.32 – 7.18 (m, 7H), 4.77 (dd, *J* = 14.6, 2.8 Hz, 1H), 4.53 (dd, *J* = 11.3, 2.4 Hz, 1H), 3.79 (dd, *J* = 14.6, 4.1 Hz, 1H), 2.62 (dq, *J* = 12.4, 6.1 Hz, 1H), 2.42 (s, 3H), 2.09 (ddd, *J* = 13.4, 5.1, 2.4 Hz, 1H), 1.59 (q, *J* = 12.4, 11.7 Hz, 1H), 1.13 (d, *J* = 6.4 Hz, 3H).

^{13}C NMR (126 MHz, $CDCl_3$): δ 159.7, 144.2, 141.1, 135.0, 129.5, 128.6, 128.5, 127.9, 125.9, 79.2, 63.4, 42.5, 36.7, 21.7, 16.4.

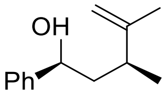

relative configuration

36 mg hydrazide was suspended in 5 mL dried toluene and cooled to -78 ^{o}C. Then 0.4 mL MeLi (1 M in diethyl ether) was added drop wise. The mixture was stirred at r.t. overnight. 10 mL aqueous NH_4Cl was added, then extracted with EA, washed with brine, and dried over $MgSO_4$. The alkenol was obtained by silica gel chromatography eluted with EA/hexane=1:6 (85% yield by NMR).

^{1}H NMR (500 MHz, $CDCl_3$) : δ 7.38 – 7.26 (m, 5H), 4.75 (d, *J* = 1.2 Hz, 2H), 4.68 (t, *J* = 7.2 Hz, 1H), 2.32 – 2.21 (m, 1H), 2.01 – 1.92 (m, 2H), 1.73 (s, 3H), 1.64 (dt, *J* = 13.9, 6.1 Hz, 1H), 1.04 (d, *J* = 6.9 Hz, 3H).

^{13}C NMR (126 MHz, $CDCl_3$) : δ 150.4, 145.0, 128.6, 127.7, 126.2, 110.3, 73.4, 44.5, 38.5, 20.0, 19.0.

HRMS ESI (m/z): $[M+H-H_2O]^+$ calcd for $C_{13}H_{17}$, 173.1330; found, 173.1325.

Supplementary References

1 Arduengo, A. J. *et al.* Imidazolylidenes, imidazolinylidenes and imidazolidines. *Tetrahedron* **55**, 14523-14534 (1999).

2 Ryan, S. J., Schimler, S. D., Bland, D. C. & Sanford, M. S. Acyl Azolium Fluorides for Room Temperature Nucleophilic Aromatic Fluorination of Chloro- and Nitroarenes. *Org. Lett.* **17**, 1866-1869 (2015).

3 Zhao, J.-P., Chan, S.-C. & Ho, C.-Y. Substituted 1,3-cyclohexadiene synthesis by NHC-Nickel(0) catalyzed 2+2+2 cycloaddition of 1,n-Enyne. *Tetrahedron* **71**, 4426-4431 (2015).

4 Zhao, L.-M. *et al.* Zinc-mediated α-regioselective Barbier-type cinnamylation reactions of aldehydes, ketones and esters. *Org. Biomol. Chem.* **15**, 4359-4366 (2017).

5 Fan, R., Pu, D., Wen, F., Ye, Y. & Wang, X. A Facile Synthesis of N-Sulfonyl and N-Sulfinyl Aldimines under Barbier-Type Conditions. *J. Org. Chem.* **73**, 3623-3625 (2008).

6 Ghorai, M. K., Das, S., Das, K. & Kumar, A. Stereoselective synthesis of activated 2-arylazetidines via imino-aldol reaction. *Org. Biomol. Chem.* **13**, 9042-9049 (2015).

7 Shikora, J. M. & Chemler, S. R. Synthesis of Benzyl Amines via Copper-Catalyzed Enantioselective Aza-Friedel–Crafts Addition of Phenols to N-Sulfonyl Aldimines. *Org. Lett.* **20**, 2133-2137 (2018).

8 Chang, Z., Jing, X., He, C., Liu, X. & Duan, C. Silver Clusters as Robust Nodes and π–Activation Sites for the Construction of Heterogeneous Catalysts for the Cycloaddition of Propargylamines. *ACS Catal.* **8**, 1384-1391 (2018).

9 Martzel, T., Lohier, J.-F., Gaumont, A.-C., Brière, J.-F. & Perrio, S. Sulfinate-Organocatalyzed (3+2) Annulation Reaction of Propargyl or Allenyl Sulfones with Activated Imines. *Eur. J. Org. Chem.* **2018**, 5069-5073 (2018).

10 Saavedra-Olavarría, J., Arteaga, G. C., López, J. J. & Pérez, E. G. Copper-catalyzed intermolecular and regioselective aminofluorination of styrenes: facile access to β-fluoro-N-protected phenethylamines. *Chem. Commun.* **51**, 3379-3382 (2015).

11 Wen, K., Wu, Z., Chen, B., Chen, J. & Zhang, W. Pd(ii)-Catalyzed aerobic 1,2-difunctionalization of conjugated dienes: efficient synthesis of morpholines and 2-morpholones. *Org. Biomol. Chem.* **16**, 5618-5625 (2018).

12 Tatton, M. R., Simpson, I. & Donohoe, T. J. De Novo Synthesis of Multisubstituted Aryl Amines Using Alkene Cross Metathesis. *Org. Lett.* **16**, 1920-1923 (2014).

13 Nicolai, S., Sedigh-Zadeh, R. & Waser, J. Pd(0)-Catalyzed Alkene Oxy- and Aminoalkynylation with Aliphatic Bromoacetylenes. *J. Org. Chem.* **78**, 3783-3801 (2013).

14 Kawashima, H., Kaneko, Y., Sakai, M. & Kobayashi, Y. Synthesis of Cyclobakuchiols A, B, and C by Using Conformation-Controlled Stereoselective Reactions. *Chem. Eur. J.* **20**, 272-278 (2014).

15 Hecker, S. J. & Heathcock, C. H. TOTAL SYNTHESIS OF (+)-DIHYDROMEVINOLIN. *J. Am. Chem. Soc.* **108**, 4586-4594 (1986).
