# Supplementary material for: NHC-Ni catalyzed 1,3- and 1,4-diastereodivergent heterocycle synthesis from hetero-substituted enyne
Source: Commun Chem. 2020 Apr 30;3:50. doi: 10.1038/s42004-020-0299-9 (PMC9814851; doi:10.1038/s42004-020-0299-9)
Supplement: Supplementary file 2 — Description of Additional Supplementary Files [file 42004_2020_299_MOESM2_ESM.pdf]

Supplementary Data 1. Source data underlying the study.
