# Supplementary material for: NHC-Ni catalyzed 1,3- and 1,4-diastereodivergent heterocycle synthesis from hetero-substituted enyne
Source: Commun Chem. 2020 Apr 30;3:50. doi: 10.1038/s42004-020-0299-9 (PMC9814851; doi:10.1038/s42004-020-0299-9)

Parameter	Value
Data File Name	E:/ NMR/ 2017/ 2017-5(10-17)/ xfy-0517-1/ 1/ fid
Comment	
Origin	Bruker BioSpin GmbH
Owner	nmr
Site	
Instrument	spect
Solvent	CDCl3
Temperature	295.3
Pulse Sequence	zg30
Experiment	1D
Number of Scans	8
Receiver Gain	87.6
Relaxation Delay	1.0000
Pulse Width	9.6000
Presaturation Frequency	
Acquisition Time	1.9999
Class	
Spectrometer Frequency	400.13
Spectral Width	8012.8
Lowest Frequency	-1545.8
Nucleus	1H
Acquired Size	16025
Spectral Size	32768

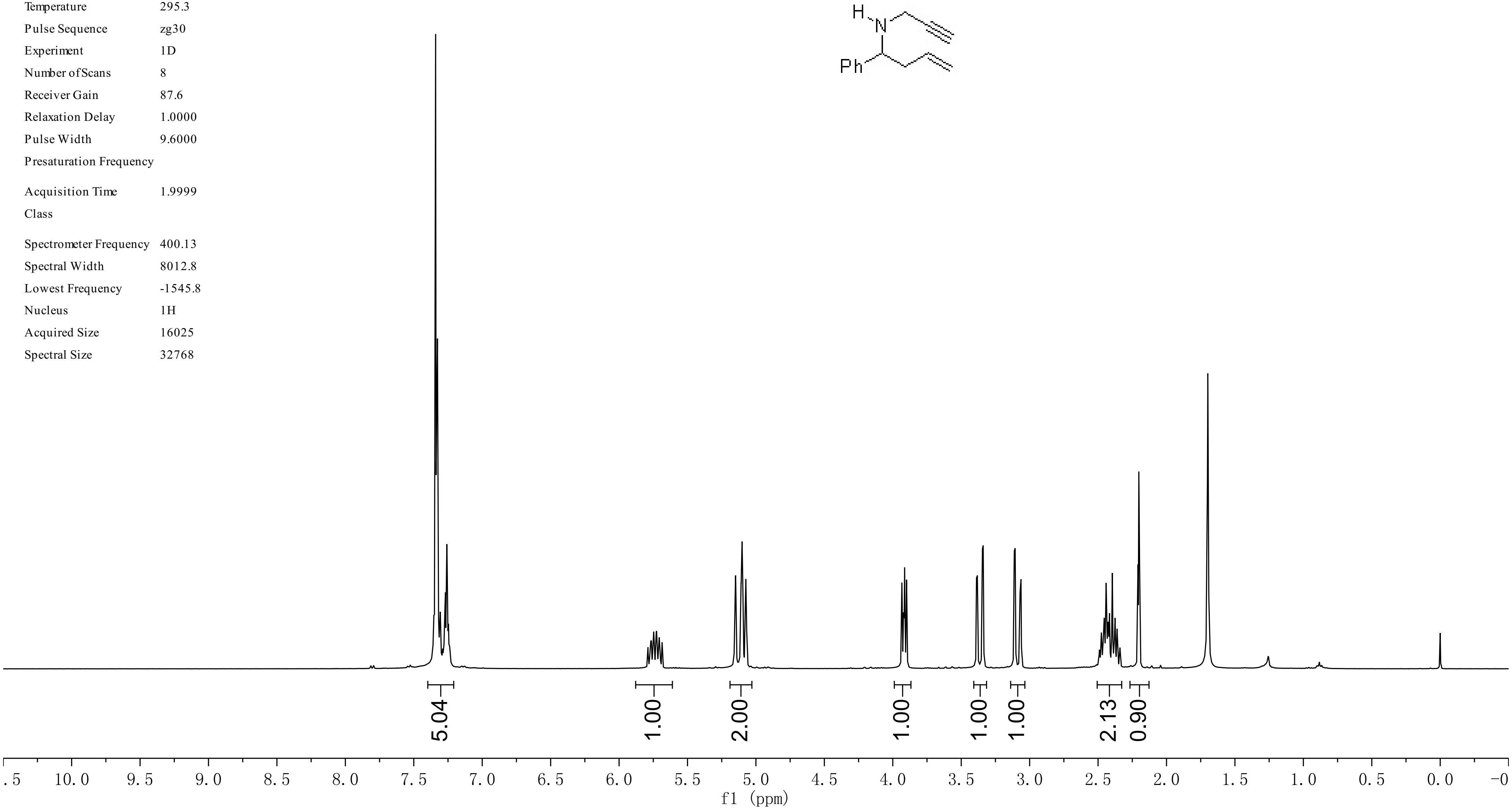

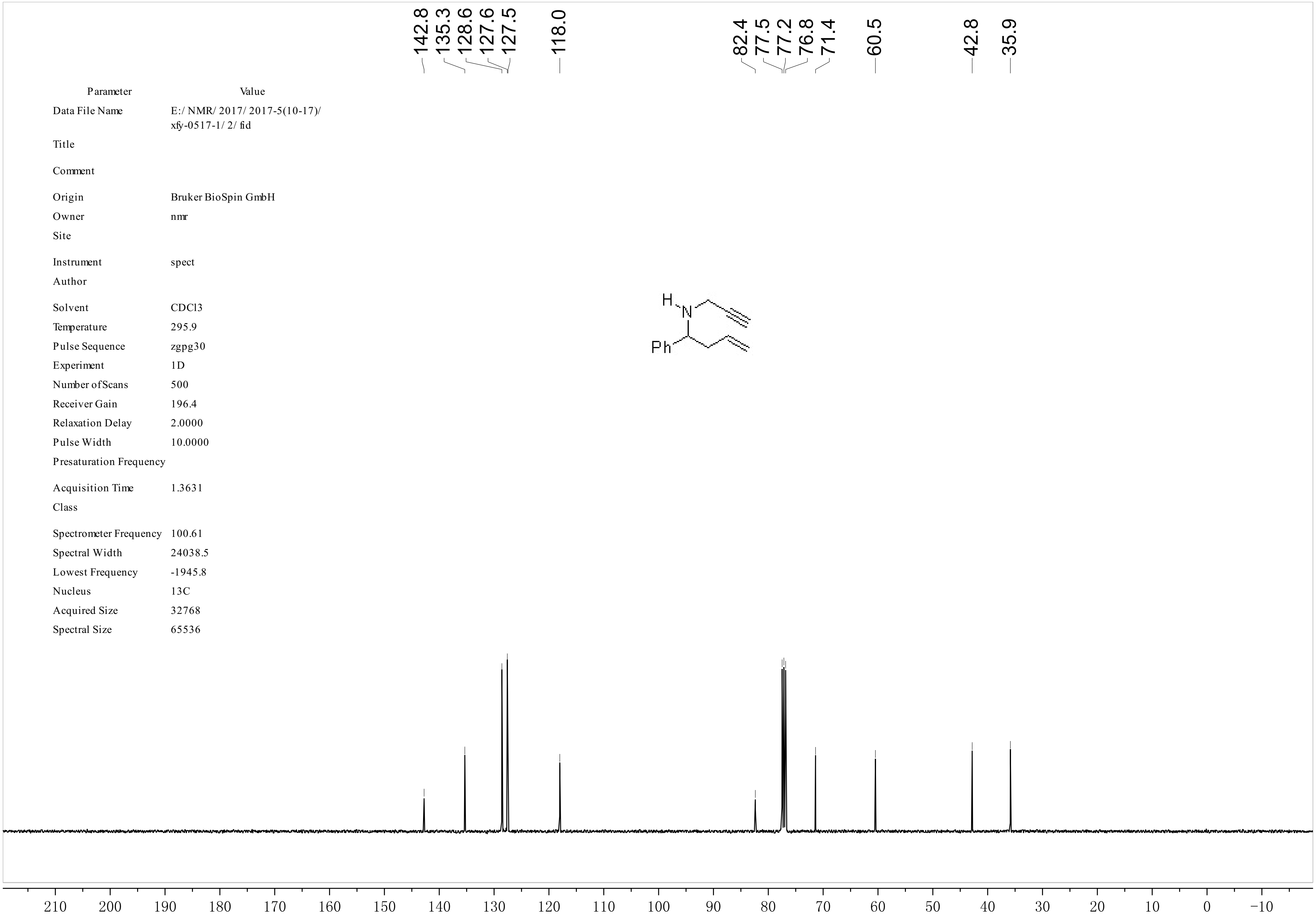
142.8
135.3
128.6
127.6
127.5
118.0
82.4
77.5
77.2
76.8
71.4
60.5
42.8
35.9
Parameter
Value
Data File Name
E:/ NMR/ 2017/ 2017-5(10-17)/ xfy-0517-1/ 2/ fid
Title
Comment
Origin
Bruker BioSpin GmbH
Owner
nmr
Site
Instrument
spect
Author
Solvent
CDCl3
Temperature
295.9
Pulse Sequence
zgpg30
Experiment
1D
Number of Scans
500
Receiver Gain
196.4
Relaxation Delay
2.0000
Pulse Width
10.0000
Presaturation Frequency
Acquisition Time
1.3631
Class
Spectrometer Frequency
100.61
Spectral Width
24038.5
Lowest Frequency
-1945.8
Nucleus
13C
Acquired Size
32768
Spectral Size
65536
H
N
Ph
210 200 190 180 170 160 150 140 130 120 110 100 90 80 70 60 50 40 30 20 10 0 −10

Parameter	Value
Data File Name	E:/ NMR/ 2018/ July/ 2018-7-11/ xfy-0525-3/ 21/ fid
Title	
Comment	
Origin	Bruker BioSpin GmbH
Owner	nmr
Site	
Instrument	spect
Author	
Solvent	CDCl3
Temperature	296.1
Pulse Sequence	zg30
Experiment	1D
Number of Scans	7
Receiver Gain	77.6
Relaxation Delay	1.0000
Pulse Width	10.7100
Presaturation Frequency	
Acquisition Time	3.2768
Class	
Spectrometer Frequency	500.13
Spectral Width	10000.0
Lowest Frequency	-1922.1
Nucleus	1H
Acquired Size	32768
Spectral Size	65536

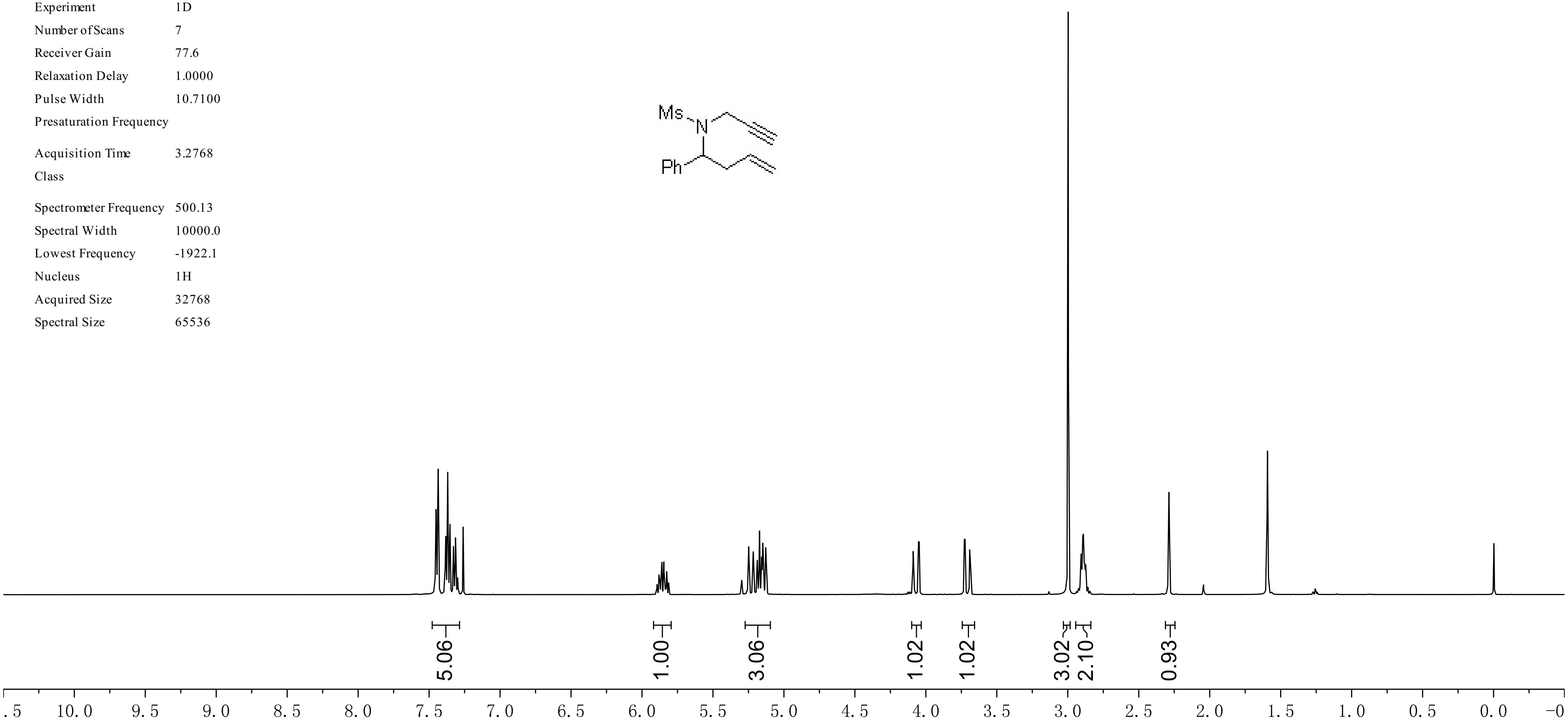

Parameter	Value
Data File Name	E:/ NMR/ 2018/ July/ 2018-7-11/ xfy-0525-3/ 22/ fid
Title	
Comment	
Origin	Bruker BioSpin GmbH
Owner	nmr
Site	
Instrument	spect
Author	
Solvent	CDCl3
Temperature	296.1
Pulse Sequence	zgpg30
Experiment	1D
Number of Scans	42
Receiver Gain	193.1
Relaxation Delay	2.0000
Pulse Width	9.6000
Presaturation Frequency	
Acquisition Time	1.1010
Class	
Spectrometer Frequency	125.77
Spectral Width	29761.9
Lowest Frequency	-2290.4
Nucleus	13C
Acquired Size	32768
Spectral Size	65536

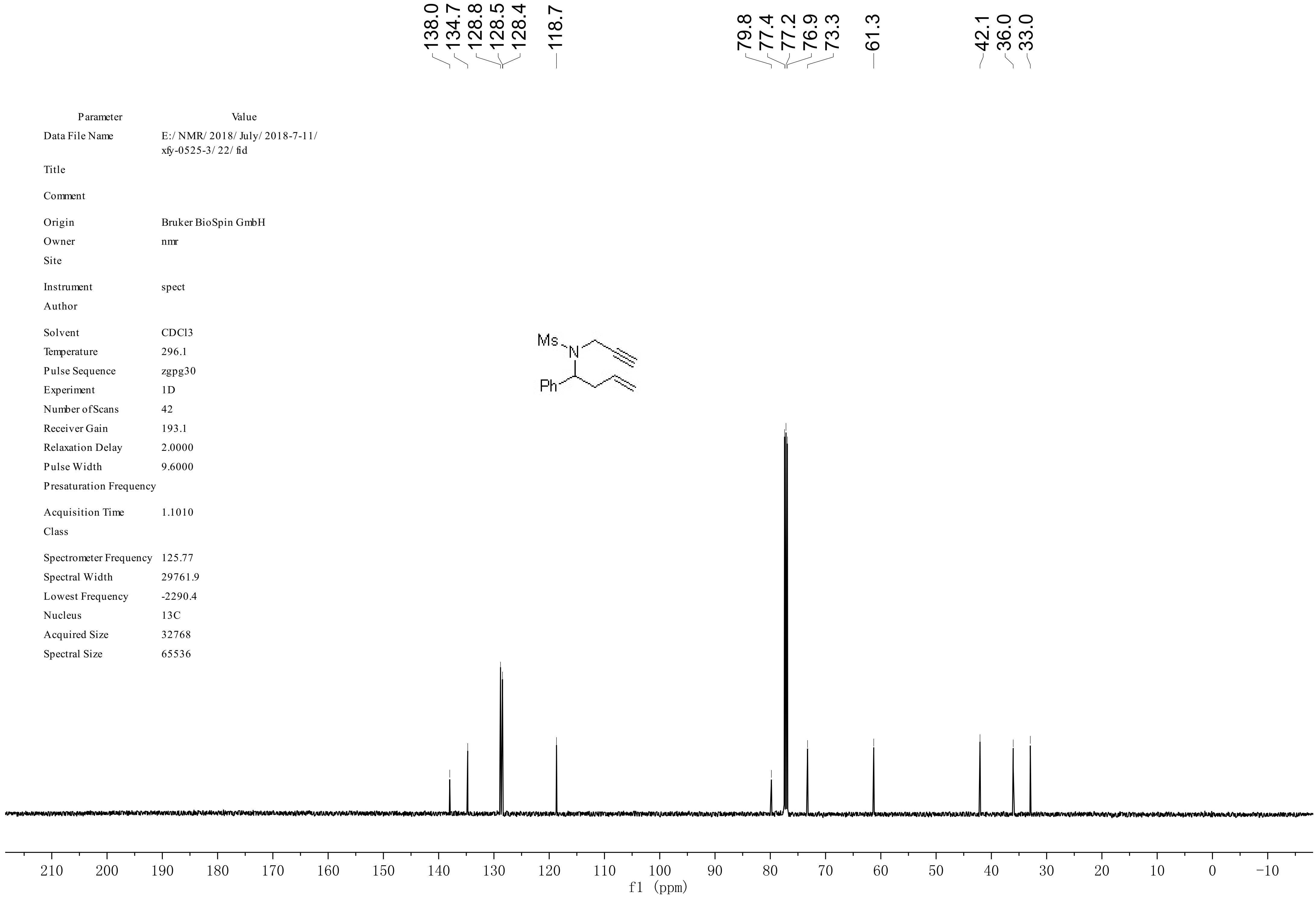

Parameter	Value
Data File Name	E:/ NMR/ 2017/ 2017-4(20-27)/ xfy-0425-x/ 4/ fid
Comment	
Origin	Bruker BioSpin GmbH
Owner	nmr
Site	
Instrument	spect
Solvent	CDCl3
Temperature	294.8
Pulse Sequence	zg30
Experiment	1D
Number of Scans	13
Receiver Gain	34.9
Relaxation Delay	1.0000
Pulse Width	9.6000
Presaturation Frequency	
Acquisition Time	1.9999
Class	
Spectrometer Frequency	400.13
Spectral Width	8012.8
Lowest Frequency	-1545.8
Nucleus	1H
Acquired Size	16025
Spectral Size	32768

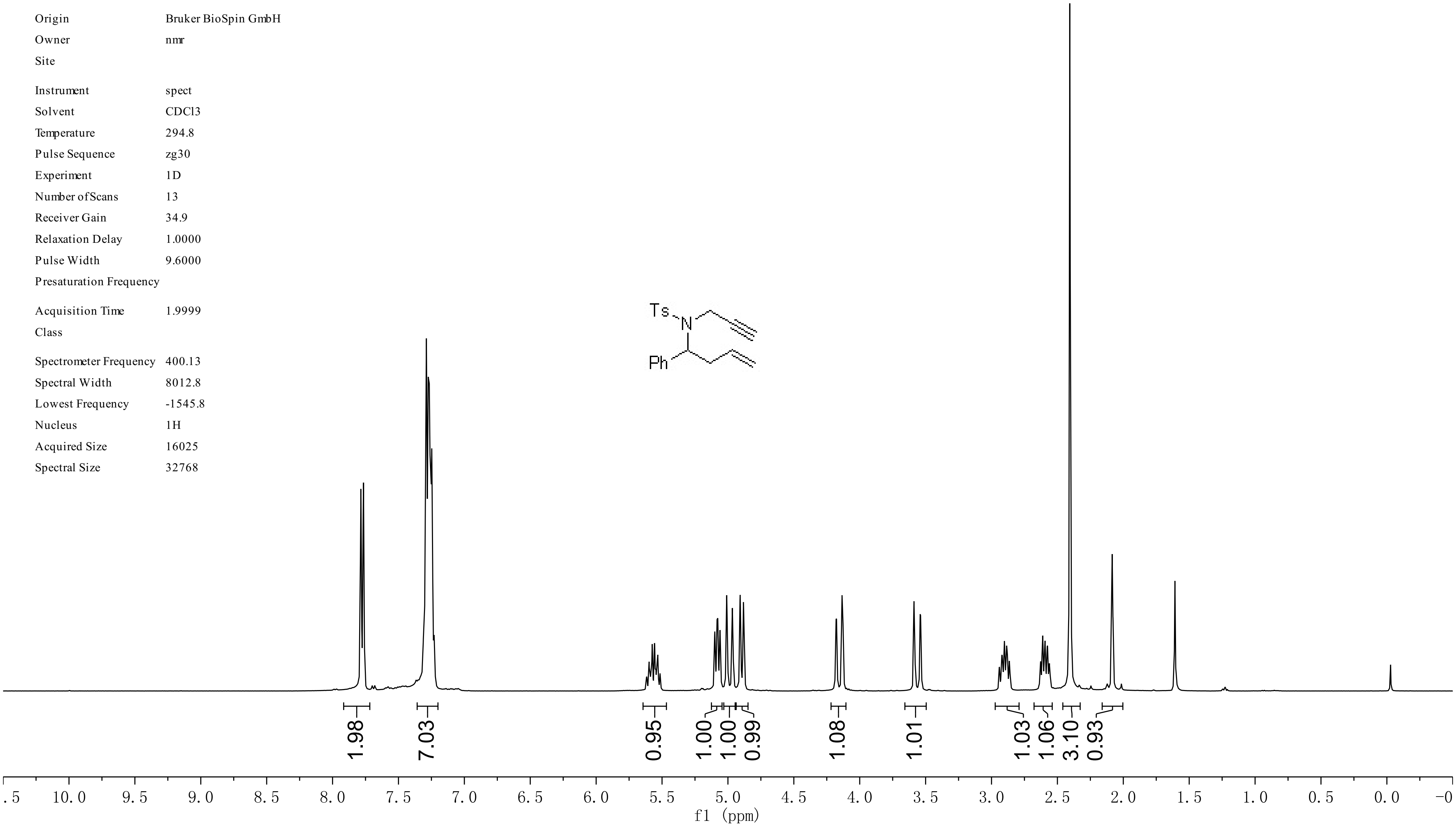

Parameter	Value
Data File Name	E:/ NMR/ 2017/ 2017-4(20-27)/ xfy-0425-x/ 5/ fid
Comment	
Origin	Bruker BioSpin GmbH
Owner	nmr
Site	
Instrument	spect
Solvent	CDCl3
Temperature	294.9
Pulse Sequence	zgpg30
Experiment	1D
Number of Scans	18
Receiver Gain	196.4
Relaxation Delay	2.0000
Pulse Width	10.0000
Presaturation Frequency	
Acquisition Time	1.3631
Class	
Spectrometer Frequency	100.61
Spectral Width	24038.5
Lowest Frequency	-1950.2
Nucleus	13C
Acquired Size	32768
Spectral Size	65536

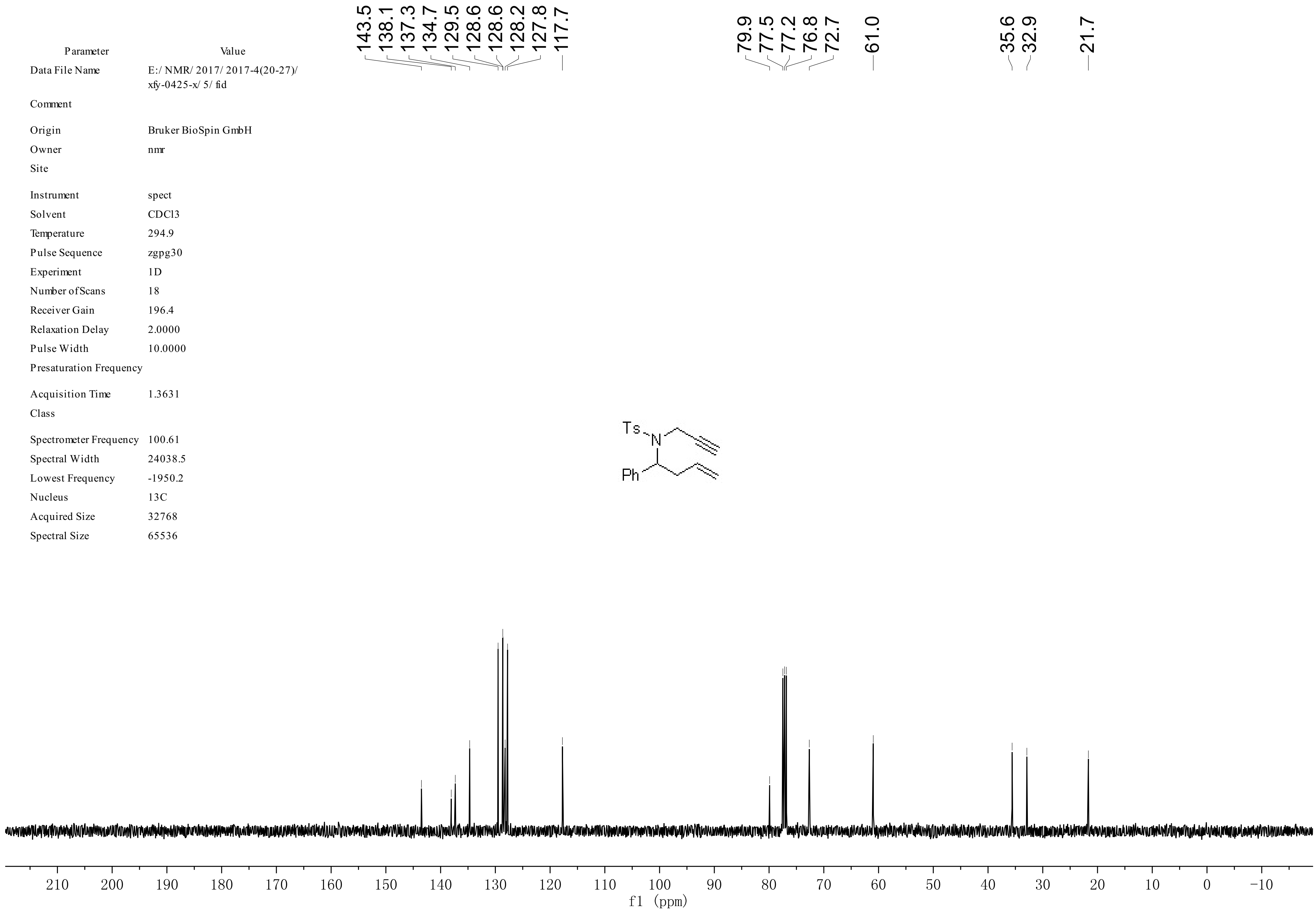

Parameter	Value
Data File Name	E:/ NMR/ 2017/ 2017-7(6-28-7-5)/ xfy-0629-1/ 1/ fid
Comment	
Origin	Bruker BioSpin GmbH
Owner	nmr
Site	
Instrument	spect
Solvent	CDCl3
Temperature	296.0
Pulse Sequence	zg30
Experiment	1D
Number of Scans	8
Receiver Gain	87.6
Relaxation Delay	1.0000
Pulse Width	9.6000
Presaturation Frequency	
Acquisition Time	1.9999
Class	
Spectrometer Frequency	400.13
Spectral Width	8012.8
Lowest Frequency	-1535.4
Nucleus	1H
Acquired Size	16025
Spectral Size	32768

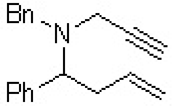

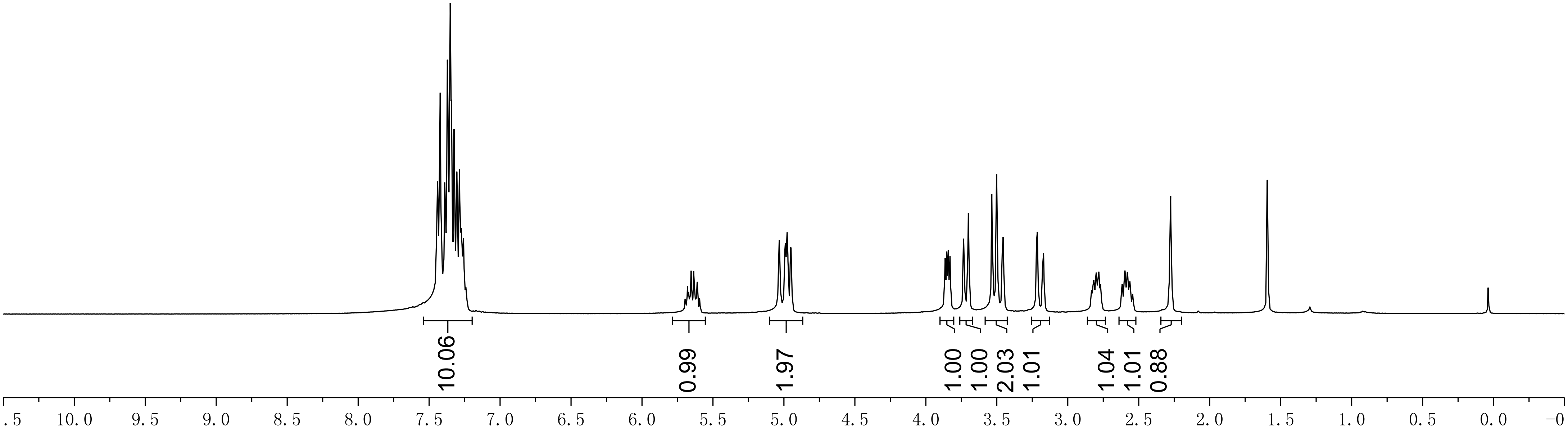

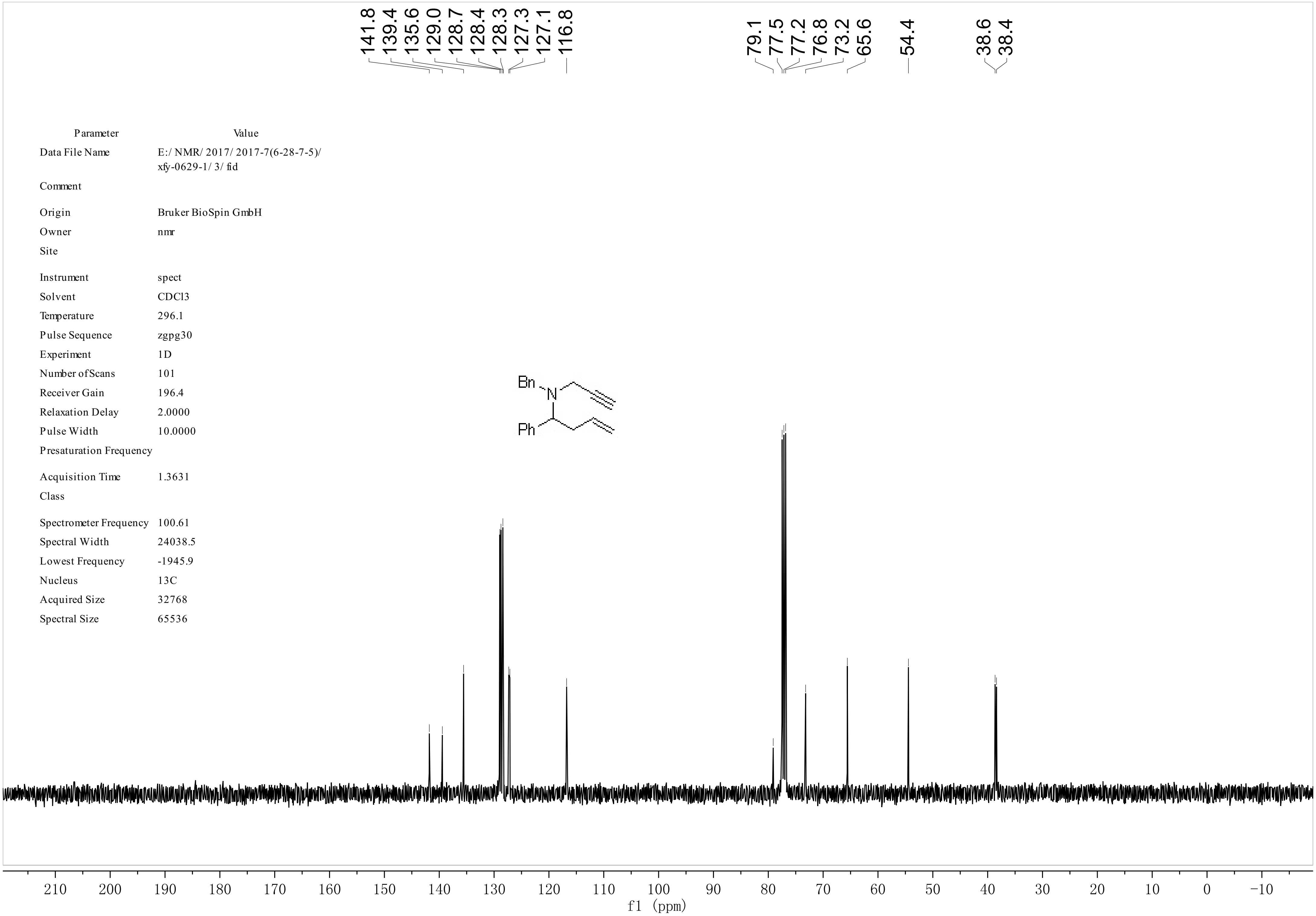

Parameter	Value
Data File Name	E:/ NMR/ 2017/ 2017-7(6-28-7-5)/ xfy-0629-1/ 3/ fid
Comment	
Origin	Bruker BioSpin GmbH
Owner	nmr
Site	
Instrument	spect
Solvent	CDCl3
Temperature	296.1
Pulse Sequence	zgpg30
Experiment	1D
Number of Scans	101
Receiver Gain	196.4
Relaxation Delay	2.0000
Pulse Width	10.0000
Presaturation Frequency	
Acquisition Time	1.3631
Class	
Spectrometer Frequency	100.61
Spectral Width	24038.5
Lowest Frequency	-1945.9
Nucleus	13C
Acquired Size	32768
Spectral Size	65536

Parameter	Value
Title	gvv288f2.1.fid
Comment	
Origin	Bruker BioSpin GmbH
Owner	nmr
Site	
Instrument	spect
Solvent	CDCl3
Temperature	292.8
Pulse Sequence	zg30
Experiment	1D
Number of Scans	8
Receiver Gain	111.4
Relaxation Delay	1.0000
Pulse Width	10.0000
Presaturation Frequency	
Acquisition Time	1.9999
Acquisition Date	2018-10-28T22:41:37
Modification Date	2018-10-30T21:47:09
Spectrometer Frequency	400.13
Spectral Width	8012.8
Lowest Frequency	-1552.2
Nucleus	1H
Acquired Size	16025
Spectral Size	65536

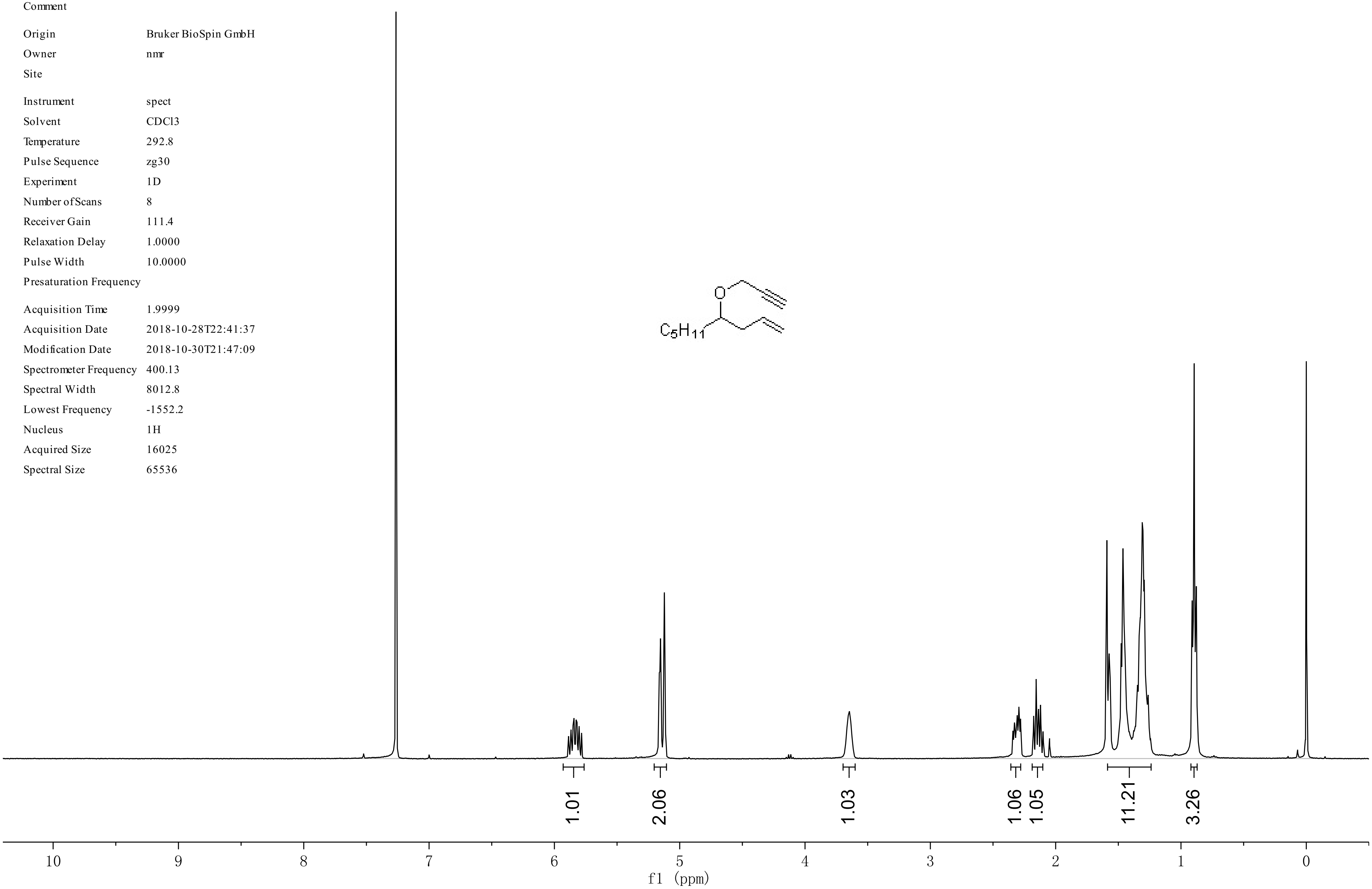

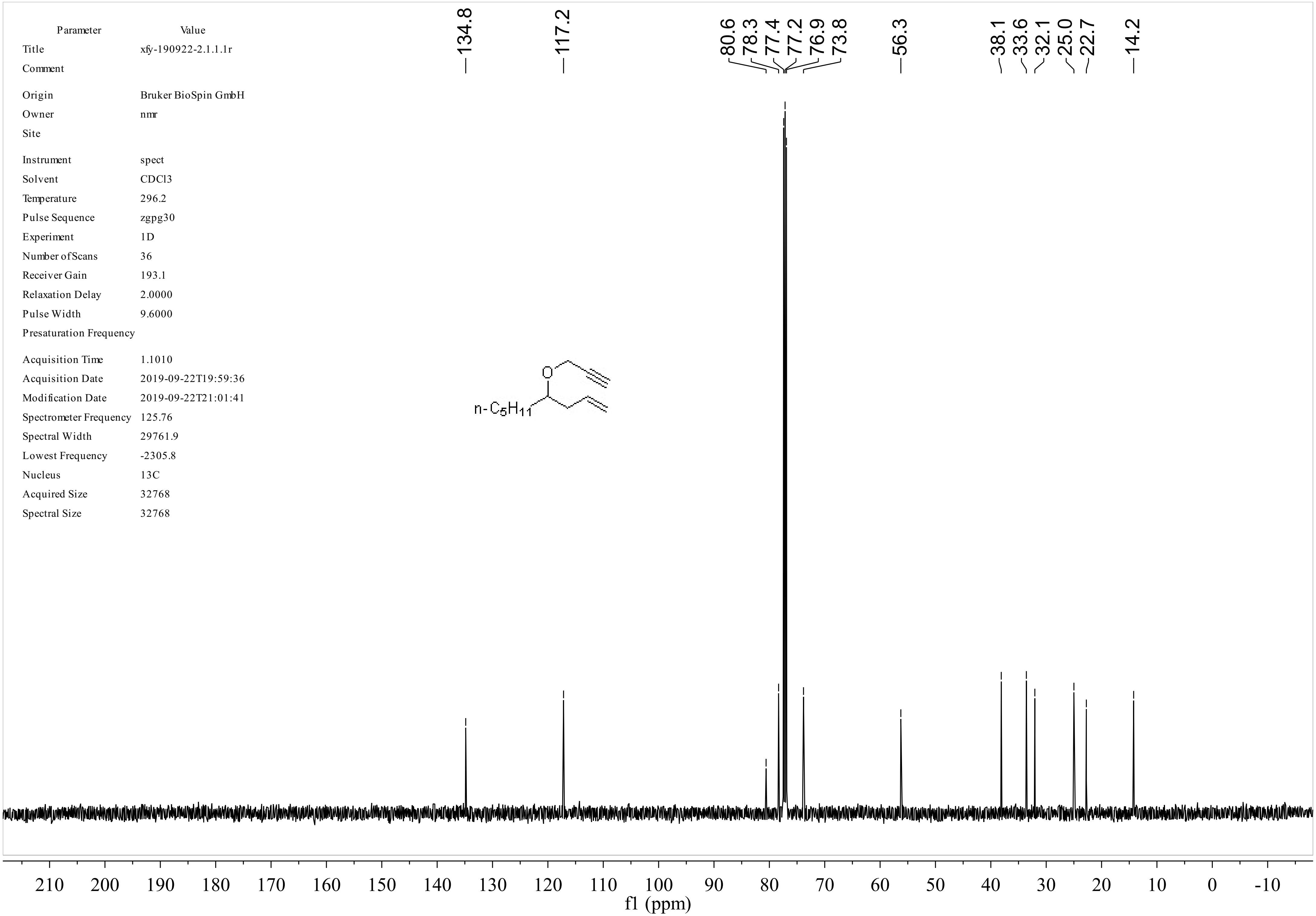

Parameter	Value
Title	xfy-190922-2.1.1.1r
Comment	
Origin	Bruker BioSpin GmbH
Owner	nmr
Site	
Instrument	spect
Solvent	CDCl3
Temperature	296.2
Pulse Sequence	zgpg30
Experiment	1D
Number of Scans	36
Receiver Gain	193.1
Relaxation Delay	2.0000
Pulse Width	9.6000
Presaturation Frequency	
Acquisition Time	1.1010
Acquisition Date	2019-09-22T19:59:36
Modification Date	2019-09-22T21:01:41
Spectrometer Frequency	125.76
Spectral Width	29761.9
Lowest Frequency	-2305.8
Nucleus	13C
Acquired Size	32768
Spectral Size	32768

Parameter	Value
Origin	Bruker BioSpin GmbH
Spectrometer	spect
Solvent	CDCl3
Temperature	293.6
Pulse Sequence	zg30
Experiment	1D
Number of Scans	8
Receiver Gain	14
Relaxation Delay	1.0000
Pulse Width	10.0000
Acquisition Time	1.9999
Spectrometer Frequency	400.13
Spectral Width	8012.8
Lowest Frequency	-1526.6
Nucleus	1H
Acquired Size	16025
Spectral Size	65536

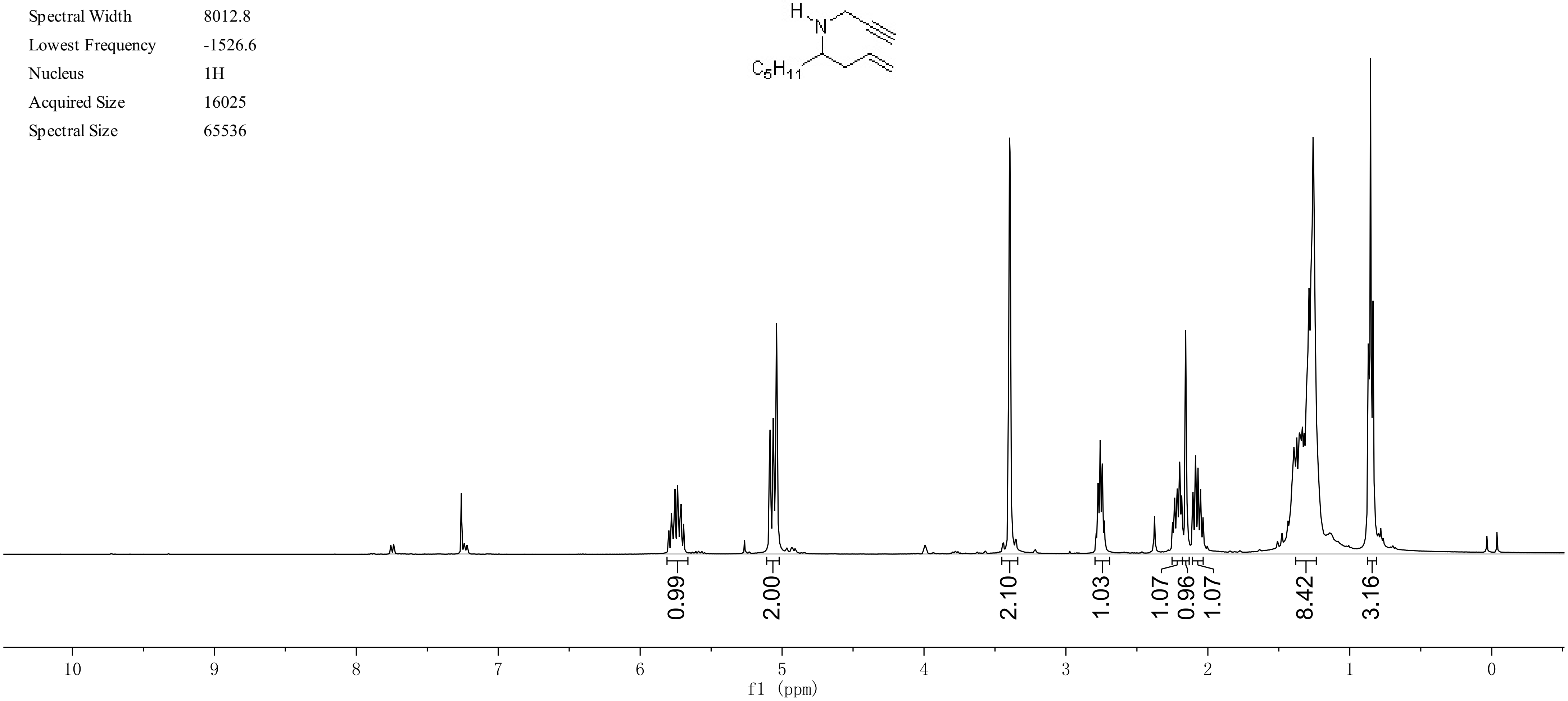

Parameter	Value
Origin	Bruker BioSpin GmbH
Spectrometer	spect
Solvent	CDCl3
Temperature	294.4
Pulse Sequence	zgpg30
Experiment	1D
Number of Scans	483
Receiver Gain	196
Relaxation Delay	2.0000
Pulse Width	10.0000
Acquisition Time	1.3631
Spectrometer Frequency	100.62
Spectral Width	24038.5
Lowest Frequency	-1941.0
Nucleus	13C
Acquired Size	32768
Spectral Size	65536

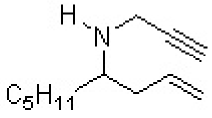

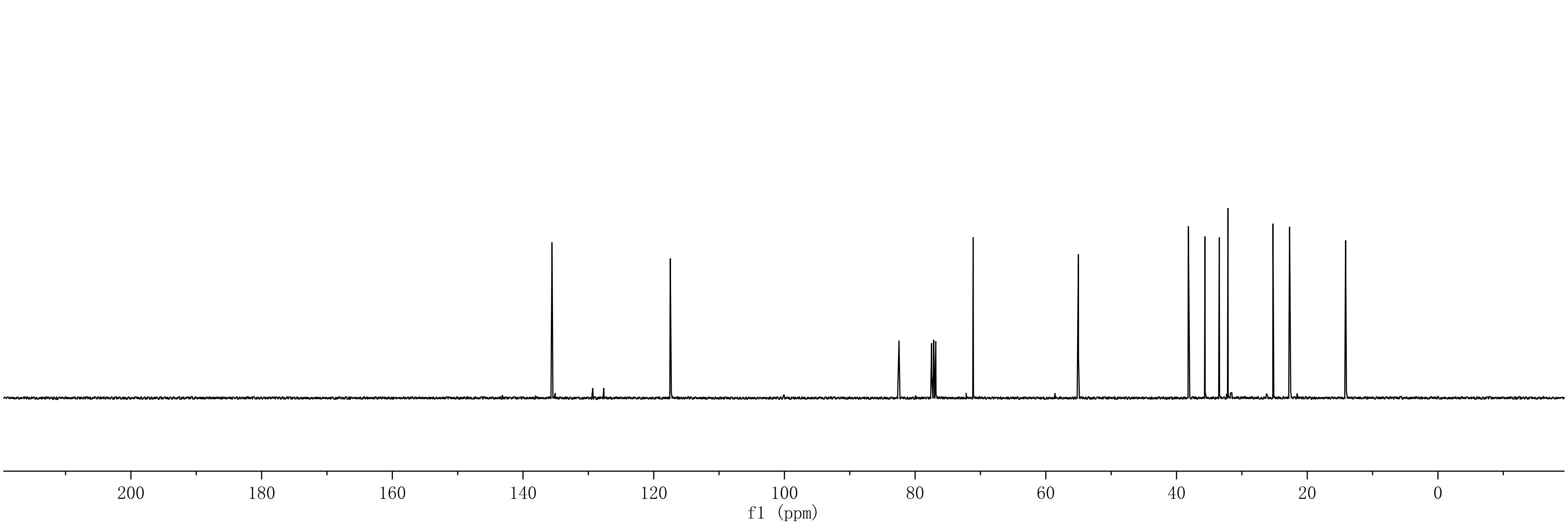

Parameter	Value
Origin	Bruker BioSpin GmbH
Spectrometer	spect
Solvent	CDCl3
Temperature	296.2
Pulse Sequence	zg30
Experiment	1D
Number of Scans	16
Receiver Gain	9
Relaxation Delay	1.0000
Pulse Width	10.7100
Acquisition Time	3.2768
Spectrometer Frequency	500.13
Spectral Width	10000.0
Lowest Frequency	-1876.5
Nucleus	1H
Acquired Size	32768
Spectral Size	65536

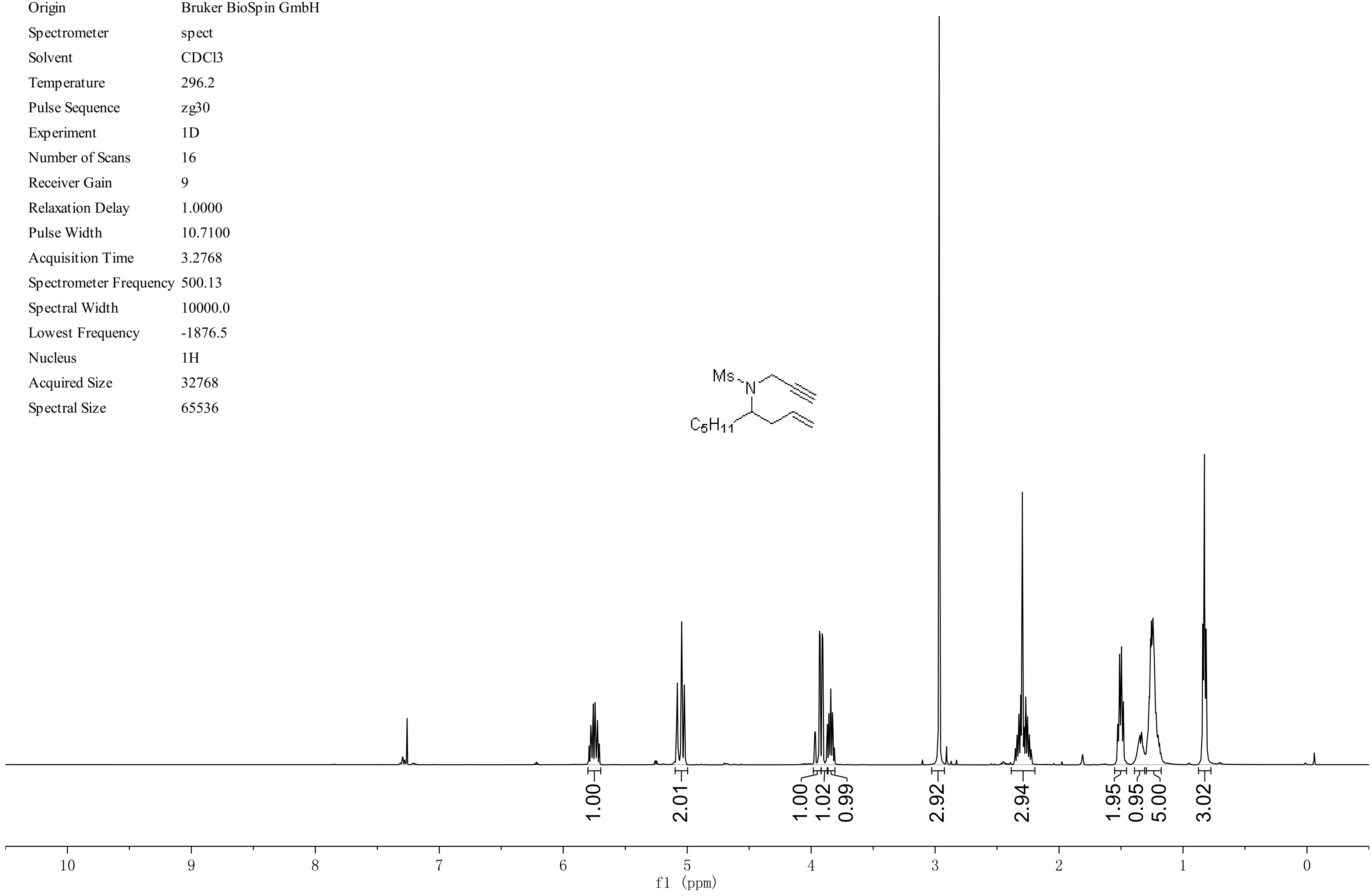

Parameter	Value
Origin	Bruker BioSpin GmbH
Spectrometer	spect
Solvent	CDCl3
Temperature	296.2
Pulse Sequence	zgpg30
Experiment	1D
Number of Scans	24
Receiver Gain	193
Relaxation Delay	2.0000
Pulse Width	9.6000
Acquisition Time	1.1010
Spectrometer Frequency	125.77
Spectral Width	29761.9
Lowest Frequency	-2313.8
Nucleus	13C
Acquired Size	32768
Spectral Size	65536

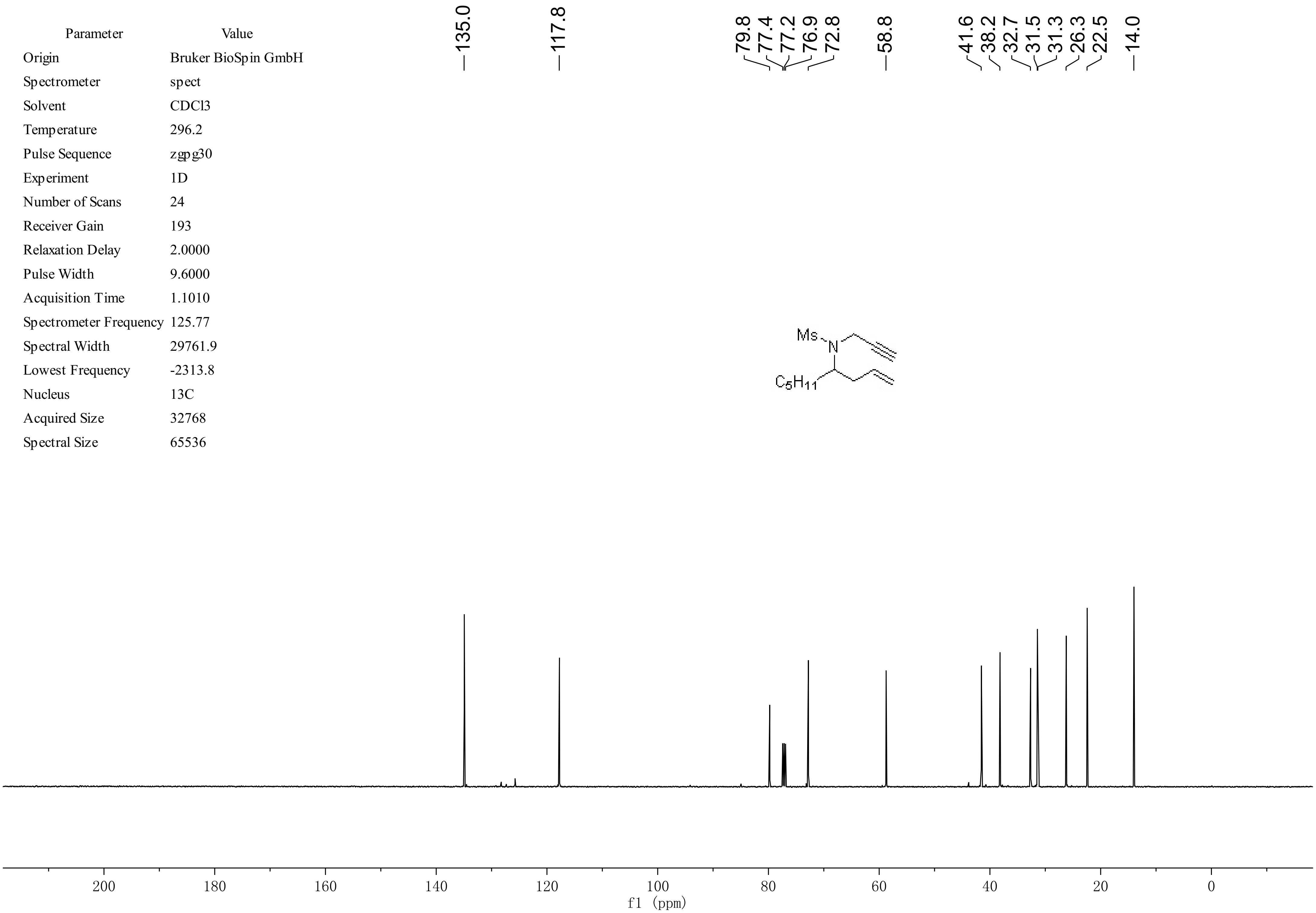

Parameter	Value
Data File Name	E:/ NMR/ 2017/ 2017-5(3-8)/ xfy-0508-2-1/ 1/ fid
Comment	
Origin	Bruker BioSpin GmbH
Owner	nmr
Site	
Instrument	spect
Solvent	CDCl3
Temperature	295.5
Pulse Sequence	zg30
Experiment	1D
Number of Scans	8
Receiver Gain	111.4
Relaxation Delay	1.0000
Pulse Width	9.6000
Presaturation Frequency	
Acquisition Time	1.9999
Class	
Spectrometer Frequency	400.13
Spectral Width	8012.8
Lowest Frequency	-1543.8
Nucleus	1H
Acquired Size	16025
Spectral Size	32768

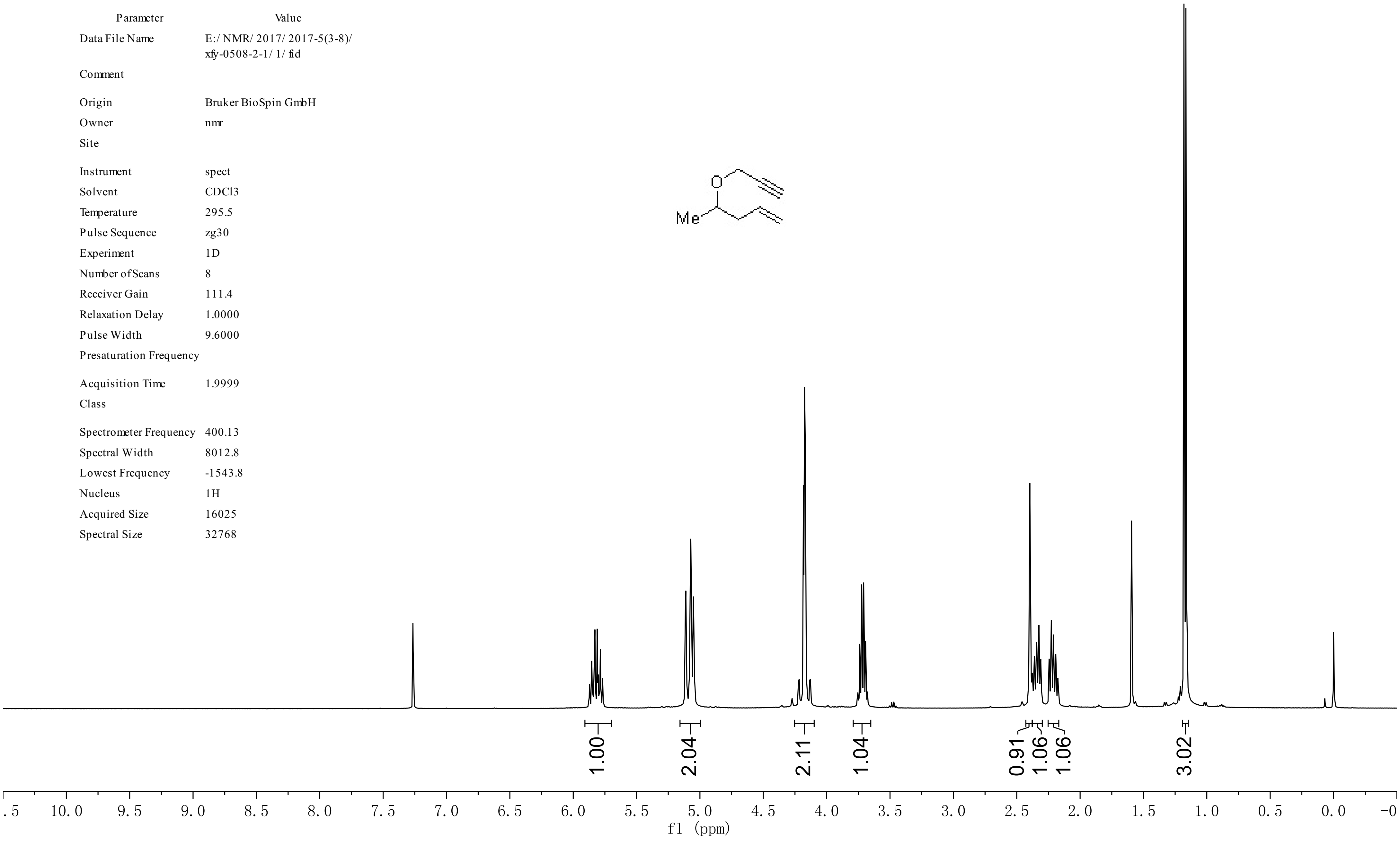

	Parameter	Value
1	Data File Name	E:/ NMR/ 2017/ 2017-5(3-8)/ xfy-0508-2-1/ 2/ fid
2	Title	xfy-0508-2-1
3	Comment	
4	Origin	Bruker BioSpin GmbH
5	Owner	nmr
6	Site	
7	Spectrometer	spect
8	Author	
9	Solvent	CDCl3
10	Temperature	295.8
11	Pulse Sequence	zgpg30
12	Experiment	1D
13	Number of Scans	500
14	Receiver Gain	196
15	Relaxation Delay	2.0000
16	Pulse Width	10.0000
17	Acquisition Time	1.3631
18	Acquisition Date	2017-05-09T07:02:40
19	Modification Date	2017-05-09T09:18:20
20	Spectrometer Frequency	100.62
21	Spectral Width	24038.5
22	Lowest Frequency	-1943.6
23	Nucleus	13C
24	Acquired Size	32768
25	Spectral Size	65536

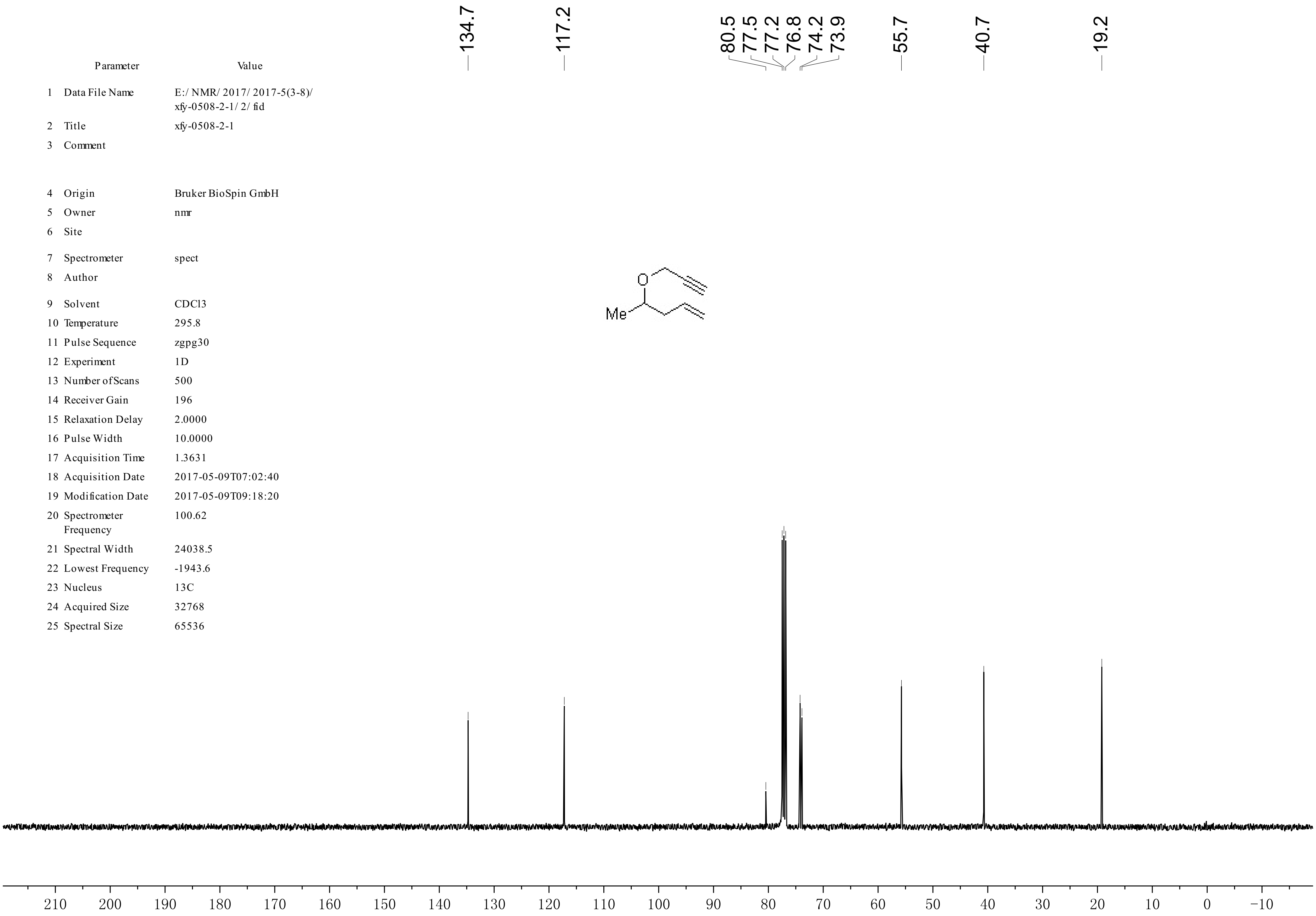

Parameter	Value
Data File Name	E:/ NMR/ 2017/ 2017-5(26-31)/ xfy-0529-2/ 1/ fid
Comment	
Origin	Bruker BioSpin GmbH
Owner	nmr
Site	
Instrument	spect
Solvent	CDCl3
Temperature	295.2
Pulse Sequence	zg30
Experiment	1D
Number of Scans	8
Receiver Gain	70.3
Relaxation Delay	1.0000
Pulse Width	9.6000
Presaturation Frequency	
Acquisition Time	1.9999
Class	
Spectrometer Frequency	400.13
Spectral Width	8012.8
Lowest Frequency	-1539.4
Nucleus	1H
Acquired Size	16025
Spectral Size	32768

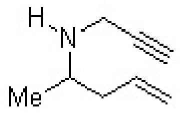

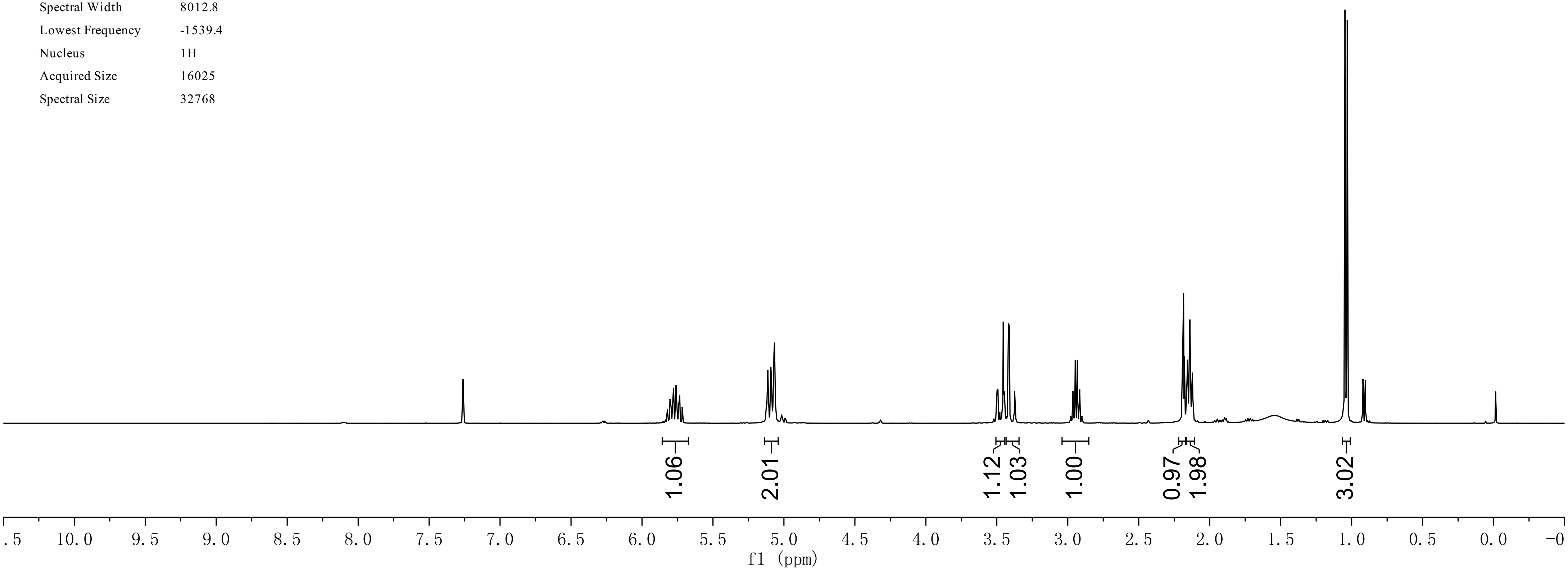

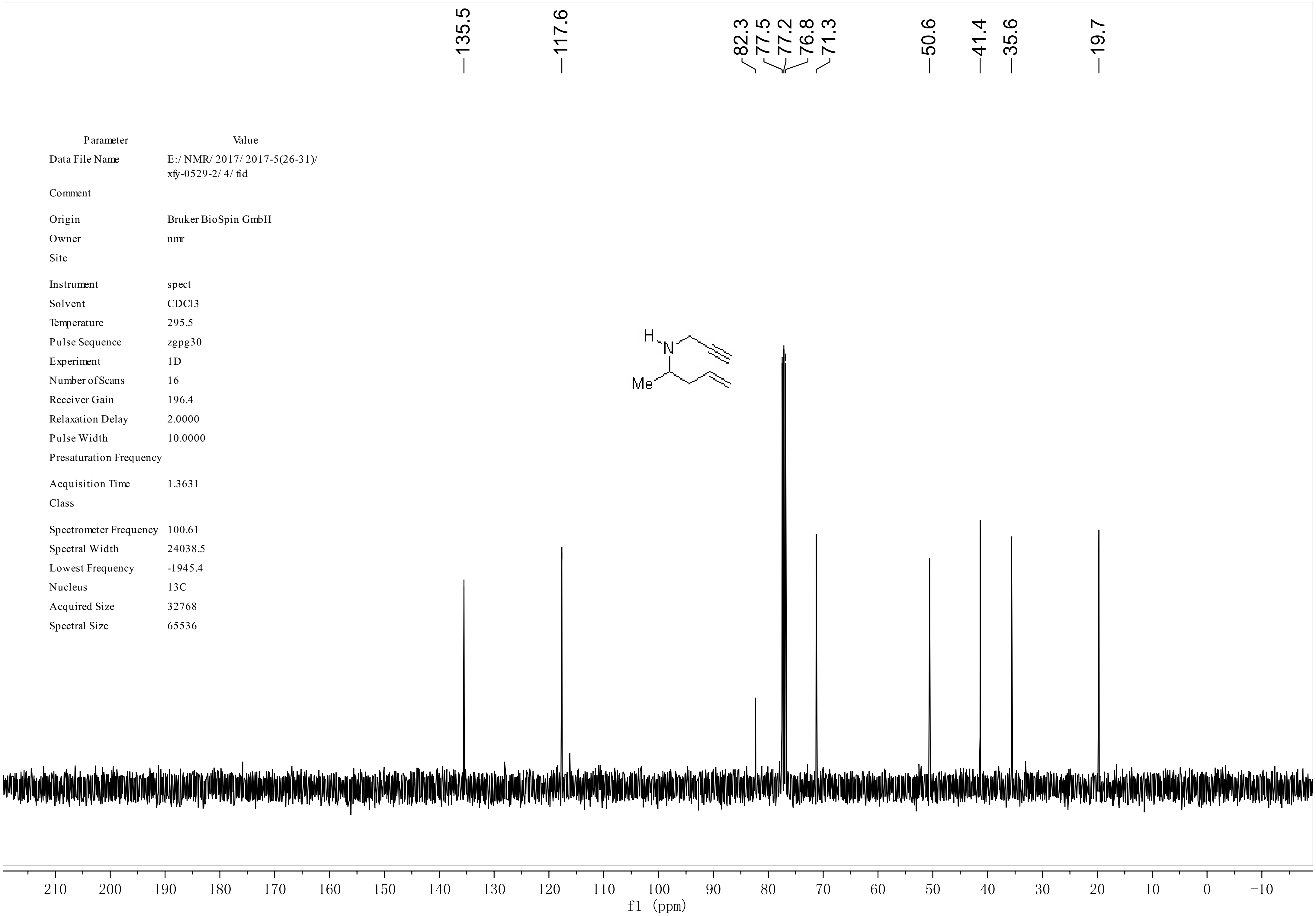

Parameter	Value
Data File Name	E:/ NMR/ 2017/ 2017-5(26-31)/ xfy-0529-2/ 4/ fid
Comment	
Origin	Bruker BioSpin GmbH
Owner	nmr
Site	
Instrument	spect
Solvent	CDCl3
Temperature	295.5
Pulse Sequence	zgpg30
Experiment	1D
Number of Scans	16
Receiver Gain	196.4
Relaxation Delay	2.0000
Pulse Width	10.0000
Presaturation Frequency	
Acquisition Time	1.3631
Class	
Spectrometer Frequency	100.61
Spectral Width	24038.5
Lowest Frequency	-1945.4
Nucleus	13C
Acquired Size	32768
Spectral Size	65536

Parameter	Value
Data File Name	E:/ NMR/ 2017/ 2017-6-（8-14）/ xfy-0612-1/ 1/ fid
Comment	
Origin	Bruker BioSpin GmbH
Owner	nmr
Site	
Instrument	spect
Solvent	CDCl3
Temperature	296.1
Pulse Sequence	zg30
Experiment	1D
Number of Scans	16
Receiver Gain	31.1
Relaxation Delay	1.0000
Pulse Width	11.2900
Presaturation Frequency	
Acquisition Time	3.2768
Class	
Spectrometer Frequency	500.13
Spectral Width	10000.0
Lowest Frequency	-1915.0
Nucleus	1H
Acquired Size	32768
Spectral Size	65536

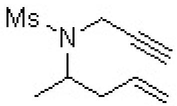

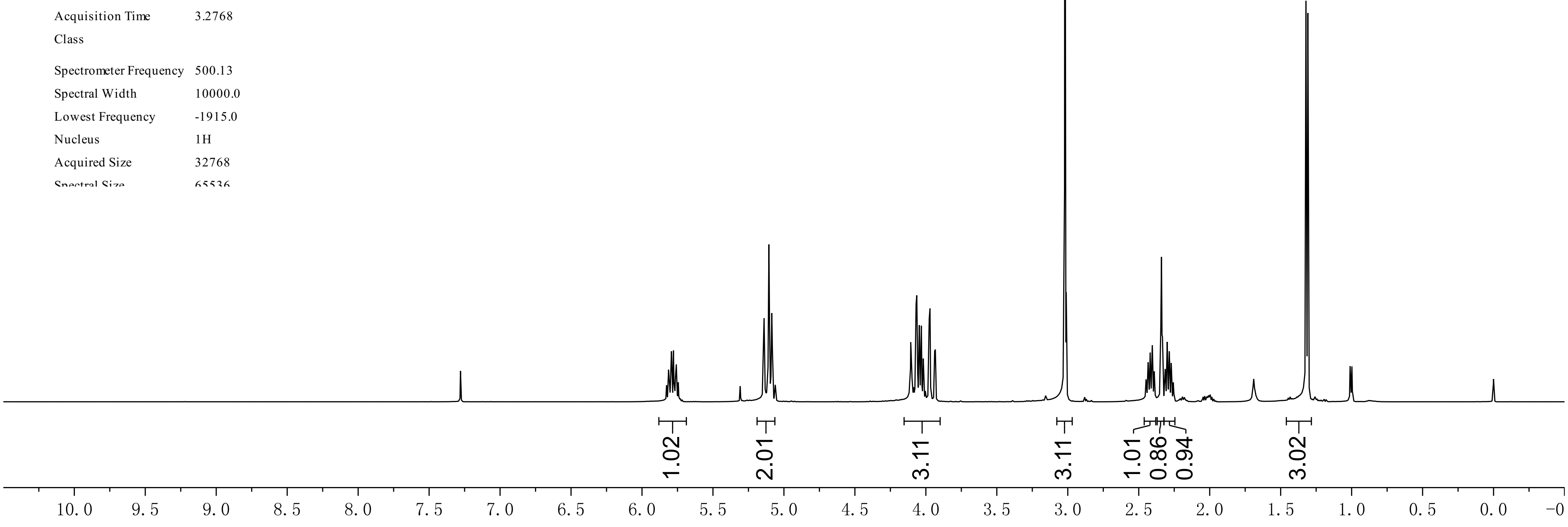

Parameter	Value
Title	xfy-190922-3.1.fid
Comment	
Origin	Bruker BioSpin GmbH
Owner	nmr
Site	
Instrument	spect
Solvent	CDCl3
Temperature	296.5
Pulse Sequence	zgpg30
Experiment	1D
Number of Scans	47
Receiver Gain	196.4
Relaxation Delay	2.0000
Pulse Width	10.0000
Presaturation Frequency	
Acquisition Time	1.3631
Acquisition Date	2019-09-22T20:00:24
Modification Date	2019-09-22T21:01:40
Spectrometer Frequency	100.62
Spectral Width	24038.5
Lowest Frequency	-1945.4
Nucleus	13C
Acquired Size	32768
Spectral Size	65536

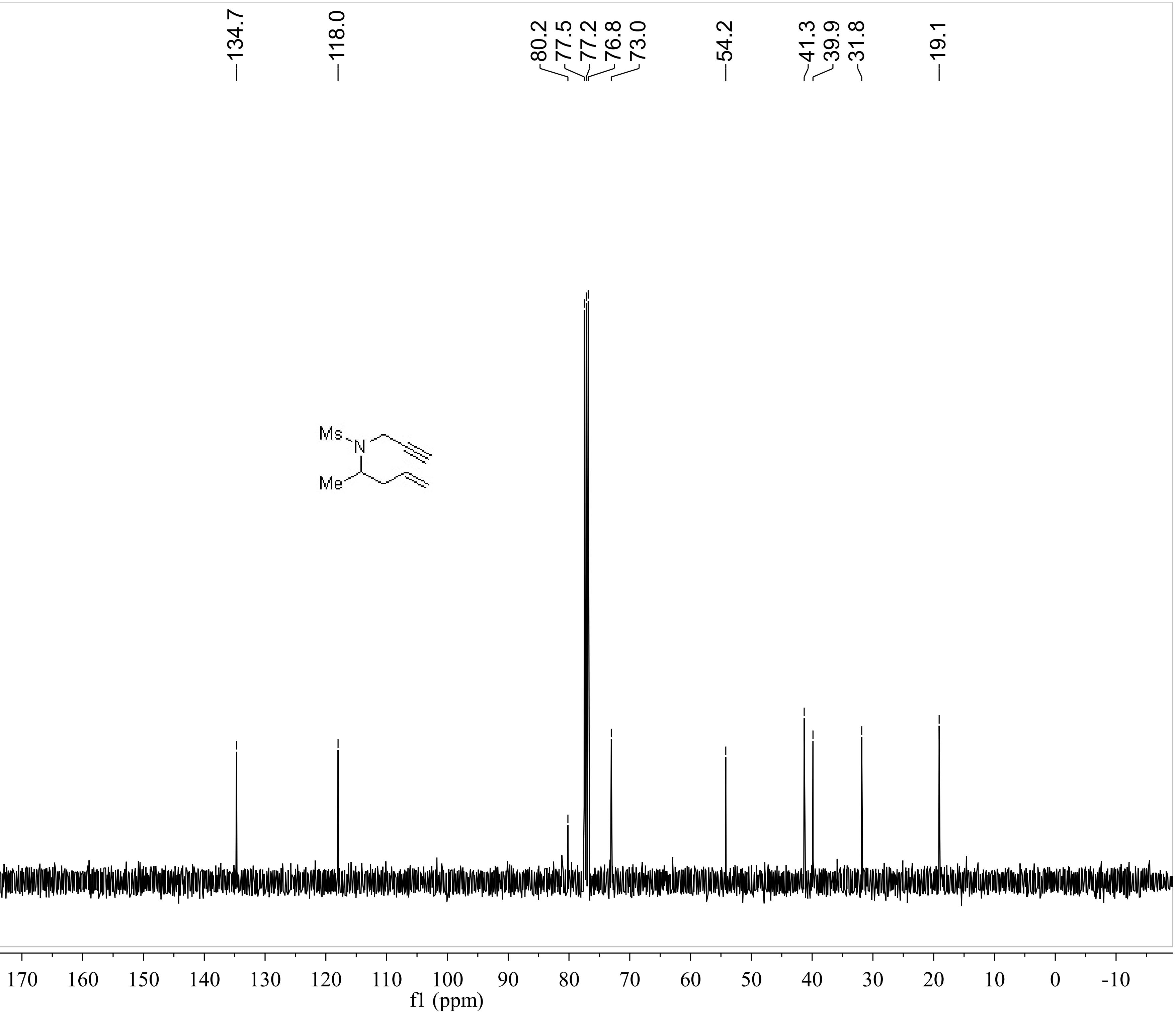

Parameter	Value
Data File Name	E:/ NMR/ 2017/ 2017-5(26-31)/ xfy-0529-3/ 1/ fid
Comment	
Origin	Bruker BioSpin GmbH
Owner	nmr
Site	
Instrument	spect
Solvent	CDCl3
Temperature	295.3
Pulse Sequence	zg30
Experiment	1D
Number of Scans	8
Receiver Gain	22.4
Relaxation Delay	1.0000
Pulse Width	9.6000
Presaturation Frequency	
Acquisition Time	1.9999
Class	
Spectrometer Frequency	400.13
Spectral Width	8012.8
Lowest Frequency	-1535.4
Nucleus	1H
Acquired Size	16025
Spectral Size	32768

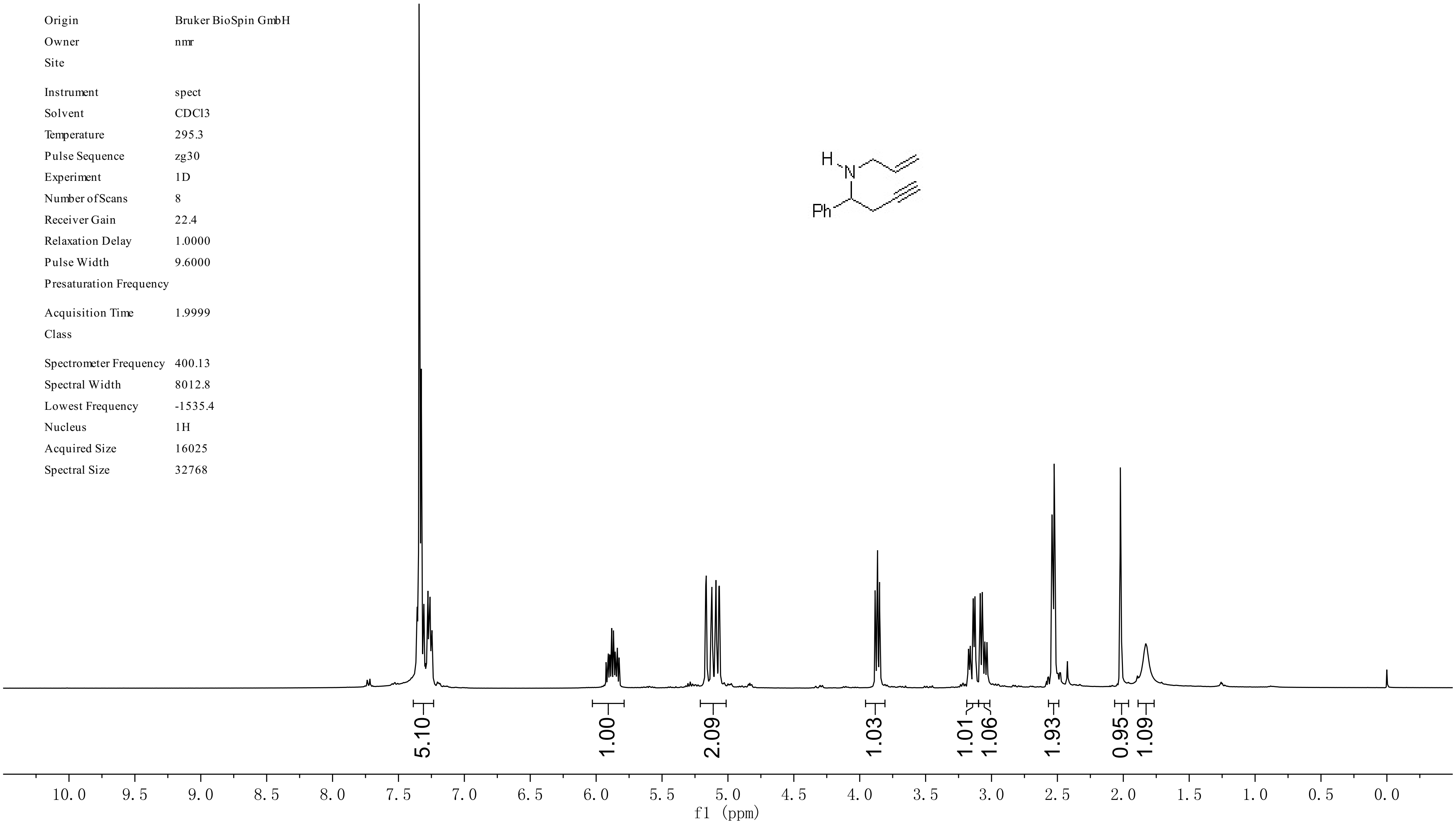

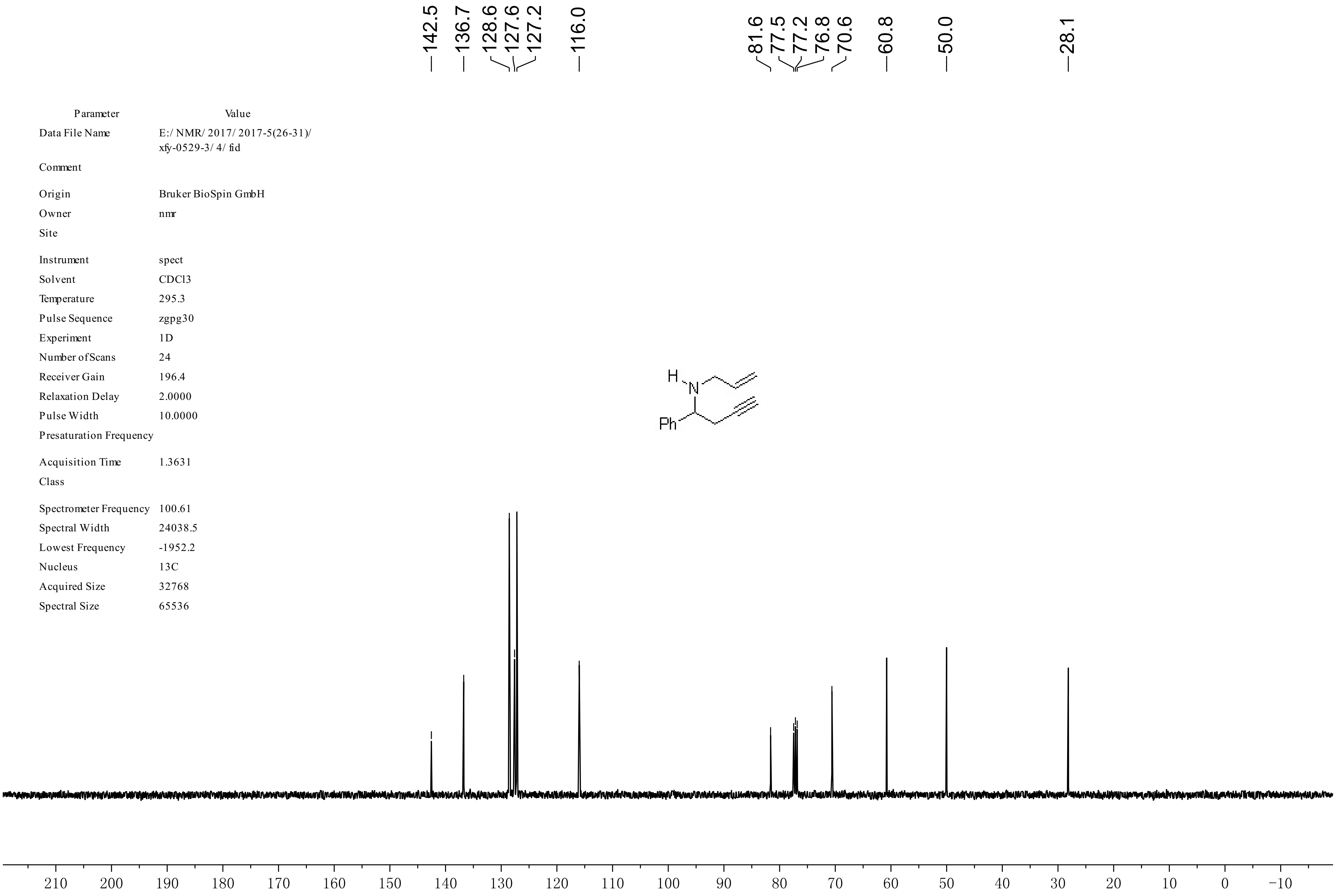

Parameter	Value
Data File Name	E:/ NMR/ 2017/ 2017-5(26-31)/ xfy-0529-3/ 4/ fid
Comment	
Origin	Bruker BioSpin GmbH
Owner	nmr
Site	
Instrument	spect
Solvent	CDCl3
Temperature	295.3
Pulse Sequence	zgpg30
Experiment	1D
Number of Scans	24
Receiver Gain	196.4
Relaxation Delay	2.0000
Pulse Width	10.0000
Presaturation Frequency	
Acquisition Time	1.3631
Class	
Spectrometer Frequency	100.61
Spectral Width	24038.5
Lowest Frequency	-1952.2
Nucleus	13C
Acquired Size	32768
Spectral Size	65536

Parameter	Value
Data File Name	E:/ NMR/ 2017/ 2017-6-(15-21)/ xfy-0614-2/ 1/ fid
Comment	
Origin	Bruker BioSpin GmbH
Owner	nmr
Site	
Instrument	spect
Solvent	CDCl3
Temperature	296.2
Pulse Sequence	zg30
Experiment	1D
Number of Scans	9
Receiver Gain	31.1
Relaxation Delay	1.0000
Pulse Width	11.2900
Presaturation Frequency	
Acquisition Time	3.2768
Class	
Spectrometer Frequency	500.13
Spectral Width	10000.0
Lowest Frequency	-1922.0
Nucleus	1H
Acquired Size	32768
Spectral Size	65536

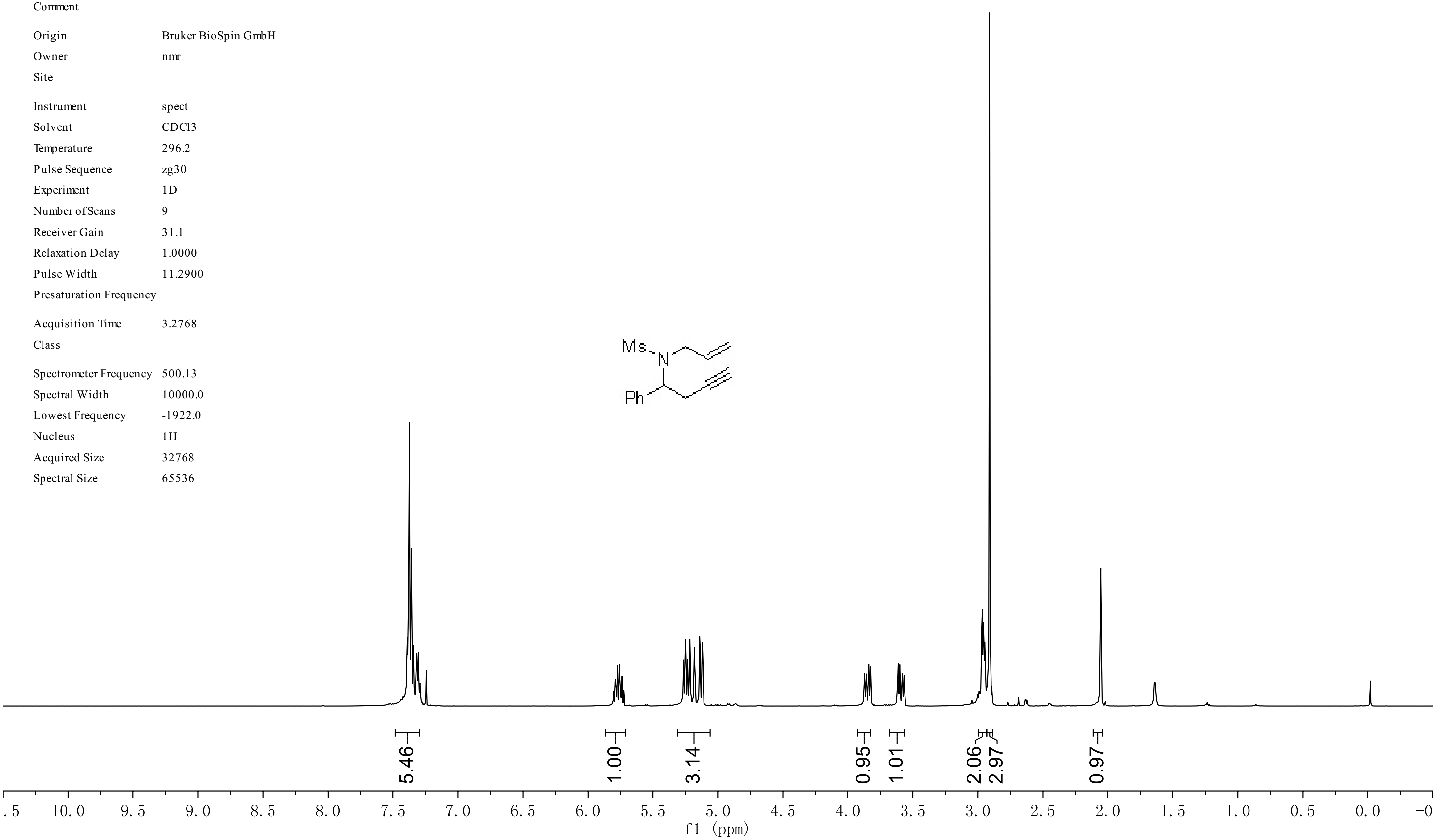

Parameter	Value
Data File Name	E:/ NMR/ 2017/ 2017-6-(15-21)/ xfy-0614-2/ 2/ fid
Comment	
Origin	Bruker BioSpin GmbH
Owner	nmr
Site	
Instrument	spect
Solvent	CDCl3
Temperature	296.2
Pulse Sequence	zgpg30
Experiment	1D
Number of Scans	21
Receiver Gain	193.1
Relaxation Delay	2.0000
Pulse Width	9.6000
Presaturation Frequency	
Acquisition Time	1.1010
Class	
Spectrometer Frequency	125.76
Spectral Width	29761.9
Lowest Frequency	-2296.6
Nucleus	13C
Acquired Size	32768
Spectral Size	65536

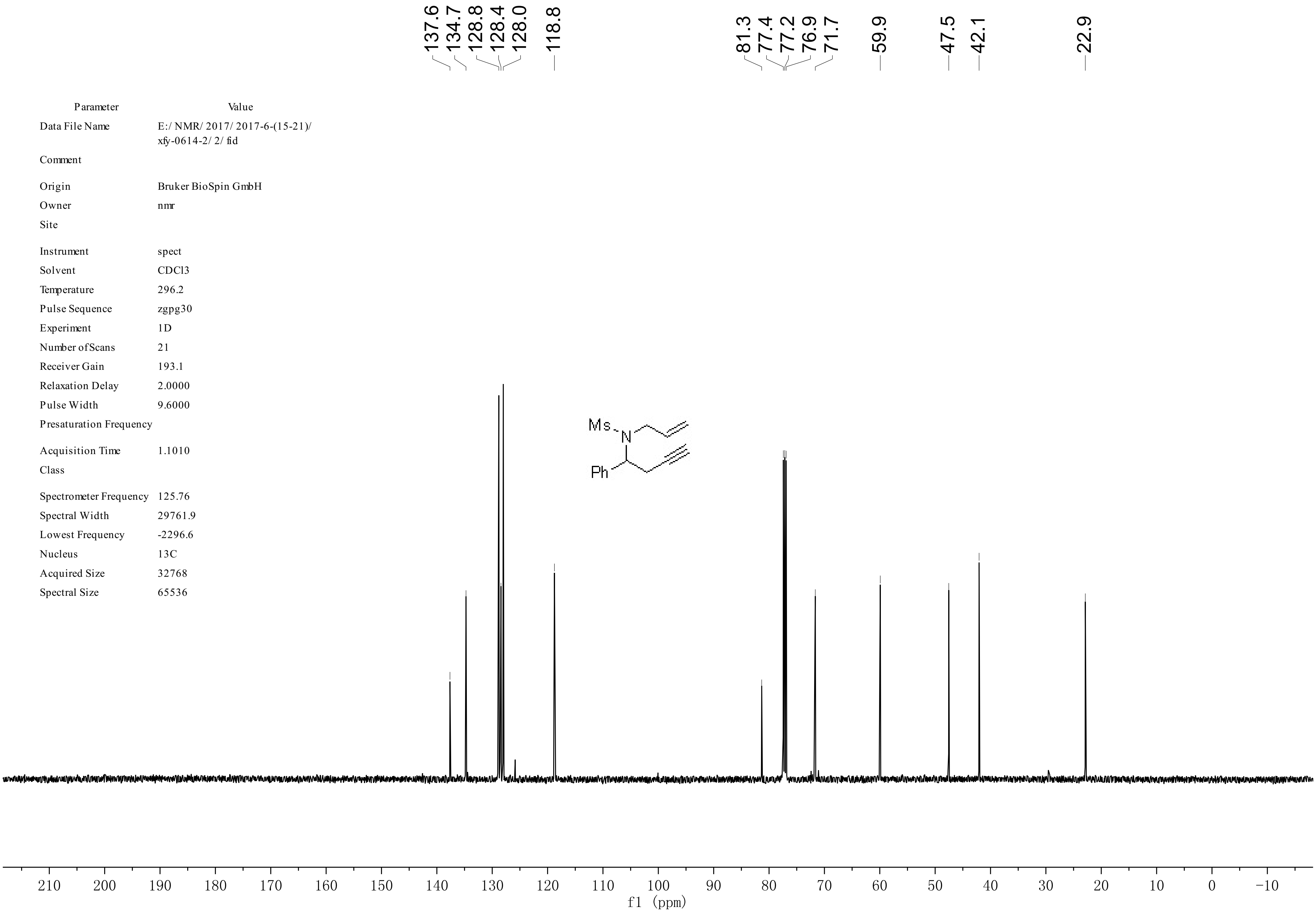

Parameter	Value
Title	gvv295f1.fid
Comment	
Origin	Bruker BioSpin GmbH
Owner	nmr
Site	
Instrument	spect
Solvent	CDCl3
Temperature	294.4
Pulse Sequence	zg30
Experiment	1D
Number of Scans	8
Receiver Gain	14.3
Relaxation Delay	1.0000
Pulse Width	10.0000
Presaturation Frequency	
Acquisition Time	1.9999
Acquisition Date	2018-11-03T23:52:18
Modification Date	2018-11-04T17:46:26
Spectrometer Frequency	400.13
Spectral Width	8012.8
Lowest Frequency	-1537.8
Nucleus	1H
Acquired Size	16025
Spectral Size	65536

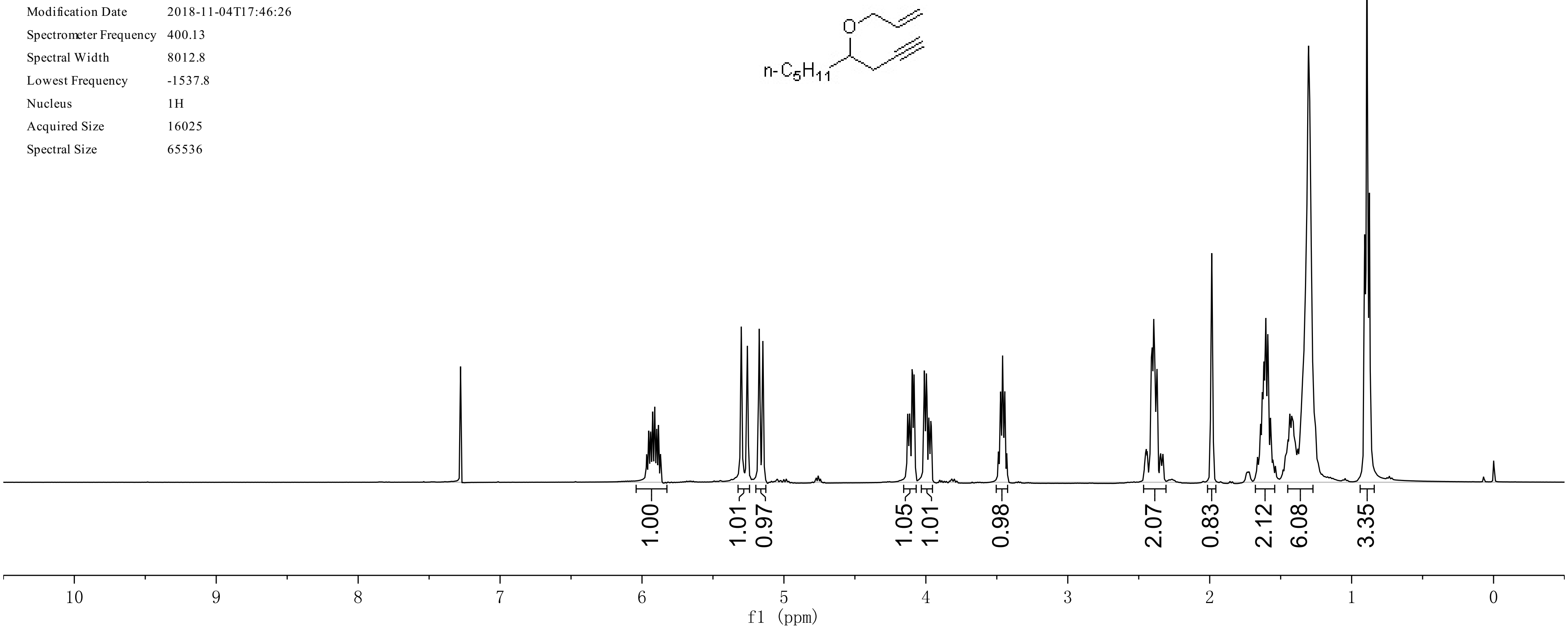

Parameter	Value
Title	gvv295f2.fid
Comment	
Origin	Bruker BioSpin GmbH
Owner	nmr
Site	
Instrument	spect
Solvent	CDCl3
Temperature	295.0
Pulse Sequence	zgpg30
Experiment	1D
Number of Scans	200
Receiver Gain	196.4
Relaxation Delay	2.0000
Pulse Width	10.0000
Presaturation Frequency	
Acquisition Time	1.3631
Acquisition Date	2018-11-04T00:04:55
Modification Date	2018-11-04T17:46:27
Spectrometer Frequency	100.62
Spectral Width	24038.5
Lowest Frequency	-1947.7
Nucleus	13C
Acquired Size	32768
Spectral Size	65536

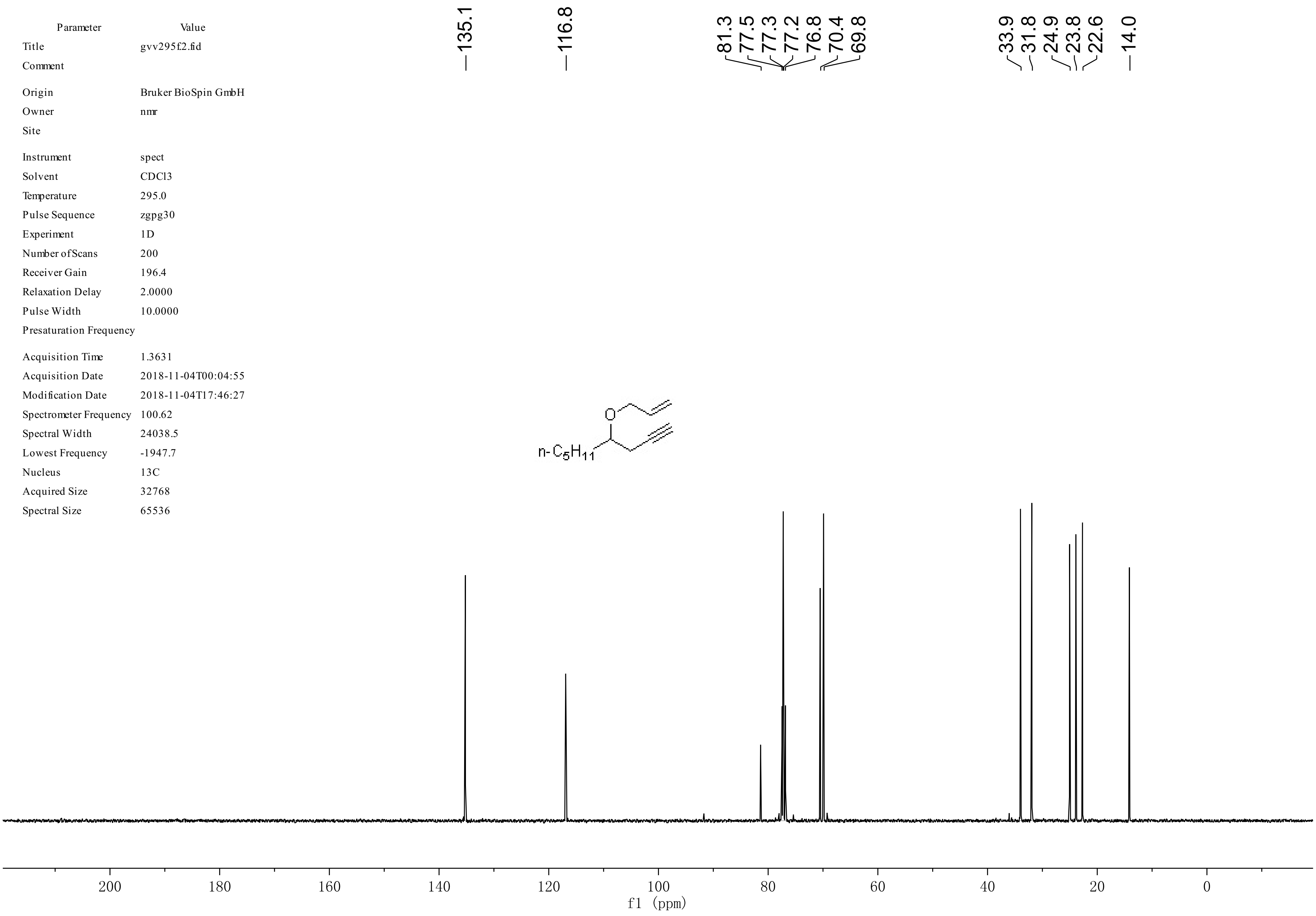

Parameter	Value
Title	xfy-190226-8.11.1.1r
Comment	
Origin	Bruker BioSpin GmbH
Owner	nmr
Site	
Instrument	spect
Solvent	CDCl3
Temperature	291.6
Pulse Sequence	zg30
Experiment	1D
Number of Scans	8
Receiver Gain	31.7
Relaxation Delay	1.0000
Pulse Width	8.7300
Presaturation Frequency	
Acquisition Time	1.9999
Acquisition Date	2019-02-26T21:34:39
Modification Date	2019-02-26T21:52:02
Spectrometer Frequency	400.13
Spectral Width	8012.8
Lowest Frequency	-1545.4
Nucleus	1H
Acquired Size	16025
Spectral Size	65536

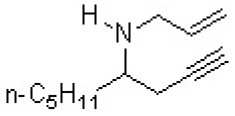

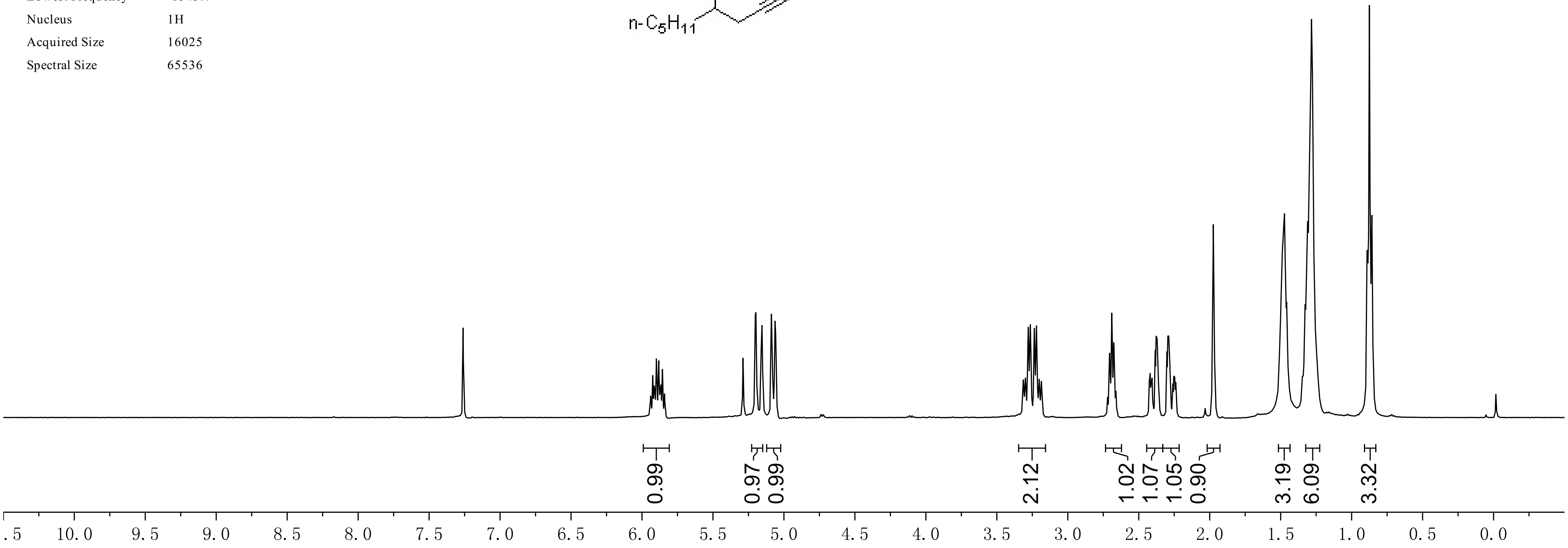

Parameter	Value
Origin	Bruker BioSpin GmbH
Spectrometer	spect
Solvent	CDCl3
Temperature	291.8
Pulse Sequence	zgpg30
Experiment	1D
Number of Scans	14
Receiver Gain	196
Relaxation Delay	2.0000
Pulse Width	10.0000
Acquisition Time	1.3631
Spectrometer Frequency	100.61
Spectral Width	24038.5
Lowest Frequency	-1937.6
Nucleus	13C
Acquired Size	32768
Spectral Size	32768

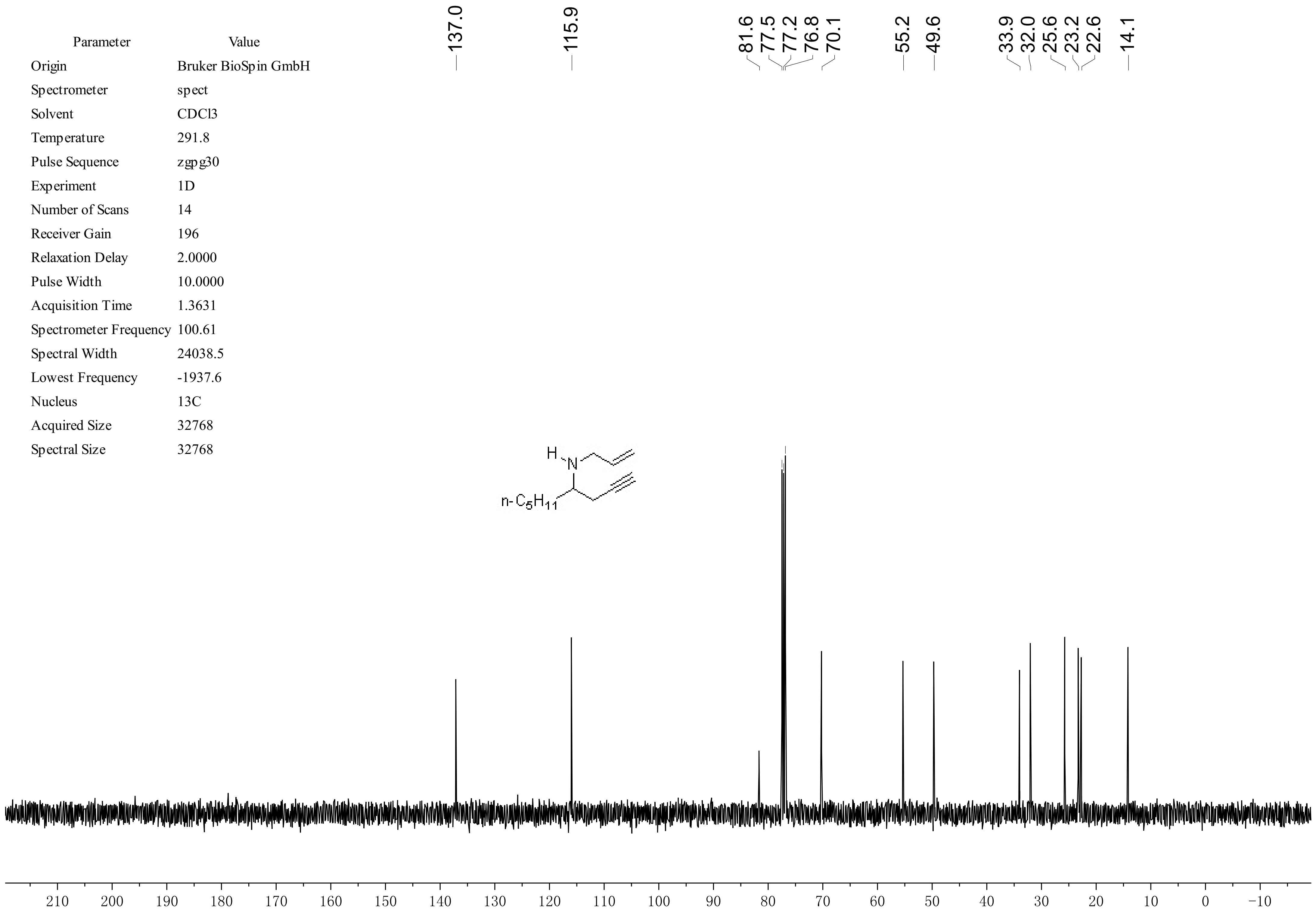

Parameter	Value
Title	xfy-190224-4.1.1.1r
Comment	
Origin	Bruker BioSpin GmbH
Owner	nmr
Site	
Instrument	spect
Solvent	CDCl3
Temperature	296.1
Pulse Sequence	zg30
Experiment	1D
Number of Scans	7
Receiver Gain	31.1
Relaxation Delay	1.0000
Pulse Width	10.7100
Presaturation Frequency	
Acquisition Time	3.2768
Class	
Spectrometer Frequency	500.13
Spectral Width	10000.0
Lowest Frequency	-1911.5
Nucleus	1H
Acquired Size	32768
Spectral Size	65536

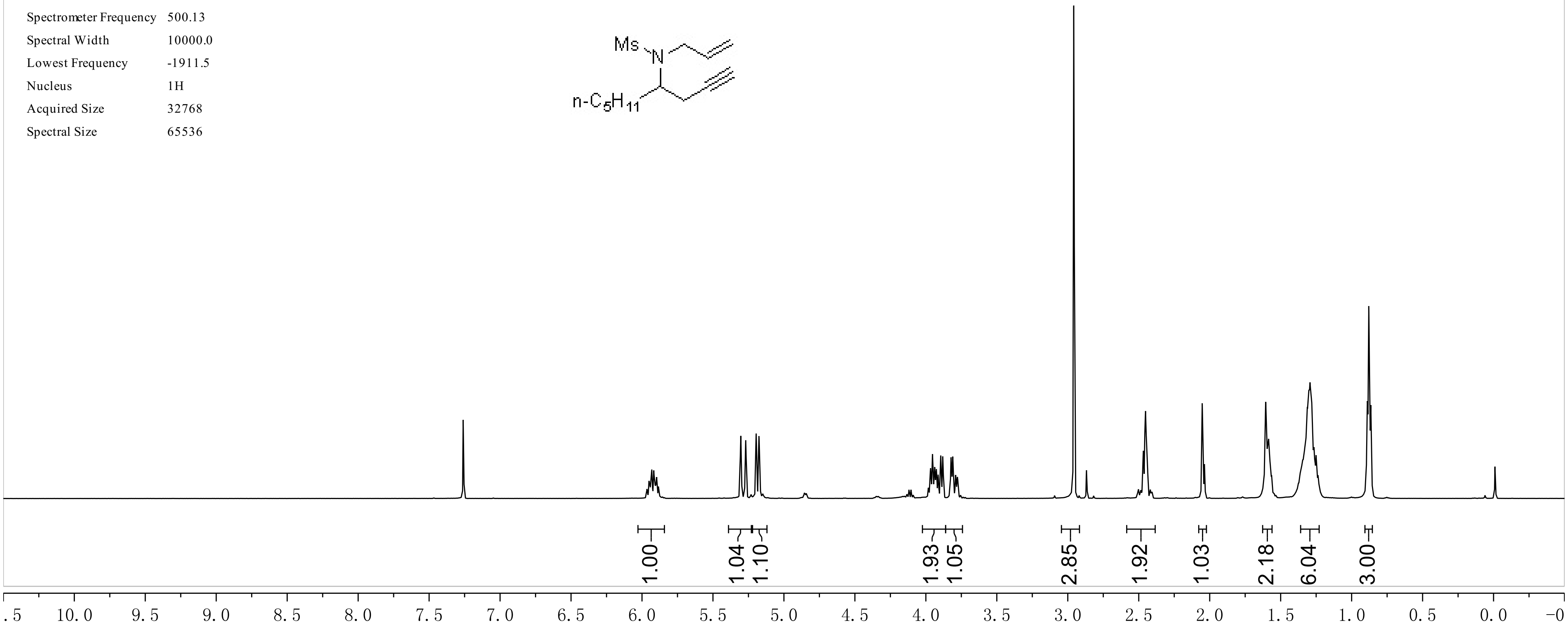

Parameter	Value
Title	xfy-190224-4.2.1.1r
Comment	
Origin	Bruker BioSpin GmbH
Owner	nmr
Site	
Instrument	spect
Solvent	CDCl3
Temperature	296.2
Pulse Sequence	zgpg30
Experiment	1D
Number of Scans	18
Receiver Gain	193.1
Relaxation Delay	2.0000
Pulse Width	9.6000
Presaturation Frequency	
Acquisition Time	1.1010
Class	
Spectrometer Frequency	125.76
Spectral Width	29761.9
Lowest Frequency	-2305.8
Nucleus	13C
Acquired Size	32768
Spectral Size	32768

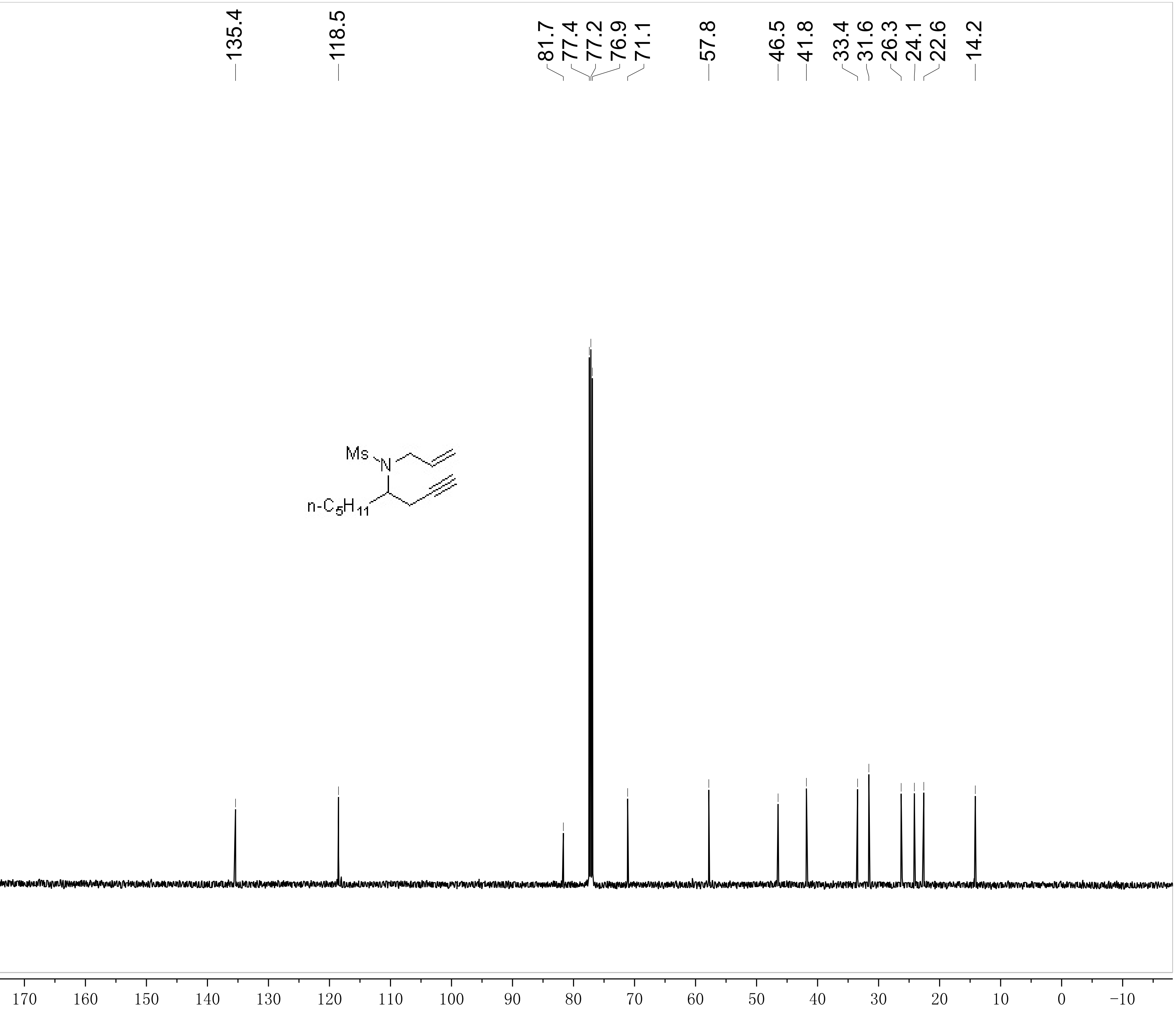

Parameter	Value
Data File Name	E:/ NMR/ 2017/ August/ 2017-8(10-17)/ xfy-0814-5/ 11/ fid
Title	
Comment	
Origin	Bruker BioSpin GmbH
Owner	nmr
Site	
Instrument	spect
Author	
Solvent	CDCl3
Temperature	296.1
Pulse Sequence	zg30
Experiment	1D
Number of Scans	9
Receiver Gain	31.1
Relaxation Delay	1.0000
Pulse Width	11.2900
Presaturation Frequency	
Acquisition Time	3.2768
Class	
Spectrometer Frequency	500.13
Spectral Width	10000.0
Lowest Frequency	-1918.5
Nucleus	1H
Acquired Size	32768
Spectral Size	65536

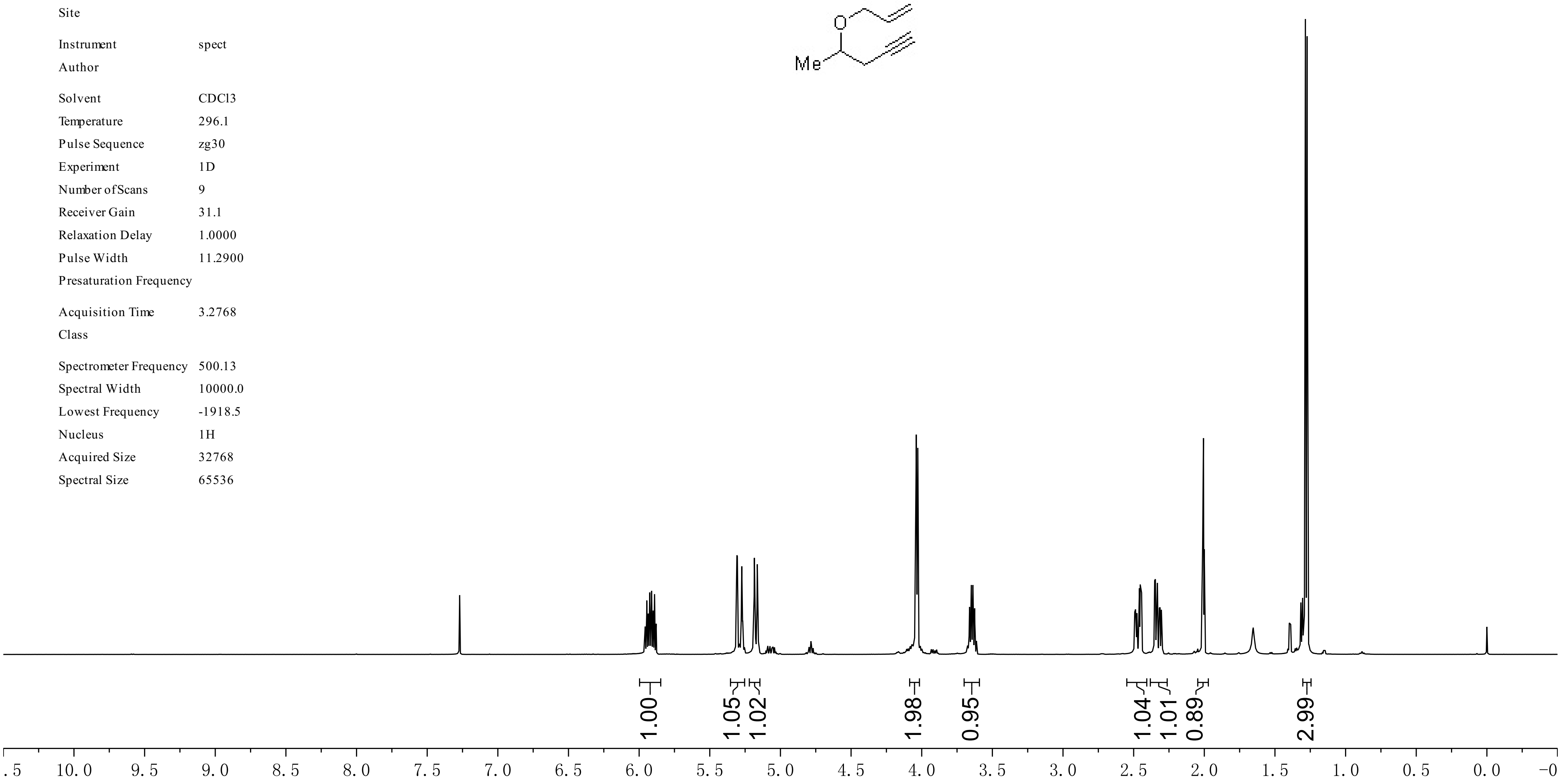

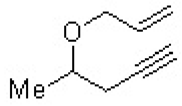

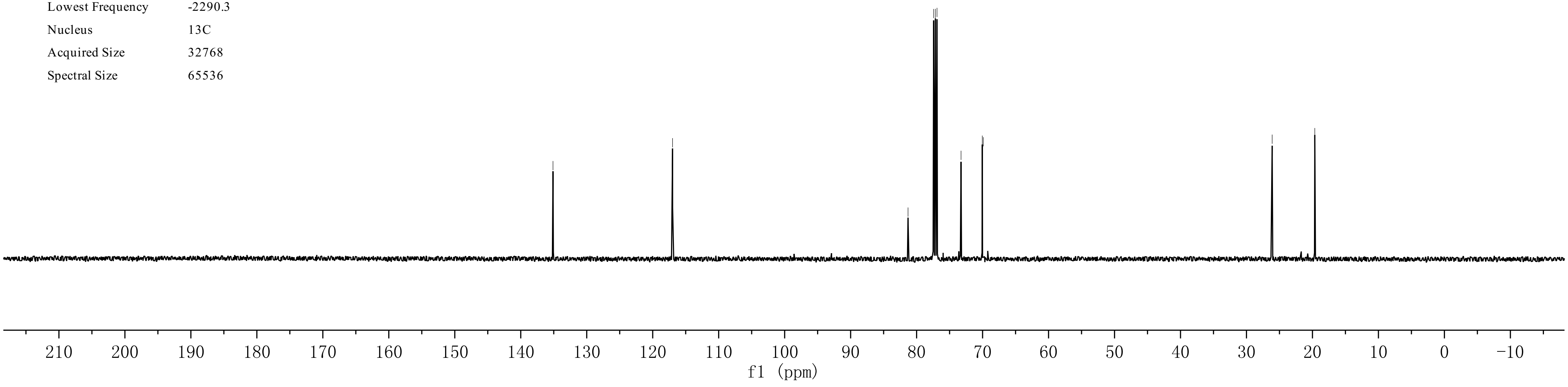

Parameter	Value
Data File Name	E:/ NMR/ 2017/ August/ 2017-8(10-17)/ xfy-0814-5/ 12/ fid
Comment	
Origin	Bruker BioSpin GmbH
Owner	nmr
Site	
Instrument	spect
Solvent	CDCl3
Temperature	296.1
Pulse Sequence	zgpg30
Experiment	1D
Number of Scans	14
Receiver Gain	193.1
Relaxation Delay	2.0000
Pulse Width	9.6000
Presaturation Frequency	
Acquisition Time	1.1010
Class	
Spectrometer Frequency	125.77
Spectral Width	29761.9
Lowest Frequency	-2290.3
Nucleus	13C
Acquired Size	32768
Spectral Size	65536

Parameter	Value
Data File Name	E:/ NMR/ 2017/ 2017-8(10-17)/ xfy-0815-1/ 1/ fid
Comment	
Origin	Bruker BioSpin GmbH
Owner	nmr
Site	
Instrument	spect
Solvent	CDCl3
Temperature	296.6
Pulse Sequence	zg30
Experiment	1D
Number of Scans	8
Receiver Gain	25.3
Relaxation Delay	1.0000
Pulse Width	9.6000
Presaturation Frequency	
Acquisition Time	1.9999
Class	
Spectrometer Frequency	400.13
Spectral Width	8012.8
Lowest Frequency	-1532.6
Nucleus	1H
Acquired Size	16025
Spectral Size	32768

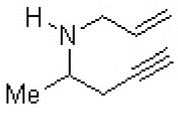

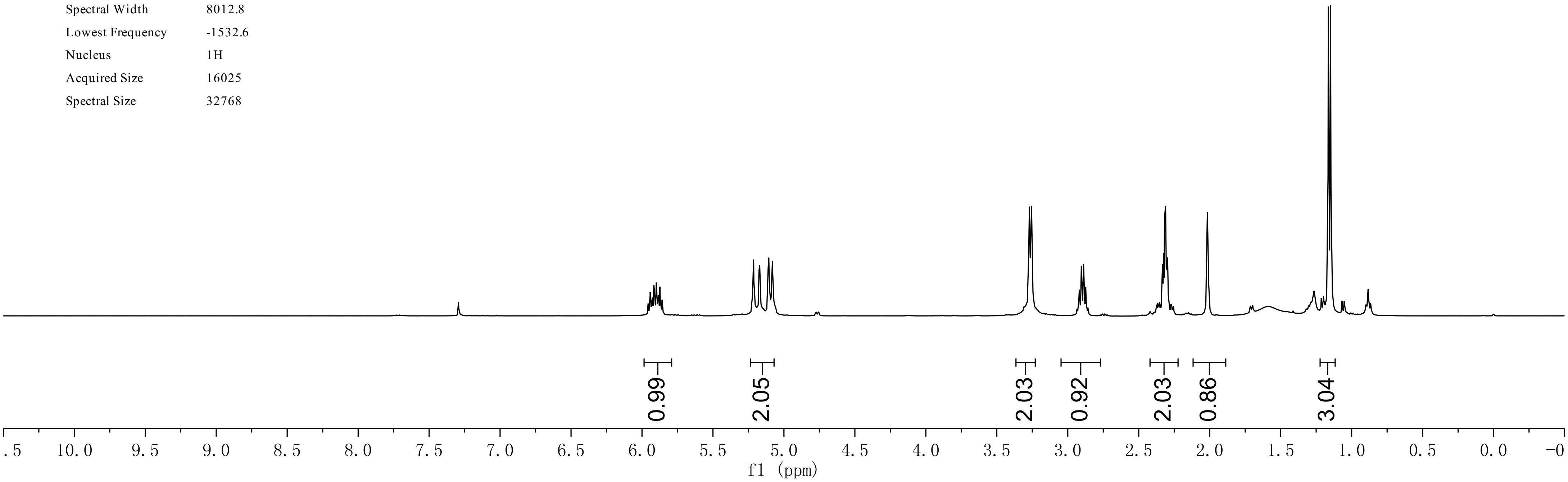

Parameter	Value
Data File Name	E:/ NMR/ 2017/ 2017-8(10-17)/ xfy-0815-1/ 2/ fid
Comment	
Origin	Bruker BioSpin GmbH
Owner	nmr
Site	
Instrument	spect
Solvent	CDCl3
Temperature	296.7
Pulse Sequence	zgpg30
Experiment	1D
Number of Scans	13
Receiver Gain	196.4
Relaxation Delay	2.0000
Pulse Width	10.0000
Presaturation Frequency	
Acquisition Time	1.3631
Class	
Spectrometer Frequency	100.61
Spectral Width	24038.5
Lowest Frequency	-1948.5
Nucleus	13C
Acquired Size	32768
Spectral Size	65536

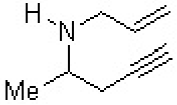

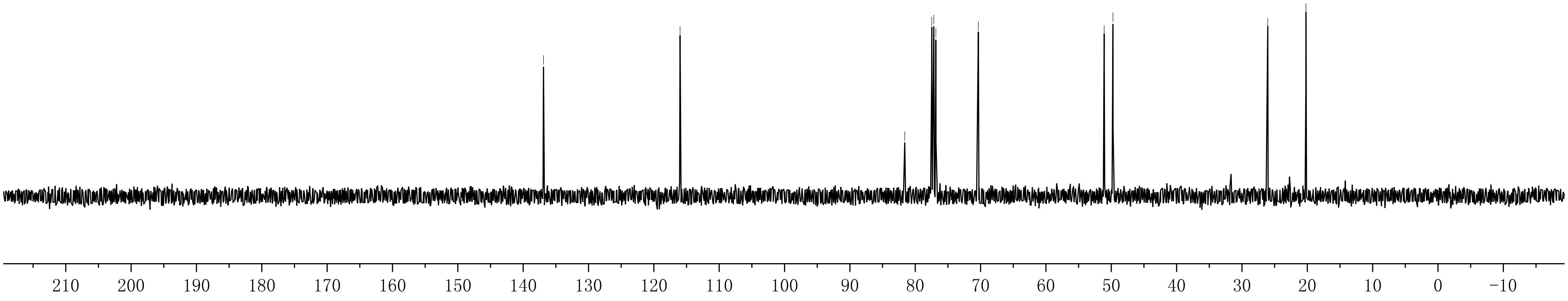

Parameter	Value
Data File Name	E:/ NMR/ 2017/ August/ 2017-8(10-17)/ xfy-0814-7/ 1/ fid
Comment	
Origin	Bruker BioSpin GmbH
Owner	nmr
Site	
Instrument	spect
Solvent	CDCl3
Temperature	296.1
Pulse Sequence	zg30
Experiment	1D
Number of Scans	13
Receiver Gain	31.1
Relaxation Delay	1.0000
Pulse Width	11.2900
Presaturation Frequency	
Acquisition Time	3.2768
Class	
Spectrometer Frequency	500.13
Spectral Width	10000.0
Lowest Frequency	-1911.5
Nucleus	1H
Acquired Size	32768
Spectral Size	65536

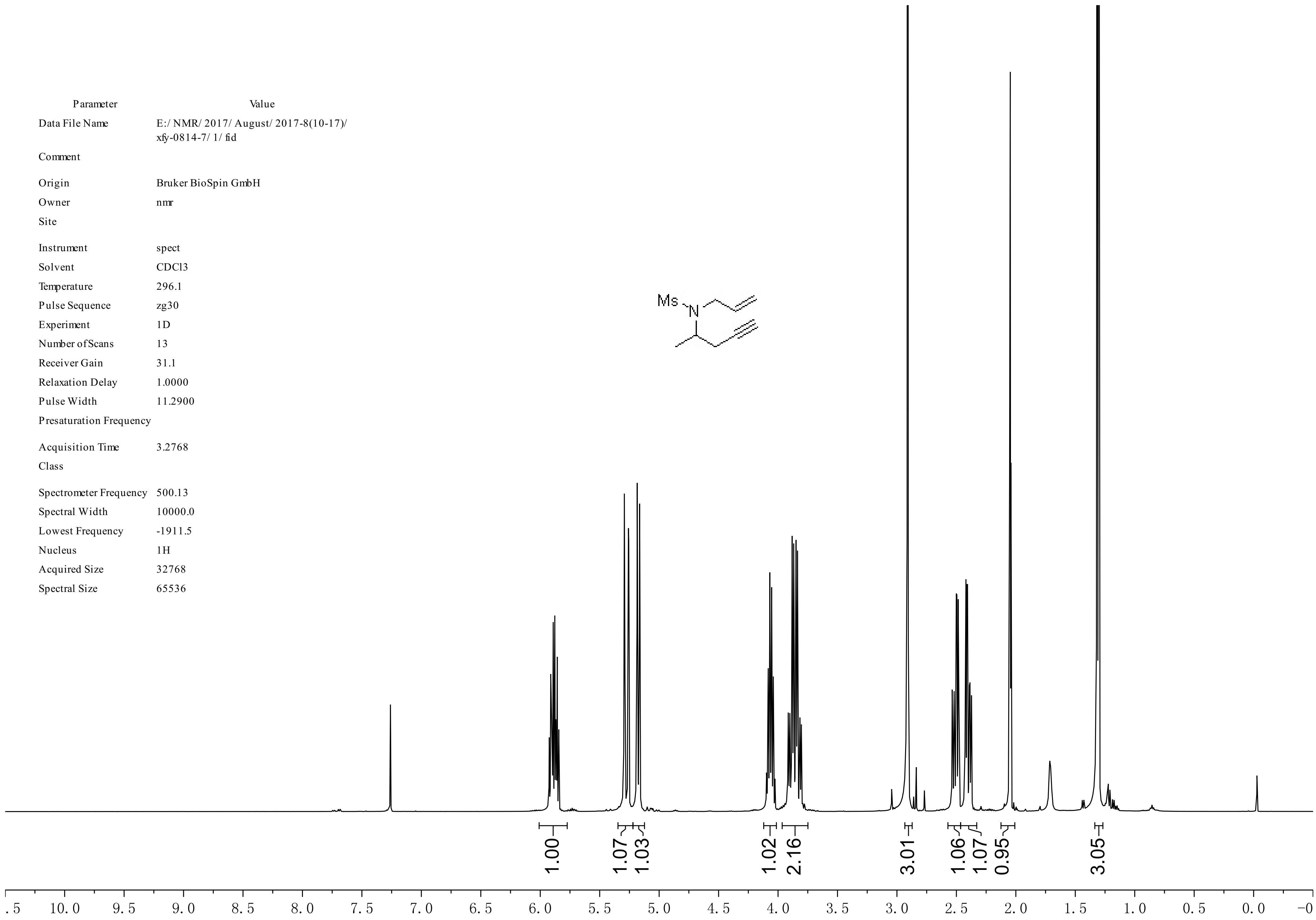

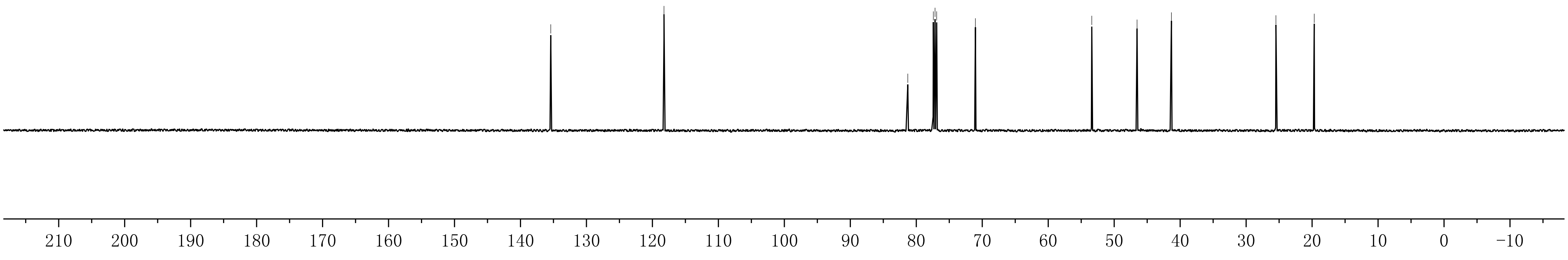

Parameter	Value
Data File Name	E:/ NMR/ 2017/ 2017-8(10-17)/ xfy-0814-7/ 2/ fid
Comment	
Origin	Bruker BioSpin GmbH
Owner	nmr
Site	
Instrument	spect
Solvent	CDCl3
Temperature	296.2
Pulse Sequence	zgpg30
Experiment	1D
Number of Scans	17
Receiver Gain	193.1
Relaxation Delay	2.0000
Pulse Width	9.6000
Presaturation Frequency	
Acquisition Time	1.1010
Class	
Spectrometer Frequency	125.76
Spectral Width	29761.9
Lowest Frequency	-2297.3
Nucleus	13C
Acquired Size	32768
Spectral Size	65536

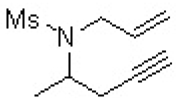

Parameter	Value
Title	
Comment	
Origin	Bruker BioSpin GmbH
Owner	nmr
Site	
Instrument	spect
Solvent	CDCl3
Temperature	296.2
Pulse Sequence	zg30
Experiment	1D
Number of Scans	16
Receiver Gain	77.6
Relaxation Delay	1.0000
Pulse Width	10.7100
Presaturation Frequency	
Acquisition Time	3.2768
Acquisition Date	2018-01-17T10:38:00
Modification Date	2018-01-17T10:47:43
Spectrometer Frequency	500.13
Spectral Width	10000.0
Lowest Frequency	-1920.7
Nucleus	1H
Acquired Size	32768
Spectral Size	65536

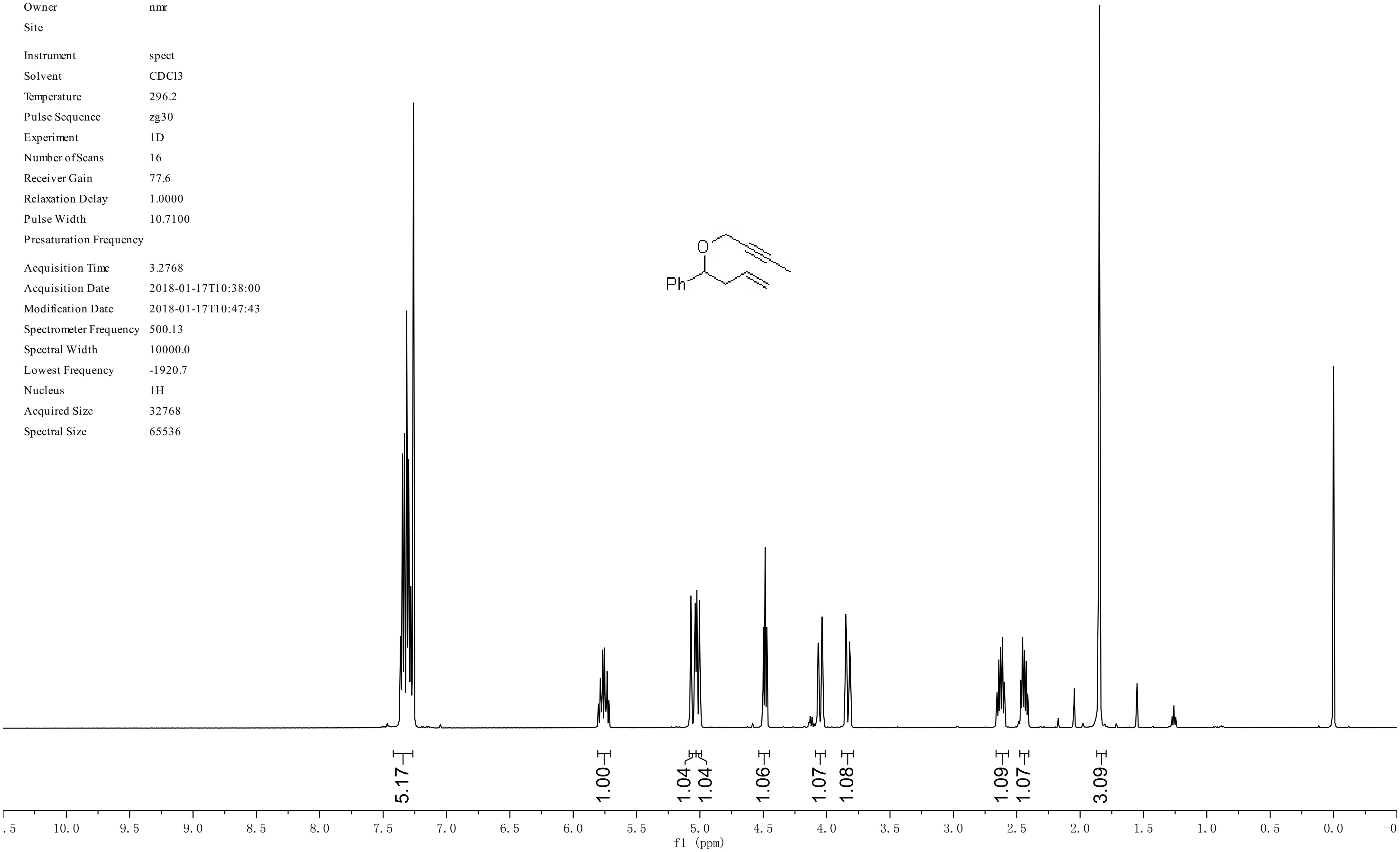

Parameter	Value
Title	
Comment	
Origin	Bruker BioSpin GmbH
Owner	nmr
Site	
Instrument	spect
Solvent	CDCl3
Temperature	294.3
Pulse Sequence	zgpg30
Experiment	1D
Number of Scans	7
Receiver Gain	196.4
Relaxation Delay	2.0000
Pulse Width	10.0000
Presaturation Frequency	
Acquisition Time	1.3631
Acquisition Date	2018-04-16T16:26:41
Modification Date	2018-04-16T16:34:06
Spectrometer Frequency	100.61
Spectral Width	24038.5
Lowest Frequency	-1949.5
Nucleus	13C
Acquired Size	32768
Spectral Size	65536

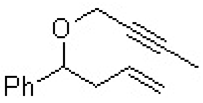

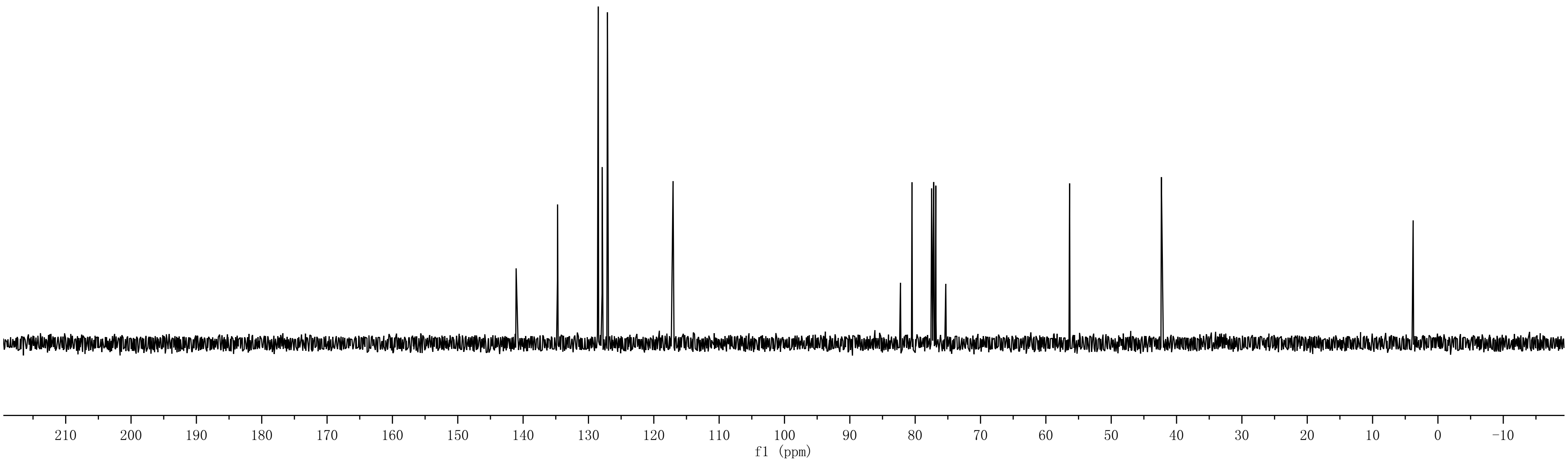

Parameter	Value
Title	
Comment	
Origin	Bruker BioSpin GmbH
Owner	nmr
Site	
Instrument	spect
Solvent	CDCl3
Temperature	296.2
Pulse Sequence	zg30
Experiment	1D
Number of Scans	16
Receiver Gain	77.6
Relaxation Delay	1.0000
Pulse Width	10.7100
Presaturation Frequency	
Acquisition Time	3.2768
Acquisition Date	2018-03-10T13:19:12
Modification Date	2018-03-10T21:21:07
Spectrometer Frequency	500.13
Spectral Width	10000.0
Lowest Frequency	-1922.5
Nucleus	1H
Acquired Size	32768
Spectral Size	65536

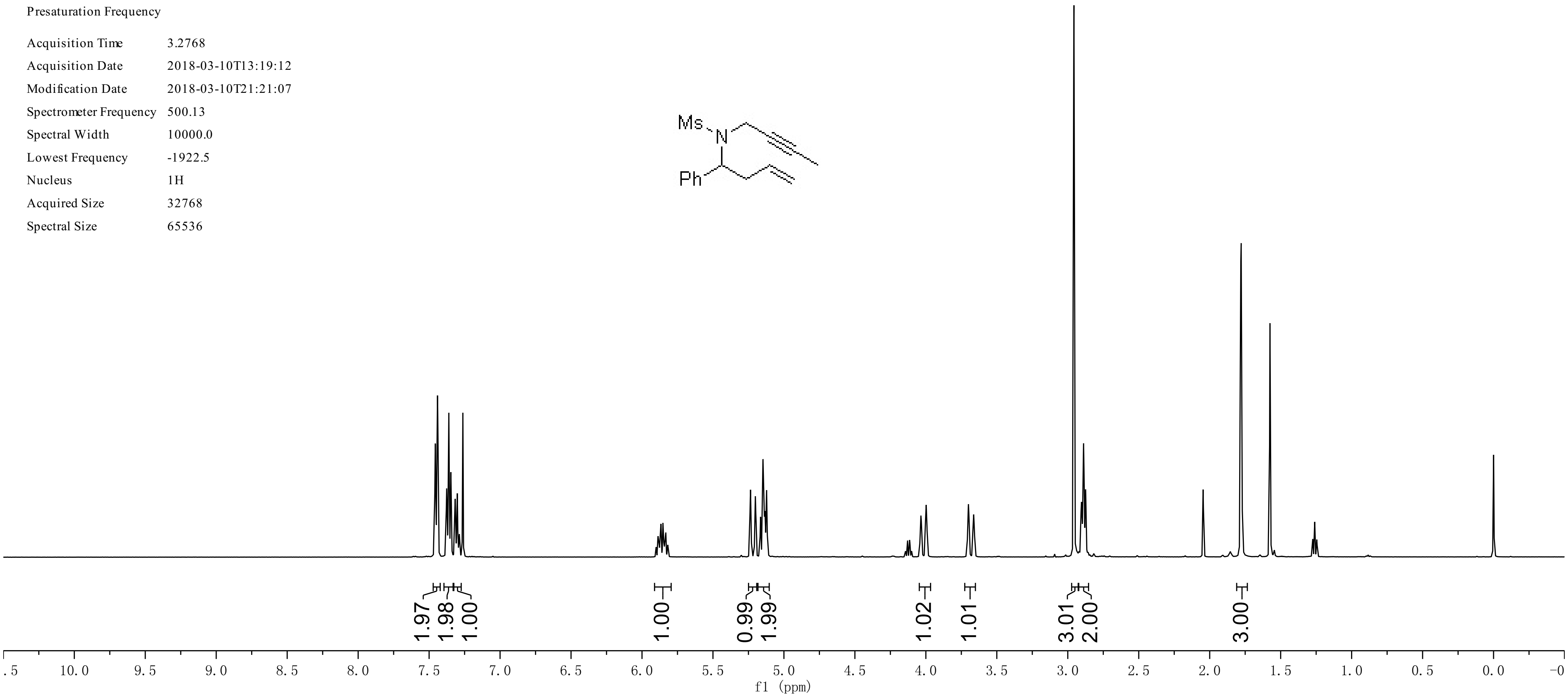

Parameter	Value
Title	
Comment	
Origin	Bruker BioSpin GmbH
Owner	nmr
Site	
Instrument	spect
Solvent	CDCl3
Temperature	296.2
Pulse Sequence	zgpg30
Experiment	1D
Number of Scans	50
Receiver Gain	193.1
Relaxation Delay	2.0000
Pulse Width	9.6000
Presaturation Frequency	
Acquisition Time	1.1010
Acquisition Date	2018-04-16T03:02:40
Modification Date	2018-04-16T09:48:32
Spectrometer Frequency	125.77
Spectral Width	29761.9
Lowest Frequency	-2290.9
Nucleus	13C
Acquired Size	32768
Spectral Size	65536

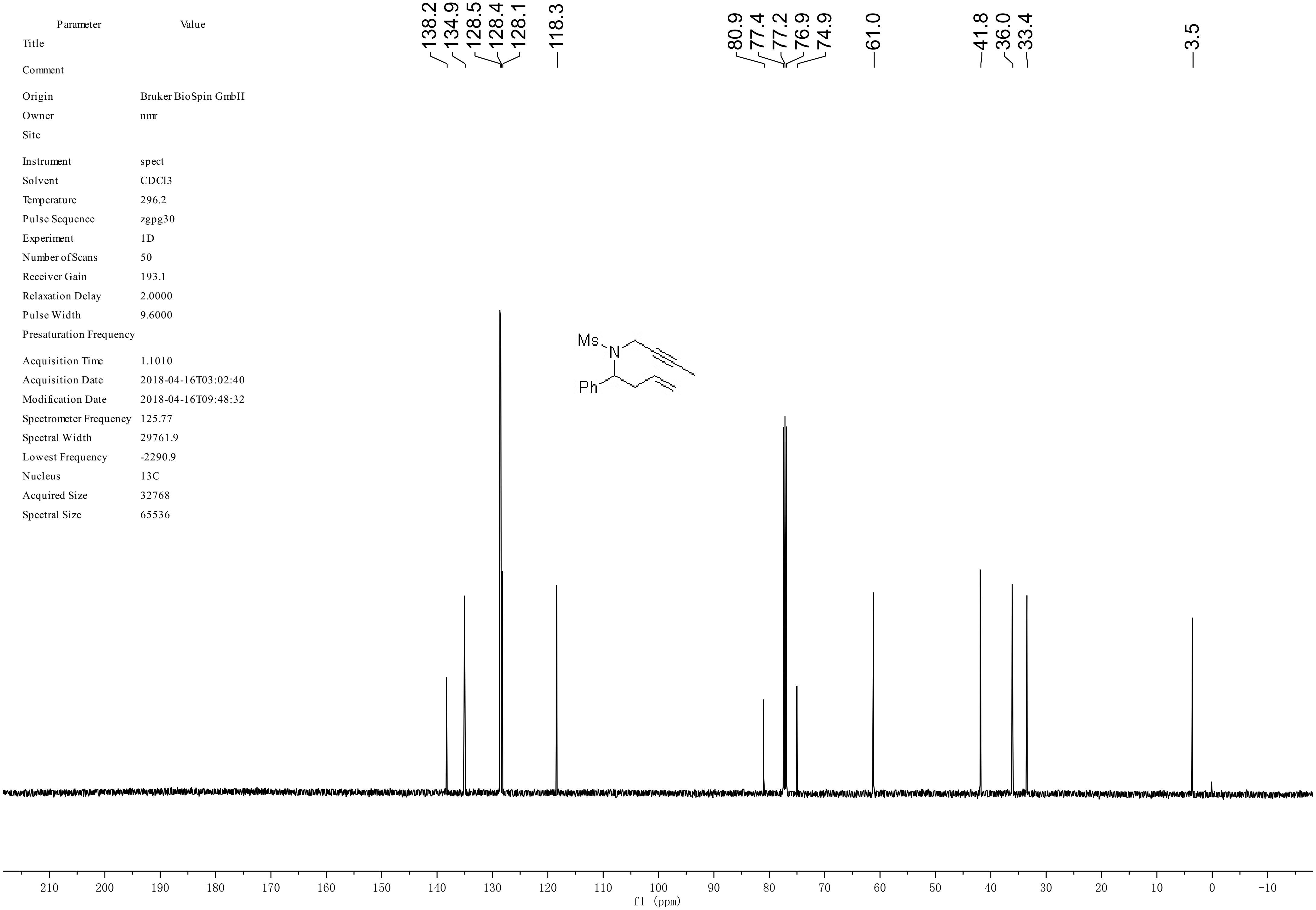

Parameter	Value
Title	
Comment	
Origin	Bruker BioSpin GmbH
Owner	nmr
Site	
Instrument	spect
Solvent	CDCl3
Temperature	296.2
Pulse Sequence	zg30
Experiment	1D
Number of Scans	16
Receiver Gain	48.5
Relaxation Delay	1.0000
Pulse Width	10.7100
Presaturation Frequency	
Acquisition Time	3.2768
Acquisition Date	2018-06-12T22:17:44
Modification Date	2018-06-13T10:25:21
Spectrometer Frequency	500.13
Spectral Width	10000.0
Lowest Frequency	-1911.5
Nucleus	1H
Acquired Size	32768
Spectral Size	65536

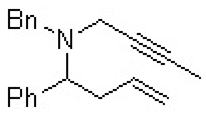

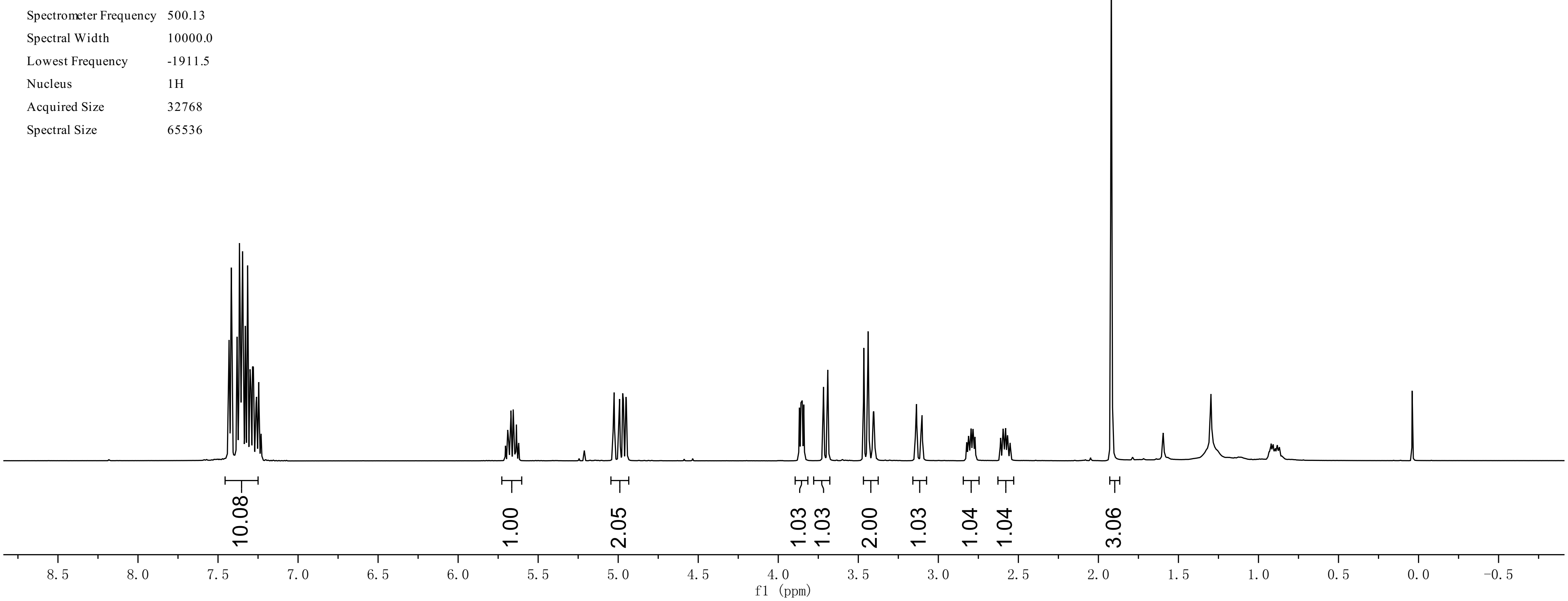

Parameter	Value
Title	
Comment	
Origin	Bruker BioSpin GmbH
Owner	nmr
Site	
Instrument	spect
Solvent	CDCl3
Temperature	296.2
Pulse Sequence	zgpg30
Experiment	1D
Number of Scans	250
Receiver Gain	193.1
Relaxation Delay	2.0000
Pulse Width	9.6000
Presaturation Frequency	
Acquisition Time	1.1010
Acquisition Date	2018-06-12T22:32:13
Modification Date	2018-06-13T10:25:21
Spectrometer Frequency	125.77
Spectral Width	29761.9
Lowest Frequency	-2276.8
Nucleus	13C
Acquired Size	32768
Spectral Size	65536

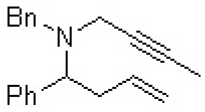

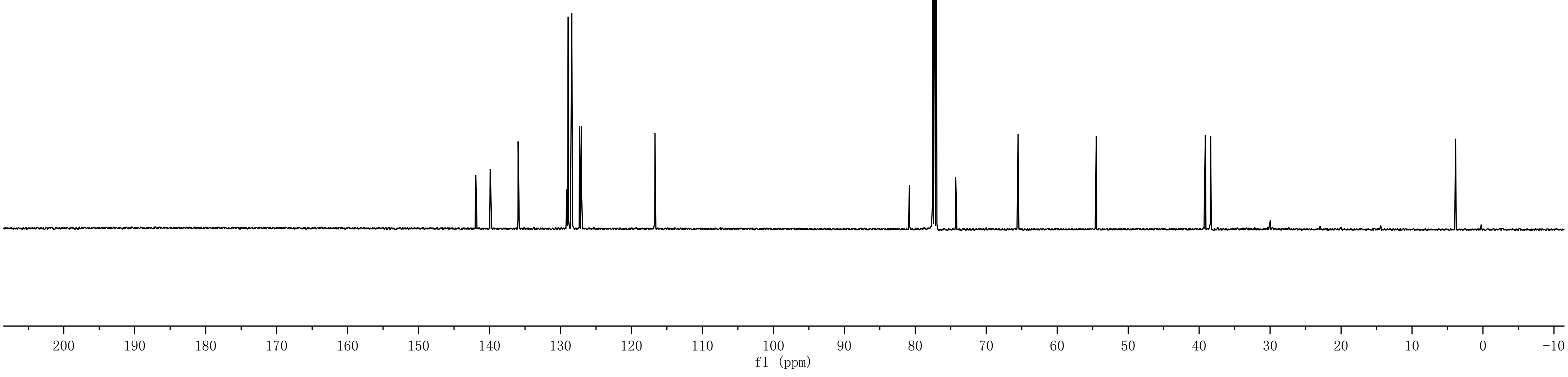

Parameter	Value
标题	xfy-190326-5-s2.2.fid
Comment	
Origin	Bruker BioSpin GmbH
Owner	nmr
Site	
Instrument	spect
Author	
Solvent	CDCl3
Temperature	296.1
Pulse Sequence	zg30
Experiment	1D
Number of Scans	4
Receiver Gain	95.3
Relaxation Delay	1.0000
Pulse Width	10.7100
Presaturation Frequency	
Acquisition Time	3.2768
Class	
Spectrometer Frequency	500.13
Spectral Width	10000.0
Lowest Frequency	-1911.5
Nucleus	1H
Acquired Size	32768
Spectral Size	65536

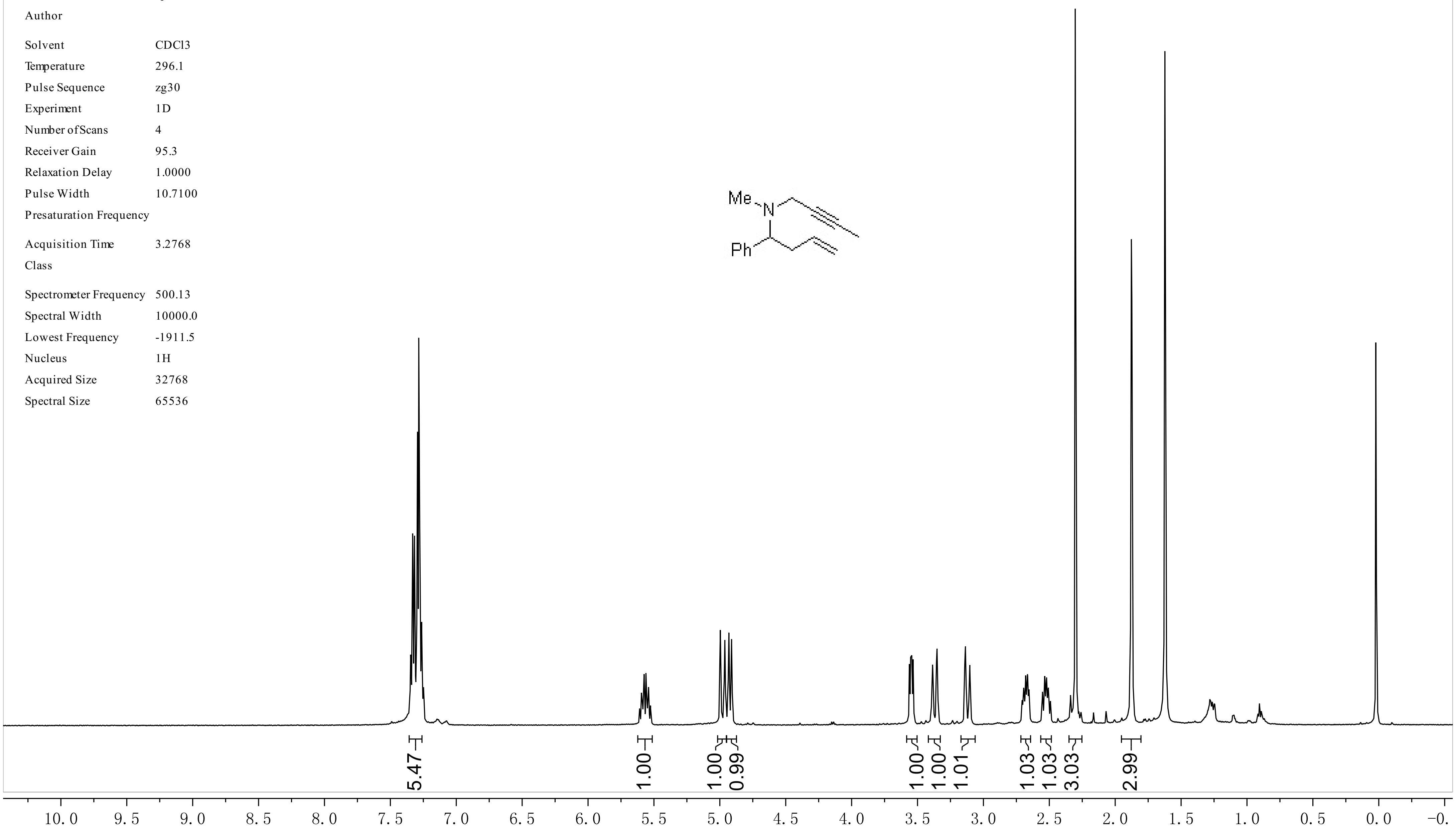

Parameter	Value
标题	xfy-190326-5-s2.3.fid
Comment	
Origin	Bruker BioSpin GmbH
Owner	nmr
Site	
Instrument	spect
Author	
Solvent	CDCl3
Temperature	296.2
Pulse Sequence	zgpg30
Experiment	1D
Number of Scans	75
Receiver Gain	193.1
Relaxation Delay	2.0000
Pulse Width	9.6000
Presaturation Frequency	
Acquisition Time	1.1010
Class	
Spectrometer Frequency	125.77
Spectral Width	29761.9
Lowest Frequency	-2288.8
Nucleus	13C
Acquired Size	32768
Spectral Size	65536

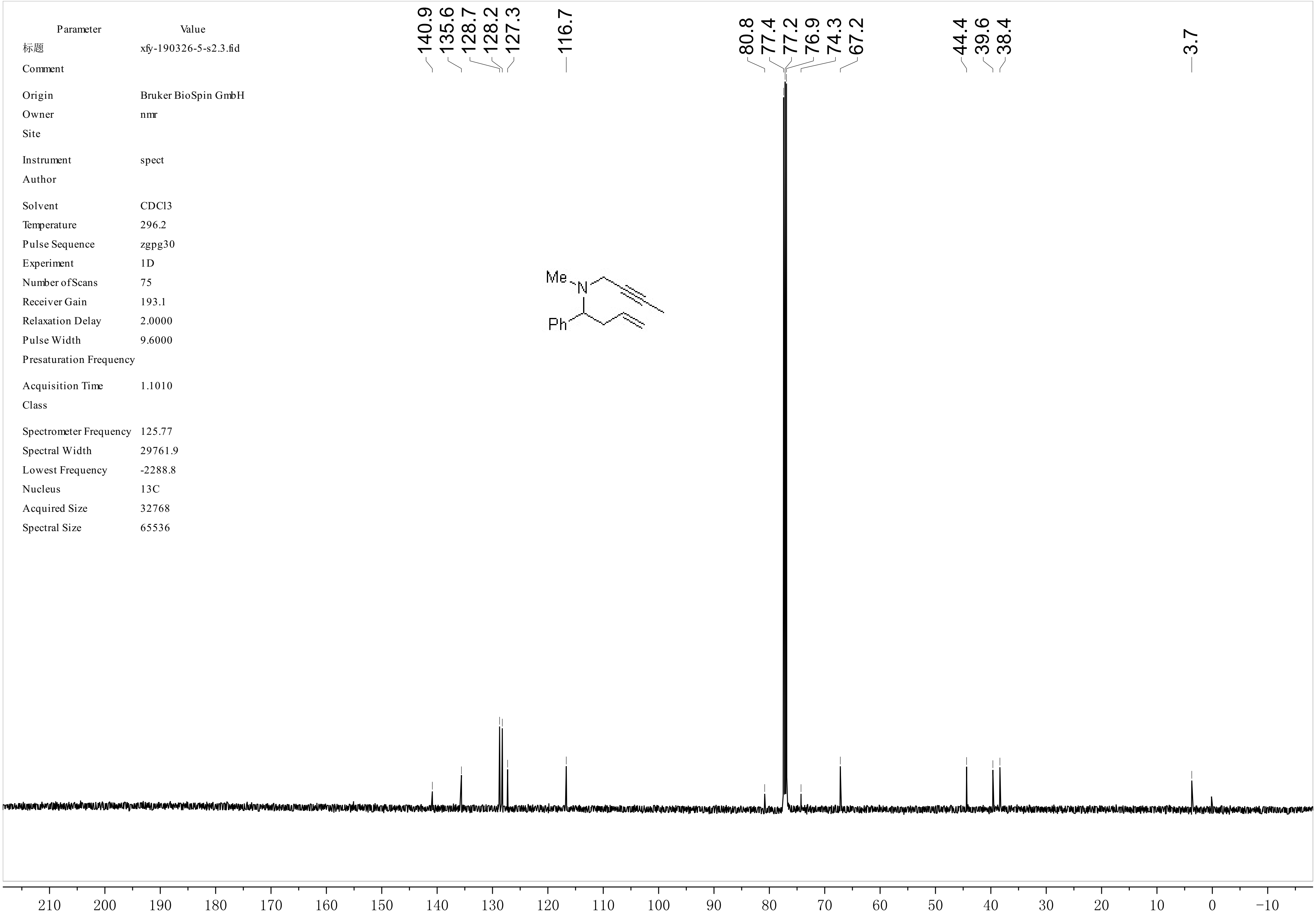

Parameter	Value
标题	xfy-190307-5.3.1.1r
Comment	
Origin	Bruker BioSpin GmbH
Owner	nmr
Site	
Instrument	spect
Author	
Solvent	CDCl3
Temperature	296.1
Pulse Sequence	zg30
Experiment	1D
Number of Scans	8
Receiver Gain	62.9
Relaxation Delay	1.0000
Pulse Width	10.7100
Presaturation Frequency	
Acquisition Time	3.2768
Class	
Spectrometer Frequency	500.13
Spectral Width	10000.0
Lowest Frequency	-1911.5
Nucleus	1H
Acquired Size	32768
Spectral Size	65536

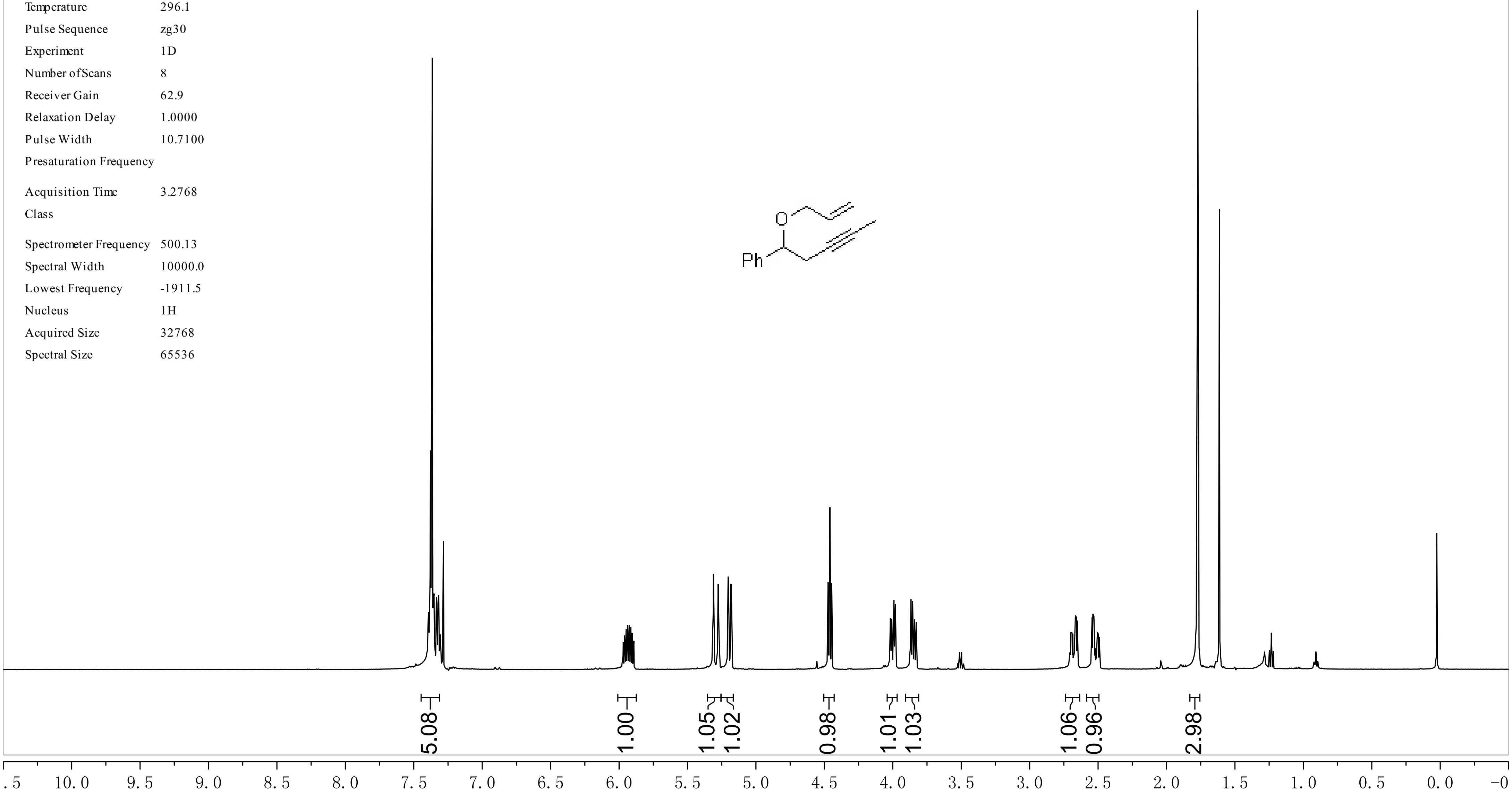

Parameter	Value
Title	xfy-190307-5.4.fid
Comment	
Origin	Bruker BioSpin GmbH
Owner	nmr
Site	
Instrument	spect
Solvent	CDCl3
Temperature	296.1
Pulse Sequence	zgpg30
Experiment	1D
Number of Scans	25
Receiver Gain	193.1
Relaxation Delay	2.0000
Pulse Width	9.6000
Presaturation Frequency	
Acquisition Time	1.1010
Class	
Spectrometer Frequency	125.77
Spectral Width	29761.9
Lowest Frequency	-2290.4
Nucleus	13C
Acquired Size	32768
Spectral Size	65536

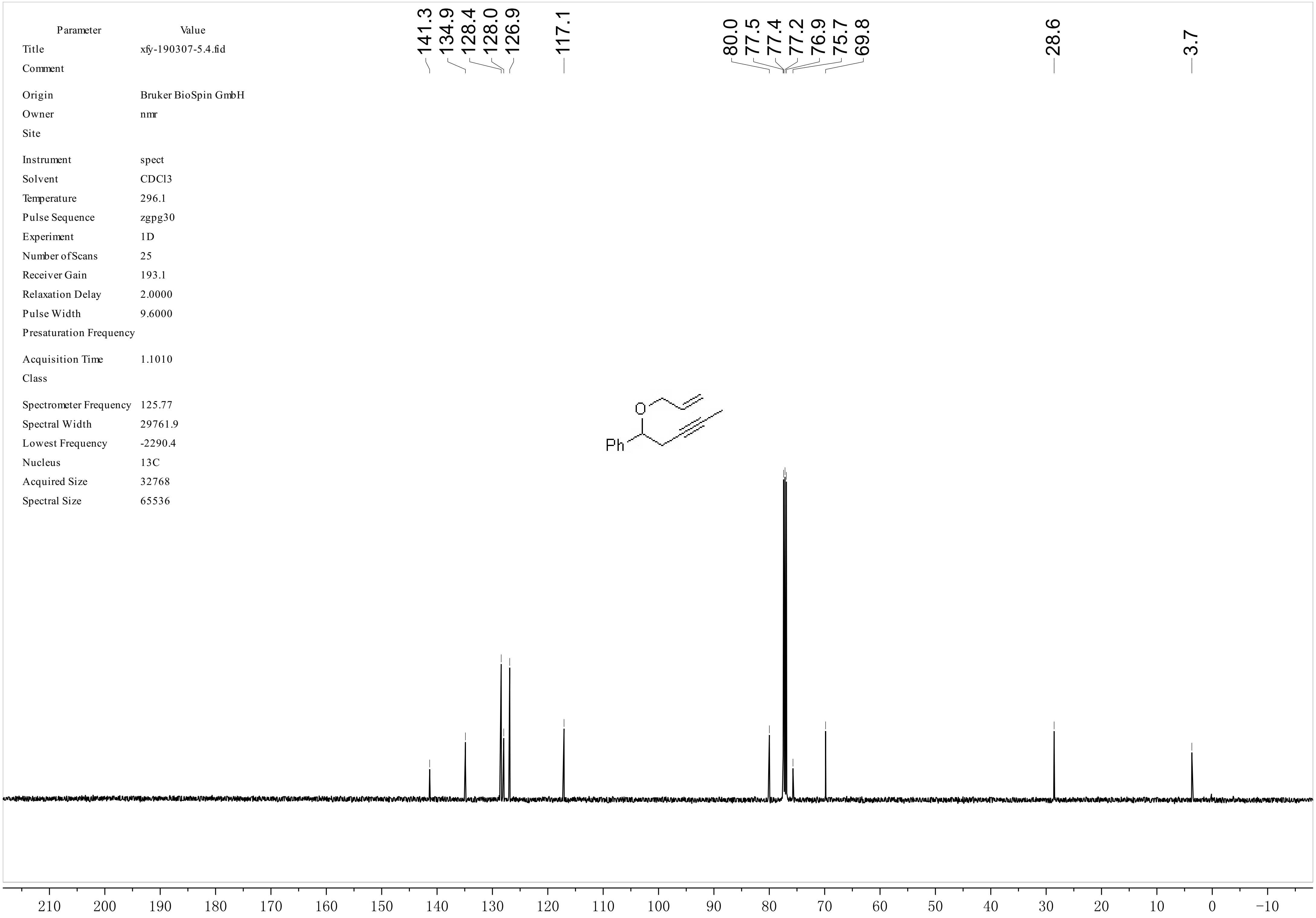

Parameter	Value
Title	xfy-190313-2.11.1.1r
Comment	
Origin	Bruker BioSpin GmbH
Owner	nmr
Site	
Instrument	spect
Solvent	CDCl3
Temperature	294.0
Pulse Sequence	zg30
Experiment	1D
Number of Scans	8
Receiver Gain	31.7
Relaxation Delay	1.0000
Pulse Width	8.7300
Presaturation Frequency	
Acquisition Time	1.9999
Class	
Spectrometer Frequency	400.13
Spectral Width	8012.8
Lowest Frequency	-1535.4
Nucleus	1H
Acquired Size	16025
Spectral Size	65536

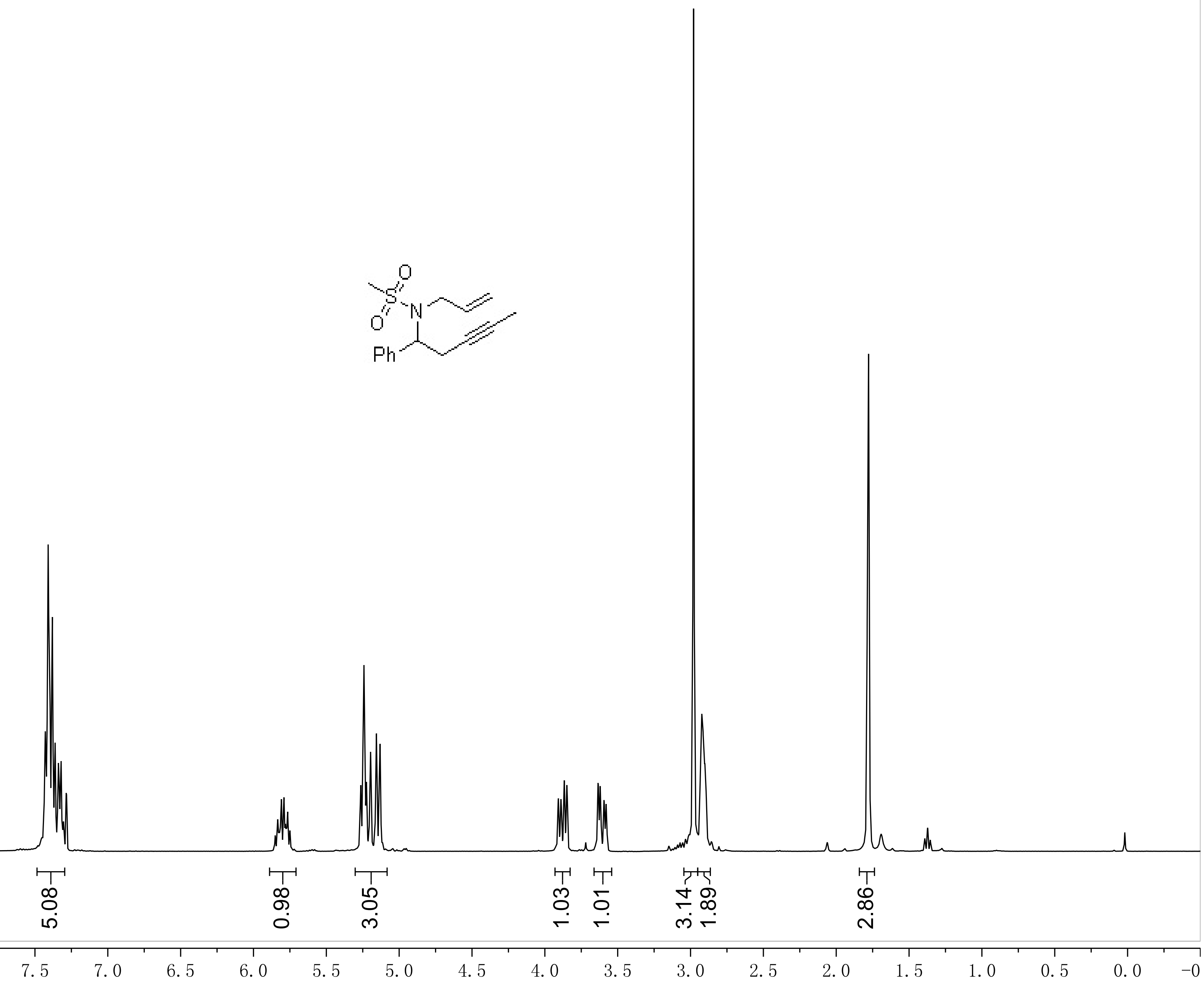

Parameter	Value
Title	xfy-190313-2.12.fid
Comment	
Origin	Bruker BioSpin GmbH
Owner	nmr
Site	
Instrument	spect
Solvent	CDCl3
Temperature	294.2
Pulse Sequence	zgpg30
Experiment	1D
Number of Scans	18
Receiver Gain	196.4
Relaxation Delay	2.0000
Pulse Width	10.0000
Presaturation Frequency	
Acquisition Time	1.3631
Class	
Spectrometer Frequency	100.62
Spectral Width	24038.5
Lowest Frequency	-1950.4
Nucleus	13C
Acquired Size	32768
Spectral Size	65536

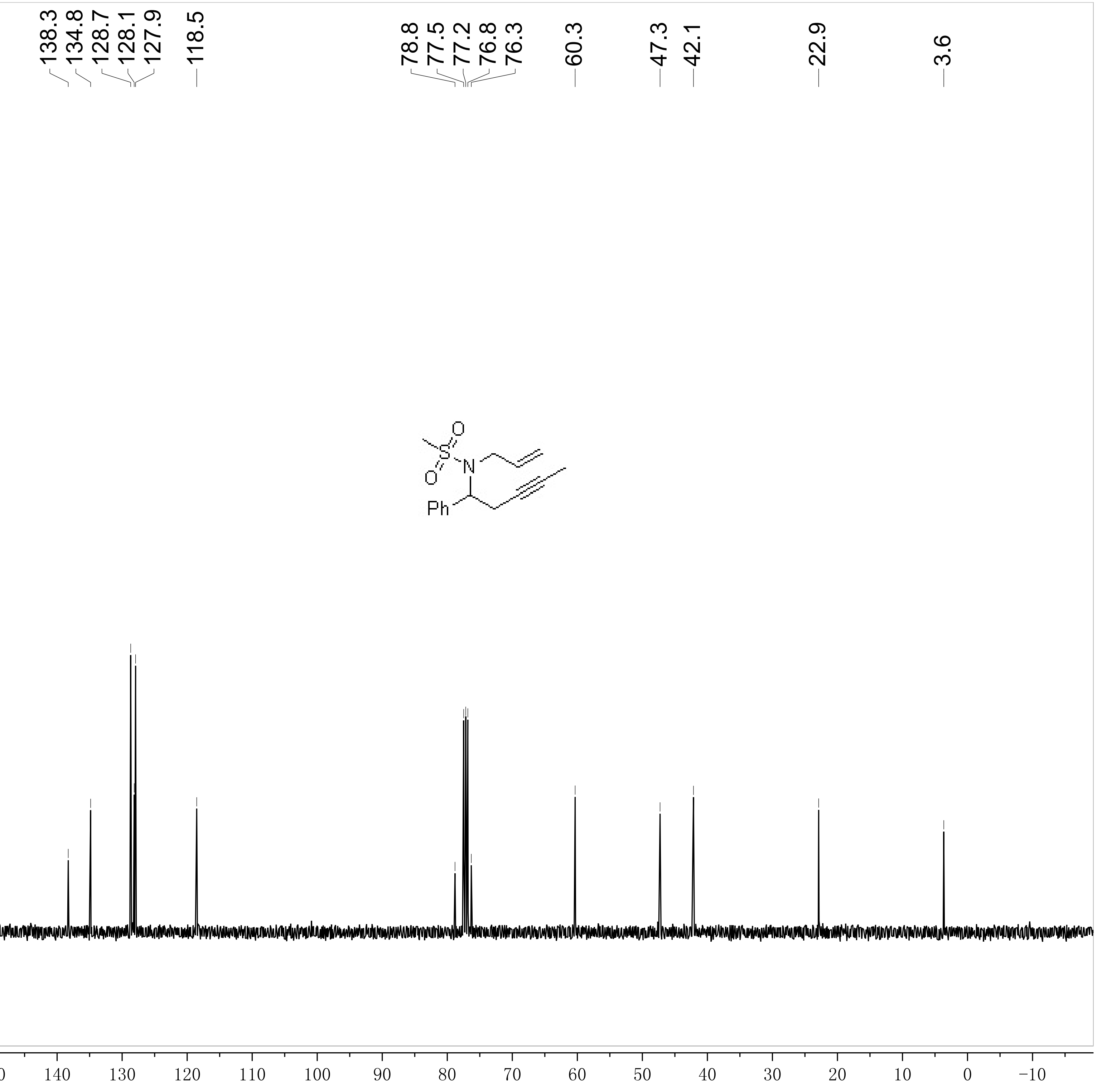

Parameter	Value
Title	xfy-190309-2.21.1.1r
Comment	
Origin	Bruker BioSpin GmbH
Owner	nmr
Site	
Instrument	spect
Solvent	CDCl3
Temperature	296.1
Pulse Sequence	zg30
Experiment	1D
Number of Scans	16
Receiver Gain	31.1
Relaxation Delay	1.0000
Pulse Width	10.7100
Presaturation Frequency	
Acquisition Time	3.2768
Class	
Spectrometer Frequency	500.13
Spectral Width	10000.0
Lowest Frequency	-1921.3
Nucleus	1H
Acquired Size	32768
Spectral Size	65536

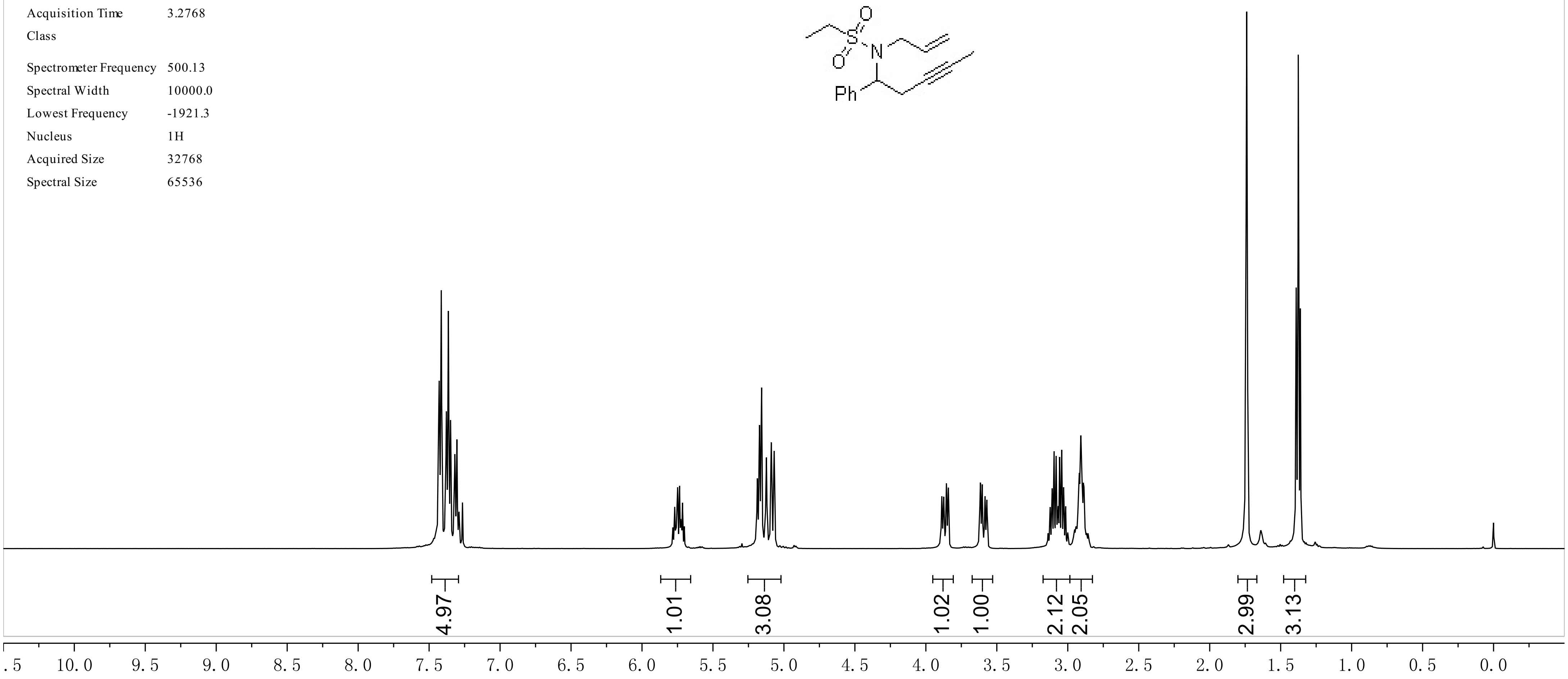

Parameter	Value
Title	xfy-190309-2.22.1.1r
Comment	
Origin	Bruker BioSpin GmbH
Owner	nmr
Site	
Instrument	spect
Solvent	CDCl3
Temperature	296.2
Pulse Sequence	zgpg30
Experiment	1D
Number of Scans	24
Receiver Gain	193.1
Relaxation Delay	2.0000
Pulse Width	9.6000
Presaturation Frequency	
Acquisition Time	1.1010
Class	
Spectrometer Frequency	125.76
Spectral Width	29761.9
Lowest Frequency	-2294.4
Nucleus	13C
Acquired Size	32768
Spectral Size	32768

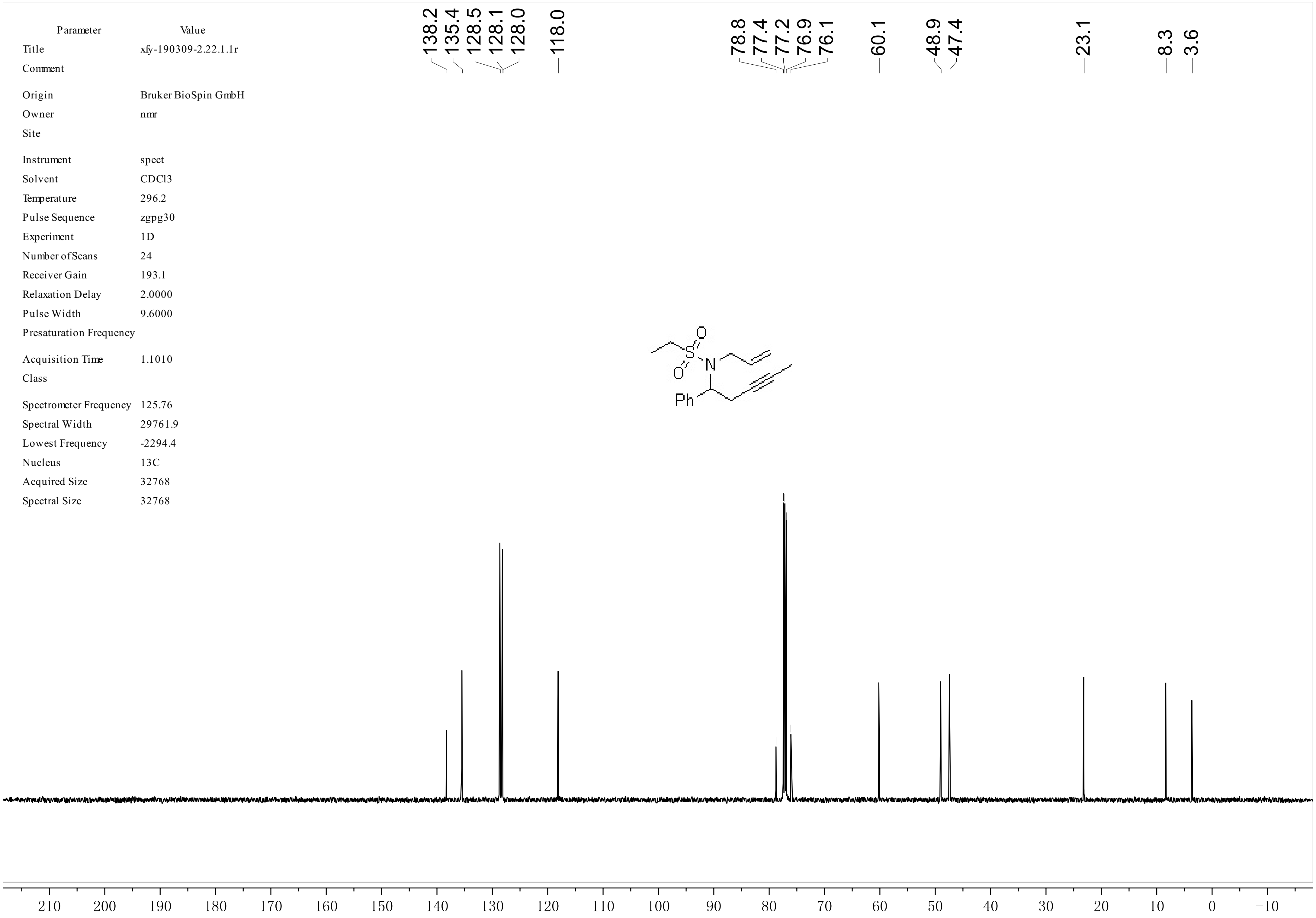

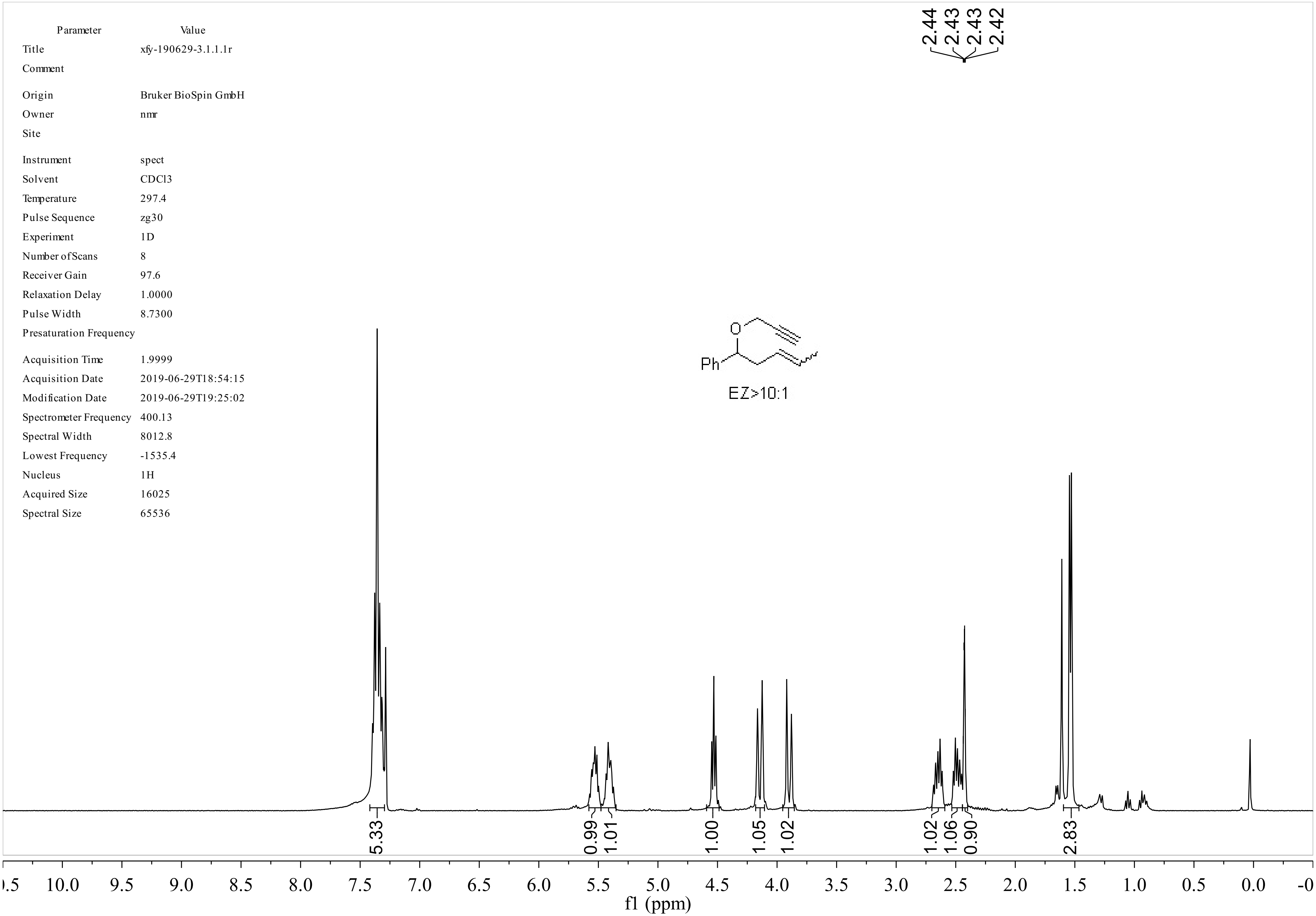

Parameter Value
Title xfy-190629-3.1.1.1r
Comment
Origin Bruker BioSpin GmbH
Owner nmr
Site
Instrument spect
Solvent CDCl3
Temperature 297.4
Pulse Sequence zg30
Experiment 1D
Number of Scans 8
Receiver Gain 97.6
Relaxation Delay 1.0000
Pulse Width 8.7300
Presaturation Frequency
Acquisition Time 1.9999
Acquisition Date 2019-06-29T18:54:15
Modification Date 2019-06-29T19:25:02
Spectrometer Frequency 400.13
Spectral Width 8012.8
Lowest Frequency -1535.4
Nucleus 1H
Acquired Size 16025
Spectral Size 65536
2.44
2.43
2.43
2.42
O
Ph
EZ>10:1
5.33
0.99
1.01
1.00
1.05
1.02
1.02
1.06
0.90
2.83
10.0 9.5 9.0 8.5 8.0 7.5 7.0 6.5 6.0 5.5 5.0 4.5 4.0 3.5 3.0 2.5 2.0 1.5 1.0 0.5 0.0
f1 (ppm)

Parameter	Value
Title	xfy-190629-3.2.fid
Comment	
Origin	Bruker BioSpin GmbH
Owner	nmr
Site	
Instrument	spect
Solvent	CDCl3
Temperature	297.7
Pulse Sequence	zgpg30
Experiment	1D
Number of Scans	32
Receiver Gain	196.4
Relaxation Delay	2.0000
Pulse Width	10.0000
Presaturation Frequency	
Acquisition Time	1.3631
Acquisition Date	2019-06-29T18:56:13
Modification Date	2019-06-29T19:25:03
Spectrometer Frequency	100.62
Spectral Width	24038.5
Lowest Frequency	-1958.0
Nucleus	13C
Acquired Size	32768
Spectral Size	65536

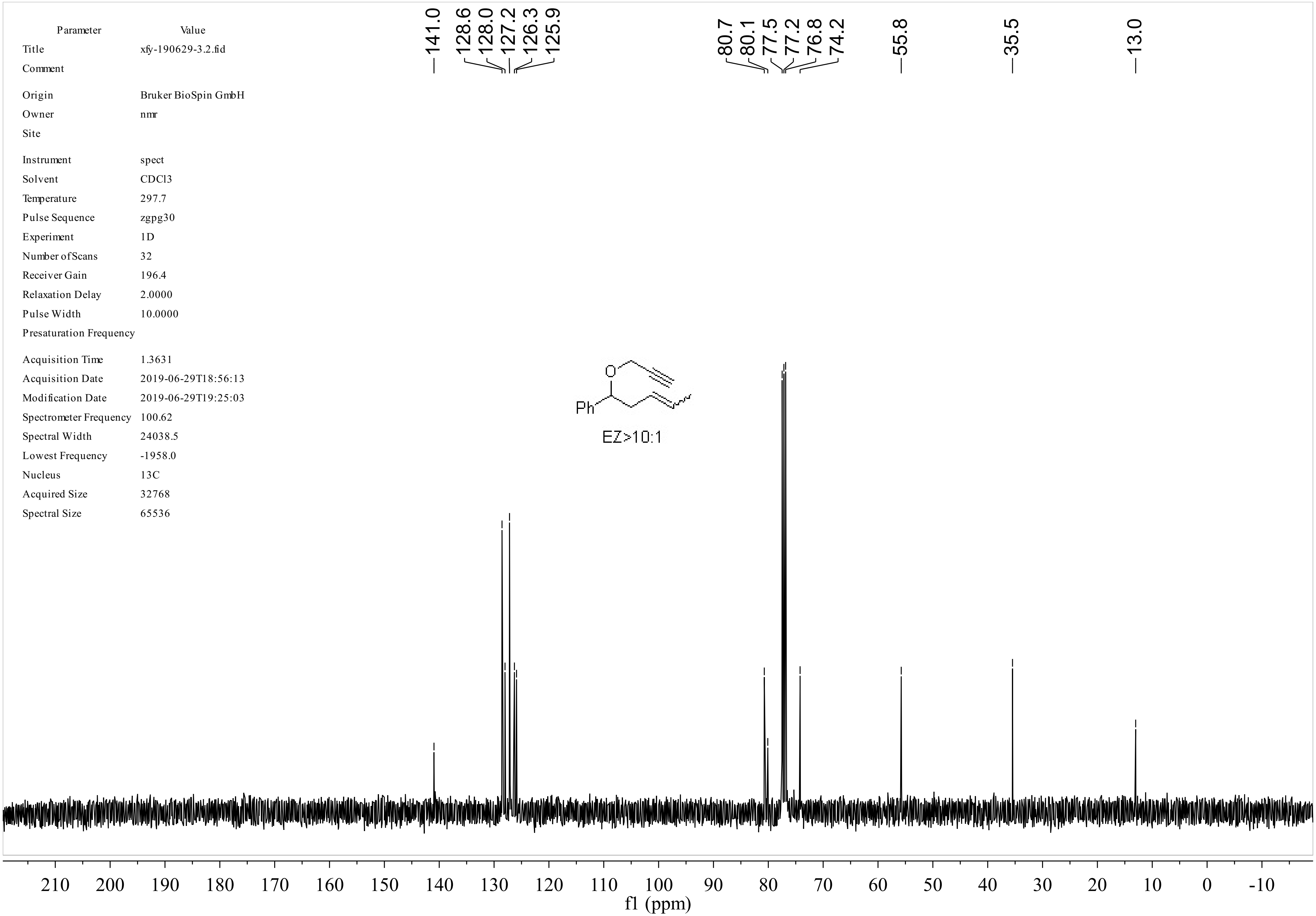

Parameter	Value
Title	xfy-191025-0.41.fid
Comment	
Origin	Bruker BioSpin GmbH
Owner	nmr
Site	
Instrument	spect
Solvent	CDCl3
Temperature	296.3
Pulse Sequence	zg30
Experiment	1D
Number of Scans	8
Receiver Gain	111.4
Relaxation Delay	1.0000
Pulse Width	8.7300
Presaturation Frequency	
Acquisition Time	1.9999
Acquisition Date	2019-10-27T20:48:54
Modification Date	2019-10-28T09:32:48
Spectrometer Frequency	400.13
Spectral Width	8012.8
Lowest Frequency	-1535.4
Nucleus	1H
Acquired Size	16025
Spectral Size	65536

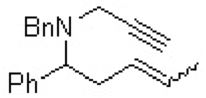

EZ=1.2:1

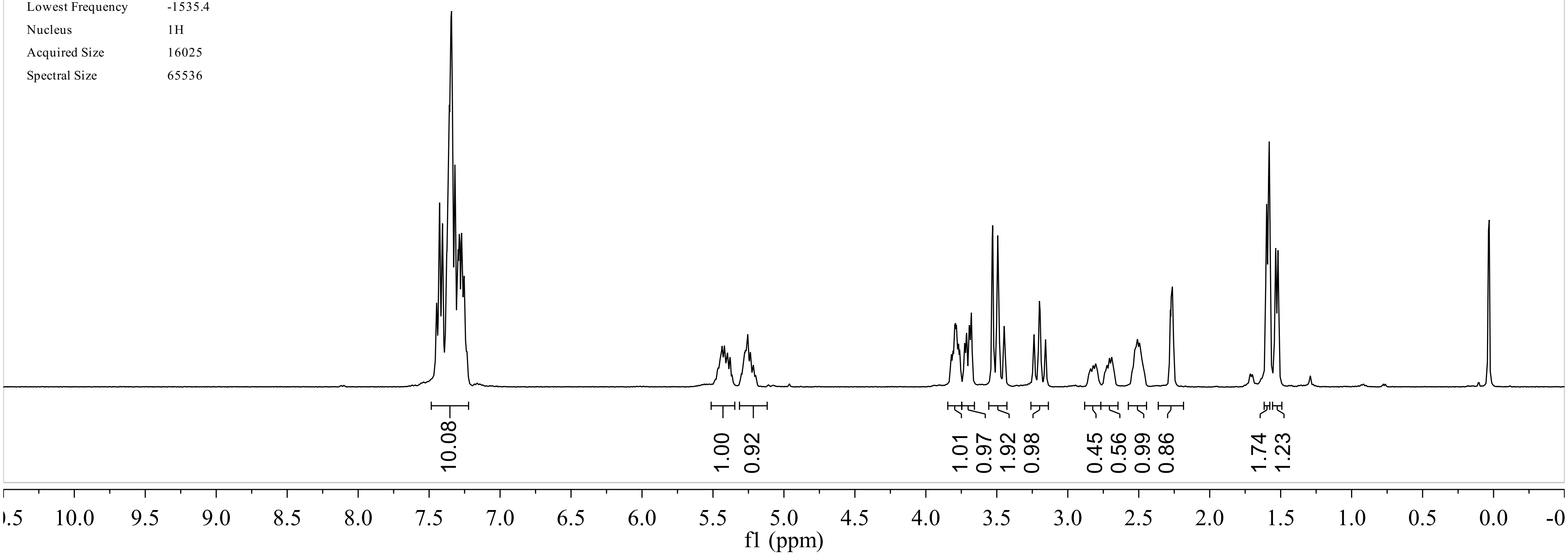

Parameter	Value
Title	xfy-191025-0.42.fid
Comment	
Origin	Bruker BioSpin GmbH
Owner	nmr
Site	
Instrument	spect
Solvent	CDCl3
Temperature	297.0
Pulse Sequence	zgpg30
Experiment	1D
Number of Scans	256
Receiver Gain	196.4
Relaxation Delay	2.0000
Pulse Width	10.0000
Presaturation Frequency	
Acquisition Time	1.3631
Acquisition Date	2019-10-27T21:04:49
Modification Date	2019-10-28T09:32:49
Spectrometer Frequency	100.62
Spectral Width	24038.5
Lowest Frequency	-1944.7
Nucleus	13C
Acquired Size	32768
Spectral Size	65536

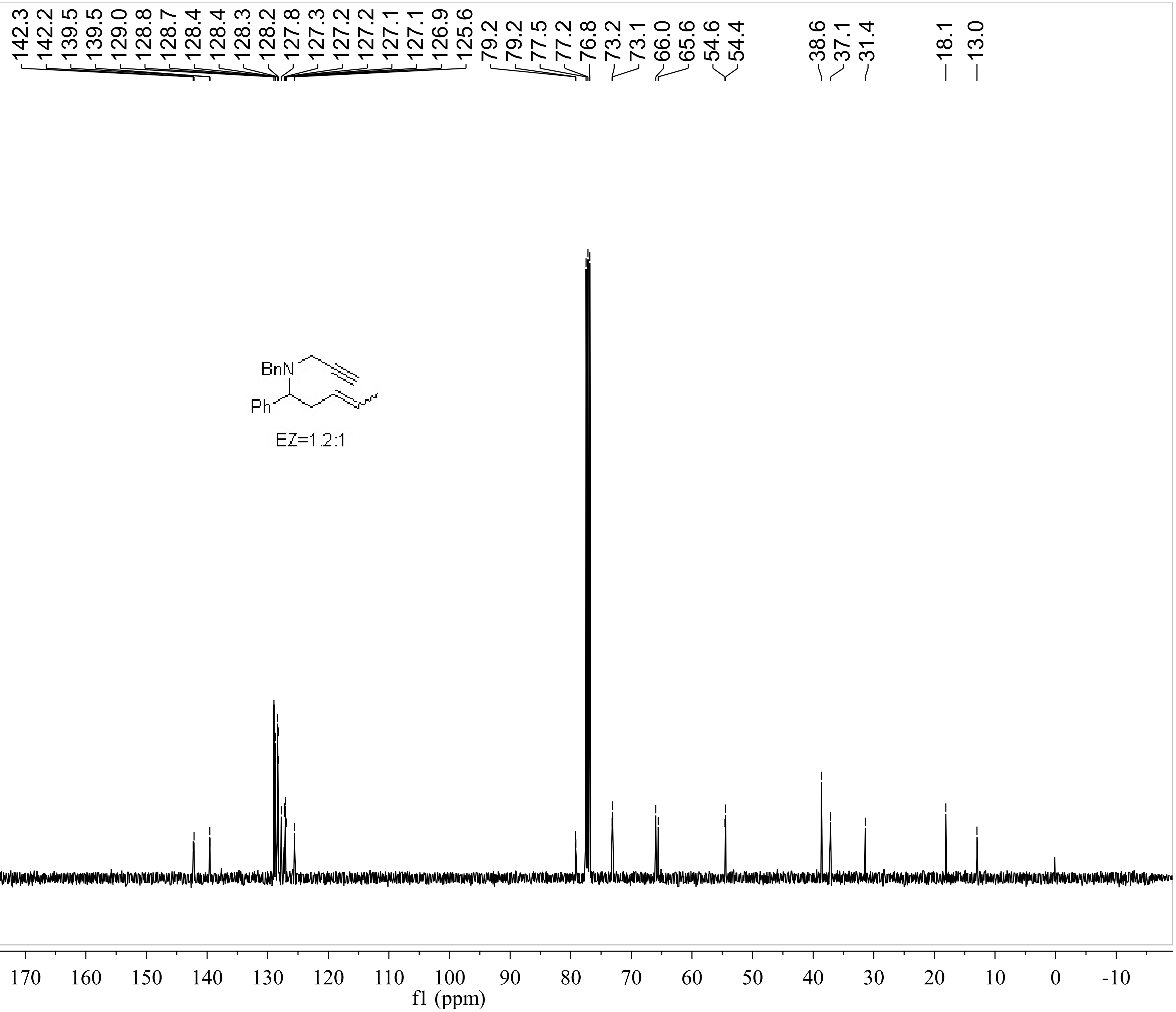

Parameter	Value
标题	xfy-190626-2-s1.1.1.1r
Comment	
Origin	Bruker BioSpin GmbH
Owner	nmr
Site	
Instrument	spect
Author	
Solvent	CDCl3
Temperature	296.1
Pulse Sequence	zg30
Experiment	1D
Number of Scans	8
Receiver Gain	70.5
Relaxation Delay	1.0000
Pulse Width	10.7100
Presaturation Frequency	
Acquisition Time	3.2768
Class	
Spectrometer Frequency	500.13
Spectral Width	10000.0
Lowest Frequency	-1923.4
Nucleus	1H
Acquired Size	32768
Spectral Size	65536

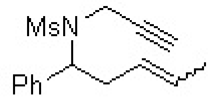

EZ mixture=7:1

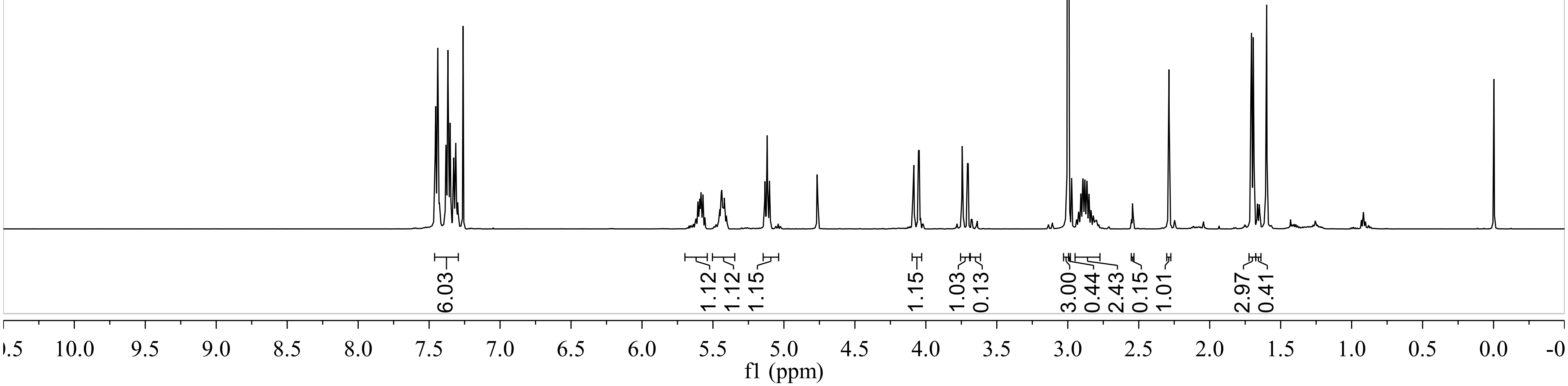

Parameter	Value
标题	xfy-190626-2-s1.2.fid
Comment	
Origin	Bruker BioSpin GmbH
Owner	nmr
Site	
Instrument	spect
Author	
Solvent	CDCl3
Temperature	296.1
Pulse Sequence	zgpg30
Experiment	1D
Number of Scans	20
Receiver Gain	193.1
Relaxation Delay	2.0000
Pulse Width	9.6000
Presaturation Frequency	
Acquisition Time	1.1010
Class	
Spectrometer Frequency	125.77
Spectral Width	29761.9
Lowest Frequency	-2290.8
Nucleus	13C
Acquired Size	32768
Spectral Size	65536

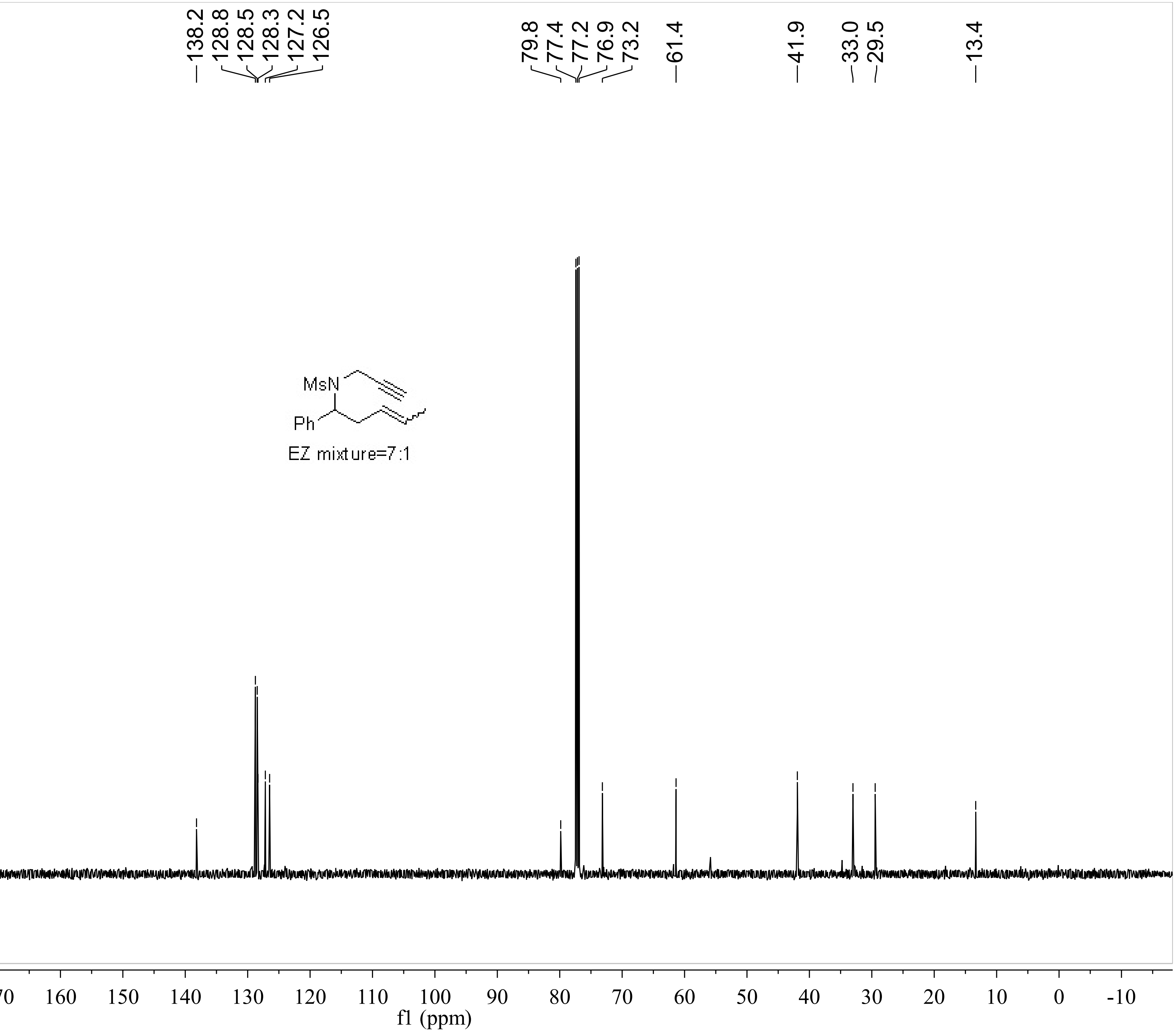

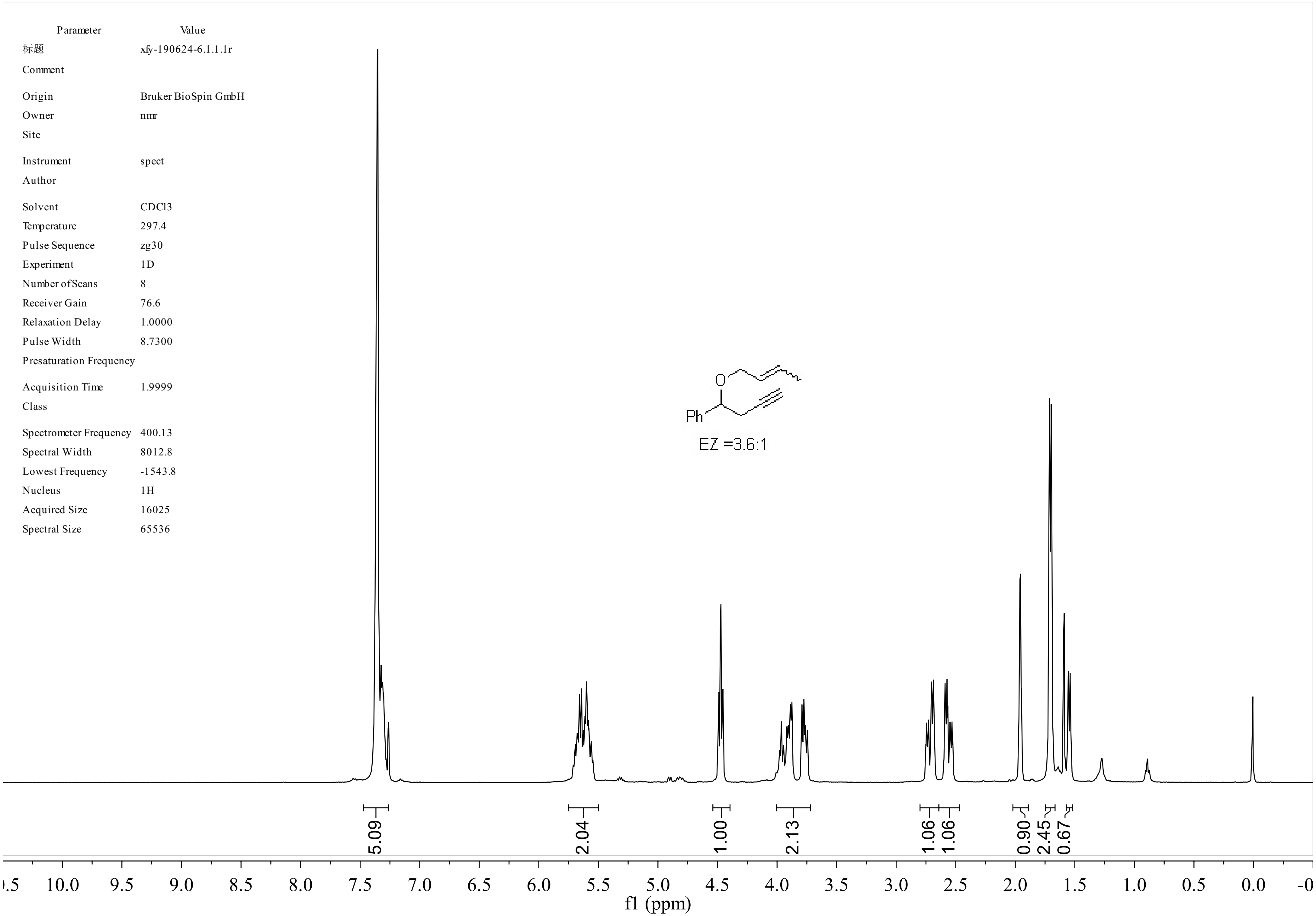
Parameter
Value
标题
xfy-190624-6.1.1.1r
Comment
Origin
Bruker BioSpin GmbH
Owner
nmr
Site
Instrument
spect
Author
Solvent
CDCl3
Temperature
297.4
Pulse Sequence
zg30
Experiment
1D
Number of Scans
8
Receiver Gain
76.6
Relaxation Delay
1.0000
Pulse Width
8.7300
Presaturation Frequency
Acquisition Time
1.9999
Class
Spectrometer Frequency
400.13
Spectral Width
8012.8
Lowest Frequency
-1543.8
Nucleus
1H
Acquired Size
16025
Spectral Size
65536
O
Ph
EZ =3.6:1
5.09
2.04
1.00
2.13
1.06
1.06
0.90
2.45
0.67
10.0
9.5
9.0
8.5
8.0
7.5
7.0
6.5
6.0
5.5
5.0
4.5
4.0
3.5
3.0
2.5
2.0
1.5
1.0
0.5
0.0
f1 (ppm)

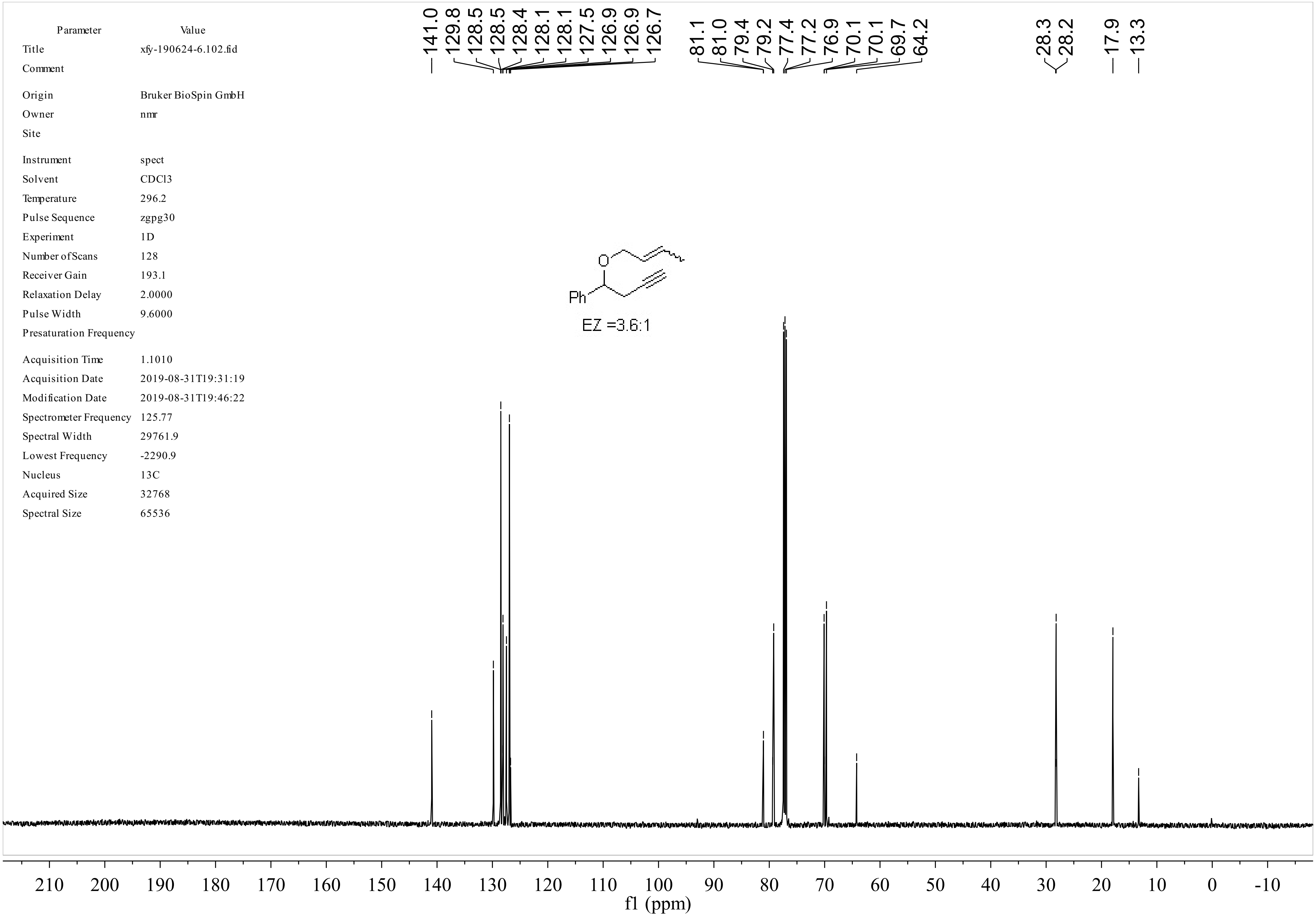
Parameter Value
Title xfy-190624-6.102.fid
Comment
Origin Bruker BioSpin GmbH
Owner nmr
Site
Instrument spect
Solvent CDCl3
Temperature 296.2
Pulse Sequence zgpg30
Experiment 1D
Number of Scans 128
Receiver Gain 193.1
Relaxation Delay 2.0000
Pulse Width 9.6000
Presaturation Frequency
Acquisition Time 1.1010
Acquisition Date 2019-08-31T19:31:19
Modification Date 2019-08-31T19:46:22
Spectrometer Frequency 125.77
Spectral Width 29761.9
Lowest Frequency -2290.9
Nucleus 13C
Acquired Size 32768
Spectral Size 65536
141.0 129.8 128.5 128.5 128.4 128.1 128.1 127.5 126.9 126.9 126.7
81.1 81.0 79.4 79.2 77.4 77.2 76.9 70.1 70.1 69.7 64.2
28.3 28.2 17.9 13.3
O
Ph
EZ =3.6:1
f1 (ppm)
210 200 190 180 170 160 150 140 130 120 110 100 90 80 70 60 50 40 30 20 10 0 -10

Parameter	Value
标题	xfy-190704-1.1.1.1r
Comment	
Origin	Bruker BioSpin GmbH
Owner	nmr
Site	
Instrument	spect
Author	
Solvent	CDCl3
Temperature	296.1
Pulse Sequence	zg30
Experiment	1D
Number of Scans	16
Receiver Gain	54.3
Relaxation Delay	1.0000
Pulse Width	10.7100
Presaturation Frequency	
Acquisition Time	3.2768
Class	
Spectrometer Frequency	500.13
Spectral Width	10000.0
Lowest Frequency	-1923.9
Nucleus	1H
Acquired Size	32768
Spectral Size	65536

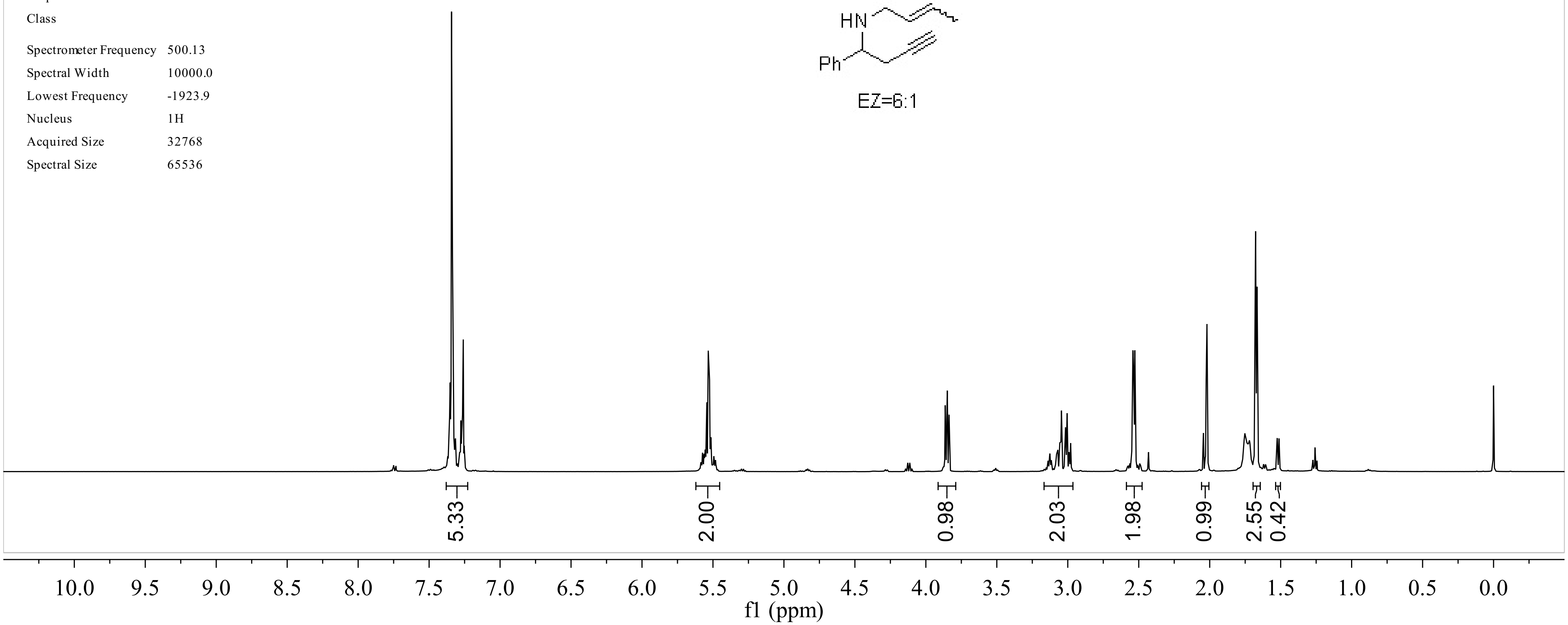

Parameter	Value
标题	xfy-190704-1.2.1.1r
Comment	
Origin	Bruker BioSpin GmbH
Owner	nmr
Site	
Instrument	spect
Author	
Solvent	CDCl3
Temperature	296.2
Pulse Sequence	zgpg30
Experiment	1D
Number of Scans	37
Receiver Gain	193.1
Relaxation Delay	2.0000
Pulse Width	9.6000
Presaturation Frequency	
Acquisition Time	1.1010
Class	
Spectrometer Frequency	125.76
Spectral Width	29761.9
Lowest Frequency	-2292.6
Nucleus	13C
Acquired Size	32768
Spectral Size	32768

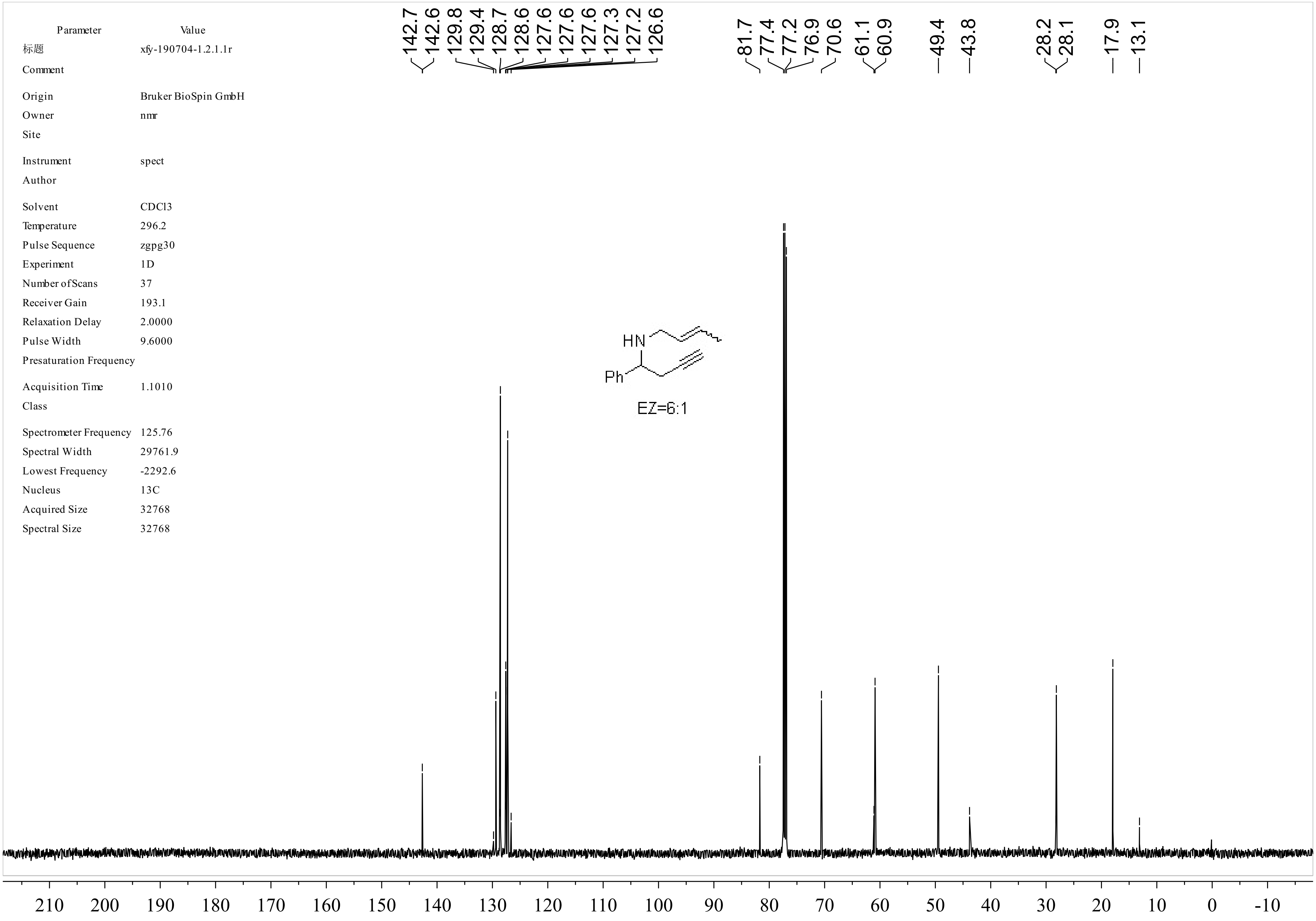

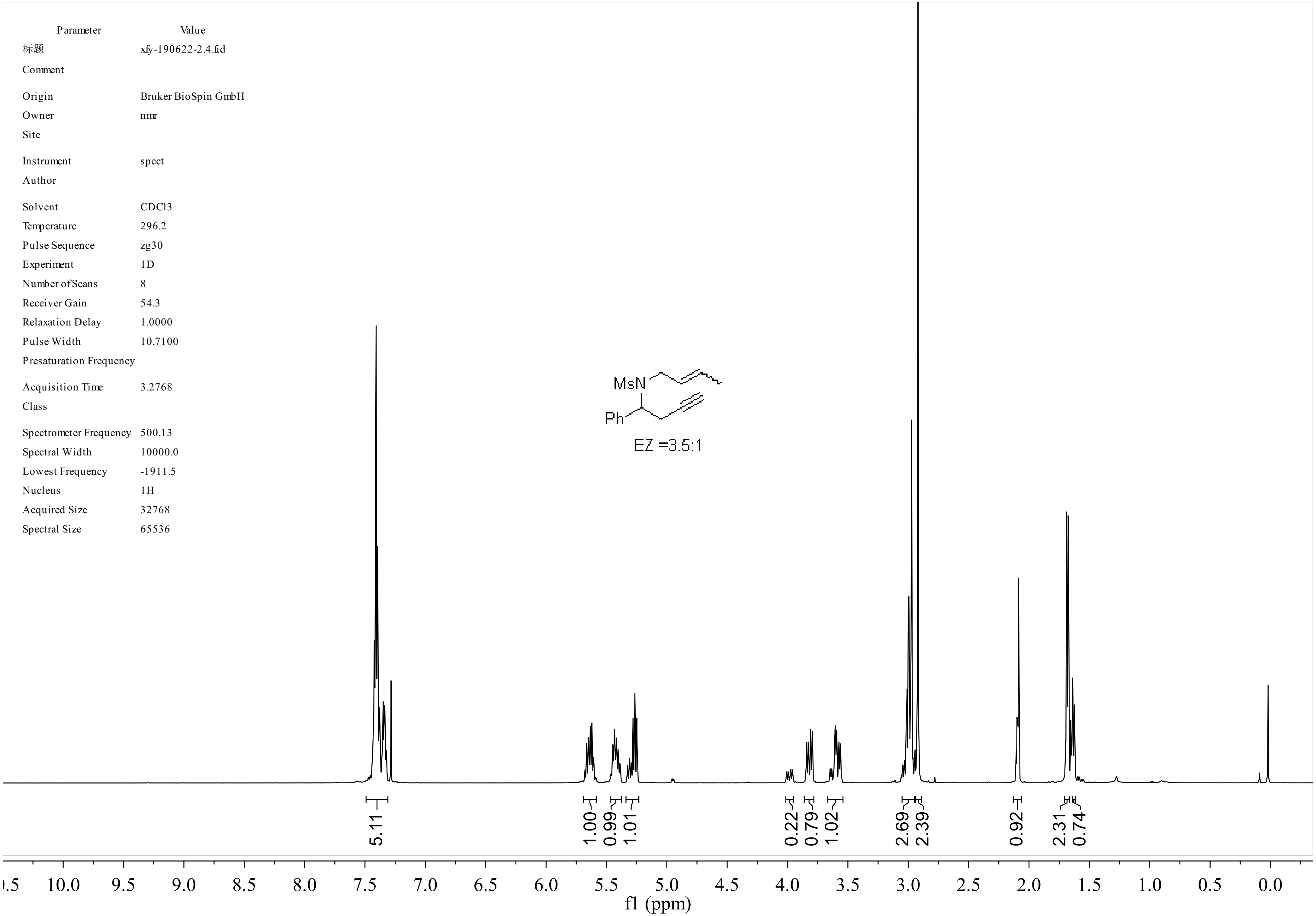
Parameter Value
标题 xfy-190622-2.4.fid
Comment
Origin Bruker BioSpin GmbH
Owner nmr
Site
Instrument spect
Author
Solvent CDCl3
Temperature 296.2
Pulse Sequence zg30
Experiment 1D
Number of Scans 8
Receiver Gain 54.3
Relaxation Delay 1.0000
Pulse Width 10.7100
Presaturation Frequency
Acquisition Time 3.2768
Class
Spectrometer Frequency 500.13
Spectral Width 10000.0
Lowest Frequency -1911.5
Nucleus 1H
Acquired Size 32768
Spectral Size 65536
MsN
Ph
EZ =3.5:1
5.11
1.00
0.99
1.01
0.22
0.79
1.02
2.69
2.39
0.92
2.31
0.74
10.0
9.5
9.0
8.5
8.0
7.5
7.0
6.5
6.0
5.5
5.0
4.5
4.0
3.5
3.0
2.5
2.0
1.5
1.0
0.5
0.0
f1 (ppm)

Parameter	Value
标题	xfy-190622-2.5.fid
Comment	
Origin	Bruker BioSpin GmbH
Owner	nmr
Site	
Instrument	spect
Author	
Solvent	CDCl3
Temperature	296.2
Pulse Sequence	zgpg30
Experiment	1D
Number of Scans	64
Receiver Gain	193.1
Relaxation Delay	2.0000
Pulse Width	9.6000
Presaturation Frequency	
Acquisition Time	1.1010
Class	
Spectrometer Frequency	125.77
Spectral Width	29761.9
Lowest Frequency	-2294.1
Nucleus	13C
Acquired Size	32768
Spectral Size	65536

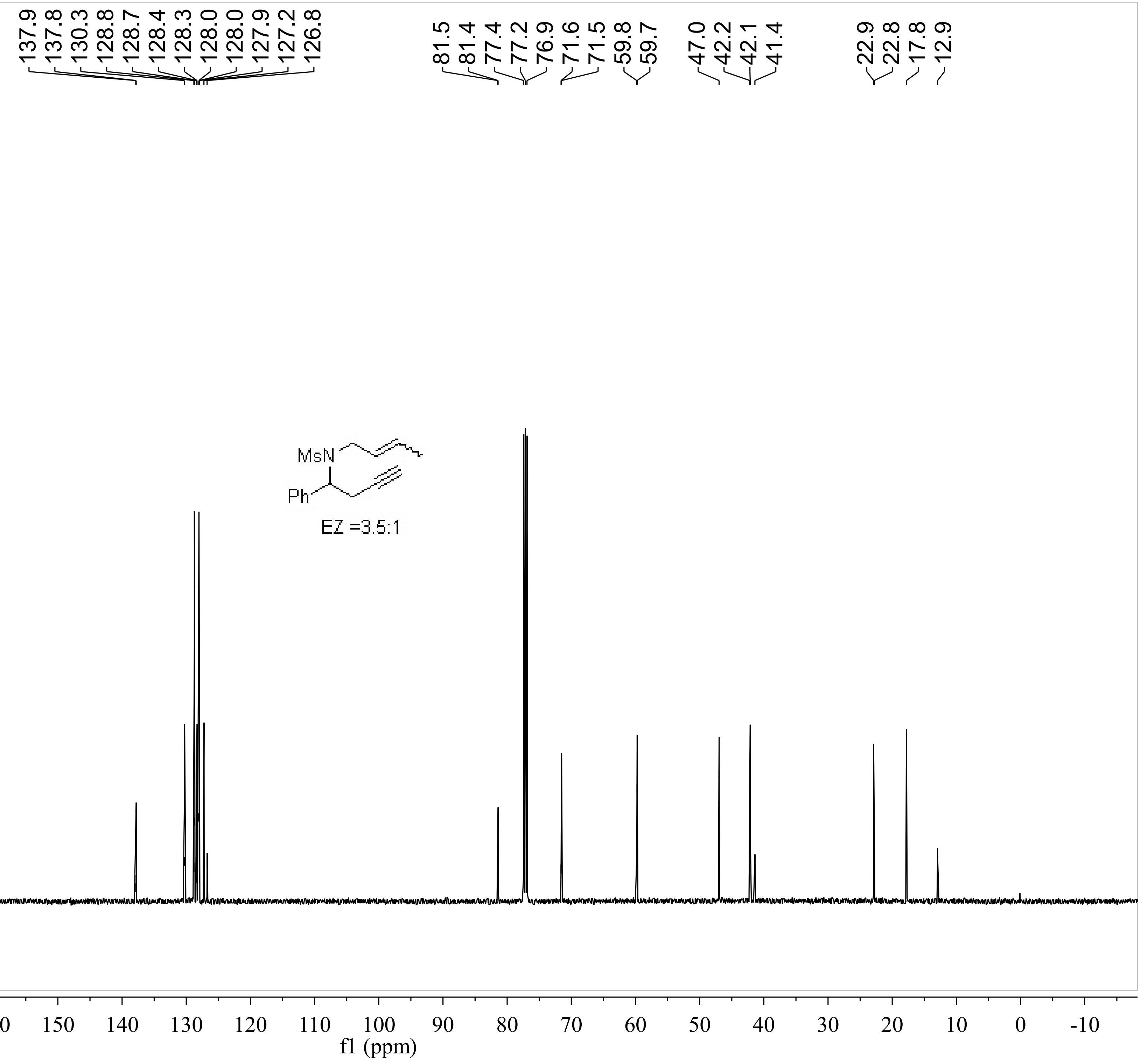

Parameter	Value
Title	xfy-0818-5.1.fid
Comment	
Origin	Bruker BioSpin GmbH
Owner	nmr
Site	
Instrument	spect
Solvent	CDCl3
Temperature	296.1
Pulse Sequence	zg30
Experiment	1D
Number of Scans	6
Receiver Gain	48.5
Relaxation Delay	1.0000
Pulse Width	10.7100
Presaturation Frequency	
Acquisition Time	3.2768
Class	
Spectrometer Frequency	500.13
Spectral Width	10000.0
Lowest Frequency	-1924.5
Nucleus	1H
Acquired Size	32768
Spectral Size	65536

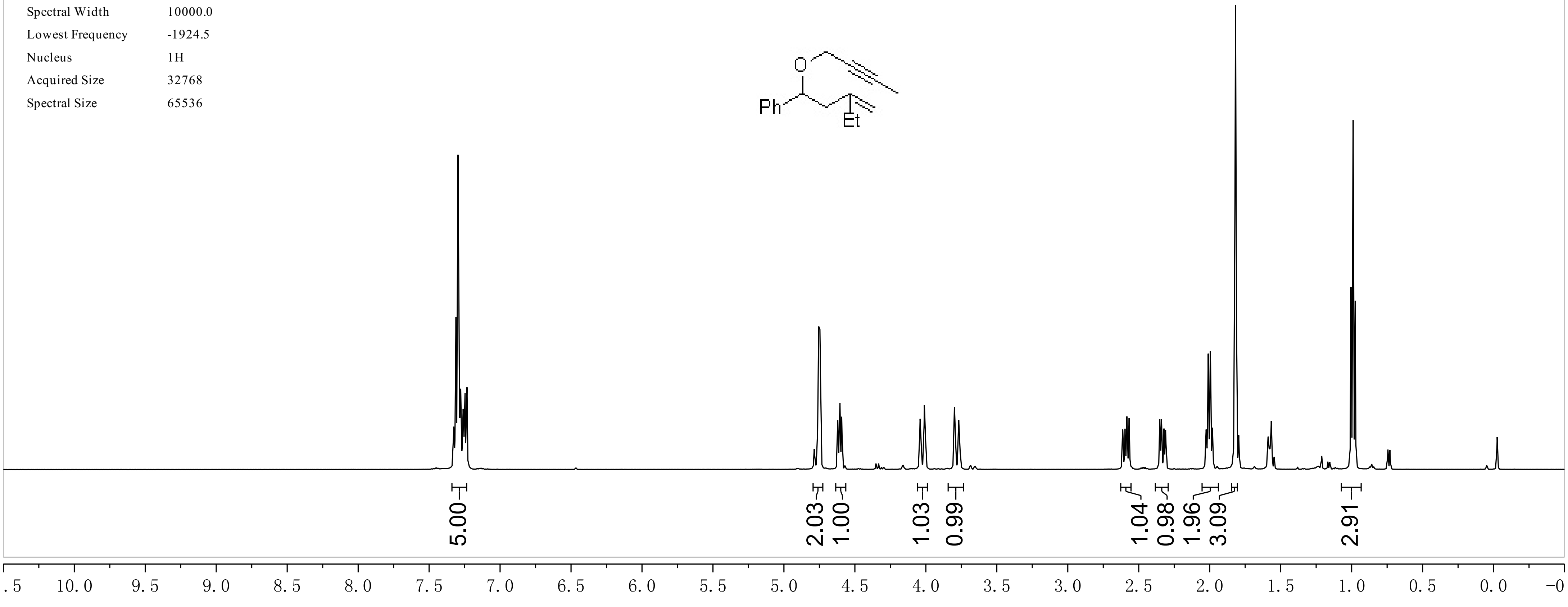

Parameter	Value
Title	xfy-0818-5.2.fid
Comment	
Origin	Bruker BioSpin GmbH
Owner	nmr
Site	
Instrument	spect
Solvent	CDCl3
Temperature	296.1
Pulse Sequence	zgpg30
Experiment	1D
Number of Scans	10
Receiver Gain	193.1
Relaxation Delay	2.0000
Pulse Width	9.6000
Presaturation Frequency	
Acquisition Time	1.1010
Class	
Spectrometer Frequency	125.77
Spectral Width	29761.9
Lowest Frequency	-2291.1
Nucleus	13C
Acquired Size	32768
Spectral Size	65536

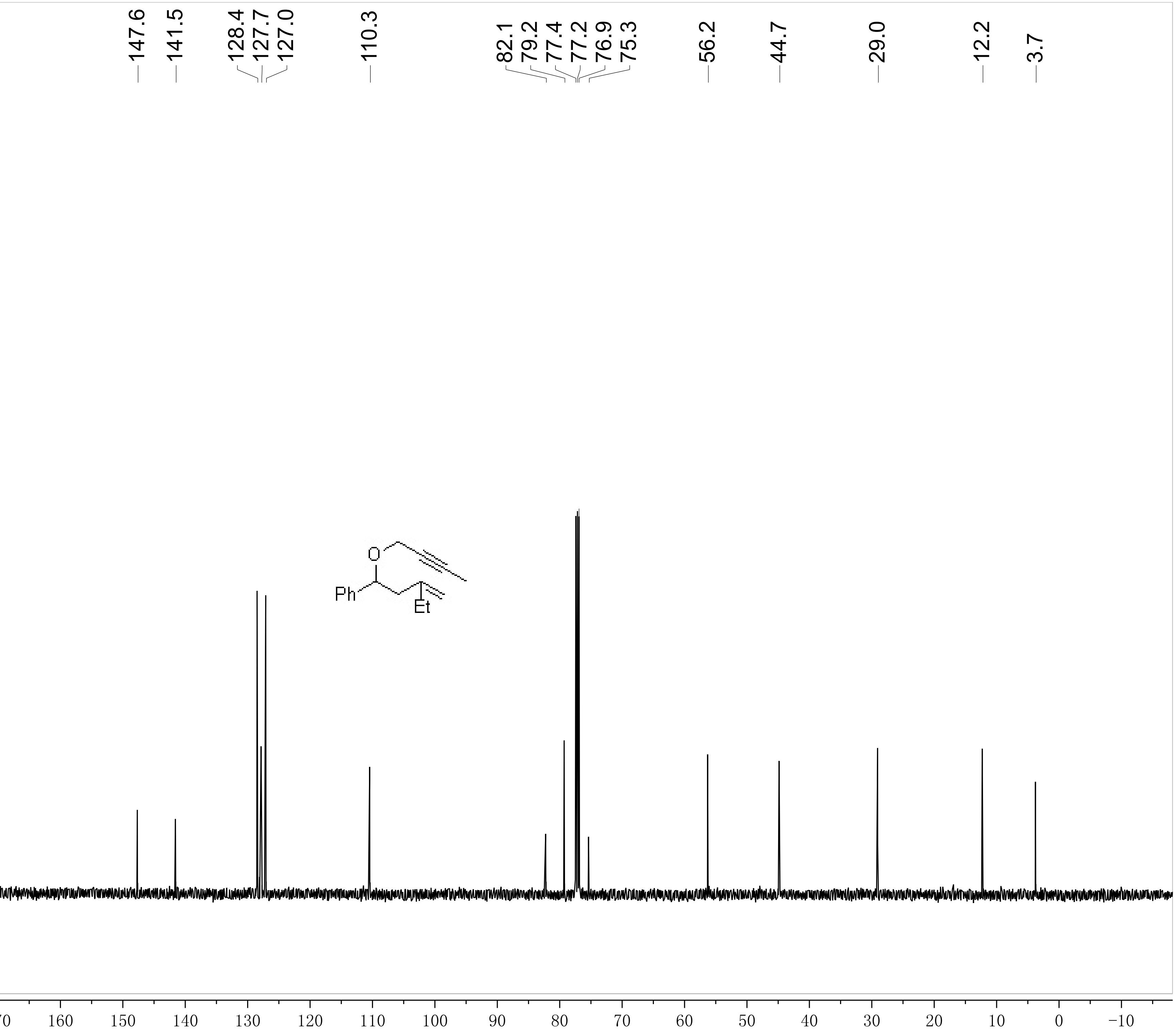

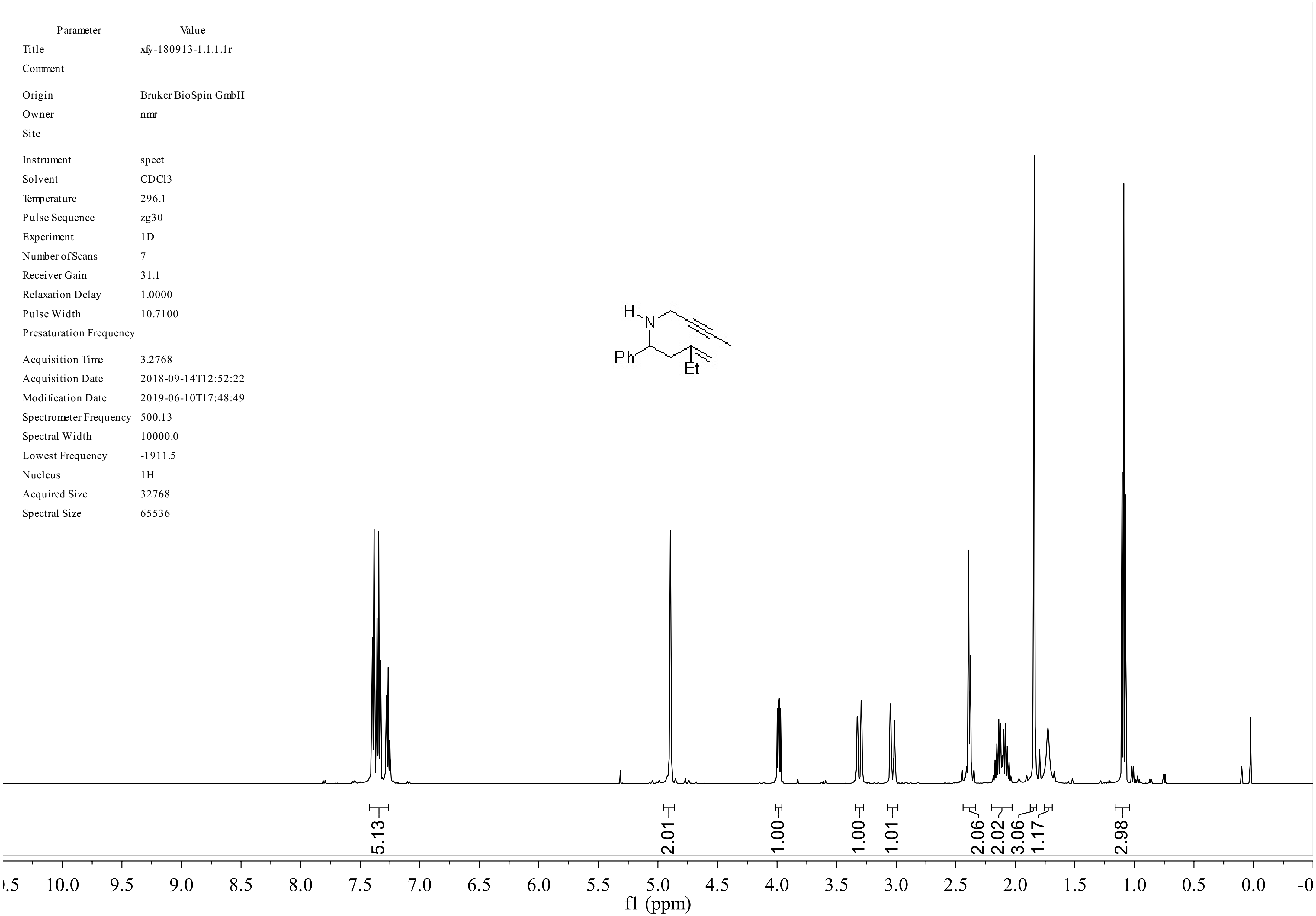
Parameter Value
Title xfy-180913-1.1.1.1r
Comment
Origin Bruker BioSpin GmbH
Owner nmr
Site
Instrument spect
Solvent CDCl3
Temperature 296.1
Pulse Sequence zg30
Experiment 1D
Number of Scans 7
Receiver Gain 31.1
Relaxation Delay 1.0000
Pulse Width 10.7100
Presaturation Frequency
Acquisition Time 3.2768
Acquisition Date 2018-09-14T12:52:22
Modification Date 2019-06-10T17:48:49
Spectrometer Frequency 500.13
Spectral Width 10000.0
Lowest Frequency -1911.5
Nucleus 1H
Acquired Size 32768
Spectral Size 65536
H
N
Ph
Et
5.13
2.01
1.00
1.00
1.01
2.06
2.02
3.06
1.17
2.98
10.0 9.5 9.0 8.5 8.0 7.5 7.0 6.5 6.0 5.5 5.0 4.5 4.0 3.5 3.0 2.5 2.0 1.5 1.0 0.5 0.0
f1 (ppm)

Parameter	Value
Title	xfy-180913-1.2.fid
Comment	
Origin	Bruker BioSpin GmbH
Owner	nmr
Site	
Instrument	spect
Solvent	CDCl3
Temperature	296.1
Pulse Sequence	zgpg30
Experiment	1D
Number of Scans	32
Receiver Gain	193.1
Relaxation Delay	2.0000
Pulse Width	9.6000
Presaturation Frequency	
Acquisition Time	1.1010
Acquisition Date	2018-09-14T12:54:07
Modification Date	2019-06-10T17:48:49
Spectrometer Frequency	125.77
Spectral Width	29761.9
Lowest Frequency	-2292.3
Nucleus	13C
Acquired Size	32768
Spectral Size	65536

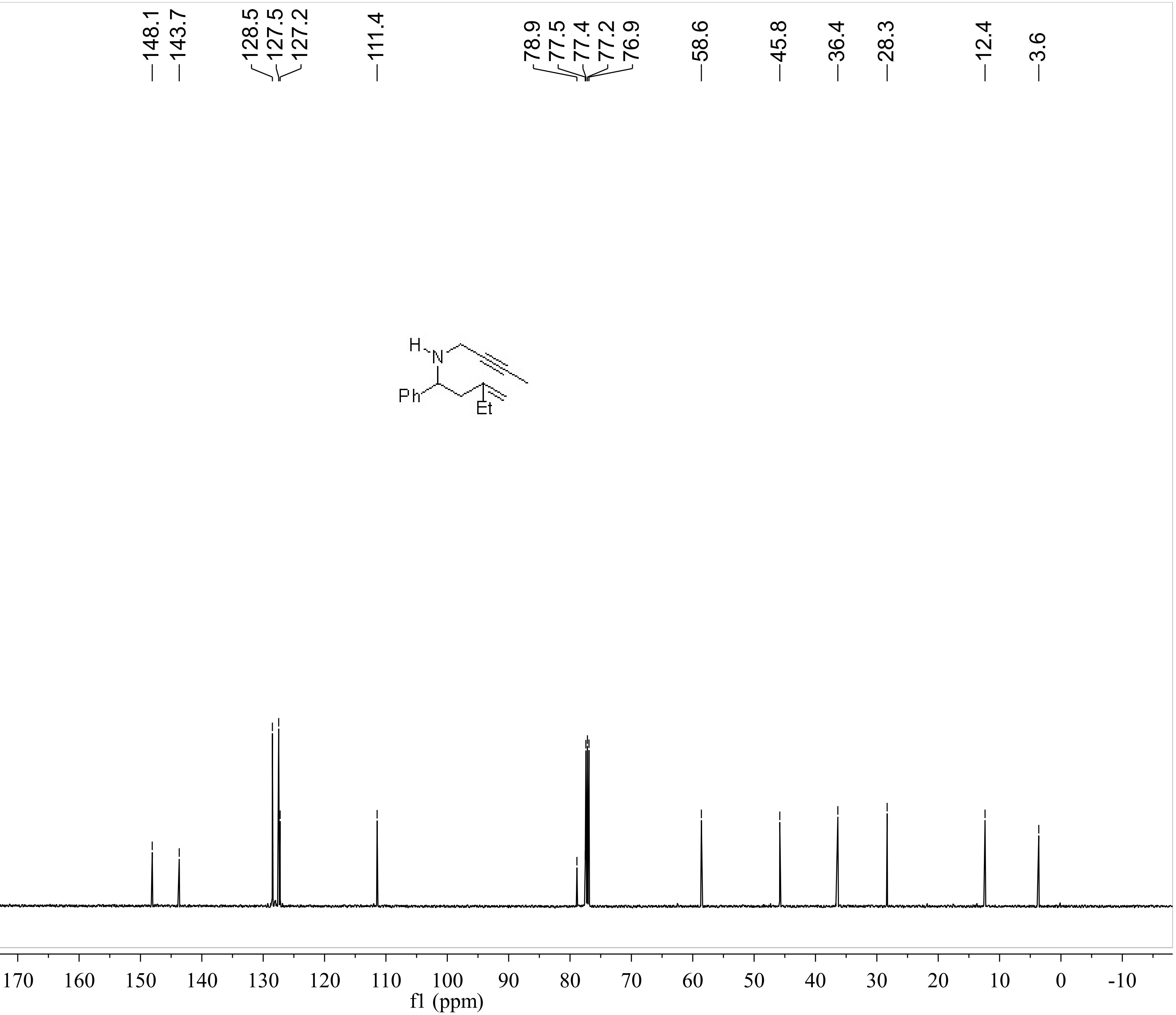

Parameter	Value
Title	xfy-180827-4.11.fid
Comment	
Origin	Bruker BioSpin GmbH
Owner	nmr
Site	
Instrument	spect
Solvent	CDCl3
Temperature	296.2
Pulse Sequence	zg30
Experiment	1D
Number of Scans	9
Receiver Gain	54.3
Relaxation Delay	1.0000
Pulse Width	10.7100
Presaturation Frequency	
Acquisition Time	3.2768
Acquisition Date	2018-08-27T14:47:00
Modification Date	2018-08-28T14:08:08
Spectrometer Frequency	500.13
Spectral Width	10000.0
Lowest Frequency	-1922.0
Nucleus	1H
Acquired Size	32768
Spectral Size	65536

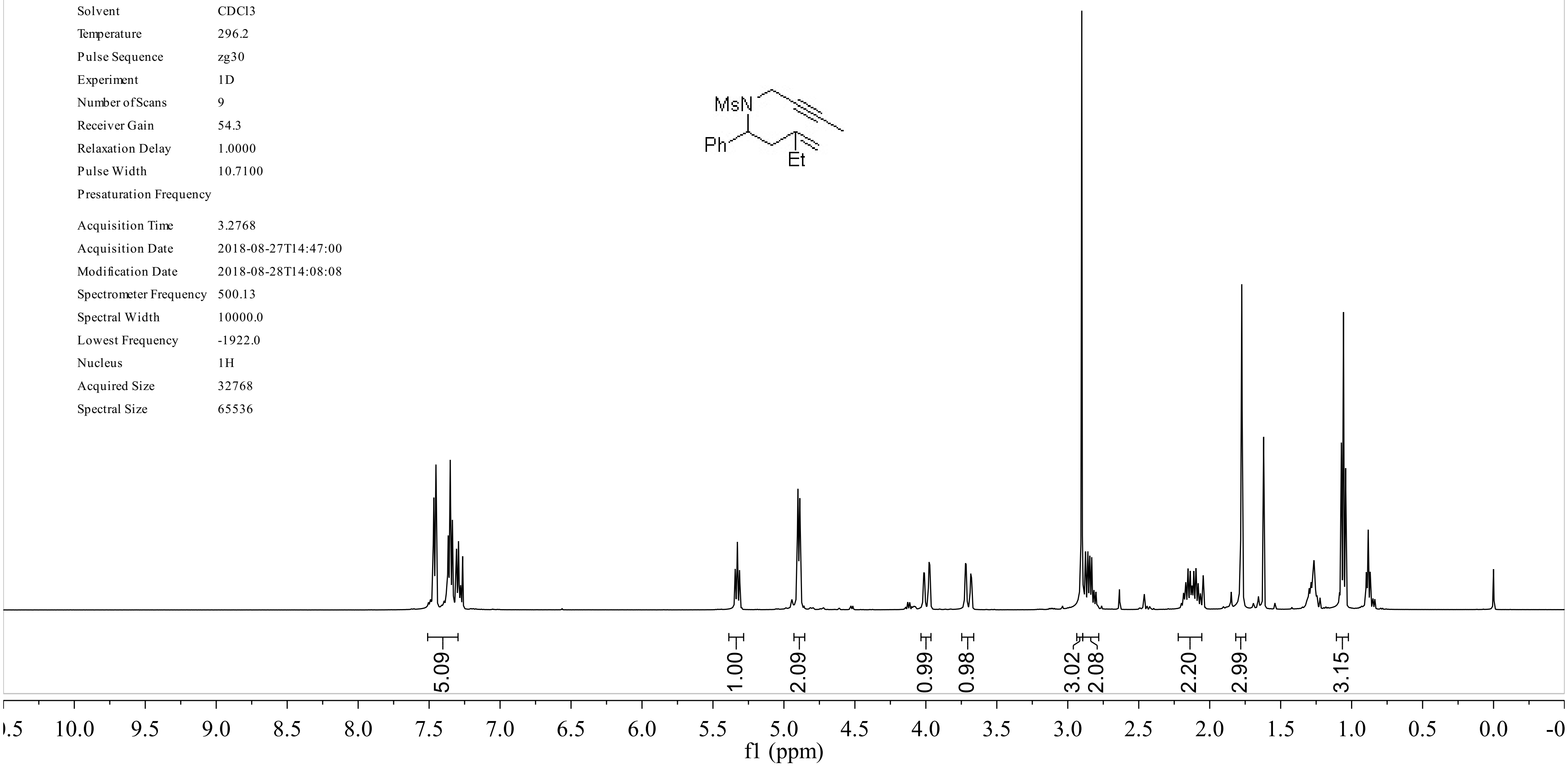

Parameter	Value
Title	xfy-180827-4.12.fid
Comment	
Origin	Bruker BioSpin GmbH
Owner	nmr
Site	
Instrument	spect
Solvent	CDCl3
Temperature	296.2
Pulse Sequence	zgpg30
Experiment	1D
Number of Scans	10
Receiver Gain	193.1
Relaxation Delay	2.0000
Pulse Width	9.6000
Presaturation Frequency	
Acquisition Time	1.1010
Acquisition Date	2018-08-27T14:49:00
Modification Date	2018-08-28T14:08:08
Spectrometer Frequency	125.77
Spectral Width	29761.9
Lowest Frequency	-2291.8
Nucleus	13C
Acquired Size	32768
Spectral Size	65536

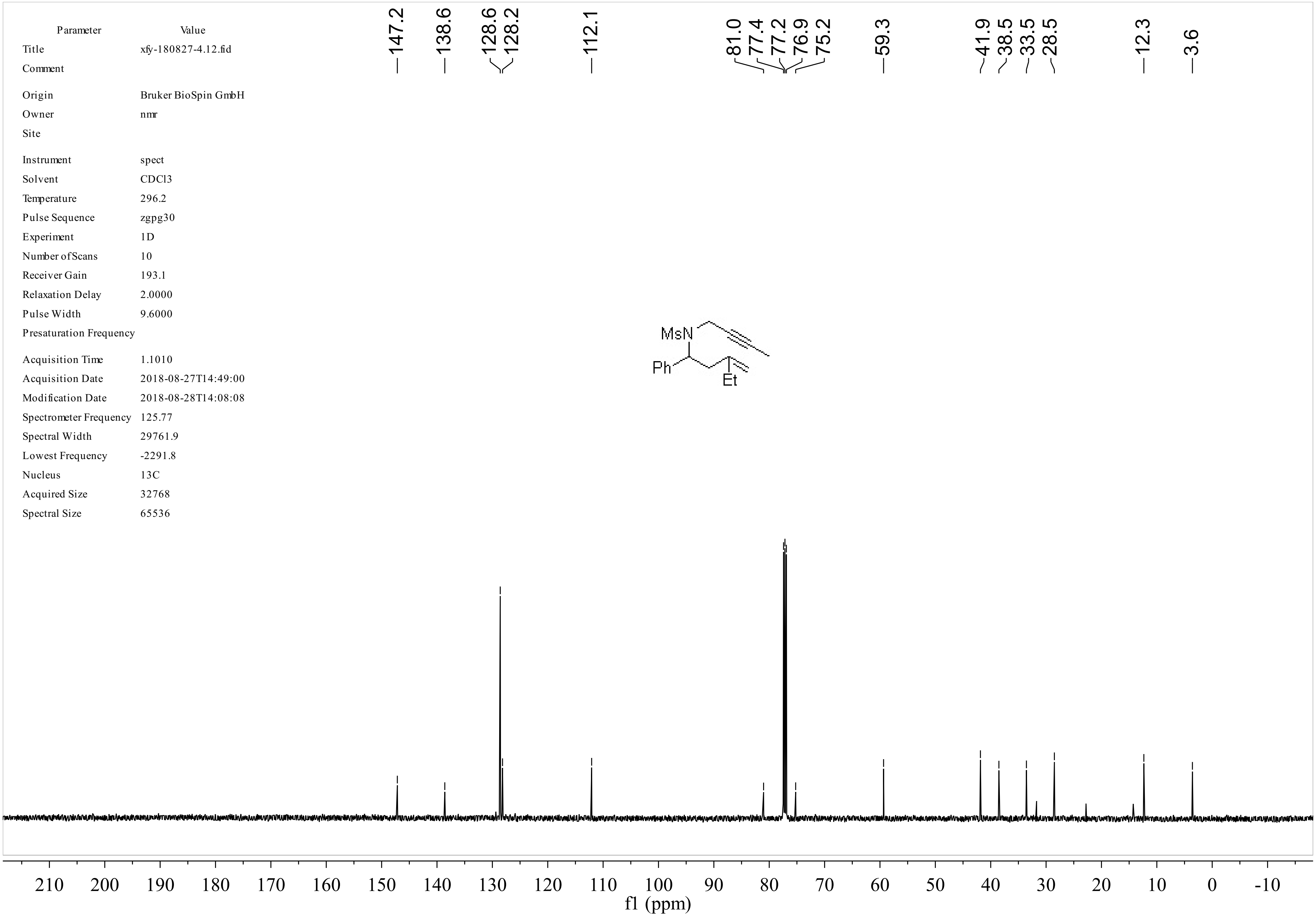

Parameter	Value
Title	xfy-190403-1.1.1.1r
Comment	
Origin	Bruker BioSpin GmbH
Owner	nmr
Site	
Instrument	spect
Solvent	CDCl3
Temperature	296.2
Pulse Sequence	zg30
Experiment	1D
Number of Scans	16
Receiver Gain	87.5
Relaxation Delay	1.0000
Pulse Width	10.7100
Presaturation Frequency	
Acquisition Time	3.2768
Class	
Spectrometer Frequency	500.13
Spectral Width	10000.0
Lowest Frequency	-1911.5
Nucleus	1H
Acquired Size	32768
Spectral Size	65536

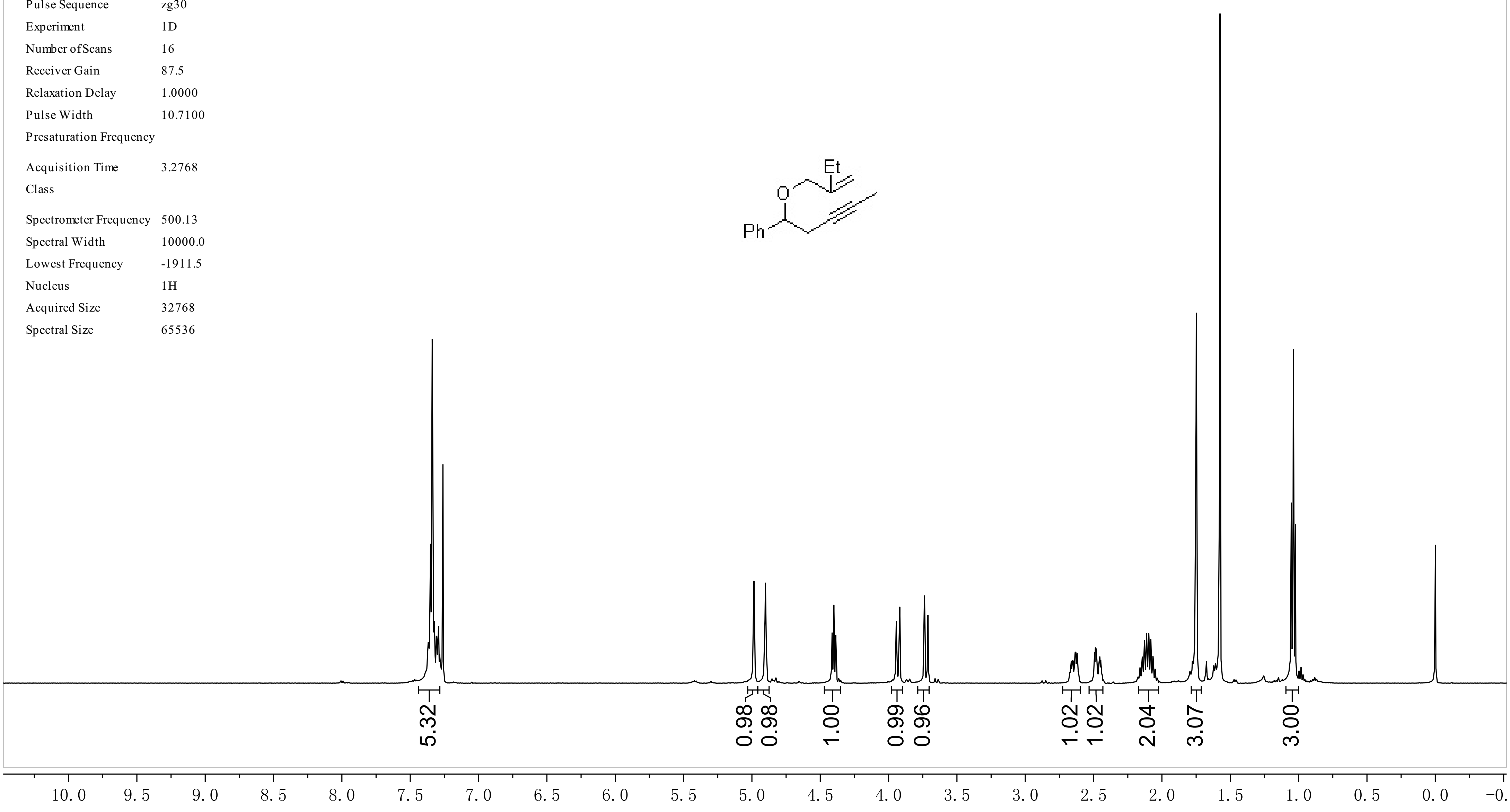

Parameter	Value
Title	xfy-190922-1.1.1.1r
Comment	
Origin	Bruker BioSpin GmbH
Owner	nmr
Site	
Instrument	spect
Solvent	CD2Cl2
Temperature	296.2
Pulse Sequence	zgpg30
Experiment	1D
Number of Scans	36
Receiver Gain	193.1
Relaxation Delay	2.0000
Pulse Width	9.6000
Presaturation Frequency	
Acquisition Time	1.1010
Acquisition Date	2019-09-22T19:53:37
Modification Date	2019-09-22T21:01:39
Spectrometer Frequency	125.76
Spectral Width	29761.9
Lowest Frequency	-2296.2
Nucleus	13C
Acquired Size	32768
Spectral Size	32768

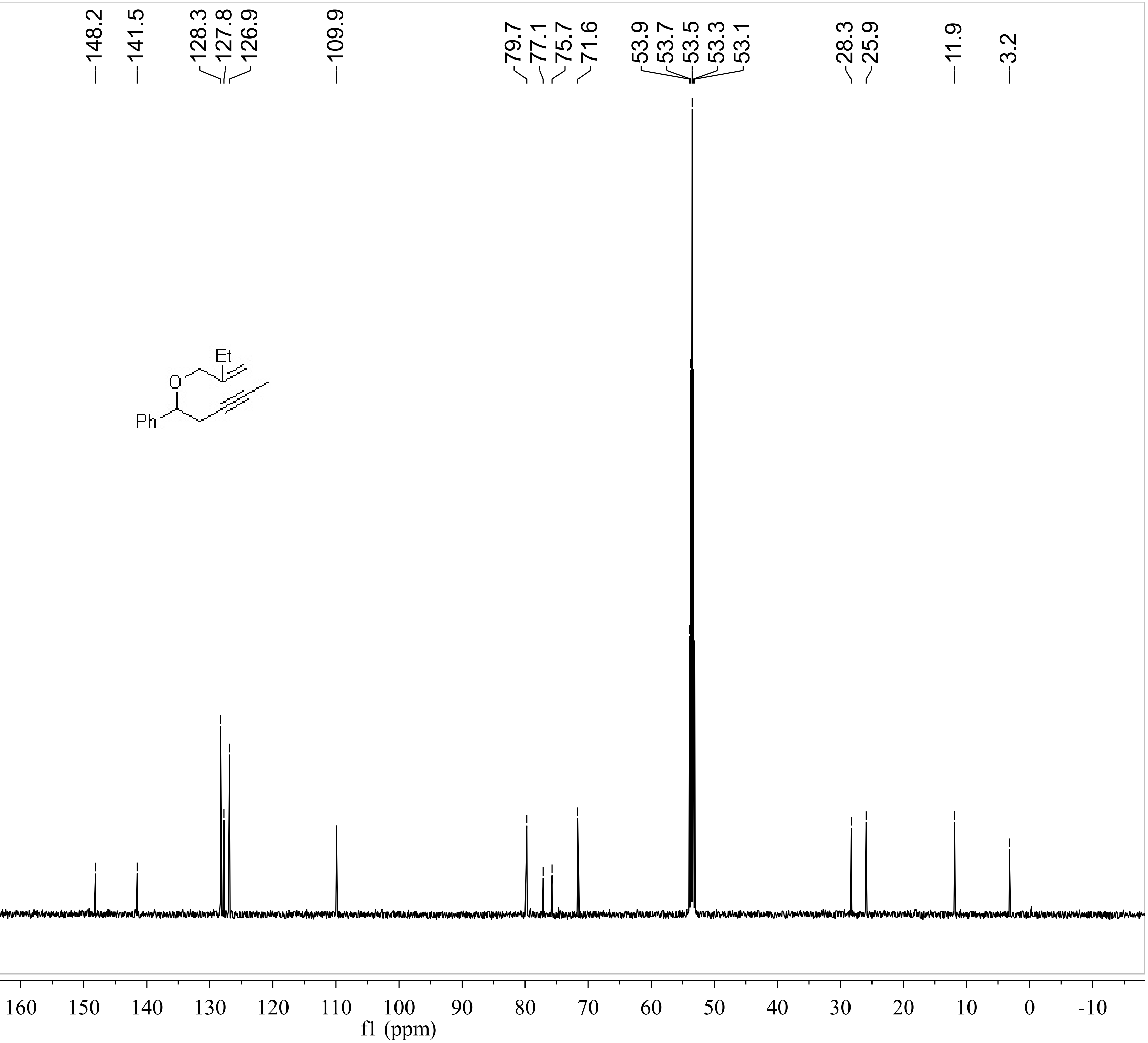

Parameter	Value
Title	xfy-190405-2.1.1.1r
Comment	
Origin	Bruker BioSpin GmbH
Owner	nmr
Site	
Instrument	spect
Solvent	CDCl3
Temperature	296.1
Pulse Sequence	zg30
Experiment	1D
Number of Scans	4
Receiver Gain	77.6
Relaxation Delay	1.0000
Pulse Width	10.7100
Presaturation Frequency	
Acquisition Time	3.2768
Class	
Spectrometer Frequency	500.13
Spectral Width	10000.0
Lowest Frequency	-1911.5
Nucleus	1H
Acquired Size	32768
Spectral Size	65536

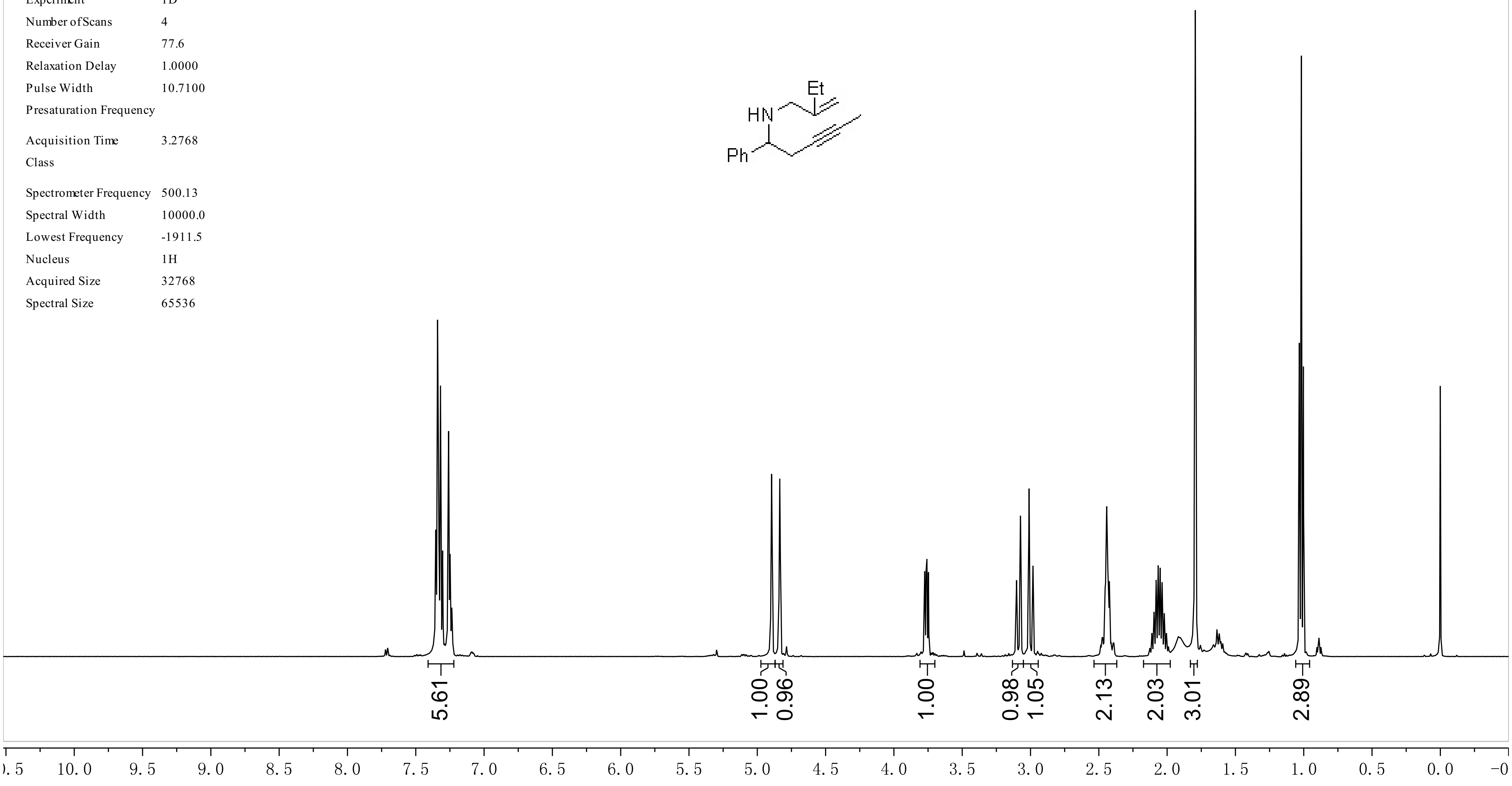

Parameter	Value
Title	xfy-190405-2.2.1.1r
Comment	
Origin	Bruker BioSpin GmbH
Owner	nmr
Site	
Instrument	spect
Solvent	CDCl3
Temperature	296.2
Pulse Sequence	zgpg30
Experiment	1D
Number of Scans	64
Receiver Gain	193.1
Relaxation Delay	2.0000
Pulse Width	9.6000
Presaturation Frequency	
Acquisition Time	1.1010
Class	
Spectrometer Frequency	125.76
Spectral Width	29761.9
Lowest Frequency	-2290.4
Nucleus	13C
Acquired Size	32768
Spectral Size	32768

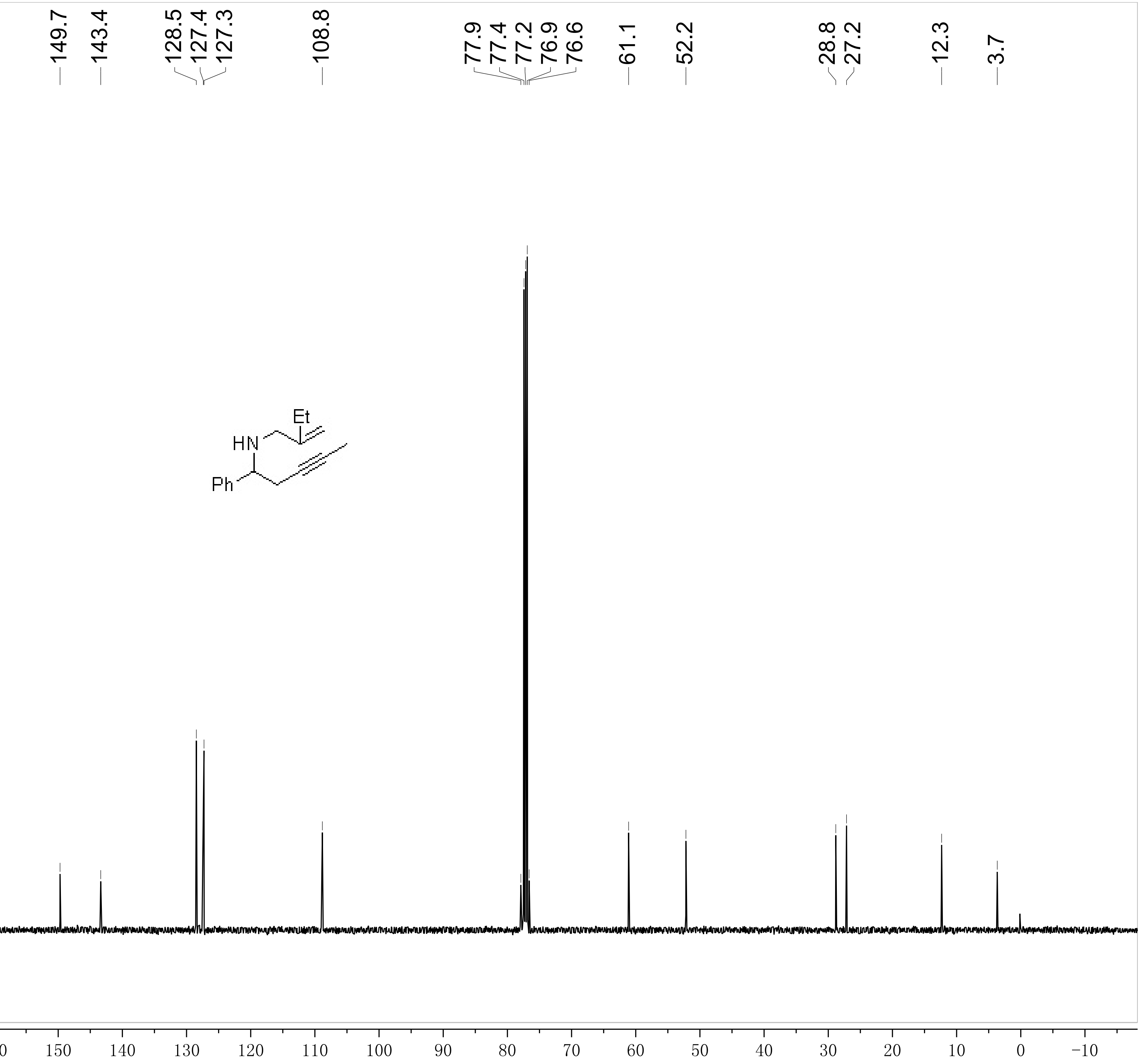

Parameter	Value
Title	xfy-190402-2.1.fid
Comment	
Origin	Bruker BioSpin GmbH
Owner	nmr
Site	
Instrument	spect
Solvent	CDCl3
Temperature	296.7
Pulse Sequence	zg30
Experiment	1D
Number of Scans	5
Receiver Gain	142.1
Relaxation Delay	1.0000
Pulse Width	8.7300
Presaturation Frequency	
Acquisition Time	1.9999
Class	
Spectrometer Frequency	400.13
Spectral Width	8012.8
Lowest Frequency	-1535.4
Nucleus	1H
Acquired Size	16025
Spectral Size	65536

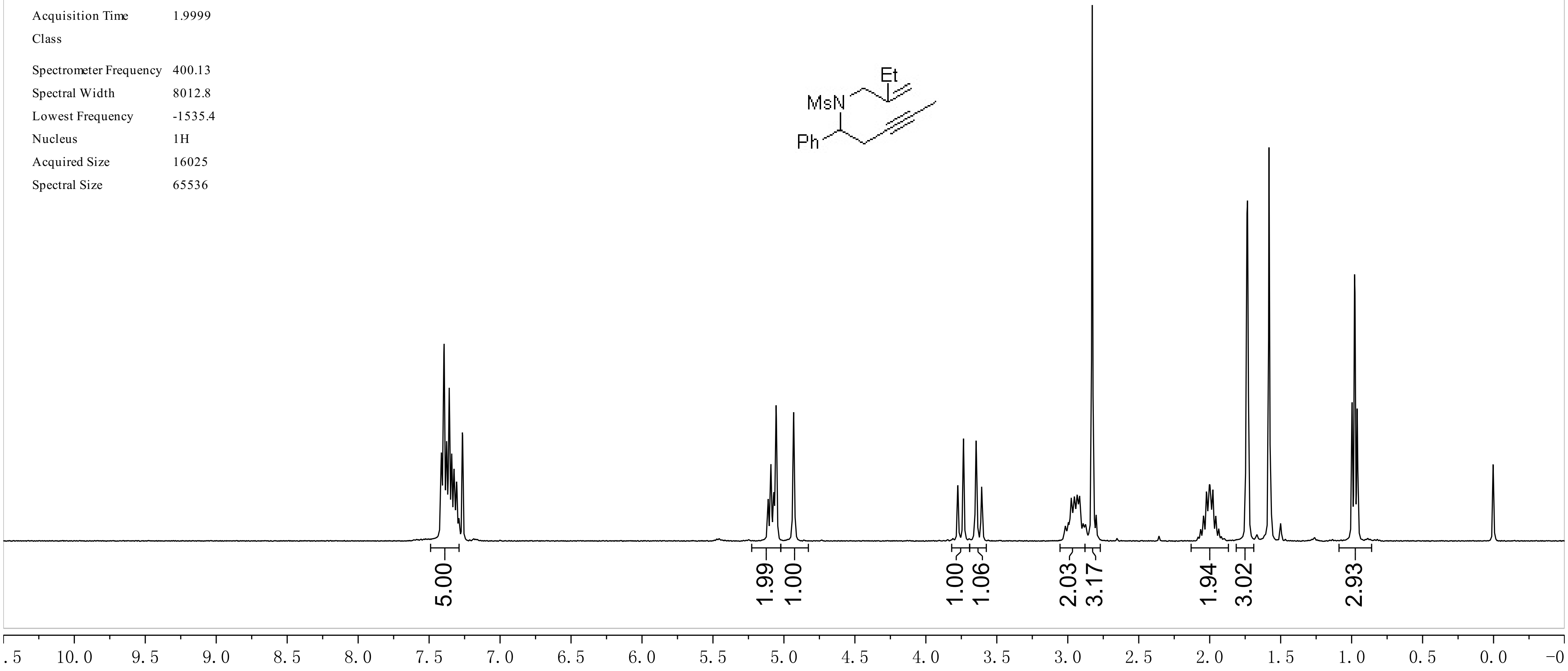

Parameter	Value
Title	xfy-190402-2.2.fid
Comment	
Origin	Bruker BioSpin GmbH
Owner	nmr
Site	
Instrument	spect
Solvent	CDCl3
Temperature	296.2
Pulse Sequence	zgpg30
Experiment	1D
Number of Scans	46
Receiver Gain	193.1
Relaxation Delay	2.0000
Pulse Width	9.6000
Presaturation Frequency	
Acquisition Time	1.1010
Class	
Spectrometer Frequency	125.77
Spectral Width	29761.9
Lowest Frequency	-2305.8
Nucleus	13C
Acquired Size	32768
Spectral Size	65536

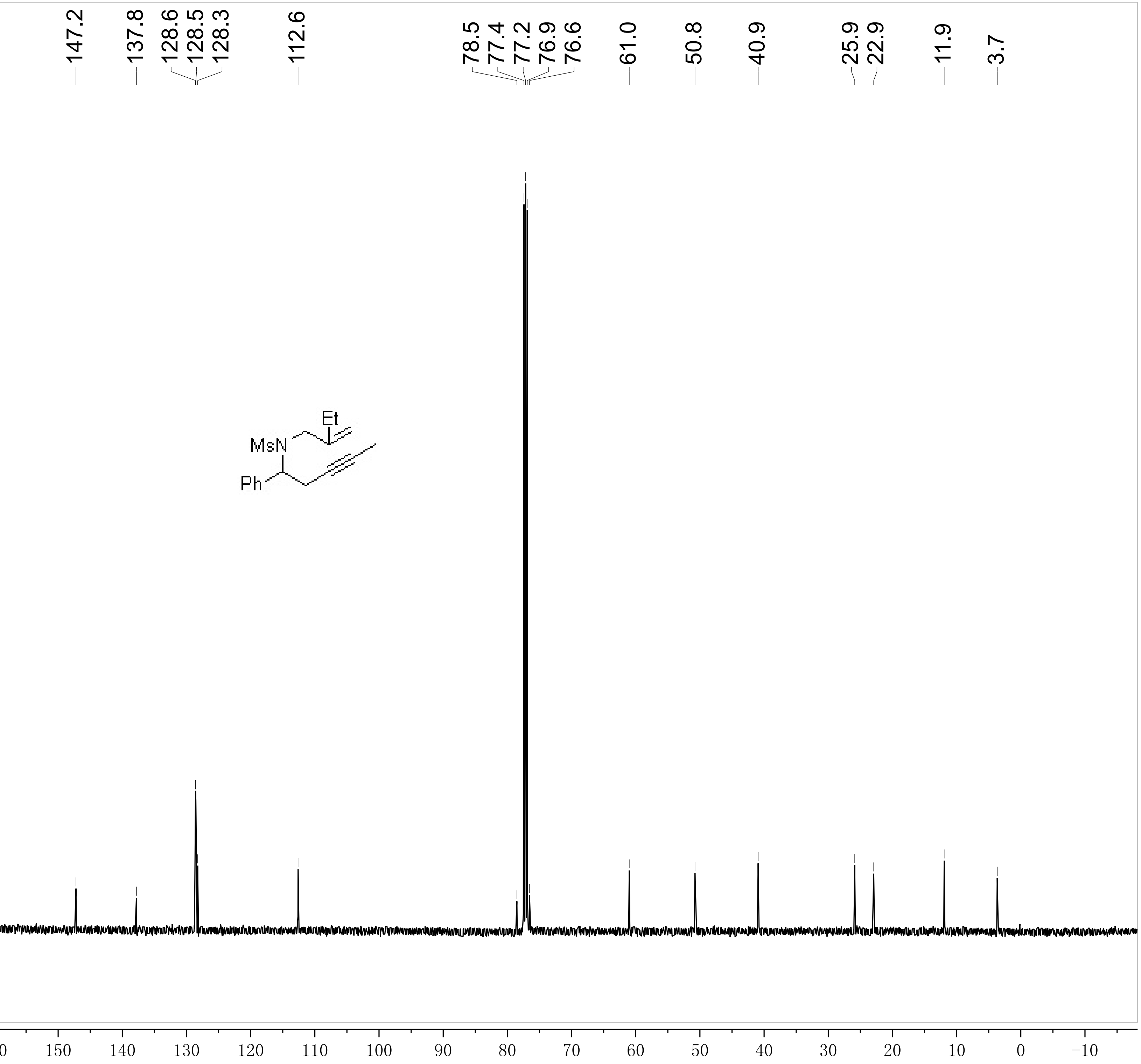

Parameter	Value
Title	xfy-190406-5.1.fid
Comment	
Origin	Bruker BioSpin GmbH
Owner	nmr
Site	
Instrument	spect
Solvent	CDCl3
Temperature	296.2
Pulse Sequence	zg30
Experiment	1D
Number of Scans	8
Receiver Gain	77.6
Relaxation Delay	1.0000
Pulse Width	10.7100
Presaturation Frequency	
Acquisition Time	3.2768
Class	
Spectrometer Frequency	500.13
Spectral Width	10000.0
Lowest Frequency	-1923.6
Nucleus	1H
Acquired Size	32768
Spectral Size	65536

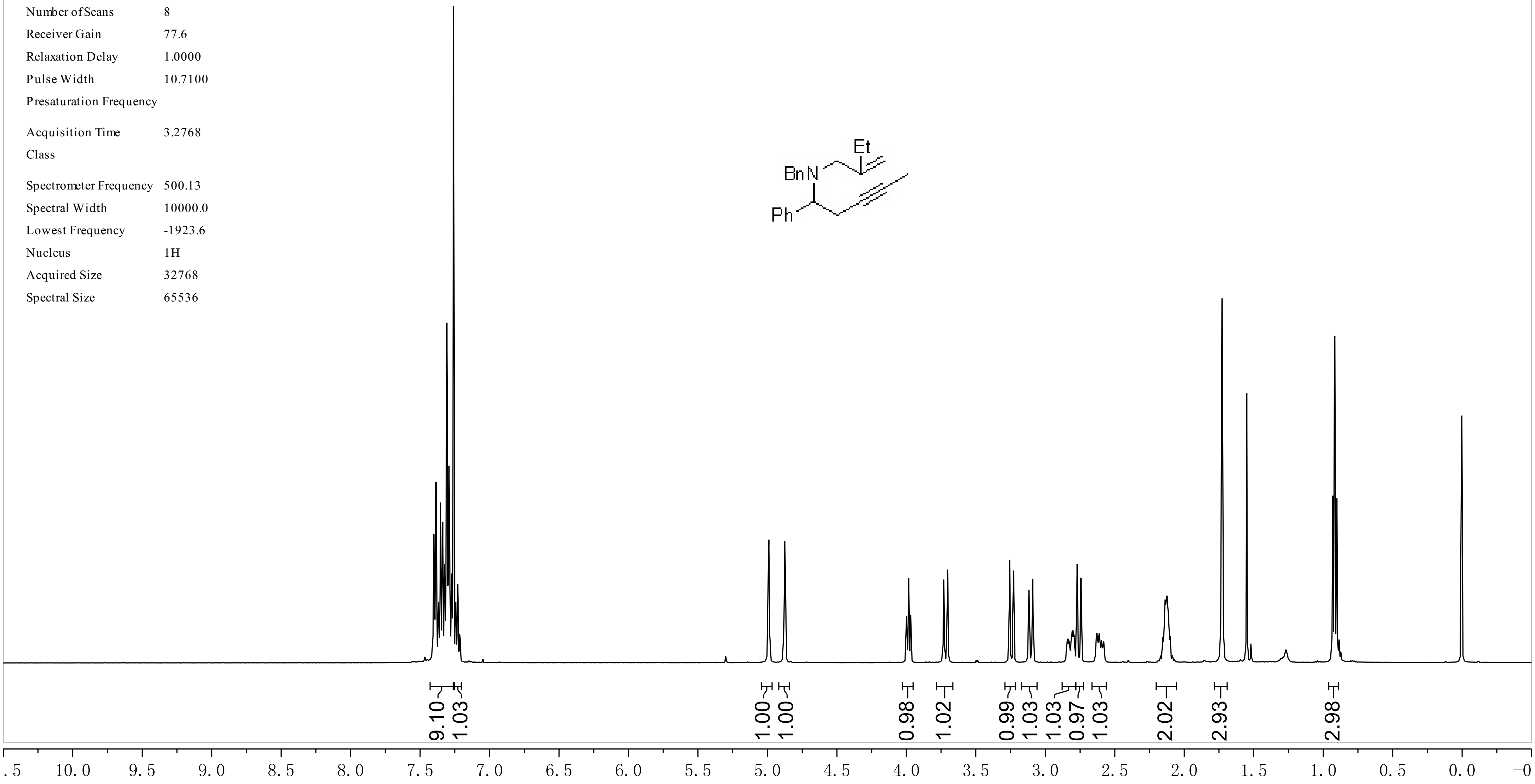

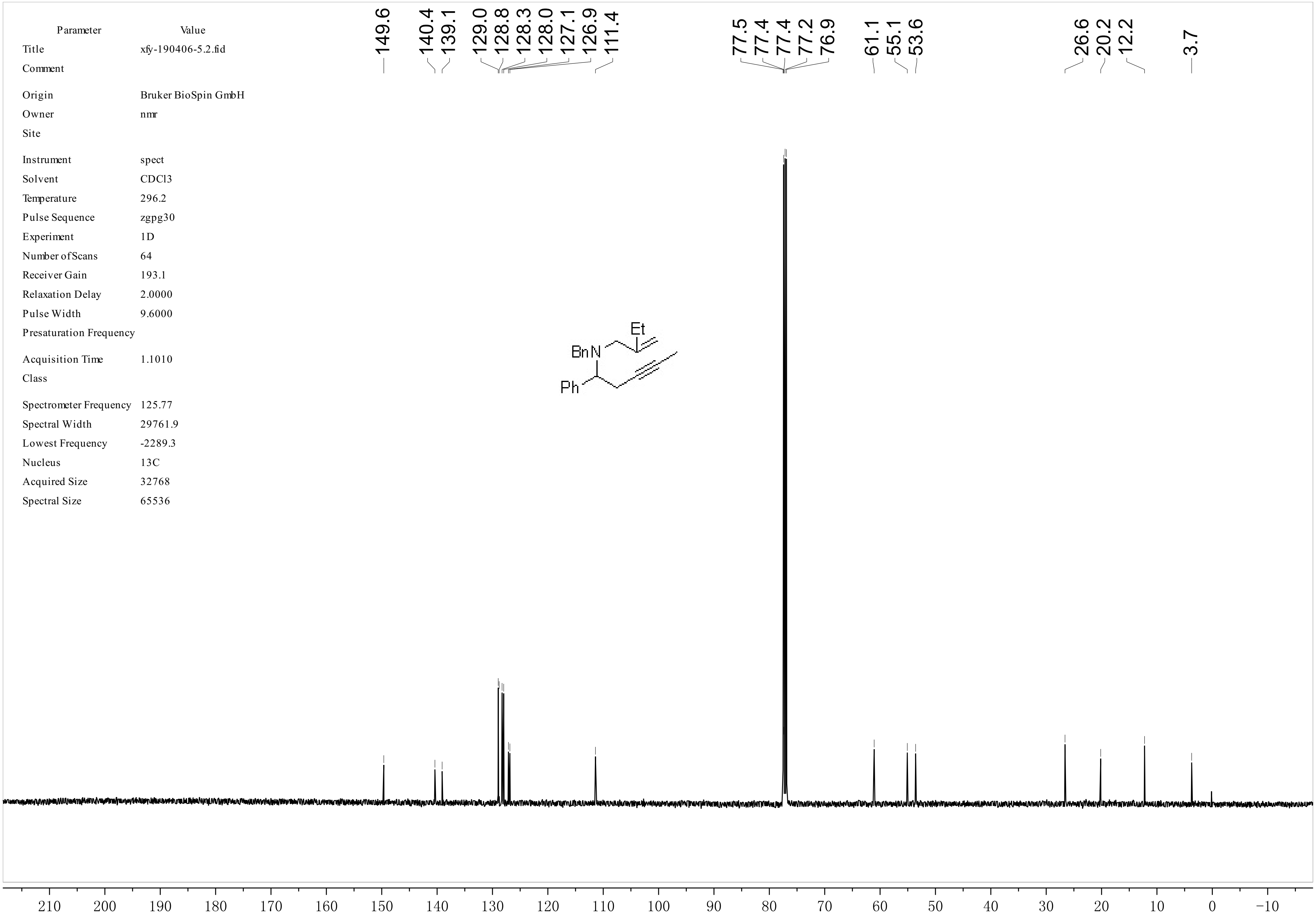

Parameter Value
Title xfy-190406-5.2.fid
Comment
Origin Bruker BioSpin GmbH
Owner nmr
Site
Instrument spect
Solvent CDCl3
Temperature 296.2
Pulse Sequence zgpg30
Experiment 1D
Number of Scans 64
Receiver Gain 193.1
Relaxation Delay 2.0000
Pulse Width 9.6000
Presaturation Frequency
Acquisition Time 1.1010
Class
Spectrometer Frequency 125.77
Spectral Width 29761.9
Lowest Frequency -2289.3
Nucleus 13C
Acquired Size 32768
Spectral Size 65536
149.6
140.4
139.1
129.0
128.8
128.3
128.0
127.1
126.9
111.4
77.5
77.4
77.4
77.2
76.9
61.1
55.1
53.6
26.6
20.2
12.2
3.7
Et
BnN
Ph
210 200 190 180 170 160 150 140 130 120 110 100 90 80 70 60 50 40 30 20 10 0 −10

Parameter	Value
Title	gvv228-2f/1
Origin	Bruker BioSpin GmbH
Owner	nmr
Site	
Spectrometer	spect
Author	
Solvent	CDCl3
Temperature	296.0
Pulse Sequence	zg30
Experiment	1D
Number of Scans	8
Receiver Gain	111
Relaxation Delay	1.0000
Pulse Width	8.7300
Acquisition Time	1.9999
Spectrometer Frequency	400.13
Spectral Width	8012.8
Lowest Frequency	-1544.6
Nucleus	1H
Acquired Size	16025
Spectral Size	65536

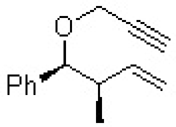

relative configuration

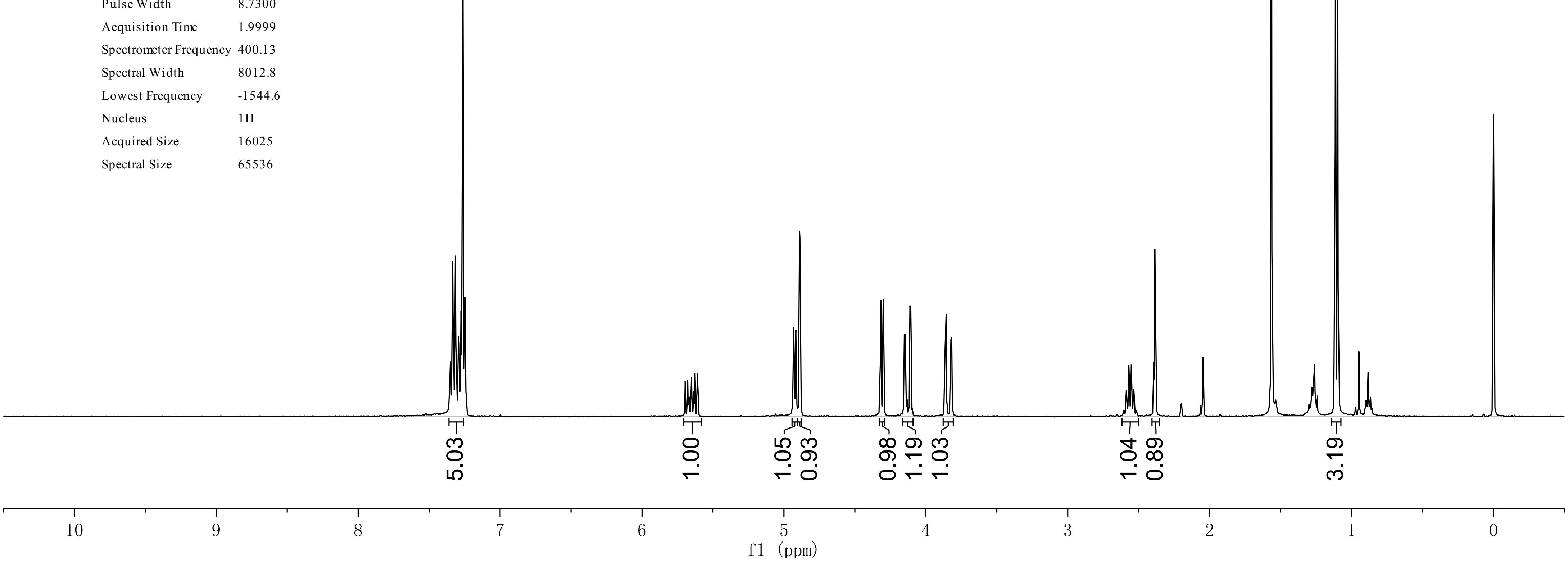

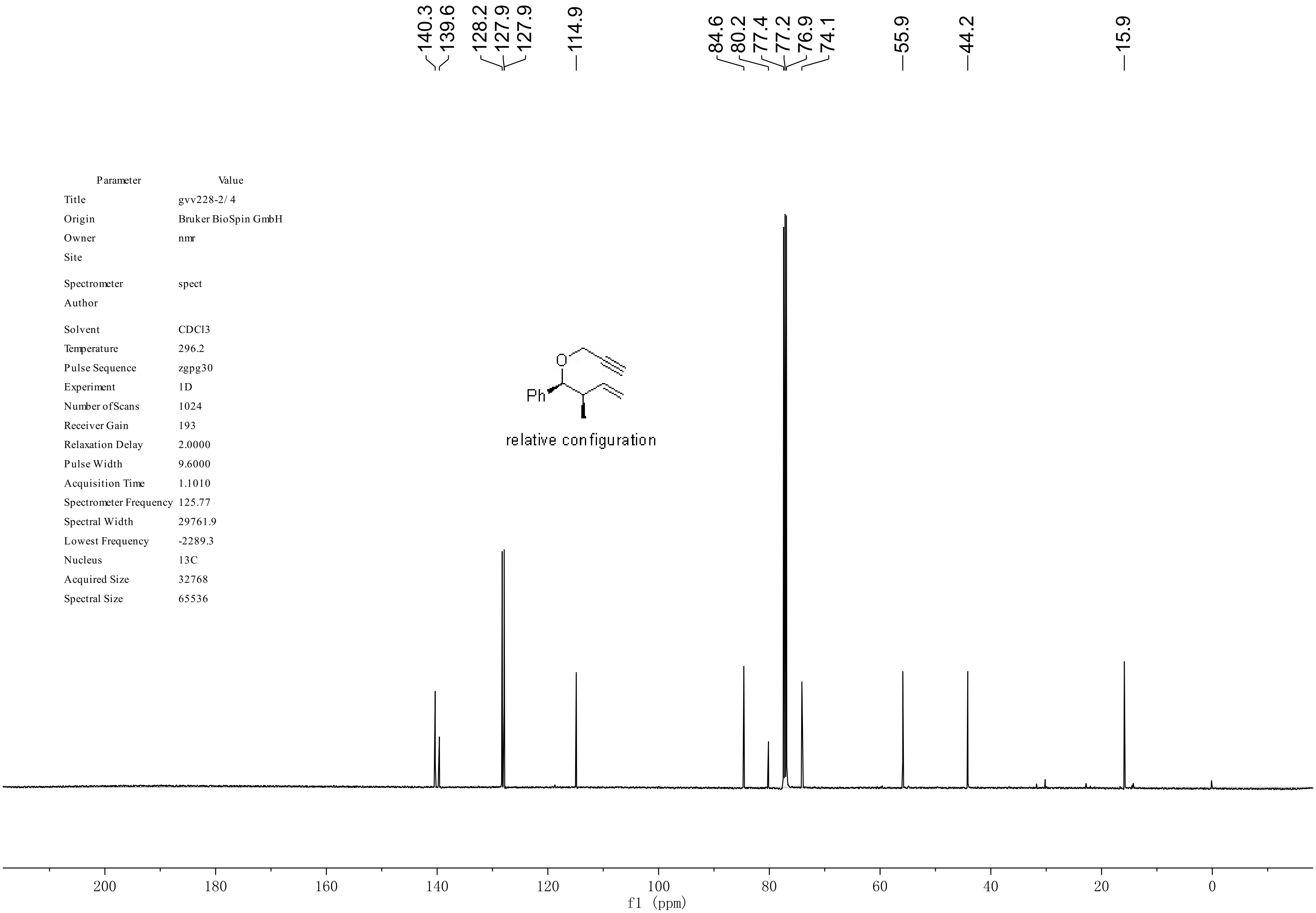

Parameter	Value
Title	gvv228-2/ 4
Origin	Bruker BioSpin GmbH
Owner	nmr
Site	
Spectrometer	spect
Author	
Solvent	CDCl3
Temperature	296.2
Pulse Sequence	zgpg30
Experiment	1D
Number of Scans	1024
Receiver Gain	193
Relaxation Delay	2.0000
Pulse Width	9.6000
Acquisition Time	1.1010
Spectrometer Frequency	125.77
Spectral Width	29761.9
Lowest Frequency	-2289.3
Nucleus	13C
Acquired Size	32768
Spectral Size	65536

Parameter	Value
Data File Name	E:/ NMR/ 2017/ 2017-9(1-6)/ xfy-0905-2/ 1/ fid
Comment	
Origin	Bruker BioSpin GmbH
Owner	nmr
Site	
Instrument	spect
Solvent	CDCl3
Temperature	296.3
Pulse Sequence	zg30
Experiment	1D
Number of Scans	8
Receiver Gain	25.3
Relaxation Delay	1.0000
Pulse Width	9.6000
Presaturation Frequency	
Acquisition Time	1.9999
Class	
Spectrometer Frequency	400.13
Spectral Width	8012.8
Lowest Frequency	-1547.0
Nucleus	1H
Acquired Size	16025
Spectral Size	32768

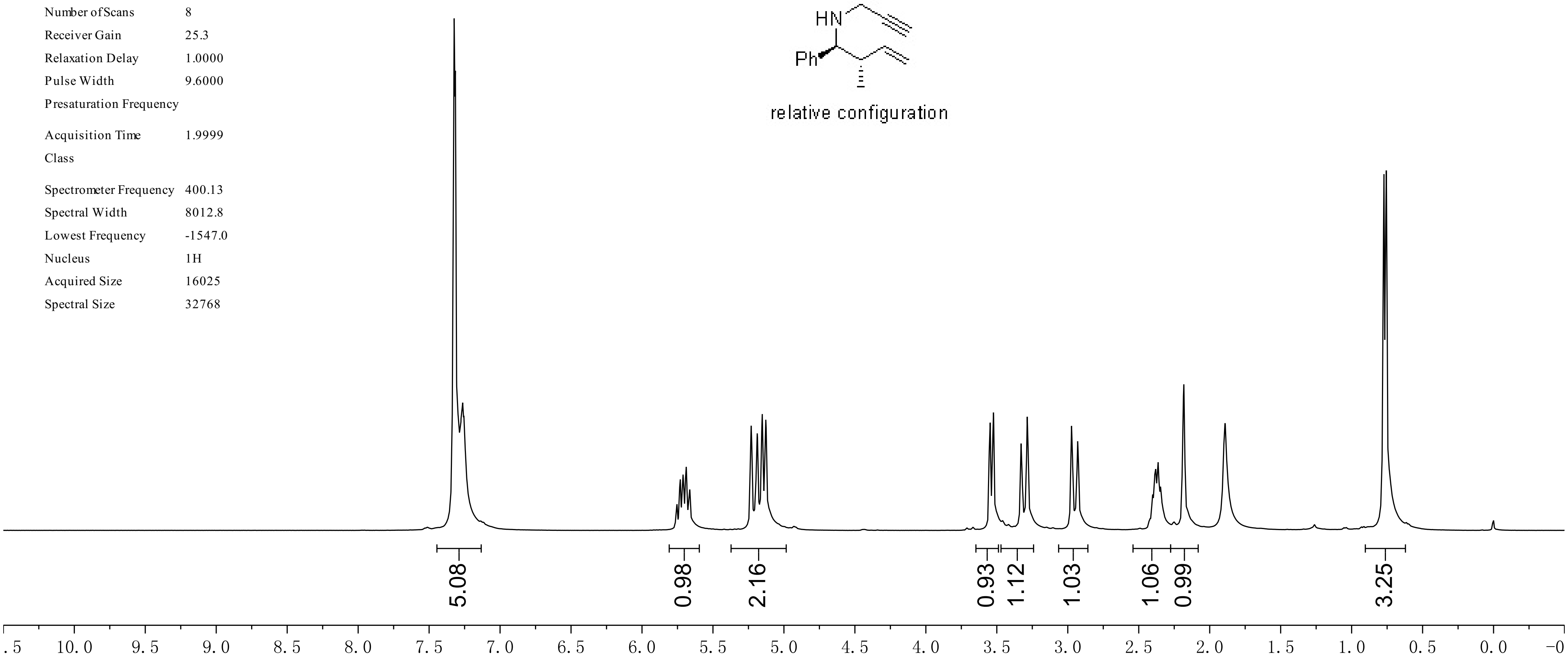

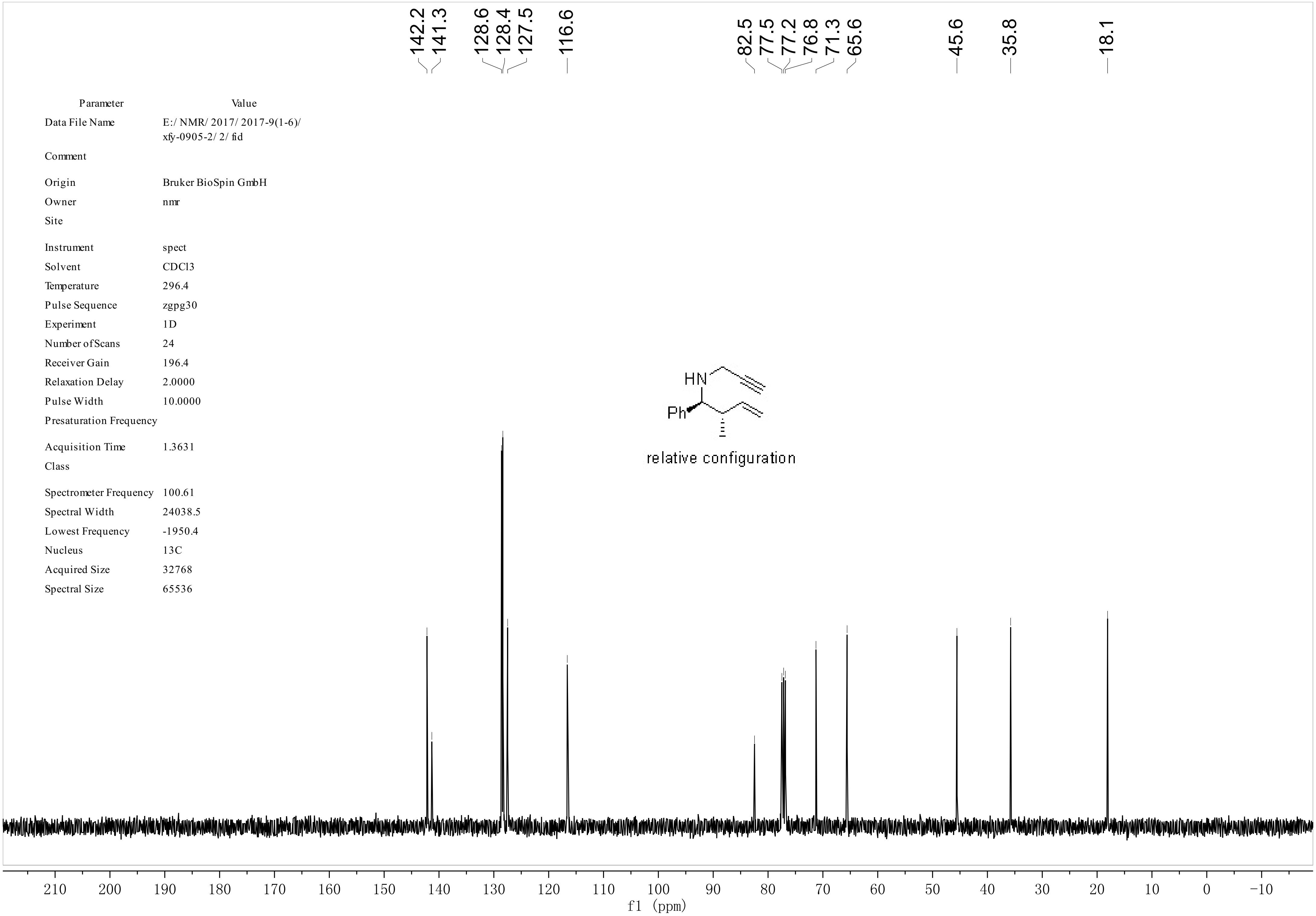

Parameter	Value
Data File Name	E:/ NMR/ 2017/ 2017-9(1-6)/ xfy-0905-2/ 2/ fid
Comment	
Origin	Bruker BioSpin GmbH
Owner	nmr
Site	
Instrument	spect
Solvent	CDCl3
Temperature	296.4
Pulse Sequence	zgpg30
Experiment	1D
Number of Scans	24
Receiver Gain	196.4
Relaxation Delay	2.0000
Pulse Width	10.0000
Presaturation Frequency	
Acquisition Time	1.3631
Class	
Spectrometer Frequency	100.61
Spectral Width	24038.5
Lowest Frequency	-1950.4
Nucleus	13C
Acquired Size	32768
Spectral Size	65536

Parameter	Value
Data File Name	E:/ NMR/ 2017/ 2017-9(14-21)/ xfy-0914-2/ 3/ pdata/ 1/ 1r
Comment	
Origin	Bruker BioSpin GmbH
Owner	nmr
Site	
Instrument	spect
Solvent	CDCl3
Temperature	296.1
Pulse Sequence	zg30
Experiment	1D
Number of Scans	16
Receiver Gain	31.1
Relaxation Delay	1.0000
Pulse Width	11.2900
Presaturation Frequency	
Acquisition Time	3.2768
Class	
Spectrometer Frequency	500.13
Spectral Width	10000.0
Lowest Frequency	-1911.5
Nucleus	1H
Acquired Size	32768
Spectral Size	65536

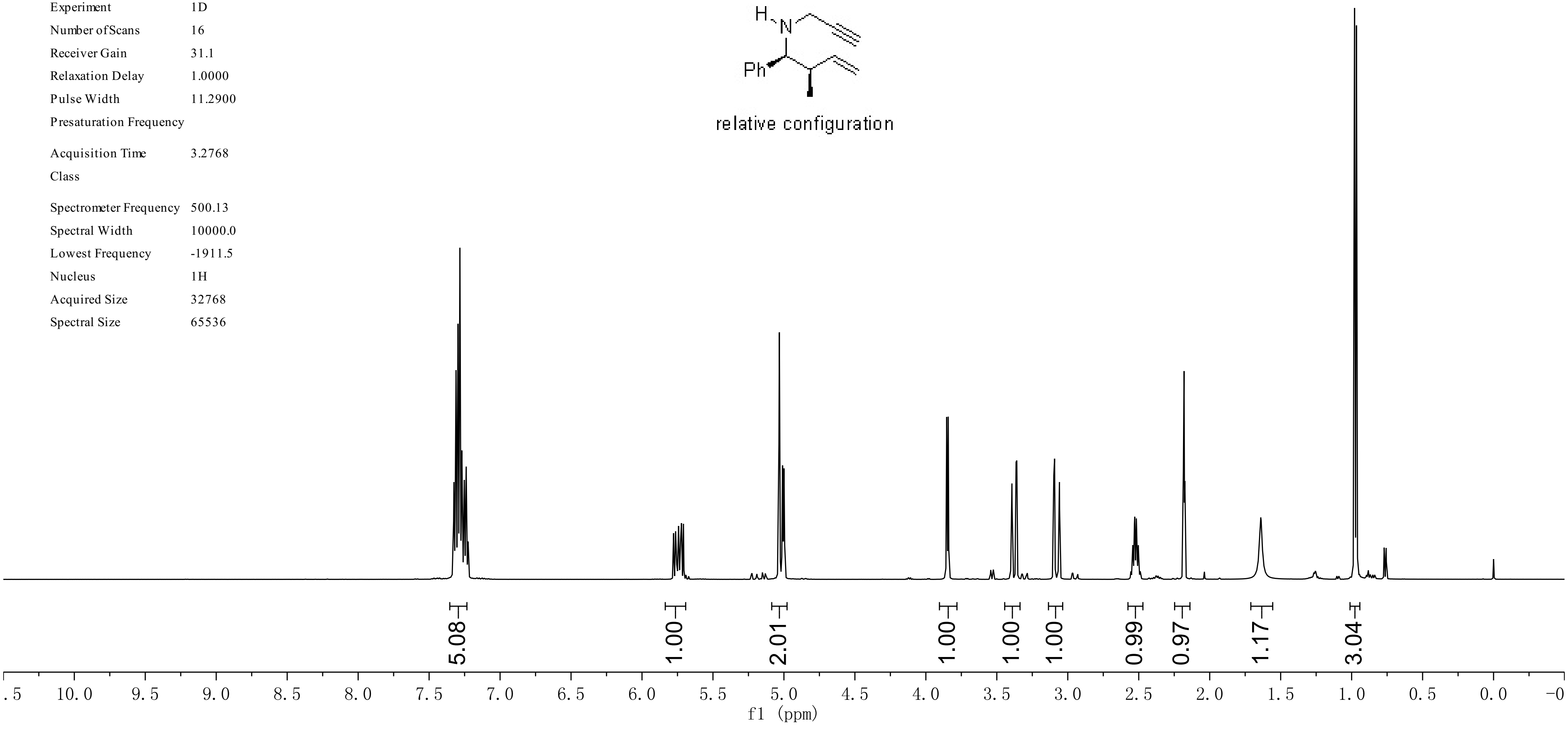

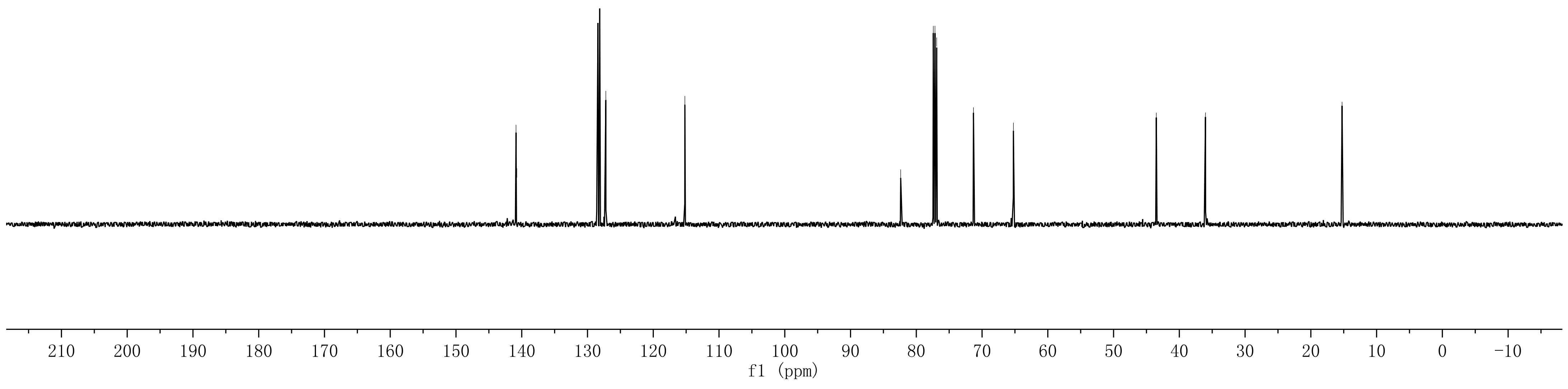

Parameter	Value
Data File Name	E:/ NMR/ 2017/ 2017-9(14-21)/ xfy-0914-2/ 4/ pdata/ 1/ 1r
Comment	
Origin	Bruker BioSpin GmbH
Owner	nmr
Site	
Instrument	spect
Solvent	CDCl3
Temperature	296.1
Pulse Sequence	zgpg30
Experiment	1D
Number of Scans	5
Receiver Gain	193.1
Relaxation Delay	2.0000
Pulse Width	9.6000
Presaturation Frequency	
Acquisition Time	1.1010
Class	
Spectrometer Frequency	125.76
Spectral Width	29761.9
Lowest Frequency	-2296.2
Nucleus	13C
Acquired Size	32768
Spectral Size	32768

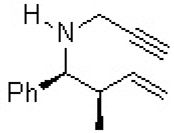

relative configuration

Parameter	Value
Data File Name	E:/ NMR/ 2018/ July/ 2018-7-18/ xfy-0904-8/ 21/ fid
Comment	
Origin	Bruker BioSpin GmbH
Owner	nmr
Site	
Instrument	spect
Solvent	CDCl3
Temperature	296.1
Pulse Sequence	zg30
Experiment	1D
Number of Scans	8
Receiver Gain	87.5
Relaxation Delay	1.0000
Pulse Width	10.7100
Presaturation Frequency	
Acquisition Time	3.2768
Class	
Spectrometer Frequency	500.13
Spectral Width	10000.0
Lowest Frequency	-1911.5
Nucleus	1H
Acquired Size	32768
Spectral Size	65536

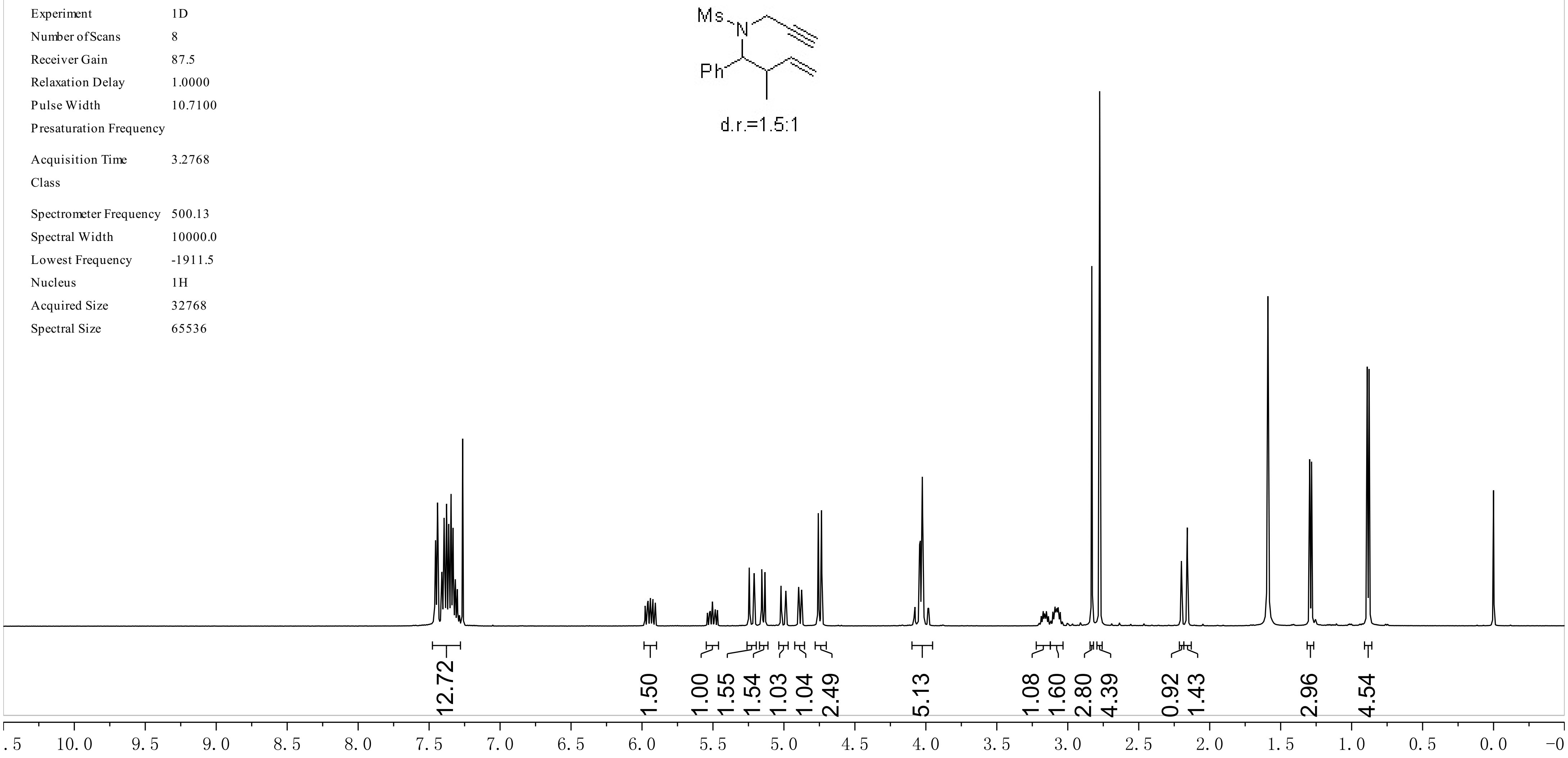

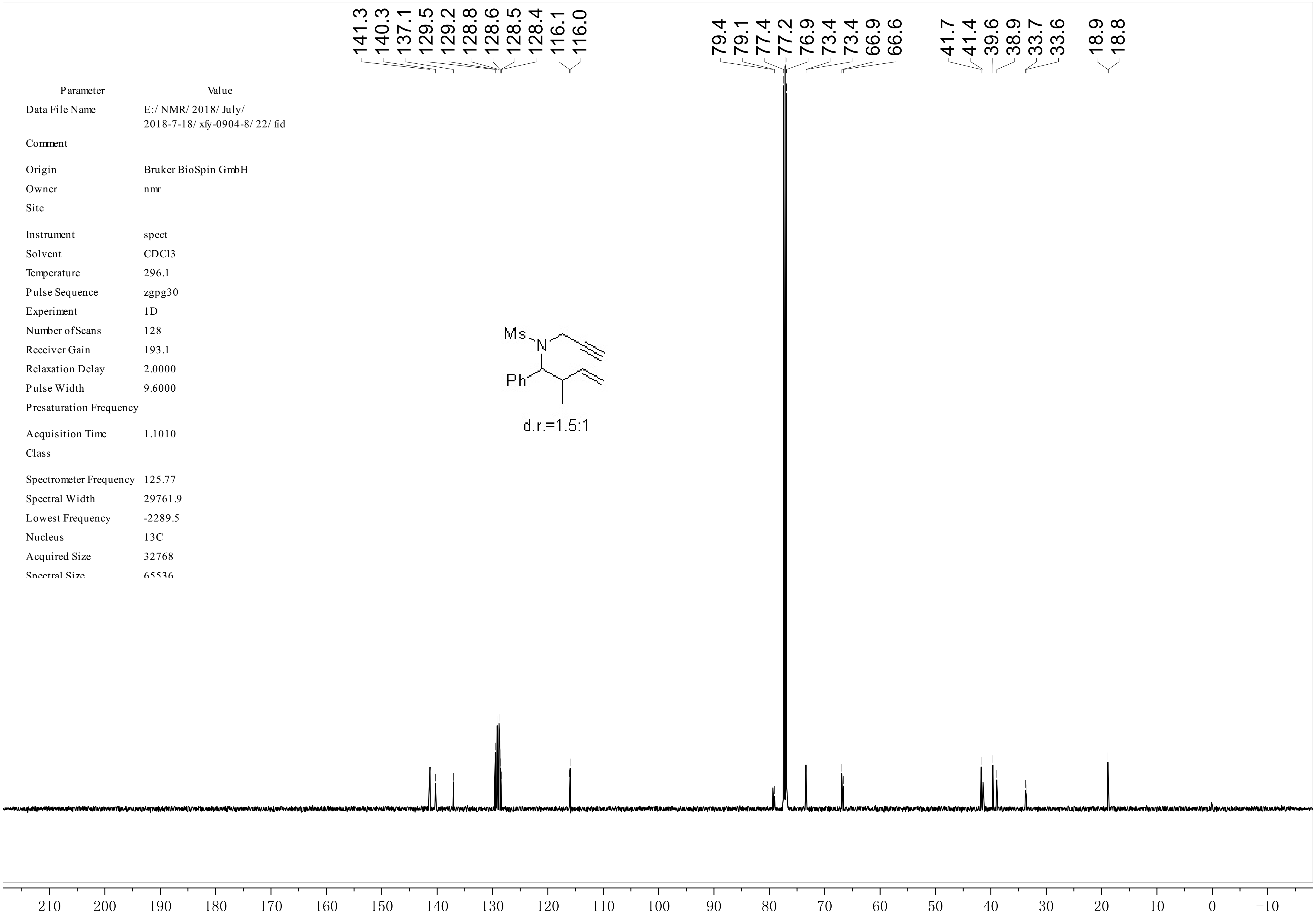

Parameter	Value
Data File Name	E:/ NMR/ 2018/ July/ 2018-7-18/ xfy-0904-8/ 22/ fid
Comment	
Origin	Bruker BioSpin GmbH
Owner	nmr
Site	
Instrument	spect
Solvent	CDCl3
Temperature	296.1
Pulse Sequence	zgpg30
Experiment	1D
Number of Scans	128
Receiver Gain	193.1
Relaxation Delay	2.0000
Pulse Width	9.6000
Presaturation Frequency	
Acquisition Time	1.1010
Class	
Spectrometer Frequency	125.77
Spectral Width	29761.9
Lowest Frequency	-2289.5
Nucleus	13C
Acquired Size	32768
Spectral Size	65536

Parameter	Value
Origin	Bruker BioSpin GmbH
Spectrometer	spect
Solvent	CDCl3
Temperature	296.1
Pulse Sequence	zg30
Experiment	1D
Number of Scans	16
Receiver Gain	88
Relaxation Delay	1.0000
Pulse Width	10.7100
Acquisition Time	3.2768
Spectrometer Frequency	500.13
Spectral Width	10000.0
Lowest Frequency	-1923.3
Nucleus	1H
Acquired Size	32768
Spectral Size	65536

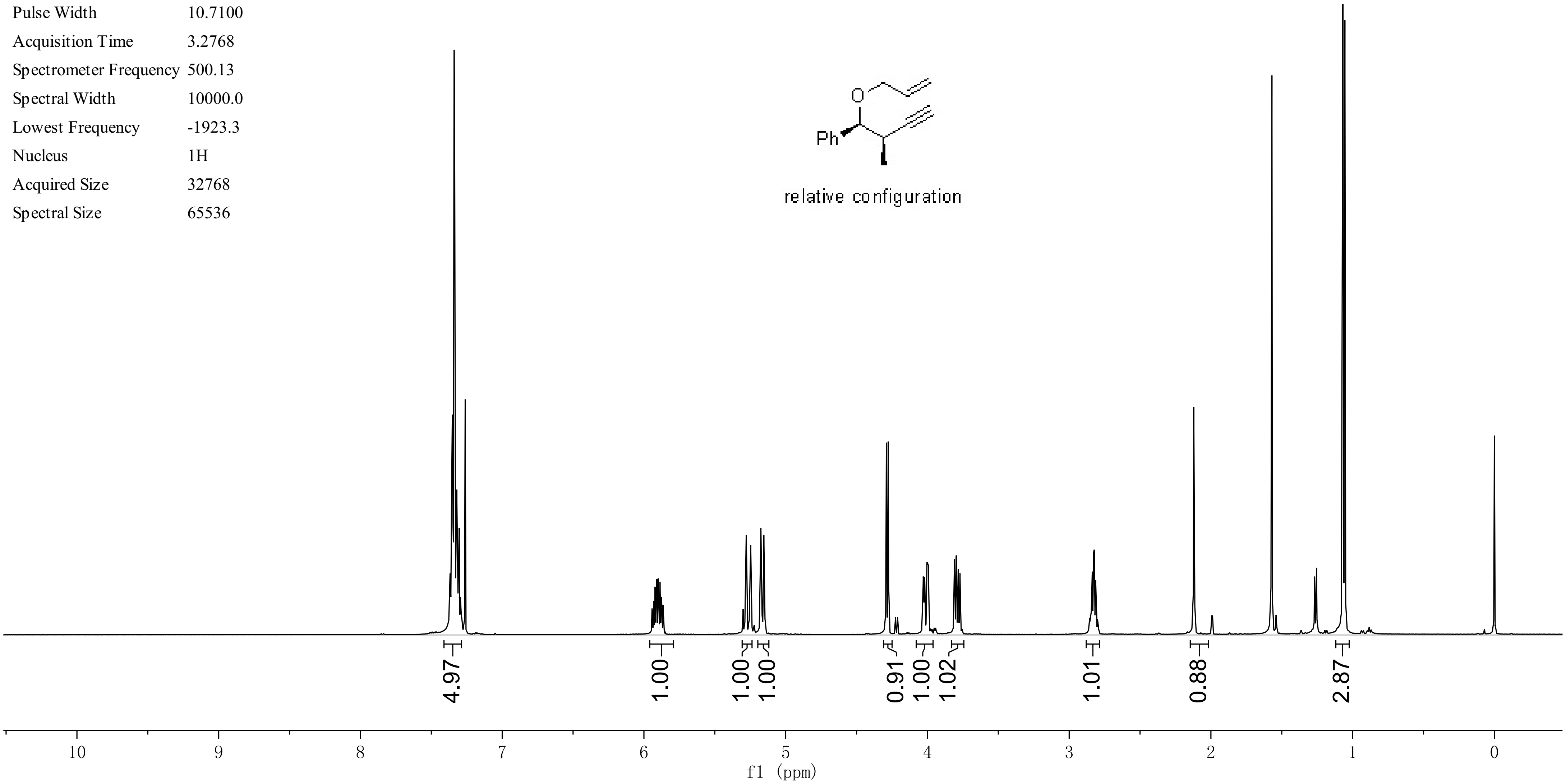

Parameter	Value
Origin	Bruker BioSpin GmbH
Spectrometer	spect
Solvent	CDCl3
Temperature	296.7
Pulse Sequence	zgpg30
Experiment	1D
Number of Scans	6
Receiver Gain	196
Relaxation Delay	2.0000
Pulse Width	10.0000
Acquisition Time	1.3631
Spectrometer Frequency	100.62
Spectral Width	24038.5
Lowest Frequency	-1943.7
Nucleus	13C
Acquired Size	32768
Spectral Size	65536

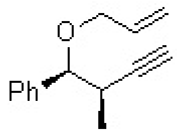

relative configuration

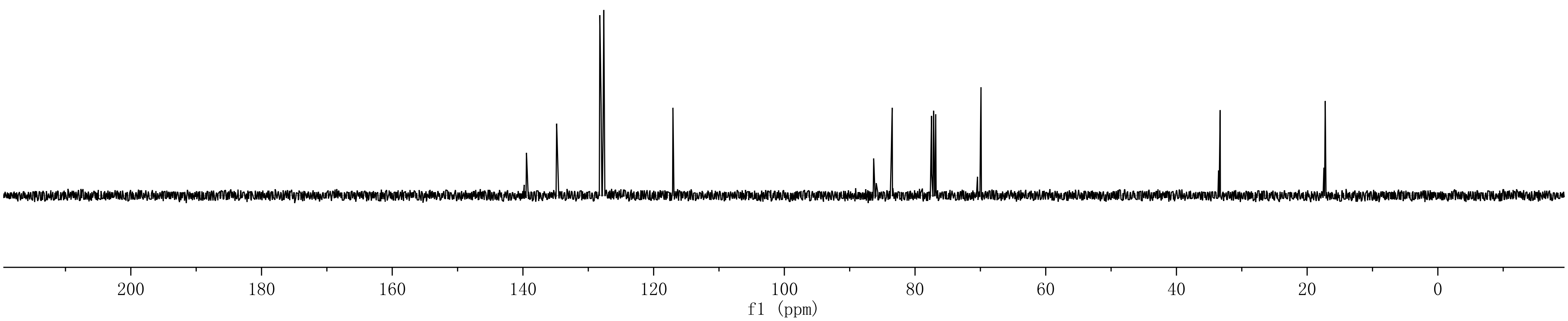

Parameter	Value
Data File Name	E:/ NMR/ 2017/ December/ 2017-12-2/ xfy-1202-1/ 1/ fid
Comment	
Origin	Bruker BioSpin GmbH
Owner	nmr
Site	
Instrument	spect
Solvent	CDCl3
Temperature	296.2
Pulse Sequence	zg30
Experiment	1D
Number of Scans	9
Receiver Gain	48.5
Relaxation Delay	1.0000
Pulse Width	10.7100
Presaturation Frequency	
Acquisition Time	3.2768
Class	
Spectrometer Frequency	500.13
Spectral Width	10000.0
Lowest Frequency	-1923.5
Nucleus	1H
Acquired Size	32768
Spectral Size	65536

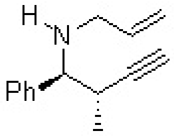

relative configuration

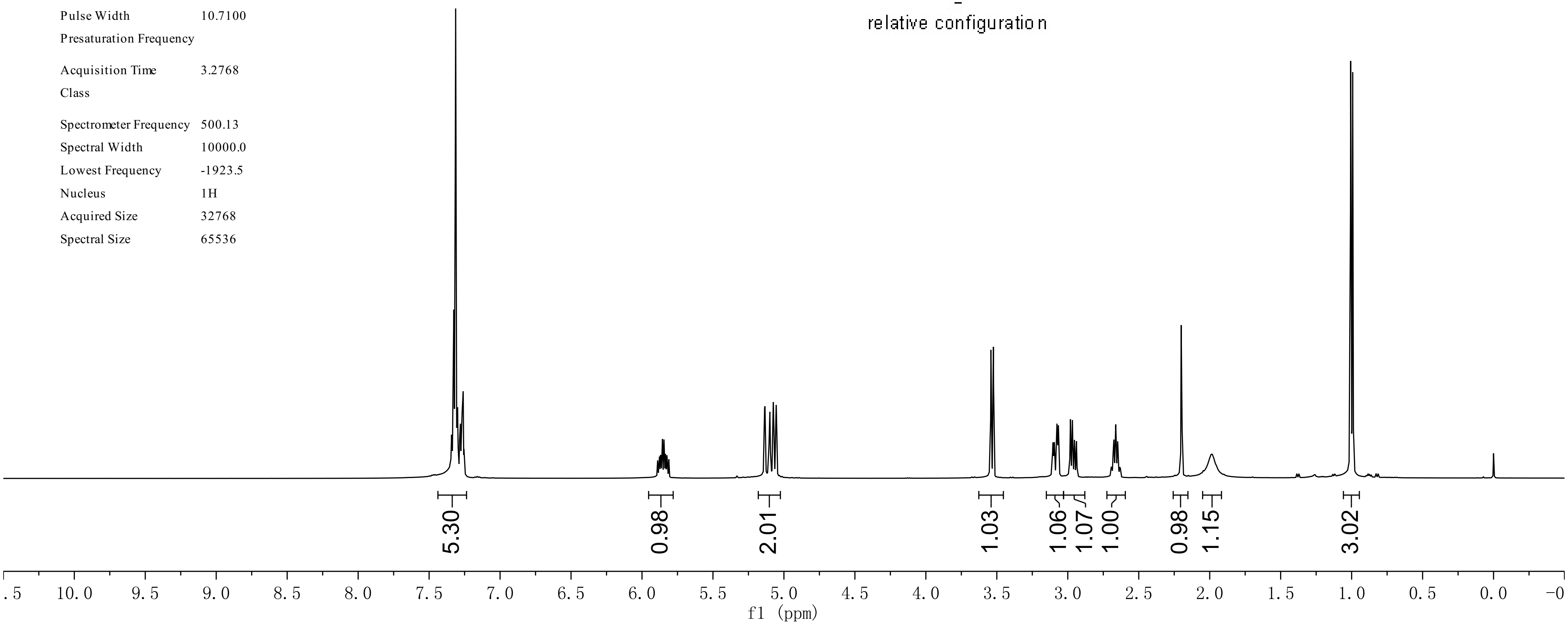

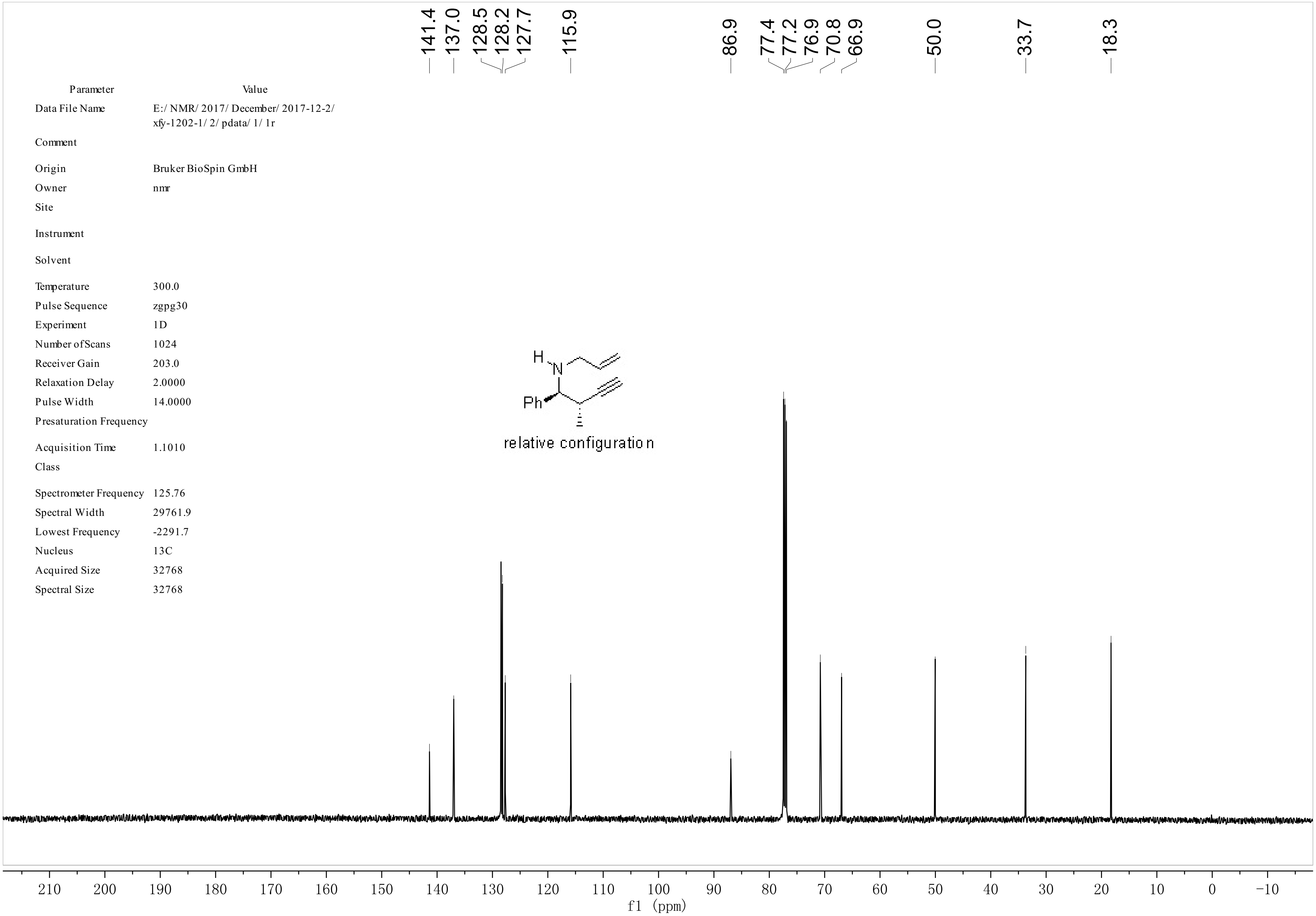
141.4
137.0
128.5
128.2
127.7
115.9
86.9
77.4
77.2
76.9
70.8
66.9
50.0
33.7
18.3
Parameter Value
Data File Name E:/ NMR/ 2017/ December/ 2017-12-2/ xfy-1202-1/ 2/ pdata/ 1/ 1r
Comment
Origin Bruker BioSpin GmbH
Owner nmr
Site
Instrument
Solvent
Temperature 300.0
Pulse Sequence zgpg30
Experiment 1D
Number of Scans 1024
Receiver Gain 203.0
Relaxation Delay 2.0000
Pulse Width 14.0000
Presaturation Frequency
Acquisition Time 1.1010
Class
Spectrometer Frequency 125.76
Spectral Width 29761.9
Lowest Frequency -2291.7
Nucleus 13C
Acquired Size 32768
Spectral Size 32768
H
N
Ph
relative configuration
210 200 190 180 170 160 150 140 130 120 110 100 90 80 70 60 50 40 30 20 10 0 −10
f1 (ppm)

Parameter	Value
Title	gvv210f4.2.fid
Origin	Bruker BioSpin GmbH
Owner	nmr
Site	
Spectrometer	spect
Author	
Solvent	CDCl3
Temperature	296.2
Pulse Sequence	zg30
Experiment	1D
Number of Scans	13
Receiver Gain	78
Relaxation Delay	1.0000
Pulse Width	10.7100
Acquisition Time	3.2768
Spectrometer Frequency	500.13
Spectral Width	10000.0
Lowest Frequency	-1923.2
Nucleus	1H
Acquired Size	32768
Spectral Size	65536

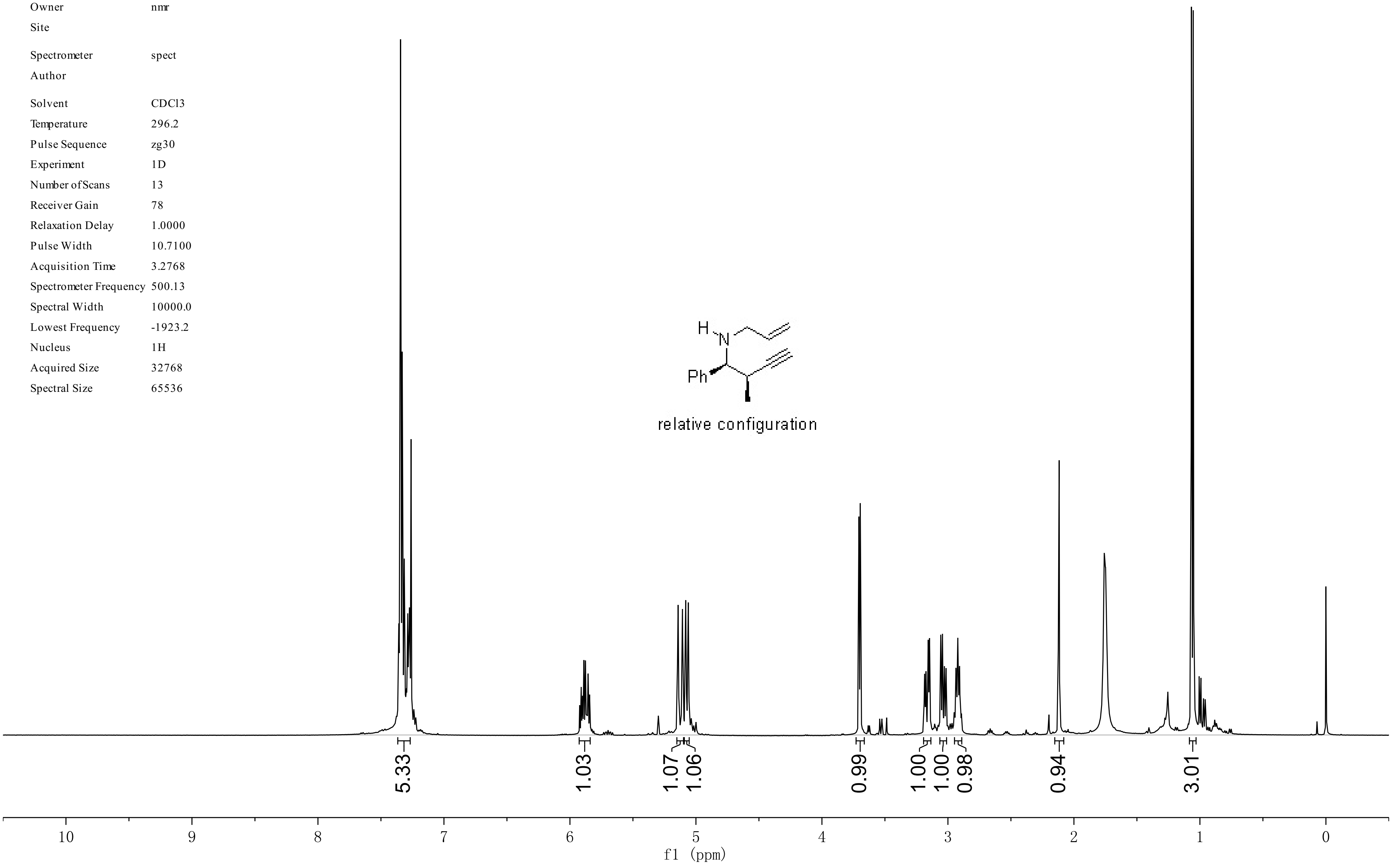

Parameter	Value
Title	gvv210f4.3.fid
Origin	Bruker BioSpin GmbH
Owner	nmr
Site	
Spectrometer	spect
Author	
Solvent	CDCl3
Temperature	296.1
Pulse Sequence	zgpg30
Experiment	1D
Number of Scans	51
Receiver Gain	193
Relaxation Delay	2.0000
Pulse Width	9.6000
Acquisition Time	1.1010
Spectrometer Frequency	125.77
Spectral Width	29761.9
Lowest Frequency	-2289.8
Nucleus	13C
Acquired Size	32768
Spectral Size	65536

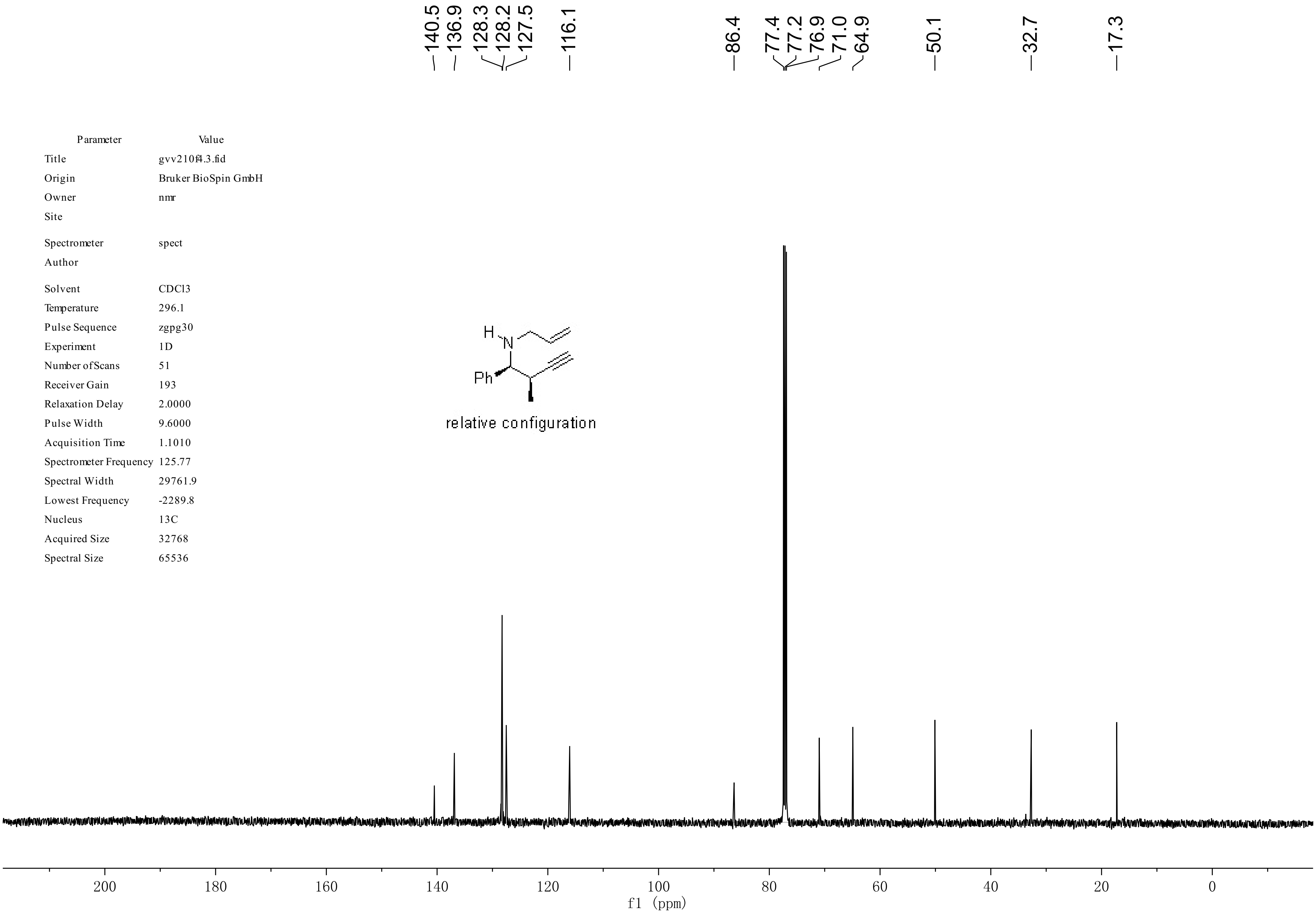

relative configuration

Parameter	Value
Title	gvv199fl/ 1
Origin	Bruker BioSpin GmbH
Owner	nmr
Site	
Spectrometer	spect
Author	
Solvent	CDCl3
Temperature	298.2
Pulse Sequence	zg30
Experiment	1D
Number of Scans	8
Receiver Gain	174
Relaxation Delay	1.0000
Pulse Width	8.7300
Acquisition Time	1.9999
Spectrometer Frequency	400.13
Spectral Width	8012.8
Lowest Frequency	-1545.0
Nucleus	1H
Acquired Size	16025
Spectral Size	65536

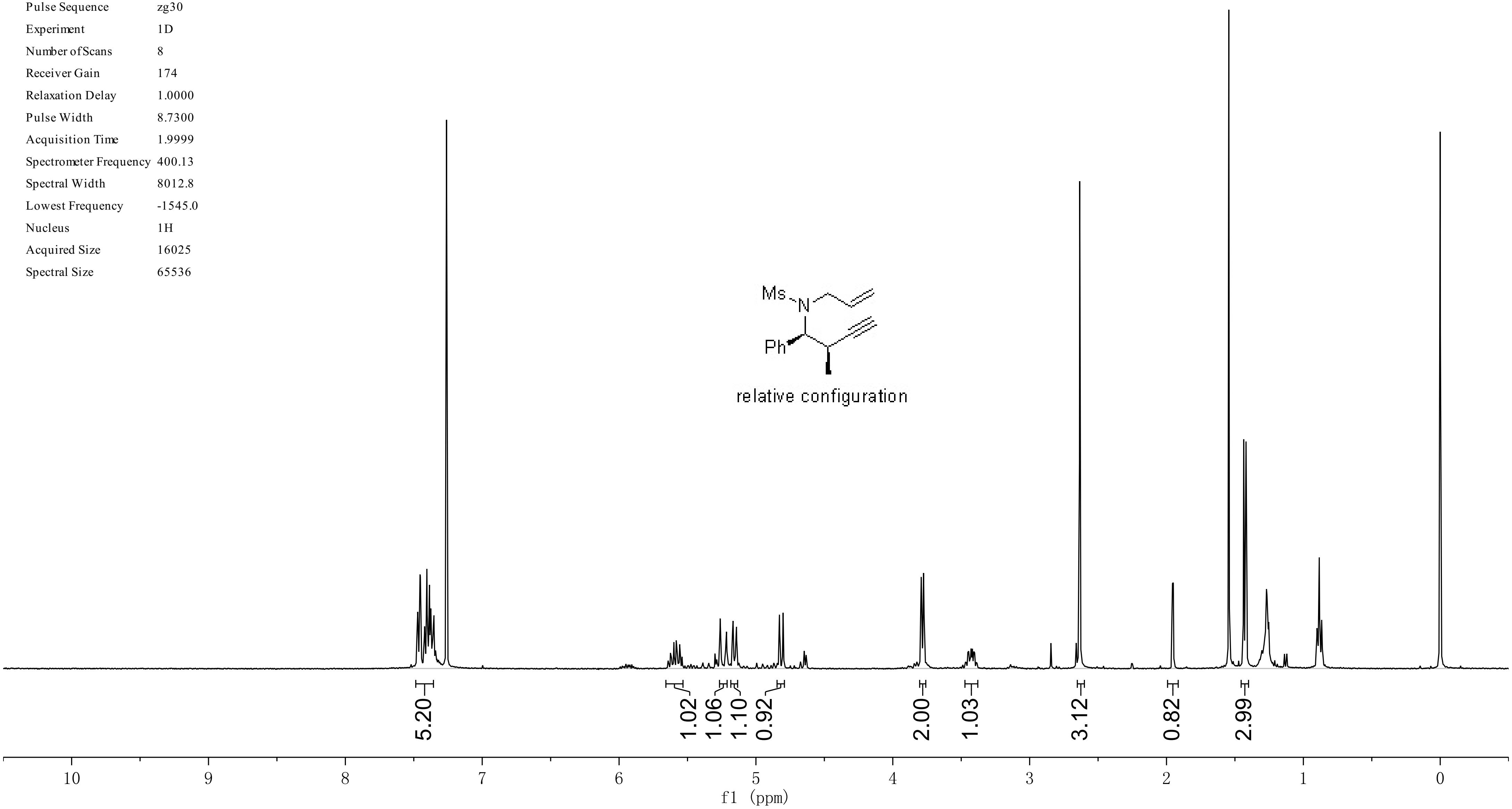

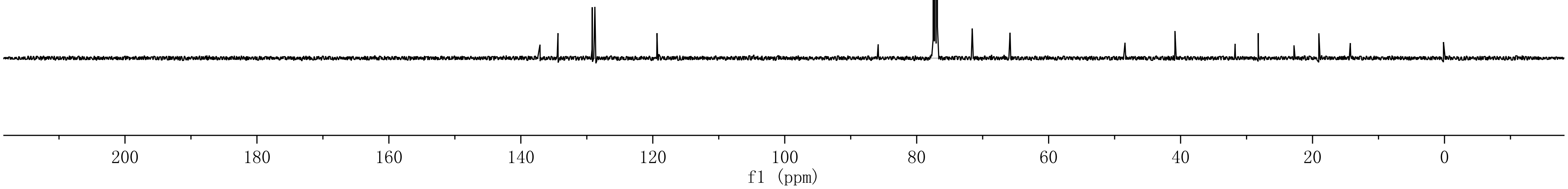

Parameter	Value
Title	gvv199fl-500/ 1
Origin	Bruker BioSpin GmbH
Owner	nmr
Site	
Spectrometer	spect
Author	
Solvent	CDCl3
Temperature	296.1
Pulse Sequence	zgpg30
Experiment	1D
Number of Scans	527
Receiver Gain	193
Relaxation Delay	2.0000
Pulse Width	9.6000
Acquisition Time	1.1010
Spectrometer Frequency	125.77
Spectral Width	29761.9
Lowest Frequency	-2288.7
Nucleus	13C
Acquired Size	32768
Spectral Size	65536

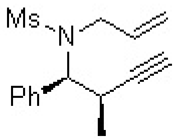

relative configuration

Parameter	Value
Title	lxl-0705-1/ 1
Origin	Bruker BioSpin GmbH
Owner	nmr
Site	
Spectrometer	spect
Author	
Solvent	CDCl3
Temperature	296.1
Pulse Sequence	zg30
Experiment	1D
Number of Scans	9
Receiver Gain	31
Relaxation Delay	1.0000
Pulse Width	10.7100
Acquisition Time	3.2768
Spectrometer Frequency	500.13
Spectral Width	10000.0
Lowest Frequency	-1929.5
Nucleus	1H
Acquired Size	32768
Spectral Size	65536

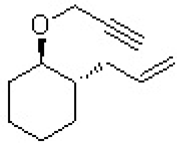

relative configuration only

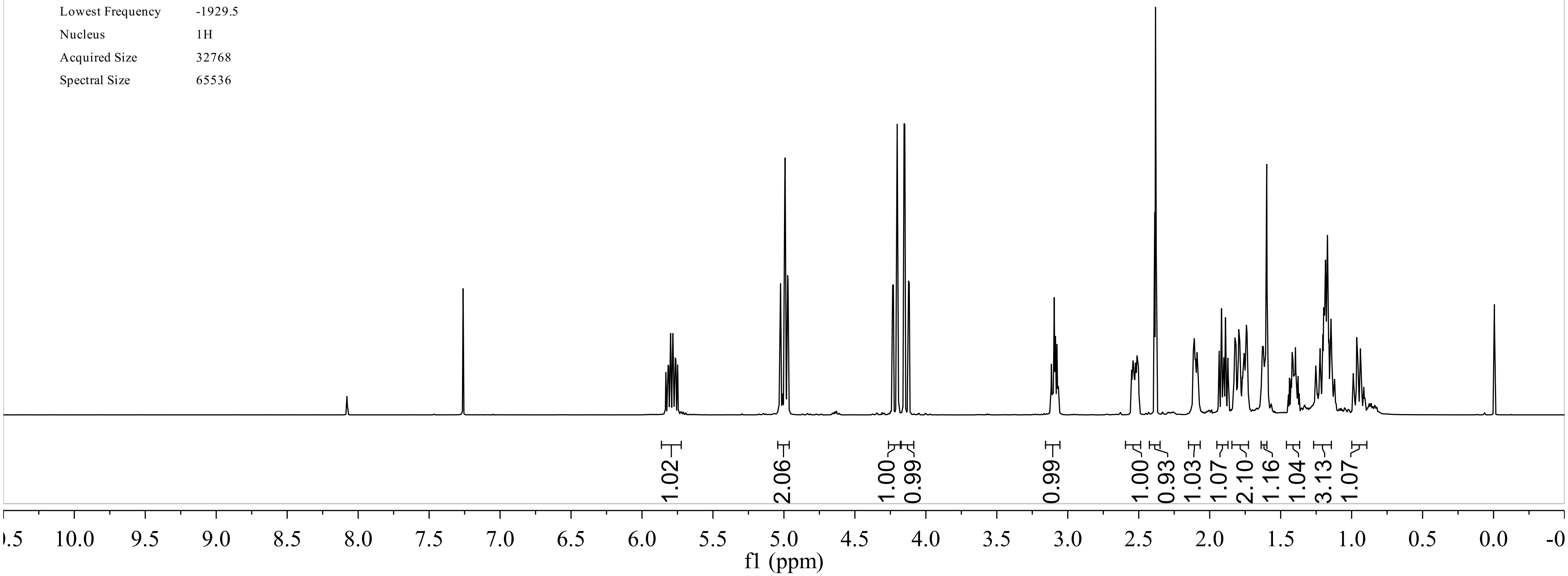

Parameter	Value
Title	lxl-0705-1/ 2
Origin	Bruker BioSpin GmbH
Owner	nmr
Site	
Spectrometer	spect
Author	
Solvent	CDCl3
Temperature	296.1
Pulse Sequence	zgpg30
Experiment	1D
Number of Scans	20
Receiver Gain	193
Relaxation Delay	2.0000
Pulse Width	9.6000
Acquisition Time	1.1010
Spectrometer Frequency	125.77
Spectral Width	29761.9
Lowest Frequency	-2273.8
Nucleus	13C
Acquired Size	32768
Spectral Size	65536

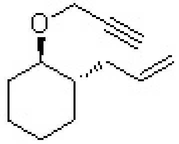

relative configuration only

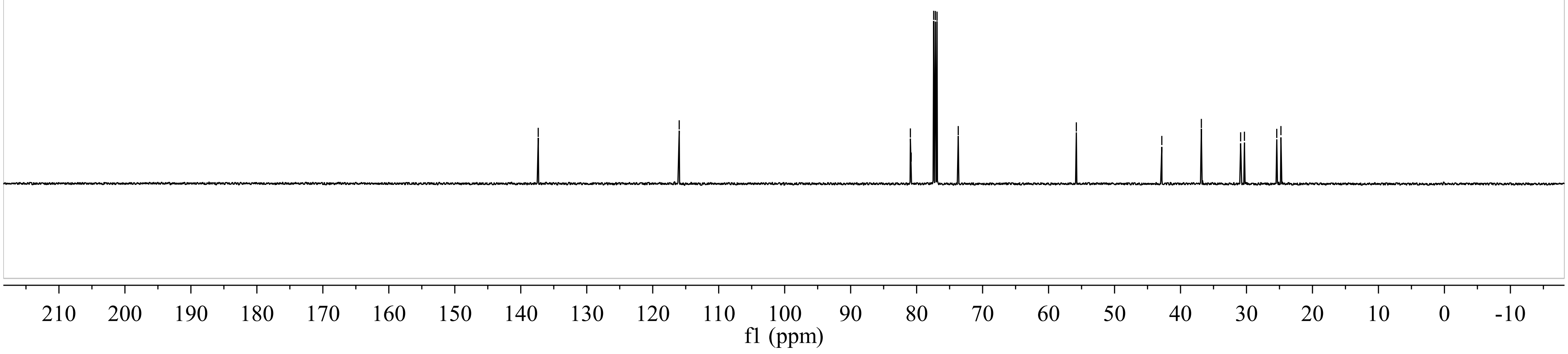

Parameter	Value
标题	xfy-190719-2.23.fid
Comment	
Origin	Bruker BioSpin GmbH
Owner	nmr
Site	
Instrument	spect
Author	
Solvent	CDCl3
Temperature	296.1
Pulse Sequence	zg30
Experiment	1D
Number of Scans	8
Receiver Gain	70.5
Relaxation Delay	1.0000
Pulse Width	10.7100
Presaturation Frequency	
Acquisition Time	3.2768
Class	
Spectrometer Frequency	500.13
Spectral Width	10000.0
Lowest Frequency	-1911.5
Nucleus	1H
Acquired Size	32768
Spectral Size	65536

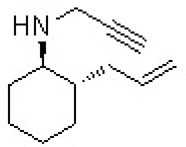

relative configuration

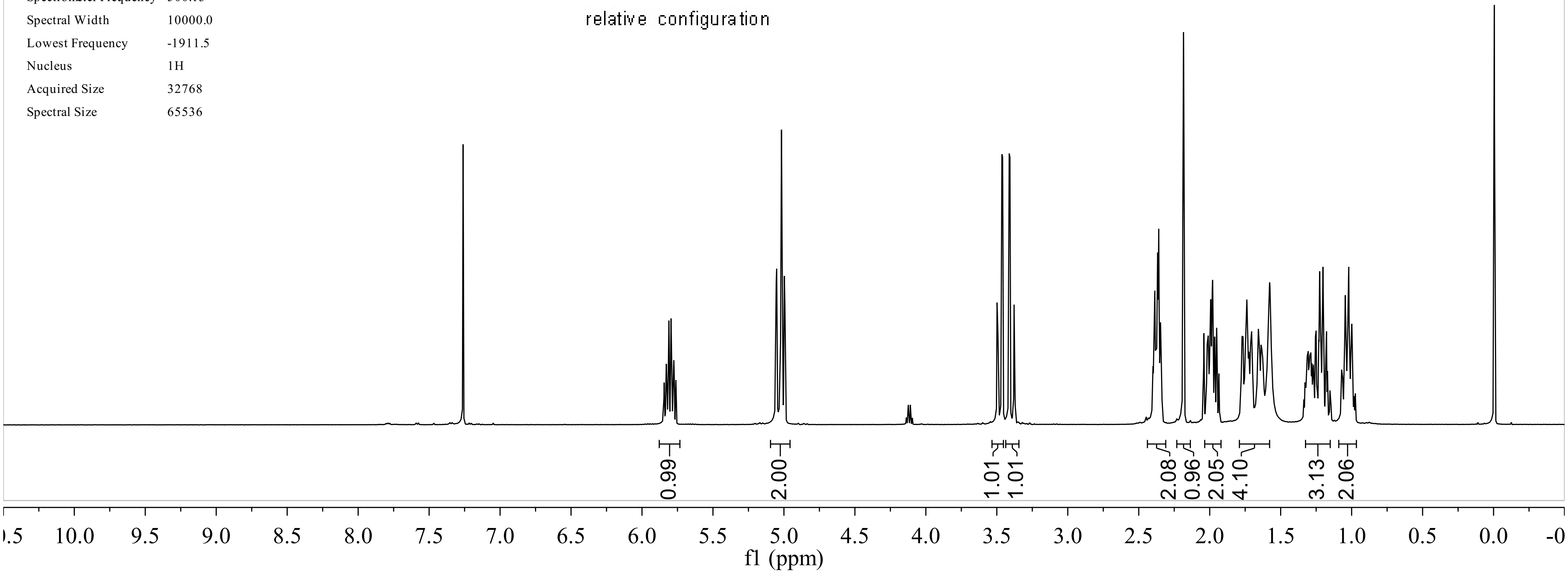

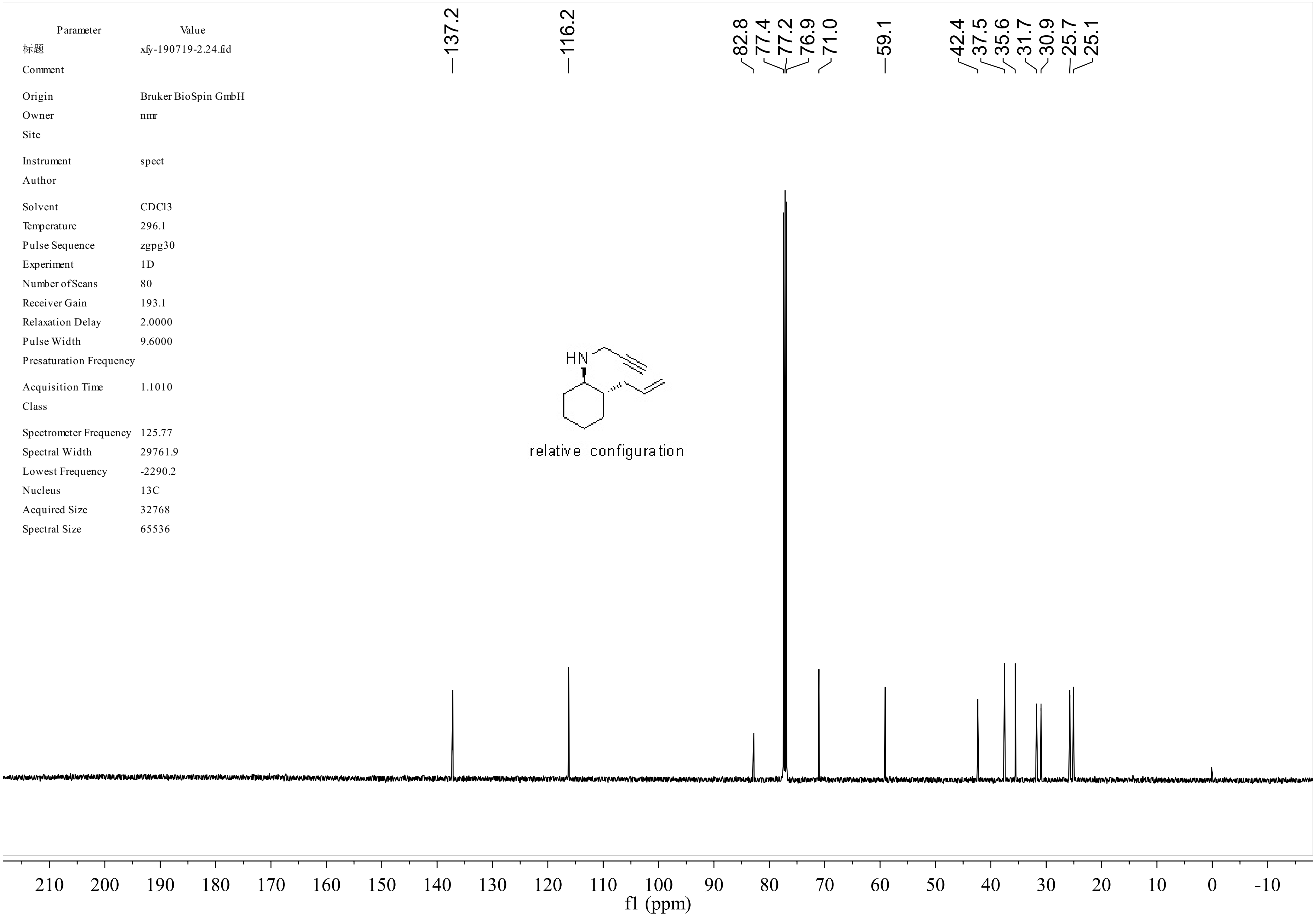

Parameter Value
标题 xfy-190719-2.24.fid
Comment
Origin Bruker BioSpin GmbH
Owner nmr
Site
Instrument spect
Author
Solvent CDCl3
Temperature 296.1
Pulse Sequence zgpg30
Experiment 1D
Number of Scans 80
Receiver Gain 193.1
Relaxation Delay 2.0000
Pulse Width 9.6000
Presaturation Frequency
Acquisition Time 1.1010
Class
Spectrometer Frequency 125.77
Spectral Width 29761.9
Lowest Frequency -2290.2
Nucleus 13C
Acquired Size 32768
Spectral Size 65536
137.2
116.2
82.8
77.4
77.2
76.9
71.0
59.1
42.4
37.5
35.6
31.7
30.9
25.7
25.1
HN
relative configuration
210 200 190 180 170 160 150 140 130 120 110 100 90 80 70 60 50 40 30 20 10 0 -10
f1 (ppm)

Parameter	Value
Title	lxl-0705-2/ 11
Origin	Bruker BioSpin GmbH
Owner	nmr
Site	
Spectrometer	spect
Author	
Solvent	CDCl3
Temperature	296.1
Pulse Sequence	zg30
Experiment	1D
Number of Scans	10
Receiver Gain	31
Relaxation Delay	1.0000
Pulse Width	10.7100
Acquisition Time	3.2768
Spectrometer Frequency	500.13
Spectral Width	10000.0
Lowest Frequency	-1916.5
Nucleus	1H
Acquired Size	32768
Spectral Size	65536

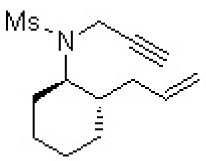

relative configuration only

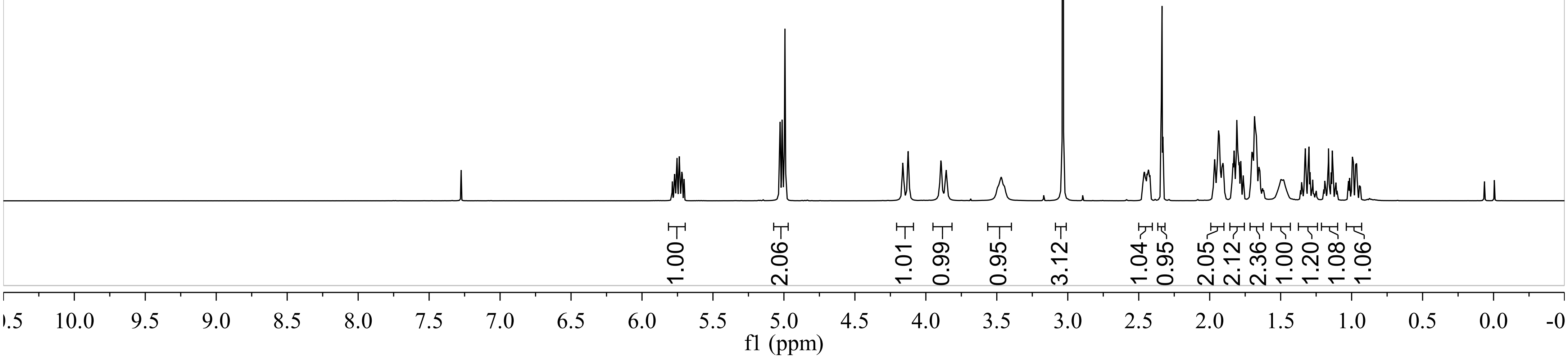

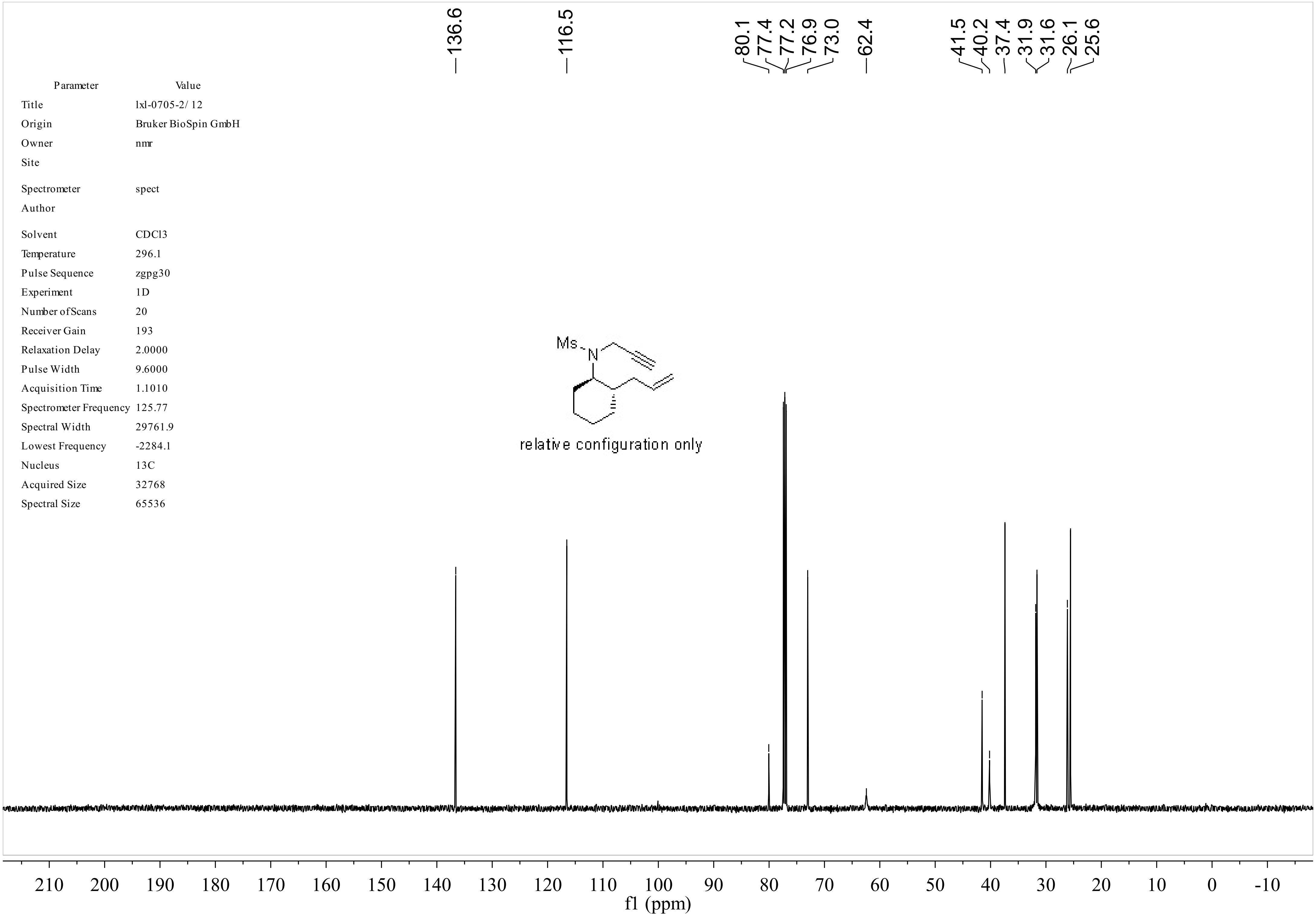

Parameter	Value
Title	lxl-0705-2/ 12
Origin	Bruker BioSpin GmbH
Owner	nmr
Site	
Spectrometer	spect
Author	
Solvent	CDCl3
Temperature	296.1
Pulse Sequence	zgpg30
Experiment	1D
Number of Scans	20
Receiver Gain	193
Relaxation Delay	2.0000
Pulse Width	9.6000
Acquisition Time	1.1010
Spectrometer Frequency	125.77
Spectral Width	29761.9
Lowest Frequency	-2284.1
Nucleus	13C
Acquired Size	32768
Spectral Size	65536

Parameter	Value
Origin	Bruker BioSpin GmbH
Spectrometer	spect
Solvent	CDCl3
Temperature	296.2
Pulse Sequence	zg30
Experiment	1D
Number of Scans	16
Receiver Gain	88
Relaxation Delay	1.0000
Pulse Width	10.7100
Acquisition Time	3.2768
Spectrometer Frequency	500.13
Spectral Width	10000.0
Lowest Frequency	-1924.0
Nucleus	1H
Acquired Size	32768
Spectral Size	65536

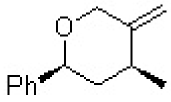

relative configuration

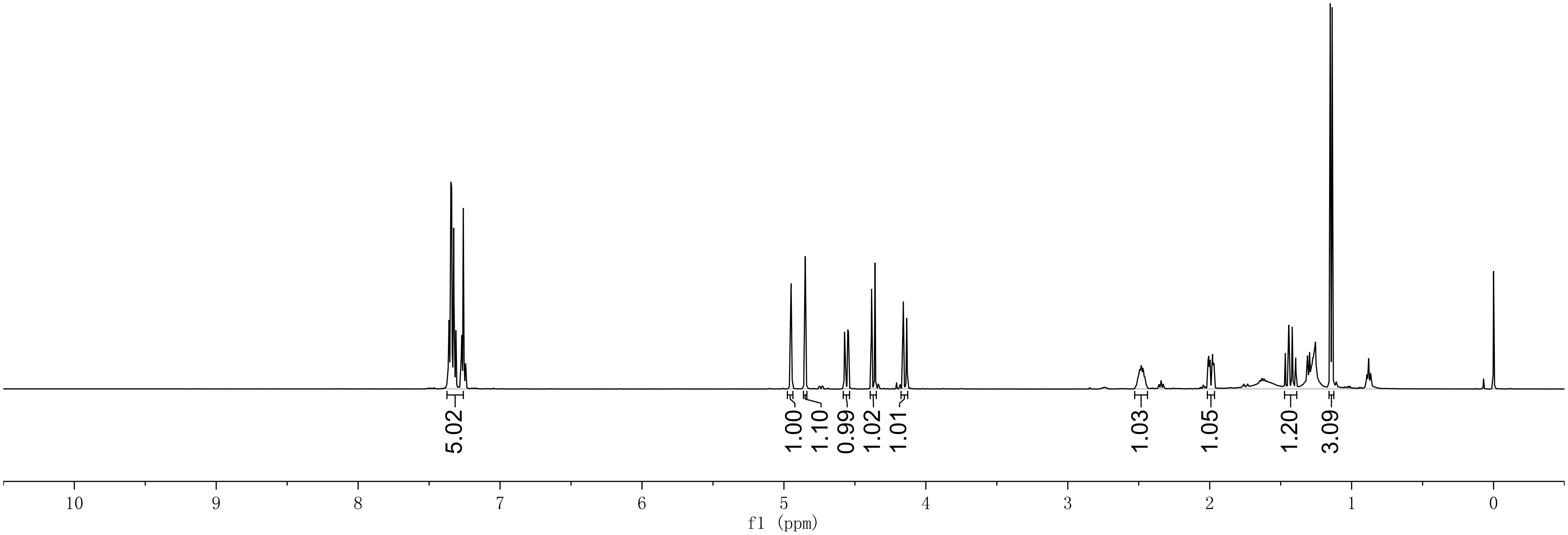

Parameter	Value
Origin	Bruker BioSpin GmbH
Spectrometer	spect
Solvent	CDCl3
Temperature	296.1
Pulse Sequence	zgpg30
Experiment	1D
Number of Scans	1024
Receiver Gain	193
Relaxation Delay	2.0000
Pulse Width	9.6000
Acquisition Time	1.1010
Spectrometer Frequency	125.77
Spectral Width	29761.9
Lowest Frequency	-2273.3
Nucleus	13C
Acquired Size	32768
Spectral Size	65536

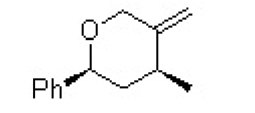

relative configuration

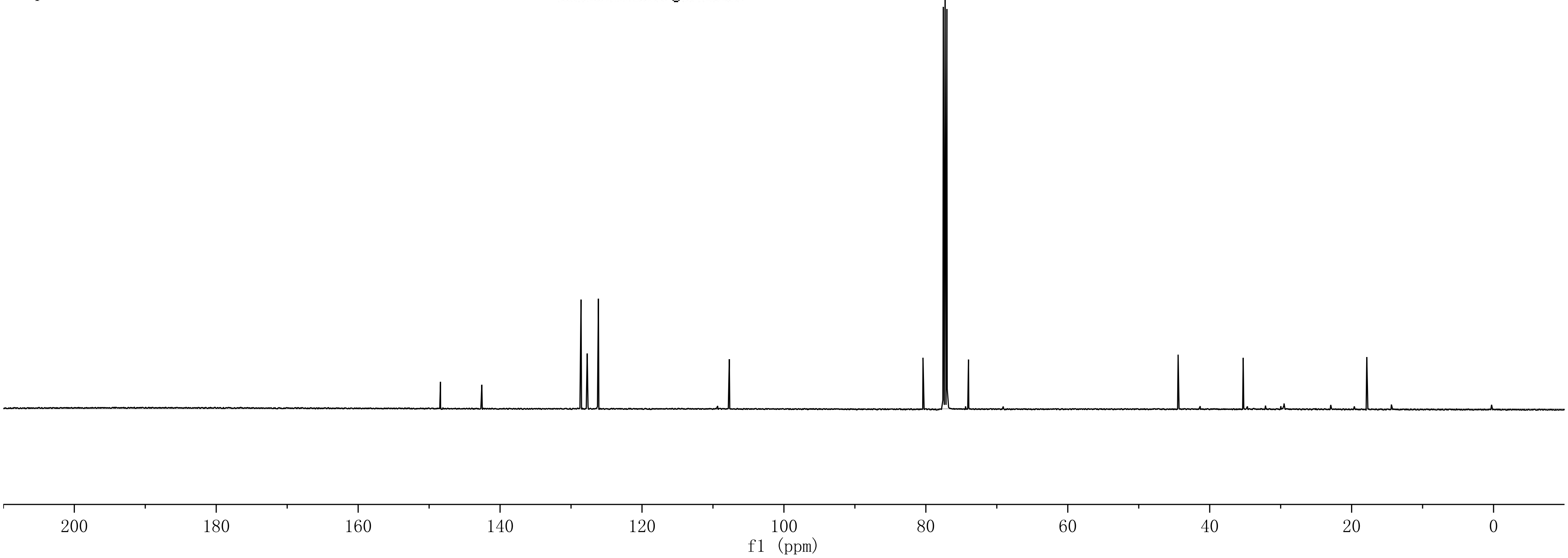

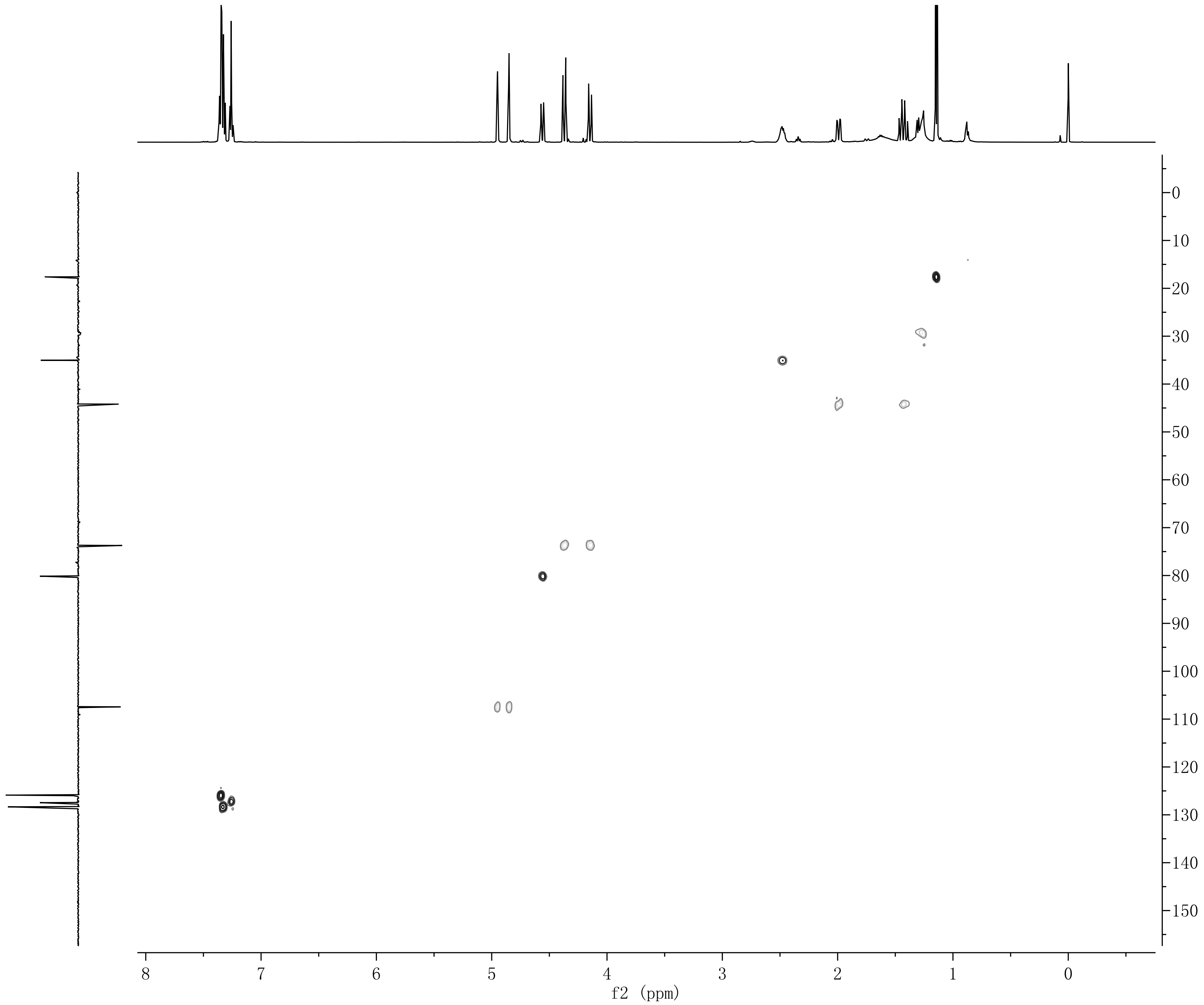

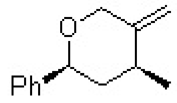

relative configuration

Parameter	Value
Origin	Bruker BioSpin GmbH
Spectrometer	spect
Solvent	CDCl3
Temperature	296.1
Pulse Sequence	hsqcedetgp
Experiment	HSQC-EDITED
Number of Scans	2
Receiver Gain	193
Relaxation Delay	1.4631
Pulse Width	10.7100
Acquisition Time	0.1157
Spectrometer Frequency	(500.13, 125.77)
Spectral Width	(4424.8, 20833.3)
Lowest Frequency	(-387.2, -1037.0)
Nucleus	(1H, 13C)
Acquired Size	(512, 256)
Spectral Size	(512, 512)

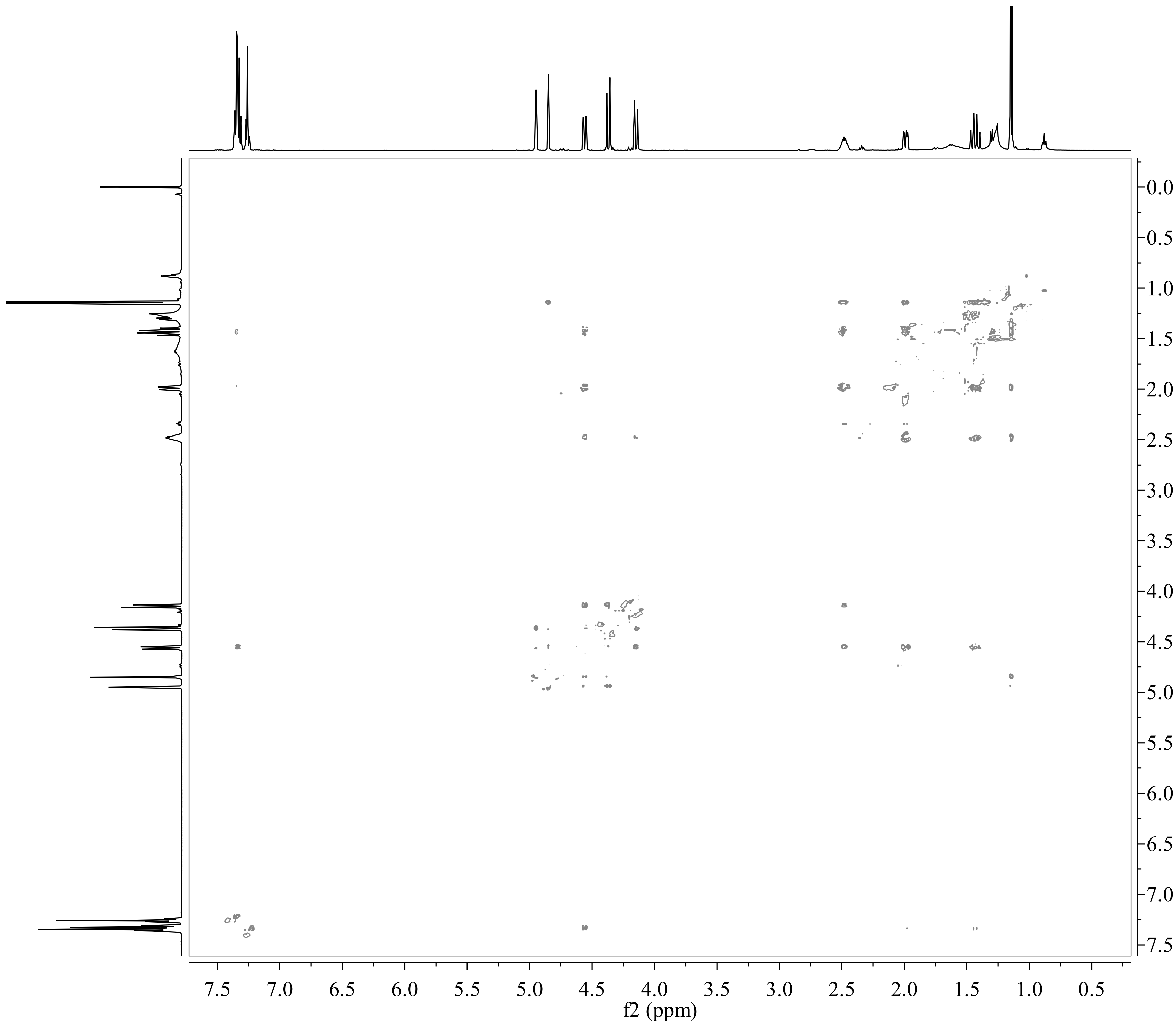

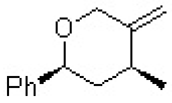

relative configuration

Parameter	Value
Title	gvv-e-70ff6.ser
Comment	
Origin	Bruker BioSpin GmbH
Owner	nmr
Site	
Instrument	spect
Solvent	CDCl3
Temperature	296.1
Pulse Sequence	noesygpphpp
Experiment	NOESY
Number of Scans	4
Receiver Gain	31.1
Relaxation Delay	1.9734
Pulse Width	10.7100
Presaturation Frequency	
Acquisition Time	0.2314
Acquisition Date	2018-07-20T05:29:52
Modification Date	2019-09-23T21:41:29
Spectrometer Frequency	(500.13, 500.13)
Spectral Width	(4424.8, 4424.8)
Lowest Frequency	(-387.2, -387.2)
Nucleus	(1H, 1H)
Acquired Size	(1024, 256)
Spectral Size	(1024, 1024)

	Parameter	Value
1	Title	xfy-0614-4-s1.1.fid
2	Comment	
3	Origin	Bruker BioSpin GmbH
4	Owner	nmr
5	Site	
6	Instrument	spect
7	Author	
8	Solvent	CDCl3
9	Temperature	296.1
10	Pulse Sequence	zg30
11	Experiment	1D
12	Number of Scans	8
13	Receiver Gain	62.9
14	Relaxation Delay	1.0000
15	Pulse Width	10.7100
16	Presaturation Frequency	
17	Acquisition Time	3.2768
18	Class	
19	Spectrometer Frequency	500.13
20	Spectral Width	10000.0
21	Lowest Frequency	-1924.6
22	Nucleus	1H
23	Acquired Size	32768
24	Spectral Size	65536

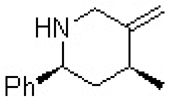

relative configuration

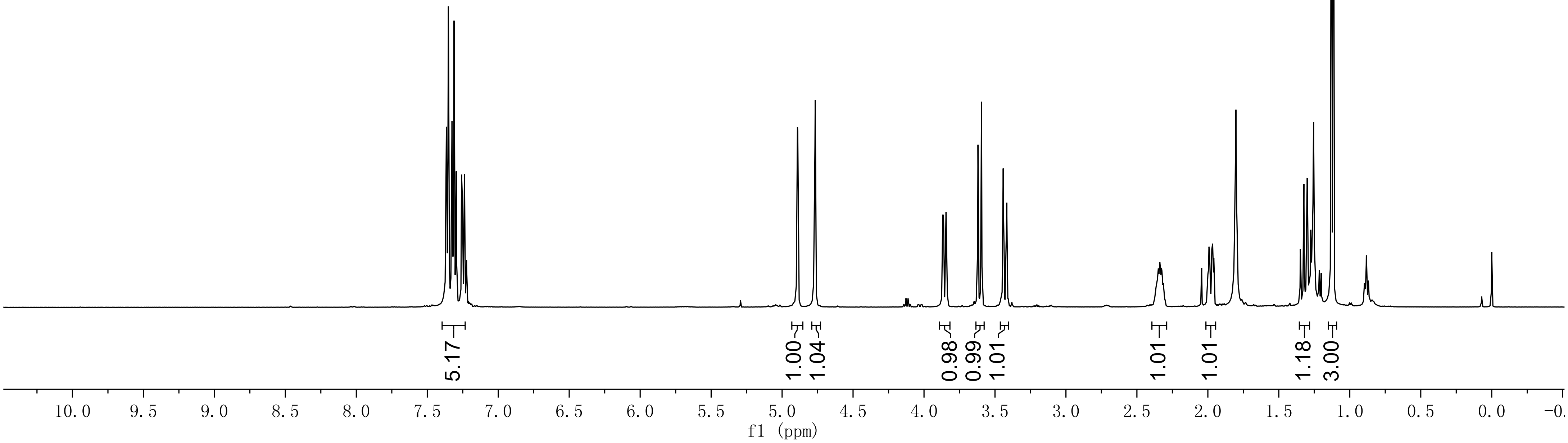

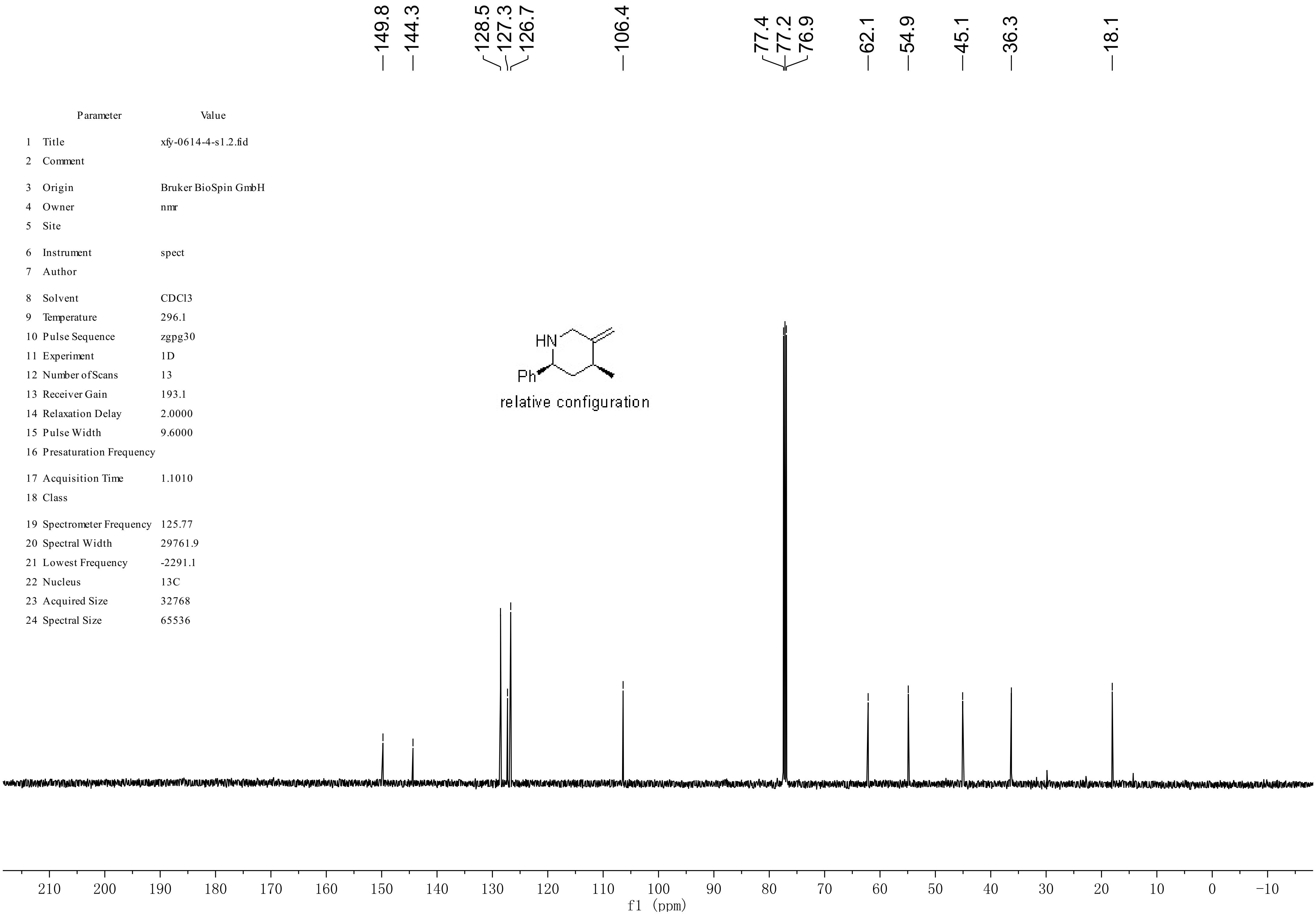

	Parameter	Value
1	Title	xfy-0614-4-s1.2.fid
2	Comment	
3	Origin	Bruker BioSpin GmbH
4	Owner	nmr
5	Site	
6	Instrument	spect
7	Author	
8	Solvent	CDCl3
9	Temperature	296.1
10	Pulse Sequence	zgpg30
11	Experiment	1D
12	Number of Scans	13
13	Receiver Gain	193.1
14	Relaxation Delay	2.0000
15	Pulse Width	9.6000
16	Presaturation Frequency	
17	Acquisition Time	1.1010
18	Class	
19	Spectrometer Frequency	125.77
20	Spectral Width	29761.9
21	Lowest Frequency	-2291.1
22	Nucleus	13C
23	Acquired Size	32768
24	Spectral Size	65536

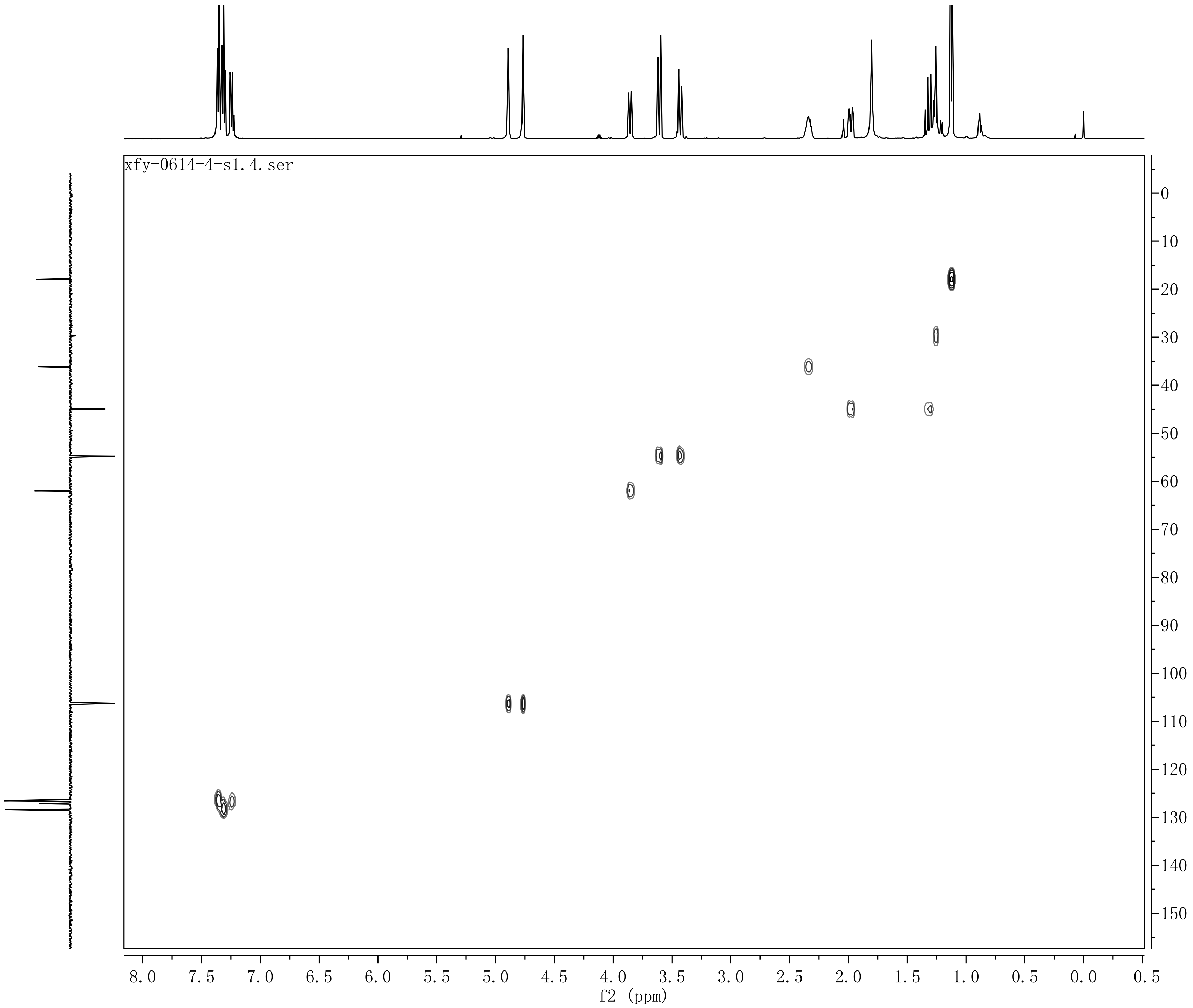

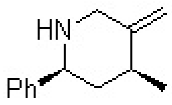

relative configuration

Parameter	Value
Title	xfy-0614-4-s1.4.ser
Comment	
Origin	Bruker BioSpin GmbH
Owner	nmr
Site	
Instrument	spect
Solvent	CDCl3
Temperature	296.2
Pulse Sequence	hsqcetgp
Experiment	HSQC
Number of Scans	2
Receiver Gain	193.1
Relaxation Delay	1.4611
Pulse Width	10.7100
Presaturation Frequency	
Acquisition Time	0.1178
Class	
Spectrometer Frequency	(500.13, 125.77)
Spectral Width	(4347.8, 20833.3)
Lowest Frequency	(-267.2, -1037.0)
Nucleus	(1H, 13C)
Acquired Size	(512, 129)
Spectral Size	(512, 512)

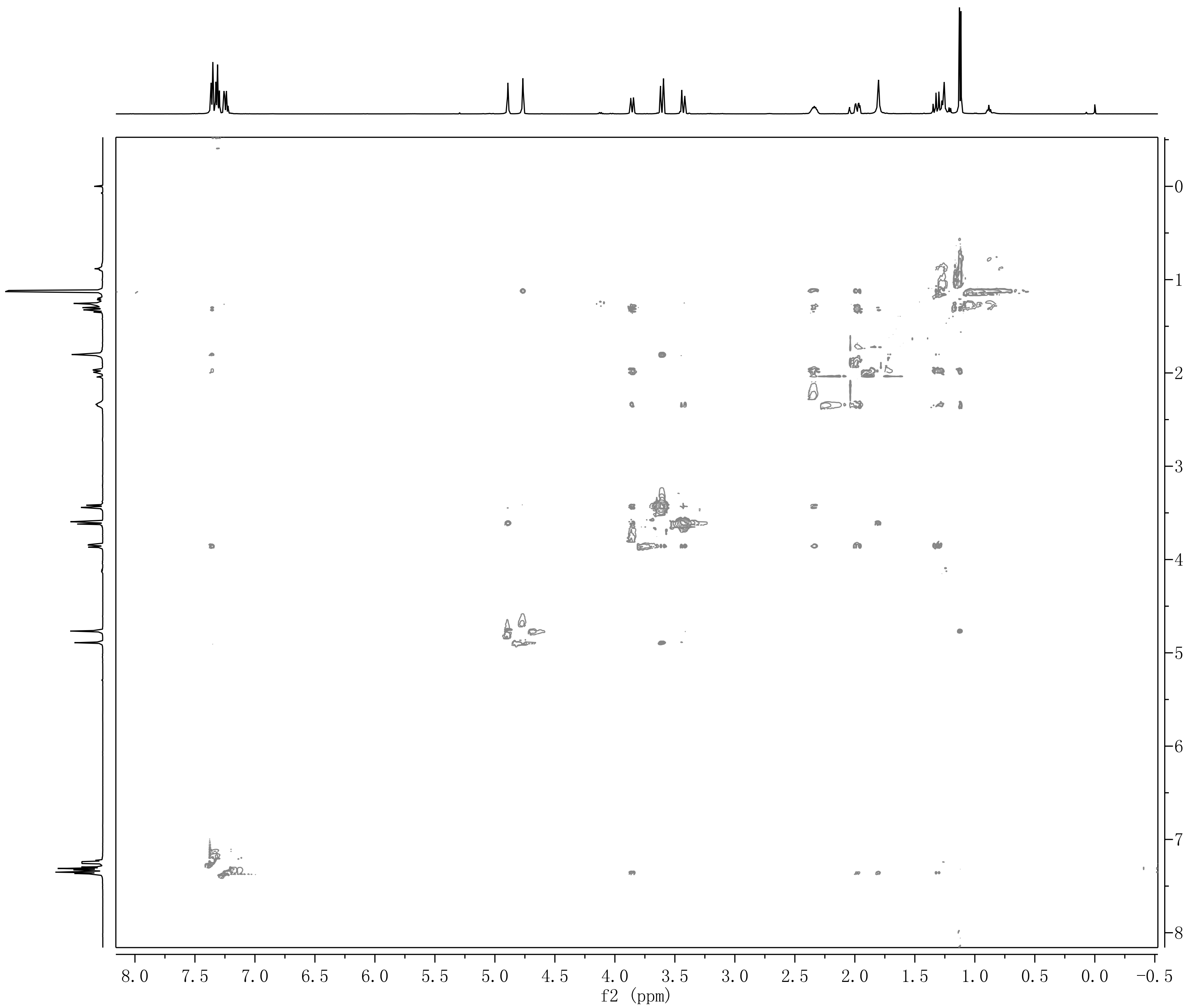

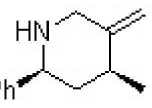

relative configuration

	Parameter	Value
1	Title	xfy-0614-4-s1.5.ser
2	Comment	
3	Origin	Bruker BioSpin GmbH
4	Owner	nmr
5	Site	
6	Instrument	spect
7	Author	
8	Solvent	CDCl3
9	Temperature	296.1
10	Pulse Sequence	noesygpphpp
11	Experiment	NOESY
12	Number of Scans	4
13	Receiver Gain	48.5
14	Relaxation Delay	1.9693
15	Pulse Width	10.7100
16	Presaturation Frequency	
17	Acquisition Time	0.2355
18	Class	
19	Spectrometer Frequency	(500.13, 500.13)
20	Spectral Width	(4347.8, 4347.8)
21	Lowest Frequency	(-267.2, -267.2)
22	Nucleus	(1H, 1H)
23	Acquired Size	(1024, 256)
24	Spectral Size	(1024, 1024)

	Parameter	Value
1	Title	xfy-0614-4-s1-dMs.11.fid
2	Comment	
3	Origin	Bruker BioSpin GmbH
4	Owner	nmr
5	Site	
6	Instrument	spect
7	Author	
8	Solvent	CDCl3
9	Temperature	296.1
10	Pulse Sequence	zg30
11	Experiment	1D
12	Number of Scans	4
13	Receiver Gain	54.3
14	Relaxation Delay	1.0000
15	Pulse Width	10.7100
16	Presaturation Frequency	
17	Acquisition Time	3.2768
18	Class	
19	Spectrometer Frequency	500.13
20	Spectral Width	10000.0
21	Lowest Frequency	-1923.1
22	Nucleus	1H
23	Acquired Size	32768
24	Spectral Size	65536

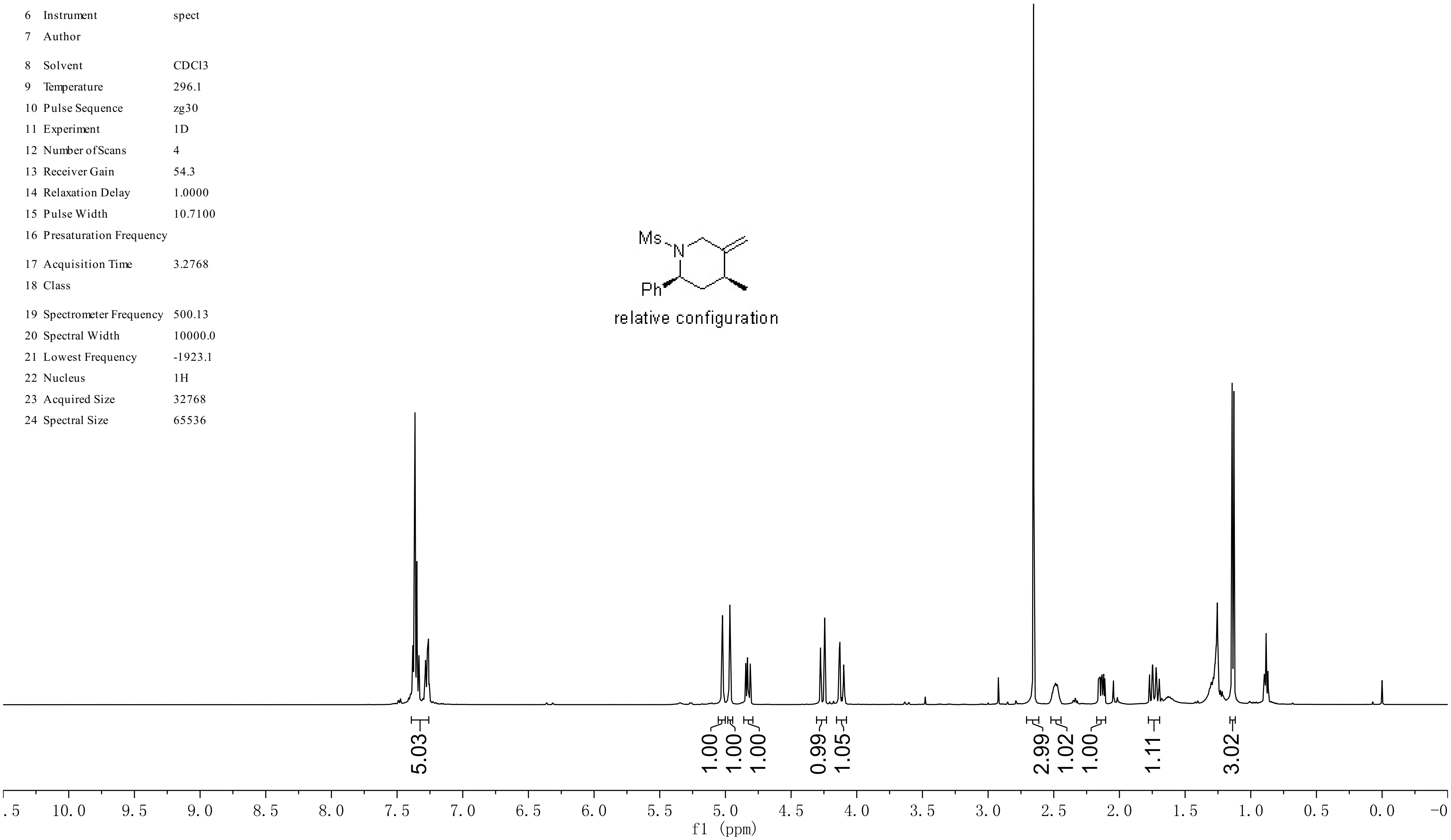

	Parameter	Value
1	Title	xfy-0614-4-s1-dMs.12.fid
2	Comment	
3	Origin	Bruker BioSpin GmbH
4	Owner	nmr
5	Site	
6	Instrument	spect
7	Author	
8	Solvent	CDCl3
9	Temperature	296.1
10	Pulse Sequence	zgpg30
11	Experiment	1D
12	Number of Scans	80
13	Receiver Gain	193.1
14	Relaxation Delay	2.0000
15	Pulse Width	9.6000
16	Presaturation Frequency	
17	Acquisition Time	1.1010
18	Class	
19	Spectrometer Frequency	125.77
20	Spectral Width	29761.9
21	Lowest Frequency	-2292.2
22	Nucleus	13C
23	Acquired Size	32768
24	Spectral Size	65536

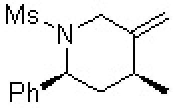

relative configuration

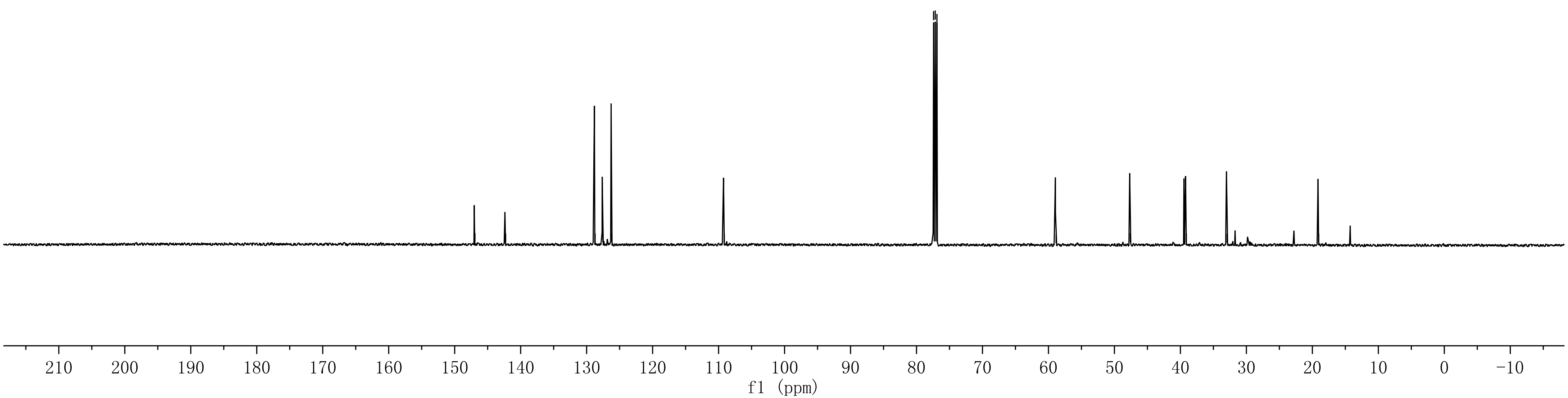

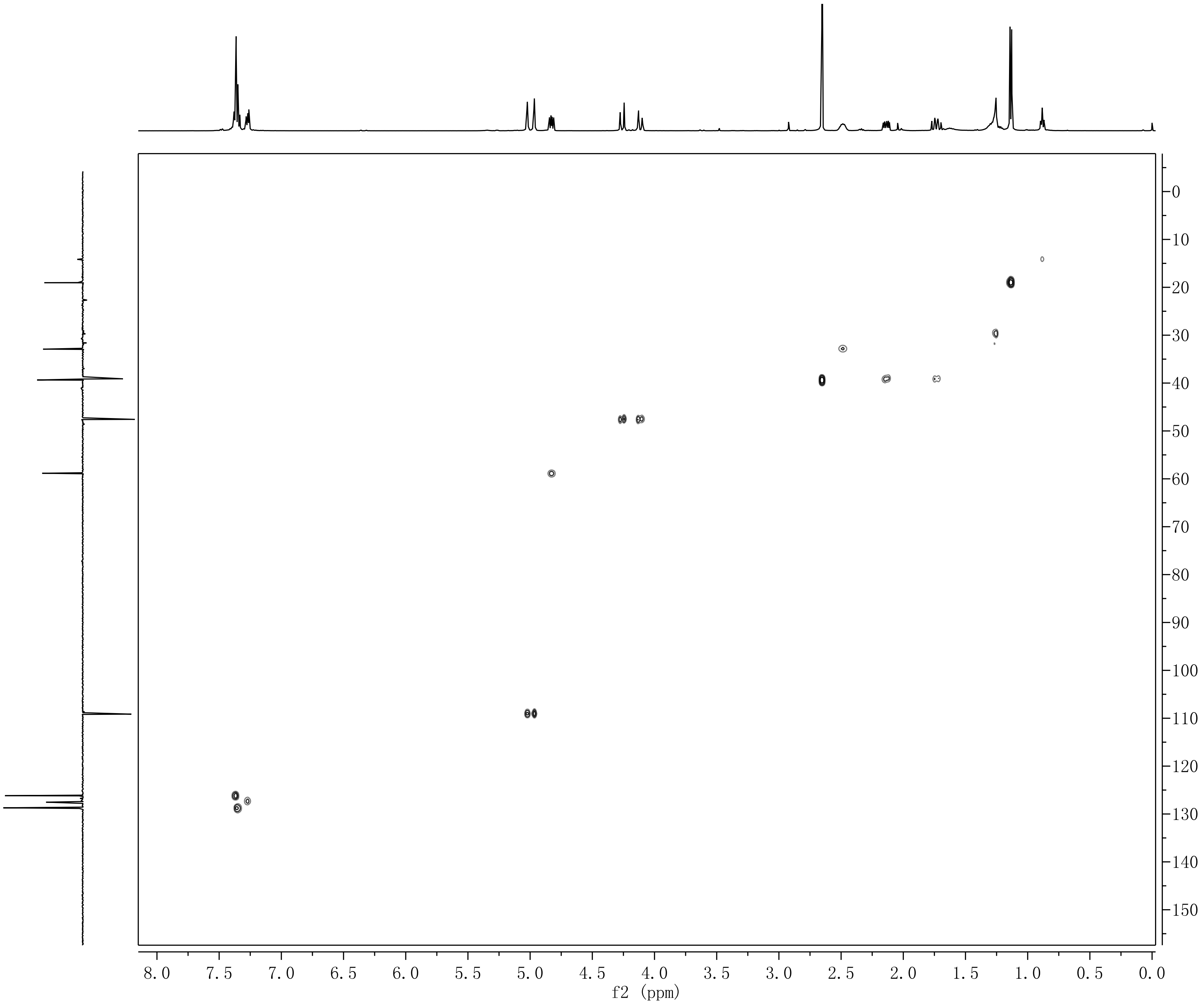

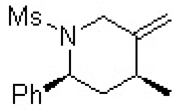

relative configuration

Parameter	Value
Title	xfy-0614-4-s1-dMs.14.ser
Comment	
Origin	Bruker BioSpin GmbH
Owner	nmr
Site	
Instrument	spect
Solvent	CDCl3
Temperature	296.2
Pulse Sequence	hsqcetgp
Experiment	HSQC
Number of Scans	2
Receiver Gain	193.1
Relaxation Delay	1.4539
Pulse Width	10.7100
Presaturation Frequency	
Acquisition Time	0.1249
Class	
Spectrometer Frequency	(500.13, 125.77)
Spectral Width	(4098.4, 20833.3)
Lowest Frequency	(-22.2, -1037.0)
Nucleus	(1H, 13C)
Acquired Size	(512, 256)
Spectral Size	(512, 512)

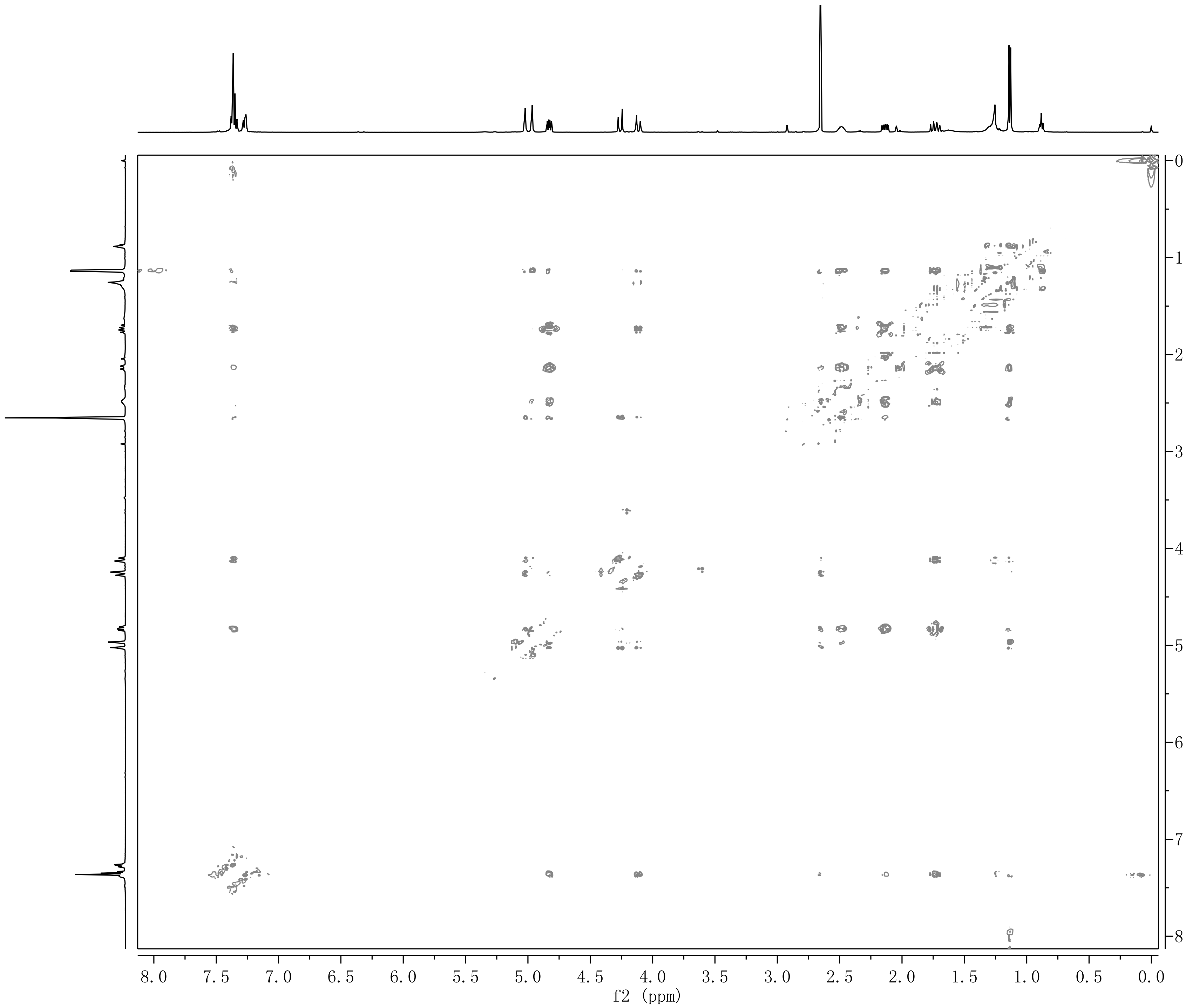

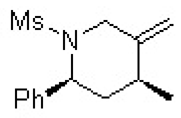

relative configuration

	Parameter	Value
1	Title	xfy-0614-4-s1-dMs.15.ser
2	Comment	
3	Origin	Bruker BioSpin GmbH
4	Owner	nmr
5	Site	
6	Instrument	spect
7	Author	
8	Solvent	CDCl3
9	Temperature	296.2
10	Pulse Sequence	noesygpphpp
11	Experiment	NOESY
12	Number of Scans	4
13	Receiver Gain	27.5
14	Relaxation Delay	1.9549
15	Pulse Width	10.7100
16	Presaturation Frequency	
17	Acquisition Time	0.2499
18	Class	
19	Spectrometer Frequency	(500.13, 500.13)
20	Spectral Width	(4098.4, 4098.4)
21	Lowest Frequency	(-32.4, -32.4)
22	Nucleus	(1H, 1H)
23	Acquired Size	(1024, 256)
24	Spectral Size	(1024, 1024)

	Parameter	Value
1	Title	xfy-0617-41-s1.11.fid
2	Comment	
3	Origin	Bruker BioSpin GmbH
4	Owner	nmr
5	Site	
6	Instrument	spect
7	Author	
8	Solvent	CDCl3
9	Temperature	296.1
10	Pulse Sequence	zg30
11	Experiment	1D
12	Number of Scans	4
13	Receiver Gain	54.3
14	Relaxation Delay	1.0000
15	Pulse Width	10.7100
16	Presaturation Frequency	
17	Acquisition Time	3.2768
18	Class	
19	Spectrometer Frequency	500.13
20	Spectral Width	10000.0
21	Lowest Frequency	-1922.7
22	Nucleus	1H
23	Acquired Size	32768
24	Spectral Size	65536

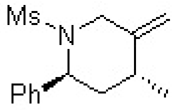

relative configuration

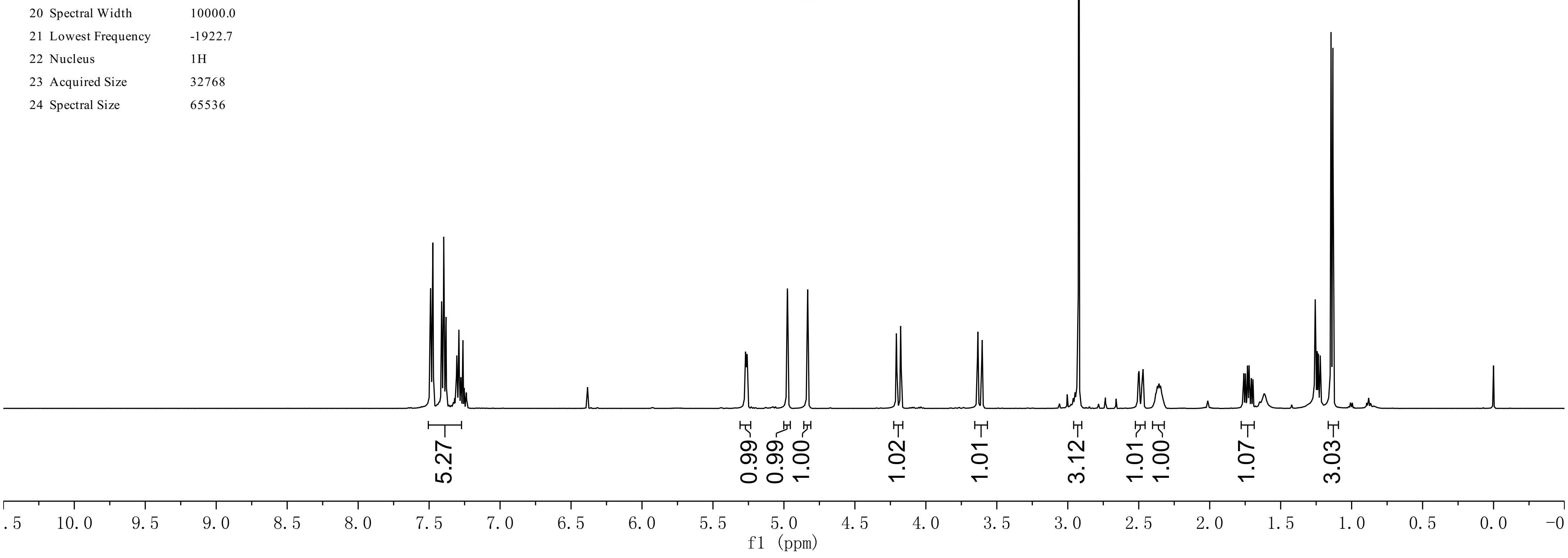

	Parameter	Value
1	Title	xfy-0617-41-s1.12.fid
2	Comment	
3	Origin	Bruker BioSpin GmbH
4	Owner	nmr
5	Site	
6	Instrument	spect
7	Author	
8	Solvent	CDCl3
9	Temperature	296.1
10	Pulse Sequence	zgpg30
11	Experiment	1D
12	Number of Scans	40
13	Receiver Gain	193.1
14	Relaxation Delay	2.0000
15	Pulse Width	9.6000
16	Presaturation Frequency	
17	Acquisition Time	1.1010
18	Class	
19	Spectrometer Frequency	125.77
20	Spectral Width	29761.9
21	Lowest Frequency	-2292.1
22	Nucleus	13C
23	Acquired Size	32768
24	Spectral Size	65536

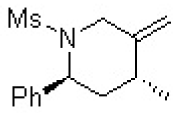

relative configuration

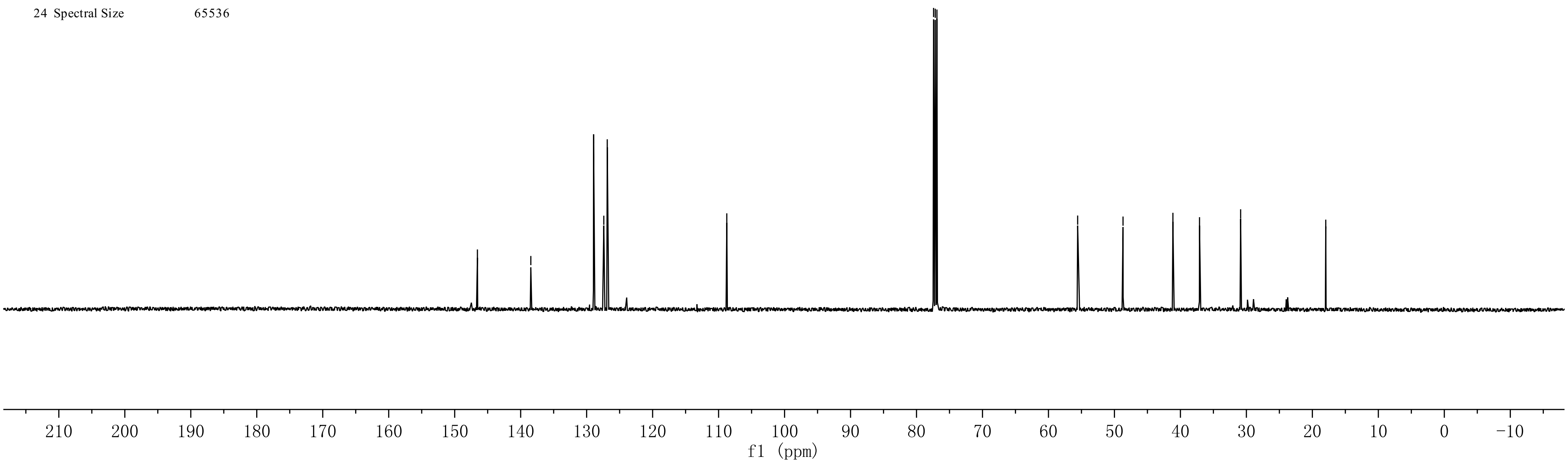

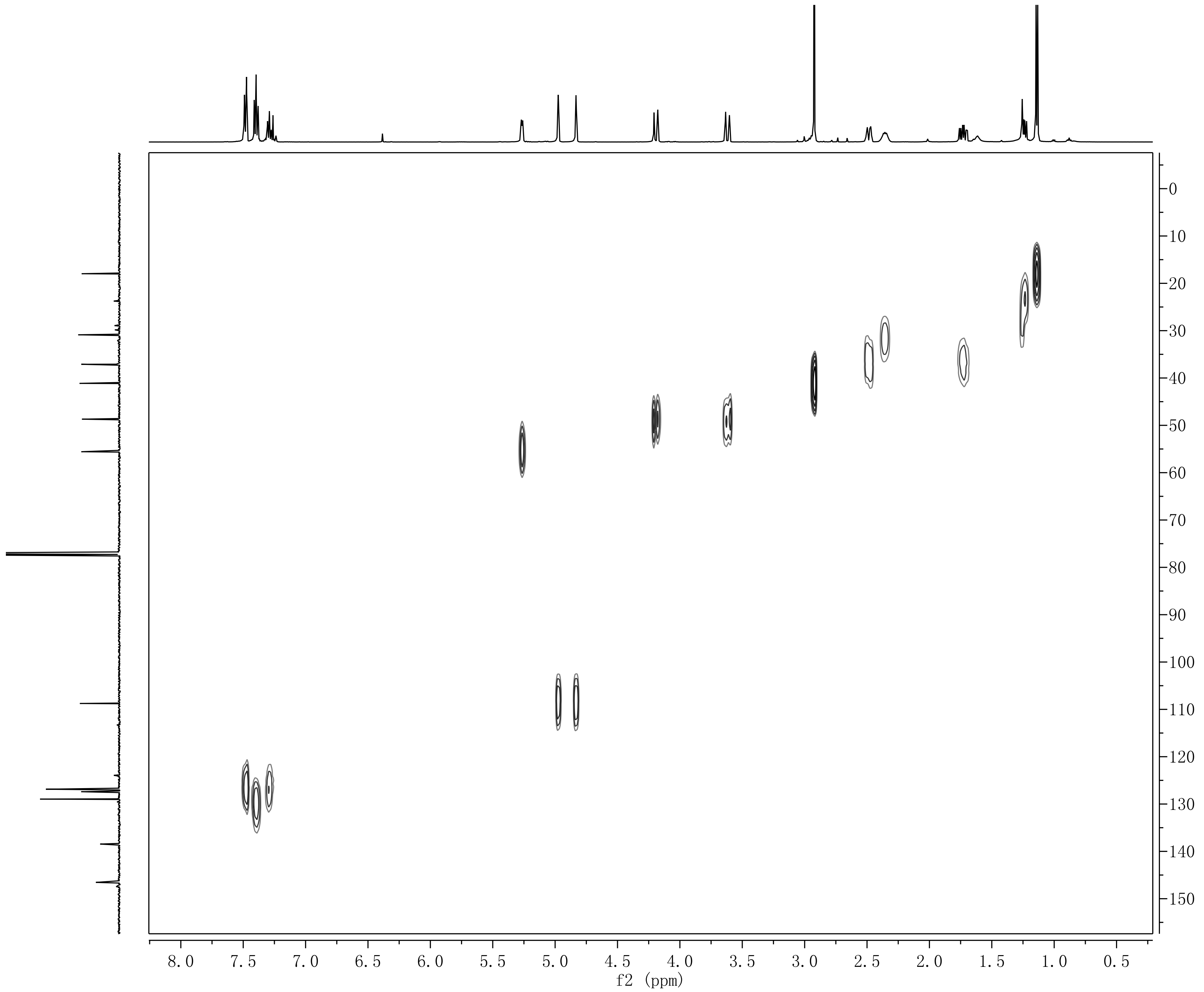

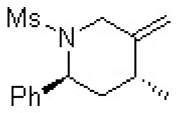

relative configuration

Parameter	Value
Title	xfy-0617-41-s1.16.ser
Comment	
Origin	Bruker BioSpin GmbH
Owner	nmr
Site	
Instrument	spect
Solvent	CDCl3
Temperature	296.1
Pulse Sequence	hsqcetgp
Experiment	HSQC
Number of Scans	2
Receiver Gain	193.1
Relaxation Delay	1.4519
Pulse Width	10.7100
Presaturation Frequency	
Acquisition Time	0.1270
Class	
Spectrometer Frequency	(500.13, 125.77)
Spectral Width	(4032.3, 20833.3)
Lowest Frequency	(97.0, -1037.0)
Nucleus	(1H, 13C)
Acquired Size	(512, 45)
Spectral Size	(512, 256)

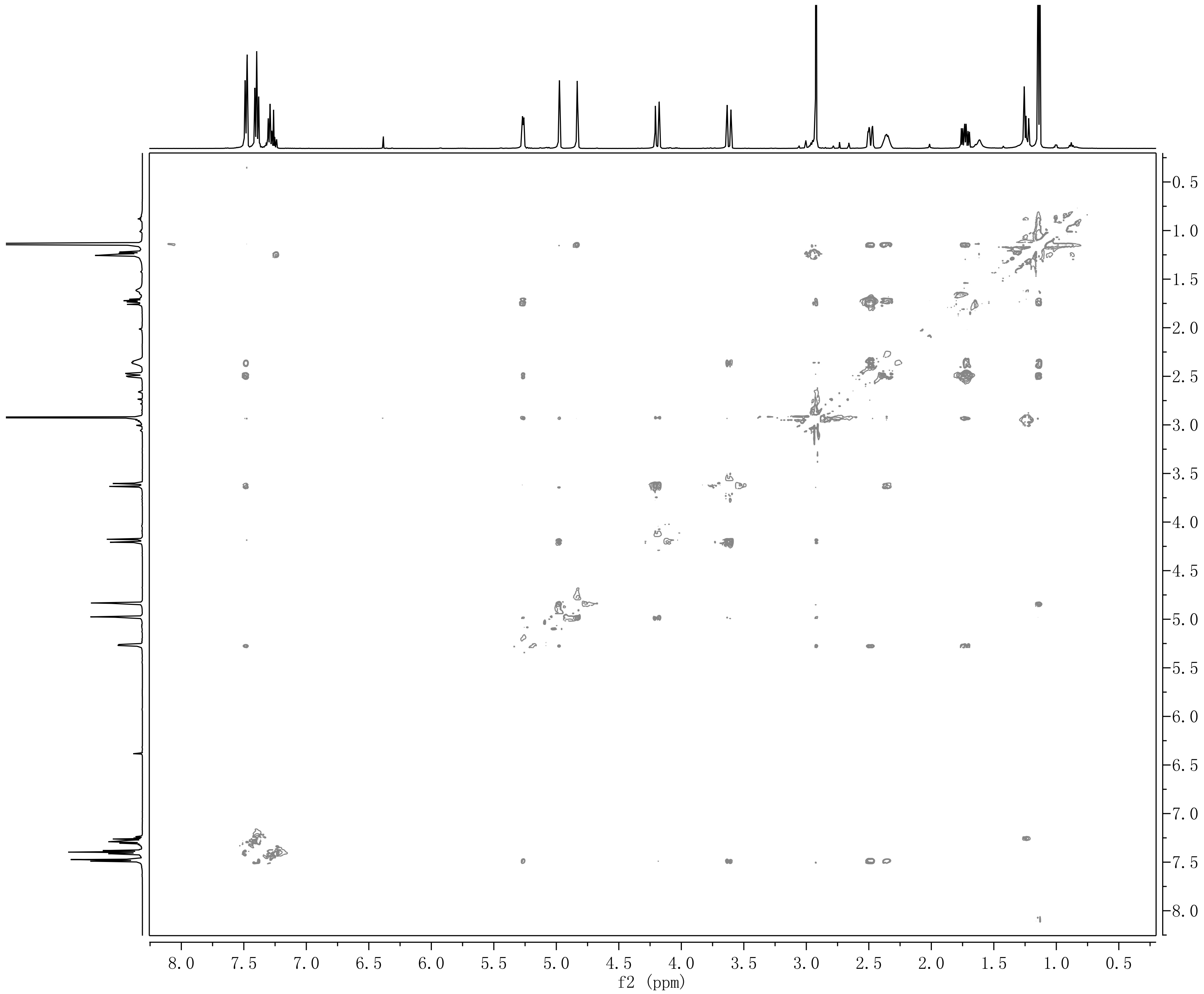

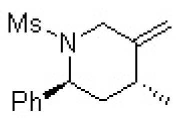

relative configuration

	Parameter	Value
1	Title	xfy-0617-41-s1.15.ser
2	Comment	
3	Origin	Bruker BioSpin GmbH
4	Owner	nmr
5	Site	
6	Instrument	spect
7	Author	
8	Solvent	CDCl3
9	Temperature	296.1
10	Pulse Sequence	noesygpphpp
11	Experiment	NOESY
12	Number of Scans	4
13	Receiver Gain	27.5
14	Relaxation Delay	1.9508
15	Pulse Width	10.7100
16	Presaturation Frequency	
17	Acquisition Time	0.2540
18	Class	
19	Spectrometer Frequency	(500.13, 500.13)
20	Spectral Width	(4032.3, 4032.3)
21	Lowest Frequency	(97.0, 97.0)
22	Nucleus	(1H, 1H)
23	Acquired Size	(1024, 256)
24	Spectral Size	(1024, 1024)

Parameter	Value
Title	xfy-190810-2-s4.1.1.1r
Comment	
Origin	Bruker BioSpin GmbH
Owner	nmr
Site	
Instrument	spect
Solvent	CDCl3
Temperature	296.2
Pulse Sequence	zg30
Experiment	1D
Number of Scans	16
Receiver Gain	87.5
Relaxation Delay	1.0000
Pulse Width	10.7100
Presaturation Frequency	
Acquisition Time	3.2768
Acquisition Date	2019-08-10T14:25:57
Modification Date	2019-08-10T21:31:35
Spectrometer Frequency	500.13
Spectral Width	10000.0
Lowest Frequency	-1923.0
Nucleus	1H
Acquired Size	32768
Spectral Size	65536

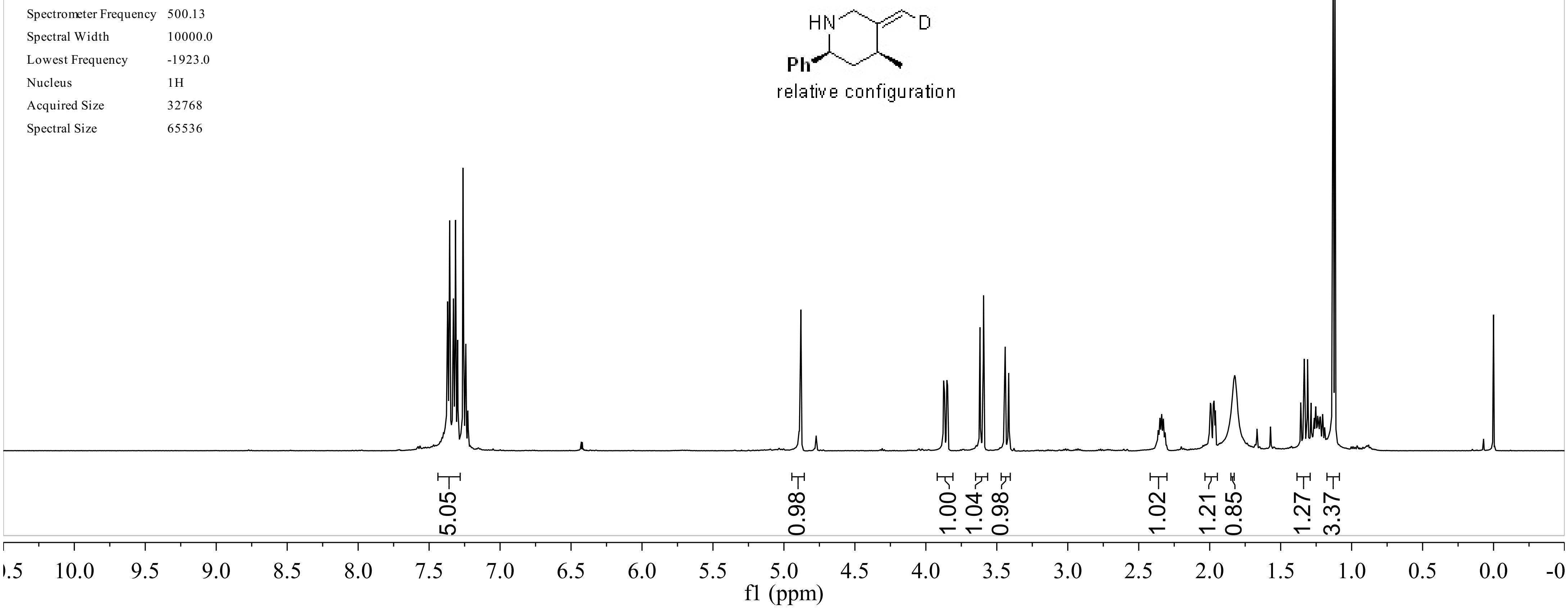

Parameter	Value
Title	xfy-190810-2-s4.2.fid
Comment	
Origin	Bruker BioSpin GmbH
Owner	nmr
Site	
Instrument	spect
Solvent	CDCl3
Temperature	296.2
Pulse Sequence	zgpg30
Experiment	1D
Number of Scans	200
Receiver Gain	193.1
Relaxation Delay	2.0000
Pulse Width	9.6000
Presaturation Frequency	
Acquisition Time	1.1010
Acquisition Date	2019-08-10T14:29:03
Modification Date	2019-08-10T21:31:38
Spectrometer Frequency	125.77
Spectral Width	29761.9
Lowest Frequency	-2289.5
Nucleus	13C
Acquired Size	32768
Spectral Size	65536

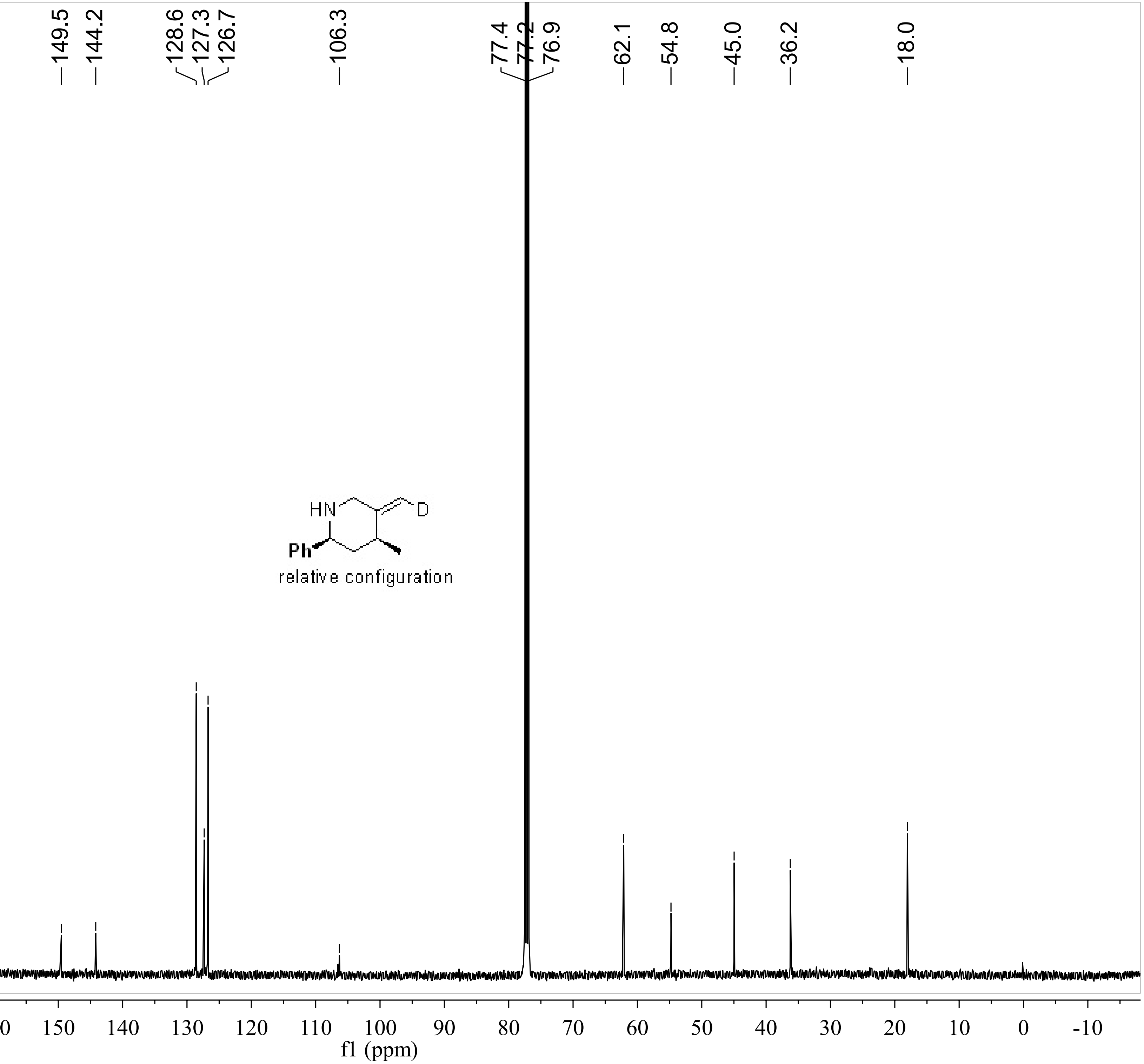

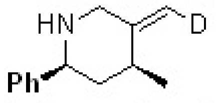

relative configuration

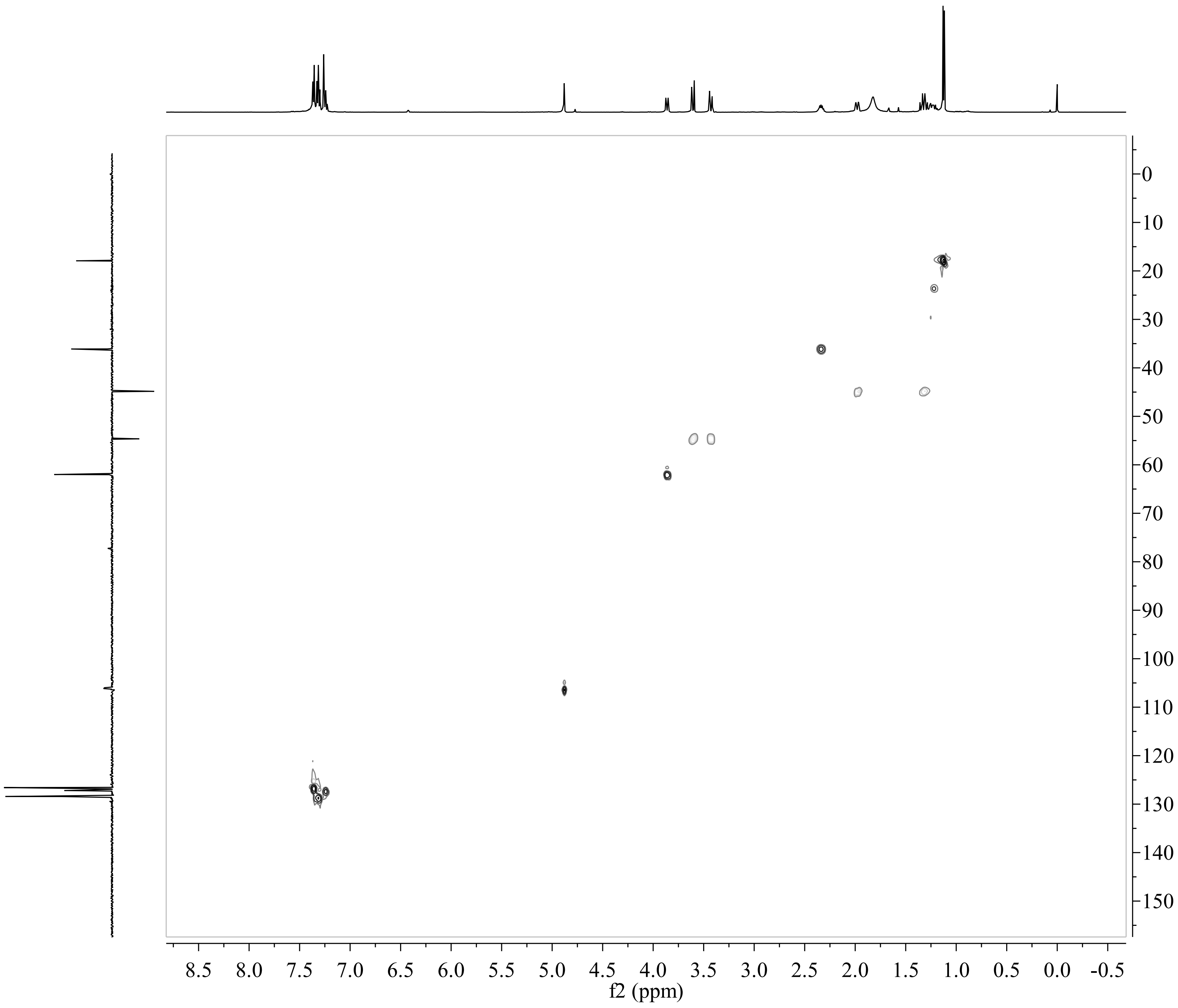

Parameter	Value
Title	xfy-190810-2-s4.3.ser
Comment	
Origin	Bruker BioSpin GmbH
Owner	nmr
Site	
Instrument	spect
Solvent	CDCl3
Temperature	296.1
Pulse Sequence	hsqcedetgp
Experiment	HSQC-EDITED
Number of Scans	2
Receiver Gain	193.1
Relaxation Delay	1.4713
Pulse Width	10.7100
Presaturation Frequency	
Acquisition Time	0.1075
Acquisition Date	2019-08-10T14:40:11
Modification Date	2019-08-10T21:31:38
Spectrometer Frequency	(500.13, 125.77)
Spectral Width	(4761.9, 20833.3)
Lowest Frequency	(-350.4, -1037.0)
Nucleus	(1H, 13C)
Acquired Size	(512, 256)
Spectral Size	(512, 512)

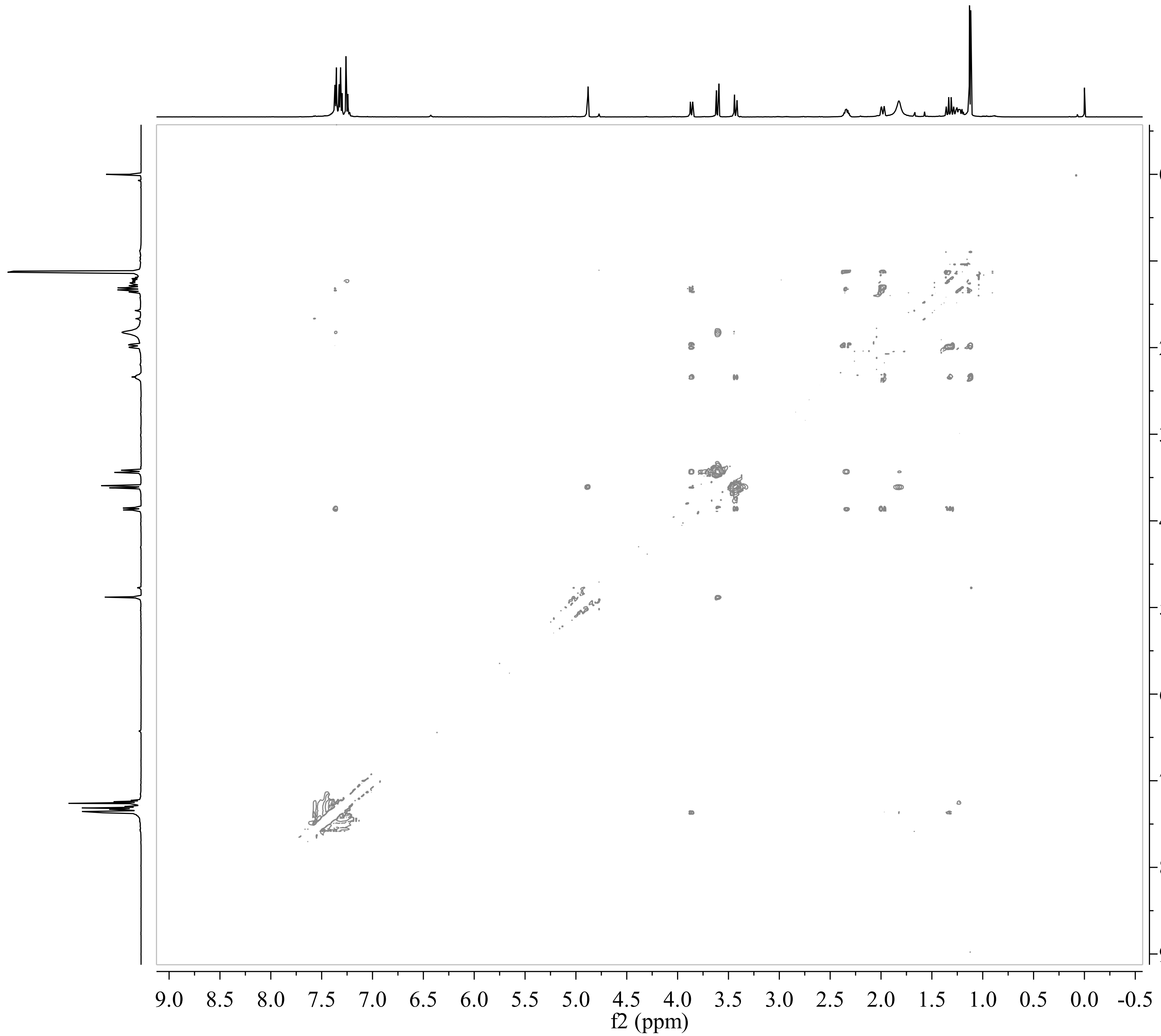

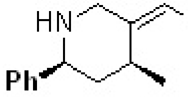

relative configuration

Parameter	Value
Title	xfy-190810-2-s4.12.ser
Comment	
Origin	Bruker BioSpin GmbH
Owner	nmr
Site	
Instrument	spect
Solvent	CDCl3
Temperature	296.1
Pulse Sequence	noesygpphpp
Experiment	NOESY
Number of Scans	4
Receiver Gain	31.1
Relaxation Delay	1.9939
Pulse Width	10.7100
Presaturation Frequency	
Acquisition Time	0.2109
Acquisition Date	2019-08-10T17:46:19
Modification Date	2019-08-10T21:31:36
Spectrometer Frequency	(500.13, 500.13)
Spectral Width	(4854.4, 4854.4)
Lowest Frequency	(-291.2, -291.2)
Nucleus	(1H, 1H)
Acquired Size	(1024, 256)
Spectral Size	(1024, 1024)

Parameter	Value
Title	xfy-190810-2-dMs.1.1.1r
Comment	
Origin	Bruker BioSpin GmbH
Owner	nmr
Site	
Instrument	spect
Solvent	CDCl3
Temperature	299.3
Pulse Sequence	zg30
Experiment	1D
Number of Scans	8
Receiver Gain	126.1
Relaxation Delay	1.0000
Pulse Width	8.7300
Presaturation Frequency	
Acquisition Time	1.9999
Acquisition Date	2019-08-10T20:34:37
Modification Date	2019-08-10T21:51:30
Spectrometer Frequency	400.13
Spectral Width	8012.8
Lowest Frequency	-1535.4
Nucleus	1H
Acquired Size	16025
Spectral Size	65536

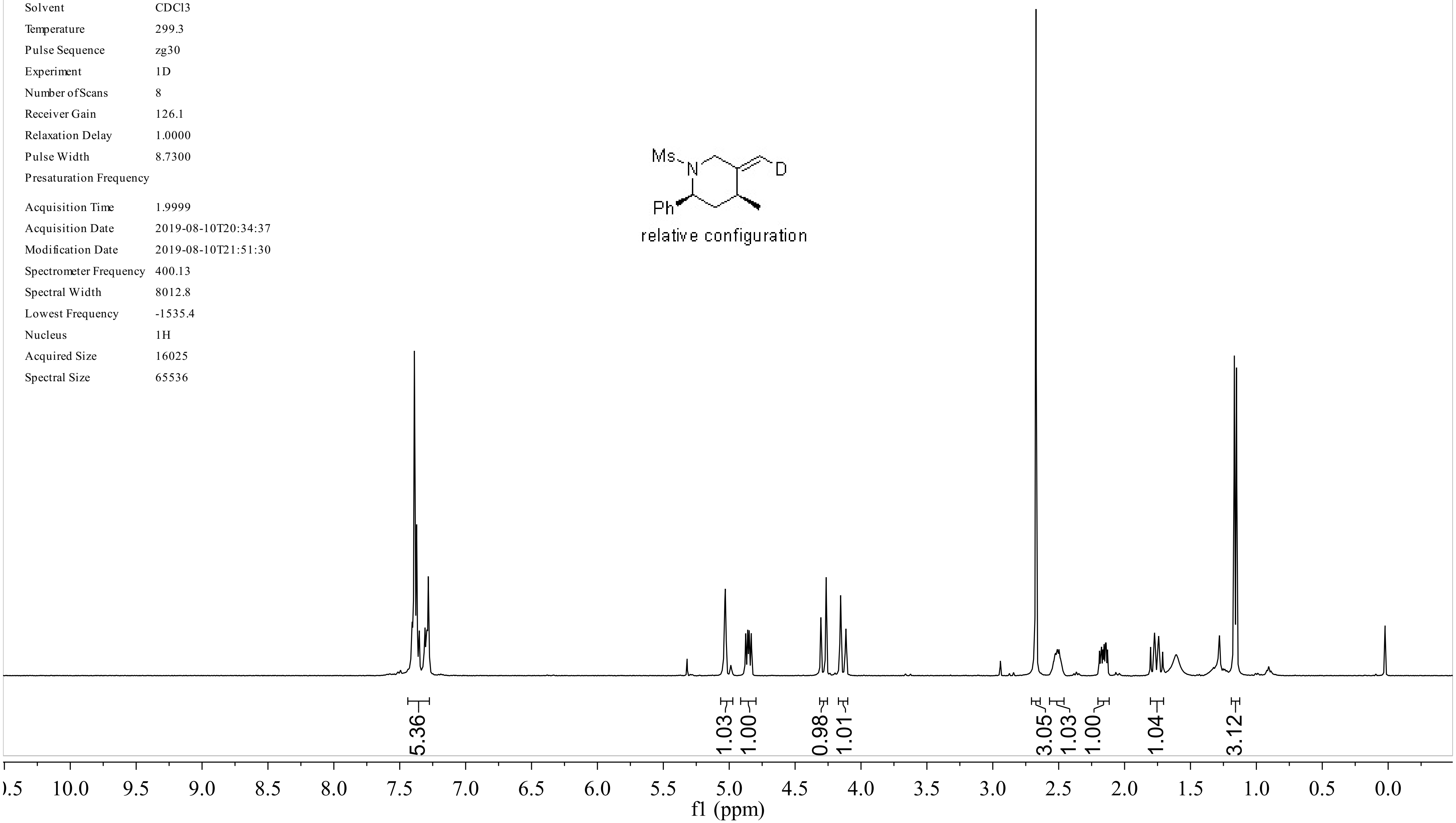

Parameter	Value
Title	xfy-190810-2-dMs.2.fid
Comment	
Origin	Bruker BioSpin GmbH
Owner	nmr
Site	
Instrument	spect
Solvent	CDCl3
Temperature	299.5
Pulse Sequence	zgpg30
Experiment	1D
Number of Scans	440
Receiver Gain	196.4
Relaxation Delay	2.0000
Pulse Width	10.0000
Presaturation Frequency	
Acquisition Time	1.3631
Acquisition Date	2019-08-10T20:36:41
Modification Date	2019-08-10T21:51:30
Spectrometer Frequency	100.62
Spectral Width	24038.5
Lowest Frequency	-1943.9
Nucleus	13C
Acquired Size	32768
Spectral Size	65536

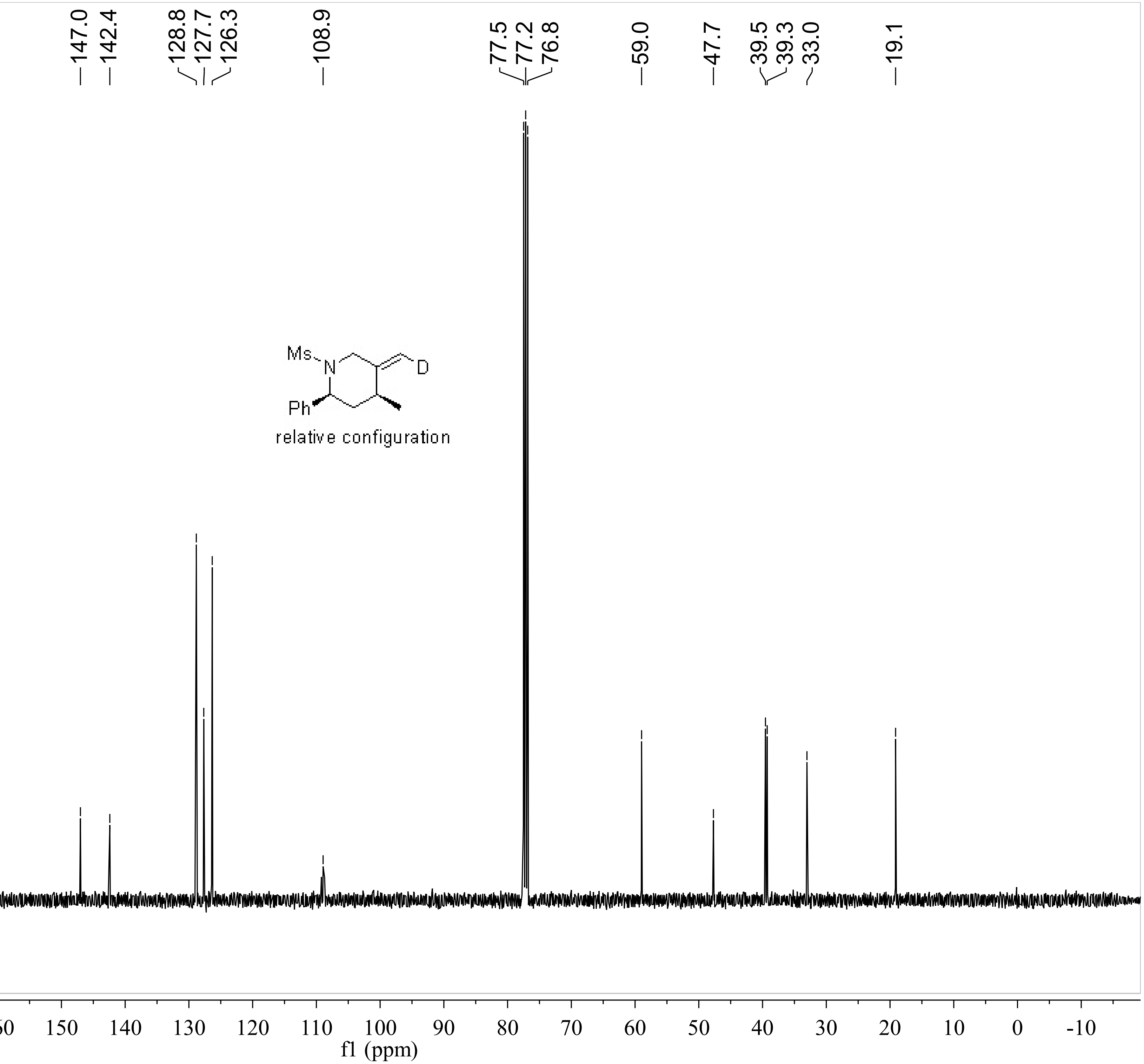

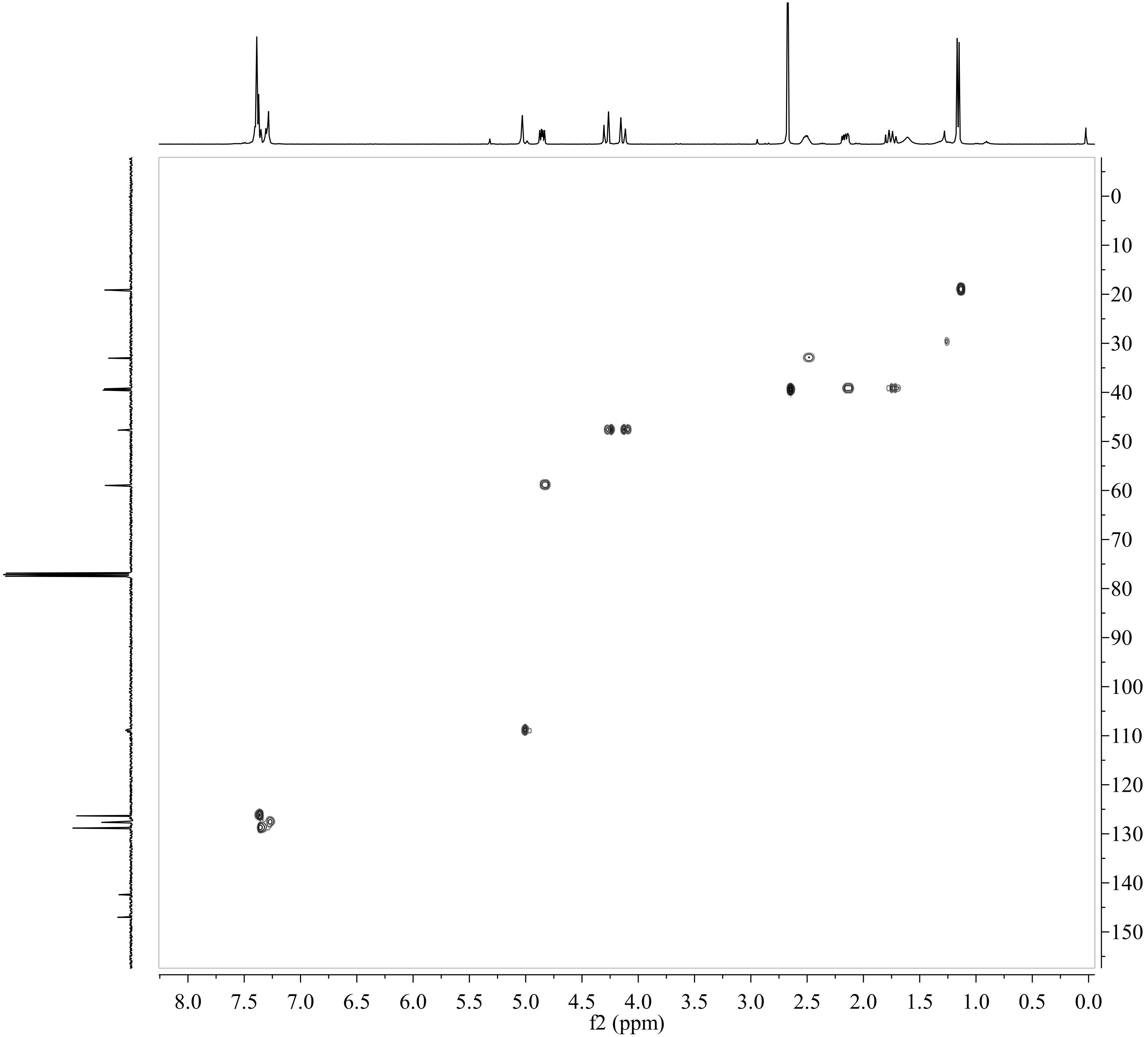

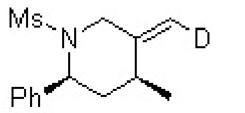

relative configuration

Parameter	Value
Title	xfy-190810-2-dMs.8.ser
Comment	
Origin	Bruker BioSpin GmbH
Owner	nmr
Site	
Instrument	spect
Solvent	CDCl3
Temperature	298.7
Pulse Sequence	hsqcetgp
Experiment	HSQC
Number of Scans	8
Receiver Gain	196.4
Relaxation Delay	1.4449
Pulse Width	8.7300
Presaturation Frequency	
Acquisition Time	0.1536
Acquisition Date	2019-08-11T07:56:58
Modification Date	2019-08-11T10:32:38
Spectrometer Frequency	(400.13, 100.62)
Spectral Width	(3333.3, 16666.7)
Lowest Frequency	(-28.9, -829.1)
Nucleus	(1H, 13C)
Acquired Size	(512, 256)
Spectral Size	(512, 512)

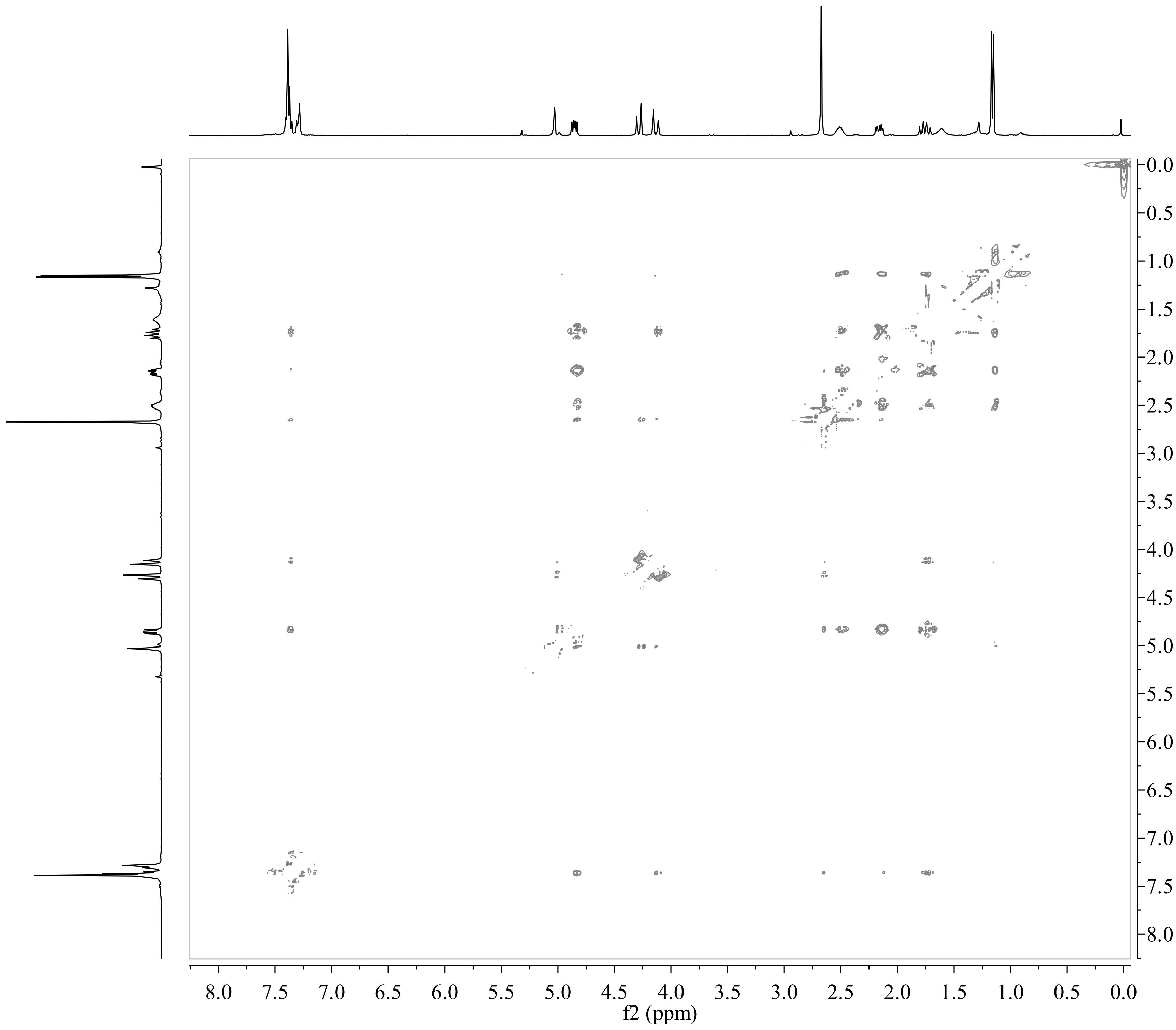

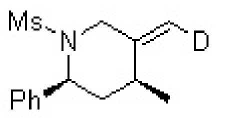

relative configuration

Parameter	Value
Title	xfy-190810-2-dMs.6.ser
Comment	
Origin	Bruker BioSpin GmbH
Owner	nmr
Site	
Instrument	spect
Solvent	CDCl3
Temperature	298.6
Pulse Sequence	noesygpphpp
Experiment	NOESY
Number of Scans	20
Receiver Gain	49.4
Relaxation Delay	1.9488
Pulse Width	8.7300
Presaturation Frequency	
Acquisition Time	0.3072
Acquisition Date	2019-08-11T03:57:06
Modification Date	2019-08-11T10:03:31
Spectrometer Frequency	(400.13, 400.13)
Spectral Width	(3333.3, 3333.3)
Lowest Frequency	(-28.9, -28.9)
Nucleus	(1H, 1H)
Acquired Size	(1024, 256)
Spectral Size	(1024, 1024)

Parameter	Value
Title	xfy-190804-3-s.21.1.1r
Comment	
Origin	Bruker BioSpin GmbH
Owner	nmr
Site	
Instrument	spect
Solvent	CDCl3
Temperature	297.1
Pulse Sequence	zg30
Experiment	1D
Number of Scans	8
Receiver Gain	173.6
Relaxation Delay	1.0000
Pulse Width	8.7300
Presaturation Frequency	
Acquisition Time	1.9999
Acquisition Date	2019-08-04T21:47:20
Modification Date	2019-08-09T17:08:36
Spectrometer Frequency	400.13
Spectral Width	8012.8
Lowest Frequency	-1545.0
Nucleus	1H
Acquired Size	16025
Spectral Size	65536

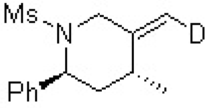

relative configuration

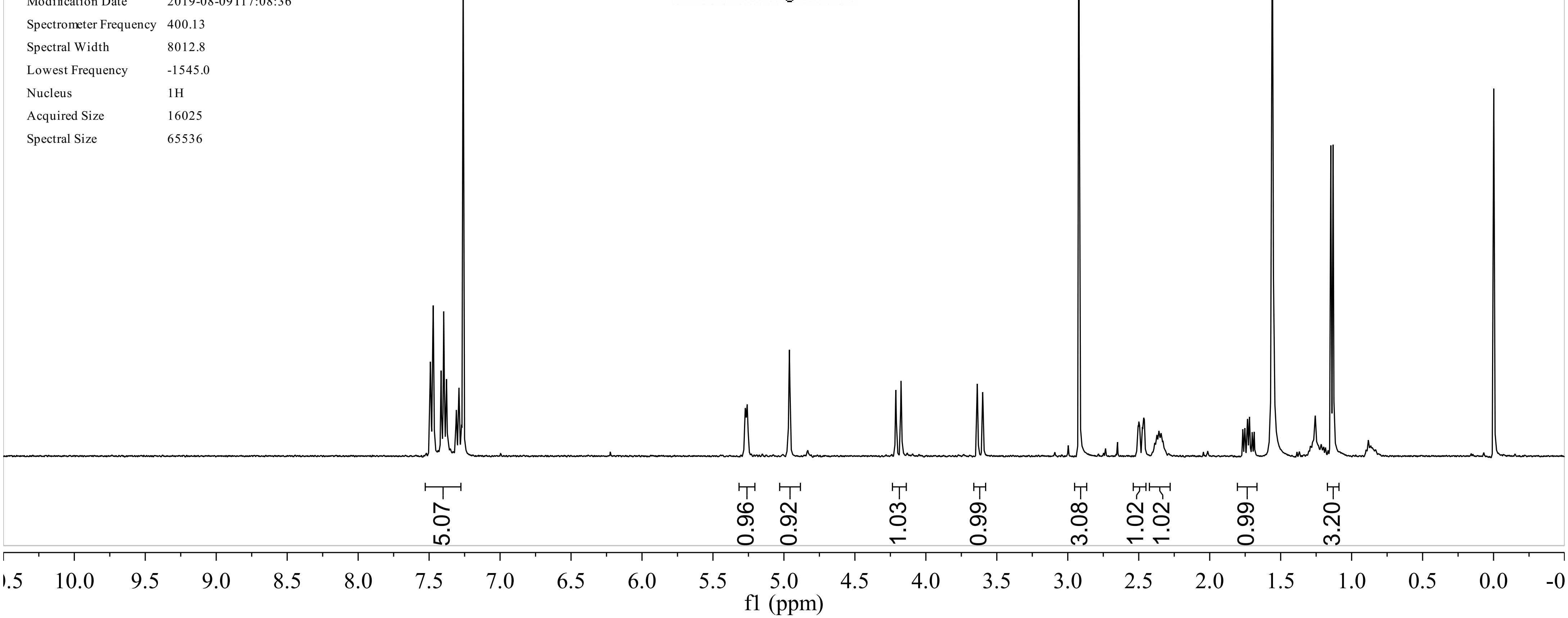

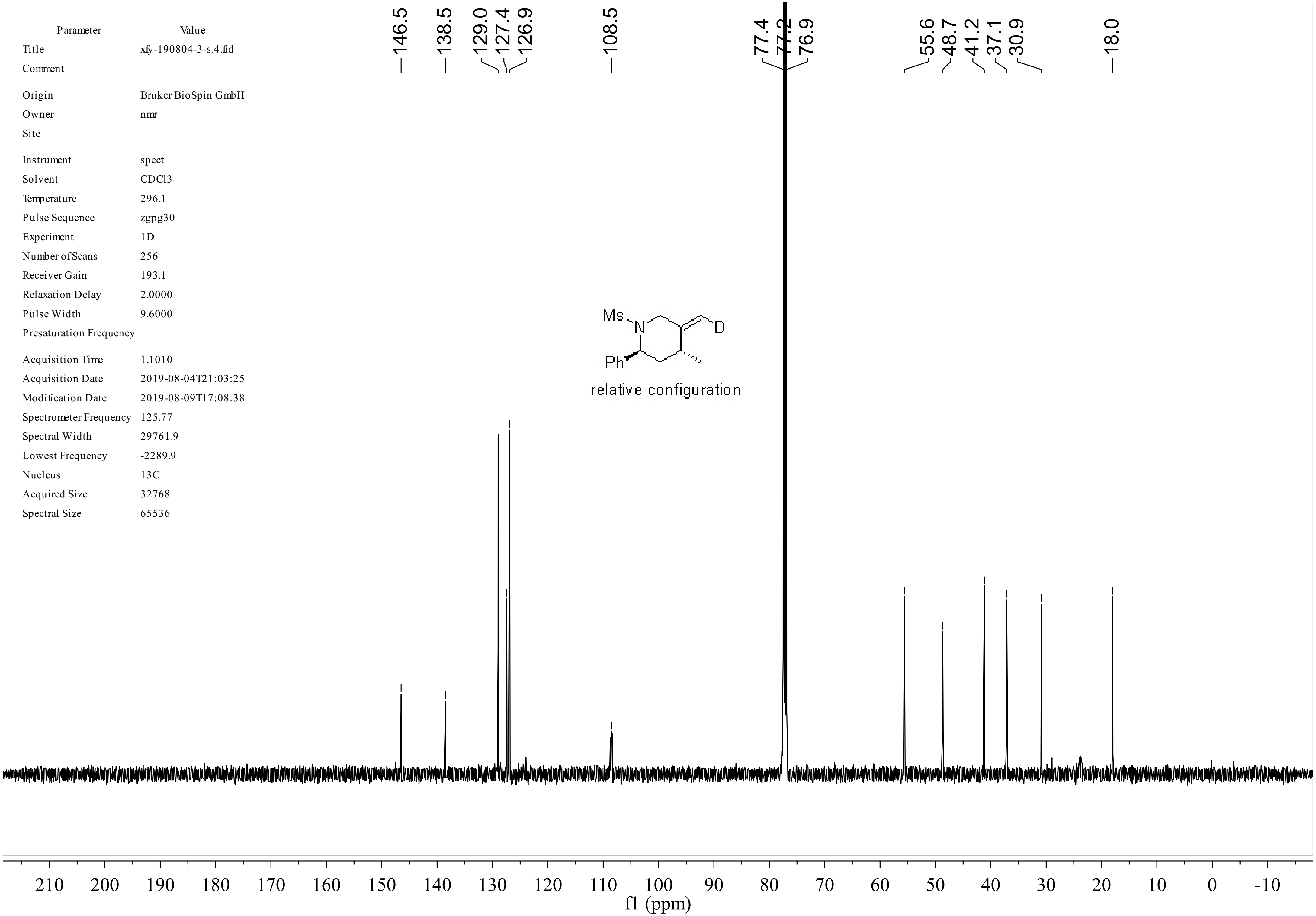
Parameter Value
Title xfy-190804-3-s.4.fid
Comment
Origin Bruker BioSpin GmbH
Owner nmr
Site
Instrument spect
Solvent CDCl3
Temperature 296.1
Pulse Sequence zgpg30
Experiment 1D
Number of Scans 256
Receiver Gain 193.1
Relaxation Delay 2.0000
Pulse Width 9.6000
Presaturation Frequency
Acquisition Time 1.1010
Acquisition Date 2019-08-04T21:03:25
Modification Date 2019-08-09T17:08:38
Spectrometer Frequency 125.77
Spectral Width 29761.9
Lowest Frequency -2289.9
Nucleus 13C
Acquired Size 32768
Spectral Size 65536
146.5
138.5
129.0
127.4
126.9
108.5
77.4
77.2
76.9
55.6
48.7
41.2
37.1
30.9
18.0
Ms
N
D
Ph
relative configuration
210 200 190 180 170 160 150 140 130 120 110 100 90 80 70 60 50 40 30 20 10 0 -10
f1 (ppm)

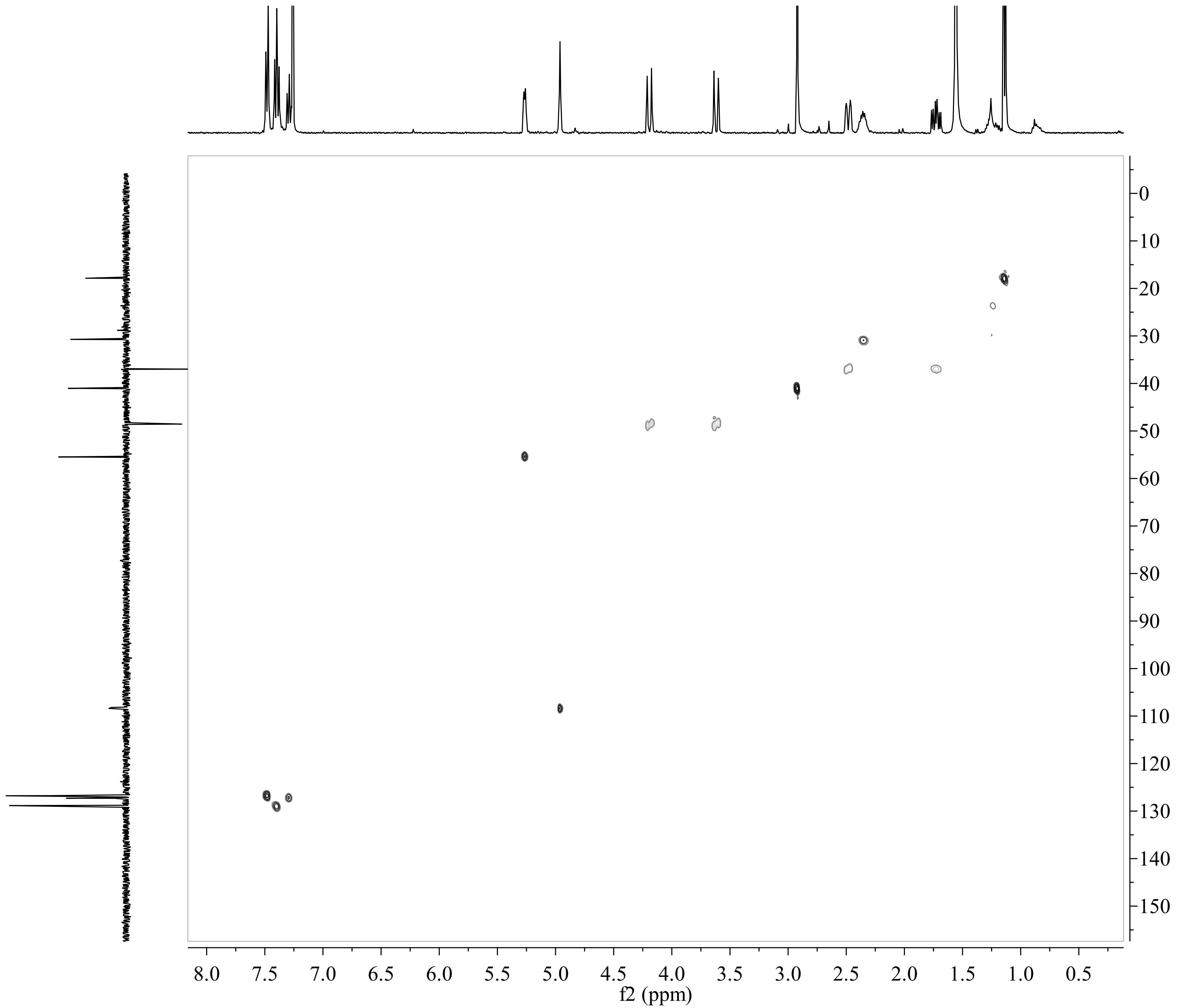

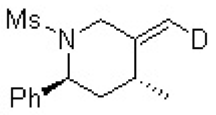

relative configuration

Parameter	Value
标题	xfy-190804-3-s.6.ser
Comment	
Origin	Bruker BioSpin GmbH
Owner	nmr
Site	
Instrument	spect
Author	
Solvent	CDCl3
Temperature	296.1
Pulse Sequence	hsqcedetgp
Experiment	HSQC-EDITED
Number of Scans	2
Receiver Gain	193.1
Relaxation Delay	1.4519
Pulse Width	10.7100
Presaturation Frequency	
Acquisition Time	0.1270
Class	
Spectrometer Frequency	(500.13, 125.77)
Spectral Width	(4032.3, 20833.3)
Lowest Frequency	(49.8, -1037.0)
Nucleus	(1H, 13C)
Acquired Size	(512, 256)
Spectral Size	(512, 512)

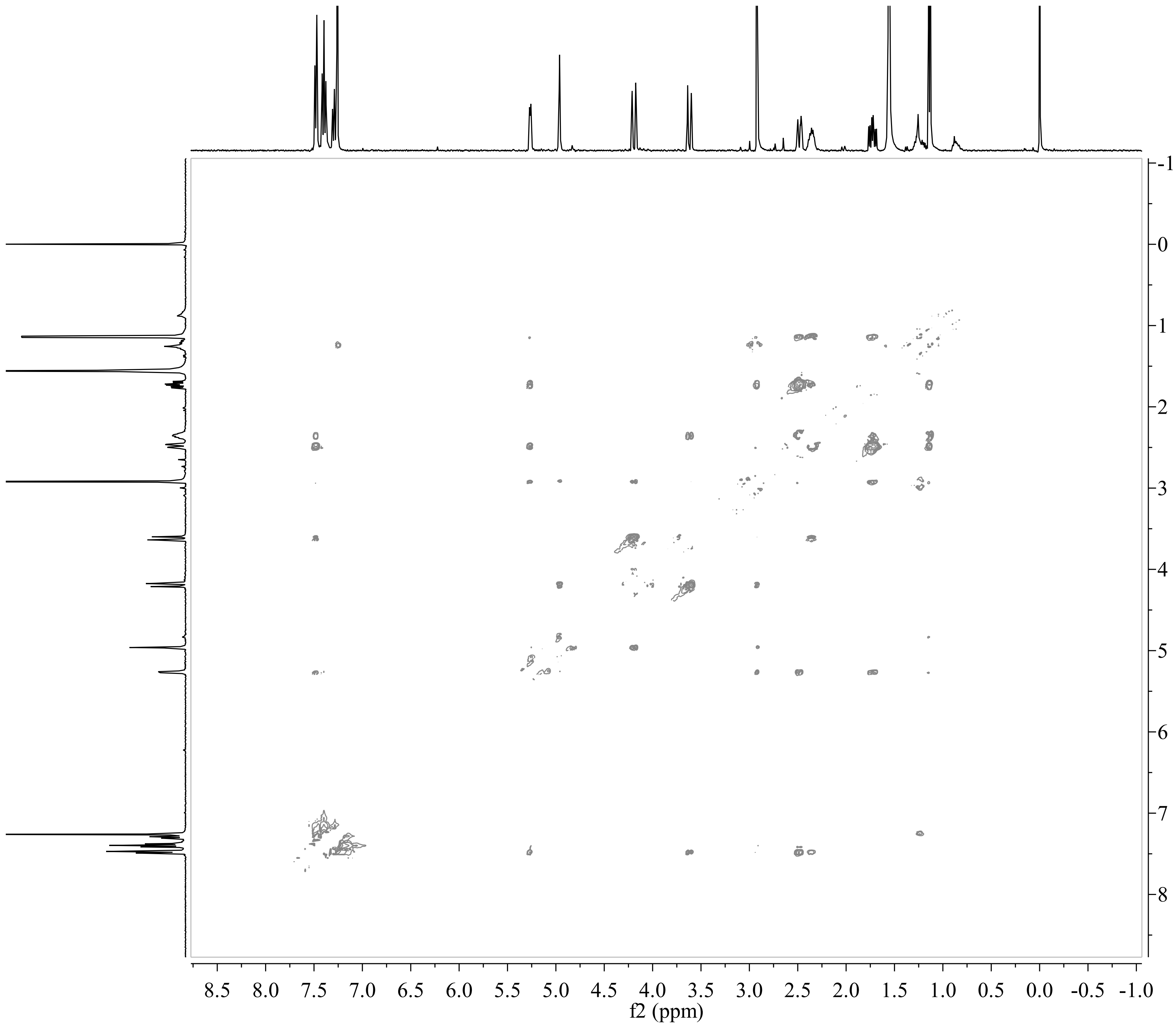

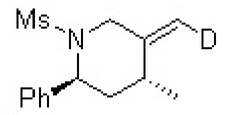

relative configuration

Parameter	Value
Title	xfy-190804-3-s.44.ser
Comment	
Origin	Bruker BioSpin GmbH
Owner	nmr
Site	
Instrument	spect
Solvent	CDCl3
Temperature	297.1
Pulse Sequence	noesygpphpp
Experiment	NOESY
Number of Scans	24
Receiver Gain	34.9
Relaxation Delay	1.9959
Pulse Width	8.7300
Presaturation Frequency	
Acquisition Time	0.2601
Acquisition Date	2019-08-04T23:46:38
Modification Date	2019-08-09T17:08:40
Spectrometer Frequency	(400.13, 400.13)
Spectral Width	(3937.0, 3937.0)
Lowest Frequency	(-426.9, -426.9)
Nucleus	(1H, 1H)
Acquired Size	(1024, 256)
Spectral Size	(1024, 1024)

Parameter	Value
Title	xfy-190614-1-s.11.fid
Comment	
Origin	Bruker BioSpin GmbH
Owner	nmr
Site	
Instrument	spect
Solvent	CDCl3
Temperature	296.2
Pulse Sequence	zg30
Experiment	1D
Number of Scans	8
Receiver Gain	54.3
Relaxation Delay	1.0000
Pulse Width	10.7100
Presaturation Frequency	
Acquisition Time	3.2768
Acquisition Date	2019-06-16T18:05:23
Modification Date	2019-06-16T18:44:19
Spectrometer Frequency	500.13
Spectral Width	10000.0
Lowest Frequency	-1923.6
Nucleus	1H
Acquired Size	32768
Spectral Size	65536

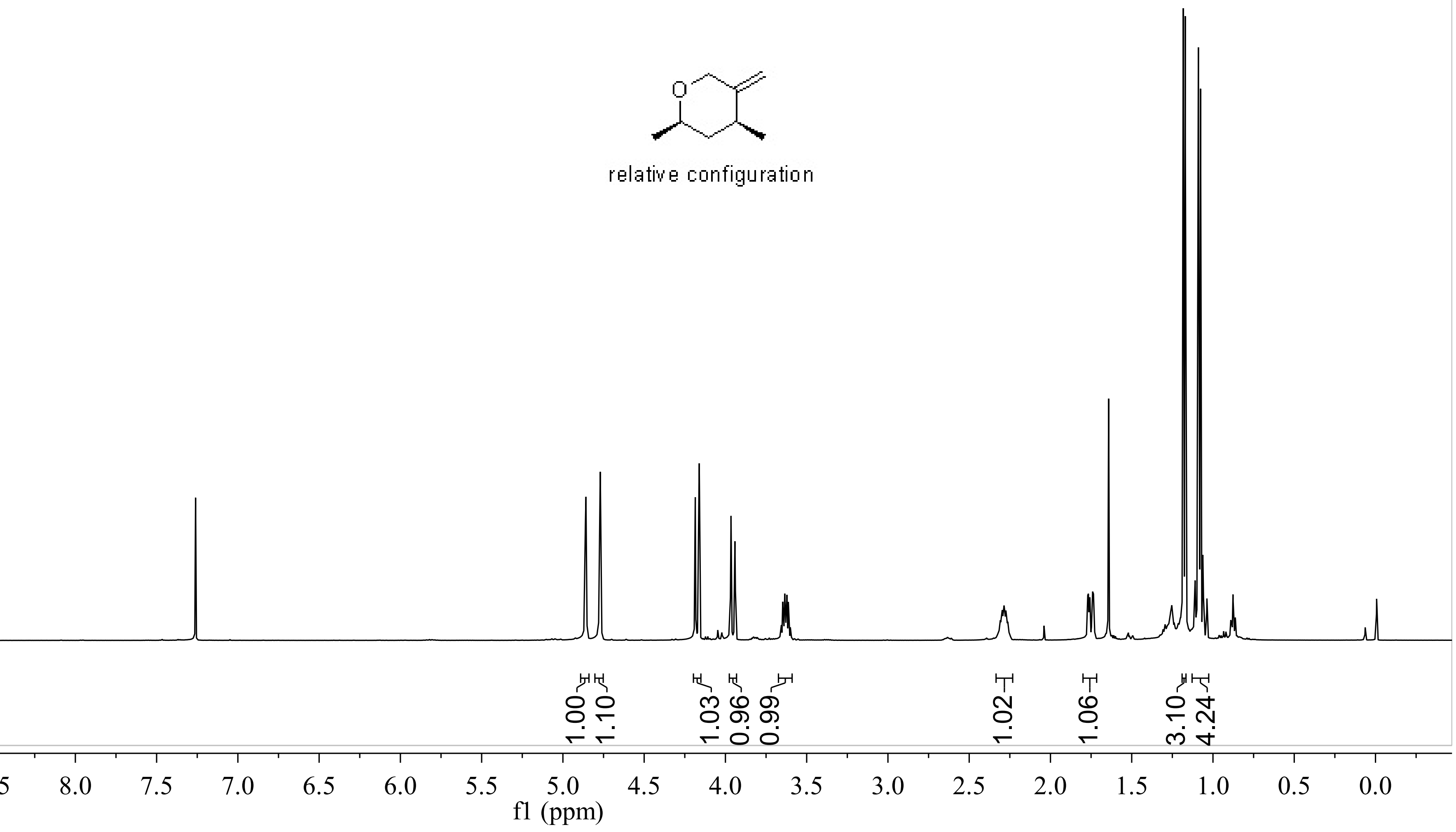

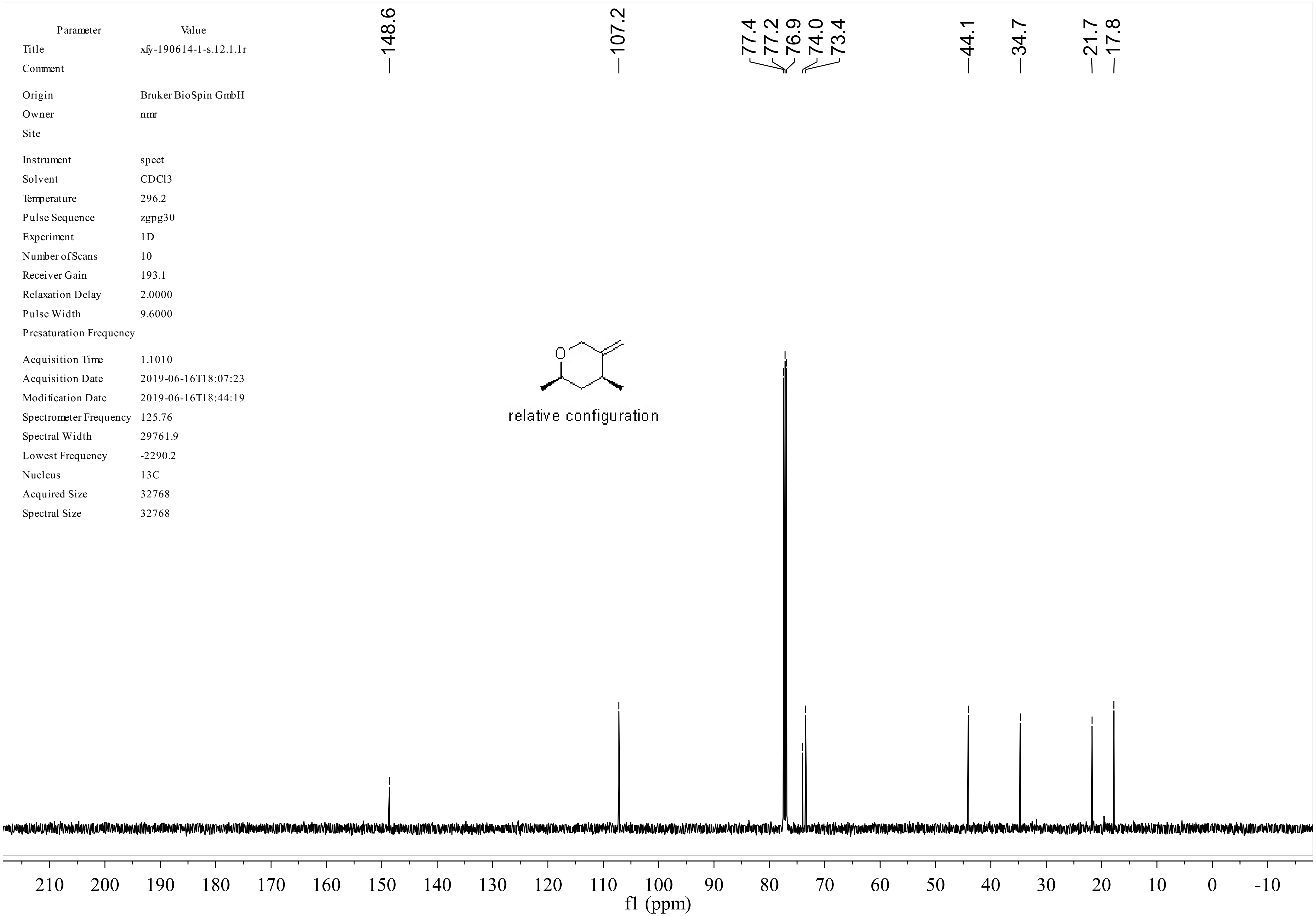
Parameter Value
Title xfy-190614-1-s.12.1.1r
Comment
Origin Bruker BioSpin GmbH
Owner nmr
Site
Instrument spect
Solvent CDCl3
Temperature 296.2
Pulse Sequence zgpg30
Experiment 1D
Number of Scans 10
Receiver Gain 193.1
Relaxation Delay 2.0000
Pulse Width 9.6000
Presaturation Frequency
Acquisition Time 1.1010
Acquisition Date 2019-06-16T18:07:23
Modification Date 2019-06-16T18:44:19
Spectrometer Frequency 125.76
Spectral Width 29761.9
Lowest Frequency -2290.2
Nucleus 13C
Acquired Size 32768
Spectral Size 32768
148.6
107.2
77.4
77.2
76.9
74.0
73.4
44.1
34.7
21.7
17.8
O
relative configuration
210 200 190 180 170 160 150 140 130 120 110 100 90 80 70 60 50 40 30 20 10 0 -10
f1 (ppm)

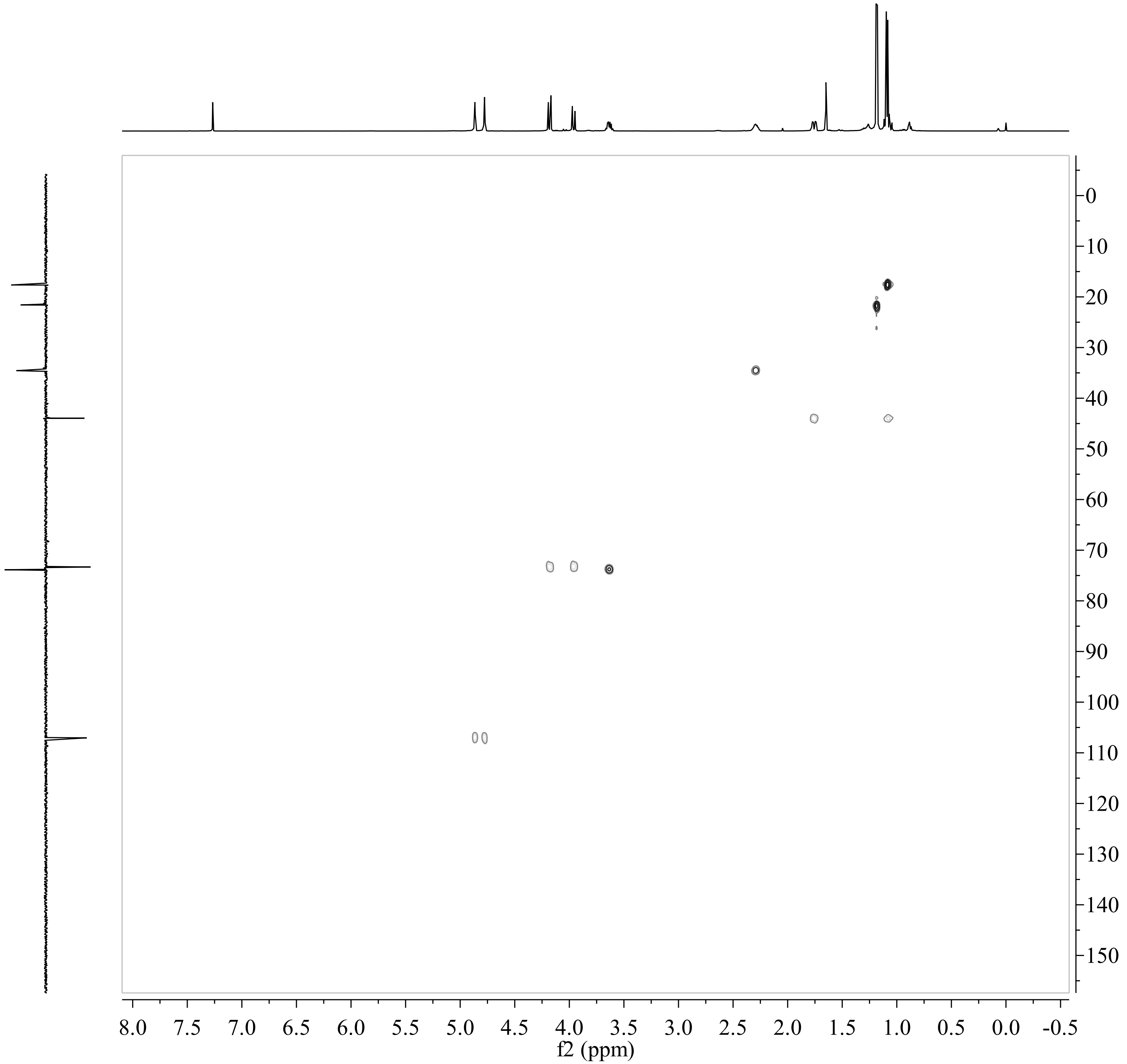

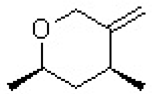

relative configuration

Parameter	Value
Title	xfy-190614-1-s.14.ser
Comment	
Origin	Bruker BioSpin GmbH
Owner	nmr
Site	
Instrument	spect
Solvent	CDCl3
Temperature	296.2
Pulse Sequence	hsqcedetgp
Experiment	HSQC-EDITED
Number of Scans	2
Receiver Gain	193.1
Relaxation Delay	1.4611
Pulse Width	10.7100
Presaturation Frequency	
Acquisition Time	0.1178
Acquisition Date	2019-06-16T18:10:57
Modification Date	2019-06-16T18:44:20
Spectrometer Frequency	(500.13, 125.77)
Spectral Width	(4347.8, 20833.3)
Lowest Frequency	(-298.1, -1037.0)
Nucleus	(1H, 13C)
Acquired Size	(512, 256)
Spectral Size	(512, 512)

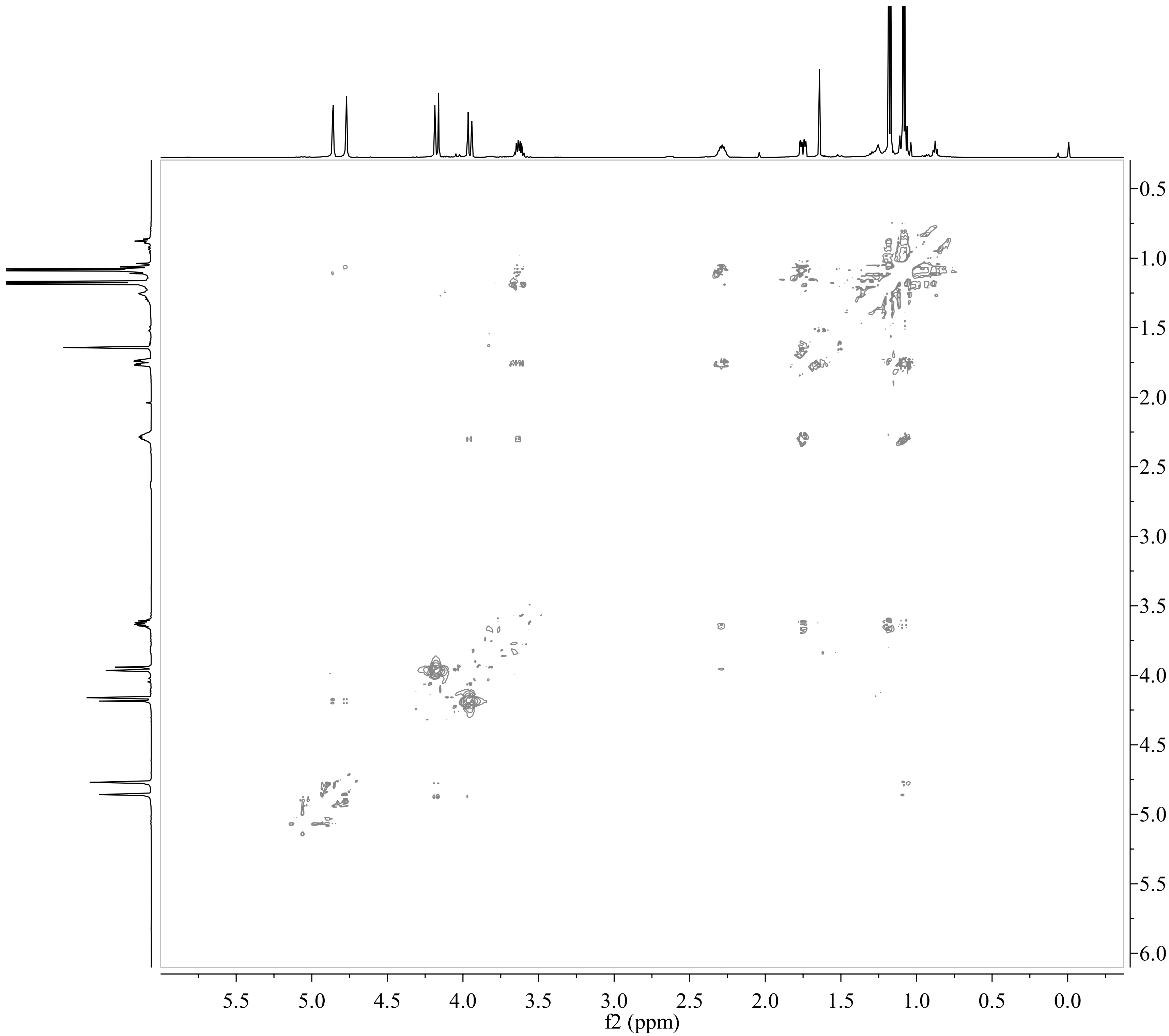

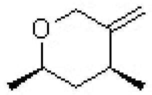

relative configuration

Parameter	Value
Title	xfy-190614-1-s.15.ser
Comment	
Origin	Bruker BioSpin GmbH
Owner	nmr
Site	
Instrument	spect
Solvent	CDCl3
Temperature	296.2
Pulse Sequence	noesygpphpp
Experiment	NOESY
Number of Scans	4
Receiver Gain	48.5
Relaxation Delay	1.9693
Pulse Width	10.7100
Presaturation Frequency	
Acquisition Time	0.2355
Acquisition Date	2019-06-16T18:27:03
Modification Date	2019-06-17T18:14:30
Spectrometer Frequency	(500.13, 500.13)
Spectral Width	(4347.8, 4347.8)
Lowest Frequency	(-298.1, -298.1)
Nucleus	(1H, 1H)
Acquired Size	(1024, 256)
Spectral Size	(1024, 1024)

Parameter	Value
Title	xfy-190602-3-s.1.1.1r
Comment	
Origin	Bruker BioSpin GmbH
Owner	nmr
Site	
Instrument	spect
Solvent	CDCl3
Temperature	296.1
Pulse Sequence	zg30
Experiment	1D
Number of Scans	4
Receiver Gain	54.3
Relaxation Delay	1.0000
Pulse Width	10.7100
Presaturation Frequency	
Acquisition Time	3.2768
Acquisition Date	2019-06-04T18:06:45
Modification Date	2019-06-04T19:04:11
Spectrometer Frequency	500.13
Spectral Width	10000.0
Lowest Frequency	-1911.5
Nucleus	1H
Acquired Size	32768
Spectral Size	65536

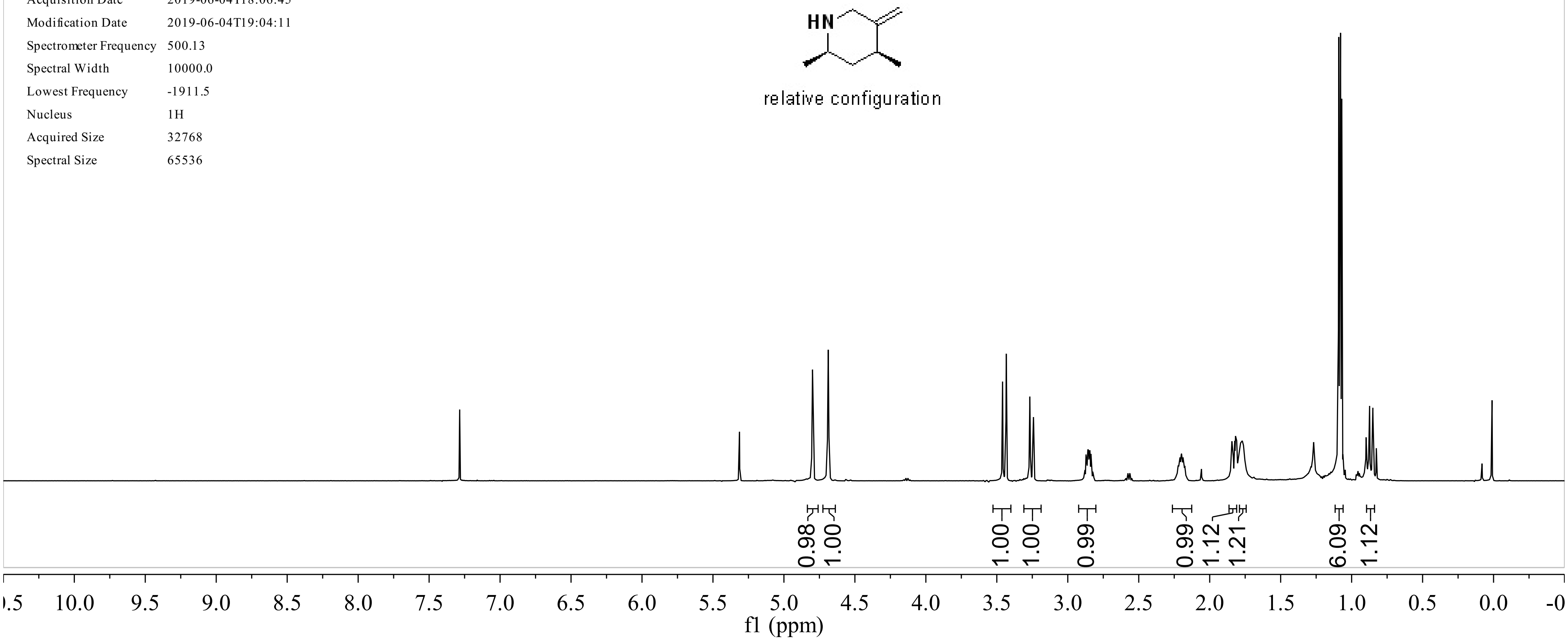

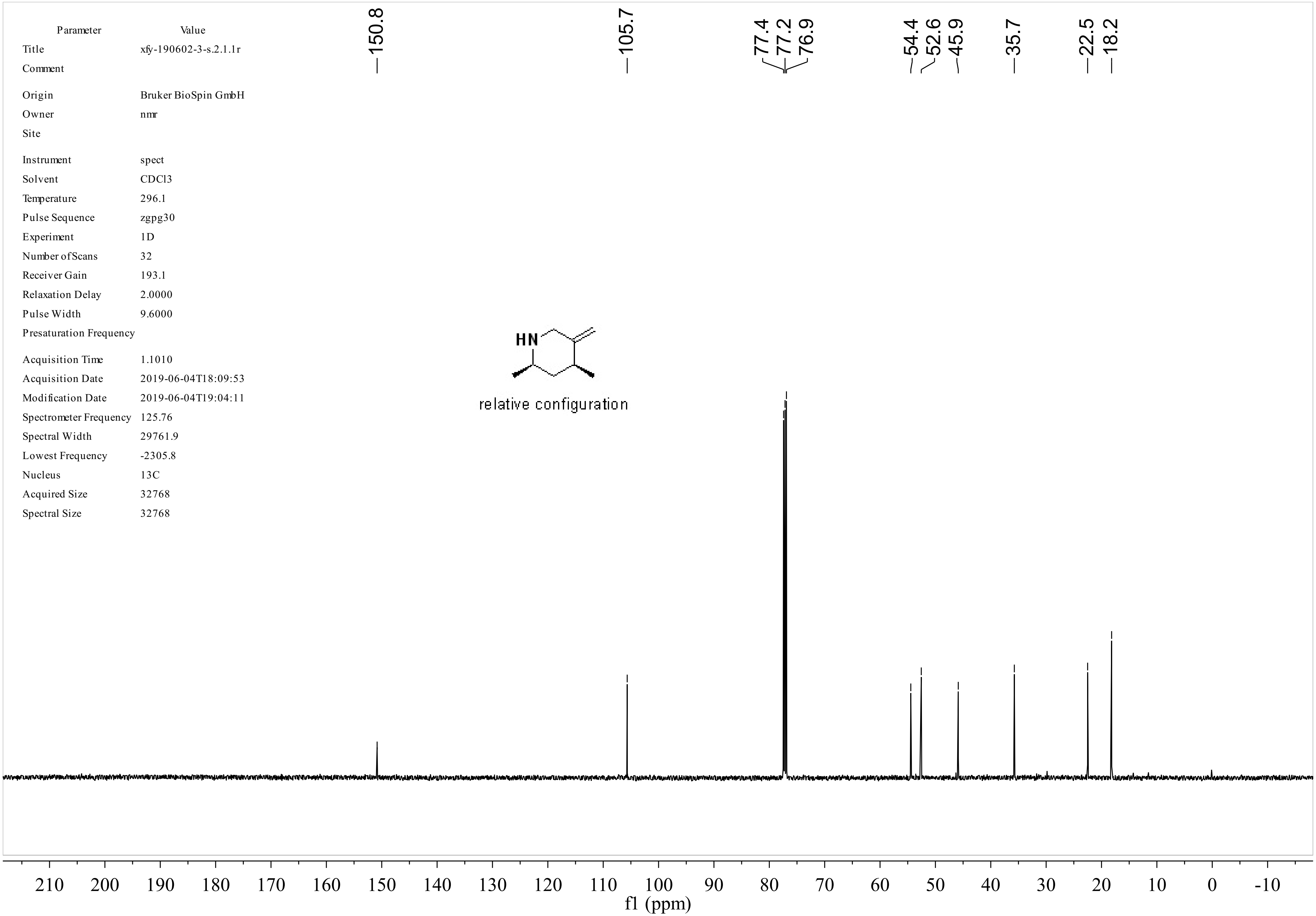
Parameter Value
Title xfy-190602-3-s.2.1.1r
Comment
Origin Bruker BioSpin GmbH
Owner nmr
Site
Instrument spect
Solvent CDCl3
Temperature 296.1
Pulse Sequence zgpg30
Experiment 1D
Number of Scans 32
Receiver Gain 193.1
Relaxation Delay 2.0000
Pulse Width 9.6000
Presaturation Frequency
Acquisition Time 1.1010
Acquisition Date 2019-06-04T18:09:53
Modification Date 2019-06-04T19:04:11
Spectrometer Frequency 125.76
Spectral Width 29761.9
Lowest Frequency -2305.8
Nucleus 13C
Acquired Size 32768
Spectral Size 32768
150.8
105.7
77.4
77.2
76.9
54.4
52.6
45.9
35.7
22.5
18.2
HN
relative configuration
210 200 190 180 170 160 150 140 130 120 110 100 90 80 70 60 50 40 30 20 10 0 -10
f1 (ppm)

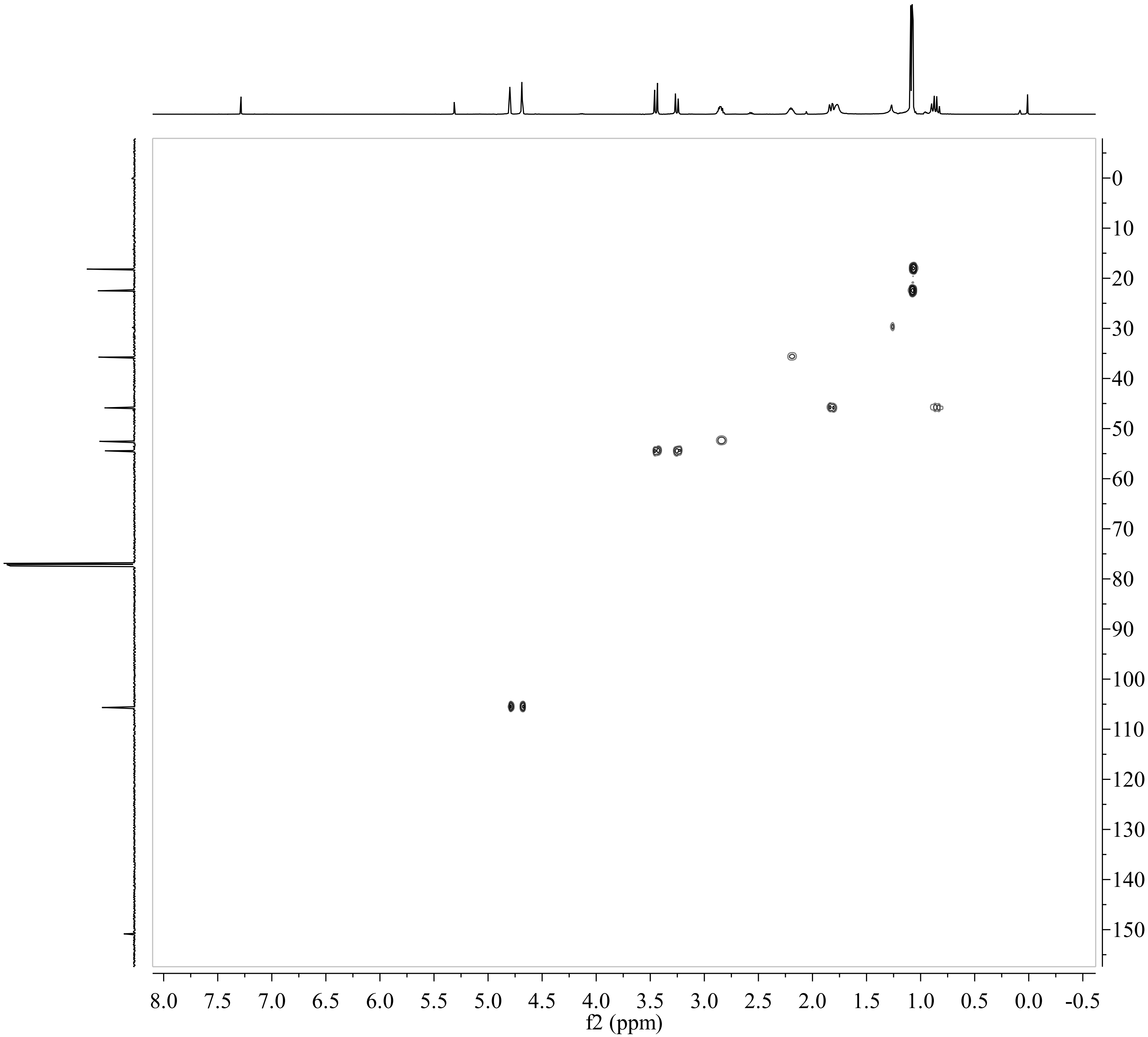

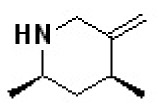

relative configuration

Parameter	Value
Title	xfy-190602-3-s.34.ser
Comment	
Origin	Bruker BioSpin GmbH
Owner	nmr
Site	
Instrument	spect
Solvent	CDCl3
Temperature	296.8
Pulse Sequence	hsqcetgp
Experiment	HSQC
Number of Scans	2
Receiver Gain	196.4
Relaxation Delay	1.4521
Pulse Width	8.7300
Presaturation Frequency	
Acquisition Time	0.1464
Acquisition Date	2019-06-05T04:42:11
Modification Date	2019-06-05T09:35:45
Spectrometer Frequency	(400.13, 100.62)
Spectral Width	(3496.5, 16666.7)
Lowest Frequency	(-254.6, -829.1)
Nucleus	(1H, 13C)
Acquired Size	(512, 256)
Spectral Size	(512, 512)

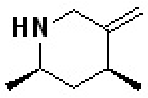

relative configuration

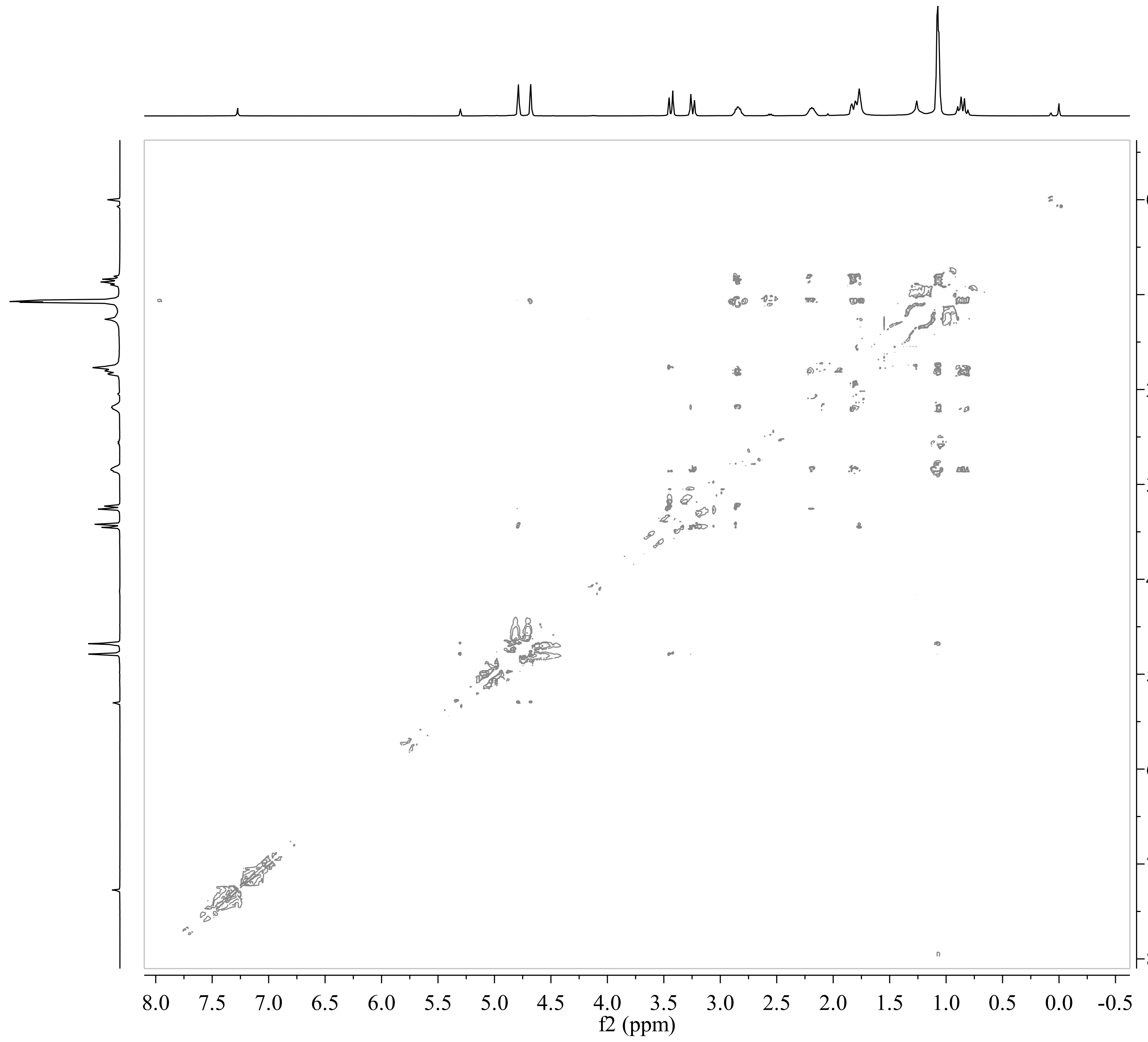

Parameter	Value
Title	xfy-190602-3-s.32.ser
Comment	
Origin	Bruker BioSpin GmbH
Owner	nmr
Site	
Instrument	spect
Solvent	CDCl3
Temperature	296.2
Pulse Sequence	noesygpphpp
Experiment	NOESY
Number of Scans	12
Receiver Gain	45.6
Relaxation Delay	1.9631
Pulse Width	8.7300
Presaturation Frequency	
Acquisition Time	0.2929
Acquisition Date	2019-06-05T02:11:26
Modification Date	2019-06-05T09:35:45
Spectrometer Frequency	(400.13, 400.13)
Spectral Width	(3496.5, 3496.5)
Lowest Frequency	(-254.6, -254.6)
Nucleus	(1H, 1H)
Acquired Size	(1024, 256)
Spectral Size	(1024, 1024)

Parameter	Value
Title	xfy-190602-3-dMs.1.1.1r
Comment	
Origin	Bruker BioSpin GmbH
Owner	nmr
Site	
Instrument	spect
Solvent	CDCl3
Temperature	296.8
Pulse Sequence	zg30
Experiment	1D
Number of Scans	8
Receiver Gain	126.1
Relaxation Delay	1.0000
Pulse Width	8.7300
Presaturation Frequency	
Acquisition Time	1.9999
Acquisition Date	2019-06-04T23:14:55
Modification Date	2019-06-05T09:22:48
Spectrometer Frequency	400.13
Spectral Width	8012.8
Lowest Frequency	-1543.1
Nucleus	1H
Acquired Size	16025
Spectral Size	65536

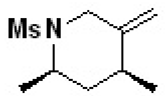

relative configuration

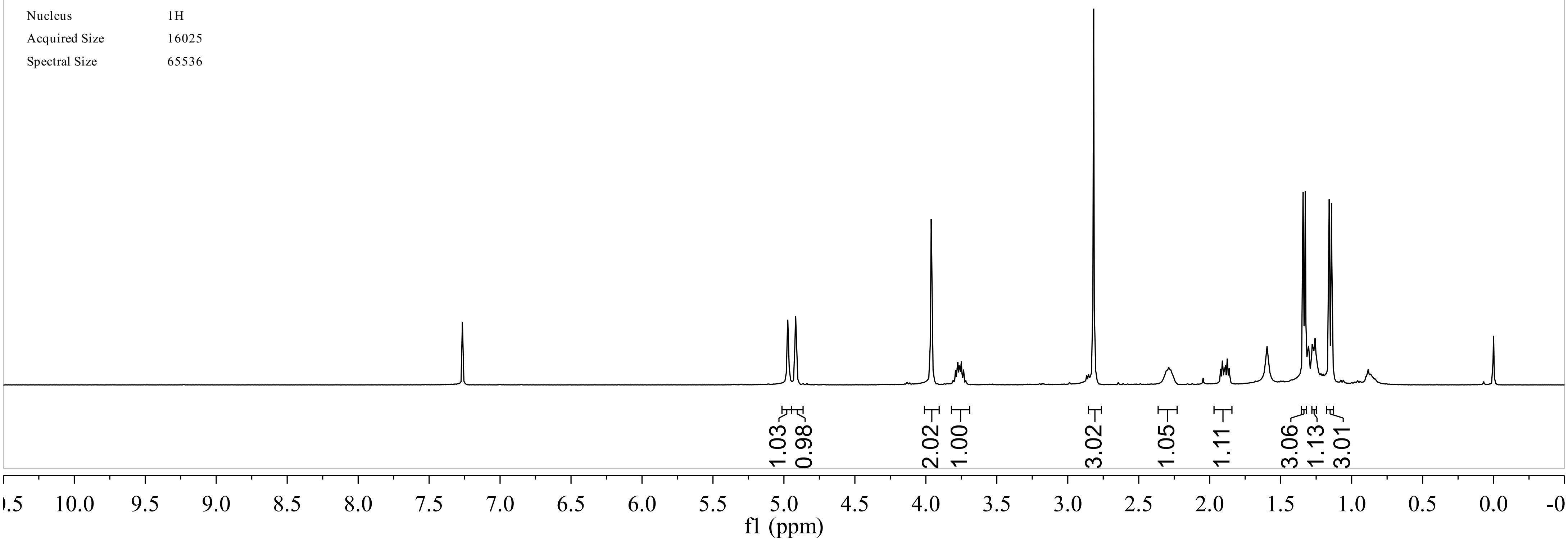

Parameter	Value
Title	xfy-190602-3-dMs.2.1.1r
Comment	
Origin	Bruker BioSpin GmbH
Owner	nmr
Site	
Instrument	spect
Solvent	CDCl3
Temperature	297.2
Pulse Sequence	zgpg30
Experiment	1D
Number of Scans	128
Receiver Gain	196.4
Relaxation Delay	2.0000
Pulse Width	10.0000
Presaturation Frequency	
Acquisition Time	1.3631
Acquisition Date	2019-06-04T23:17:58
Modification Date	2019-06-05T09:22:48
Spectrometer Frequency	100.61
Spectral Width	24038.5
Lowest Frequency	-1958.0
Nucleus	13C
Acquired Size	32768
Spectral Size	32768

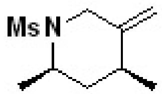

relative configuration

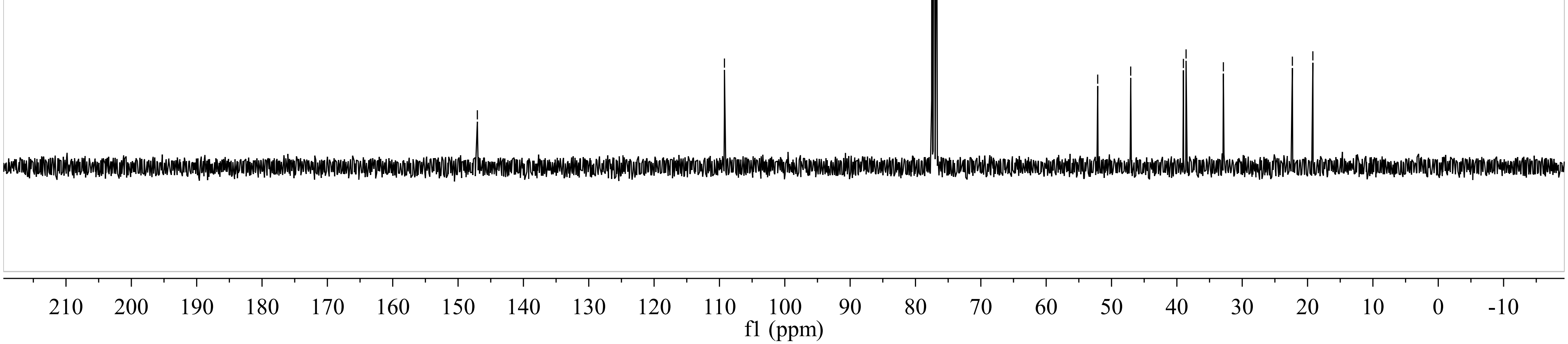

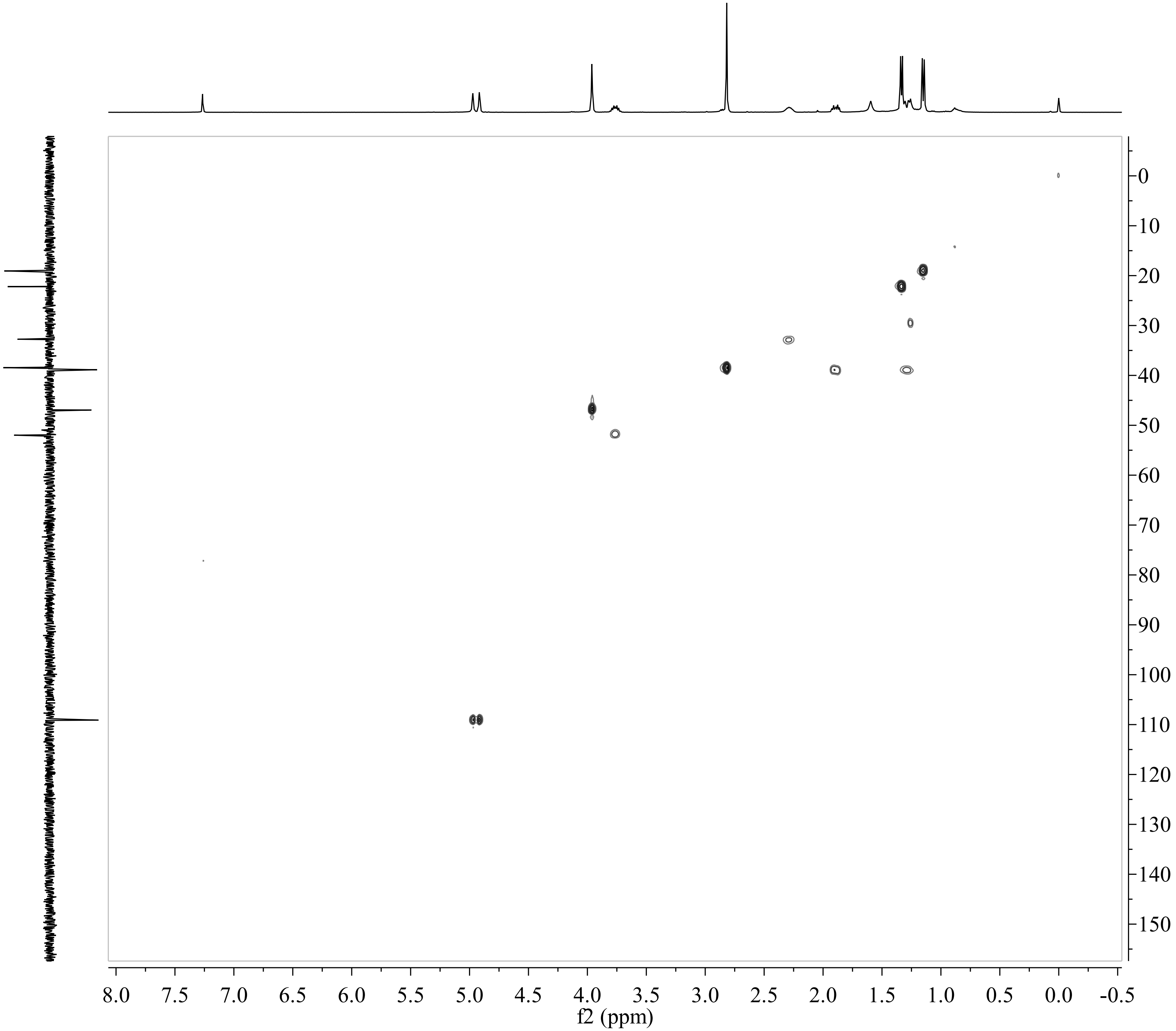

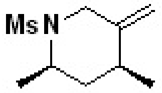

relative configuration

Parameter	Value
Title	xfy-190602-3-dMs.4.ser
Comment	
Origin	Bruker BioSpin GmbH
Owner	nmr
Site	
Instrument	spect
Solvent	CDCl3
Temperature	297.0
Pulse Sequence	hsqcetgp
Experiment	HSQC
Number of Scans	2
Receiver Gain	196.4
Relaxation Delay	1.4500
Pulse Width	8.7300
Presaturation Frequency	
Acquisition Time	0.1485
Acquisition Date	2019-06-04T23:32:07
Modification Date	2019-06-05T09:22:49
Spectrometer Frequency	(400.13, 100.62)
Spectral Width	(3448.3, 16666.7)
Lowest Frequency	(-221.0, -829.1)
Nucleus	(1H, 13C)
Acquired Size	(512, 256)
Spectral Size	(512, 512)

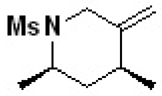

relative configuration

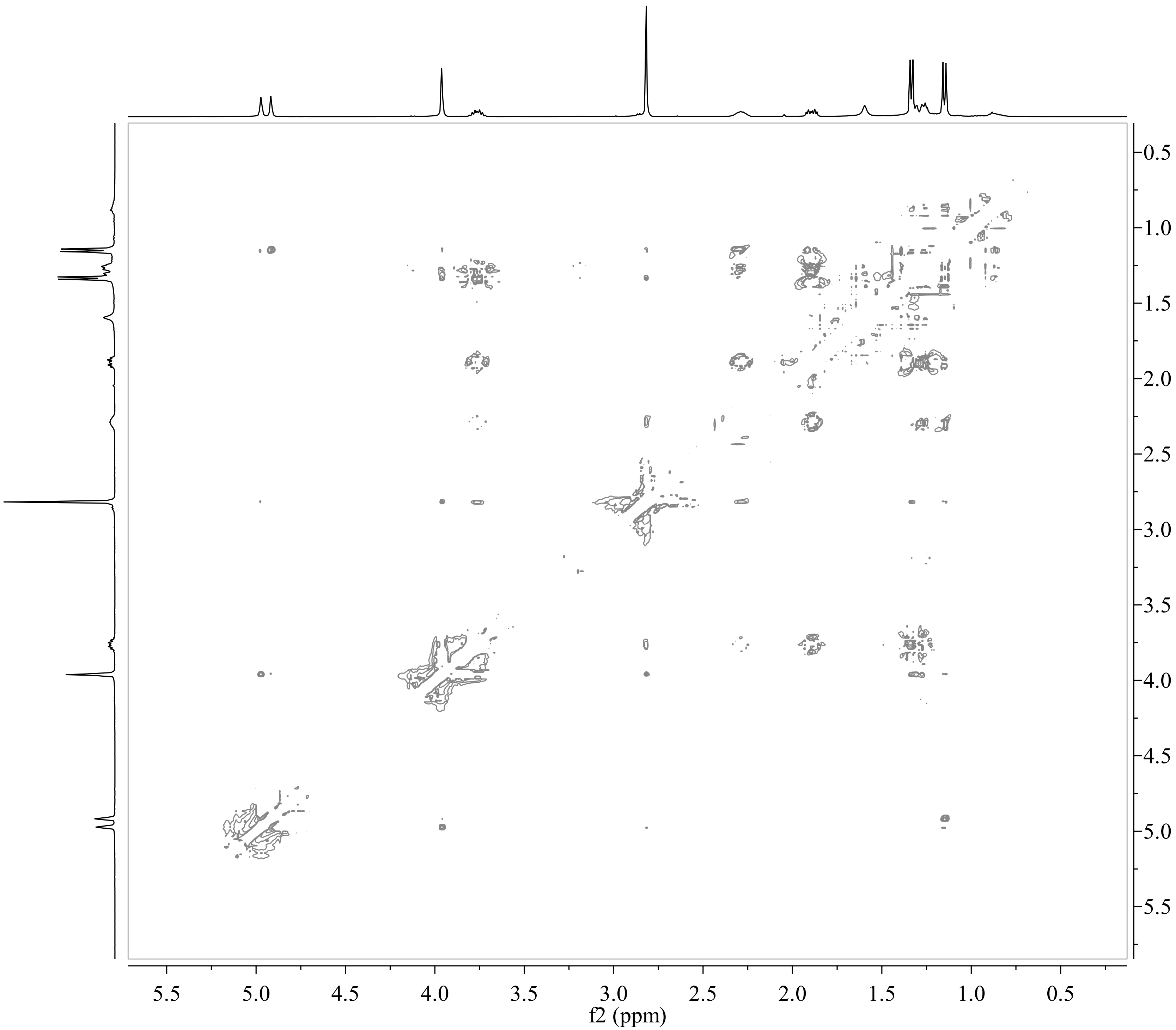

Parameter	Value
Title	xfy-190602-3-dMs.5.ser
Comment	
Origin	Bruker BioSpin GmbH
Owner	nmr
Site	
Instrument	spect
Solvent	CDCl3
Temperature	296.8
Pulse Sequence	noesygpphpp
Experiment	NOESY
Number of Scans	12
Receiver Gain	62.3
Relaxation Delay	1.9590
Pulse Width	8.7300
Presaturation Frequency	
Acquisition Time	0.2970
Acquisition Date	2019-06-04T23:48:36
Modification Date	2019-06-05T09:22:49
Spectrometer Frequency	(400.13, 400.13)
Spectral Width	(3448.3, 3448.3)
Lowest Frequency	(-221.0, -221.0)
Nucleus	(1H, 1H)
Acquired Size	(1024, 256)
Spectral Size	(1024, 1024)

Parameter	Value
标题	xfy-190602-2-s.64.fid
Comment	
Origin	Bruker BioSpin GmbH
Owner	nmr
Site	
Instrument	spect
Author	
Solvent	CDCl3
Temperature	296.2
Pulse Sequence	zg30
Experiment	1D
Number of Scans	4
Receiver Gain	70.5
Relaxation Delay	1.0000
Pulse Width	10.7100
Presaturation Frequency	
Acquisition Time	3.2768
Class	
Spectrometer Frequency	500.13
Spectral Width	10000.0
Lowest Frequency	-1923.5
Nucleus	1H
Acquired Size	32768
Spectral Size	65536

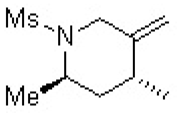

relative configuration

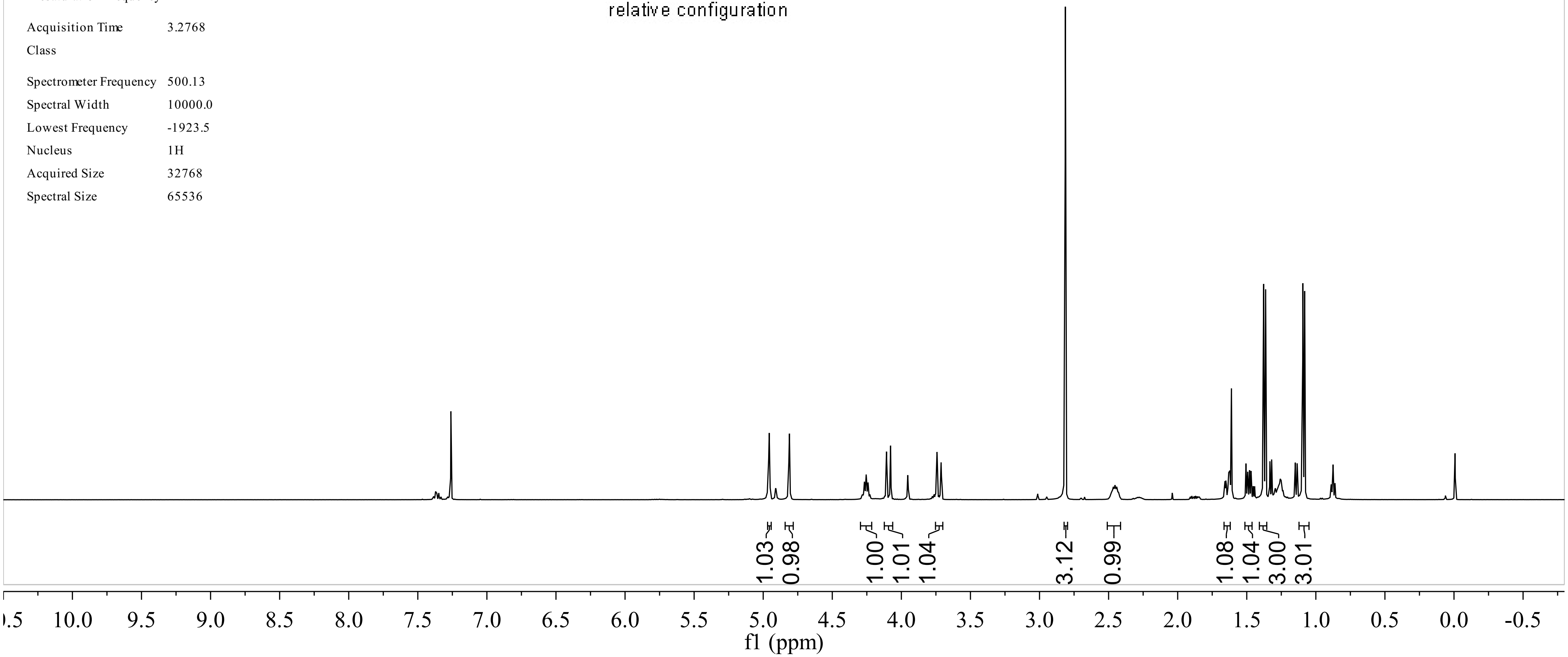

Parameter	Value
标题	xfy-190602-2-s.70.fid
Comment	
Origin	Bruker BioSpin GmbH
Owner	nmr
Site	
Instrument	spect
Author	
Solvent	CDCl3
Temperature	296.1
Pulse Sequence	zgpg30
Experiment	1D
Number of Scans	32
Receiver Gain	193.1
Relaxation Delay	2.0000
Pulse Width	9.6000
Presaturation Frequency	
Acquisition Time	1.1010
Class	
Spectrometer Frequency	125.77
Spectral Width	29761.9
Lowest Frequency	-2290.4
Nucleus	13C
Acquired Size	32768
Spectral Size	65536

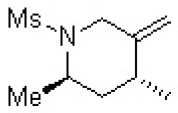

relative configuration

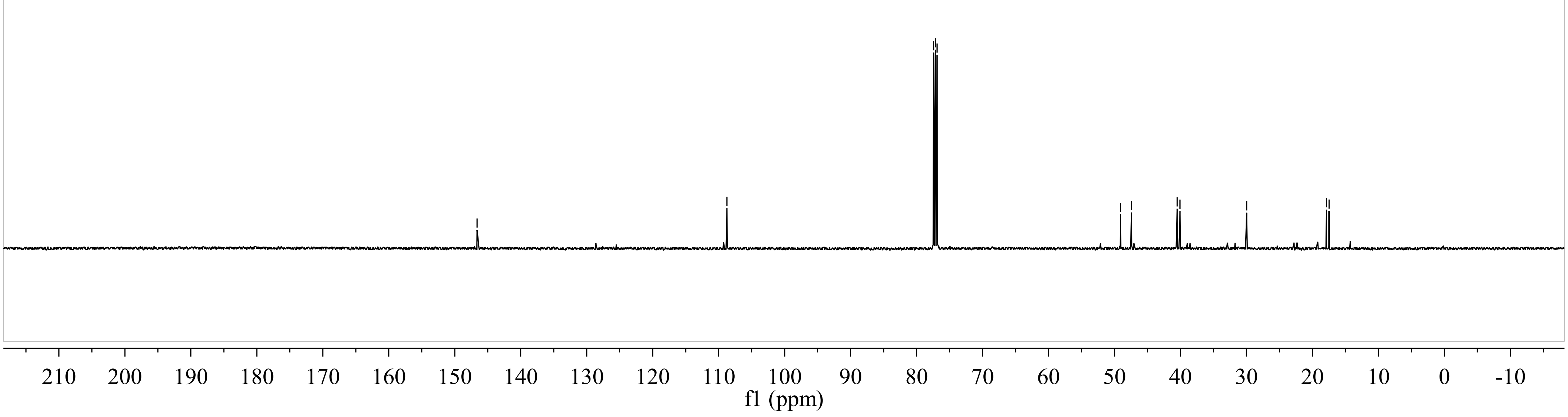

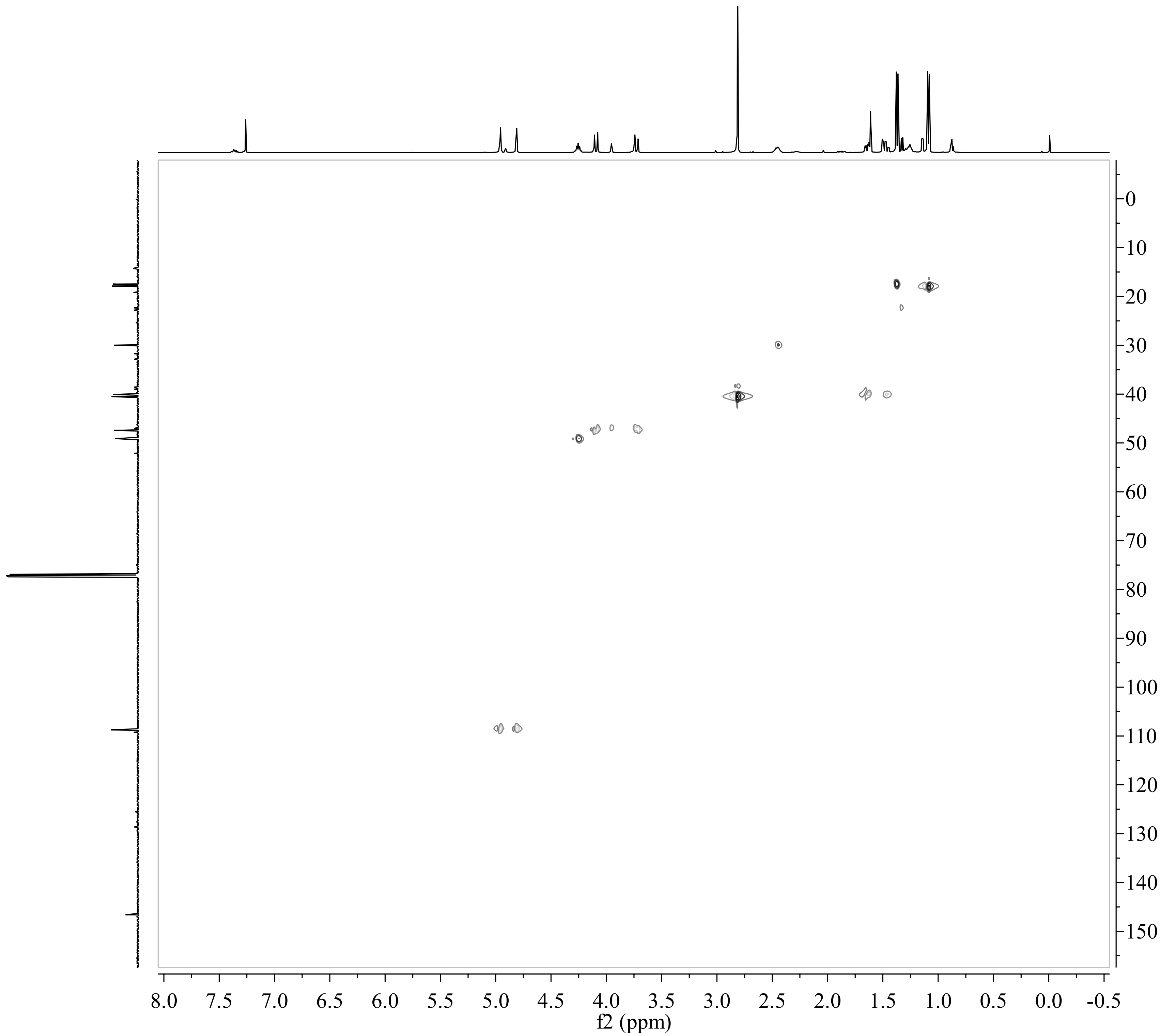

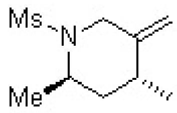

relative configuration

Parameter	Value
标题	xfy-190602-2-s.71.ser
Comment	
Origin	Bruker BioSpin GmbH
Owner	nmr
Site	
Instrument	spect
Author	
Solvent	CDCl3
Temperature	296.1
Pulse Sequence	hsqcedetgp
Experiment	HSQC-EDITED
Number of Scans	2
Receiver Gain	193.1
Relaxation Delay	1.4601
Pulse Width	10.7100
Presaturation Frequency	
Acquisition Time	0.1188
Class	
Spectrometer Frequency	(500.13, 125.77)
Spectral Width	(4310.3, 20833.3)
Lowest Frequency	(-283.5, -1037.0)
Nucleus	(1H, 13C)
Acquired Size	(512, 256)
Spectral Size	(512, 512)

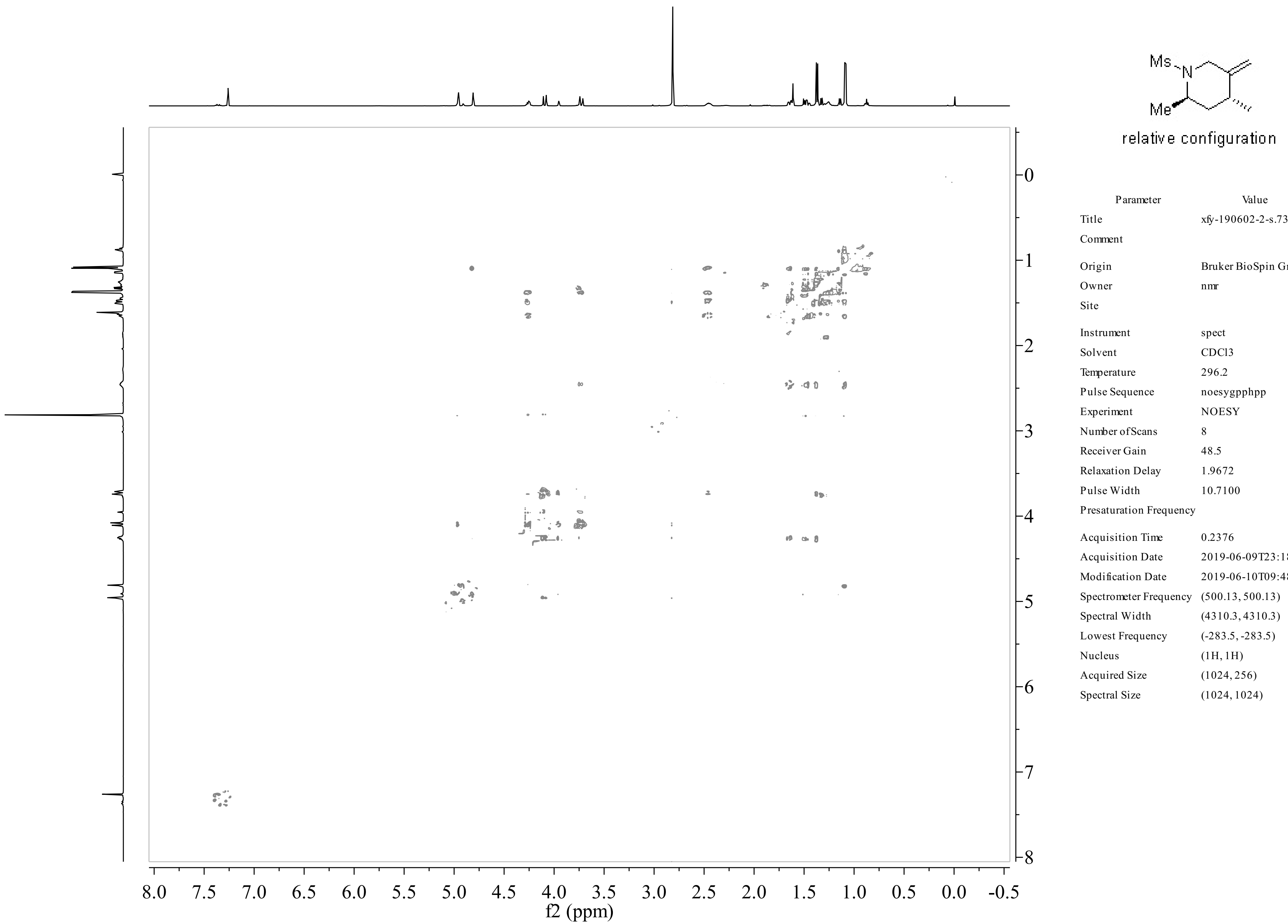

Parameter	Value
Title	xfy-190602-2-s.73.ser
Comment	
Origin	Bruker BioSpin GmbH
Owner	nmr
Site	
Instrument	spect
Solvent	CDCl3
Temperature	296.2
Pulse Sequence	noesygpphpp
Experiment	NOESY
Number of Scans	8
Receiver Gain	48.5
Relaxation Delay	1.9672
Pulse Width	10.7100
Presaturation Frequency	
Acquisition Time	0.2376
Acquisition Date	2019-06-09T23:18:40
Modification Date	2019-06-10T09:48:15
Spectrometer Frequency	(500.13, 500.13)
Spectral Width	(4310.3, 4310.3)
Lowest Frequency	(-283.5, -283.5)
Nucleus	(1H, 1H)
Acquired Size	(1024, 256)
Spectral Size	(1024, 1024)

Parameter	Value
Title	xfy-190514-3-dBn.3.fid
Comment	
Origin	Bruker BioSpin GmbH
Owner	nmr
Site	
Instrument	spect
Solvent	CDCl3
Temperature	298.5
Pulse Sequence	zg30
Experiment	1D
Number of Scans	8
Receiver Gain	173.6
Relaxation Delay	1.0000
Pulse Width	8.7300
Presaturation Frequency	
Acquisition Time	1.9999
Acquisition Date	2019-05-18T03:57:25
Modification Date	2019-05-18T11:01:34
Spectrometer Frequency	400.13
Spectral Width	8012.8
Lowest Frequency	-1545.8
Nucleus	1H
Acquired Size	16025
Spectral Size	65536

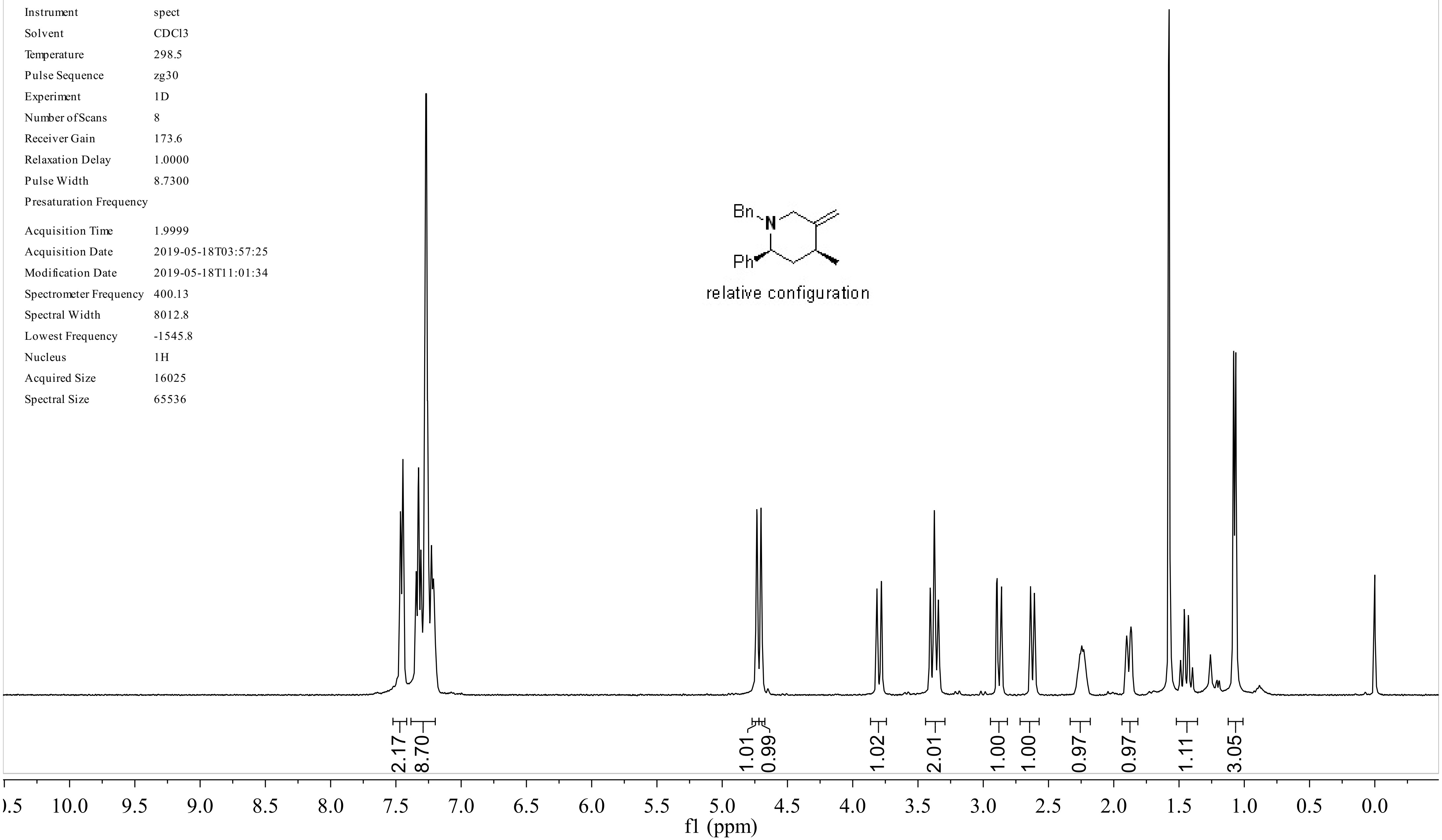

Parameter	Value
Title	xfy-190514-3-dBn.4.1.1r
Comment	
Origin	Bruker BioSpin GmbH
Owner	nmr
Site	
Instrument	spect
Solvent	CDCl3
Temperature	299.2
Pulse Sequence	zgpg30
Experiment	1D
Number of Scans	2000
Receiver Gain	196.4
Relaxation Delay	2.0000
Pulse Width	10.0000
Presaturation Frequency	
Acquisition Time	1.3631
Acquisition Date	2019-05-18T05:52:50
Modification Date	2019-05-18T11:01:35
Spectrometer Frequency	100.61
Spectral Width	24038.5
Lowest Frequency	-1944.1
Nucleus	13C
Acquired Size	32768
Spectral Size	32768

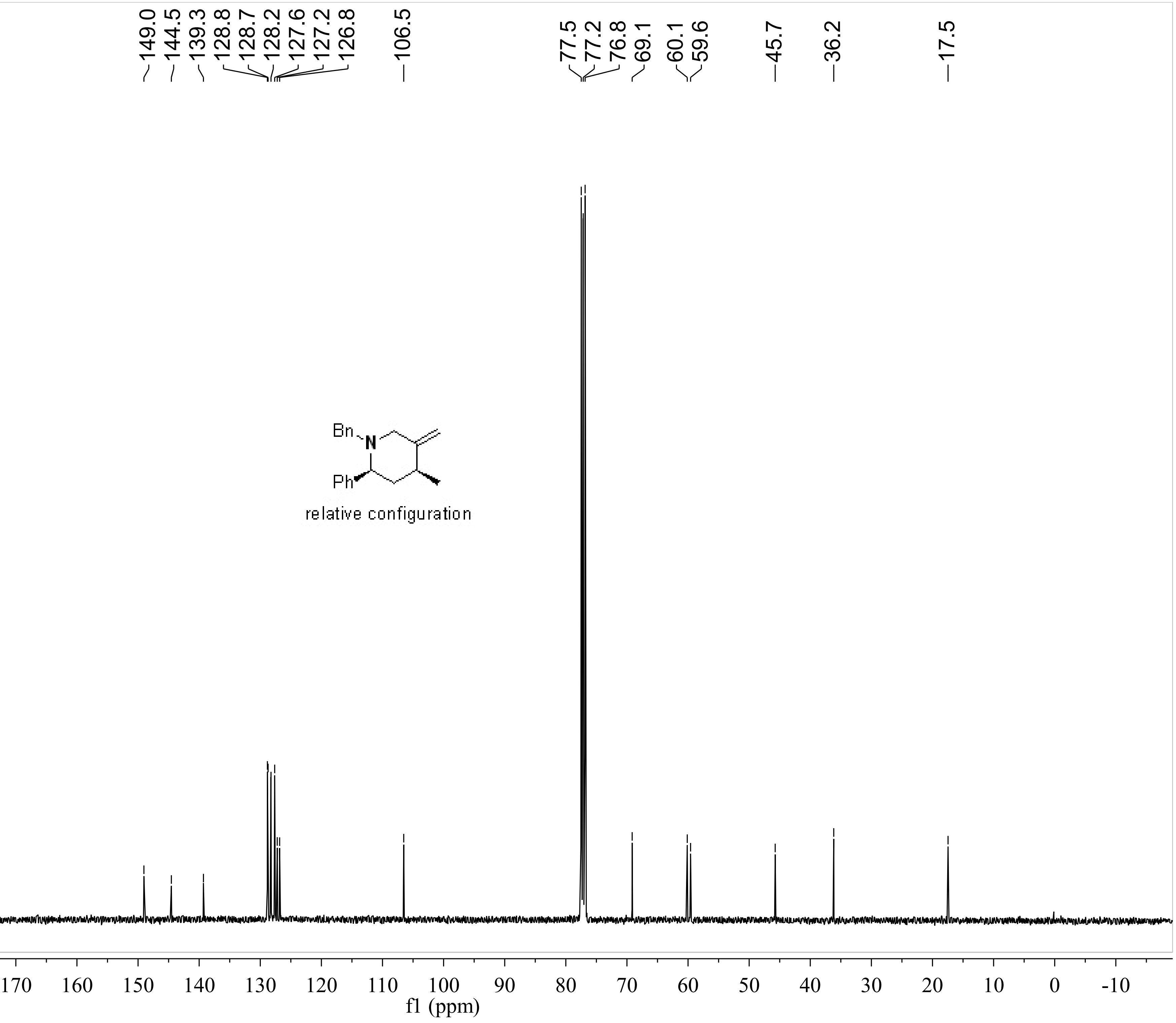

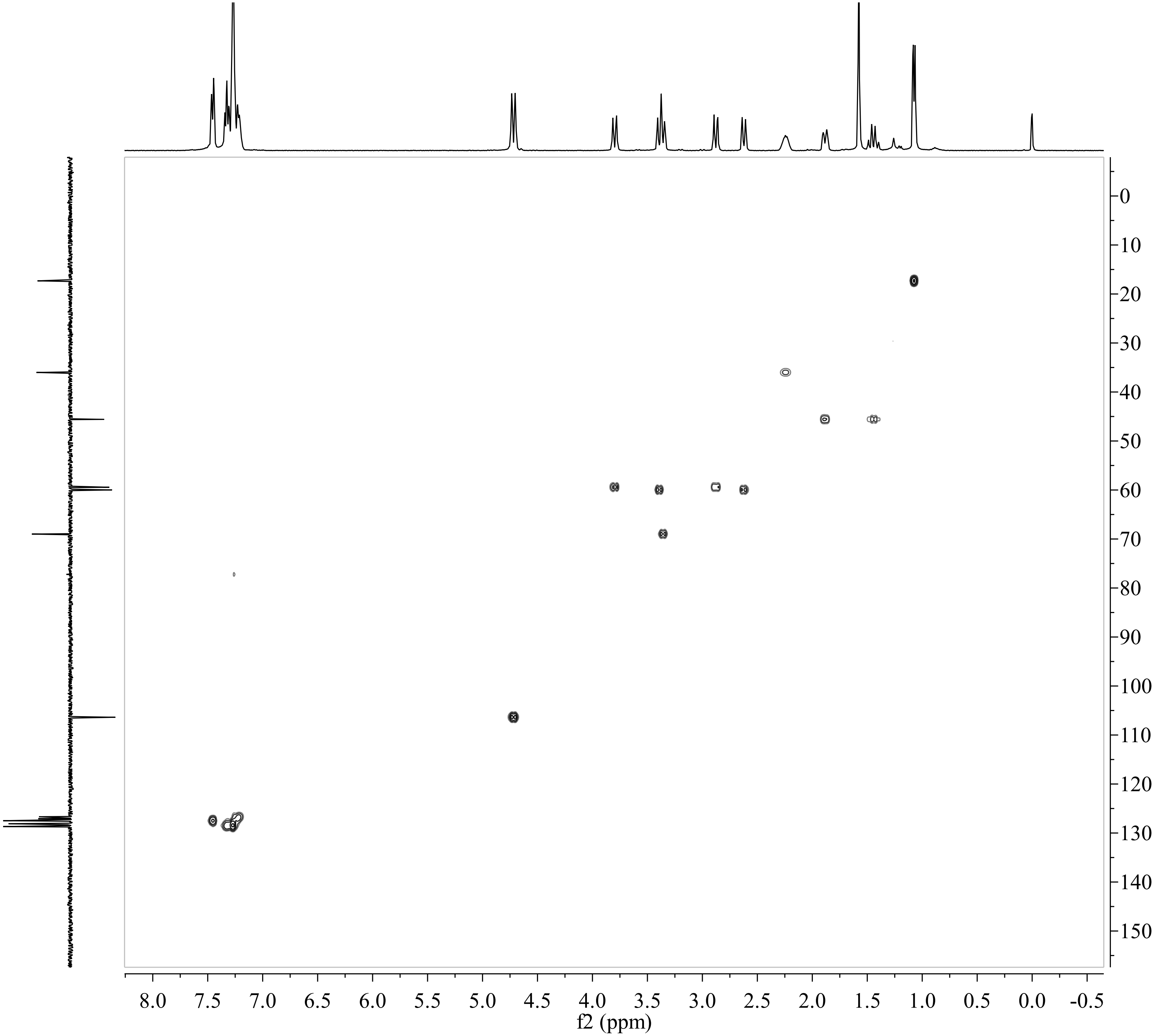

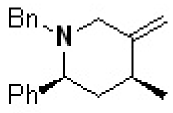

relative configuration

Parameter	Value
Title	xfy-190514-3-dBn.6.ser
Comment	
Origin	Bruker BioSpin GmbH
Owner	nmr
Site	
Instrument	spect
Solvent	CDCl3
Temperature	298.9
Pulse Sequence	hsqcetgp
Experiment	HSQC
Number of Scans	8
Receiver Gain	196.4
Relaxation Delay	1.4551
Pulse Width	8.7300
Presaturation Frequency	
Acquisition Time	0.1434
Acquisition Date	2019-05-18T06:25:35
Modification Date	2019-05-18T11:01:32
Spectrometer Frequency	(400.13, 100.62)
Spectral Width	(3571.4, 16666.7)
Lowest Frequency	(-267.7, -829.1)
Nucleus	(1H, 13C)
Acquired Size	(512, 256)
Spectral Size	(512, 512)

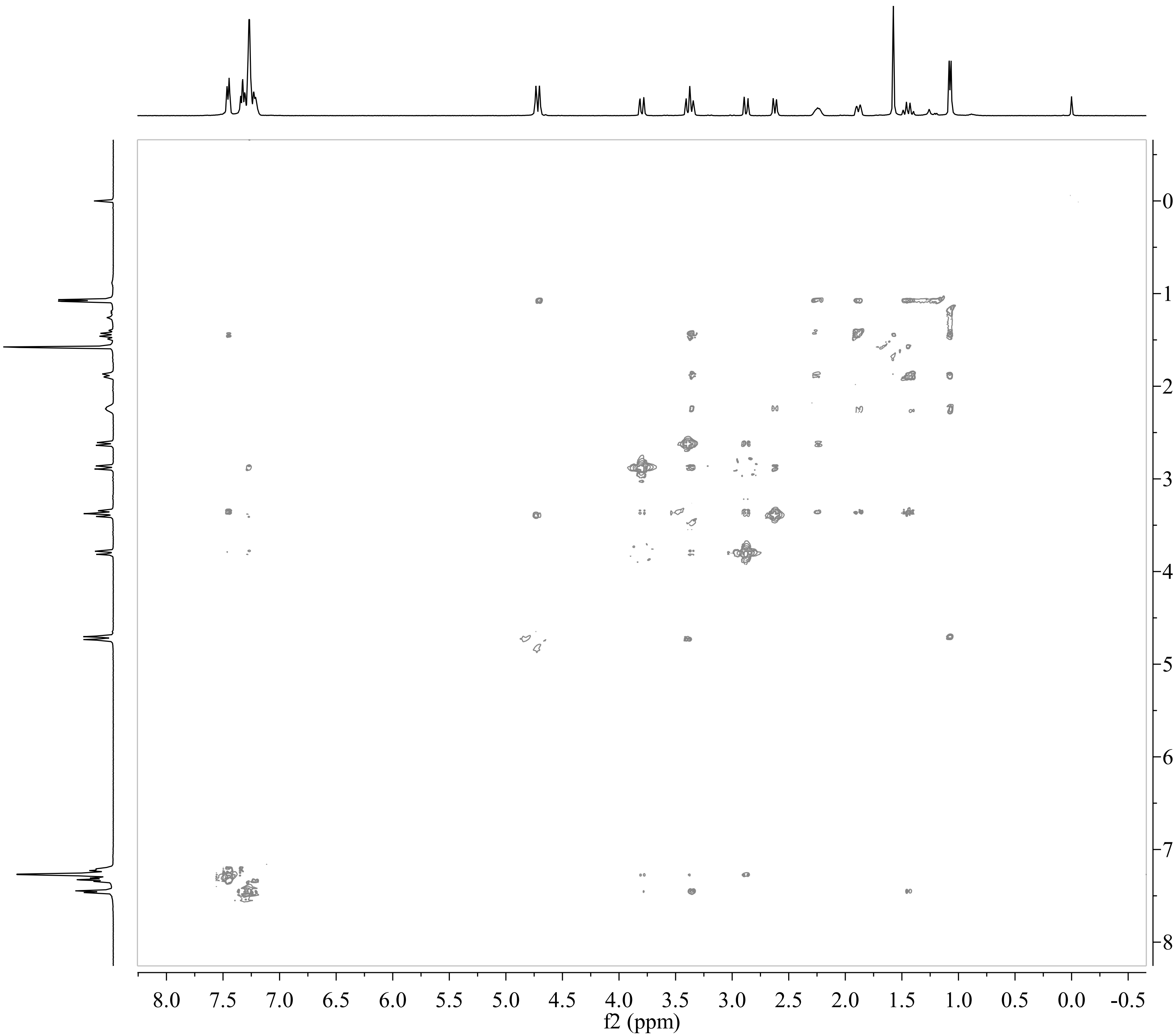

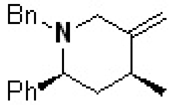

relative configuration

Parameter	Value
Title	xfy-190514-3-dBn.7.ser
Comment	
Origin	Bruker BioSpin GmbH
Owner	nmr
Site	
Instrument	spect
Solvent	CDCl3
Temperature	298.7
Pulse Sequence	noesygpphpp
Experiment	NOESY
Number of Scans	8
Receiver Gain	62.3
Relaxation Delay	1.9693
Pulse Width	8.7300
Presaturation Frequency	
Acquisition Time	0.2867
Acquisition Date	2019-05-18T07:22:58
Modification Date	2019-05-18T11:01:34
Spectrometer Frequency	(400.13, 400.13)
Spectral Width	(3571.4, 3571.4)
Lowest Frequency	(-267.7, -267.7)
Nucleus	(1H, 1H)
Acquired Size	(1024, 256)
Spectral Size	(1024, 1024)

Parameter	Value
Title	xfy-190514-3-dTs.41.fid
Comment	
Origin	Bruker BioSpin GmbH
Owner	nmr
Site	
Instrument	spect
Solvent	CDCl3
Temperature	298.8
Pulse Sequence	zg30
Experiment	1D
Number of Scans	8
Receiver Gain	126.1
Relaxation Delay	1.0000
Pulse Width	8.7300
Presaturation Frequency	
Acquisition Time	1.9999
Acquisition Date	2019-05-17T23:55:12
Modification Date	2019-05-18T11:01:14
Spectrometer Frequency	400.13
Spectral Width	8012.8
Lowest Frequency	-1544.6
Nucleus	1H
Acquired Size	16025
Spectral Size	65536

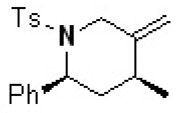

relative configuration

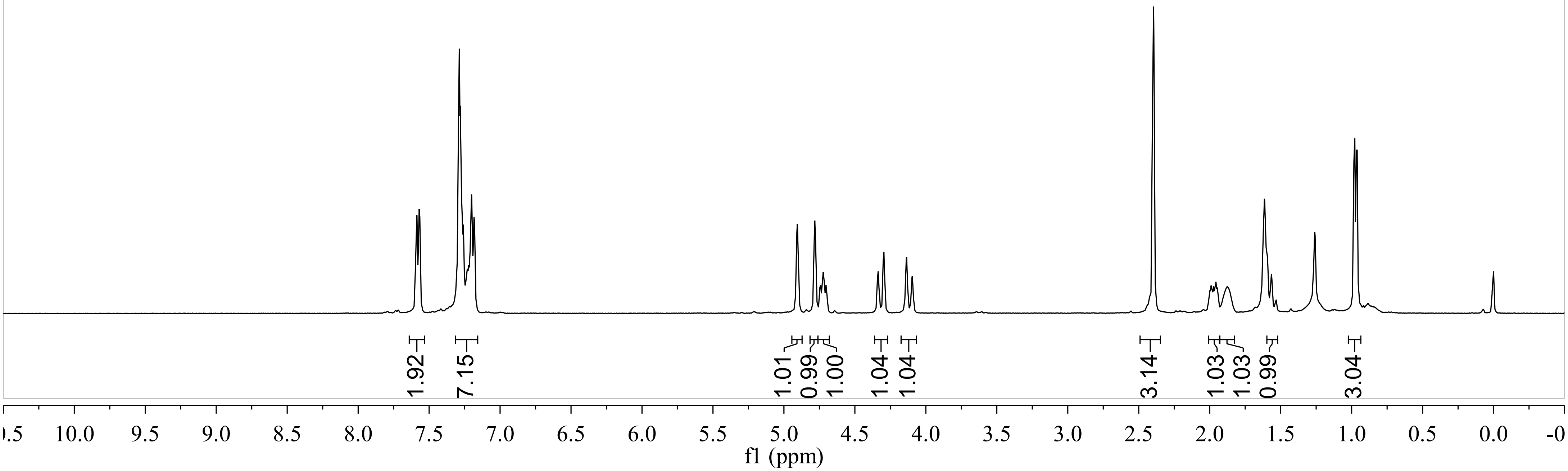

Parameter	Value
Title	xfy-190514-3-dTs.22.fid
Comment	
Origin	Bruker BioSpin GmbH
Owner	nmr
Site	
Instrument	spect
Solvent	CDCl3
Temperature	296.2
Pulse Sequence	zgpg30
Experiment	1D
Number of Scans	24
Receiver Gain	193.1
Relaxation Delay	2.0000
Pulse Width	9.6000
Presaturation Frequency	
Acquisition Time	1.1010
Acquisition Date	2019-05-16T09:35:00
Modification Date	2019-05-18T11:01:22
Spectrometer Frequency	125.77
Spectral Width	29761.9
Lowest Frequency	-2305.8
Nucleus	13C
Acquired Size	32768
Spectral Size	65536

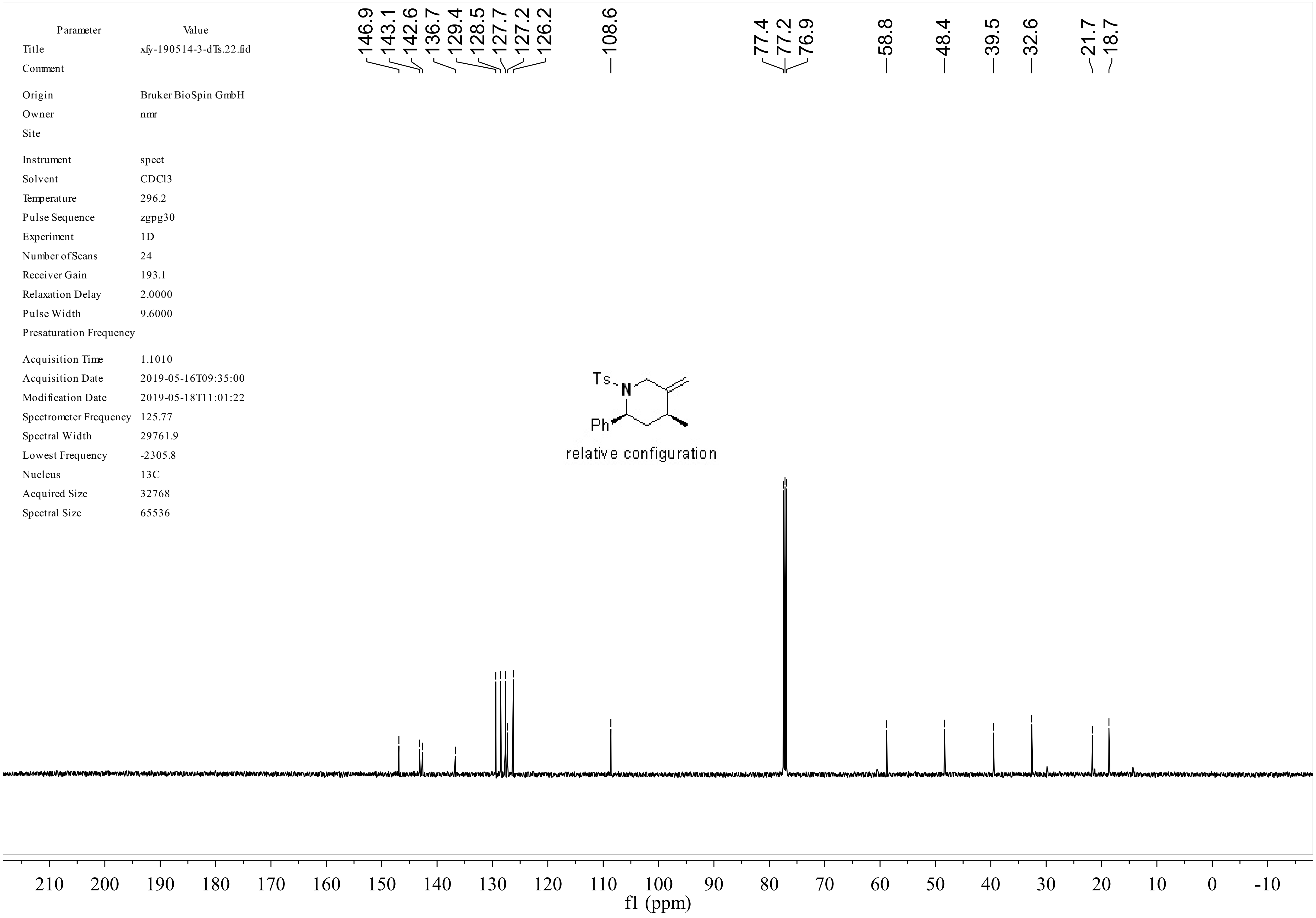

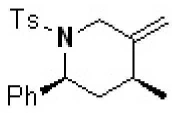

relative configuration

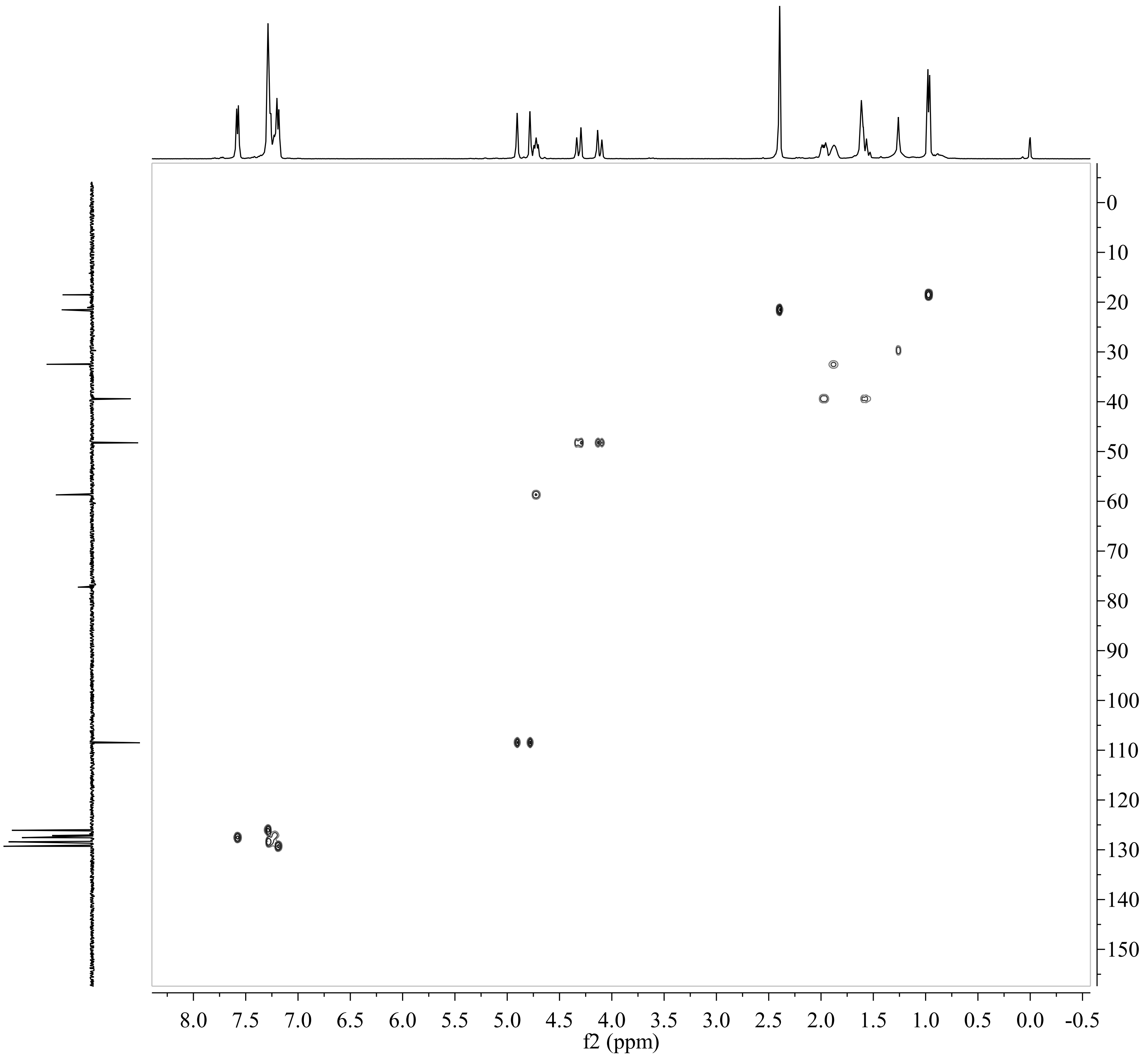

Parameter	Value
Title	xfy-190514-3-dTs.44.ser
Comment	
Origin	Bruker BioSpin GmbH
Owner	nmr
Site	
Instrument	spect
Solvent	CDCl3
Temperature	299.1
Pulse Sequence	hsqcetgp
Experiment	HSQC
Number of Scans	8
Receiver Gain	196.4
Relaxation Delay	1.4562
Pulse Width	8.7300
Presaturation Frequency	
Acquisition Time	0.1423
Acquisition Date	2019-05-18T01:27:05
Modification Date	2019-05-18T11:01:18
Spectrometer Frequency	(400.13, 100.62)
Spectral Width	(3597.1, 16666.7)
Lowest Frequency	(-237.3, -829.1)
Nucleus	(1H, 13C)
Acquired Size	(512, 256)
Spectral Size	(512, 512)

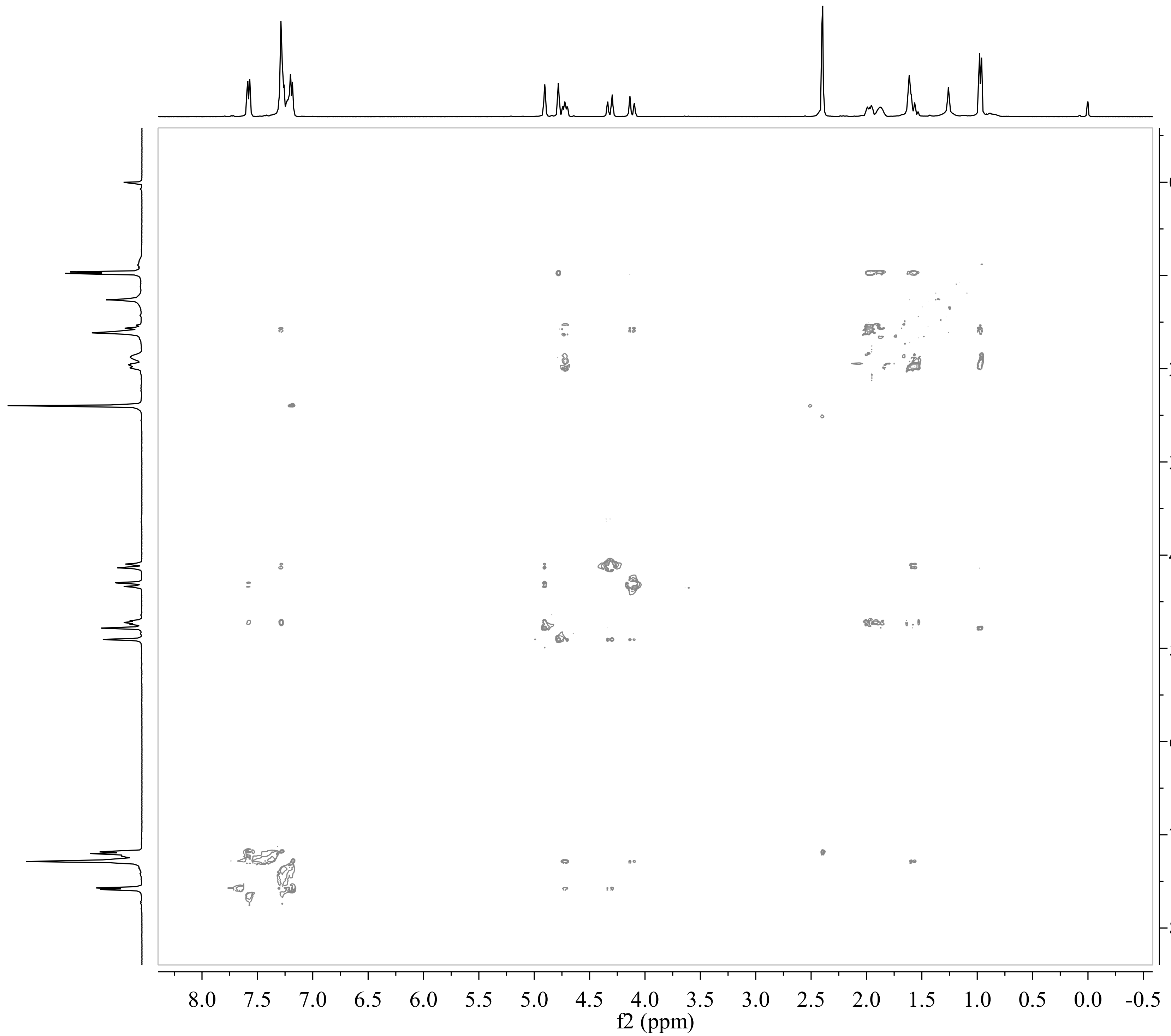

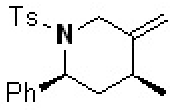

relative configuration

Parameter	Value
Title	xfy-190514-3-dTs.45.ser
Comment	
Origin	Bruker BioSpin GmbH
Owner	nmr
Site	
Instrument	spect
Solvent	CDCl3
Temperature	298.9
Pulse Sequence	noesygpphpp
Experiment	NOESY
Number of Scans	8
Receiver Gain	31.7
Relaxation Delay	1.9713
Pulse Width	8.7300
Presaturation Frequency	
Acquisition Time	0.2847
Acquisition Date	2019-05-18T02:24:38
Modification Date	2019-05-18T11:01:19
Spectrometer Frequency	(400.13, 400.13)
Spectral Width	(3597.1, 3597.1)
Lowest Frequency	(-237.3, -237.3)
Nucleus	(1H, 1H)
Acquired Size	(1024, 256)
Spectral Size	(1024, 1024)

Parameter	Value
Title	gvv-e-140-1-noesy.3.fid
Comment	
Origin	Bruker BioSpin GmbH
Owner	nmr
Site	
Instrument	spect
Solvent	CDCl3
Temperature	295.4
Pulse Sequence	zg30
Experiment	1D
Number of Scans	8
Receiver Gain	34.9
Relaxation Delay	1.0000
Pulse Width	10.0000
Presaturation Frequency	
Acquisition Time	1.9999
Acquisition Date	2018-11-25T10:57:29
Modification Date	2018-11-26T15:12:37
Spectrometer Frequency	400.13
Spectral Width	8012.8
Lowest Frequency	-1543.2
Nucleus	1H
Acquired Size	16025
Spectral Size	65536

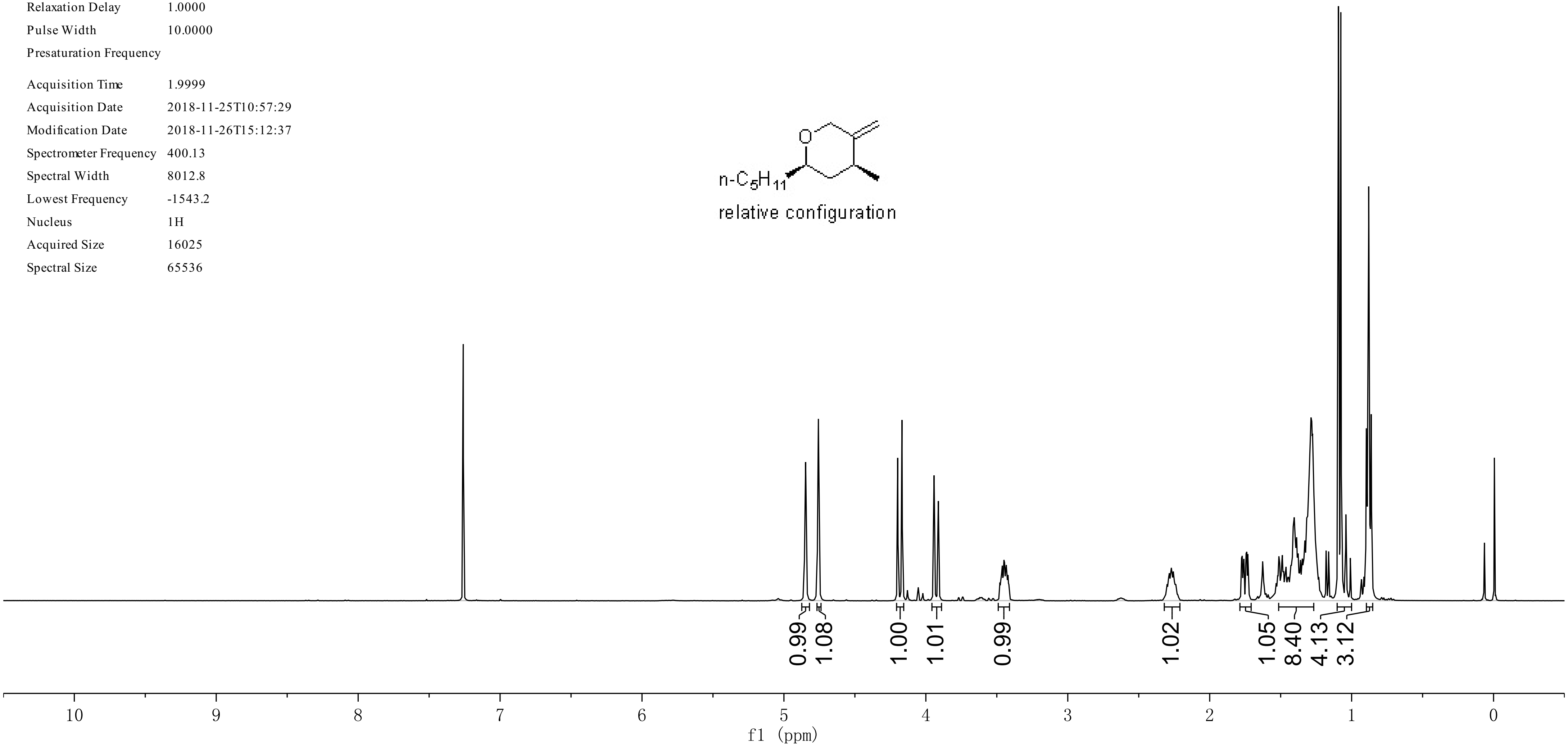

Parameter	Value
Title	gvv-e-140-1f-HSQC.3.fid
Comment	
Origin	Bruker BioSpin GmbH
Owner	nmr
Site	
Instrument	spect
Solvent	CDCl3
Temperature	296.2
Pulse Sequence	zgpg30
Experiment	1D
Number of Scans	50
Receiver Gain	193.1
Relaxation Delay	2.0000
Pulse Width	9.6000
Presaturation Frequency	
Acquisition Time	1.1010
Acquisition Date	2018-11-14T09:58:02
Modification Date	2019-01-23T17:14:08
Spectrometer Frequency	125.77
Spectral Width	29761.9
Lowest Frequency	-2289.7
Nucleus	13C
Acquired Size	32768
Spectral Size	65536

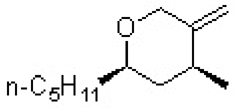

relative configuration

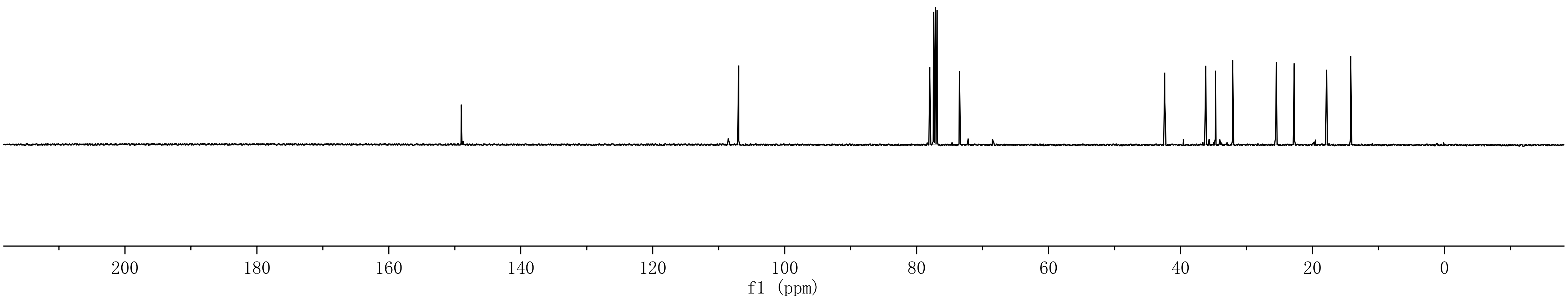

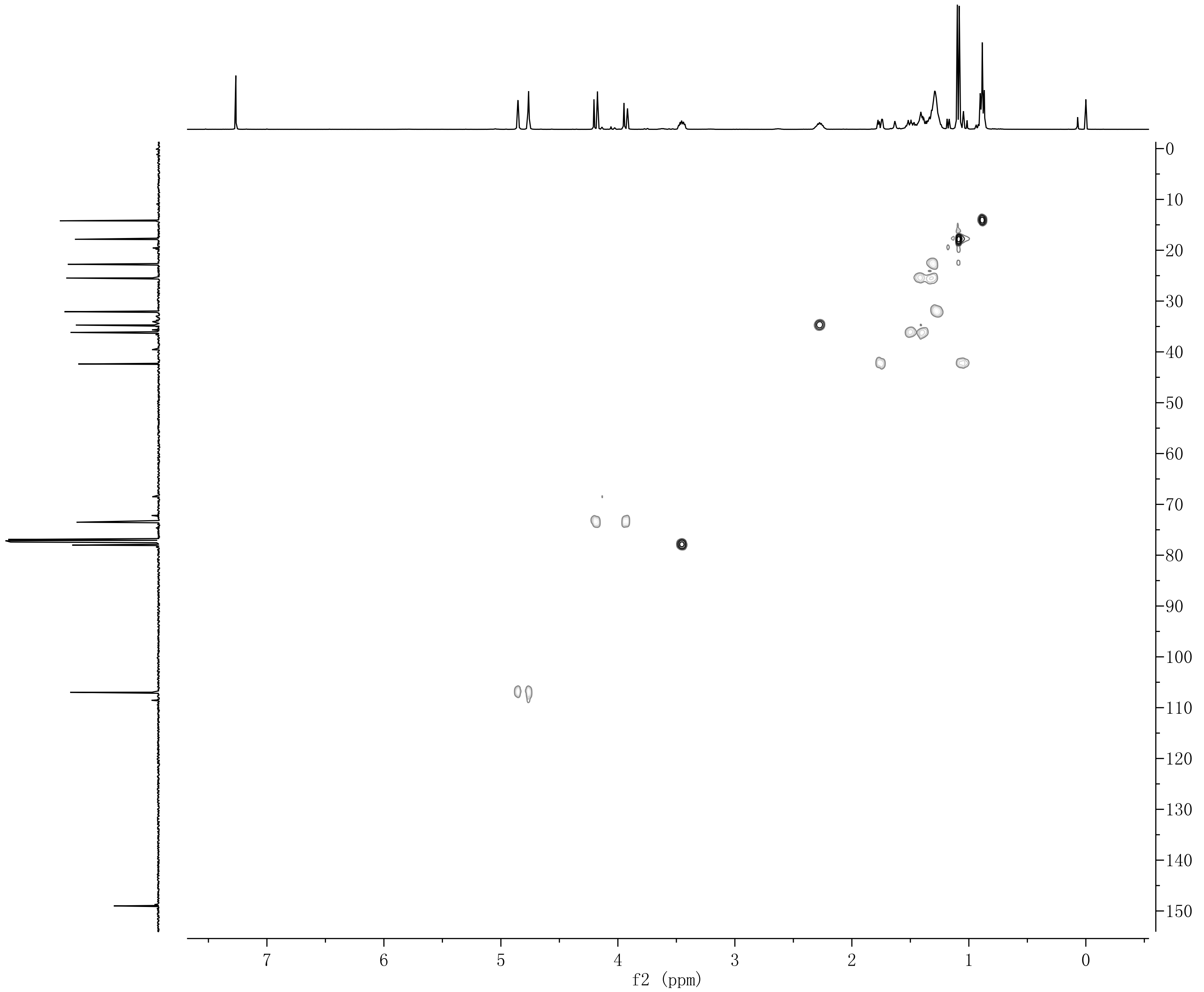

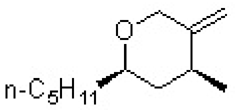

relative configuration

Parameter	Value
Title	gvv-e-140-1f-HSQC.4.ser
Comment	
Origin	Bruker BioSpin GmbH
Owner	nmr
Site	
Instrument	spect
Solvent	CDCl3
Temperature	296.1
Pulse Sequence	hsqcedetgp
Experiment	HSQC-EDITED
Number of Scans	2
Receiver Gain	193.1
Relaxation Delay	1.4590
Pulse Width	10.7100
Presaturation Frequency	
Acquisition Time	0.1198
Acquisition Date	2018-11-14T09:59:40
Modification Date	2019-01-23T17:14:10
Spectrometer Frequency	(500.13, 125.77)
Spectral Width	(4273.5, 20833.3)
Lowest Frequency	(-278.4, -1037.0)
Nucleus	(1H, 13C)
Acquired Size	(512, 256)
Spectral Size	(512, 512)

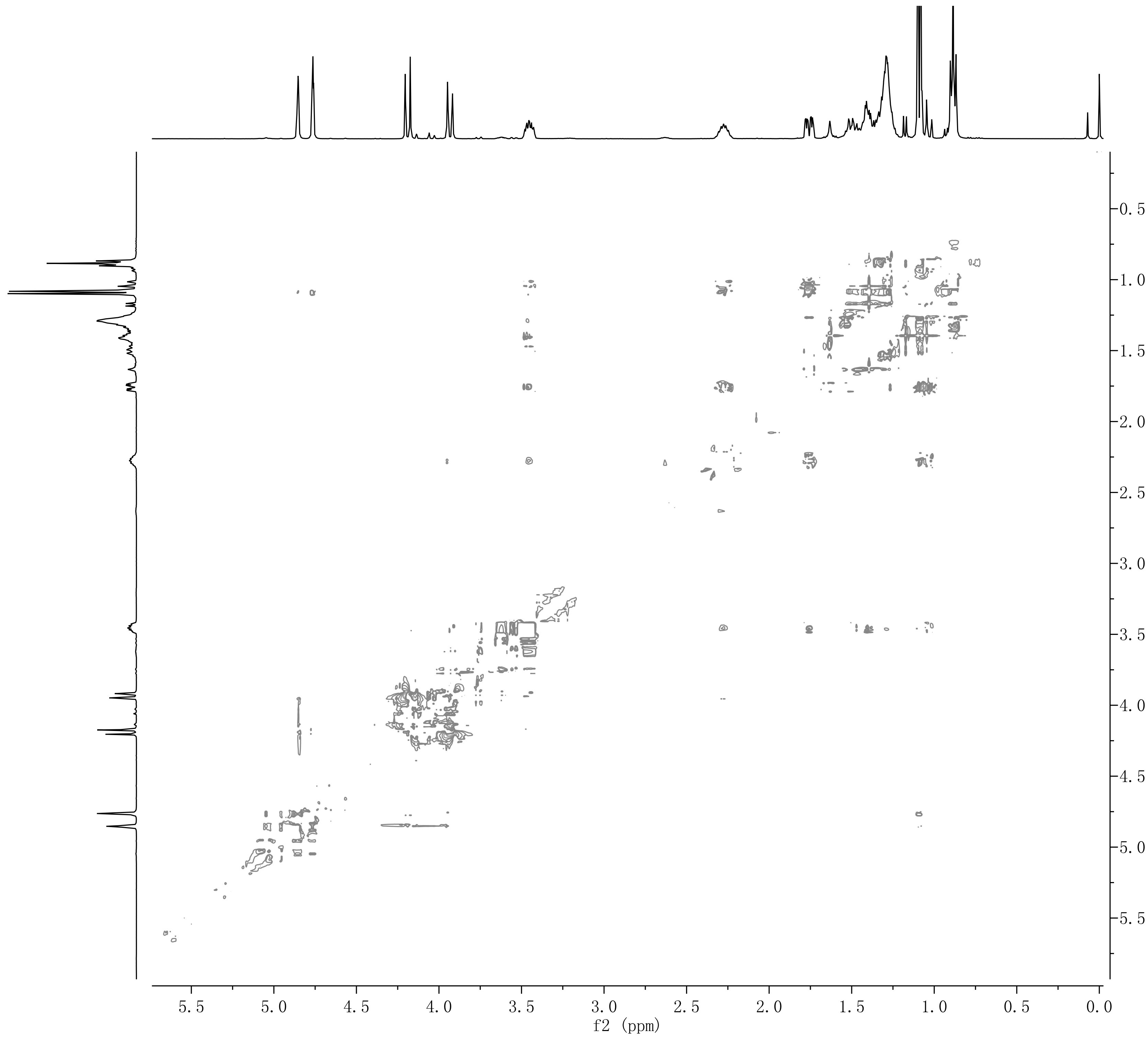

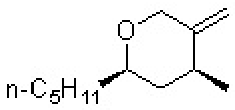

relative configuration

Parameter	Value
Title	gvv-e-140-1-noesy.5.ser
Comment	
Origin	Bruker BioSpin GmbH
Owner	nmr
Site	
Instrument	spect
Solvent	CDCl3
Temperature	295.4
Pulse Sequence	noesygpphpp
Experiment	NOESY
Number of Scans	4
Receiver Gain	34.9
Relaxation Delay	1.9631
Pulse Width	10.0000
Presaturation Frequency	
Acquisition Time	0.2929
Acquisition Date	2018-11-25T10:59:51
Modification Date	2018-11-26T15:12:37
Spectrometer Frequency	(400.13, 400.13)
Spectral Width	(3496.5, 3496.5)
Lowest Frequency	(-298.8, -298.8)
Nucleus	(1H, 1H)
Acquired Size	(1024, 256)
Spectral Size	(1024, 1024)

Parameter	Value
Title	xfy-190318-gvv.11.fid
Comment	
Origin	Bruker BioSpin GmbH
Owner	nmr
Site	
Instrument	spect
Solvent	CDCl3
Temperature	296.2
Pulse Sequence	zg30
Experiment	1D
Number of Scans	16
Receiver Gain	87.5
Relaxation Delay	1.0000
Pulse Width	10.7100
Presaturation Frequency	
Acquisition Time	3.2768
Acquisition Date	2019-03-25T07:51:38
Modification Date	2019-04-11T10:18:48
Spectrometer Frequency	500.13
Spectral Width	10000.0
Lowest Frequency	-1923.0
Nucleus	1H
Acquired Size	32768
Spectral Size	65536

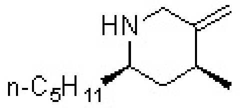

relative configuration

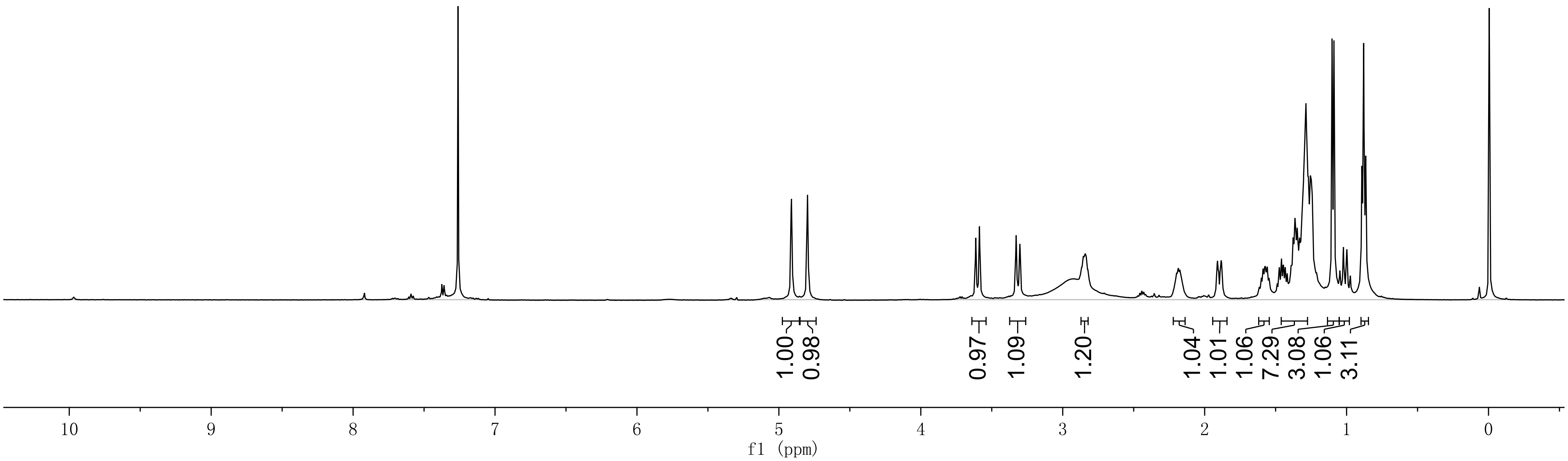

Parameter	Value
Origin	Bruker BioSpin GmbH
Spectrometer	spect
Solvent	CDCl3
Temperature	296.1
Pulse Sequence	zgpg30
Experiment	1D
Number of Scans	200
Receiver Gain	193
Relaxation Delay	2.0000
Pulse Width	9.6000
Acquisition Time	1.1010
Spectrometer Frequency	125.77
Spectral Width	29761.9
Lowest Frequency	-2305.8
Nucleus	13C
Acquired Size	32768
Spectral Size	65536

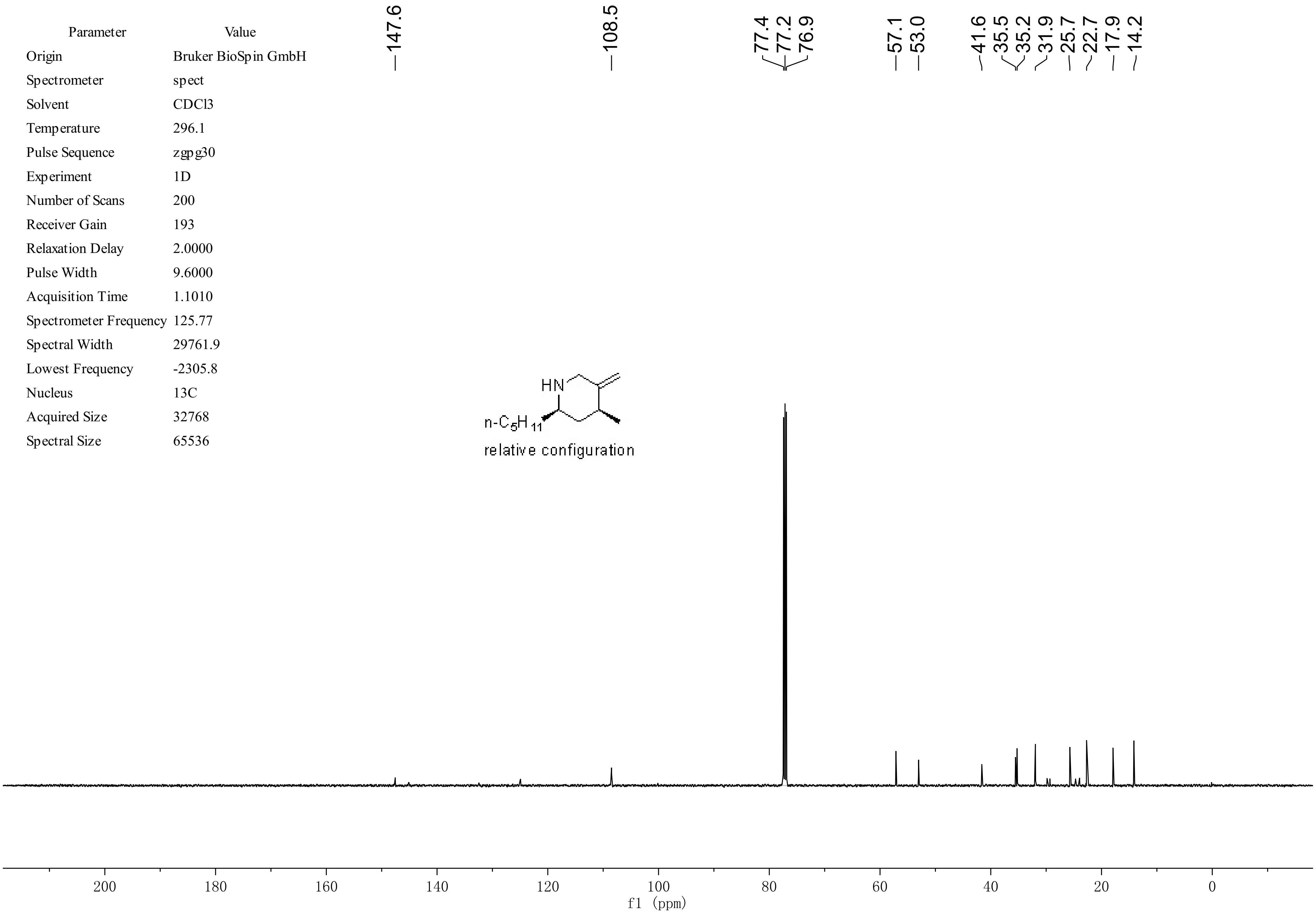

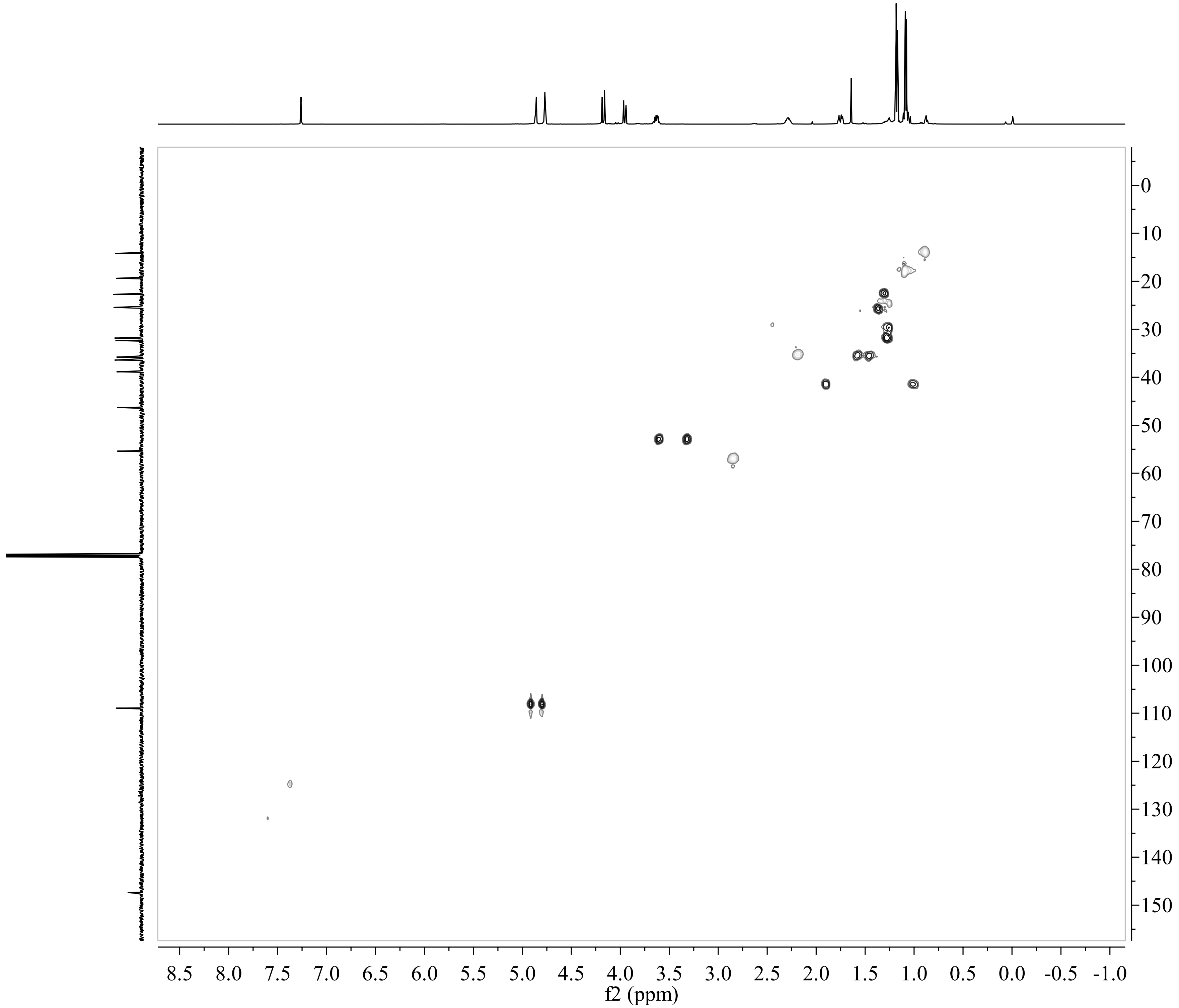

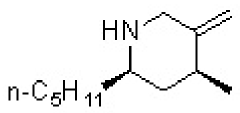

relative configuration

Parameter	Value
Title	xfy-190318-gvv.14.ser
Comment	
Origin	Bruker BioSpin GmbH
Owner	nmr
Site	
Instrument	spect
Solvent	CDCl3
Temperature	296.2
Pulse Sequence	hsqcedetgp
Experiment	HSQC-EDITED
Number of Scans	2
Receiver Gain	193.1
Relaxation Delay	1.4754
Pulse Width	10.7100
Presaturation Frequency	
Acquisition Time	0.1034
Acquisition Date	2019-03-25T08:47:18
Modification Date	2019-09-23T22:11:17
Spectrometer Frequency	(500.13, 125.77)
Spectral Width	(4950.5, 20833.3)
Lowest Frequency	(-588.0, -1037.0)
Nucleus	(1H, 13C)
Acquired Size	(512, 256)
Spectral Size	(512, 512)

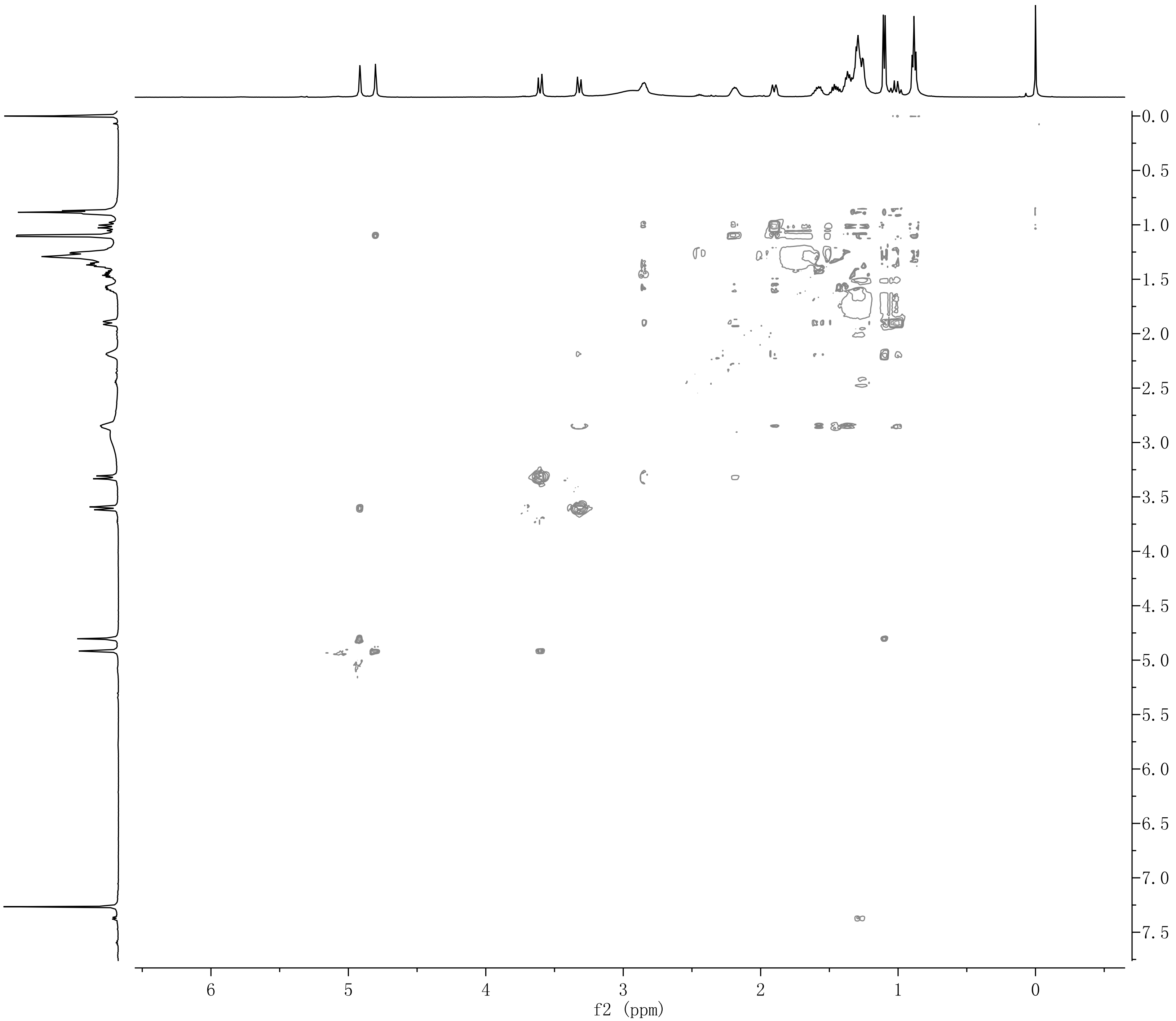

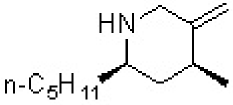

relative configuration

Parameter	Value
Title	xfy-190318-gvv.13.ser
Comment	
Origin	Bruker BioSpin GmbH
Owner	nmr
Site	
Instrument	spect
Solvent	CDCl3
Temperature	296.1
Pulse Sequence	noesygpphpp
Experiment	NOESY
Number of Scans	4
Receiver Gain	48.5
Relaxation Delay	1.9980
Pulse Width	10.7100
Presaturation Frequency	
Acquisition Time	0.2068
Acquisition Date	2019-03-25T08:02:10
Modification Date	2019-04-11T10:18:49
Spectrometer Frequency	(500.13, 500.13)
Spectral Width	(4950.5, 4950.5)
Lowest Frequency	(-588.0, -588.0)
Nucleus	(1H, 1H)
Acquired Size	(1024, 256)
Spectral Size	(1024, 1024)

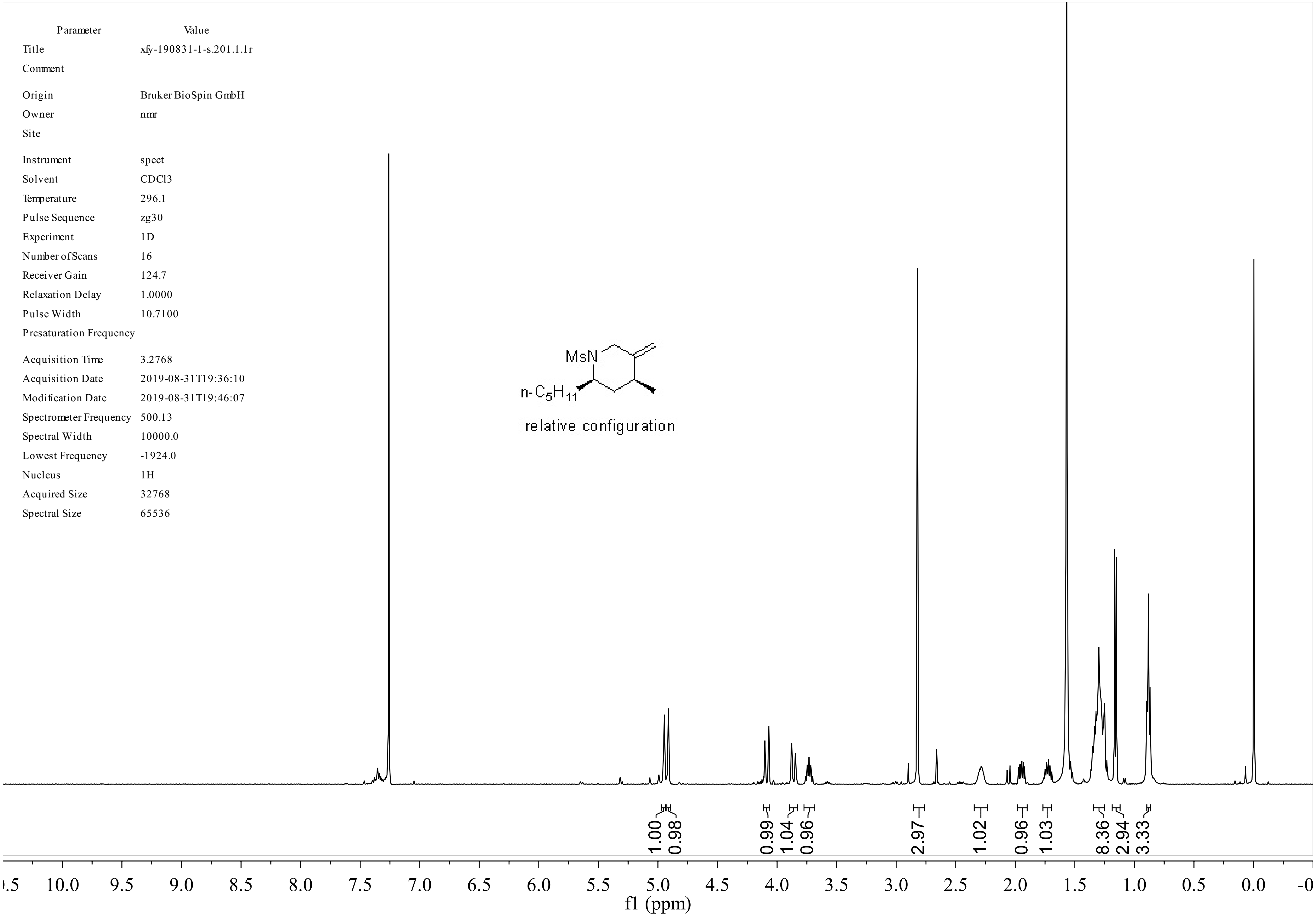

Parameter Value
Title xfy-190831-1-s.201.1.1r
Comment
Origin Bruker BioSpin GmbH
Owner nmr
Site
Instrument spect
Solvent CDCl3
Temperature 296.1
Pulse Sequence zg30
Experiment 1D
Number of Scans 16
Receiver Gain 124.7
Relaxation Delay 1.0000
Pulse Width 10.7100
Presaturation Frequency
Acquisition Time 3.2768
Acquisition Date 2019-08-31T19:36:10
Modification Date 2019-08-31T19:46:07
Spectrometer Frequency 500.13
Spectral Width 10000.0
Lowest Frequency -1924.0
Nucleus 1H
Acquired Size 32768
Spectral Size 65536
MsN
n-C5H11
relative configuration
1.00
0.98
0.99
1.04
0.96
2.97
1.02
0.96
1.03
8.36
2.94
3.33
).5 10.0 9.5 9.0 8.5 8.0 7.5 7.0 6.5 6.0 5.5 5.0 4.5 4.0 3.5 3.0 2.5 2.0 1.5 1.0 0.5 0.0 -0
f1 (ppm)

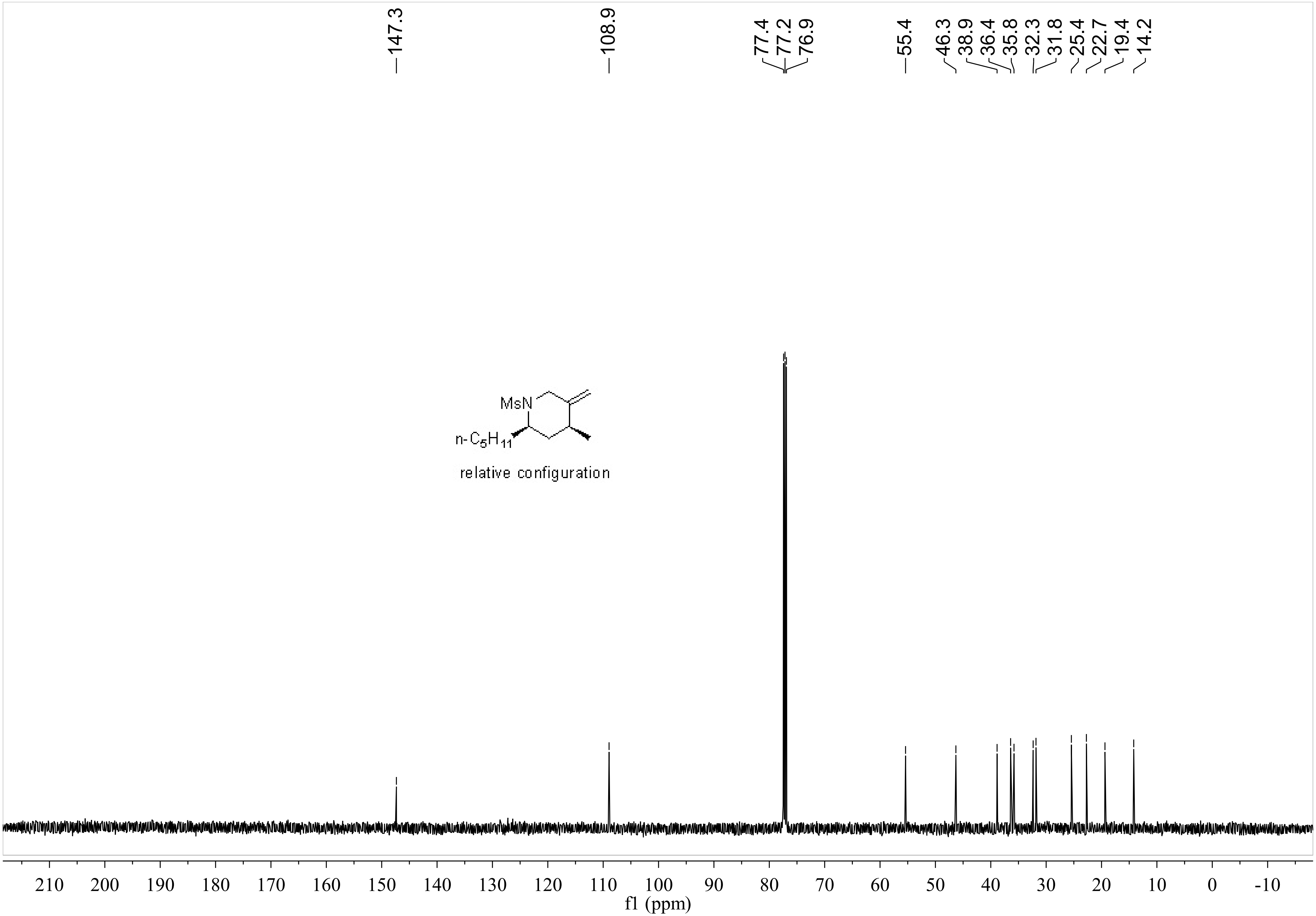
147.3
108.9
77.4
77.2
76.9
55.4
46.3
38.9
36.4
35.8
32.3
31.8
25.4
22.7
19.4
14.2
MsN
n-C5H11
relative configuration
210 200 190 180 170 160 150 140 130 120 110 100 90 80 70 60 50 40 30 20 10 0 -10
f1 (ppm)

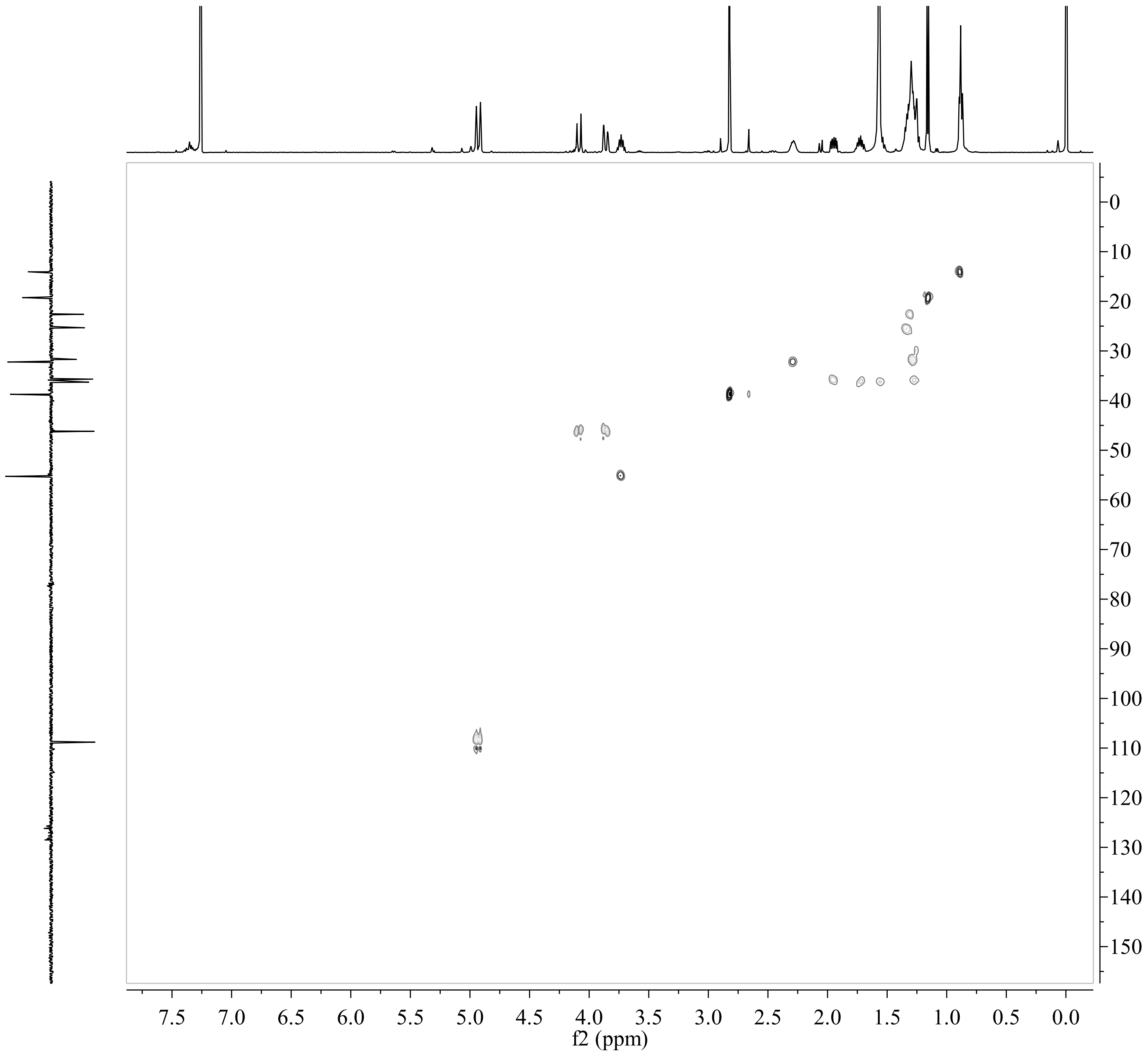

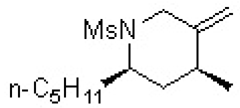

relative configuration

Parameter	Value
Title	xfy-190831-1-s.112.ser
Comment	
Origin	Bruker BioSpin GmbH
Owner	nmr
Site	
Instrument	spect
Solvent	CDCl3
Temperature	296.1
Pulse Sequence	hsqcedetgp
Experiment	HSQC-EDITED
Number of Scans	2
Receiver Gain	193.1
Relaxation Delay	1.4529
Pulse Width	10.7100
Presaturation Frequency	
Acquisition Time	0.1260
Acquisition Date	2019-09-02T12:04:13
Modification Date	2019-09-02T16:31:33
Spectrometer Frequency	(500.13, 125.77)
Spectral Width	(4065.0, 20833.3)
Lowest Frequency	(-123.0, -1037.0)
Nucleus	(1H, 13C)
Acquired Size	(512, 221)
Spectral Size	(512, 512)

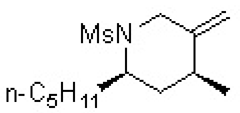

relative configuration

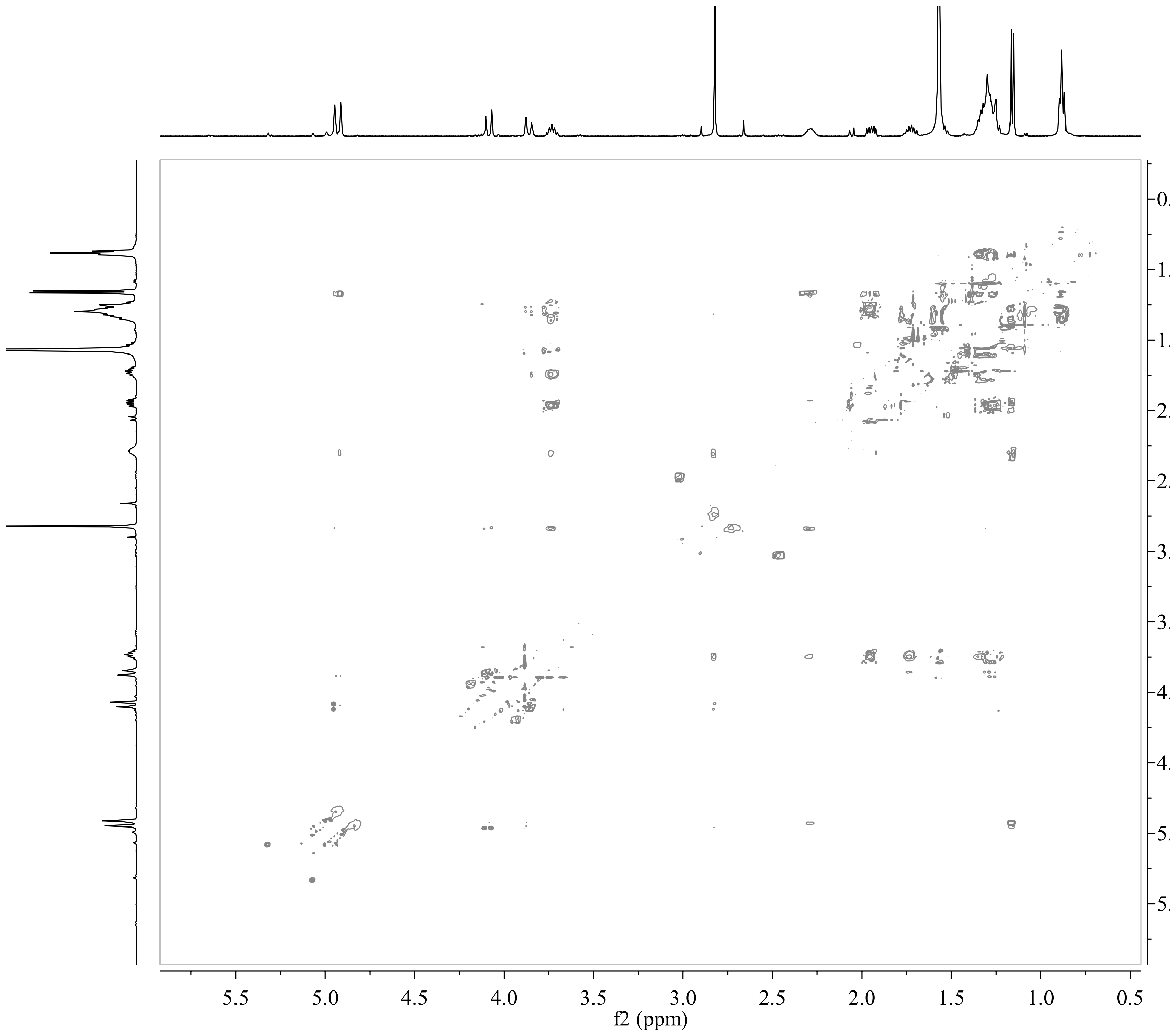

Parameter	Value
Title	xfy-190831-1-s.402.ser
Comment	
Origin	Bruker BioSpin GmbH
Owner	nmr
Site	
Instrument	spect
Solvent	CDCl3
Temperature	297.2
Pulse Sequence	noesygpphpp
Experiment	NOESY
Number of Scans	20
Receiver Gain	62.3
Relaxation Delay	1.9672
Pulse Width	8.7300
Presaturation Frequency	
Acquisition Time	0.2888
Acquisition Date	2019-09-01T22:14:04
Modification Date	2019-09-02T10:00:21
Spectrometer Frequency	(400.13, 400.13)
Spectral Width	(3546.1, 3546.1)
Lowest Frequency	(-339.6, -339.6)
Nucleus	(1H, 1H)
Acquired Size	(1024, 256)
Spectral Size	(1024, 1024)

Parameter	Value
Title	gvv-e-160fl1.1.fid
Comment	
Origin	Bruker BioSpin GmbH
Owner	nmr
Site	
Instrument	spect
Solvent	CDCl3
Temperature	296.2
Pulse Sequence	zg30
Experiment	1D
Number of Scans	16
Receiver Gain	77.6
Relaxation Delay	1.0000
Pulse Width	10.7100
Presaturation Frequency	
Acquisition Time	3.2768
Acquisition Date	2018-12-24T17:46:09
Modification Date	2018-12-25T19:27:04
Spectrometer Frequency	500.13
Spectral Width	10000.0
Lowest Frequency	-1931.5
Nucleus	1H
Acquired Size	32768
Spectral Size	65536

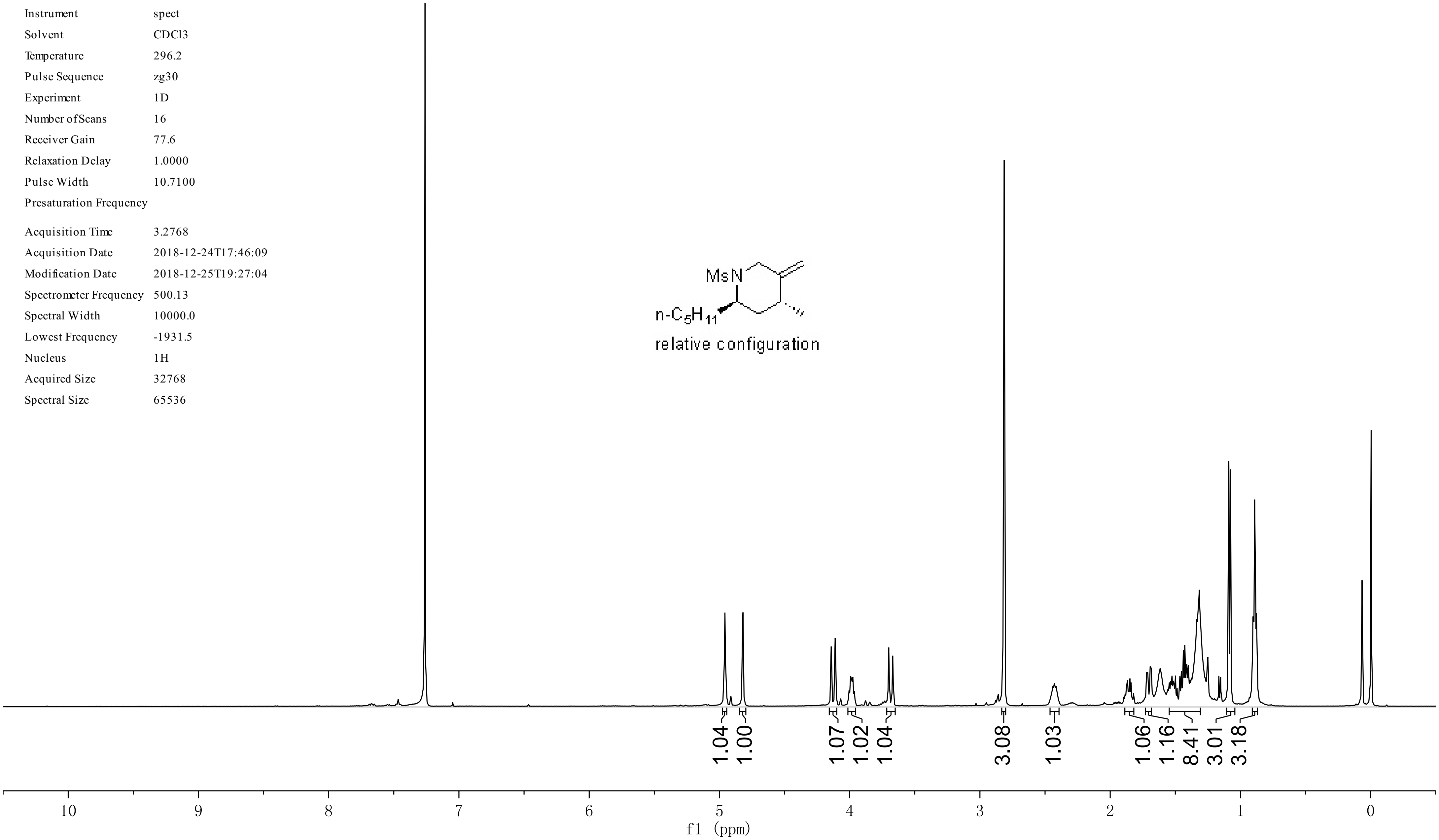

Parameter	Value
Title	gvv-e-160f11-1.5.fid
Comment	
Origin	Bruker BioSpin GmbH
Owner	nmr
Site	
Instrument	spect
Solvent	CDCl3
Temperature	296.1
Pulse Sequence	zgpg30
Experiment	1D
Number of Scans	500
Receiver Gain	193.1
Relaxation Delay	2.0000
Pulse Width	9.6000
Presaturation Frequency	
Acquisition Time	1.1010
Acquisition Date	2018-12-26T09:58:44
Modification Date	2018-12-26T13:55:59
Spectrometer Frequency	125.77
Spectral Width	29761.9
Lowest Frequency	-2289.2
Nucleus	13C
Acquired Size	32768
Spectral Size	65536

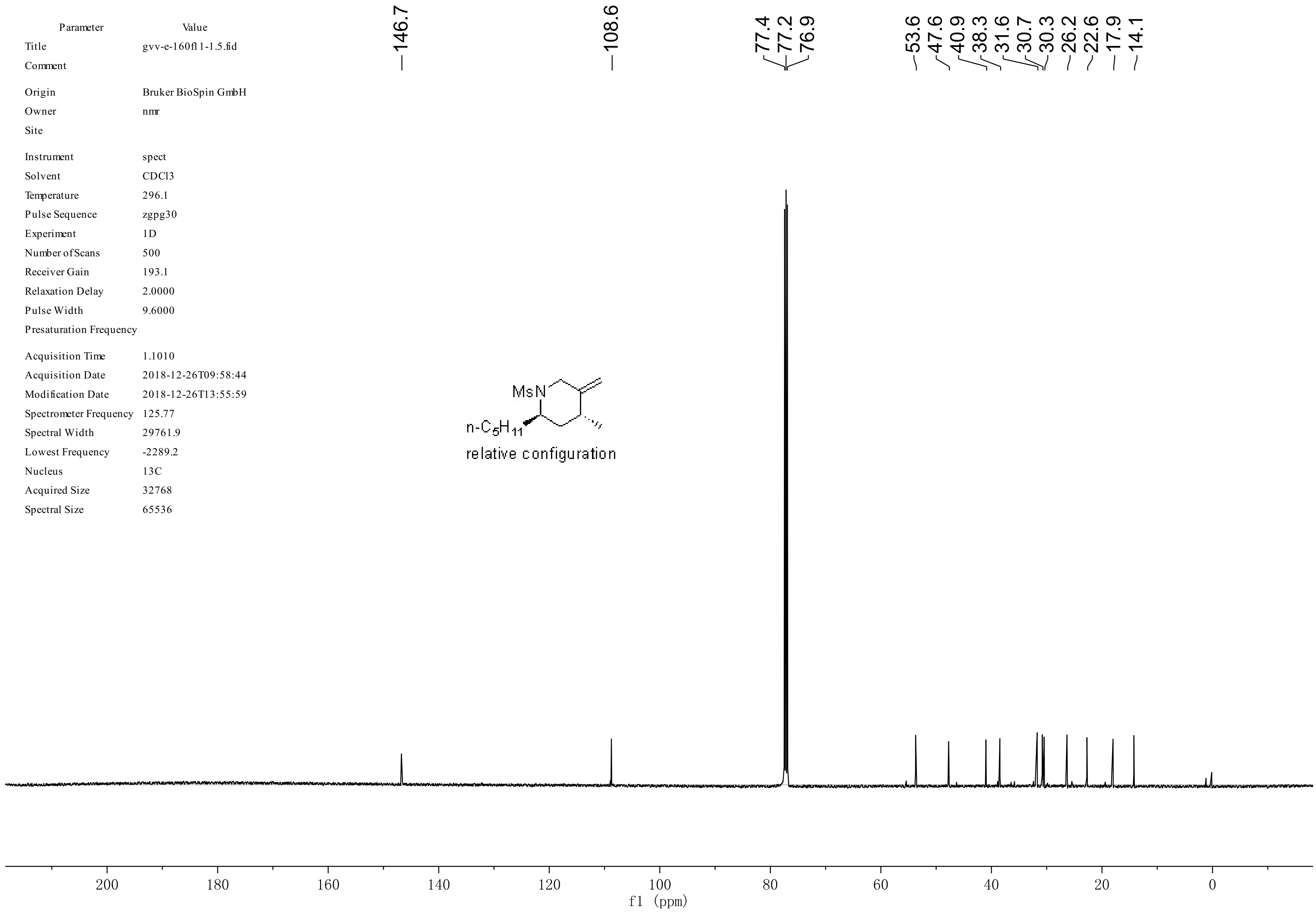

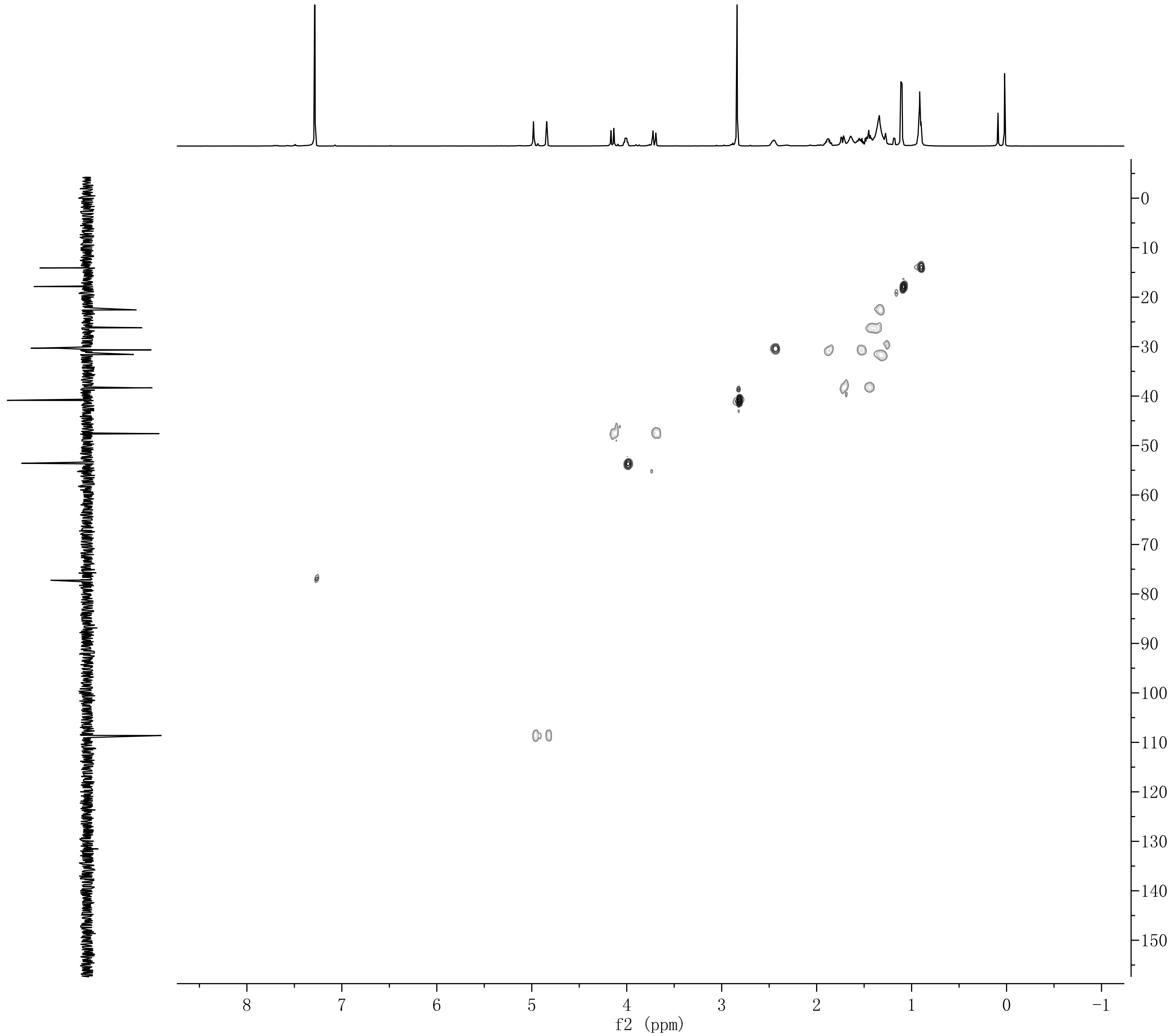

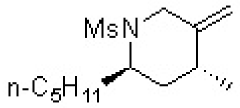

relative configuration

Parameter	Value
Title	gvv-e-160fl1-1.7.ser
Comment	
Origin	Bruker BioSpin GmbH
Owner	nmr
Site	
Instrument	spect
Solvent	CDCl3
Temperature	296.1
Pulse Sequence	hsqcedetgp
Experiment	HSQC-EDITED
Number of Scans	2
Receiver Gain	193.1
Relaxation Delay	1.4764
Pulse Width	10.7100
Presaturation Frequency	
Acquisition Time	0.1024
Acquisition Date	2018-12-26T10:02:33
Modification Date	2018-12-26T13:56:00
Spectrometer Frequency	(500.13, 125.77)
Spectral Width	(5000.0, 20833.3)
Lowest Frequency	(-630.1, -1037.0)
Nucleus	(1H, 13C)
Acquired Size	(512, 256)
Spectral Size	(512, 512)

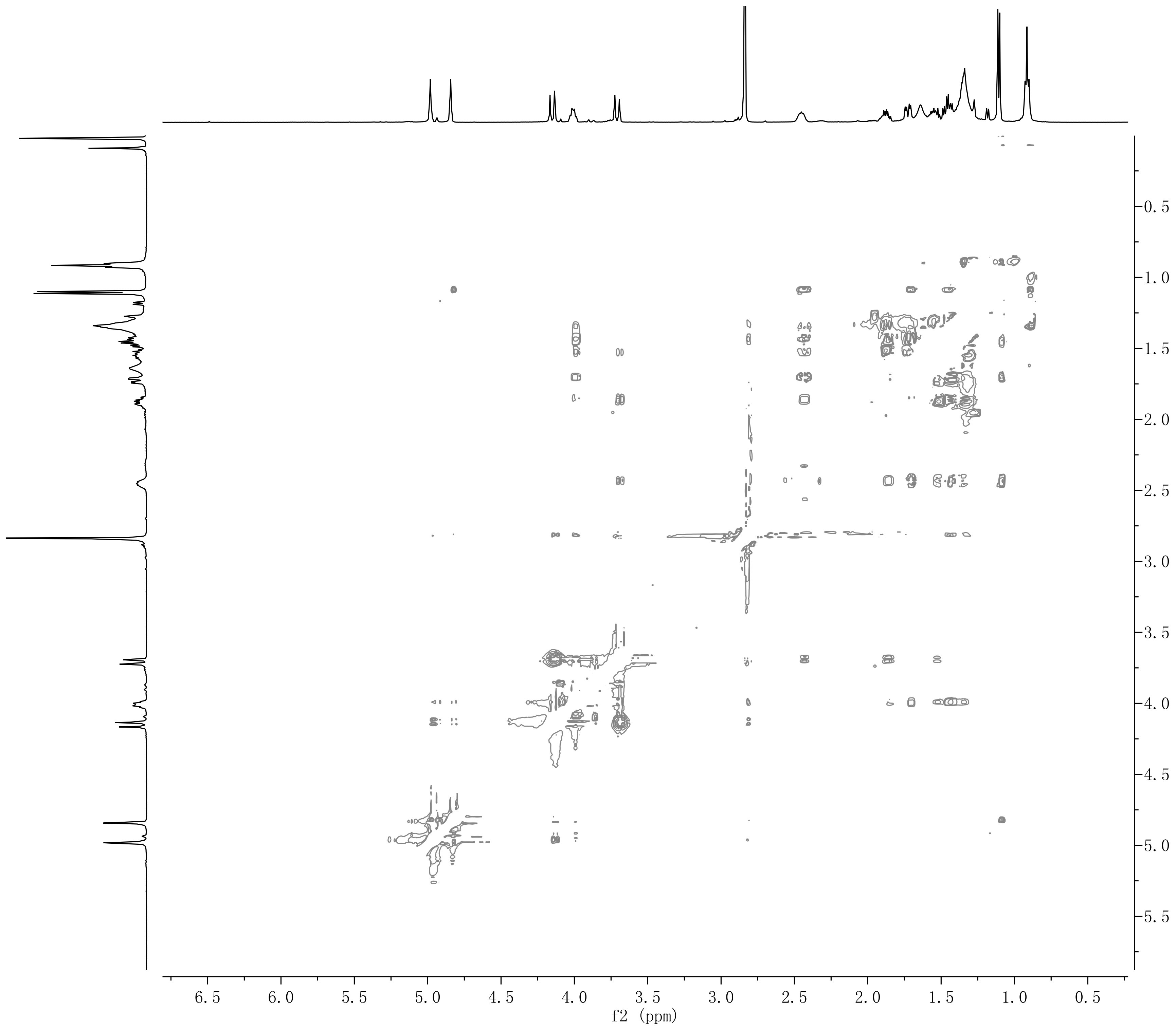

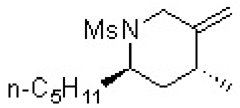

relative configuration

Parameter	Value
Title	gvv-e-160fl1.4.ser
Comment	
Origin	Bruker BioSpin GmbH
Owner	nmr
Site	
Instrument	spect
Solvent	CDCl3
Temperature	296.2
Pulse Sequence	noesygpphpp
Experiment	NOESY
Number of Scans	4
Receiver Gain	48.5
Relaxation Delay	1.9795
Pulse Width	10.7100
Presaturation Frequency	
Acquisition Time	0.2253
Acquisition Date	2018-12-24T18:01:48
Modification Date	2018-12-25T19:27:05
Spectrometer Frequency	(500.13, 500.13)
Spectral Width	(4545.5, 4545.5)
Lowest Frequency	(-343.1, -343.1)
Nucleus	(1H, 1H)
Acquired Size	(1024, 256)
Spectral Size	(1024, 1024)

Parameter	Value
Title	gvv-e-149f-full.1.fid
Comment	
Origin	Bruker BioSpin GmbH
Owner	nmr
Site	
Instrument	spect
Solvent	CDCl3
Temperature	295.8
Pulse Sequence	zg30
Experiment	1D
Number of Scans	8
Receiver Gain	62.3
Relaxation Delay	1.0000
Pulse Width	10.0000
Presaturation Frequency	
Acquisition Time	1.9999
Acquisition Date	2018-11-27T15:30:54
Modification Date	2018-11-27T19:23:12
Spectrometer Frequency	400.13
Spectral Width	8012.8
Lowest Frequency	-1549.4
Nucleus	1H
Acquired Size	16025
Spectral Size	65536

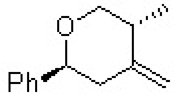

relative configuration

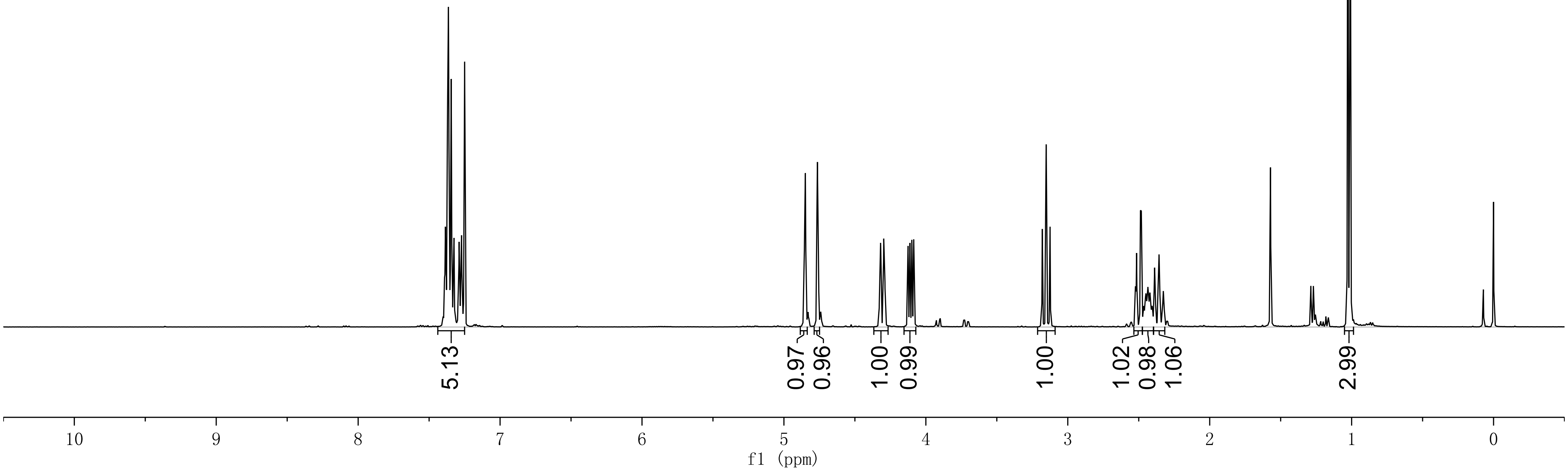

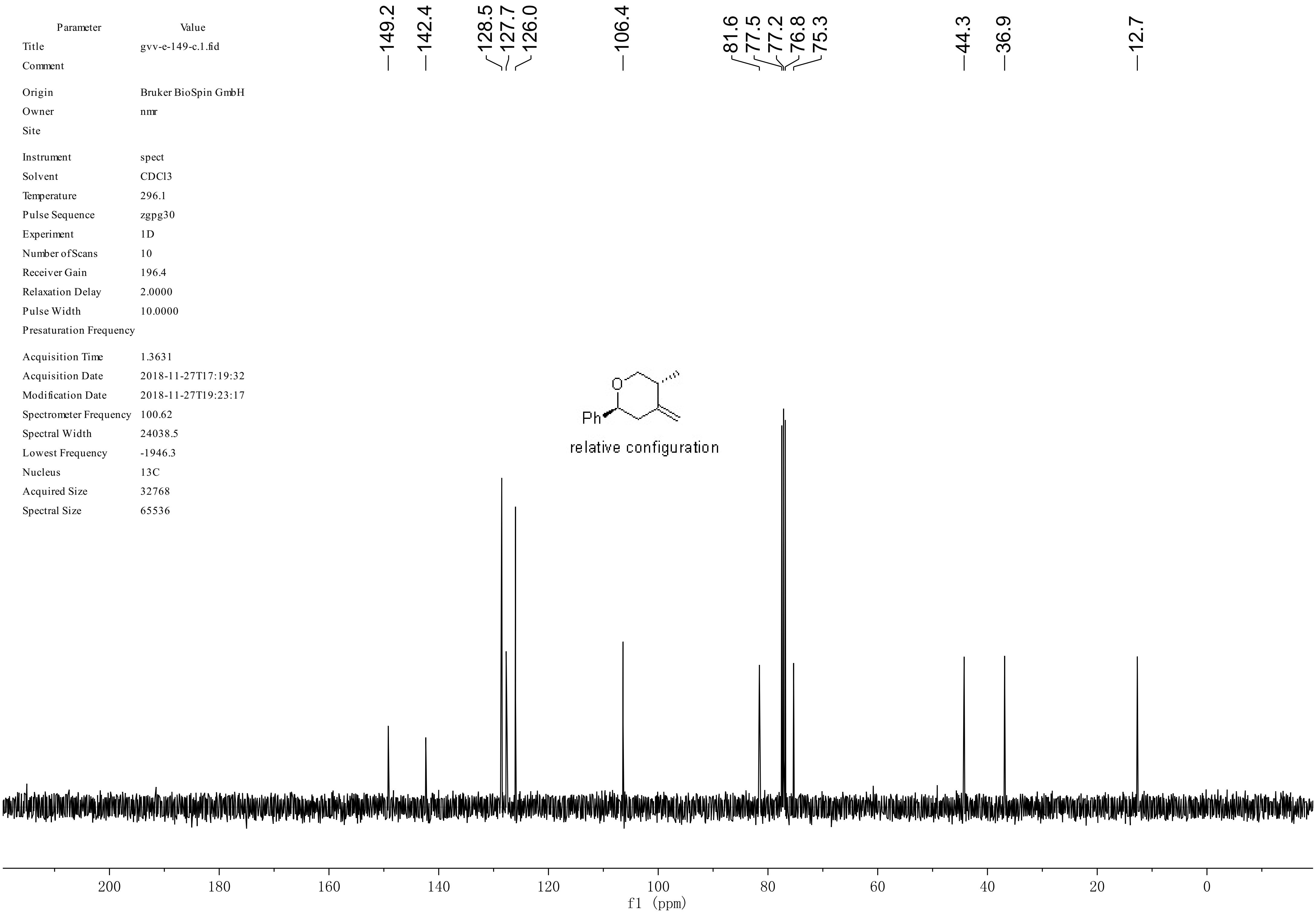

Parameter	Value
Title	gvv-e-149-c.1.fid
Comment	
Origin	Bruker BioSpin GmbH
Owner	nmr
Site	
Instrument	spect
Solvent	CDCl3
Temperature	296.1
Pulse Sequence	zgpg30
Experiment	1D
Number of Scans	10
Receiver Gain	196.4
Relaxation Delay	2.0000
Pulse Width	10.0000
Presaturation Frequency	
Acquisition Time	1.3631
Acquisition Date	2018-11-27T17:19:32
Modification Date	2018-11-27T19:23:17
Spectrometer Frequency	100.62
Spectral Width	24038.5
Lowest Frequency	-1946.3
Nucleus	13C
Acquired Size	32768
Spectral Size	65536

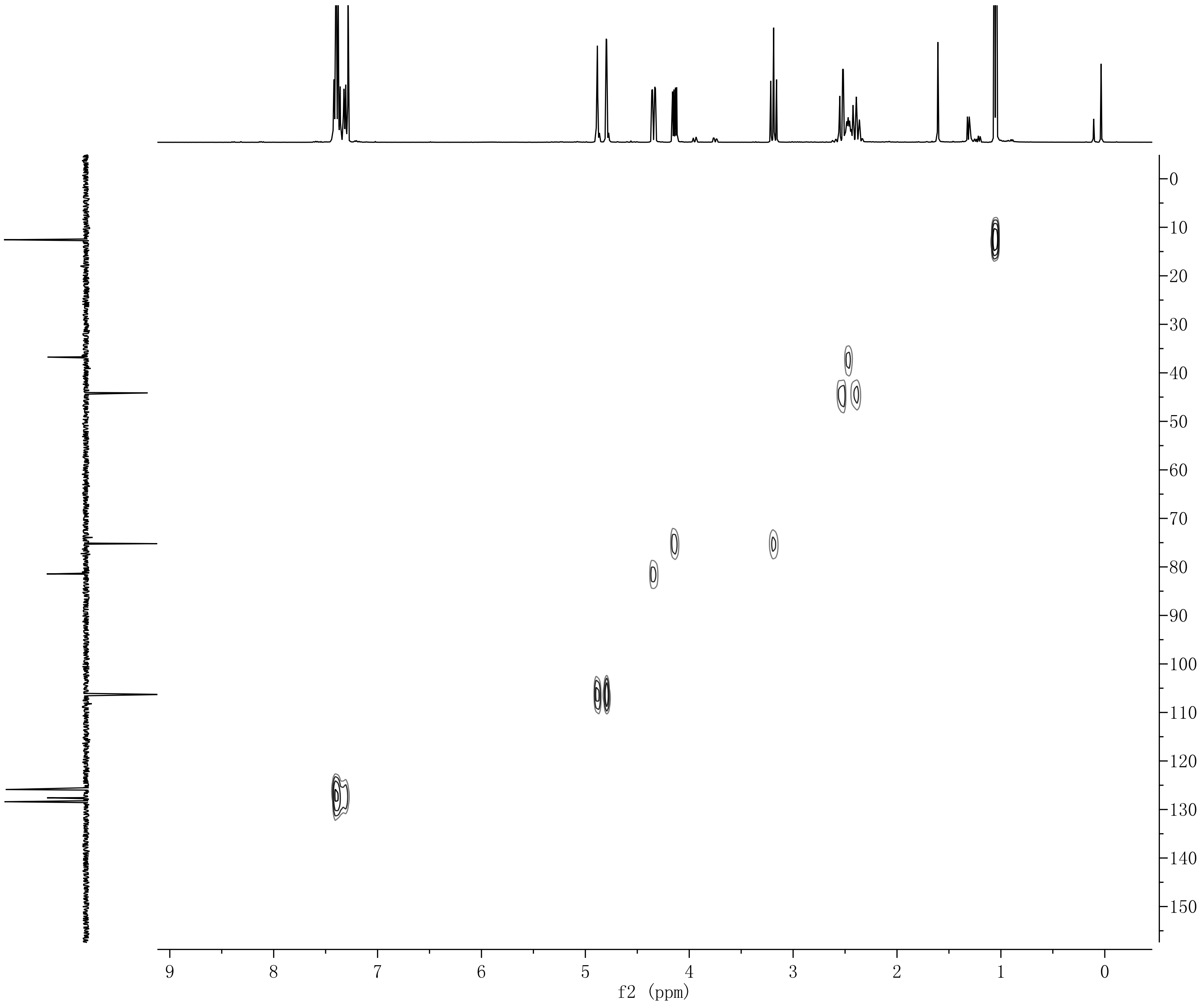

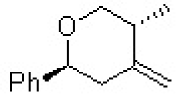

relative configuration

Parameter	Value
Comment	
Origin	Bruker BioSpin GmbH
Owner	nmr
Site	
Instrument	spect
Solvent	CDCl3
Temperature	295.9
Pulse Sequence	hsqcetgp
Experiment	HSQC
Number of Scans	2
Receiver Gain	196.4
Relaxation Delay	1.5000
Pulse Width	10.0000
Presaturation Frequency	
Acquisition Time	0.0985
Acquisition Date	2018-11-27T16:33:00
Modification Date	2018-11-27T19:23:13
Spectrometer Frequency	(400.13, 100.62)
Spectral Width	(5197.5, 16666.7)
Lowest Frequency	(-193.1, -829.1)
Nucleus	(1H, 13C)
Acquired Size	(512, 65)
Spectral Size	(512, 512)

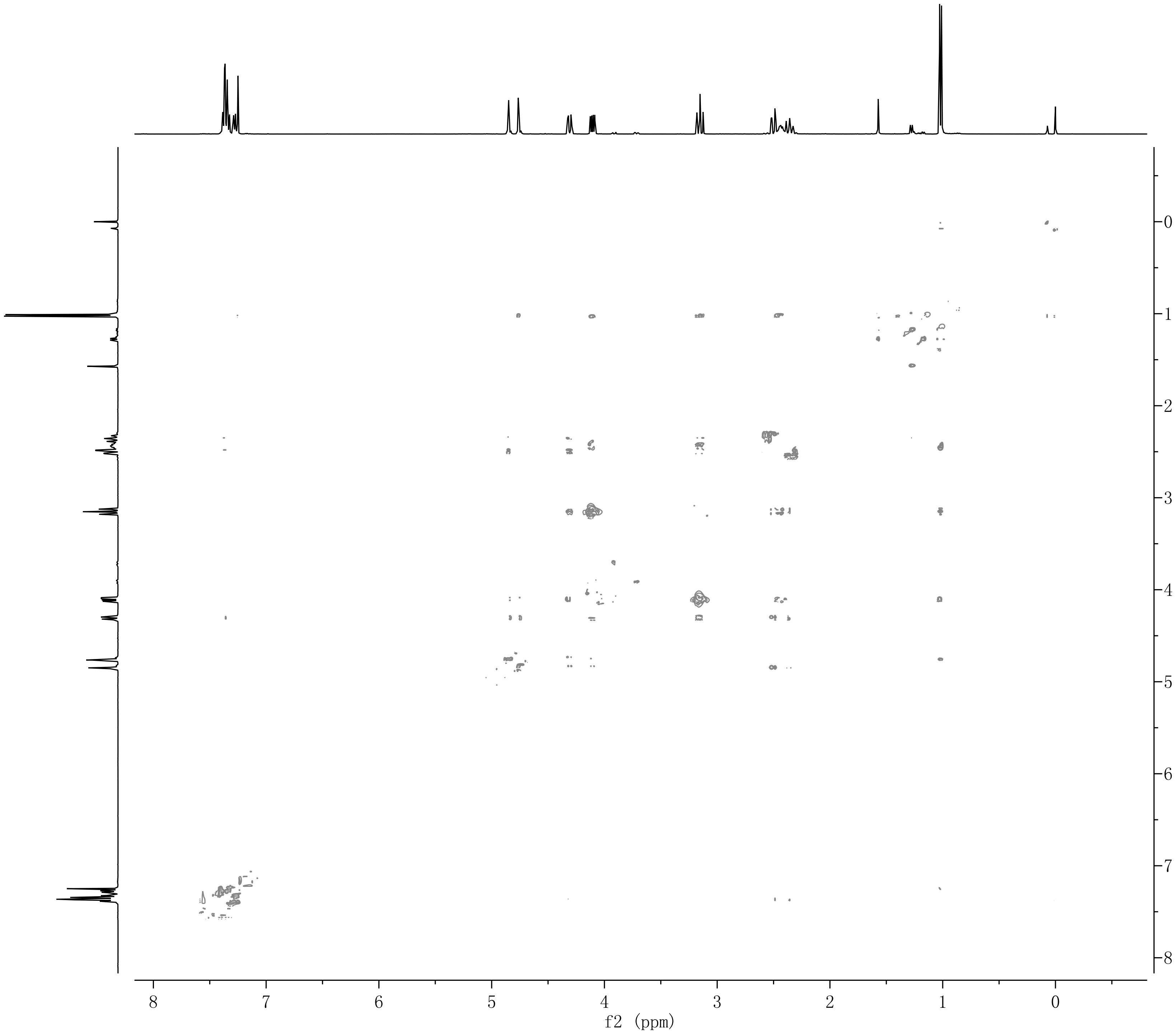

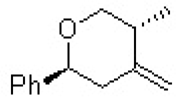

relative configuration

Parameter	Value
Comment	
Origin	Bruker BioSpin GmbH
Owner	nmr
Site	
Instrument	spect
Solvent	CDCl3
Temperature	295.9
Pulse Sequence	noesygpphpp
Experiment	NOESY
Number of Scans	4
Receiver Gain	49.4
Relaxation Delay	1.9713
Pulse Width	10.0000
Presaturation Frequency	
Acquisition Time	0.2847
Acquisition Date	2018-11-27T15:37:00
Modification Date	2018-11-27T19:23:14
Spectrometer Frequency	(400.13, 400.13)
Spectral Width	(3597.1, 3597.1)
Lowest Frequency	(-315.3, -315.3)
Nucleus	(1H, 1H)
Acquired Size	(1024, 220)
Spectral Size	(1024, 1024)

Parameter	Value
Comment	
Origin	Bruker BioSpin GmbH
Owner	nmrsu
Site	
Instrument	Avance NEO 600
Solvent	CDCl3
Temperature	296.9
Pulse Sequence	zg30
Experiment	1D
Number of Scans	16
Receiver Gain	92.3
Relaxation Delay	1.0000
Pulse Width	10.0000
Presaturation Frequency	
Acquisition Time	2.7525
Acquisition Date	2018-11-11T21:41:24
Modification Date	2018-11-14T12:27:36
Spectrometer Frequency	600.15
Spectral Width	11904.8
Lowest Frequency	-2260.8
Nucleus	1H
Acquired Size	32768
Spectral Size	65536

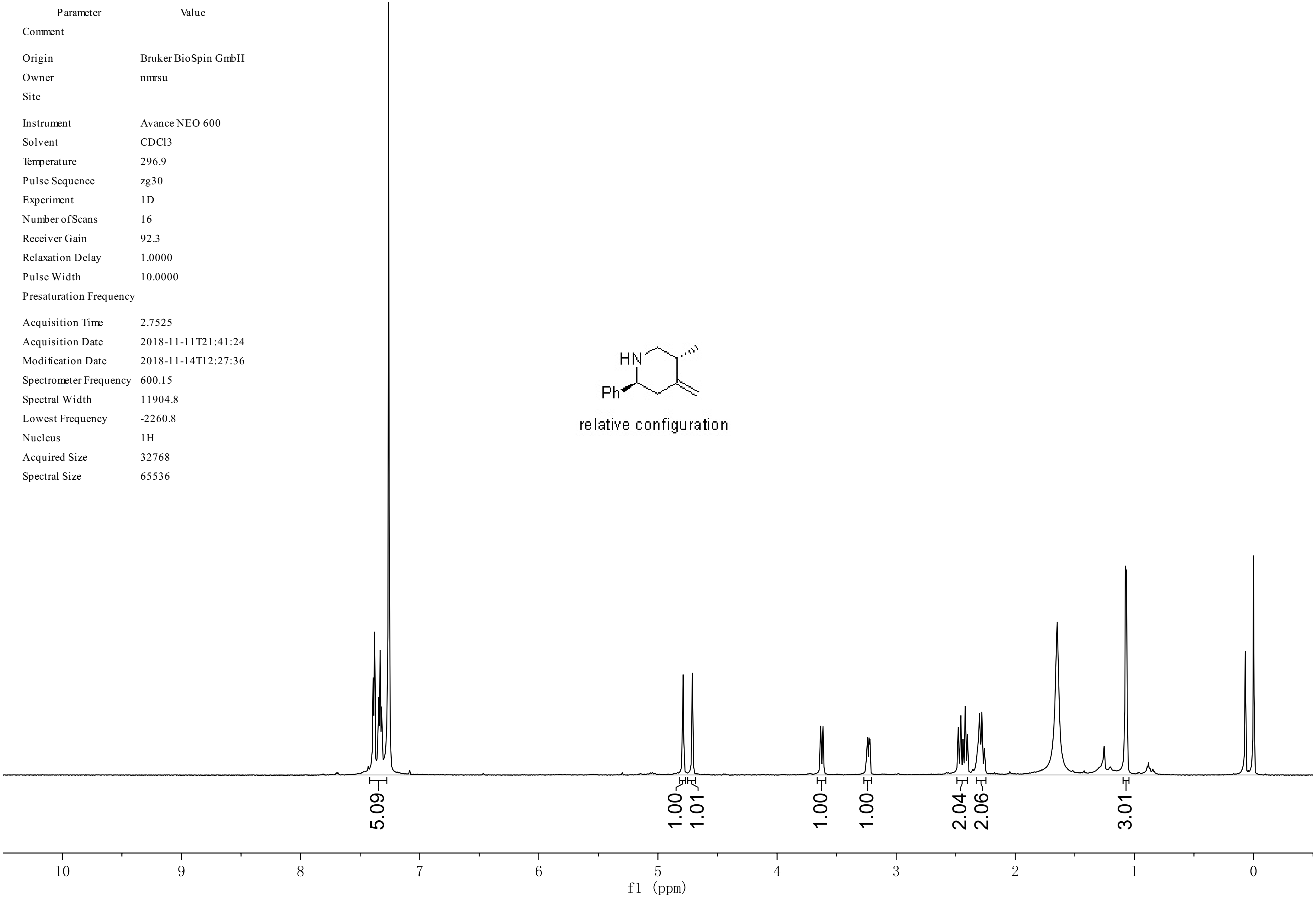

Parameter	Value
Comment	
Origin	Bruker BioSpin GmbH
Owner	nmr
Site	
Instrument	spect
Solvent	CDCl3
Temperature	293.7
Pulse Sequence	zgpg30
Experiment	1D
Number of Scans	142
Receiver Gain	196.4
Relaxation Delay	2.0000
Pulse Width	10.0000
Presaturation Frequency	
Acquisition Time	1.3631
Acquisition Date	2018-11-03T09:22:14
Modification Date	2018-11-04T20:42:09
Spectrometer Frequency	100.62
Spectral Width	24038.5
Lowest Frequency	-1945.4
Nucleus	13C
Acquired Size	32768
Spectral Size	65536

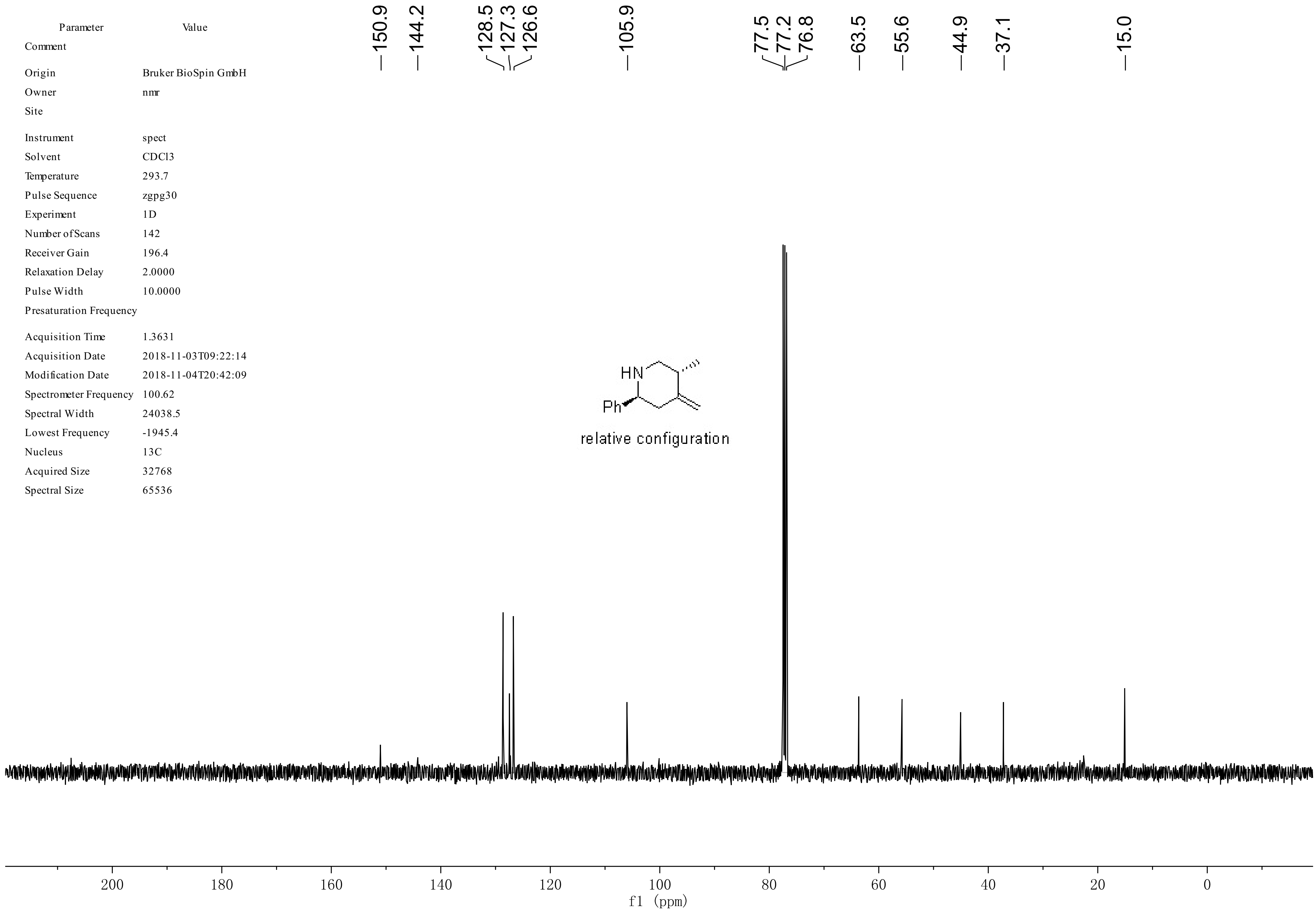

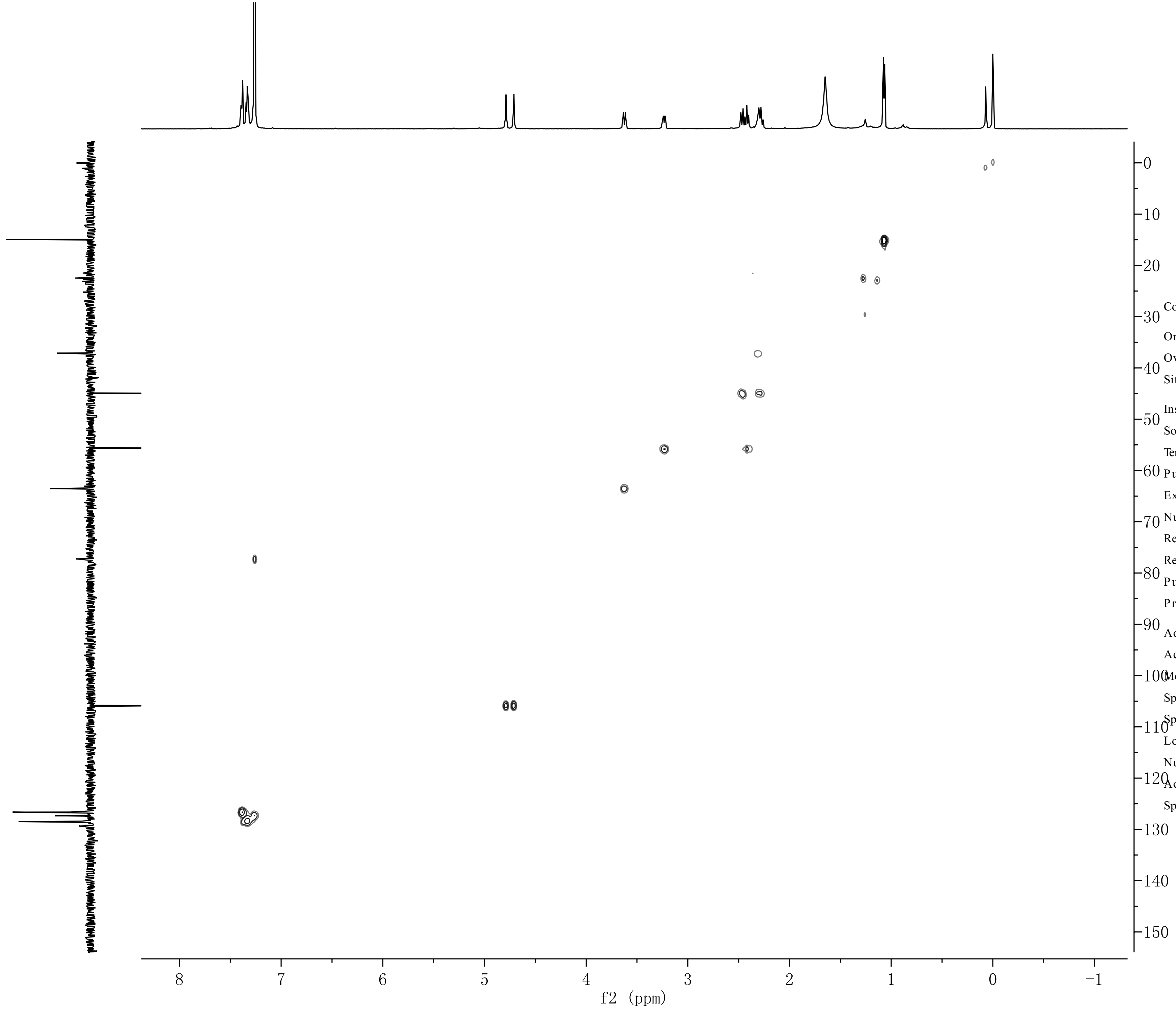

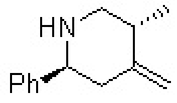

relative configuration

Parameter	Value
Comment	
Origin	Bruker BioSpin GmbH
Owner	nmr
Site	
Instrument	spect
Solvent	CDCl3
Temperature	293.8
Pulse Sequence	hsqcetgp
Experiment	HSQC
Number of Scans	2
Receiver Gain	196.4
Relaxation Delay	1.4736
Pulse Width	10.0000
Presaturation Frequency	
Acquisition Time	0.1249
Acquisition Date	2018-11-03T09:37:37
Modification Date	2018-11-04T20:42:09
Spectrometer Frequency	(400.13, 100.62)
Spectral Width	(4098.4, 16666.7)
Lowest Frequency	(-592.7, -829.1)
Nucleus	(1H, 13C)
Acquired Size	(512, 256)
Spectral Size	(512, 512)

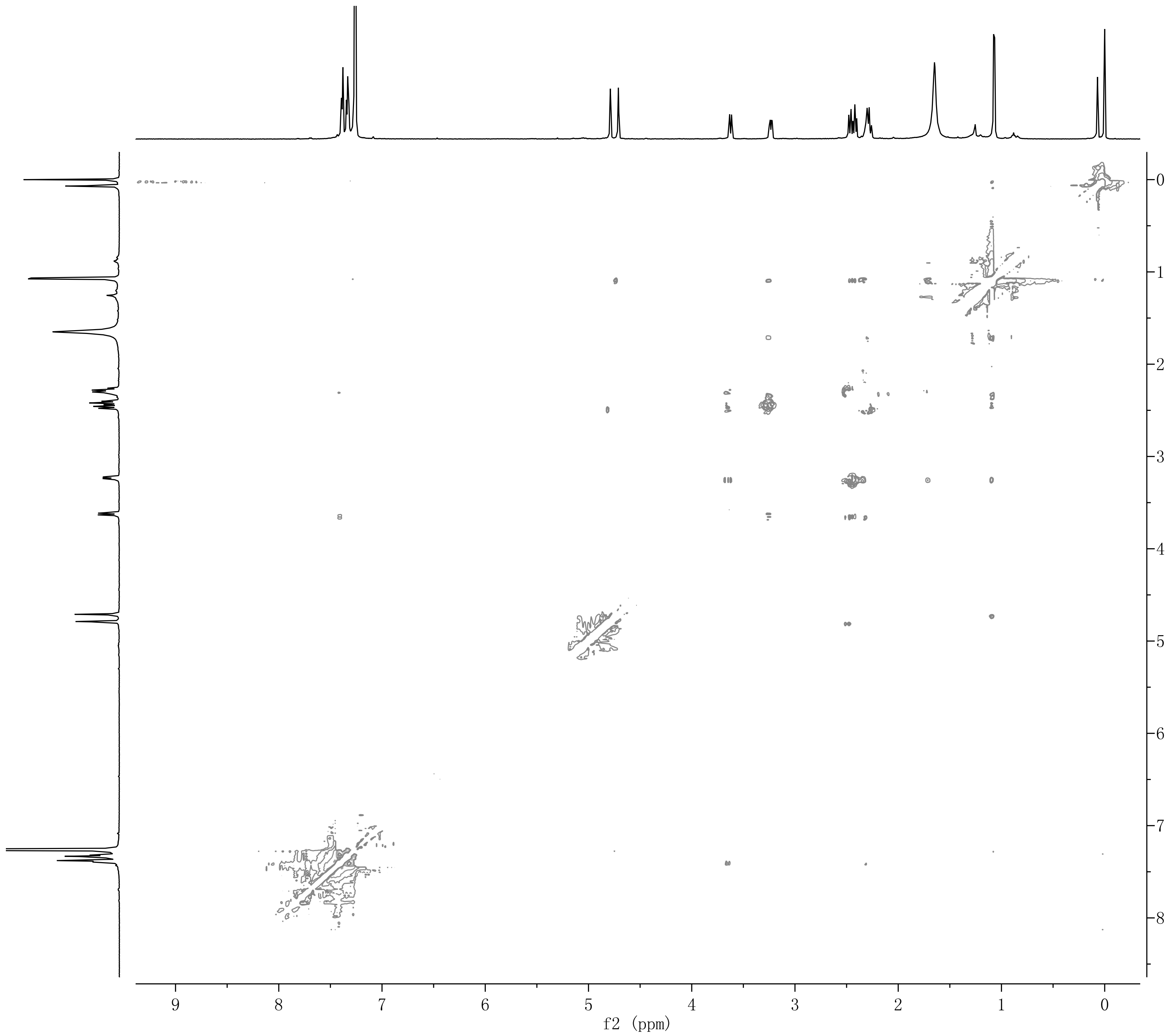

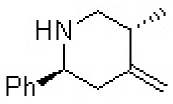

relative configuration

Parameter	Value
Comment	
Origin	Bruker BioSpin GmbH
Owner	nmr
Site	
Instrument	spect
Solvent	CDCl3
Temperature	293.5
Pulse Sequence	noesygpphpp
Experiment	NOESY
Number of Scans	4
Receiver Gain	62.3
Relaxation Delay	2.0000
Pulse Width	10.0000
Presaturation Frequency	
Acquisition Time	0.2560
Acquisition Date	2018-11-21T09:23:08
Modification Date	2018-11-21T12:09:12
Spectrometer Frequency	(400.13, 400.13)
Spectral Width	(4000.0, 4000.0)
Lowest Frequency	(-158.1, -158.1)
Nucleus	(1H, 1H)
Acquired Size	(1024, 242)
Spectral Size	(1024, 1024)

Parameter	Value
Title	xfy-190809-6.31.1.1r
Comment	
Origin	Bruker BioSpin GmbH
Owner	nmr
Site	
Instrument	spect
Solvent	CDCl3
Temperature	296.1
Pulse Sequence	zg30
Experiment	1D
Number of Scans	16
Receiver Gain	95.3
Relaxation Delay	1.0000
Pulse Width	10.7100
Presaturation Frequency	
Acquisition Time	3.2768
Acquisition Date	2019-08-11T11:32:49
Modification Date	2019-08-11T12:19:26
Spectrometer Frequency	500.13
Spectral Width	10000.0
Lowest Frequency	-1924.0
Nucleus	1H
Acquired Size	32768
Spectral Size	65536

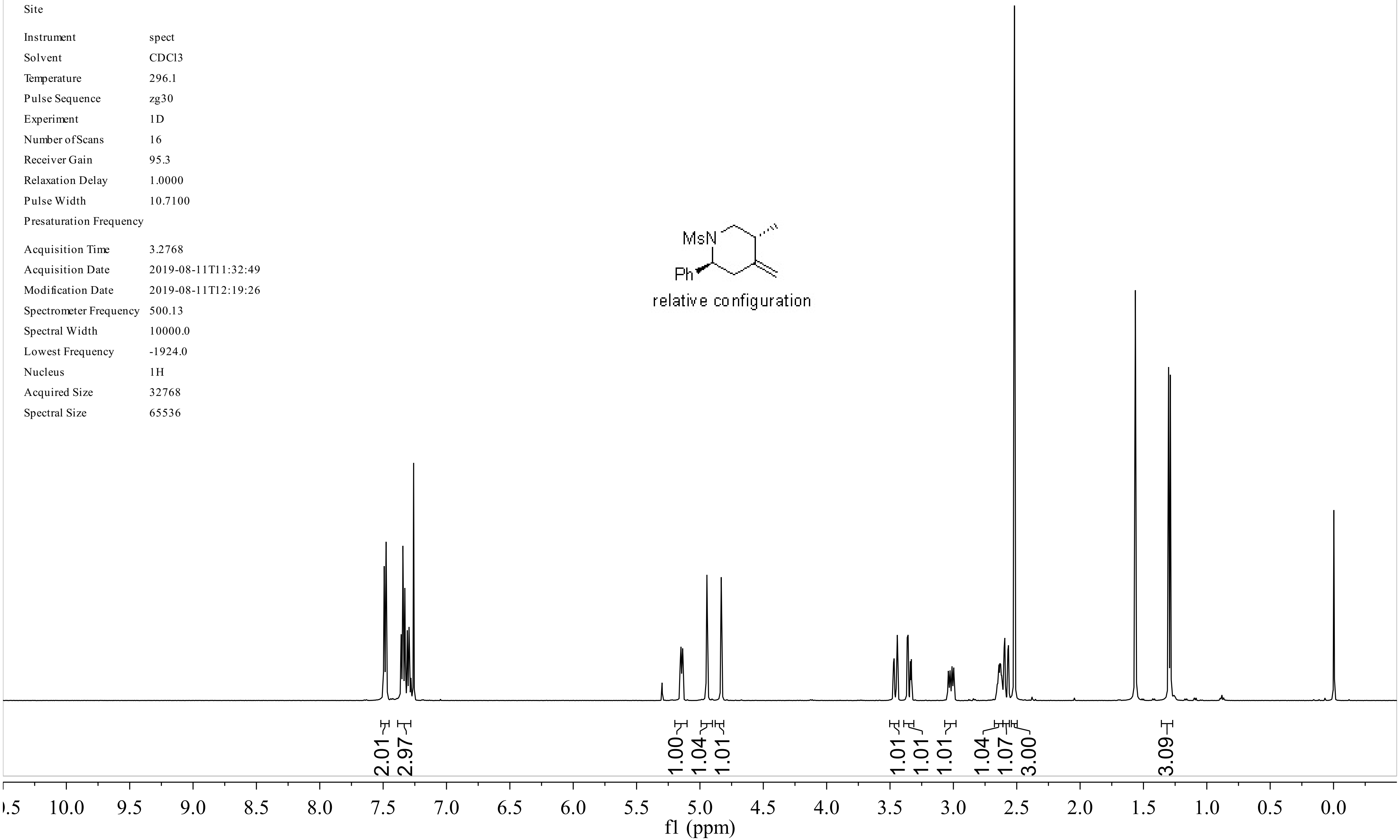

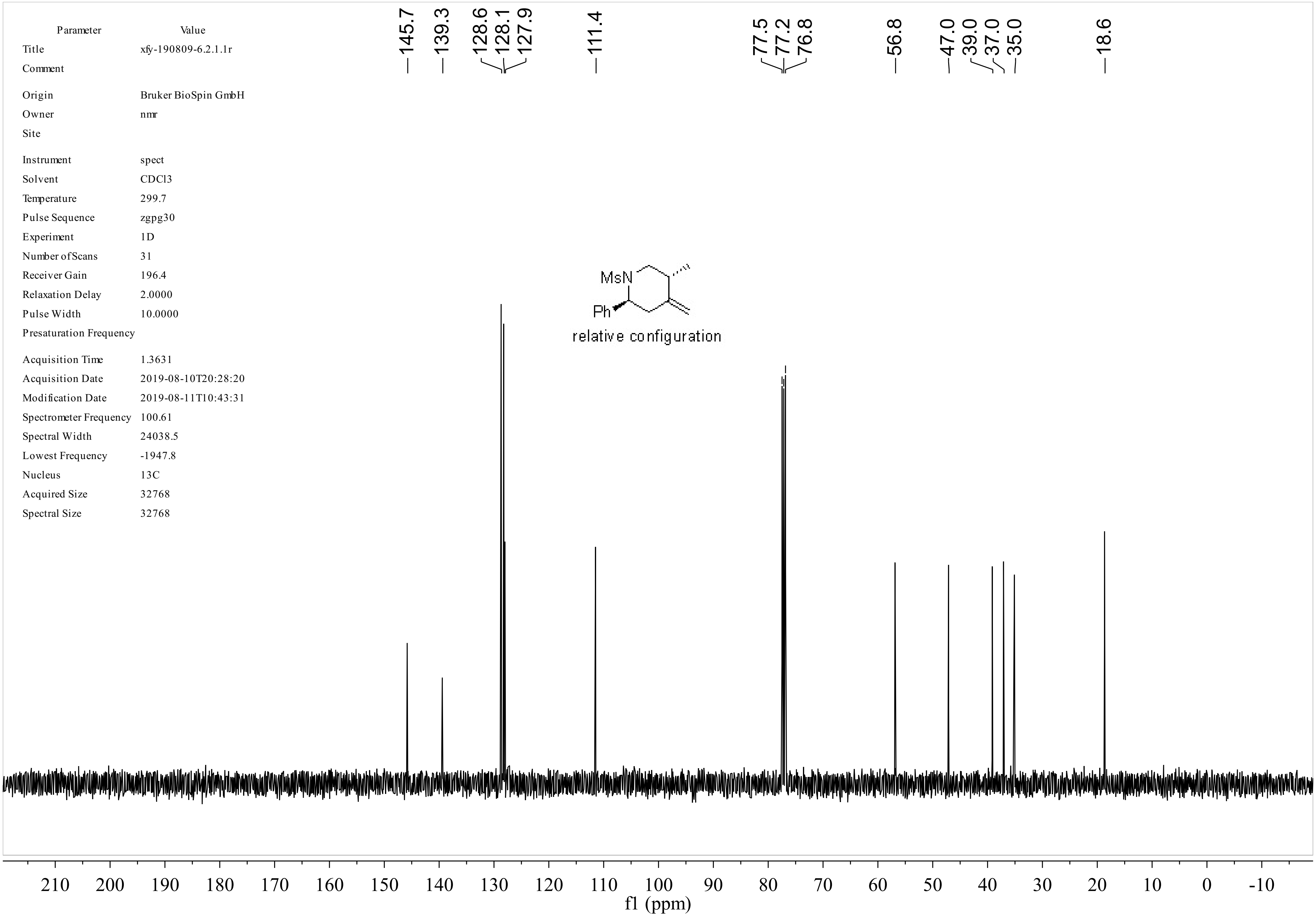

Parameter Value
Title xfy-190809-6.2.1.1r
Comment
Origin Bruker BioSpin GmbH
Owner nmr
Site
Instrument spect
Solvent CDCl3
Temperature 299.7
Pulse Sequence zgpg30
Experiment 1D
Number of Scans 31
Receiver Gain 196.4
Relaxation Delay 2.0000
Pulse Width 10.0000
Presaturation Frequency
Acquisition Time 1.3631
Acquisition Date 2019-08-10T20:28:20
Modification Date 2019-08-11T10:43:31
Spectrometer Frequency 100.61
Spectral Width 24038.5
Lowest Frequency -1947.8
Nucleus 13C
Acquired Size 32768
Spectral Size 32768
145.7
139.3
128.6
128.1
127.9
111.4
77.5
77.2
76.8
56.8
47.0
39.0
37.0
35.0
18.6
MsN
Ph
relative configuration
210 200 190 180 170 160 150 140 130 120 110 100 90 80 70 60 50 40 30 20 10 0 -10
f1 (ppm)

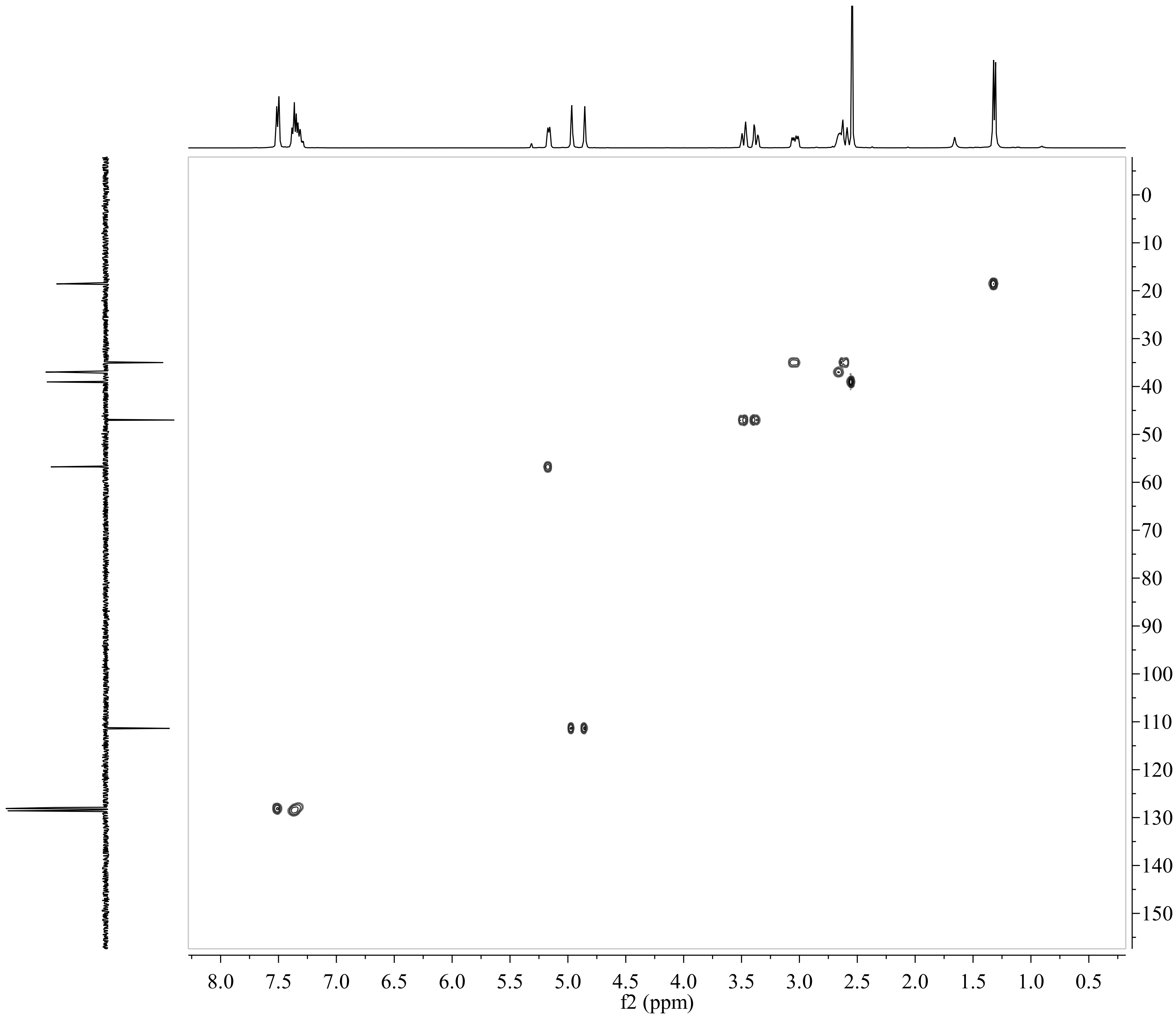

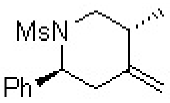

relative configuration

Parameter	Value
Title	xfy-190809-6.14.ser
Comment	
Origin	Bruker BioSpin GmbH
Owner	nmr
Site	
Instrument	spect
Solvent	CDCl3
Temperature	299.6
Pulse Sequence	hsqcetgp
Experiment	HSQC
Number of Scans	8
Receiver Gain	196.4
Relaxation Delay	1.4408
Pulse Width	8.7300
Presaturation Frequency	
Acquisition Time	0.1577
Acquisition Date	2019-08-10T23:10:08
Modification Date	2019-08-11T10:43:30
Spectrometer Frequency	(400.13, 100.62)
Spectral Width	(3246.8, 16666.7)
Lowest Frequency	(54.3, -829.1)
Nucleus	(1H, 13C)
Acquired Size	(512, 256)
Spectral Size	(512, 512)

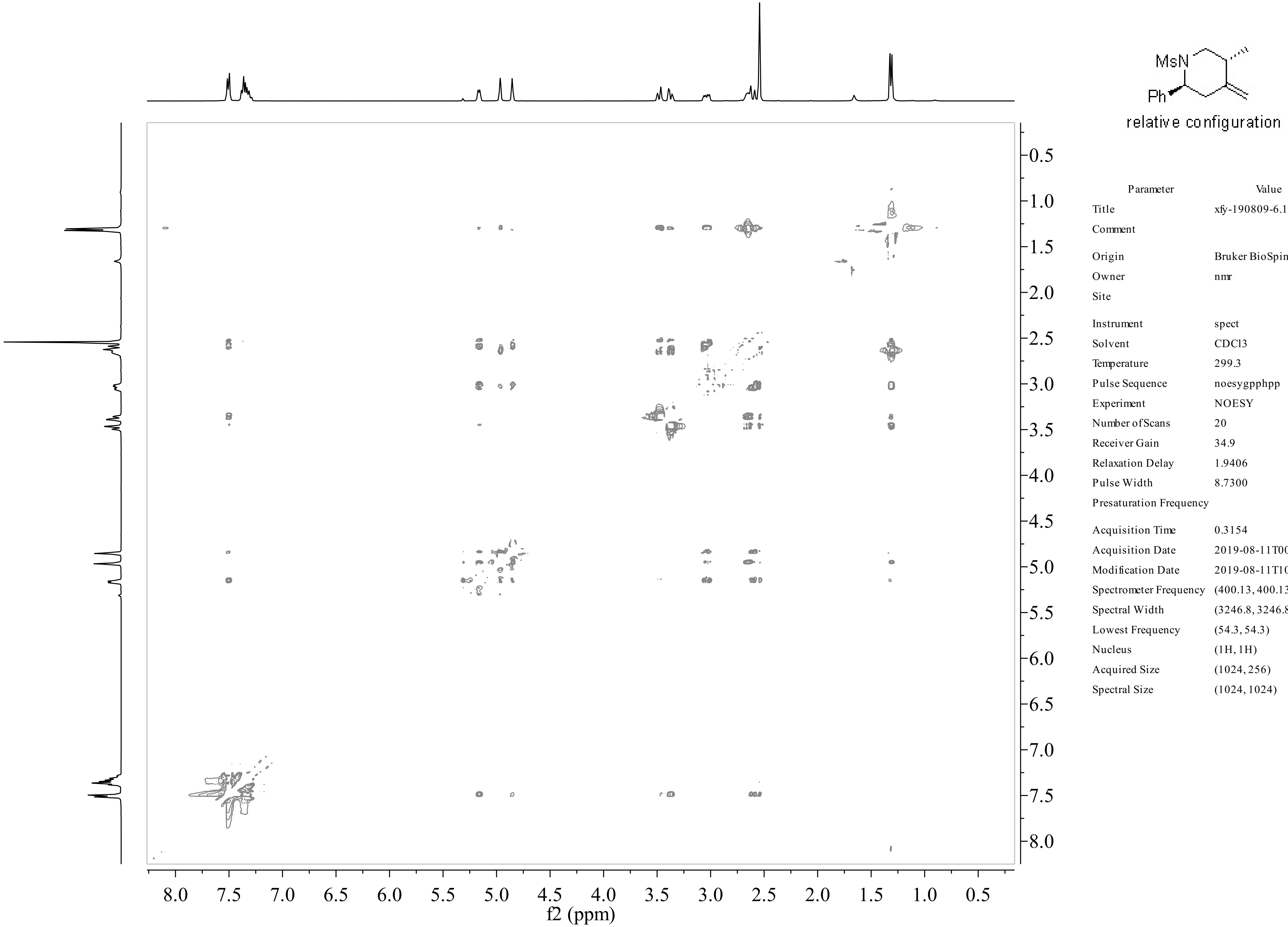

Parameter	Value
Title	xfy-190809-6.15.ser
Comment	
Origin	Bruker BioSpin GmbH
Owner	nmr
Site	
Instrument	spect
Solvent	CDCl3
Temperature	299.3
Pulse Sequence	noesygpphpp
Experiment	NOESY
Number of Scans	20
Receiver Gain	34.9
Relaxation Delay	1.9406
Pulse Width	8.7300
Presaturation Frequency	
Acquisition Time	0.3154
Acquisition Date	2019-08-11T00:08:10
Modification Date	2019-08-11T10:43:30
Spectrometer Frequency	(400.13, 400.13)
Spectral Width	(3246.8, 3246.8)
Lowest Frequency	(54.3, 54.3)
Nucleus	(1H, 1H)
Acquired Size	(1024, 256)
Spectral Size	(1024, 1024)

Parameter	Value
Comment	
Origin	Bruker BioSpin GmbH
Owner	nmr
Site	
Instrument	spect
Solvent	CDCl3
Temperature	296.2
Pulse Sequence	zg30
Experiment	1D
Number of Scans	16
Receiver Gain	87.5
Relaxation Delay	1.0000
Pulse Width	10.7100
Presaturation Frequency	
Acquisition Time	3.2768
Acquisition Date	2018-11-01T12:25:31
Modification Date	2018-11-05T15:21:40
Spectrometer Frequency	500.13
Spectral Width	10000.0
Lowest Frequency	-1911.5
Nucleus	1H
Acquired Size	32768
Spectral Size	65536

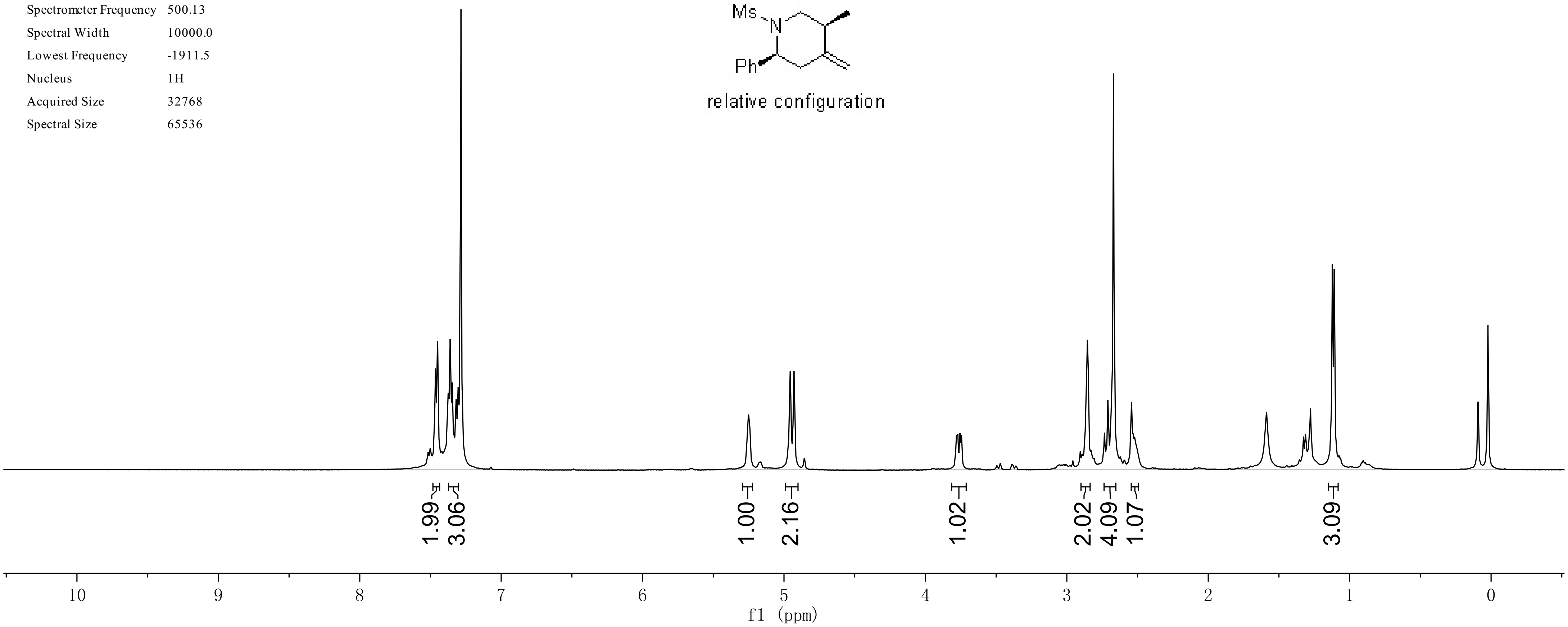

Parameter	Value
Comment	
Origin	Bruker BioSpin GmbH
Owner	nmr
Site	
Instrument	spect
Solvent	CDCl3
Temperature	296.1
Pulse Sequence	zgpg30
Experiment	1D
Number of Scans	176
Receiver Gain	193.1
Relaxation Delay	2.0000
Pulse Width	9.6000
Presaturation Frequency	
Acquisition Time	1.1010
Acquisition Date	2018-11-01T12:36:46
Modification Date	2018-11-05T15:21:40
Spectrometer Frequency	125.77
Spectral Width	29761.9
Lowest Frequency	-2289.2
Nucleus	13C
Acquired Size	32768
Spectral Size	65536

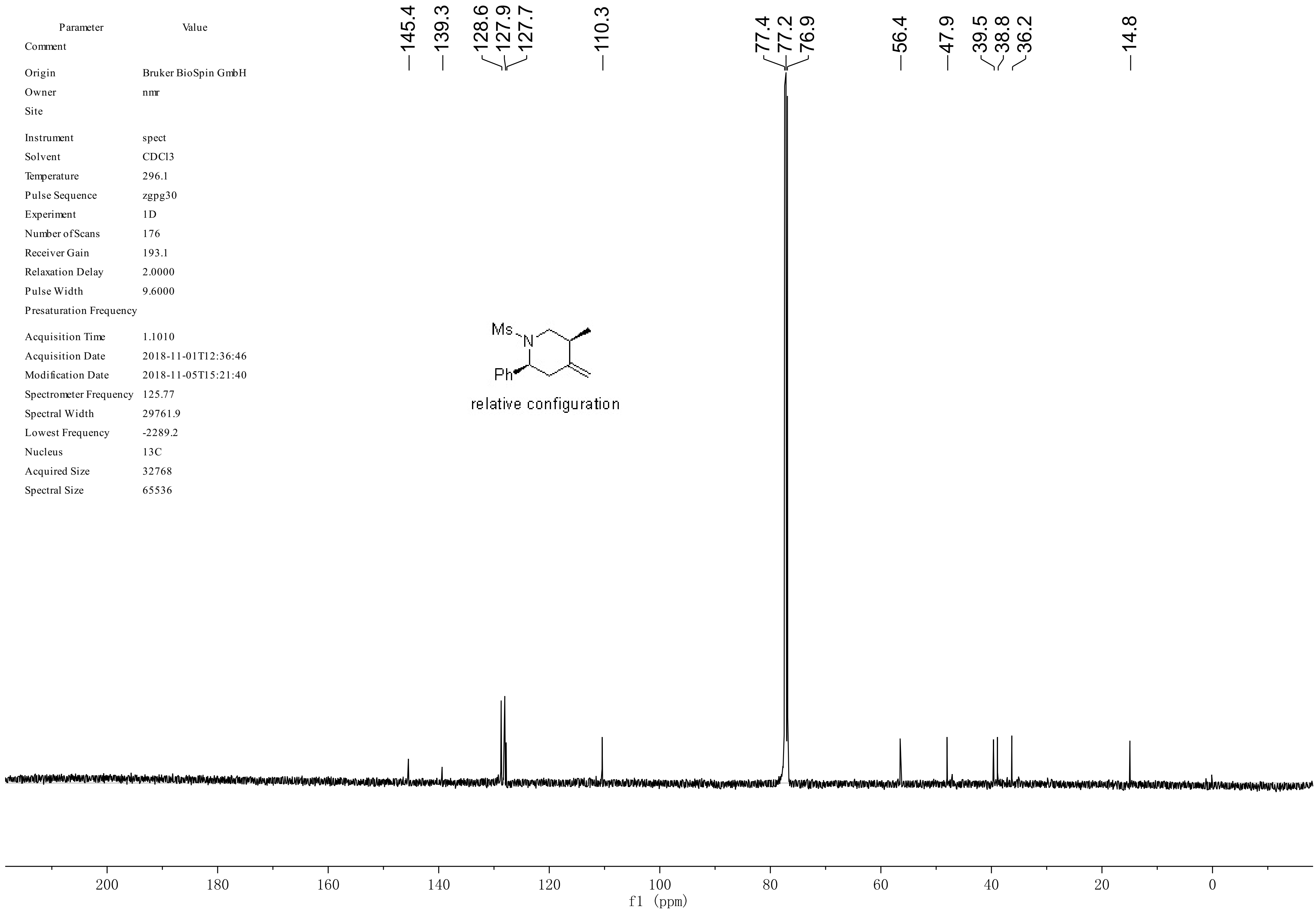

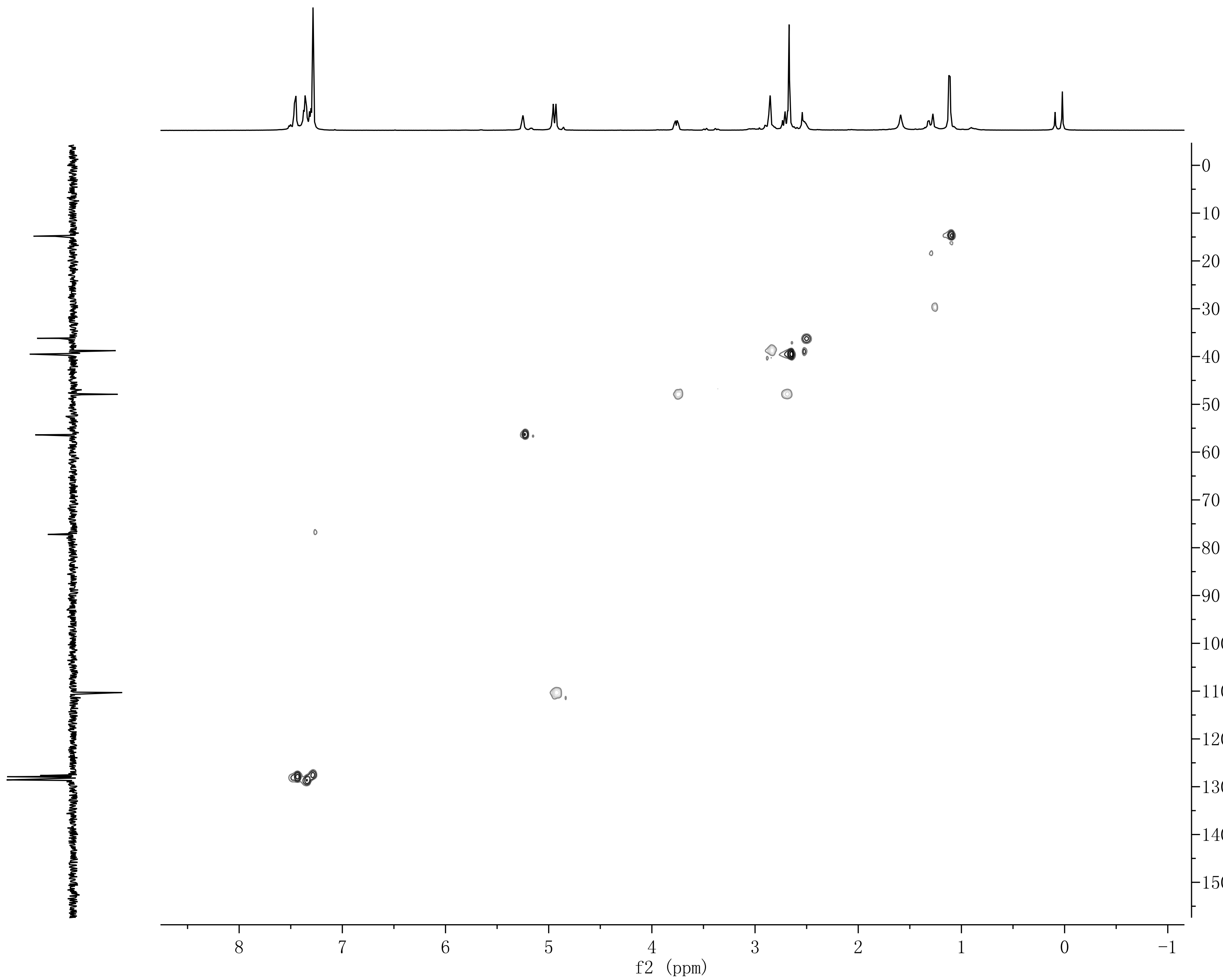

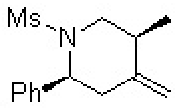

relative configuration

Parameter	Value
Comment	
Origin	Bruker BioSpin GmbH
Owner	nmr
Site	
Instrument	spect
Solvent	CDCl3
Temperature	296.1
Pulse Sequence	hsqcedetgp
Experiment	HSQC-EDITED
Number of Scans	2
Receiver Gain	193.1
Relaxation Delay	1.4785
Pulse Width	10.7100
Presaturation Frequency	
Acquisition Time	0.1004
Acquisition Date	2018-11-01T12:58:28
Modification Date	2018-11-05T15:21:43
Spectrometer Frequency	(500.13, 125.77)
Spectral Width	(5102.0, 20833.3)
Lowest Frequency	(-590.9, -1037.0)
Nucleus	(1H, 13C)
Acquired Size	(512, 256)
Spectral Size	(512, 512)

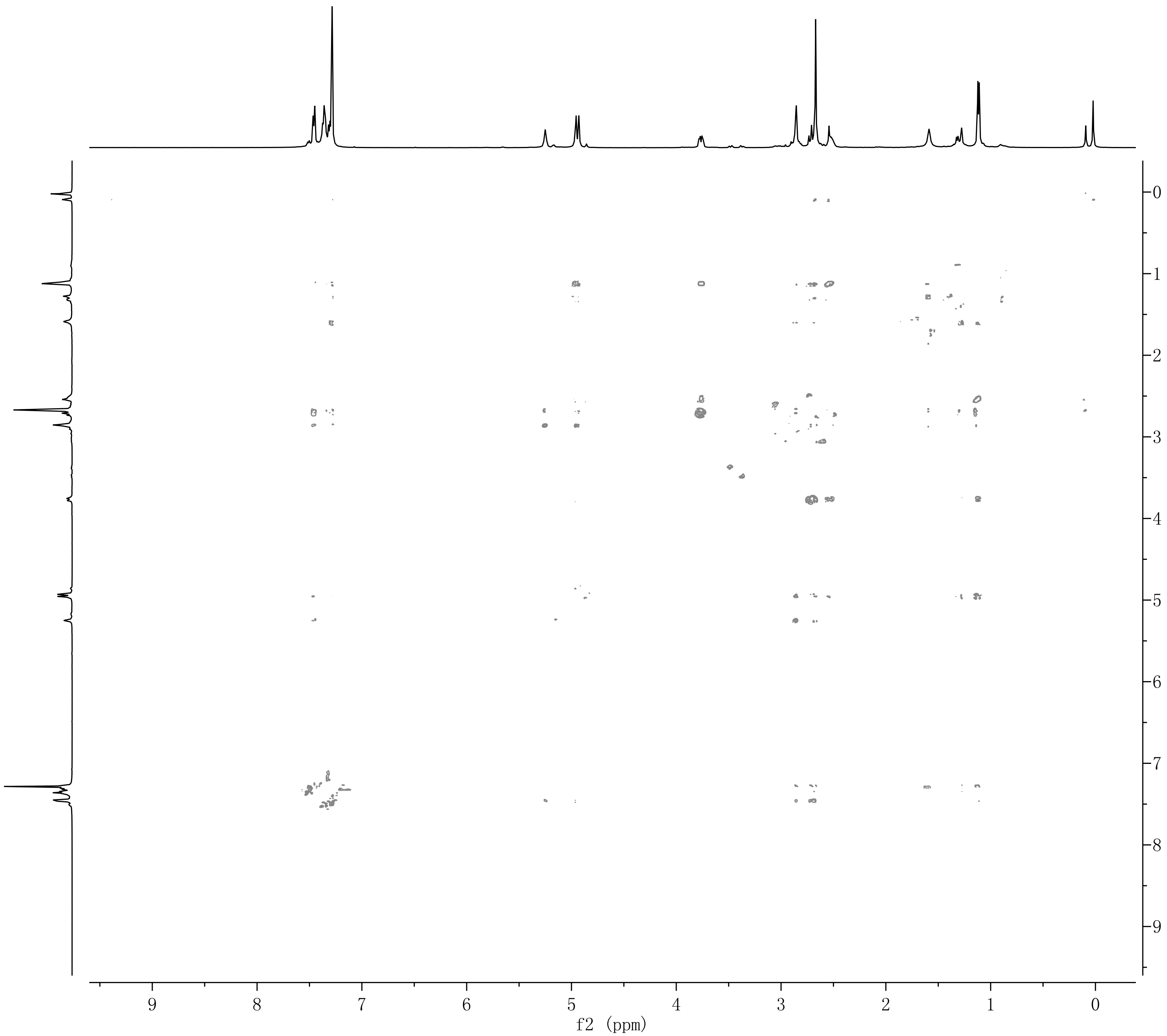

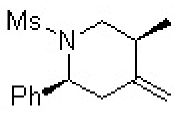

relative configuration

Parameter	Value
Comment	
Origin	Bruker BioSpin GmbH
Owner	nmr
Site	
Instrument	spect
Solvent	CDCl3
Temperature	295.0
Pulse Sequence	noesygpphpp
Experiment	NOESY
Number of Scans	4
Receiver Gain	62.3
Relaxation Delay	2.0000
Pulse Width	10.0000
Presaturation Frequency	
Acquisition Time	0.2560
Acquisition Date	2018-11-12T20:10:43
Modification Date	2018-11-13T09:58:50
Spectrometer Frequency	(400.13, 400.13)
Spectral Width	(4000.0, 4000.0)
Lowest Frequency	(-158.1, -158.1)
Nucleus	(1H, 1H)
Acquired Size	(1024, 256)
Spectral Size	(1024, 1024)

Parameter	Value
Title	xfy-190910-1-s2.11.fid
Comment	
Origin	Bruker BioSpin GmbH
Owner	nmr
Site	
Instrument	spect
Solvent	CDCl3
Temperature	296.8
Pulse Sequence	zg30
Experiment	1D
Number of Scans	8
Receiver Gain	142.1
Relaxation Delay	1.0000
Pulse Width	8.7300
Presaturation Frequency	
Acquisition Time	1.9999
Acquisition Date	2019-09-10T23:14:03
Modification Date	2019-09-11T09:34:56
Spectrometer Frequency	400.13
Spectral Width	8012.8
Lowest Frequency	-1544.5
Nucleus	1H
Acquired Size	16025
Spectral Size	65536

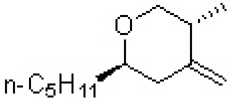

relative configuration

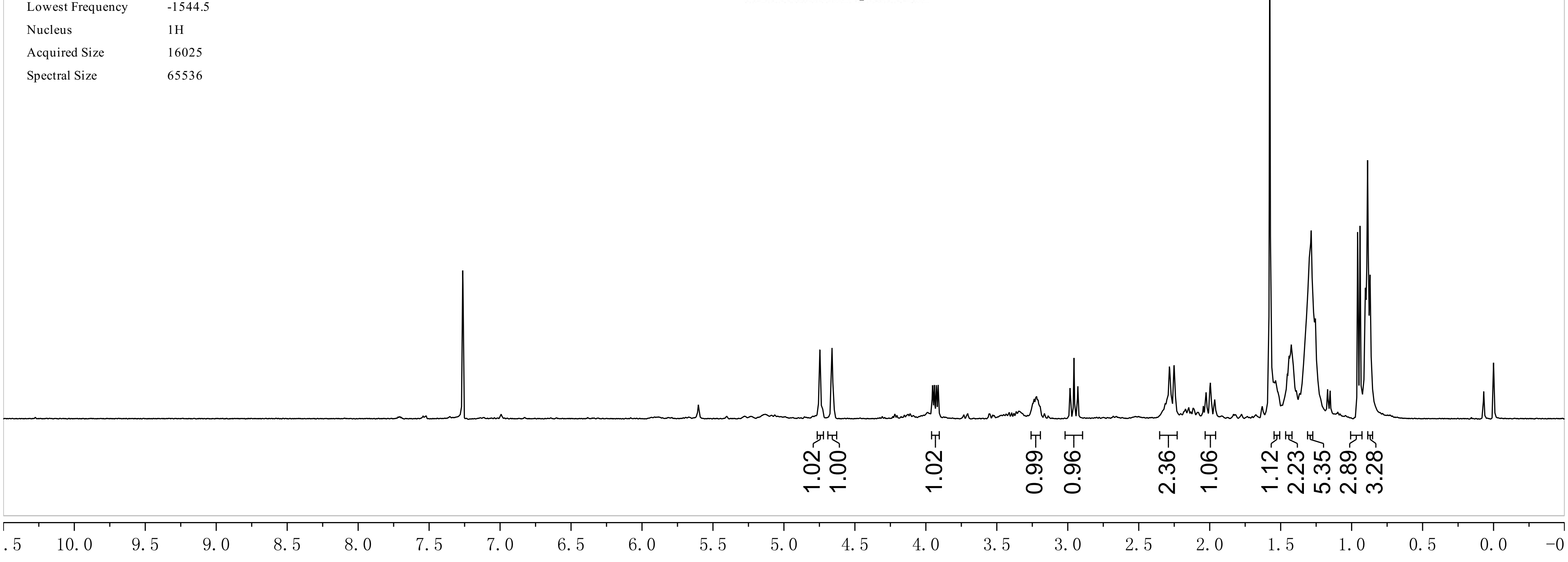

Parameter	Value
Title	xfy-190910-1-s2.12.fid
Comment	
Origin	Bruker BioSpin GmbH
Owner	nmr
Site	
Instrument	spect
Solvent	CDCl3
Temperature	297.0
Pulse Sequence	zgpg30
Experiment	1D
Number of Scans	800
Receiver Gain	196.4
Relaxation Delay	2.0000
Pulse Width	10.0000
Presaturation Frequency	
Acquisition Time	1.3631
Acquisition Date	2019-09-11T06:38:05
Modification Date	2019-09-11T09:34:57
Spectrometer Frequency	100.62
Spectral Width	24038.5
Lowest Frequency	-1943.6
Nucleus	13C
Acquired Size	32768
Spectral Size	65536

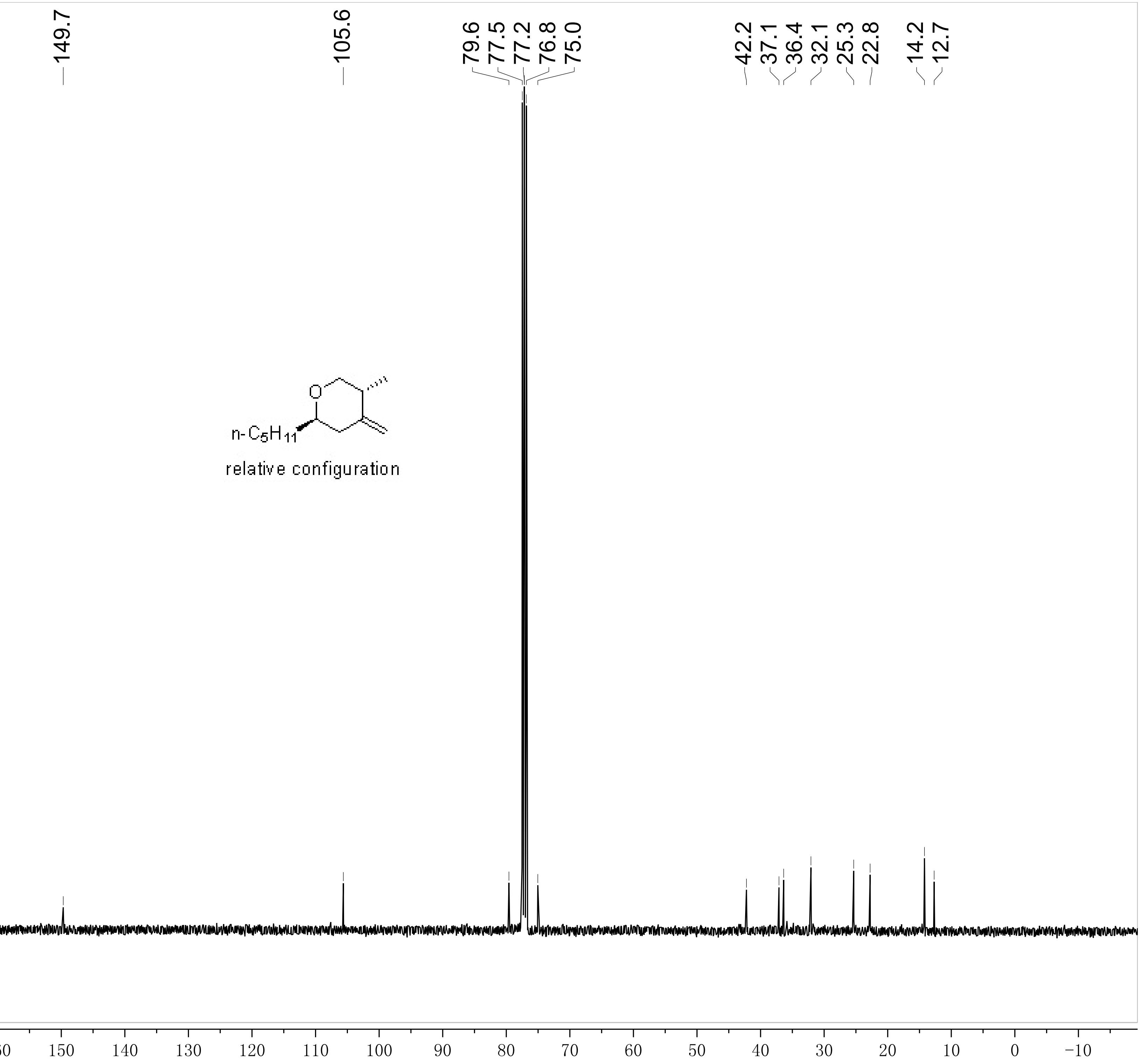

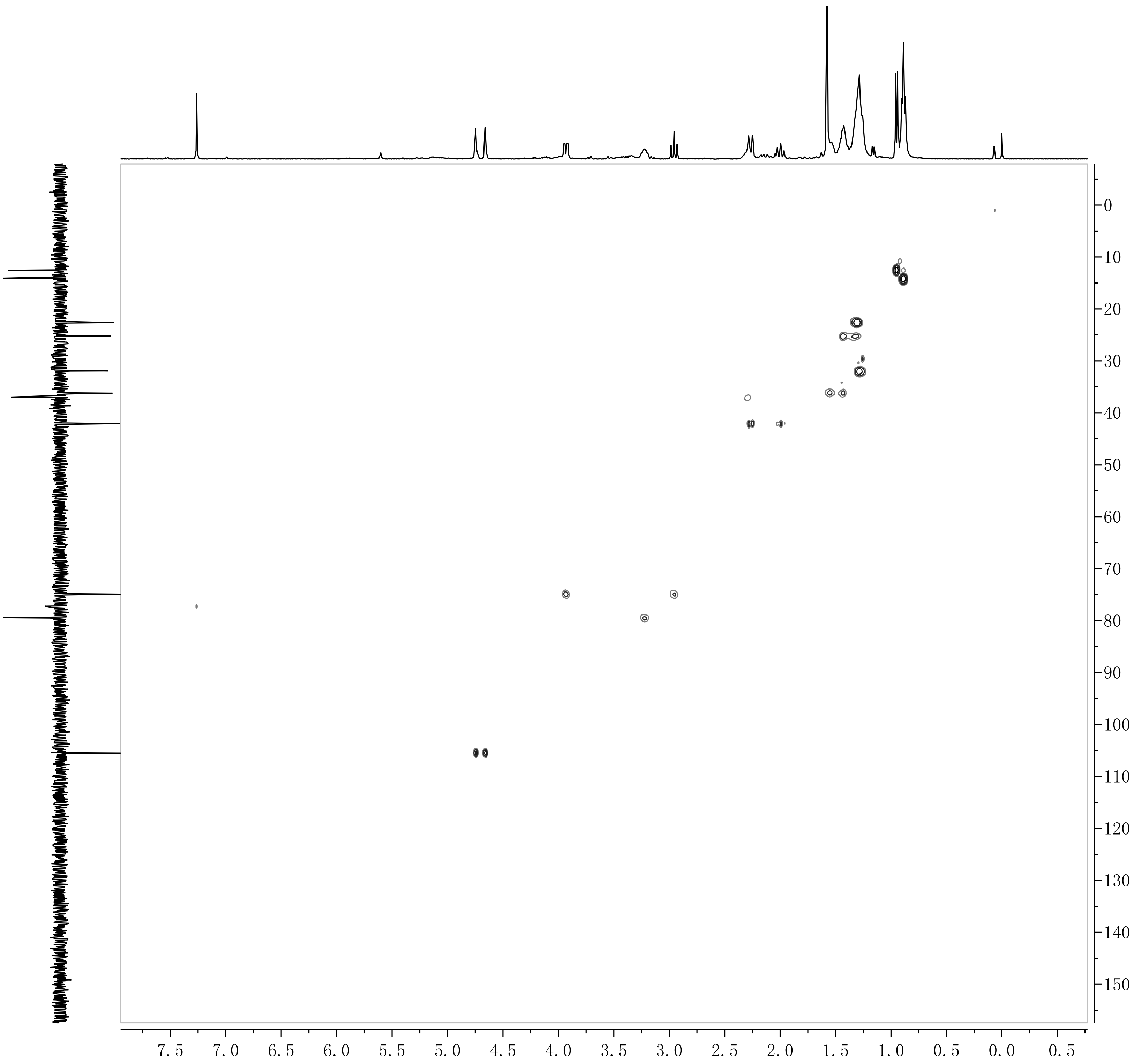

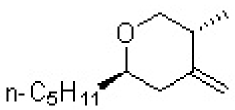

relative configuration

Parameter	Value
Title	xfy-190910-1-s2.14.ser
Comment	
Origin	Bruker BioSpin GmbH
Owner	nmr
Site	
Instrument	spect
Solvent	CDCl3
Temperature	296.7
Pulse Sequence	hsqcetgp
Experiment	HSQC
Number of Scans	2
Receiver Gain	196.4
Relaxation Delay	1.4521
Pulse Width	8.7300
Presaturation Frequency	
Acquisition Time	0.1464
Acquisition Date	2019-09-11T06:55:16
Modification Date	2019-09-11T09:34:57
Spectrometer Frequency	(400.13, 100.62)
Spectral Width	(3496.5, 16666.7)
Lowest Frequency	(-316.0, -829.1)
Nucleus	(1H, 13C)
Acquired Size	(512, 256)
Spectral Size	(512, 512)

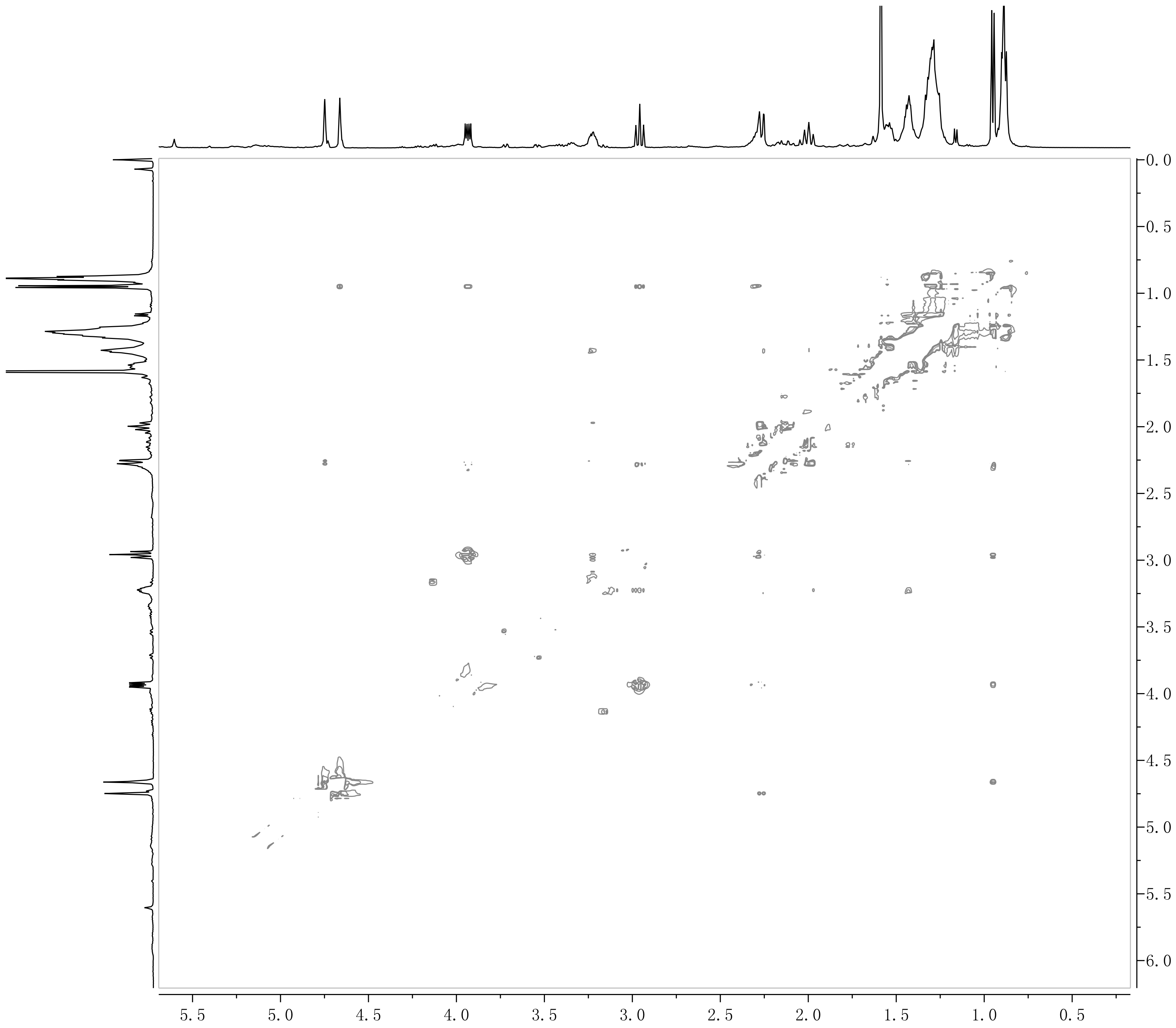

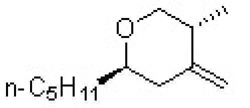

relative configuration

Parameter	Value
Title	xfy-190910-1-s2.102.ser
Comment	
Origin	Bruker BioSpin GmbH
Owner	nmr
Site	
Instrument	spect
Solvent	CDCl3
Temperature	296.1
Pulse Sequence	noesygpphpp
Experiment	NOESY
Number of Scans	20
Receiver Gain	31.1
Relaxation Delay	1.9754
Pulse Width	10.7100
Presaturation Frequency	
Acquisition Time	0.2294
Acquisition Date	2019-09-12T04:40:45
Modification Date	2019-09-12T09:10:16
Spectrometer Frequency	(500.13, 500.13)
Spectral Width	(4464.3, 4464.3)
Lowest Frequency	(-397.0, -397.0)
Nucleus	(1H, 1H)
Acquired Size	(1024, 256)
Spectral Size	(1024, 1024)

Parameter	Value
Comment	
Origin	Bruker BioSpin GmbH
Owner	nmr
Site	
Instrument	spect
Solvent	CDCl3
Temperature	296.1
Pulse Sequence	zg30
Experiment	1D
Number of Scans	15
Receiver Gain	77.6
Relaxation Delay	1.0000
Pulse Width	10.7100
Presaturation Frequency	
Acquisition Time	3.2768
Acquisition Date	2019-05-16T11:26:10
Modification Date	2019-05-19T16:23:05
Spectrometer Frequency	500.13
Spectral Width	10000.0
Lowest Frequency	-1920.1
Nucleus	1H
Acquired Size	32768
Spectral Size	65536

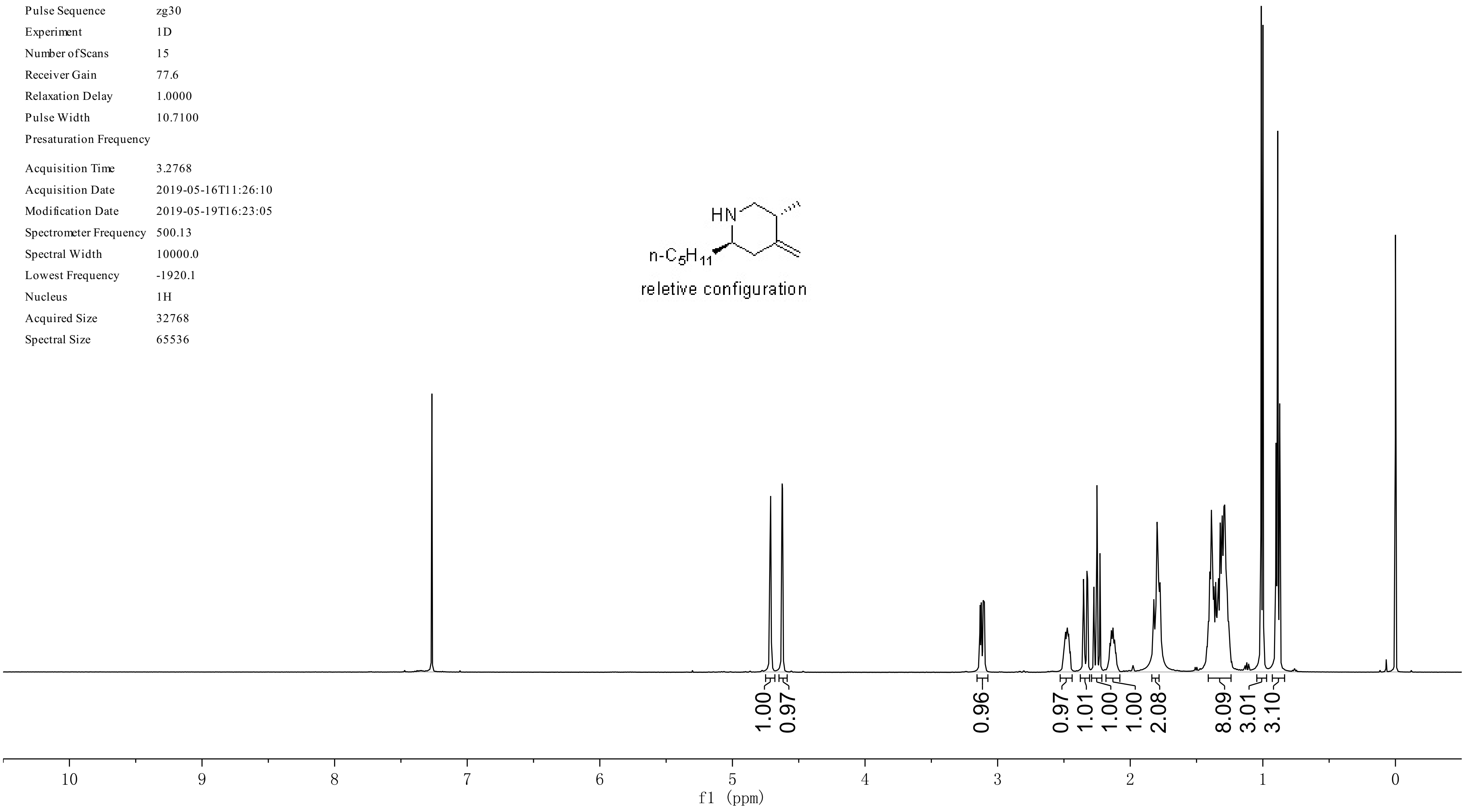

Parameter	Value
Comment	
Origin	Bruker BioSpin GmbH
Owner	nmr
Site	
Instrument	spect
Solvent	CDCl3
Temperature	296.2
Pulse Sequence	zgpg30
Experiment	1D
Number of Scans	103
Receiver Gain	193.1
Relaxation Delay	2.0000
Pulse Width	9.6000
Presaturation Frequency	
Acquisition Time	1.1010
Acquisition Date	2019-05-16T11:28:33
Modification Date	2019-05-19T16:23:06
Spectrometer Frequency	125.77
Spectral Width	29761.9
Lowest Frequency	-2289.0
Nucleus	13C
Acquired Size	32768
Spectral Size	65536

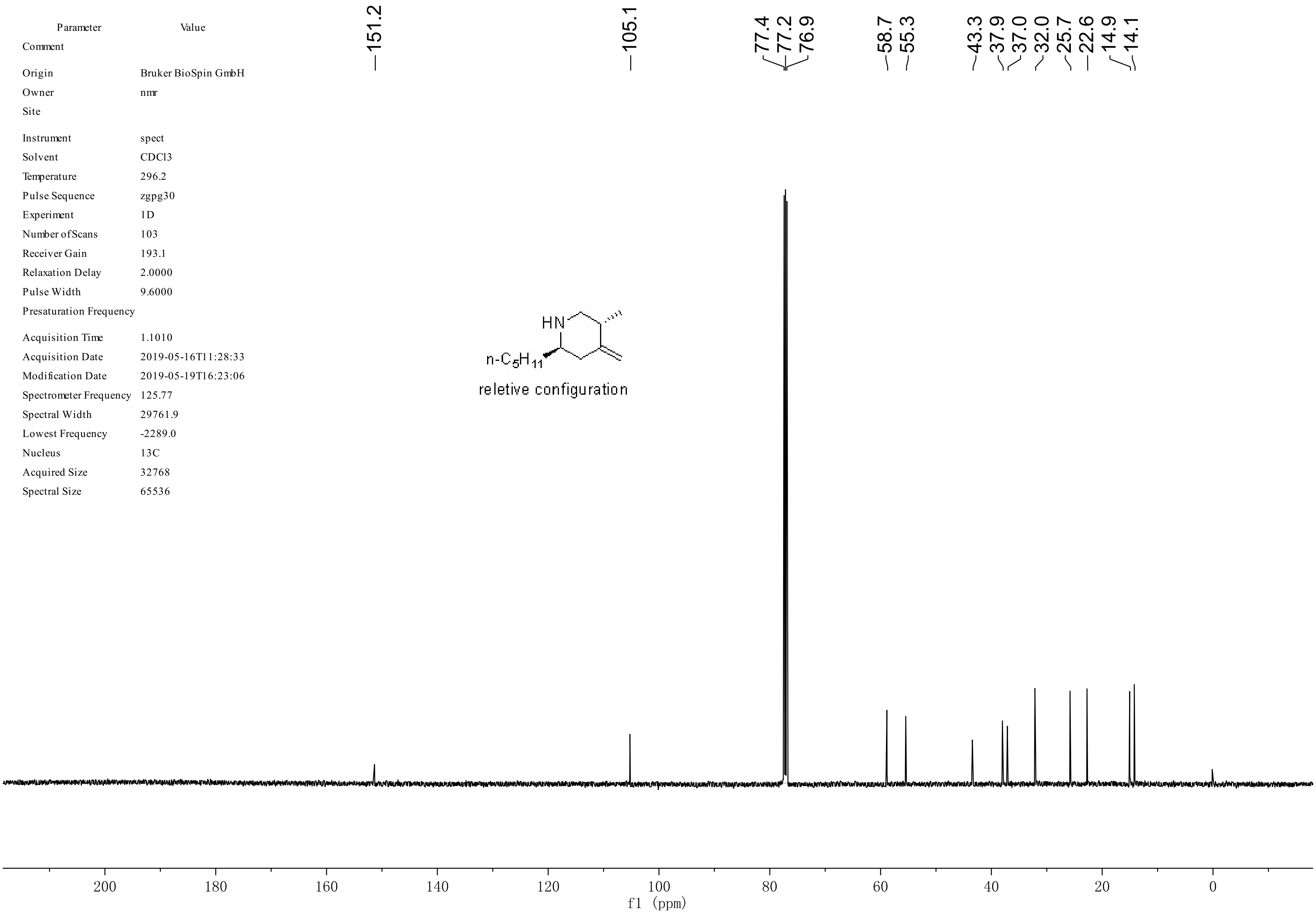

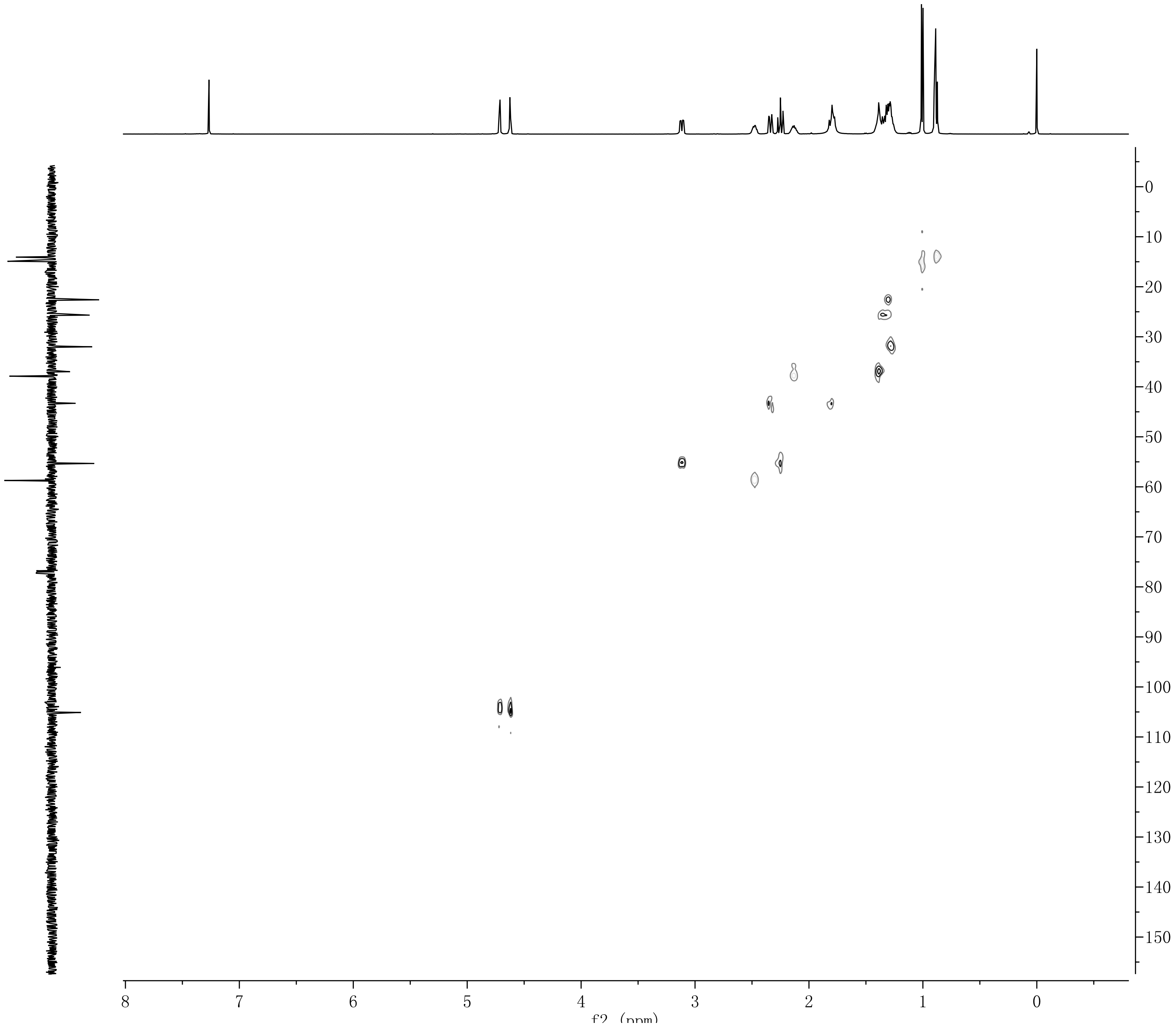

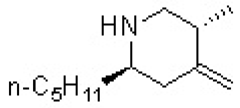

reletive configuration

Parameter	Value
Comment	
Origin	Bruker BioSpin GmbH
Owner	nmr
Site	
Instrument	spect
Solvent	CDCl3
Temperature	296.1
Pulse Sequence	hsqcedetgp
Experiment	HSQC-EDITED
Number of Scans	2
Receiver Gain	31.1
Relaxation Delay	1.4631
Pulse Width	10.7100
Presaturation Frequency	
Acquisition Time	0.1157
Acquisition Date	2019-05-16T11:37:12
Modification Date	2019-05-19T16:23:06
Spectrometer Frequency	(500.13, 125.77)
Spectral Width	(4424.8, 20833.3)
Lowest Frequency	(-412.8, -1037.0)
Nucleus	(1H, 13C)
Acquired Size	(512, 256)
Spectral Size	(512, 512)

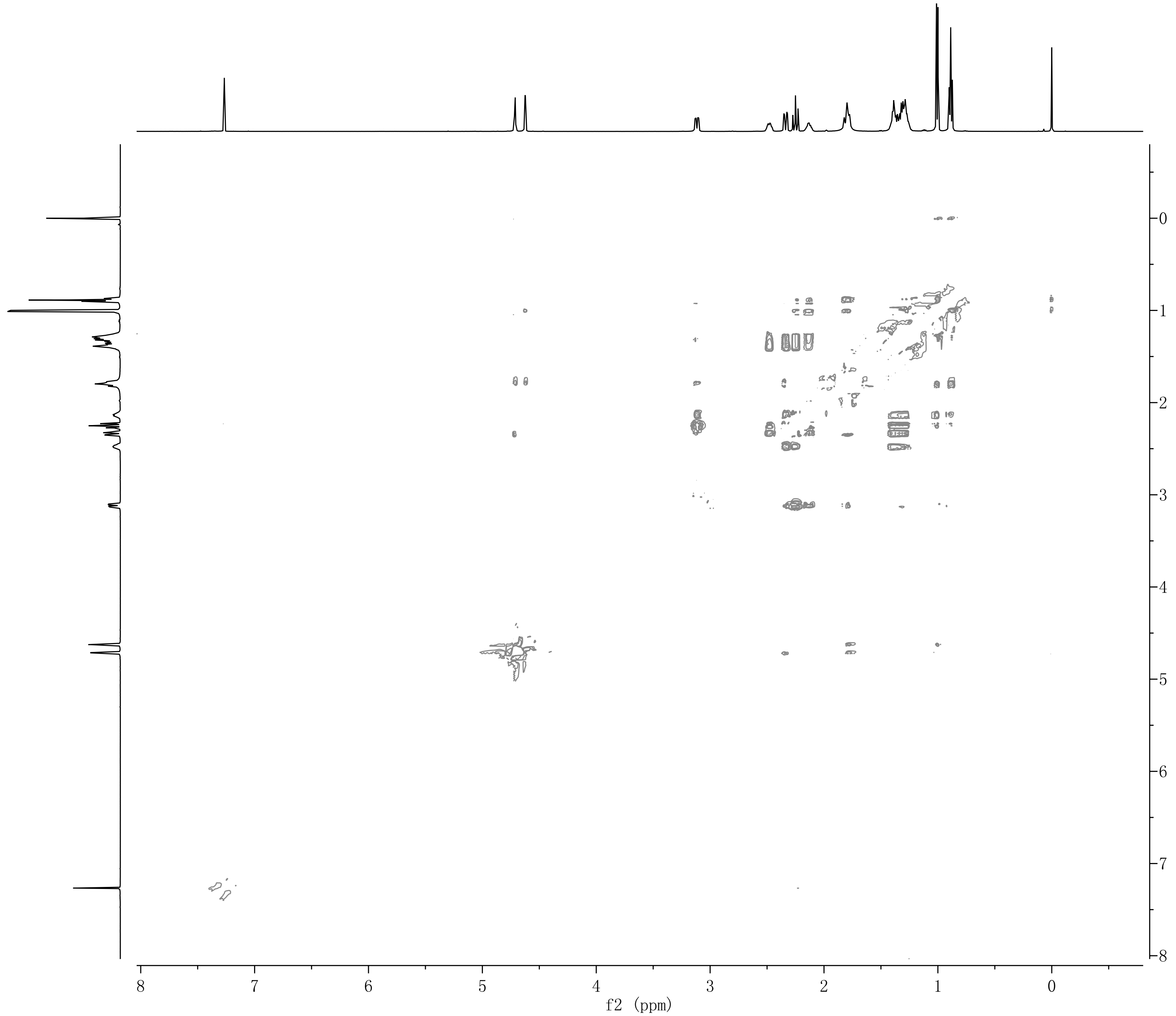

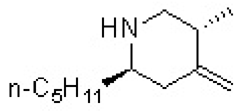

reletive configuration

Parameter	Value
Title	gvv-e198f-full.5.ser
Comment	
Origin	Bruker BioSpin GmbH
Owner	nmr
Site	
Instrument	spect
Solvent	CDCl3
Temperature	296.2
Pulse Sequence	noesygpphpp
Experiment	NOESY
Number of Scans	4
Receiver Gain	31.1
Relaxation Delay	1.9734
Pulse Width	10.7100
Presaturation Frequency	
Acquisition Time	0.2314
Acquisition Date	2019-05-16T11:53:20
Modification Date	2019-05-19T16:23:09
Spectrometer Frequency	(500.13, 500.13)
Spectral Width	(4424.8, 4424.8)
Lowest Frequency	(-405.8, -405.8)
Nucleus	(1H, 1H)
Acquired Size	(1024, 162)
Spectral Size	(1024, 1024)

Parameter	Value
Title	xfy-190523-6-s2.41.fid
Comment	
Origin	Bruker BioSpin GmbH
Owner	nmr
Site	
Instrument	spect
Solvent	CDCl3
Temperature	296.1
Pulse Sequence	zg30
Experiment	1D
Number of Scans	16
Receiver Gain	31.1
Relaxation Delay	1.0000
Pulse Width	10.7100
Presaturation Frequency	
Acquisition Time	3.2768
Acquisition Date	2019-05-24T21:37:51
Modification Date	2019-05-24T21:54:13
Spectrometer Frequency	500.13
Spectral Width	10000.0
Lowest Frequency	-1911.5
Nucleus	1H
Acquired Size	32768
Spectral Size	65536

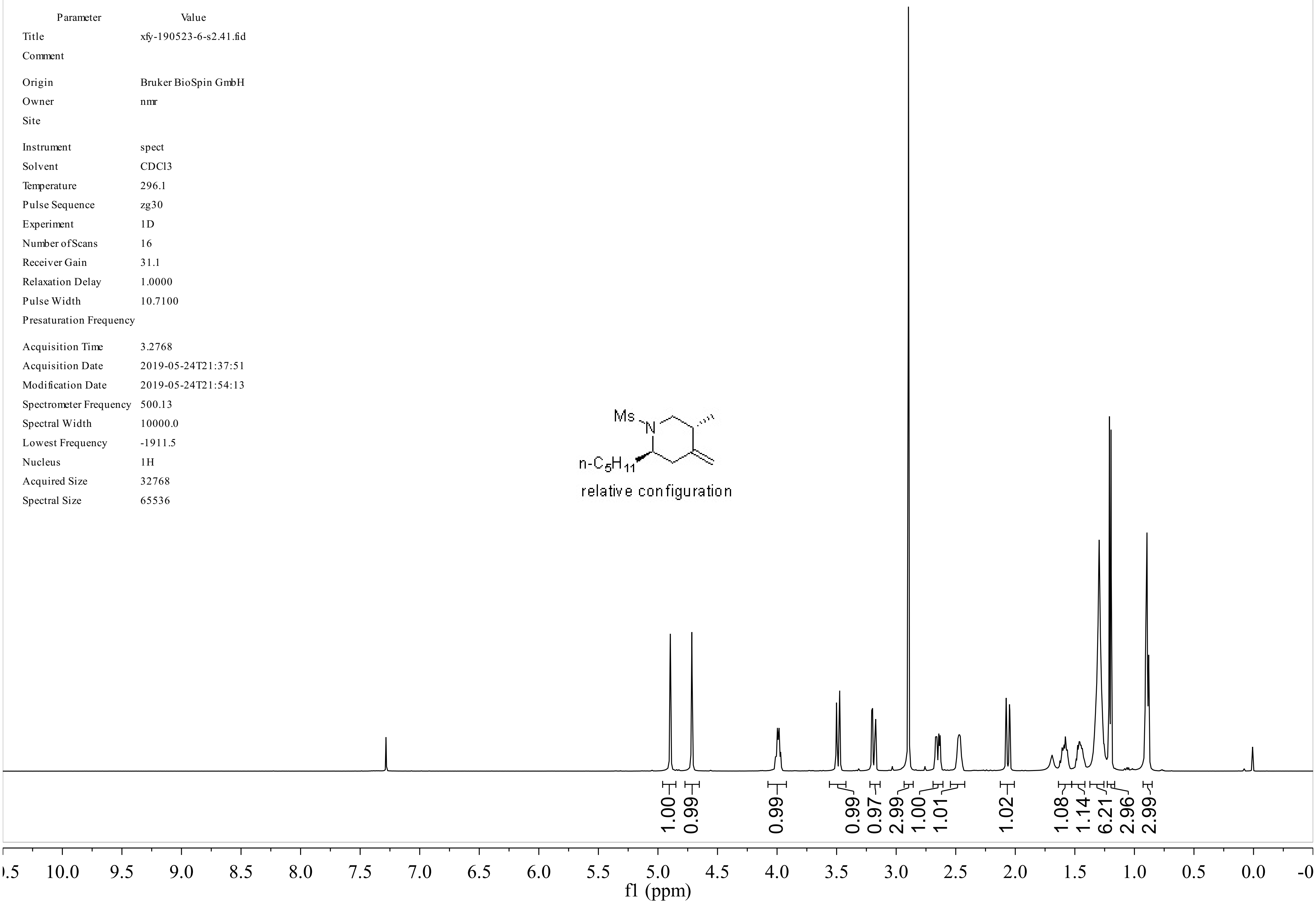

Parameter	Value
Title	xfy-190523-6-s2.42.fid
Comment	
Origin	Bruker BioSpin GmbH
Owner	nmr
Site	
Instrument	spect
Solvent	CDCl3
Temperature	296.2
Pulse Sequence	zgpg30
Experiment	1D
Number of Scans	26
Receiver Gain	193.1
Relaxation Delay	2.0000
Pulse Width	9.6000
Presaturation Frequency	
Acquisition Time	1.1010
Acquisition Date	2019-05-24T21:40:07
Modification Date	2019-05-24T21:54:14
Spectrometer Frequency	125.77
Spectral Width	29761.9
Lowest Frequency	-2292.1
Nucleus	13C
Acquired Size	32768
Spectral Size	65536

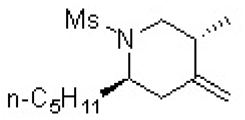

relative configuration

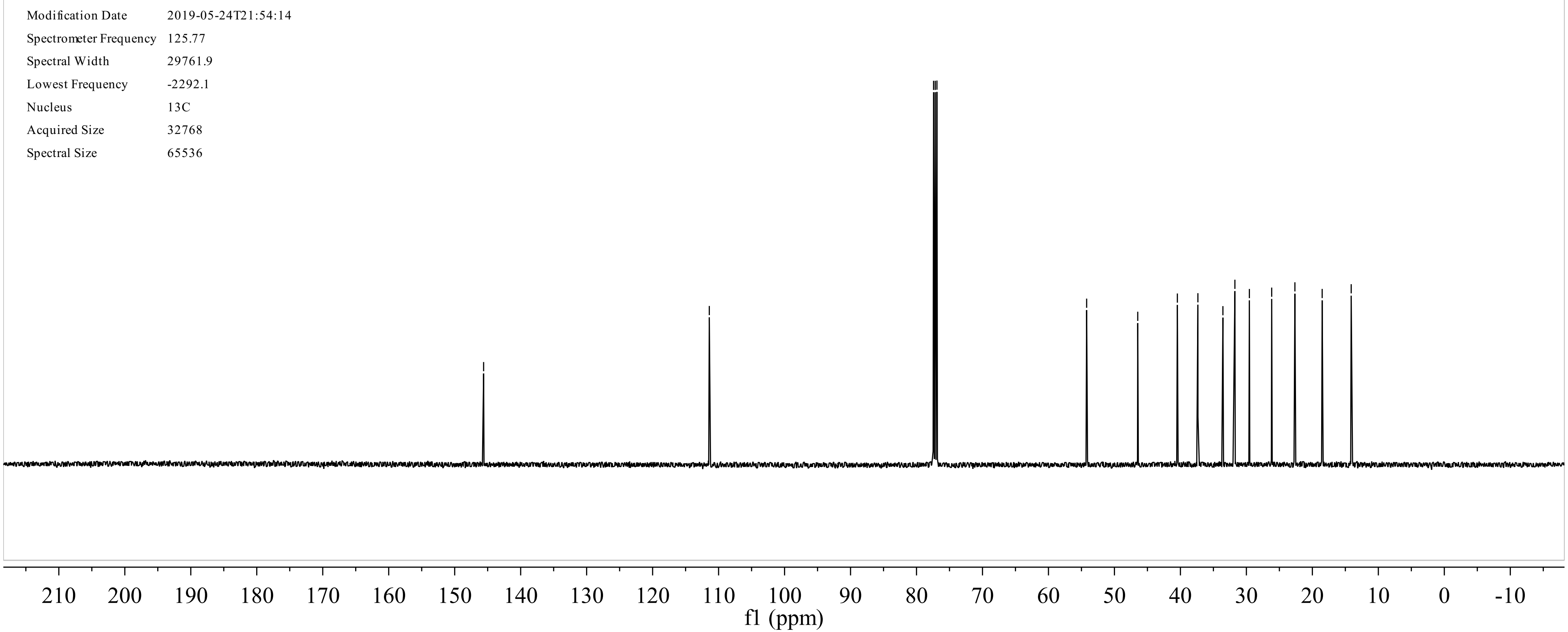

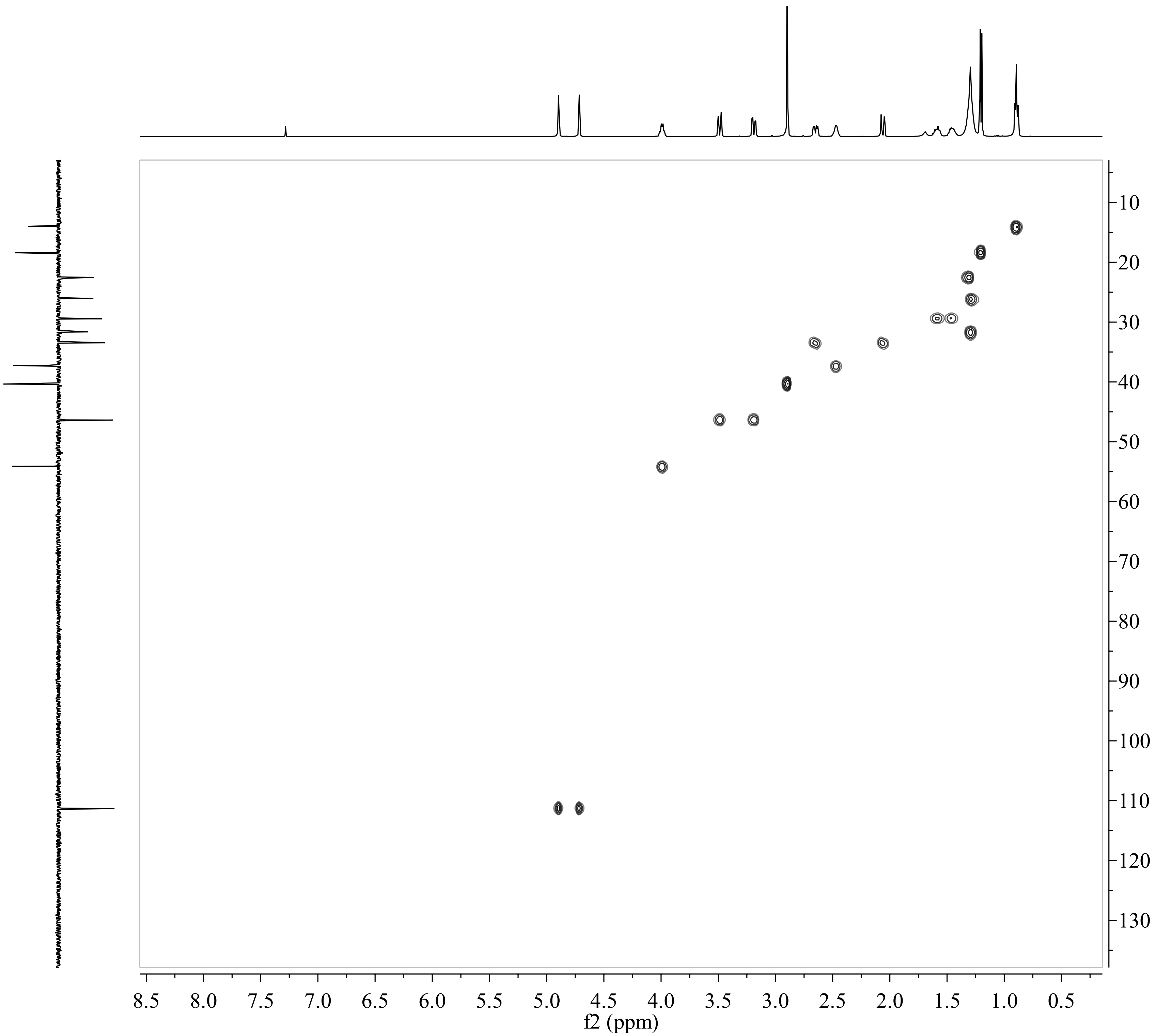

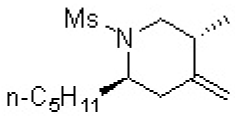

relative configuration

Parameter	Value
Title	xfy-190523-6-s1.25.ser
Comment	
Origin	Bruker BioSpin GmbH
Owner	nmr
Site	
Instrument	spect
Solvent	CDCl3
Temperature	296.3
Pulse Sequence	hsqcetgp
Experiment	HSQC
Number of Scans	2
Receiver Gain	196.4
Relaxation Delay	1.5000
Pulse Width	8.7300
Presaturation Frequency	
Acquisition Time	0.0985
Acquisition Date	2019-05-24T19:03:20
Modification Date	2019-05-24T19:24:50
Spectrometer Frequency	(400.13, 100.62)
Spectral Width	(5197.5, 16666.7)
Lowest Frequency	(-193.1, -829.1)
Nucleus	(1H, 13C)
Acquired Size	(512, 256)
Spectral Size	(512, 512)

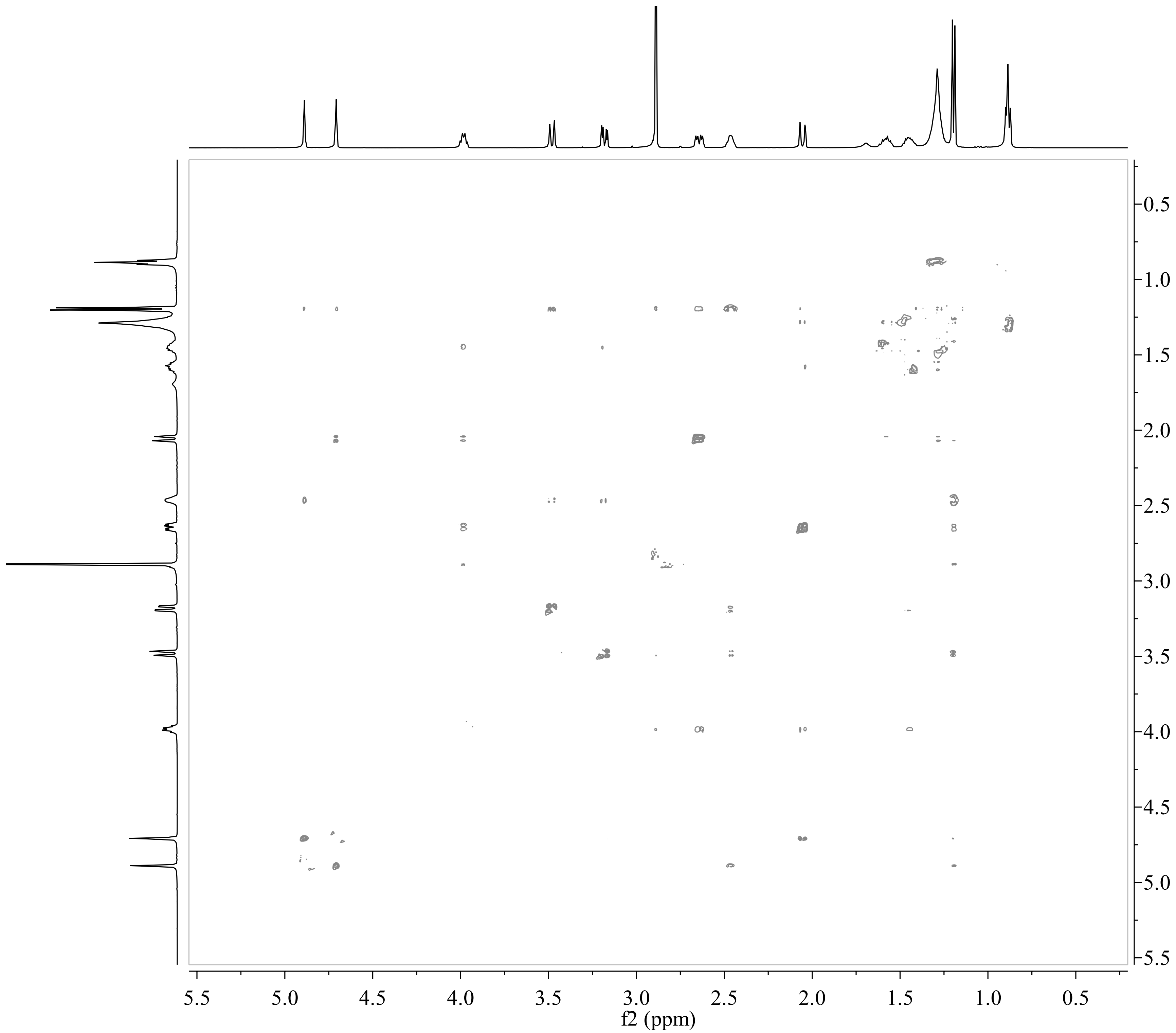

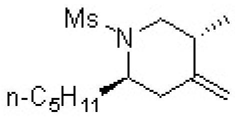

relative configuration

Parameter	Value
Title	xfy-190523-6-s2.2.ser
Comment	
Origin	Bruker BioSpin GmbH
Owner	nmr
Site	
Instrument	spect
Solvent	CDCl3
Temperature	296.1
Pulse Sequence	noesygpphpp
Experiment	NOESY
Number of Scans	4
Receiver Gain	16.0
Relaxation Delay	1.8218
Pulse Width	10.7100
Presaturation Frequency	
Acquisition Time	0.3830
Acquisition Date	2019-05-26T09:10:29
Modification Date	2019-05-26T10:08:33
Spectrometer Frequency	(500.13, 500.13)
Spectral Width	(2673.8, 2673.8)
Lowest Frequency	(99.8, 99.8)
Nucleus	(1H, 1H)
Acquired Size	(1024, 256)
Spectral Size	(1024, 1024)

Parameter	Value
Origin	Bruker BioSpin GmbH
Spectrometer	spect
Solvent	CDCl3
Temperature	296.1
Pulse Sequence	zg30
Experiment	1D
Number of Scans	9
Receiver Gain	88
Relaxation Delay	1.0000
Pulse Width	10.7100
Acquisition Time	3.2768
Spectrometer Frequency	500.13
Spectral Width	10000.0
Lowest Frequency	-1932.5
Nucleus	1H
Acquired Size	32768
Spectral Size	65536

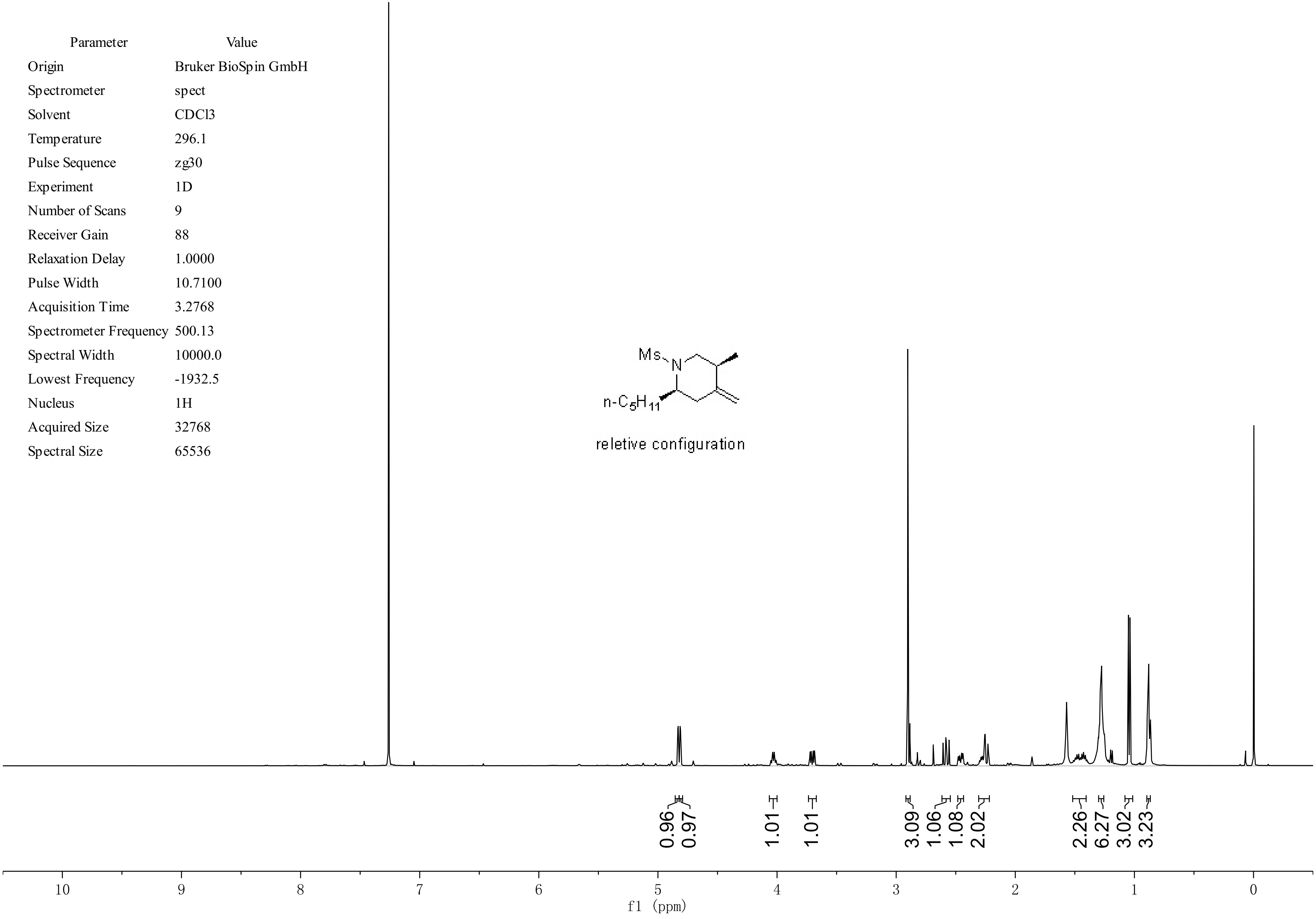

Parameter	Value
Origin	Bruker BioSpin GmbH
Spectrometer	spect
Solvent	CDCl3
Temperature	296.1
Pulse Sequence	zgpg30
Experiment	1D
Number of Scans	300
Receiver Gain	193
Relaxation Delay	2.0000
Pulse Width	9.6000
Acquisition Time	1.1010
Spectrometer Frequency	125.77
Spectral Width	29761.9
Lowest Frequency	-2272.3
Nucleus	13C
Acquired Size	32768
Spectral Size	65536

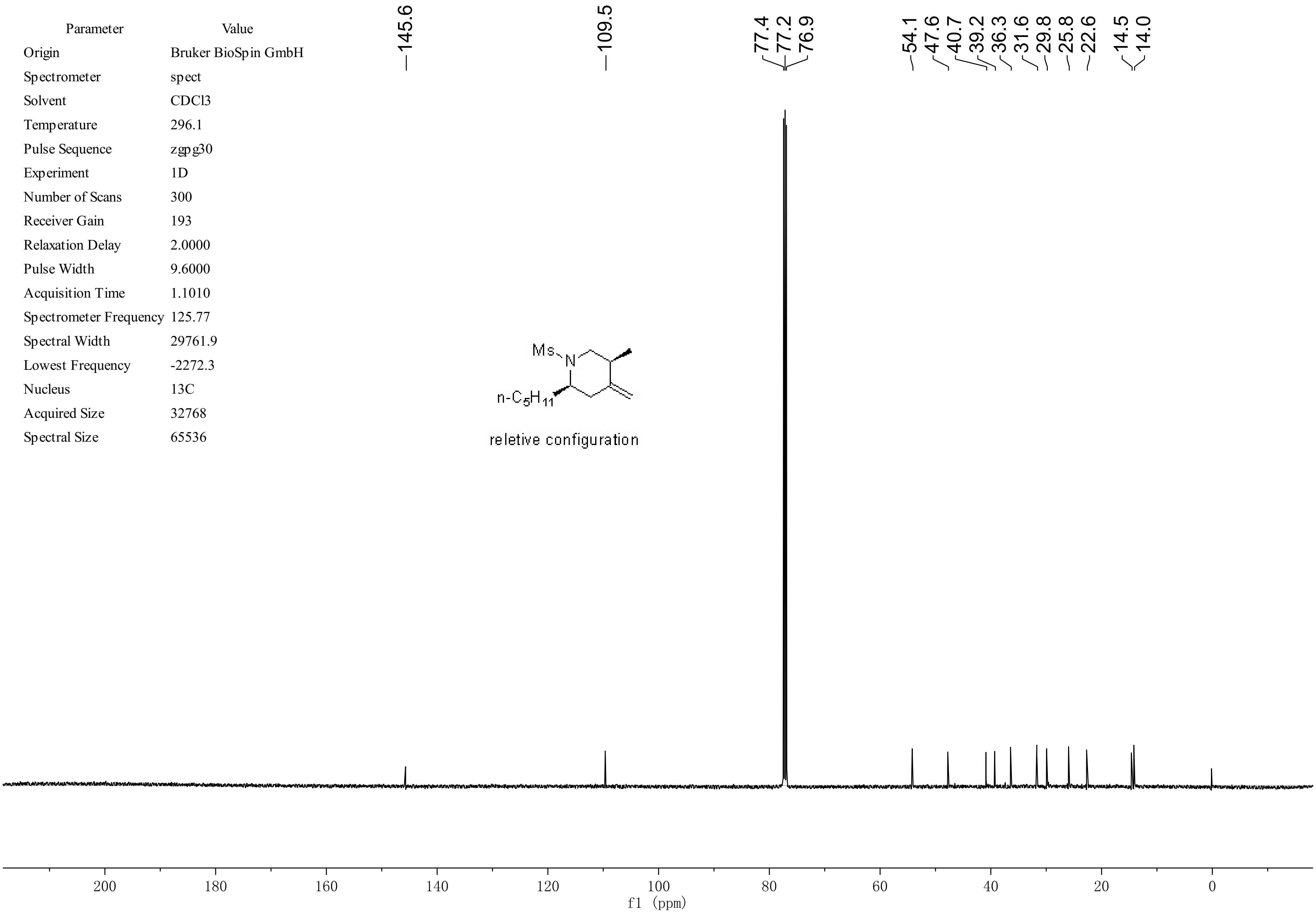

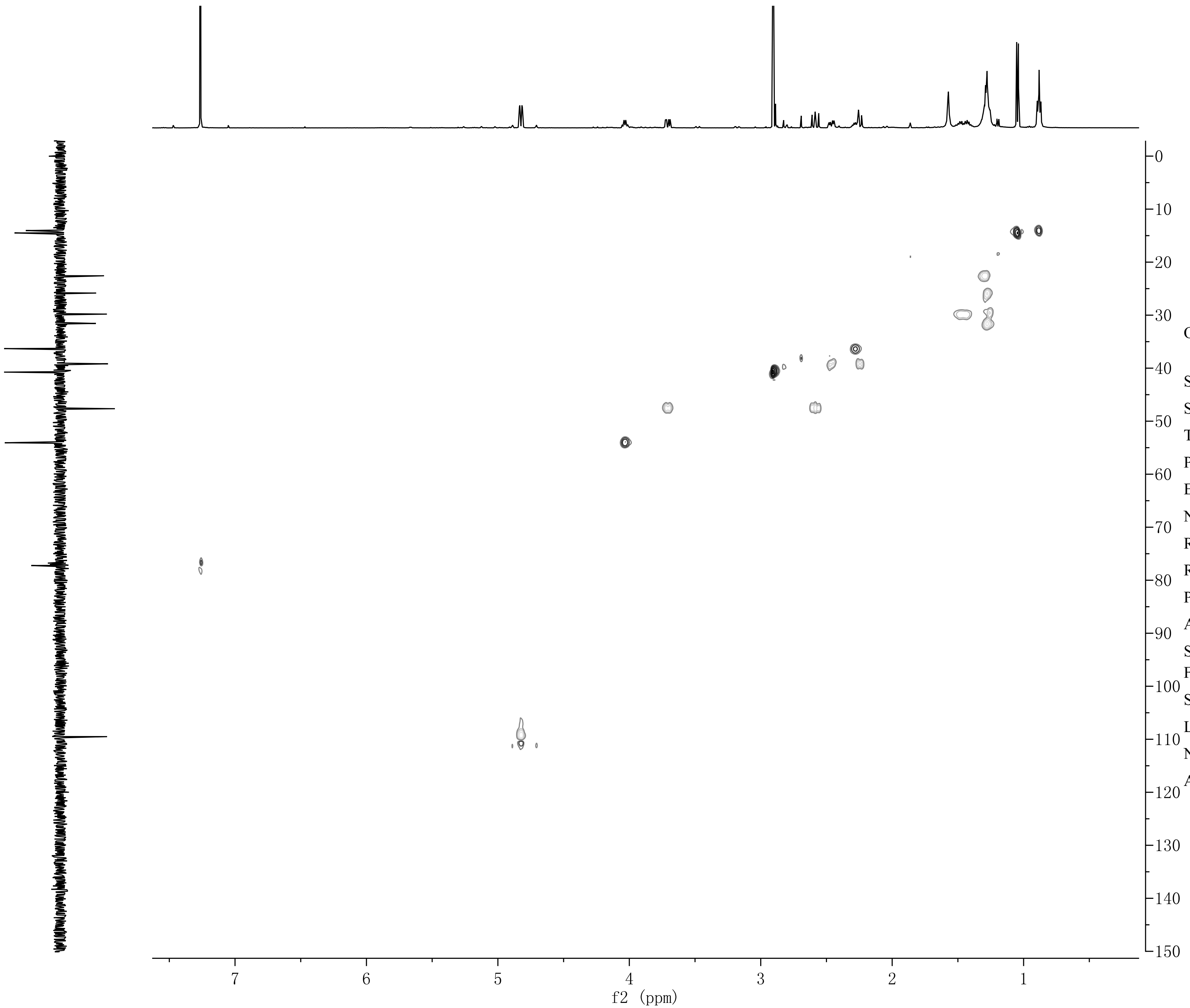

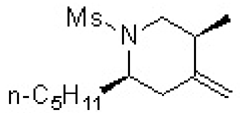

reletive configuration

Parameter	Value
Origin	Bruker BioSpin GmbH
Spectrometer	spect
Solvent	CDCl3
Temperature	296.1
Pulse Sequence	hsqcedetgp
Experiment	HSQC-EDITED
Number of Scans	2
Receiver Gain	193
Relaxation Delay	1.4488
Pulse Width	10.7100
Acquisition Time	0.1300
Spectrometer Frequency	(500.13, 125.77)
Spectral Width	(3937.0, 20833.3)
Lowest Frequency	(0.5, -1037.0)
Nucleus	(1H, 13C)
Acquired Size	(512, 256)

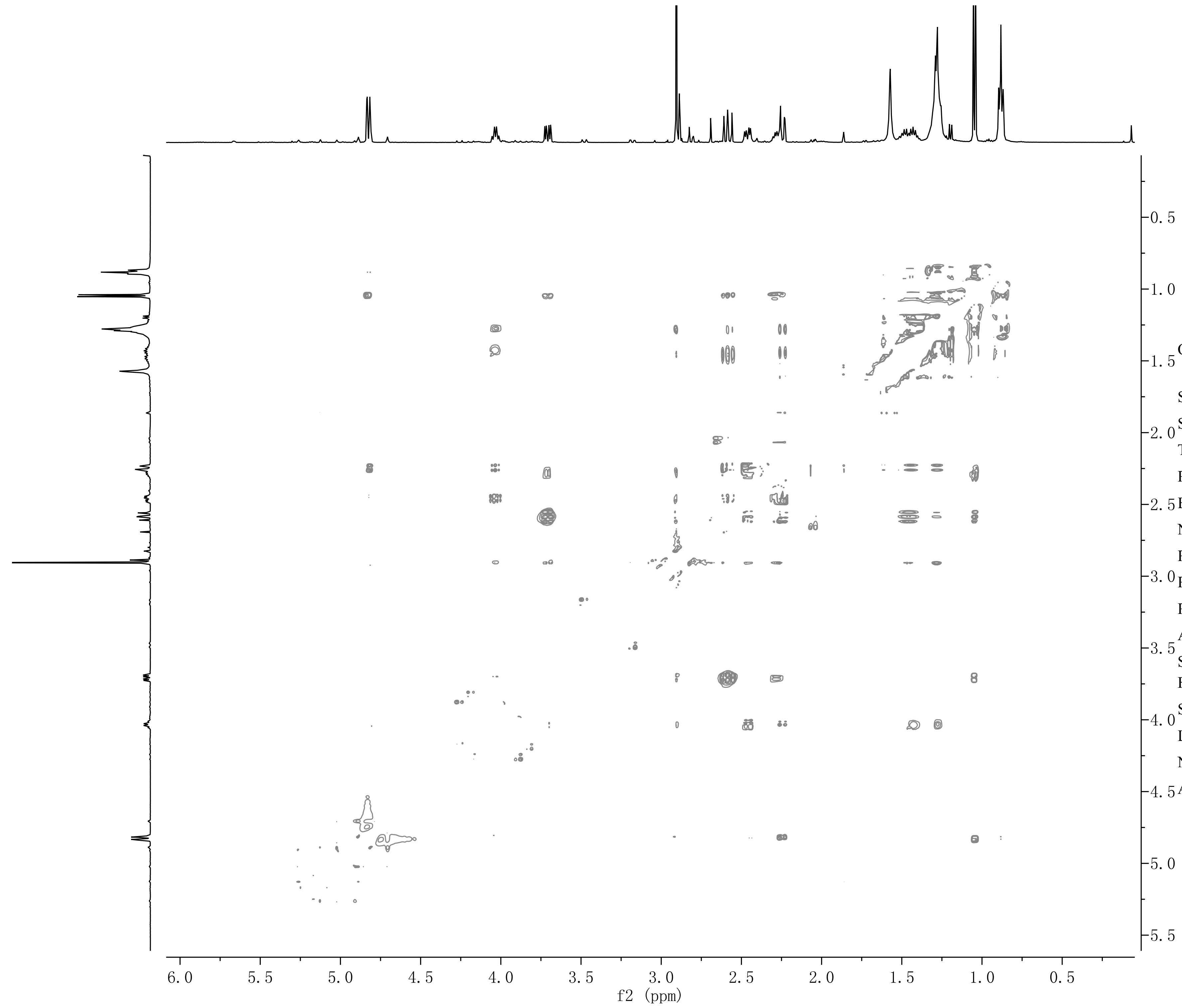

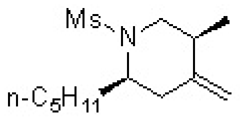

reletive configuration

Parameter	Value
Origin	Bruker BioSpin GmbH
Spectrometer	spect
Solvent	CDCl3
Temperature	293.9
Pulse Sequence	noesygpphpp
Experiment	NOESY
Number of Scans	4
Receiver Gain	35
Relaxation Delay	1.9693
Pulse Width	8.7300
Acquisition Time	0.2867
Spectrometer Frequency	(400.13, 400.13)
Spectral Width	(3571.4, 3571.4)
Lowest Frequency	(-260.5, -260.5)
Nucleus	(1H, 1H)
Acquired Size	(1024, 256)

Parameter	Value
Title	1.1.fid
Comment	
Origin	Bruker BioSpin GmbH
Owner	nmr
Site	
Instrument	spect
Solvent	CDCl3
Temperature	297.4
Pulse Sequence	zg30
Experiment	1D
Number of Scans	8
Receiver Gain	142.1
Relaxation Delay	1.0000
Pulse Width	8.7300
Presaturation Frequency	
Acquisition Time	1.9999
Acquisition Date	2019-09-11T23:14:49
Modification Date	2019-09-12T09:10:06
Spectrometer Frequency	400.13
Spectral Width	8012.8
Lowest Frequency	-1544.6
Nucleus	1H
Acquired Size	16025
Spectral Size	65536

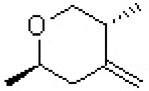

relative configuration

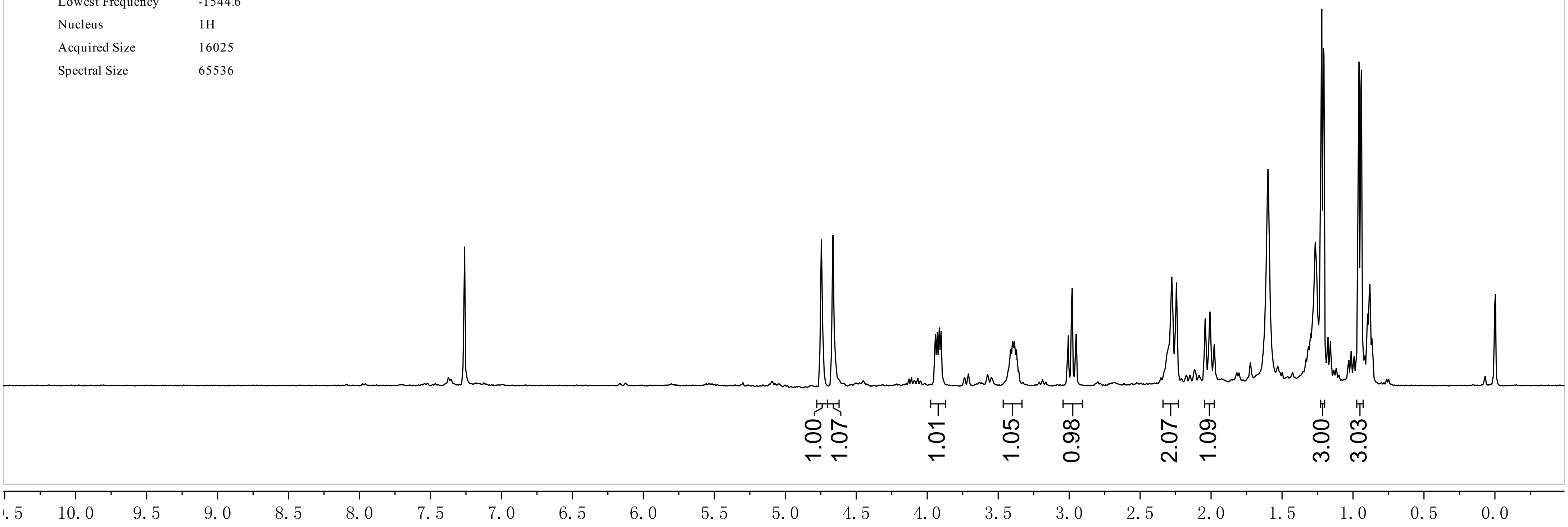

Parameter	Value
Title	1.2.fid
Comment	
Origin	Bruker BioSpin GmbH
Owner	nmr
Site	
Instrument	spect
Solvent	CDCl3
Temperature	297.6
Pulse Sequence	zgpg30
Experiment	1D
Number of Scans	49
Receiver Gain	196.4
Relaxation Delay	2.0000
Pulse Width	10.0000
Presaturation Frequency	
Acquisition Time	1.3631
Acquisition Date	2019-09-11T23:16:25
Modification Date	2019-09-12T09:10:06
Spectrometer Frequency	100.62
Spectral Width	24038.5
Lowest Frequency	-1943.5
Nucleus	13C
Acquired Size	32768
Spectral Size	65536

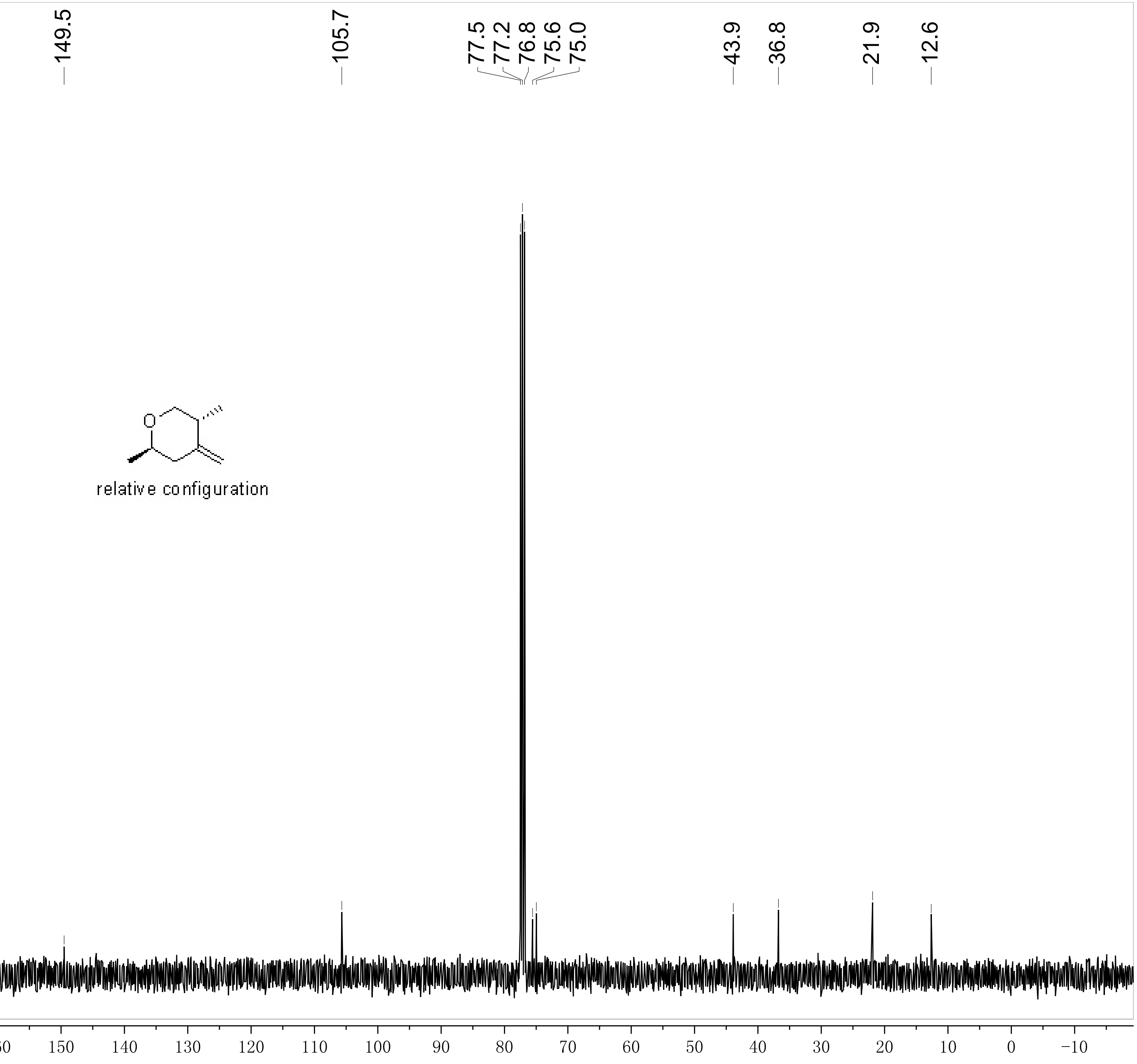

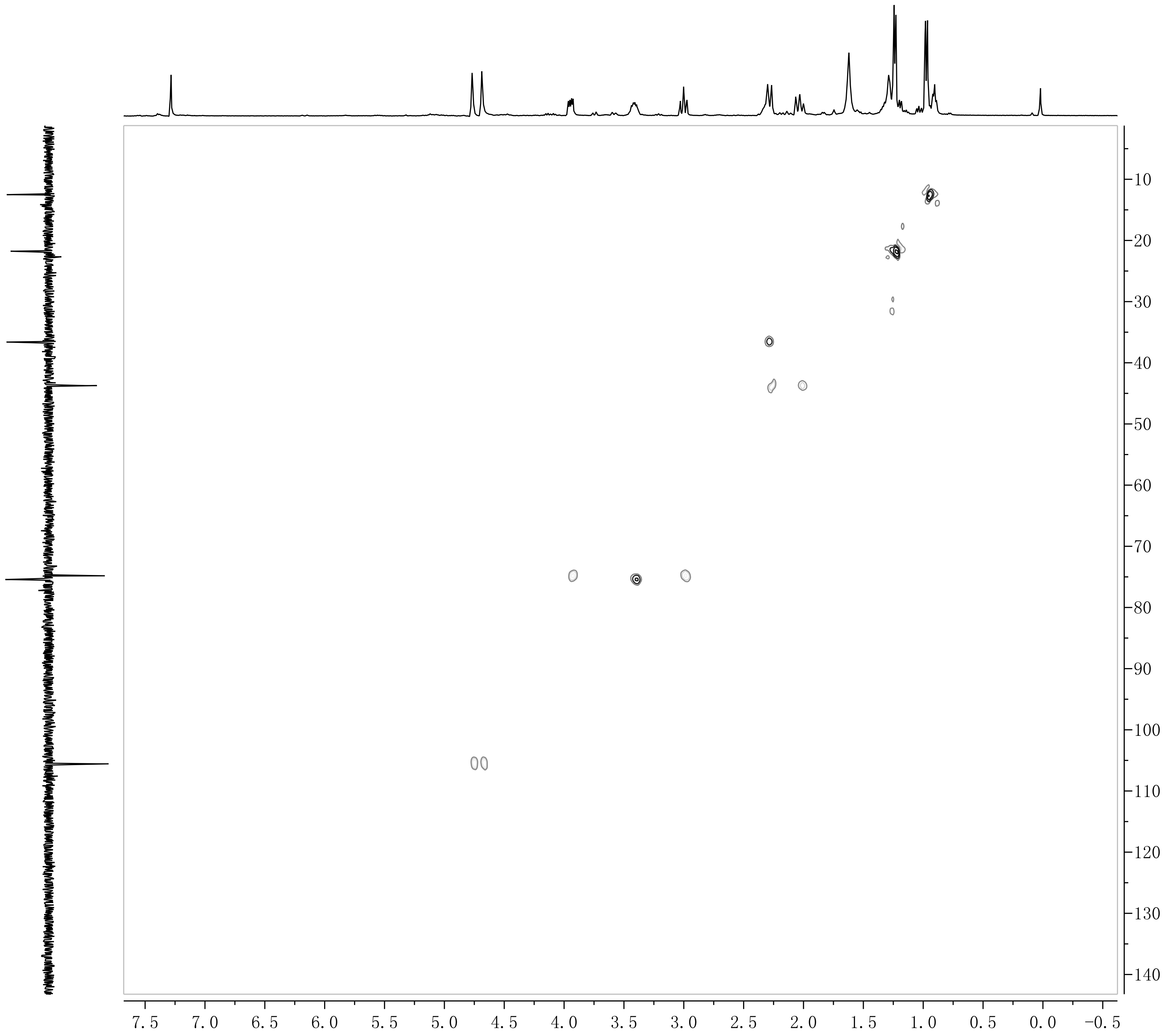

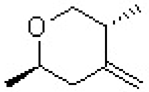

relative configuration

Parameter	Value
Title	xfy-190911-2-s2.35.ser
Comment	
Origin	Bruker BioSpin GmbH
Owner	nmr
Site	
Instrument	spect
Solvent	CDCl3
Temperature	296.2
Pulse Sequence	hsqcedetgp
Experiment	HSQC-EDITED
Number of Scans	2
Receiver Gain	193.1
Relaxation Delay	1.4785
Pulse Width	10.7100
Presaturation Frequency	
Acquisition Time	0.1004
Acquisition Date	2019-09-11T23:34:25
Modification Date	2019-09-12T09:10:07
Spectrometer Frequency	(500.13, 125.77)
Spectral Width	(5102.0, 20833.3)
Lowest Frequency	(-1008.1, -1037.0)
Nucleus	(1H, 13C)
Acquired Size	(512, 256)
Spectral Size	(512, 512)

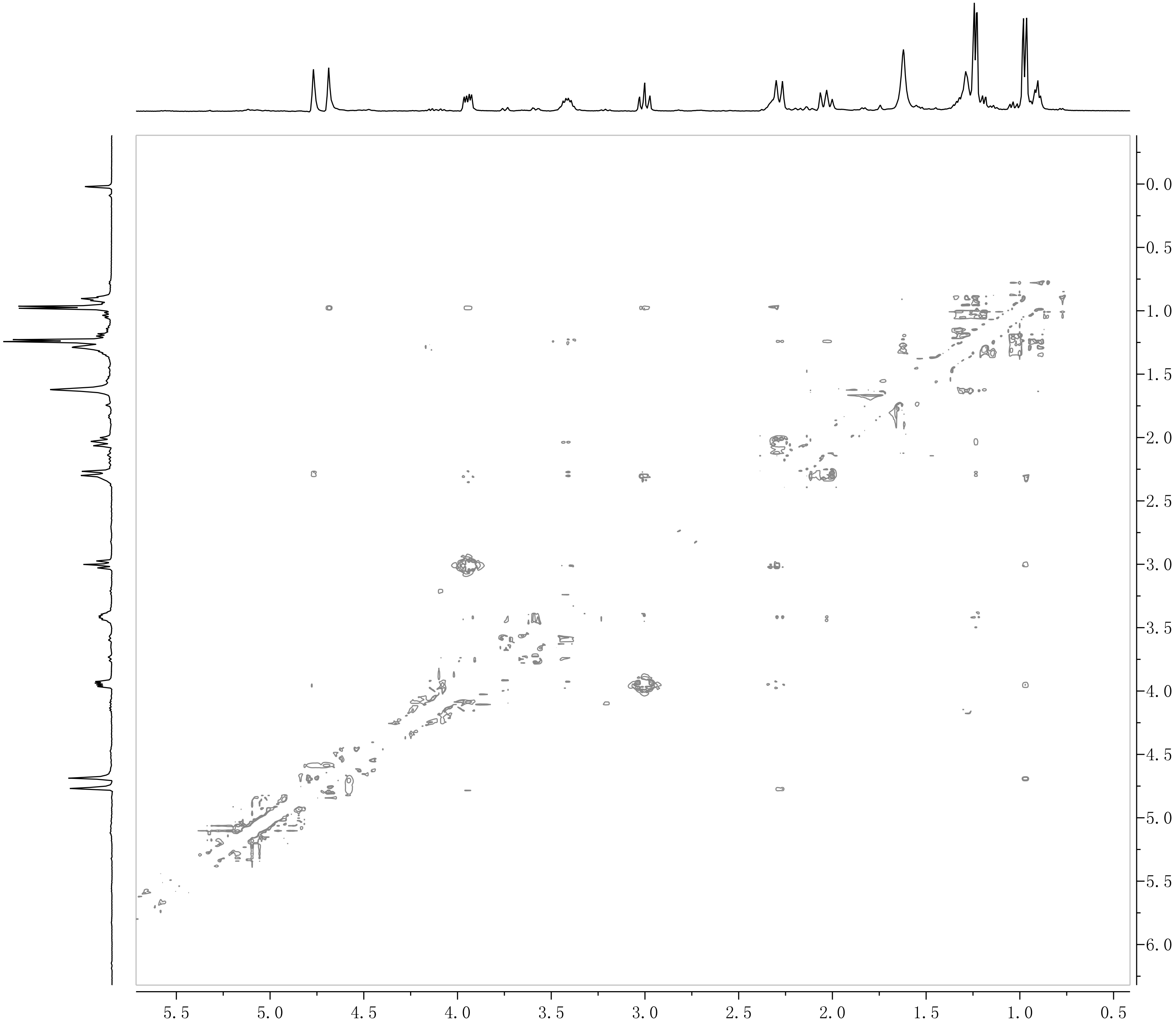

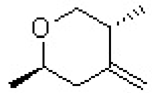

relative configuration

Parameter	Value
Title	xfy-190911-2-s2.36.ser
Comment	
Origin	Bruker BioSpin GmbH
Owner	nmr
Site	
Instrument	spect
Solvent	CDCl3
Temperature	296.1
Pulse Sequence	noesygpphpp
Experiment	NOESY
Number of Scans	24
Receiver Gain	34.4
Relaxation Delay	2.0041
Pulse Width	10.7100
Presaturation Frequency	
Acquisition Time	0.2007
Acquisition Date	2019-09-11T23:51:17
Modification Date	2019-09-12T09:10:08
Spectrometer Frequency	(500.13, 500.13)
Spectral Width	(5102.0, 5102.0)
Lowest Frequency	(-1008.0, -1008.0)
Nucleus	(1H, 1H)
Acquired Size	(1024, 256)
Spectral Size	(1024, 1024)

Parameter	Value
Title	xfy-190602-4-s.1.1.1r
Comment	
Origin	Bruker BioSpin GmbH
Owner	nmr
Site	
Instrument	spect
Solvent	CDCl3
Temperature	296.1
Pulse Sequence	zg30
Experiment	1D
Number of Scans	16
Receiver Gain	54.3
Relaxation Delay	1.0000
Pulse Width	10.7100
Presaturation Frequency	
Acquisition Time	3.2768
Acquisition Date	2019-06-07T17:29:00
Modification Date	2019-06-07T18:45:36
Spectrometer Frequency	500.13
Spectral Width	10000.0
Lowest Frequency	-1915.0
Nucleus	1H
Acquired Size	32768
Spectral Size	65536

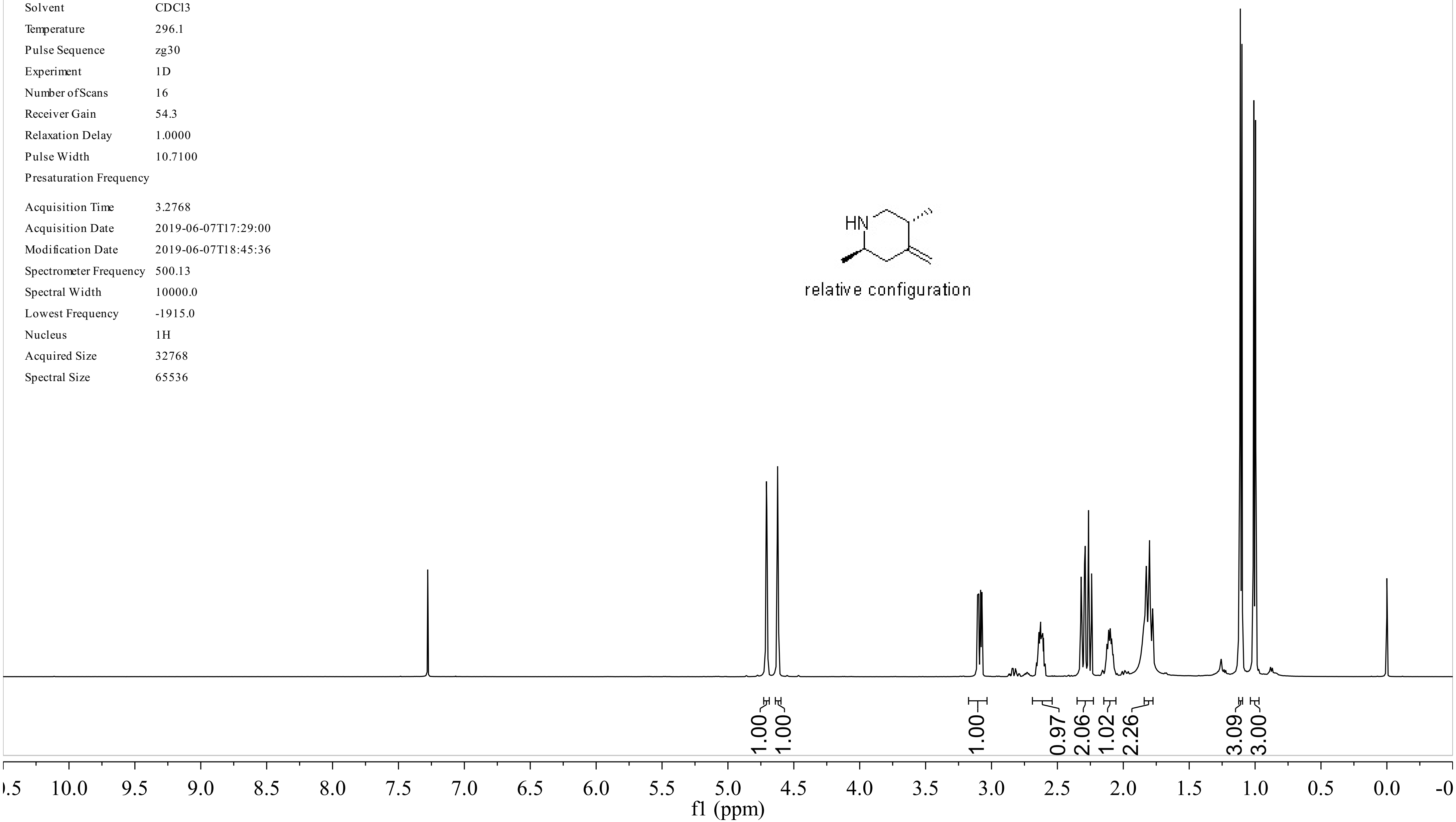

Parameter	Value
Title	xfy-190602-4-s.2.1.1r
Comment	
Origin	Bruker BioSpin GmbH
Owner	nmr
Site	
Instrument	spect
Solvent	CDCl3
Temperature	296.1
Pulse Sequence	zgpg30
Experiment	1D
Number of Scans	12
Receiver Gain	193.1
Relaxation Delay	2.0000
Pulse Width	9.6000
Presaturation Frequency	
Acquisition Time	1.1010
Acquisition Date	2019-06-07T17:31:00
Modification Date	2019-06-07T18:45:36
Spectrometer Frequency	125.76
Spectral Width	29761.9
Lowest Frequency	-2291.4
Nucleus	13C
Acquired Size	32768
Spectral Size	32768

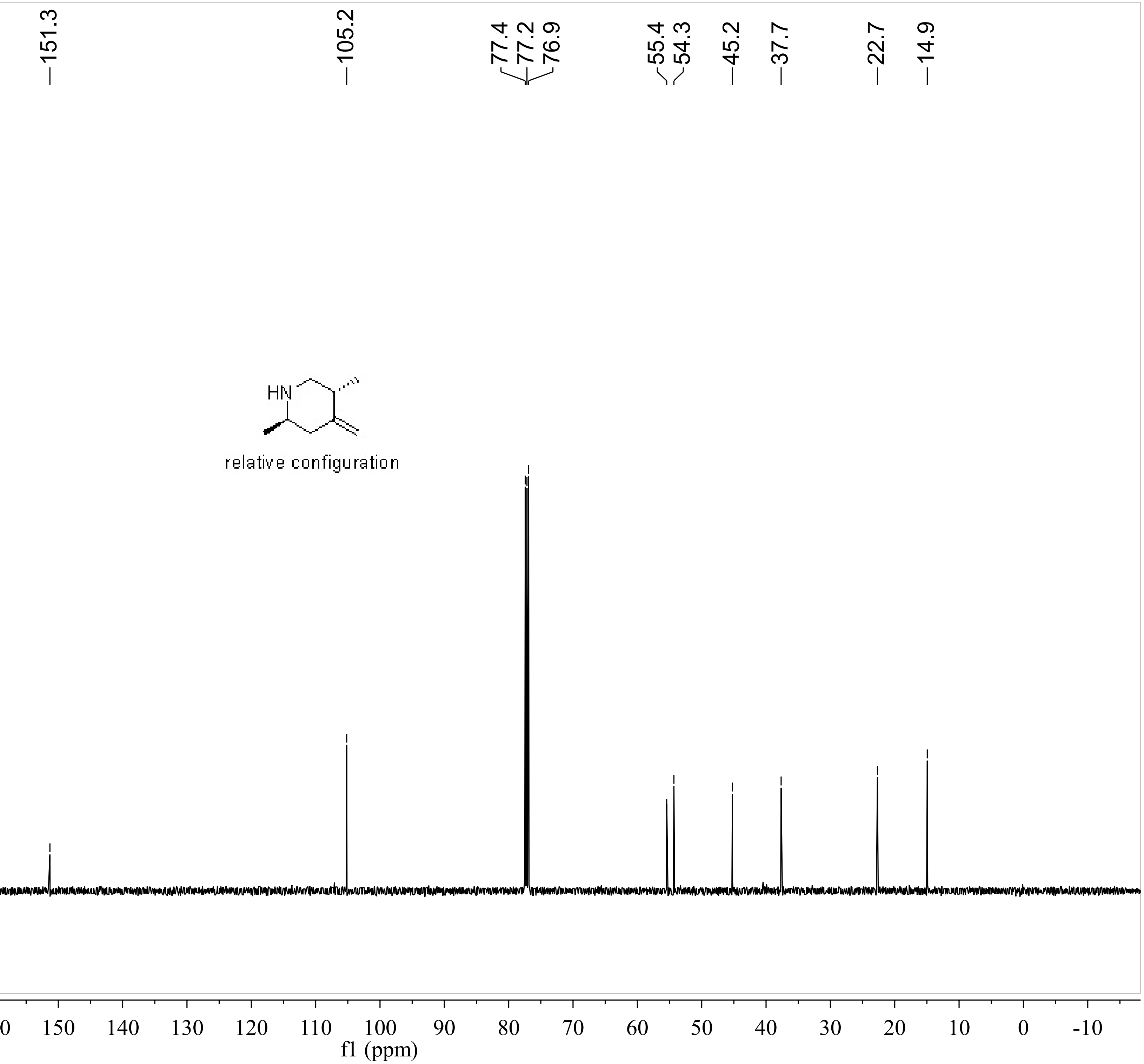

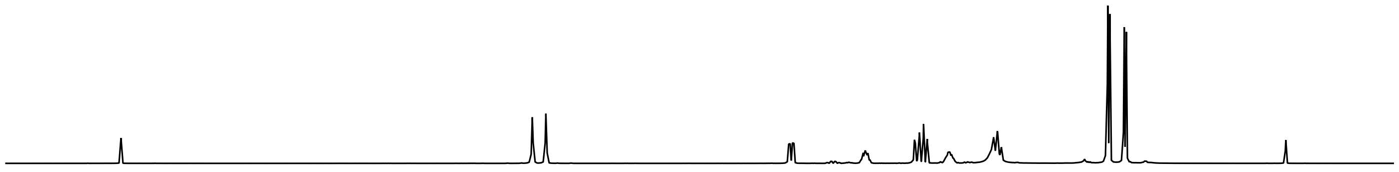

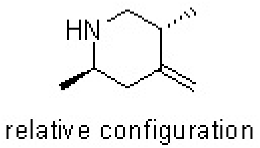

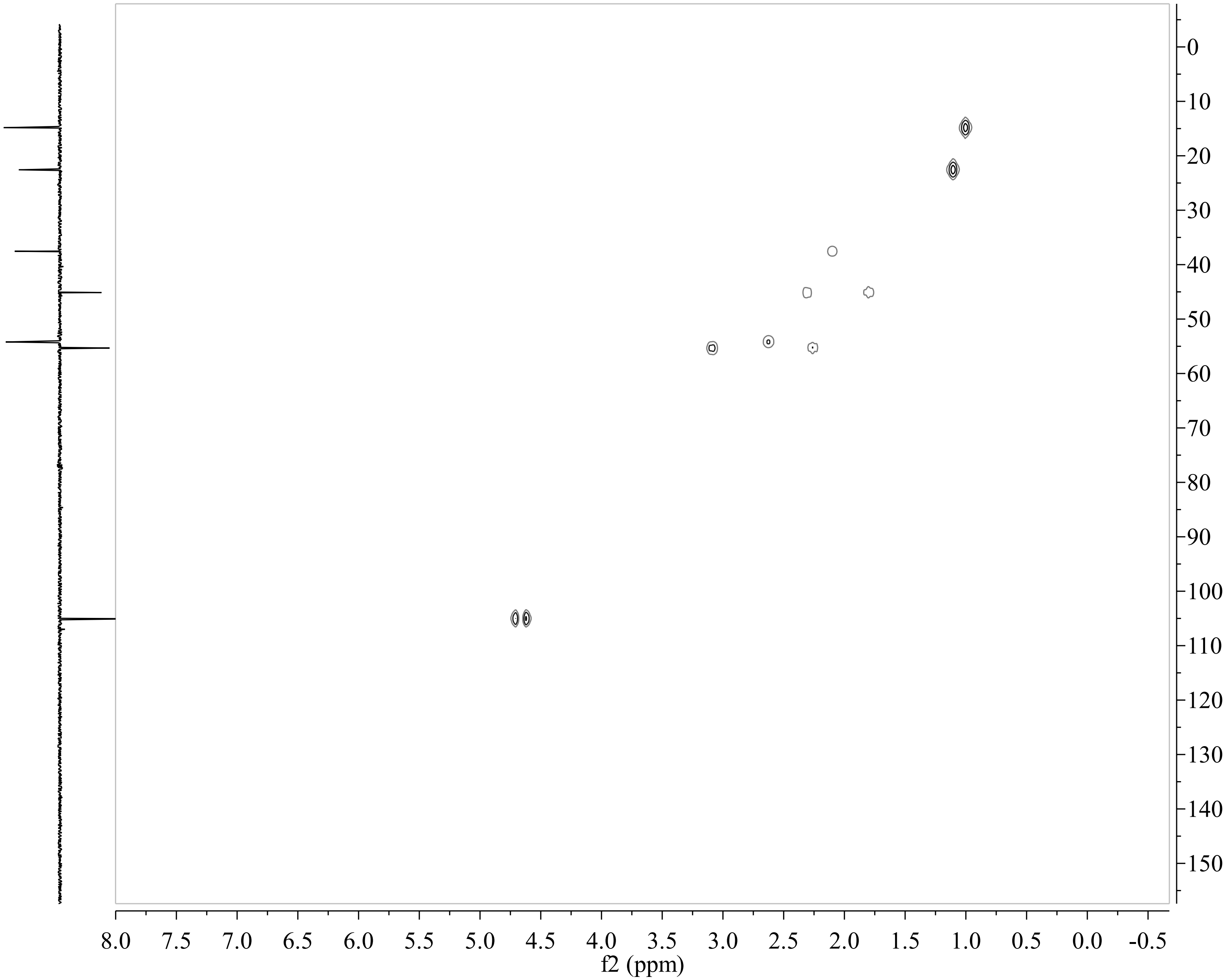

Parameter	Value
Title	xfy-190602-4-s.4.ser
Comment	
Origin	Bruker BioSpin GmbH
Owner	nmr
Site	
Instrument	spect
Solvent	CDCl3
Temperature	296.2
Pulse Sequence	hsqcedetgp
Experiment	HSQC-EDITED
Number of Scans	2
Receiver Gain	193.1
Relaxation Delay	1.4611
Pulse Width	10.7100
Presaturation Frequency	
Acquisition Time	0.1178
Acquisition Date	2019-06-07T17:34:00
Modification Date	2019-06-07T18:45:37
Spectrometer Frequency	(500.13, 125.77)
Spectral Width	(4347.8, 20833.3)
Lowest Frequency	(-346.4, -1037.0)
Nucleus	(1H, 13C)
Acquired Size	(512, 256)
Spectral Size	(512, 512)

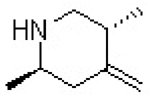

relative configuration

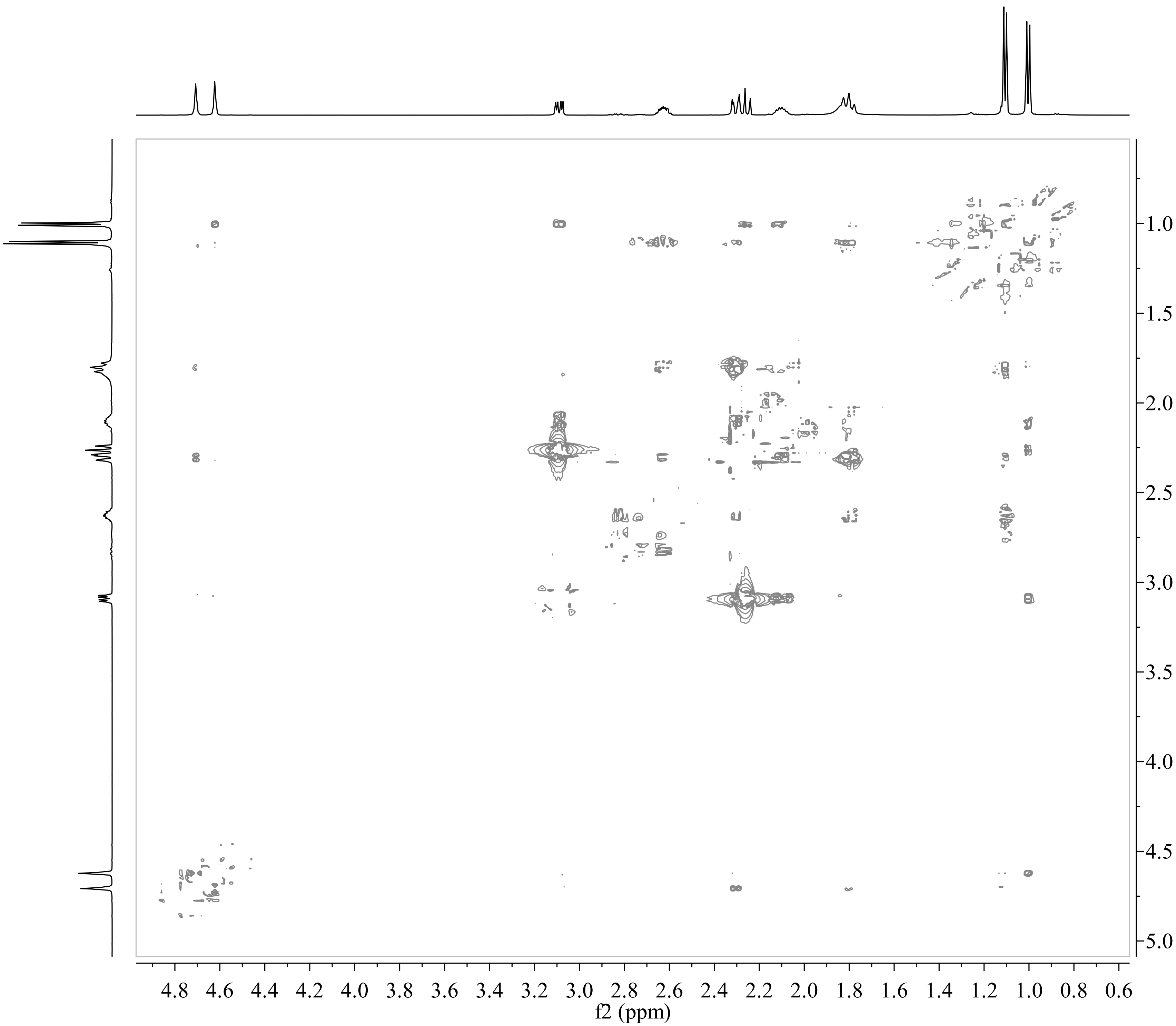

Parameter	Value
Title	xfy-190602-4-s.5.ser
Comment	
Origin	Bruker BioSpin GmbH
Owner	nmr
Site	
Instrument	spect
Solvent	CDCl3
Temperature	296.2
Pulse Sequence	noesygpphpp
Experiment	NOESY
Number of Scans	4
Receiver Gain	44.7
Relaxation Delay	1.9693
Pulse Width	10.7100
Presaturation Frequency	
Acquisition Time	0.2355
Acquisition Date	2019-06-07T17:50:00
Modification Date	2019-06-07T18:45:39
Spectrometer Frequency	(500.13, 500.13)
Spectral Width	(4347.8, 4347.8)
Lowest Frequency	(-346.4, -346.4)
Nucleus	(1H, 1H)
Acquired Size	(1024, 256)
Spectral Size	(1024, 1024)

Parameter	Value
Title	xfy-190602-1-s.41.1.1r
Comment	
Origin	Bruker BioSpin GmbH
Owner	nmr
Site	
Instrument	spect
Solvent	CDCl3
Temperature	296.1
Pulse Sequence	zg30
Experiment	1D
Number of Scans	4
Receiver Gain	54.3
Relaxation Delay	1.0000
Pulse Width	10.7100
Presaturation Frequency	
Acquisition Time	3.2768
Acquisition Date	2019-06-04T18:13:13
Modification Date	2019-06-04T19:13:05
Spectrometer Frequency	500.13
Spectral Width	10000.0
Lowest Frequency	-1911.5
Nucleus	1H
Acquired Size	32768
Spectral Size	65536

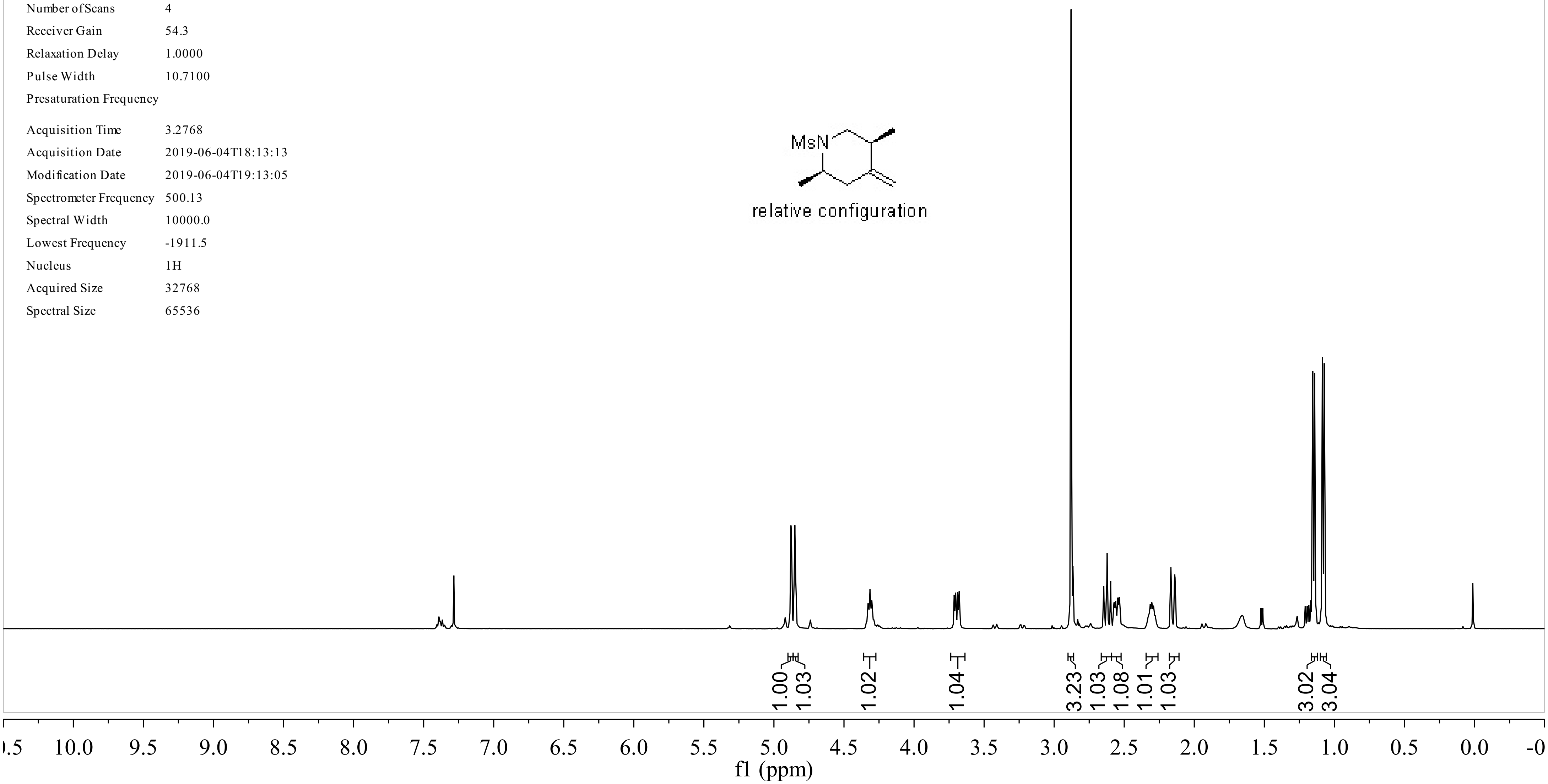

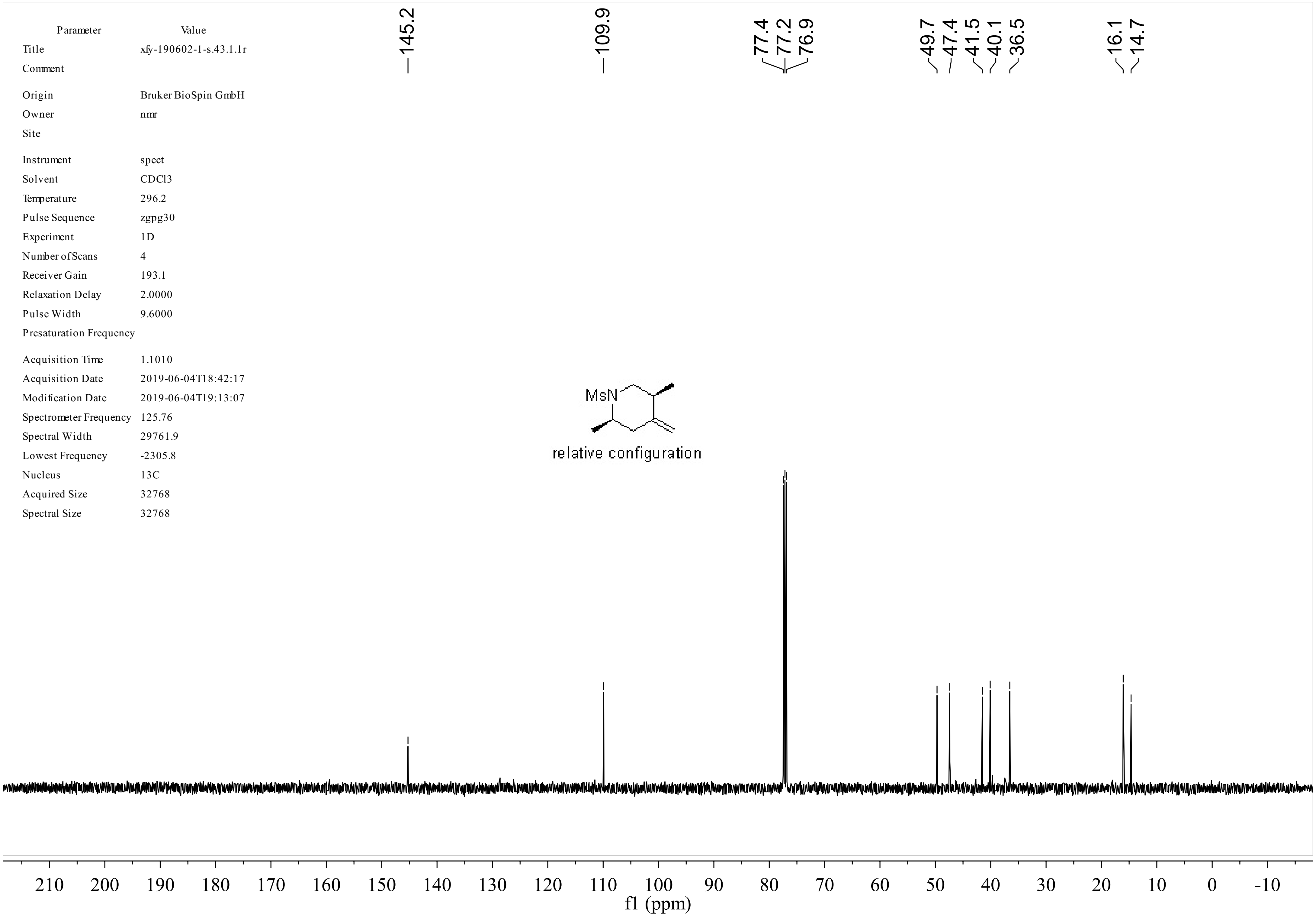

Parameter	Value
Title	xfy-190602-1-s.43.1.1r
Comment	
Origin	Bruker BioSpin GmbH
Owner	nmr
Site	
Instrument	spect
Solvent	CDCl3
Temperature	296.2
Pulse Sequence	zgpg30
Experiment	1D
Number of Scans	4
Receiver Gain	193.1
Relaxation Delay	2.0000
Pulse Width	9.6000
Presaturation Frequency	
Acquisition Time	1.1010
Acquisition Date	2019-06-04T18:42:17
Modification Date	2019-06-04T19:13:07
Spectrometer Frequency	125.76
Spectral Width	29761.9
Lowest Frequency	-2305.8
Nucleus	13C
Acquired Size	32768
Spectral Size	32768

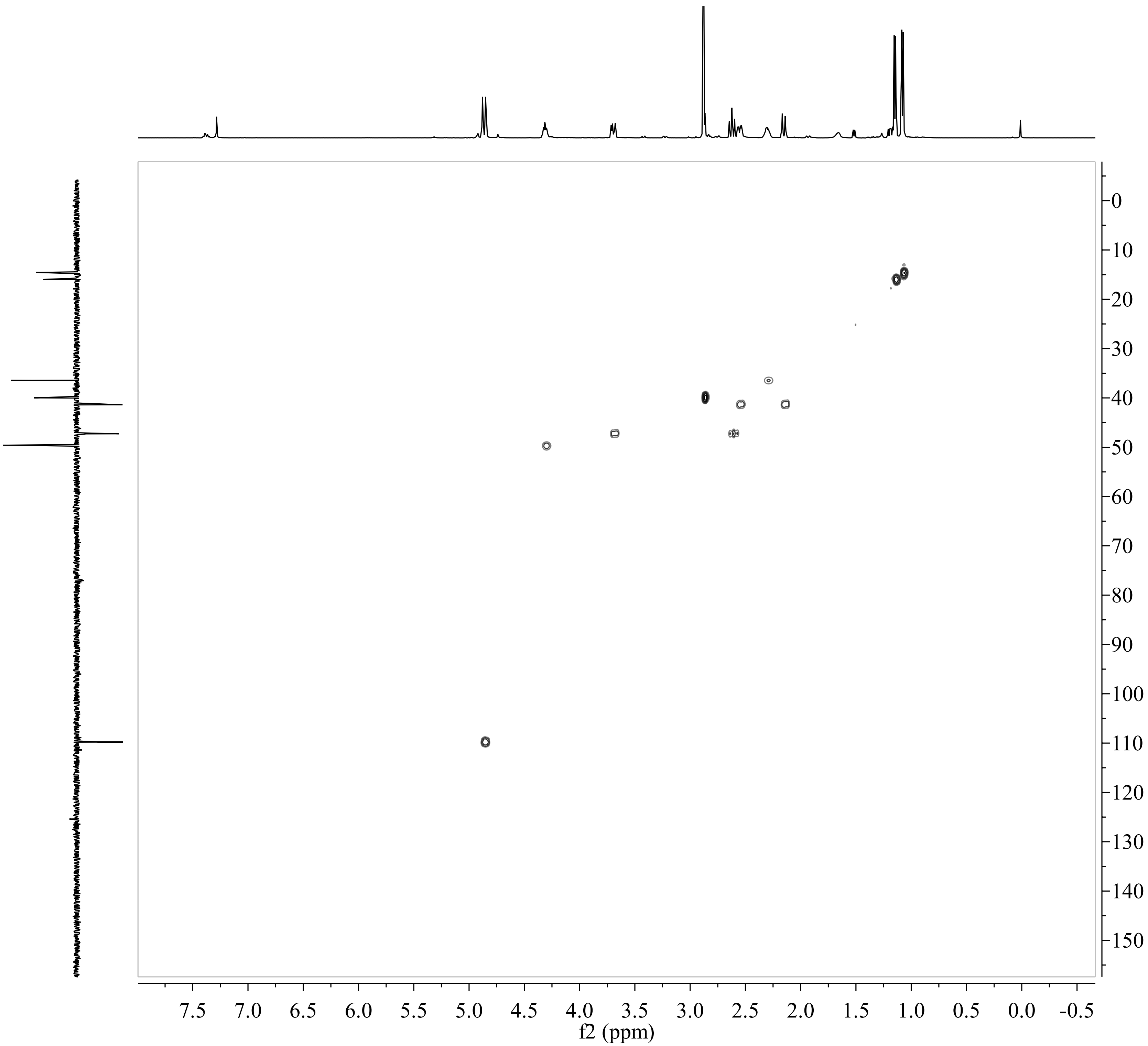

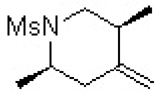

relative configuration

Parameter	Value
Title	xfy-190602-4-dMs.33.ser
Comment	
Origin	Bruker BioSpin GmbH
Owner	nmr
Site	
Instrument	spect
Solvent	CDCl3
Temperature	296.8
Pulse Sequence	hsqcetgp
Experiment	HSQC
Number of Scans	2
Receiver Gain	196.4
Relaxation Delay	1.4511
Pulse Width	8.7300
Presaturation Frequency	
Acquisition Time	0.1475
Acquisition Date	2019-06-05T08:34:03
Modification Date	2019-06-05T09:42:40
Spectrometer Frequency	(400.13, 100.62)
Spectral Width	(3472.2, 16666.7)
Lowest Frequency	(-273.2, -829.1)
Nucleus	(1H, 13C)
Acquired Size	(512, 256)
Spectral Size	(512, 512)

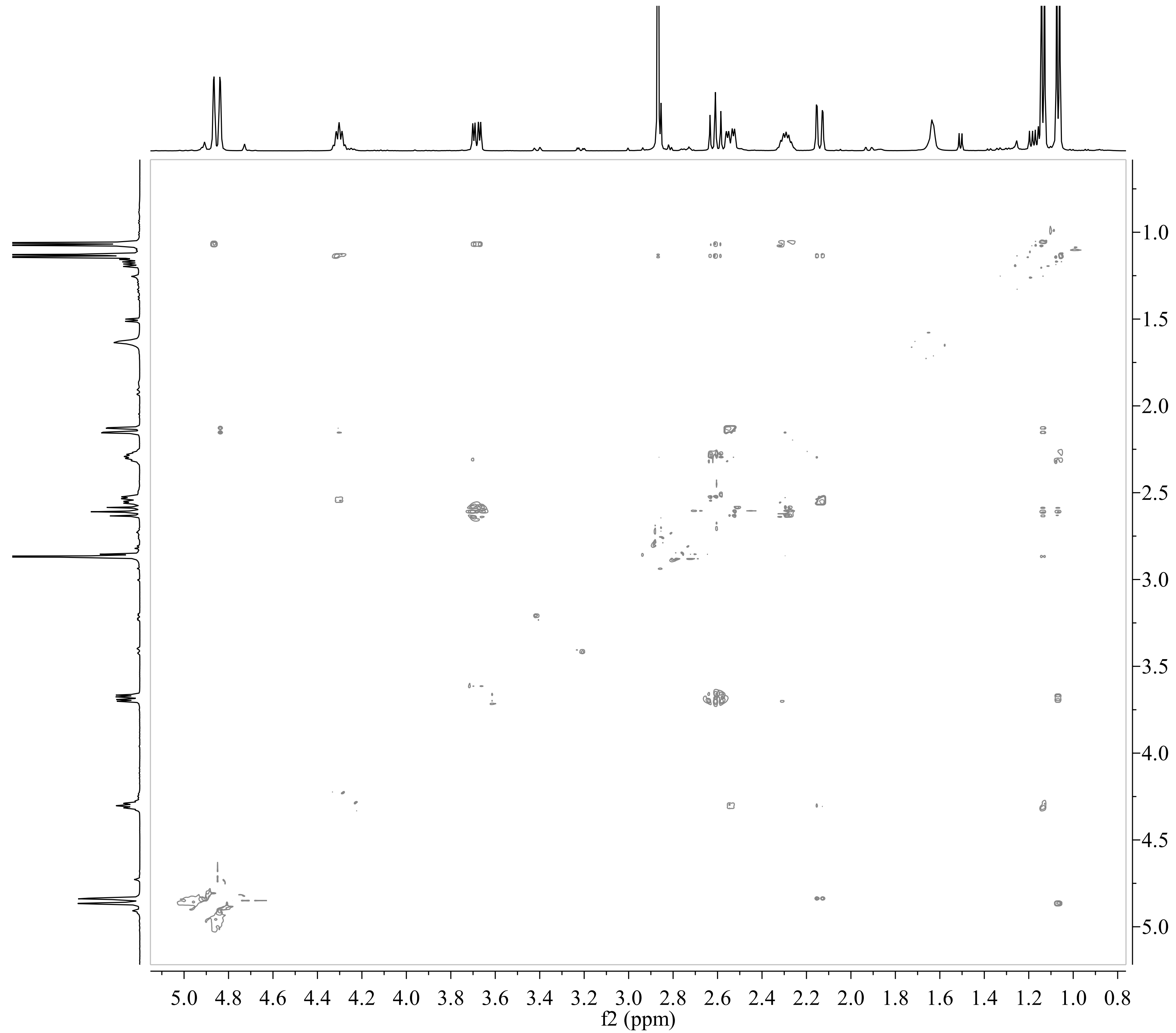

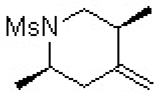

relative configuration

Parameter	Value
Title	xfy-190601-1-s.42.ser
Comment	
Origin	Bruker BioSpin GmbH
Owner	nmr
Site	
Instrument	spect
Solvent	CDCl3
Temperature	296.1
Pulse Sequence	noesygpphpp
Experiment	NOESY
Number of Scans	8
Receiver Gain	31.1
Relaxation Delay	1.9652
Pulse Width	10.7100
Presaturation Frequency	
Acquisition Time	0.2396
Acquisition Date	2019-06-08T22:48:00
Modification Date	2019-06-09T21:05:17
Spectrometer Frequency	(500.13, 500.13)
Spectral Width	(4273.5, 4273.5)
Lowest Frequency	(-274.8, -274.8)
Nucleus	(1H, 1H)
Acquired Size	(1024, 256)
Spectral Size	(1024, 1024)

Parameter	Value
Title	xfy-190602-1-s.51.1.1r
Comment	
Origin	Bruker BioSpin GmbH
Owner	nmr
Site	
Instrument	spect
Solvent	CDCl3
Temperature	296.5
Pulse Sequence	zg30
Experiment	1D
Number of Scans	8
Receiver Gain	70.3
Relaxation Delay	1.0000
Pulse Width	8.7300
Presaturation Frequency	
Acquisition Time	1.9999
Acquisition Date	2019-06-05T06:22:15
Modification Date	2019-06-05T10:18:43
Spectrometer Frequency	400.13
Spectral Width	8012.8
Lowest Frequency	-1541.5
Nucleus	1H
Acquired Size	16025
Spectral Size	65536

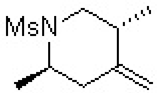

relative configuration

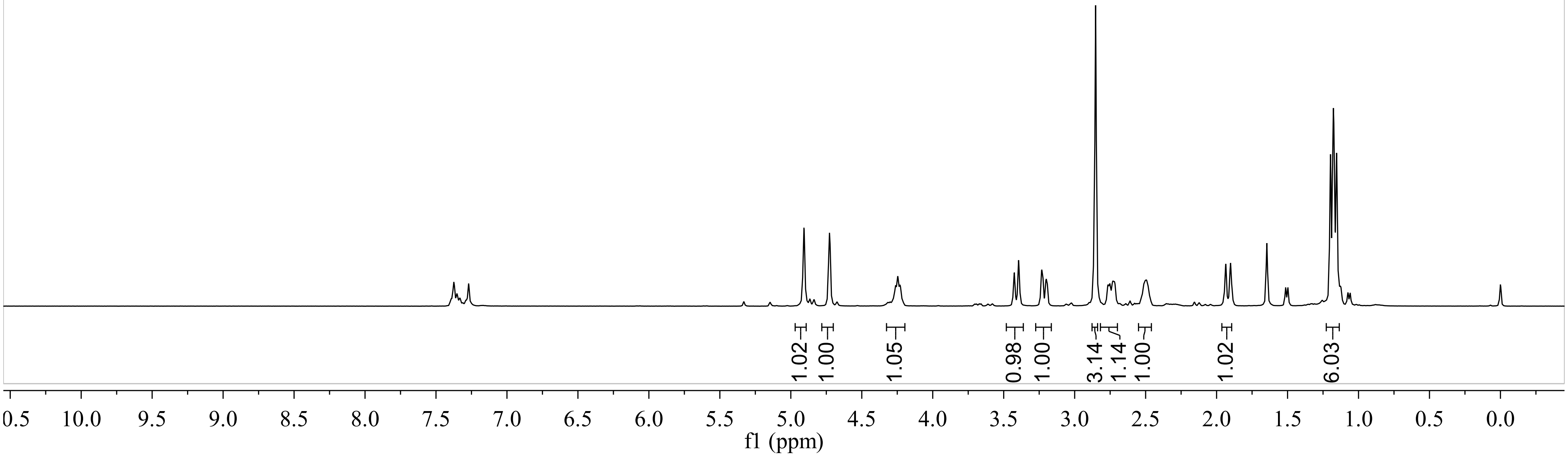

Parameter	Value
Title	xfy-190602-1-s.53.fid
Comment	
Origin	Bruker BioSpin GmbH
Owner	nmr
Site	
Instrument	spect
Solvent	CDCl3
Temperature	296.8
Pulse Sequence	zgpg30
Experiment	1D
Number of Scans	24
Receiver Gain	196.4
Relaxation Delay	2.0000
Pulse Width	10.0000
Presaturation Frequency	
Acquisition Time	1.3631
Acquisition Date	2019-06-05T06:24:51
Modification Date	2019-06-05T10:18:44
Spectrometer Frequency	100.62
Spectral Width	24038.5
Lowest Frequency	-1958.0
Nucleus	13C
Acquired Size	32768
Spectral Size	65536

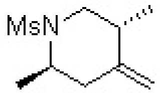

relative configuration

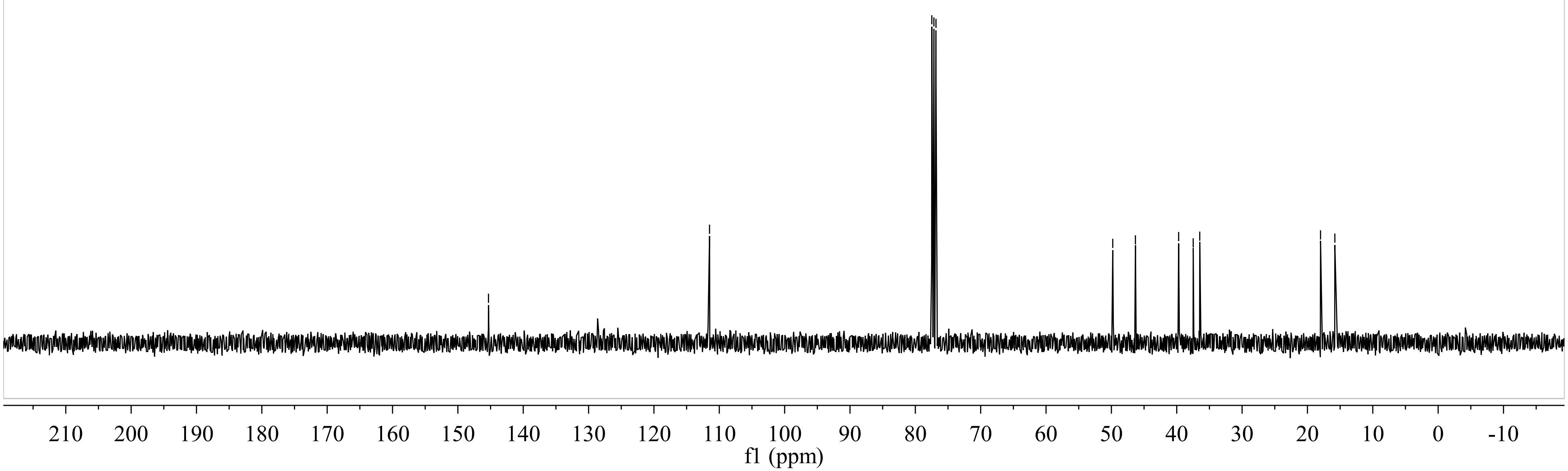

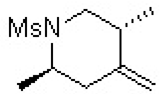

relative configuration

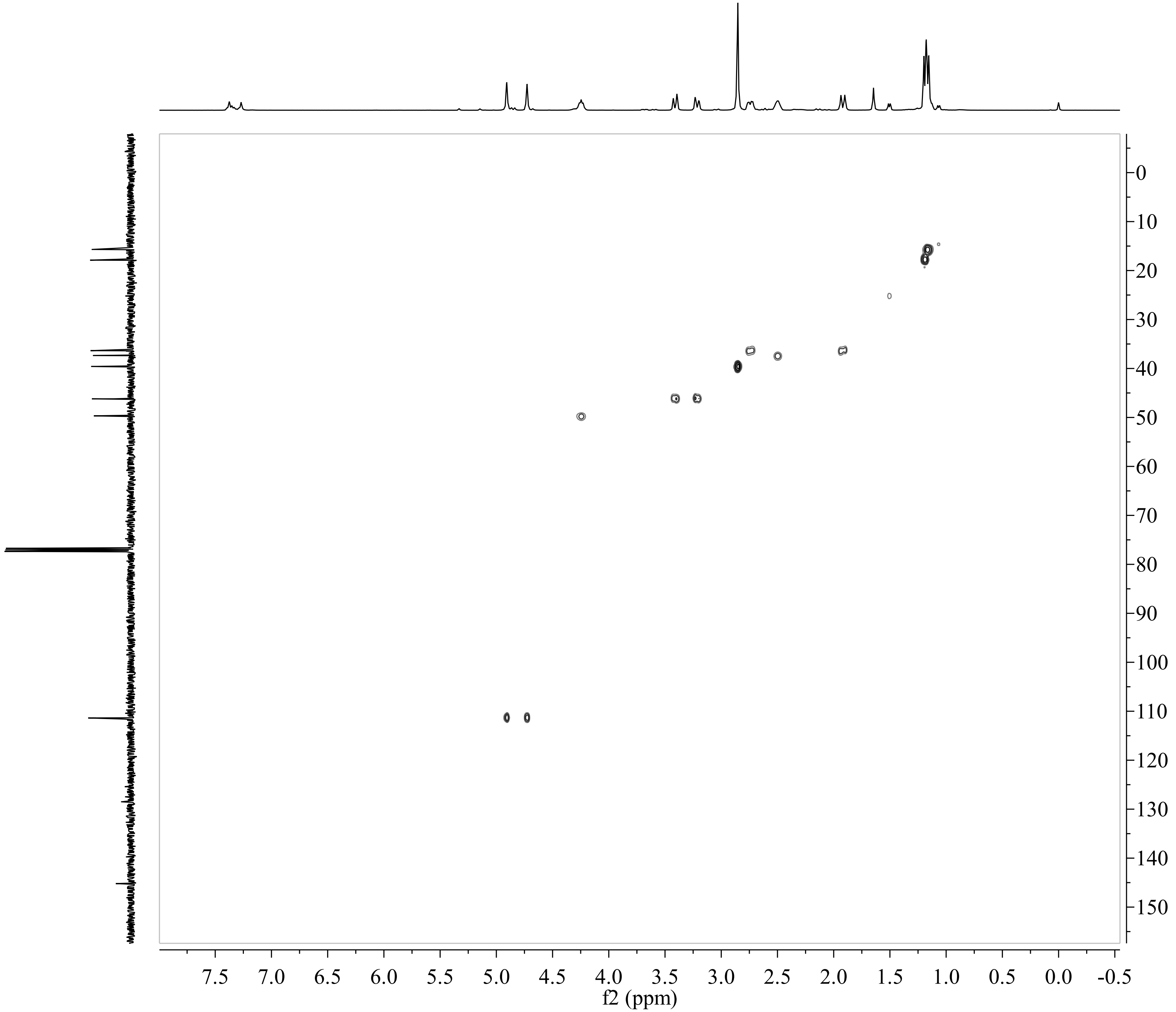

Parameter	Value
Title	xfy-190602-1-s.54.ser
Comment	
Origin	Bruker BioSpin GmbH
Owner	nmr
Site	
Instrument	spect
Solvent	CDCl3
Temperature	296.8
Pulse Sequence	hsqcetgp
Experiment	HSQC
Number of Scans	2
Receiver Gain	196.4
Relaxation Delay	1.4490
Pulse Width	8.7300
Presaturation Frequency	
Acquisition Time	0.1495
Acquisition Date	2019-06-05T06:26:24
Modification Date	2019-06-05T10:18:44
Spectrometer Frequency	(400.13, 100.62)
Spectral Width	(3424.7, 16666.7)
Lowest Frequency	(-225.2, -829.1)
Nucleus	(1H, 13C)
Acquired Size	(512, 256)
Spectral Size	(512, 512)

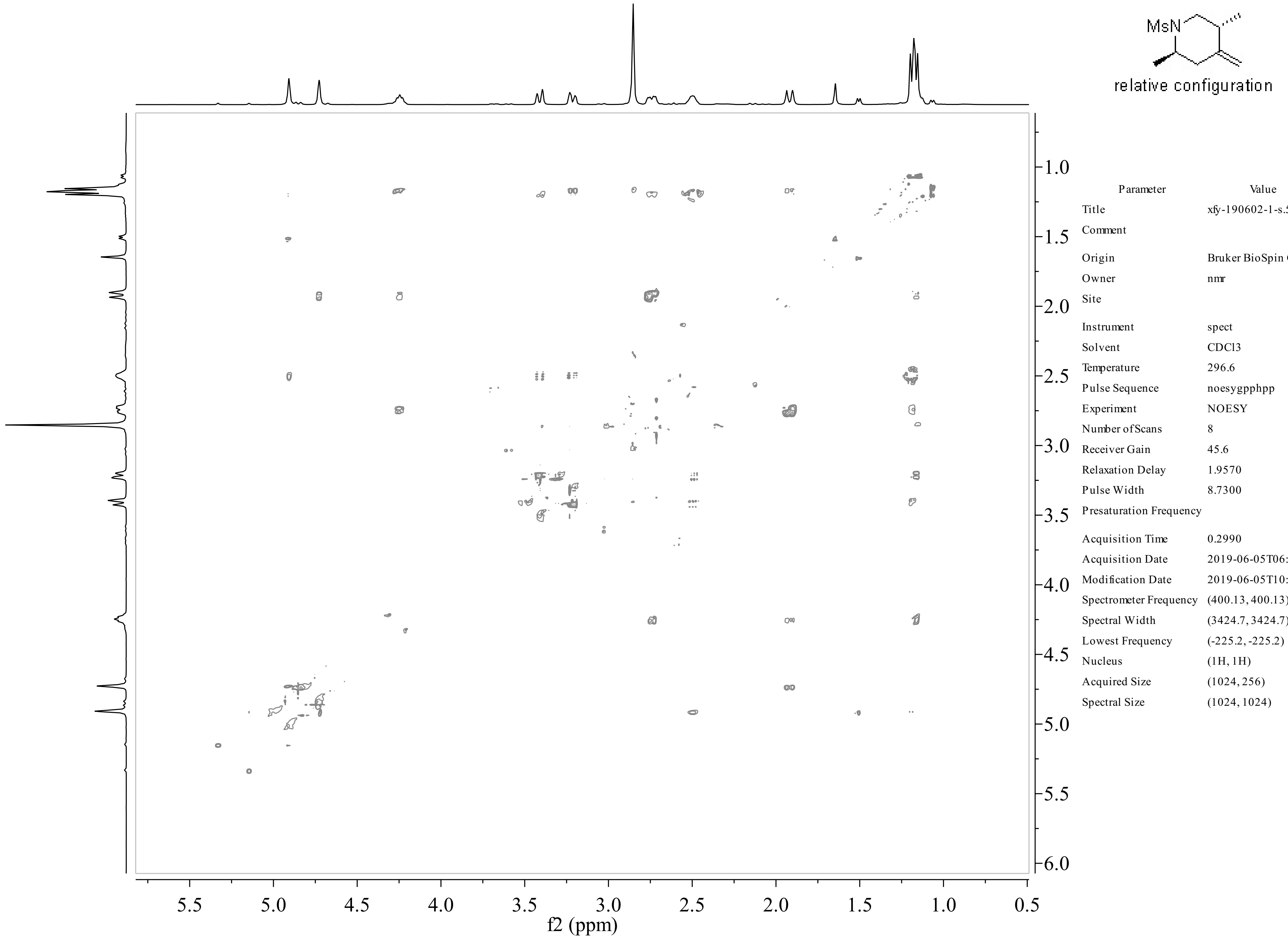

Parameter	Value
Title	xfy-190602-1-s.55.ser
Comment	
Origin	Bruker BioSpin GmbH
Owner	nmr
Site	
Instrument	spect
Solvent	CDCl3
Temperature	296.6
Pulse Sequence	noesygpphpp
Experiment	NOESY
Number of Scans	8
Receiver Gain	45.6
Relaxation Delay	1.9570
Pulse Width	8.7300
Presaturation Frequency	
Acquisition Time	0.2990
Acquisition Date	2019-06-05T06:42:43
Modification Date	2019-06-05T10:18:44
Spectrometer Frequency	(400.13, 400.13)
Spectral Width	(3424.7, 3424.7)
Lowest Frequency	(-225.2, -225.2)
Nucleus	(1H, 1H)
Acquired Size	(1024, 256)
Spectral Size	(1024, 1024)

Parameter	Value
Origin	Bruker BioSpin GmbH
Spectrometer	spect
Solvent	CDCl3
Temperature	299.9
Pulse Sequence	zg30
Experiment	1D
Number of Scans	8
Receiver Gain	55
Relaxation Delay	1.0000
Pulse Width	10.0000
Acquisition Time	1.9999
Spectrometer Frequency	400.13
Spectral Width	8012.8
Lowest Frequency	-1548.2
Nucleus	1H
Acquired Size	16025
Spectral Size	65536

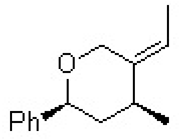

reletive configuration only

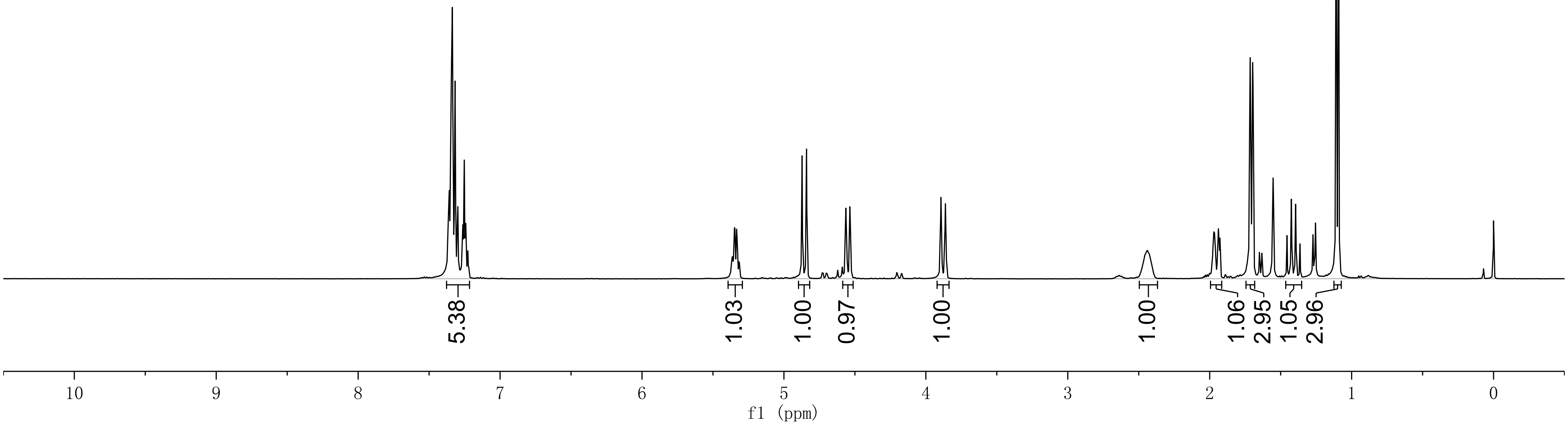

Parameter	Value
Origin	Bruker BioSpin GmbH
Spectrometer	spect
Solvent	CDCl3
Temperature	300.6
Pulse Sequence	zgpg30
Experiment	1D
Number of Scans	250
Receiver Gain	196
Relaxation Delay	2.0000
Pulse Width	10.0000
Acquisition Time	1.3631
Spectrometer Frequency	100.62
Spectral Width	24038.5
Lowest Frequency	-1929.6
Nucleus	13C
Acquired Size	32768
Spectral Size	65536

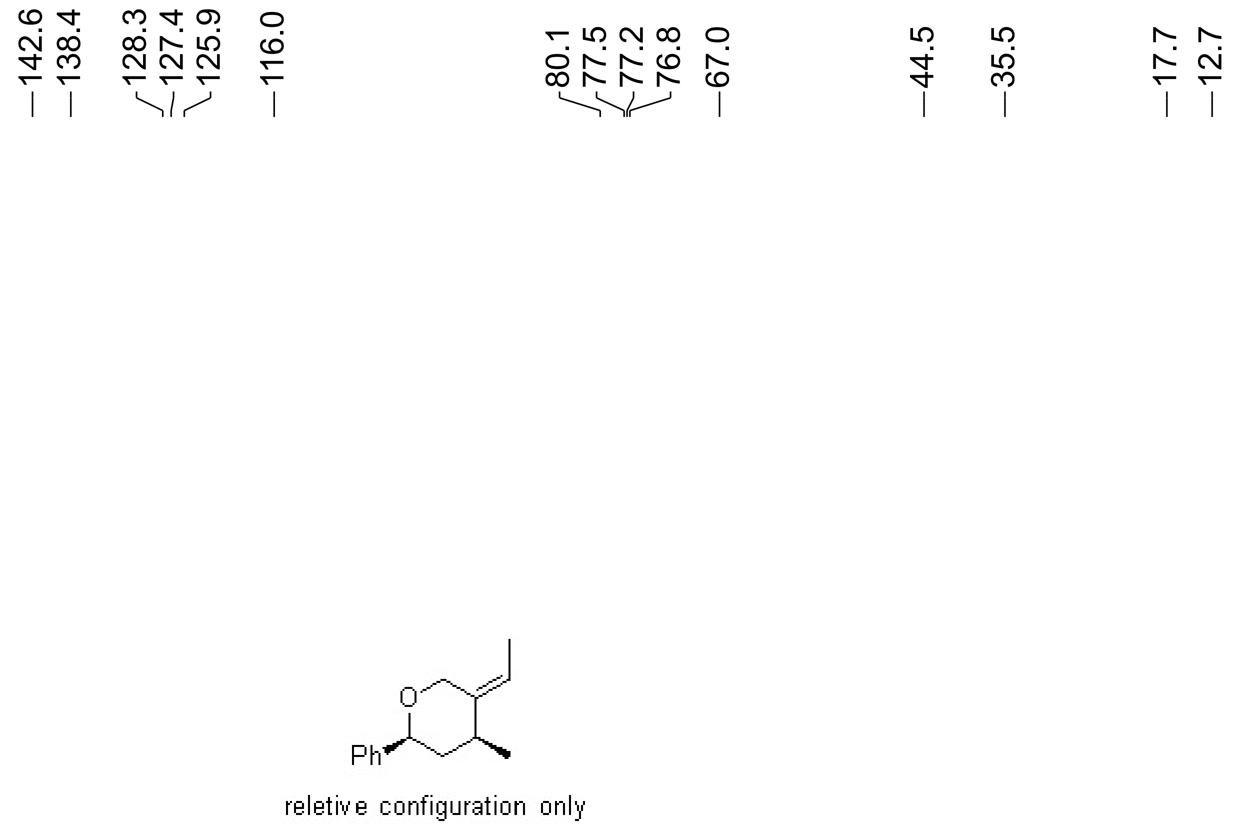

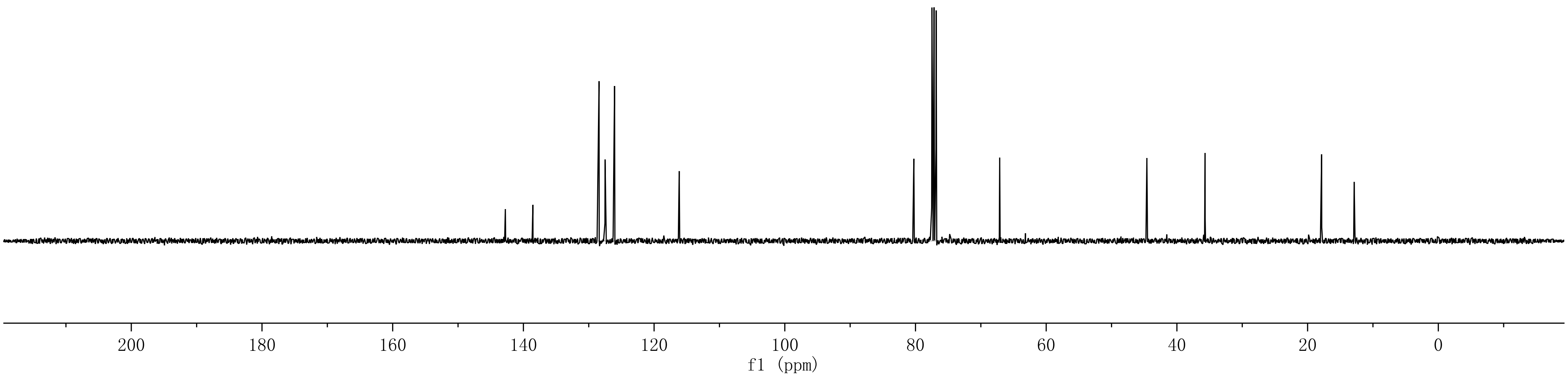

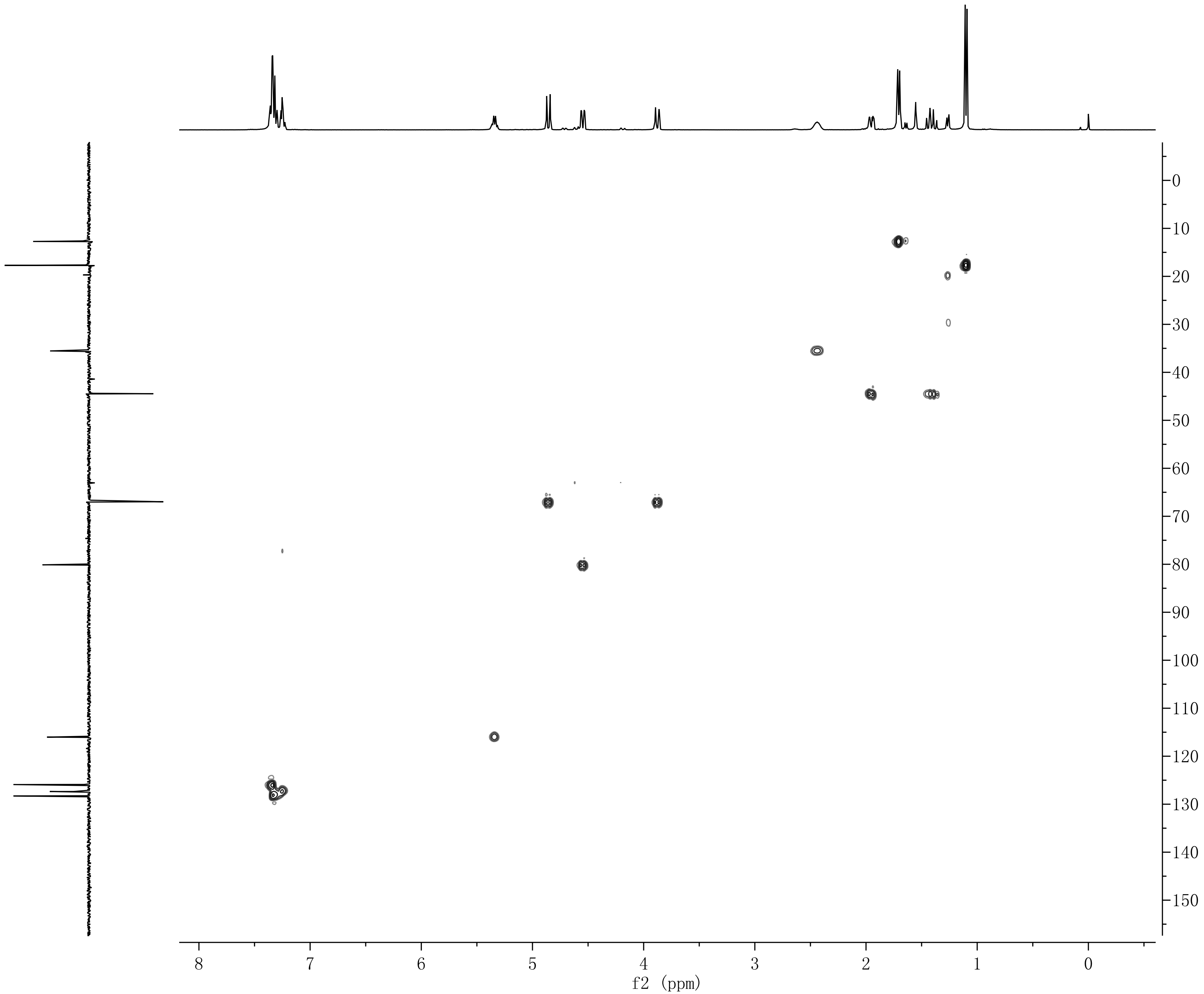

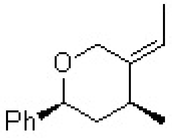

reletive configuration only

Parameter	Value
Origin	Bruker BioSpin GmbH
Spectrometer	spect
Solvent	CDCl3
Temperature	300.2
Pulse Sequence	hsqcetgp
Experiment	HSQC
Number of Scans	2
Receiver Gain	196
Relaxation Delay	1.4531
Pulse Width	10.0000
Acquisition Time	0.1454
Spectrometer Frequency	(400.13, 100.62)
Spectral Width	(3521.1, 16666.7)
Lowest Frequency	(-249.1, -829.1)
Nucleus	(1H, 13C)
Acquired Size	(512, 256)
Spectral Size	(512, 512)

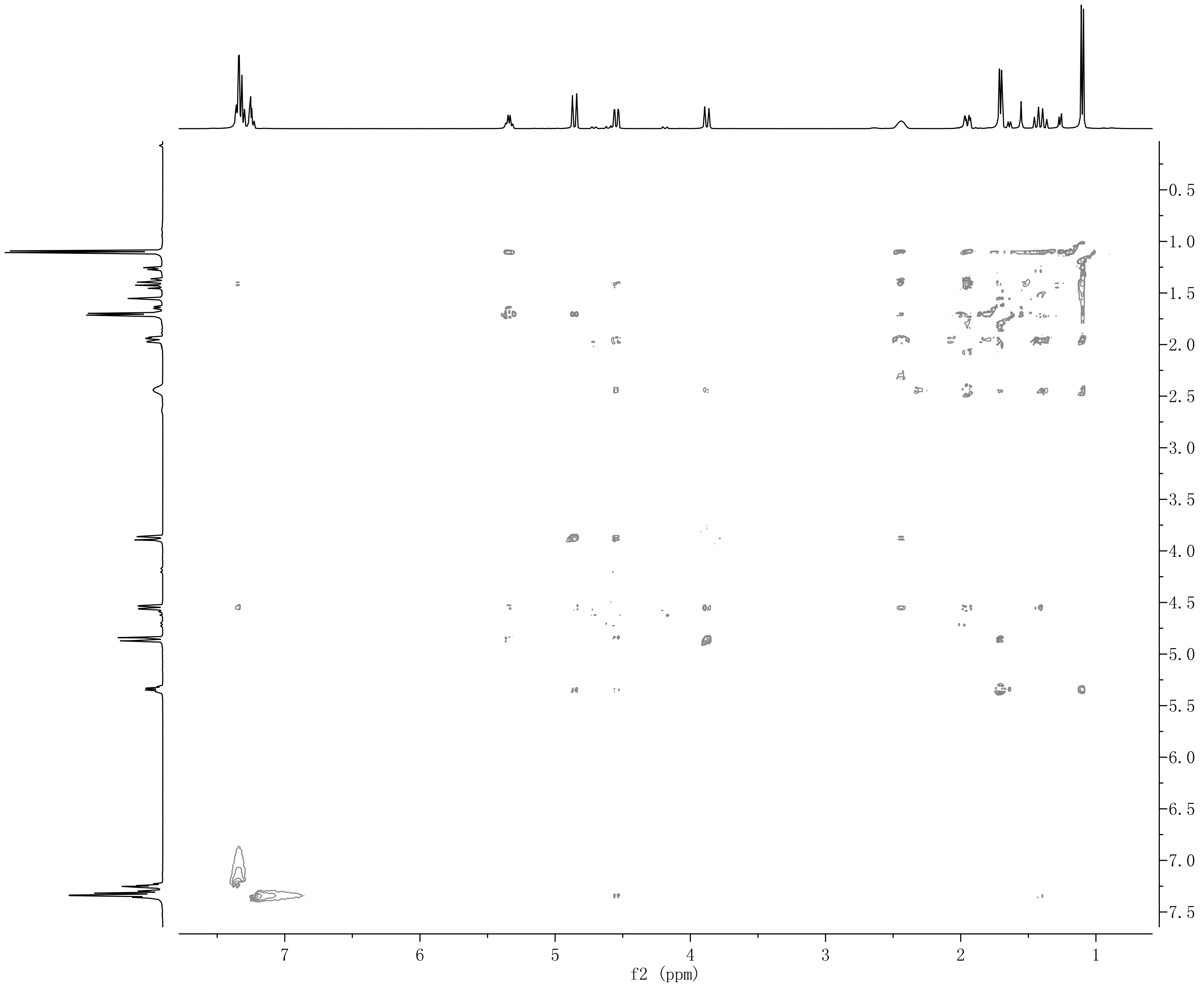

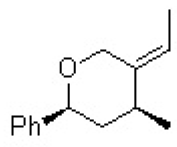

reletive configuration only

Parameter	Value
Origin	Bruker BioSpin GmbH
Spectrometer	spect
Solvent	CDCl3
Temperature	300.0
Pulse Sequence	noesygpphpp
Experiment	NOESY
Number of Scans	4
Receiver Gain	32
Relaxation Delay	1.9631
Pulse Width	10.0000
Acquisition Time	0.2929
Spectrometer Frequency	(400.13, 400.13)
Spectral Width	(3496.5, 3496.5)
Lowest Frequency	(-229.9, -229.9)
Nucleus	(1H, 1H)
Acquired Size	(1024, 256)
Spectral Size	(1024, 1024)

Parameter	Value
Title	xfy-190327-1-s.1.fid
Comment	
Origin	Bruker BioSpin GmbH
Owner	nmr
Site	
Instrument	spect
Solvent	CDCl3
Temperature	296.1
Pulse Sequence	zg30
Experiment	1D
Number of Scans	4
Receiver Gain	31.1
Relaxation Delay	1.0000
Pulse Width	10.7100
Presaturation Frequency	
Acquisition Time	3.2768
Acquisition Date	2019-03-27T18:08:35
Modification Date	2019-03-28T08:47:27
Spectrometer Frequency	500.13
Spectral Width	10000.0
Lowest Frequency	-1926.6
Nucleus	1H
Acquired Size	32768
Spectral Size	65536

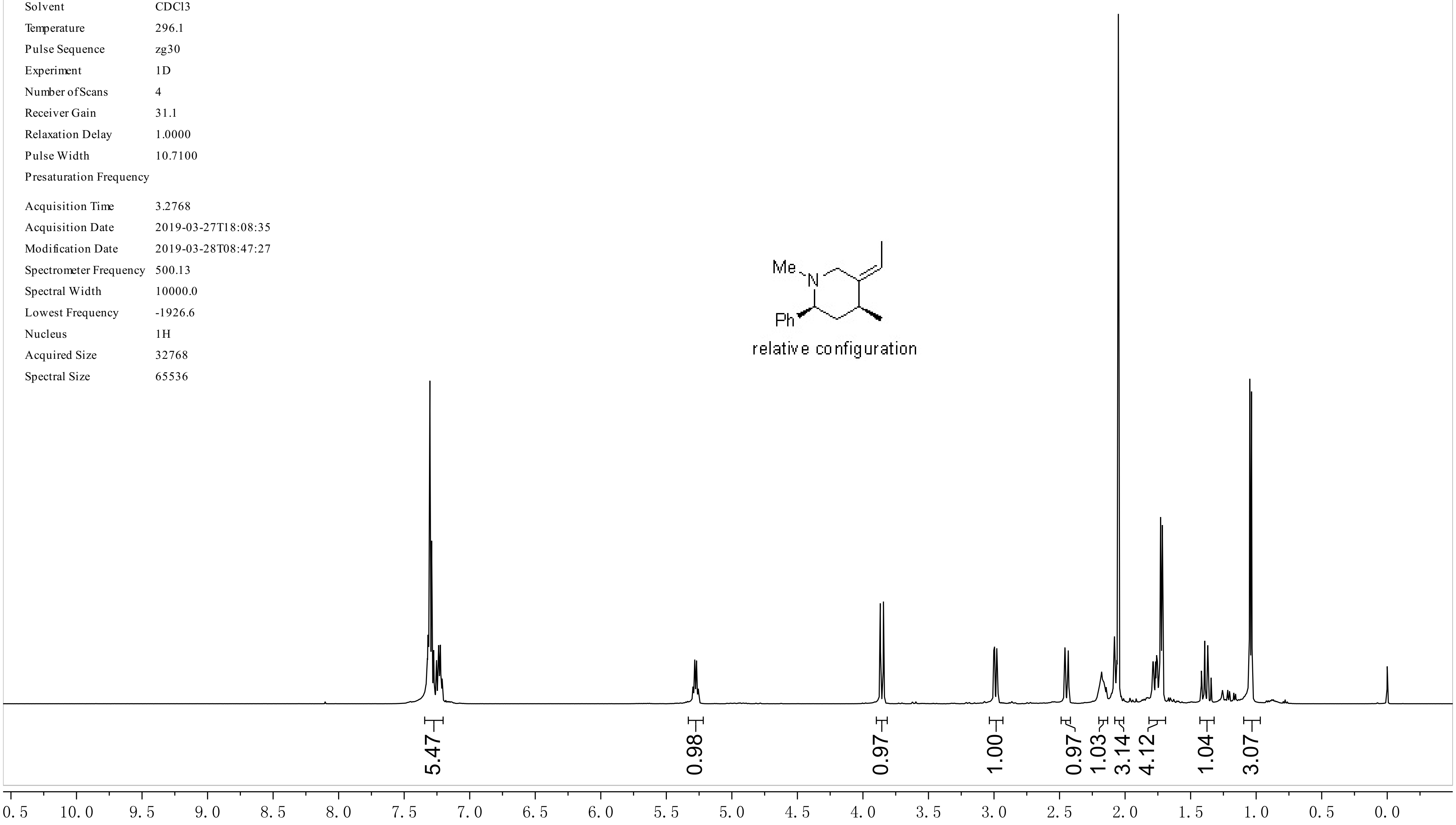

Parameter	Value
Title	xfy-190327-1-s.2.fid
Comment	
Origin	Bruker BioSpin GmbH
Owner	nmr
Site	
Instrument	spect
Solvent	CDCl3
Temperature	296.1
Pulse Sequence	zgpg30
Experiment	1D
Number of Scans	6
Receiver Gain	193.1
Relaxation Delay	2.0000
Pulse Width	9.6000
Presaturation Frequency	
Acquisition Time	1.1010
Acquisition Date	2019-03-27T18:10:07
Modification Date	2019-03-28T08:47:27
Spectrometer Frequency	125.77
Spectral Width	29761.9
Lowest Frequency	-2305.8
Nucleus	13C
Acquired Size	32768
Spectral Size	65536

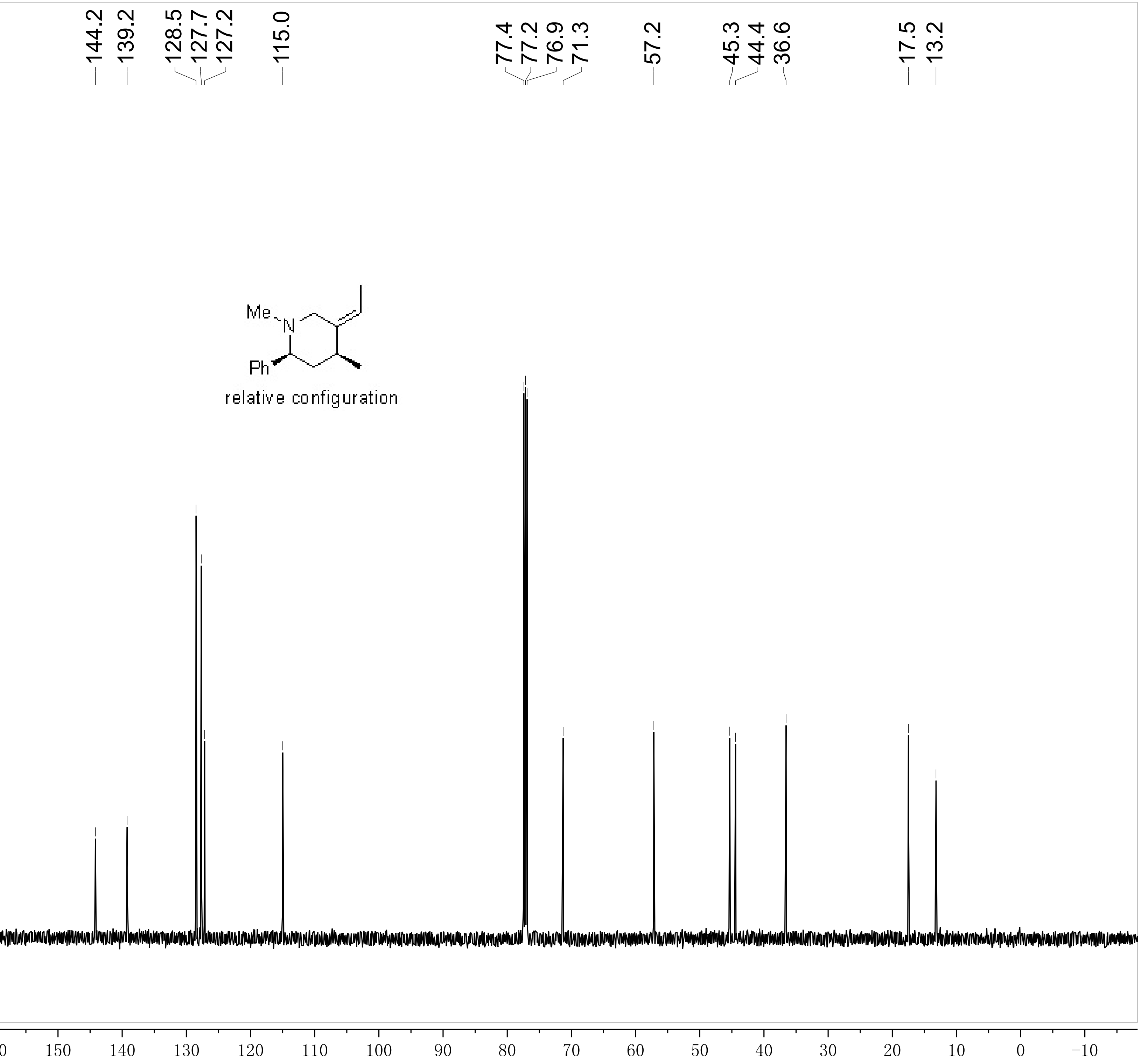

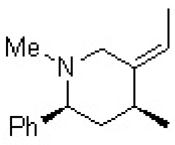

relative configuration

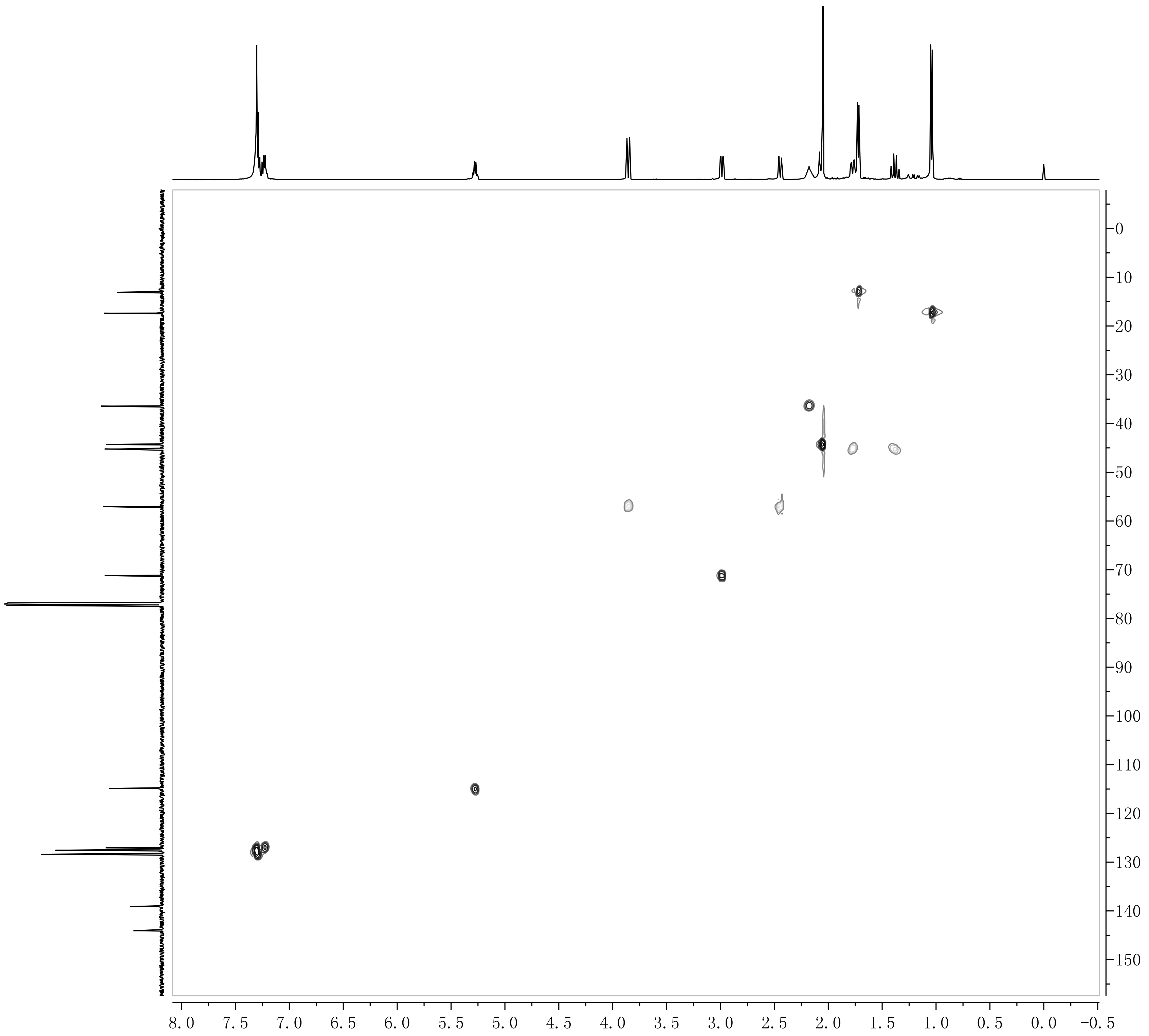

Parameter	Value
Title	xfy-190327-1-s.3.ser
Comment	
Origin	Bruker BioSpin GmbH
Owner	nmr
Site	
Instrument	spect
Solvent	CDCl3
Temperature	296.1
Pulse Sequence	hsqcedetgp
Experiment	HSQC-EDITED
Number of Scans	2
Receiver Gain	193.1
Relaxation Delay	1.4601
Pulse Width	10.7100
Presaturation Frequency	
Acquisition Time	0.1188
Acquisition Date	2019-03-27T18:11:56
Modification Date	2019-03-28T08:47:28
Spectrometer Frequency	(500.13, 125.77)
Spectral Width	(4310.3, 20833.3)
Lowest Frequency	(-266.7, -1037.0)
Nucleus	(1H, 13C)
Acquired Size	(512, 239)
Spectral Size	(512, 512)

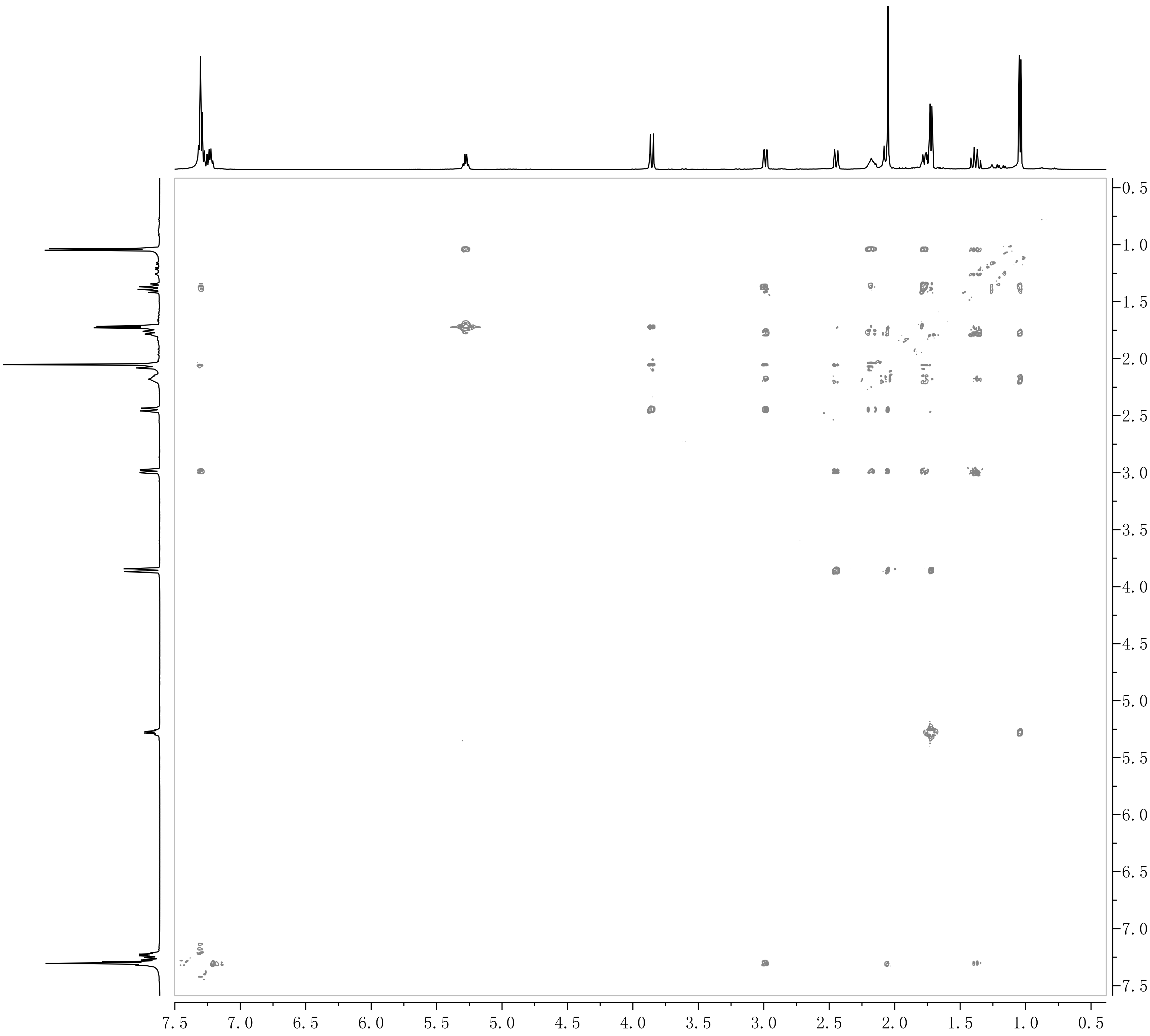

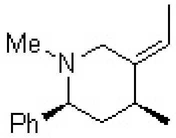

relative configuration

Parameter	Value
Title	xfy-190327-1-s.6.ser
Comment	
Origin	Bruker BioSpin GmbH
Owner	nmr
Site	
Instrument	spect
Solvent	CDCl3
Temperature	296.2
Pulse Sequence	noesygpphpp
Experiment	NOESY
Number of Scans	4
Receiver Gain	17.9
Relaxation Delay	1.9529
Pulse Width	10.7100
Presaturation Frequency	
Acquisition Time	0.2519
Acquisition Date	2019-03-28T07:42:00
Modification Date	2019-03-28T08:47:31
Spectrometer Frequency	(500.13, 500.13)
Spectral Width	(4065.0, 4065.0)
Lowest Frequency	(-51.0, -51.0)
Nucleus	(1H, 1H)
Acquired Size	(1024, 256)
Spectral Size	(1024, 1024)

Parameter	Value
Origin	Bruker BioSpin GmbH
Spectrometer	spect
Solvent	CDCl3
Temperature	292.4
Pulse Sequence	zg30
Experiment	1D
Number of Scans	8
Receiver Gain	35
Relaxation Delay	1.0000
Pulse Width	8.7300
Acquisition Time	1.9999
Spectrometer Frequency	400.13
Spectral Width	8012.8
Lowest Frequency	-1554.7
Nucleus	1H
Acquired Size	16025
Spectral Size	65536

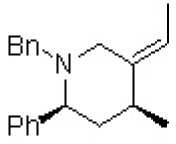

reletive configuration only

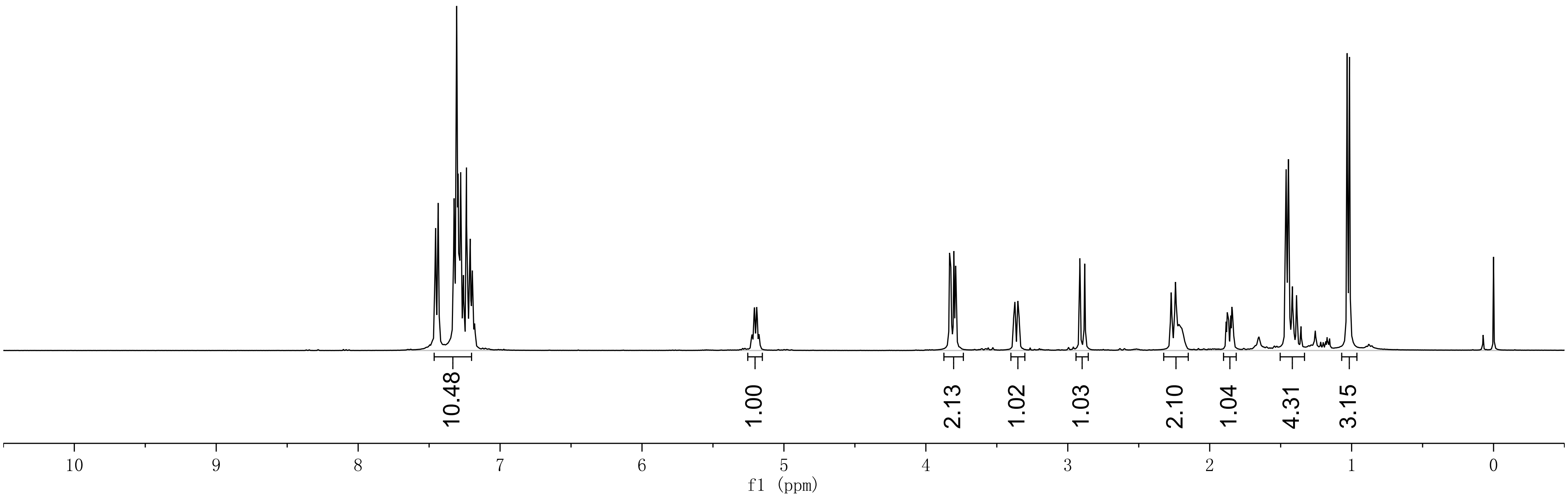

Parameter	Value
Origin	Bruker BioSpin GmbH
Spectrometer	spect
Solvent	CDCl3
Temperature	292.9
Pulse Sequence	zgpg30
Experiment	1D
Number of Scans	26
Receiver Gain	196
Relaxation Delay	2.0000
Pulse Width	10.0000
Acquisition Time	1.3631
Spectrometer Frequency	100.62
Spectral Width	24038.5
Lowest Frequency	-1938.2
Nucleus	13C
Acquired Size	32768
Spectral Size	65536

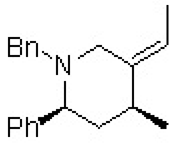

reletive configuration only

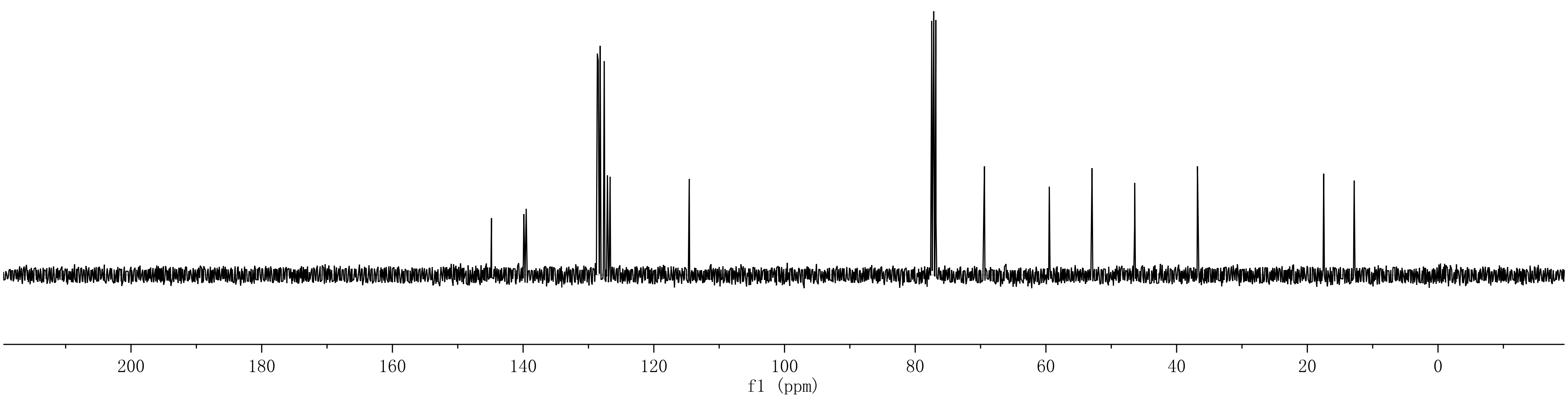

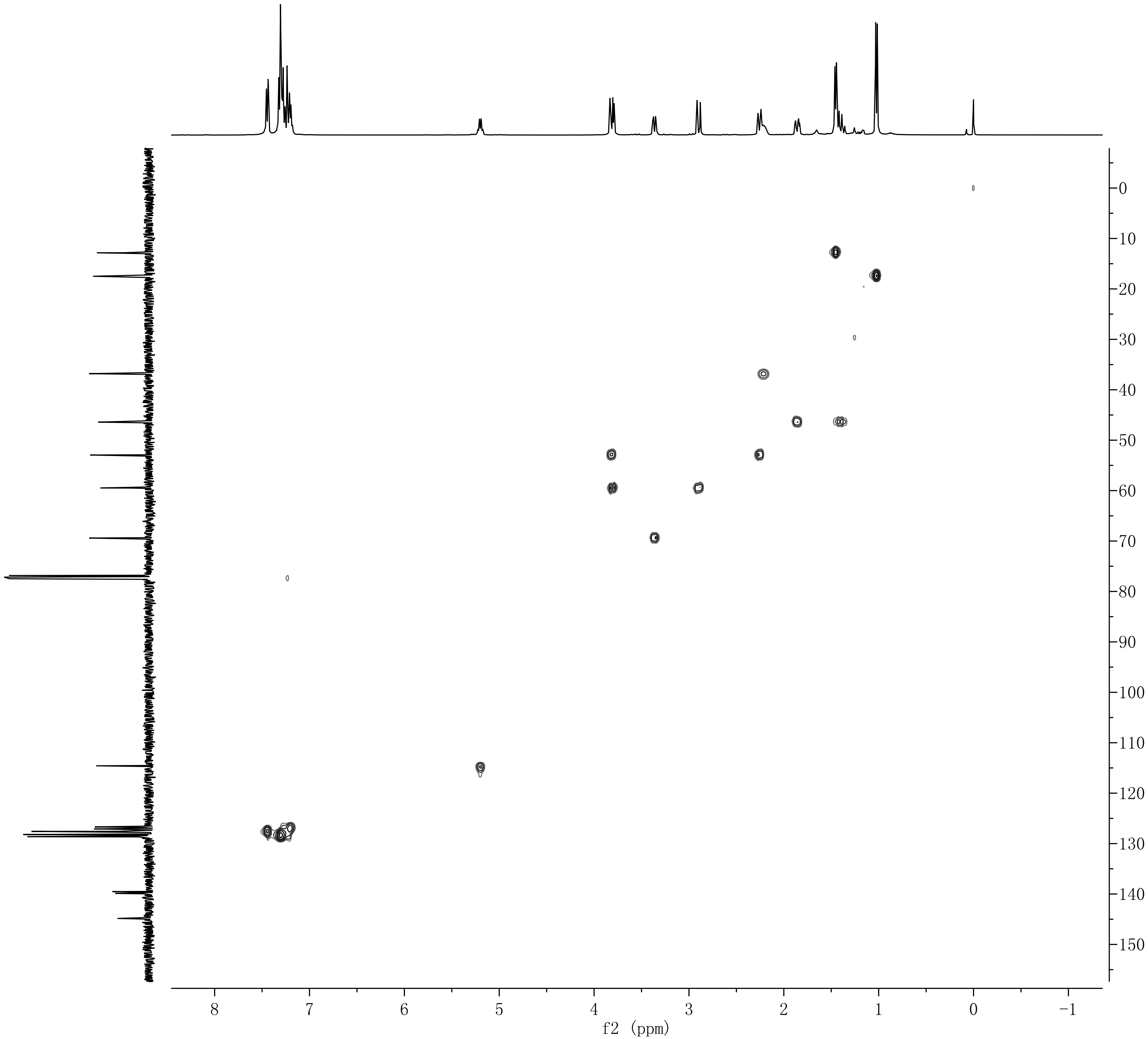

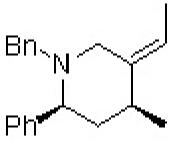

reletive configuration only

Parameter	Value
Origin	Bruker BioSpin GmbH
Spectrometer	spect
Solvent	CDCl3
Temperature	292.7
Pulse Sequence	hsqcetgp
Experiment	HSQC
Number of Scans	2
Receiver Gain	196
Relaxation Delay	1.4685
Pulse Width	8.7300
Acquisition Time	0.1300
Spectrometer Frequency	(400.13, 100.62)
Spectral Width	(3937.0, 16666.7)
Lowest Frequency	(-552.4, -829.1)
Nucleus	(1H, 13C)
Acquired Size	(512, 252)
Spectral Size	(512, 512)

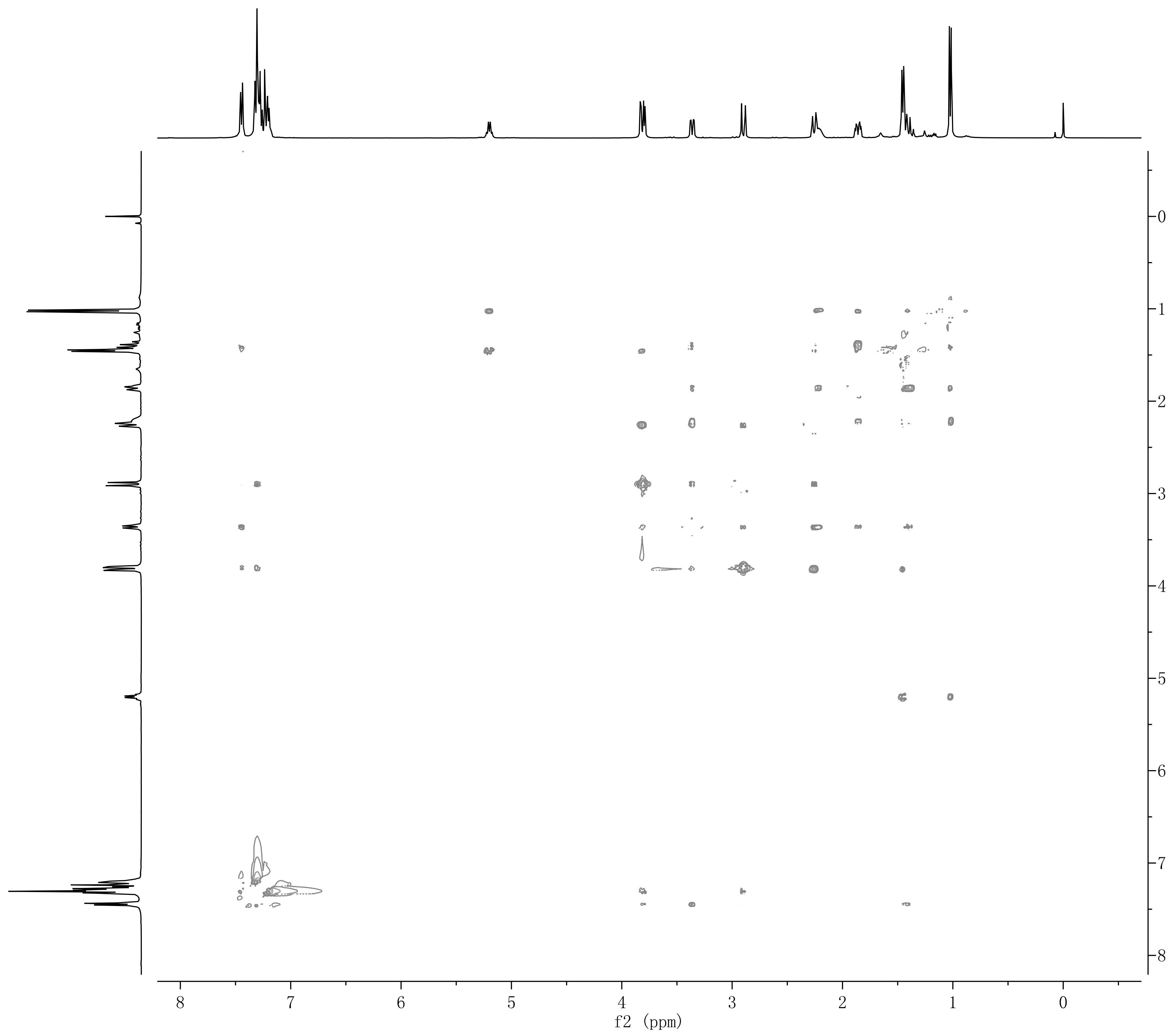

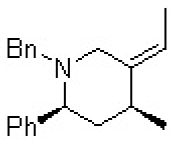

Parameter	Value
Origin	Bruker BioSpin GmbH
Spectrometer	spect
Solvent	CDCl3
Temperature	296.1
Pulse Sequence	noesygpphpp
Experiment	NOESY
Number of Scans	4
Receiver Gain	19
Relaxation Delay	1.9754
Pulse Width	10.7100
Acquisition Time	0.2294
Spectrometer Frequency	(500.13, 500.13)
Spectral Width	(4464.3, 4464.3)
Lowest Frequency	(-358.7, -358.7)
Nucleus	(1H, 1H)
Acquired Size	(1024, 256)

Parameter	Value
Title	xfy-190826-2-s1.50.1.1r
Comment	
Origin	Bruker BioSpin GmbH
Owner	nmr
Site	
Instrument	spect
Solvent	CDCl3
Temperature	296.1
Pulse Sequence	zg30
Experiment	1D
Number of Scans	8
Receiver Gain	95.3
Relaxation Delay	1.0000
Pulse Width	10.7100
Presaturation Frequency	
Acquisition Time	3.2768
Acquisition Date	2019-08-27T11:58:10
Modification Date	2019-08-27T12:34:41
Spectrometer Frequency	500.13
Spectral Width	10000.0
Lowest Frequency	-1923.5
Nucleus	1H
Acquired Size	32768
Spectral Size	65536

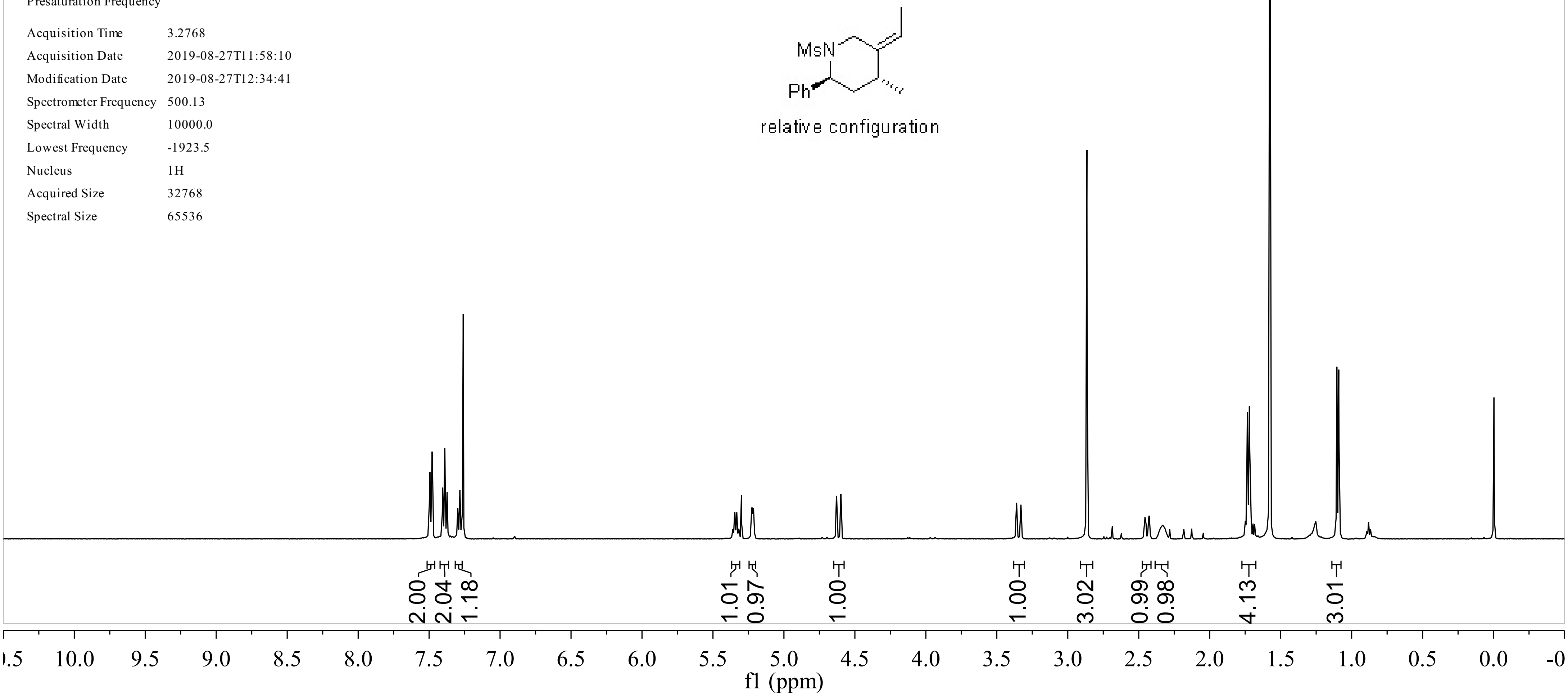

Parameter	Value
Title	xfy-190826-2-s1.12.1.1r
Comment	
Origin	Bruker BioSpin GmbH
Owner	nmr
Site	
Instrument	spect
Solvent	CDCl3
Temperature	296.5
Pulse Sequence	zgpg30
Experiment	1D
Number of Scans	80
Receiver Gain	196.4
Relaxation Delay	2.0000
Pulse Width	10.0000
Presaturation Frequency	
Acquisition Time	1.3631
Acquisition Date	2019-08-26T23:25:49
Modification Date	2019-08-27T09:19:30
Spectrometer Frequency	100.61
Spectral Width	24038.5
Lowest Frequency	-1946.6
Nucleus	13C
Acquired Size	32768
Spectral Size	32768

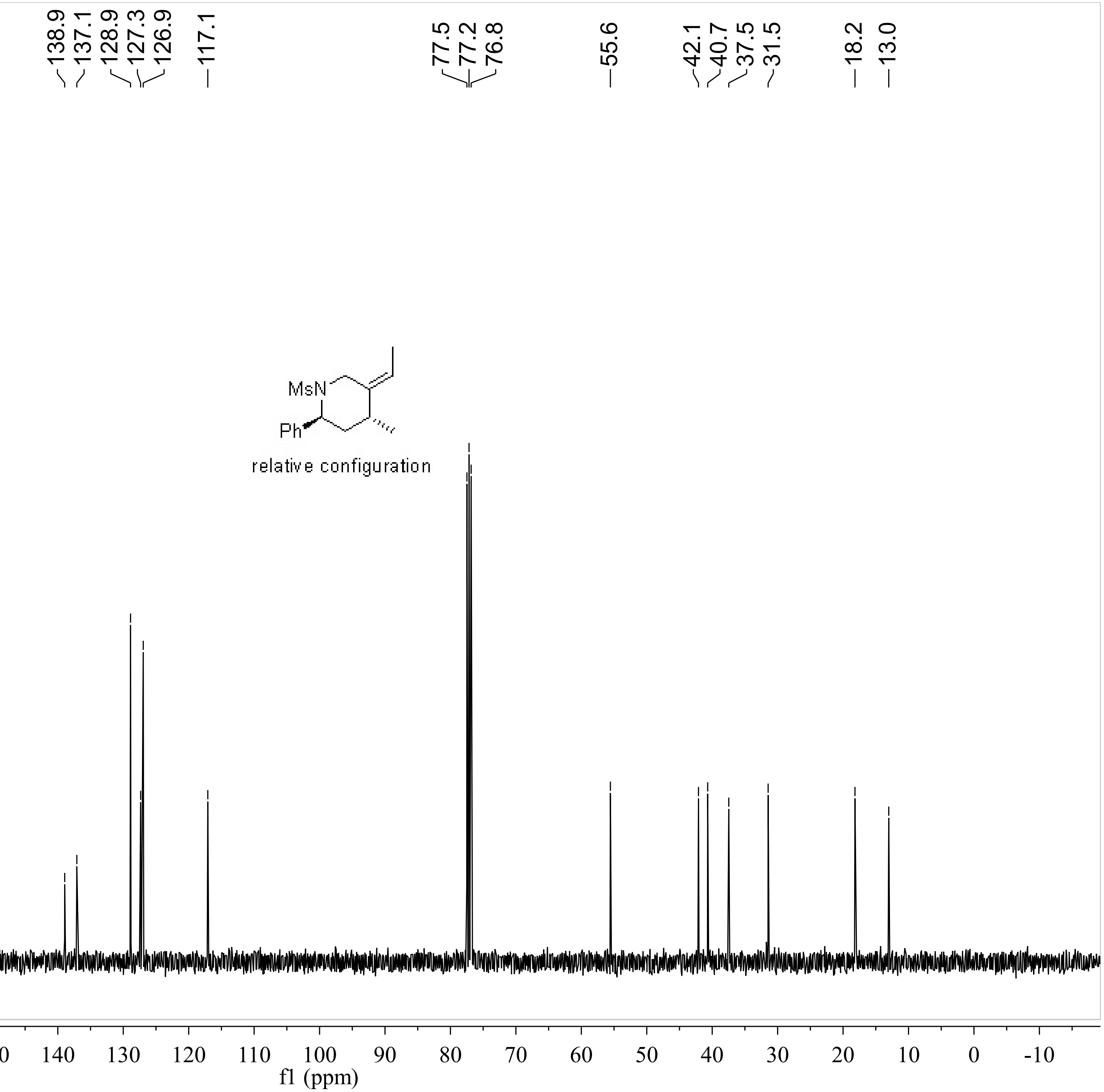

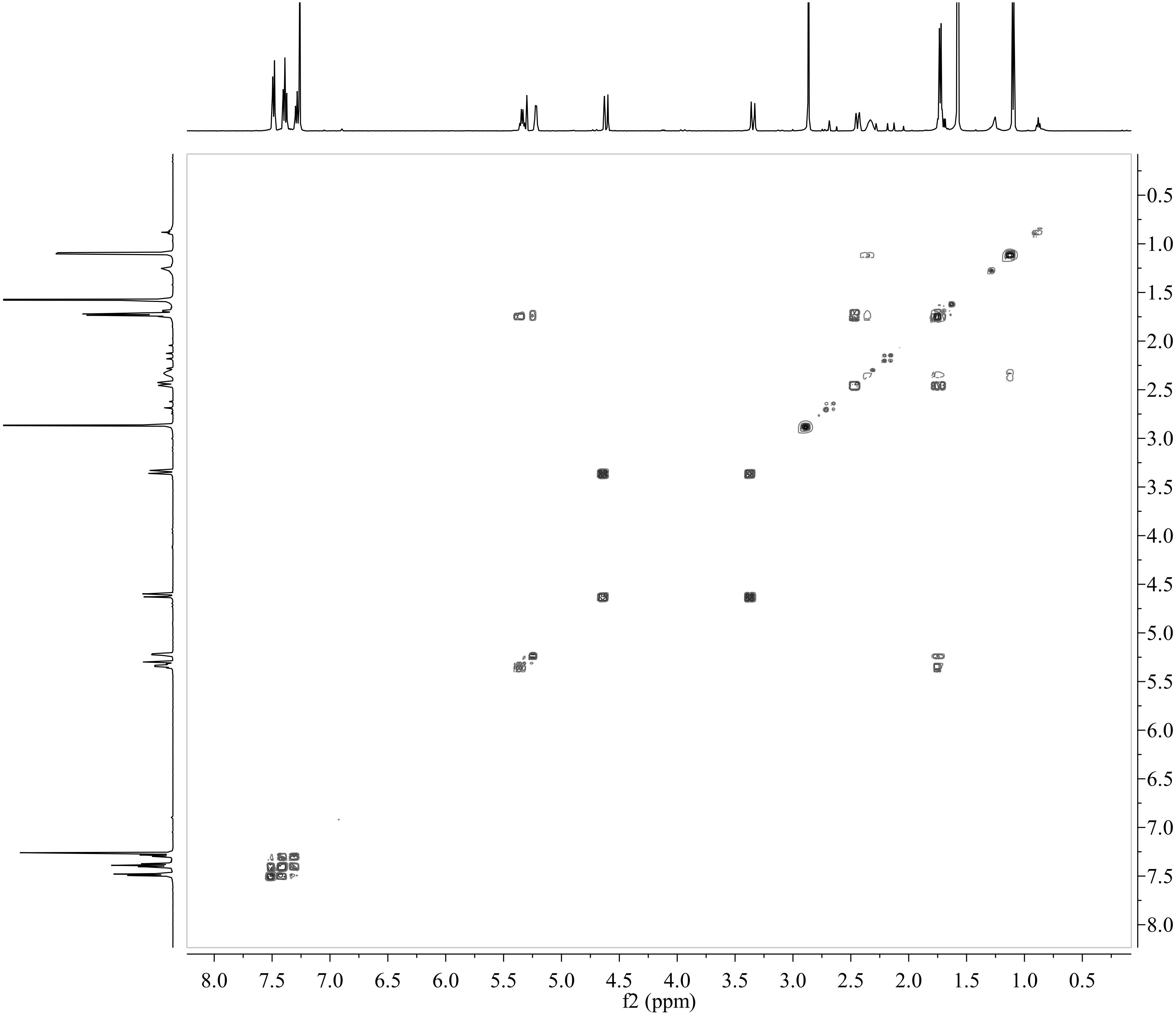

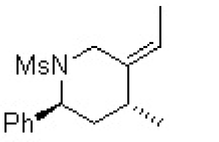

relative configuration

Parameter	Value
Title	xfy-190826-2-s1.14.ser
Comment	
Origin	Bruker BioSpin GmbH
Owner	nmr
Site	
Instrument	spect
Solvent	CDCl3
Temperature	296.5
Pulse Sequence	cosygpppqf
Experiment	COSY
Number of Scans	4
Receiver Gain	49.4
Relaxation Delay	1.8783
Pulse Width	8.7300
Presaturation Frequency	
Acquisition Time	0.3133
Acquisition Date	2019-08-26T23:47:08
Modification Date	2019-08-27T09:19:30
Spectrometer Frequency	(400.13, 400.13)
Spectral Width	(3268.0, 3268.0)
Lowest Frequency	(27.5, 27.5)
Nucleus	(1H, 1H)
Acquired Size	(1024, 128)
Spectral Size	(1024, 1024)

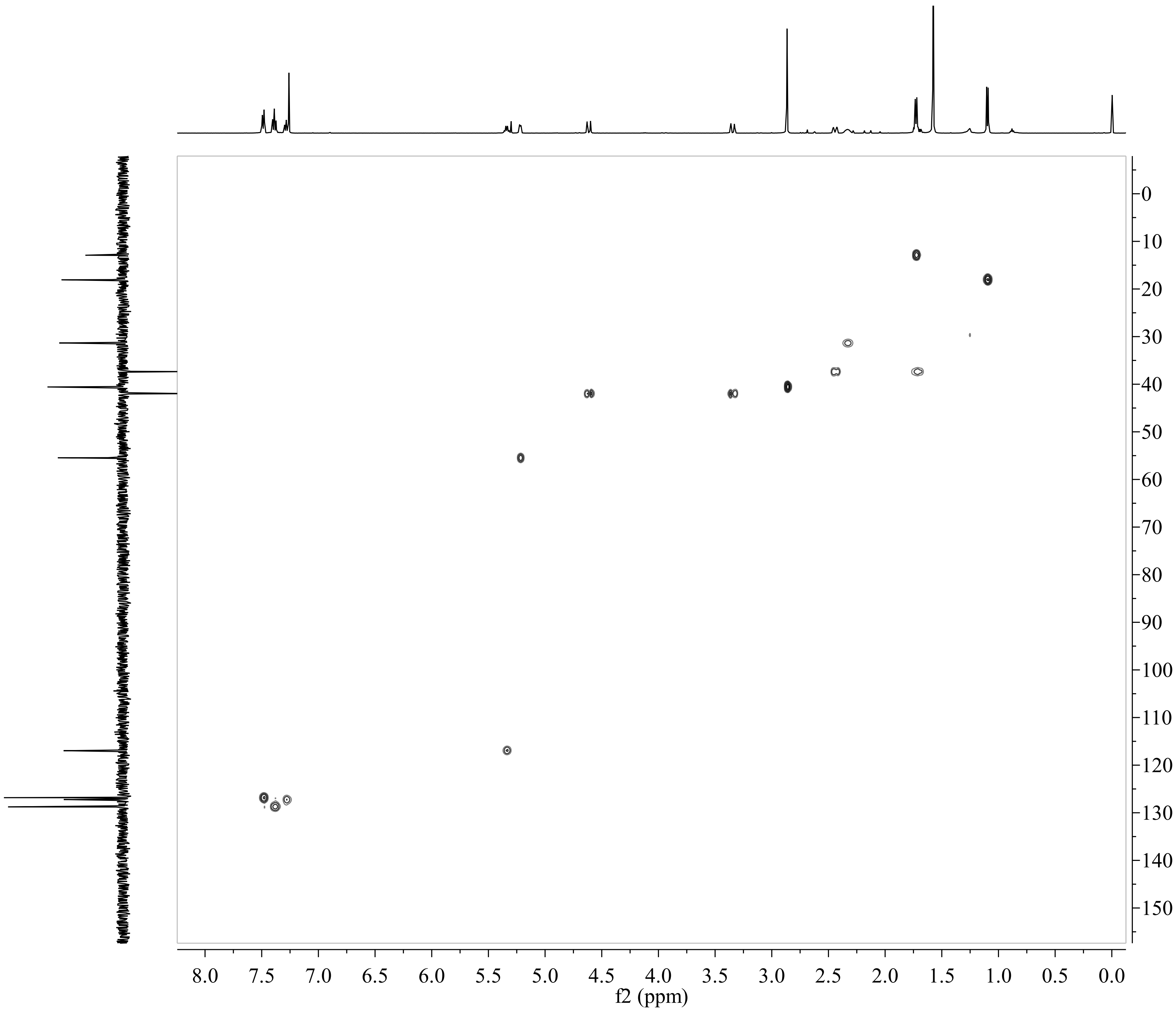

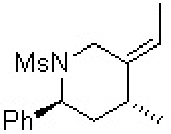

relative configuration

Parameter	Value
Title	xfy-190826-2-s1.15.ser
Comment	
Origin	Bruker BioSpin GmbH
Owner	nmr
Site	
Instrument	spect
Solvent	CDCl3
Temperature	296.6
Pulse Sequence	hsqcetgp
Experiment	HSQC
Number of Scans	6
Receiver Gain	196.4
Relaxation Delay	1.4459
Pulse Width	8.7300
Presaturation Frequency	
Acquisition Time	0.1526
Acquisition Date	2019-08-27T00:07:51
Modification Date	2019-08-27T09:19:30
Spectrometer Frequency	(400.13, 100.62)
Spectral Width	(3355.7, 16666.7)
Lowest Frequency	(-56.5, -829.1)
Nucleus	(1H, 13C)
Acquired Size	(512, 256)
Spectral Size	(512, 512)

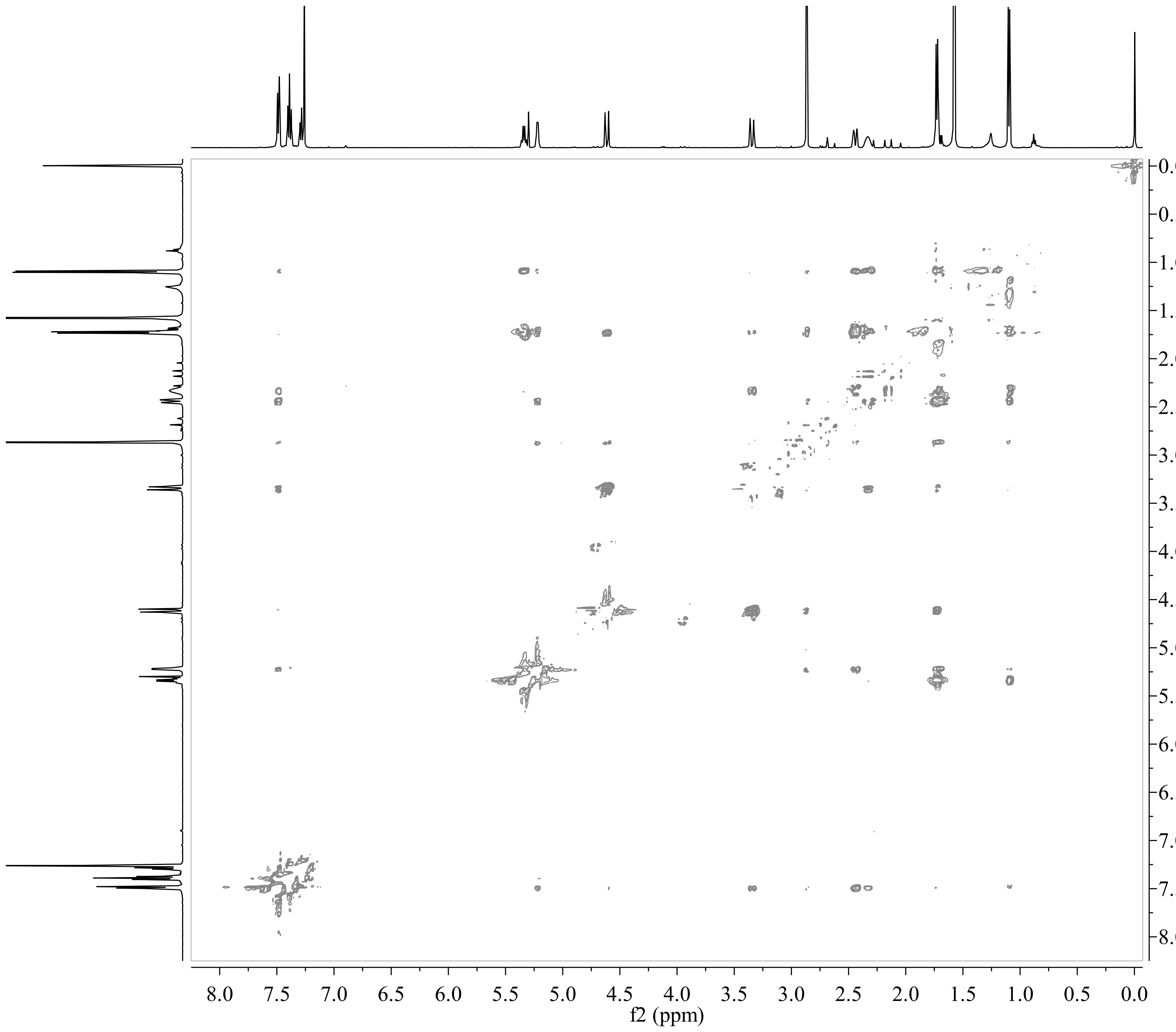

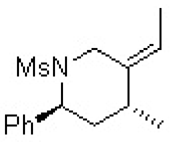

relative configuration

Parameter	Value
Title	xfy-190826-2-s1.16.ser
Comment	
Origin	Bruker BioSpin GmbH
Owner	nmr
Site	
Instrument	spect
Solvent	CDCl3
Temperature	296.6
Pulse Sequence	noesygpphpp
Experiment	NOESY
Number of Scans	12
Receiver Gain	49.4
Relaxation Delay	1.9488
Pulse Width	8.7300
Presaturation Frequency	
Acquisition Time	0.3072
Acquisition Date	2019-08-27T00:51:47
Modification Date	2019-08-27T09:19:30
Spectrometer Frequency	(400.13, 400.13)
Spectral Width	(3333.3, 3333.3)
Lowest Frequency	(-32.3, -32.3)
Nucleus	(1H, 1H)
Acquired Size	(1024, 256)
Spectral Size	(1024, 1024)

Parameter	Value
Title	xfy-190308-1-s.1.fid
Comment	
Origin	Bruker BioSpin GmbH
Owner	nmr
Site	
Instrument	spect
Solvent	CDCl3
Temperature	296.1
Pulse Sequence	zg30
Experiment	1D
Number of Scans	4
Receiver Gain	31.1
Relaxation Delay	1.0000
Pulse Width	10.7100
Presaturation Frequency	
Acquisition Time	3.2768
Acquisition Date	2019-03-09T10:30:35
Modification Date	2019-03-11T10:13:02
Spectrometer Frequency	500.13
Spectral Width	10000.0
Lowest Frequency	-1911.5
Nucleus	1H
Acquired Size	32768
Spectral Size	65536

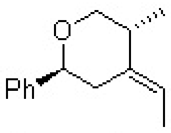

relative configuration

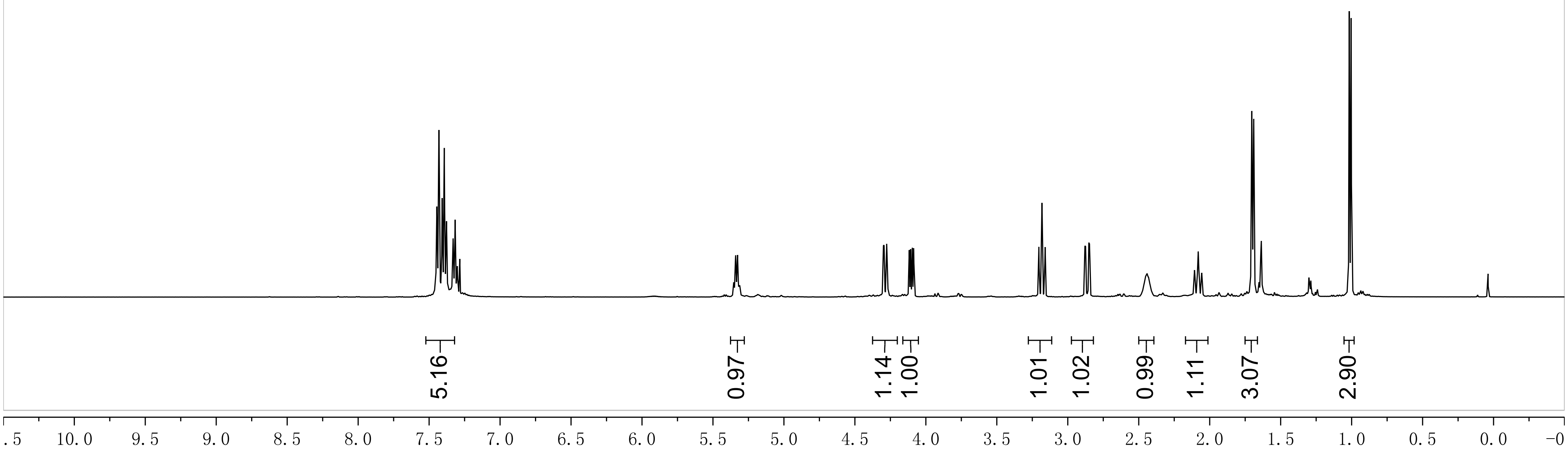

Parameter	Value
Title	xfy-190308-1-s.18.fid
Comment	
Origin	Bruker BioSpin GmbH
Owner	nmr
Site	
Instrument	spect
Solvent	CDCl3
Temperature	296.2
Pulse Sequence	zgpg30
Experiment	1D
Number of Scans	1024
Receiver Gain	193.1
Relaxation Delay	2.0000
Pulse Width	9.6000
Presaturation Frequency	
Acquisition Time	1.1010
Acquisition Date	2019-03-11T06:54:56
Modification Date	2019-03-11T10:13:04
Spectrometer Frequency	125.77
Spectral Width	29761.9
Lowest Frequency	-2289.3
Nucleus	13C
Acquired Size	32768
Spectral Size	65536

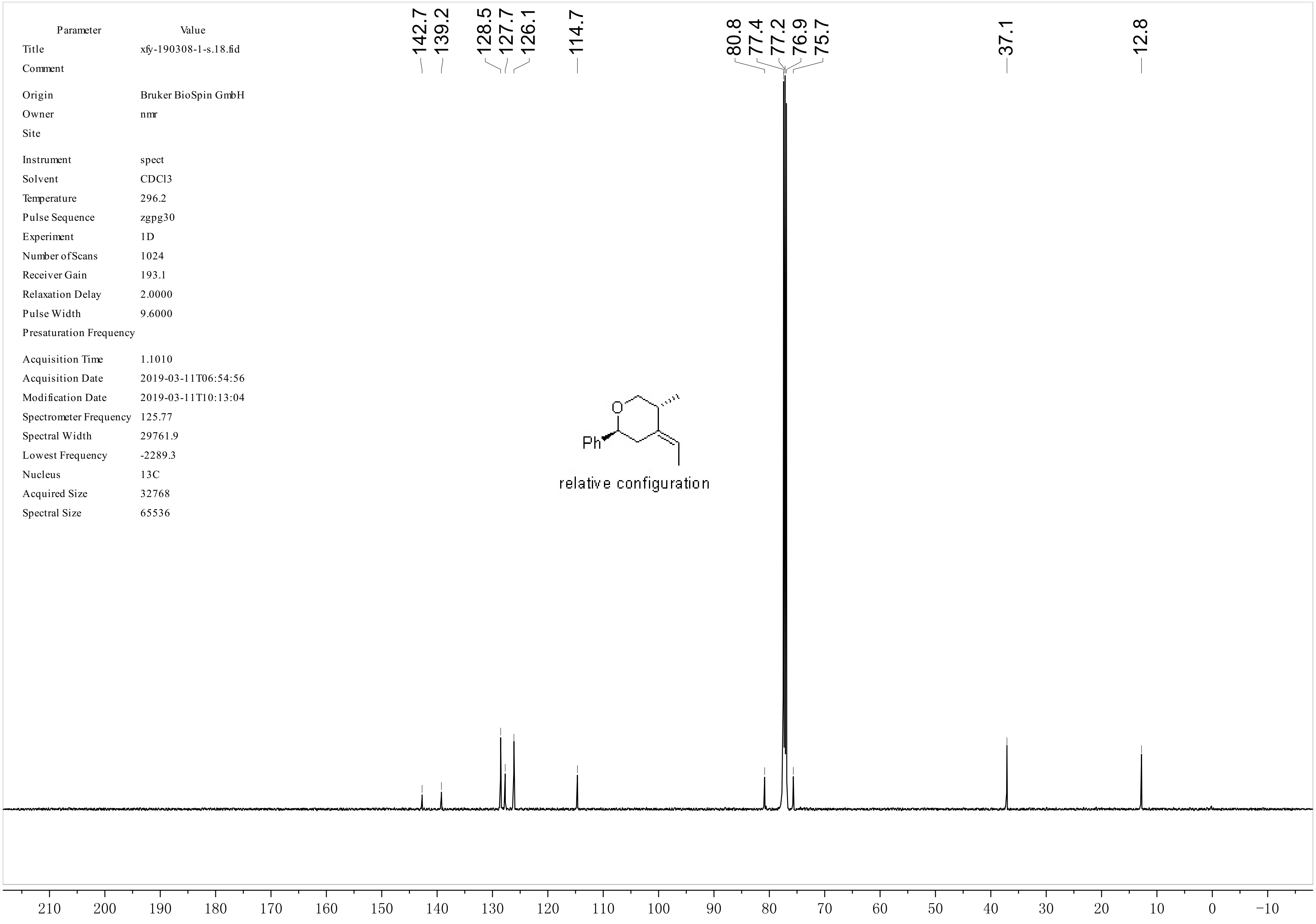

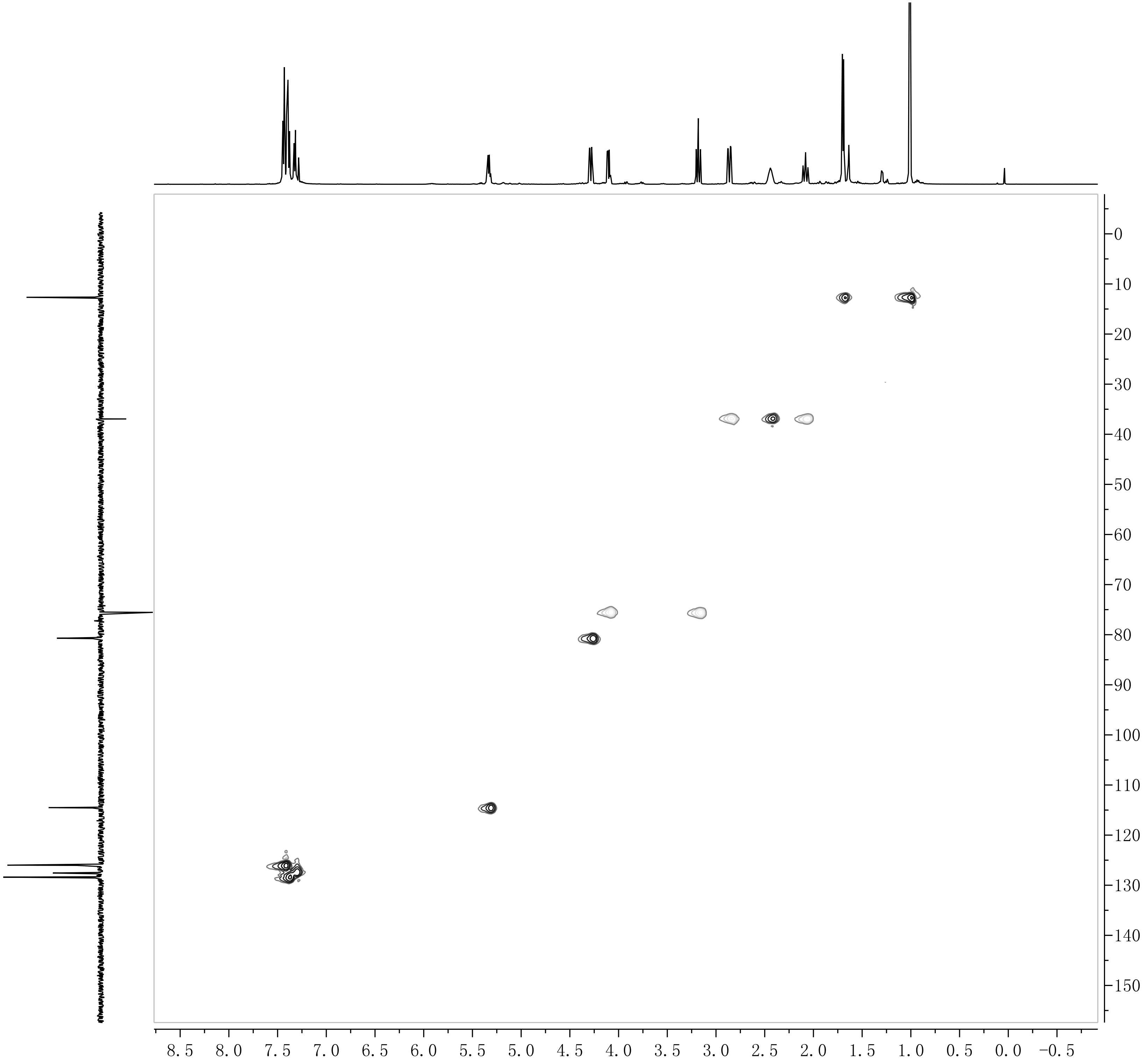

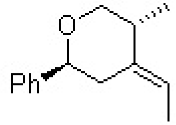

relative configuration

Parameter	Value
Title	xfy-190308-1-s.15.ser
Comment	
Origin	Bruker BioSpin GmbH
Owner	nmr
Site	
Instrument	spect
Solvent	CDCl3
Temperature	296.2
Pulse Sequence	hsqcedetgp
Experiment	HSQC-EDITED
Number of Scans	2
Receiver Gain	193.1
Relaxation Delay	1.4734
Pulse Width	10.7100
Presaturation Frequency	
Acquisition Time	0.1055
Acquisition Date	2019-03-11T04:14:03
Modification Date	2019-03-11T10:13:04
Spectrometer Frequency	(500.13, 125.77)
Spectral Width	(4854.4, 20833.3)
Lowest Frequency	(-469.1, -1037.0)
Nucleus	(1H, 13C)
Acquired Size	(512, 256)
Spectral Size	(512, 512)

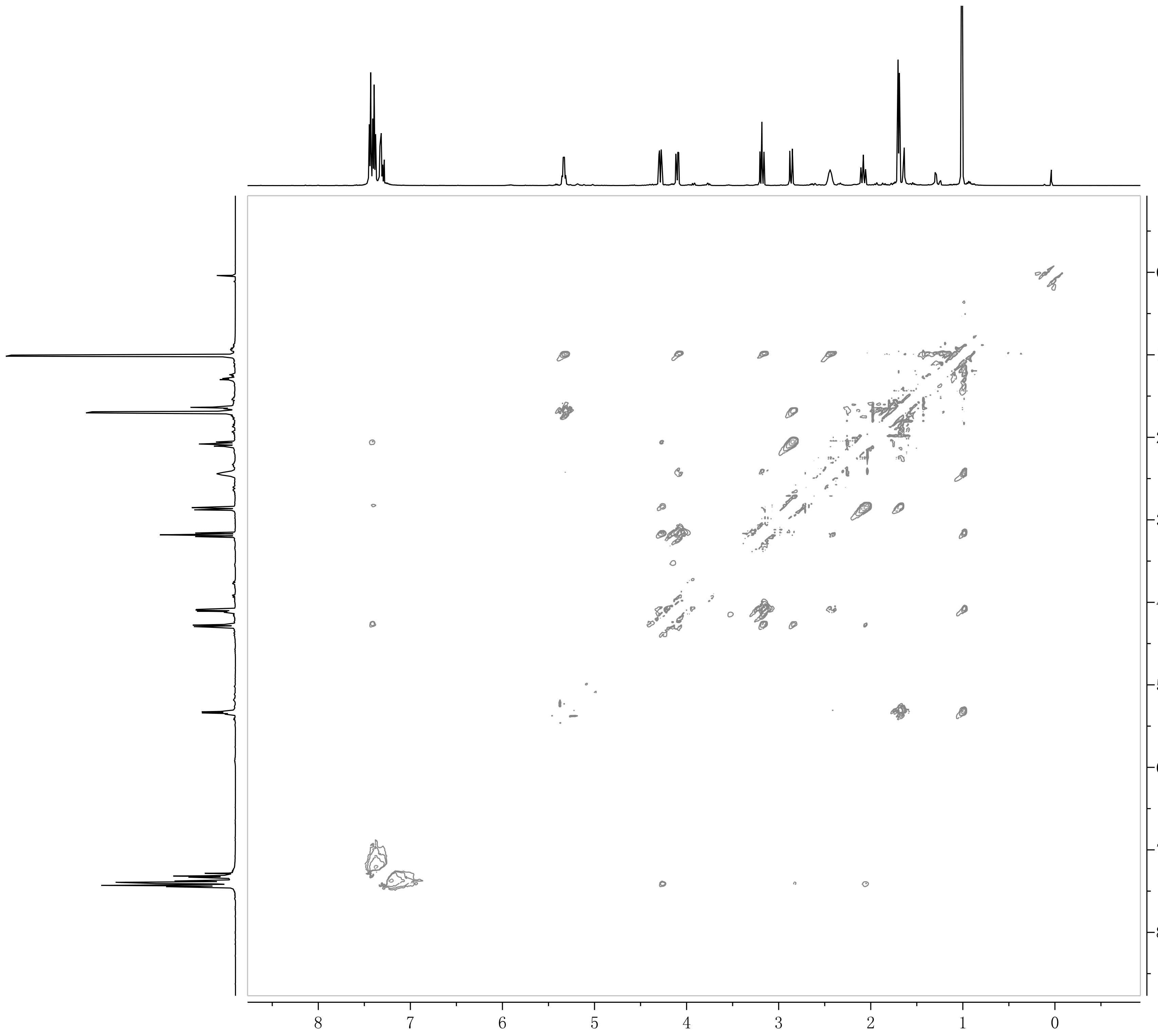

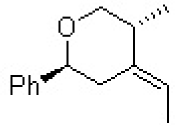

relative configuration

Parameter	Value
Title	xfy-190308-1-s.16.ser
Comment	
Origin	Bruker BioSpin GmbH
Owner	nmr
Site	
Instrument	spect
Solvent	CDCl3
Temperature	296.1
Pulse Sequence	noesygpphpp
Experiment	NOESY
Number of Scans	8
Receiver Gain	54.3
Relaxation Delay	1.9939
Pulse Width	10.7100
Presaturation Frequency	
Acquisition Time	0.2109
Acquisition Date	2019-03-11T04:30:17
Modification Date	2019-03-11T10:13:04
Spectrometer Frequency	(500.13, 500.13)
Spectral Width	(4854.4, 4854.4)
Lowest Frequency	(-469.1, -469.1)
Nucleus	(1H, 1H)
Acquired Size	(1024, 256)
Spectral Size	(1024, 1024)

Parameter	Value
Title	xfy-190316-1-s.44.fid
Comment	
Origin	Bruker BioSpin GmbH
Owner	nmr
Site	
Instrument	spect
Solvent	CDCl3
Temperature	296.2
Pulse Sequence	zg30
Experiment	1D
Number of Scans	16
Receiver Gain	31.1
Relaxation Delay	1.0000
Pulse Width	10.7100
Presaturation Frequency	
Acquisition Time	3.2768
Acquisition Date	2019-03-17T11:54:04
Modification Date	2019-03-20T09:26:21
Spectrometer Frequency	500.13
Spectral Width	10000.0
Lowest Frequency	-1921.1
Nucleus	1H
Acquired Size	32768
Spectral Size	65536

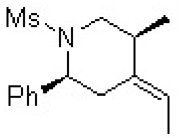

relative configuration

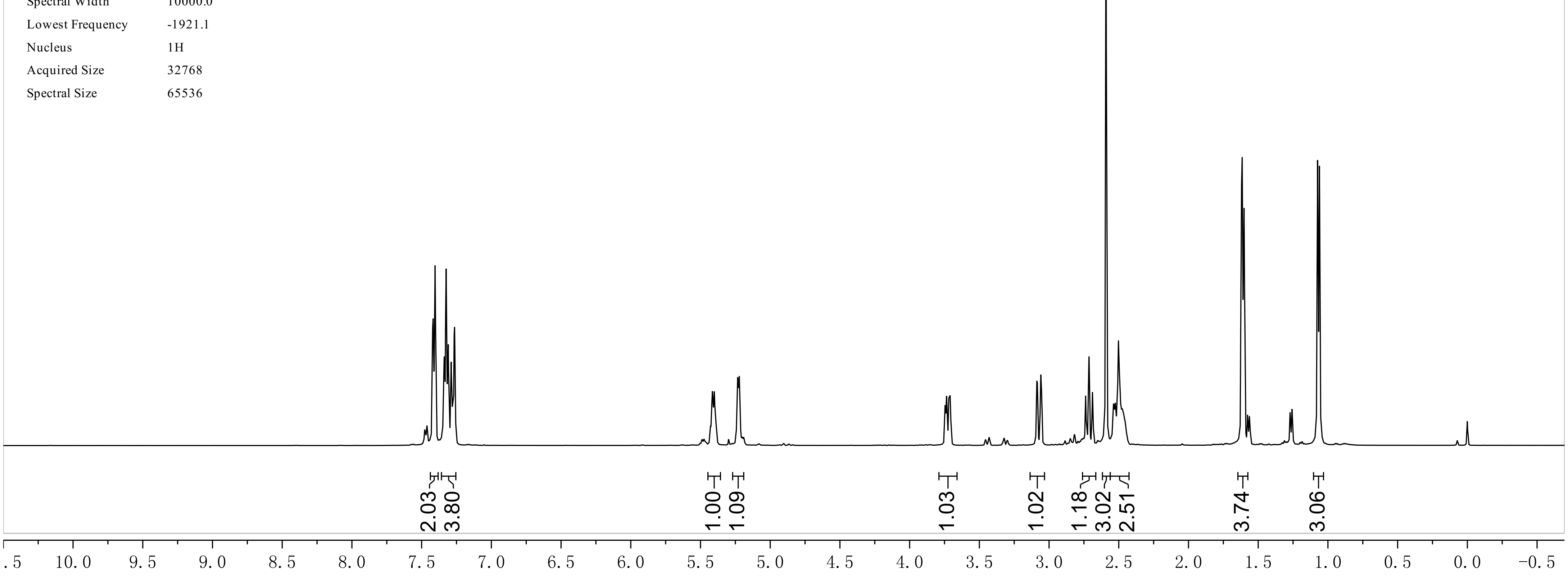

Parameter	Value
Title	xfy-190316-1-s.45.fid
Comment	
Origin	Bruker BioSpin GmbH
Owner	nmr
Site	
Instrument	spect
Solvent	CDCl3
Temperature	296.2
Pulse Sequence	zgpg30
Experiment	1D
Number of Scans	24
Receiver Gain	193.1
Relaxation Delay	2.0000
Pulse Width	9.6000
Presaturation Frequency	
Acquisition Time	1.1010
Acquisition Date	2019-03-17T12:04:19
Modification Date	2019-03-20T09:26:21
Spectrometer Frequency	125.77
Spectral Width	29761.9
Lowest Frequency	-2292.4
Nucleus	13C
Acquired Size	32768
Spectral Size	65536

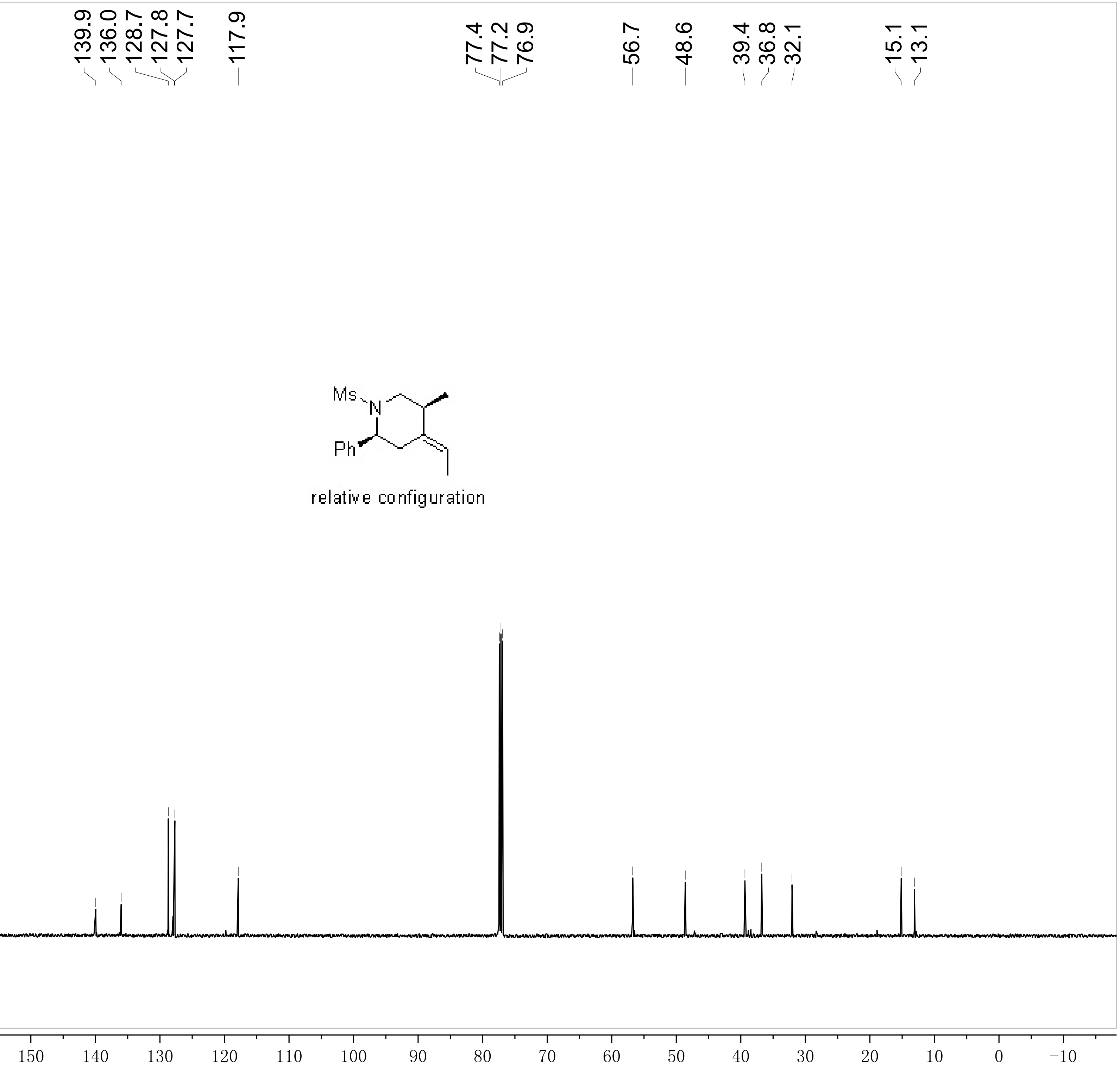

relative configuration

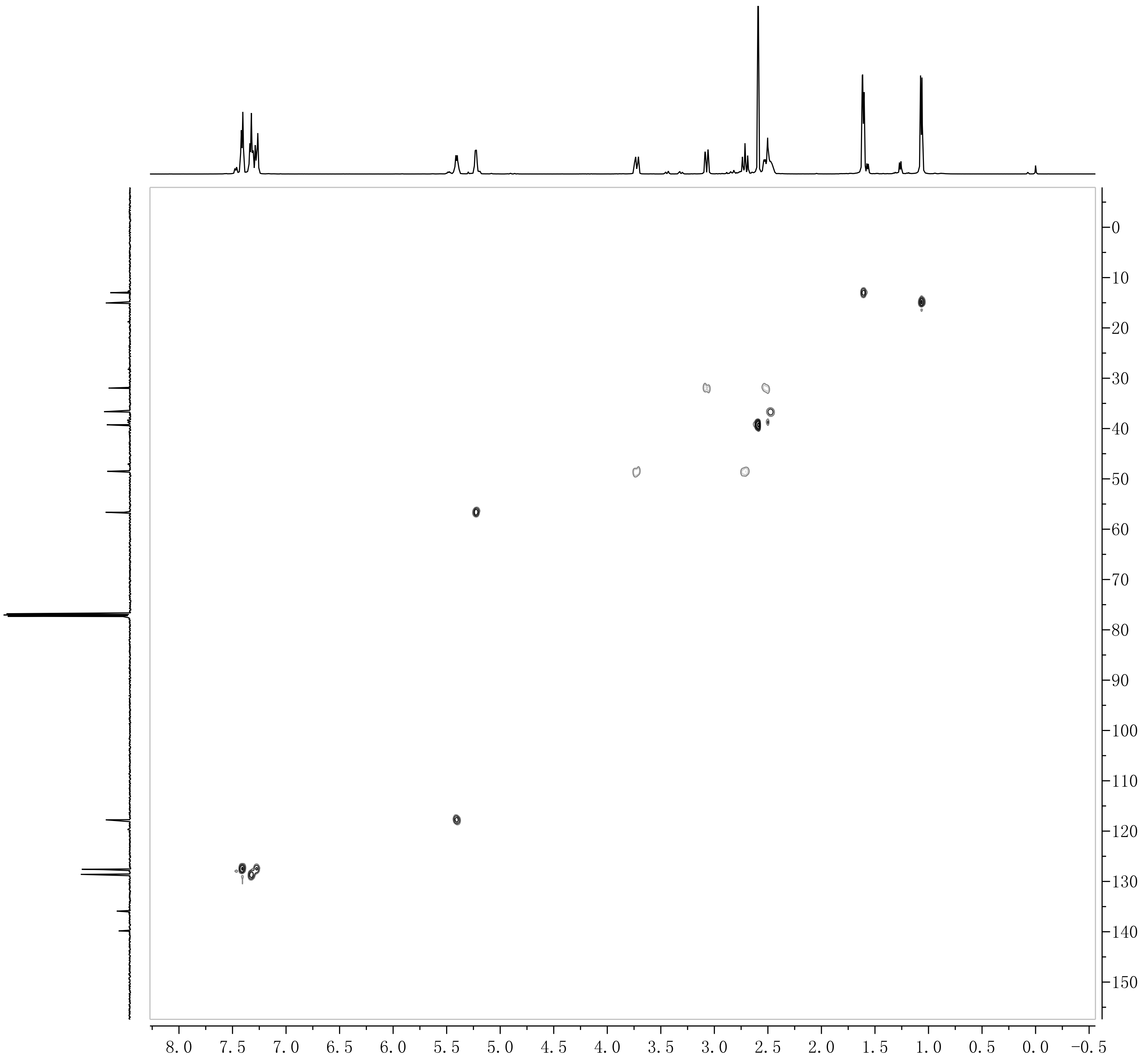

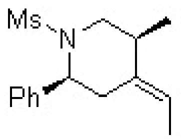

relative configuration

Parameter	Value
Title	xfy-190316-1-s.46.ser
Comment	
Origin	Bruker BioSpin GmbH
Owner	nmr
Site	
Instrument	spect
Solvent	CDCl3
Temperature	296.1
Pulse Sequence	hsqcedetgp
Experiment	HSQC-EDITED
Number of Scans	2
Receiver Gain	193.1
Relaxation Delay	1.4631
Pulse Width	10.7100
Presaturation Frequency	
Acquisition Time	0.1157
Class	
Spectrometer Frequency	(500.13, 125.77)
Spectral Width	(4424.8, 20833.3)
Lowest Frequency	(-288.2, -1037.0)
Nucleus	(1H, 13C)
Acquired Size	(512, 256)
Spectral Size	(512, 512)

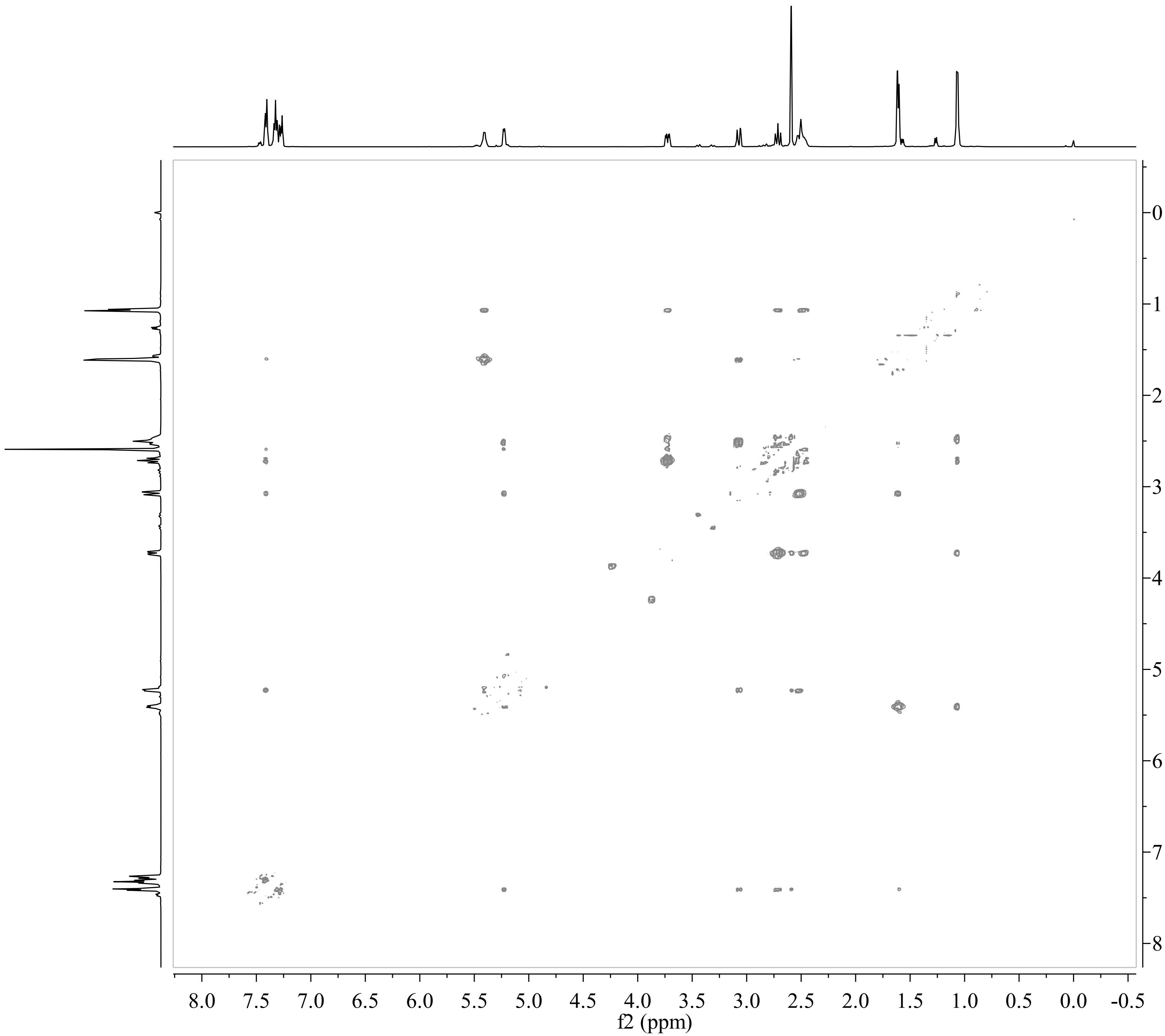

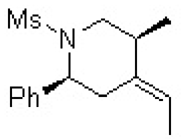

relative configuration

Parameter	Value
Title	xfy-190316-1-s.42.ser
Comment	
Origin	Bruker BioSpin GmbH
Owner	nmr
Site	
Instrument	spect
Solvent	CDCl3
Temperature	296.2
Pulse Sequence	noesygpphpp
Experiment	NOESY
Number of Scans	4
Receiver Gain	25.2
Relaxation Delay	1.9734
Pulse Width	10.7100
Presaturation Frequency	
Acquisition Time	0.2314
Acquisition Date	2019-09-24T17:49:34
Modification Date	2019-09-24T20:27:21
Spectrometer Frequency	(500.13, 500.13)
Spectral Width	(4424.8, 4424.8)
Lowest Frequency	(-292.1, -292.1)
Nucleus	(1H, 1H)
Acquired Size	(1024, 256)
Spectral Size	(1024, 1024)

Parameter	Value
Title	xfy-190310-3-s.11.fid
Comment	
Origin	Bruker BioSpin GmbH
Owner	nmr
Site	
Instrument	spect
Solvent	CDCl3
Temperature	296.2
Pulse Sequence	zg30
Experiment	1D
Number of Scans	8
Receiver Gain	62.9
Relaxation Delay	1.0000
Pulse Width	10.7100
Presaturation Frequency	
Acquisition Time	3.2768
Acquisition Date	2019-03-11T01:05:33
Modification Date	2019-03-11T09:47:44
Spectrometer Frequency	500.13
Spectral Width	10000.0
Lowest Frequency	-1922.1
Nucleus	1H
Acquired Size	32768
Spectral Size	65536

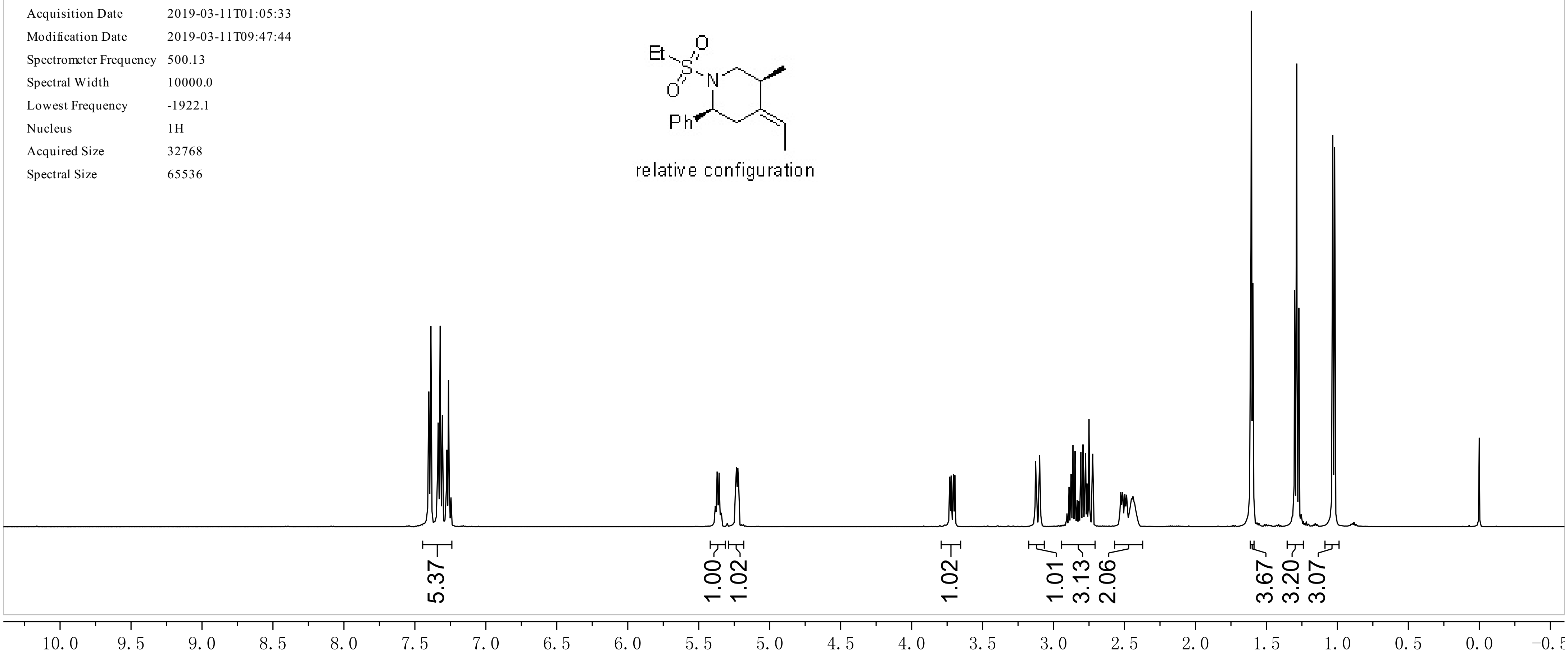

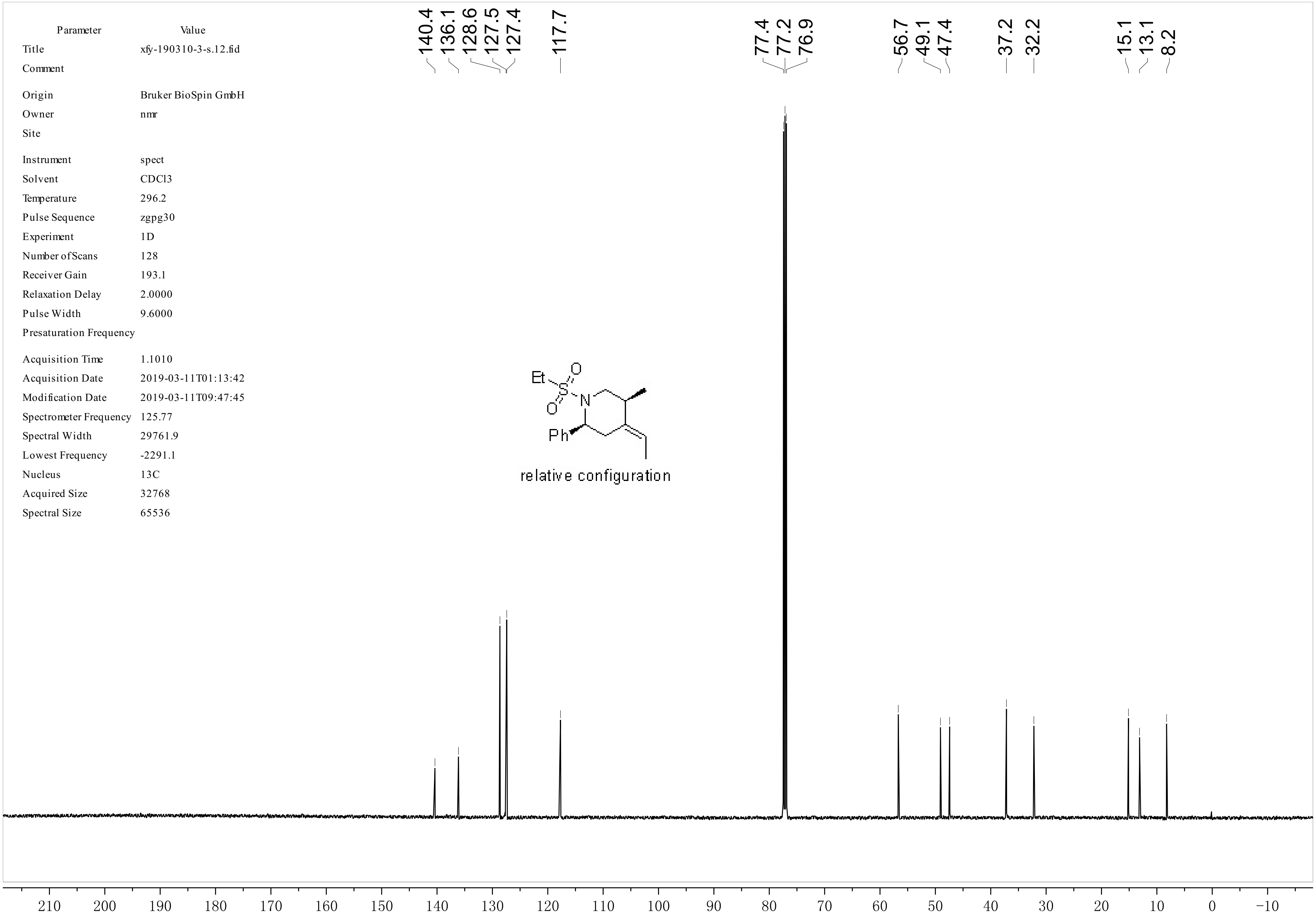
Parameter Value
Title xfy-190310-3-s.12.fid
Comment
Origin Bruker BioSpin GmbH
Owner nmr
Site
Instrument spect
Solvent CDCl3
Temperature 296.2
Pulse Sequence zgpg30
Experiment 1D
Number of Scans 128
Receiver Gain 193.1
Relaxation Delay 2.0000
Pulse Width 9.6000
Presaturation Frequency
Acquisition Time 1.1010
Acquisition Date 2019-03-11T01:13:42
Modification Date 2019-03-11T09:47:45
Spectrometer Frequency 125.77
Spectral Width 29761.9
Lowest Frequency -2291.1
Nucleus 13C
Acquired Size 32768
Spectral Size 65536
140.4
136.1
128.6
127.5
127.4
117.7
77.4
77.2
76.9
56.7
49.1
47.4
37.2
32.2
15.1
13.1
8.2
Et
S
O
O
N
Ph
relative configuration
210 200 190 180 170 160 150 140 130 120 110 100 90 80 70 60 50 40 30 20 10 0 −10

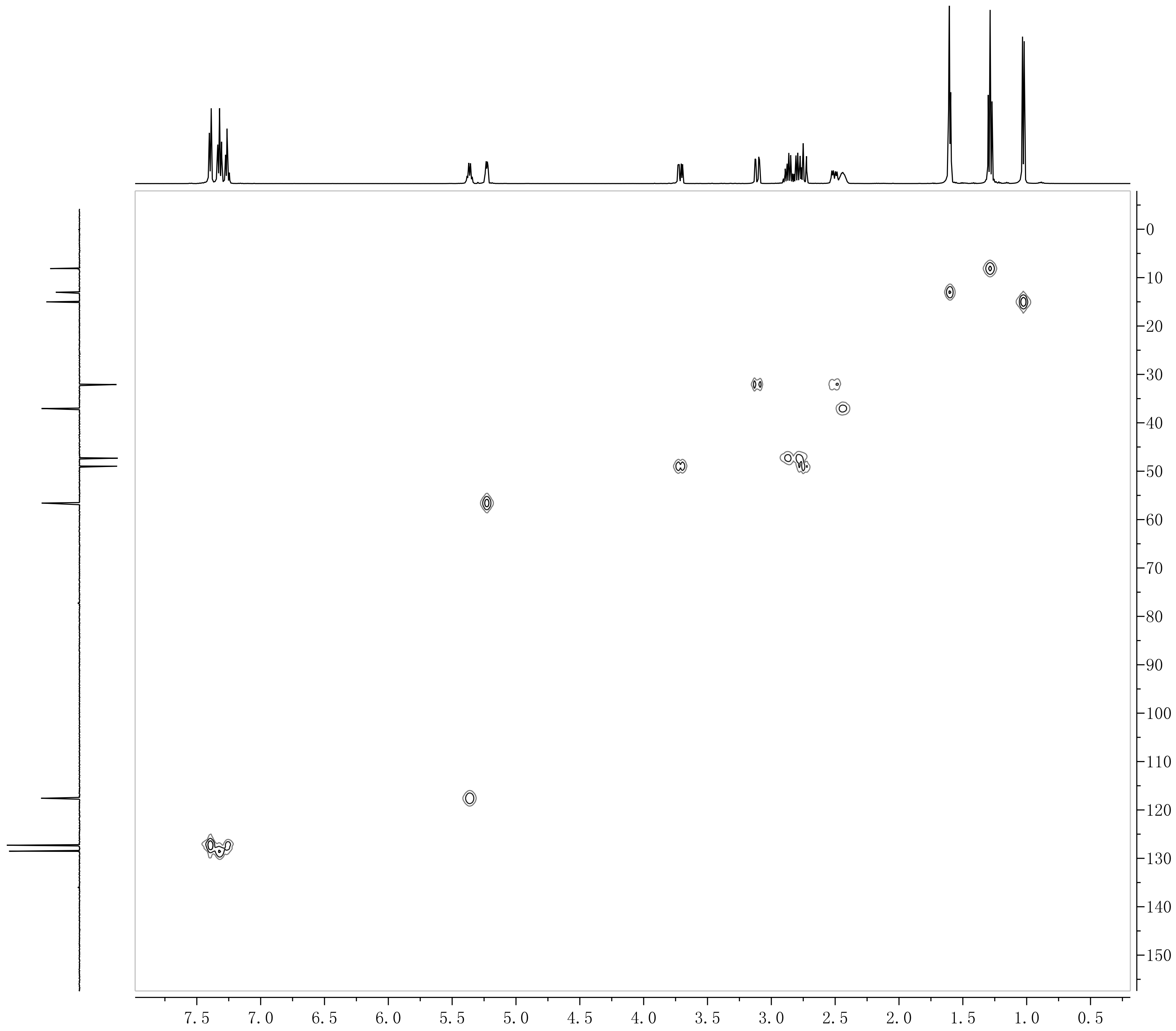

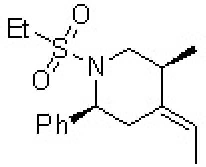

relative configuration

Parameter	Value
Title	xfy-190310-3-s.14.ser
Comment	
Origin	Bruker BioSpin GmbH
Owner	nmr
Site	
Instrument	spect
Solvent	CDCl3
Temperature	296.1
Pulse Sequence	hsqcedetgp
Experiment	HSQC-EDITED
Number of Scans	4
Receiver Gain	193.1
Relaxation Delay	1.4478
Pulse Width	10.7100
Presaturation Frequency	
Acquisition Time	0.1311
Acquisition Date	2019-03-11T01:23:14
Modification Date	2019-03-11T09:47:45
Spectrometer Frequency	(500.13, 125.77)
Spectral Width	(3906.2, 20833.3)
Lowest Frequency	(86.4, -1037.0)
Nucleus	(1H, 13C)
Acquired Size	(512, 256)
Spectral Size	(512, 512)

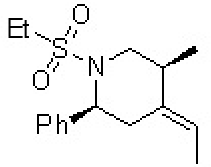

relative configuration

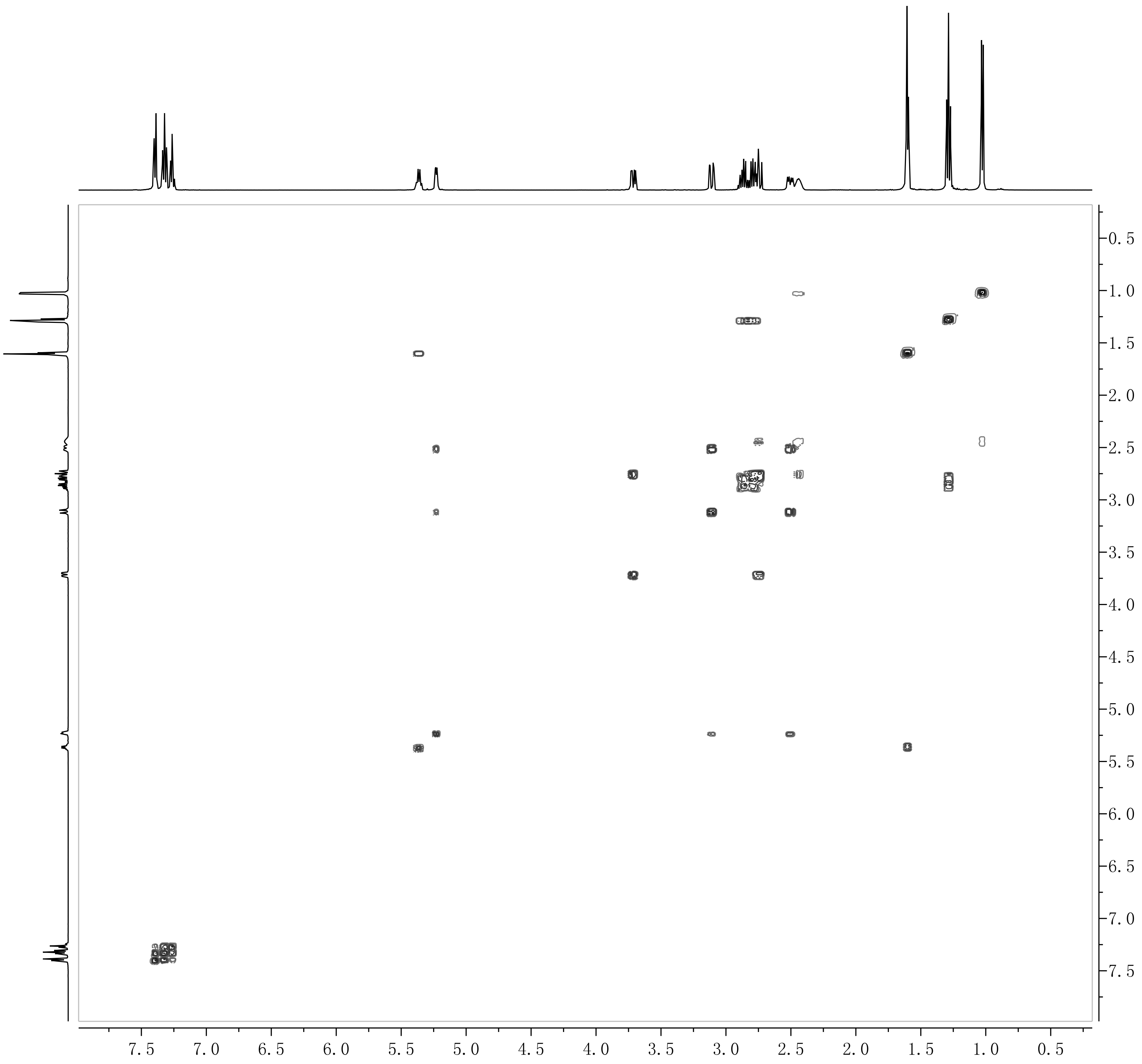

Parameter	Value
Title	xfy-190310-3-s.15.ser
Comment	
Origin	Bruker BioSpin GmbH
Owner	nmr
Site	
Instrument	spect
Solvent	CDCl3
Temperature	296.2
Pulse Sequence	cosygpppqf
Experiment	COSY
Number of Scans	4
Receiver Gain	44.7
Relaxation Delay	1.8910
Pulse Width	10.7100
Presaturation Frequency	
Acquisition Time	0.2621
Acquisition Date	2019-03-11T01:51:48
Modification Date	2019-03-11T09:47:45
Spectrometer Frequency	(500.13, 500.13)
Spectral Width	(3906.3, 3906.2)
Lowest Frequency	(86.3, 86.3)
Nucleus	(1H, 1H)
Acquired Size	(1024, 128)
Spectral Size	(1024, 1024)

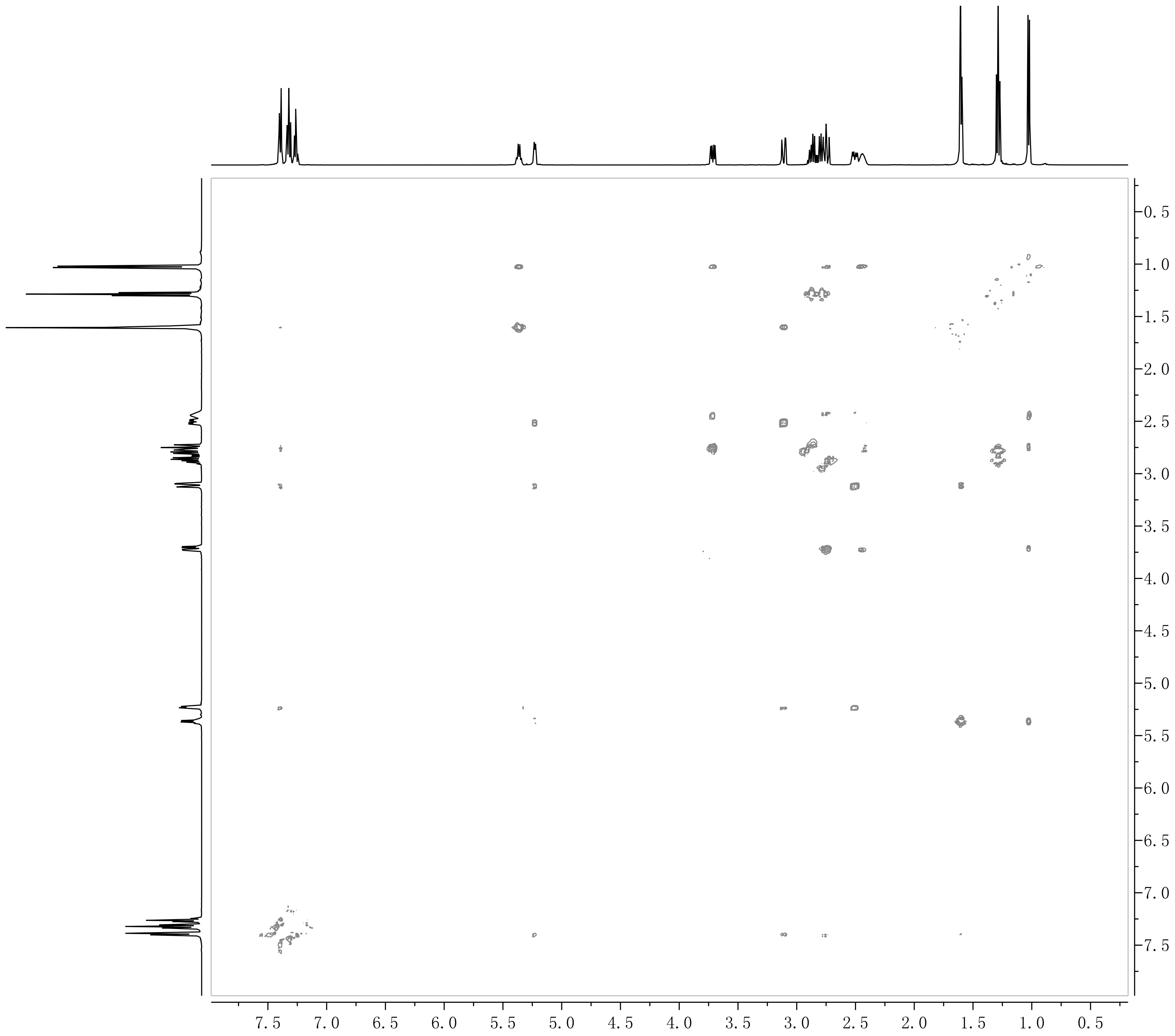

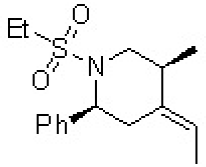

relative configuration

Parameter	Value
Title	xfy-190310-3-s.16.ser
Comment	
Origin	Bruker BioSpin GmbH
Owner	nmr
Site	
Instrument	spect
Solvent	CDCl3
Temperature	296.1
Pulse Sequence	noesygpphpp
Experiment	NOESY
Number of Scans	8
Receiver Gain	44.7
Relaxation Delay	1.9427
Pulse Width	10.7100
Presaturation Frequency	
Acquisition Time	0.2621
Class	
Spectrometer Frequency	(500.13, 500.13)
Spectral Width	(3906.3, 3906.2)
Lowest Frequency	(86.3, 86.3)
Nucleus	(1H, 1H)
Acquired Size	(1024, 256)
Spectral Size	(1024, 1024)

Parameter	Value
Title	xfy-190625-1-s.11.fid
Comment	
Origin	Bruker BioSpin GmbH
Owner	nmr
Site	
Instrument	spect
Solvent	CDCl3
Temperature	297.5
Pulse Sequence	zg30
Experiment	1D
Number of Scans	4
Receiver Gain	45.6
Relaxation Delay	1.0000
Pulse Width	8.7300
Presaturation Frequency	
Acquisition Time	1.9999
Acquisition Date	2019-06-25T22:02:22
Modification Date	2019-06-26T09:55:04
Spectrometer Frequency	400.13
Spectral Width	8012.8
Lowest Frequency	-1551.9
Nucleus	1H
Acquired Size	16025
Spectral Size	65536

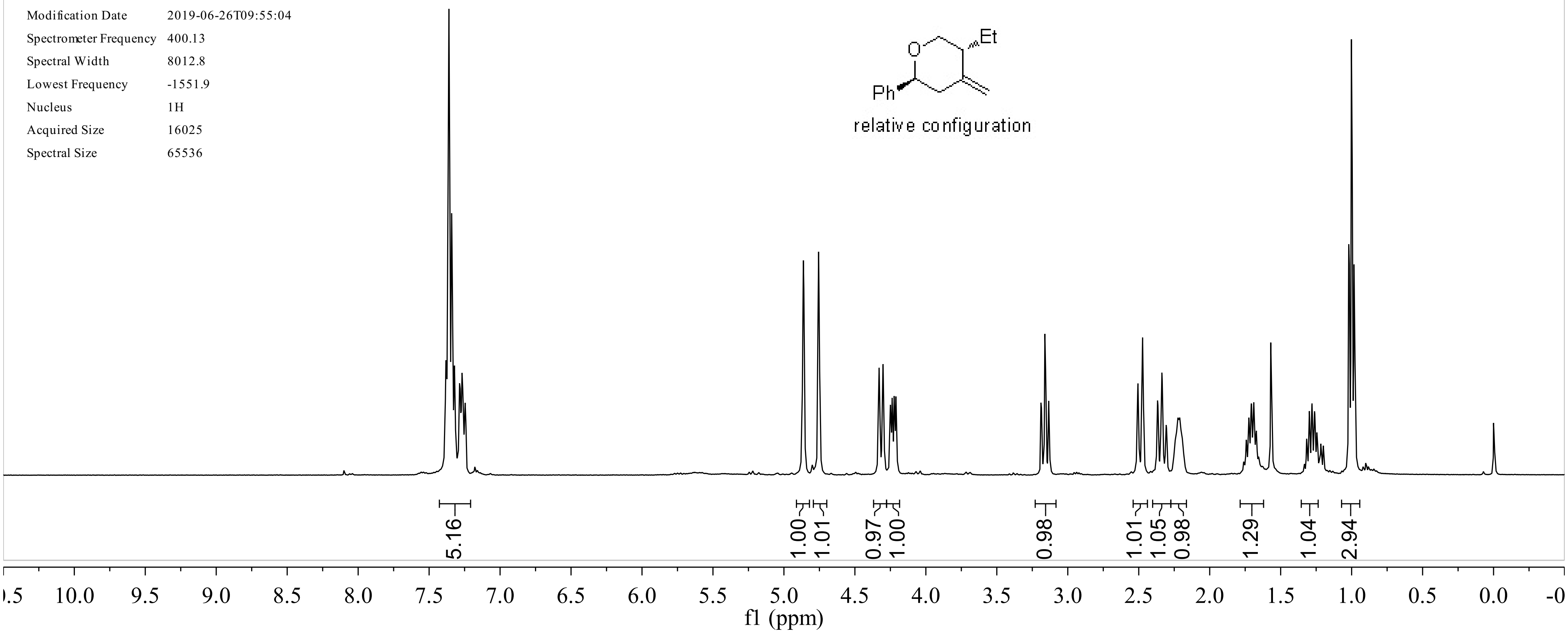

Parameter	Value
Title	xfy-190625-1-s.12.fid
Comment	
Origin	Bruker BioSpin GmbH
Owner	nmr
Site	
Instrument	spect
Solvent	CDCl3
Temperature	297.9
Pulse Sequence	zgpg30
Experiment	1D
Number of Scans	64
Receiver Gain	196.4
Relaxation Delay	2.0000
Pulse Width	10.0000
Presaturation Frequency	
Acquisition Time	1.3631
Acquisition Date	2019-06-25T22:07:15
Modification Date	2019-06-26T09:55:04
Spectrometer Frequency	100.62
Spectral Width	24038.5
Lowest Frequency	-1958.0
Nucleus	13C
Acquired Size	32768
Spectral Size	65536

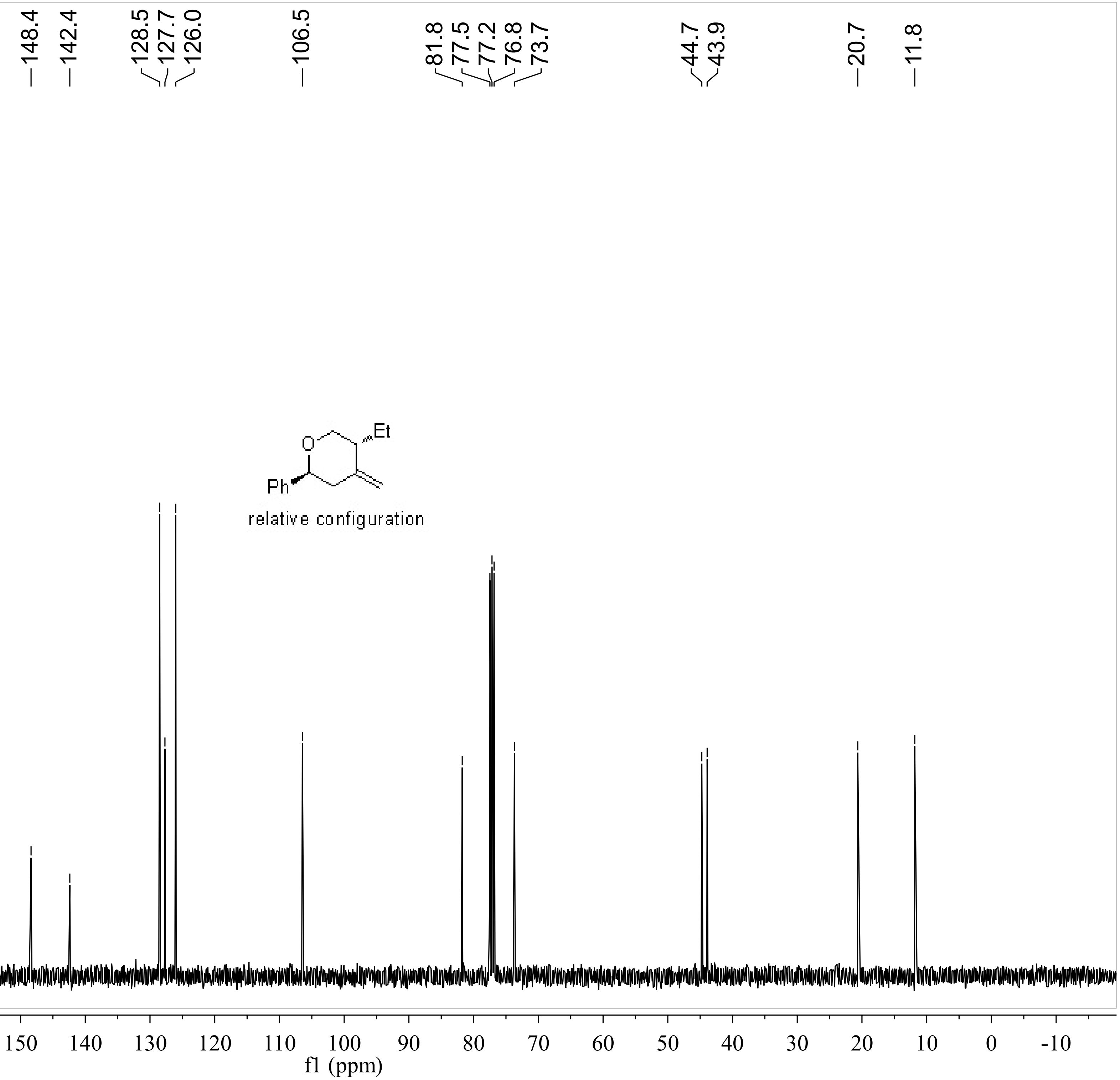

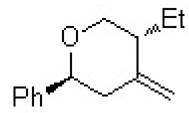

relative configuration

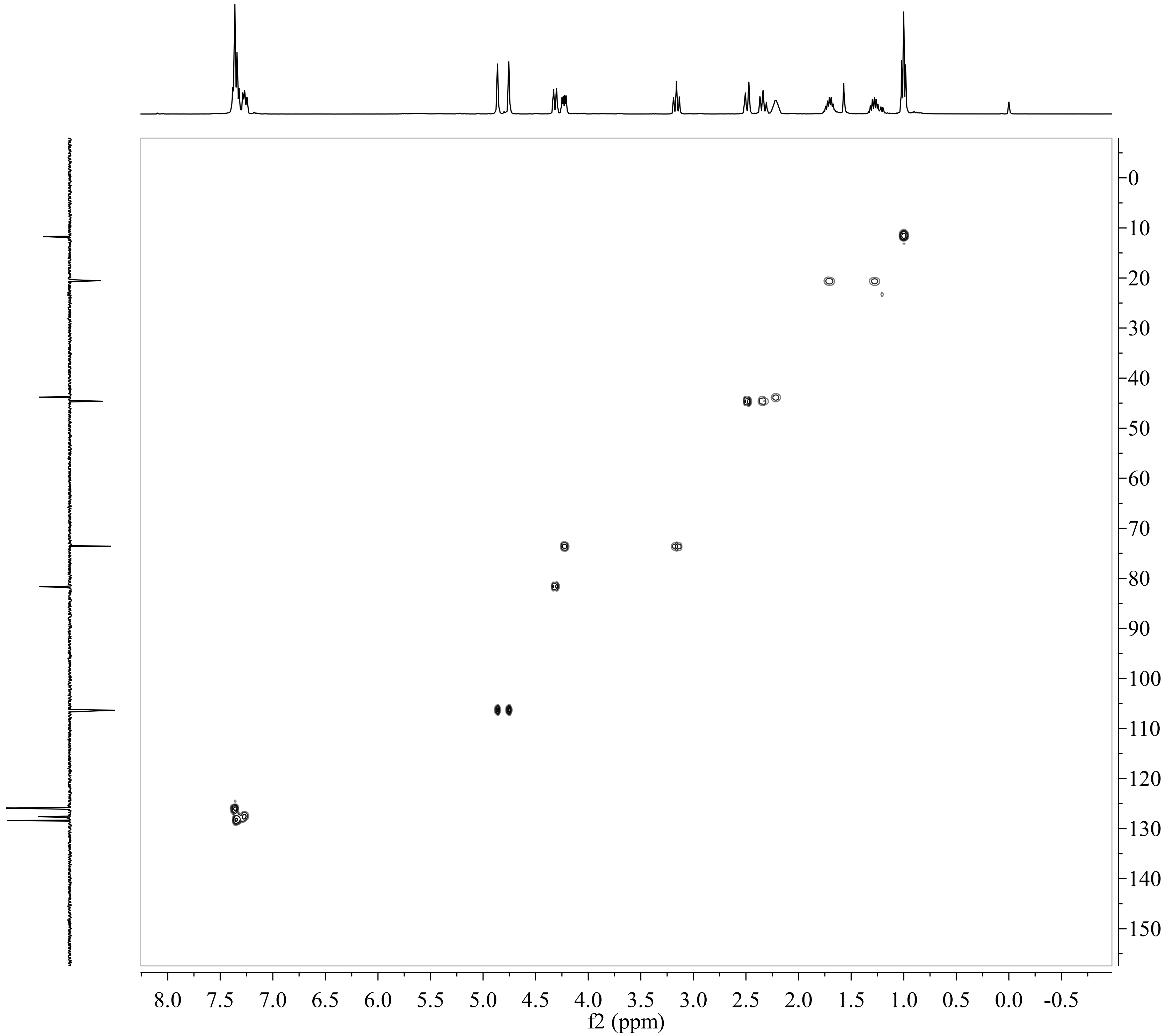

Parameter	Value
Title	xfy-190625-1-s.14.ser
Comment	
Origin	Bruker BioSpin GmbH
Owner	nmr
Site	
Instrument	spect
Solvent	CDCl3
Temperature	297.7
Pulse Sequence	hsqcetgp
Experiment	HSQC
Number of Scans	2
Receiver Gain	196.4
Relaxation Delay	1.4603
Pulse Width	8.7300
Presaturation Frequency	
Acquisition Time	0.1382
Acquisition Date	2019-06-25T22:11:42
Modification Date	2019-06-26T09:55:05
Spectrometer Frequency	(400.13, 100.62)
Spectral Width	(3703.7, 16666.7)
Lowest Frequency	(-399.4, -829.1)
Nucleus	(1H, 13C)
Acquired Size	(512, 256)
Spectral Size	(512, 512)

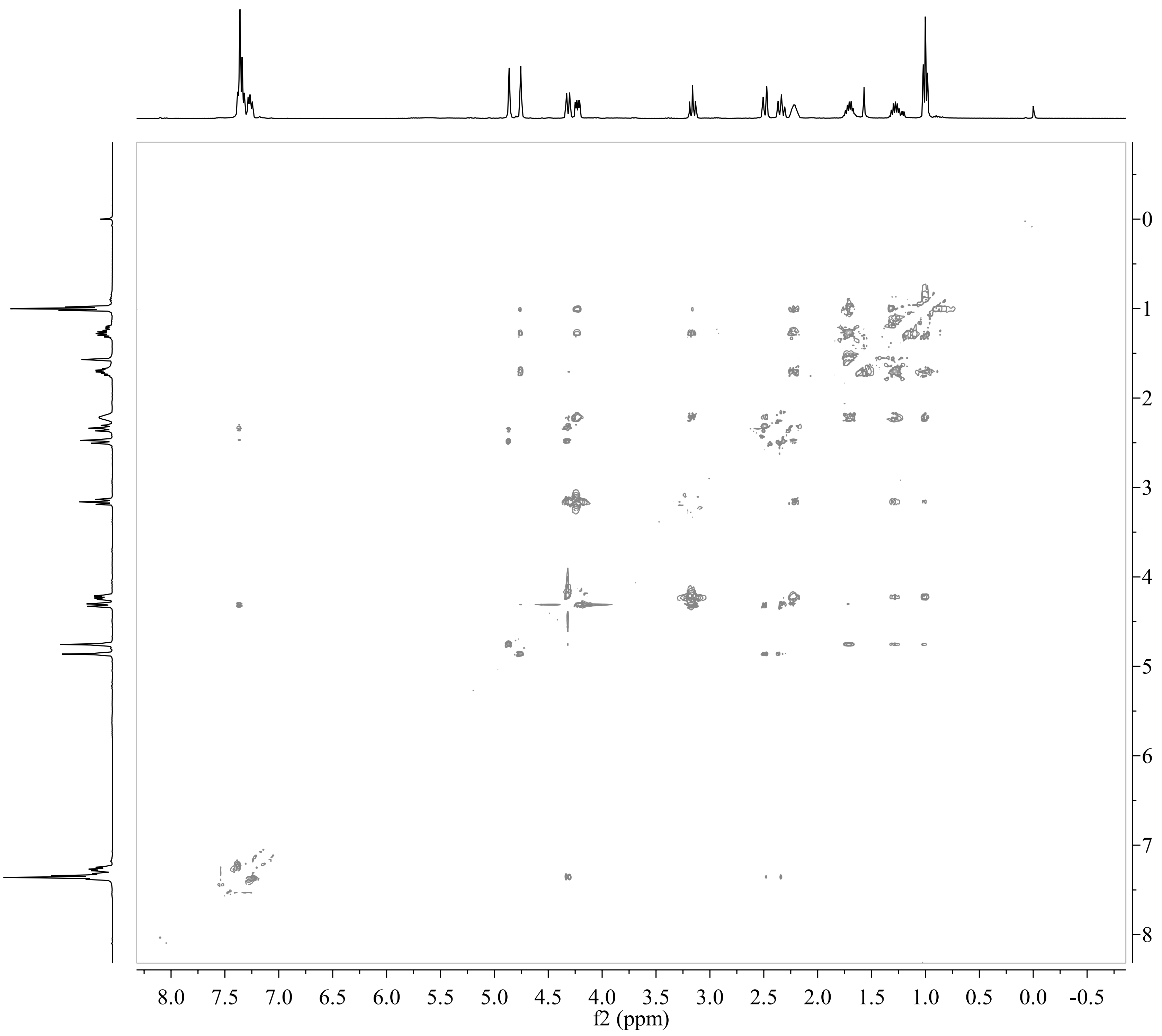

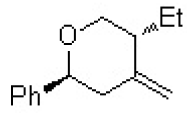

relative configuration

Parameter	Value
Title	xfy-190625-1-s.32.ser
Comment	
Origin	Bruker BioSpin GmbH
Owner	nmr
Site	
Instrument	spect
Solvent	CDCl3
Temperature	297.6
Pulse Sequence	noesygpphpp
Experiment	NOESY
Number of Scans	20
Receiver Gain	54.9
Relaxation Delay	1.9775
Pulse Width	8.7300
Presaturation Frequency	
Acquisition Time	0.2785
Acquisition Date	2019-06-28T00:34:52
Modification Date	2019-06-28T13:10:13
Spectrometer Frequency	(400.13, 400.13)
Spectral Width	(3676.5, 3676.5)
Lowest Frequency	(-347.4, -347.4)
Nucleus	(1H, 1H)
Acquired Size	(1024, 256)
Spectral Size	(1024, 1024)

Parameter	Value
Title	xfy-190623-1-s.1.1.1r
Comment	
Origin	Bruker BioSpin GmbH
Owner	nmr
Site	
Instrument	spect
Solvent	CDCl3
Temperature	298.7
Pulse Sequence	zg30
Experiment	1D
Number of Scans	8
Receiver Gain	87.6
Relaxation Delay	1.0000
Pulse Width	8.7300
Presaturation Frequency	
Acquisition Time	1.9999
Acquisition Date	2019-06-23T16:55:03
Modification Date	2019-06-23T18:44:04
Spectrometer Frequency	400.13
Spectral Width	8012.8
Lowest Frequency	-1535.4
Nucleus	1H
Acquired Size	16025
Spectral Size	65536

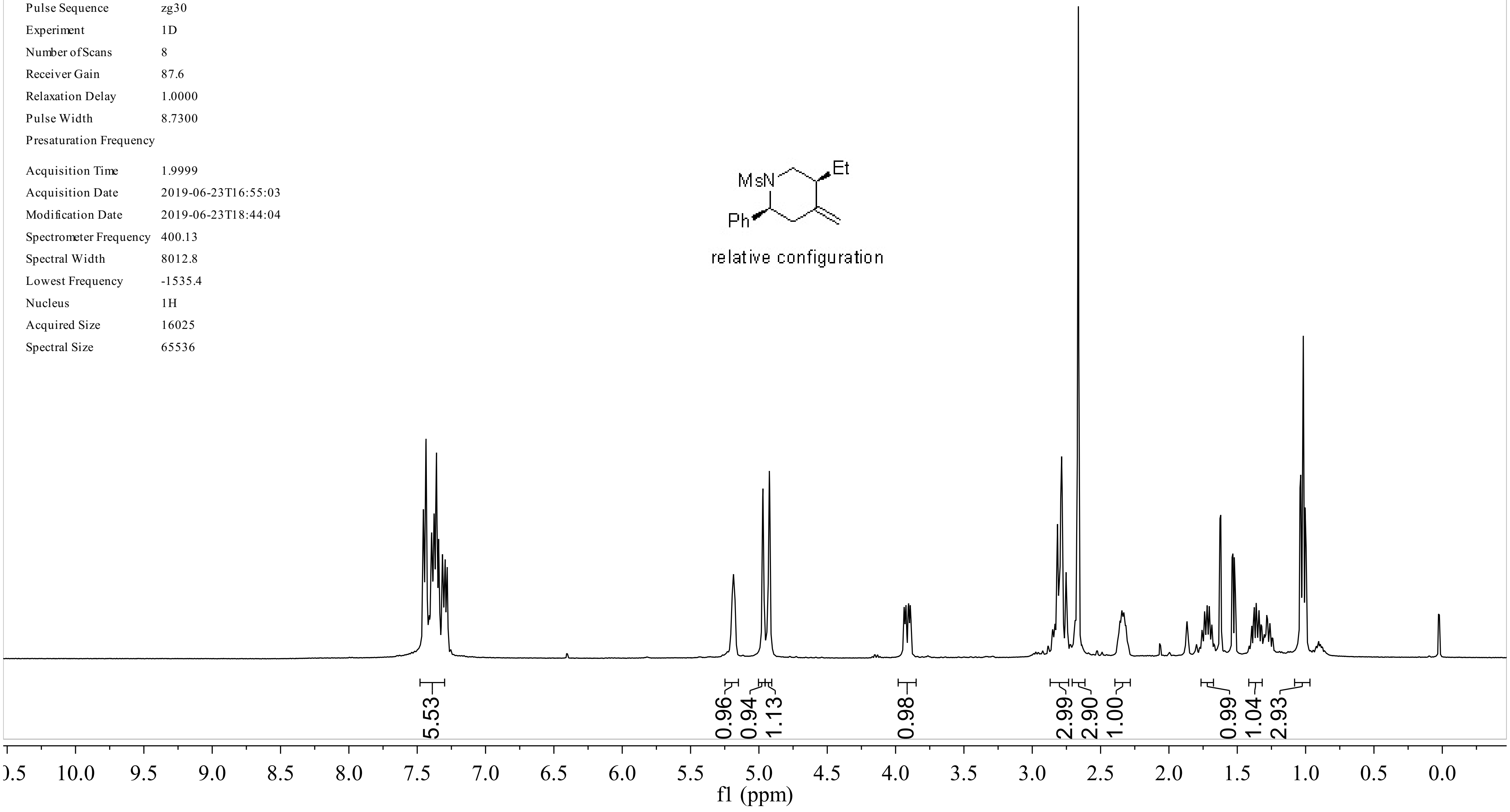

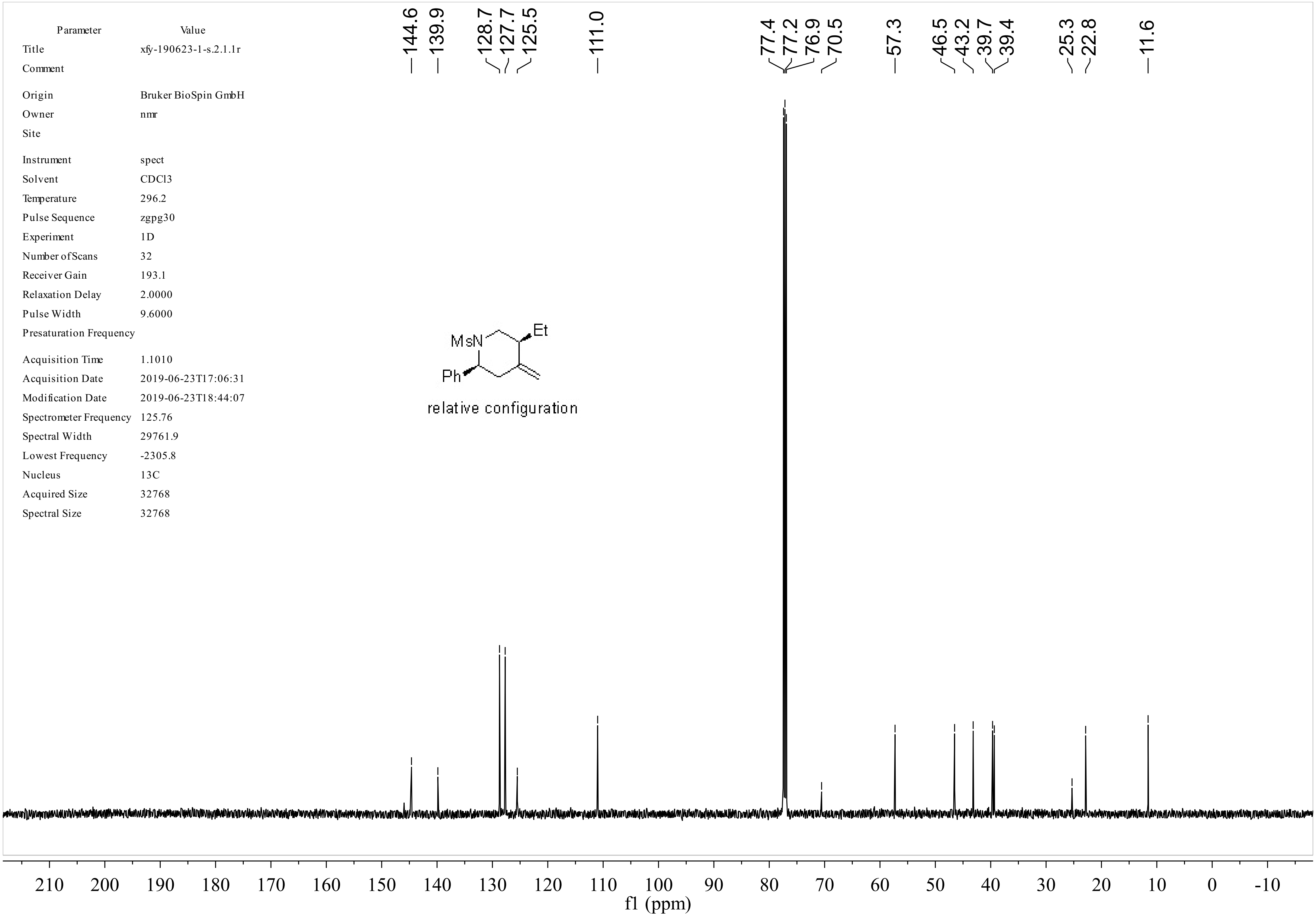

Parameter Value
Title xfy-190623-1-s.2.1.1r
Comment
Origin Bruker BioSpin GmbH
Owner nmr
Site
Instrument spect
Solvent CDCl3
Temperature 296.2
Pulse Sequence zgpg30
Experiment 1D
Number of Scans 32
Receiver Gain 193.1
Relaxation Delay 2.0000
Pulse Width 9.6000
Presaturation Frequency
Acquisition Time 1.1010
Acquisition Date 2019-06-23T17:06:31
Modification Date 2019-06-23T18:44:07
Spectrometer Frequency 125.76
Spectral Width 29761.9
Lowest Frequency -2305.8
Nucleus 13C
Acquired Size 32768
Spectral Size 32768
144.6
139.9
128.7
127.7
125.5
111.0
77.4
77.2
76.9
70.5
57.3
46.5
43.2
39.7
39.4
25.3
22.8
11.6
MsN
Et
Ph
relative configuration
210 200 190 180 170 160 150 140 130 120 110 100 90 80 70 60 50 40 30 20 10 0 -10
f1 (ppm)

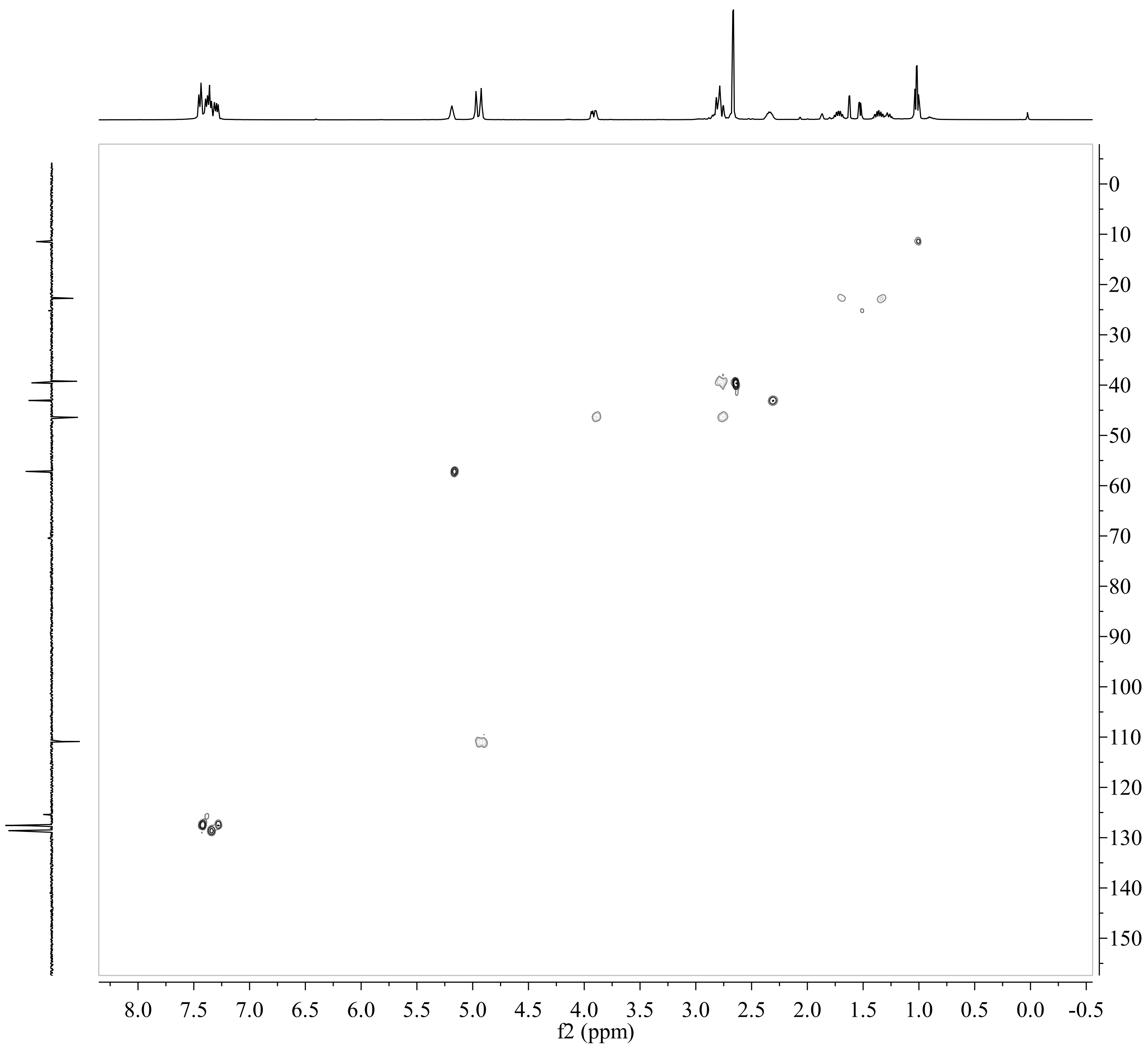

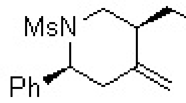

relative configuration

Parameter	Value
Title	xfy-190623-1-s.4.ser
Comment	
Origin	Bruker BioSpin GmbH
Owner	nmr
Site	
Instrument	spect
Solvent	CDCl3
Temperature	296.2
Pulse Sequence	hsqcedetgp
Experiment	HSQC-EDITED
Number of Scans	2
Receiver Gain	193.1
Relaxation Delay	1.4642
Pulse Width	10.7100
Presaturation Frequency	
Acquisition Time	0.1147
Acquisition Date	2019-06-23T17:11:40
Modification Date	2019-06-23T18:44:07
Spectrometer Frequency	(500.13, 125.77)
Spectral Width	(4464.3, 20833.3)
Lowest Frequency	(-287.3, -1037.0)
Nucleus	(1H, 13C)
Acquired Size	(512, 256)
Spectral Size	(512, 512)

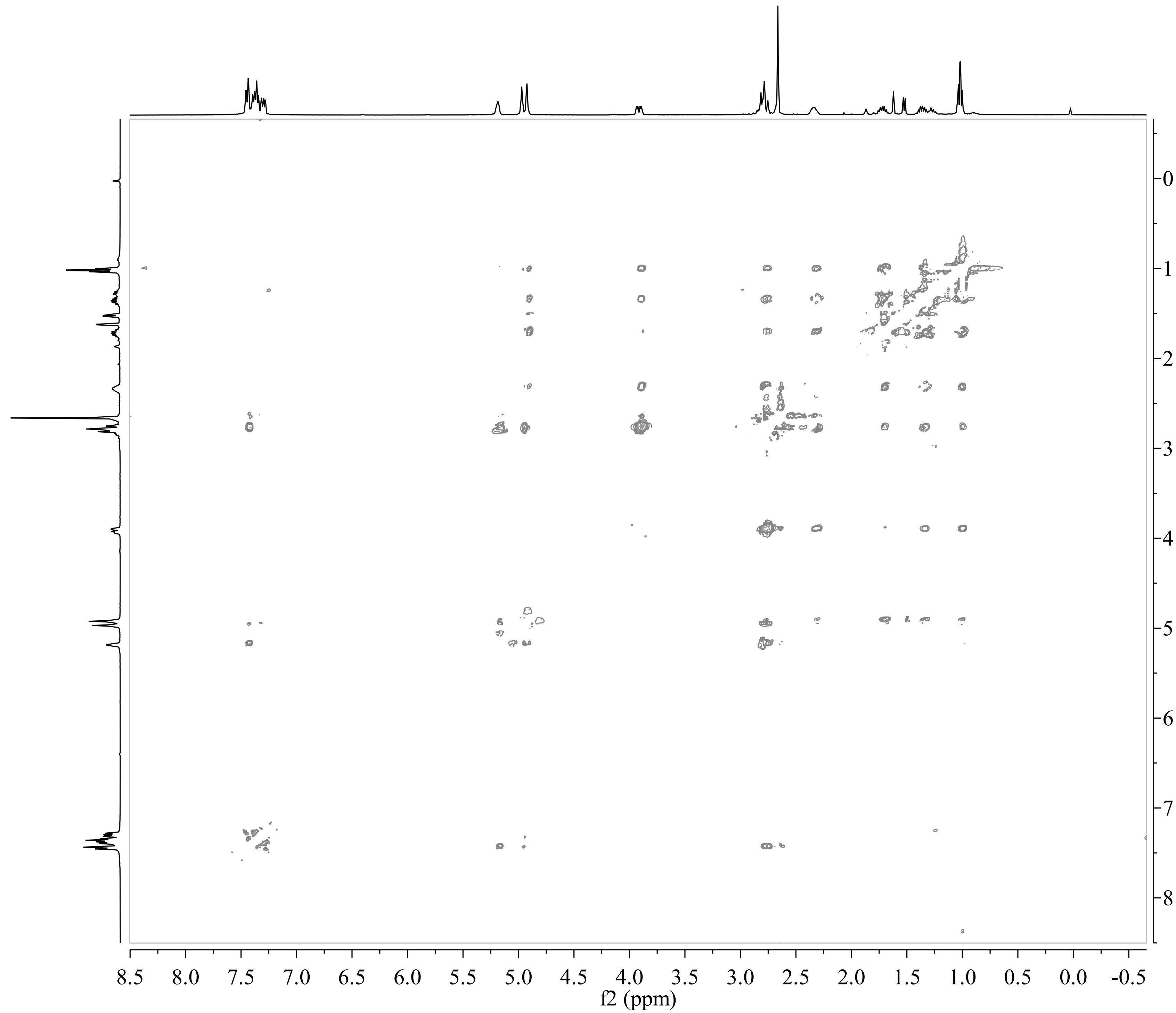

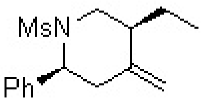

relative configuration

Parameter	Value
Title	xfy-190623-1-s.14.ser
Comment	
Origin	Bruker BioSpin GmbH
Owner	nmr
Site	
Instrument	spect
Solvent	CDCl3
Temperature	296.1
Pulse Sequence	noesygpphpp
Experiment	NOESY
Number of Scans	4
Receiver Gain	25.2
Relaxation Delay	1.9816
Pulse Width	10.7100
Presaturation Frequency	
Acquisition Time	0.2232
Acquisition Date	2019-06-23T17:54:27
Modification Date	2019-06-23T18:44:05
Spectrometer Frequency	(500.13, 500.13)
Spectral Width	(4587.2, 4587.2)
Lowest Frequency	(-334.6, -334.6)
Nucleus	(1H, 1H)
Acquired Size	(1024, 256)
Spectral Size	(1024, 1024)

Parameter	Value
Title	xfy-190704-4-s.1.fid
Comment	
Origin	Bruker BioSpin GmbH
Owner	nmr
Site	
Instrument	spect
Solvent	CDCl3
Temperature	296.2
Pulse Sequence	zg30
Experiment	1D
Number of Scans	16
Receiver Gain	87.5
Relaxation Delay	1.0000
Pulse Width	10.7100
Presaturation Frequency	
Acquisition Time	3.2768
Acquisition Date	2019-07-05T22:23:16
Modification Date	2019-07-06T10:54:40
Spectrometer Frequency	500.13
Spectral Width	10000.0
Lowest Frequency	-1923.4
Nucleus	1H
Acquired Size	32768
Spectral Size	65536

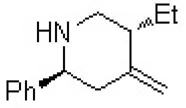

relative configuration

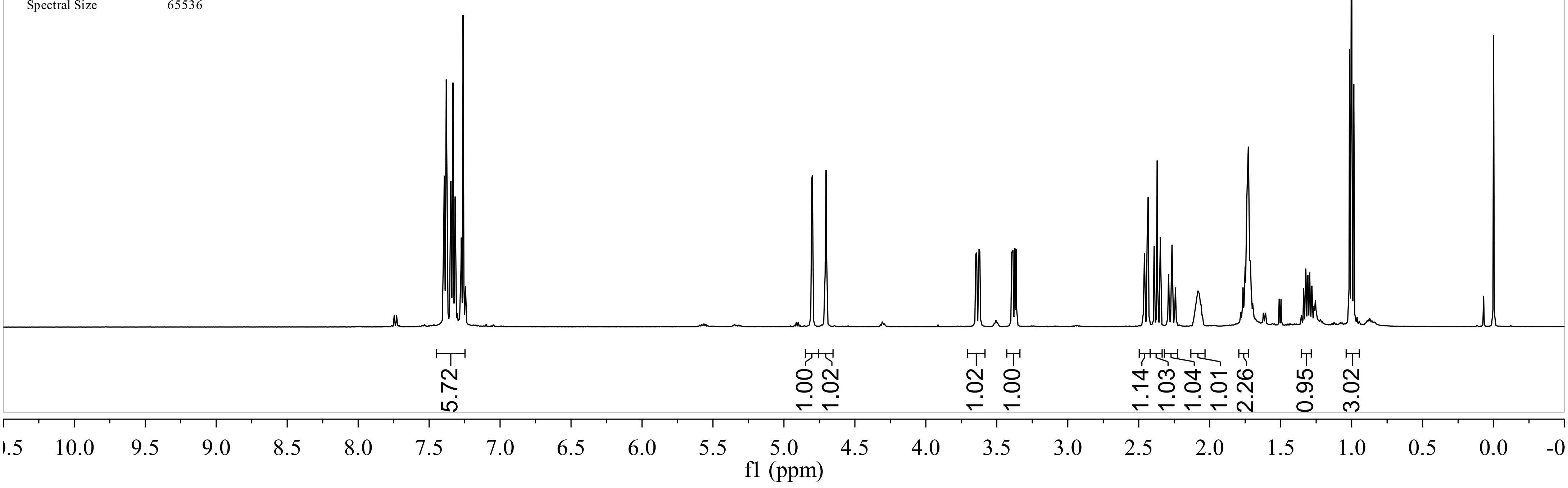

Parameter	Value
Title	xfy-190704-4-s.4.fid
Comment	
Origin	Bruker BioSpin GmbH
Owner	nmr
Site	
Instrument	spect
Solvent	CDCl3
Temperature	296.2
Pulse Sequence	zgpg30
Experiment	1D
Number of Scans	80
Receiver Gain	193.1
Relaxation Delay	2.0000
Pulse Width	9.6000
Presaturation Frequency	
Acquisition Time	1.1010
Acquisition Date	2019-07-05T22:58:35
Modification Date	2019-07-06T10:54:41
Spectrometer Frequency	125.77
Spectral Width	29761.9
Lowest Frequency	-2305.8
Nucleus	13C
Acquired Size	32768
Spectral Size	65536

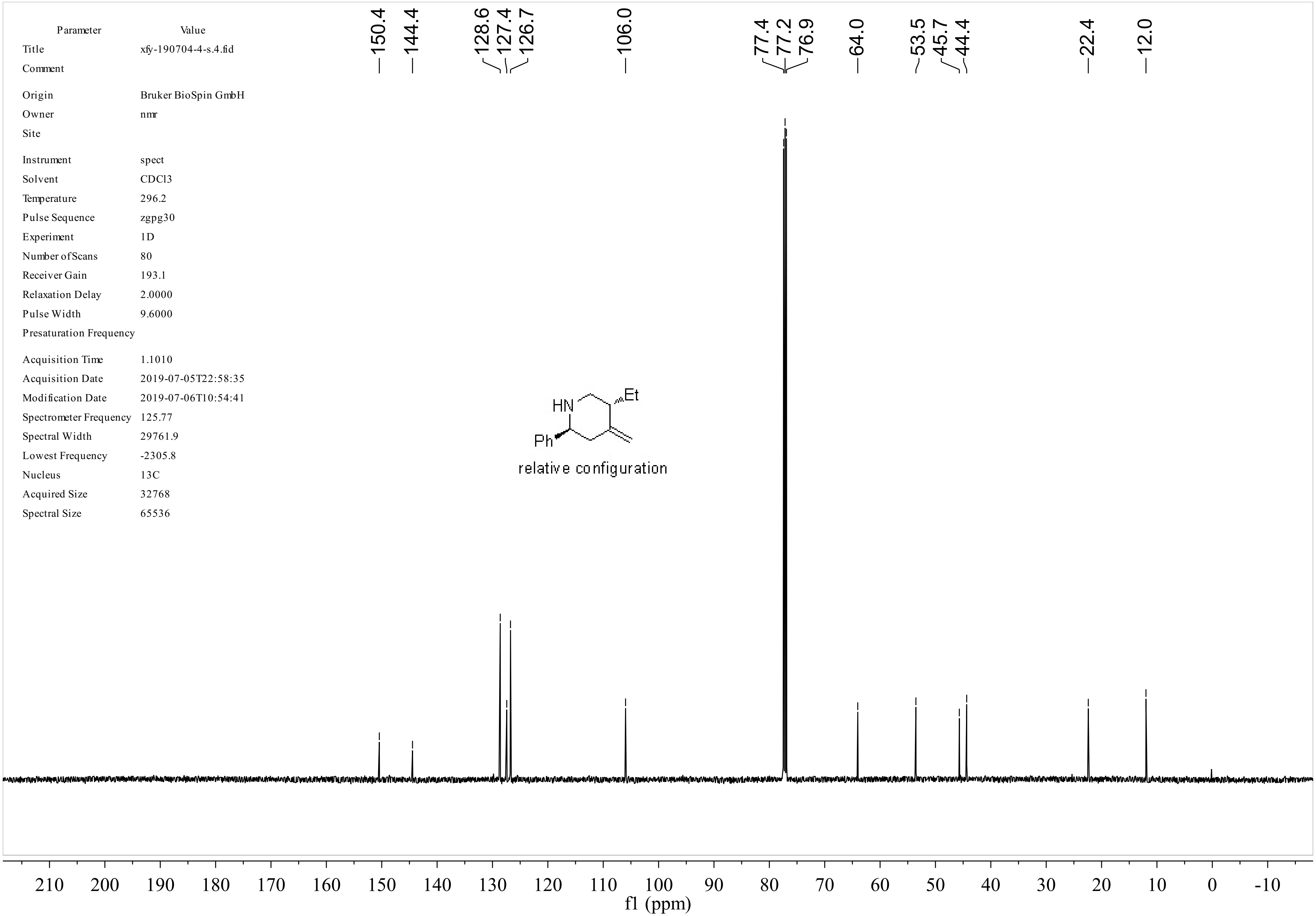

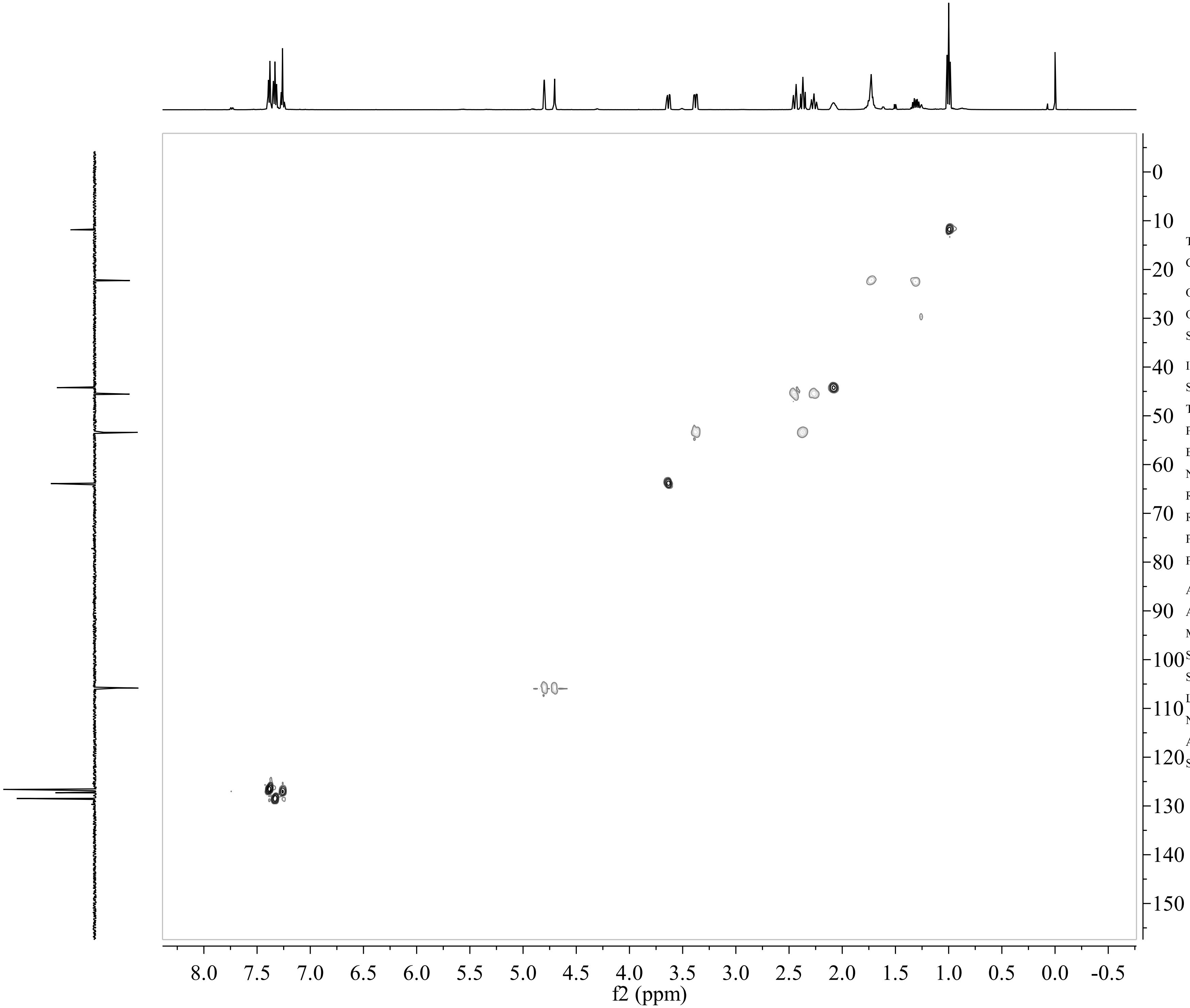

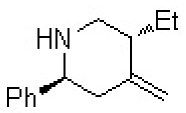

relative configuration

Parameter	Value
Title	xfy-190704-4-s.5.ser
Comment	
Origin	Bruker BioSpin GmbH
Owner	nmr
Site	
Instrument	spect
Solvent	CDCl3
Temperature	296.1
Pulse Sequence	hsqcedetgp
Experiment	HSQC-EDITED
Number of Scans	4
Receiver Gain	193.1
Relaxation Delay	1.4672
Pulse Width	10.7100
Presaturation Frequency	
Acquisition Time	0.1116
Acquisition Date	2019-07-05T23:00:19
Modification Date	2019-07-06T10:54:41
Spectrometer Frequency	(500.13, 125.77)
Spectral Width	(4587.2, 20833.3)
Lowest Frequency	(-391.7, -1037.0)
Nucleus	(1H, 13C)
Acquired Size	(512, 256)
Spectral Size	(512, 512)

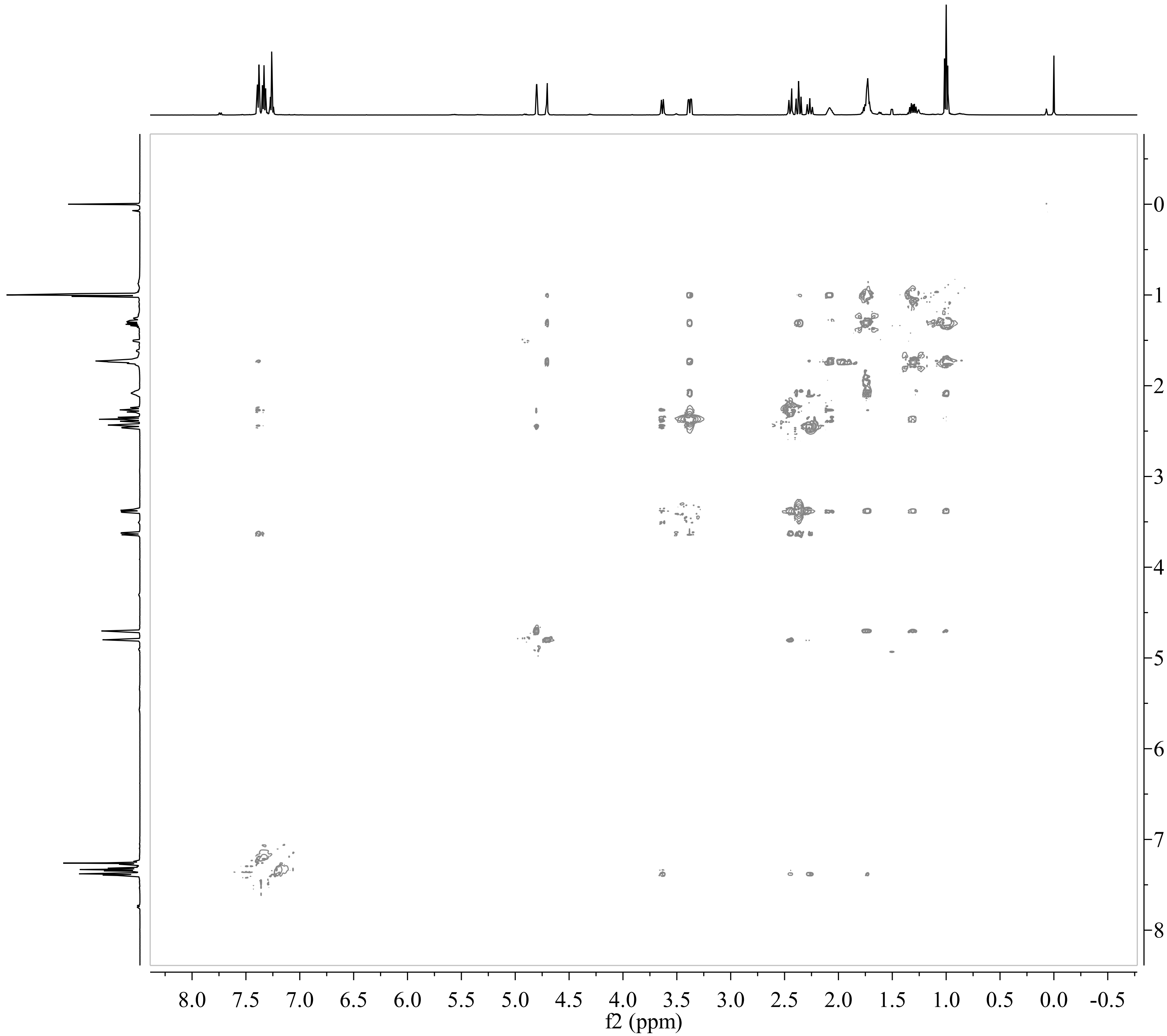

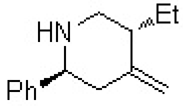

relative configuration

Parameter	Value
Title	xfy-190704-4-s.6.ser
Comment	
Origin	Bruker BioSpin GmbH
Owner	nmr
Site	
Instrument	spect
Solvent	CDCl3
Temperature	296.1
Pulse Sequence	noesygpphpp
Experiment	NOESY
Number of Scans	20
Receiver Gain	31.1
Relaxation Delay	1.9816
Pulse Width	10.7100
Presaturation Frequency	
Acquisition Time	0.2232
Acquisition Date	2019-07-05T23:30:48
Modification Date	2019-07-06T10:54:43
Spectrometer Frequency	(500.13, 500.13)
Spectral Width	(4587.2, 4587.2)
Lowest Frequency	(-391.6, -391.6)
Nucleus	(1H, 1H)
Acquired Size	(1024, 256)
Spectral Size	(1024, 1024)

Parameter	Value
标题	xfy-190704-4-dMs.11.1.1r
Comment	
Origin	Bruker BioSpin GmbH
Owner	nmr
Site	
Instrument	spect
Author	
Solvent	CDCl3
Temperature	296.1
Pulse Sequence	zg30
Experiment	1D
Number of Scans	16
Receiver Gain	87.5
Relaxation Delay	1.0000
Pulse Width	10.7100
Presaturation Frequency	
Acquisition Time	3.2768
Class	
Spectrometer Frequency	500.13
Spectral Width	10000.0
Lowest Frequency	-1920.5
Nucleus	1H
Acquired Size	32768
Spectral Size	65536

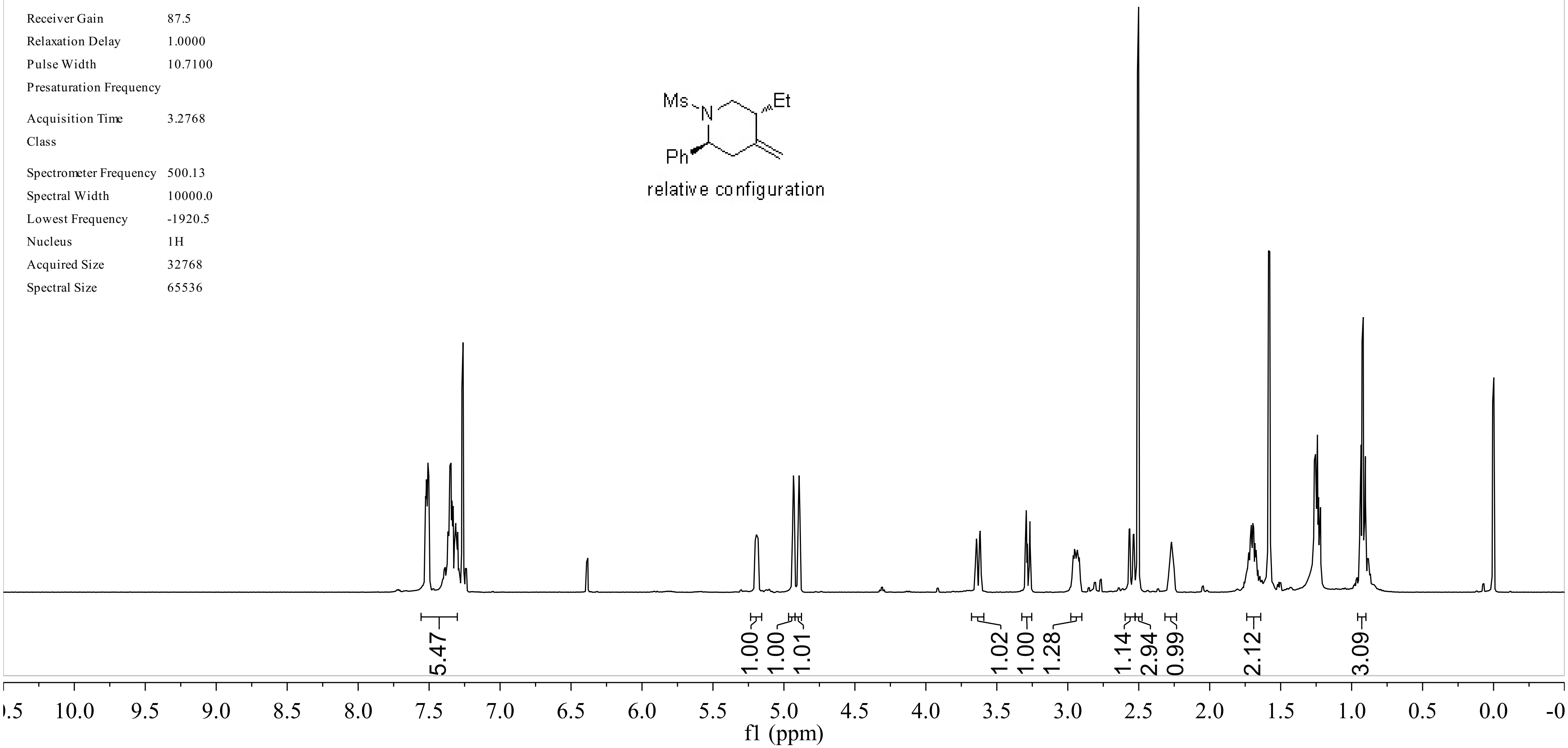

Parameter	Value
Title	xfy-190704-4-dMs.12.fid
Comment	
Origin	Bruker BioSpin GmbH
Owner	nmr
Site	
Instrument	spect
Solvent	CDCl3
Temperature	296.1
Pulse Sequence	zgpg30
Experiment	1D
Number of Scans	128
Receiver Gain	193.1
Relaxation Delay	2.0000
Pulse Width	9.6000
Presaturation Frequency	
Acquisition Time	1.1010
Acquisition Date	2019-07-06T06:07:58
Modification Date	2019-07-06T10:54:45
Spectrometer Frequency	125.77
Spectral Width	29761.9
Lowest Frequency	-2305.8
Nucleus	13C
Acquired Size	32768
Spectral Size	65536

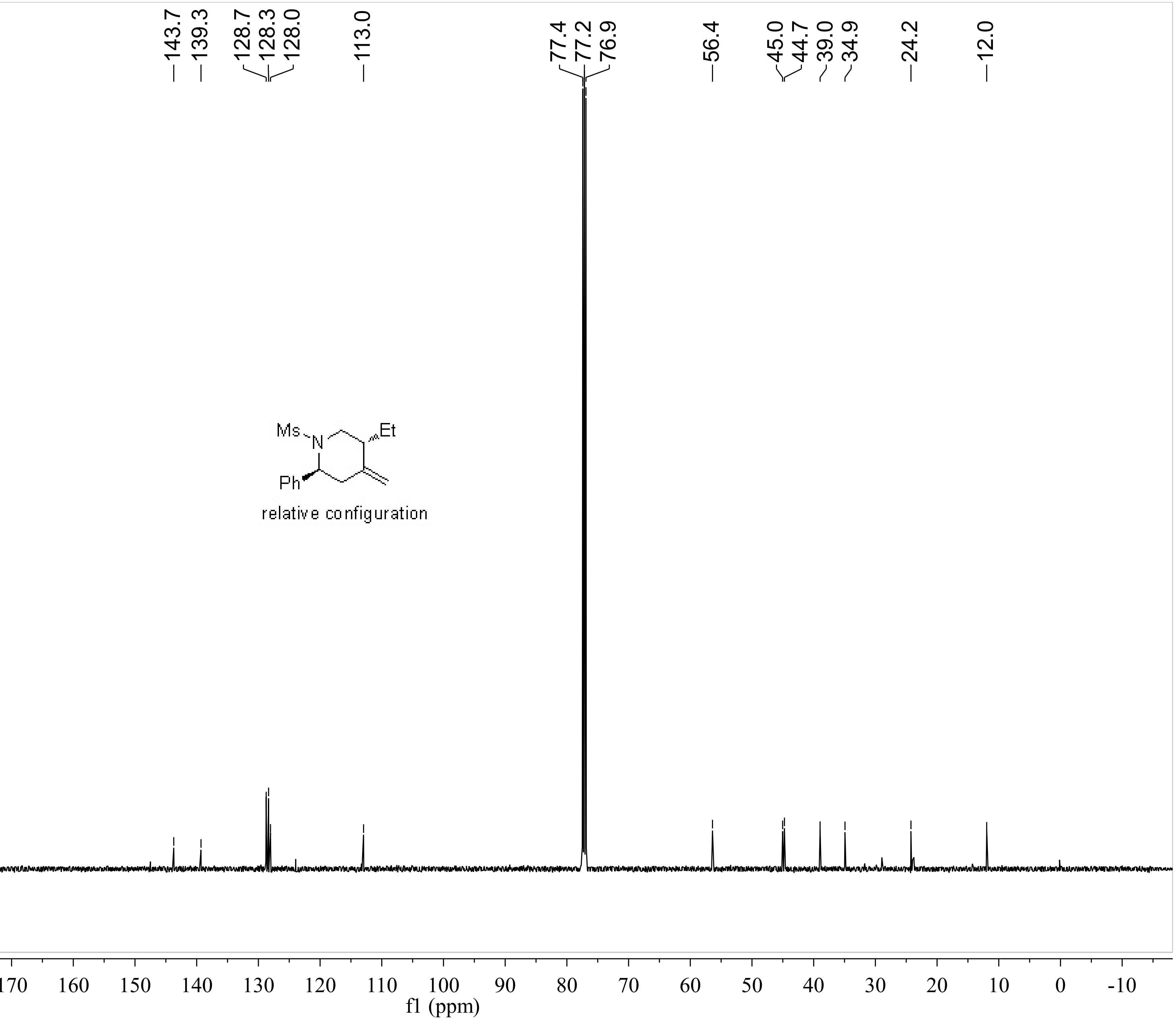

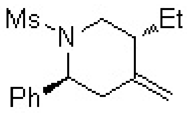

relative configuration

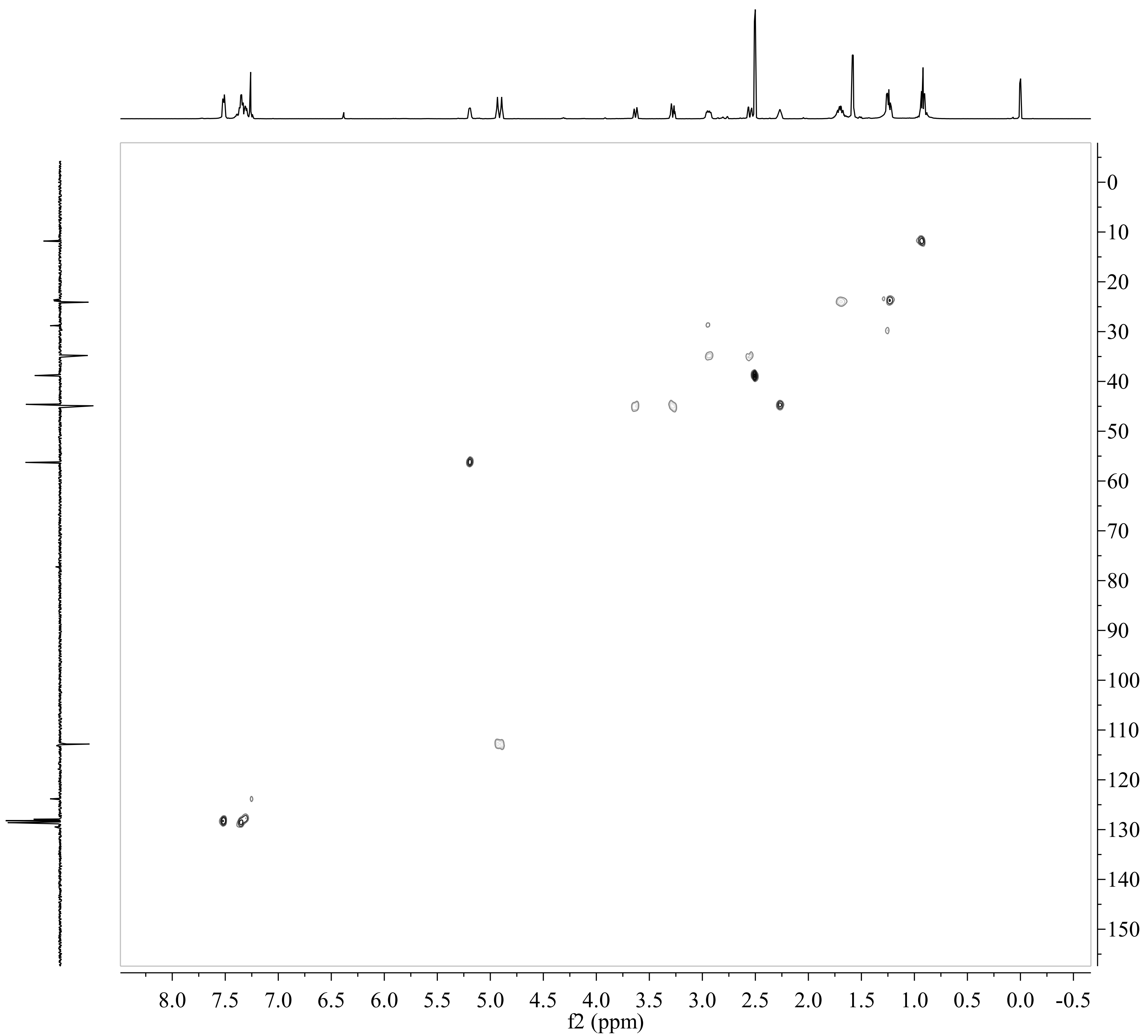

Parameter	Value
标题	xfy-190704-4-dMs.14.ser
Comment	
Origin	Bruker BioSpin GmbH
Owner	nmr
Site	
Instrument	spect
Author	
Solvent	CDCl3
Temperature	296.2
Pulse Sequence	hsqcedetgp
Experiment	HSQC-EDITED
Number of Scans	4
Receiver Gain	193.1
Relaxation Delay	1.4672
Pulse Width	10.7100
Presaturation Frequency	
Acquisition Time	0.1116
Class	
Spectrometer Frequency	(500.13, 125.77)
Spectral Width	(4587.2, 20833.3)
Lowest Frequency	(-341.5, -1037.0)
Nucleus	(1H, 13C)
Acquired Size	(512, 256)
Spectral Size	(512, 512)

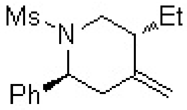

relative configuration

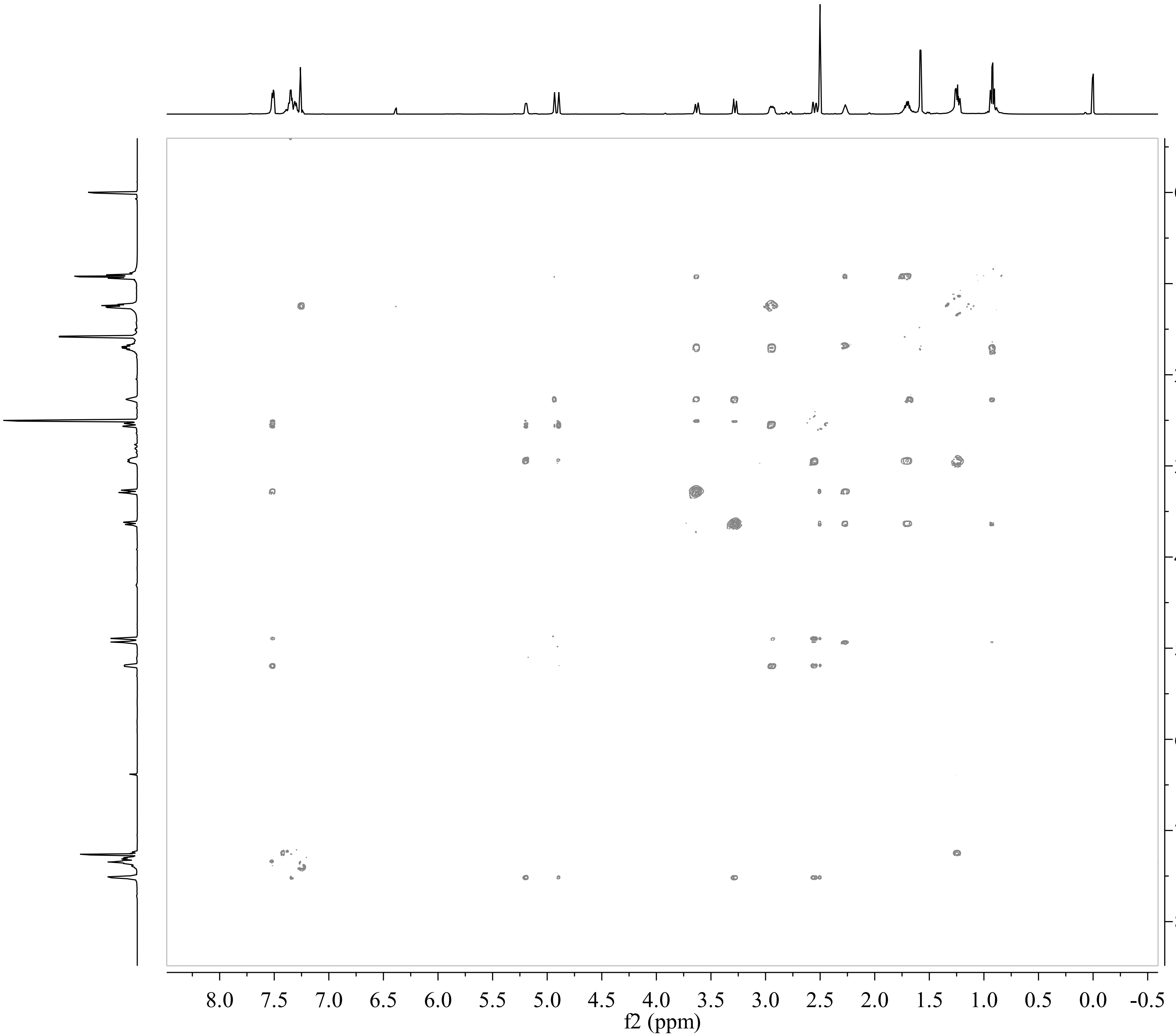

Parameter	Value
标题	xfy-190704-4-dMs.15.ser
Comment	
Origin	Bruker BioSpin GmbH
Owner	nmr
Site	
Instrument	spect
Author	
Solvent	CDCl3
Temperature	296.2
Pulse Sequence	noesygpphpp
Experiment	NOESY
Number of Scans	8
Receiver Gain	31.1
Relaxation Delay	1.9795
Pulse Width	10.7100
Presaturation Frequency	
Acquisition Time	0.2253
Class	
Spectrometer Frequency	(500.13, 500.13)
Spectral Width	(4545.5, 4545.5)
Lowest Frequency	(-302.0, -302.0)
Nucleus	(1H, 1H)
Acquired Size	(1024, 256)
Spectral Size	(1024, 1024)

Parameter	Value
Title	xfy-190918-1-s.1.1.1r
Comment	
Origin	Bruker BioSpin GmbH
Owner	nmr
Site	
Instrument	spect
Solvent	CDCl3
Temperature	296.2
Pulse Sequence	zg30
Experiment	1D
Number of Scans	12
Receiver Gain	95.3
Relaxation Delay	1.0000
Pulse Width	10.7100
Presaturation Frequency	
Acquisition Time	3.2768
Acquisition Date	2019-09-20T00:24:42
Modification Date	2019-09-20T08:56:27
Spectrometer Frequency	500.13
Spectral Width	10000.0
Lowest Frequency	-1922.5
Nucleus	1H
Acquired Size	32768
Spectral Size	65536

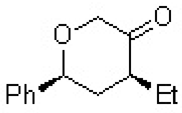

relative configuration

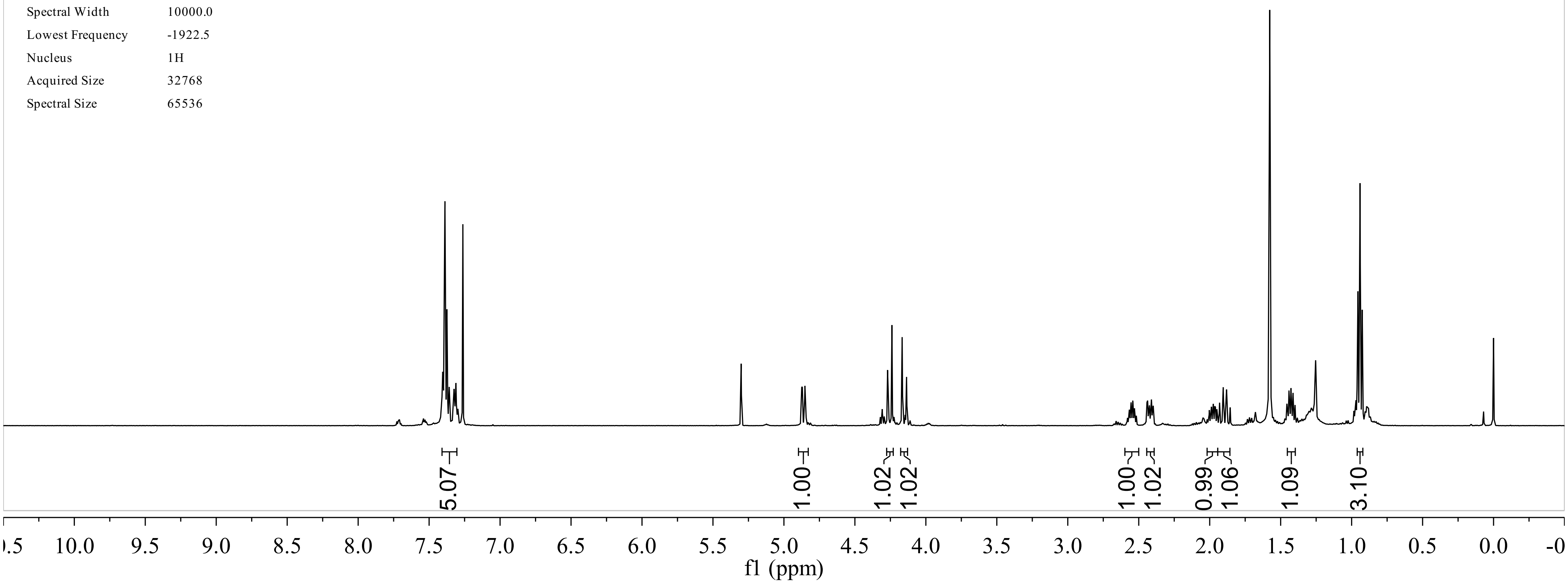

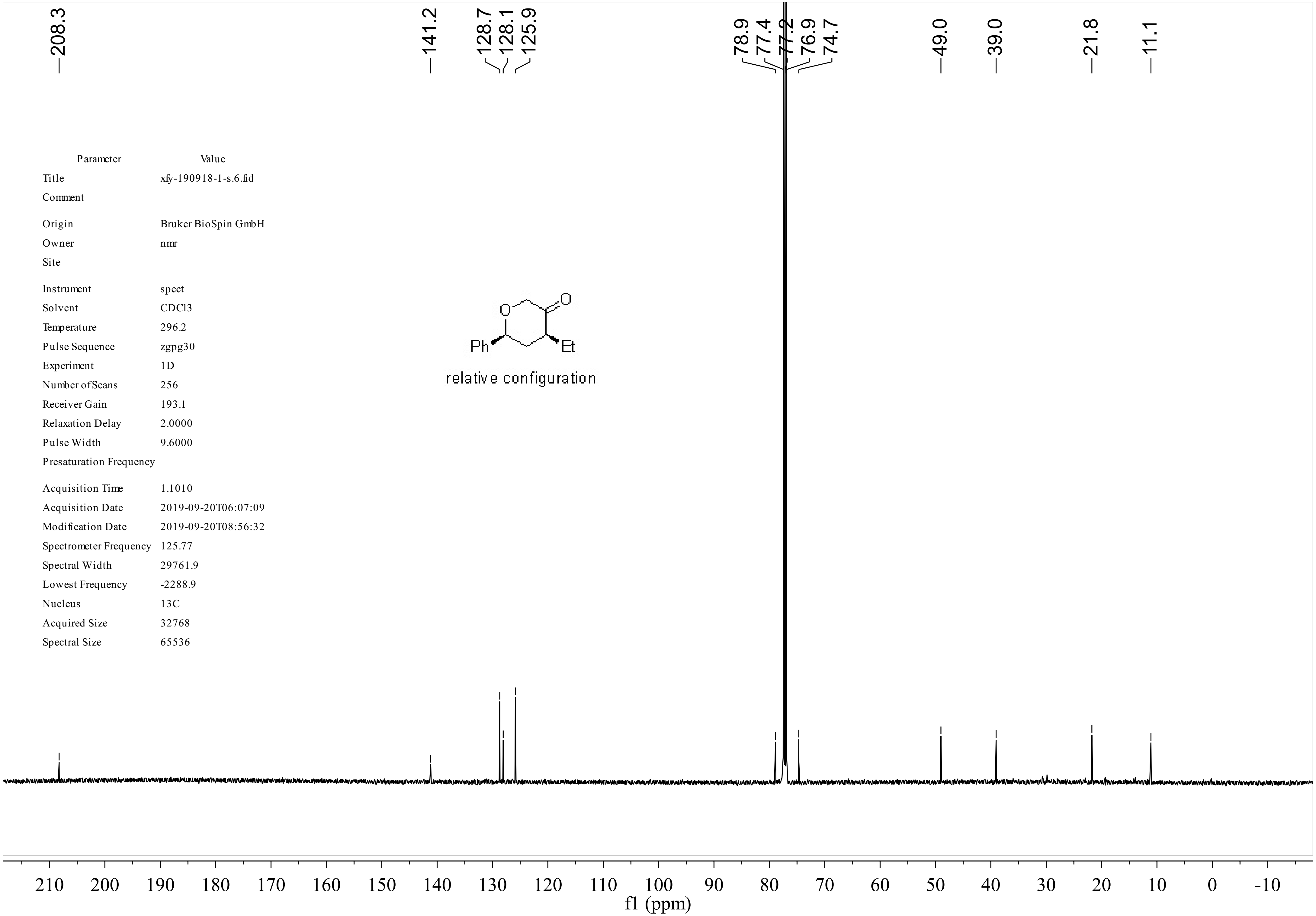

Parameter	Value
Title	xfy-190918-1-s.6.fid
Comment	
Origin	Bruker BioSpin GmbH
Owner	nmr
Site	
Instrument	spect
Solvent	CDCl3
Temperature	296.2
Pulse Sequence	zgpg30
Experiment	1D
Number of Scans	256
Receiver Gain	193.1
Relaxation Delay	2.0000
Pulse Width	9.6000
Presaturation Frequency	
Acquisition Time	1.1010
Acquisition Date	2019-09-20T06:07:09
Modification Date	2019-09-20T08:56:32
Spectrometer Frequency	125.77
Spectral Width	29761.9
Lowest Frequency	-2288.9
Nucleus	13C
Acquired Size	32768
Spectral Size	65536

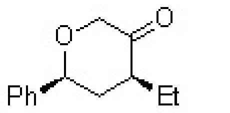

relative configuration

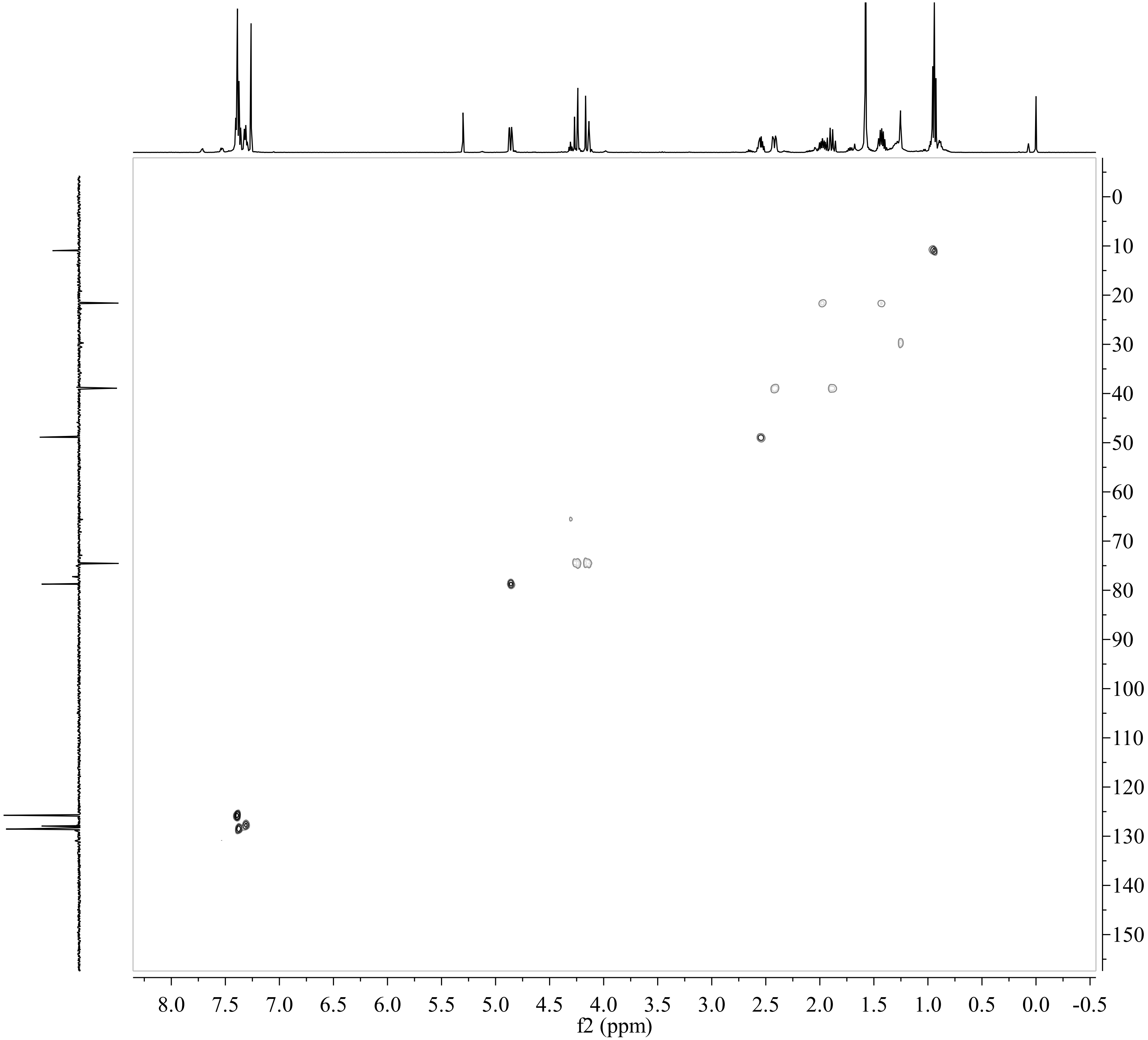

Parameter	Value
Title	xfy-190918-1-s.4.ser
Comment	
Origin	Bruker BioSpin GmbH
Owner	nmr
Site	
Instrument	spect
Solvent	CDCl3
Temperature	296.2
Pulse Sequence	hsqcedetgp
Experiment	HSQC-EDITED
Number of Scans	4
Receiver Gain	193.1
Relaxation Delay	1.4642
Pulse Width	10.7100
Presaturation Frequency	
Acquisition Time	0.1147
Acquisition Date	2019-09-20T02:28:09
Modification Date	2019-09-20T08:56:28
Spectrometer Frequency	(500.13, 125.77)
Spectral Width	(4464.3, 20833.3)
Lowest Frequency	(-286.1, -1037.0)
Nucleus	(1H, 13C)
Acquired Size	(512, 256)
Spectral Size	(512, 512)

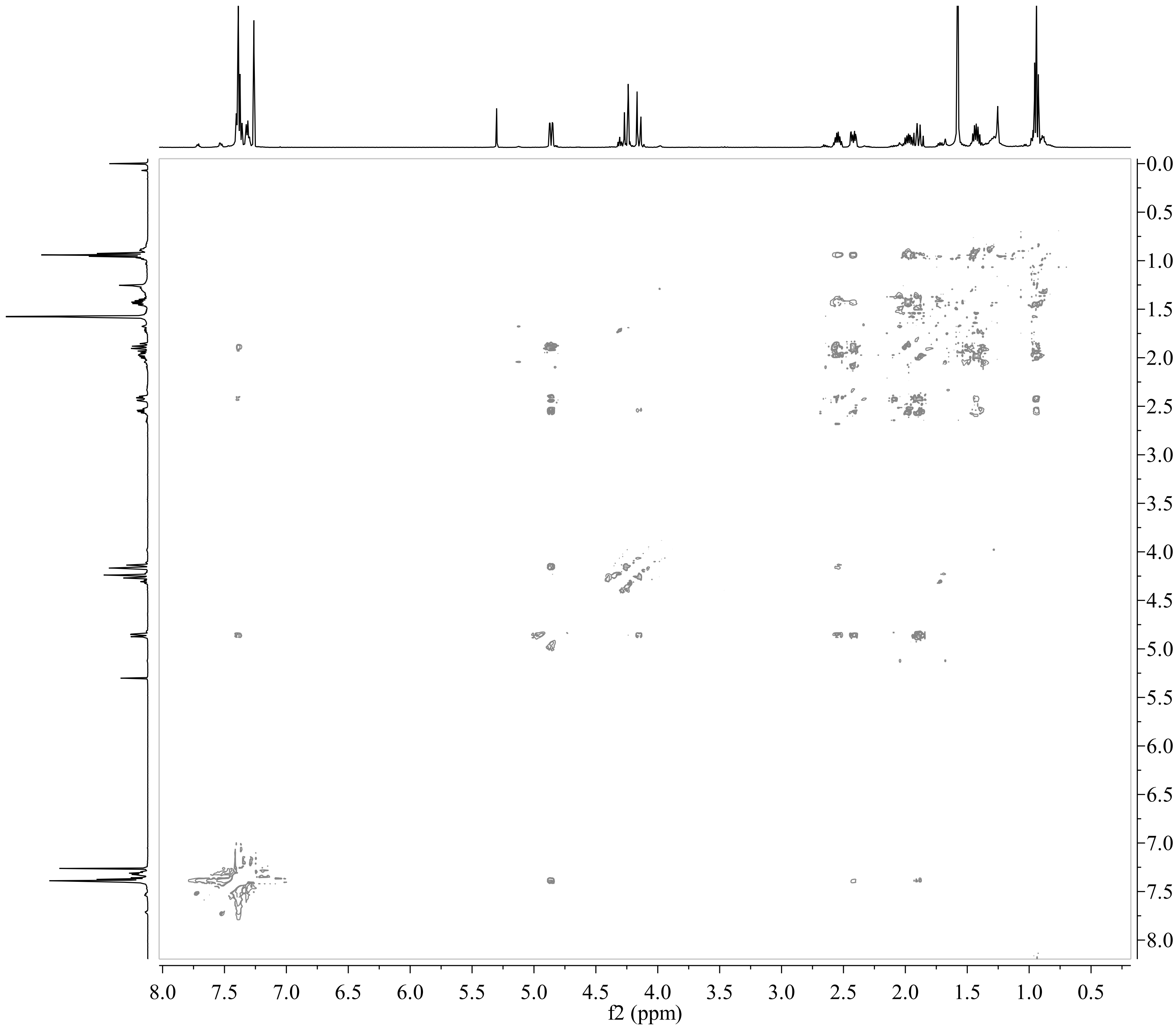

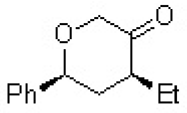

relative configuration

Parameter	Value
Title	xfy-190918-1-s.5.ser
Comment	
Origin	Bruker BioSpin GmbH
Owner	nmr
Site	
Instrument	spect
Solvent	CDCl3
Temperature	296.2
Pulse Sequence	noesygpphpp
Experiment	NOESY
Number of Scans	16
Receiver Gain	62.9
Relaxation Delay	1.9754
Pulse Width	10.7100
Presaturation Frequency	
Acquisition Time	0.2294
Acquisition Date	2019-09-20T02:58:18
Modification Date	2019-09-20T08:56:30
Spectrometer Frequency	(500.13, 500.13)
Spectral Width	(4464.3, 4464.3)
Lowest Frequency	(-286.1, -286.1)
Nucleus	(1H, 1H)
Acquired Size	(1024, 256)
Spectral Size	(1024, 1024)

Parameter	Value
Title	xfy-191027-1-s.31.fid
Comment	
Origin	Bruker BioSpin GmbH
Owner	nmr
Site	
Instrument	spect
Solvent	CDCl3
Temperature	296.2
Pulse Sequence	zg30
Experiment	1D
Number of Scans	16
Receiver Gain	87.5
Relaxation Delay	1.0000
Pulse Width	10.7100
Presaturation Frequency	
Acquisition Time	3.2768
Acquisition Date	2019-10-27T17:57:02
Modification Date	2019-10-27T18:31:48
Spectrometer Frequency	500.13
Spectral Width	10000.0
Lowest Frequency	-1924.3
Nucleus	1H
Acquired Size	32768
Spectral Size	65536

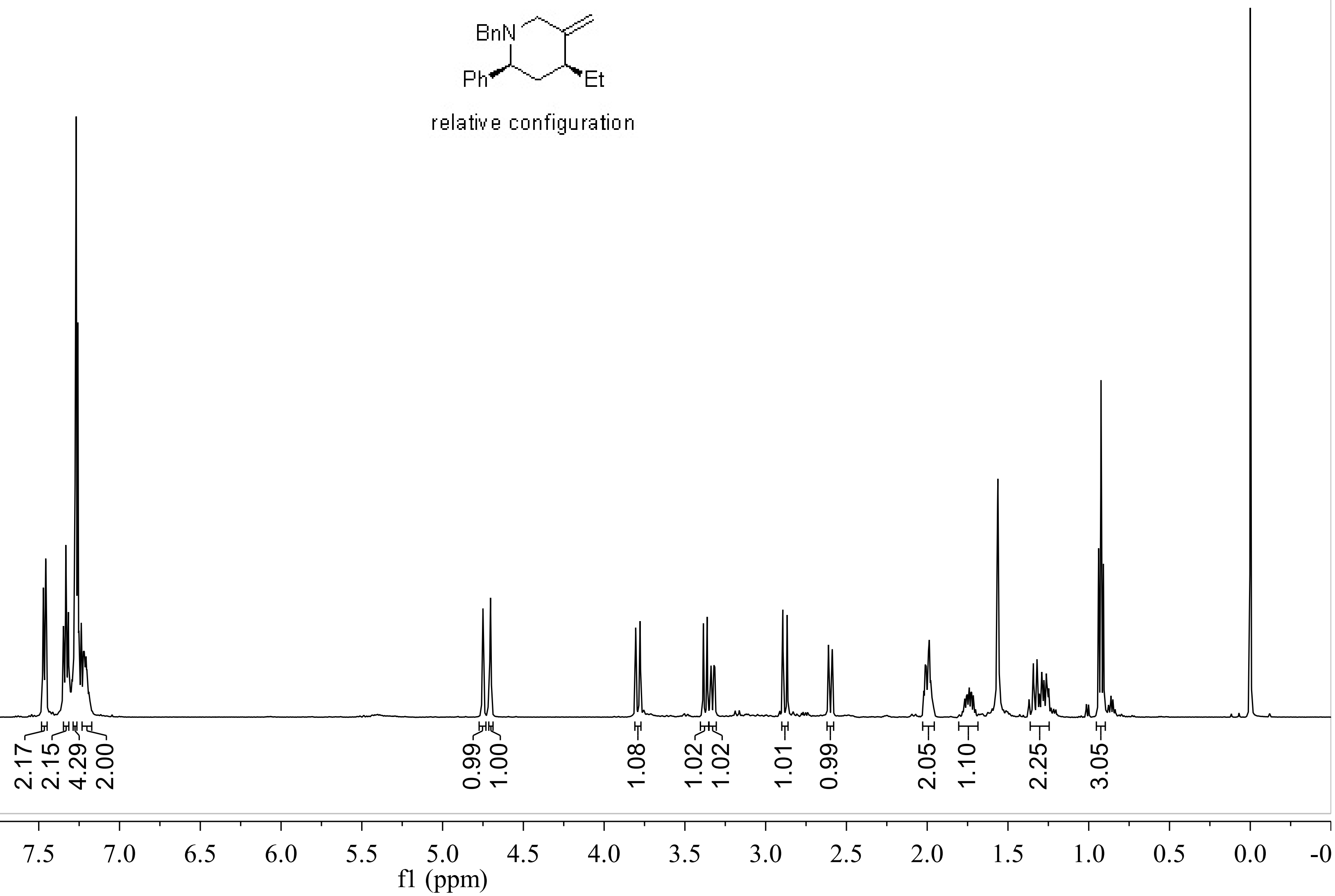

Parameter	Value
Title	xfy-191027-1-s.22.fid
Comment	
Origin	Bruker BioSpin GmbH
Owner	nmr
Site	
Instrument	spect
Solvent	CDCl3
Temperature	296.5
Pulse Sequence	zgpg30
Experiment	1D
Number of Scans	32
Receiver Gain	196.4
Relaxation Delay	2.0000
Pulse Width	10.0000
Presaturation Frequency	
Acquisition Time	1.3631
Acquisition Date	2019-10-26T22:31:18
Modification Date	2019-10-27T16:03:48
Spectrometer Frequency	100.62
Spectral Width	24038.5
Lowest Frequency	-1946.7
Nucleus	13C
Acquired Size	32768
Spectral Size	65536

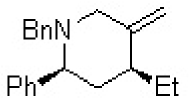

relative configuration

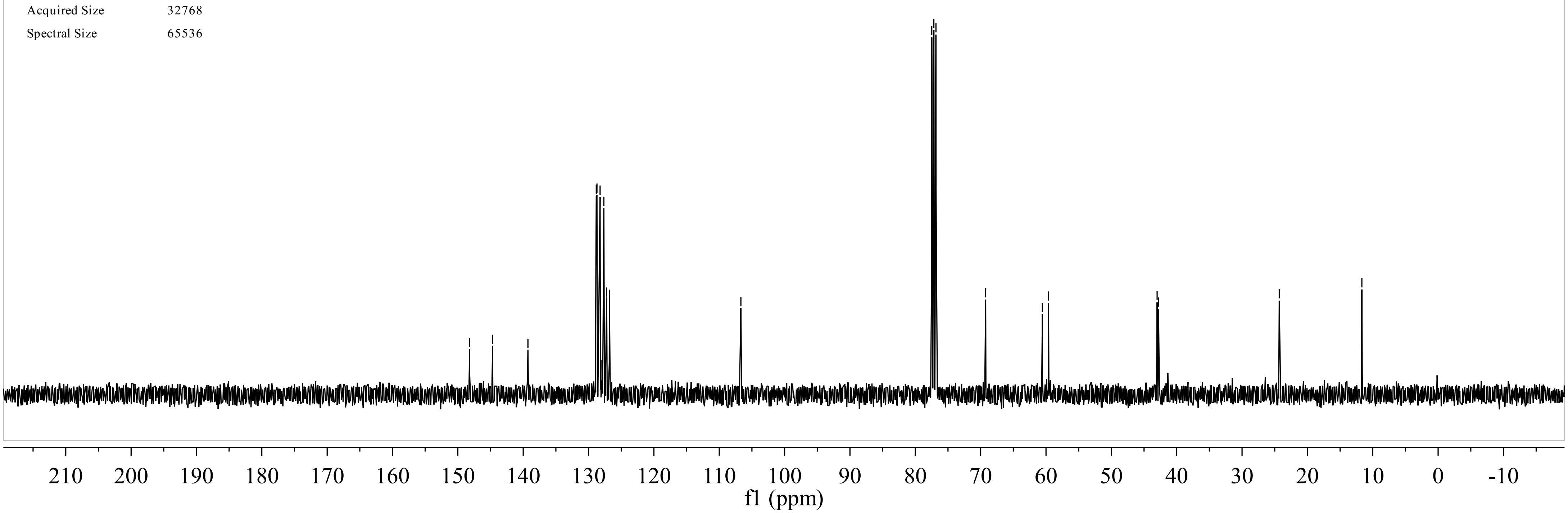

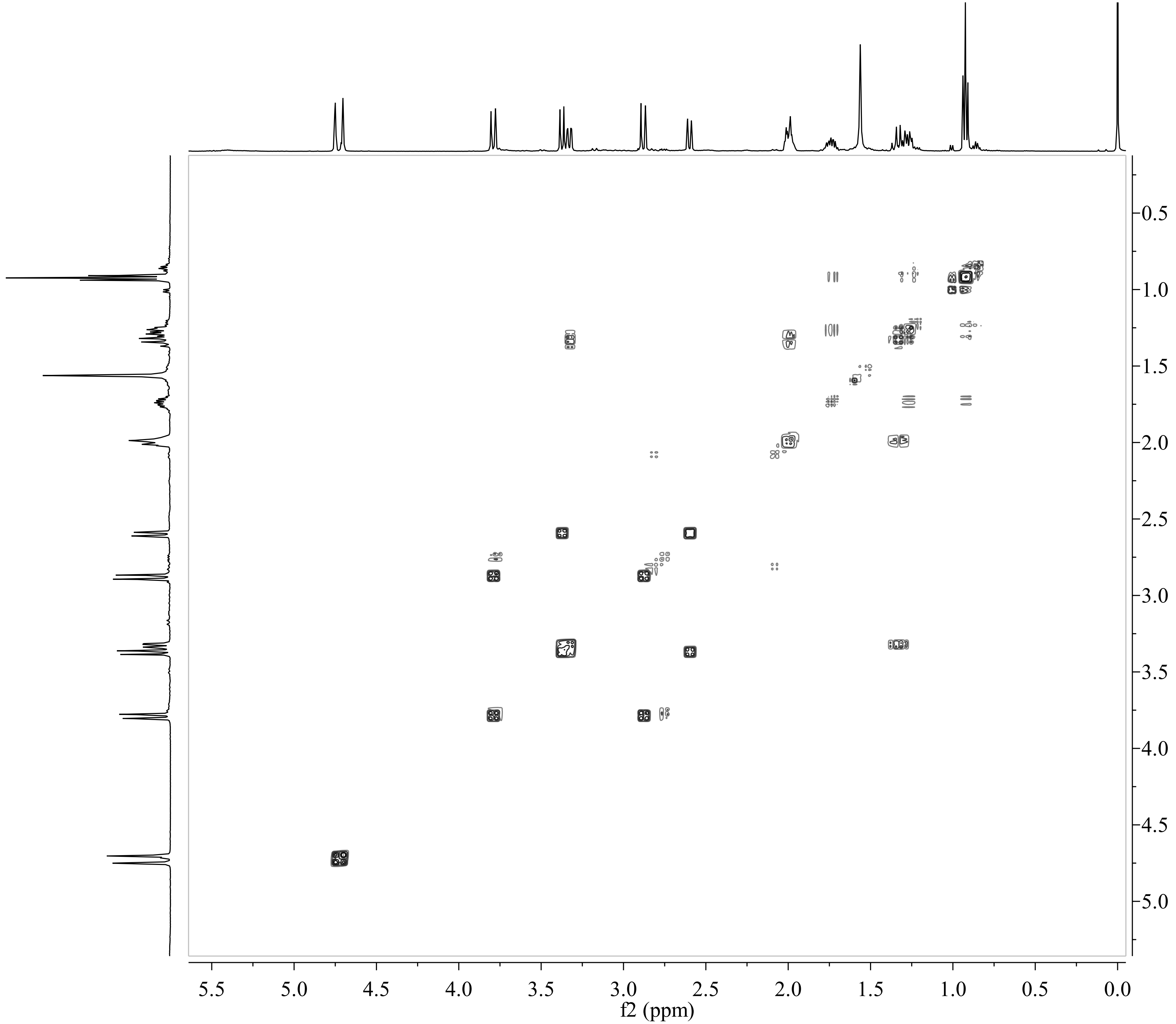

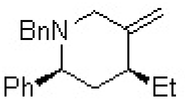

relative configuration

Parameter	Value
Title	xfy-191027-1-s.24.ser
Comment	
Origin	Bruker BioSpin GmbH
Owner	nmr
Site	
Instrument	spect
Solvent	CDCl3
Temperature	296.4
Pulse Sequence	cosygpppqf
Experiment	COSY
Number of Scans	2
Receiver Gain	34.9
Relaxation Delay	1.9050
Pulse Width	8.7300
Presaturation Frequency	
Acquisition Time	0.2867
Acquisition Date	2019-10-26T22:36:28
Modification Date	2019-10-27T16:03:48
Spectrometer Frequency	(400.13, 400.13)
Spectral Width	(3571.4, 3571.4)
Lowest Frequency	(-216.6, -216.6)
Nucleus	(1H, 1H)
Acquired Size	(1024, 128)
Spectral Size	(1024, 1024)

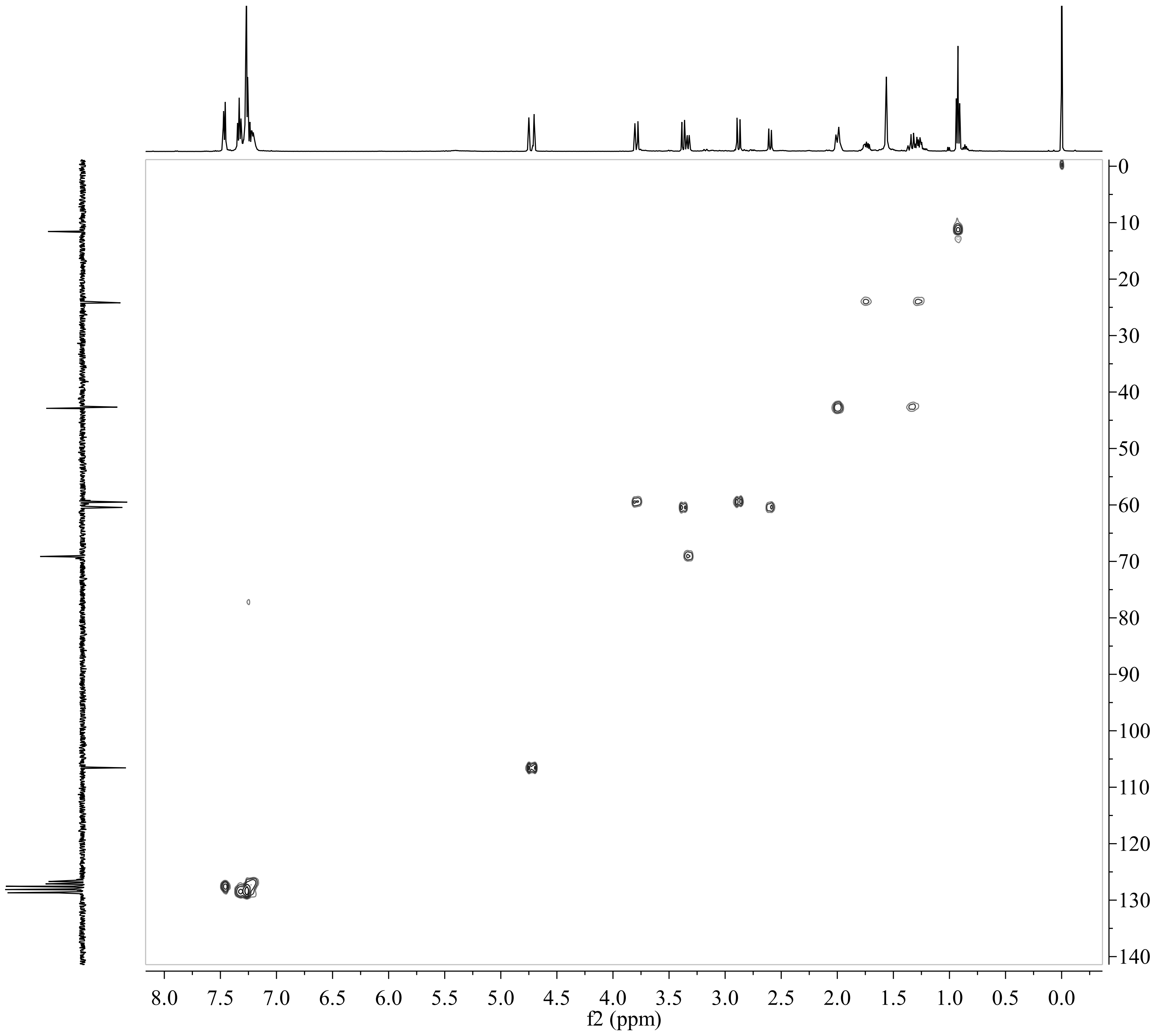

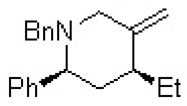

relative configuration

Parameter	Value
Title	xfy-191027-1-s.43.ser
Comment	
Origin	Bruker BioSpin GmbH
Owner	nmr
Site	
Instrument	spect
Solvent	CDCl3
Temperature	296.8
Pulse Sequence	hsqcetgp
Experiment	HSQC
Number of Scans	2
Receiver Gain	196.4
Relaxation Delay	1.4623
Pulse Width	8.7300
Presaturation Frequency	
Acquisition Time	0.1362
Acquisition Date	2019-10-27T18:57:02
Modification Date	2019-10-27T19:22:25
Spectrometer Frequency	(400.13, 100.62)
Spectral Width	(3759.4, 16666.7)
Lowest Frequency	(-419.9, -829.1)
Nucleus	(1H, 13C)
Acquired Size	(512, 256)
Spectral Size	(512, 512)

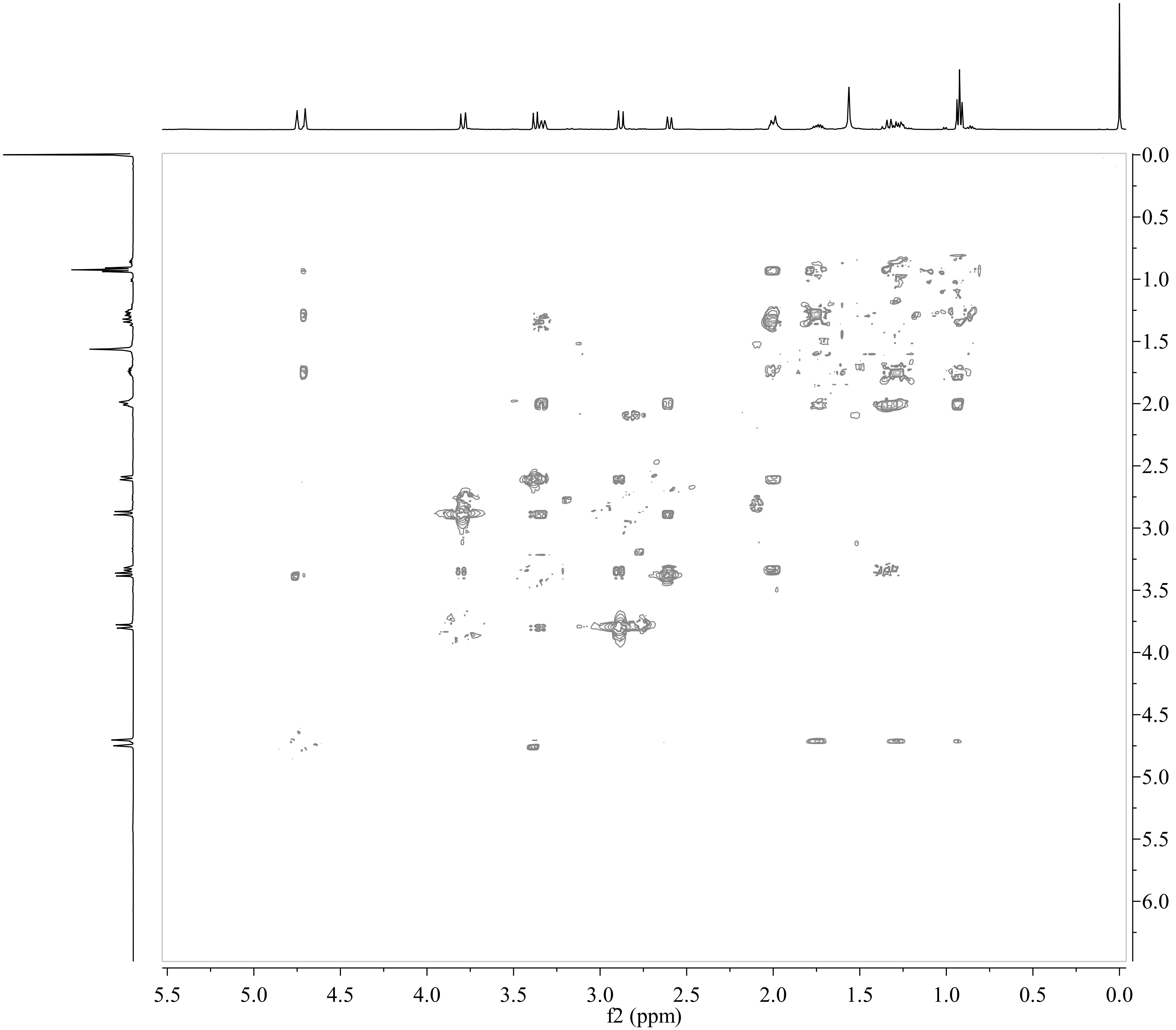

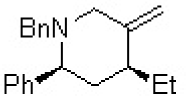

relative configuration

Parameter	Value
Title	xfy-191027-1-s.25.ser
Comment	
Origin	Bruker BioSpin GmbH
Owner	nmr
Site	
Instrument	spect
Solvent	CDCl3
Temperature	296.2
Pulse Sequence	noesygpphpp
Experiment	NOESY
Number of Scans	12
Receiver Gain	34.9
Relaxation Delay	1.9693
Pulse Width	8.7300
Presaturation Frequency	
Acquisition Time	0.2867
Acquisition Date	2019-10-26T22:48:45
Modification Date	2019-10-27T16:03:48
Spectrometer Frequency	(400.13, 400.13)
Spectral Width	(3571.4, 3571.4)
Lowest Frequency	(-230.6, -234.2)
Nucleus	(1H, 1H)
Acquired Size	(1024, 256)
Spectral Size	(1024, 1024)

Parameter	Value
标题	xfy-190629-4.1.fid
Comment	
Origin	Bruker BioSpin GmbH
Owner	nmr
Site	
Instrument	spect
Author	
Solvent	CDCl3
Temperature	296.2
Pulse Sequence	zg30
Experiment	1D
Number of Scans	8
Receiver Gain	108.2
Relaxation Delay	1.0000
Pulse Width	10.7100
Presaturation Frequency	
Acquisition Time	3.2768
Class	
Spectrometer Frequency	500.13
Spectral Width	10000.0
Lowest Frequency	-1923.5
Nucleus	1H
Acquired Size	32768
Spectral Size	65536

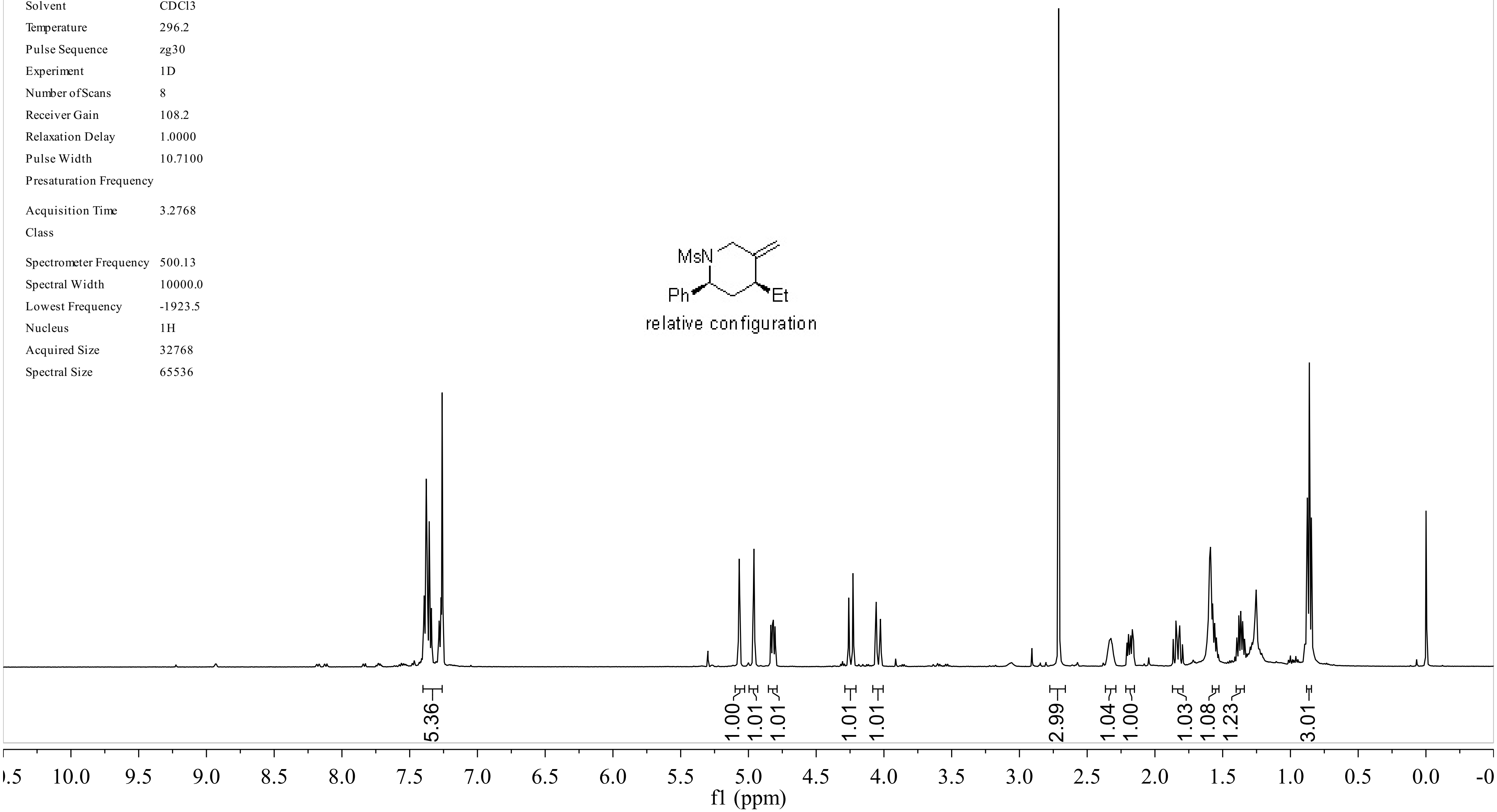

Parameter	Value
Title	xfy-190629-4.2.fid
Comment	
Origin	Bruker BioSpin GmbH
Owner	nmr
Site	
Instrument	spect
Solvent	CDCl3
Temperature	296.2
Pulse Sequence	zgpg30
Experiment	1D
Number of Scans	96
Receiver Gain	193.1
Relaxation Delay	2.0000
Pulse Width	9.6000
Presaturation Frequency	
Acquisition Time	1.1010
Acquisition Date	2019-06-29T21:40:05
Modification Date	2019-07-01T22:36:30
Spectrometer Frequency	125.77
Spectral Width	29761.9
Lowest Frequency	-2305.8
Nucleus	13C
Acquired Size	32768
Spectral Size	65536

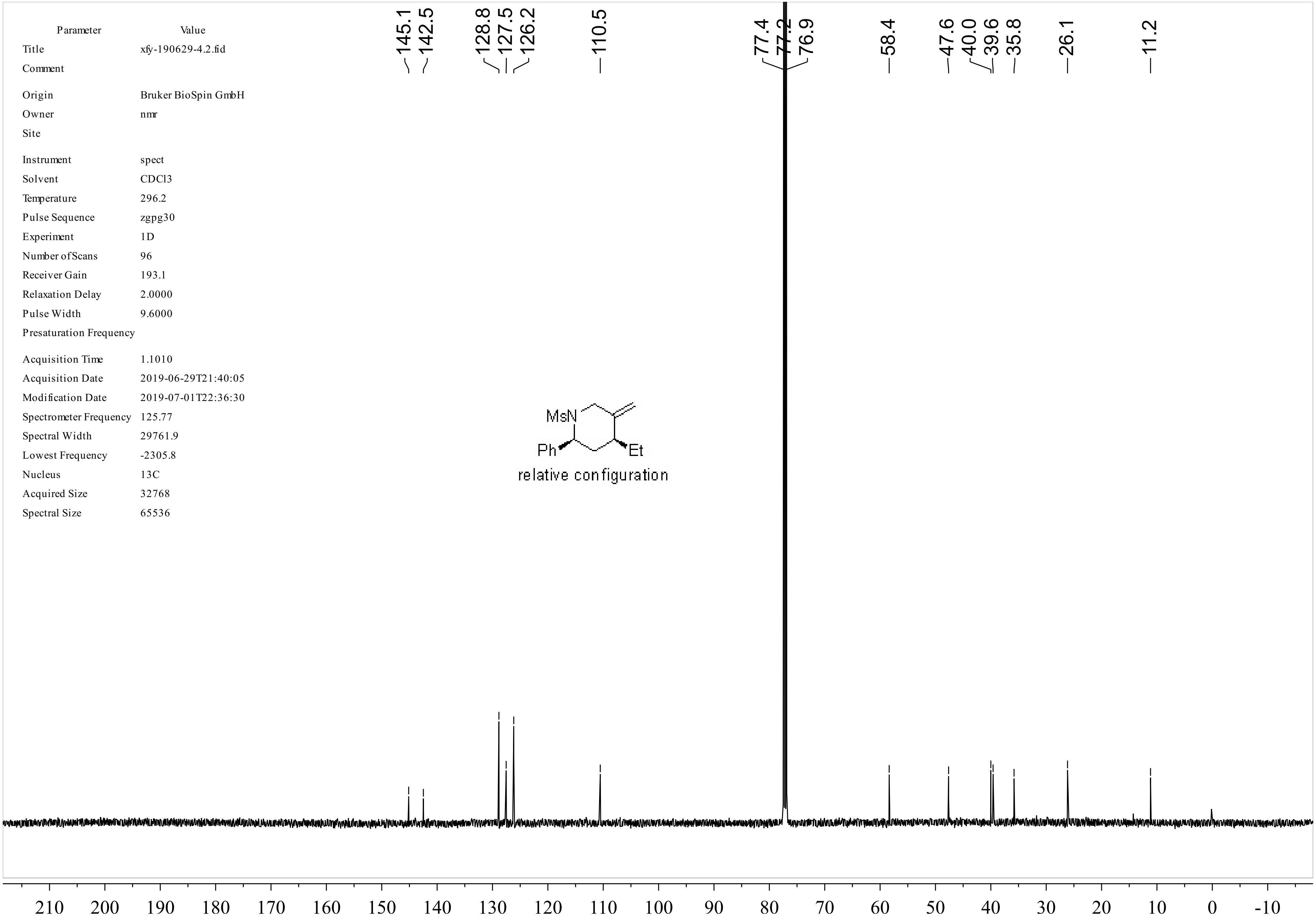

relative configuration

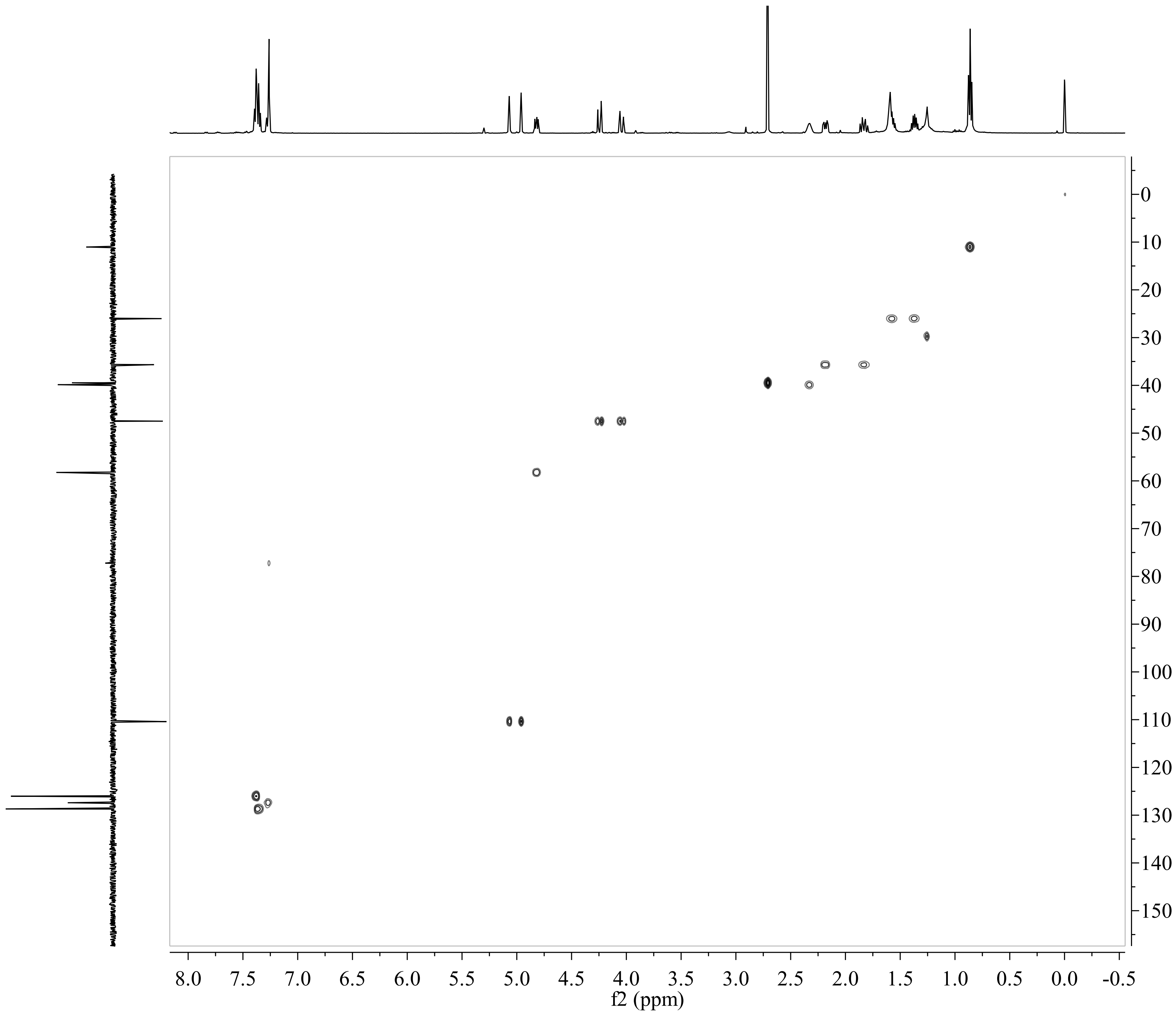

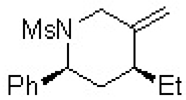

relative configuration

Parameter	Value
Title	xfy-190629-4.44.ser
Comment	
Origin	Bruker BioSpin GmbH
Owner	nmr
Site	
Instrument	spect
Solvent	CDCl3
Temperature	296.9
Pulse Sequence	hsqcetgp
Experiment	HSQC
Number of Scans	8
Receiver Gain	196.4
Relaxation Delay	1.4521
Pulse Width	8.7300
Presaturation Frequency	
Acquisition Time	0.1464
Acquisition Date	2019-07-02T06:24:11
Modification Date	2019-07-02T08:43:04
Spectrometer Frequency	(400.13, 100.62)
Spectral Width	(3496.5, 16666.7)
Lowest Frequency	(-228.2, -829.1)
Nucleus	(1H, 13C)
Acquired Size	(512, 256)
Spectral Size	(512, 512)

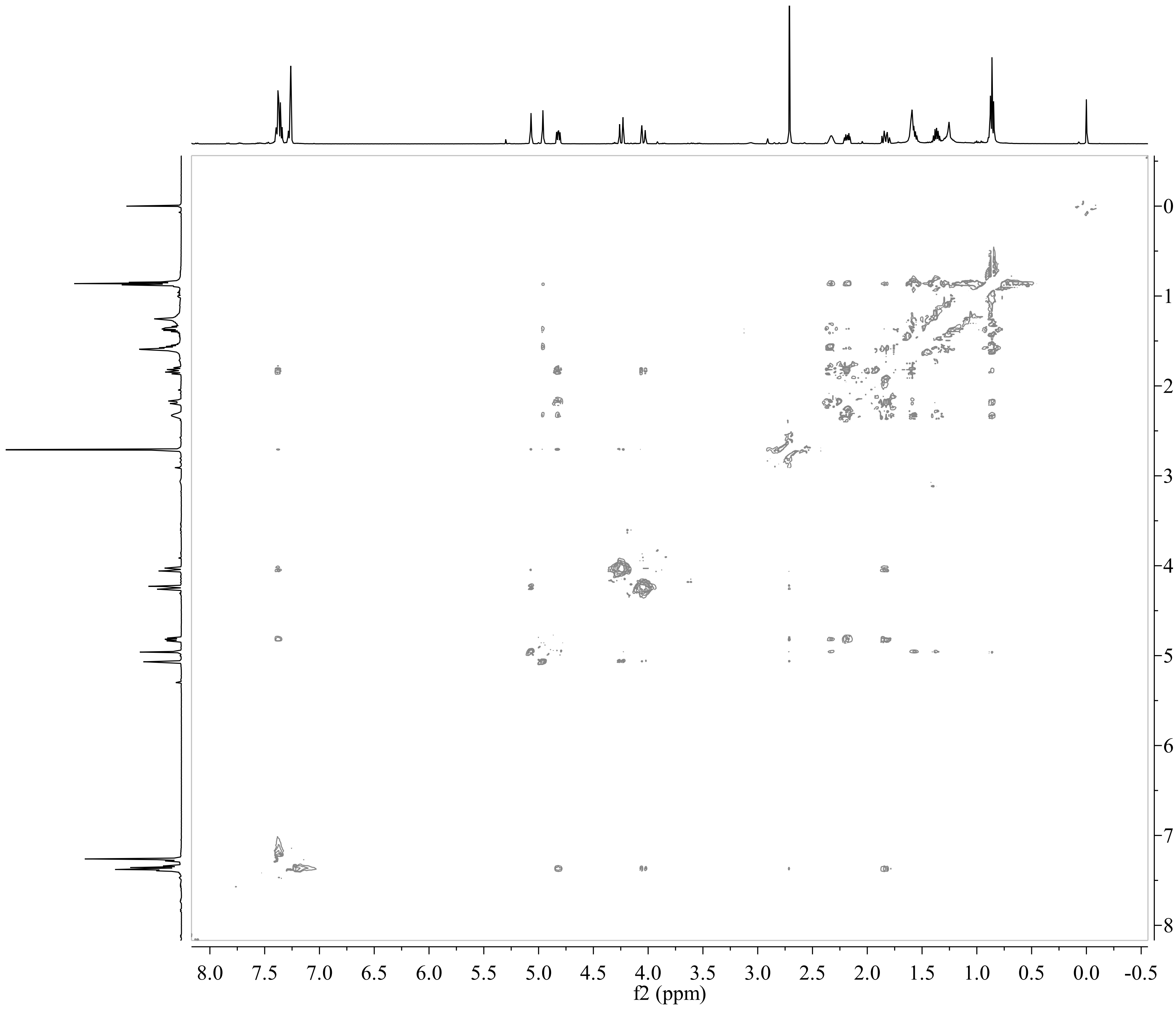

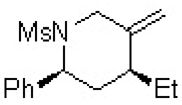

relative configuration

Parameter	Value
Title	xfy-190629-4.42.ser
Comment	
Origin	Bruker BioSpin GmbH
Owner	nmr
Site	
Instrument	spect
Solvent	CDCl3
Temperature	296.6
Pulse Sequence	noesygpphpp
Experiment	NOESY
Number of Scans	24
Receiver Gain	62.3
Relaxation Delay	1.9631
Pulse Width	8.7300
Presaturation Frequency	
Acquisition Time	0.2929
Acquisition Date	2019-07-02T01:39:54
Modification Date	2019-07-02T08:43:03
Spectrometer Frequency	(400.13, 400.13)
Spectral Width	(3496.5, 3496.5)
Lowest Frequency	(-228.2, -228.2)
Nucleus	(1H, 1H)
Acquired Size	(1024, 256)
Spectral Size	(1024, 1024)

Parameter	Value
Origin	Bruker BioSpin GmbH
Spectrometer	spect
Solvent	CDCl3
Temperature	295.9
Pulse Sequence	zg30
Experiment	1D
Number of Scans	8
Receiver Gain	111
Relaxation Delay	1.0000
Pulse Width	10.0000
Acquisition Time	1.9999
Spectrometer Frequency	400.13
Spectral Width	8012.8
Lowest Frequency	-1538.9
Nucleus	1H
Acquired Size	16025
Spectral Size	65536

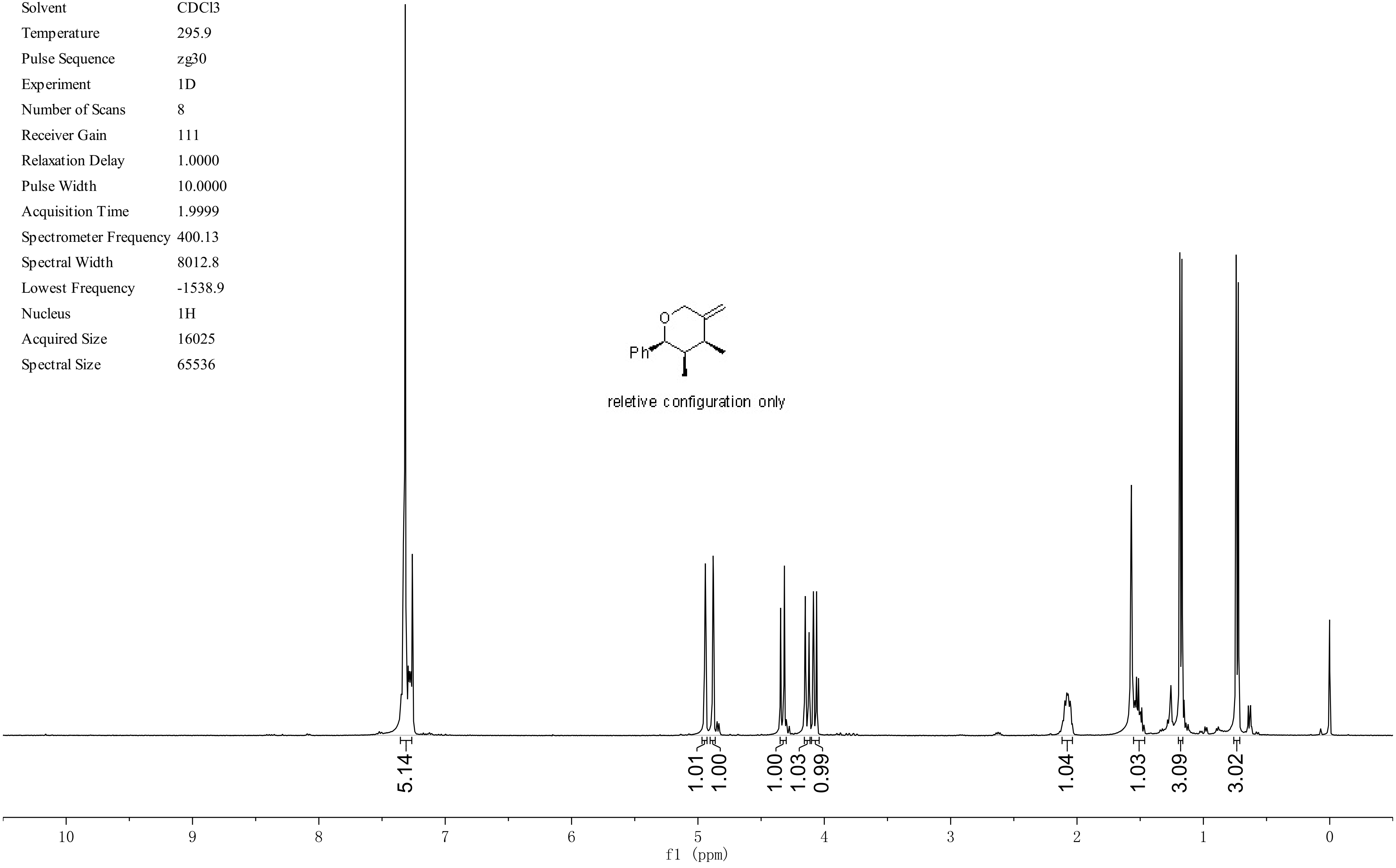

Parameter	Value
Origin	Bruker BioSpin GmbH
Spectrometer	spect
Solvent	CDCl3
Temperature	296.0
Pulse Sequence	zgpg30
Experiment	1D
Number of Scans	600
Receiver Gain	196
Relaxation Delay	2.0000
Pulse Width	10.0000
Acquisition Time	1.3631
Spectrometer Frequency	100.62
Spectral Width	24038.5
Lowest Frequency	-1928.6
Nucleus	13C
Acquired Size	32768
Spectral Size	65536

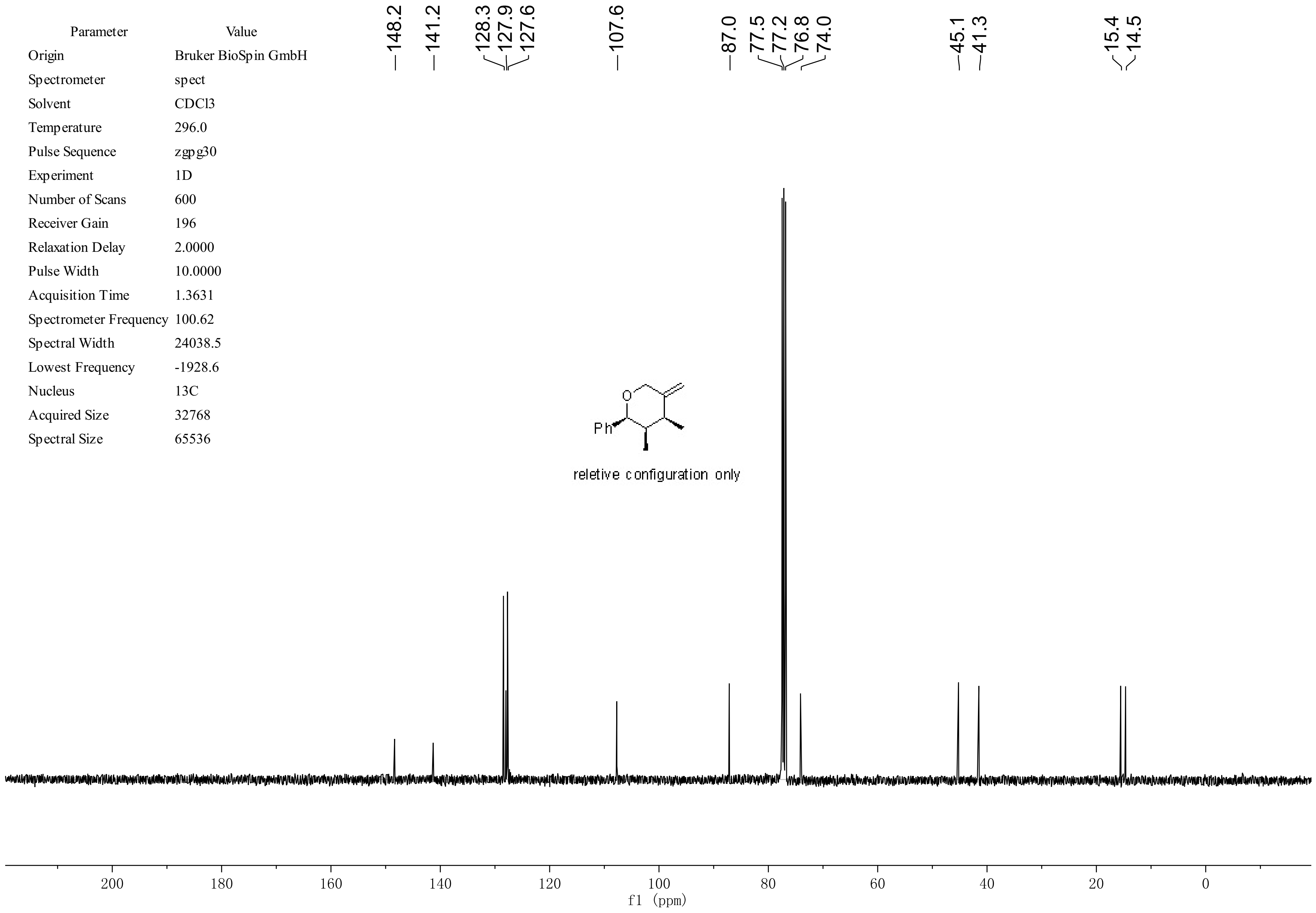

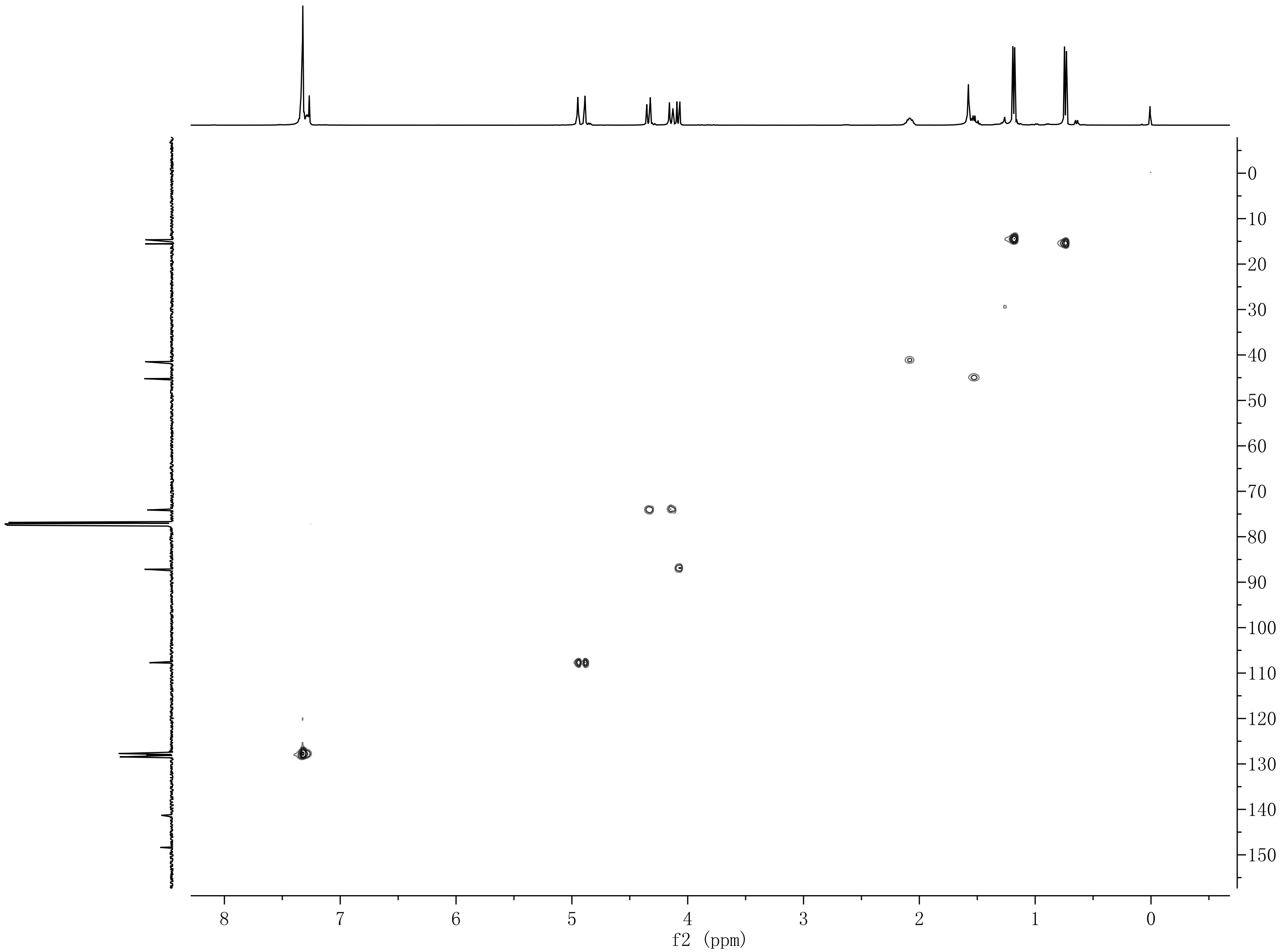

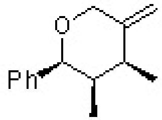

reletive configuration only

Parameter	Value
Origin	Bruker BioSpin GmbH
Spectrometer	spect
Solvent	CDCl3
Temperature	294.9
Pulse Sequence	hsqcetgp
Experiment	HSQC
Number of Scans	2
Receiver Gain	196
Relaxation Delay	1.4562
Pulse Width	10.0000
Acquisition Time	0.1423
Spectrometer Frequency	(400.13, 100.62)
Spectral Width	(3597.1, 16666.7)
Lowest Frequency	(-279.7, -829.1)
Nucleus	(1H, 13C)
Acquired Size	(512, 256)

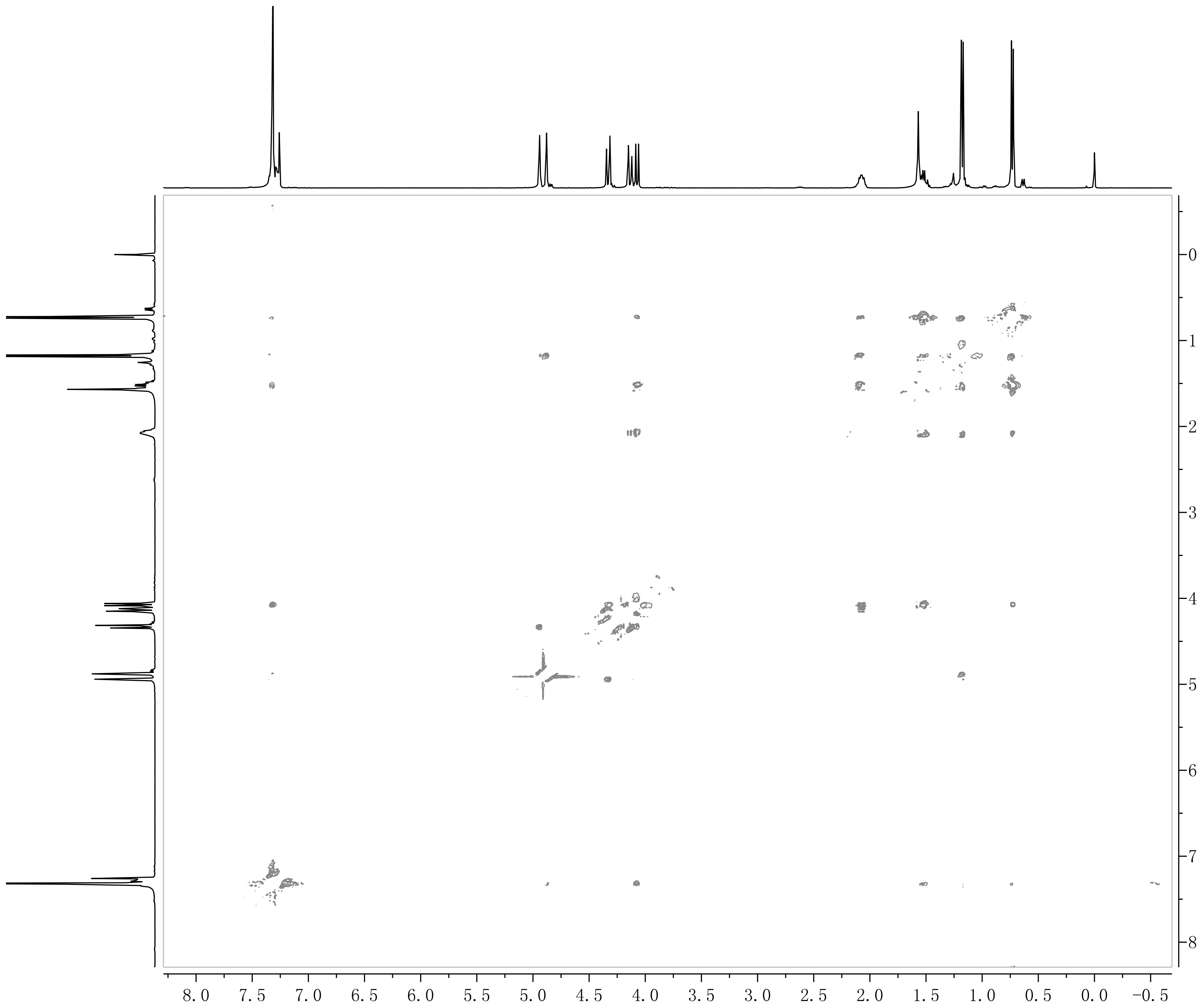

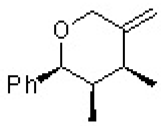

reletive configuration only

Parameter	Value
Title	gvv-e-76-2f-0730.12.ser
Comment	
Origin	Bruker BioSpin GmbH
Owner	nmr
Site	
Instrument	spect
Solvent	CDCl3
Temperature	295.7
Pulse Sequence	noesygpphpp
Experiment	NOESY
Number of Scans	4
Receiver Gain	34.9
Relaxation Delay	1.9713
Pulse Width	10.0000
Presaturation Frequency	
Acquisition Time	0.2847
Acquisition Date	2018-07-31T07:11:48
Modification Date	2019-04-22T11:11:27
Spectrometer Frequency	(400.13, 400.13)
Spectral Width	(3597.1, 3597.1)
Lowest Frequency	(-279.7, -279.7)
Nucleus	(1H, 1H)
Acquired Size	(1024, 256)
Spectral Size	(1024, 1024)

Parameter	Value
Origin	Bruker BioSpin GmbH
Spectrometer	spect
Solvent	CDCl3
Temperature	295.9
Pulse Sequence	zg30
Experiment	1D
Number of Scans	8
Receiver Gain	40
Relaxation Delay	1.0000
Pulse Width	10.0000
Acquisition Time	1.9999
Spectrometer Frequency	400.13
Spectral Width	8012.8
Lowest Frequency	-1548.1
Nucleus	1H
Acquired Size	16025
Spectral Size	65536

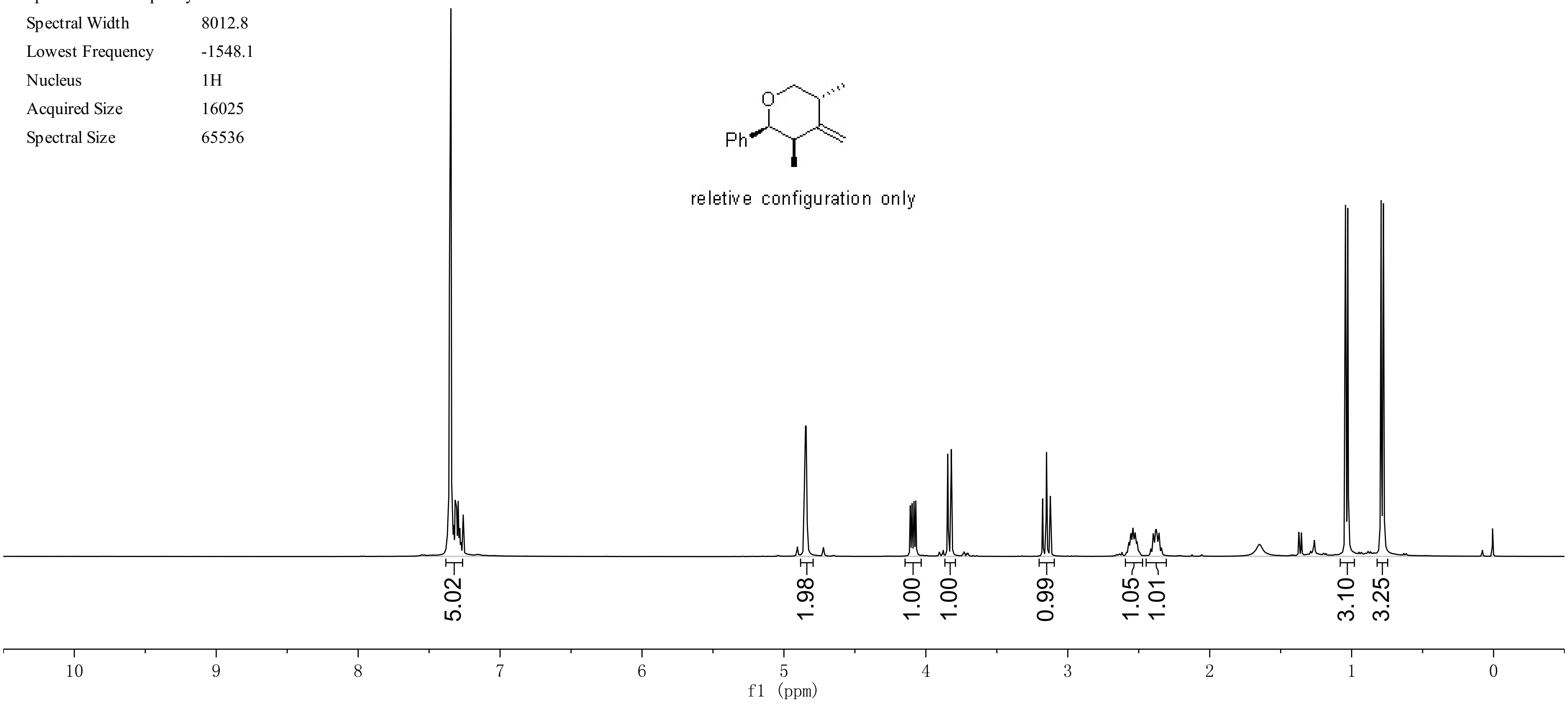

Parameter	Value
Origin	Bruker BioSpin GmbH
Spectrometer	spect
Solvent	CDCl3
Temperature	296.1
Pulse Sequence	zgpg30
Experiment	1D
Number of Scans	200
Receiver Gain	193
Relaxation Delay	2.0000
Pulse Width	9.6000
Acquisition Time	1.1010
Spectrometer Frequency	125.77
Spectral Width	29761.9
Lowest Frequency	-2277.1
Nucleus	13C
Acquired Size	32768
Spectral Size	65536

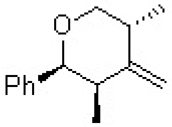

reletive configuration only

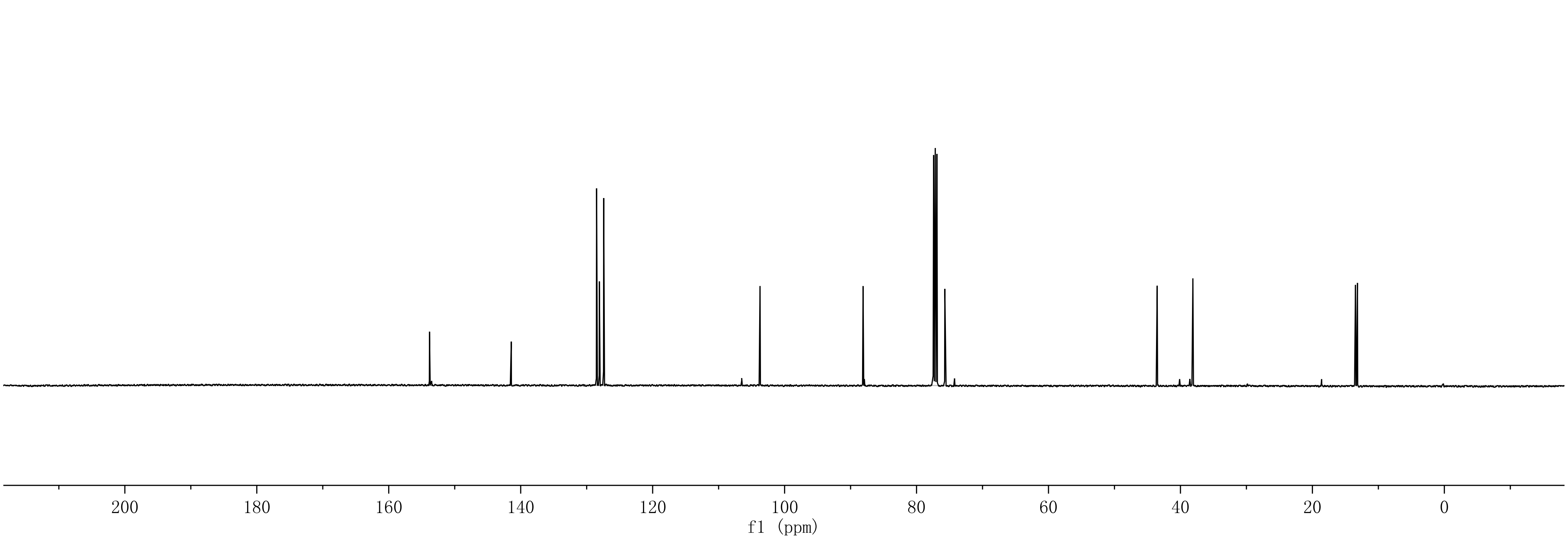

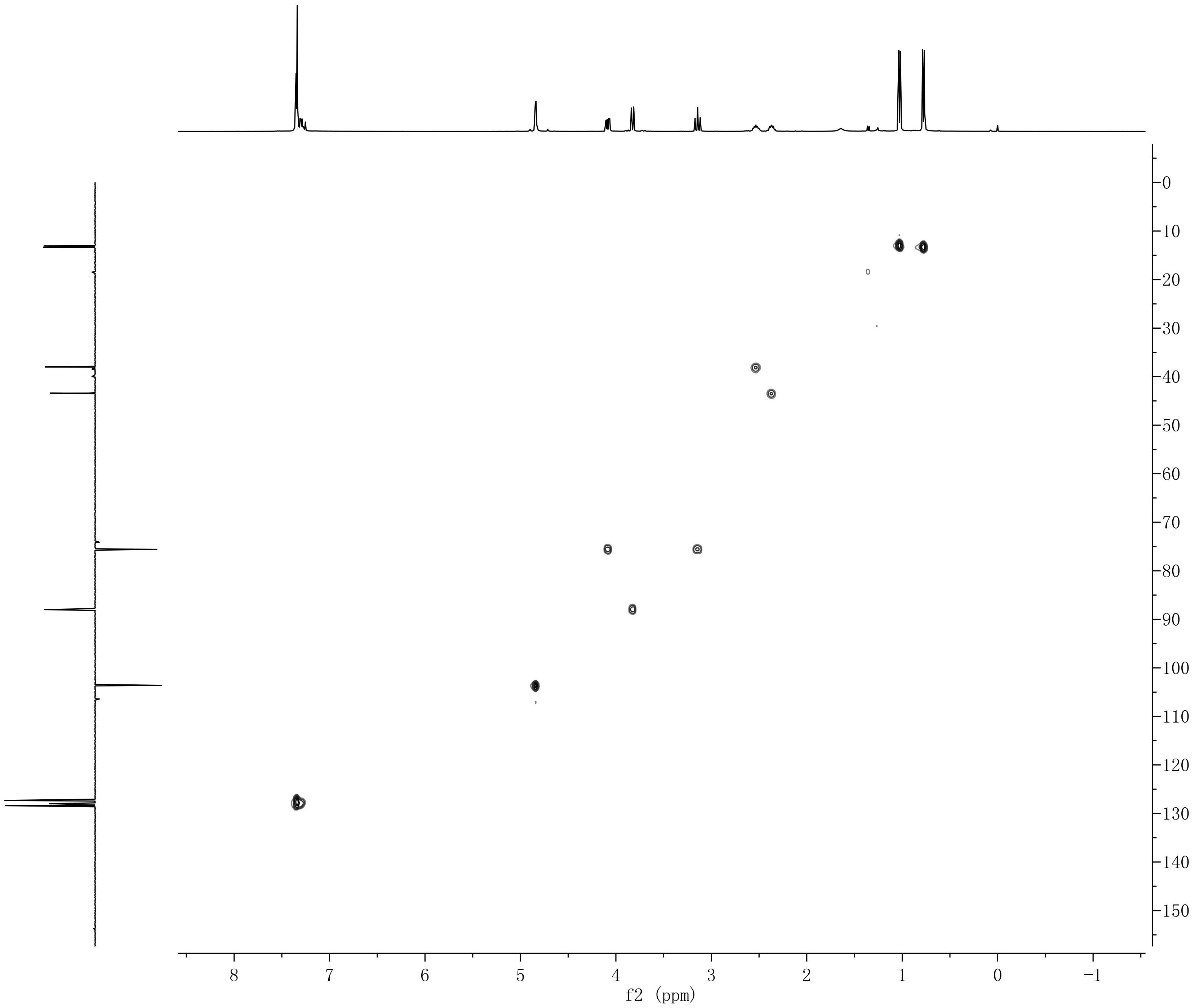

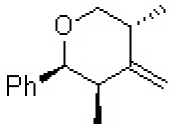

reletive configuration only

Parameter	Value
Origin	Bruker BioSpin GmbH
Spectrometer	spect
Solvent	CDCl3
Temperature	296.0
Pulse Sequence	hsqcetgp
Experiment	HSQC
Number of Scans	2
Receiver Gain	196
Relaxation Delay	1.4726
Pulse Width	10.0000
Acquisition Time	0.1260
Spectrometer Frequency	(400.13, 100.62)
Spectral Width	(4065.0, 16666.7)
Lowest Frequency	(-627.9, -829.1)
Nucleus	(1H, 13C)
Acquired Size	(512, 256)
Spectral Size	(512, 512)

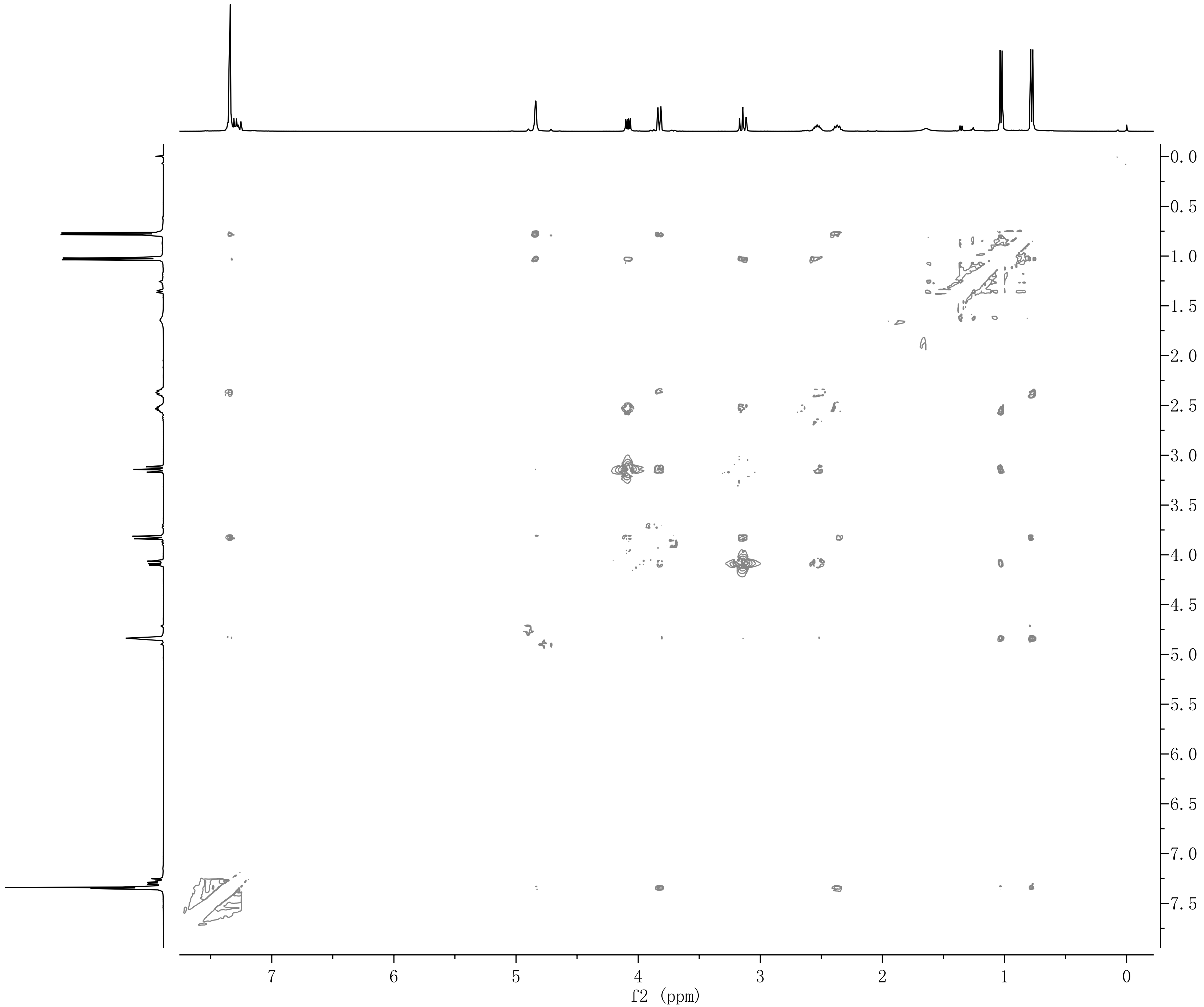

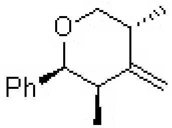

reletive configuration only

Parameter	Value
Origin	Bruker BioSpin GmbH
Spectrometer	spect
Solvent	CDCl3
Temperature	296.4
Pulse Sequence	noesygpphpp
Experiment	NOESY
Number of Scans	4
Receiver Gain	46
Relaxation Delay	2.0041
Pulse Width	10.0000
Acquisition Time	0.2519
Spectrometer Frequency	(400.13, 400.13)
Spectral Width	(4065.0, 4065.0)
Lowest Frequency	(-627.9, -627.9)
Nucleus	(1H, 1H)
Acquired Size	(1024, 256)
Spectral Size	(1024, 1024)

Parameter	Value
Title	xfy-190413-1-s.21.1.1r
Comment	
Origin	Bruker BioSpin GmbH
Owner	nmr
Site	
Instrument	spect
Solvent	CDCl3
Temperature	296.1
Pulse Sequence	zg30
Experiment	1D
Number of Scans	4
Receiver Gain	87.5
Relaxation Delay	1.0000
Pulse Width	10.7100
Presaturation Frequency	
Acquisition Time	3.2768
Acquisition Date	2019-04-14T12:12:41
Modification Date	2019-04-14T13:18:12
Spectrometer Frequency	500.13
Spectral Width	10000.0
Lowest Frequency	-1923.0
Nucleus	1H
Acquired Size	32768
Spectral Size	65536

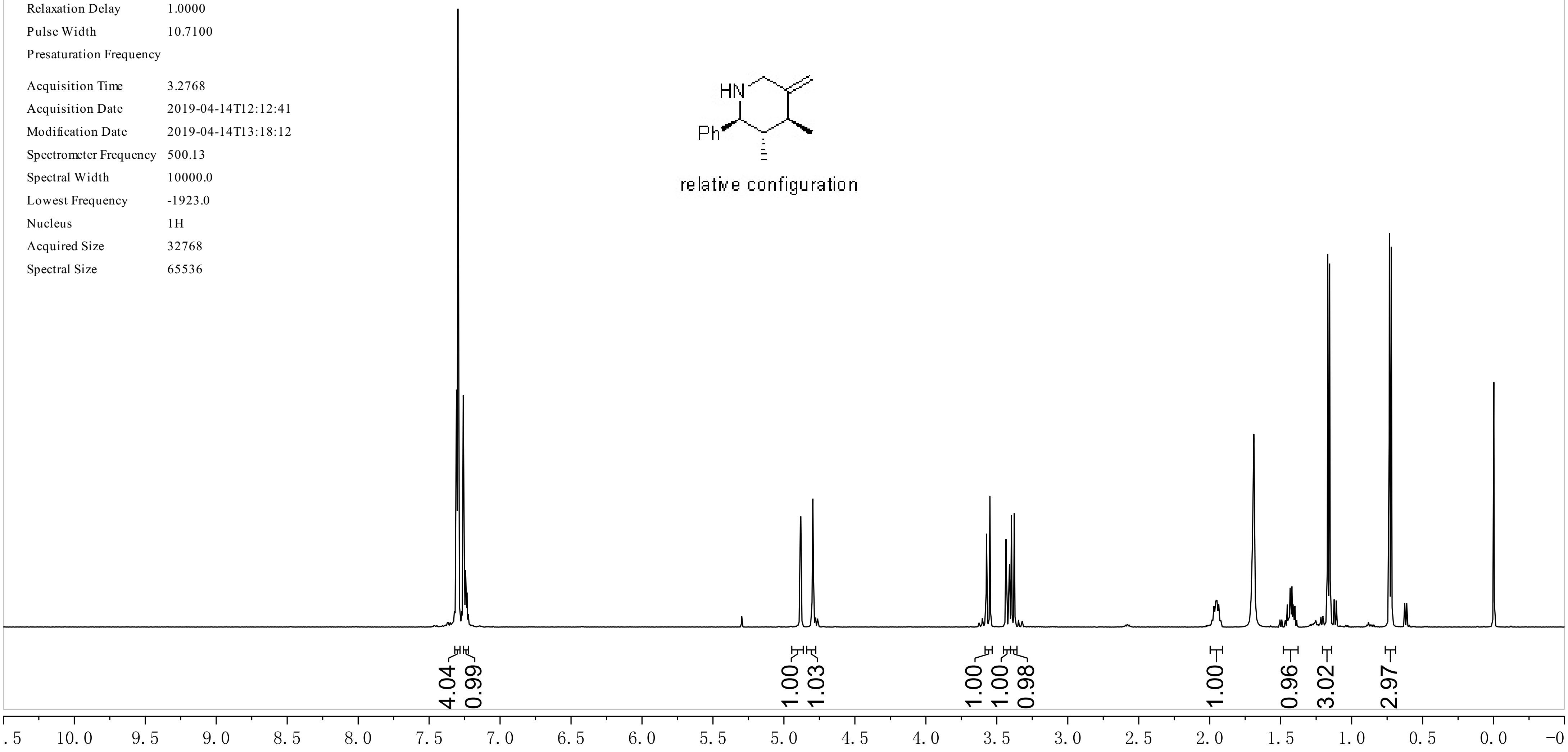

Parameter	Value
Title	xfy-190329-1-s.17.fid
Comment	
Origin	Bruker BioSpin GmbH
Owner	nmr
Site	
Instrument	spect
Solvent	CDCl3
Temperature	296.2
Pulse Sequence	zgpg30
Experiment	1D
Number of Scans	24
Receiver Gain	193.1
Relaxation Delay	2.0000
Pulse Width	9.6000
Presaturation Frequency	
Acquisition Time	1.1010
Class	
Spectrometer Frequency	125.77
Spectral Width	29761.9
Lowest Frequency	-2305.8
Nucleus	13C
Acquired Size	32768
Spectral Size	65536

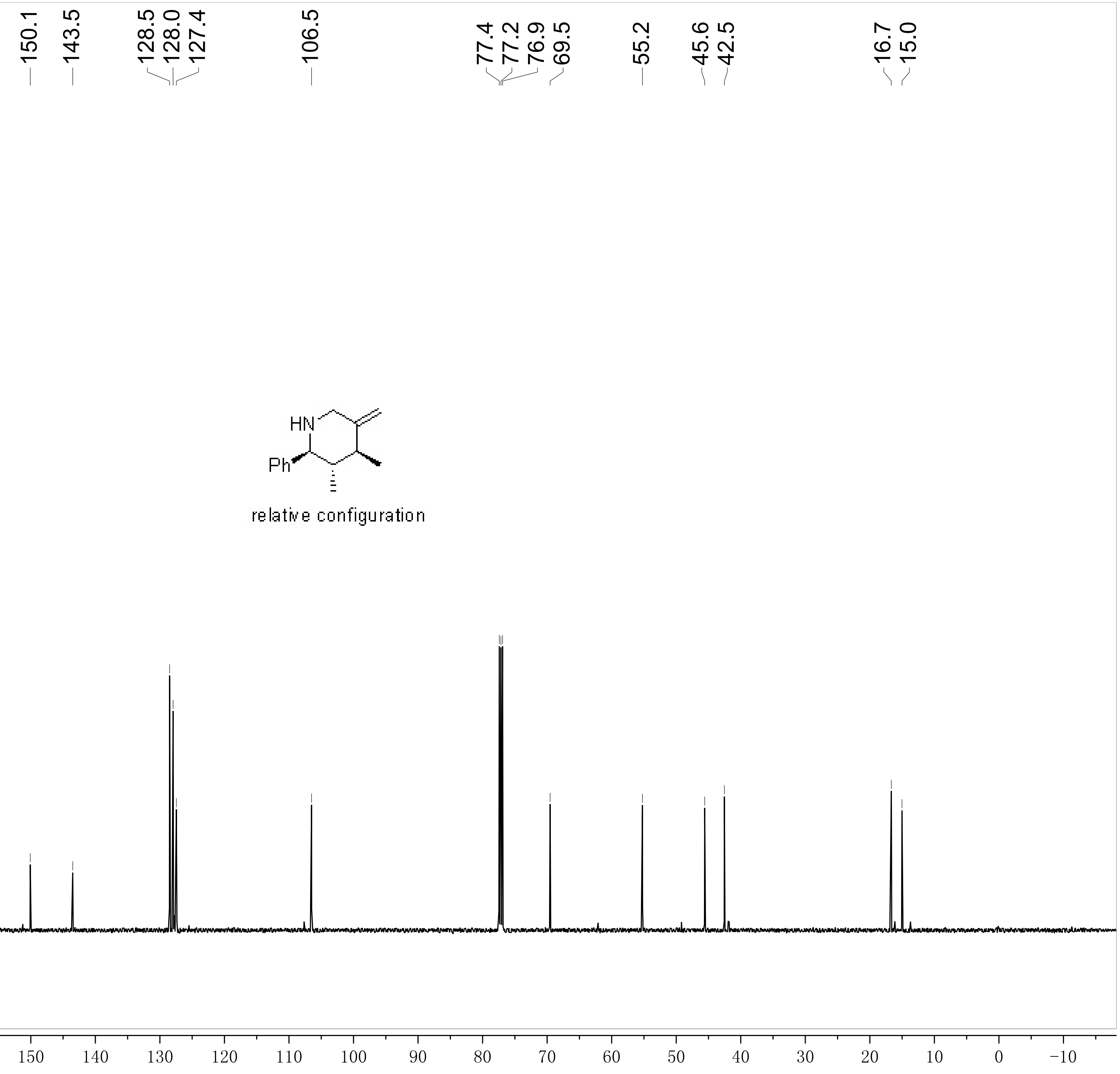

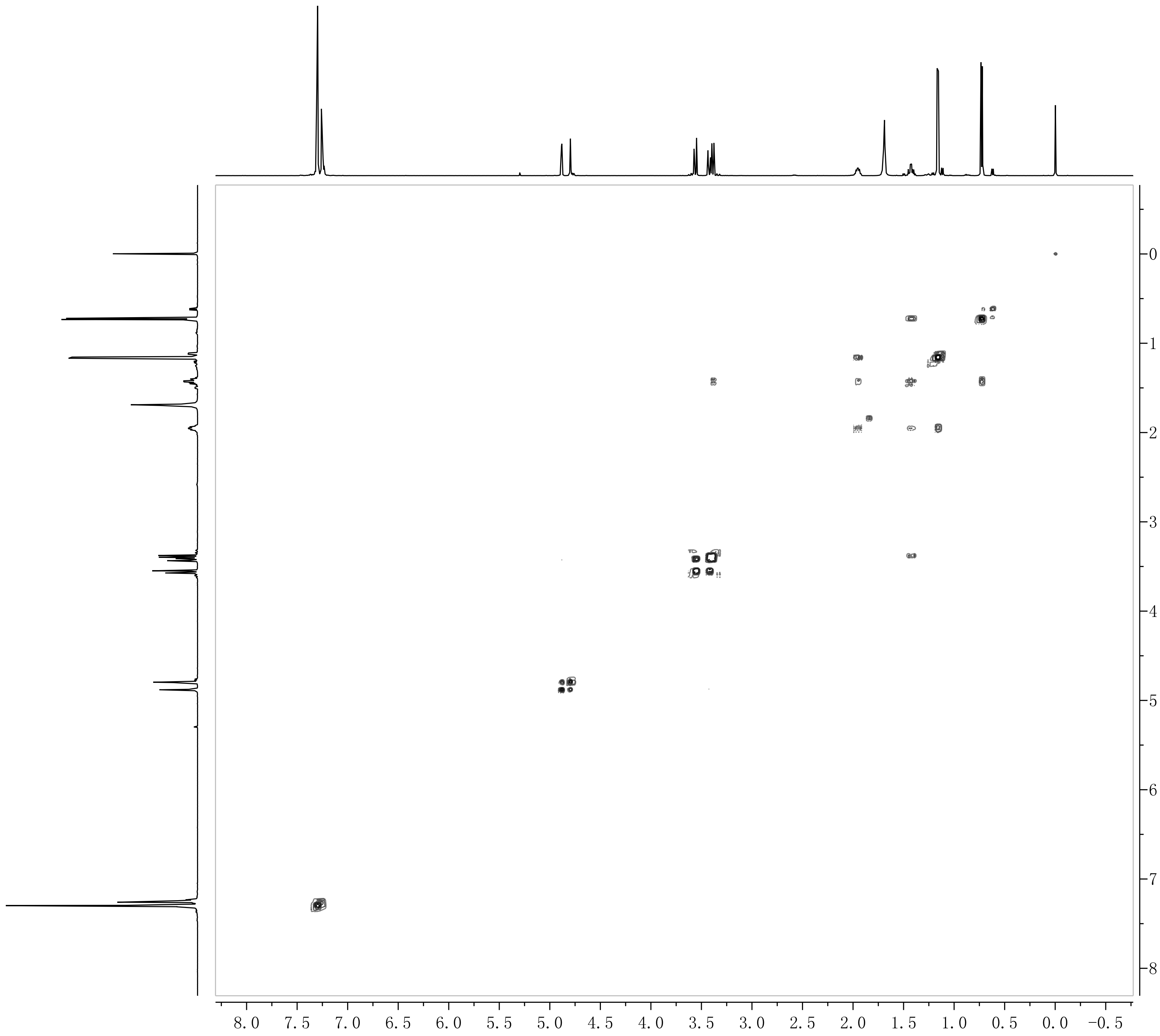

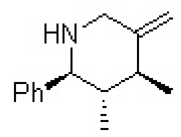

relative configuration

Parameter	Value
Title	xfy-190329-1-s.14.ser
Comment	
Origin	Bruker BioSpin GmbH
Owner	nmr
Site	
Instrument	spect
Solvent	CDCl3
Temperature	296.1
Pulse Sequence	cosygpppqf
Experiment	COSY
Number of Scans	1
Receiver Gain	21.9
Relaxation Delay	1.9279
Pulse Width	10.7100
Presaturation Frequency	
Acquisition Time	0.2253
Class	
Spectrometer Frequency	(500.13, 500.13)
Spectral Width	(4545.5, 4545.5)
Lowest Frequency	(-390.6, -390.6)
Nucleus	(1H, 1H)
Acquired Size	(1024, 128)
Spectral Size	(1024, 1024)

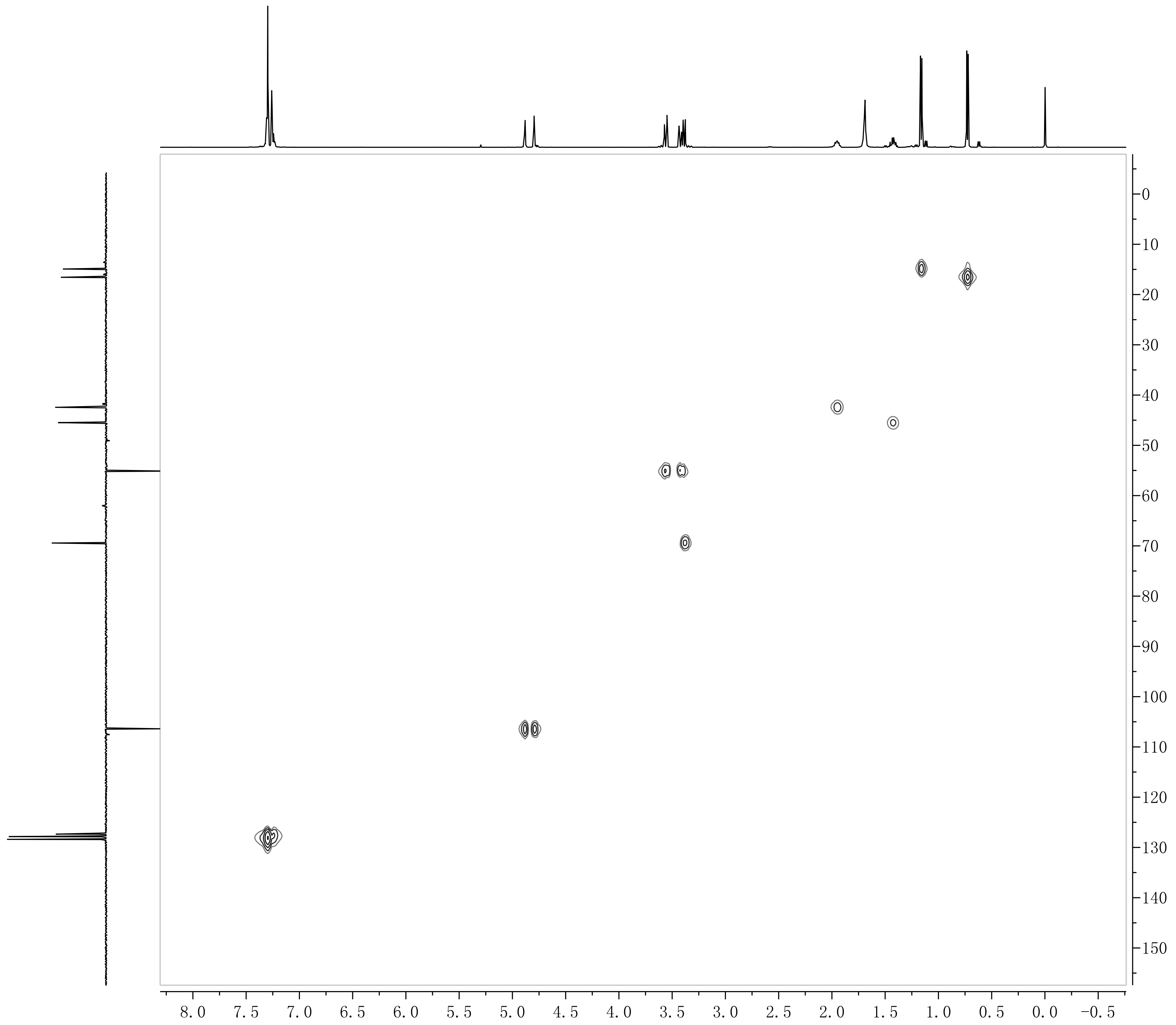

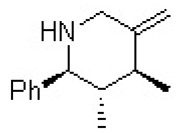

relative configuration

Parameter	Value
Title	xfy-190329-1-s.15.ser
Comment	
Origin	Bruker BioSpin GmbH
Owner	nmr
Site	
Instrument	spect
Solvent	CDCl3
Temperature	296.1
Pulse Sequence	hsqcedetgp
Experiment	HSQC-EDITED
Number of Scans	2
Receiver Gain	27.5
Relaxation Delay	1.4662
Pulse Width	10.7100
Presaturation Frequency	
Acquisition Time	0.1126
Class	
Spectrometer Frequency	(500.13, 125.77)
Spectral Width	(4545.5, 20833.3)
Lowest Frequency	(-390.6, -1037.0)
Nucleus	(1H, 13C)
Acquired Size	(512, 256)
Spectral Size	(512, 512)

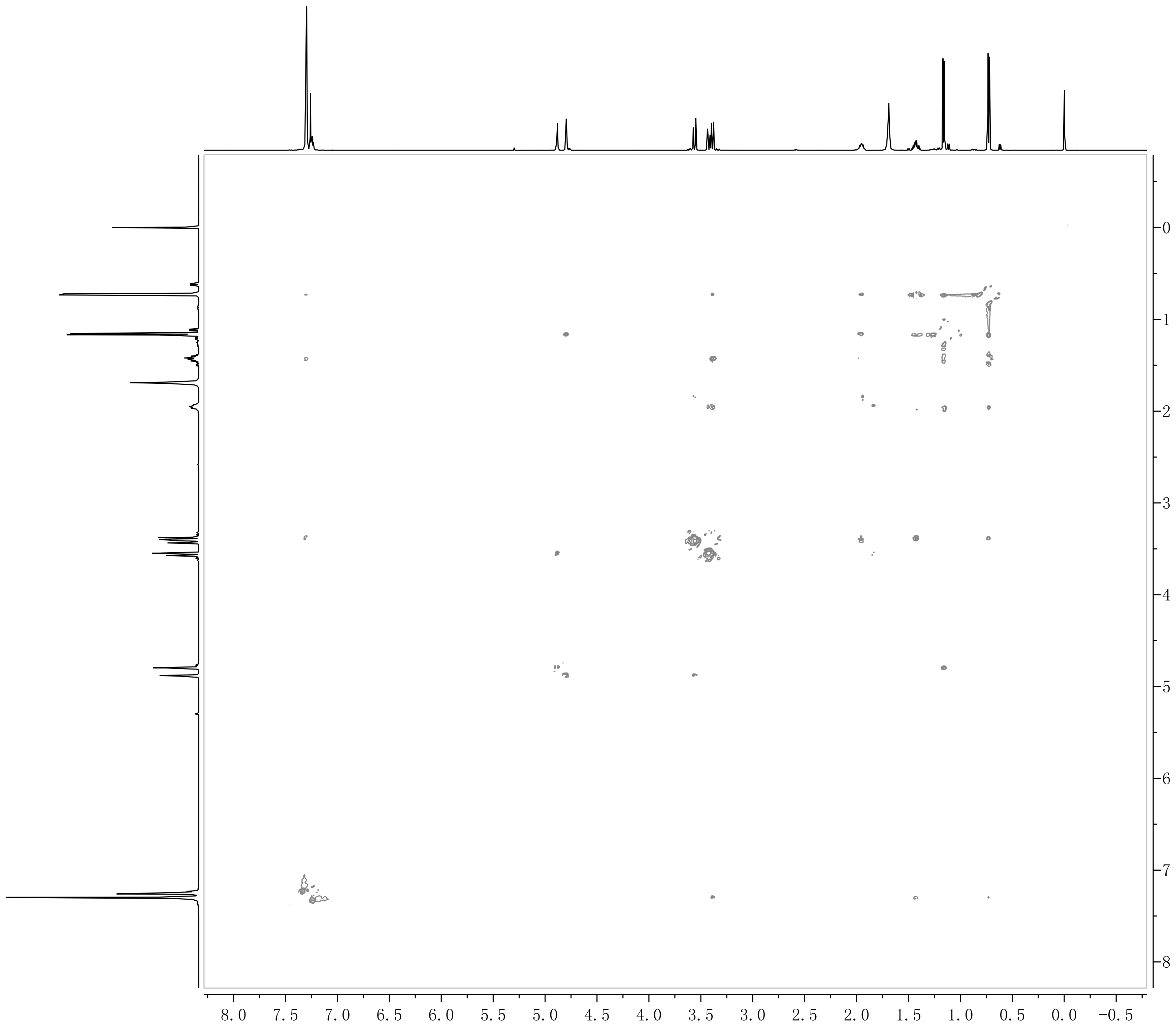

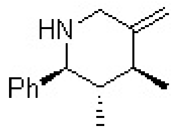

relative configuration

Parameter	Value
Title	xfy-190329-1-s.16.ser
Comment	
Origin	Bruker BioSpin GmbH
Owner	nmr
Site	
Instrument	spect
Solvent	CDCl3
Temperature	296.1
Pulse Sequence	noesygpphpp
Experiment	NOESY
Number of Scans	8
Receiver Gain	19.3
Relaxation Delay	1.9795
Pulse Width	10.7100
Presaturation Frequency	
Acquisition Time	0.2253
Class	
Spectrometer Frequency	(500.13, 500.13)
Spectral Width	(4545.5, 4545.5)
Lowest Frequency	(-401.8, -401.8)
Nucleus	(1H, 1H)
Acquired Size	(1024, 256)
Spectral Size	(1024, 1024)

Parameter	Value
Title	xfy-190329-1-s.21.1.1r
Comment	
Origin	Bruker BioSpin GmbH
Owner	nmr
Site	
Instrument	spect
Solvent	CDCl3
Temperature	296.1
Pulse Sequence	zg30
Experiment	1D
Number of Scans	4
Receiver Gain	124.7
Relaxation Delay	1.0000
Pulse Width	10.7100
Presaturation Frequency	
Acquisition Time	3.2768
Class	
Spectrometer Frequency	500.13
Spectral Width	10000.0
Lowest Frequency	-1911.5
Nucleus	1H
Acquired Size	32768
Spectral Size	65536

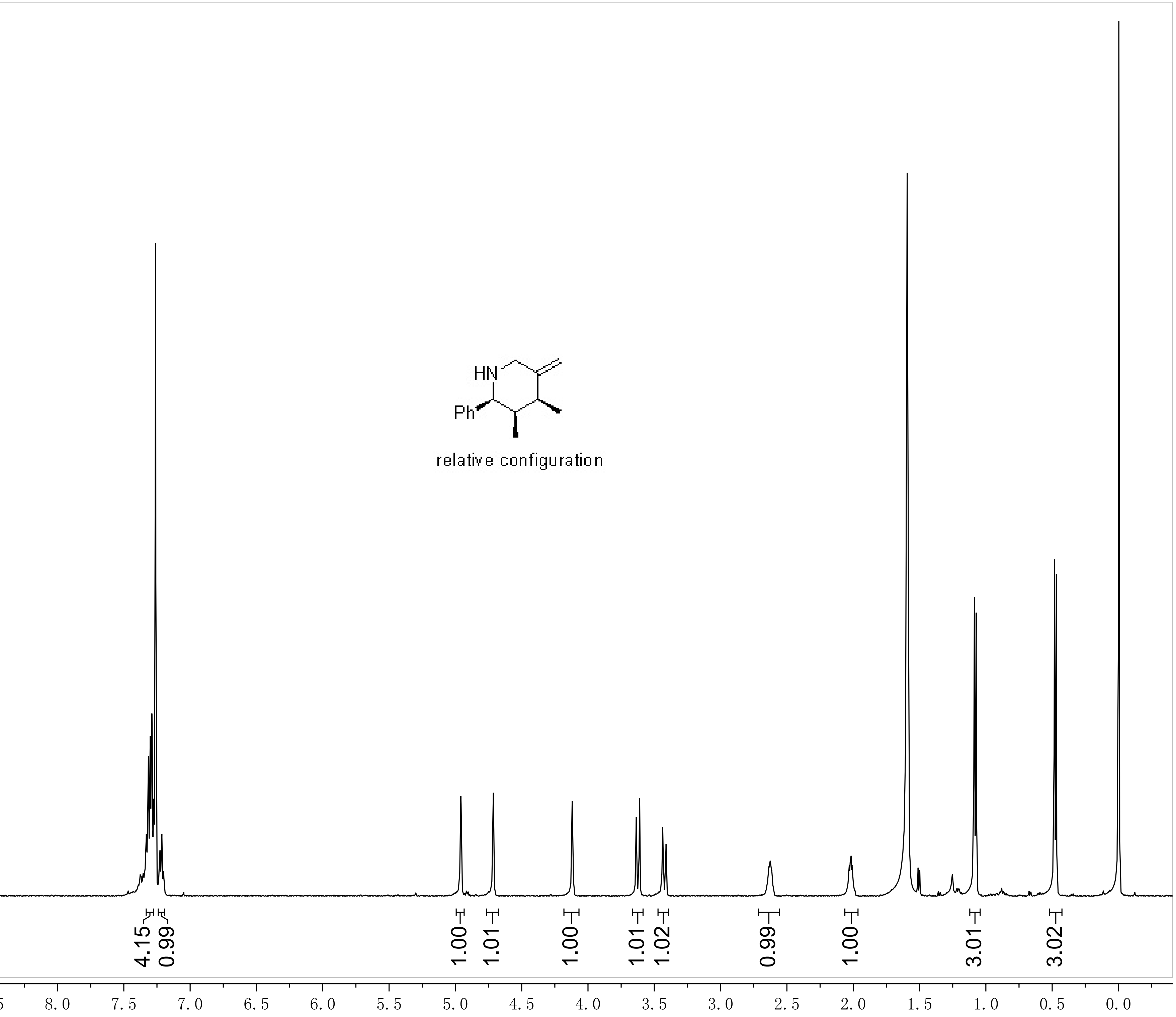

Parameter	Value
Title	xfy-190413-1-s.22.1.1r
Comment	
Origin	Bruker BioSpin GmbH
Owner	nmr
Site	
Instrument	spect
Solvent	CDCl3
Temperature	296.2
Pulse Sequence	zgpg30
Experiment	1D
Number of Scans	72
Receiver Gain	193.1
Relaxation Delay	2.0000
Pulse Width	9.6000
Presaturation Frequency	
Acquisition Time	1.1010
Class	
Spectrometer Frequency	125.76
Spectral Width	29761.9
Lowest Frequency	-2290.4
Nucleus	13C
Acquired Size	32768
Spectral Size	32768

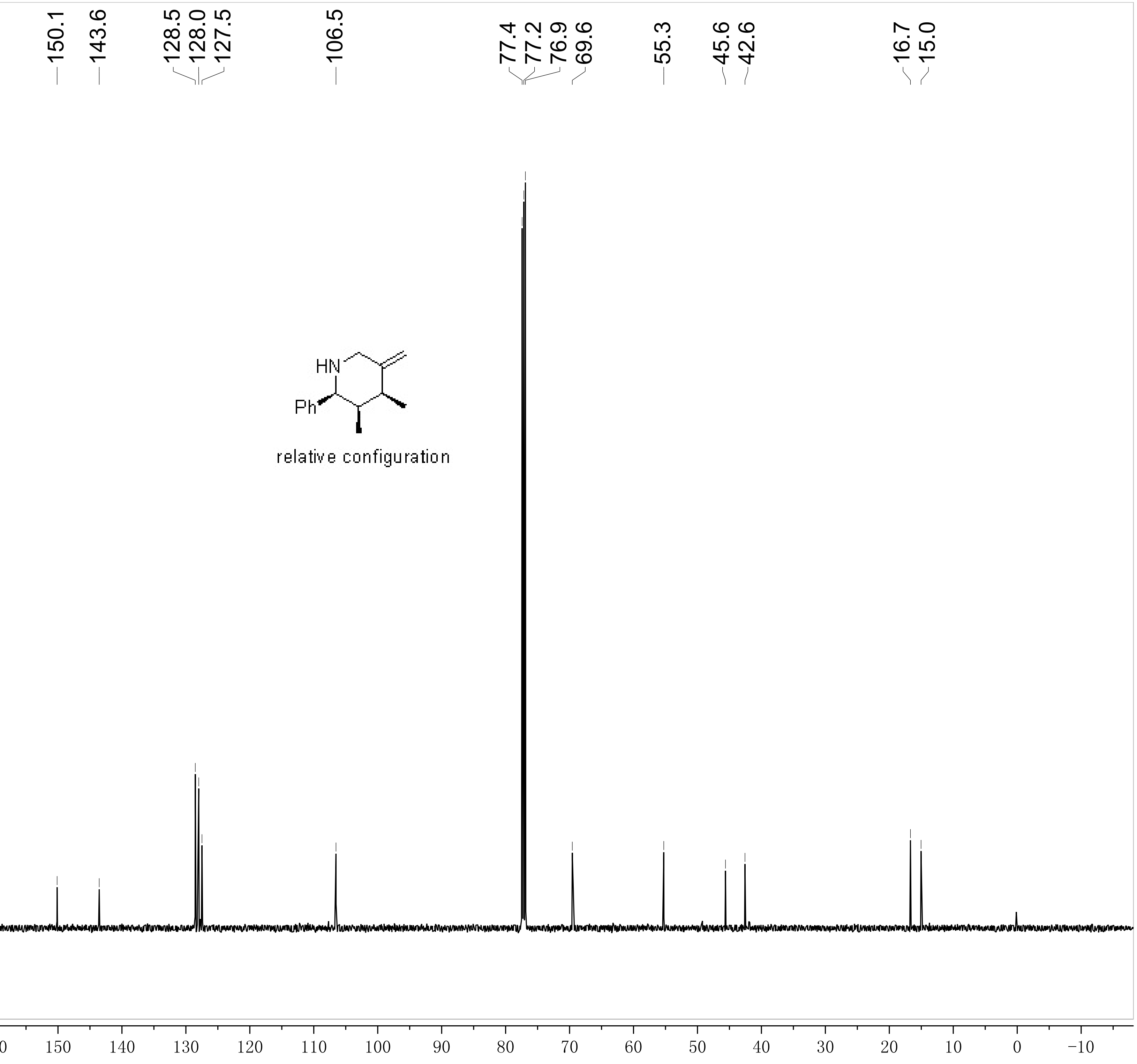

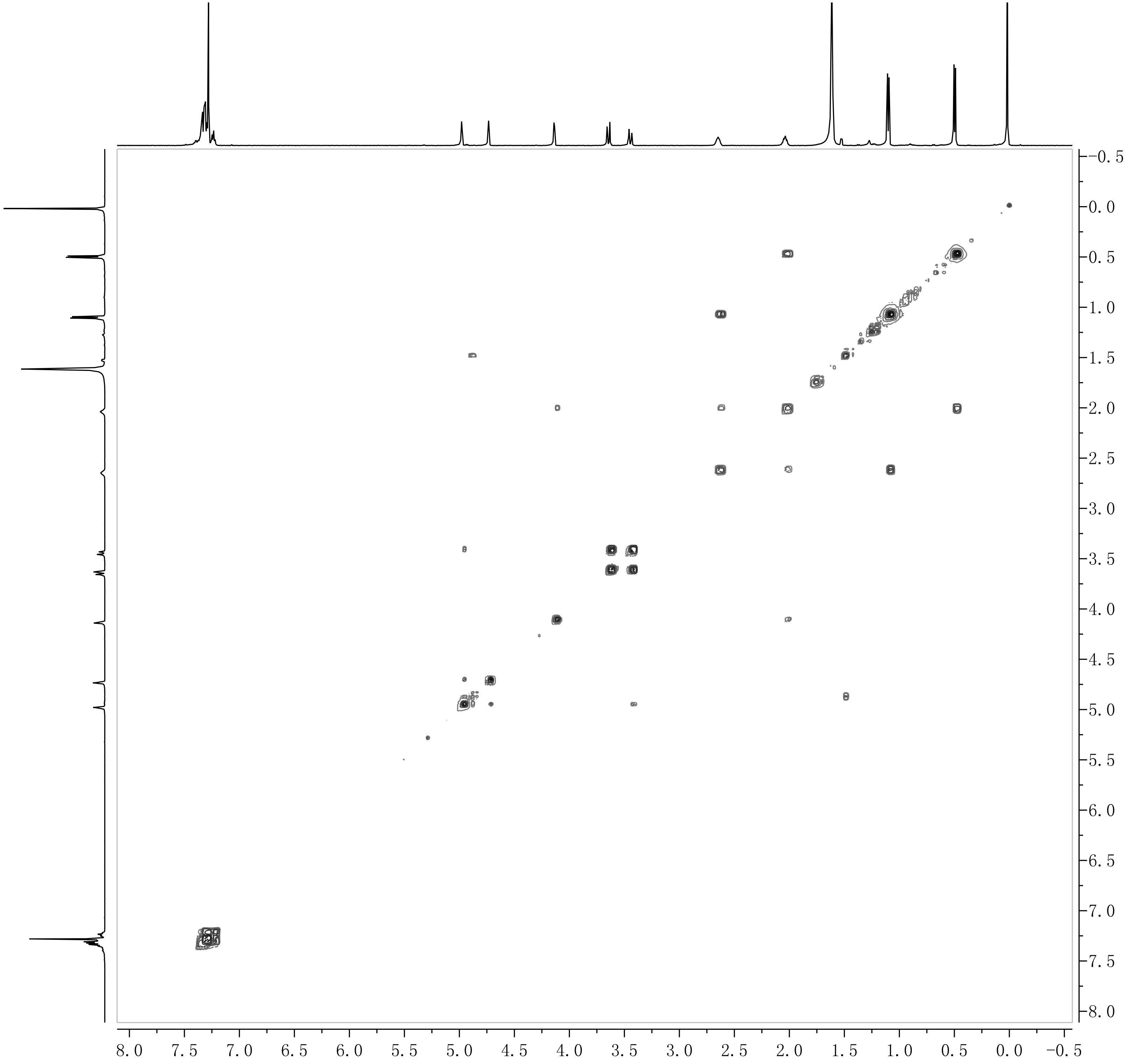

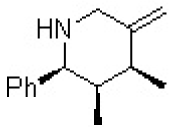

relative configuration

Parameter	Value
Title	xfy-190413-1-s.5.ser
Comment	
Origin	Bruker BioSpin GmbH
Owner	nmr
Site	
Instrument	spect
Solvent	CDCl3
Temperature	296.1
Pulse Sequence	cosygpppqf
Experiment	COSY
Number of Scans	1
Receiver Gain	25.2
Relaxation Delay	1.9177
Pulse Width	10.7100
Presaturation Frequency	
Acquisition Time	0.2355
Class	
Spectrometer Frequency	(500.13, 500.13)
Spectral Width	(4347.8, 4347.8)
Lowest Frequency	(-290.8, -290.8)
Nucleus	(1H, 1H)
Acquired Size	(1024, 128)
Spectral Size	(1024, 1024)

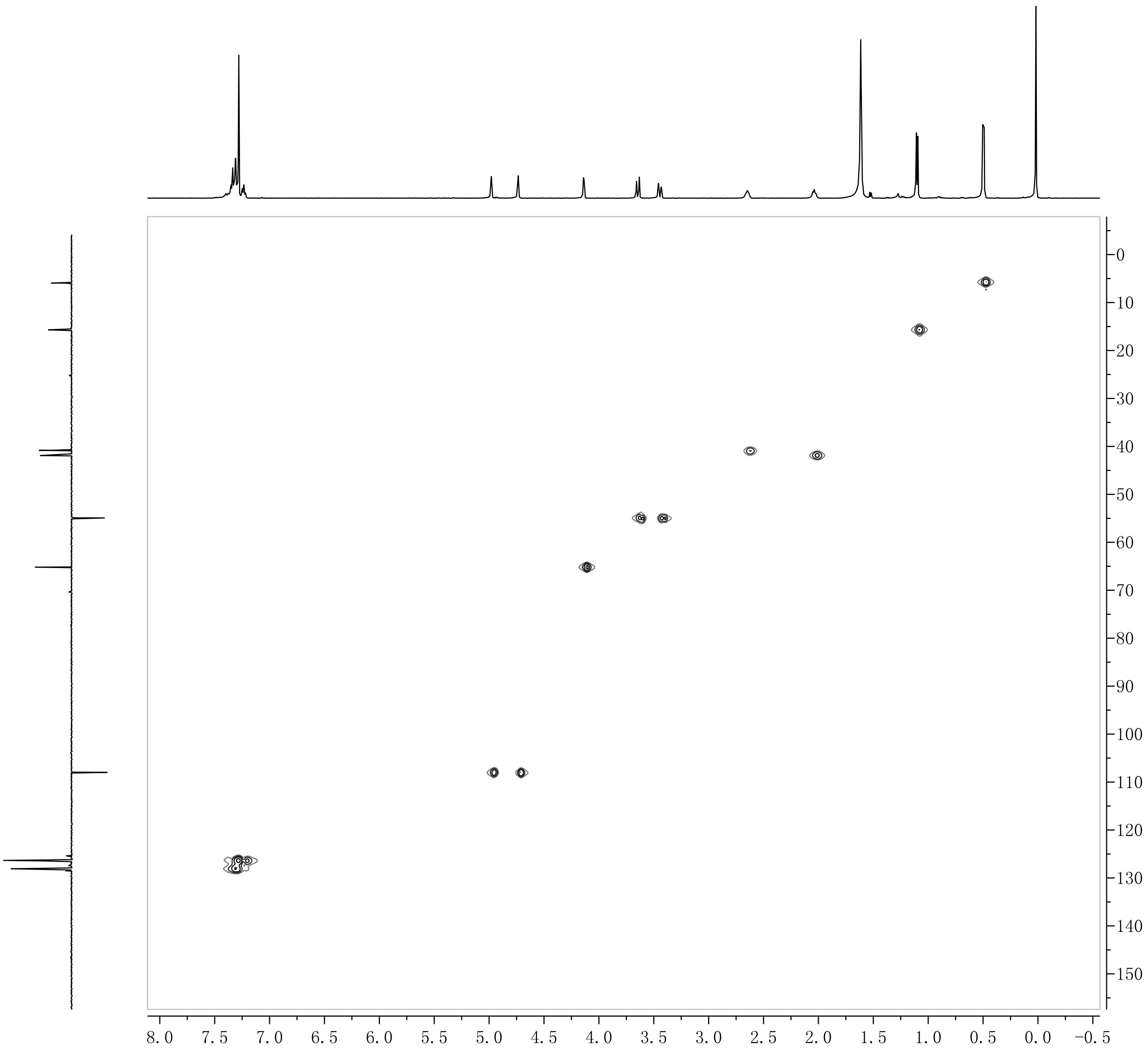

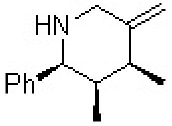

relative configuration

Parameter	Value
Title	xfy-190413-1-s.6.ser
Comment	
Origin	Bruker BioSpin GmbH
Owner	nmr
Site	
Instrument	spect
Solvent	CDCl3
Temperature	296.1
Pulse Sequence	hsqcedetgp
Experiment	HSQC-EDITED
Number of Scans	2
Receiver Gain	193.1
Relaxation Delay	1.4611
Pulse Width	10.7100
Presaturation Frequency	
Acquisition Time	0.1178
Class	
Spectrometer Frequency	(500.13, 125.77)
Spectral Width	(4347.8, 20833.3)
Lowest Frequency	(-290.8, -1037.0)
Nucleus	(1H, 13C)
Acquired Size	(512, 256)
Spectral Size	(512, 512)

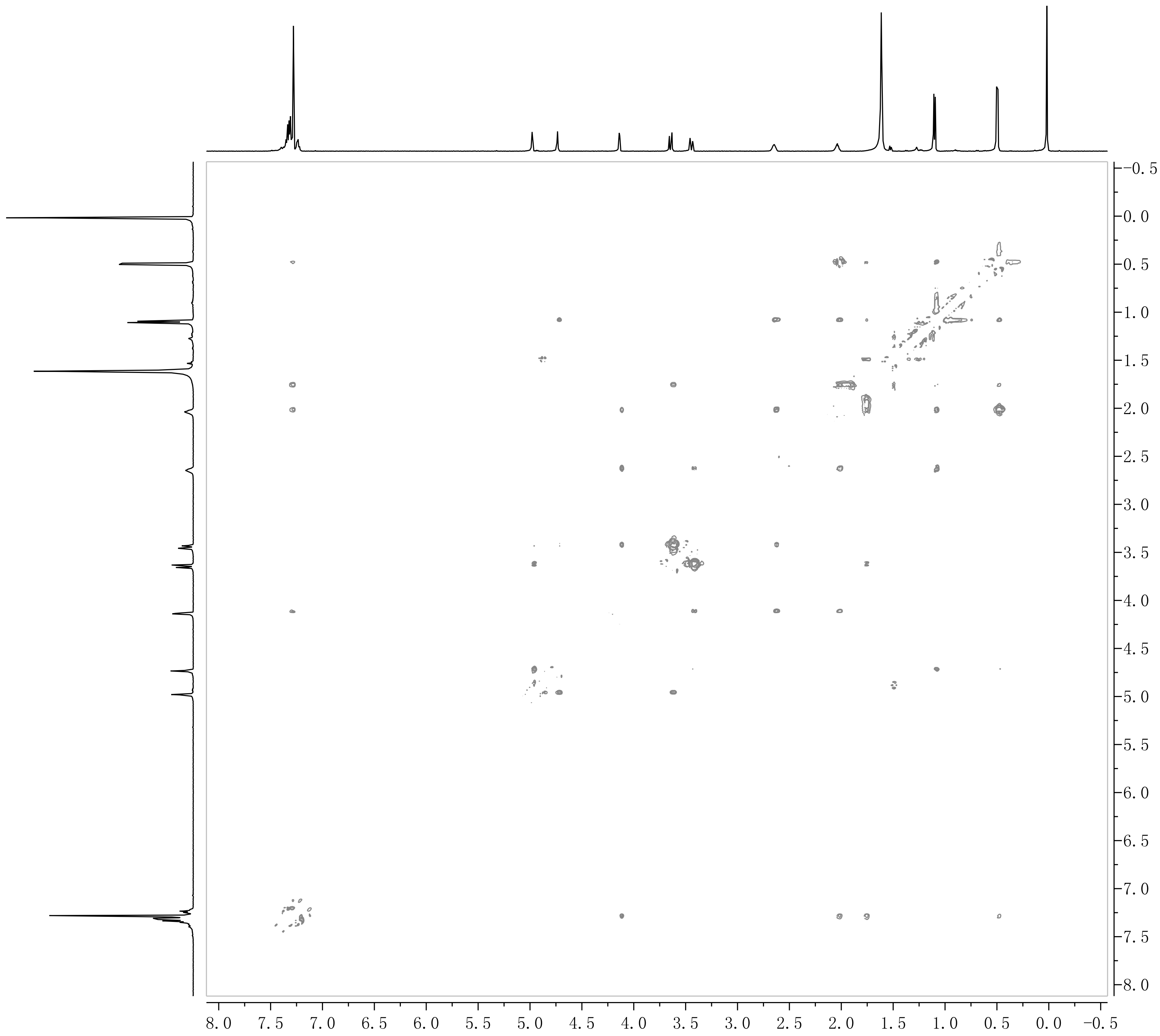

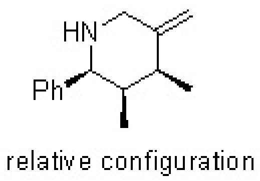

Parameter	Value
Title	xfy-190413-1-s.7.ser
Comment	
Origin	Bruker BioSpin GmbH
Owner	nmr
Site	
Instrument	spect
Solvent	CDCl3
Temperature	296.2
Pulse Sequence	noesygpphpp
Experiment	NOESY
Number of Scans	8
Receiver Gain	25.2
Relaxation Delay	1.9693
Pulse Width	10.7100
Presaturation Frequency	
Acquisition Time	0.2355
Class	
Spectrometer Frequency	(500.13, 500.13)
Spectral Width	(4347.8, 4347.8)
Lowest Frequency	(-287.5, -287.5)
Nucleus	(1H, 1H)
Acquired Size	(1024, 256)
Spectral Size	(1024, 1024)

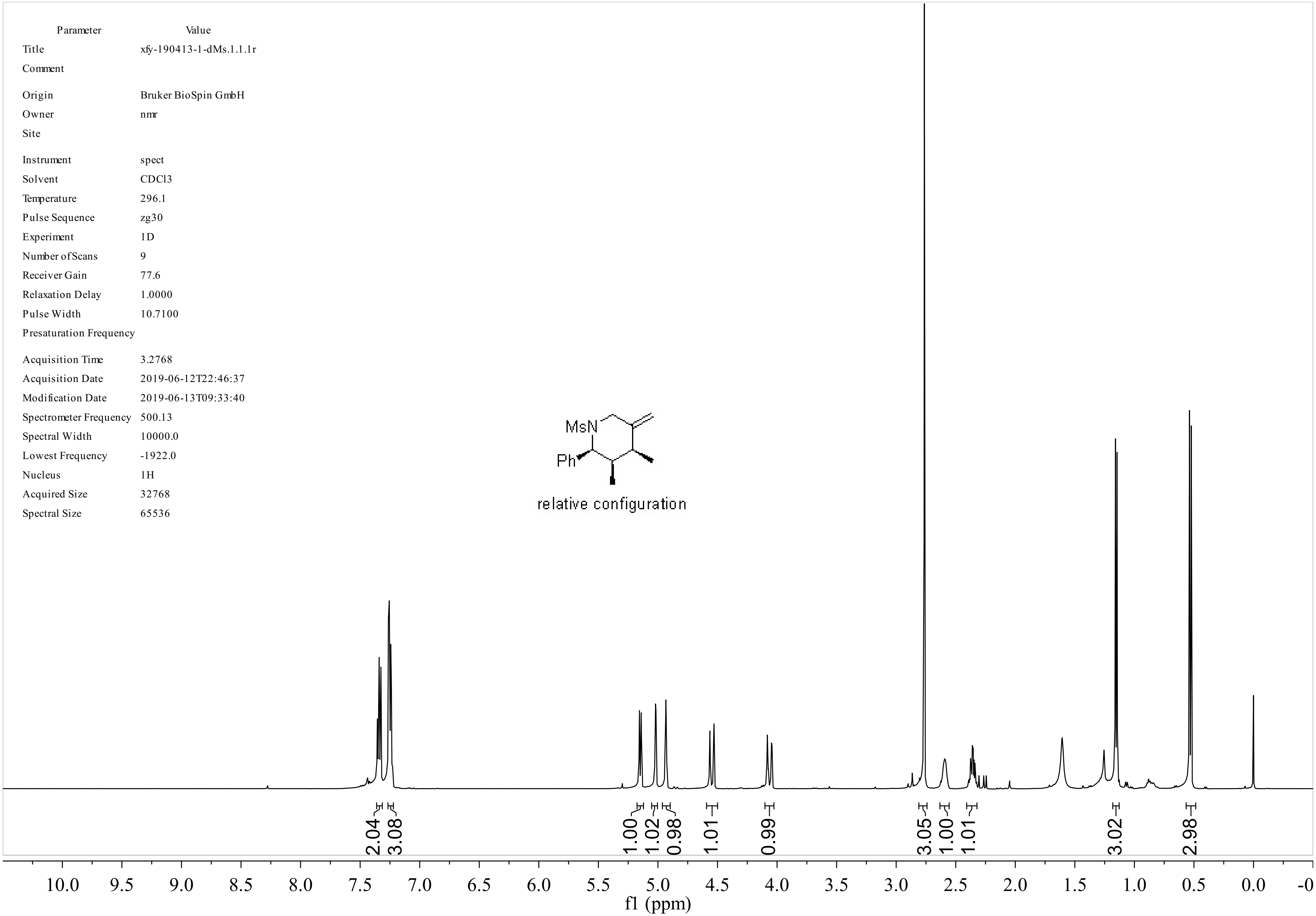

Parameter Value
Title xfy-190413-1-dMs.1.1.1r
Comment
Origin Bruker BioSpin GmbH
Owner nmr
Site
Instrument spect
Solvent CDCl3
Temperature 296.1
Pulse Sequence zg30
Experiment 1D
Number of Scans 9
Receiver Gain 77.6
Relaxation Delay 1.0000
Pulse Width 10.7100
Presaturation Frequency
Acquisition Time 3.2768
Acquisition Date 2019-06-12T22:46:37
Modification Date 2019-06-13T09:33:40
Spectrometer Frequency 500.13
Spectral Width 10000.0
Lowest Frequency -1922.0
Nucleus 1H
Acquired Size 32768
Spectral Size 65536
MsN
Ph
relative configuration
2.04
3.08
1.00
1.02
0.98
1.01
0.99
3.05
1.00
1.01
3.02
2.98
10.0 9.5 9.0 8.5 8.0 7.5 7.0 6.5 6.0 5.5 5.0 4.5 4.0 3.5 3.0 2.5 2.0 1.5 1.0 0.5 0.0 -0
f1 (ppm)

Parameter	Value
Title	xfy-190413-1-dMs.2.fid
Comment	
Origin	Bruker BioSpin GmbH
Owner	nmr
Site	
Instrument	spect
Solvent	CDCl3
Temperature	296.2
Pulse Sequence	zgpg30
Experiment	1D
Number of Scans	32
Receiver Gain	193.1
Relaxation Delay	2.0000
Pulse Width	9.6000
Presaturation Frequency	
Acquisition Time	1.1010
Acquisition Date	2019-06-12T22:49:04
Modification Date	2019-06-13T09:33:41
Spectrometer Frequency	125.77
Spectral Width	29761.9
Lowest Frequency	-2294.2
Nucleus	13C
Acquired Size	32768
Spectral Size	65536

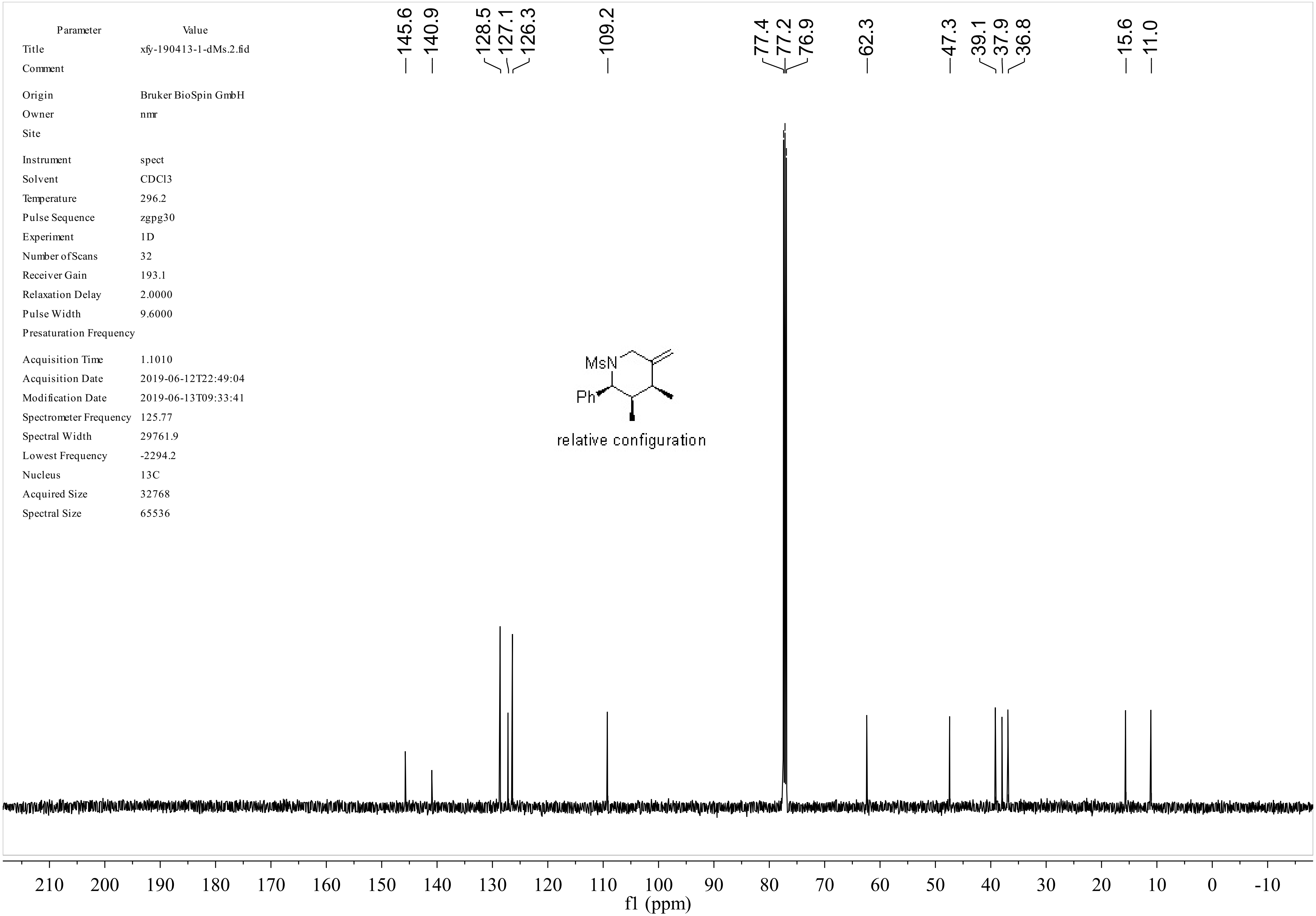

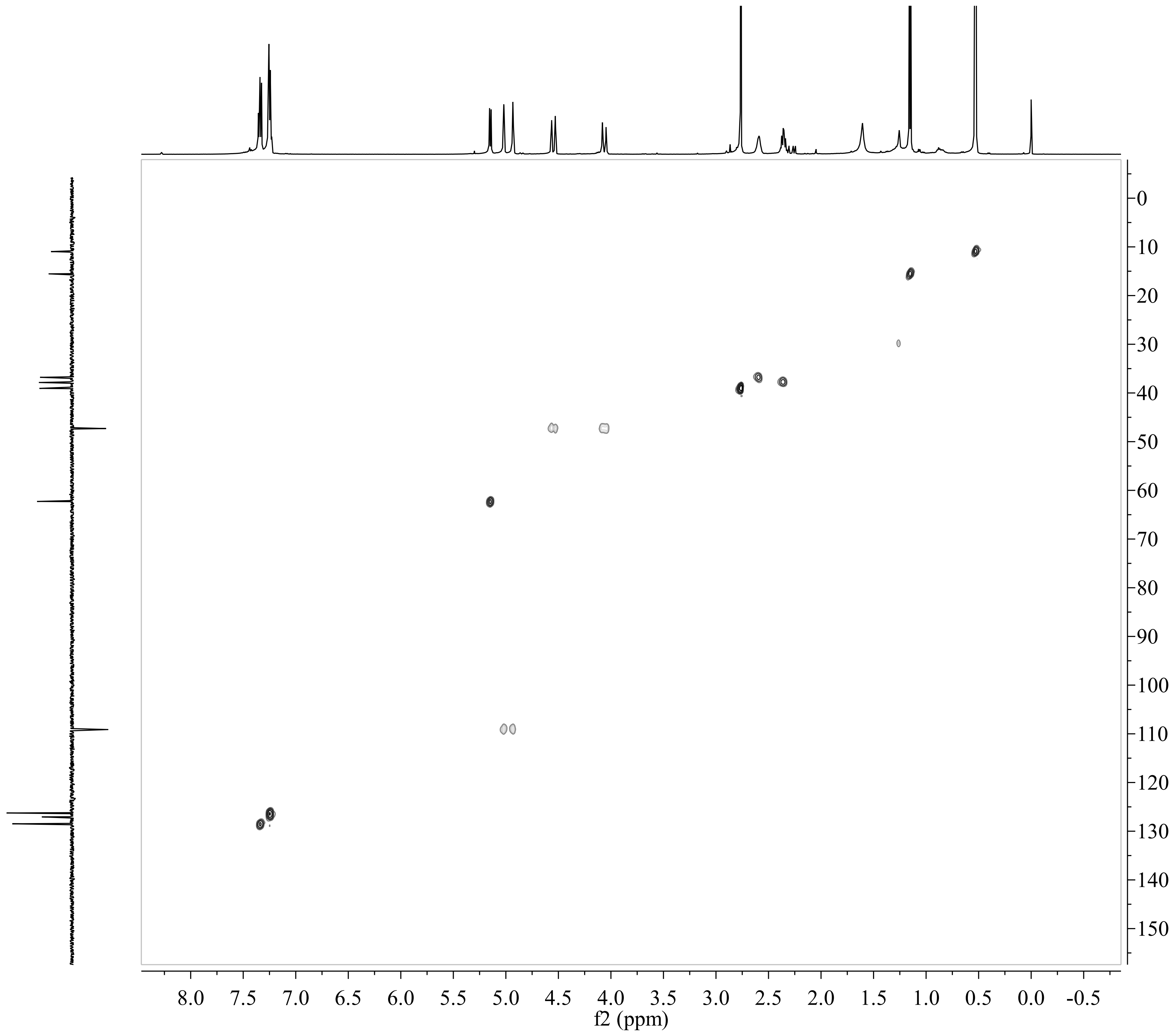

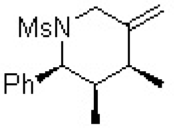

relative configuration

Parameter	Value
Title	xfy-190413-1-dMs.4.ser
Comment	
Origin	Bruker BioSpin GmbH
Owner	nmr
Site	
Instrument	spect
Solvent	CDCl3
Temperature	296.2
Pulse Sequence	hsqcedetgp
Experiment	HSQC-EDITED
Number of Scans	2
Receiver Gain	193.1
Relaxation Delay	1.4693
Pulse Width	10.7100
Presaturation Frequency	
Acquisition Time	0.1096
Acquisition Date	2019-06-12T22:53:39
Modification Date	2019-06-13T09:33:41
Spectrometer Frequency	(500.13, 125.77)
Spectral Width	(4672.9, 20833.3)
Lowest Frequency	(-436.8, -1037.0)
Nucleus	(1H, 13C)
Acquired Size	(512, 256)
Spectral Size	(512, 512)

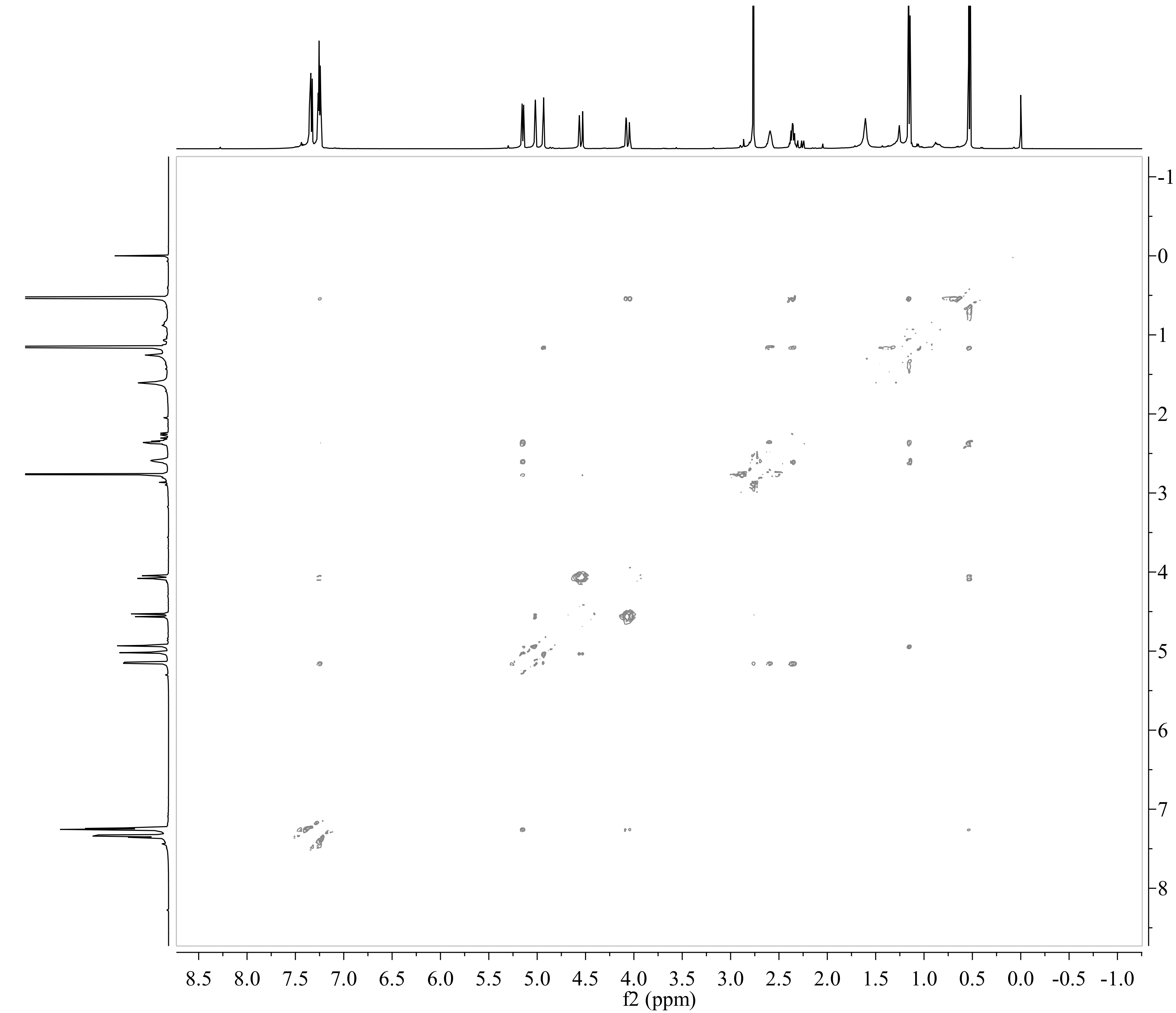

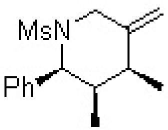

relative configuration

Parameter	Value
Title	xfy-190413-1-dMs.42.ser
Comment	
Origin	Bruker BioSpin GmbH
Owner	nmr
Site	
Instrument	spect
Solvent	CDCl3
Temperature	297.6
Pulse Sequence	noesygpphpp
Experiment	NOESY
Number of Scans	8
Receiver Gain	31.7
Relaxation Delay	2.0000
Pulse Width	8.7300
Presaturation Frequency	
Acquisition Time	0.2560
Acquisition Date	2019-06-13T06:22:03
Modification Date	2019-06-13T09:33:42
Spectrometer Frequency	(400.13, 400.13)
Spectral Width	(4000.0, 4000.0)
Lowest Frequency	(-506.6, -506.6)
Nucleus	(1H, 1H)
Acquired Size	(1024, 256)
Spectral Size	(1024, 1024)

Parameter	Value
Title	xfy-190430-2-s.41.1.1r
Comment	
Origin	Bruker BioSpin GmbH
Owner	nmr
Site	
Instrument	spect
Solvent	CDCl3
Temperature	296.1
Pulse Sequence	zg30
Experiment	1D
Number of Scans	4
Receiver Gain	87.5
Relaxation Delay	1.0000
Pulse Width	10.7100
Presaturation Frequency	
Acquisition Time	3.2768
Acquisition Date	2019-05-12T21:09:00
Modification Date	2019-05-13T14:30:24
Spectrometer Frequency	500.13
Spectral Width	10000.0
Lowest Frequency	-1922.8
Nucleus	1H
Acquired Size	32768
Spectral Size	65536

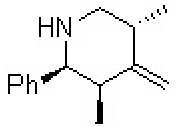

relative configuration

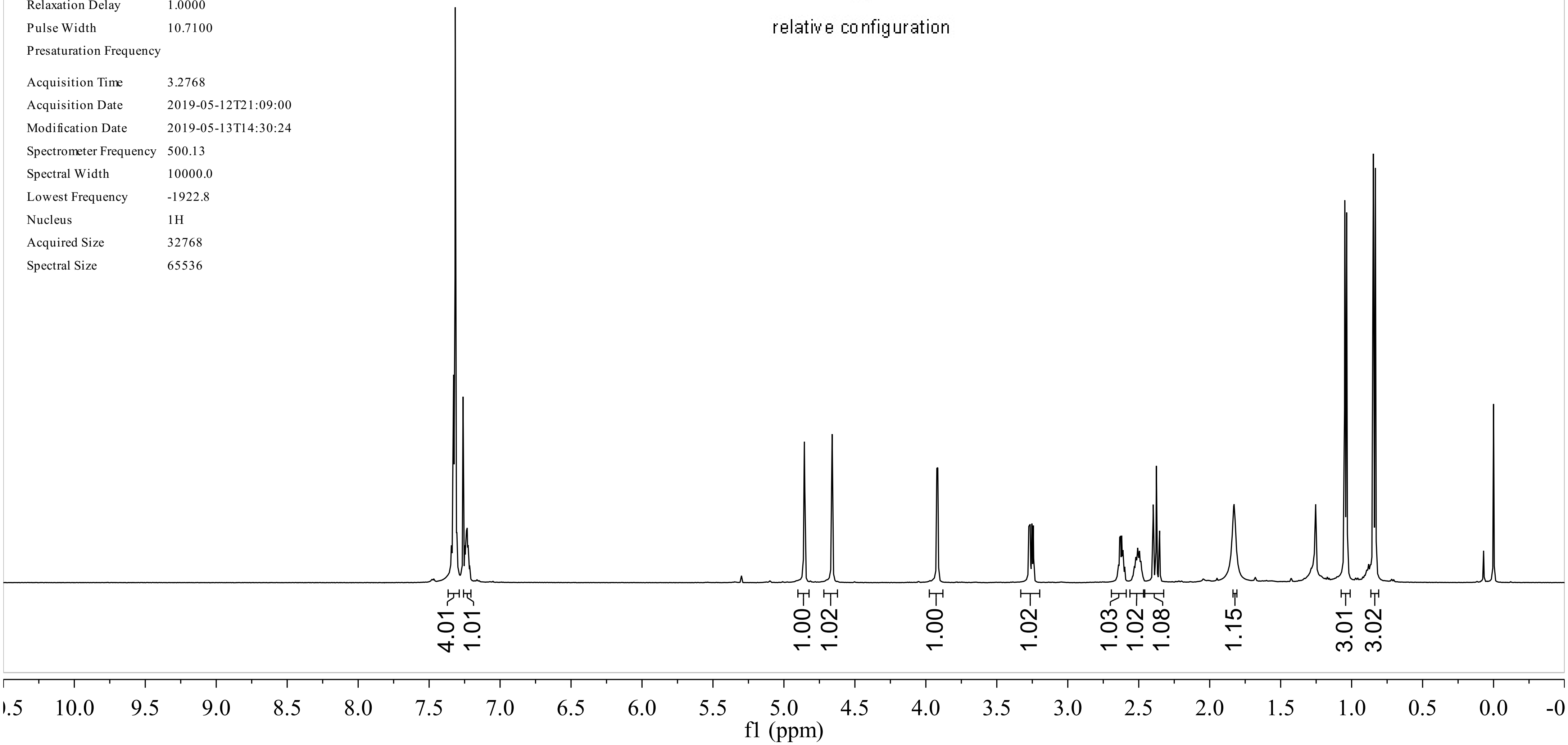

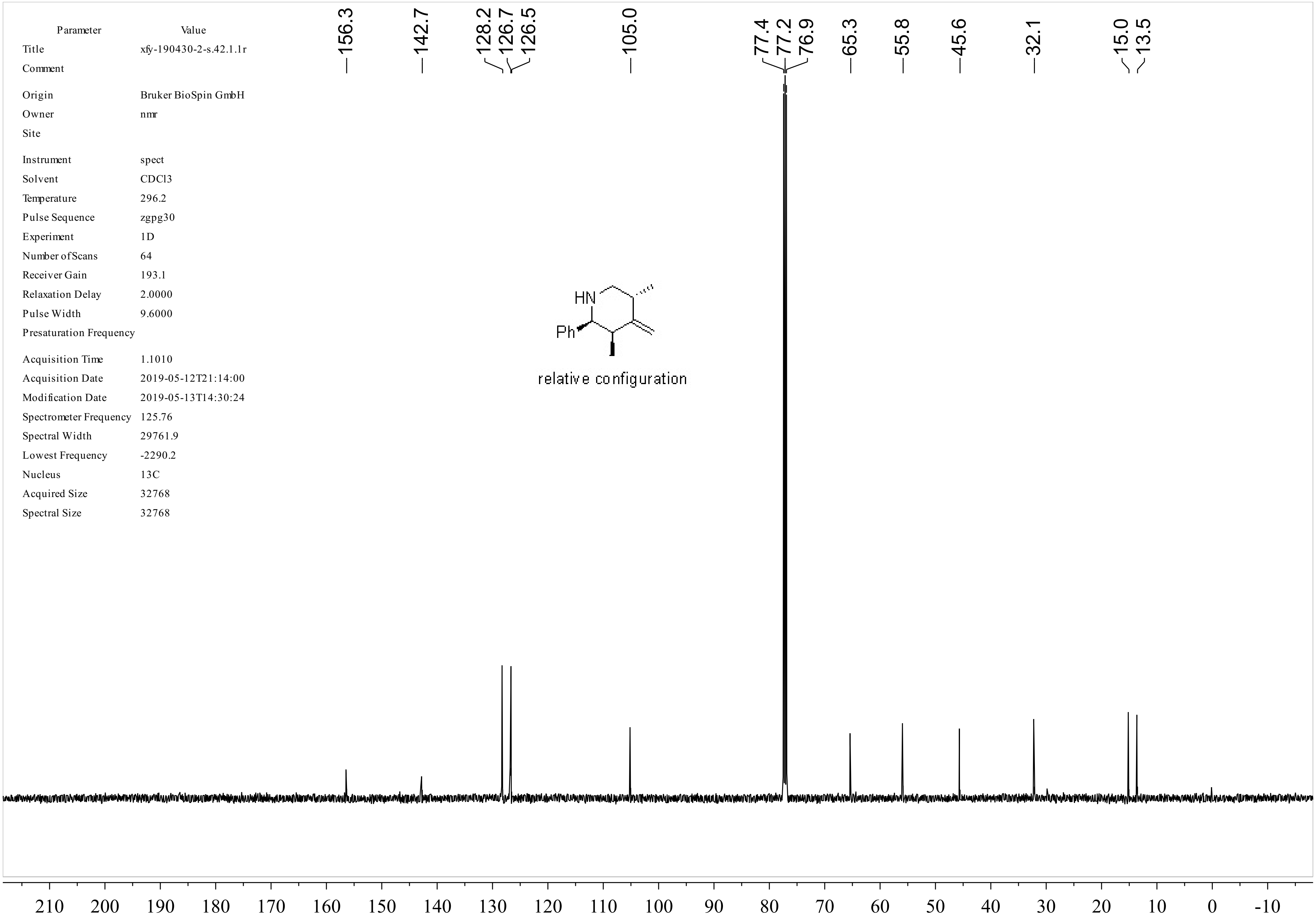

relative configuration

Parameter	Value
Title	xfy-190430-2-s.42.1.1r
Comment	
Origin	Bruker BioSpin GmbH
Owner	nmr
Site	
Instrument	spect
Solvent	CDCl3
Temperature	296.2
Pulse Sequence	zgpg30
Experiment	1D
Number of Scans	64
Receiver Gain	193.1
Relaxation Delay	2.0000
Pulse Width	9.6000
Presaturation Frequency	
Acquisition Time	1.1010
Acquisition Date	2019-05-12T21:14:00
Modification Date	2019-05-13T14:30:24
Spectrometer Frequency	125.76
Spectral Width	29761.9
Lowest Frequency	-2290.2
Nucleus	13C
Acquired Size	32768
Spectral Size	32768

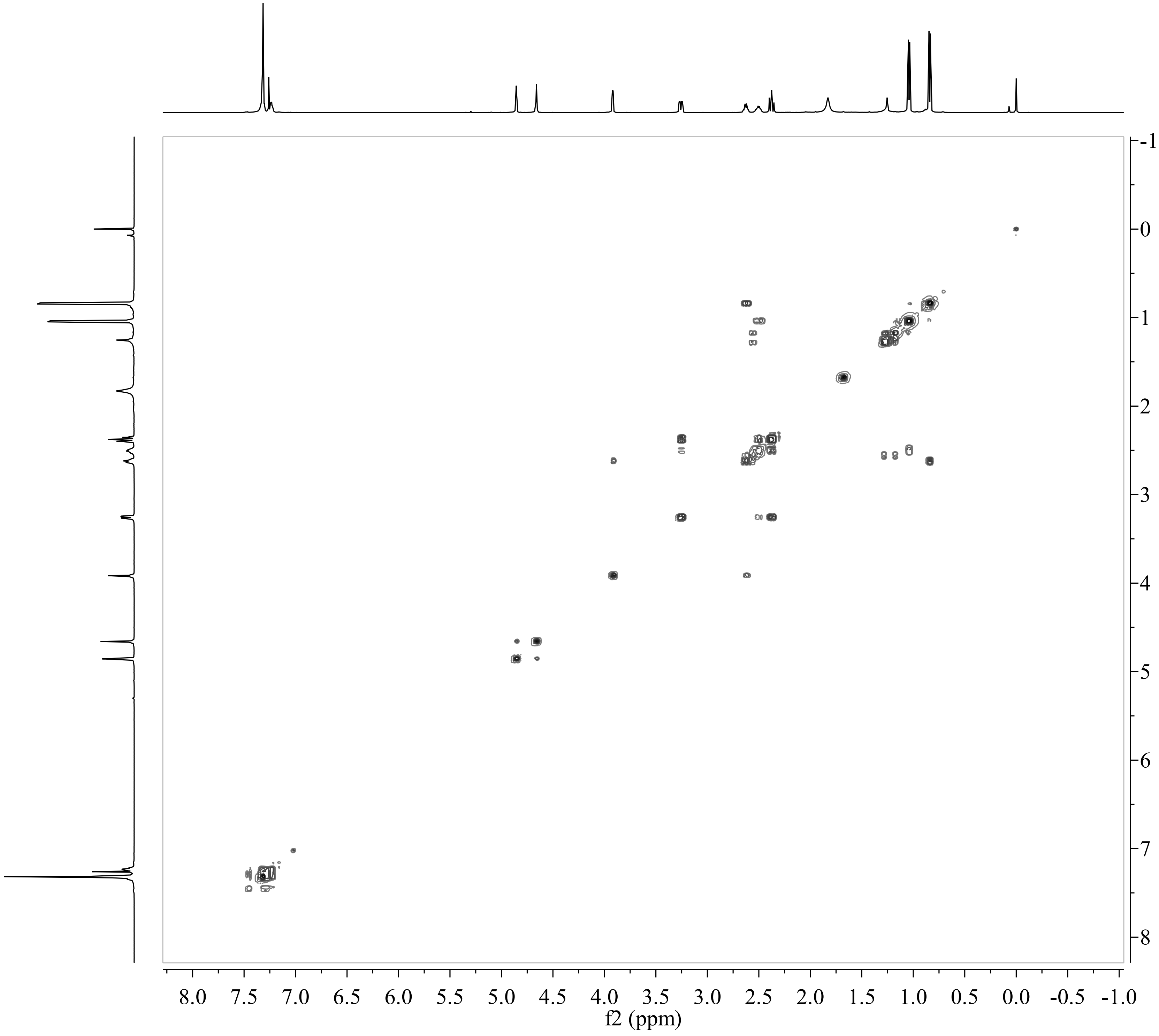

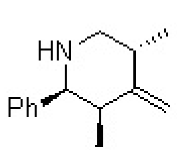

relative configuration

Parameter	Value
Title	xfy-190430-2-s.6.ser
Comment	
Origin	Bruker BioSpin GmbH
Owner	nmr
Site	
Instrument	spect
Solvent	CDCl3
Temperature	296.2
Pulse Sequence	cosygpppqf
Experiment	COSY
Number of Scans	4
Receiver Gain	54.3
Relaxation Delay	1.9341
Pulse Width	10.7100
Presaturation Frequency	
Acquisition Time	0.2191
Acquisition Date	2019-05-12T04:08:29
Modification Date	2019-05-21T19:31:25
Spectrometer Frequency	(500.13, 500.13)
Spectral Width	(4672.9, 4672.9)
Lowest Frequency	(-527.0, -527.0)
Nucleus	(1H, 1H)
Acquired Size	(1024, 128)
Spectral Size	(1024, 1024)

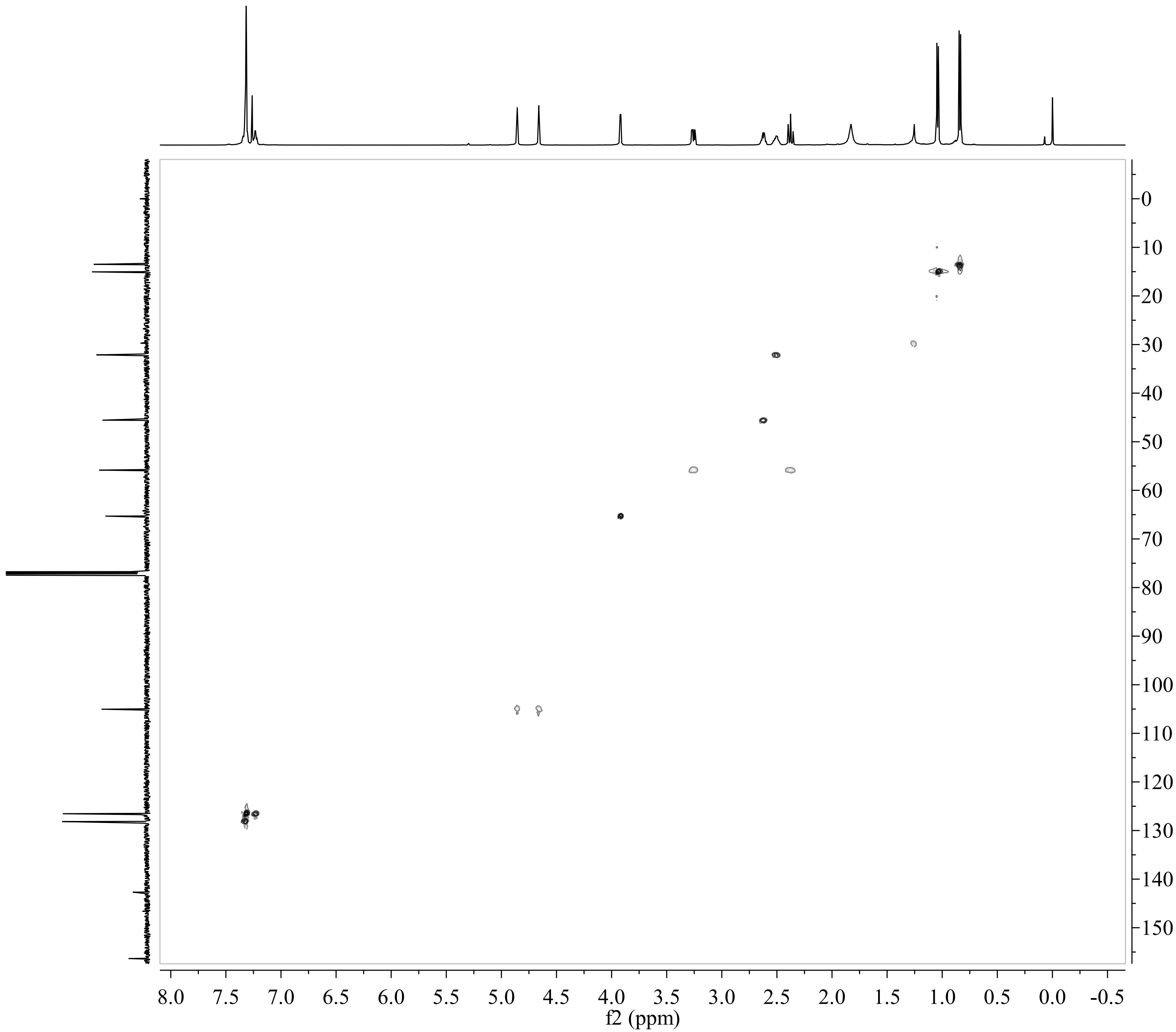

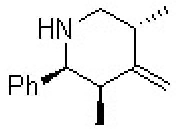

relative configuration

Parameter	Value
Title	xfy-190430-2-s.43.1.2rr
Comment	
Origin	Bruker BioSpin GmbH
Owner	nmr
Site	
Instrument	spect
Solvent	CDCl3
Temperature	296.1
Pulse Sequence	hsqcedetgp
Experiment	HSQC-EDITED
Number of Scans	2
Receiver Gain	193.1
Relaxation Delay	1.4621
Pulse Width	10.7100
Presaturation Frequency	
Acquisition Time	0.1167
Acquisition Date	2019-05-12T21:16:00
Modification Date	2019-05-13T14:30:22
Spectrometer Frequency	(500.13, 125.76)
Spectral Width	(4386.0, 20833.3)
Lowest Frequency	(-335.8, -1037.0)
Nucleus	(1H, 13C)
Acquired Size	(512, 256)
Spectral Size	(1024, 1024)

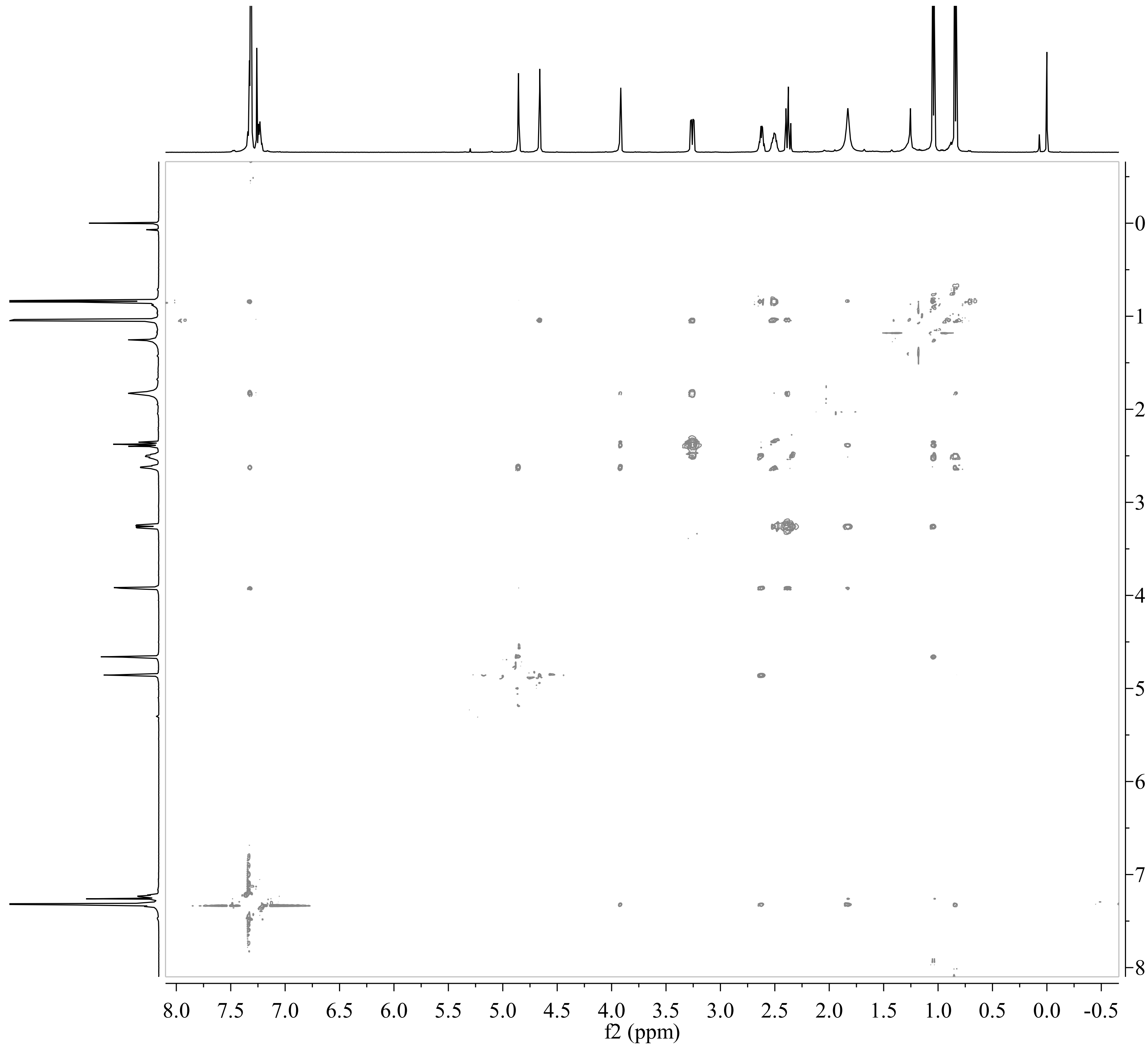

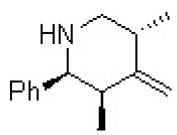

relative configuration

Parameter	Value
Title	xfy-190430-2-s.44.1.2rr
Comment	
Origin	Bruker BioSpin GmbH
Owner	nmr
Site	
Instrument	spect
Solvent	CDCl3
Temperature	296.1
Pulse Sequence	noesygpphpp
Experiment	NOESY
Number of Scans	4
Receiver Gain	31.1
Relaxation Delay	1.9713
Pulse Width	10.7100
Presaturation Frequency	
Acquisition Time	0.2335
Acquisition Date	2019-05-12T21:32:00
Modification Date	2019-05-13T14:30:20
Spectrometer Frequency	(500.13, 500.13)
Spectral Width	(4386.0, 4386.0)
Lowest Frequency	(-335.2, -335.2)
Nucleus	(1H, 1H)
Acquired Size	(1024, 176)
Spectral Size	(1024, 1024)

Parameter	Value
Title	xfy-190430-2-dMs.11.1.1r
Comment	
Origin	Bruker BioSpin GmbH
Owner	nmr
Site	
Instrument	spect
Solvent	CDCl3
Temperature	296.1
Pulse Sequence	zg30
Experiment	1D
Number of Scans	11
Receiver Gain	77.6
Relaxation Delay	1.0000
Pulse Width	10.7100
Presaturation Frequency	
Acquisition Time	3.2768
Acquisition Date	2019-05-12T17:18:33
Modification Date	2019-05-13T14:30:42
Spectrometer Frequency	500.13
Spectral Width	10000.0
Lowest Frequency	-1920.7
Nucleus	1H
Acquired Size	32768
Spectral Size	65536

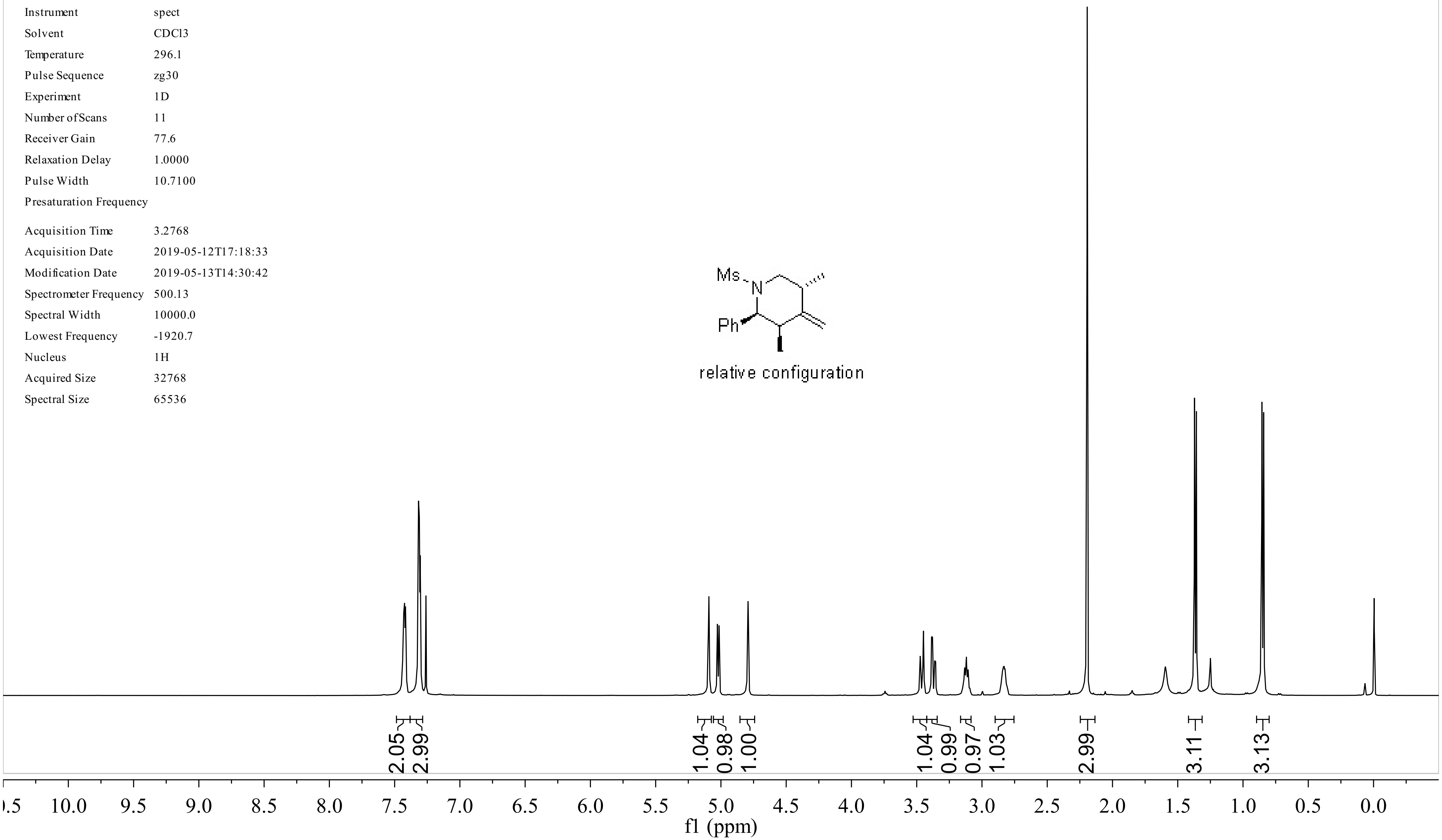

Parameter	Value
Title	xfy-190430-2-dMs.13.1.1r
Comment	
Origin	Bruker BioSpin GmbH
Owner	nmr
Site	
Instrument	spect
Solvent	CDCl3
Temperature	296.1
Pulse Sequence	deptsp135
Experiment	DEPT-135
Number of Scans	23
Receiver Gain	193.1
Relaxation Delay	2.0000
Pulse Width	9.6000
Presaturation Frequency	
Acquisition Time	1.0486
Acquisition Date	2019-05-12T17:24:09
Modification Date	2019-05-13T14:30:41
Spectrometer Frequency	125.76
Spectral Width	31250.0
Lowest Frequency	-519.2
Nucleus	13C
Acquired Size	32768
Spectral Size	32768

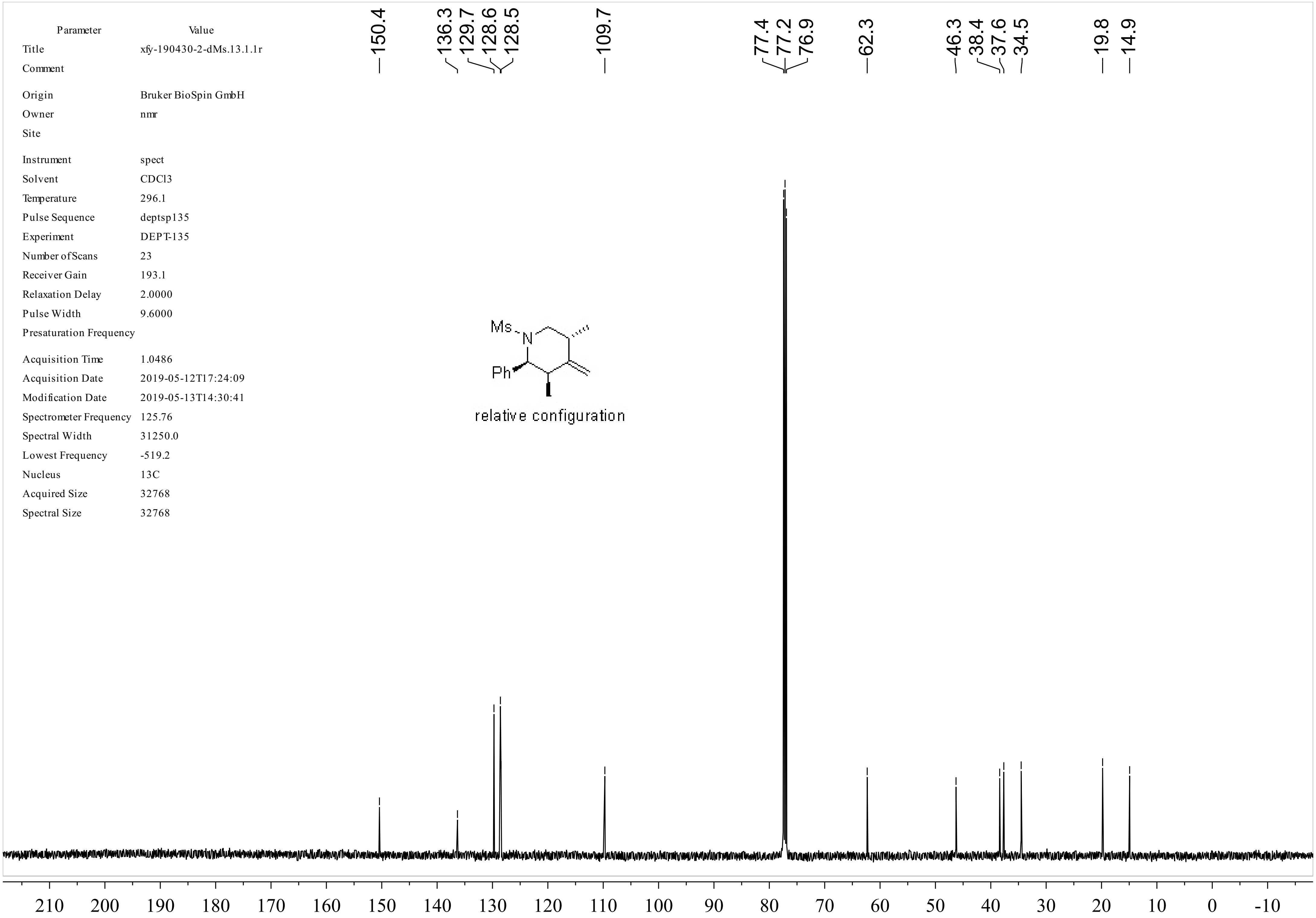

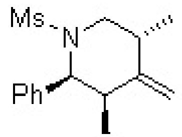

relative configuration

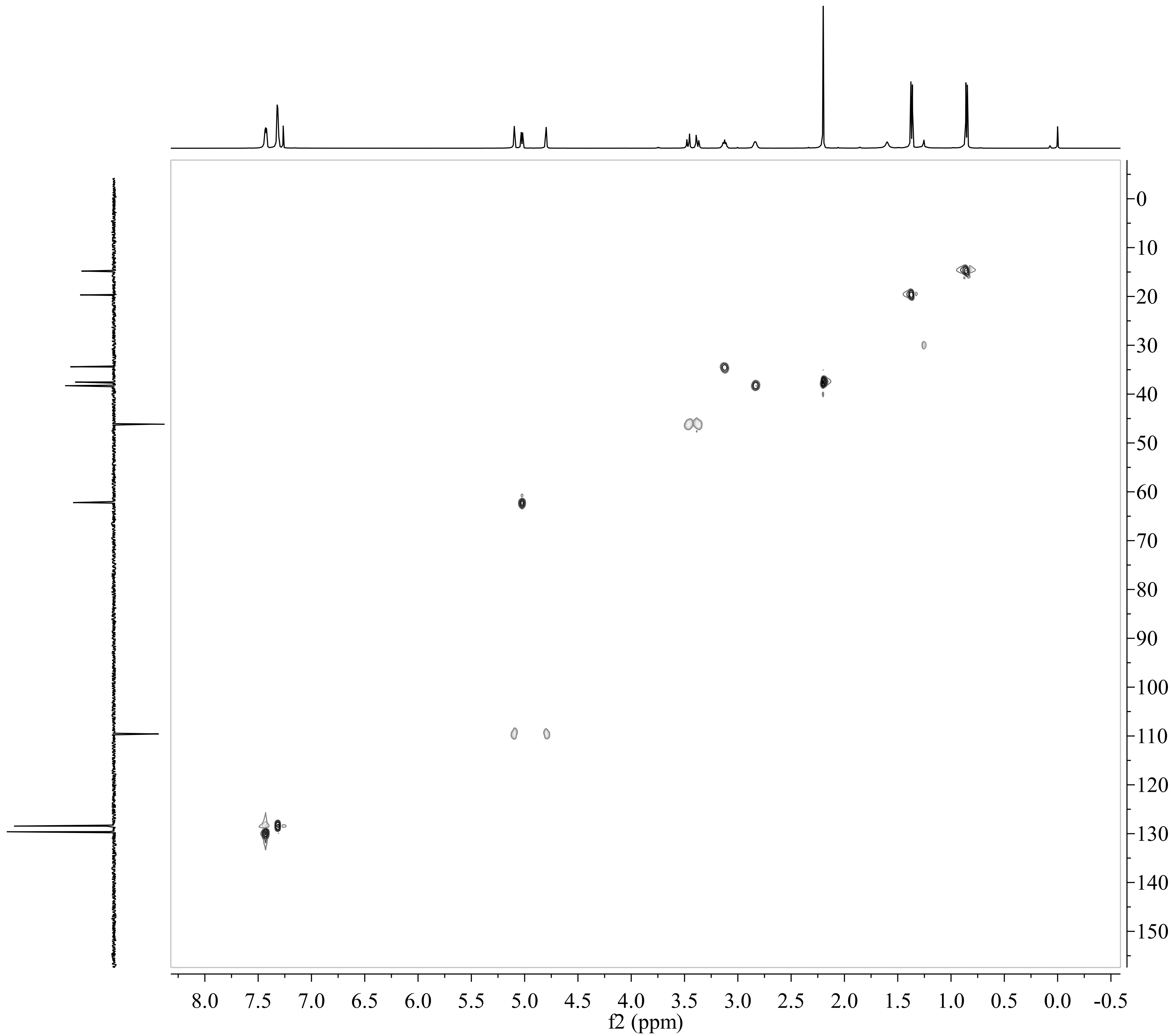

Parameter	Value
Title	xfy-190430-2-dMs.14.ser
Comment	
Origin	Bruker BioSpin GmbH
Owner	nmr
Site	
Instrument	spect
Solvent	CDCl3
Temperature	296.2
Pulse Sequence	hsqcedetgp
Experiment	HSQC-EDITED
Number of Scans	2
Receiver Gain	193.1
Relaxation Delay	1.4642
Pulse Width	10.7100
Presaturation Frequency	
Acquisition Time	0.1147
Acquisition Date	2019-05-12T17:26:31
Modification Date	2019-05-13T14:30:40
Spectrometer Frequency	(500.13, 125.77)
Spectral Width	(4464.3, 20833.3)
Lowest Frequency	(-303.5, -1037.0)
Nucleus	(1H, 13C)
Acquired Size	(512, 256)
Spectral Size	(512, 512)

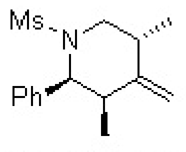

relative configuration

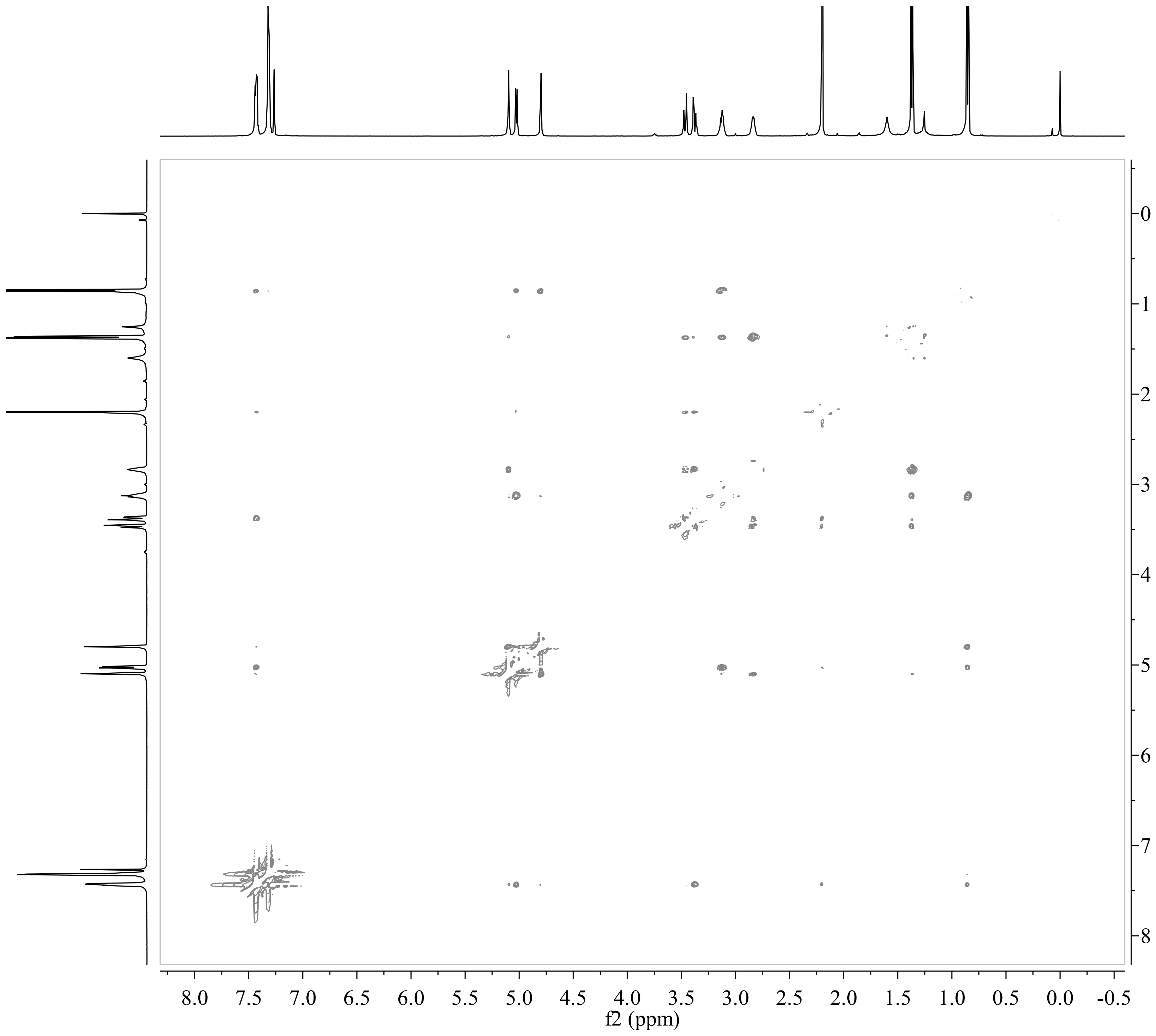

Parameter	Value
Title	xfy-190430-2-dMs.16.ser
Comment	
Origin	Bruker BioSpin GmbH
Owner	nmr
Site	
Instrument	spect
Solvent	CDCl3
Temperature	296.2
Pulse Sequence	noesygpphpp
Experiment	NOESY
Number of Scans	4
Receiver Gain	48.5
Relaxation Delay	1.9754
Pulse Width	10.7100
Presaturation Frequency	
Acquisition Time	0.2294
Acquisition Date	2019-05-12T17:44:40
Modification Date	2019-05-13T14:30:39
Spectrometer Frequency	(500.13, 500.13)
Spectral Width	(4464.3, 4464.3)
Lowest Frequency	(-303.5, -303.5)
Nucleus	(1H, 1H)
Acquired Size	(1024, 256)
Spectral Size	(1024, 1024)

Parameter	Value
Title	xfy-190427-4-s.211.1.1r
Comment	
Origin	Bruker BioSpin GmbH
Owner	nmr
Site	
Instrument	spect
Solvent	CDCl3
Temperature	296.2
Pulse Sequence	zg30
Experiment	1D
Number of Scans	16
Receiver Gain	62.9
Relaxation Delay	1.0000
Pulse Width	10.7100
Presaturation Frequency	
Acquisition Time	3.2768
Acquisition Date	2019-08-28T22:58:06
Modification Date	2019-08-29T09:37:28
Spectrometer Frequency	500.13
Spectral Width	10000.0
Lowest Frequency	-1920.6
Nucleus	1H
Acquired Size	32768
Spectral Size	65536

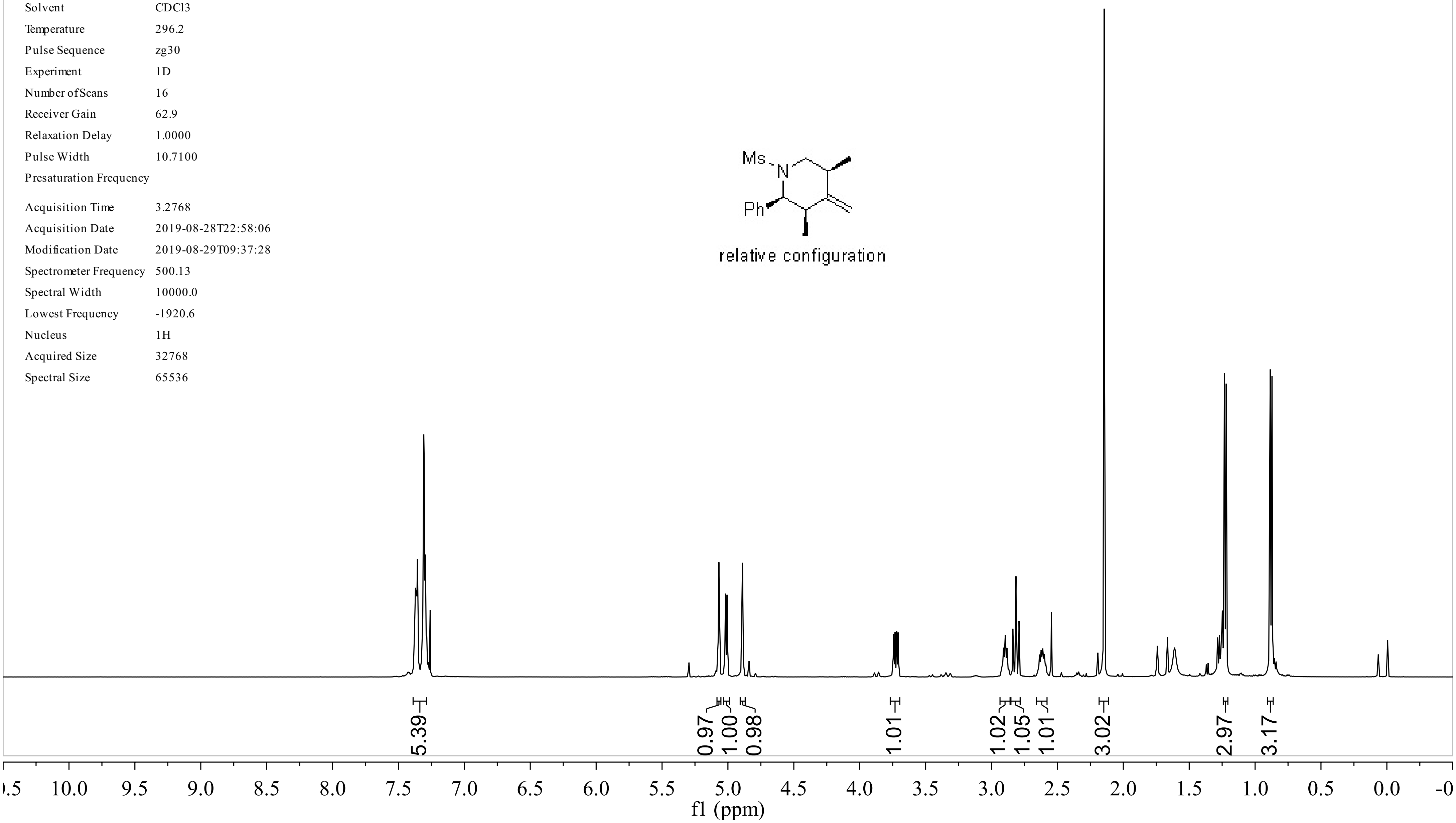

Parameter	Value
Title	xfy-190427-4-s.212.1.1r
Comment	
Origin	Bruker BioSpin GmbH
Owner	nmr
Site	
Instrument	spect
Solvent	CDCl3
Temperature	296.1
Pulse Sequence	zgpg30
Experiment	1D
Number of Scans	30
Receiver Gain	193.1
Relaxation Delay	2.0000
Pulse Width	9.6000
Presaturation Frequency	
Acquisition Time	1.1010
Acquisition Date	2019-08-28T23:00:52
Modification Date	2019-08-29T09:37:29
Spectrometer Frequency	125.76
Spectral Width	29761.9
Lowest Frequency	-2291.7
Nucleus	13C
Acquired Size	32768
Spectral Size	32768

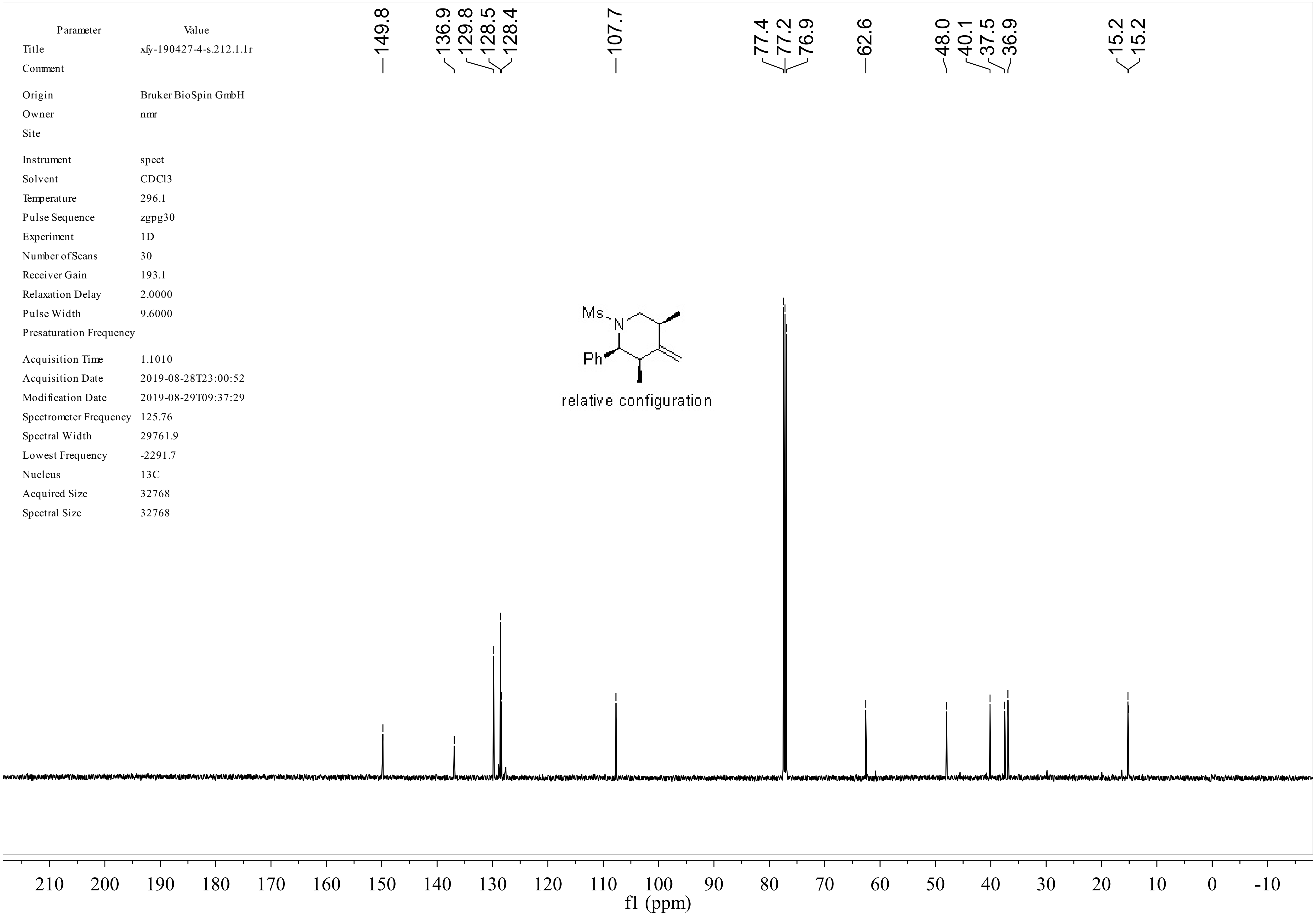

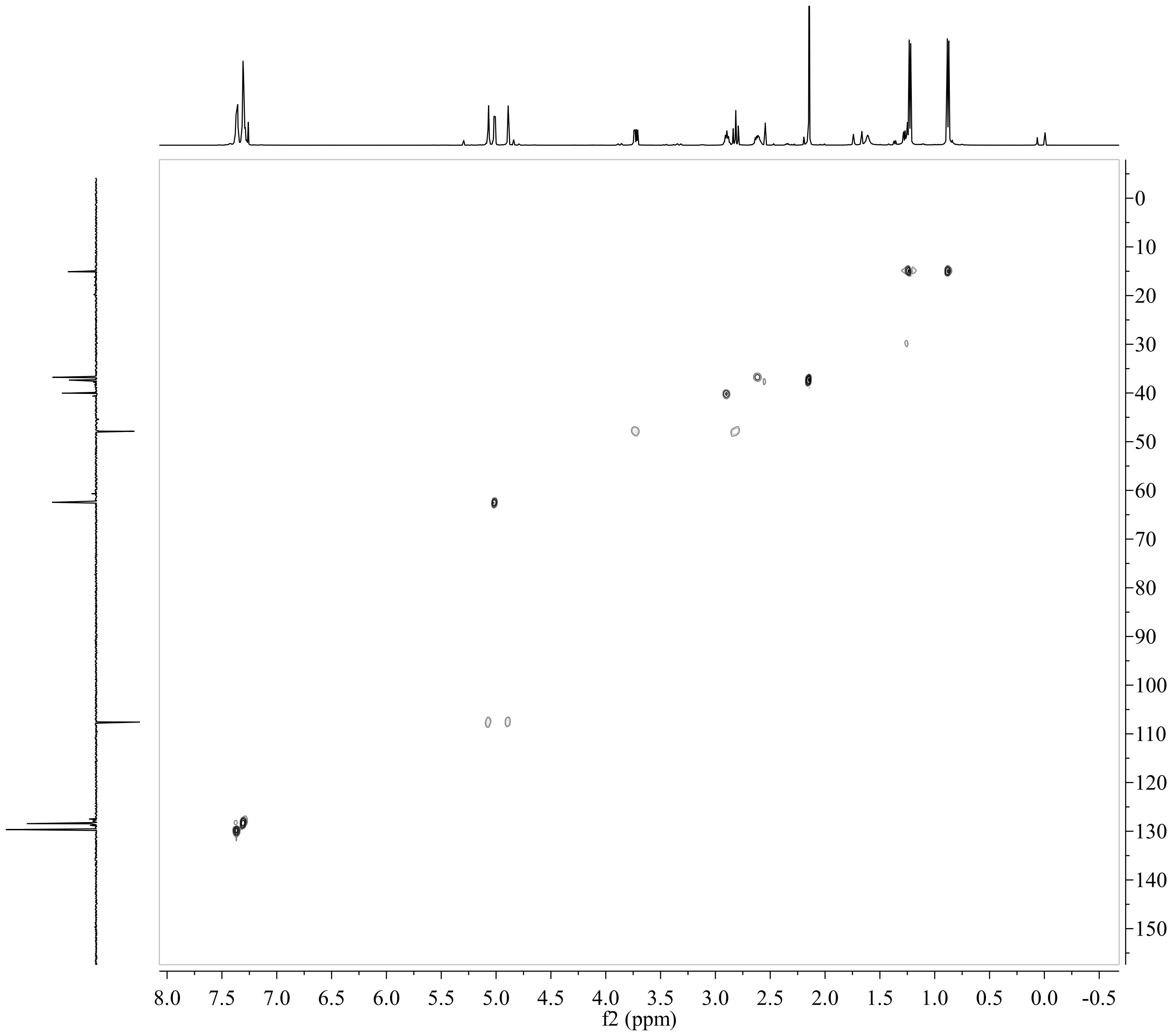

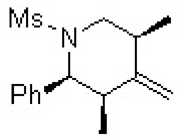

relative configuration

Parameter	Value
Title	xfy-190427-4-s.214.ser
Comment	
Origin	Bruker BioSpin GmbH
Owner	nmr
Site	
Instrument	spect
Solvent	CDCl3
Temperature	296.2
Pulse Sequence	hsqcedetgp
Experiment	HSQC-EDITED
Number of Scans	4
Receiver Gain	193.1
Relaxation Delay	1.4621
Pulse Width	10.7100
Presaturation Frequency	
Acquisition Time	0.1167
Acquisition Date	2019-08-28T23:05:39
Modification Date	2019-08-29T09:37:29
Spectrometer Frequency	(500.13, 125.77)
Spectral Width	(4386.0, 20833.3)
Lowest Frequency	(-349.6, -1037.0)
Nucleus	(1H, 13C)
Acquired Size	(512, 256)
Spectral Size	(512, 512)

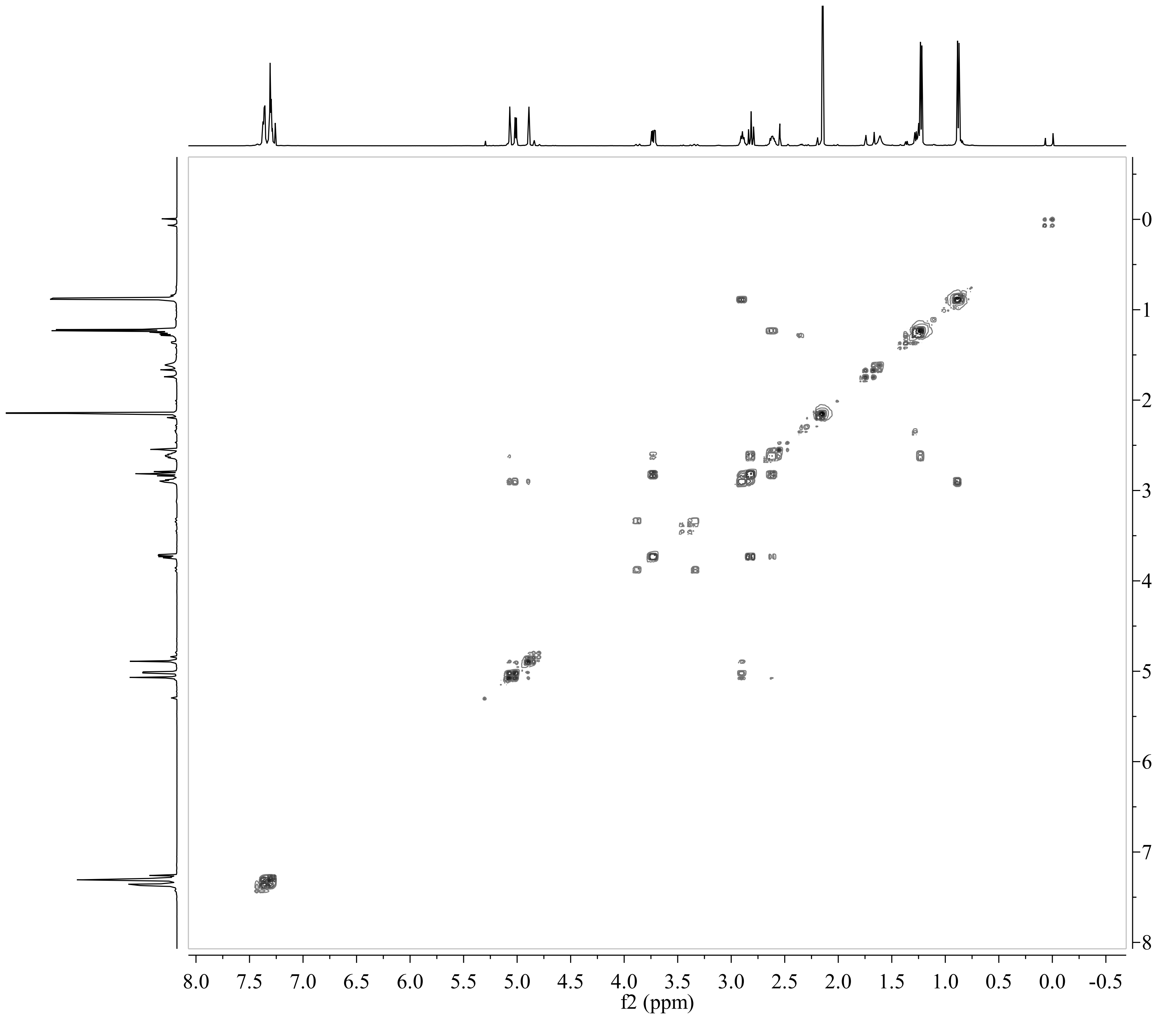

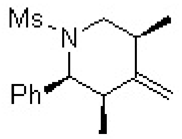

relative configuration

Parameter	Value
Title	xfy-190427-4-s.215.ser
Comment	
Origin	Bruker BioSpin GmbH
Owner	nmr
Site	
Instrument	spect
Solvent	CDCl3
Temperature	296.1
Pulse Sequence	cosygpppqf
Experiment	COSY
Number of Scans	4
Receiver Gain	44.7
Relaxation Delay	1.9197
Pulse Width	10.7100
Presaturation Frequency	
Acquisition Time	0.2335
Acquisition Date	2019-08-28T23:34:12
Modification Date	2019-08-29T09:37:31
Spectrometer Frequency	(500.13, 500.13)
Spectral Width	(4386.0, 4386.0)
Lowest Frequency	(-349.4, -349.4)
Nucleus	(1H, 1H)
Acquired Size	(1024, 128)
Spectral Size	(1024, 1024)

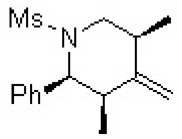

relative configuration

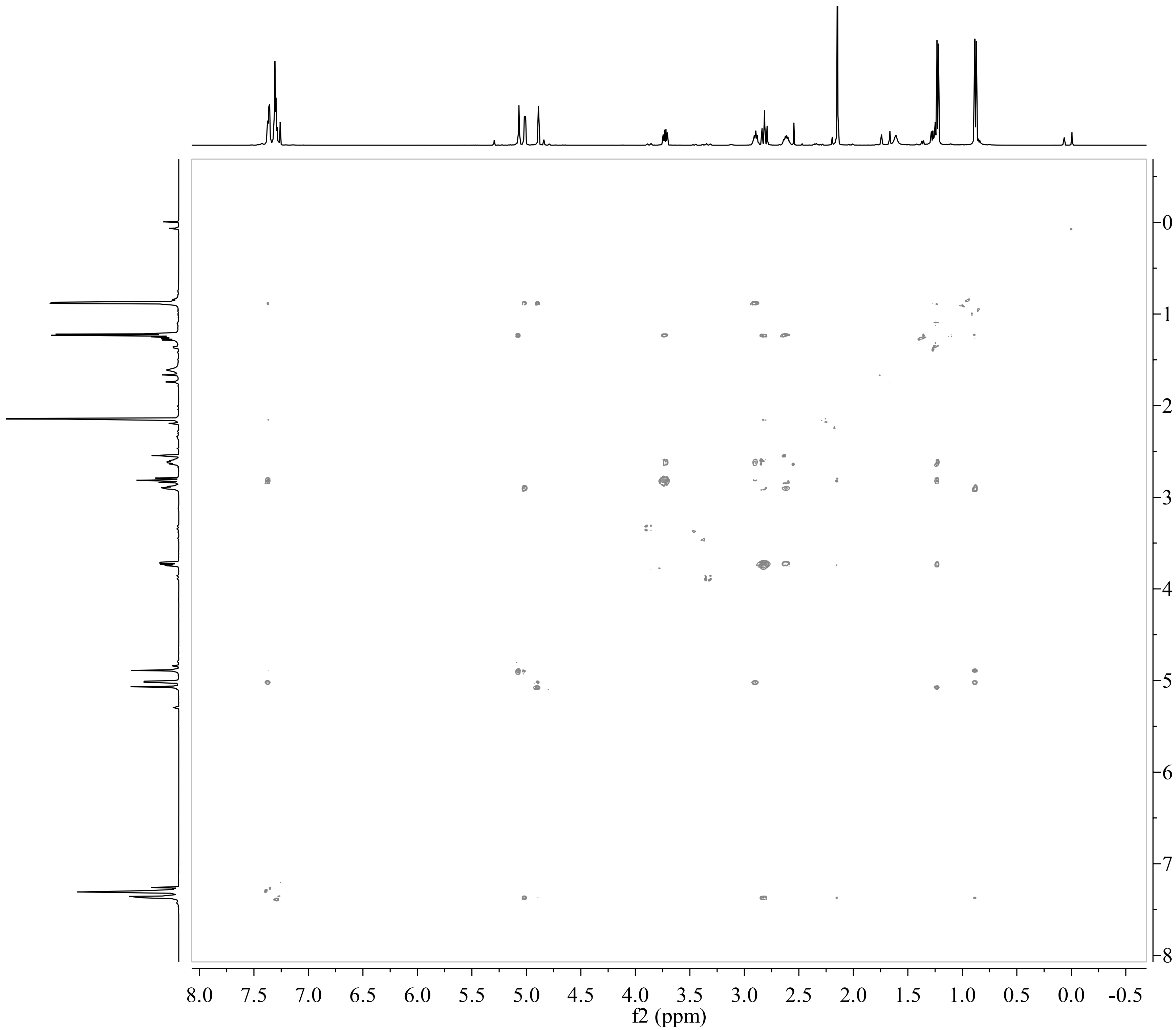

Parameter	Value
Title	xfy-190427-4-s.216.ser
Comment	
Origin	Bruker BioSpin GmbH
Owner	nmr
Site	
Instrument	spect
Solvent	CDCl3
Temperature	296.1
Pulse Sequence	noesygpphpp
Experiment	NOESY
Number of Scans	12
Receiver Gain	44.7
Relaxation Delay	1.9713
Pulse Width	10.7100
Presaturation Frequency	
Acquisition Time	0.2335
Acquisition Date	2019-08-28T23:55:41
Modification Date	2019-08-29T09:37:31
Spectrometer Frequency	(500.13, 500.13)
Spectral Width	(4386.0, 4386.0)
Lowest Frequency	(-349.4, -349.4)
Nucleus	(1H, 1H)
Acquired Size	(1024, 256)
Spectral Size	(1024, 1024)

Parameter	Value
Title	xfy-190413-2-s.21.1.1r
Comment	
Origin	Bruker BioSpin GmbH
Owner	nmr
Site	
Instrument	spect
Solvent	CDCl3
Temperature	296.1
Pulse Sequence	zg30
Experiment	1D
Number of Scans	4
Receiver Gain	108.2
Relaxation Delay	1.0000
Pulse Width	10.7100
Presaturation Frequency	
Acquisition Time	3.2768
Class	
Spectrometer Frequency	500.13
Spectral Width	10000.0
Lowest Frequency	-1911.5
Nucleus	1H
Acquired Size	32768
Spectral Size	65536

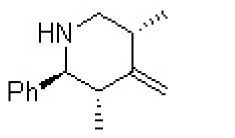

relative configuration

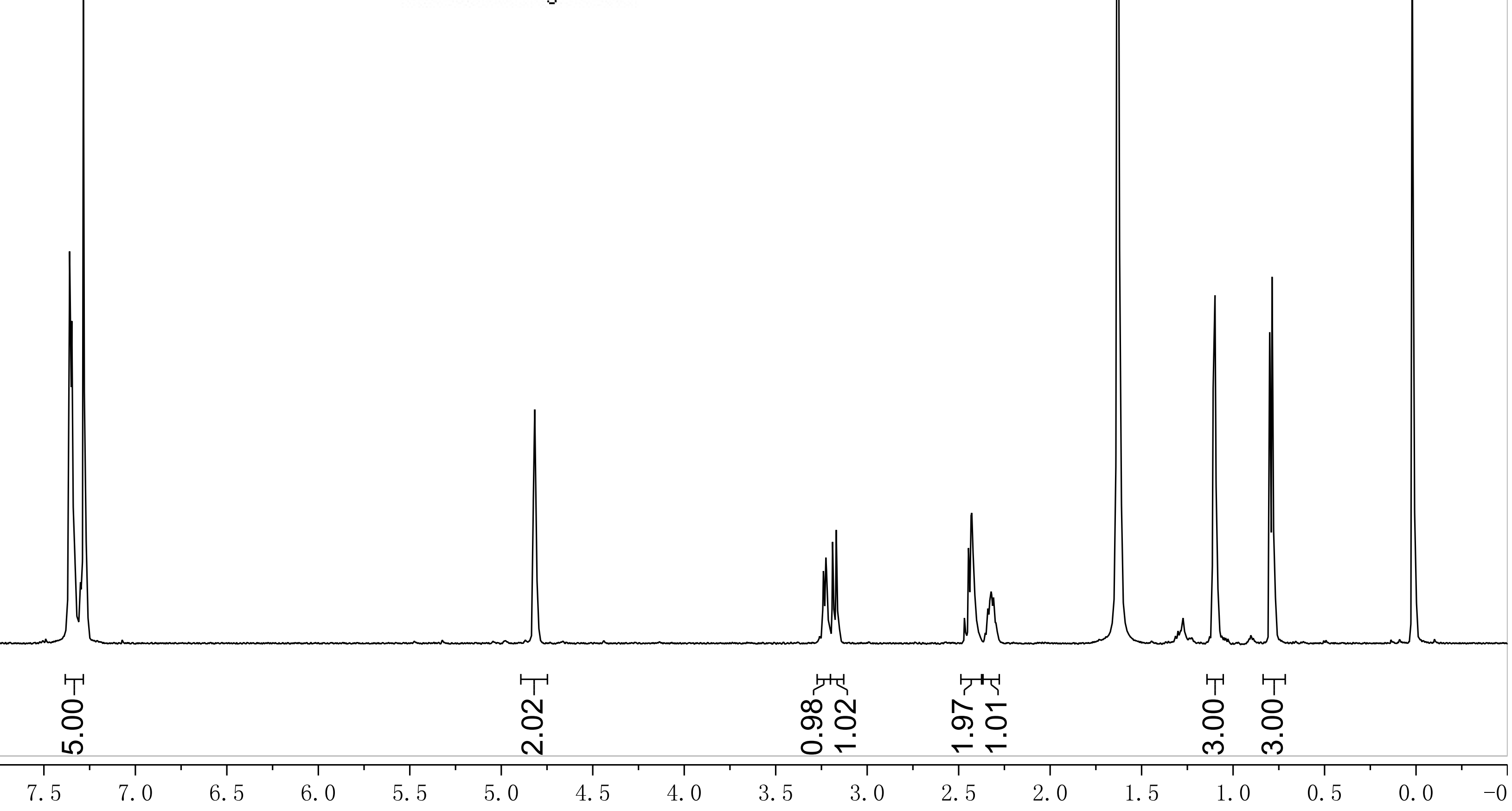

Parameter	Value
Title	xfy-190413-2-s.2.fid
Comment	
Origin	Bruker BioSpin GmbH
Owner	nmr
Site	
Instrument	spect
Solvent	CDCl3
Temperature	296.1
Pulse Sequence	zgpg30
Experiment	1D
Number of Scans	64
Receiver Gain	193.1
Relaxation Delay	2.0000
Pulse Width	9.6000
Presaturation Frequency	
Acquisition Time	1.1010
Class	
Spectrometer Frequency	125.77
Spectral Width	29761.9
Lowest Frequency	-2289.5
Nucleus	13C
Acquired Size	32768
Spectral Size	65536

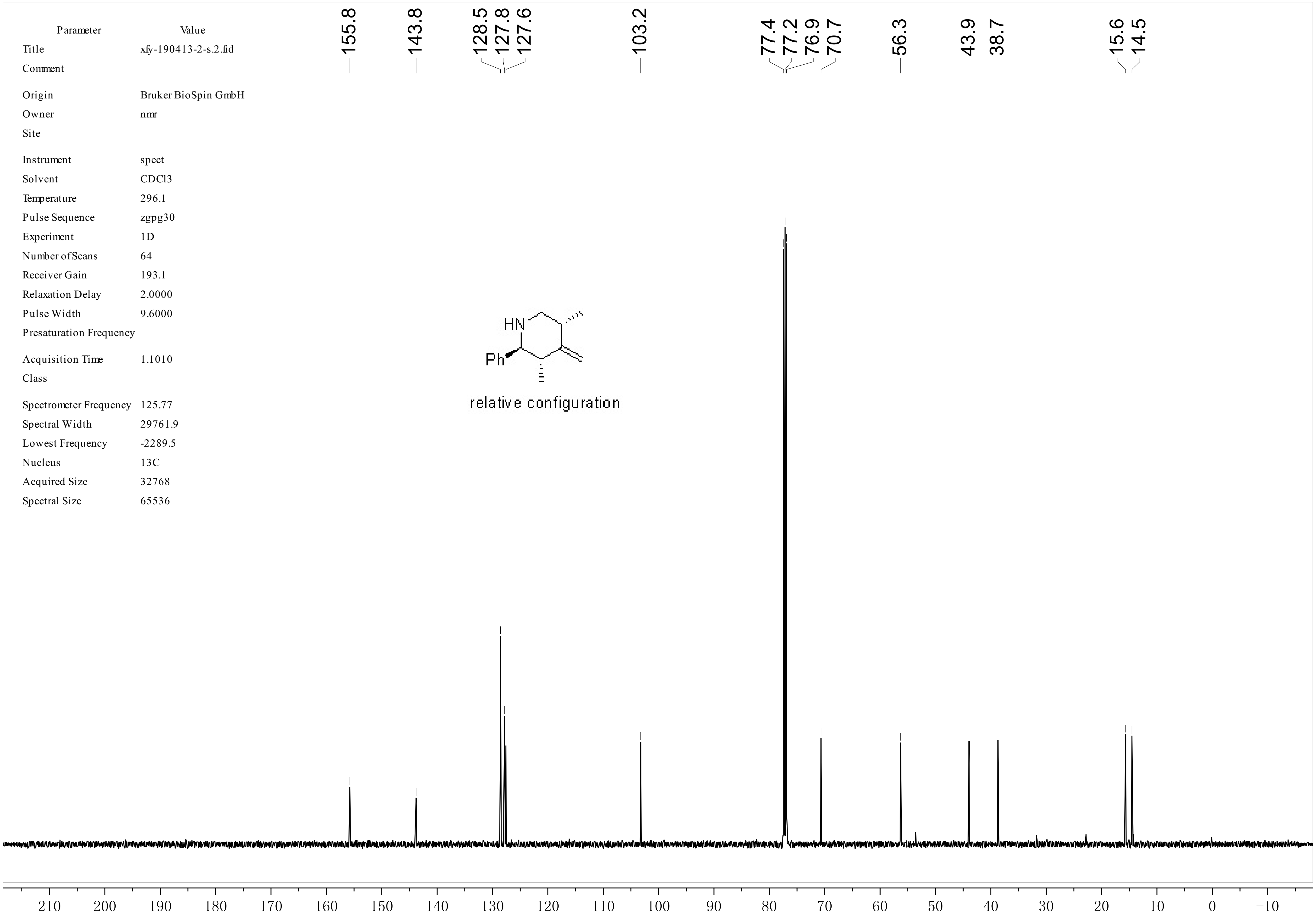

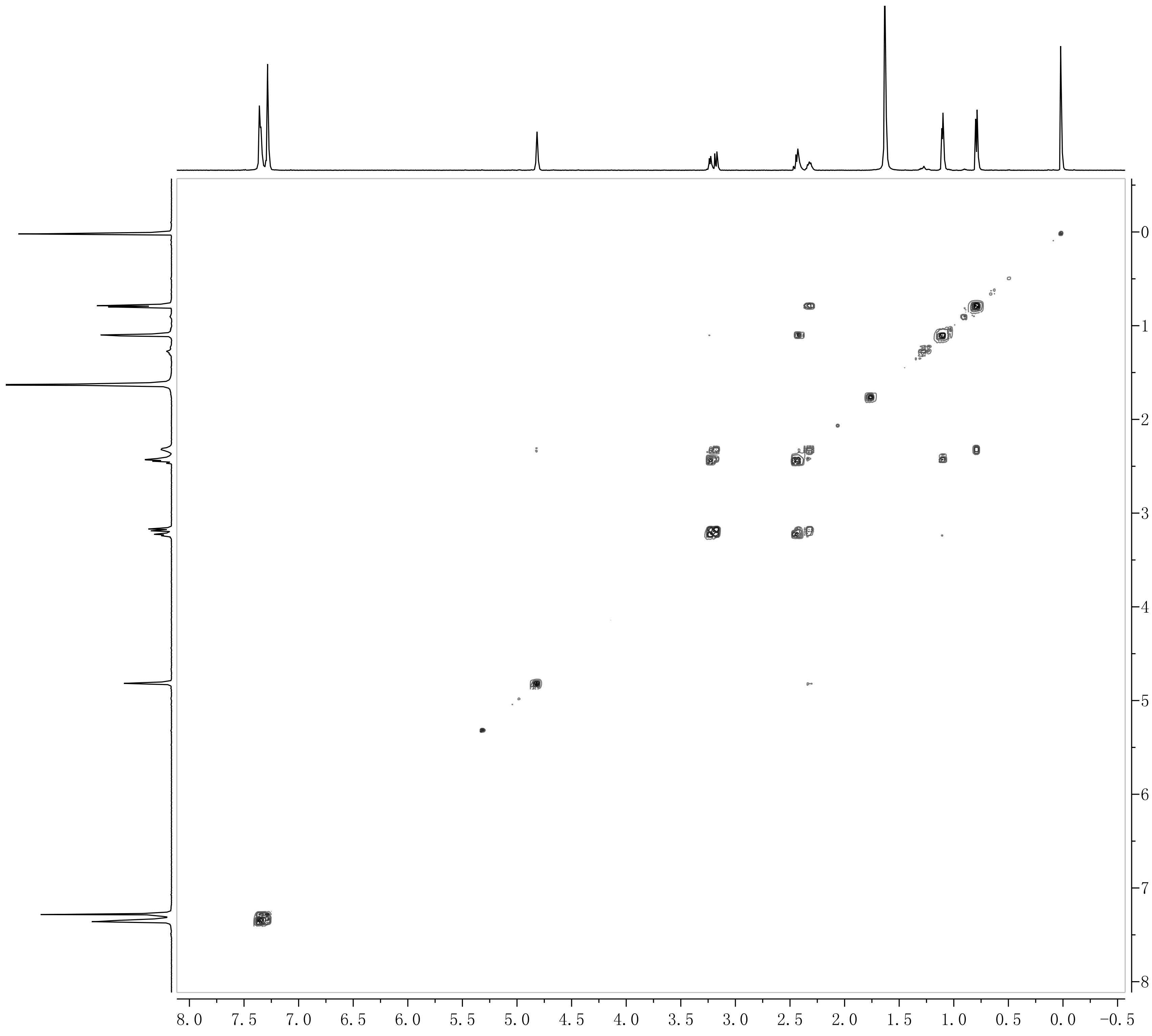

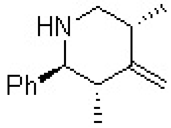

relative configuration

Parameter	Value
Title	xfy-190413-2-s.4.ser
Comment	
Origin	Bruker BioSpin GmbH
Owner	nmr
Site	
Instrument	spect
Solvent	CDCl3
Temperature	296.1
Pulse Sequence	cosygpppqf
Experiment	COSY
Number of Scans	1
Receiver Gain	54.3
Relaxation Delay	1.9177
Pulse Width	10.7100
Presaturation Frequency	
Acquisition Time	0.2355
Class	
Spectrometer Frequency	(500.13, 500.13)
Spectral Width	(4347.8, 4347.8)
Lowest Frequency	(-289.1, -289.1)
Nucleus	(1H, 1H)
Acquired Size	(1024, 128)
Spectral Size	(1024, 1024)

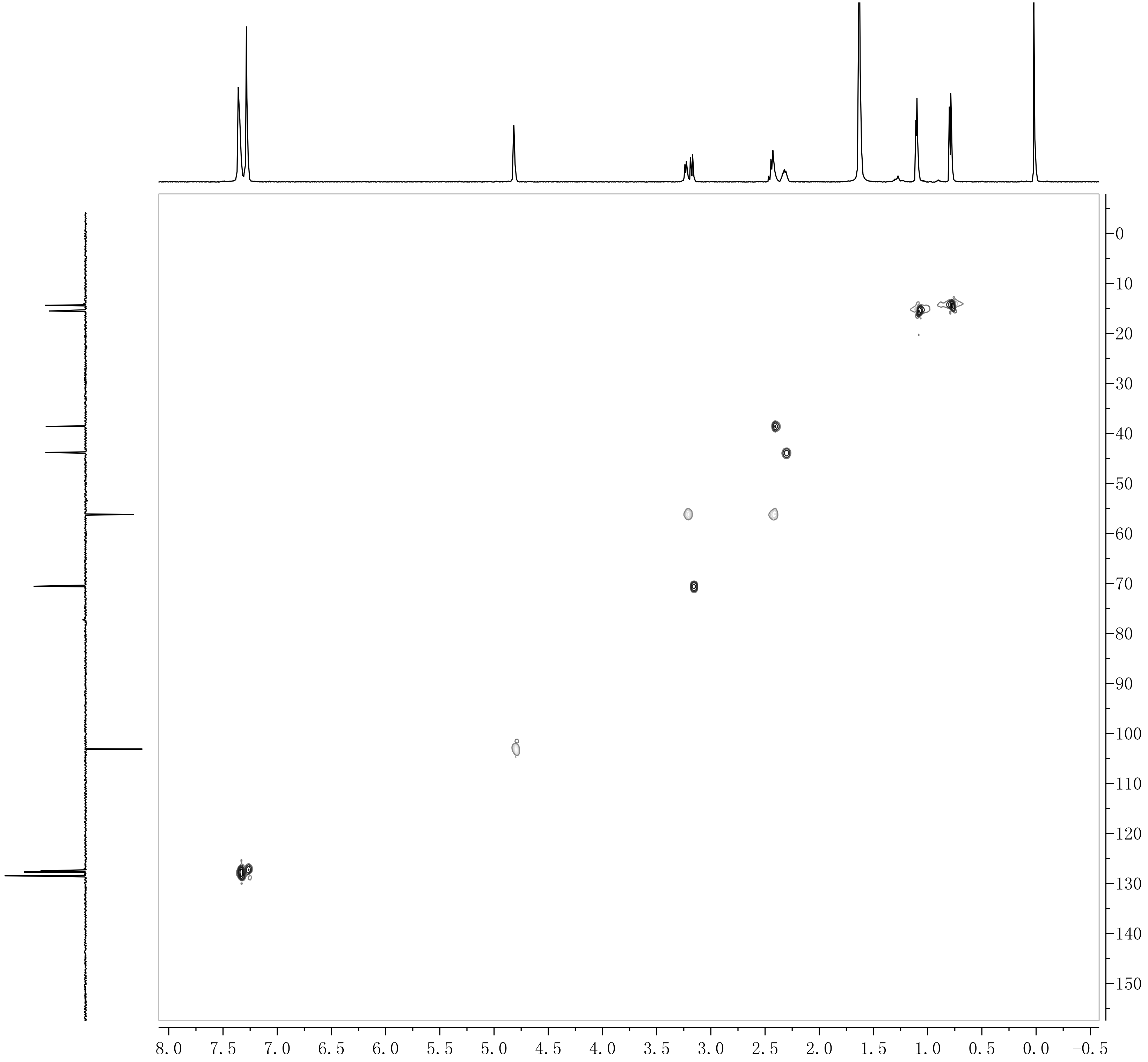

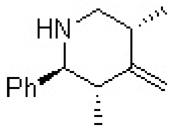

relative configuration

Parameter	Value
Title	xfy-190413-2-s.5.ser
Comment	
Origin	Bruker BioSpin GmbH
Owner	nmr
Site	
Instrument	spect
Solvent	CDCl3
Temperature	296.2
Pulse Sequence	hsqcedetgp
Experiment	HSQC-EDITED
Number of Scans	2
Receiver Gain	193.1
Relaxation Delay	1.4611
Pulse Width	10.7100
Presaturation Frequency	
Acquisition Time	0.1178
Class	
Spectrometer Frequency	(500.13, 125.77)
Spectral Width	(4347.8, 20833.3)
Lowest Frequency	(-299.5, -1037.0)
Nucleus	(1H, 13C)
Acquired Size	(512, 256)
Spectral Size	(512, 512)

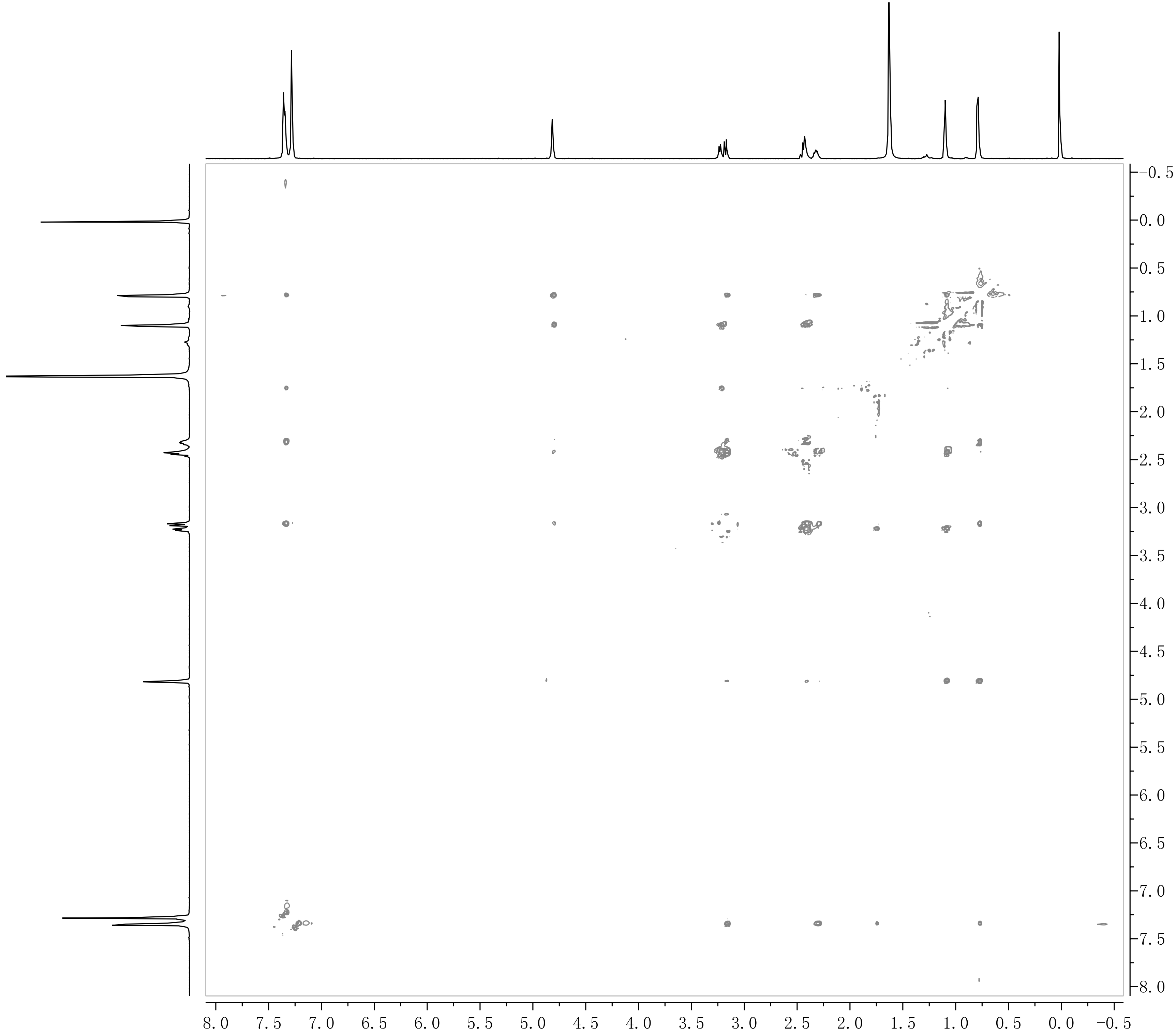

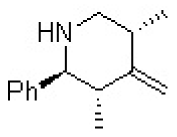

relative configuration

Parameter	Value
Title	xfy-190413-2-s.6.ser
Comment	
Origin	Bruker BioSpin GmbH
Owner	nmr
Site	
Instrument	spect
Solvent	CDCl3
Temperature	296.2
Pulse Sequence	noesygpphpp
Experiment	NOESY
Number of Scans	8
Receiver Gain	54.3
Relaxation Delay	1.9693
Pulse Width	10.7100
Presaturation Frequency	
Acquisition Time	0.2355
Class	
Spectrometer Frequency	(500.13, 500.13)
Spectral Width	(4347.8, 4347.8)
Lowest Frequency	(-298.4, -298.4)
Nucleus	(1H, 1H)
Acquired Size	(1024, 256)
Spectral Size	(1024, 1024)

Parameter	Value
Title	xfy-180821-0.1.fid
Comment	
Origin	Bruker BioSpin GmbH
Owner	nmr
Site	
Instrument	spect
Solvent	CDCl3
Temperature	296.1
Pulse Sequence	zg30
Experiment	1D
Number of Scans	8
Receiver Gain	31.1
Relaxation Delay	1.0000
Pulse Width	10.7100
Presaturation Frequency	
Acquisition Time	3.2768
Class	
Spectrometer Frequency	500.13
Spectral Width	10000.0
Lowest Frequency	-1929.4
Nucleus	1H
Acquired Size	32768
Spectral Size	65536

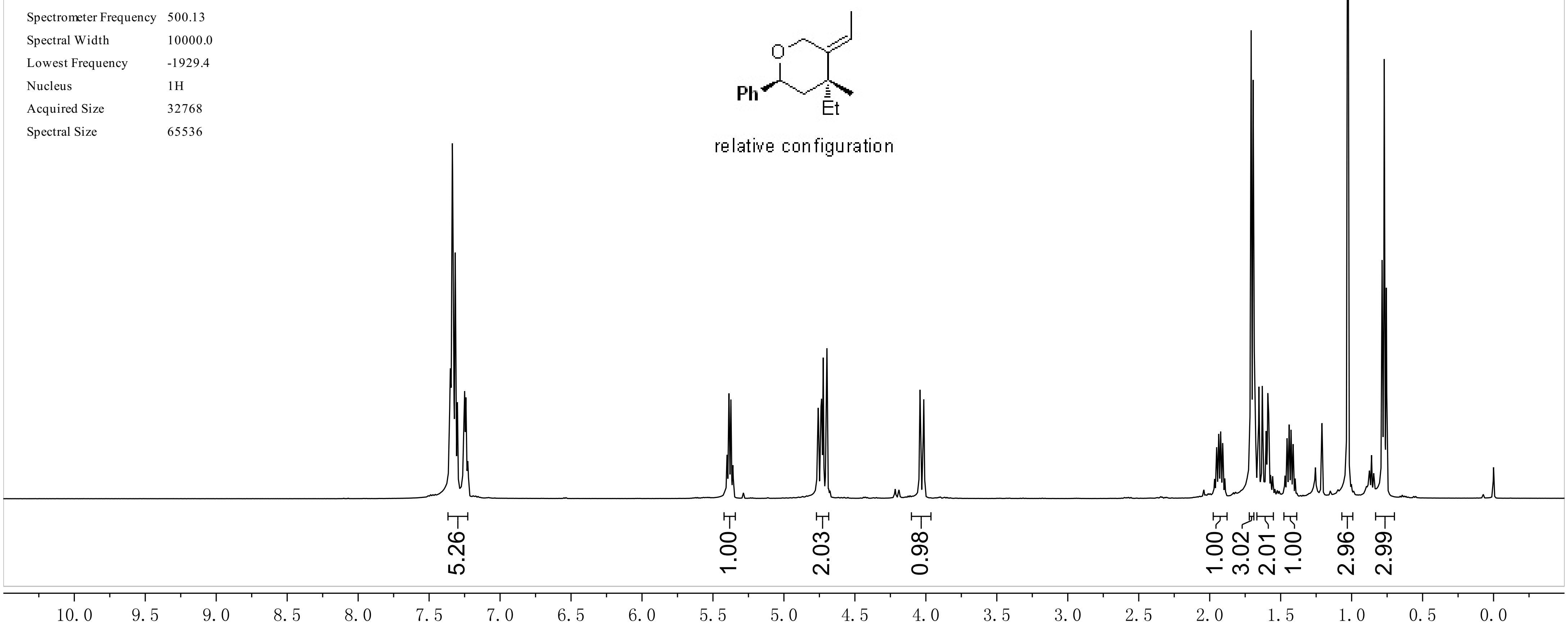

Parameter	Value
Title	xfy-180821-0.2.fid
Comment	
Origin	Bruker BioSpin GmbH
Owner	nmr
Site	
Instrument	spect
Solvent	CDCl3
Temperature	296.2
Pulse Sequence	zgpg30
Experiment	1D
Number of Scans	9
Receiver Gain	193.1
Relaxation Delay	2.0000
Pulse Width	9.6000
Presaturation Frequency	
Acquisition Time	1.1010
Class	
Spectrometer Frequency	125.77
Spectral Width	29761.9
Lowest Frequency	-2291.9
Nucleus	13C
Acquired Size	32768
Spectral Size	65536

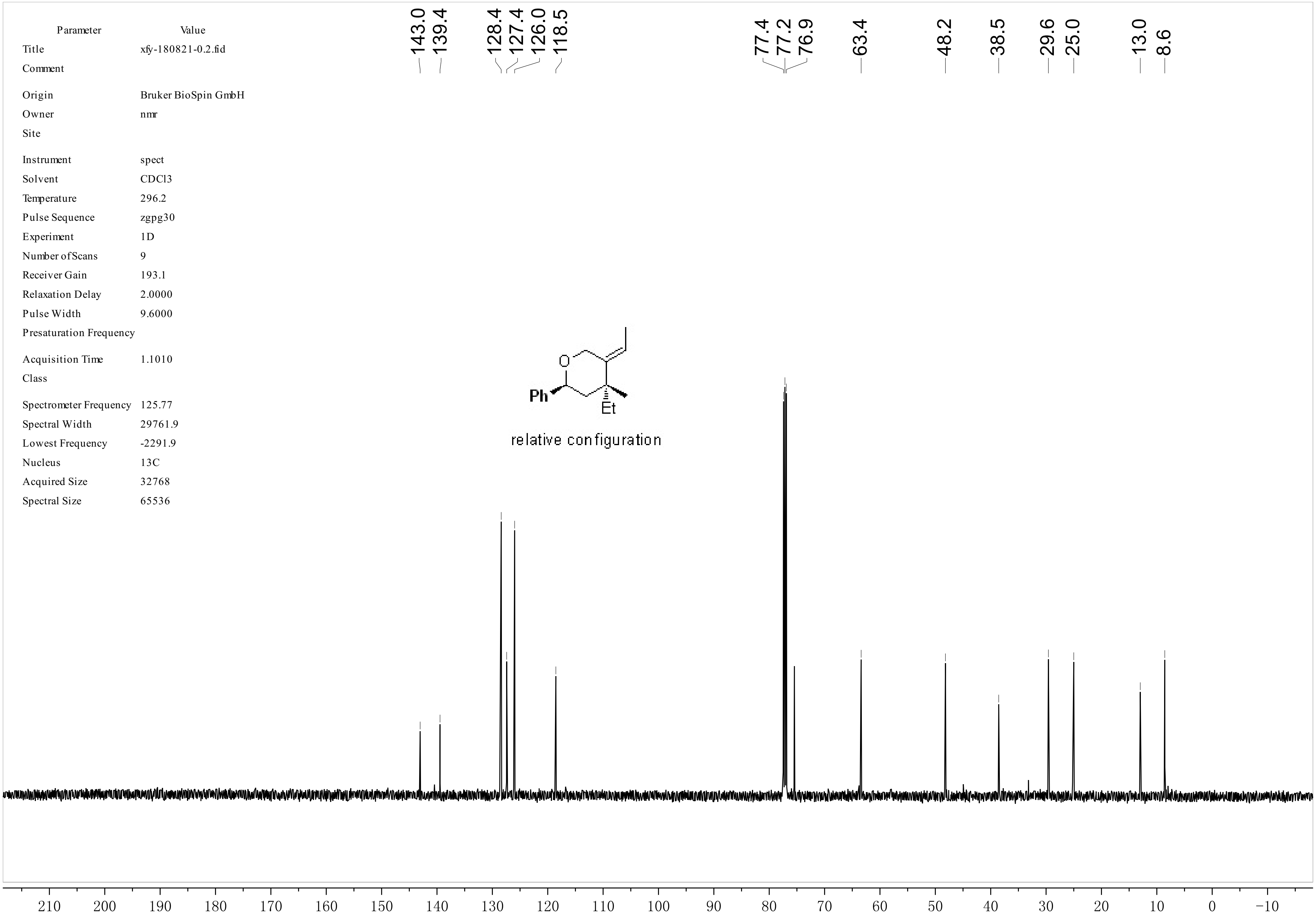

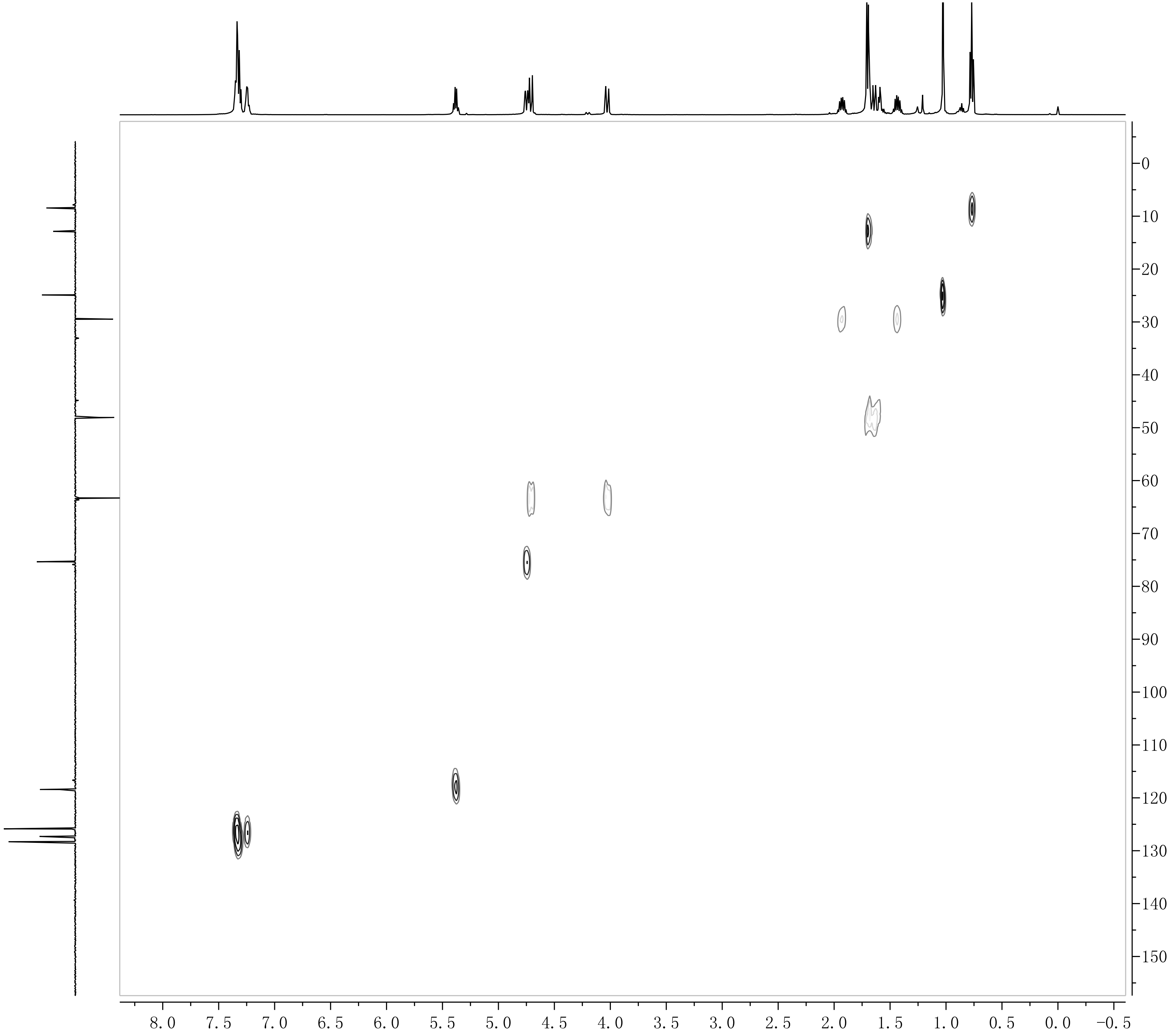

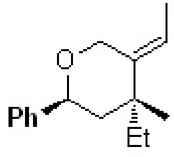

relative configuration

Parameter	Value
Title	xfy-180821-0.4.ser
Comment	
Origin	Bruker BioSpin GmbH
Owner	nmr
Site	
Instrument	spect
Solvent	CDCl3
Temperature	296.1
Pulse Sequence	hsqcedetgp
Experiment	HSQC-EDITED
Number of Scans	2
Receiver Gain	193.1
Relaxation Delay	1.4652
Pulse Width	10.7100
Presaturation Frequency	
Acquisition Time	0.1137
Class	
Spectrometer Frequency	(500.13, 125.77)
Spectral Width	(4504.5, 20833.3)
Lowest Frequency	(-311.4, -1037.0)
Nucleus	(1H, 13C)
Acquired Size	(512, 72)
Spectral Size	(512, 512)

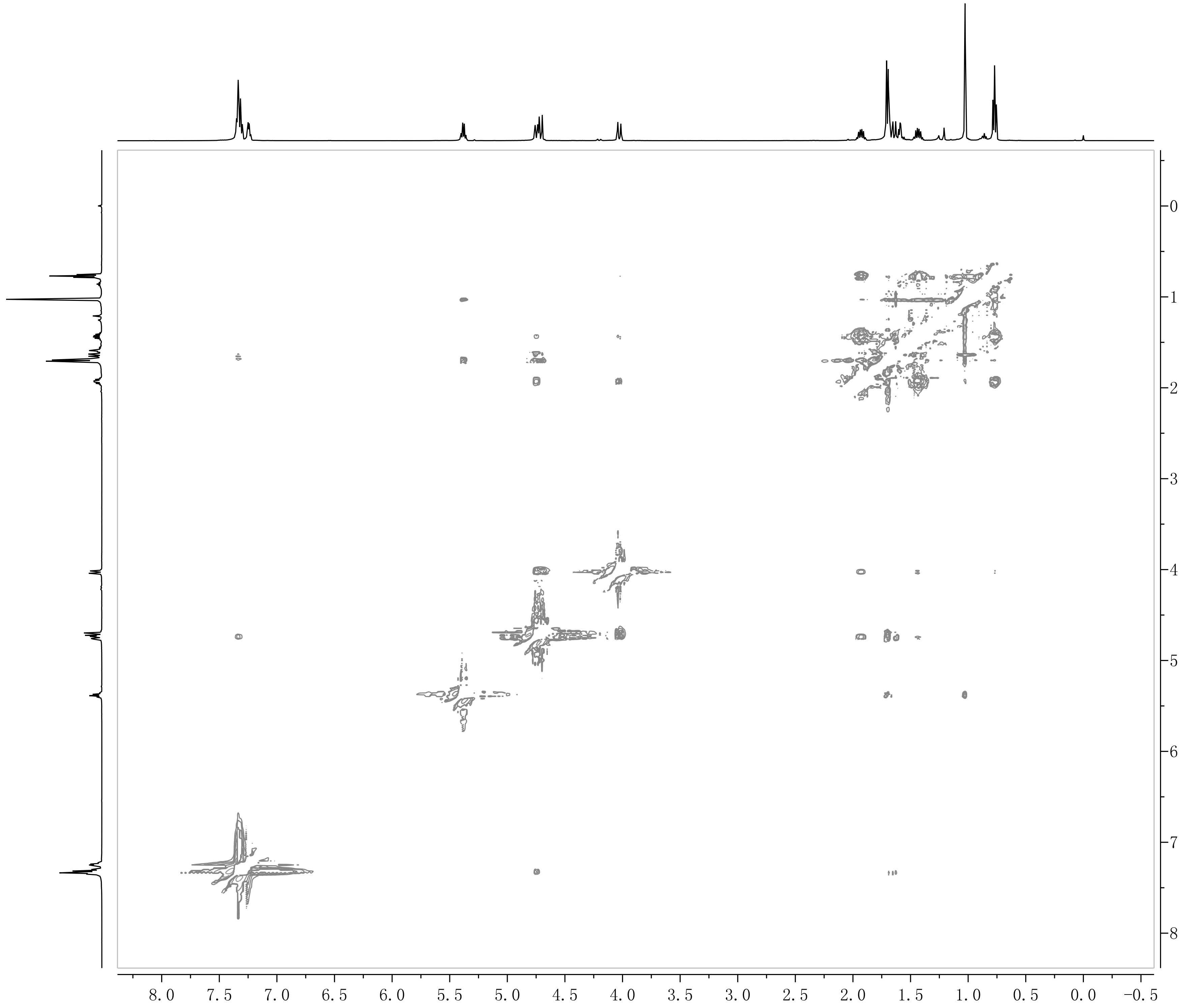

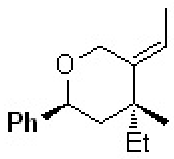

relative configuration

Parameter	Value
Title	xfy-180821-0.5.ser
Comment	
Origin	Bruker BioSpin GmbH
Owner	nmr
Site	
Instrument	spect
Solvent	CDCl3
Temperature	296.2
Pulse Sequence	noesygpphpp
Experiment	NOESY
Number of Scans	4
Receiver Gain	21.9
Relaxation Delay	1.9775
Pulse Width	10.7100
Presaturation Frequency	
Acquisition Time	0.2273
Class	
Spectrometer Frequency	(500.13, 500.13)
Spectral Width	(4504.5, 4504.5)
Lowest Frequency	(-311.4, -311.4)
Nucleus	(1H, 1H)
Acquired Size	(1024, 173)
Spectral Size	(1024, 1024)

Parameter	Value
Title	xfy-180914-2-s.2.fid
Comment	
Origin	Bruker BioSpin GmbH
Owner	nmr
Site	
Instrument	spect
Solvent	CDCl3
Temperature	296.2
Pulse Sequence	zg30
Experiment	1D
Number of Scans	4
Receiver Gain	62.9
Relaxation Delay	1.0000
Pulse Width	10.7100
Presaturation Frequency	
Acquisition Time	3.2768
Acquisition Date	2018-09-18T12:38:00
Modification Date	2018-09-19T09:24:10
Spectrometer Frequency	500.13
Spectral Width	10000.0
Lowest Frequency	-1922.5
Nucleus	1H
Acquired Size	32768
Spectral Size	65536

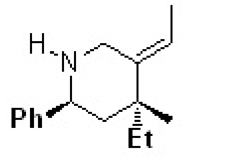

relative configuration

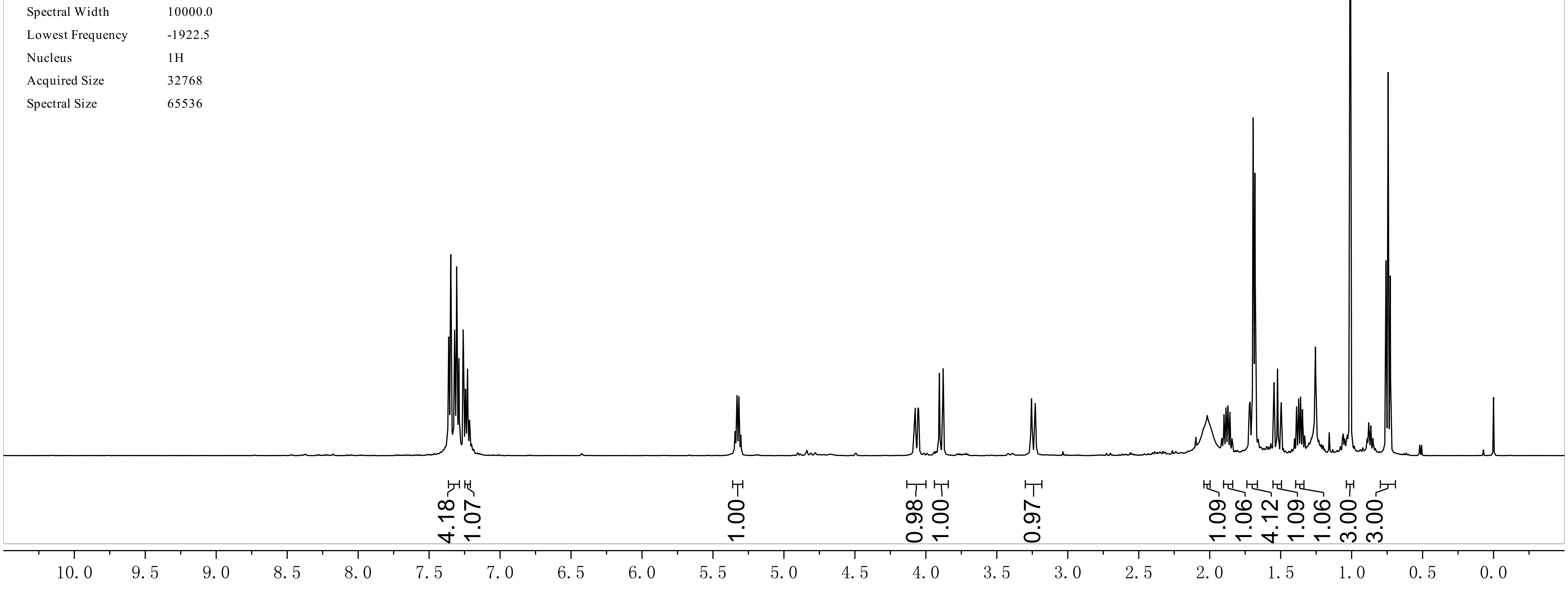

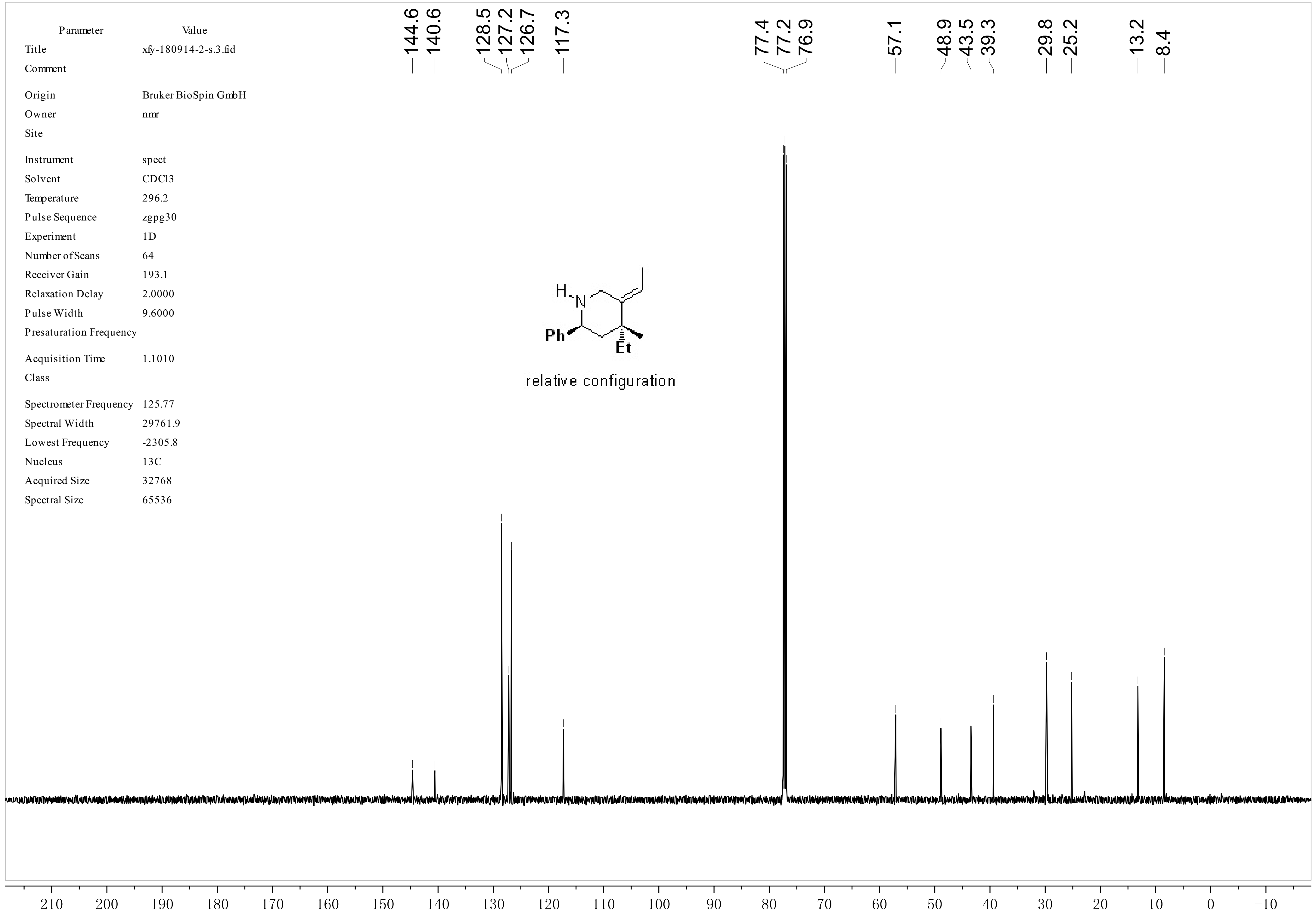
Parameter Value
Title xfy-180914-2-s.3.fid
Comment
Origin Bruker BioSpin GmbH
Owner nmr
Site
Instrument spect
Solvent CDCl3
Temperature 296.2
Pulse Sequence zgpg30
Experiment 1D
Number of Scans 64
Receiver Gain 193.1
Relaxation Delay 2.0000
Pulse Width 9.6000
Presaturation Frequency
Acquisition Time 1.1010
Class
Spectrometer Frequency 125.77
Spectral Width 29761.9
Lowest Frequency -2305.8
Nucleus 13C
Acquired Size 32768
Spectral Size 65536
144.6
140.6
128.5
127.2
126.7
117.3
77.4
77.2
76.9
57.1
48.9
43.5
39.3
29.8
25.2
13.2
8.4
H
N
Ph
Et
relative configuration
210 200 190 180 170 160 150 140 130 120 110 100 90 80 70 60 50 40 30 20 10 0 −10

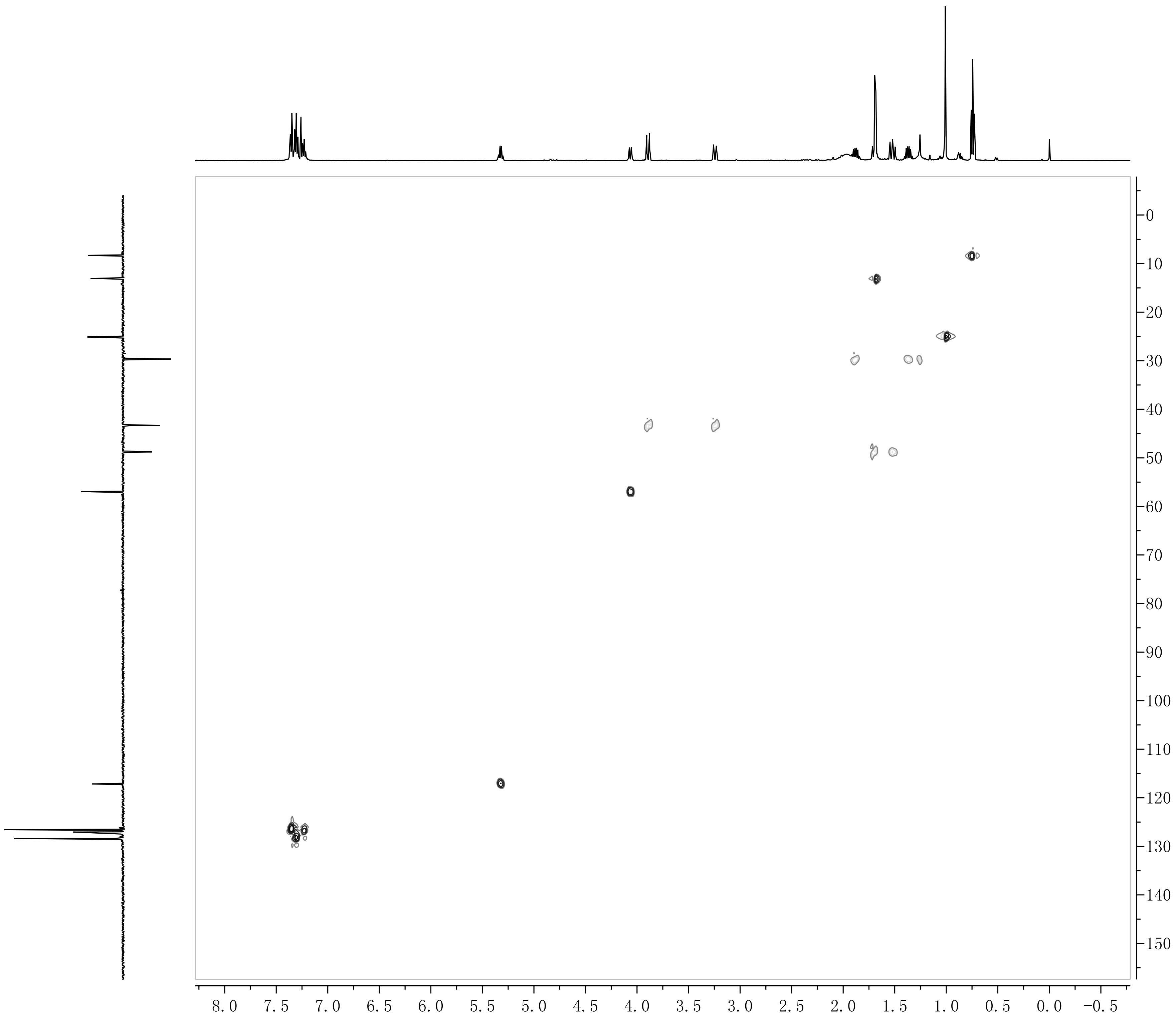

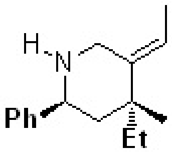

relative configuration

Parameter	Value
Title	xfy-180914-2-s.5.ser
Comment	
Origin	Bruker BioSpin GmbH
Owner	nmr
Site	
Instrument	spect
Solvent	CDCl3
Temperature	296.2
Pulse Sequence	hsqcedetgp
Experiment	HSQC-EDITED
Number of Scans	2
Receiver Gain	193.1
Relaxation Delay	1.4662
Pulse Width	10.7100
Presaturation Frequency	
Acquisition Time	0.1126
Class	
Spectrometer Frequency	(500.13, 125.77)
Spectral Width	(4545.5, 20833.3)
Lowest Frequency	(-401.8, -1037.0)
Nucleus	(1H, 13C)
Acquired Size	(512, 256)
Spectral Size	(512, 512)

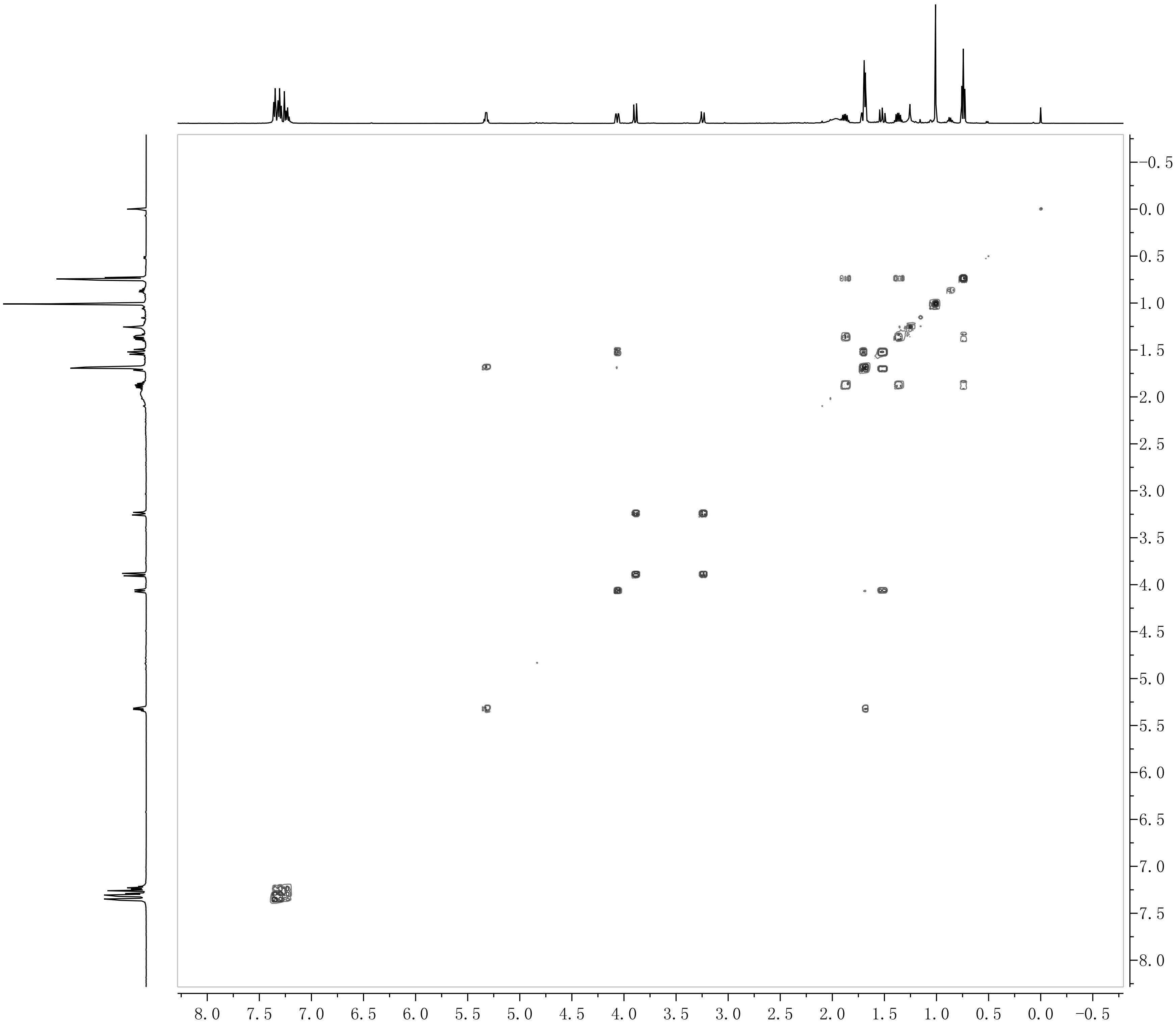

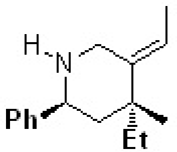

relative configuration

Parameter	Value
Title	xfy-180914-2-s.6.ser
Comment	
Origin	Bruker BioSpin GmbH
Owner	nmr
Site	
Instrument	spect
Solvent	CDCl3
Temperature	296.1
Pulse Sequence	cosygpppqf
Experiment	COSY
Number of Scans	1
Receiver Gain	27.5
Relaxation Delay	1.9279
Pulse Width	10.7100
Presaturation Frequency	
Acquisition Time	0.2253
Class	
Spectrometer Frequency	(500.13, 500.13)
Spectral Width	(4545.5, 4545.5)
Lowest Frequency	(-401.8, -401.8)
Nucleus	(1H, 1H)
Acquired Size	(1024, 128)
Spectral Size	(1024, 1024)

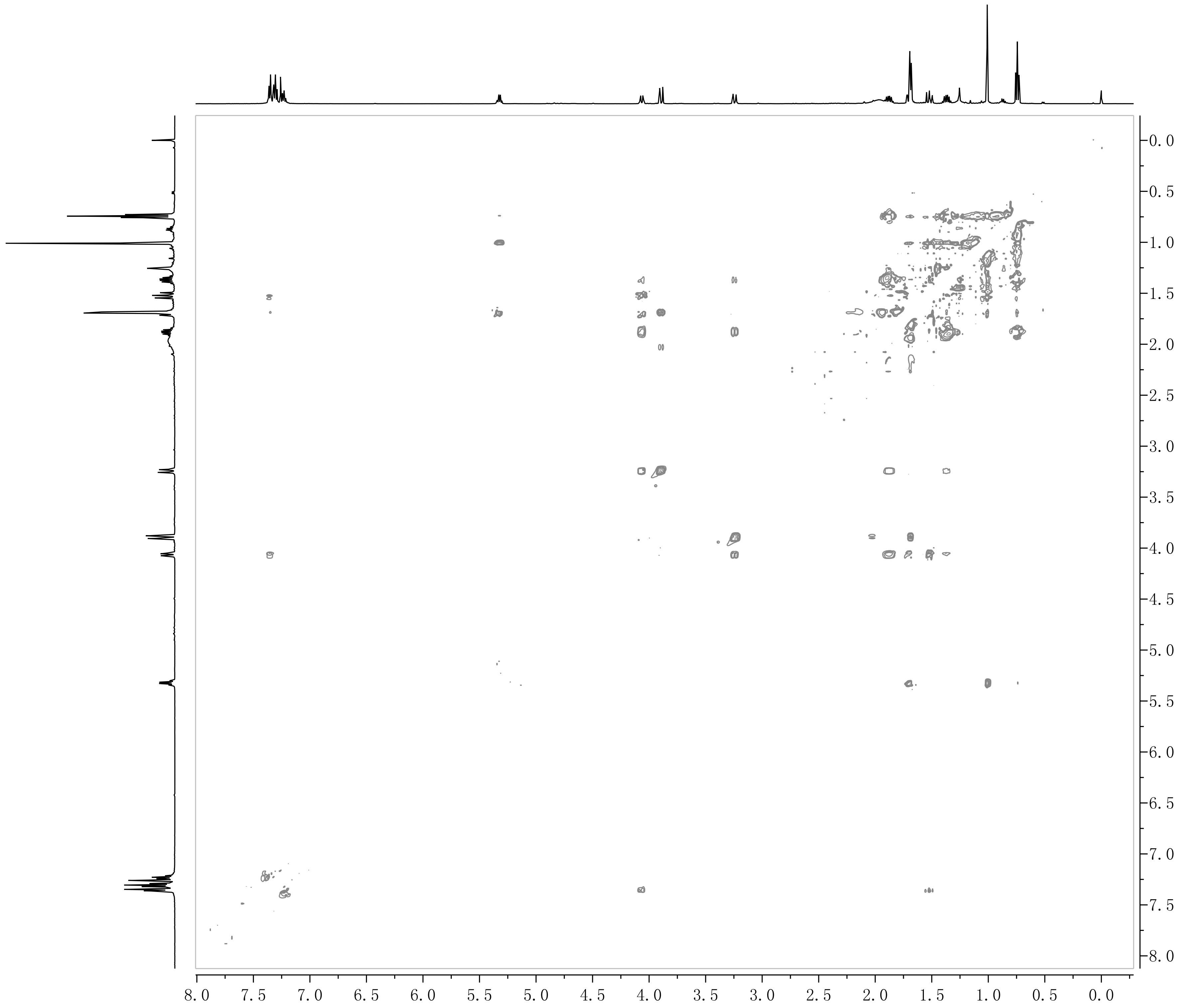

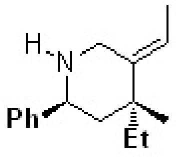

relative configuration

Parameter	Value
Title	xfy-180914-2-s.22.ser
Comment	
Origin	Bruker BioSpin GmbH
Owner	nmr
Site	
Instrument	spect
Solvent	CDCl3
Temperature	296.2
Pulse Sequence	noesygpphpp
Experiment	NOESY
Number of Scans	8
Receiver Gain	45.6
Relaxation Delay	2.0205
Pulse Width	10.0000
Presaturation Frequency	
Acquisition Time	0.2355
Class	
Spectrometer Frequency	(400.13, 400.13)
Spectral Width	(4347.8, 4347.8)
Lowest Frequency	(-672.3, -672.3)
Nucleus	(1H, 1H)
Acquired Size	(1024, 256)
Spectral Size	(1024, 1024)

Parameter	Value
标题	xfy-180827-6-s.1.fid
Comment	
Origin	Bruker BioSpin GmbH
Owner	nmr
Site	
Instrument	spect
Author	
Solvent	CDCl3
Temperature	296.1
Pulse Sequence	zg30
Experiment	1D
Number of Scans	9
Receiver Gain	31.1
Relaxation Delay	1.0000
Pulse Width	10.7100
Presaturation Frequency	
Acquisition Time	3.2768
Class	
Spectrometer Frequency	500.13
Spectral Width	10000.0
Lowest Frequency	-1924.1
Nucleus	1H
Acquired Size	32768
Spectral Size	65536

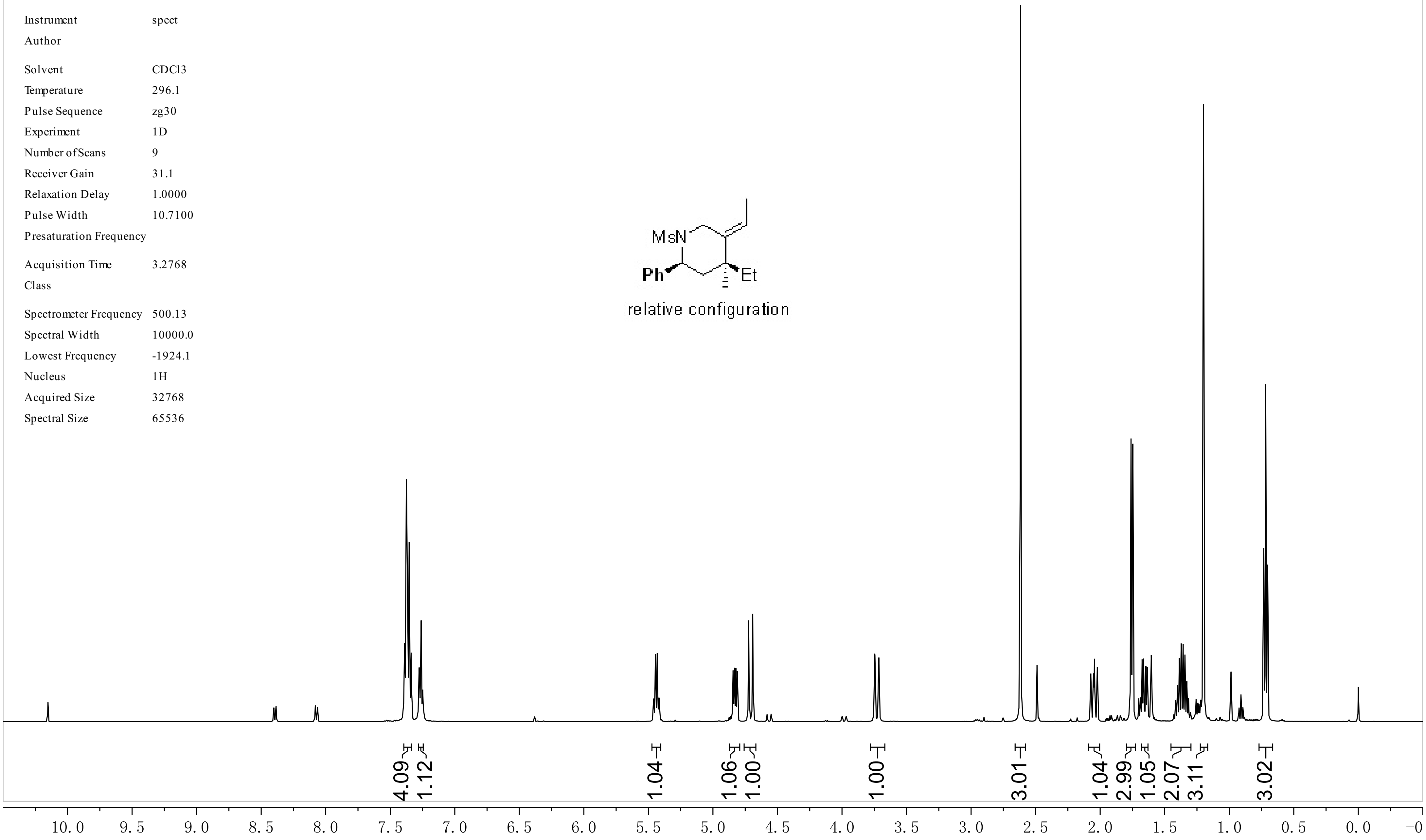

Parameter	Value
标题	xfy-180827-6-s.2.fid
Comment	
Origin	Bruker BioSpin GmbH
Owner	nmr
Site	
Instrument	spect
Author	
Solvent	CDCl3
Temperature	296.1
Pulse Sequence	zgpg30
Experiment	1D
Number of Scans	5
Receiver Gain	193.1
Relaxation Delay	2.0000
Pulse Width	9.6000
Presaturation Frequency	
Acquisition Time	1.1010
Class	
Spectrometer Frequency	125.77
Spectral Width	29761.9
Lowest Frequency	-2305.8
Nucleus	13C
Acquired Size	32768
Spectral Size	65536

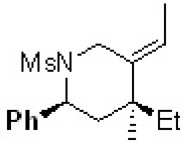

relative configuration

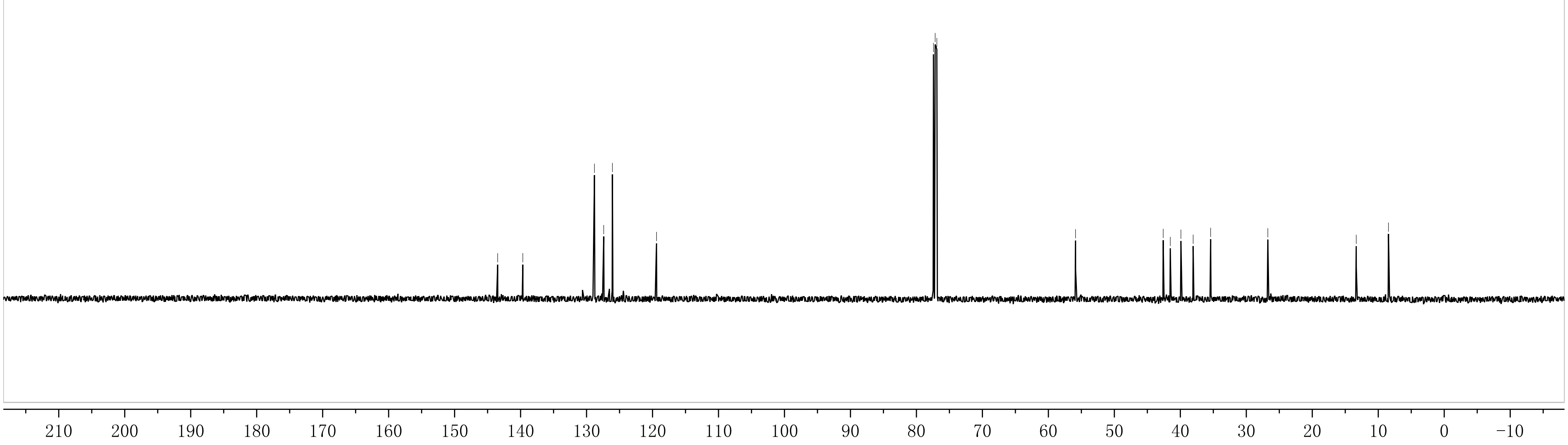

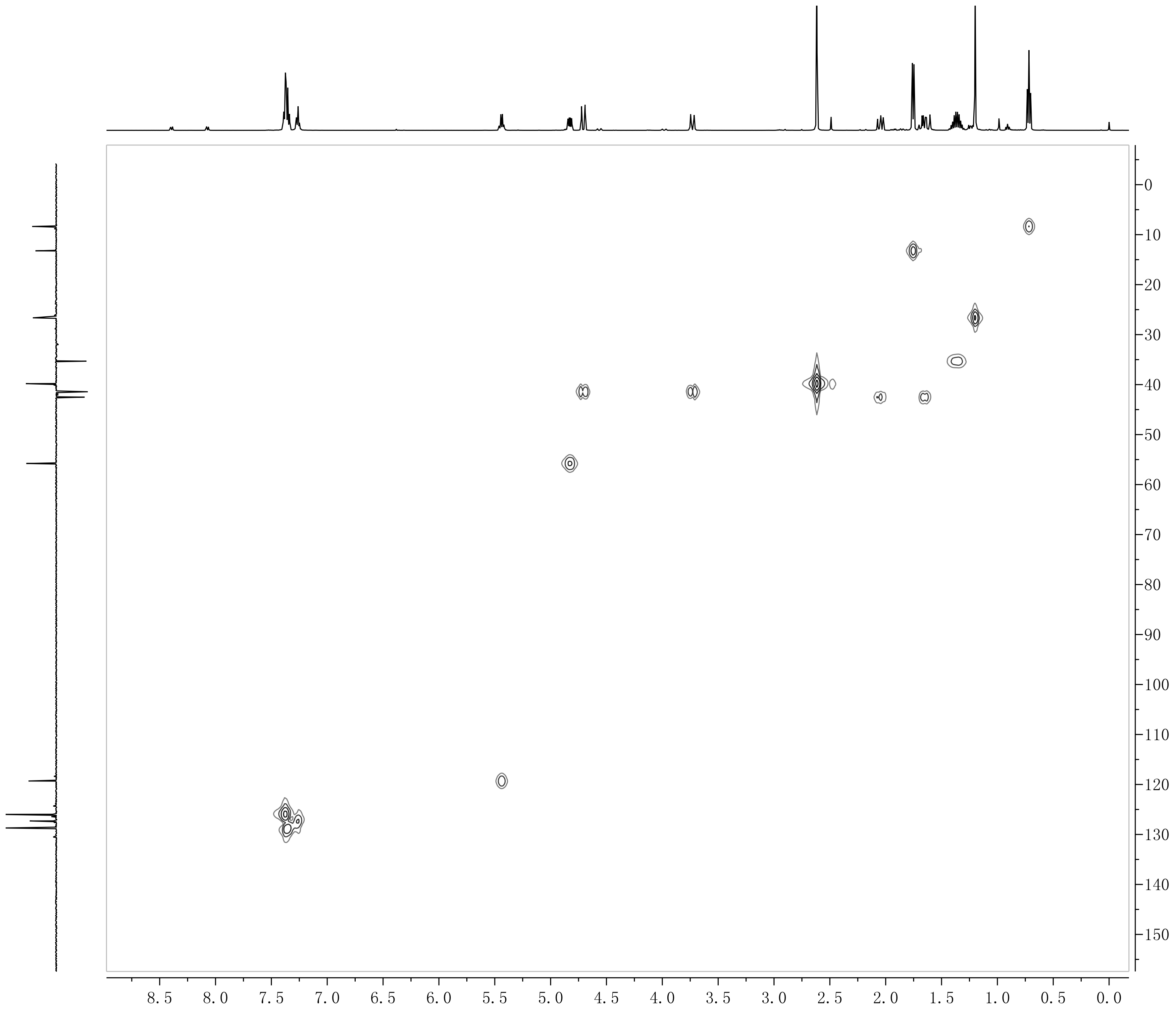

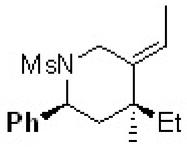

relative configuration

Parameter	Value
标题	xfy-180827-6-s.4.ser
Comment	
Origin	Bruker BioSpin GmbH
Owner	nmr
Site	
Instrument	spect
Author	
Solvent	CDCl3
Temperature	296.1
Pulse Sequence	hsqcedetgp
Experiment	HSQC-EDITED
Number of Scans	2
Receiver Gain	193.1
Relaxation Delay	1.4672
Pulse Width	10.7100
Presaturation Frequency	
Acquisition Time	0.1116
Class	
Spectrometer Frequency	(500.13, 125.77)
Spectral Width	(4587.2, 20833.3)
Lowest Frequency	(-97.9, -1037.0)
Nucleus	(1H, 13C)
Acquired Size	(512, 256)
Spectral Size	(512, 512)

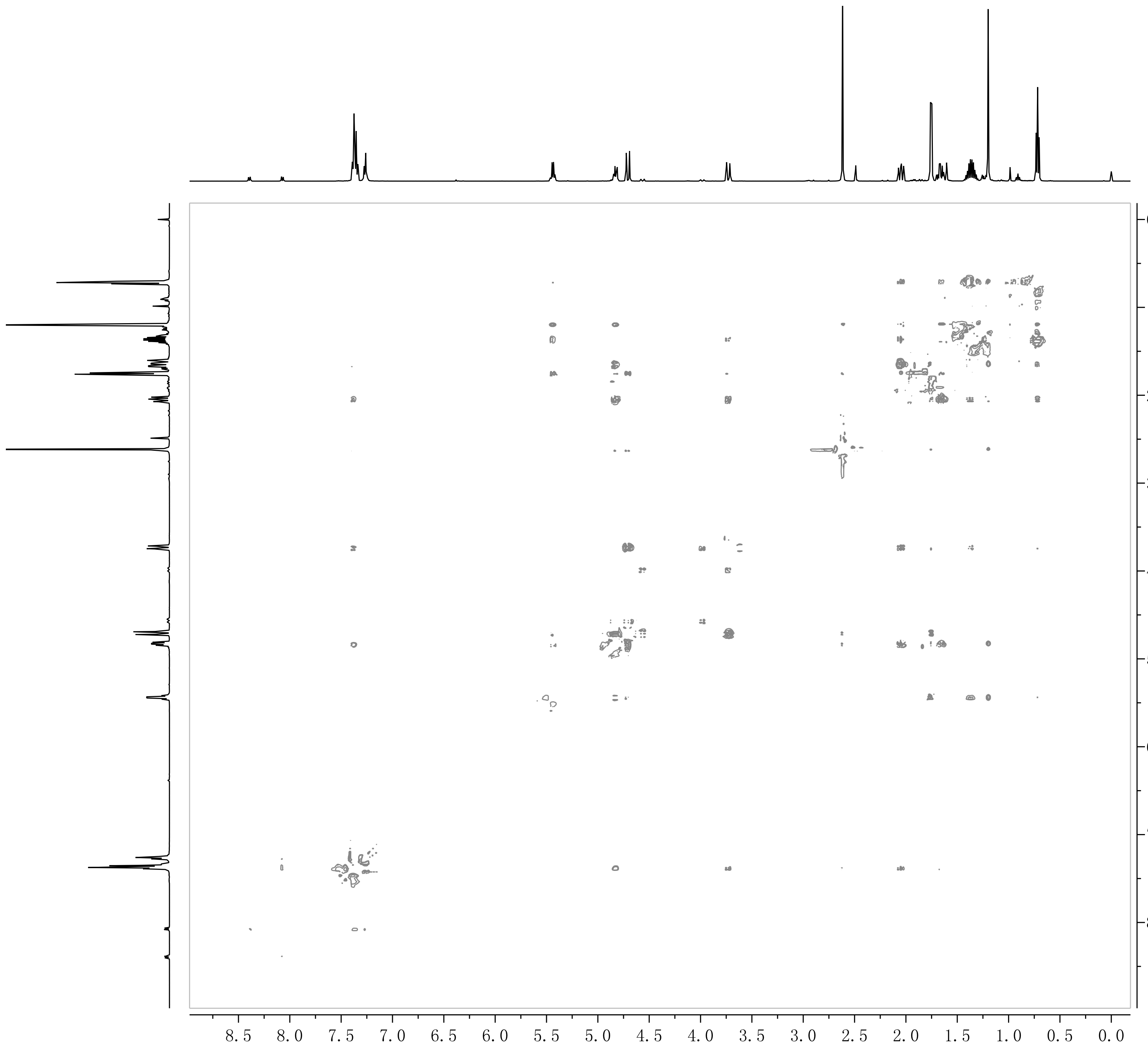

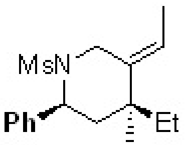

relative configuration

Parameter	Value
标题	xfy-180827-6-s.5.ser
Comment	
Origin	Bruker BioSpin GmbH
Owner	nmr
Site	
Instrument	spect
Author	
Solvent	CDCl3
Temperature	296.2
Pulse Sequence	noesygpphpp
Experiment	NOESY
Number of Scans	4
Receiver Gain	21.9
Relaxation Delay	1.9816
Pulse Width	10.7100
Presaturation Frequency	
Acquisition Time	0.2232
Class	
Spectrometer Frequency	(500.13, 500.13)
Spectral Width	(4587.2, 4587.2)
Lowest Frequency	(-97.9, -97.9)
Nucleus	(1H, 1H)
Acquired Size	(1024, 231)
Spectral Size	(1024, 1024)

Parameter	Value
Title	xfy-180914-2-dMs.1.1.1r
Comment	
Origin	Bruker BioSpin GmbH
Owner	nmr
Site	
Instrument	spect
Solvent	CDCl3
Temperature	296.2
Pulse Sequence	zg30
Experiment	1D
Number of Scans	13
Receiver Gain	87.5
Relaxation Delay	1.0000
Pulse Width	10.7100
Presaturation Frequency	
Acquisition Time	3.2768
Acquisition Date	2019-09-16T21:23:00
Modification Date	2019-09-16T22:39:13
Spectrometer Frequency	500.13
Spectral Width	10000.0
Lowest Frequency	-1924.3
Nucleus	1H
Acquired Size	32768
Spectral Size	65536

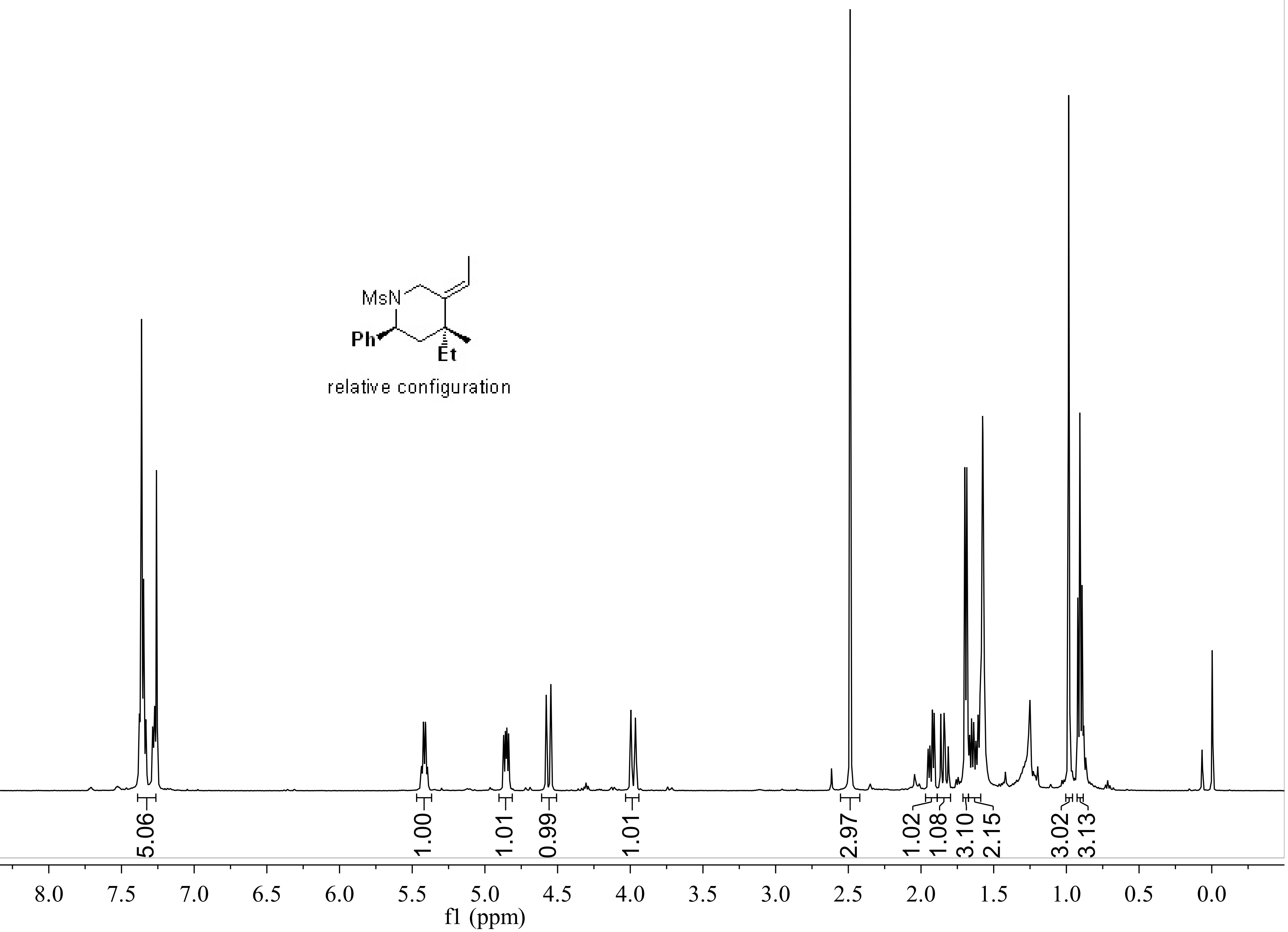

Parameter	Value
Title	xfy-180914-2-dMs.2.1.1r
Comment	
Origin	Bruker BioSpin GmbH
Owner	nmr
Site	
Instrument	spect
Solvent	CDCl3
Temperature	296.1
Pulse Sequence	zgpg30
Experiment	1D
Number of Scans	203
Receiver Gain	193.1
Relaxation Delay	2.0000
Pulse Width	9.6000
Presaturation Frequency	
Acquisition Time	1.1010
Acquisition Date	2019-09-16T21:26:00
Modification Date	2019-09-16T22:39:14
Spectrometer Frequency	125.76
Spectral Width	29761.9
Lowest Frequency	-2290.2
Nucleus	13C
Acquired Size	32768
Spectral Size	32768

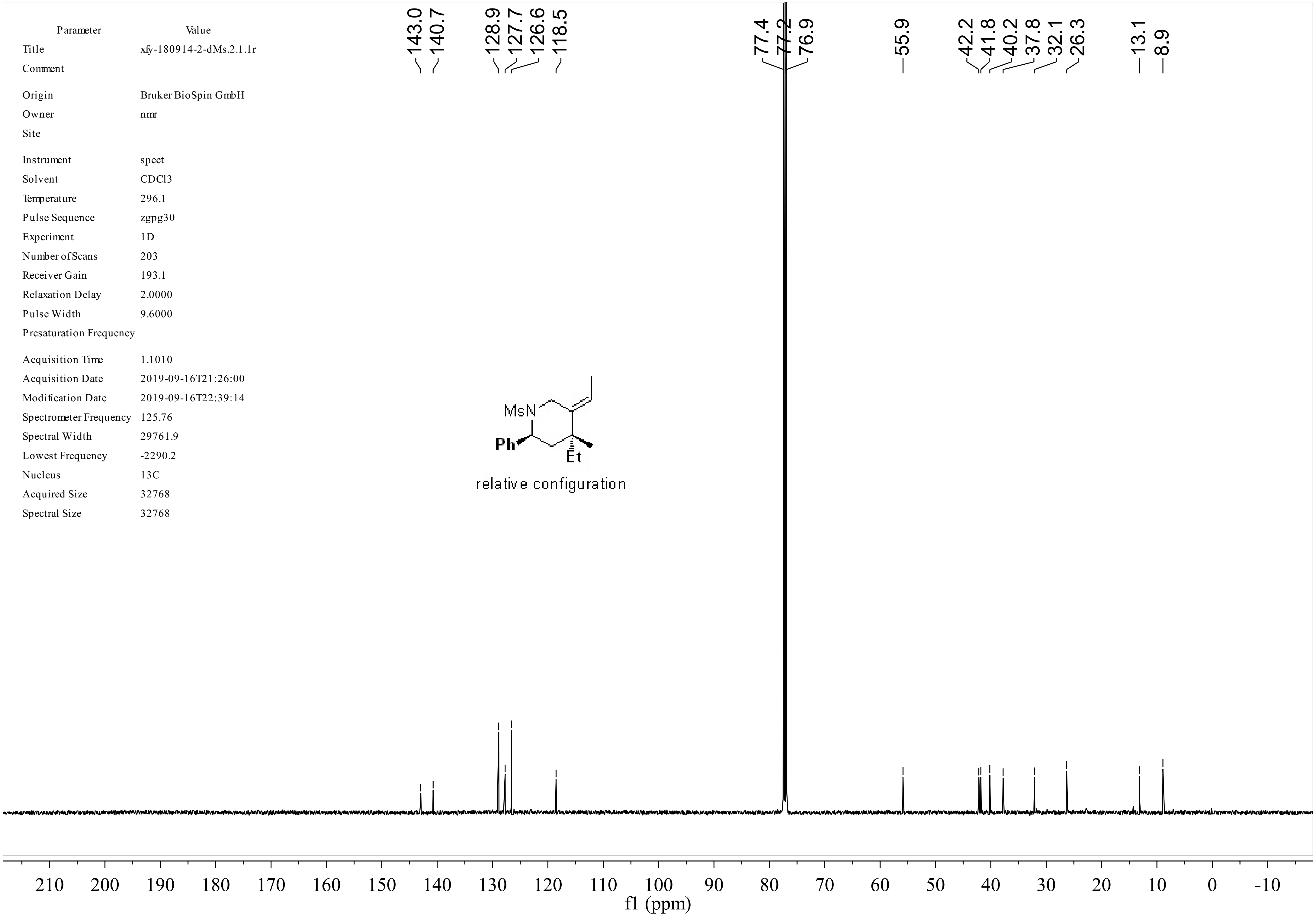

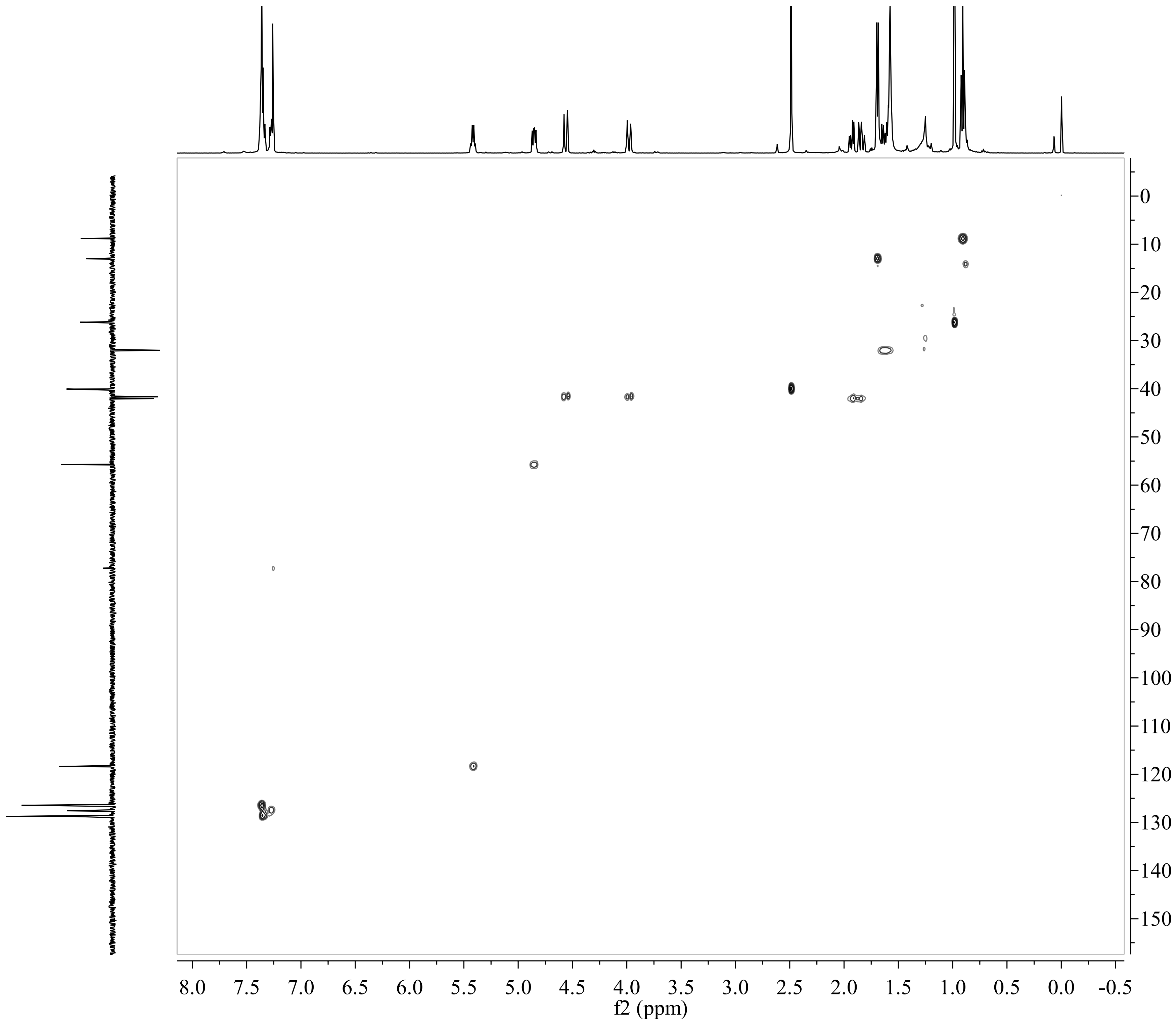

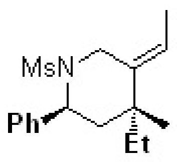

relative configuration

Parameter	Value
Title	xfy-180914-2-dMs.6.ser
Comment	
Origin	Bruker BioSpin GmbH
Owner	nmr
Site	
Instrument	spect
Solvent	CDCl3
Temperature	297.4
Pulse Sequence	hsqcetgp
Experiment	HSQC
Number of Scans	2
Receiver Gain	196.4
Relaxation Delay	1.4521
Pulse Width	8.7300
Presaturation Frequency	
Acquisition Time	0.1464
Acquisition Date	2019-09-17T03:46:30
Modification Date	2019-09-17T09:22:42
Spectrometer Frequency	(400.13, 100.62)
Spectral Width	(3496.5, 16666.7)
Lowest Frequency	(-239.2, -829.1)
Nucleus	(1H, 13C)
Acquired Size	(512, 256)
Spectral Size	(512, 512)

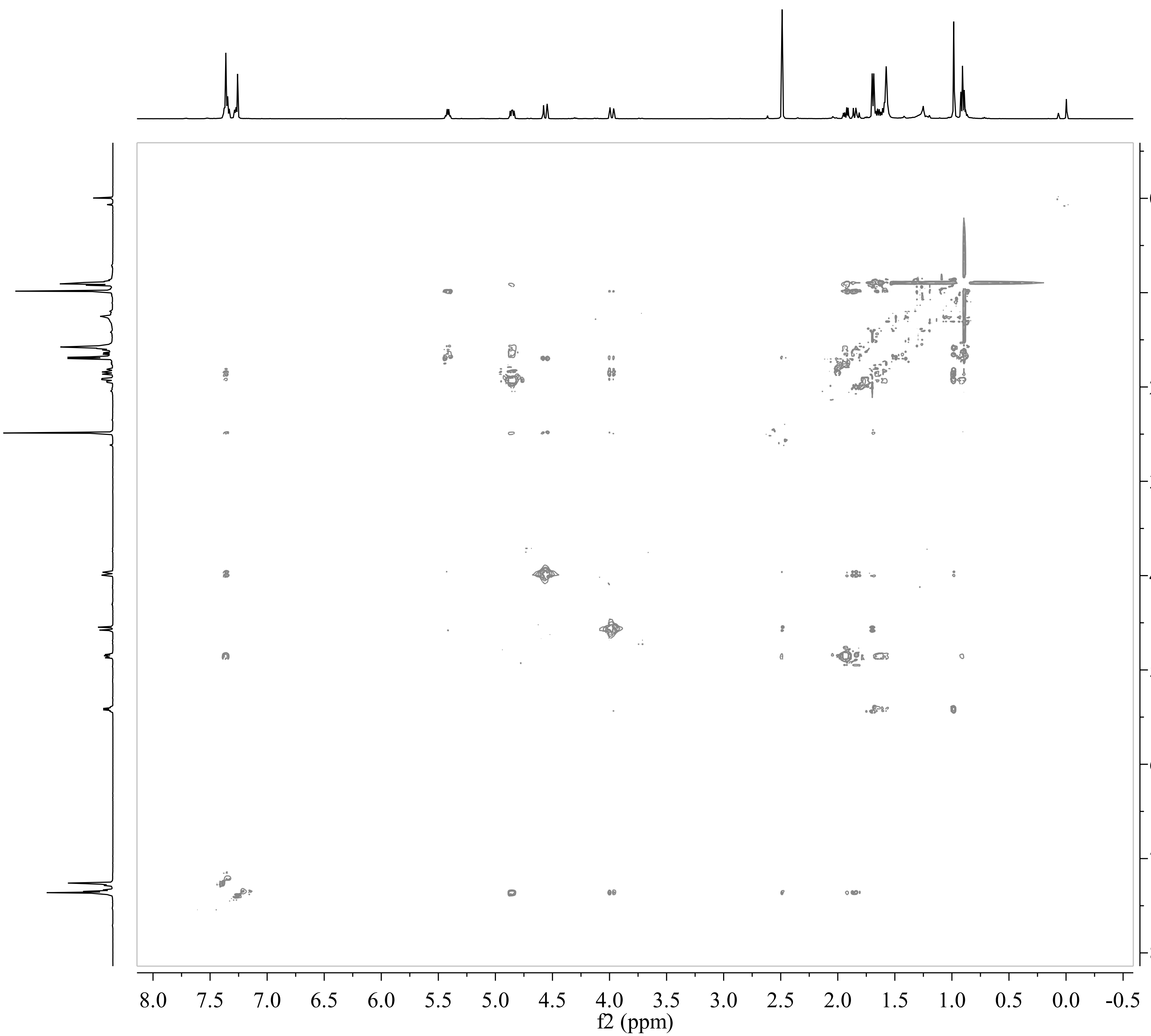

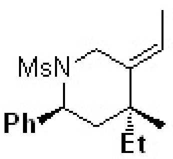

relative configuration

Parameter	Value
Title	xfy-180914-2-dMs.7.ser
Comment	
Origin	Bruker BioSpin GmbH
Owner	nmr
Site	
Instrument	spect
Solvent	CDCl3
Temperature	297.1
Pulse Sequence	noesygpphpp
Experiment	NOESY
Number of Scans	8
Receiver Gain	62.3
Relaxation Delay	1.9631
Pulse Width	8.7300
Presaturation Frequency	
Acquisition Time	0.2929
Acquisition Date	2019-09-17T04:02:41
Modification Date	2019-09-17T09:22:42
Spectrometer Frequency	(400.13, 400.13)
Spectral Width	(3496.5, 3496.5)
Lowest Frequency	(-239.2, -239.2)
Nucleus	(1H, 1H)
Acquired Size	(1024, 256)
Spectral Size	(1024, 1024)

Parameter	Value
Title	xfy-190403-2-s.1.fid
Comment	
Origin	Bruker BioSpin GmbH
Owner	nmr
Site	
Instrument	spect
Solvent	CDCl3
Temperature	297.2
Pulse Sequence	zg30
Experiment	1D
Number of Scans	8
Receiver Gain	111.4
Relaxation Delay	1.0000
Pulse Width	8.7300
Presaturation Frequency	
Acquisition Time	1.9999
Class	
Spectrometer Frequency	400.13
Spectral Width	8012.8
Lowest Frequency	-1535.4
Nucleus	1H
Acquired Size	16025
Spectral Size	65536

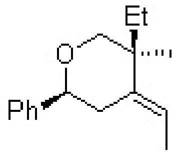

relative configuration

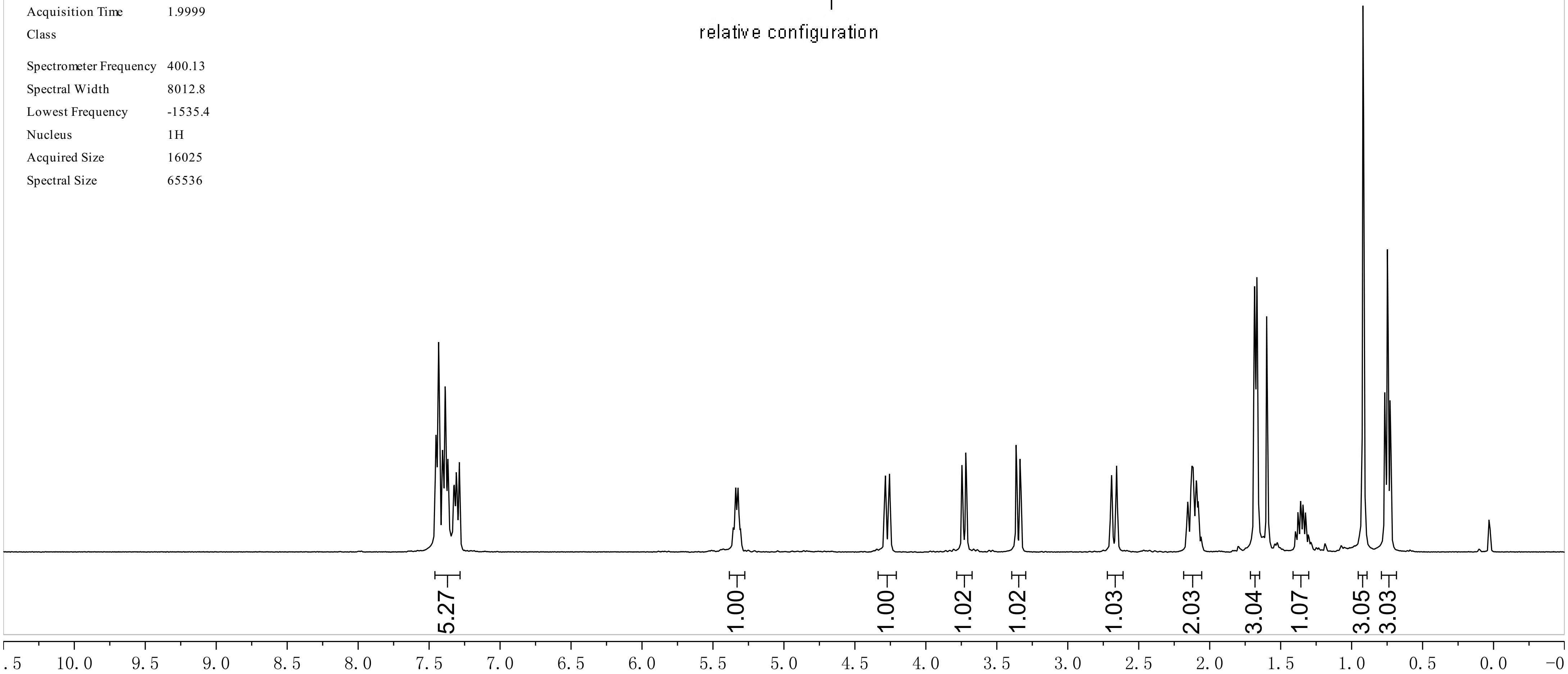

Parameter	Value
Title	xfy-190403-2-s.2.fid
Comment	
Origin	Bruker BioSpin GmbH
Owner	nmr
Site	
Instrument	spect
Solvent	CDCl3
Temperature	297.4
Pulse Sequence	zgpg30
Experiment	1D
Number of Scans	89
Receiver Gain	196.4
Relaxation Delay	2.0000
Pulse Width	10.0000
Presaturation Frequency	
Acquisition Time	1.3631
Class	
Spectrometer Frequency	100.62
Spectral Width	24038.5
Lowest Frequency	-1944.3
Nucleus	13C
Acquired Size	32768
Spectral Size	65536

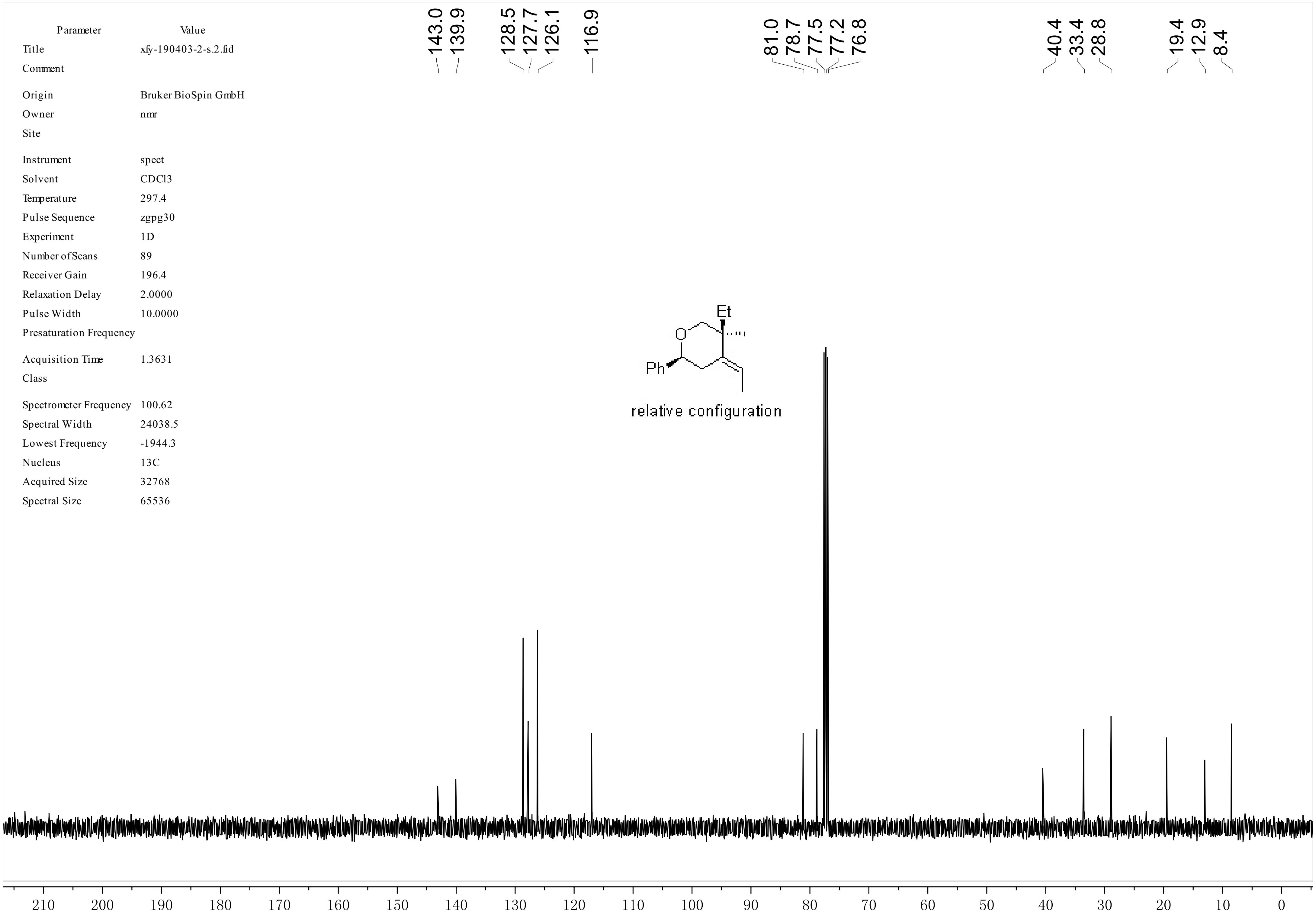

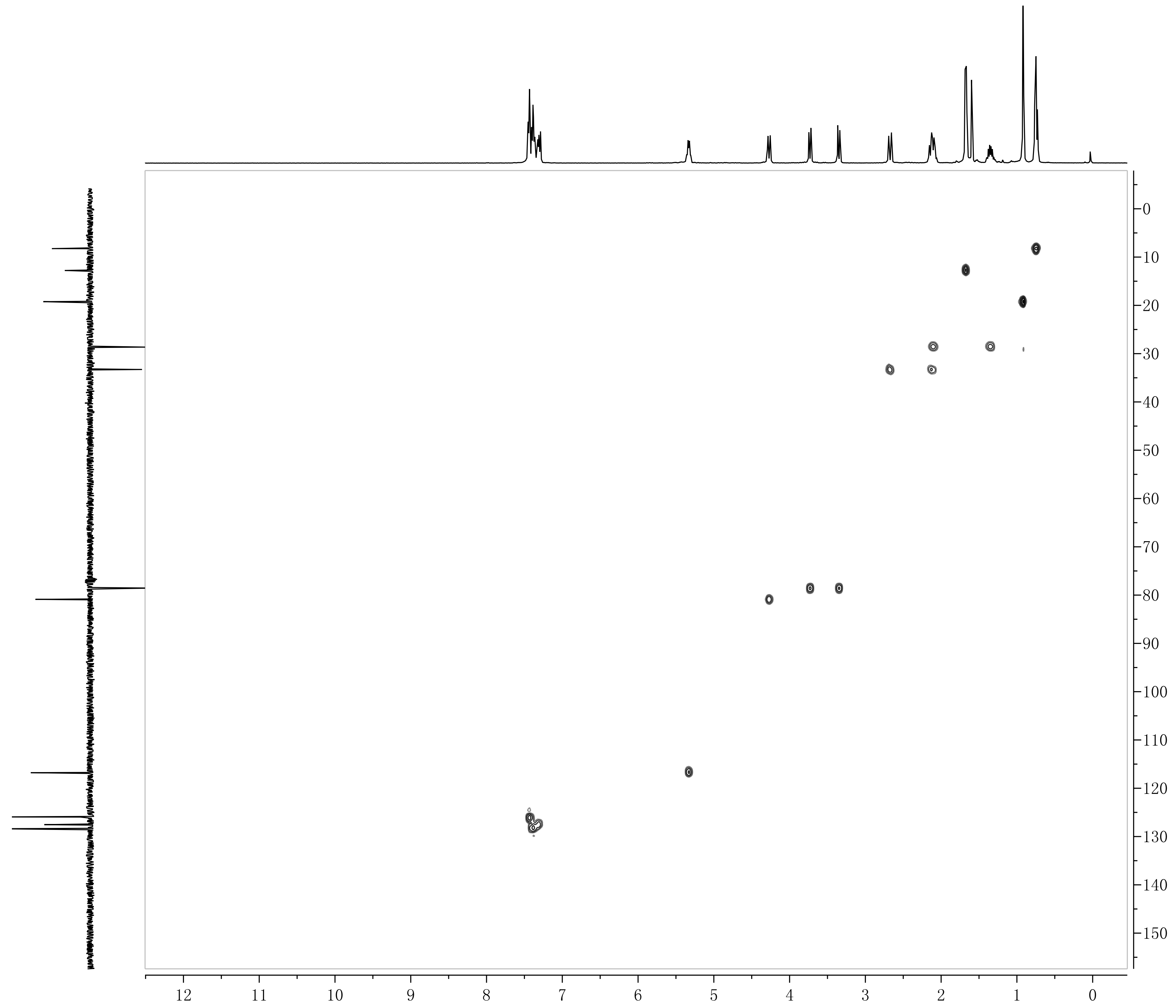

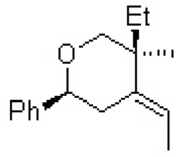

relative configuration

Parameter	Value
Title	xfy-190403-2-s.3.ser
Comment	
Origin	Bruker BioSpin GmbH
Owner	nmr
Site	
Instrument	spect
Solvent	CDCl3
Temperature	297.5
Pulse Sequence	hsqcetgp
Experiment	HSQC
Number of Scans	2
Receiver Gain	196.4
Relaxation Delay	1.5000
Pulse Width	8.7300
Presaturation Frequency	
Acquisition Time	0.0985
Class	
Spectrometer Frequency	(400.13, 100.62)
Spectral Width	(5197.5, 16666.7)
Lowest Frequency	(-193.1, -829.1)
Nucleus	(1H, 13C)
Acquired Size	(512, 256)
Spectral Size	(512, 512)

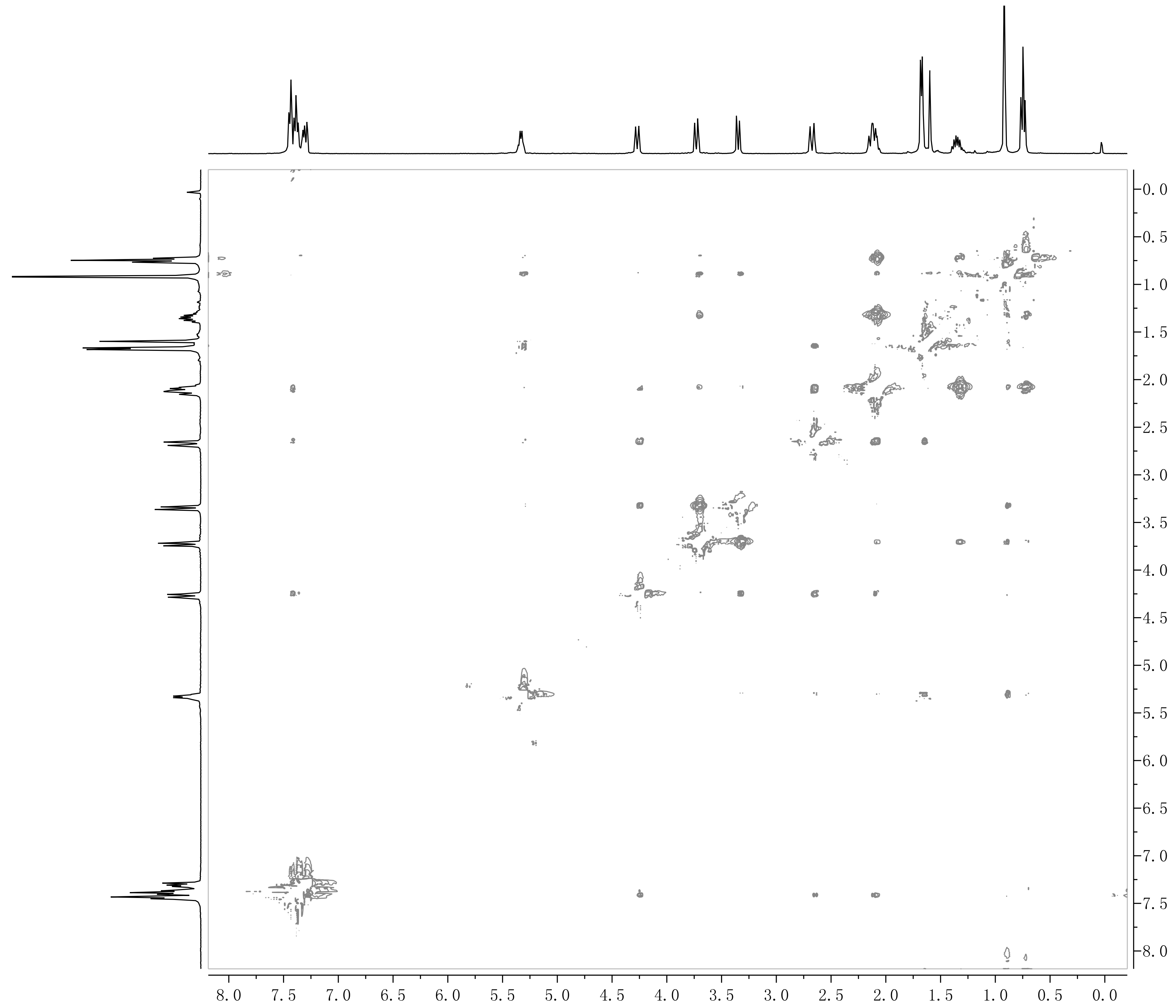

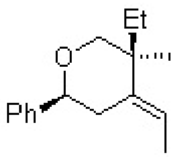

relative configuration

Parameter	Value
Title	xfy-190403-2-s.12.ser
Comment	
Origin	Bruker BioSpin GmbH
Owner	nmr
Site	
Instrument	spect
Solvent	CDCl3
Temperature	296.2
Pulse Sequence	noesygpphpp
Experiment	NOESY
Number of Scans	4
Receiver Gain	48.5
Relaxation Delay	1.9611
Pulse Width	10.7100
Presaturation Frequency	
Acquisition Time	0.2437
Class	
Spectrometer Frequency	(500.13, 500.13)
Spectral Width	(4201.7, 4201.7)
Lowest Frequency	(-106.8, -106.8)
Nucleus	(1H, 1H)
Acquired Size	(1024, 208)
Spectral Size	(1024, 1024)

Parameter	Value
Title	xfy-190909-1-s.11.fid
Comment	
Origin	Bruker BioSpin GmbH
Owner	nmr
Site	
Instrument	spect
Solvent	CDCl3
Temperature	296.1
Pulse Sequence	zg30
Experiment	1D
Number of Scans	11
Receiver Gain	70.5
Relaxation Delay	1.0000
Pulse Width	10.7100
Presaturation Frequency	
Acquisition Time	3.2768
Acquisition Date	2019-09-10T09:31:45
Modification Date	2019-09-10T15:55:27
Spectrometer Frequency	500.13
Spectral Width	10000.0
Lowest Frequency	-1923.7
Nucleus	1H
Acquired Size	32768
Spectral Size	65536

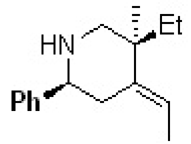

relative configuration

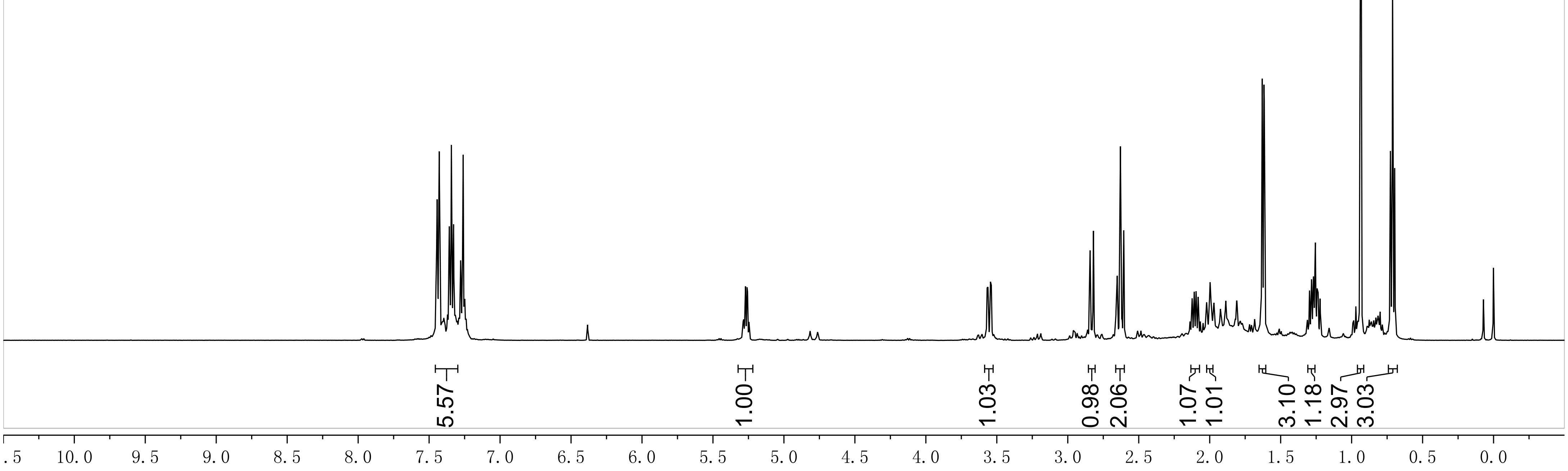

Parameter	Value
Title	xfy-190909-1-s.12.fid
Comment	
Origin	Bruker BioSpin GmbH
Owner	nmr
Site	
Instrument	spect
Solvent	CDCl3
Temperature	296.1
Pulse Sequence	zgpg30
Experiment	1D
Number of Scans	128
Receiver Gain	193.1
Relaxation Delay	2.0000
Pulse Width	9.6000
Presaturation Frequency	
Acquisition Time	1.1010
Acquisition Date	2019-09-10T09:33:27
Modification Date	2019-09-10T15:55:28
Spectrometer Frequency	125.77
Spectral Width	29761.9
Lowest Frequency	-2289.7
Nucleus	13C
Acquired Size	32768
Spectral Size	65536

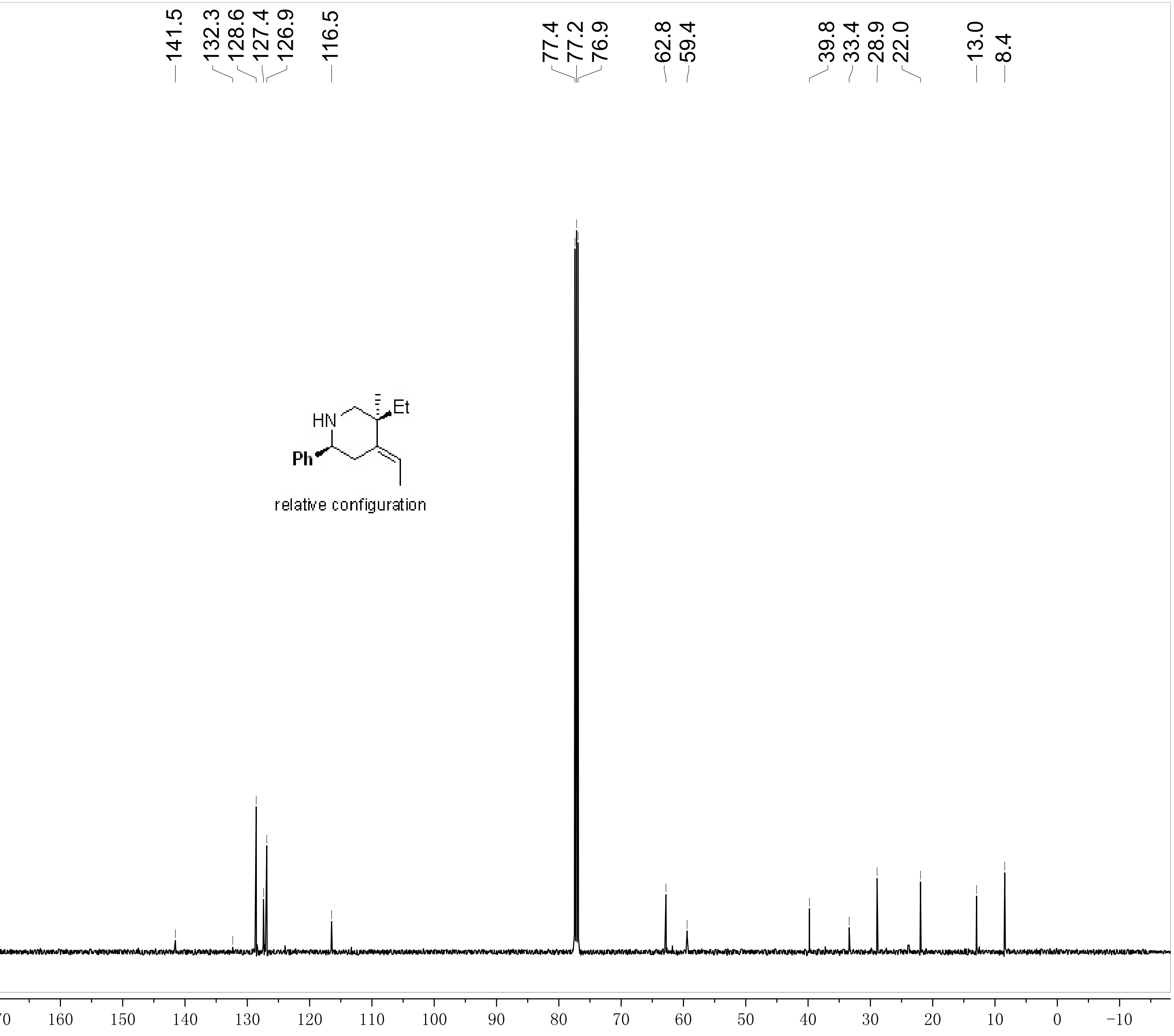

relative configuration

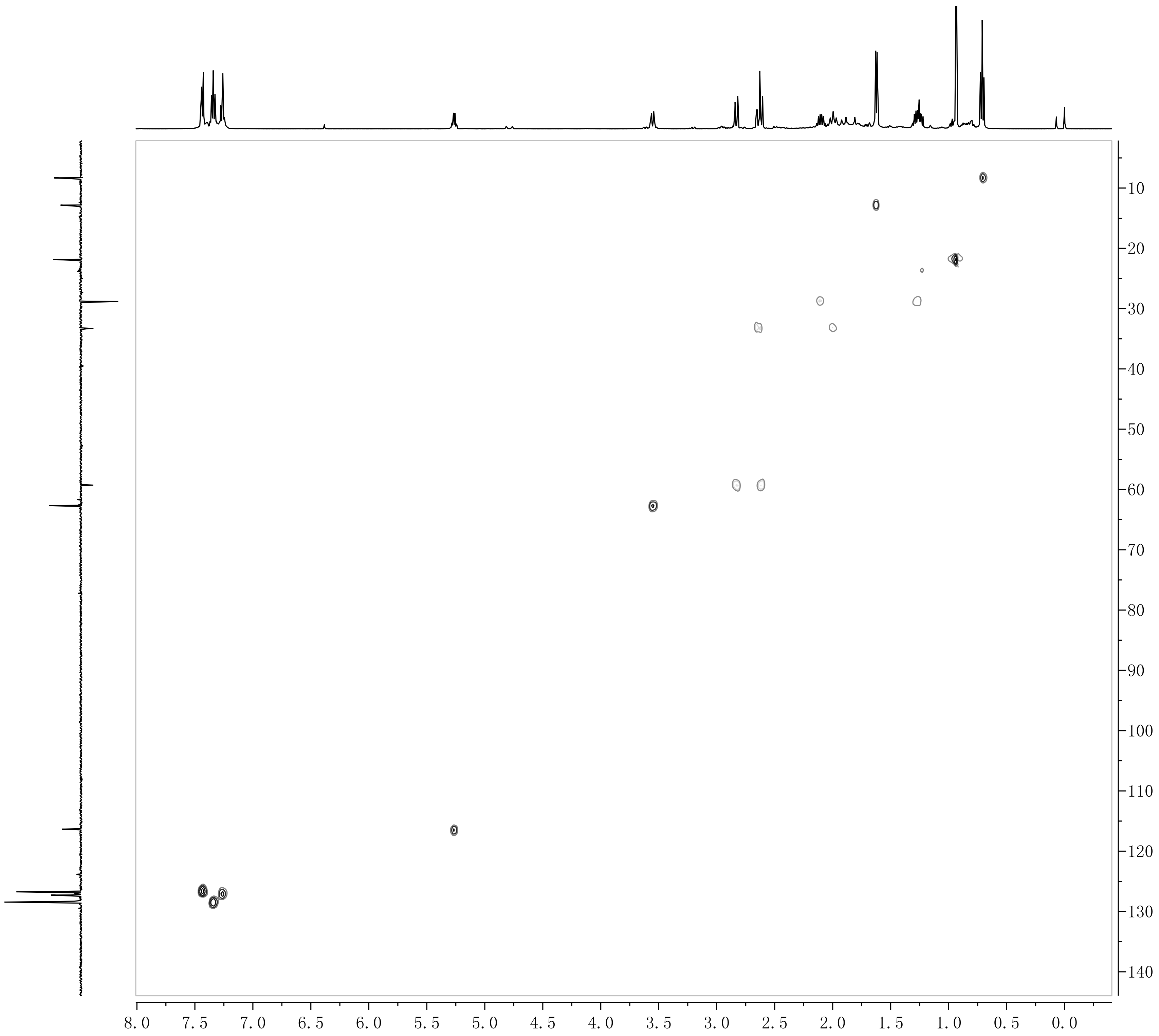

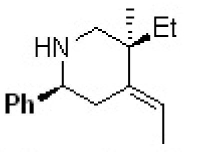

relative configuration

Parameter	Value
Title	xfy-190909-1-s.14.ser
Comment	
Origin	Bruker BioSpin GmbH
Owner	nmr
Site	
Instrument	spect
Solvent	CDCl3
Temperature	296.1
Pulse Sequence	hsqcedetgp
Experiment	HSQC-EDITED
Number of Scans	2
Receiver Gain	193.1
Relaxation Delay	1.4754
Pulse Width	10.7100
Presaturation Frequency	
Acquisition Time	0.1034
Acquisition Date	2019-09-10T09:47:28
Modification Date	2019-09-10T15:55:28
Spectrometer Frequency	(500.13, 125.77)
Spectral Width	(4950.5, 20833.3)
Lowest Frequency	(-797.5, -1037.0)
Nucleus	(1H, 13C)
Acquired Size	(512, 256)
Spectral Size	(512, 512)

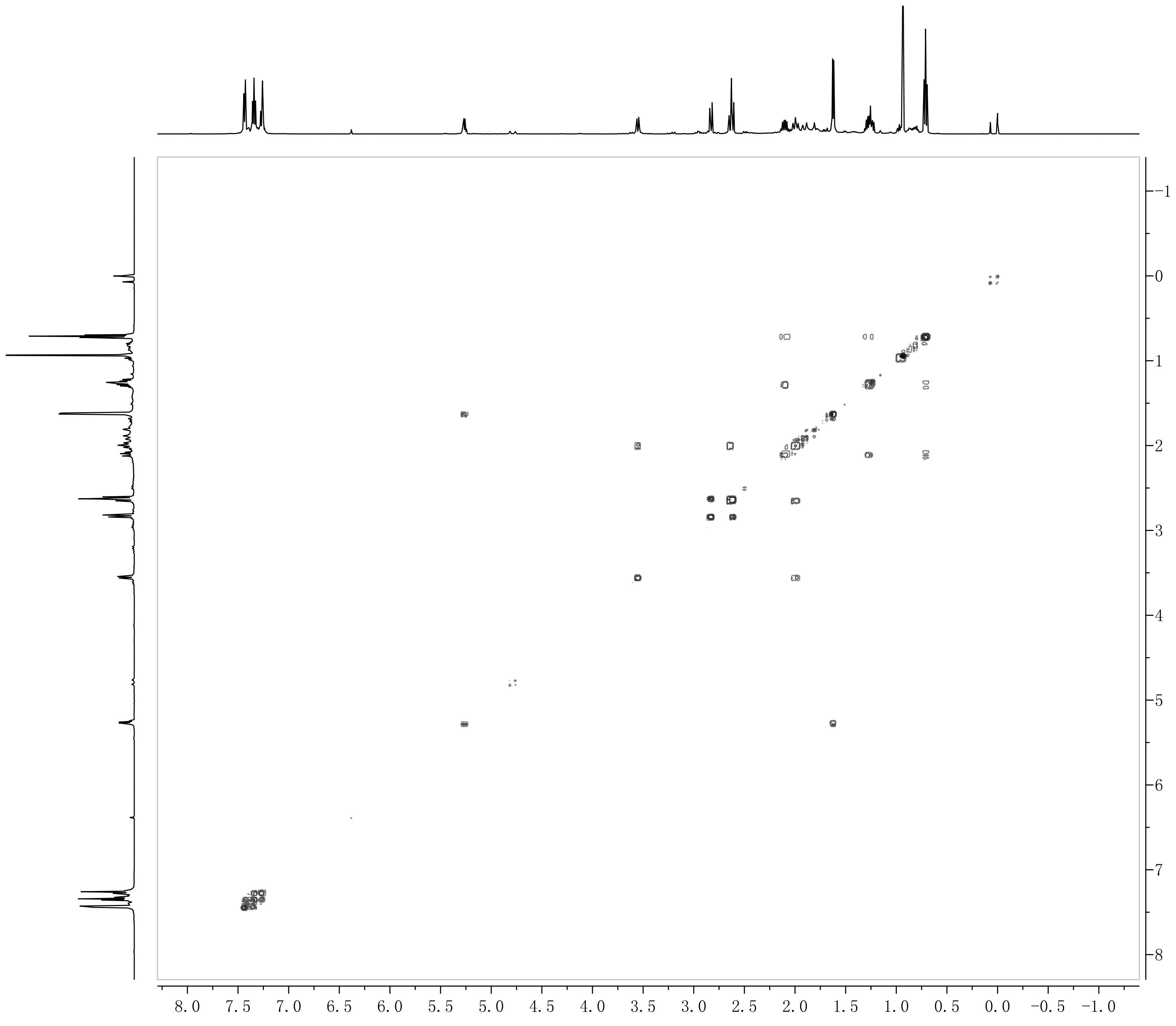

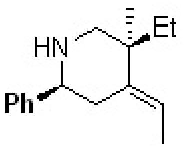

relative configuration

Parameter	Value
Title	xfy-190909-1-s.15.ser
Comment	
Origin	Bruker BioSpin GmbH
Owner	nmr
Site	
Instrument	spect
Solvent	CDCl3
Temperature	296.2
Pulse Sequence	cosygpppqf
Experiment	COSY
Number of Scans	1
Receiver Gain	48.5
Relaxation Delay	1.9422
Pulse Width	10.7100
Presaturation Frequency	
Acquisition Time	0.2109
Acquisition Date	2019-09-10T10:02:20
Modification Date	2019-09-10T15:55:30
Spectrometer Frequency	(500.13, 500.13)
Spectral Width	(4854.4, 4854.4)
Lowest Frequency	(-705.4, -705.4)
Nucleus	(1H, 1H)
Acquired Size	(1024, 128)
Spectral Size	(1024, 1024)

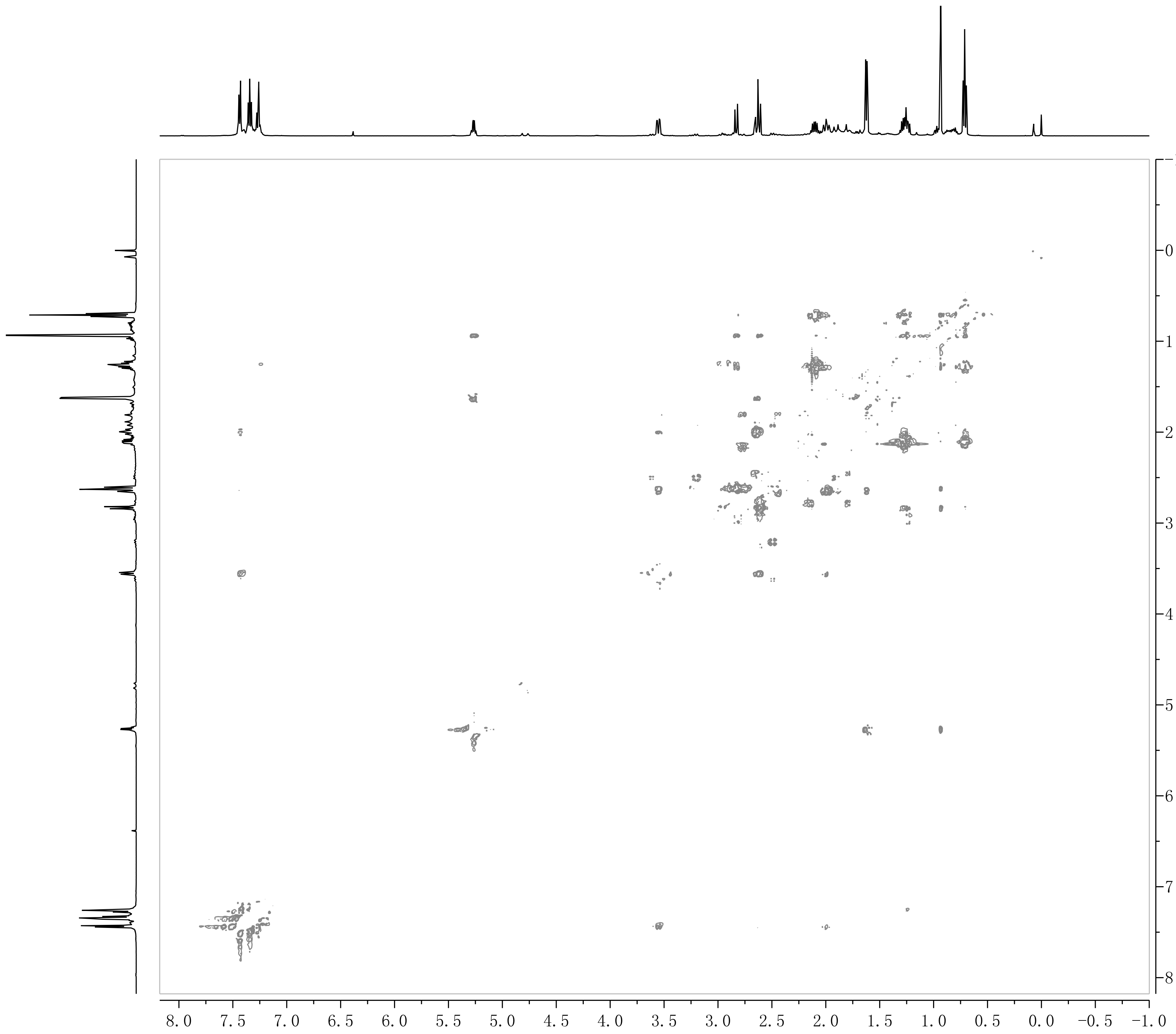

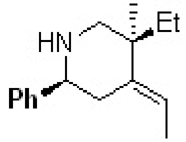

relative configuration

Parameter	Value
Title	xfy-190909-1-s.32.ser
Comment	
Origin	Bruker BioSpin GmbH
Owner	nmr
Site	
Instrument	spect
Solvent	CDCl3
Temperature	296.7
Pulse Sequence	noesygpphpp
Experiment	NOESY
Number of Scans	16
Receiver Gain	31.7
Relaxation Delay	1.9775
Pulse Width	8.7300
Presaturation Frequency	
Acquisition Time	0.2785
Acquisition Date	2019-09-10T23:24:01
Modification Date	2019-09-11T09:25:25
Spectrometer Frequency	(400.13, 400.13)
Spectral Width	(3676.5, 3676.5)
Lowest Frequency	(-404.1, -404.1)
Nucleus	(1H, 1H)
Acquired Size	(1024, 256)
Spectral Size	(1024, 1024)

Parameter	Value
Title	xfy-190909-1-dMs.1.1.1r
Comment	
Origin	Bruker BioSpin GmbH
Owner	nmr
Site	
Instrument	spect
Solvent	CDCl3
Temperature	298.4
Pulse Sequence	zg30
Experiment	1D
Number of Scans	8
Receiver Gain	142.1
Relaxation Delay	1.0000
Pulse Width	8.7300
Presaturation Frequency	
Acquisition Time	1.9999
Acquisition Date	2019-09-10T17:44:54
Modification Date	2019-09-10T19:56:32
Spectrometer Frequency	400.13
Spectral Width	8012.8
Lowest Frequency	-1544.2
Nucleus	1H
Acquired Size	16025
Spectral Size	65536

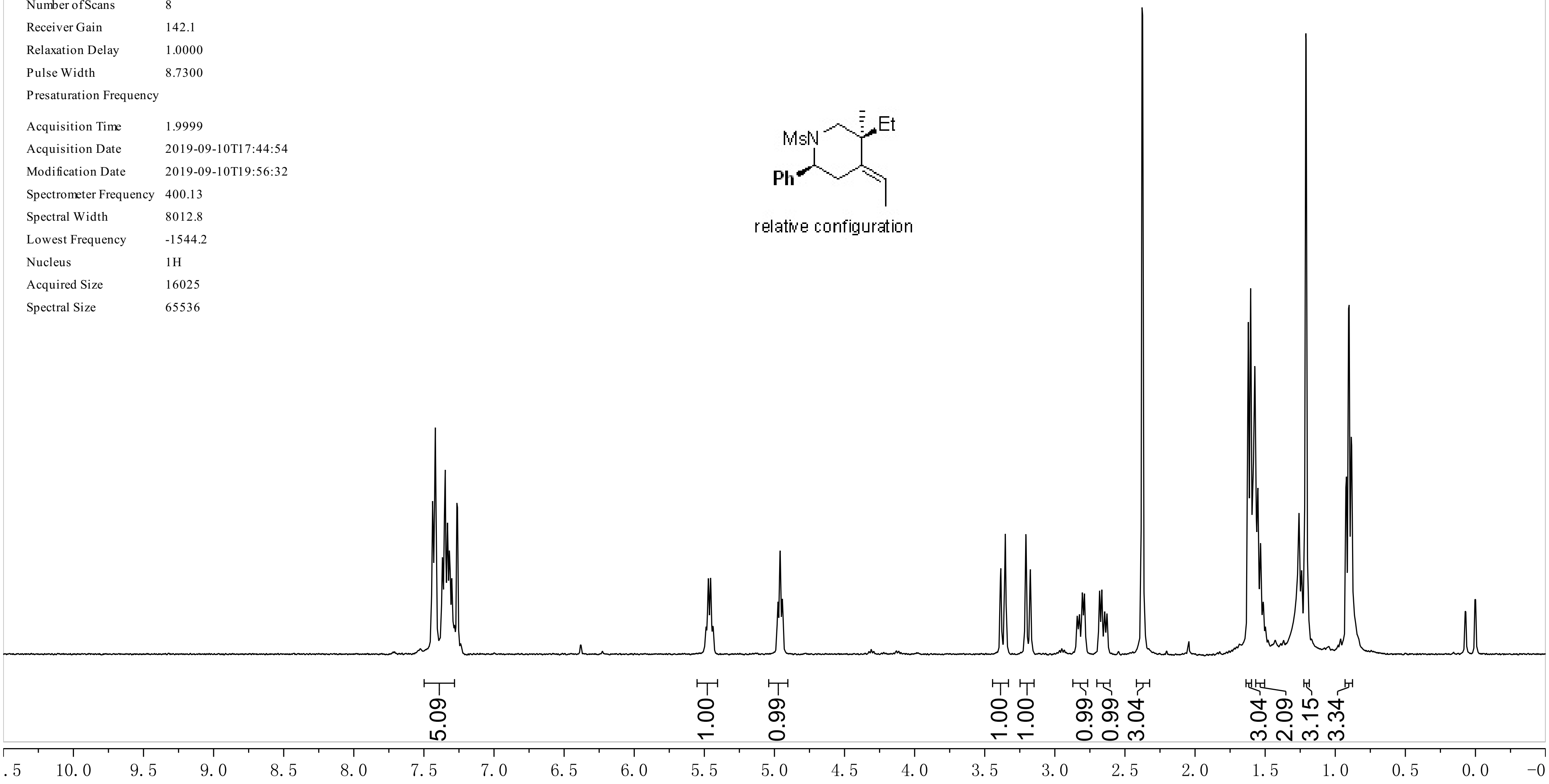

Parameter	Value
Title	xfy-190909-1-dMs.4.fid
Comment	
Origin	Bruker BioSpin GmbH
Owner	nmr
Site	
Instrument	spect
Solvent	CDCl3
Temperature	297.0
Pulse Sequence	zgpg30
Experiment	1D
Number of Scans	700
Receiver Gain	196.4
Relaxation Delay	2.0000
Pulse Width	10.0000
Presaturation Frequency	
Acquisition Time	1.3631
Acquisition Date	2019-09-11T03:25:03
Modification Date	2019-09-11T09:24:34
Spectrometer Frequency	100.62
Spectral Width	24038.5
Lowest Frequency	-1944.2
Nucleus	13C
Acquired Size	32768
Spectral Size	65536

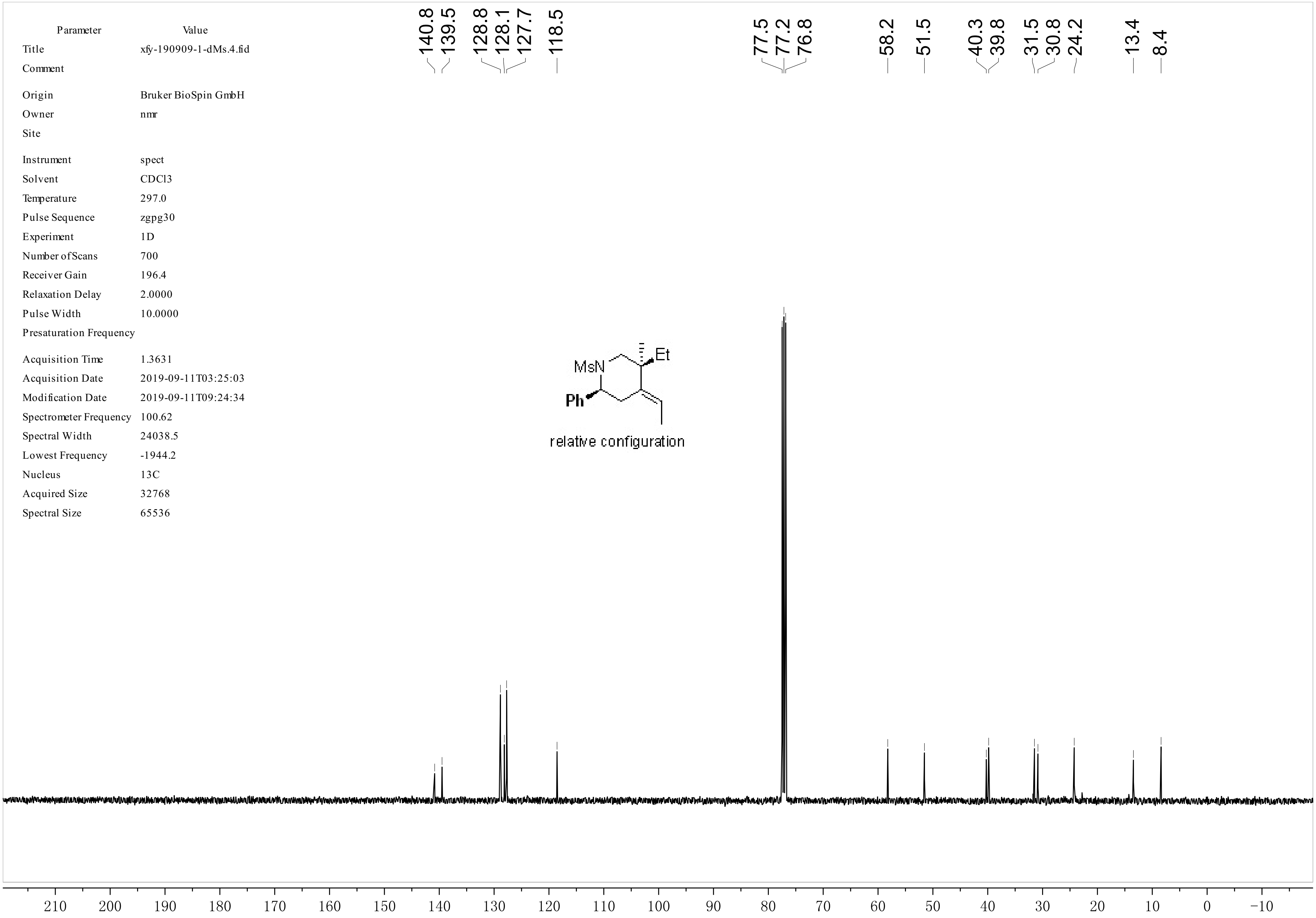

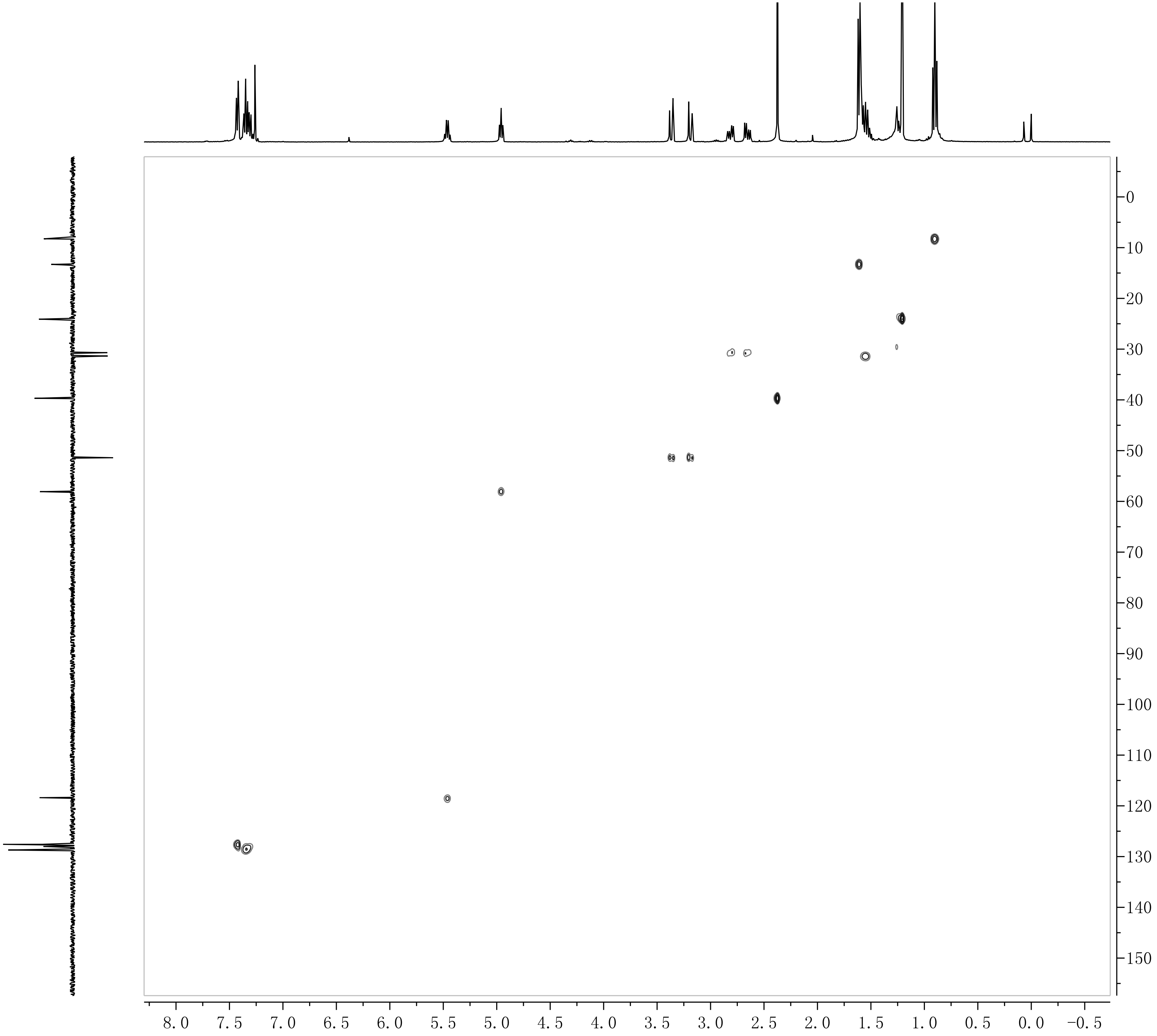

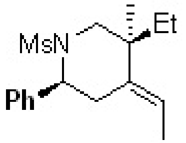

relative configuration

Parameter	Value
Title	xfy-190909-1-dMs.6.ser
Comment	
Origin	Bruker BioSpin GmbH
Owner	nmr
Site	
Instrument	spect
Solvent	CDCl3
Temperature	296.7
Pulse Sequence	hsqcetgp
Experiment	HSQC
Number of Scans	4
Receiver Gain	196.4
Relaxation Delay	1.4572
Pulse Width	8.7300
Presaturation Frequency	
Acquisition Time	0.1413
Acquisition Date	2019-09-11T03:42:17
Modification Date	2019-09-11T09:24:32
Spectrometer Frequency	(400.13, 100.62)
Spectral Width	(3623.2, 16666.7)
Lowest Frequency	(-302.7, -829.1)
Nucleus	(1H, 13C)
Acquired Size	(512, 256)
Spectral Size	(512, 512)

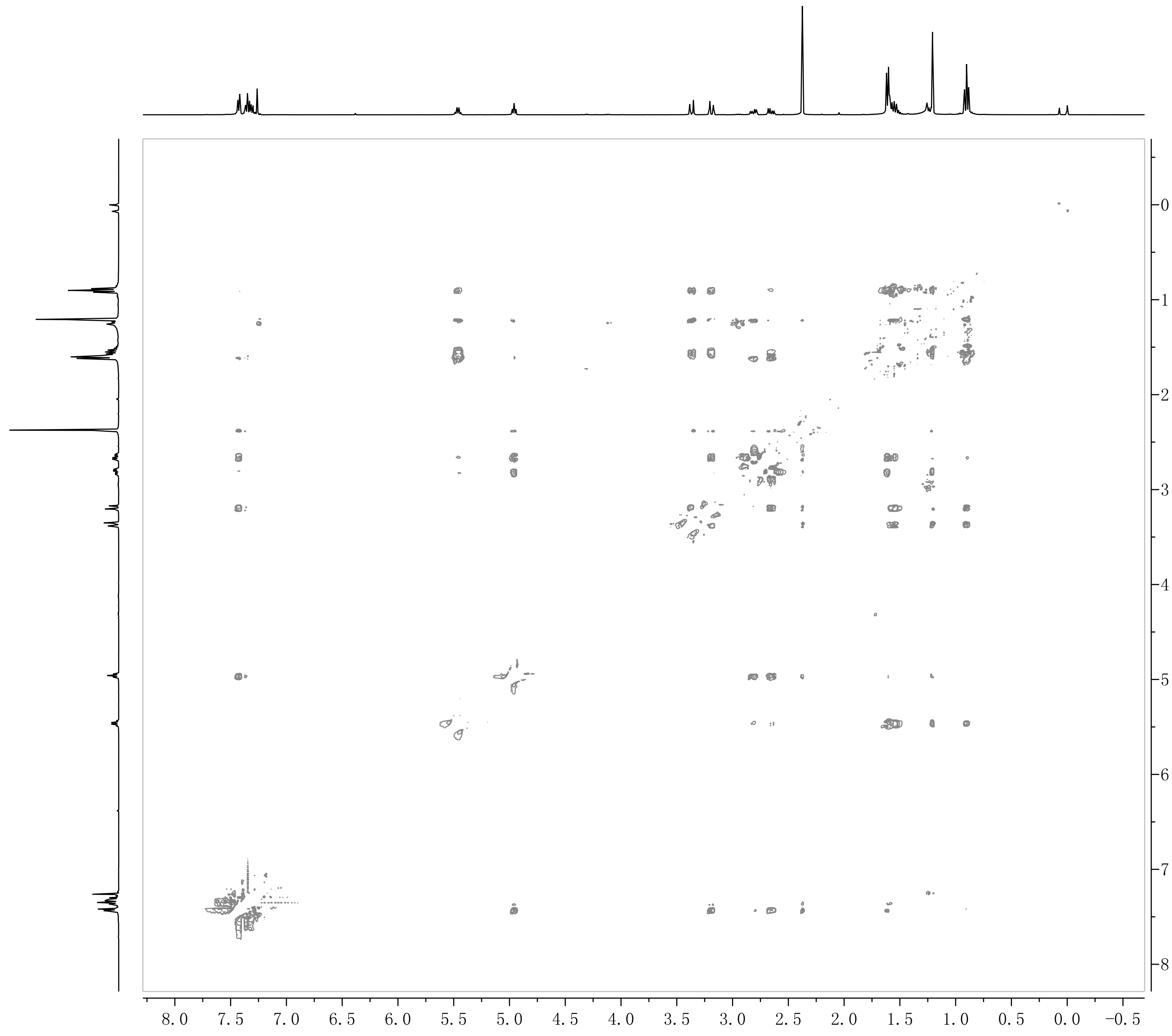

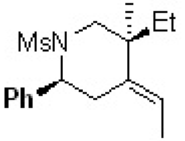

relative configuration

Parameter	Value
Title	xfy-190909-1-dMs.7.ser
Comment	
Origin	Bruker BioSpin GmbH
Owner	nmr
Site	
Instrument	spect
Solvent	CDCl3
Temperature	296.6
Pulse Sequence	noesygpphpp
Experiment	NOESY
Number of Scans	8
Receiver Gain	34.9
Relaxation Delay	1.9713
Pulse Width	8.7300
Presaturation Frequency	
Acquisition Time	0.2847
Acquisition Date	2019-09-11T04:12:23
Modification Date	2019-09-11T09:24:32
Spectrometer Frequency	(400.13, 400.13)
Spectral Width	(3597.1, 3597.1)
Lowest Frequency	(-281.3, -281.3)
Nucleus	(1H, 1H)
Acquired Size	(1024, 256)
Spectral Size	(1024, 1024)

Parameter	Value
Title	xfy-190828-6-s7.1.fid
Comment	
Origin	Bruker BioSpin GmbH
Owner	nmr
Site	
Instrument	spect
Solvent	CDCl3
Temperature	297.5
Pulse Sequence	zg30
Experiment	1D
Number of Scans	8
Receiver Gain	126.1
Relaxation Delay	1.0000
Pulse Width	8.7300
Presaturation Frequency	
Acquisition Time	1.9999
Acquisition Date	2019-08-29T12:01:49
Modification Date	2019-08-29T15:23:40
Spectrometer Frequency	400.13
Spectral Width	8012.8
Lowest Frequency	-1544.7
Nucleus	1H
Acquired Size	16025
Spectral Size	65536

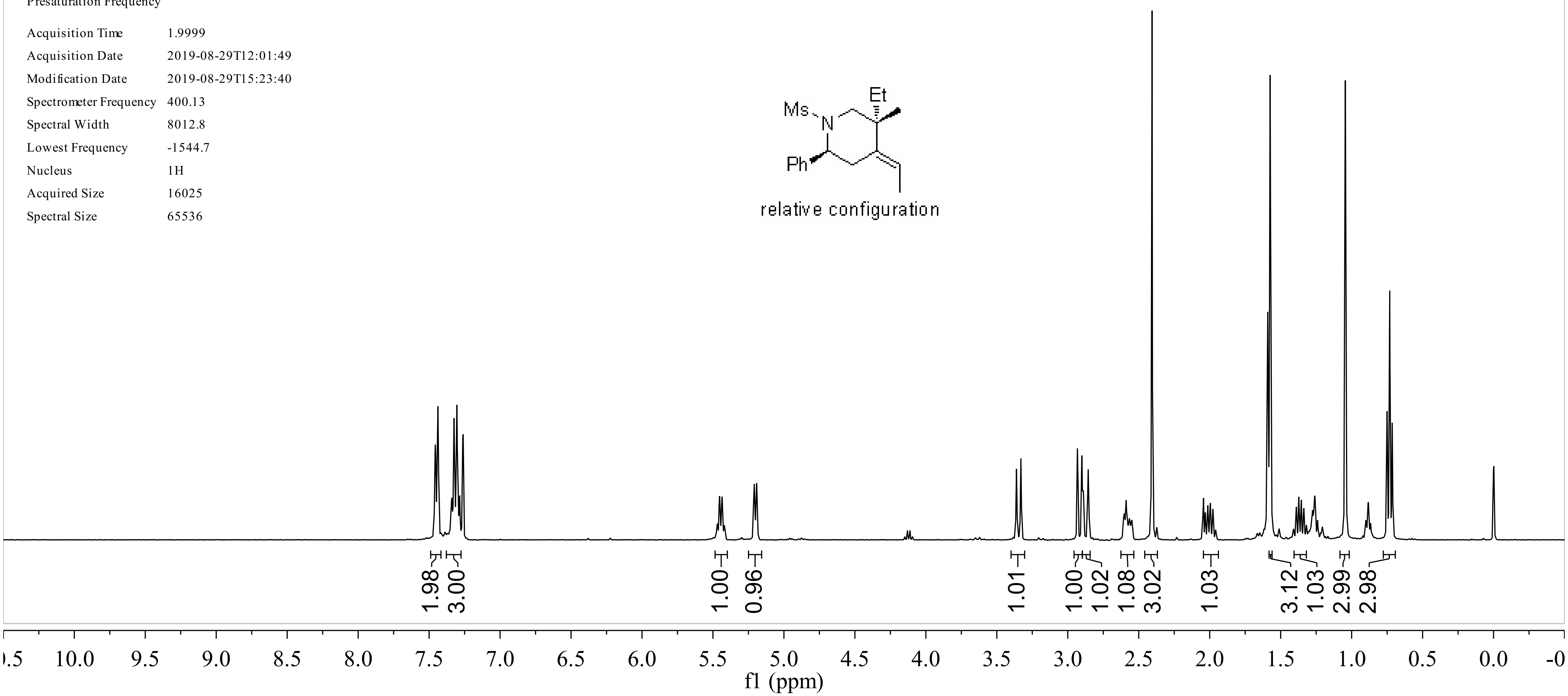

Parameter	Value
Title	xfy-190828-6-s7.5.fid
Comment	
Origin	Bruker BioSpin GmbH
Owner	nmr
Site	
Instrument	spect
Solvent	CDCl3
Temperature	298.4
Pulse Sequence	zgpg30
Experiment	1D
Number of Scans	96
Receiver Gain	196.4
Relaxation Delay	2.0000
Pulse Width	10.0000
Presaturation Frequency	
Acquisition Time	1.3631
Acquisition Date	2019-08-29T12:57:56
Modification Date	2019-08-29T15:23:43
Spectrometer Frequency	100.62
Spectral Width	24038.5
Lowest Frequency	-1943.6
Nucleus	13C
Acquired Size	32768
Spectral Size	65536

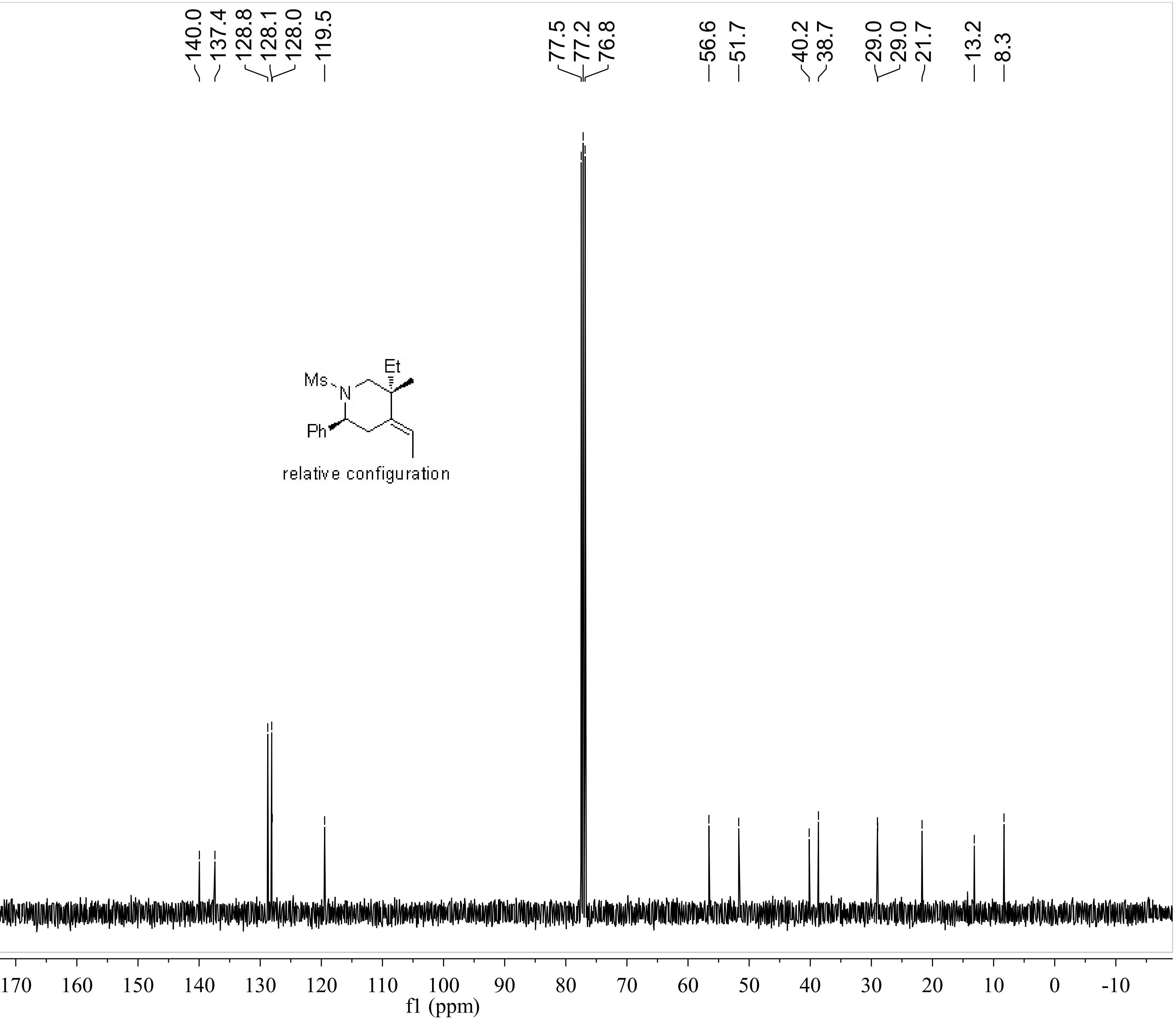

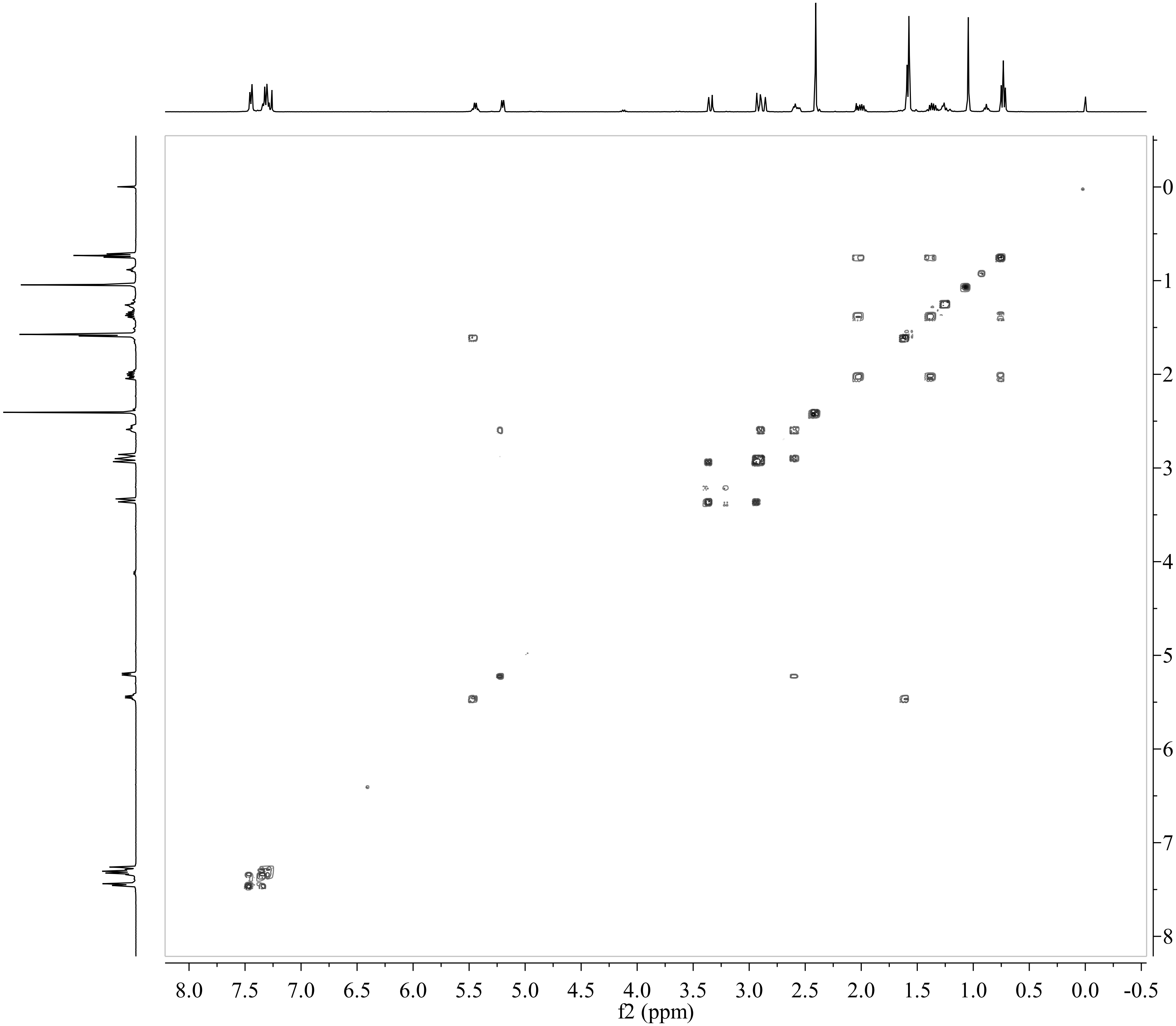

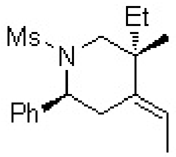

relative configuration

Parameter	Value
Title	xfy-190828-6-s2.5.ser
Comment	
Origin	Bruker BioSpin GmbH
Owner	nmr
Site	
Instrument	spect
Solvent	CDCl3
Temperature	296.1
Pulse Sequence	cosygpppqf
Experiment	COSY
Number of Scans	1
Receiver Gain	27.5
Relaxation Delay	1.9197
Pulse Width	10.7100
Presaturation Frequency	
Acquisition Time	0.2335
Acquisition Date	2019-08-29T04:35:01
Modification Date	2019-08-29T09:37:39
Spectrometer Frequency	(500.13, 500.13)
Spectral Width	(4386.0, 4386.0)
Lowest Frequency	(-278.0, -278.0)
Nucleus	(1H, 1H)
Acquired Size	(1024, 128)
Spectral Size	(1024, 1024)

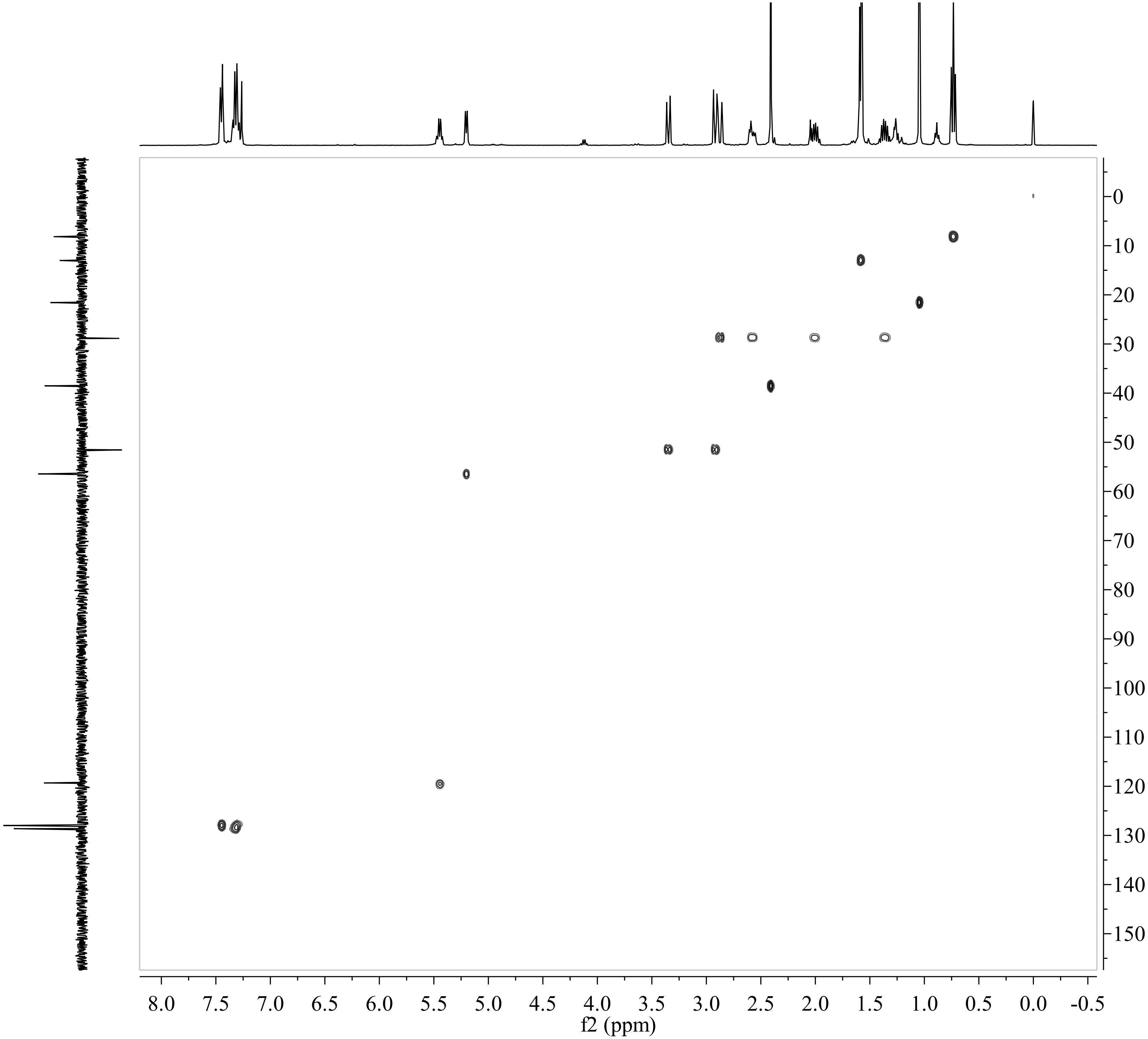

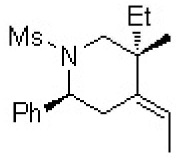

relative configuration

Parameter	Value
Title	xfy-190828-6-s7.3.ser
Comment	
Origin	Bruker BioSpin GmbH
Owner	nmr
Site	
Instrument	spect
Solvent	CDCl3
Temperature	297.9
Pulse Sequence	hsqcetgp
Experiment	HSQC
Number of Scans	2
Receiver Gain	196.4
Relaxation Delay	1.4531
Pulse Width	8.7300
Presaturation Frequency	
Acquisition Time	0.1454
Acquisition Date	2019-08-29T12:37:47
Modification Date	2019-08-29T15:23:41
Spectrometer Frequency	(400.13, 100.62)
Spectral Width	(3521.1, 16666.7)
Lowest Frequency	(-240.3, -829.1)
Nucleus	(1H, 13C)
Acquired Size	(512, 256)
Spectral Size	(512, 512)

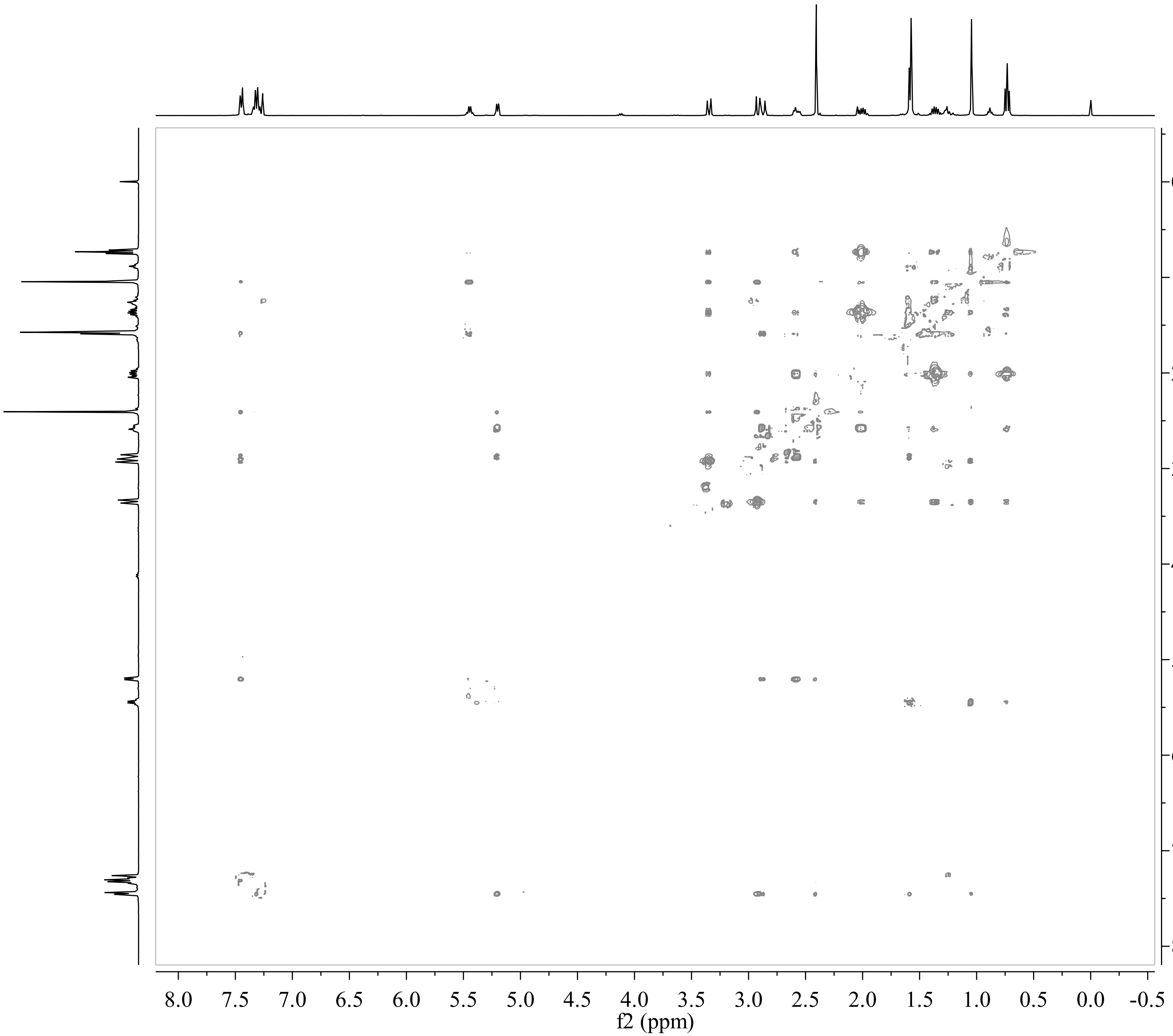

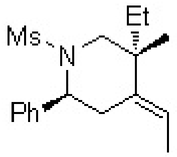

relative configuration

Parameter	Value
Title	xfy-190828-6-s2.6.ser
Comment	
Origin	Bruker BioSpin GmbH
Owner	nmr
Site	
Instrument	spect
Solvent	CDCl3
Temperature	296.1
Pulse Sequence	noesygpphpp
Experiment	NOESY
Number of Scans	8
Receiver Gain	27.5
Relaxation Delay	1.9713
Pulse Width	10.7100
Presaturation Frequency	
Acquisition Time	0.2335
Acquisition Date	2019-08-29T04:42:15
Modification Date	2019-08-29T09:37:40
Spectrometer Frequency	(500.13, 500.13)
Spectral Width	(4386.0, 4386.0)
Lowest Frequency	(-278.0, -278.0)
Nucleus	(1H, 1H)
Acquired Size	(1024, 256)
Spectral Size	(1024, 1024)

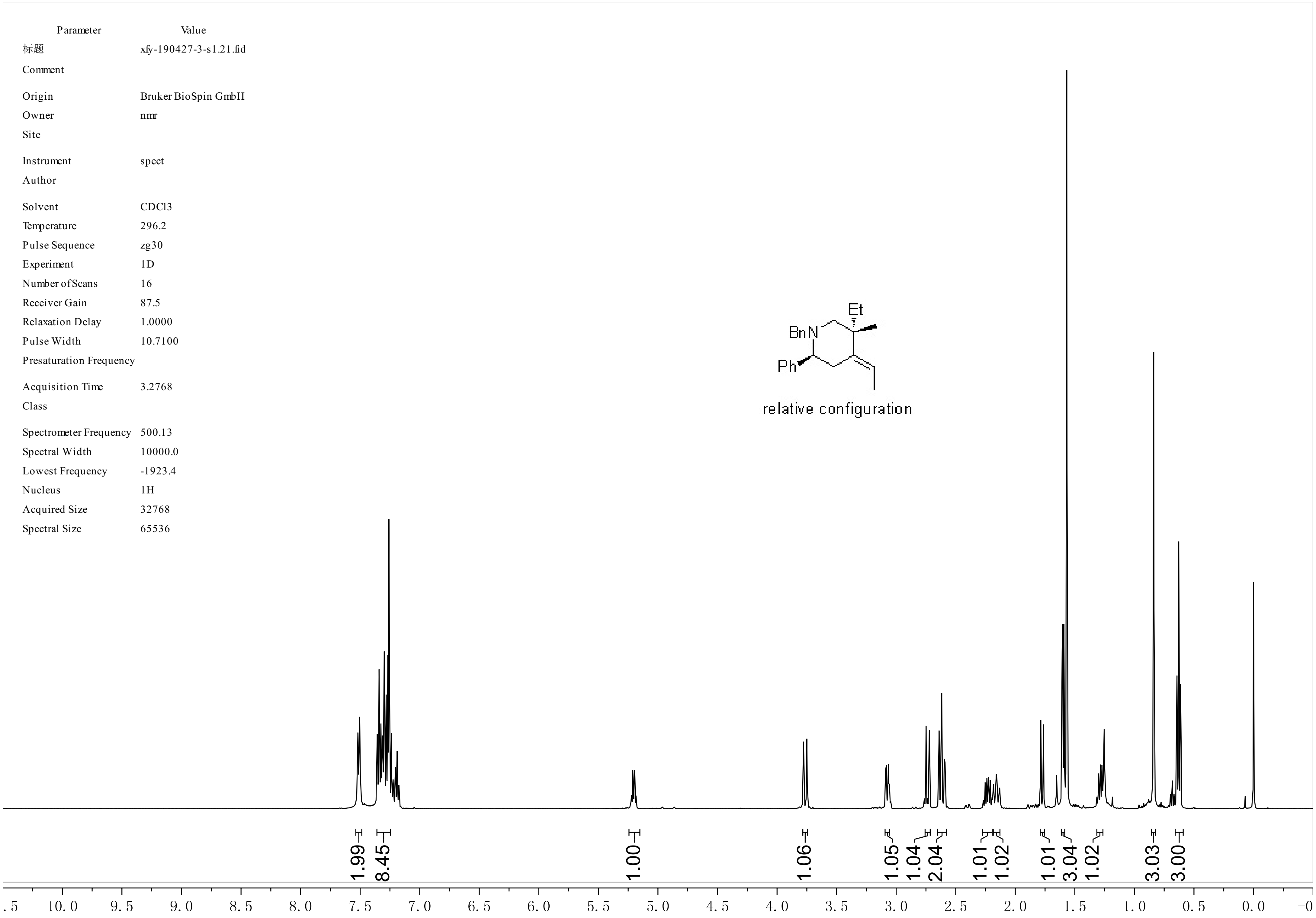

Parameter Value
标题 xfy-190427-3-s1.21.fid
Comment
Origin Bruker BioSpin GmbH
Owner nmr
Site
Instrument spect
Author
Solvent CDCl3
Temperature 296.2
Pulse Sequence zg30
Experiment 1D
Number of Scans 16
Receiver Gain 87.5
Relaxation Delay 1.0000
Pulse Width 10.7100
Presaturation Frequency
Acquisition Time 3.2768
Class
Spectrometer Frequency 500.13
Spectral Width 10000.0
Lowest Frequency -1923.4
Nucleus 1H
Acquired Size 32768
Spectral Size 65536
Et
BnN
Ph
relative configuration
1.99
8.45
1.00
1.06
1.05
1.04
2.04
1.01
1.02
1.01
3.04
1.02
3.03
3.00
.5
10.0
9.5
9.0
8.5
8.0
7.5
7.0
6.5
6.0
5.5
5.0
4.5
4.0
3.5
3.0
2.5
2.0
1.5
1.0
0.5
0.0
−0

Parameter	Value
标题	xfy-190427-3-s1.22.fid
Comment	
Origin	Bruker BioSpin GmbH
Owner	nmr
Site	
Instrument	spect
Author	
Solvent	CDCl3
Temperature	296.2
Pulse Sequence	zgpg30
Experiment	1D
Number of Scans	400
Receiver Gain	193.1
Relaxation Delay	2.0000
Pulse Width	9.6000
Presaturation Frequency	
Acquisition Time	1.1010
Class	
Spectrometer Frequency	125.77
Spectral Width	29761.9
Lowest Frequency	-2288.9
Nucleus	13C
Acquired Size	32768
Spectral Size	65536

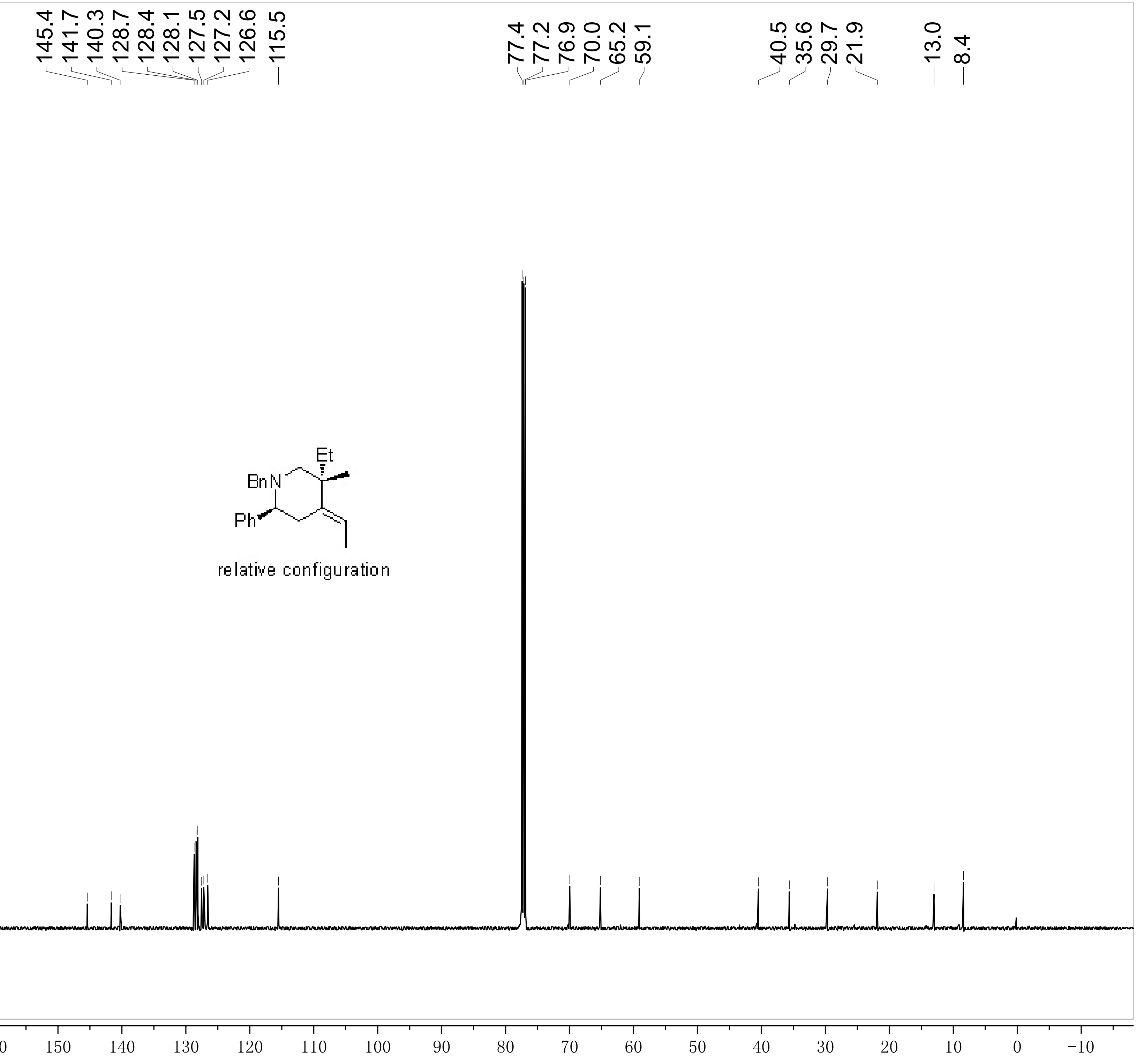

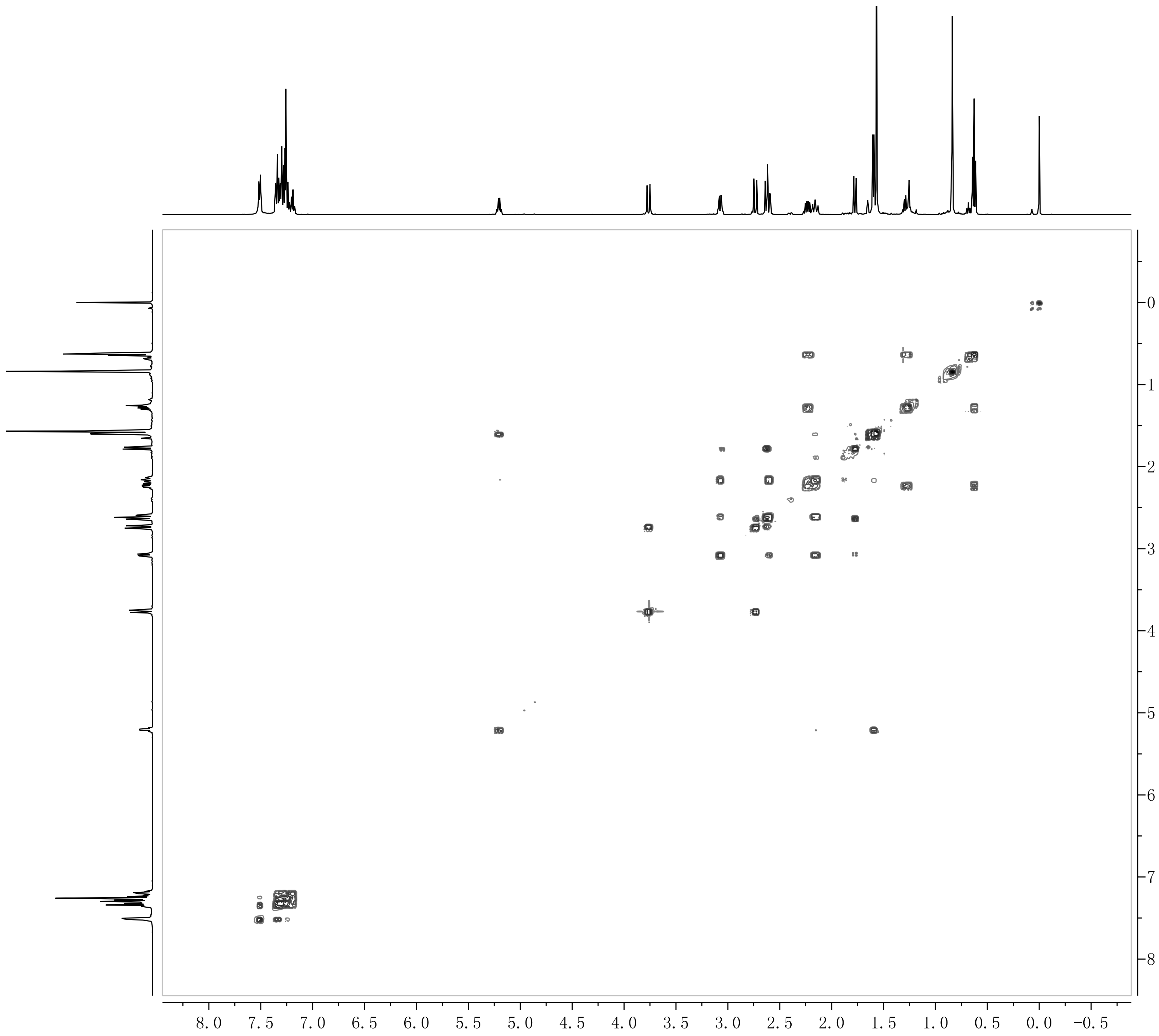

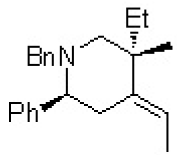

relative configuration

Parameter	Value
Title	xfy-190427-3-s1.24.ser
Comment	
Origin	Bruker BioSpin GmbH
Owner	nmr
Site	
Instrument	spect
Solvent	CDCl3
Temperature	296.1
Pulse Sequence	cosygpppqf
Experiment	COSY
Number of Scans	2
Receiver Gain	31.1
Relaxation Delay	1.9341
Pulse Width	10.7100
Presaturation Frequency	
Acquisition Time	0.2191
Acquisition Date	2019-04-30T01:09:11
Modification Date	2019-04-30T10:09:40
Spectrometer Frequency	(500.13, 500.13)
Spectral Width	(4672.9, 4672.9)
Lowest Frequency	(-448.1, -448.1)
Nucleus	(1H, 1H)
Acquired Size	(1024, 128)
Spectral Size	(1024, 1024)

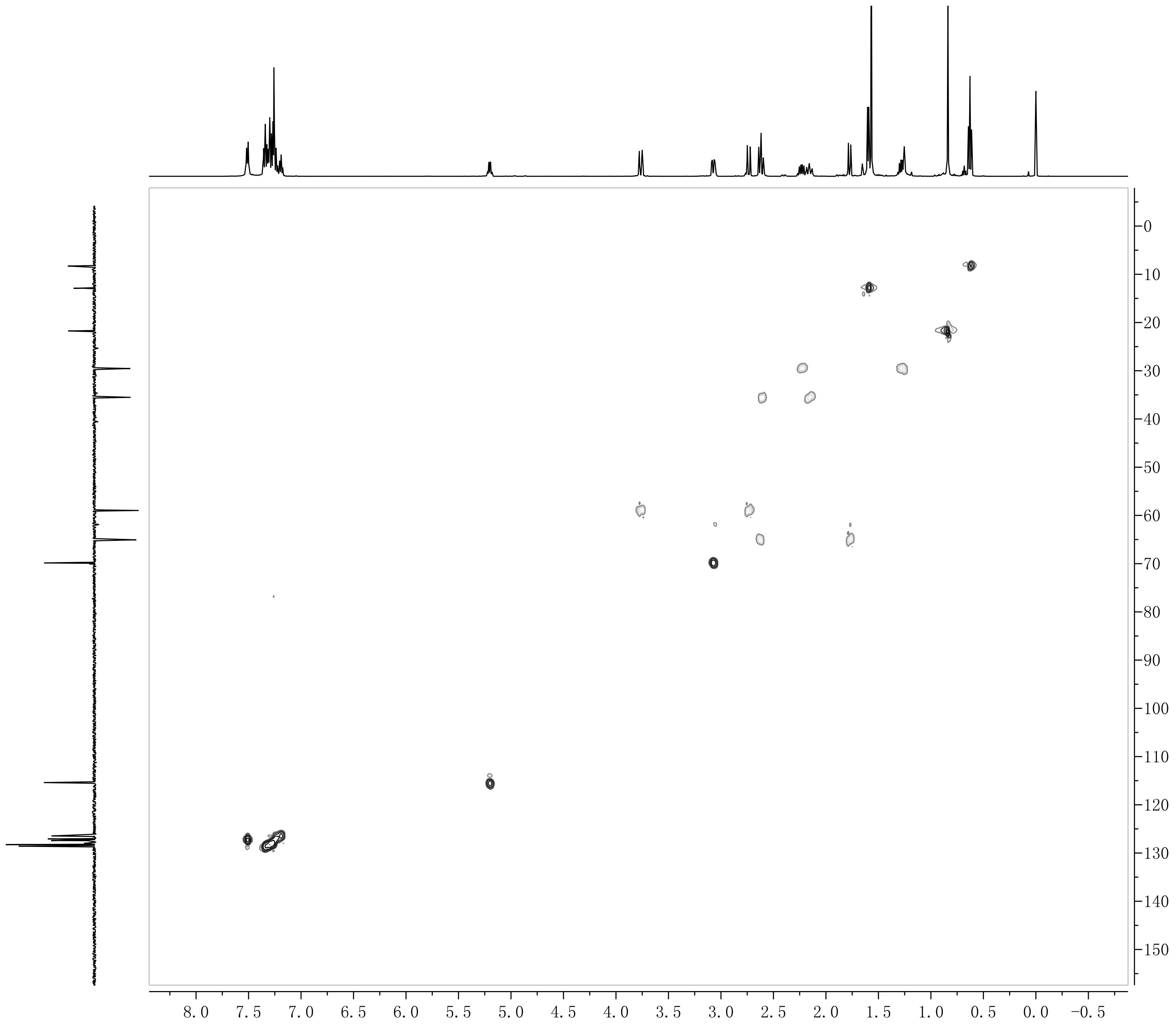

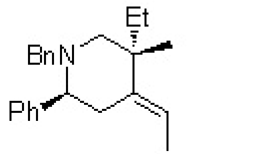

relative configuration

Parameter	Value
Title	xfy-190427-3-s1.25.ser
Comment	
Origin	Bruker BioSpin GmbH
Owner	nmr
Site	
Instrument	spect
Solvent	CDCl3
Temperature	296.2
Pulse Sequence	hsqcedetgp
Experiment	HSQC-EDITED
Number of Scans	4
Receiver Gain	193.1
Relaxation Delay	1.4693
Pulse Width	10.7100
Presaturation Frequency	
Acquisition Time	0.1096
Acquisition Date	2019-04-30T01:20:13
Modification Date	2019-04-30T10:09:40
Spectrometer Frequency	(500.13, 125.77)
Spectral Width	(4672.9, 20833.3)
Lowest Frequency	(-448.1, -1037.0)
Nucleus	(1H, 13C)
Acquired Size	(512, 256)
Spectral Size	(512, 512)

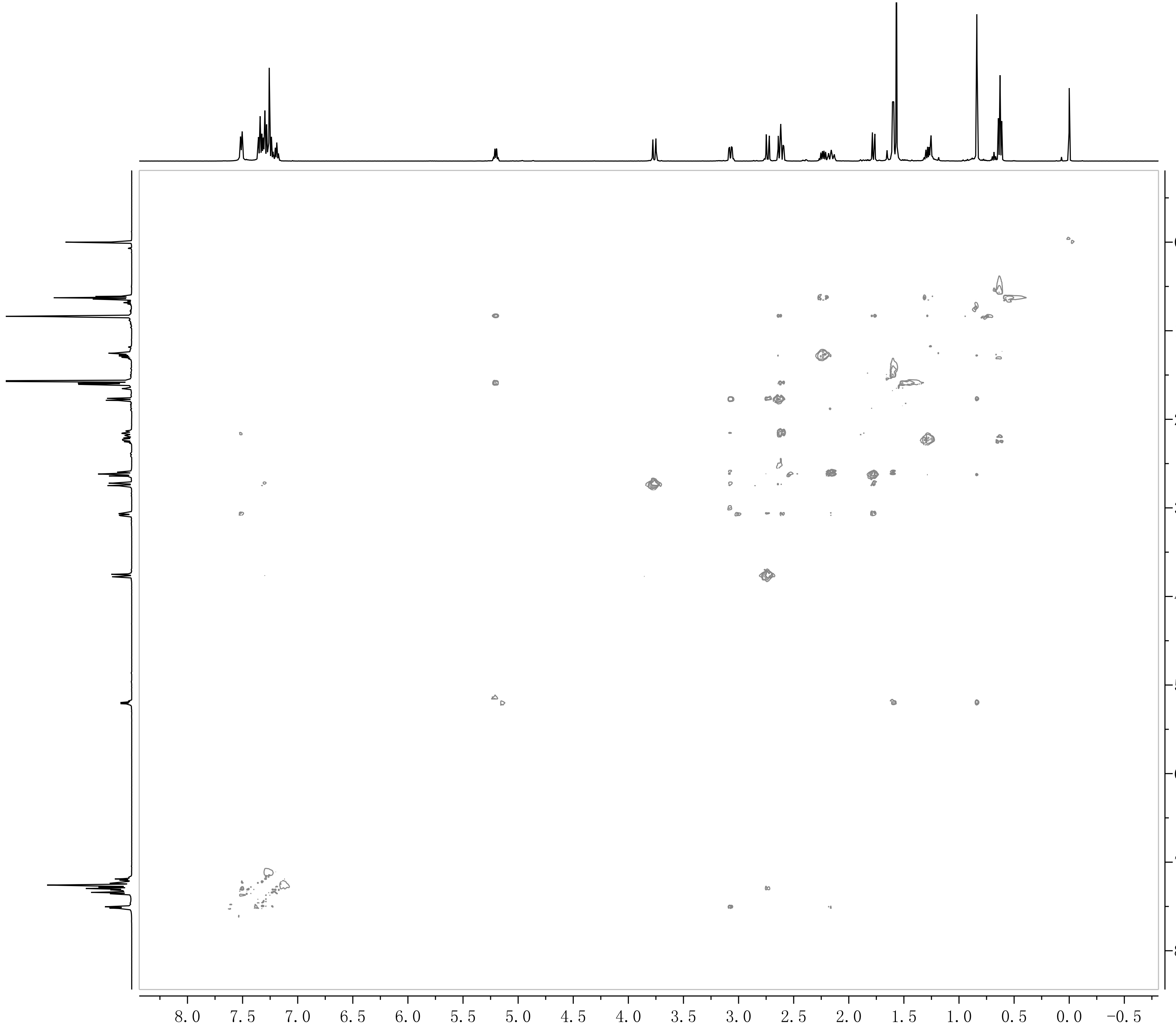

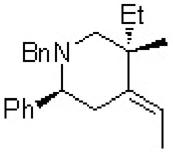

relative configuration

Parameter	Value
Title	xfy-190427-3-s1.26.ser
Comment	
Origin	Bruker BioSpin GmbH
Owner	nmr
Site	
Instrument	spect
Solvent	CDCl3
Temperature	296.1
Pulse Sequence	noesygpphpp
Experiment	NOESY
Number of Scans	8
Receiver Gain	31.1
Relaxation Delay	1.9836
Pulse Width	10.7100
Presaturation Frequency	
Acquisition Time	0.2212
Acquisition Date	2019-04-30T01:50:09
Modification Date	2019-04-30T10:09:41
Spectrometer Frequency	(500.13, 500.13)
Spectral Width	(4629.6, 4629.6)
Lowest Frequency	(-409.6, -409.6)
Nucleus	(1H, 1H)
Acquired Size	(1024, 256)
Spectral Size	(1024, 1024)

Parameter	Value
标题	xfy-190710-3-s.31.fid
Comment	
Origin	Bruker BioSpin GmbH
Owner	nmr
Site	
Instrument	spect
Author	
Solvent	CDCl3
Temperature	296.2
Pulse Sequence	zg30
Experiment	1D
Number of Scans	16
Receiver Gain	77.6
Relaxation Delay	1.0000
Pulse Width	10.7100
Presaturation Frequency	
Acquisition Time	3.2768
Class	
Spectrometer Frequency	500.13
Spectral Width	10000.0
Lowest Frequency	-1921.9
Nucleus	1H
Acquired Size	32768
Spectral Size	65536

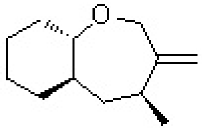

relative configuration

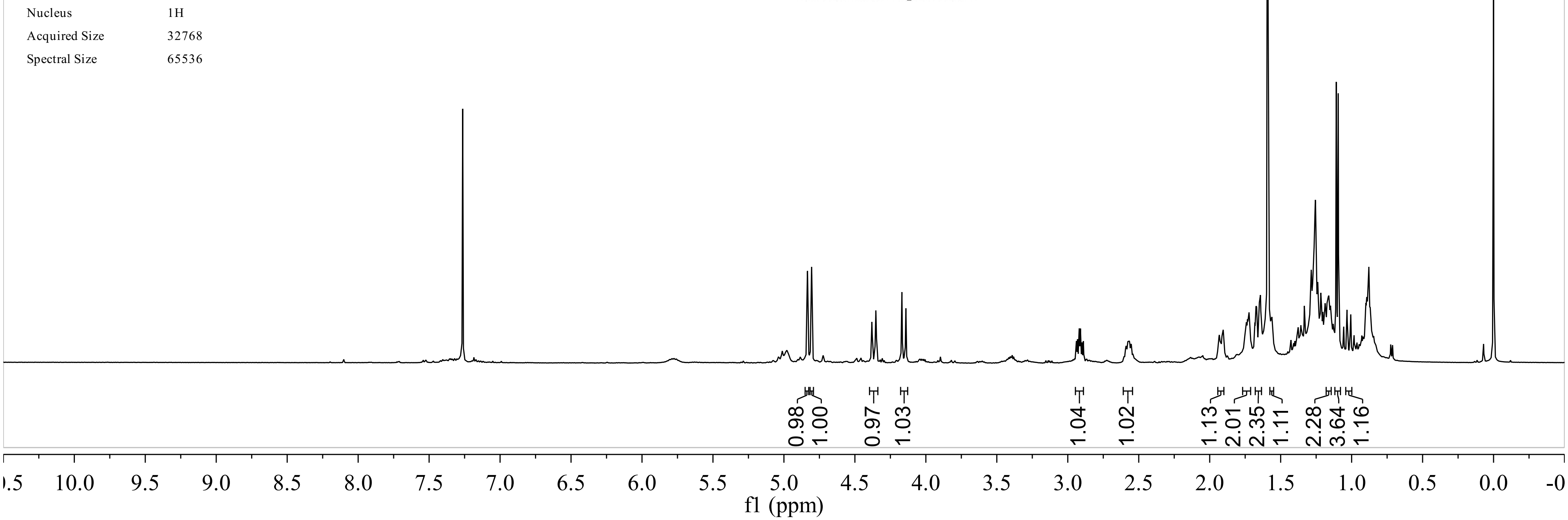

Parameter	Value
Title	xfy-190710-3-s.2.fid
Comment	
Origin	Bruker BioSpin GmbH
Owner	nmr
Site	
Instrument	spect
Solvent	CDCl3
Temperature	296.2
Pulse Sequence	zgpg30
Experiment	1D
Number of Scans	64
Receiver Gain	193.1
Relaxation Delay	2.0000
Pulse Width	9.6000
Presaturation Frequency	
Acquisition Time	1.1010
Acquisition Date	2019-07-10T18:28:25
Modification Date	2019-07-10T19:48:00
Spectrometer Frequency	125.77
Spectral Width	29761.9
Lowest Frequency	-2289.3
Nucleus	13C
Acquired Size	32768
Spectral Size	65536

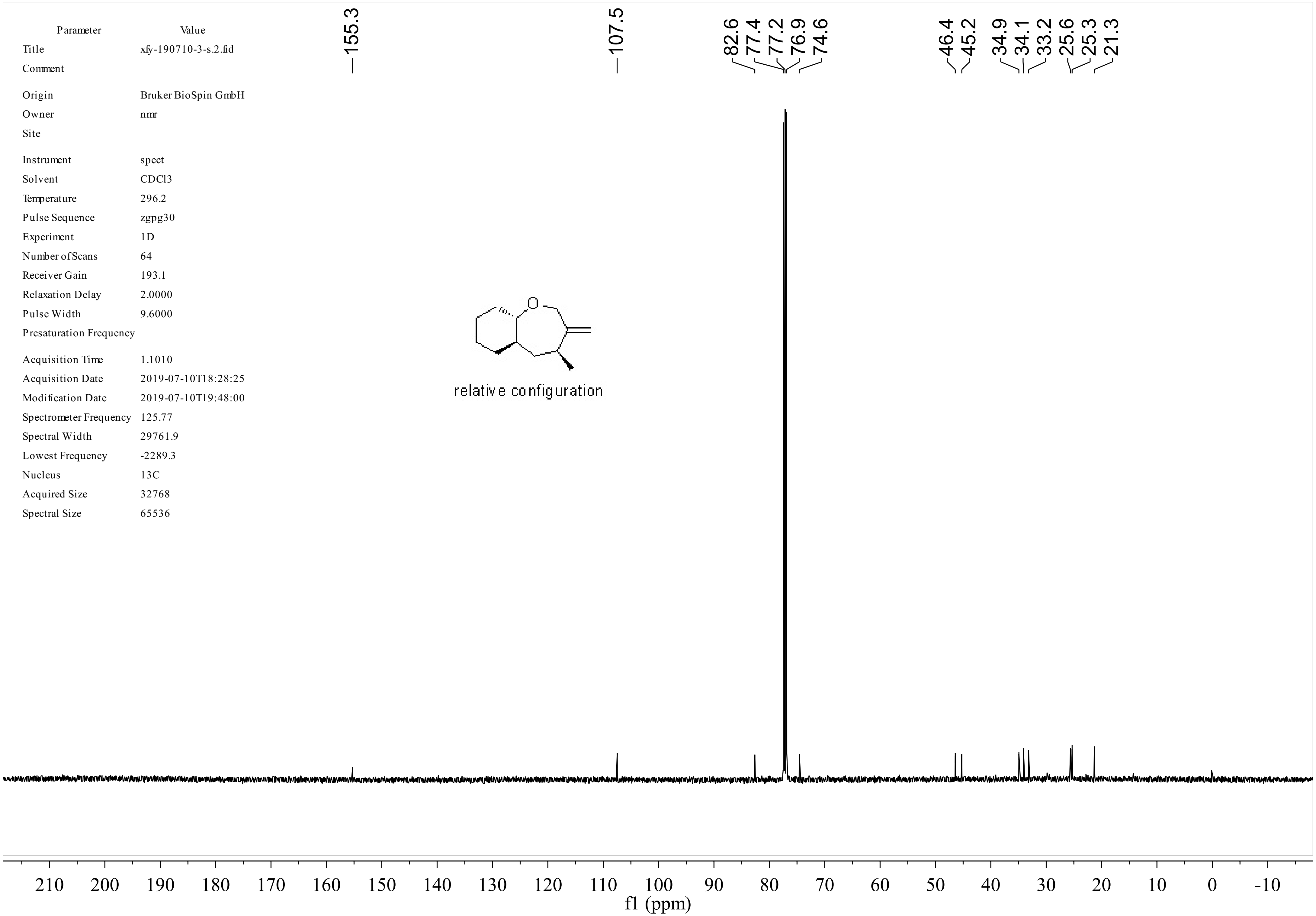

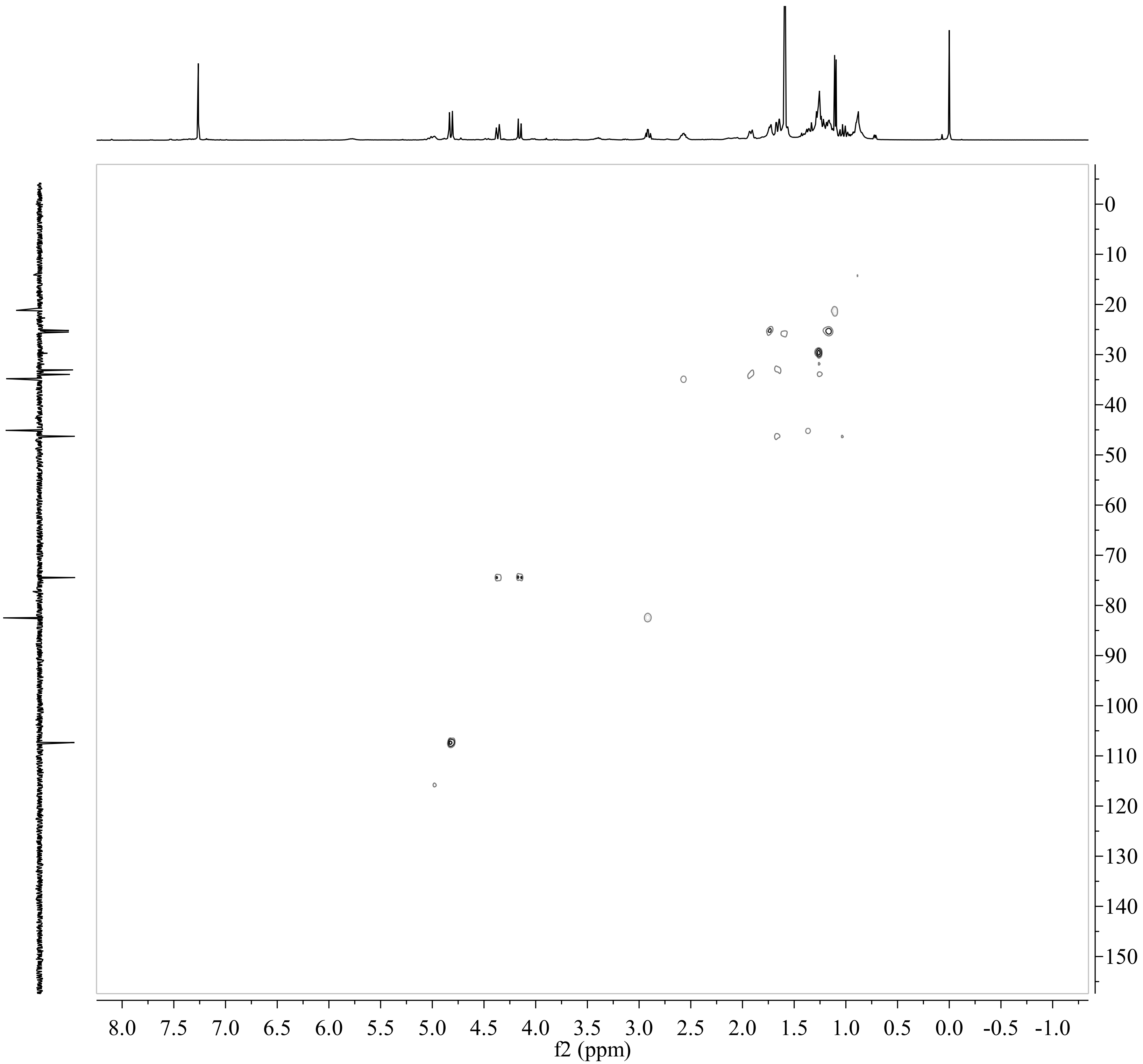

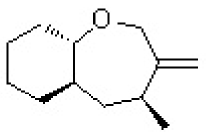

relative configuration

Parameter	Value
Title	xfy-190710-3-s.7.ser
Comment	
Origin	Bruker BioSpin GmbH
Owner	nmr
Site	
Instrument	spect
Solvent	CDCl3
Temperature	296.1
Pulse Sequence	hsqcedetgp
Experiment	HSQC-EDITED
Number of Scans	2
Receiver Gain	193.1
Relaxation Delay	1.4724
Pulse Width	10.7100
Presaturation Frequency	
Acquisition Time	0.1065
Acquisition Date	2019-07-14T12:31:26
Modification Date	2019-07-14T19:20:43
Spectrometer Frequency	(500.13, 125.77)
Spectral Width	(4807.7, 20833.3)
Lowest Frequency	(-682.8, -1037.0)
Nucleus	(1H, 13C)
Acquired Size	(512, 256)
Spectral Size	(512, 512)

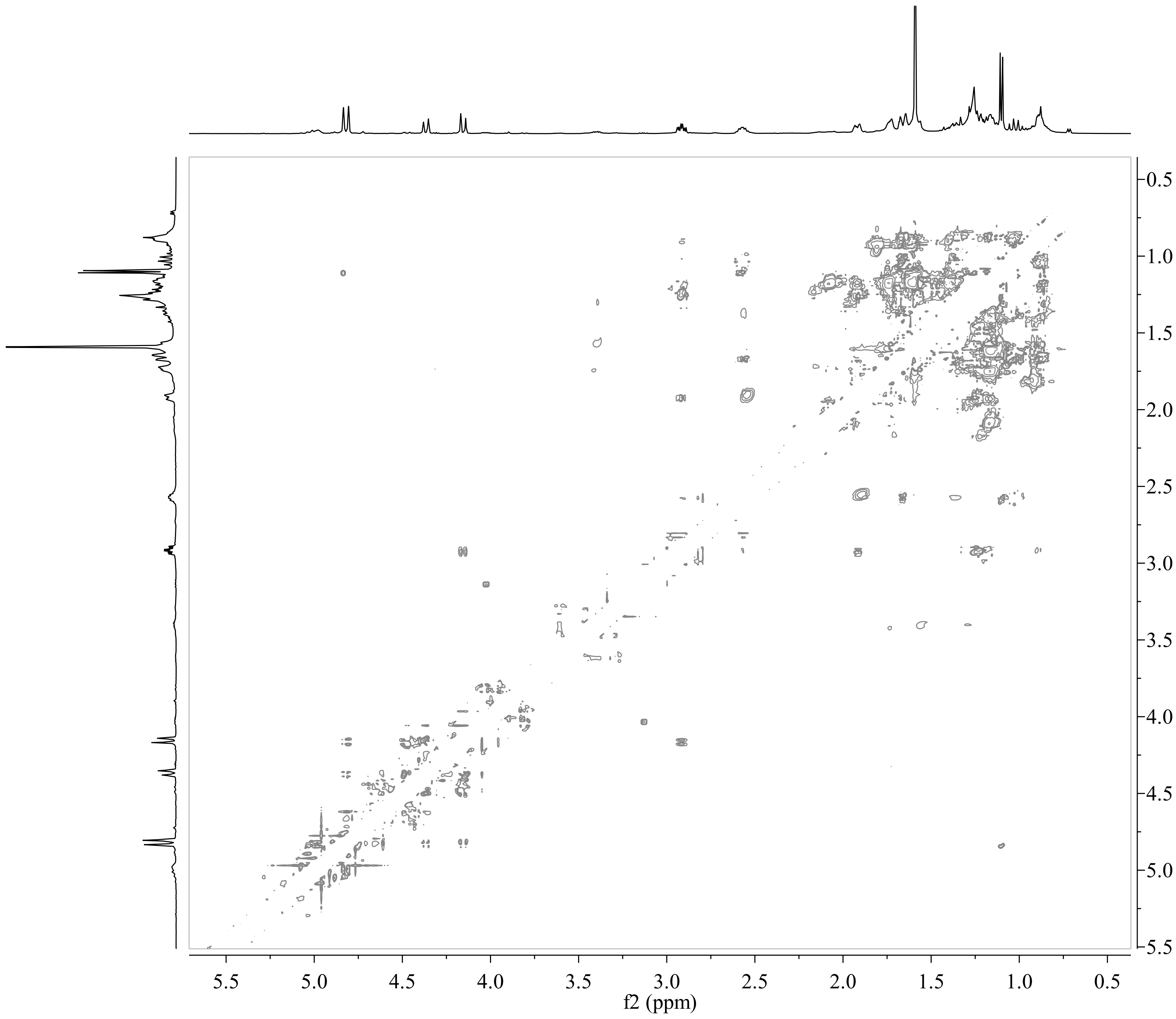

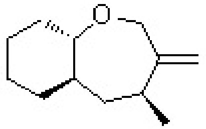

relative configuration

Parameter	Value
Title	xfy-190710-3-s.32.ser
Comment	
Origin	Bruker BioSpin GmbH
Owner	nmr
Site	
Instrument	spect
Solvent	CDCl3
Temperature	296.1
Pulse Sequence	noesygpphpp
Experiment	NOESY
Number of Scans	24
Receiver Gain	54.3
Relaxation Delay	1.9877
Pulse Width	10.7100
Presaturation Frequency	
Acquisition Time	0.2171
Acquisition Date	2019-07-23T03:40:31
Modification Date	2019-07-23T08:59:27
Spectrometer Frequency	(500.13, 500.13)
Spectral Width	(4717.0, 4717.0)
Lowest Frequency	(-642.5, -642.5)
Nucleus	(1H, 1H)
Acquired Size	(1024, 256)
Spectral Size	(1024, 1024)

Parameter	Value
Title	xfy-190720-1-s.211.1.1r
Comment	
Origin	Bruker BioSpin GmbH
Owner	nmr
Site	
Instrument	spect
Solvent	CDCl3
Temperature	297.9
Pulse Sequence	zg30
Experiment	1D
Number of Scans	8
Receiver Gain	126.1
Relaxation Delay	1.0000
Pulse Width	8.7300
Presaturation Frequency	
Acquisition Time	1.9999
Acquisition Date	2019-07-20T18:07:32
Modification Date	2019-07-21T20:24:28
Spectrometer Frequency	400.13
Spectral Width	8012.8
Lowest Frequency	-1535.4
Nucleus	1H
Acquired Size	16025
Spectral Size	65536

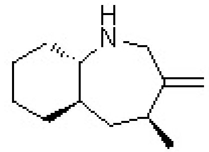

relative configuration

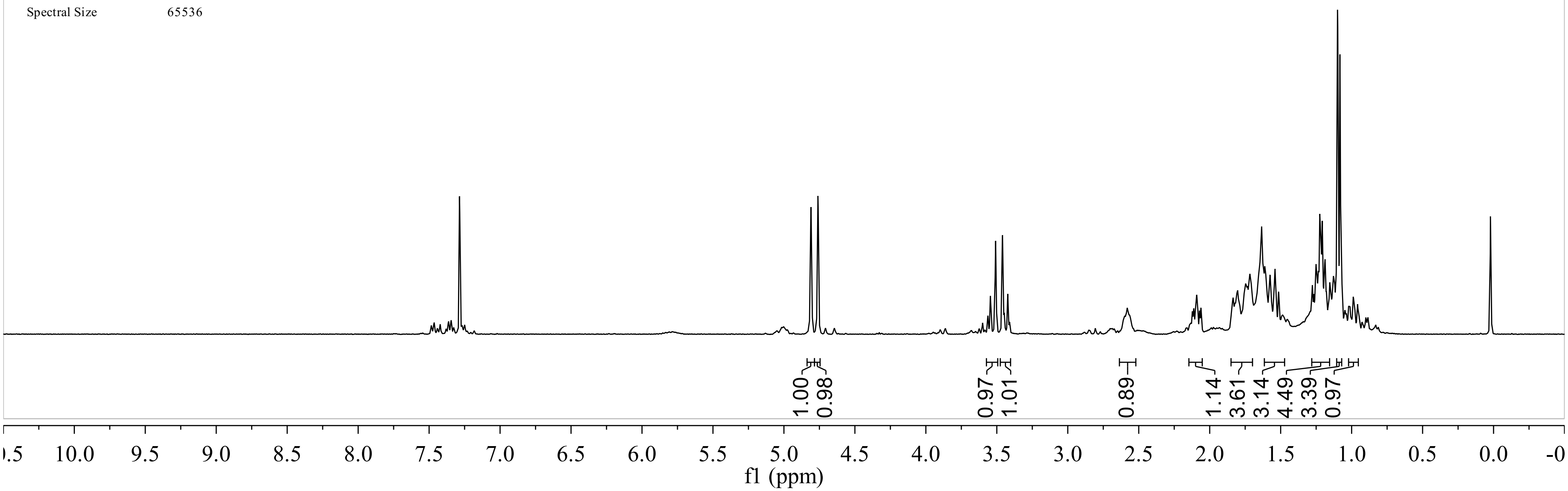

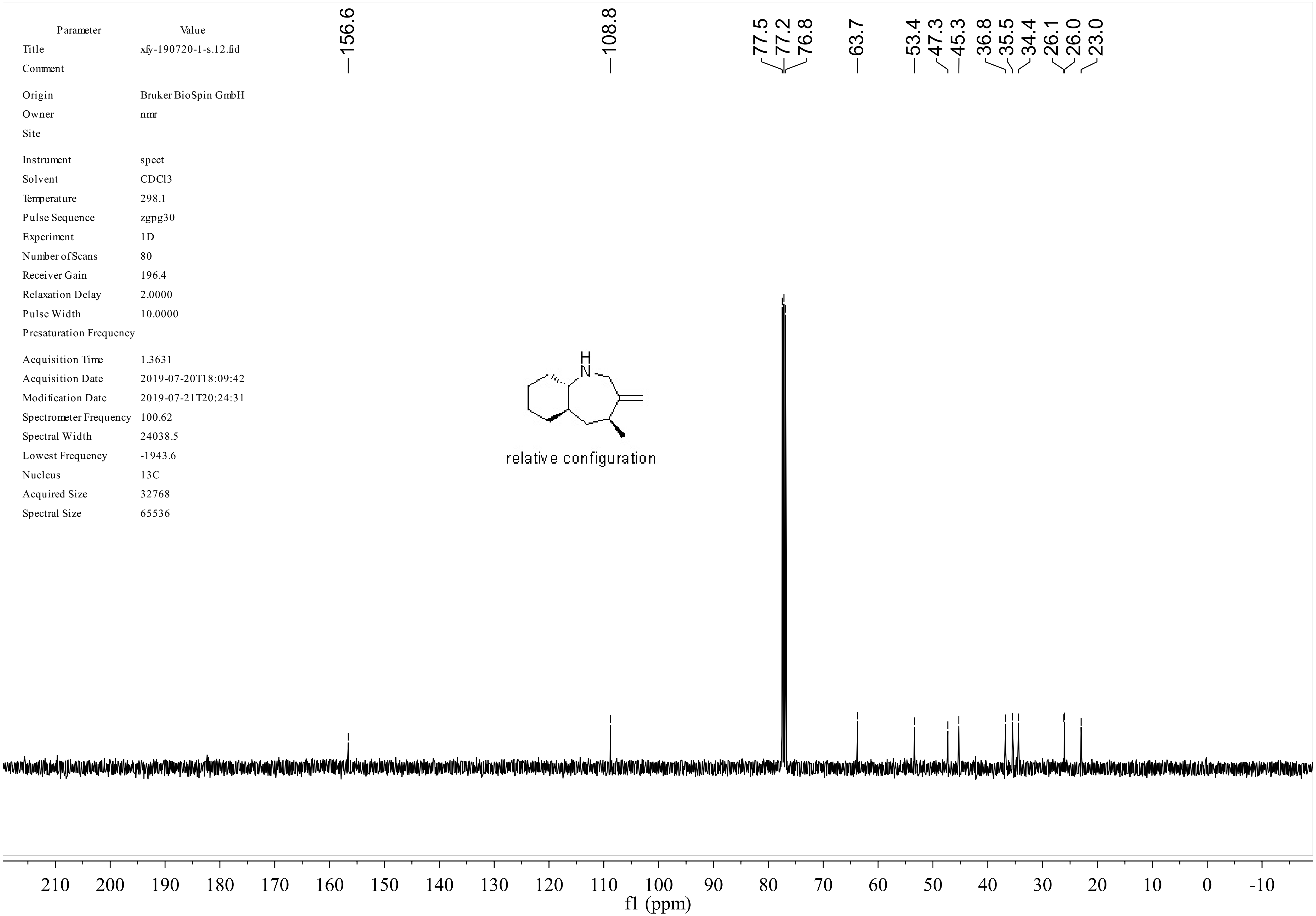

Parameter Value
Title xfy-190720-1-s.12.fid
Comment
Origin Bruker BioSpin GmbH
Owner nmr
Site
Instrument spect
Solvent CDCl3
Temperature 298.1
Pulse Sequence zgpg30
Experiment 1D
Number of Scans 80
Receiver Gain 196.4
Relaxation Delay 2.0000
Pulse Width 10.0000
Presaturation Frequency
Acquisition Time 1.3631
Acquisition Date 2019-07-20T18:09:42
Modification Date 2019-07-21T20:24:31
Spectrometer Frequency 100.62
Spectral Width 24038.5
Lowest Frequency -1943.6
Nucleus 13C
Acquired Size 32768
Spectral Size 65536
156.6
108.8
77.5
77.2
76.8
63.7
53.4
47.3
45.3
36.8
35.5
34.4
26.1
26.0
23.0
relative configuration
210 200 190 180 170 160 150 140 130 120 110 100 90 80 70 60 50 40 30 20 10 0 -10
f1 (ppm)

Parameter	Value
Title	xfy-190720-1-dMs.1.1.1r
Comment	
Origin	Bruker BioSpin GmbH
Owner	nmr
Site	
Instrument	spect
Solvent	CDCl3
Temperature	298.3
Pulse Sequence	zg30
Experiment	1D
Number of Scans	8
Receiver Gain	142.1
Relaxation Delay	1.0000
Pulse Width	8.7300
Presaturation Frequency	
Acquisition Time	1.9999
Acquisition Date	2019-07-21T12:20:11
Modification Date	2019-07-21T12:55:23
Spectrometer Frequency	400.13
Spectral Width	8012.8
Lowest Frequency	-1543.7
Nucleus	1H
Acquired Size	16025
Spectral Size	65536

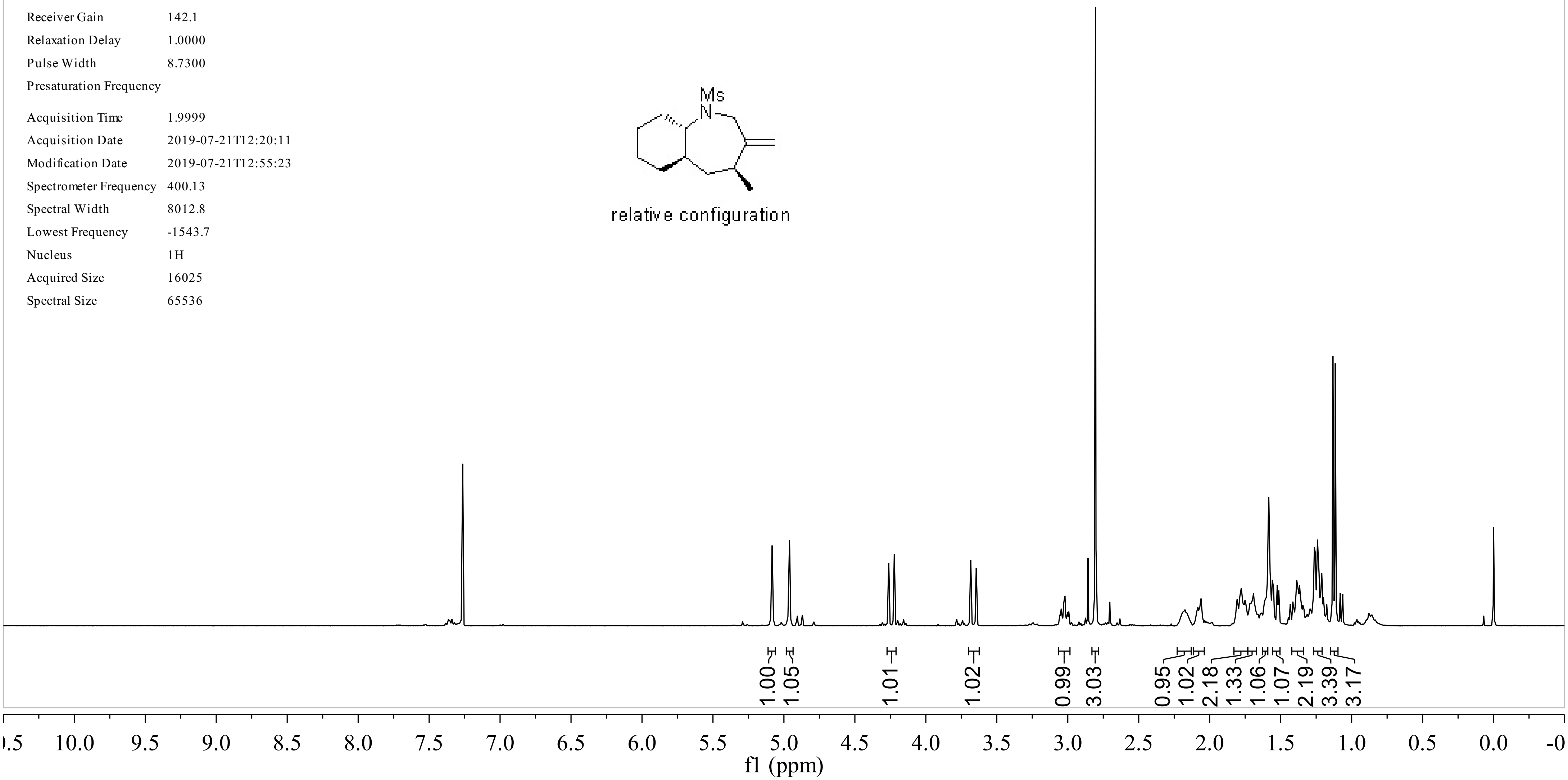

Parameter	Value
Title	xfy-190720-1-dMs.2.1.1r
Comment	
Origin	Bruker BioSpin GmbH
Owner	nmr
Site	
Instrument	spect
Solvent	CDCl3
Temperature	298.5
Pulse Sequence	zgpg30
Experiment	1D
Number of Scans	100
Receiver Gain	196.4
Relaxation Delay	2.0000
Pulse Width	10.0000
Presaturation Frequency	
Acquisition Time	1.3631
Acquisition Date	2019-07-21T12:21:47
Modification Date	2019-07-21T12:55:23
Spectrometer Frequency	100.61
Spectral Width	24038.5
Lowest Frequency	-1943.9
Nucleus	13C
Acquired Size	32768
Spectral Size	32768

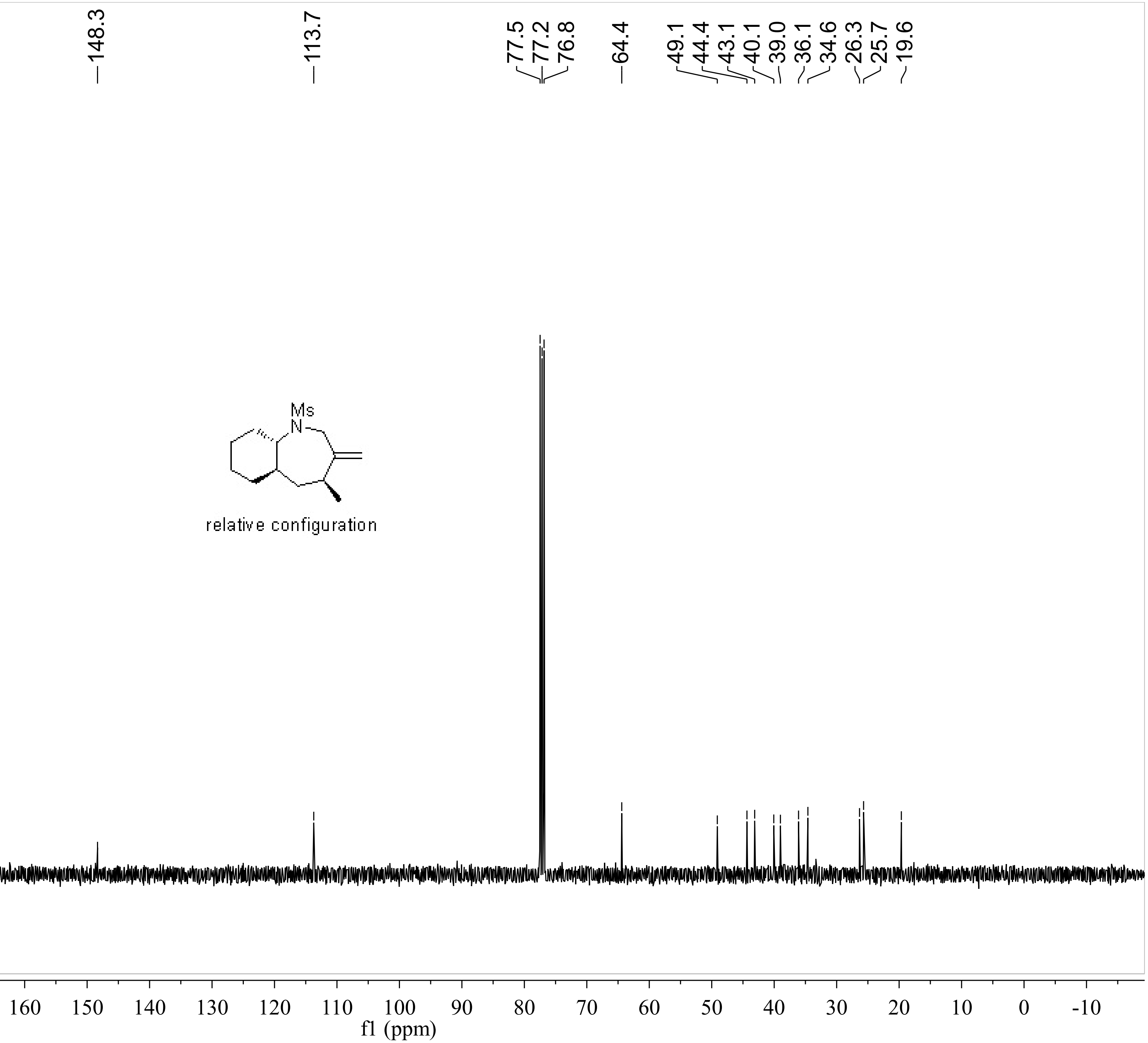

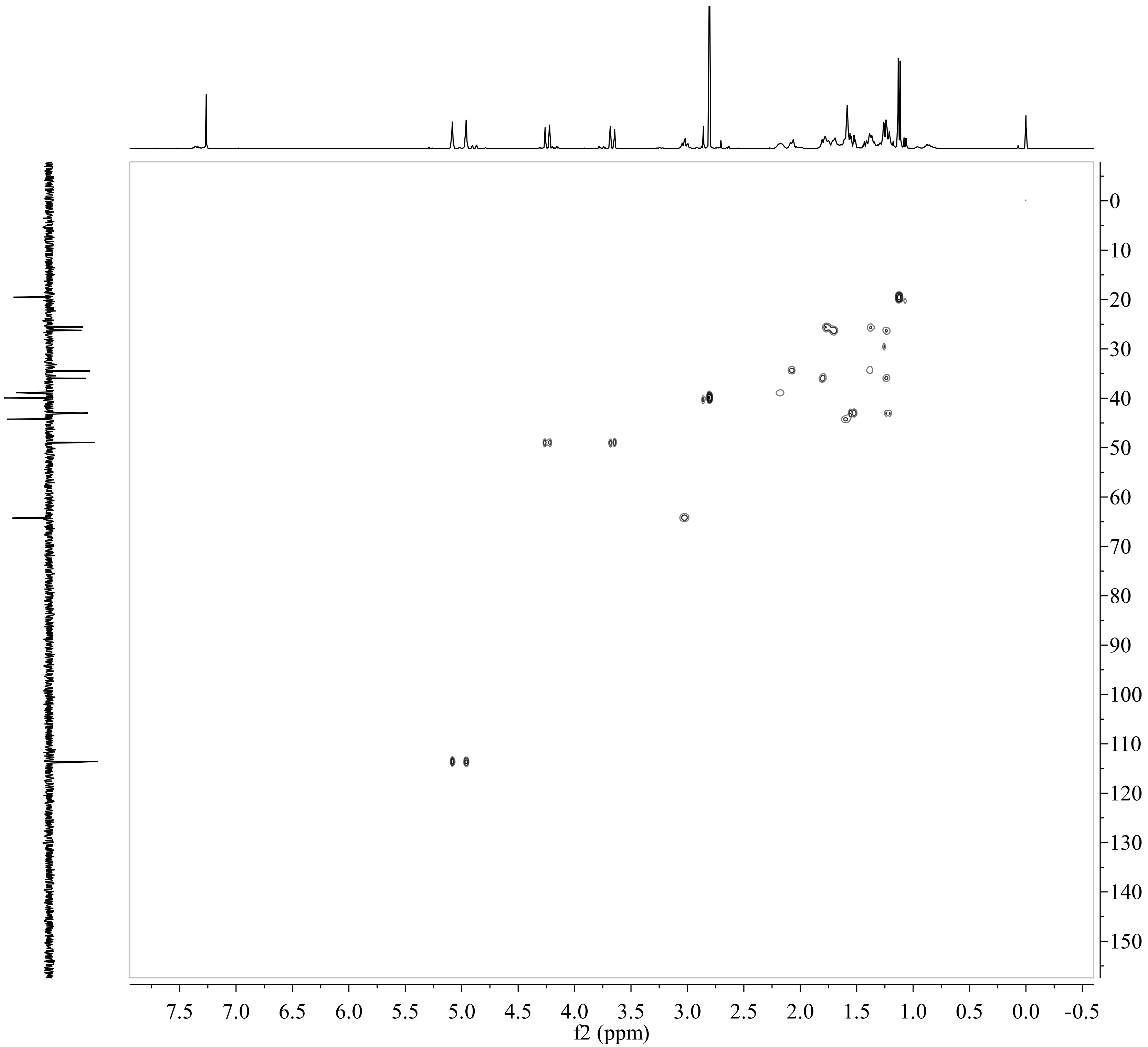

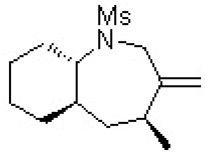

relative configuration

Parameter	Value
Title	xfy-190720-1-dMs.3.ser
Comment	
Origin	Bruker BioSpin GmbH
Owner	nmr
Site	
Instrument	spect
Solvent	CDCl3
Temperature	298.7
Pulse Sequence	hsqcetgp
Experiment	HSQC
Number of Scans	2
Receiver Gain	196.4
Relaxation Delay	1.4490
Pulse Width	8.7300
Presaturation Frequency	
Acquisition Time	0.1495
Acquisition Date	2019-07-21T12:28:43
Modification Date	2019-07-21T12:54:59
Spectrometer Frequency	(400.13, 100.62)
Spectral Width	(3424.7, 16666.7)
Lowest Frequency	(-246.6, -829.1)
Nucleus	(1H, 13C)
Acquired Size	(512, 256)
Spectral Size	(512, 512)

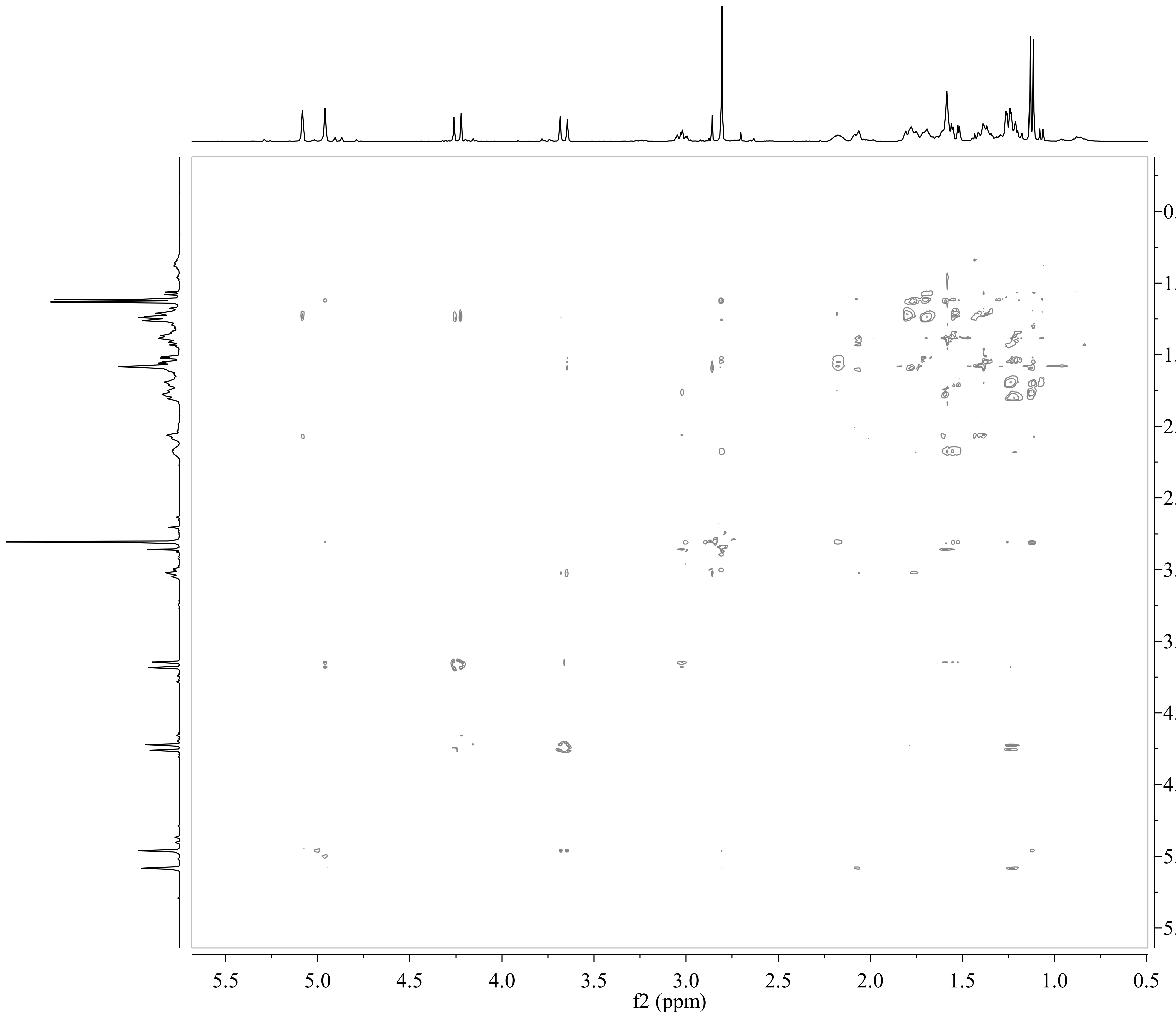

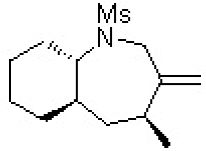

relative configuration

Parameter	Value
Title	xfy-190720-1-dMs.32.ser
Comment	
Origin	Bruker BioSpin GmbH
Owner	nmr
Site	
Instrument	spect
Solvent	CDCl3
Temperature	296.1
Pulse Sequence	noesygpphpp
Experiment	NOESY
Number of Scans	24
Receiver Gain	48.5
Relaxation Delay	1.9713
Pulse Width	10.7100
Presaturation Frequency	
Acquisition Time	0.2335
Acquisition Date	2019-07-22T23:11:38
Modification Date	2019-07-23T08:59:08
Spectrometer Frequency	(500.13, 500.13)
Spectral Width	(4386.0, 4386.0)
Lowest Frequency	(-340.0, -340.0)
Nucleus	(1H, 1H)
Acquired Size	(1024, 256)
Spectral Size	(1024, 1024)

Parameter	Value
标题	xfy-190723-1-s1.1.1.1r
Comment	
Origin	Bruker BioSpin GmbH
Owner	nmr
Site	
Instrument	spect
Author	
Solvent	CDCl3
Temperature	296.2
Pulse Sequence	zg30
Experiment	1D
Number of Scans	16
Receiver Gain	95.3
Relaxation Delay	1.0000
Pulse Width	10.7100
Presaturation Frequency	
Acquisition Time	3.2768
Class	
Spectrometer Frequency	500.13
Spectral Width	10000.0
Lowest Frequency	-1924.2
Nucleus	1H
Acquired Size	32768
Spectral Size	65536

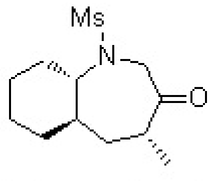

relative configuration

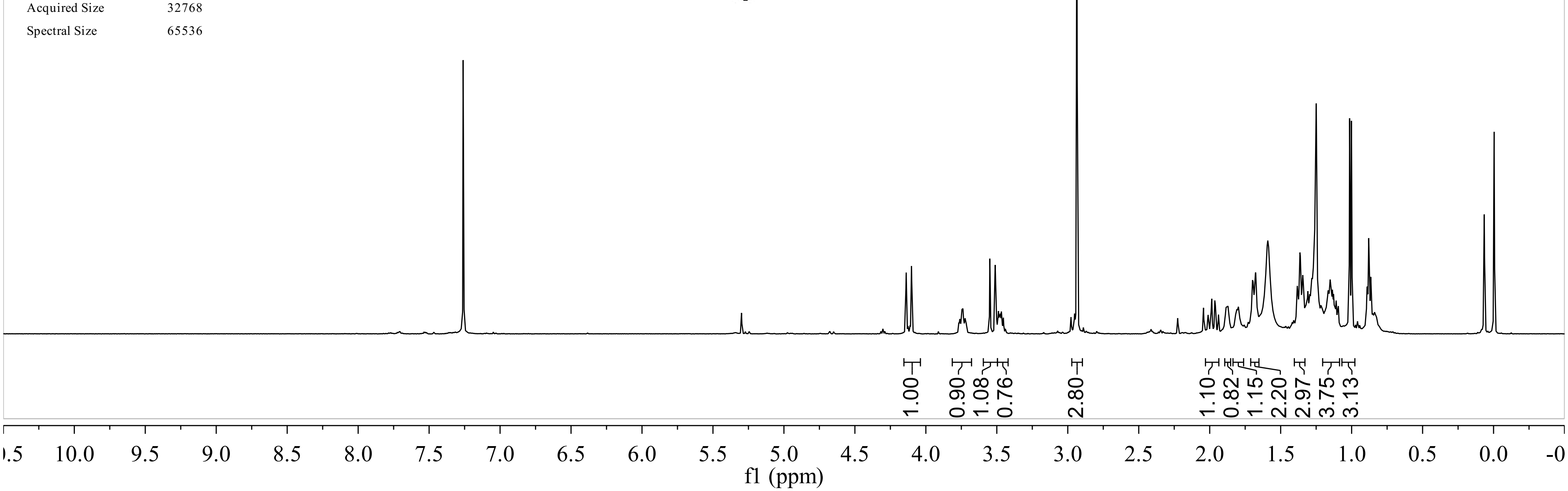

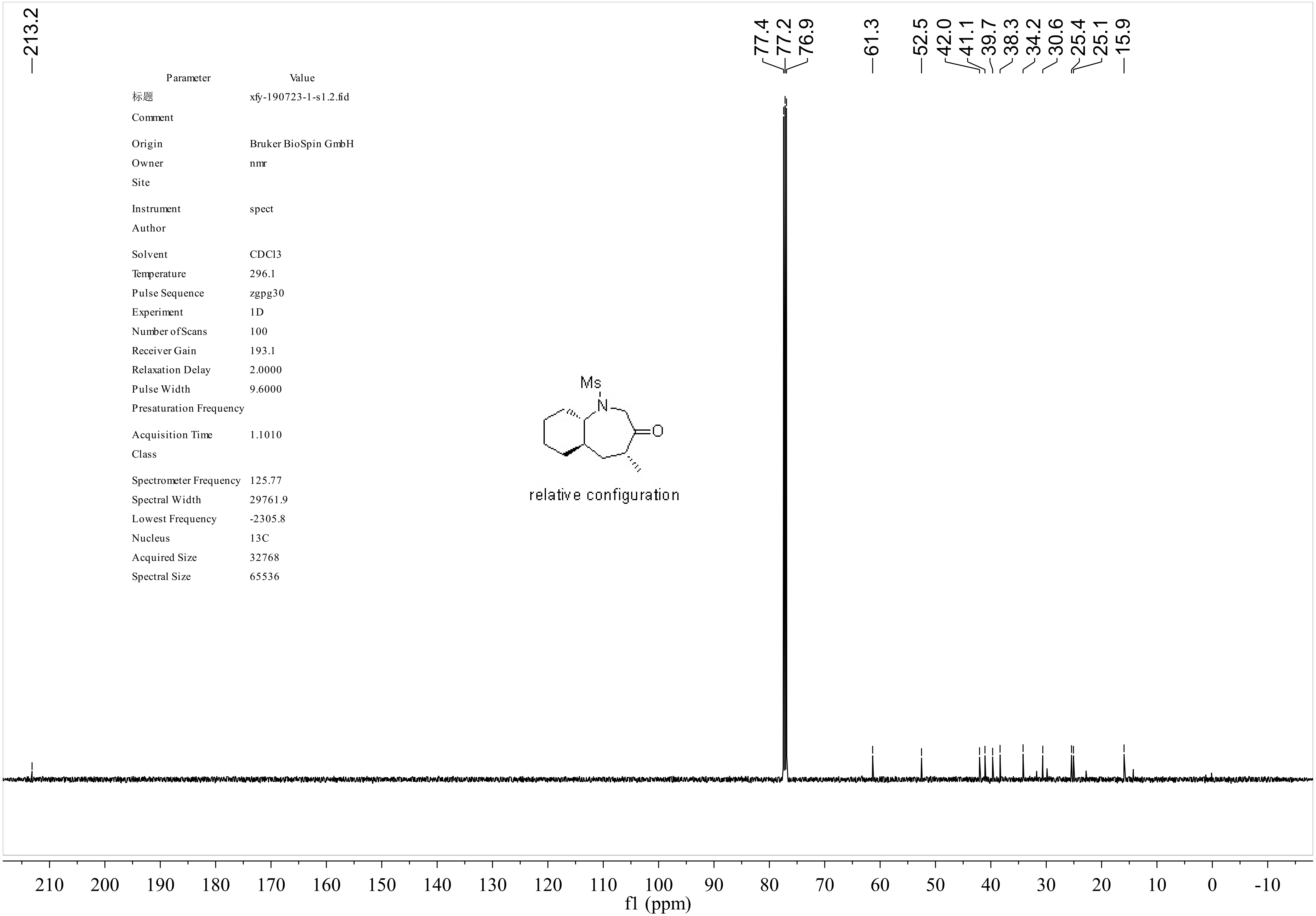

Parameter	Value
标题	xfy-190723-1-s1.2.fid
Comment	
Origin	Bruker BioSpin GmbH
Owner	nmr
Site	
Instrument	spect
Author	
Solvent	CDCl3
Temperature	296.1
Pulse Sequence	zgpg30
Experiment	1D
Number of Scans	100
Receiver Gain	193.1
Relaxation Delay	2.0000
Pulse Width	9.6000
Presaturation Frequency	
Acquisition Time	1.1010
Class	
Spectrometer Frequency	125.77
Spectral Width	29761.9
Lowest Frequency	-2305.8
Nucleus	13C
Acquired Size	32768
Spectral Size	65536

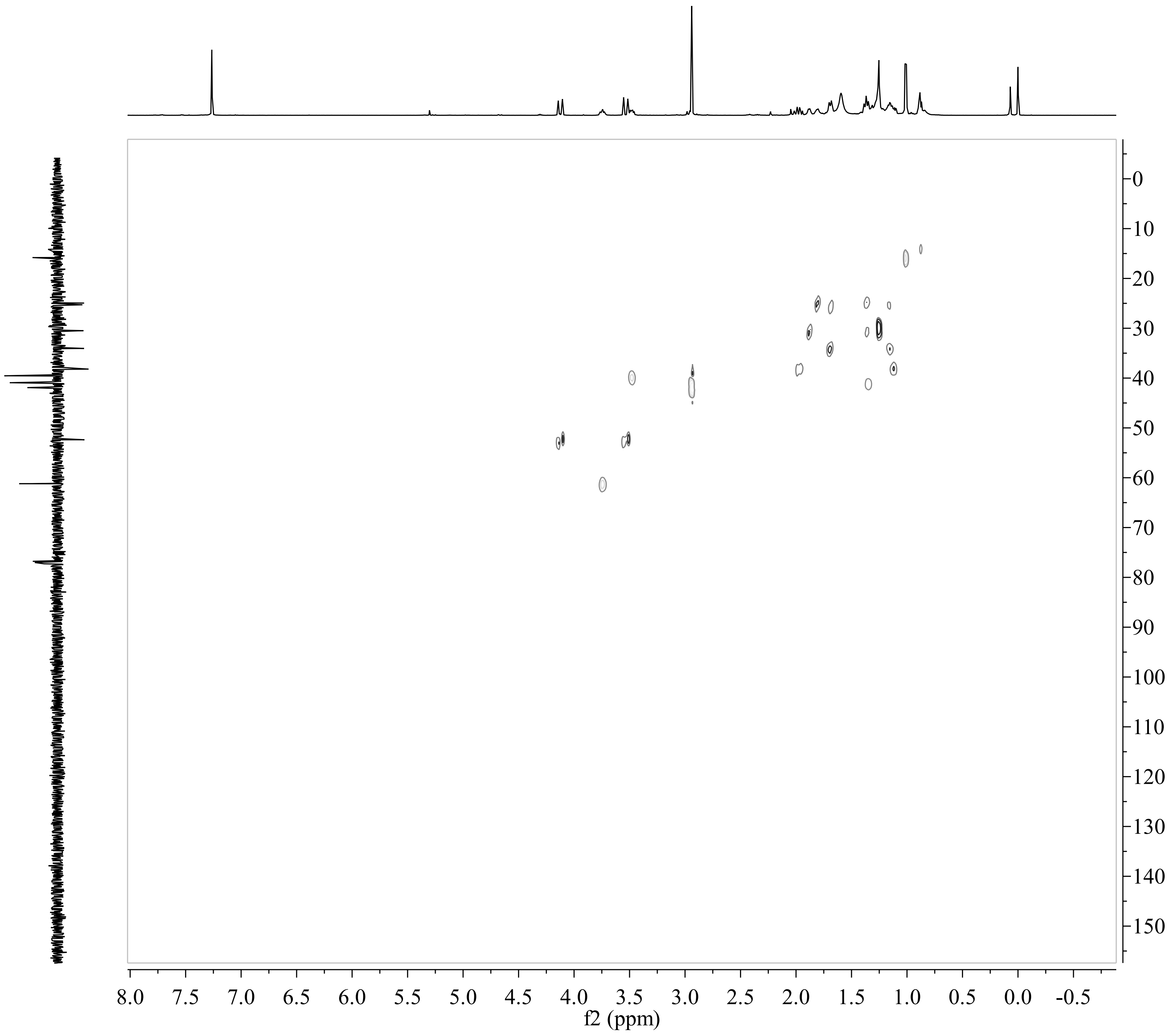

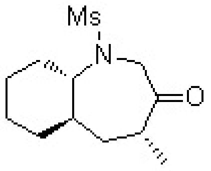

relative configuration

Parameter	Value
Title	xfy-190723-1-s1.4.ser
Comment	
Origin	Bruker BioSpin GmbH
Owner	nmr
Site	
Instrument	spect
Solvent	CDCl3
Temperature	296.1
Pulse Sequence	hsqcedetgp
Experiment	HSQC-EDITED
Number of Scans	2
Receiver Gain	193.1
Relaxation Delay	1.4642
Pulse Width	10.7100
Presaturation Frequency	
Acquisition Time	0.1147
Acquisition Date	2019-07-23T14:34:13
Modification Date	2019-07-23T15:24:29
Spectrometer Frequency	(500.13, 125.77)
Spectral Width	(4464.3, 20833.3)
Lowest Frequency	(-451.6, -1037.0)
Nucleus	(1H, 13C)
Acquired Size	(512, 132)
Spectral Size	(512, 512)

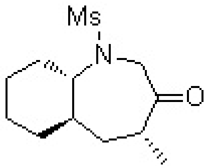

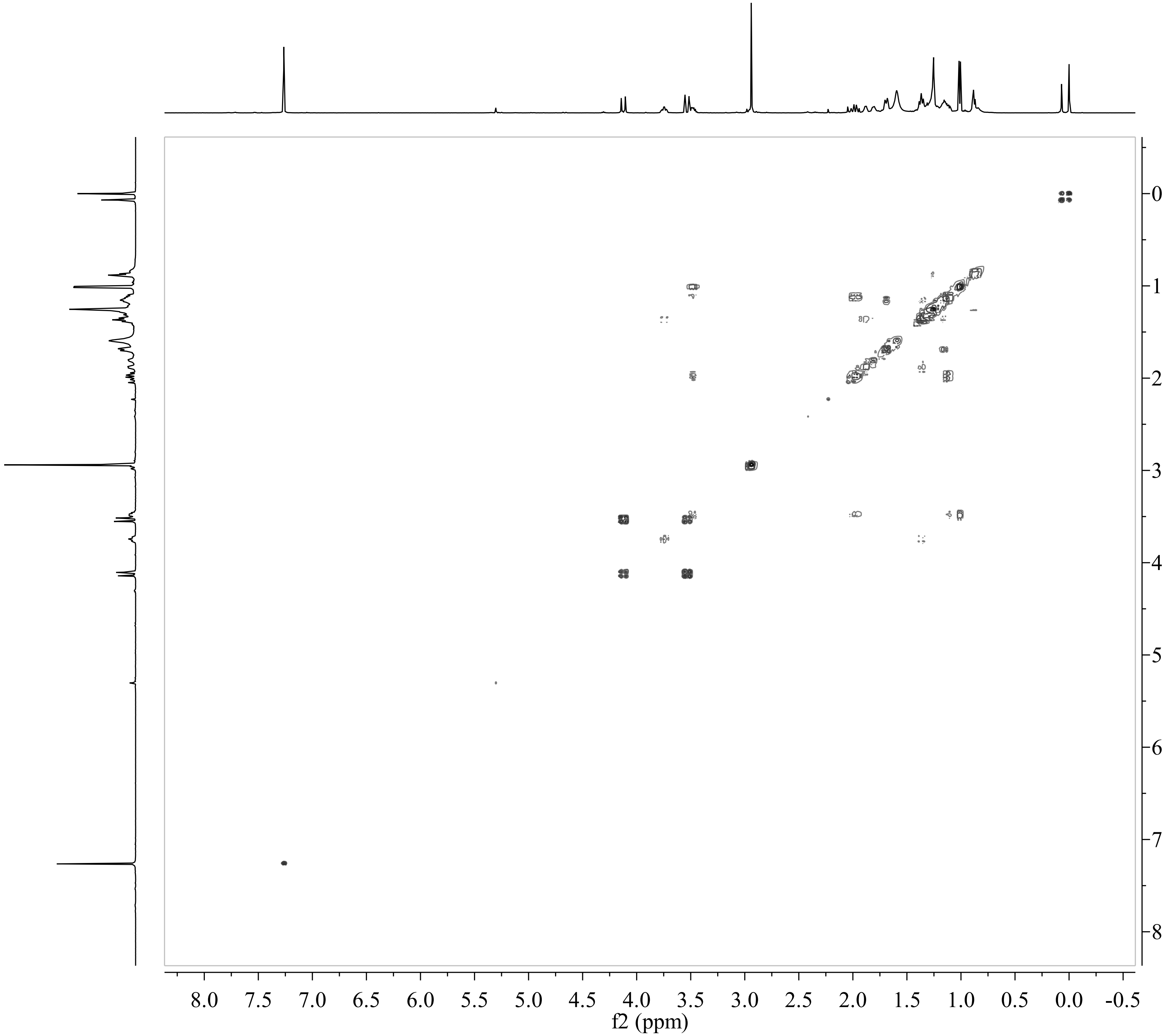

Parameter	Value
Title	xfy-190723-1-s1.24.ser
Comment	
Origin	Bruker BioSpin GmbH
Owner	nmr
Site	
Instrument	spect
Solvent	CDCl3
Temperature	297.3
Pulse Sequence	cosygpppqf
Experiment	COSY
Number of Scans	4
Receiver Gain	62.3
Relaxation Delay	1.9070
Pulse Width	8.7300
Presaturation Frequency	
Acquisition Time	0.2847
Acquisition Date	2019-07-24T01:04:06
Modification Date	2019-07-24T08:33:39
Spectrometer Frequency	(400.13, 400.13)
Spectral Width	(3597.1, 3597.1)
Lowest Frequency	(-248.9, -248.9)
Nucleus	(1H, 1H)
Acquired Size	(1024, 128)
Spectral Size	(1024, 1024)

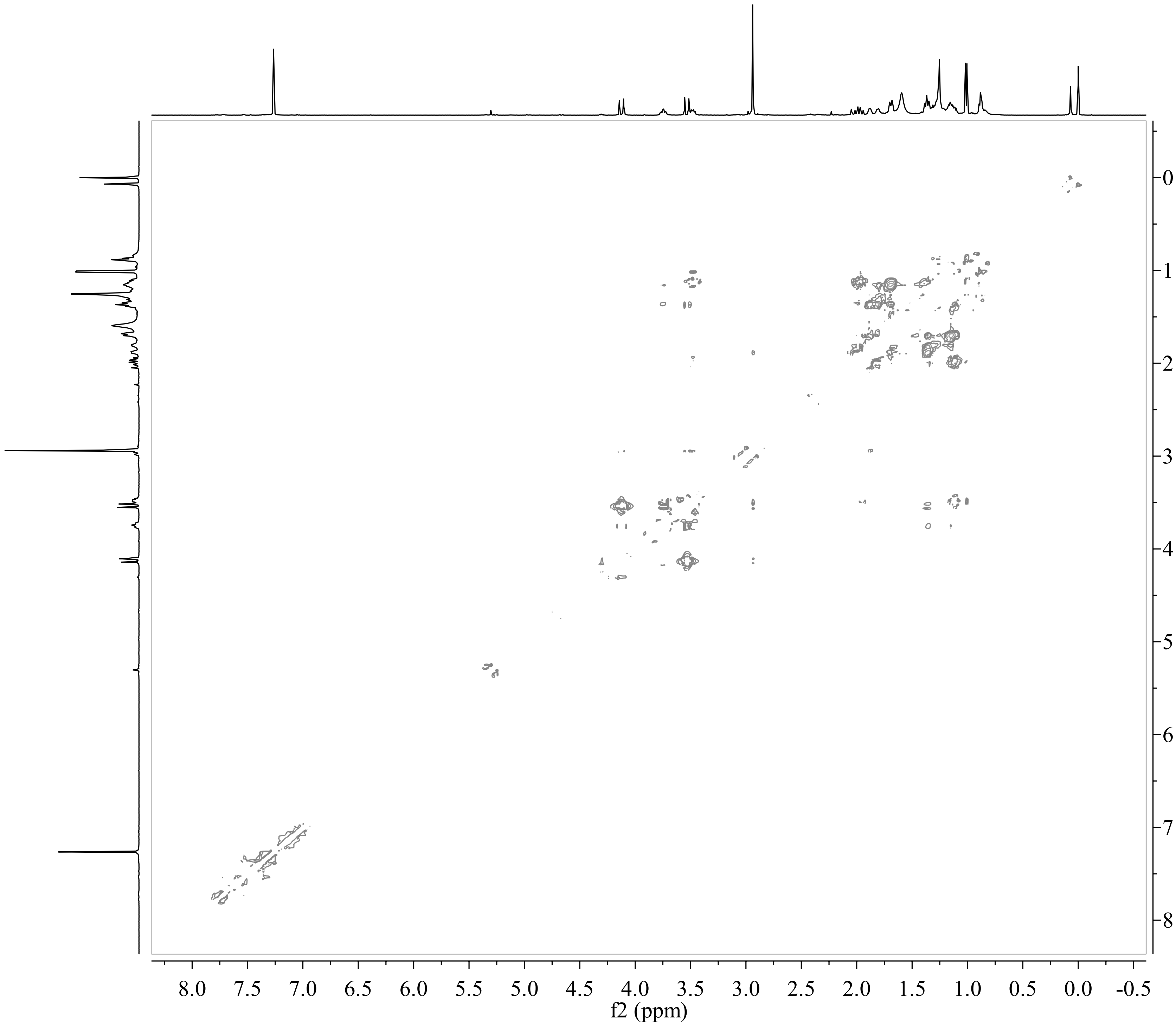

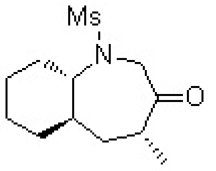

relative configuration

Parameter	Value
Title	xfy-190723-1-s1.25.ser
Comment	
Origin	Bruker BioSpin GmbH
Owner	nmr
Site	
Instrument	spect
Solvent	CDCl3
Temperature	296.9
Pulse Sequence	noesygpphpp
Experiment	NOESY
Number of Scans	36
Receiver Gain	62.3
Relaxation Delay	1.9713
Pulse Width	8.7300
Presaturation Frequency	
Acquisition Time	0.2847
Acquisition Date	2019-07-24T01:26:48
Modification Date	2019-07-24T08:33:39
Spectrometer Frequency	(400.13, 400.13)
Spectral Width	(3597.1, 3597.1)
Lowest Frequency	(-249.3, -249.3)
Nucleus	(1H, 1H)
Acquired Size	(1024, 256)
Spectral Size	(1024, 1024)

Parameter	Value
Title	xfy-190710-2-s2.1.1.1r
Comment	
Origin	Bruker BioSpin GmbH
Owner	nmr
Site	
Instrument	spect
Solvent	CDCl3
Temperature	296.2
Pulse Sequence	zg30
Experiment	1D
Number of Scans	4
Receiver Gain	87.5
Relaxation Delay	1.0000
Pulse Width	10.7100
Presaturation Frequency	
Acquisition Time	3.2768
Acquisition Date	2019-07-22T17:42:00
Modification Date	2019-07-22T20:29:45
Spectrometer Frequency	500.13
Spectral Width	10000.0
Lowest Frequency	-1921.7
Nucleus	1H
Acquired Size	32768
Spectral Size	65536

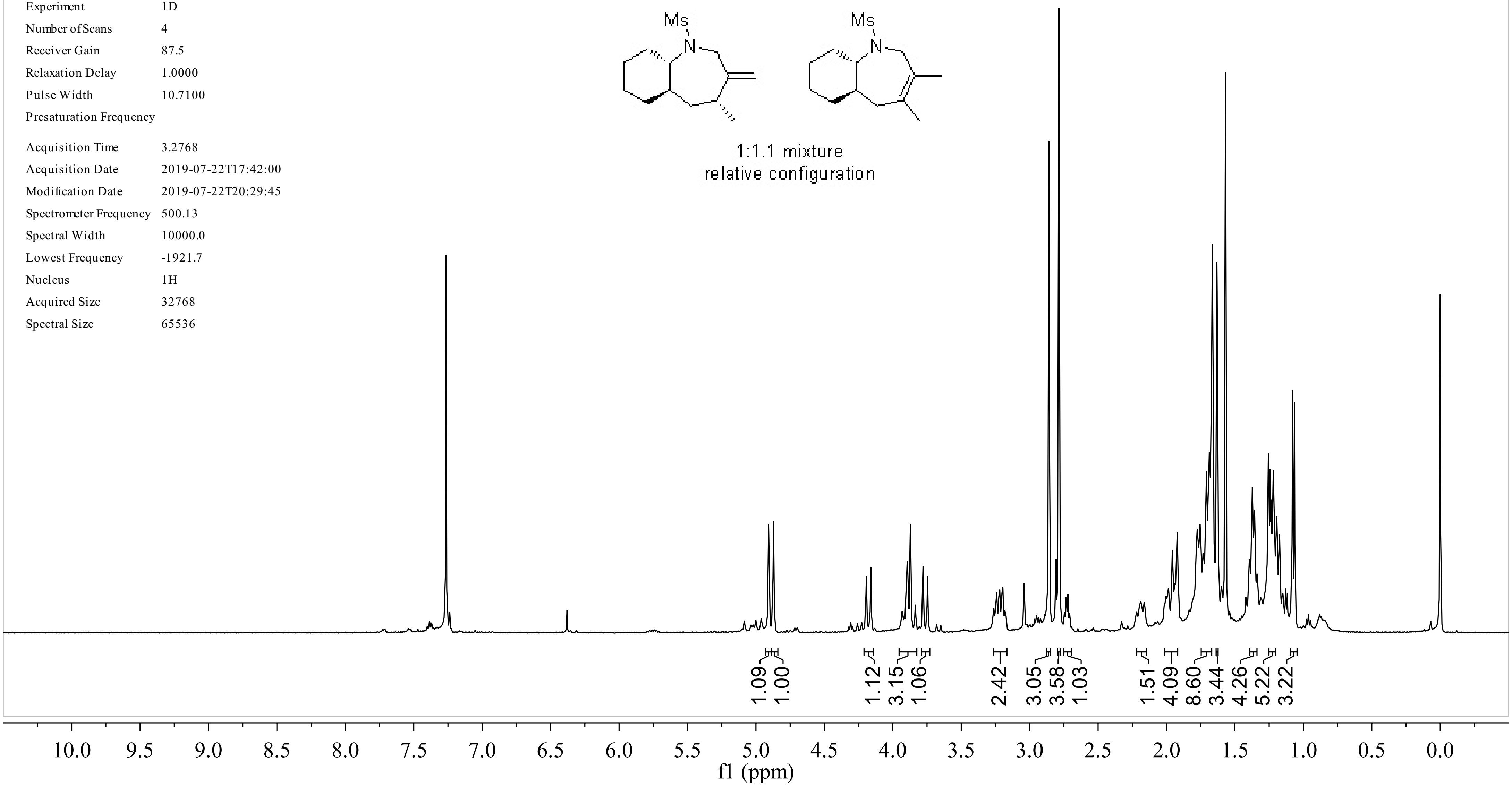

Parameter	Value
Title	xfy-190710-2-s2.3.1.1r
Comment	
Origin	Bruker BioSpin GmbH
Owner	nmr
Site	
Instrument	spect
Solvent	CDCl3
Temperature	296.2
Pulse Sequence	deptsp135
Experiment	DEPT-135
Number of Scans	86
Receiver Gain	193.1
Relaxation Delay	2.0000
Pulse Width	9.6000
Presaturation Frequency	
Acquisition Time	1.0486
Acquisition Date	2019-07-22T18:29:00
Modification Date	2019-07-22T20:29:46
Spectrometer Frequency	125.76
Spectral Width	31250.0
Lowest Frequency	-518.2
Nucleus	13C
Acquired Size	32768
Spectral Size	32768

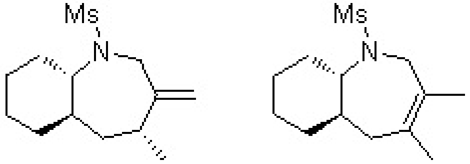

1:1.1 mixture
relative configuration

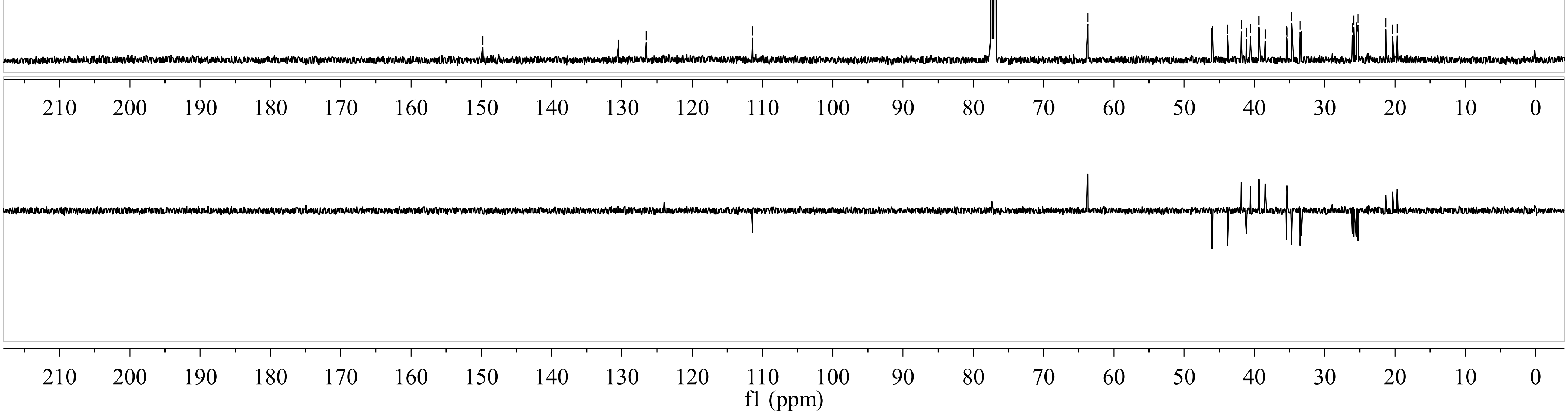

Parameter	Value
Data File Name	E:/ NMR/ 2017/ 2017-5(10-17)/ xfy-0517-2/ 1/ fid
Comment	
Origin	Bruker BioSpin GmbH
Owner	nmr
Site	
Instrument	spect
Solvent	CDCl3
Temperature	296.0
Pulse Sequence	zg30
Experiment	1D
Number of Scans	8
Receiver Gain	97.6
Relaxation Delay	1.0000
Pulse Width	9.6000
Presaturation Frequency	
Acquisition Time	1.9999
Class	
Spectrometer Frequency	400.13
Spectral Width	8012.8
Lowest Frequency	-1544.4
Nucleus	1H
Acquired Size	16025
Spectral Size	32768

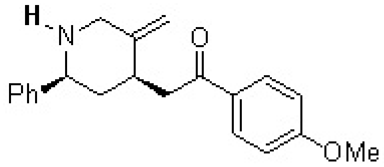

relative configuration only

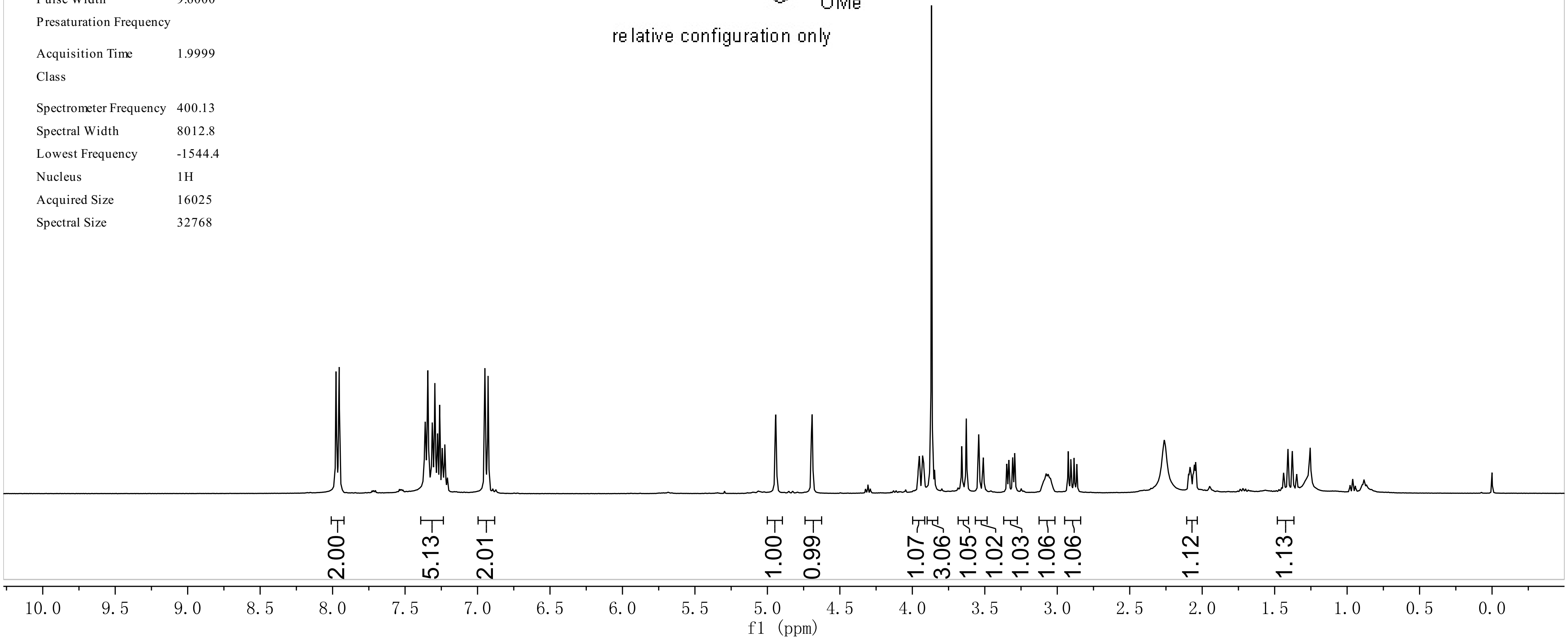

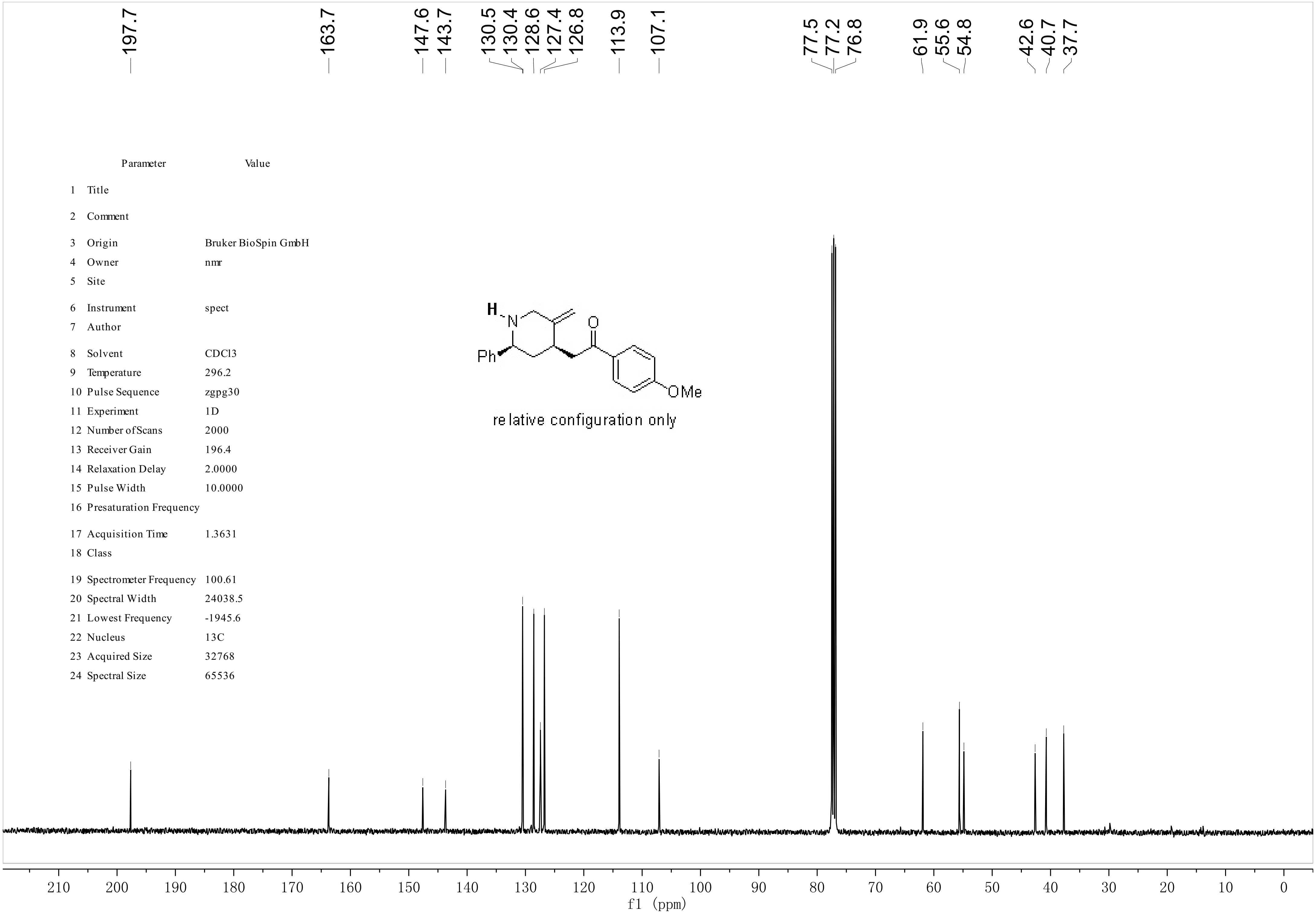
197.7
163.7
147.6
143.7
130.5
130.4
128.6
127.4
126.8
113.9
107.1
77.5
77.2
76.8
61.9
55.6
54.8
42.6
40.7
37.7
Parameter Value
1 Title
2 Comment
3 Origin Bruker BioSpin GmbH
4 Owner nmr
5 Site
6 Instrument spect
7 Author
8 Solvent CDCl3
9 Temperature 296.2
10 Pulse Sequence zgpg30
11 Experiment 1D
12 Number of Scans 2000
13 Receiver Gain 196.4
14 Relaxation Delay 2.0000
15 Pulse Width 10.0000
16 Presaturation Frequency
17 Acquisition Time 1.3631
18 Class
19 Spectrometer Frequency 100.61
20 Spectral Width 24038.5
21 Lowest Frequency -1945.6
22 Nucleus 13C
23 Acquired Size 32768
24 Spectral Size 65536
H
N
O
Ph
OMe
relative configuration only
210 200 190 180 170 160 150 140 130 120 110 100 90 80 70 60 50 40 30 20 10 0
f1 (ppm)

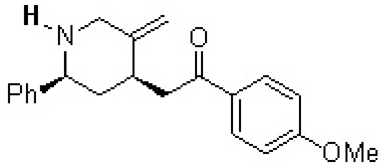

relative configuration only

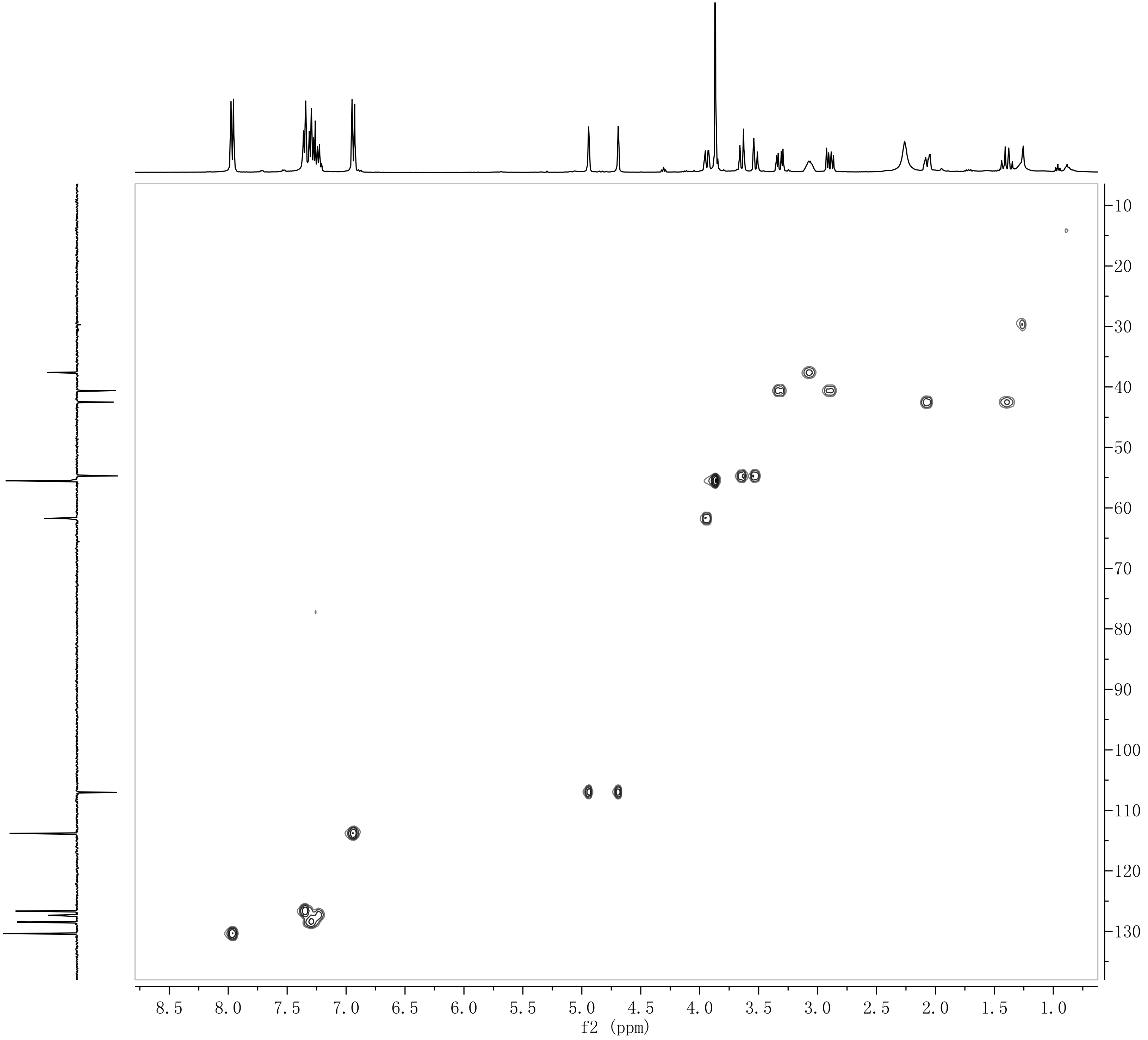

Parameter	Value
Data File Name	E:/ NMR/ 2017/ 2017-5(10-17)/ xfy-0517-2/ 4/ ser
Comment	
Origin	Bruker BioSpin GmbH
Owner	nmr
Site	
Instrument	spect
Solvent	CDCl3
Temperature	295.8
Pulse Sequence	hsqcetgp
Experiment	HSQC
Number of Scans	8
Receiver Gain	196.4
Relaxation Delay	1.4705
Pulse Width	9.6000
Presaturation Frequency	
Acquisition Time	0.1280
Class	
Spectrometer Frequency	(400.13, 100.61)
Spectral Width	(4000.0, 16666.7)
Lowest Frequency	(-314.3, -829.1)
Nucleus	(1H, 13C)
Acquired Size	(512, 256)
Spectral Size	(512, 512)

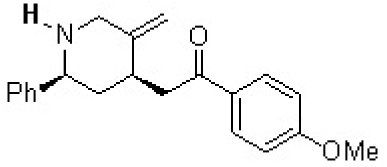

relative configuration only

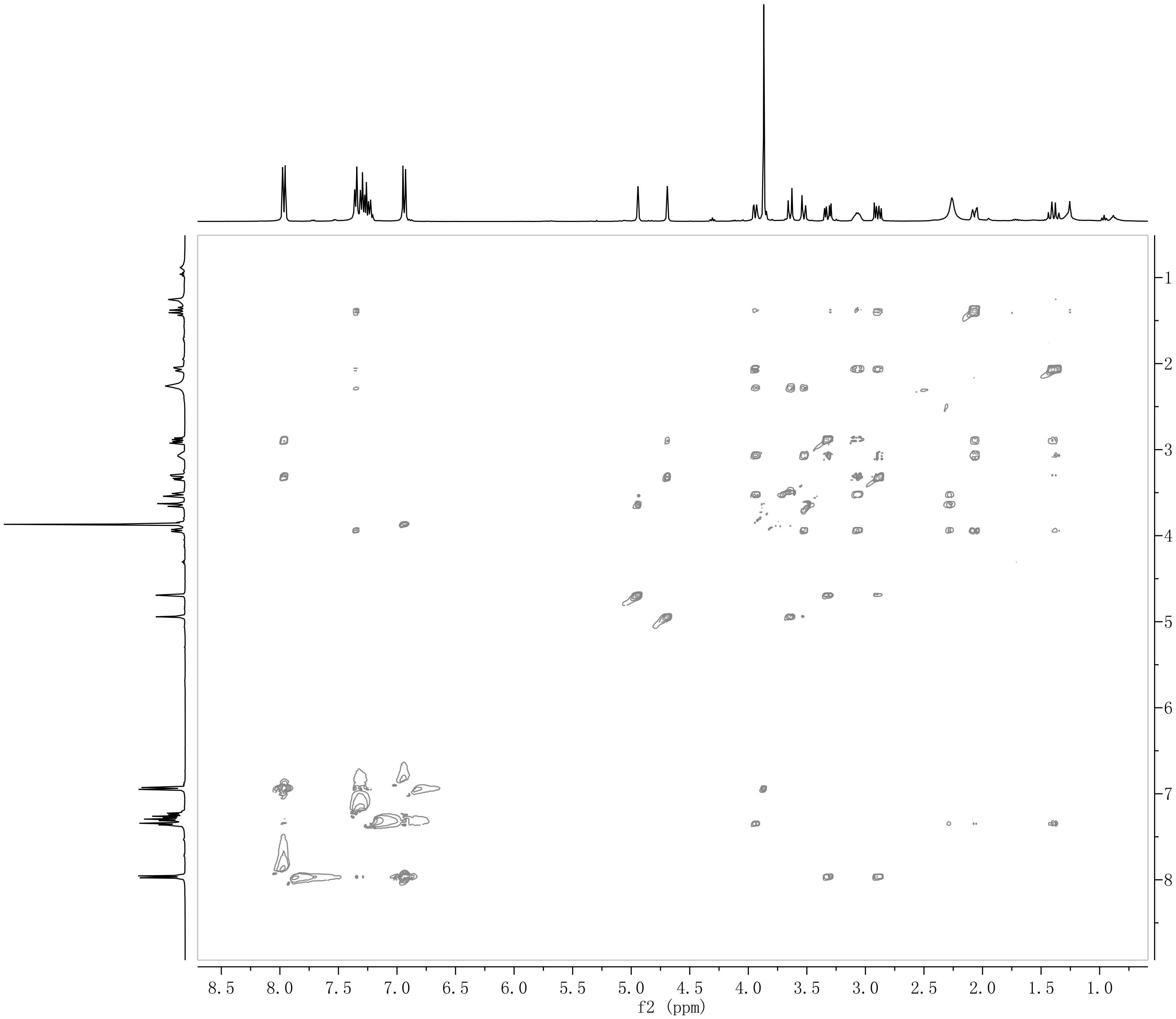

	Parameter	Value
1	Title	
2	Comment	
3	Origin	Bruker BioSpin GmbH
4	Owner	nmr
5	Site	
6	Instrument	spect
7	Author	
8	Solvent	CDCl3
9	Temperature	295.8
10	Pulse Sequence	noesygpphpp
11	Experiment	NOESY
12	Number of Scans	4
13	Receiver Gain	49.4
14	Relaxation Delay	2.0020
15	Pulse Width	9.6000
16	Presaturation Frequency	
17	Acquisition Time	0.2540
18	Class	
19	Spectrometer Frequency	(400.13, 400.13)
20	Spectral Width	(4032.3, 4032.3)
21	Lowest Frequency	(-304.2, -304.2)
22	Nucleus	(1H, 1H)
23	Acquired Size	(1024, 256)
24	Spectral Size	(1024, 1024)

Parameter	Value
Data File Name	E:/ NMR/ 2017/ 2017-6-(1-7)/ xfy-0605-2/ 11/ fid
Comment	
Origin	Bruker BioSpin GmbH
Owner	nmr
Site	
Instrument	spect
Solvent	CDCl3
Temperature	298.7
Pulse Sequence	zg30
Experiment	1D
Number of Scans	8
Receiver Gain	126.1
Relaxation Delay	1.0000
Pulse Width	9.6000
Presaturation Frequency	
Acquisition Time	1.9999
Class	
Spectrometer Frequency	400.13
Spectral Width	8012.8
Lowest Frequency	-1544.2
Nucleus	1H
Acquired Size	16025
Spectral Size	32768

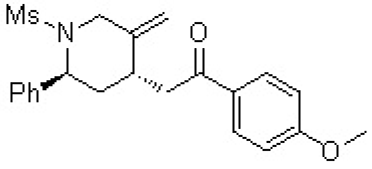

relative configuration only

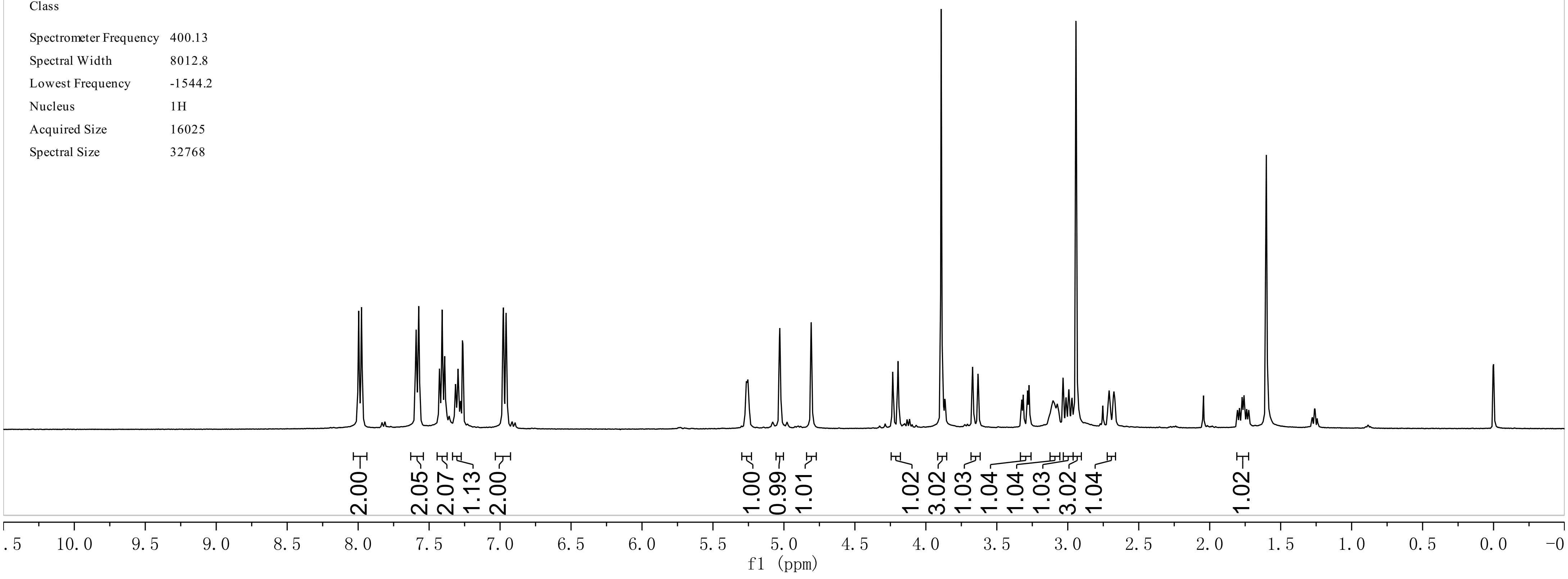

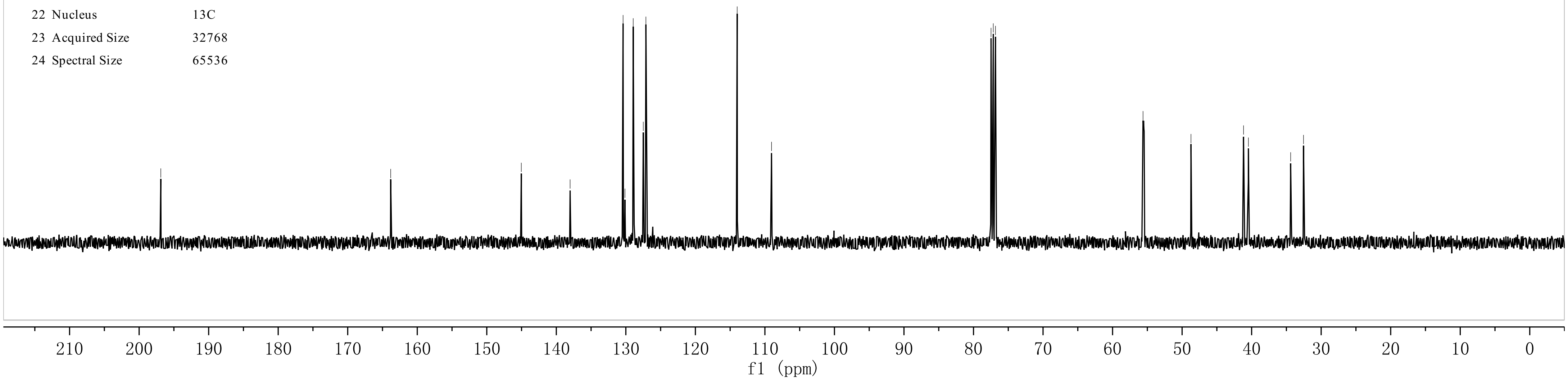

	Parameter	Value
1	Title	
2	Comment	
3	Origin	Bruker BioSpin GmbH
4	Owner	nmr
5	Site	
6	Instrument	spect
7	Author	
8	Solvent	CDCl3
9	Temperature	299.8
10	Pulse Sequence	zgpg30
11	Experiment	1D
12	Number of Scans	39
13	Receiver Gain	196.4
14	Relaxation Delay	2.0000
15	Pulse Width	10.0000
16	Presaturation Frequency	
17	Acquisition Time	1.3631
18	Class	
19	Spectrometer Frequency	100.61
20	Spectral Width	24038.5
21	Lowest Frequency	-1951.0
22	Nucleus	13C
23	Acquired Size	32768
24	Spectral Size	65536

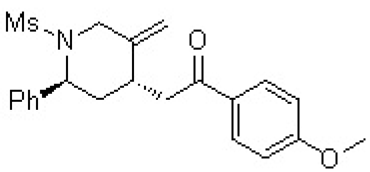

relative configuration only

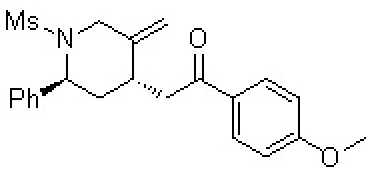

relative configuration only

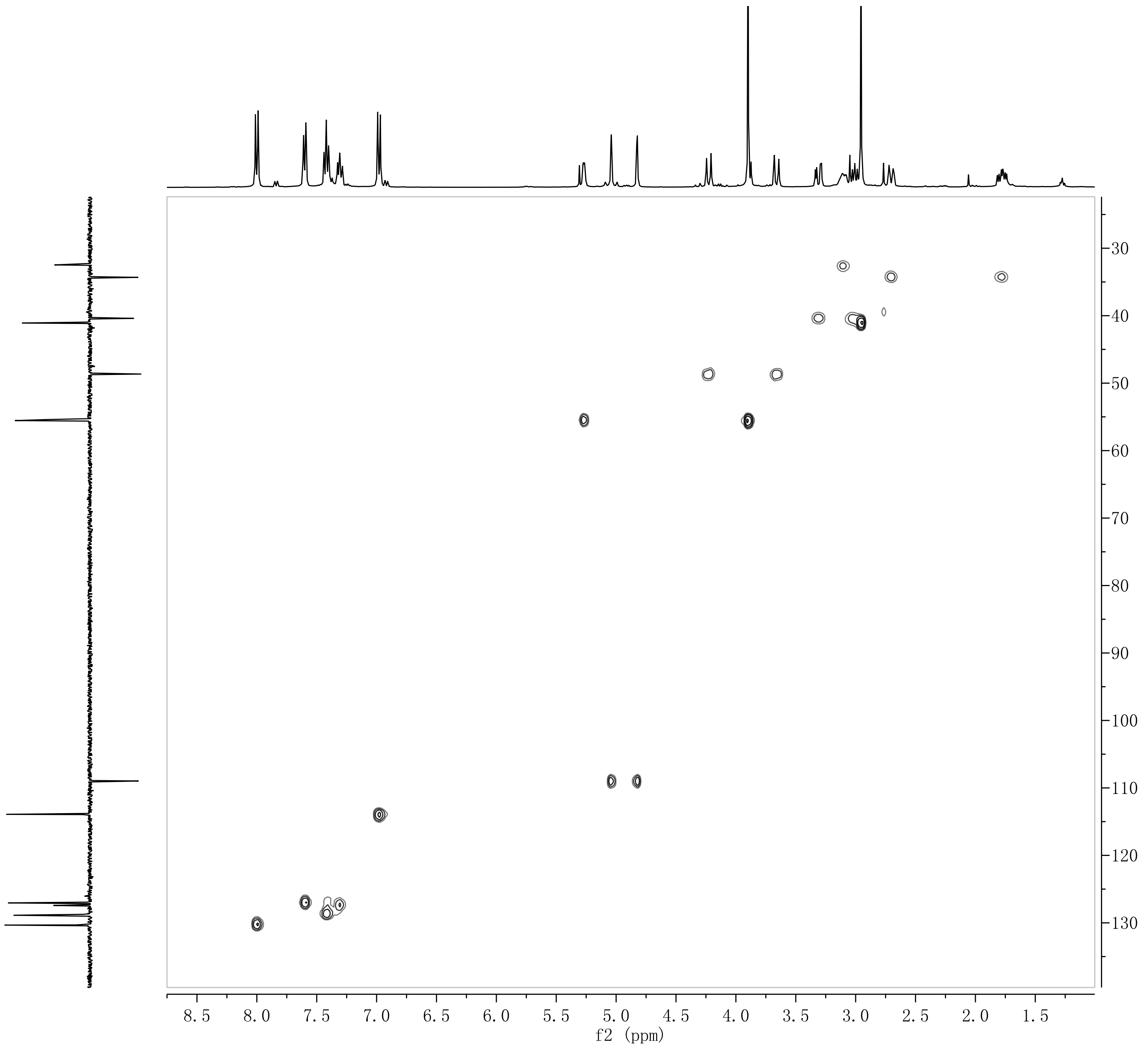

Parameter	Value
Data File Name	E:/ NMR/ 2017/ 2017-6-(1-7)/ xfy-0605-2/ 4/ 4/ ser
Comment	
Origin	Bruker BioSpin GmbH
Owner	nmr
Site	
Instrument	spect
Solvent	CDCl3
Temperature	299.8
Pulse Sequence	hsqcetgp
Experiment	HSQC
Number of Scans	2
Receiver Gain	196.4
Relaxation Delay	1.5000
Pulse Width	9.6000
Presaturation Frequency	
Acquisition Time	0.0985
Class	
Spectrometer Frequency	(400.13, 100.62)
Spectral Width	(5197.5, 16666.7)
Lowest Frequency	(-193.1, -829.1)
Nucleus	(1H, 13C)
Acquired Size	(512, 256)
Spectral Size	(512, 512)

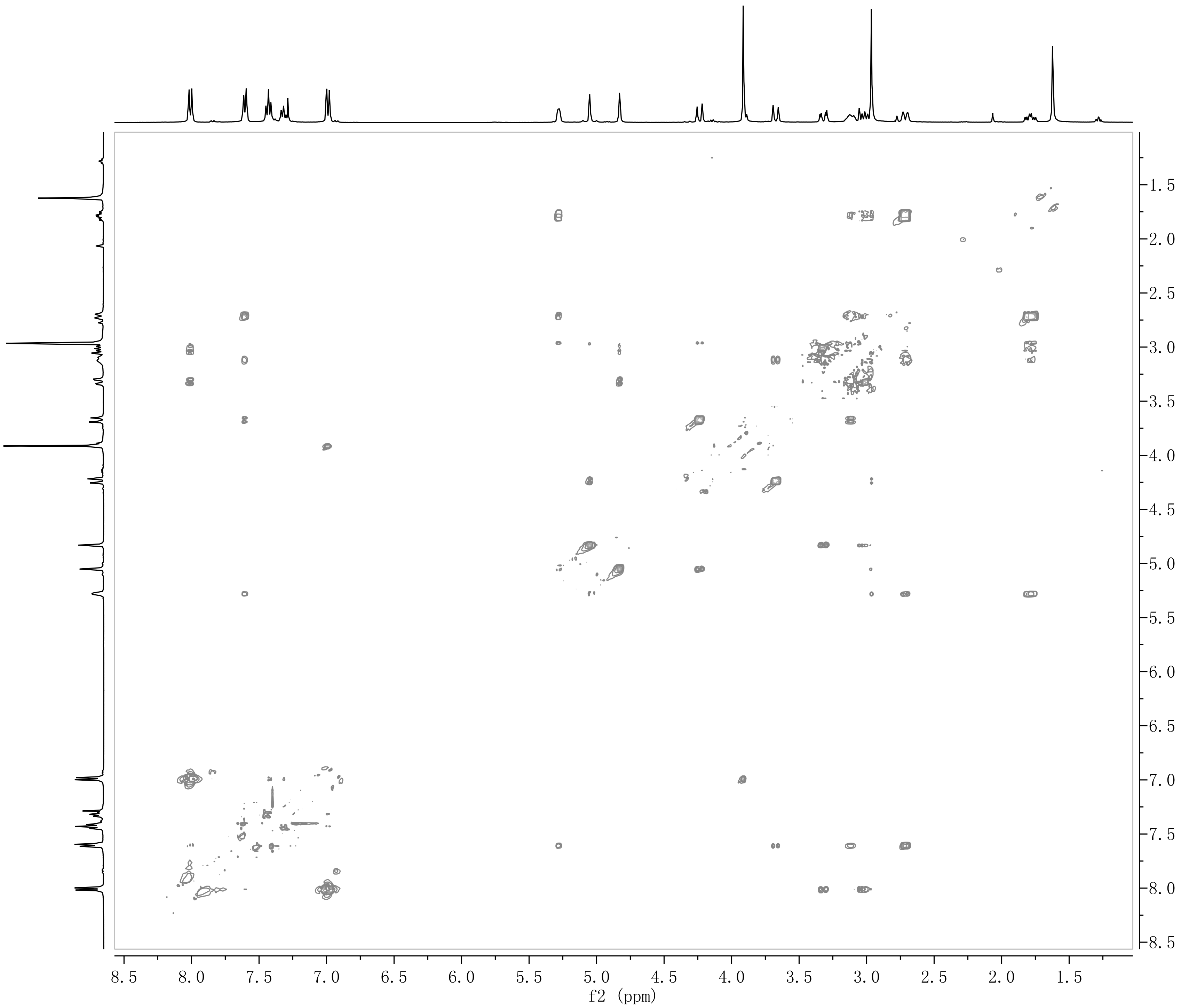

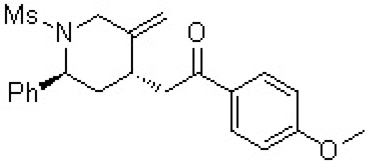

relative configuration only

	Parameter	Value
1	Title	
2	Comment	
3	Origin	Bruker BioSpin GmbH
4	Owner	nmr
5	Site	
6	Instrument	spect
7	Author	
8	Solvent	CDCl3
9	Temperature	298.7
10	Pulse Sequence	noesygpphpp
11	Experiment	NOESY
12	Number of Scans	4
13	Receiver Gain	54.9
14	Relaxation Delay	1.9939
15	Pulse Width	9.6000
16	Presaturation Frequency	
17	Acquisition Time	0.2621
18	Class	
19	Spectrometer Frequency	(400.13, 400.13)
20	Spectral Width	(3906.2, 3906.2)
21	Lowest Frequency	(-273.7, -273.7)
22	Nucleus	(1H, 1H)
23	Acquired Size	(1024, 256)
24	Spectral Size	(1024, 1024)

Parameter	Value
Data File Name	E:/ NMR/ 2017/ 2017-6-（8-14）/ xfy-0610-1/ 1/ pdata/ 1/ 1r
Comment	
Origin	Bruker BioSpin GmbH
Owner	nmr
Site	
Instrument	spect
Solvent	CDCl3
Temperature	298.6
Pulse Sequence	zg30
Experiment	1D
Number of Scans	8
Receiver Gain	111.4
Relaxation Delay	1.0000
Pulse Width	9.6000
Presaturation Frequency	
Acquisition Time	1.9999
Class	
Spectrometer Frequency	400.13
Spectral Width	8012.8
Lowest Frequency	-1544.6
Nucleus	1H
Acquired Size	16025
Spectral Size	65536

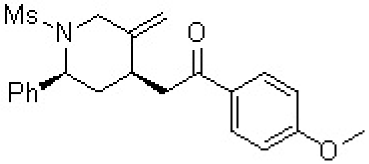

relative configuration only

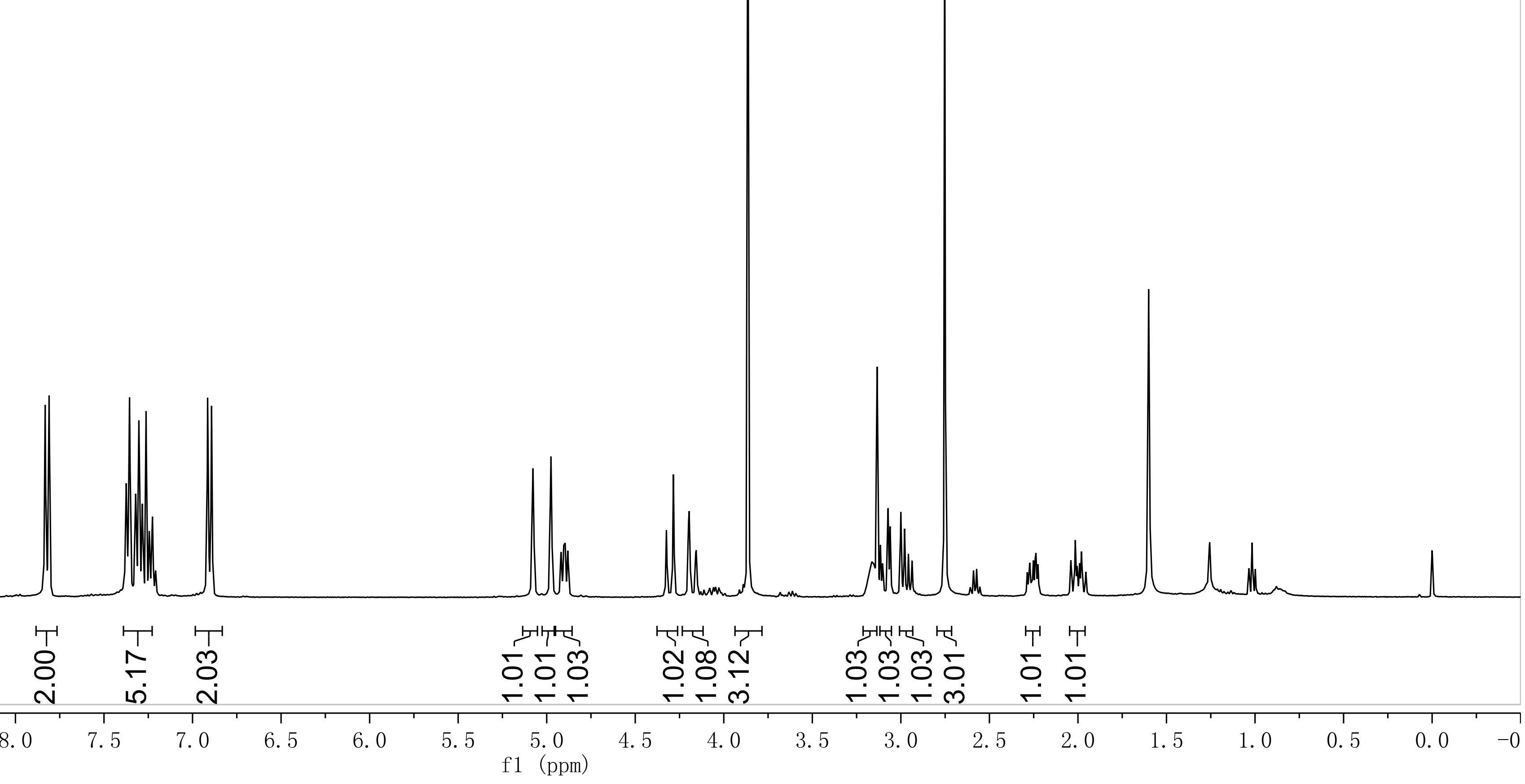

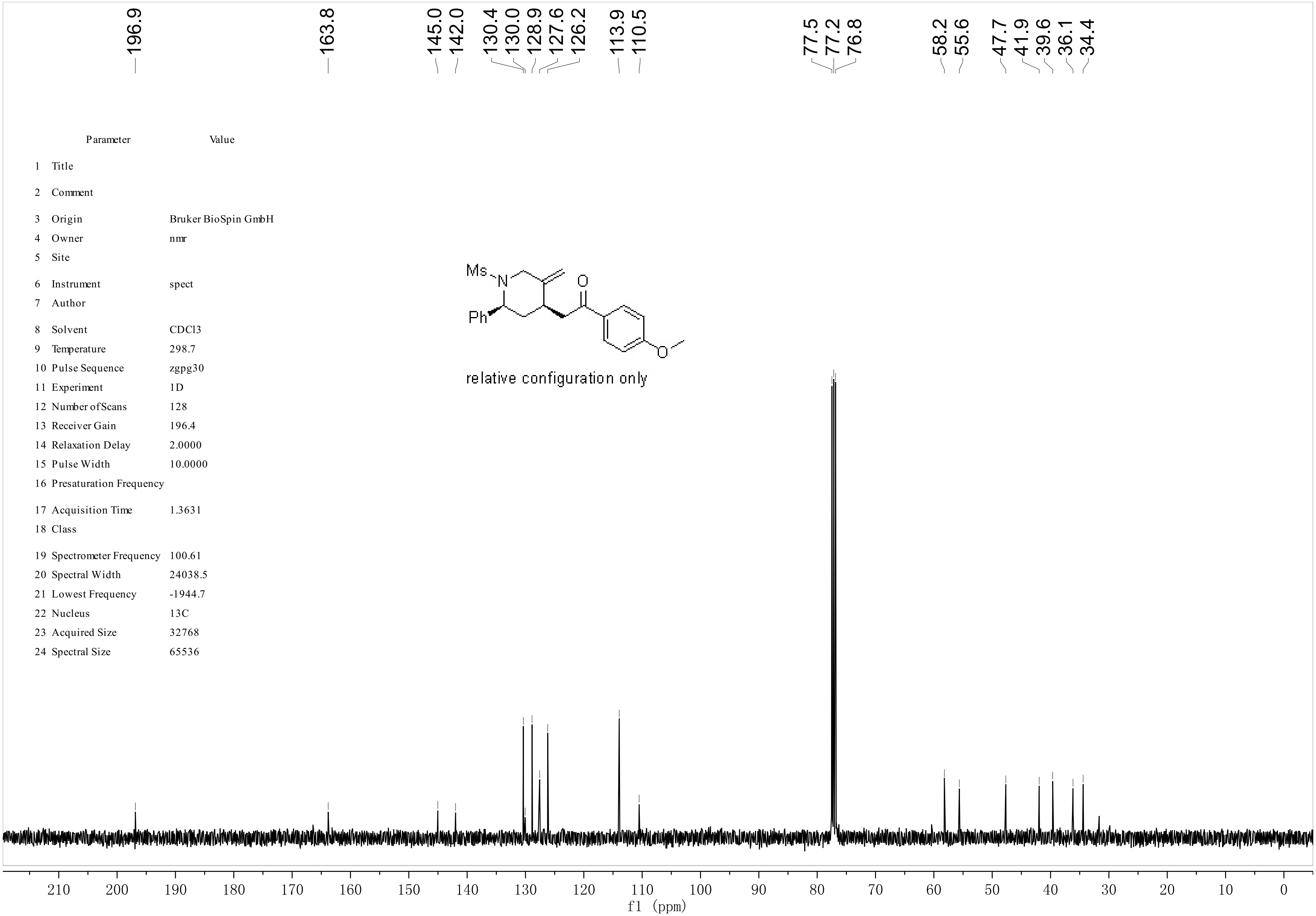

	Parameter	Value
1	Title	
2	Comment	
3	Origin	Bruker BioSpin GmbH
4	Owner	nmr
5	Site	
6	Instrument	spect
7	Author	
8	Solvent	CDCl3
9	Temperature	298.7
10	Pulse Sequence	zgpg30
11	Experiment	1D
12	Number of Scans	128
13	Receiver Gain	196.4
14	Relaxation Delay	2.0000
15	Pulse Width	10.0000
16	Presaturation Frequency	
17	Acquisition Time	1.3631
18	Class	
19	Spectrometer Frequency	100.61
20	Spectral Width	24038.5
21	Lowest Frequency	-1944.7
22	Nucleus	13C
23	Acquired Size	32768
24	Spectral Size	65536

relative configuration only

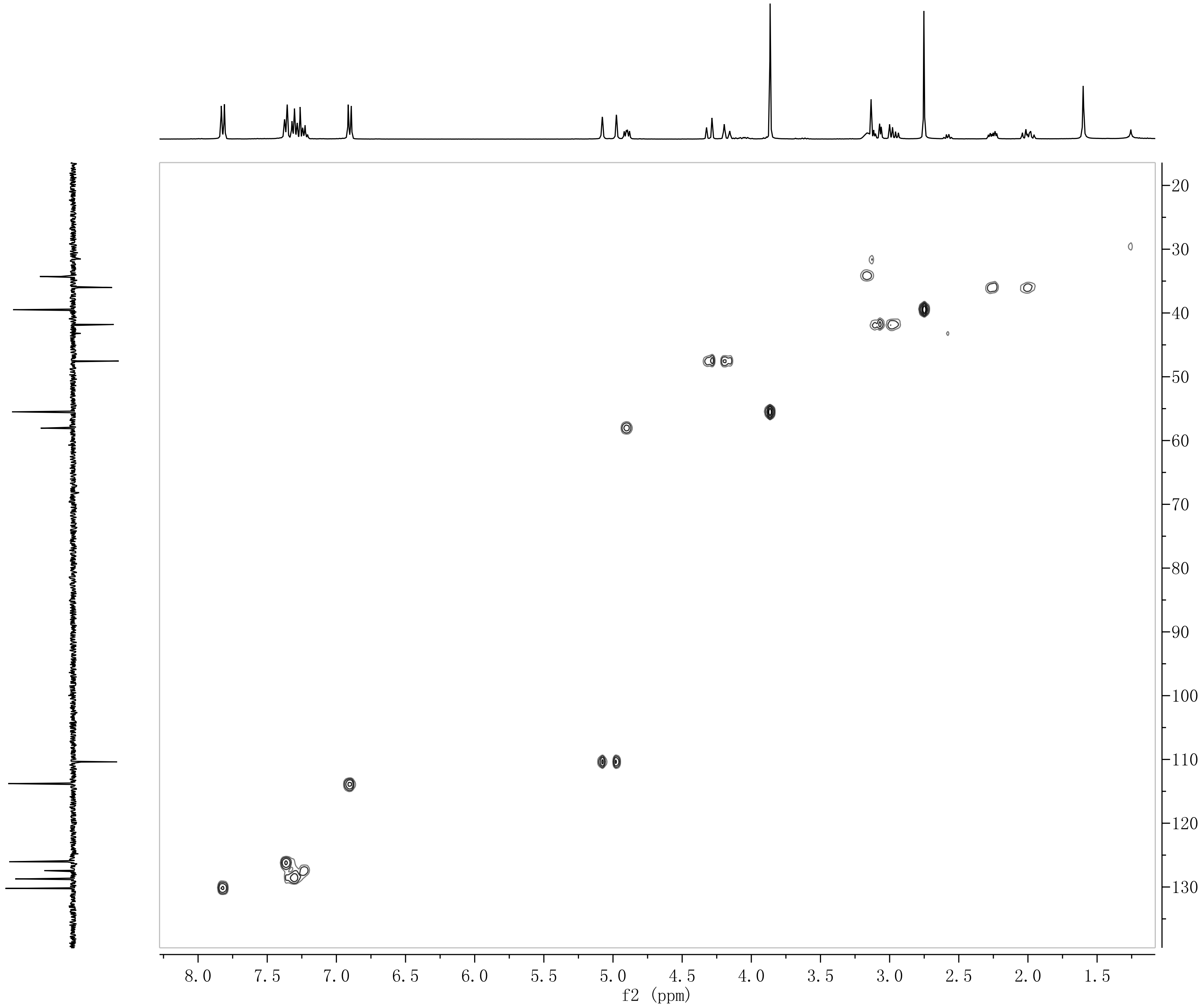

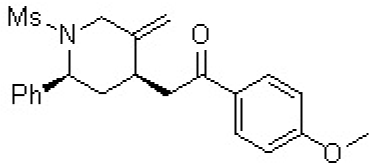

relative configuration only

Parameter	Value
Data File Name	E:/ NMR/ 2017/ 2017-6-（8-14）/ xfy-0610-1/ 4/ ser
Comment	
Origin	Bruker BioSpin GmbH
Owner	nmr
Site	
Instrument	spect
Solvent	CDCl3
Temperature	299.0
Pulse Sequence	hsqcetgp
Experiment	HSQC
Number of Scans	2
Receiver Gain	196.4
Relaxation Delay	1.4664
Pulse Width	9.6000
Presaturation Frequency	
Acquisition Time	0.1321
Class	
Spectrometer Frequency	(400.13, 100.62)
Spectral Width	(3876.0, 16666.7)
Lowest Frequency	(-314.6, -829.1)
Nucleus	(1H, 13C)
Acquired Size	(512, 256)
Spectral Size	(512, 512)

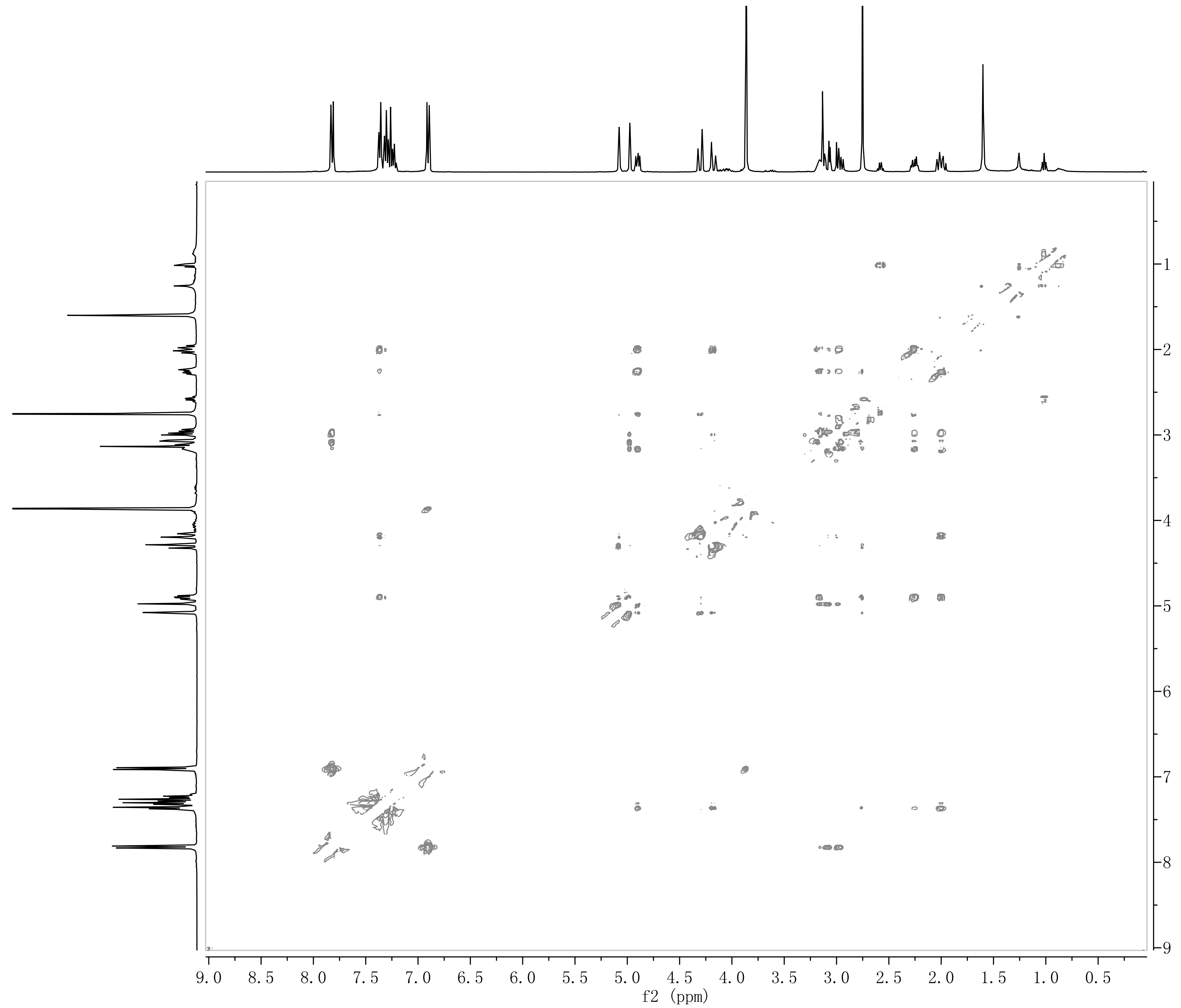

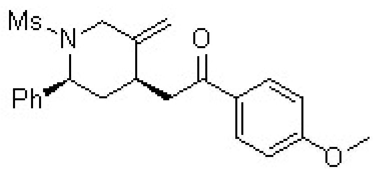

relative configuration only

	Parameter	Value
1	Title	
2	Comment	
3	Origin	Bruker BioSpin GmbH
4	Owner	nmr
5	Site	
6	Instrument	spect
7	Author	
8	Solvent	CDCl3
9	Temperature	296.2
10	Pulse Sequence	noesygpphpp
11	Experiment	NOESY
12	Number of Scans	4
13	Receiver Gain	48.5
14	Relaxation Delay	1.9775
15	Pulse Width	11.2900
16	Presaturation Frequency	
17	Acquisition Time	0.2273
18	Class	
19	Spectrometer Frequency	(500.13, 500.13)
20	Spectral Width	(4504.5, 4504.5)
21	Lowest Frequency	(11.8, 11.8)
22	Nucleus	(1H, 1H)
23	Acquired Size	(1024, 256)
24	Spectral Size	(1024, 1024)

Parameter	Value
Title	xfy-181022-1-s.1.fid
Comment	
Origin	Bruker BioSpin GmbH
Owner	nmr
Site	
Instrument	spect
Solvent	CDCl3
Temperature	296.1
Pulse Sequence	zg30
Experiment	1D
Number of Scans	10
Receiver Gain	31.1
Relaxation Delay	1.0000
Pulse Width	10.7100
Presaturation Frequency	
Acquisition Time	3.2768
Class	
Spectrometer Frequency	500.13
Spectral Width	10000.0
Lowest Frequency	-1924.1
Nucleus	1H
Acquired Size	32768
Spectral Size	65536

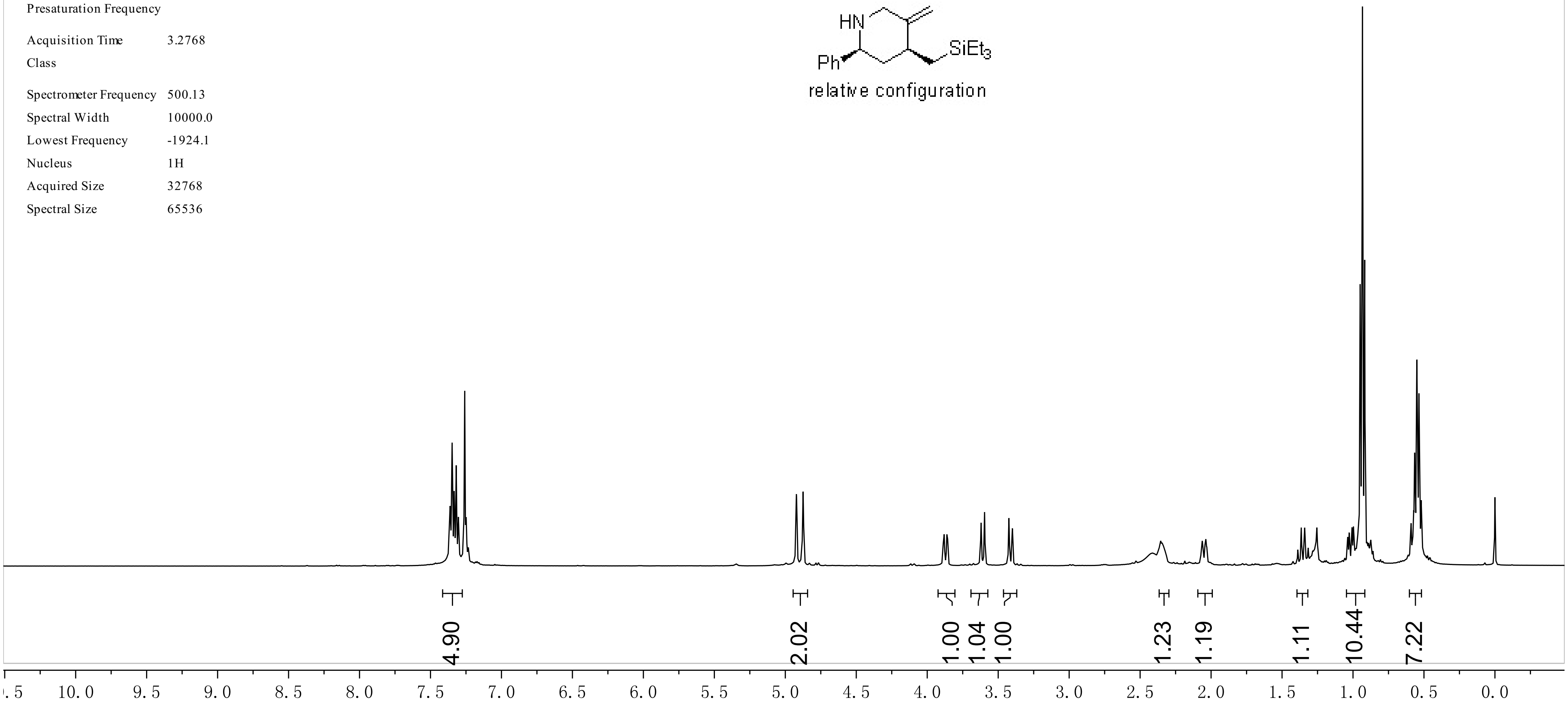

Parameter	Value
Title	xfy-181022-1-s.2.fid
Comment	
Origin	Bruker BioSpin GmbH
Owner	nmr
Site	
Instrument	spect
Solvent	CDCl3
Temperature	296.2
Pulse Sequence	zgpg30
Experiment	1D
Number of Scans	26
Receiver Gain	193.1
Relaxation Delay	2.0000
Pulse Width	9.6000
Presaturation Frequency	
Acquisition Time	1.1010
Class	
Spectrometer Frequency	125.77
Spectral Width	29761.9
Lowest Frequency	-2305.8
Nucleus	13C
Acquired Size	32768
Spectral Size	65536

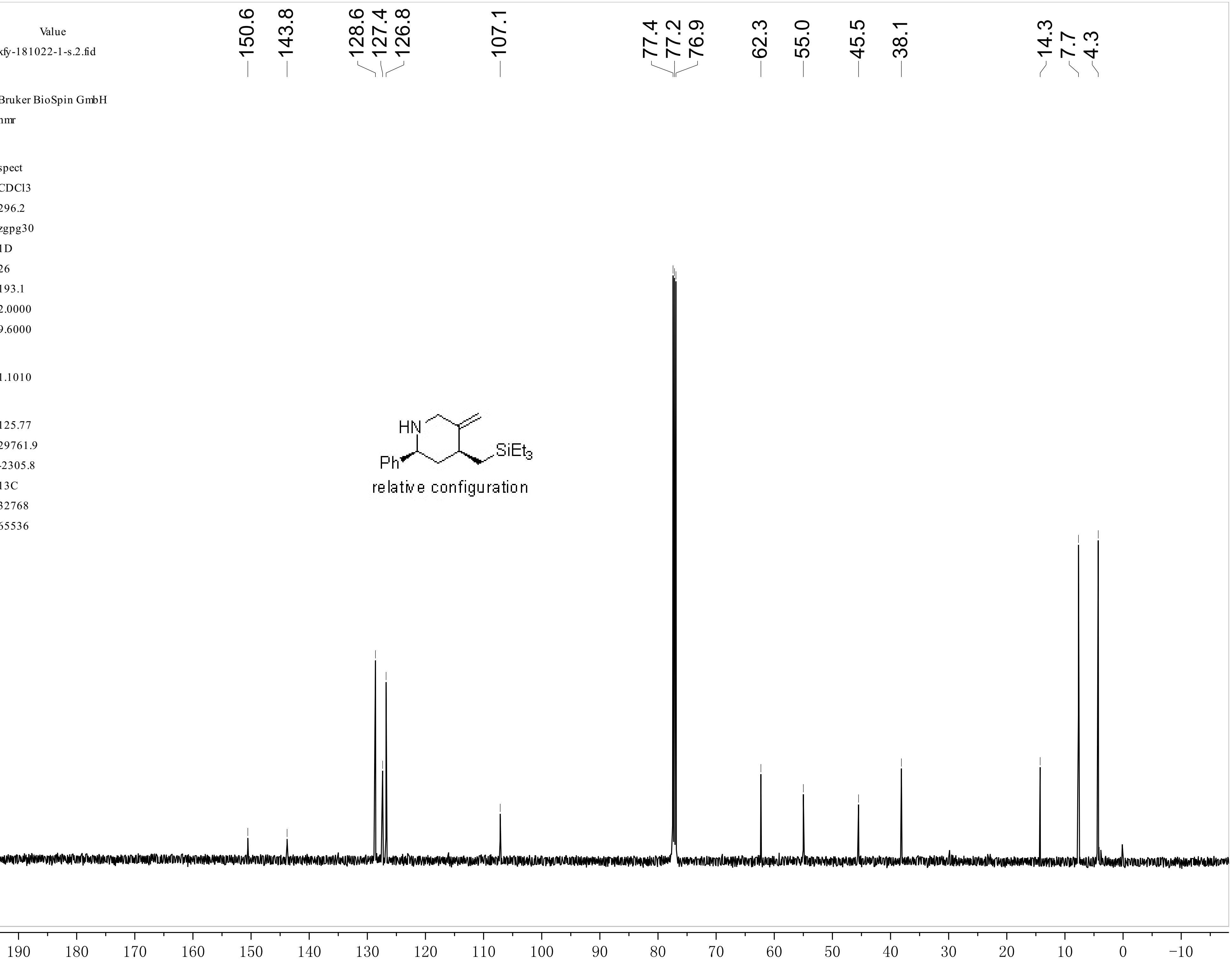

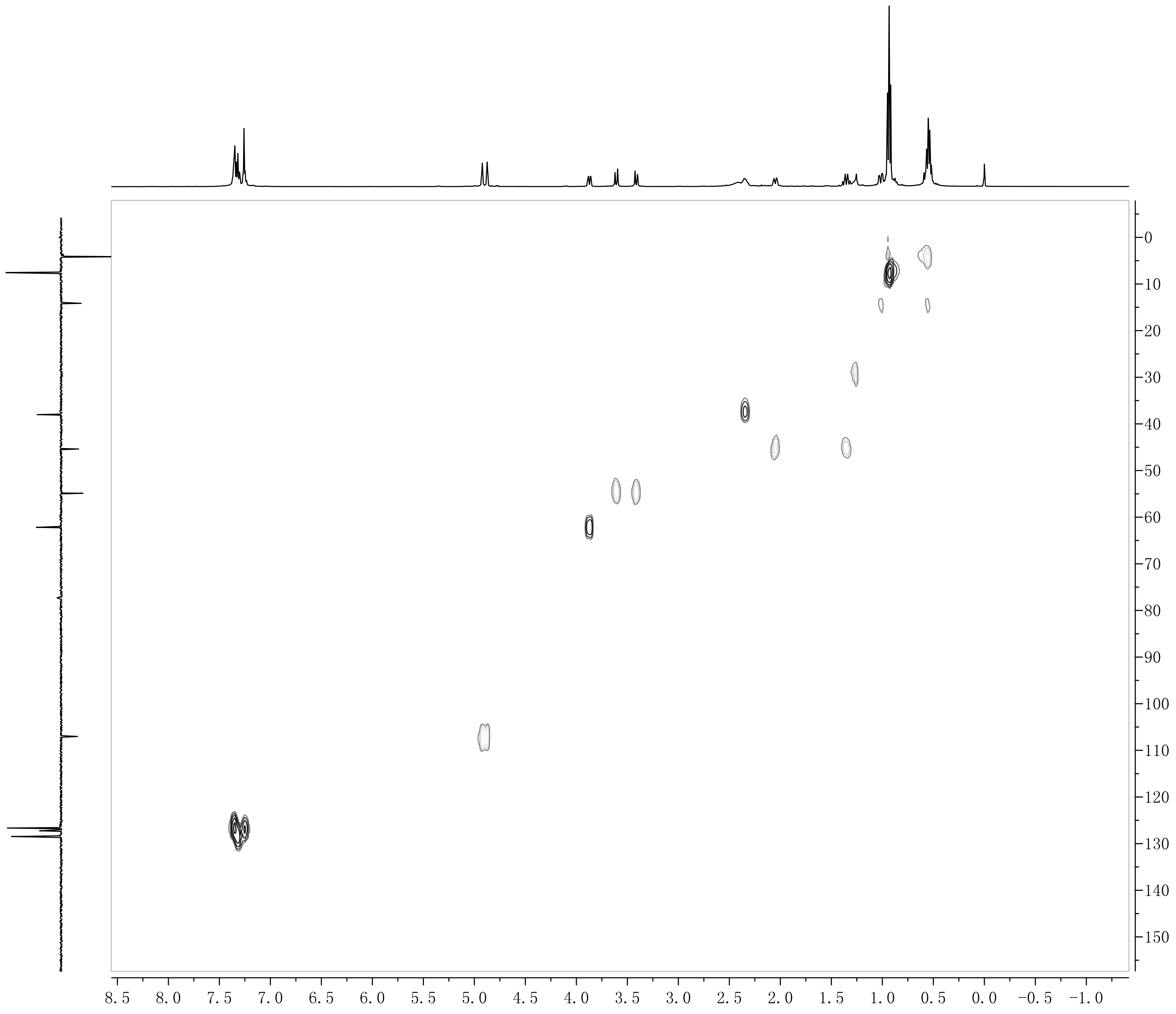

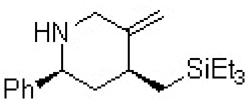

relative configuration

Parameter	Value
Title	xfy-181022-1-s.4.ser
Comment	
Origin	Bruker BioSpin GmbH
Owner	nmr
Site	
Instrument	spect
Solvent	CDCl3
Temperature	296.2
Pulse Sequence	hsqcedetgp
Experiment	HSQC-EDITED
Number of Scans	2
Receiver Gain	193.1
Relaxation Delay	1.4764
Pulse Width	10.7100
Presaturation Frequency	
Acquisition Time	0.1024
Class	
Spectrometer Frequency	(500.13, 125.77)
Spectral Width	(5000.0, 20833.3)
Lowest Frequency	(-718.8, -1037.0)
Nucleus	(1H, 13C)
Acquired Size	(512, 99)
Spectral Size	(512, 512)

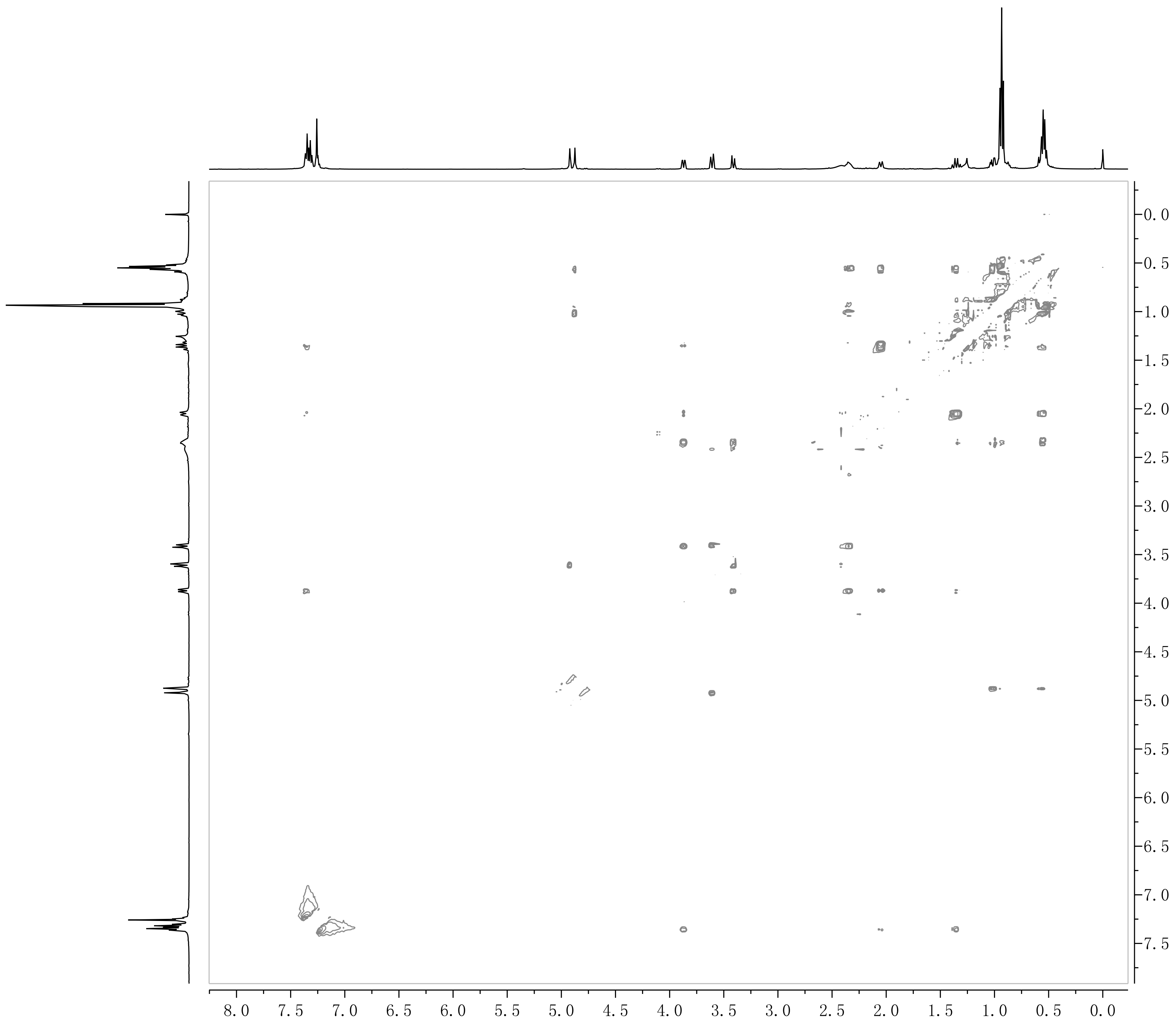

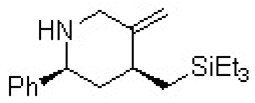

relative configuration

Parameter	Value
Title	xfy-181022-1-s.5.ser
Comment	
Origin	Bruker BioSpin GmbH
Owner	nmr
Site	
Instrument	spect
Solvent	CDCl3
Temperature	296.2
Pulse Sequence	noesygpphpp
Experiment	NOESY
Number of Scans	4
Receiver Gain	21.9
Relaxation Delay	2.0020
Pulse Width	10.7100
Presaturation Frequency	
Acquisition Time	0.2028
Class	
Spectrometer Frequency	(500.13, 500.13)
Spectral Width	(5050.5, 5050.5)
Lowest Frequency	(-749.6, -749.6)
Nucleus	(1H, 1H)
Acquired Size	(1024, 196)
Spectral Size	(1024, 1024)

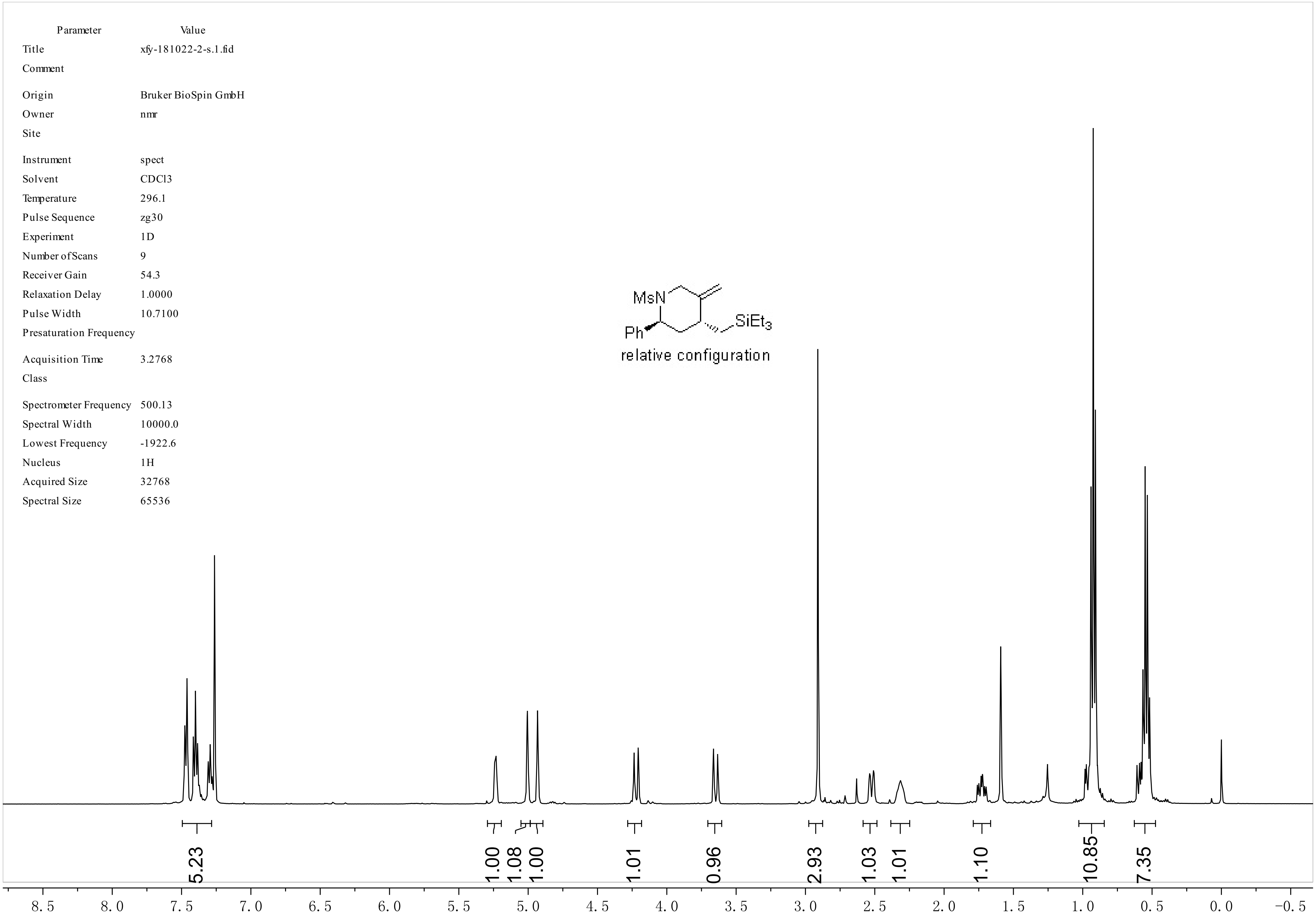
Parameter Value
Title xfy-181022-2-s.1.fid
Comment
Origin Bruker BioSpin GmbH
Owner nmr
Site
Instrument spect
Solvent CDCl3
Temperature 296.1
Pulse Sequence zg30
Experiment 1D
Number of Scans 9
Receiver Gain 54.3
Relaxation Delay 1.0000
Pulse Width 10.7100
Presaturation Frequency
Acquisition Time 3.2768
Class
Spectrometer Frequency 500.13
Spectral Width 10000.0
Lowest Frequency -1922.6
Nucleus 1H
Acquired Size 32768
Spectral Size 65536
MsN
Ph
SiEt3
relative configuration
5.23
1.00
1.08
1.00
1.01
0.96
2.93
1.03
1.01
1.10
10.85
7.35
8.5 8.0 7.5 7.0 6.5 6.0 5.5 5.0 4.5 4.0 3.5 3.0 2.5 2.0 1.5 1.0 0.5 0.0 -0.5

Parameter	Value
Title	xfy-181022-2-s.2.fid
Comment	
Origin	Bruker BioSpin GmbH
Owner	nmr
Site	
Instrument	spect
Solvent	CDCl3
Temperature	296.1
Pulse Sequence	zgpg30
Experiment	1D
Number of Scans	31
Receiver Gain	193.1
Relaxation Delay	2.0000
Pulse Width	9.6000
Presaturation Frequency	
Acquisition Time	1.1010
Class	
Spectrometer Frequency	125.77
Spectral Width	29761.9
Lowest Frequency	-2290.3
Nucleus	13C
Acquired Size	32768
Spectral Size	65536

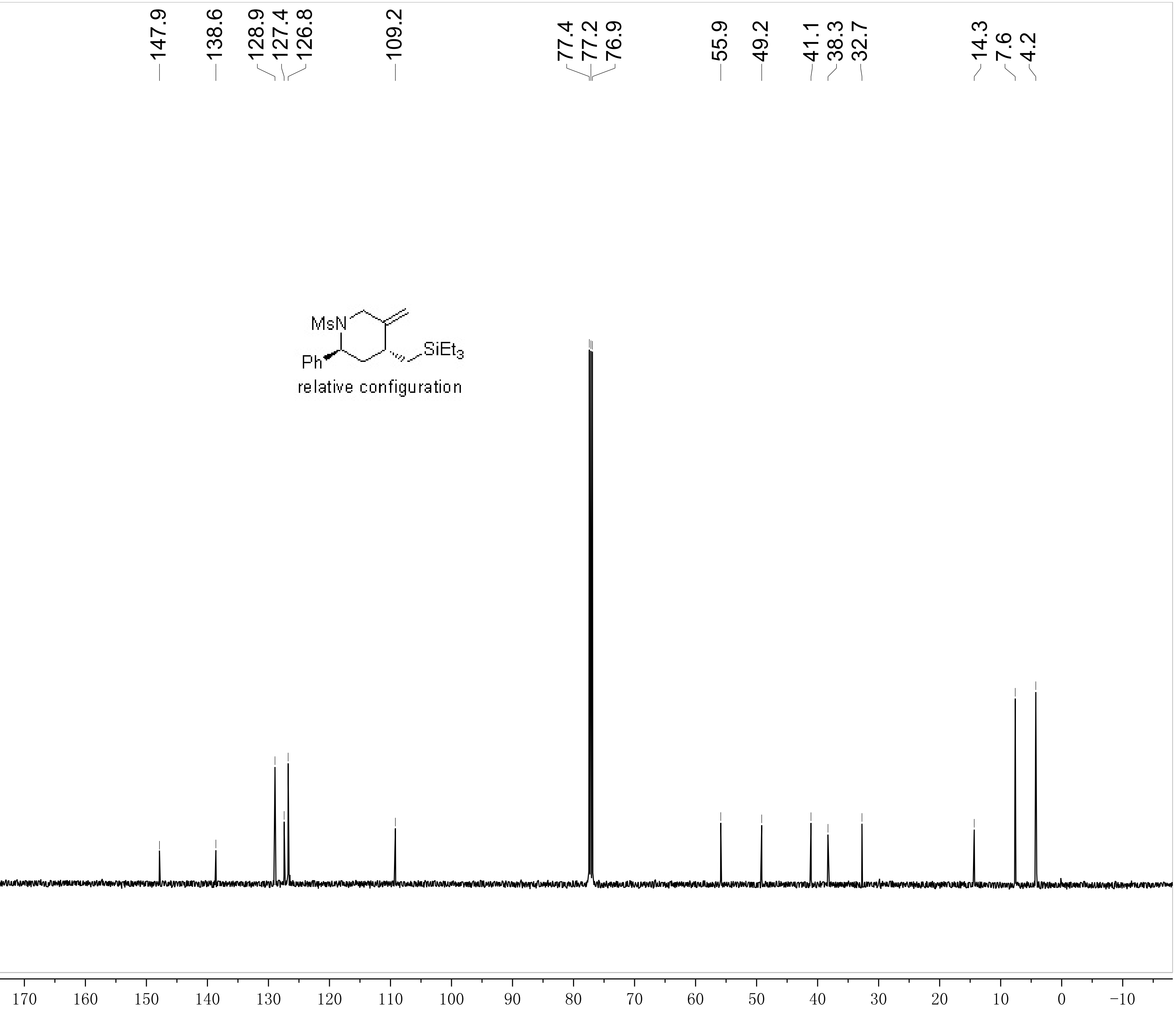

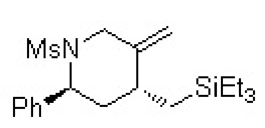

relative configuration

Parameter	Value
Title	xfy-181022-2-s.4.ser
Comment	
Origin	Bruker BioSpin GmbH
Owner	nmr
Site	
Instrument	spect
Solvent	CDCl3
Temperature	296.1
Pulse Sequence	hsqcedetgp
Experiment	HSQC-EDITED
Number of Scans	2
Receiver Gain	193.1
Relaxation Delay	1.4662
Pulse Width	10.7100
Presaturation Frequency	
Acquisition Time	0.1126
Class	
Spectrometer Frequency	(500.13, 125.77)
Spectral Width	(4545.5, 20833.3)
Lowest Frequency	(-398.3, -1037.0)
Nucleus	(1H, 13C)
Acquired Size	(512, 94)
Spectral Size	(512, 512)

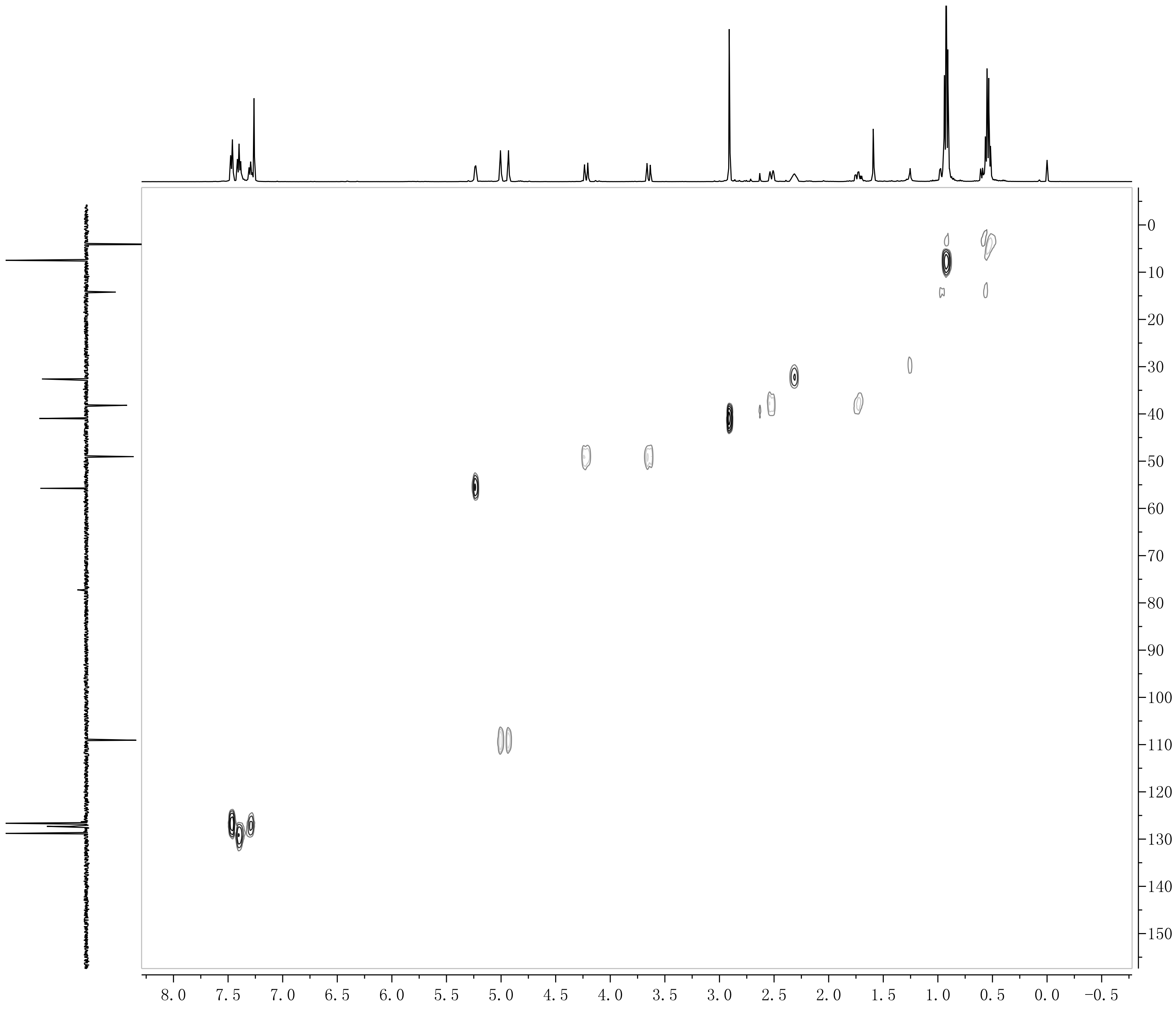

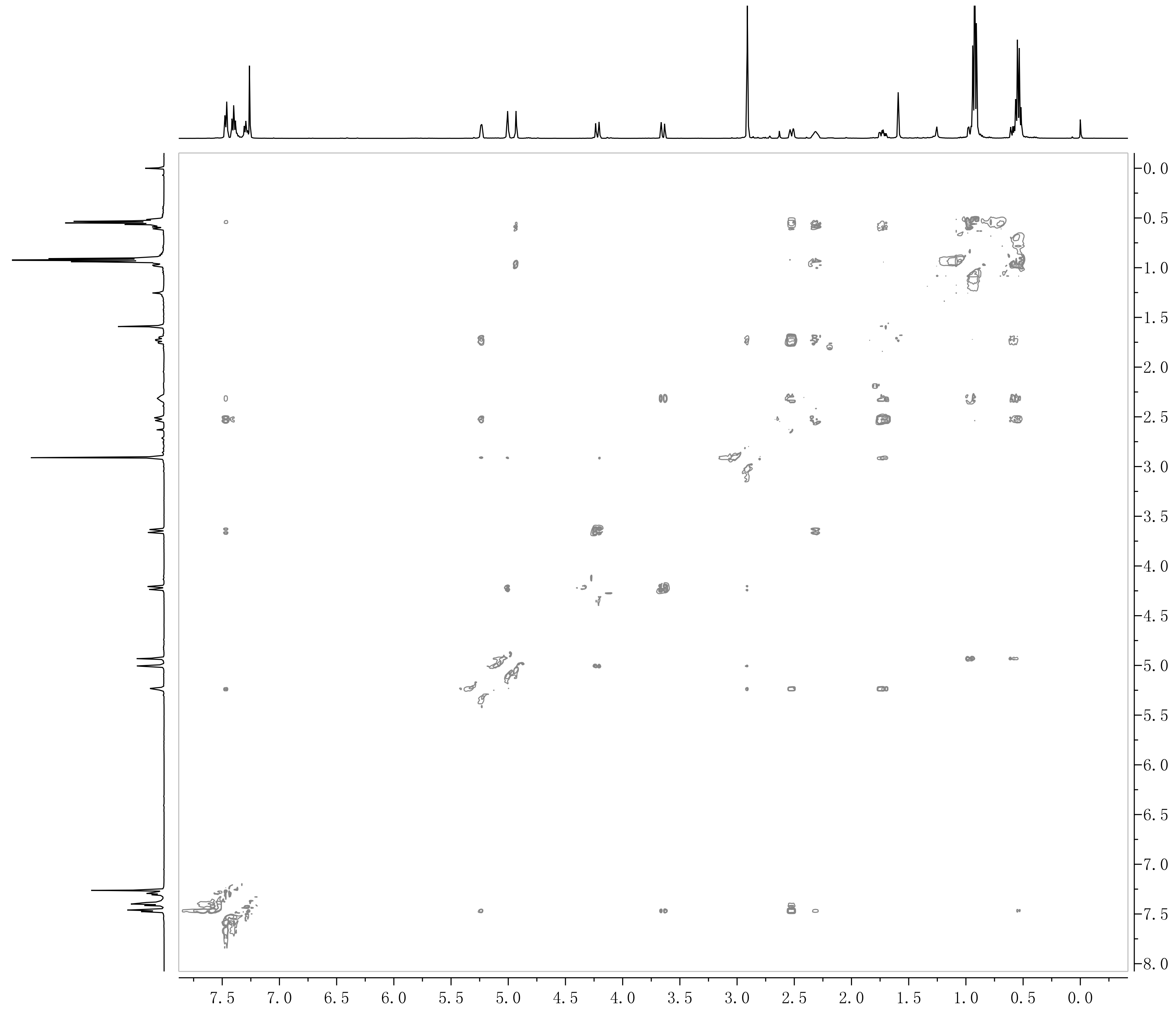

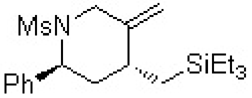

relative configuration

Parameter	Value
Title	xfy-181022-2-s.9.ser
Comment	
Origin	Bruker BioSpin GmbH
Owner	nmr
Site	
Instrument	spect
Solvent	CDCl3
Temperature	293.4
Pulse Sequence	noesygpphpp
Experiment	NOESY
Number of Scans	4
Receiver Gain	49.4
Relaxation Delay	2.0082
Pulse Width	10.0000
Presaturation Frequency	
Acquisition Time	0.2478
Class	
Spectrometer Frequency	(400.13, 400.13)
Spectral Width	(4132.2, 4132.2)
Lowest Frequency	(-655.5, -655.5)
Nucleus	(1H, 1H)
Acquired Size	(1024, 229)
Spectral Size	(1024, 1024)

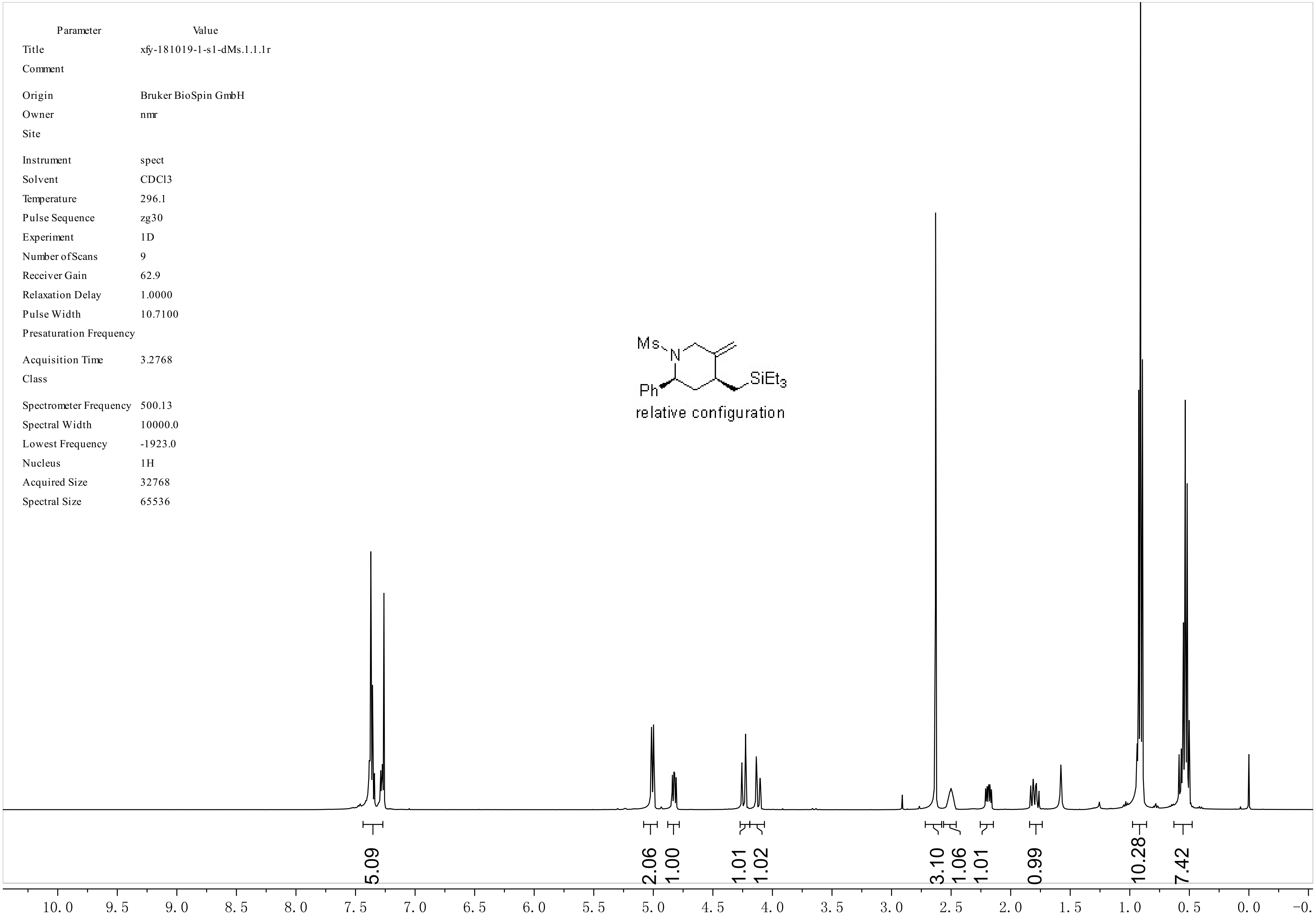
Parameter Value
Title xfy-181019-1-s1-dMs.1.1.1r
Comment
Origin Bruker BioSpin GmbH
Owner nmr
Site
Instrument spect
Solvent CDCl3
Temperature 296.1
Pulse Sequence zg30
Experiment 1D
Number of Scans 9
Receiver Gain 62.9
Relaxation Delay 1.0000
Pulse Width 10.7100
Presaturation Frequency
Acquisition Time 3.2768
Class
Spectrometer Frequency 500.13
Spectral Width 10000.0
Lowest Frequency -1923.0
Nucleus 1H
Acquired Size 32768
Spectral Size 65536
Ms
N
Ph
SiEt3
relative configuration
5.09
2.06
1.00
1.01
1.02
3.10
1.06
1.01
0.99
10.28
7.42
10.0 9.5 9.0 8.5 8.0 7.5 7.0 6.5 6.0 5.5 5.0 4.5 4.0 3.5 3.0 2.5 2.0 1.5 1.0 0.5 0.0 -0.

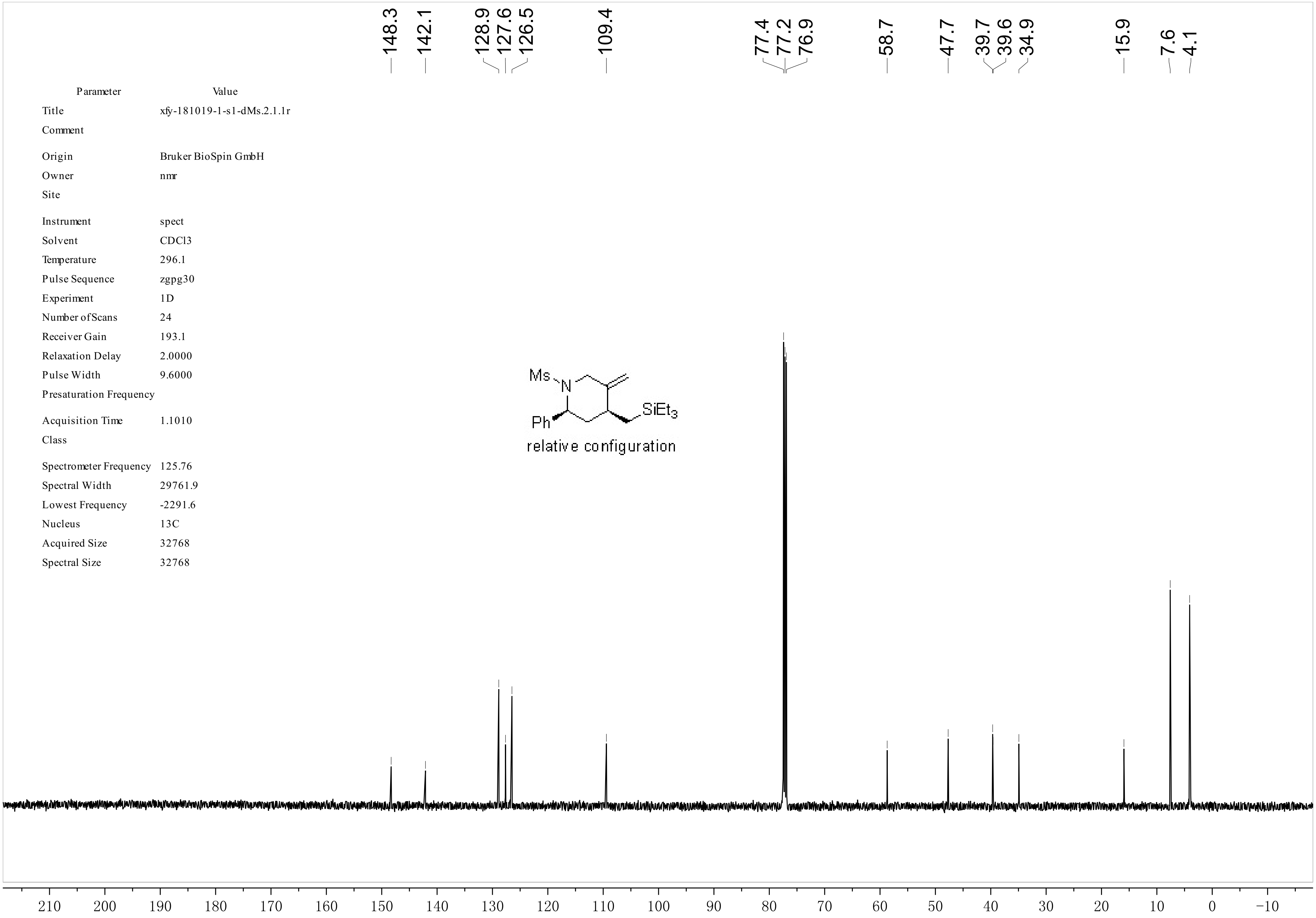

Parameter	Value
Title	xfy-181019-1-s1-dMs.2.1.1r
Comment	
Origin	Bruker BioSpin GmbH
Owner	nmr
Site	
Instrument	spect
Solvent	CDCl3
Temperature	296.1
Pulse Sequence	zgpg30
Experiment	1D
Number of Scans	24
Receiver Gain	193.1
Relaxation Delay	2.0000
Pulse Width	9.6000
Presaturation Frequency	
Acquisition Time	1.1010
Class	
Spectrometer Frequency	125.76
Spectral Width	29761.9
Lowest Frequency	-2291.6
Nucleus	13C
Acquired Size	32768
Spectral Size	32768

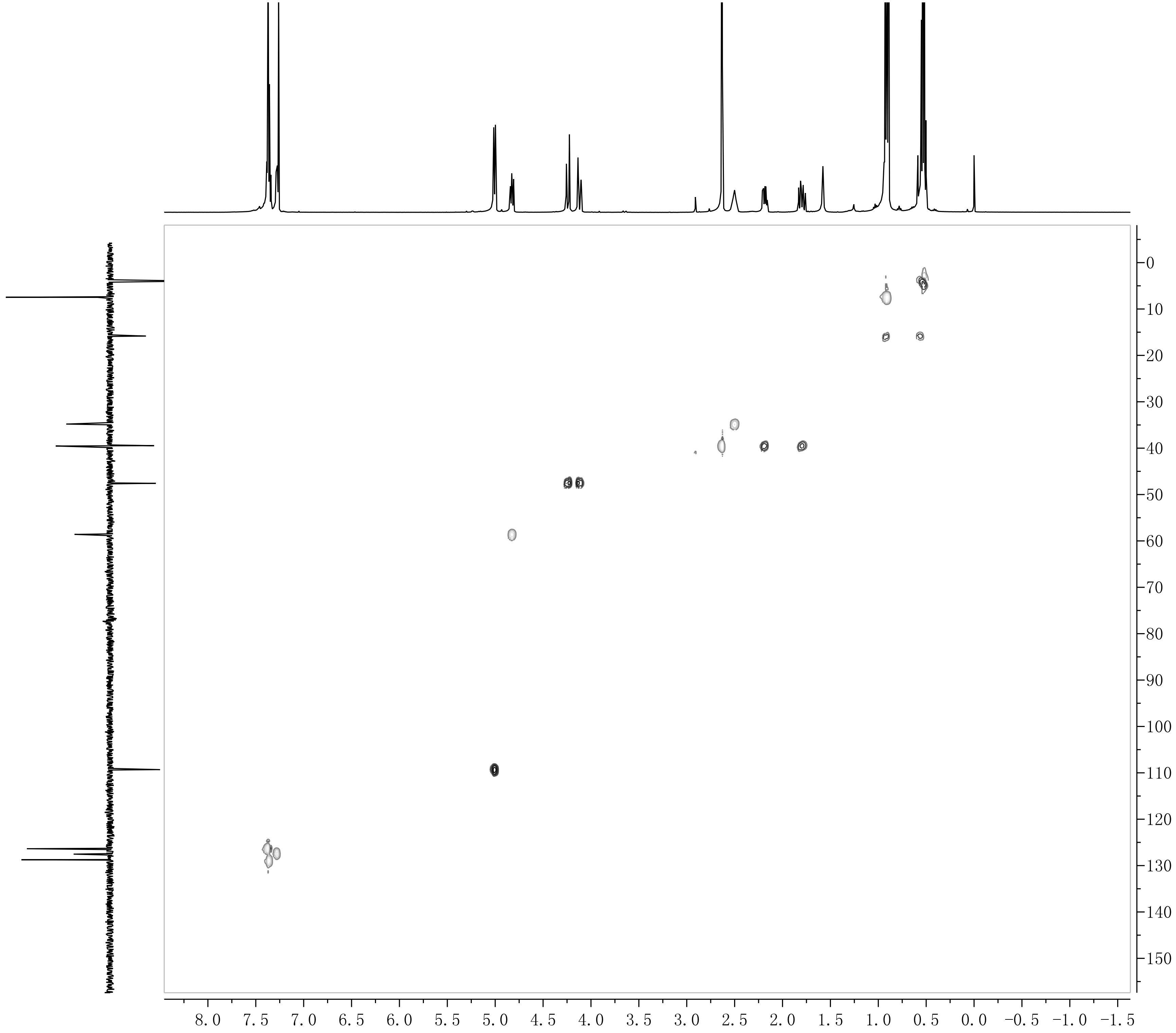

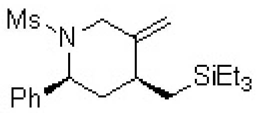

relative configuration

Parameter	Value
Title	4.4.1.2rr
Comment	
Origin	Bruker BioSpin GmbH
Owner	nmr
Site	
Instrument	spect
Solvent	CDCl3
Temperature	296.2
Pulse Sequence	hsqcedetgp
Experiment	HSQC-EDITED
Number of Scans	2
Receiver Gain	193.1
Relaxation Delay	1.4775
Pulse Width	10.7100
Presaturation Frequency	
Acquisition Time	0.1014
Class	
Spectrometer Frequency	(500.13, 125.76)
Spectral Width	(5050.5, 20833.3)
Lowest Frequency	(-821.2, -1037.0)
Nucleus	(1H, 13C)
Acquired Size	(512, 119)
Spectral Size	(1024, 1024)

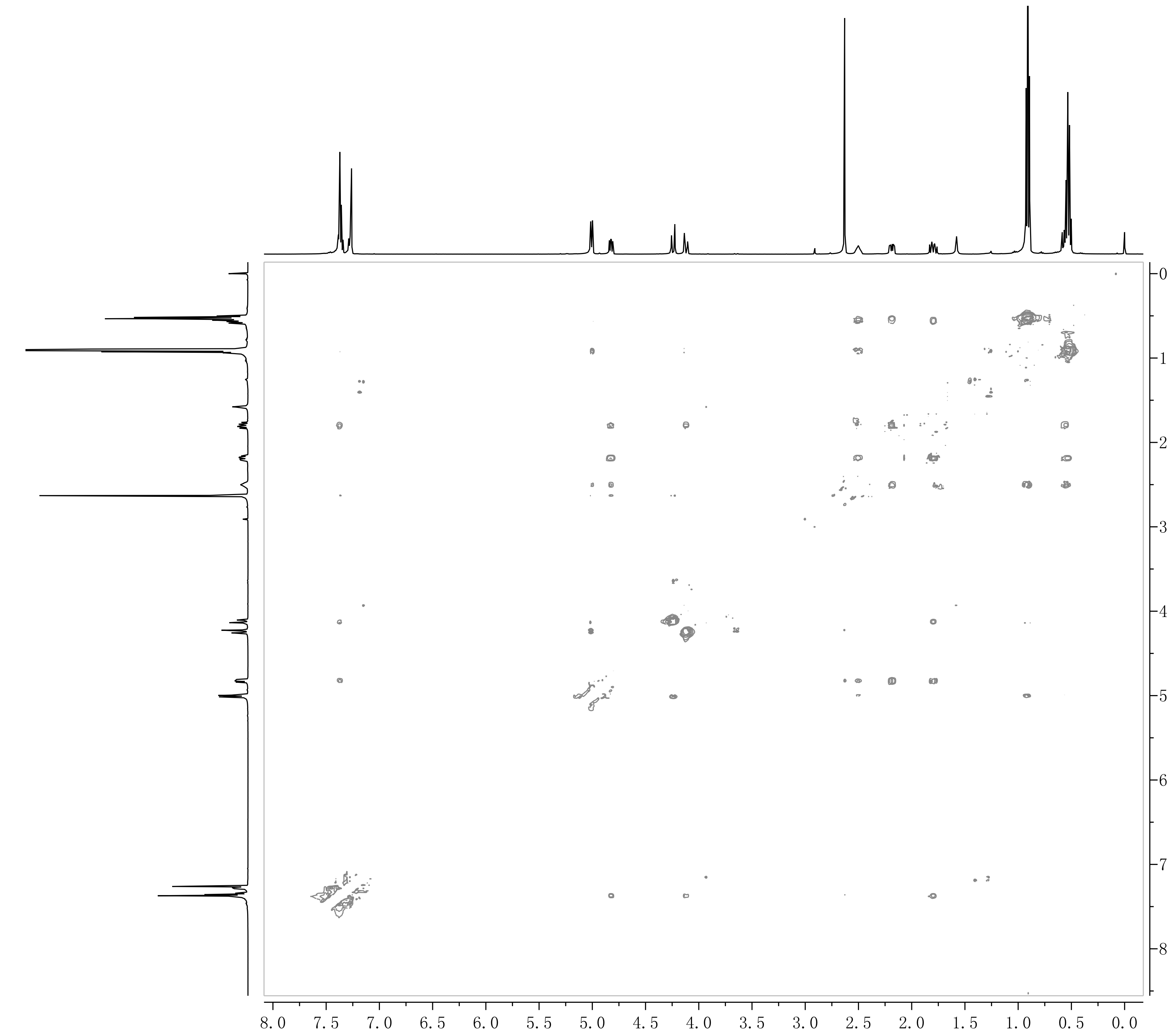

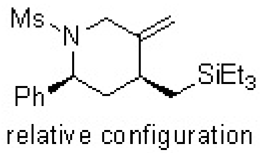

Parameter	Value
Title	xfy-181019-1-s1-dMs.5.ser
Comment	
Origin	Bruker BioSpin GmbH
Owner	nmr
Site	
Instrument	spect
Solvent	CDCl3
Temperature	296.1
Pulse Sequence	noesygpphpp
Experiment	NOESY
Number of Scans	4
Receiver Gain	54.3
Relaxation Delay	1.9939
Pulse Width	10.7100
Presaturation Frequency	
Acquisition Time	0.2109
Class	
Spectrometer Frequency	(500.13, 500.13)
Spectral Width	(4854.4, 4854.4)
Lowest Frequency	(-509.2, -509.2)
Nucleus	(1H, 1H)
Acquired Size	(1024, 256)
Spectral Size	(1024, 1024)

Parameter	Value
Title	xfy-181227-8-s2.21.1.1r
Comment	
Origin	Bruker BioSpin GmbH
Owner	nmr
Site	
Instrument	spect
Solvent	CDCl3
Temperature	293.6
Pulse Sequence	zg30
Experiment	1D
Number of Scans	4
Receiver Gain	87.6
Relaxation Delay	1.0000
Pulse Width	10.0000
Presaturation Frequency	
Acquisition Time	1.9999
Class	
Spectrometer Frequency	400.13
Spectral Width	8012.8
Lowest Frequency	-1544.6
Nucleus	1H
Acquired Size	16025
Spectral Size	65536

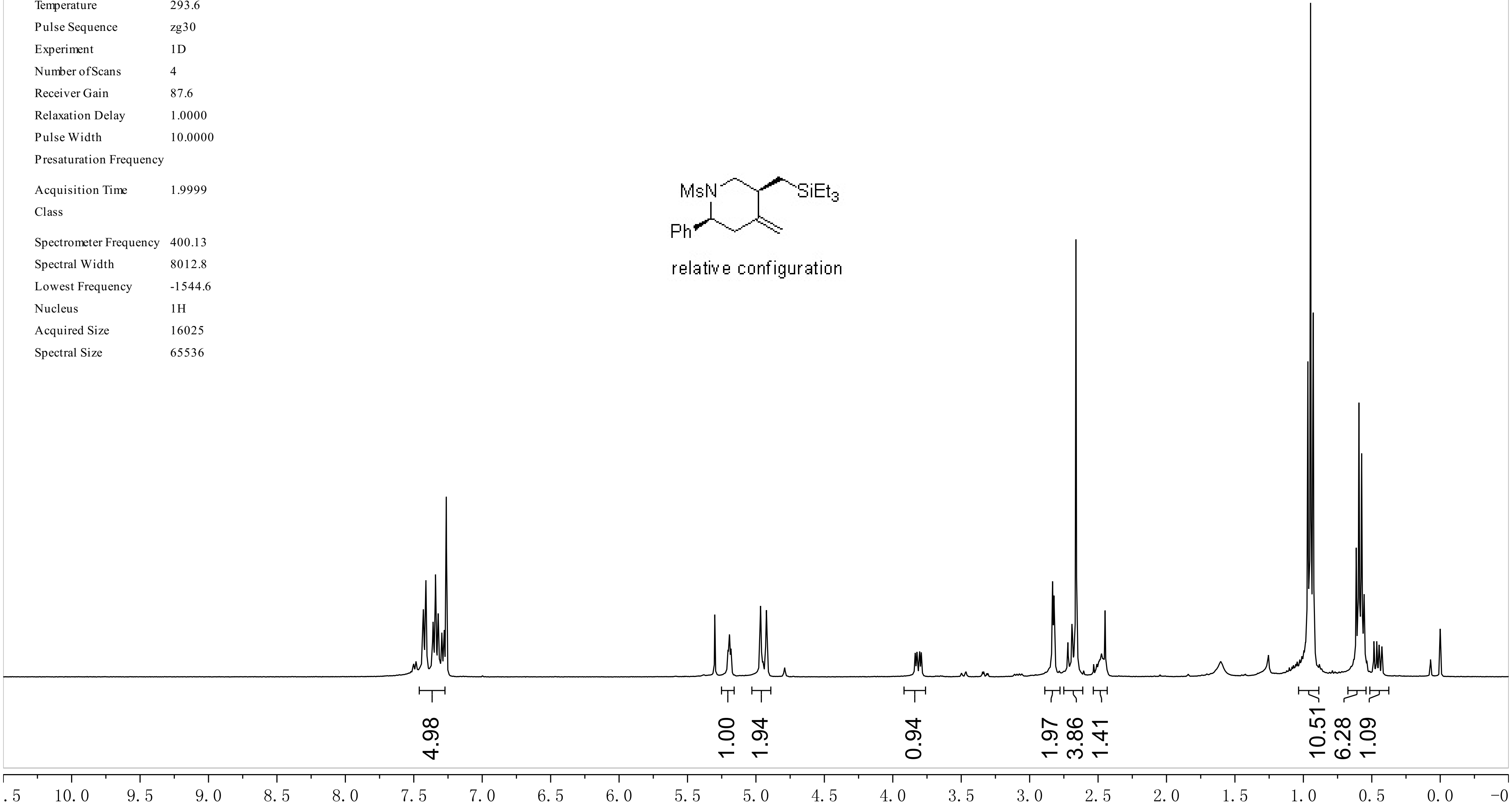

Parameter	Value
Title	xfy-181227-8-s2.22.fid
Comment	
Origin	Bruker BioSpin GmbH
Owner	nmr
Site	
Instrument	spect
Solvent	CDCl3
Temperature	294.2
Pulse Sequence	zgpg30
Experiment	1D
Number of Scans	256
Receiver Gain	196.4
Relaxation Delay	2.0000
Pulse Width	10.0000
Presaturation Frequency	
Acquisition Time	1.3631
Class	
Spectrometer Frequency	100.62
Spectral Width	24038.5
Lowest Frequency	-1958.0
Nucleus	13C
Acquired Size	32768
Spectral Size	65536

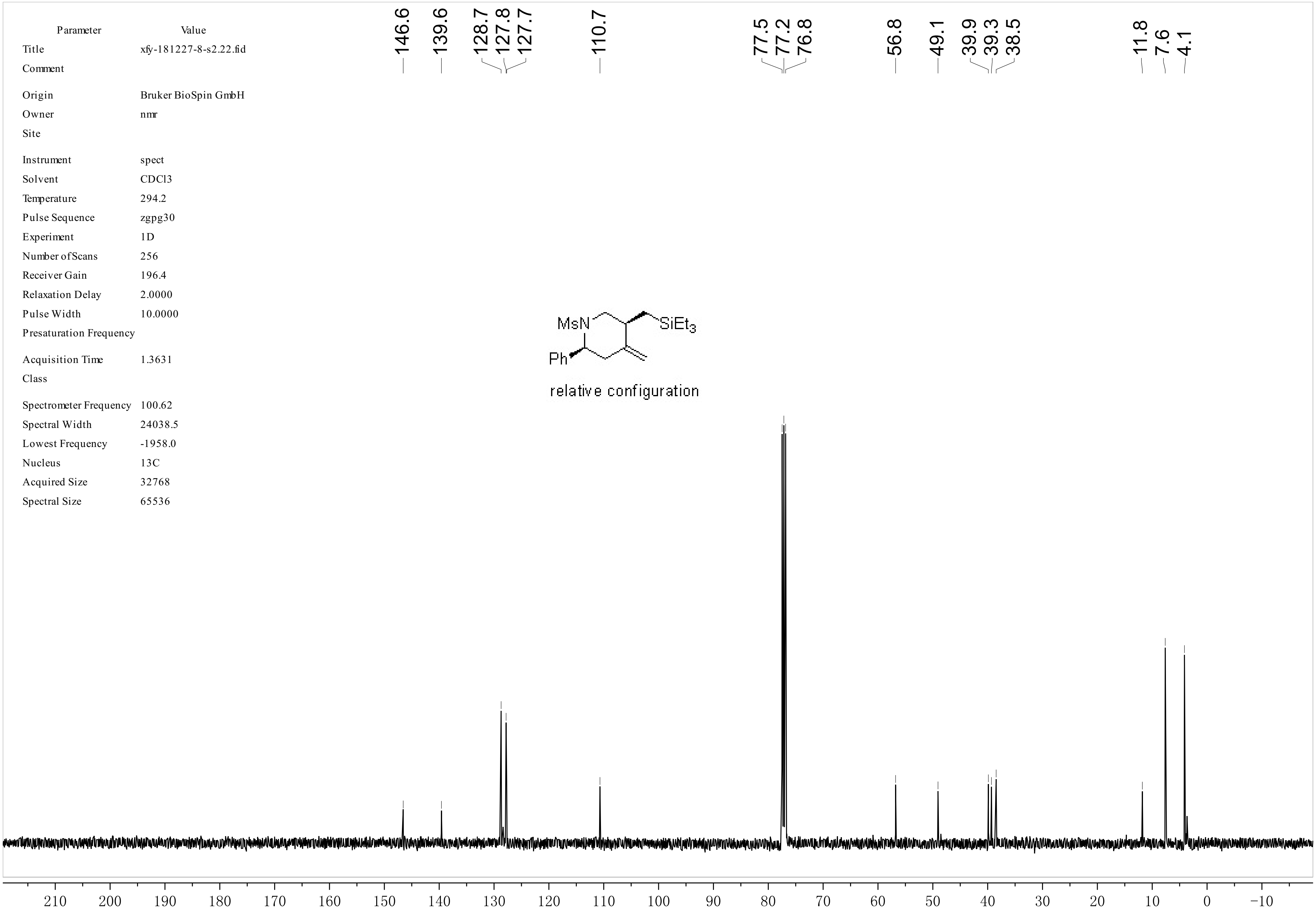

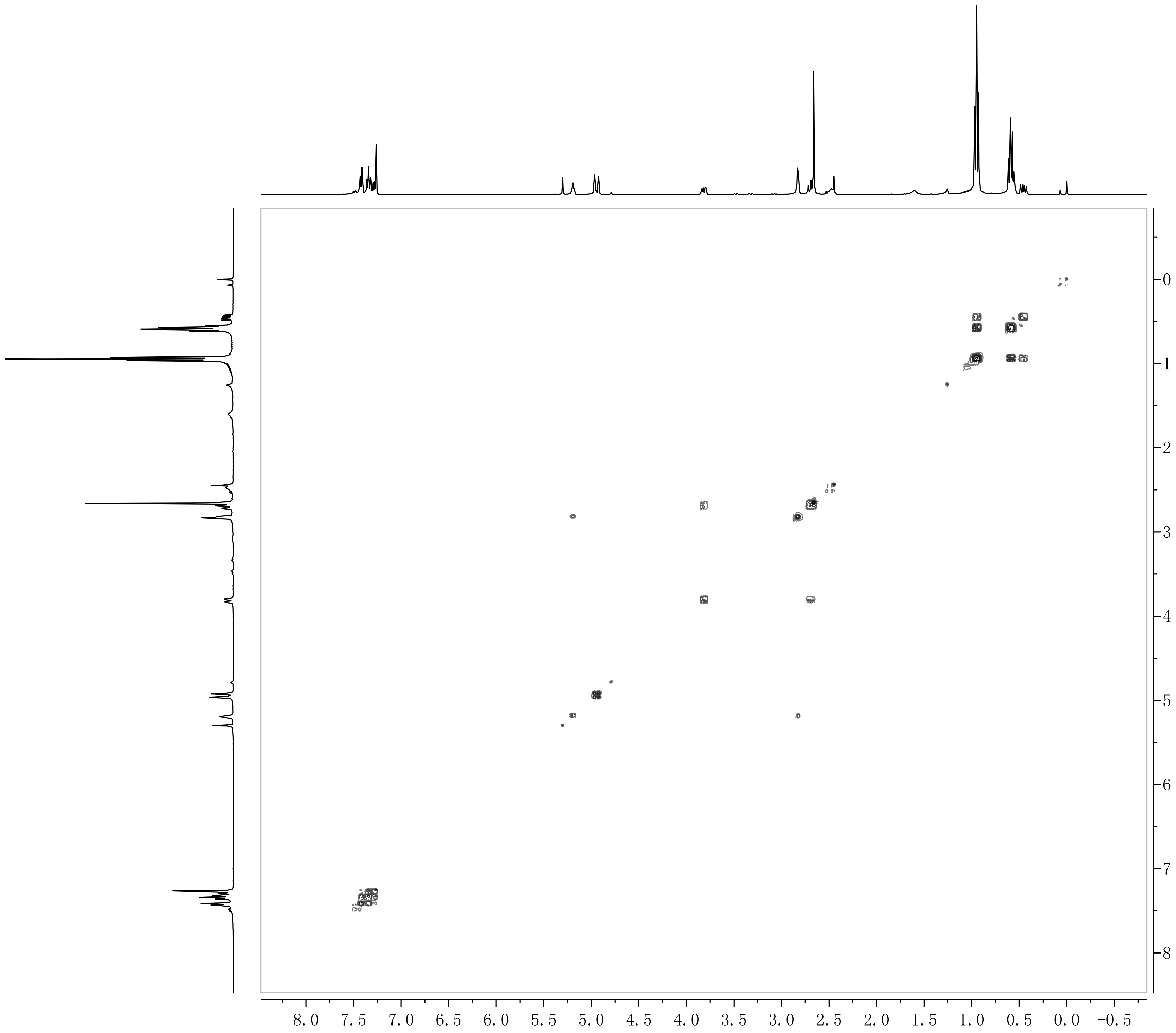

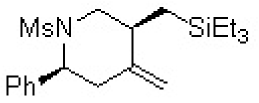

relative configuration

Parameter	Value
Title	xfy-181227-8-s2.23.ser
Comment	
Origin	Bruker BioSpin GmbH
Owner	nmr
Site	
Instrument	spect
Solvent	CDCl3
Temperature	293.9
Pulse Sequence	cosygpppqf
Experiment	COSY
Number of Scans	4
Receiver Gain	49.4
Relaxation Delay	1.9173
Pulse Width	10.0000
Presaturation Frequency	
Acquisition Time	0.2744
Class	
Spectrometer Frequency	(400.13, 400.13)
Spectral Width	(3731.3, 3731.3)
Lowest Frequency	(-341.1, -341.1)
Nucleus	(1H, 1H)
Acquired Size	(1024, 128)
Spectral Size	(1024, 1024)

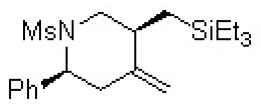

relative configuration

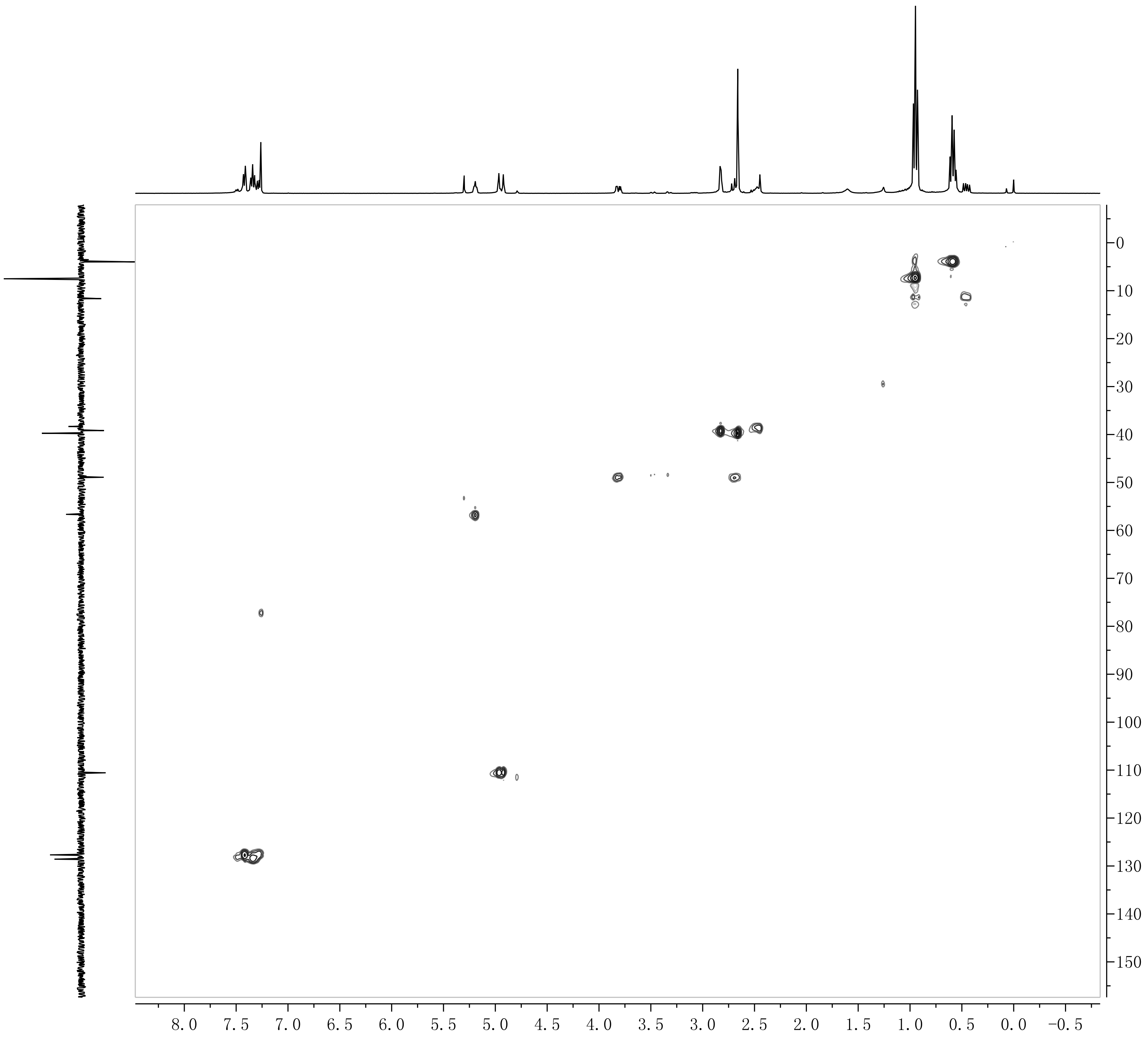

Parameter	Value
Title	xfy-181227-8-s2.25.ser
Comment	
Origin	Bruker BioSpin GmbH
Owner	nmr
Site	
Instrument	spect
Solvent	CDCl3
Temperature	293.7
Pulse Sequence	hsqcetgp
Experiment	HSQC
Number of Scans	4
Receiver Gain	196.4
Relaxation Delay	1.4613
Pulse Width	10.0000
Presaturation Frequency	
Acquisition Time	0.1372
Class	
Spectrometer Frequency	(400.13, 100.62)
Spectral Width	(3731.3, 16666.7)
Lowest Frequency	(-341.1, -829.1)
Nucleus	(1H, 13C)
Acquired Size	(512, 256)
Spectral Size	(512, 512)

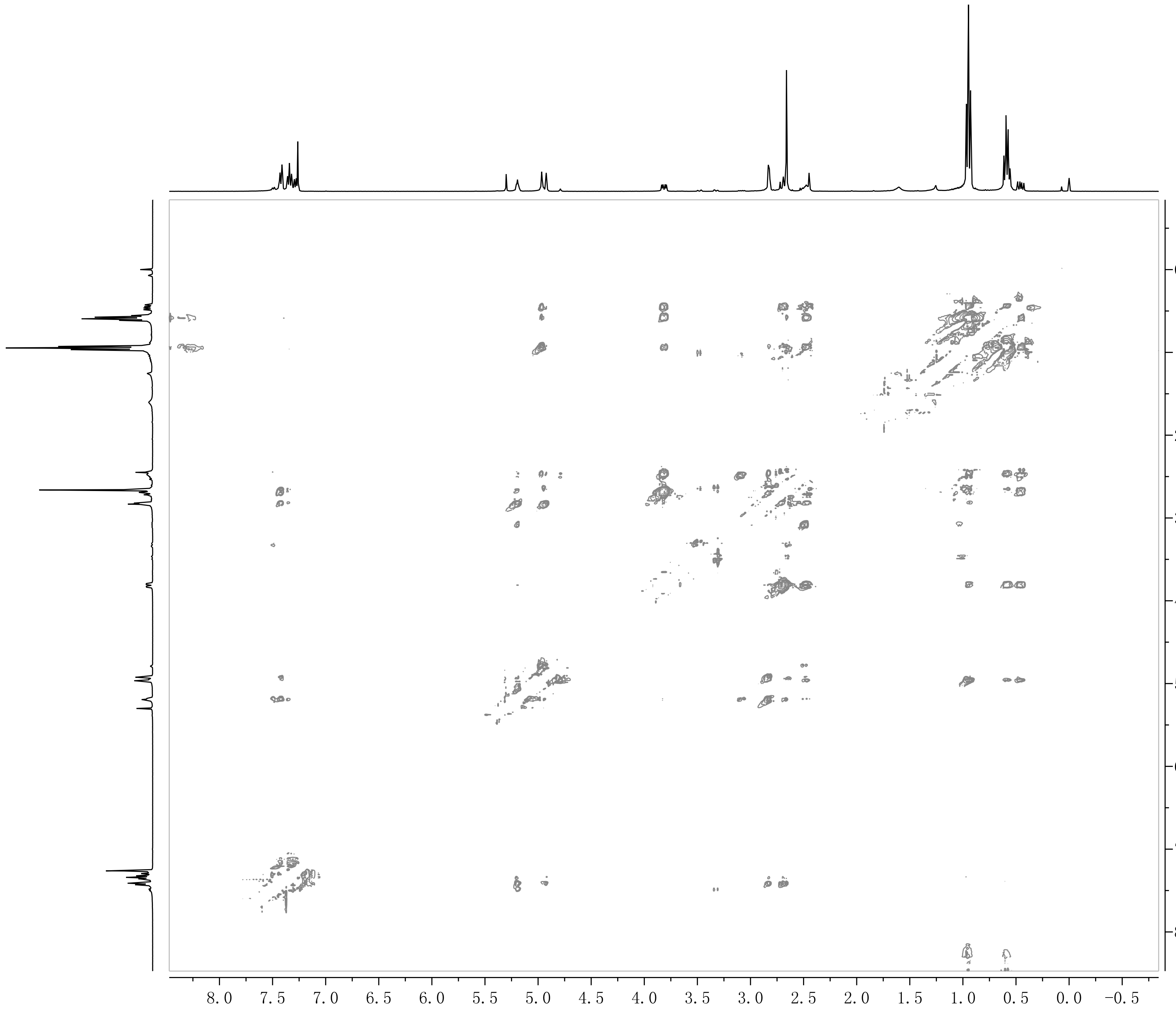

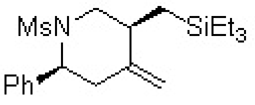

relative configuration

Parameter	Value
Title	xfy-181227-8-s2.24.ser
Comment	
Origin	Bruker BioSpin GmbH
Owner	nmr
Site	
Instrument	spect
Solvent	CDCl3
Temperature	293.6
Pulse Sequence	noesygpphpp
Experiment	NOESY
Number of Scans	8
Receiver Gain	49.4
Relaxation Delay	1.9816
Pulse Width	10.0000
Presaturation Frequency	
Acquisition Time	0.2744
Class	
Spectrometer Frequency	(400.13, 400.13)
Spectral Width	(3731.3, 3731.3)
Lowest Frequency	(-341.1, -341.1)
Nucleus	(1H, 1H)
Acquired Size	(1024, 256)
Spectral Size	(1024, 1024)

Parameter	Value
Title	xfy-181227-6-s1.1.1.1r
Comment	
Origin	Bruker BioSpin GmbH
Owner	nmr
Site	
Instrument	spect
Solvent	CDCl3
Temperature	293.7
Pulse Sequence	zg30
Experiment	1D
Number of Scans	8
Receiver Gain	76.6
Relaxation Delay	1.0000
Pulse Width	10.0000
Presaturation Frequency	
Acquisition Time	1.9999
Class	
Spectrometer Frequency	400.13
Spectral Width	8012.8
Lowest Frequency	-1545.4
Nucleus	1H
Acquired Size	16025
Spectral Size	65536

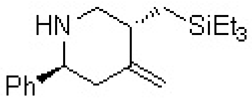

relative configuration

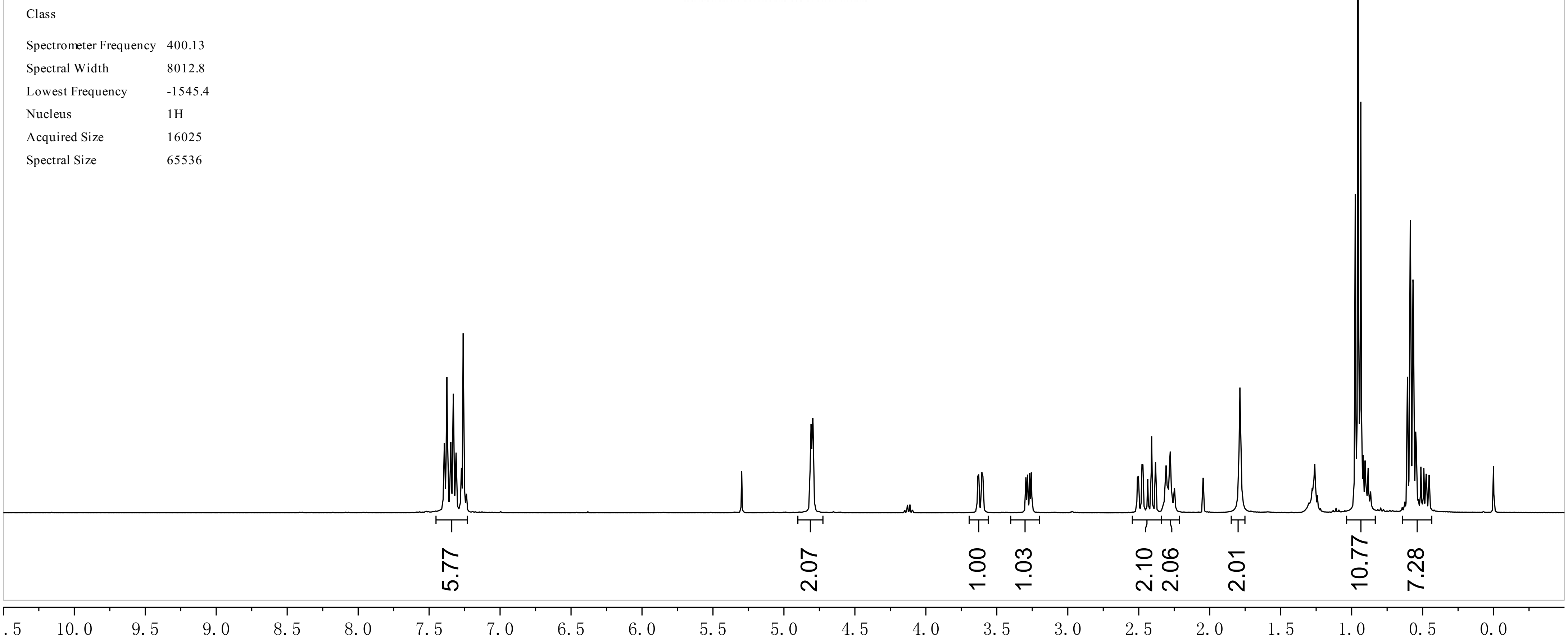

Parameter	Value
Title	xfy-181227-6-s1.2.1.1r
Comment	
Origin	Bruker BioSpin GmbH
Owner	nmr
Site	
Instrument	spect
Solvent	CDCl3
Temperature	293.9
Pulse Sequence	zgpg30
Experiment	1D
Number of Scans	80
Receiver Gain	196.4
Relaxation Delay	2.0000
Pulse Width	10.0000
Presaturation Frequency	
Acquisition Time	1.3631
Class	
Spectrometer Frequency	100.61
Spectral Width	24038.5
Lowest Frequency	-1958.0
Nucleus	13C
Acquired Size	32768
Spectral Size	32768

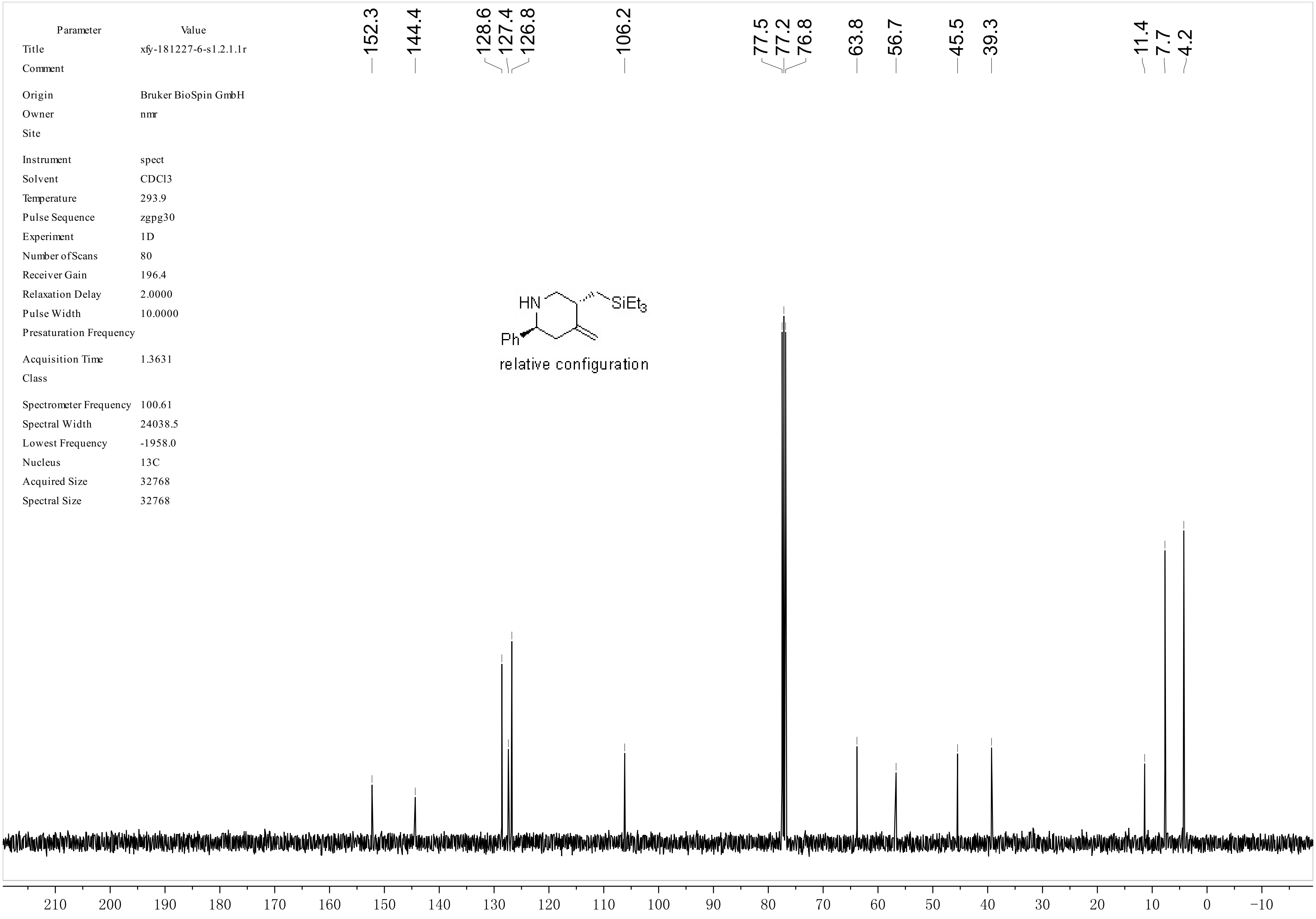

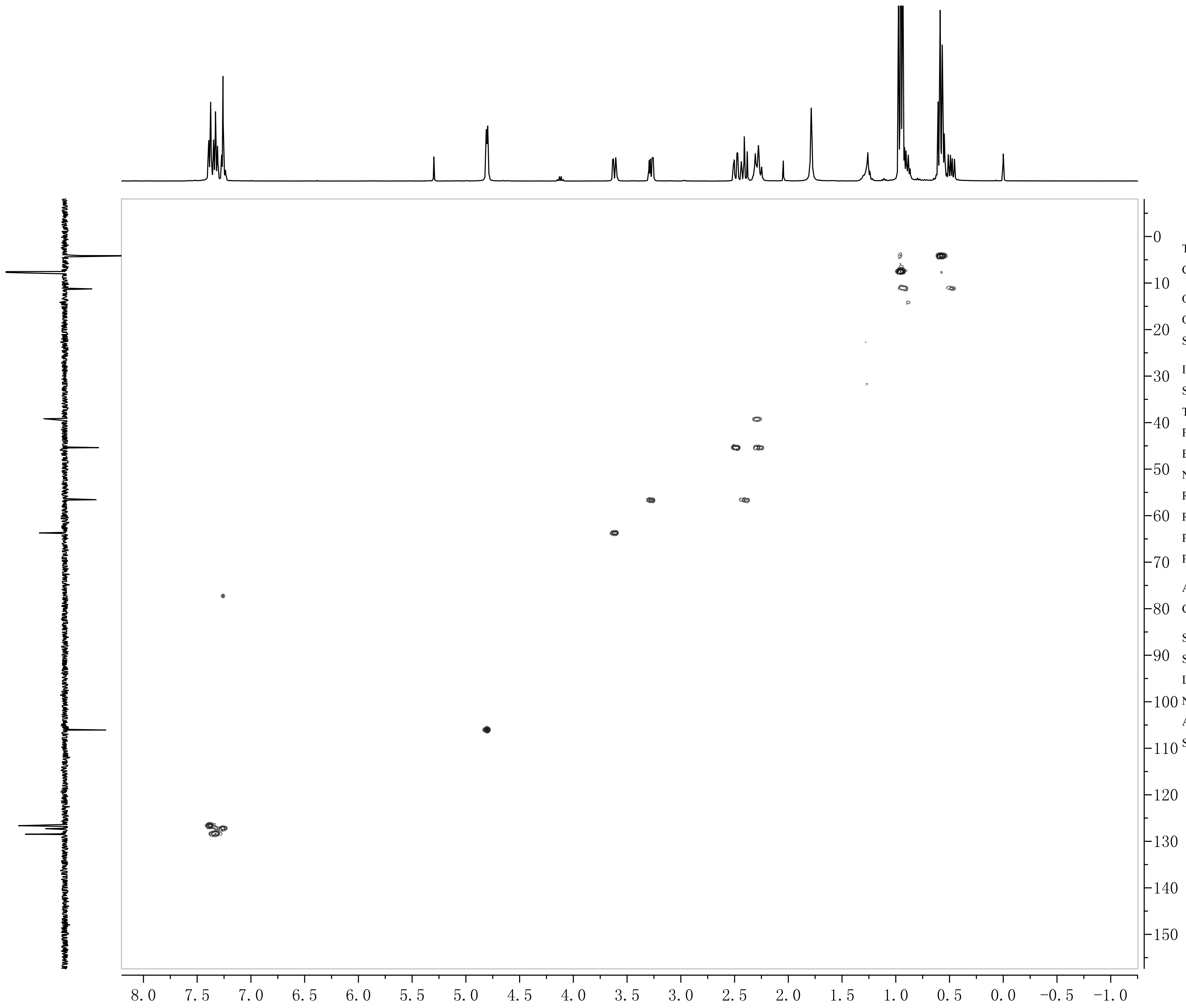

Parameter	Value
Title	xfy-181227-6-s1.4.1.2rr
Comment	
Origin	Bruker BioSpin GmbH
Owner	nmr
Site	
Instrument	spect
Solvent	CDCl3
Temperature	294.0
Pulse Sequence	hsqcetgp
Experiment	HSQC
Number of Scans	2
Receiver Gain	196.4
Relaxation Delay	1.4633
Pulse Width	10.0000
Presaturation Frequency	
Acquisition Time	0.1352
Class	
Spectrometer Frequency	(400.13, 100.61)
Spectral Width	(3787.9, 16666.7)
Lowest Frequency	(-504.9, -829.1)
Nucleus	(1H, 13C)
Acquired Size	(512, 256)
Spectral Size	(1024, 1024)

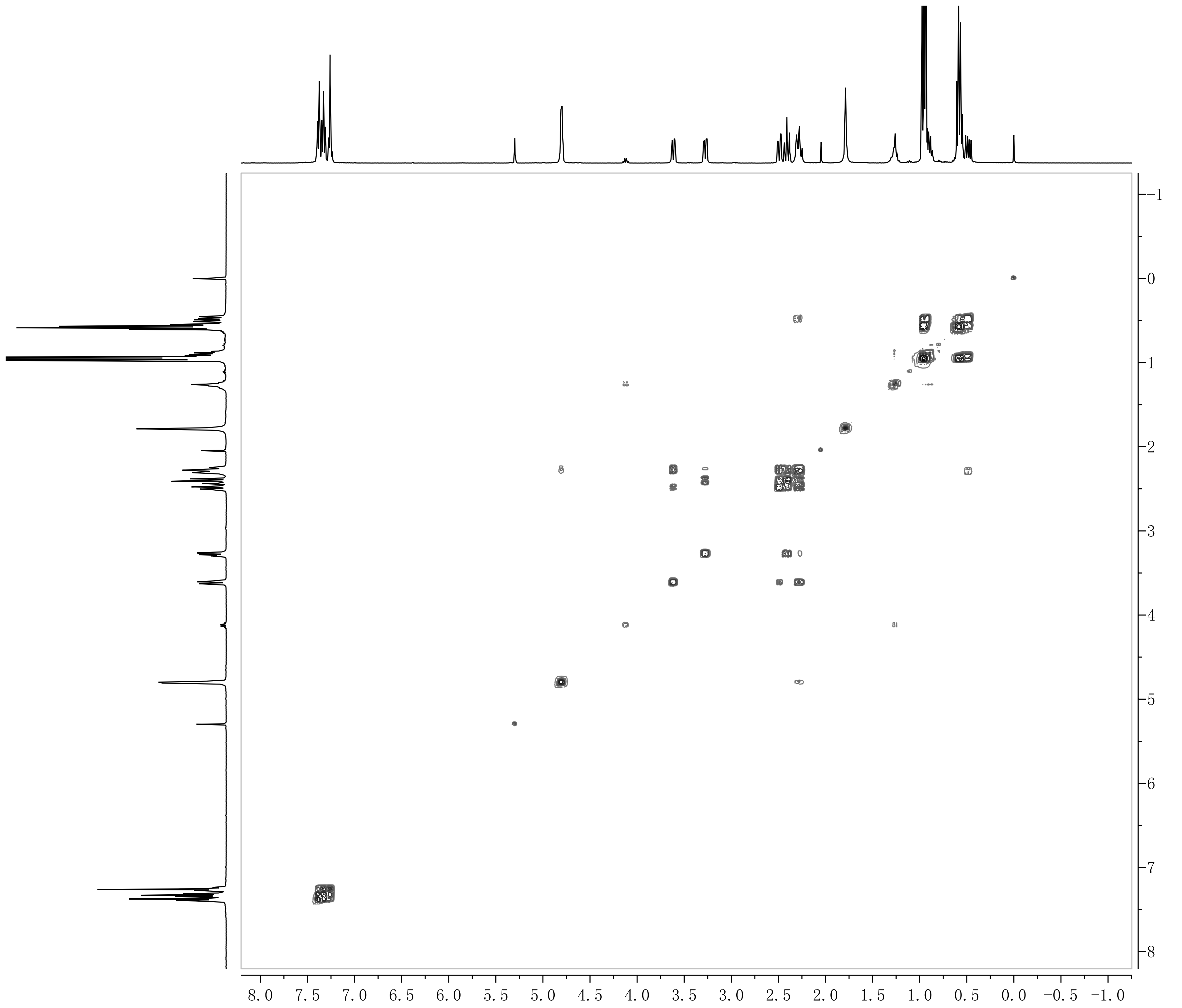

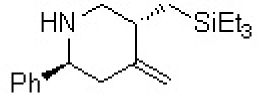

relative configuration

Parameter	Value
Title	xfy-181227-6-s1.5.ser
Comment	
Origin	Bruker BioSpin GmbH
Owner	nmr
Site	
Instrument	spect
Solvent	CDCl3
Temperature	294.0
Pulse Sequence	cosygpppqf
Experiment	COSY
Number of Scans	1
Receiver Gain	49.4
Relaxation Delay	1.9214
Pulse Width	10.0000
Presaturation Frequency	
Acquisition Time	0.2703
Class	
Spectrometer Frequency	(400.13, 400.13)
Spectral Width	(3787.9, 3787.9)
Lowest Frequency	(-504.9, -504.9)
Nucleus	(1H, 1H)
Acquired Size	(1024, 128)
Spectral Size	(1024, 1024)

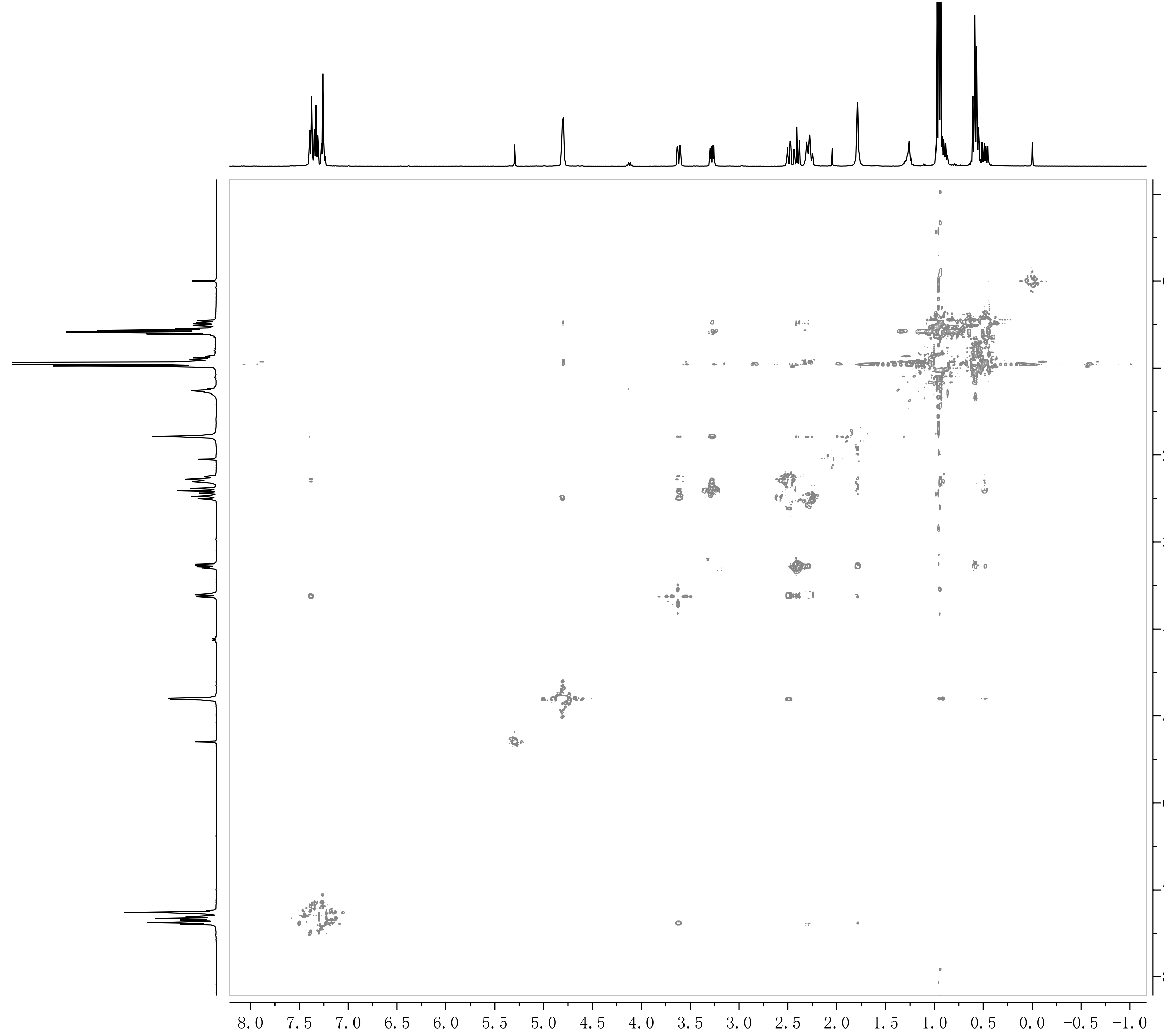

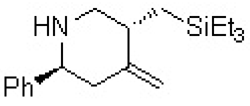

relative configuration

Parameter	Value
Title	xfy-181227-6-s1.6.1.2rr
Comment	
Origin	Bruker BioSpin GmbH
Owner	nmr
Site	
Instrument	spect
Solvent	CDCl3
Temperature	293.7
Pulse Sequence	noesygpphpp
Experiment	NOESY
Number of Scans	4
Receiver Gain	45.6
Relaxation Delay	1.9836
Pulse Width	10.0000
Presaturation Frequency	
Acquisition Time	0.2724
Class	
Spectrometer Frequency	(400.13, 400.13)
Spectral Width	(3759.4, 3759.4)
Lowest Frequency	(-472.0, -472.0)
Nucleus	(1H, 1H)
Acquired Size	(1024, 256)
Spectral Size	(1024, 1024)

Parameter	Value
Title	xfy-181227-6-dMs.11.1.1r
Comment	
Origin	Bruker BioSpin GmbH
Owner	nmr
Site	
Instrument	spect
Solvent	CDCl3
Temperature	294.2
Pulse Sequence	zg30
Experiment	1D
Number of Scans	8
Receiver Gain	25.3
Relaxation Delay	1.0000
Pulse Width	10.0000
Presaturation Frequency	
Acquisition Time	1.9999
Class	
Spectrometer Frequency	400.13
Spectral Width	8012.8
Lowest Frequency	-1544.3
Nucleus	1H
Acquired Size	16025
Spectral Size	65536

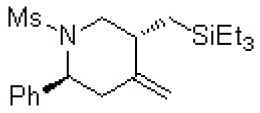

relative configuration

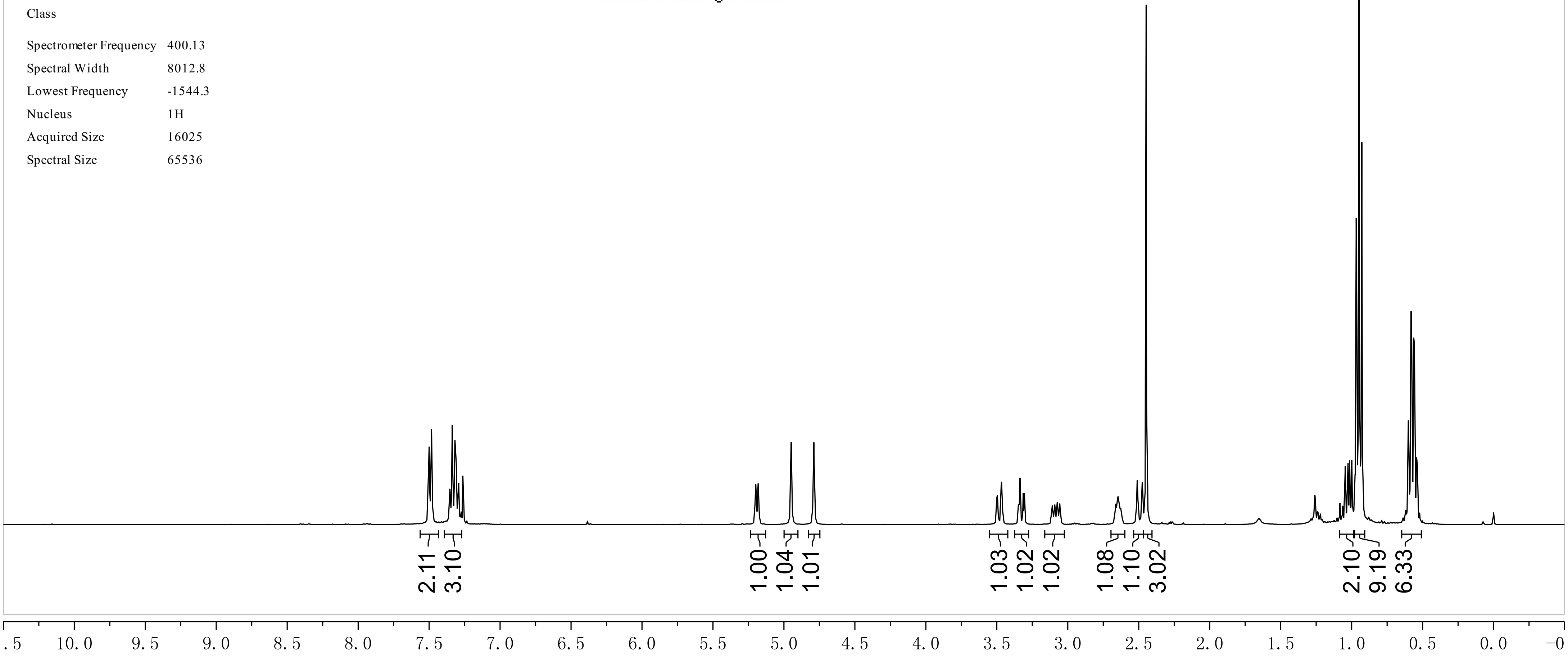

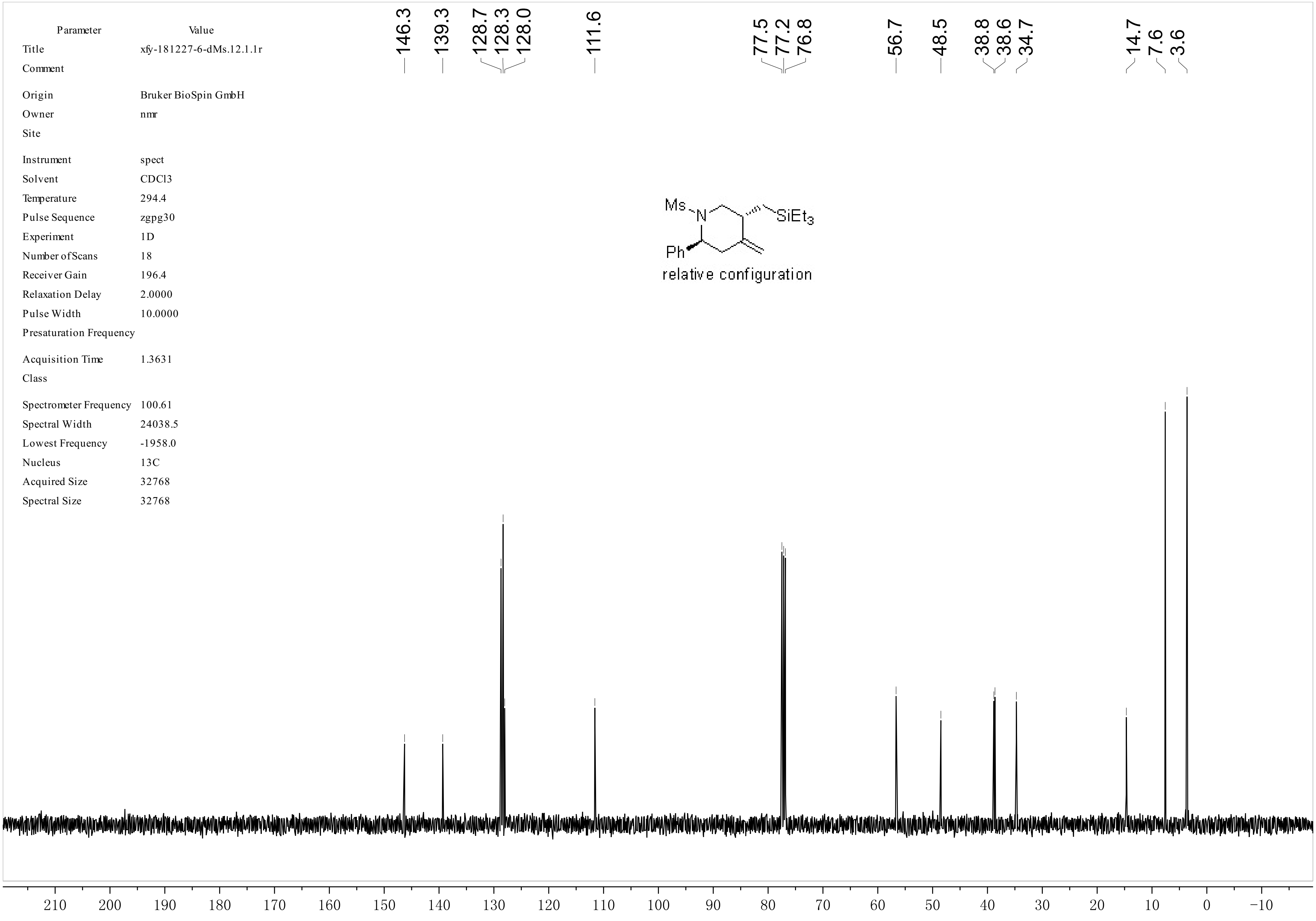

Parameter Value
Title xfy-181227-6-dMs.12.1.1r
Comment
Origin Bruker BioSpin GmbH
Owner nmr
Site
Instrument spect
Solvent CDCl3
Temperature 294.4
Pulse Sequence zgpg30
Experiment 1D
Number of Scans 18
Receiver Gain 196.4
Relaxation Delay 2.0000
Pulse Width 10.0000
Presaturation Frequency
Acquisition Time 1.3631
Class
Spectrometer Frequency 100.61
Spectral Width 24038.5
Lowest Frequency -1958.0
Nucleus 13C
Acquired Size 32768
Spectral Size 32768
146.3
139.3
128.7
128.3
128.0
111.6
77.5
77.2
76.8
56.7
48.5
38.8
38.6
34.7
14.7
7.6
3.6
Ms
N
SiEt3
Ph
relative configuration
210 200 190 180 170 160 150 140 130 120 110 100 90 80 70 60 50 40 30 20 10 0 −10

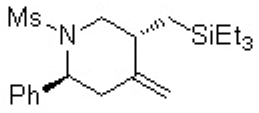

relative configuration

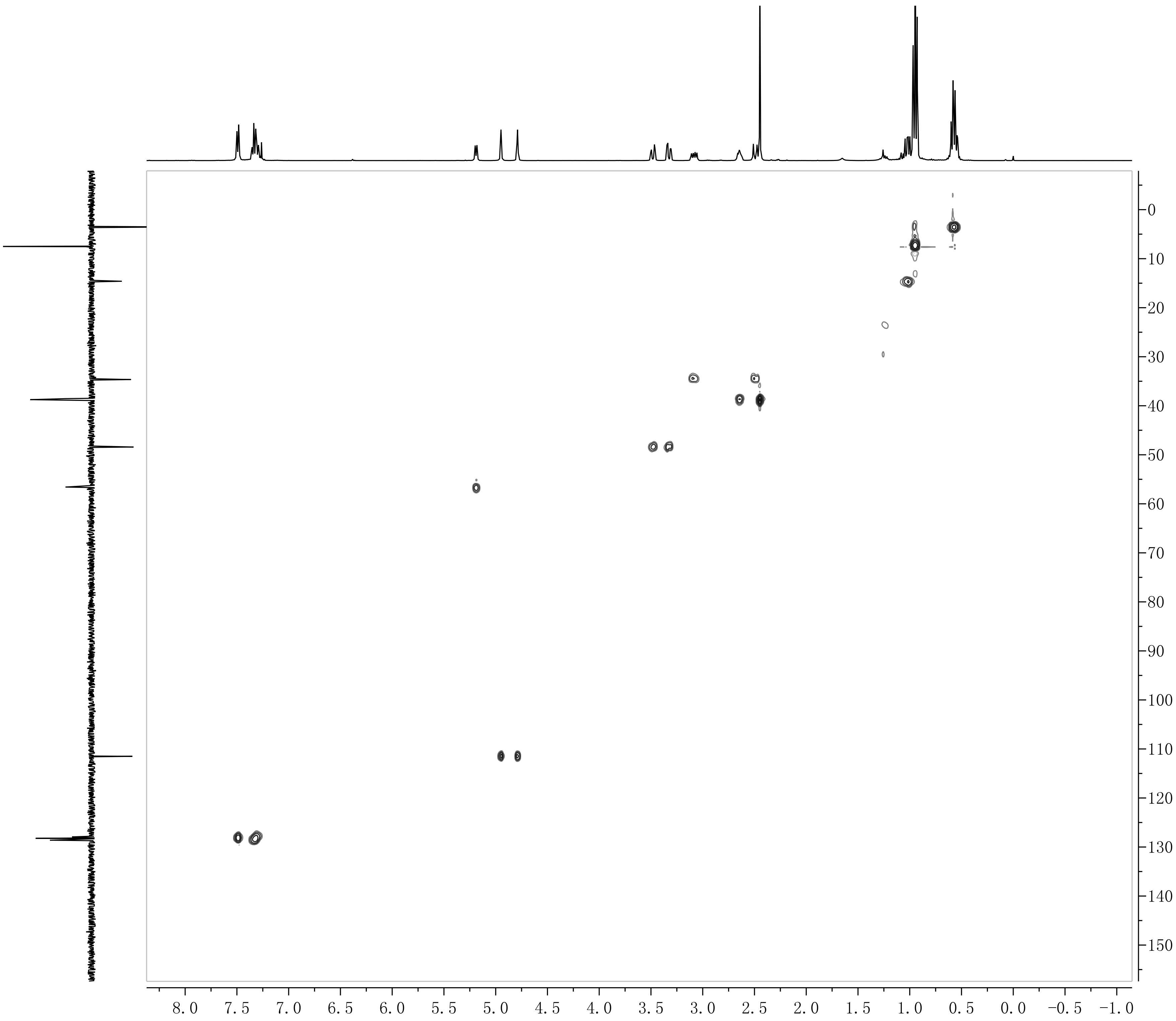

Parameter	Value
Title	xfy-181227-6-dMs.14.ser
Comment	
Origin	Bruker BioSpin GmbH
Owner	nmr
Site	
Instrument	spect
Solvent	CDCl3
Temperature	294.4
Pulse Sequence	hsqcetgp
Experiment	HSQC
Number of Scans	2
Receiver Gain	196.4
Relaxation Delay	1.4644
Pulse Width	10.0000
Presaturation Frequency	
Acquisition Time	0.1341
Class	
Spectrometer Frequency	(400.13, 100.62)
Spectral Width	(3816.8, 16666.7)
Lowest Frequency	(-466.7, -829.1)
Nucleus	(1H, 13C)
Acquired Size	(512, 256)
Spectral Size	(512, 512)

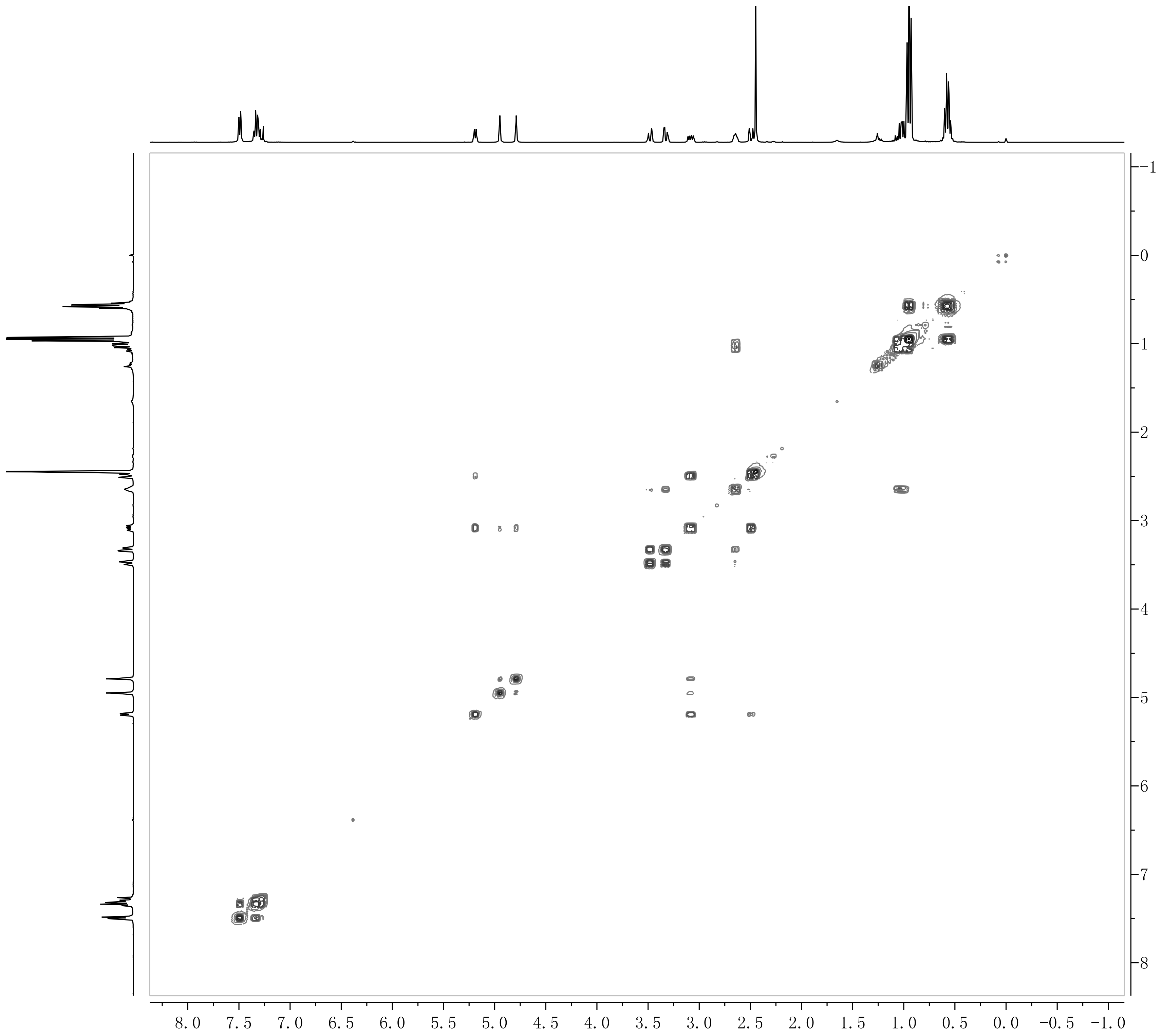

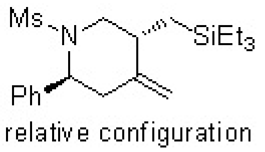

Parameter	Value
Title	xfy-181227-6-dMs.15.ser
Comment	
Origin	Bruker BioSpin GmbH
Owner	nmr
Site	
Instrument	spect
Solvent	CDCl3
Temperature	294.5
Pulse Sequence	cosygpppqf
Experiment	COSY
Number of Scans	1
Receiver Gain	19.6
Relaxation Delay	1.9234
Pulse Width	10.0000
Presaturation Frequency	
Acquisition Time	0.2683
Class	
Spectrometer Frequency	(400.13, 400.13)
Spectral Width	(3816.8, 3816.8)
Lowest Frequency	(-466.7, -466.7)
Nucleus	(1H, 1H)
Acquired Size	(1024, 128)
Spectral Size	(1024, 1024)

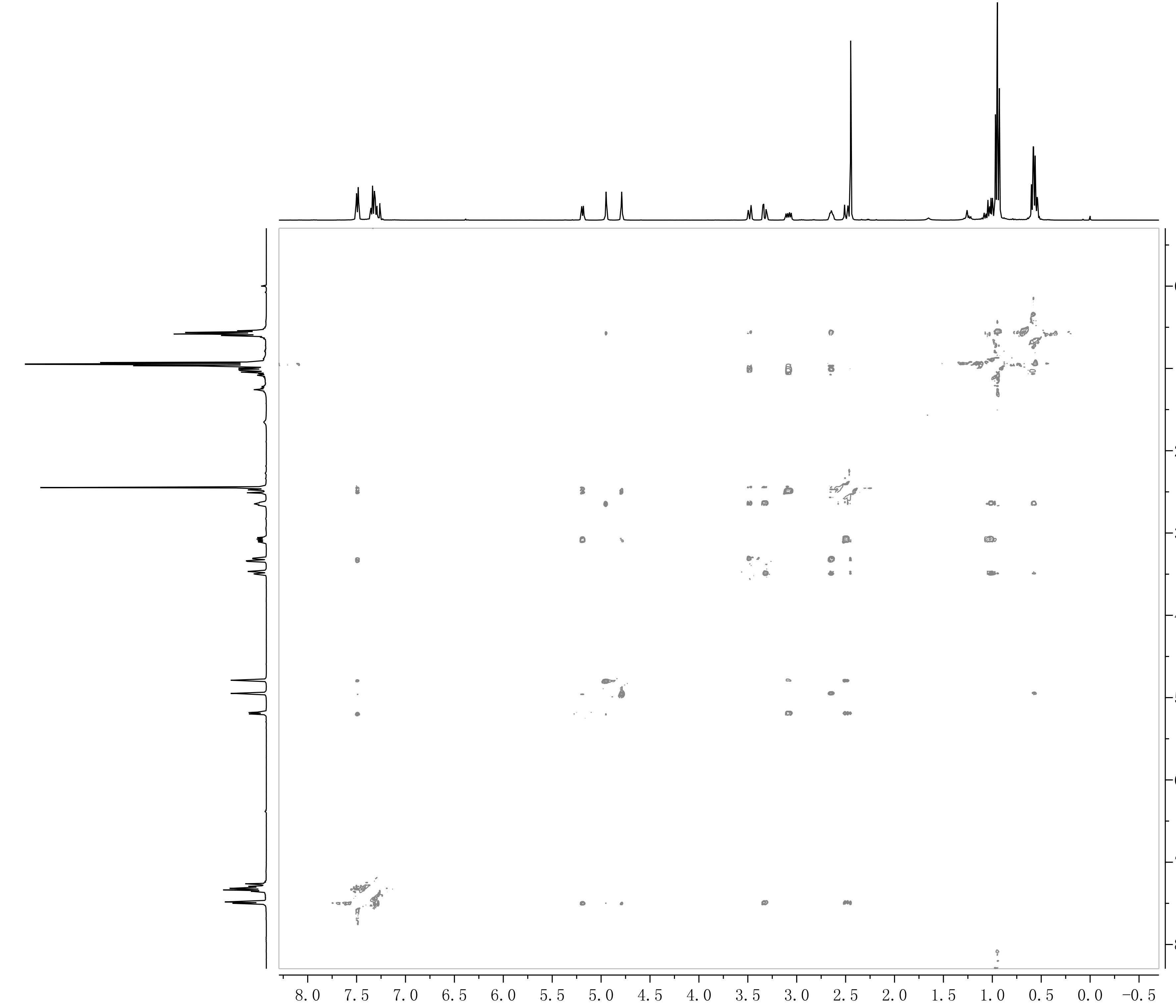

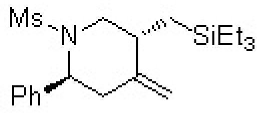

relative configuration

Parameter	Value
Title	xfy-181227-6-dMs.22.1.2rr
Comment	
Origin	Bruker BioSpin GmbH
Owner	nmr
Site	
Instrument	spect
Solvent	CDCl3
Temperature	296.2
Pulse Sequence	noesygpphpp
Experiment	NOESY
Number of Scans	4
Receiver Gain	12.7
Relaxation Delay	1.9775
Pulse Width	10.7100
Presaturation Frequency	
Acquisition Time	0.2273
Class	
Spectrometer Frequency	(500.13, 500.13)
Spectral Width	(4504.5, 4504.5)
Lowest Frequency	(-356.3, -356.3)
Nucleus	(1H, 1H)
Acquired Size	(1024, 256)
Spectral Size	(1024, 1024)

Parameter	Value
Data File Name	E:/ NMR/ 2017/ 2017-5(26-31)/ xfy-0530-2/ 1/ fid
Comment	
Origin	Bruker BioSpin GmbH
Owner	nmr
Site	
Instrument	spect
Solvent	CDCl3
Temperature	296.2
Pulse Sequence	zg30
Experiment	1D
Number of Scans	9
Receiver Gain	31.1
Relaxation Delay	1.0000
Pulse Width	11.2900
Presaturation Frequency	
Acquisition Time	3.2768
Class	
Spectrometer Frequency	500.13
Spectral Width	10000.0
Lowest Frequency	-1924.1
Nucleus	1H
Acquired Size	32768
Spectral Size	65536

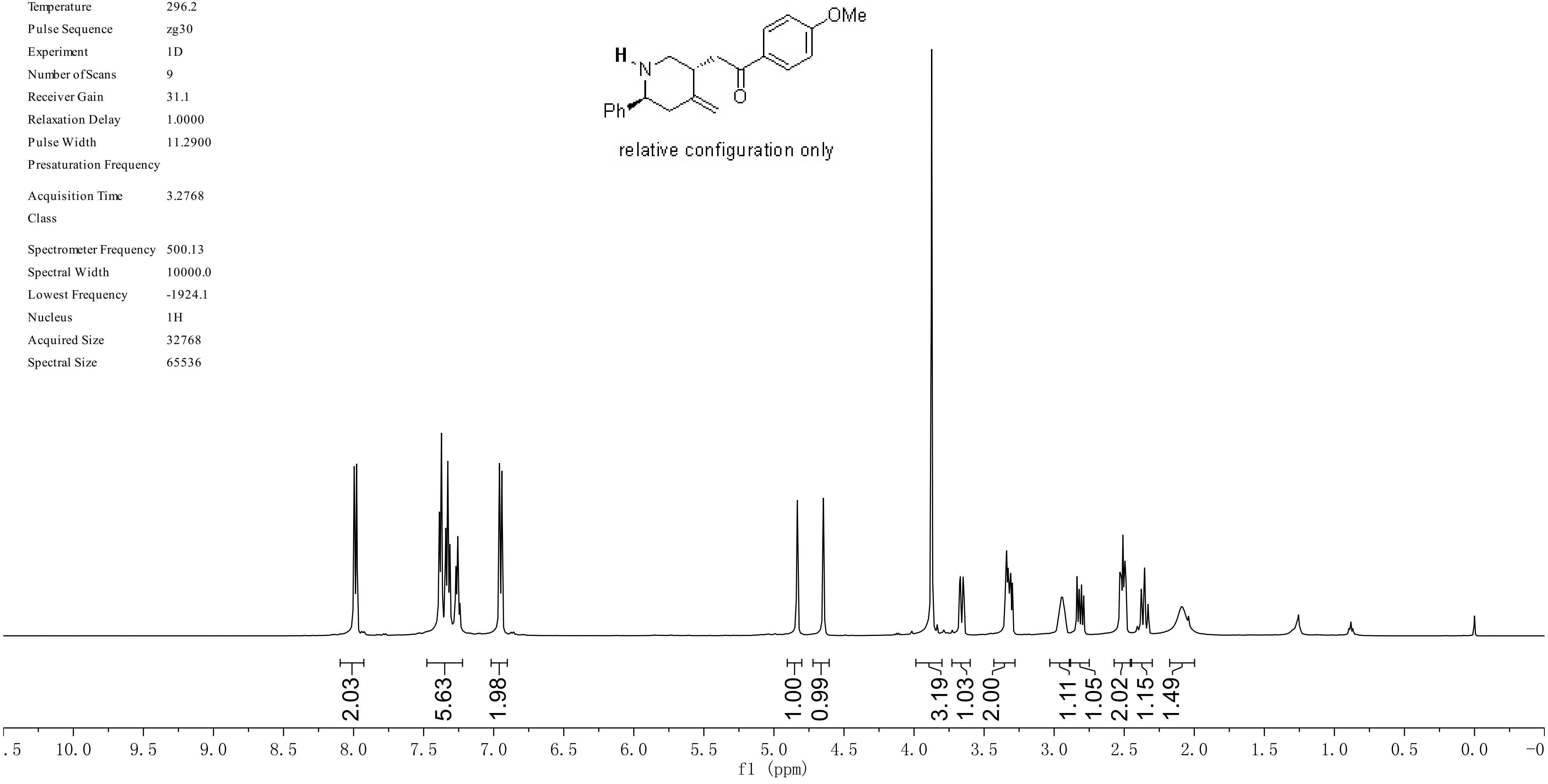

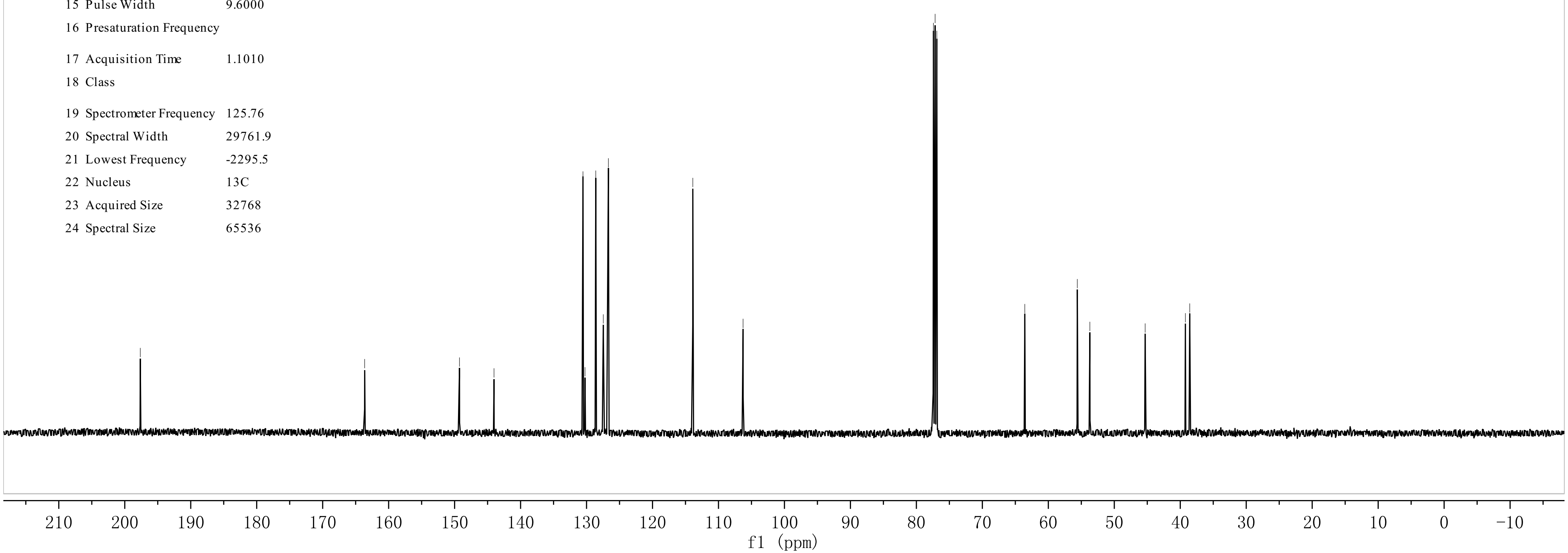

	Parameter	Value
1	Title	
2	Comment	
3	Origin	Bruker BioSpin GmbH
4	Owner	nmr
5	Site	
6	Instrument	spect
7	Author	
8	Solvent	CDCl3
9	Temperature	296.2
10	Pulse Sequence	zgpg30
11	Experiment	1D
12	Number of Scans	25
13	Receiver Gain	193.1
14	Relaxation Delay	2.0000
15	Pulse Width	9.6000
16	Presaturation Frequency	
17	Acquisition Time	1.1010
18	Class	
19	Spectrometer Frequency	125.76
20	Spectral Width	29761.9
21	Lowest Frequency	-2295.5
22	Nucleus	13C
23	Acquired Size	32768
24	Spectral Size	65536

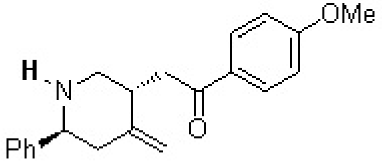

relative configuration only

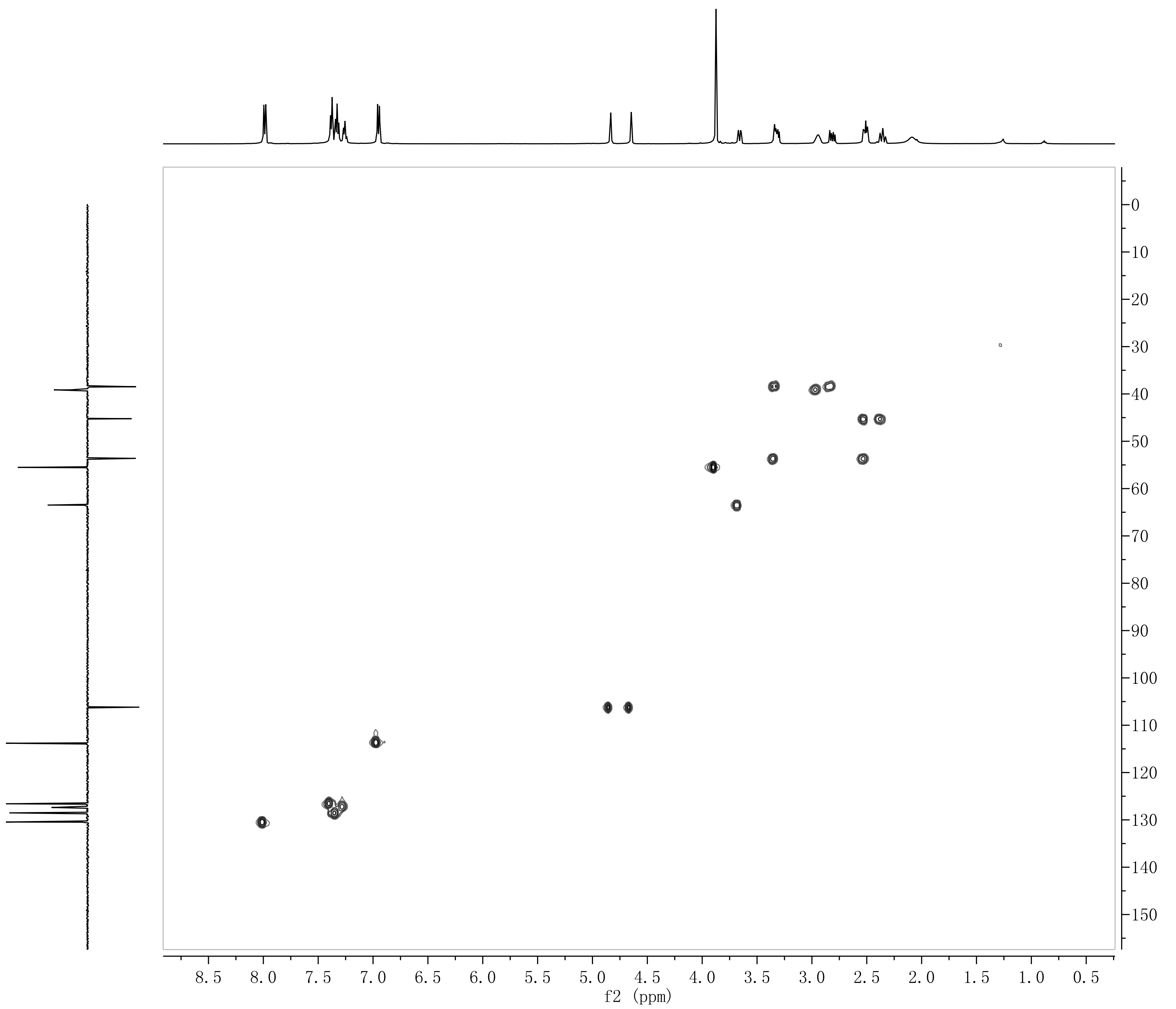

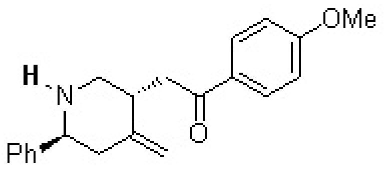

relative configuration only

Parameter	Value
Data File Name	E:/ NMR/ 2017/ 2017-5(26-31)/ xfy-0530-2/ 5/ ser
Comment	
Origin	Bruker BioSpin GmbH
Owner	nmr
Site	
Instrument	spect
Solvent	CDCl3
Temperature	296.1
Pulse Sequence	hsqcetgp
Experiment	HSQC
Number of Scans	2
Receiver Gain	193.1
Relaxation Delay	1.4611
Pulse Width	11.2900
Presaturation Frequency	
Acquisition Time	0.1178
Class	
Spectrometer Frequency	(500.13, 125.77)
Spectral Width	(4347.8, 20833.3)
Lowest Frequency	(110.1, -1037.0)
Nucleus	(1H, 13C)
Acquired Size	(512, 256)
Spectral Size	(512, 512)

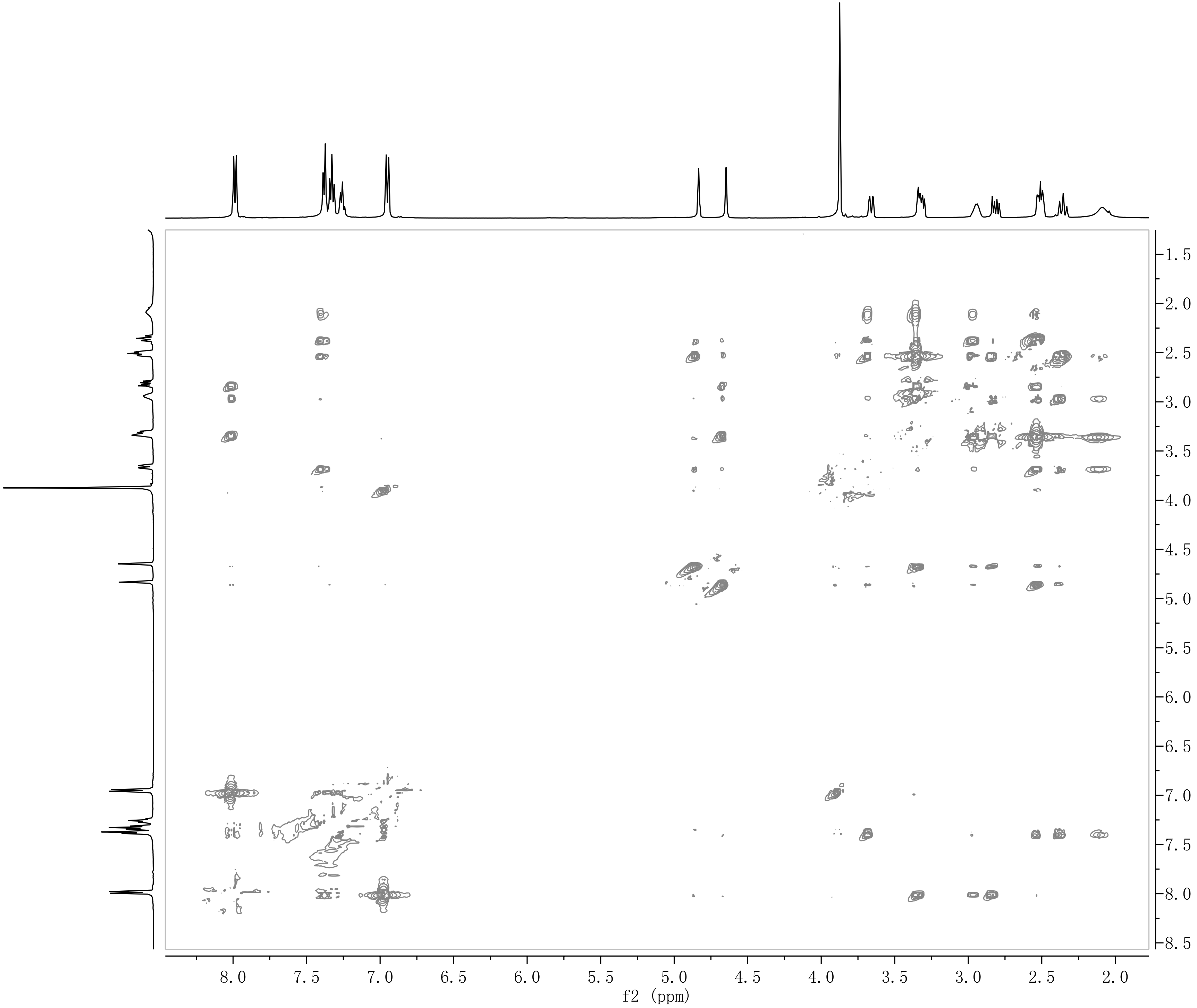

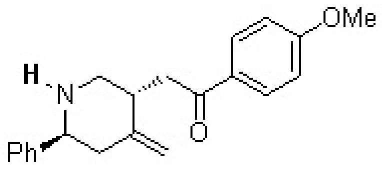

relative configuration only

	Parameter	Value
1	Title	
2	Comment	
3	Origin	Bruker BioSpin GmbH
4	Owner	nmr
5	Site	
6	Instrument	spect
7	Author	
8	Solvent	CDCl3
9	Temperature	296.2
10	Pulse Sequence	noesygpphpp
11	Experiment	NOESY
12	Number of Scans	4
13	Receiver Gain	21.9
14	Relaxation Delay	1.9693
15	Pulse Width	11.2900
16	Presaturation Frequency	
17	Acquisition Time	0.2355
18	Class	
19	Spectrometer Frequency	(500.13, 500.13)
20	Spectral Width	(4347.8, 4347.8)
21	Lowest Frequency	(112.9, 112.9)
22	Nucleus	(1H, 1H)
23	Acquired Size	(1024, 256)
24	Spectral Size	(1024, 1024)

	Parameter	Value
1	Title	xfy-0617-1.6.1
2	Comment	
3	Origin	Bruker BioSpin GmbH
4	Owner	nmr
5	Site	
6	Instrument	spect
7	Author	
8	Solvent	CDCl3
9	Temperature	296.1
10	Pulse Sequence	zg30
11	Experiment	1D
12	Number of Scans	9
13	Receiver Gain	77.6
14	Relaxation Delay	1.0000
15	Pulse Width	11.2900
16	Presaturation Frequency	
17	Acquisition Time	3.2768
18	Class	
19	Spectrometer Frequency	500.13
20	Spectral Width	10000.0
21	Lowest Frequency	-1922.6
22	Nucleus	1H
23	Acquired Size	32768
24	Spectral Size	65536

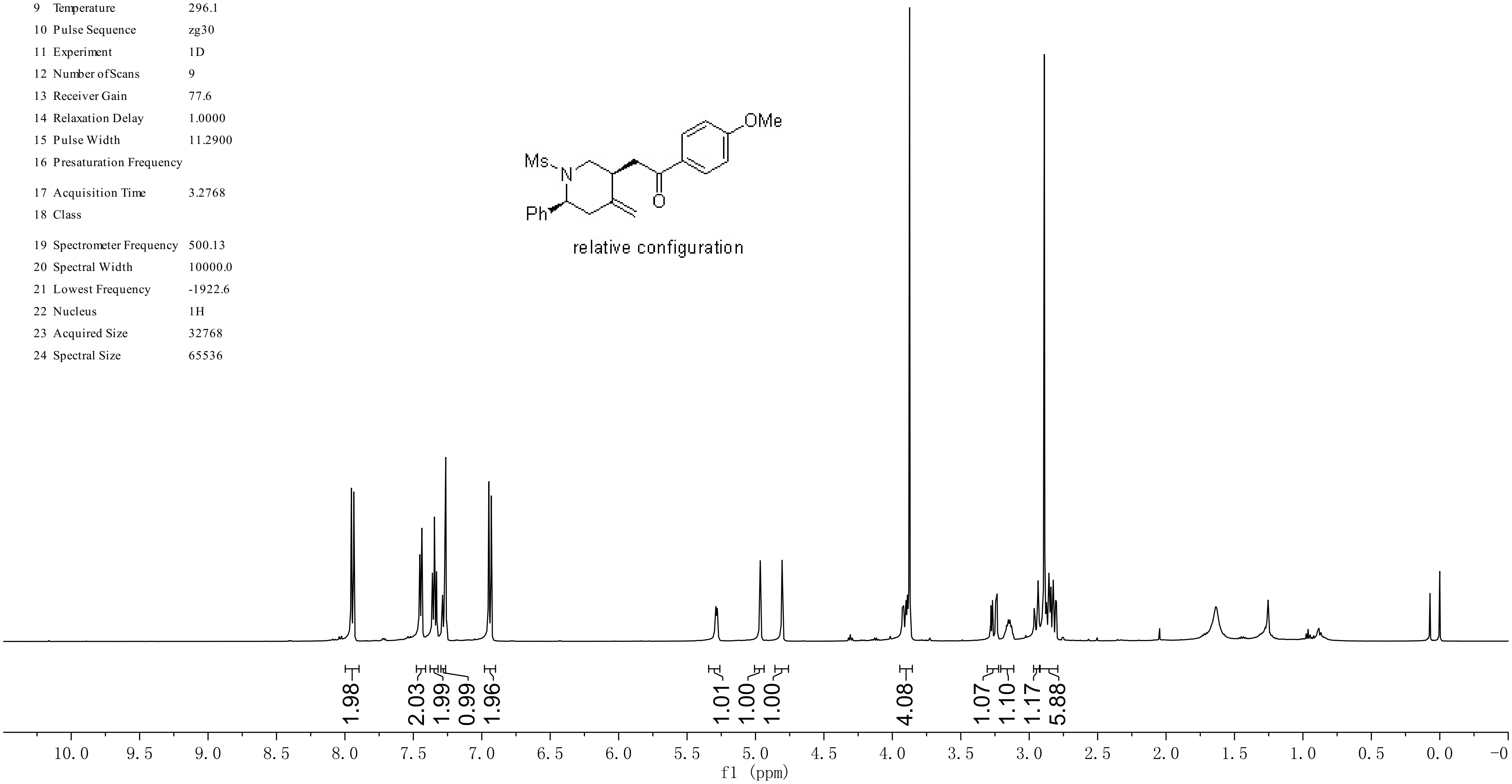

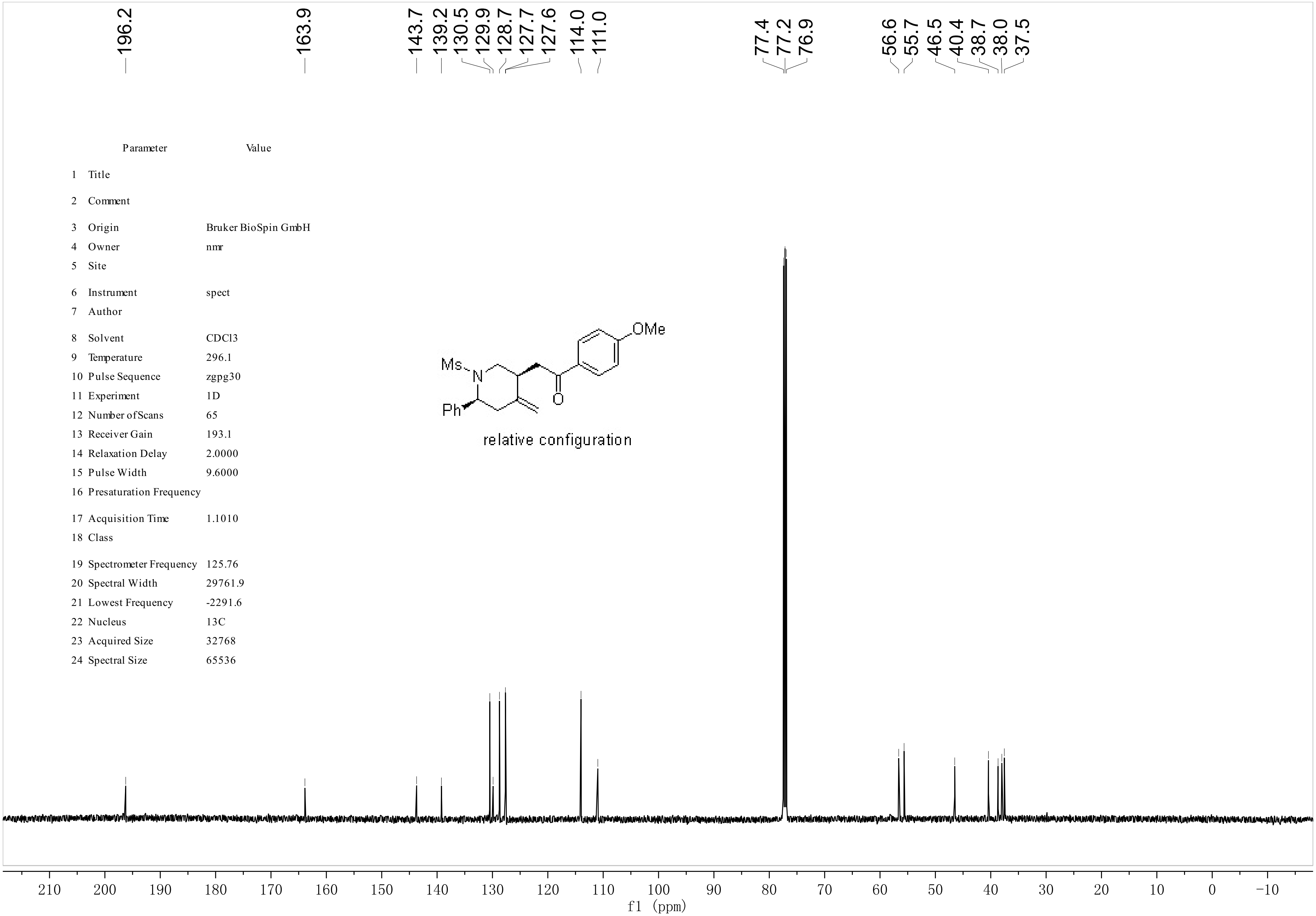

	Parameter	Value
1	Title	
2	Comment	
3	Origin	Bruker BioSpin GmbH
4	Owner	nmr
5	Site	
6	Instrument	spect
7	Author	
8	Solvent	CDCl3
9	Temperature	296.1
10	Pulse Sequence	zgpg30
11	Experiment	1D
12	Number of Scans	65
13	Receiver Gain	193.1
14	Relaxation Delay	2.0000
15	Pulse Width	9.6000
16	Presaturation Frequency	
17	Acquisition Time	1.1010
18	Class	
19	Spectrometer Frequency	125.76
20	Spectral Width	29761.9
21	Lowest Frequency	-2291.6
22	Nucleus	13C
23	Acquired Size	32768
24	Spectral Size	65536

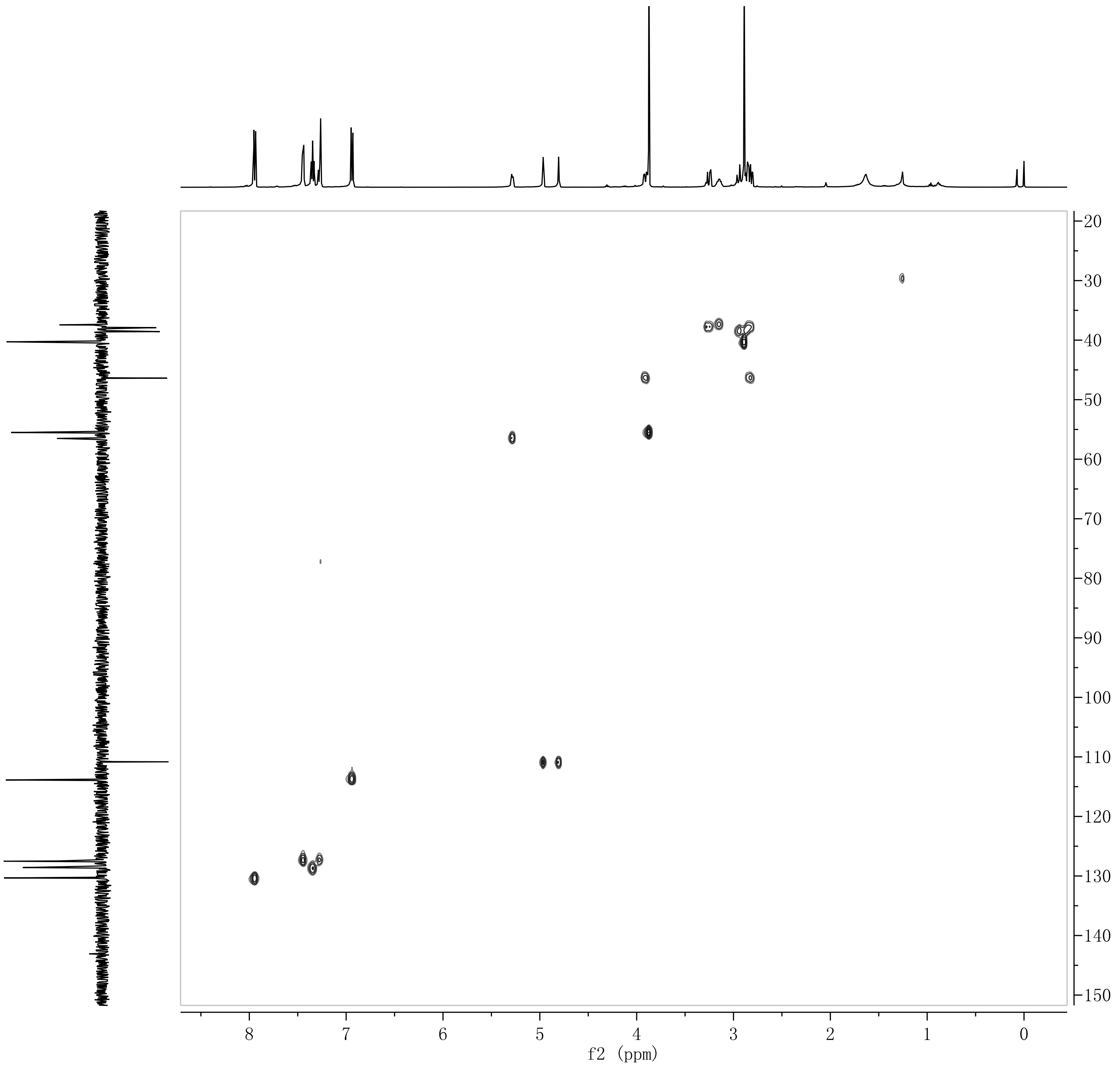

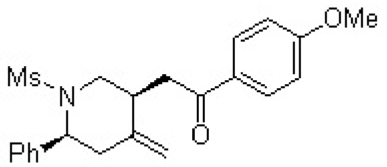

relative configuration

Parameter	Value
Data File Name	E:/ NMR/ 2017/ 2017-6-(15-21)/ xfy-0617-1/ 8/ ser
Comment	
Origin	Bruker BioSpin GmbH
Owner	nmr
Site	
Instrument	spect
Solvent	CDCl3
Temperature	296.1
Pulse Sequence	hsqcetgp
Experiment	HSQC
Number of Scans	2
Receiver Gain	193.1
Relaxation Delay	1.4744
Pulse Width	11.2900
Presaturation Frequency	
Acquisition Time	0.1044
Class	
Spectrometer Frequency	(500.13, 125.76)
Spectral Width	(4902.0, 20833.3)
Lowest Frequency	(-359.3, -1037.0)
Nucleus	(1H, 13C)
Acquired Size	(512, 256)
Spectral Size	(512, 512)

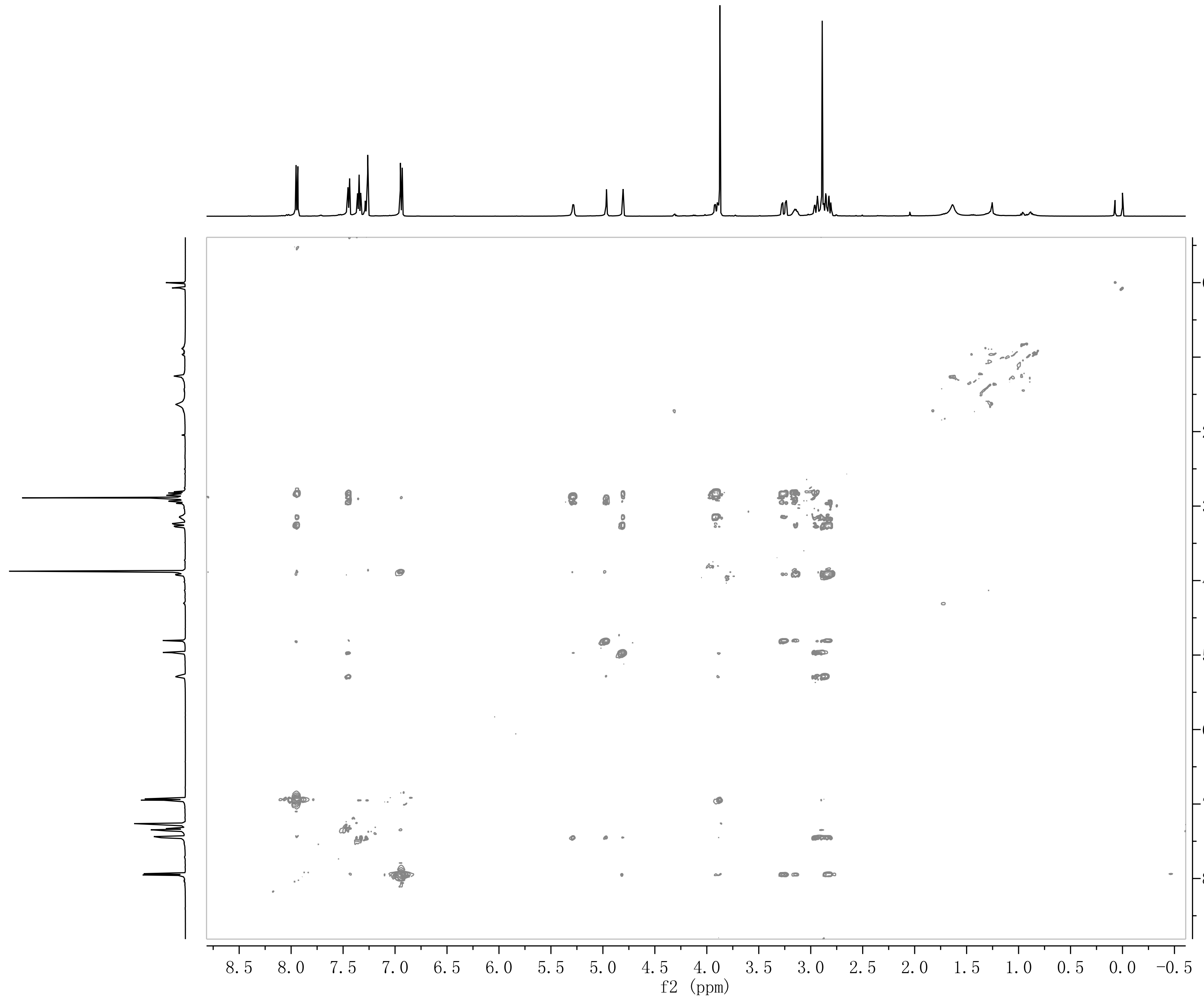

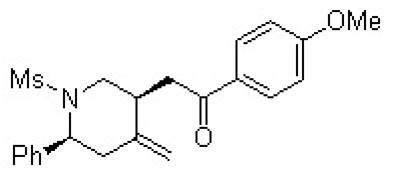

relative configuration

	Parameter	Value
1	Title	
2	Comment	
3	Origin	Bruker BioSpin GmbH
4	Owner	nmr
5	Site	
6	Instrument	spect
7	Author	
8	Solvent	CDCl3
9	Temperature	296.1
10	Pulse Sequence	noesygpphpp
11	Experiment	NOESY
12	Number of Scans	4
13	Receiver Gain	48.5
14	Relaxation Delay	1.9877
15	Pulse Width	11.2900
16	Presaturation Frequency	
17	Acquisition Time	0.2171
18	Class	
19	Spectrometer Frequency	(500.13, 500.13)
20	Spectral Width	(4717.0, 4717.0)
21	Lowest Frequency	(-308.9, -308.9)
22	Nucleus	(1H, 1H)
23	Acquired Size	(1024, 256)
24	Spectral Size	(1024, 1024)

Parameter	Value
Data File Name	E:/ NMR/ 2017/ 2017-6-(15-21)/ xfy-0618-1/ 1/ fid
Comment	
Origin	Bruker BioSpin GmbH
Owner	nmr
Site	
Instrument	spect
Solvent	CDCl3
Temperature	296.2
Pulse Sequence	zg30
Experiment	1D
Number of Scans	10
Receiver Gain	54.3
Relaxation Delay	1.0000
Pulse Width	11.2900
Presaturation Frequency	
Acquisition Time	3.2768
Class	
Spectrometer Frequency	500.13
Spectral Width	10000.0
Lowest Frequency	-1922.1
Nucleus	1H
Acquired Size	32768
Spectral Size	65536

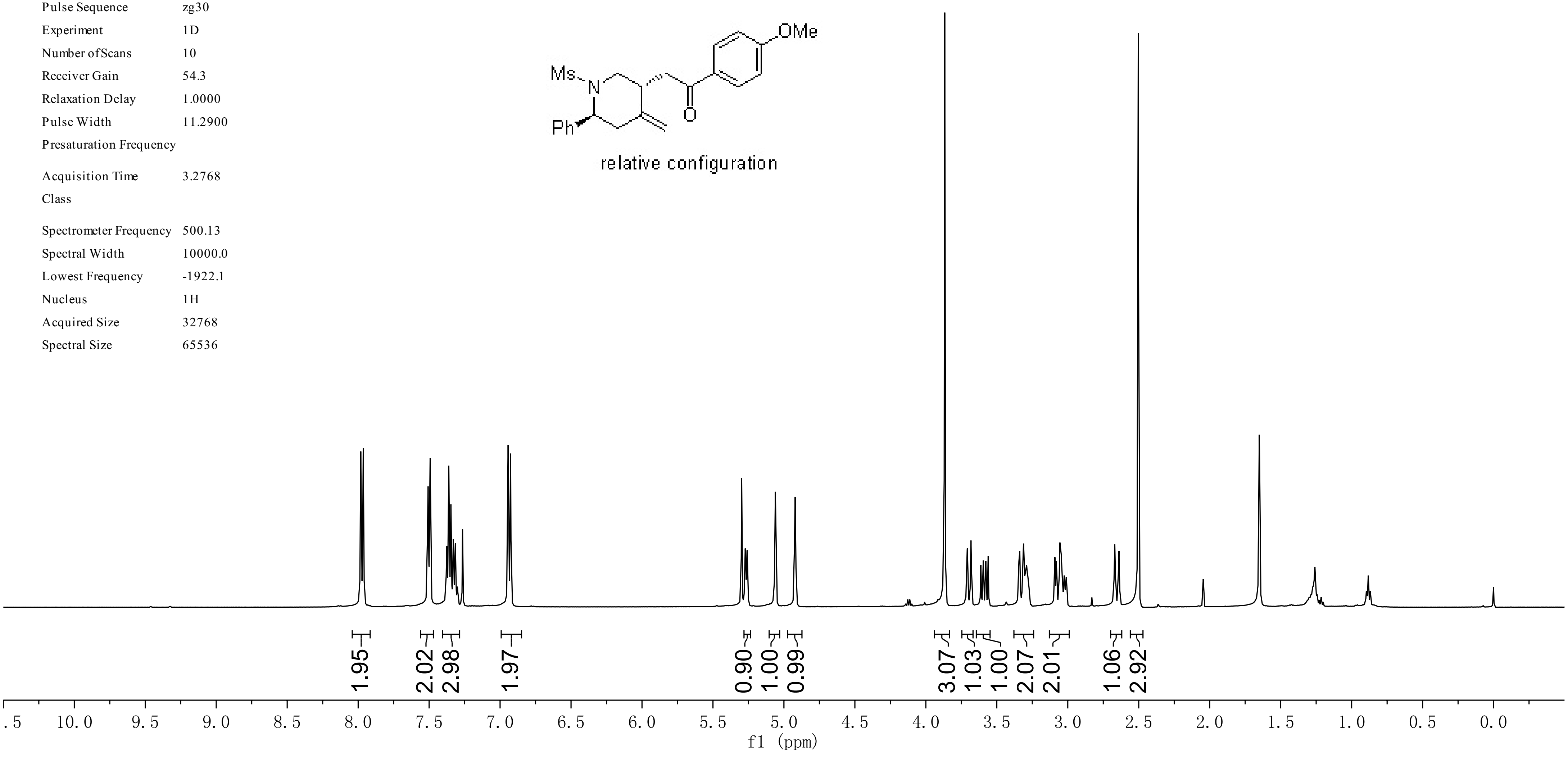

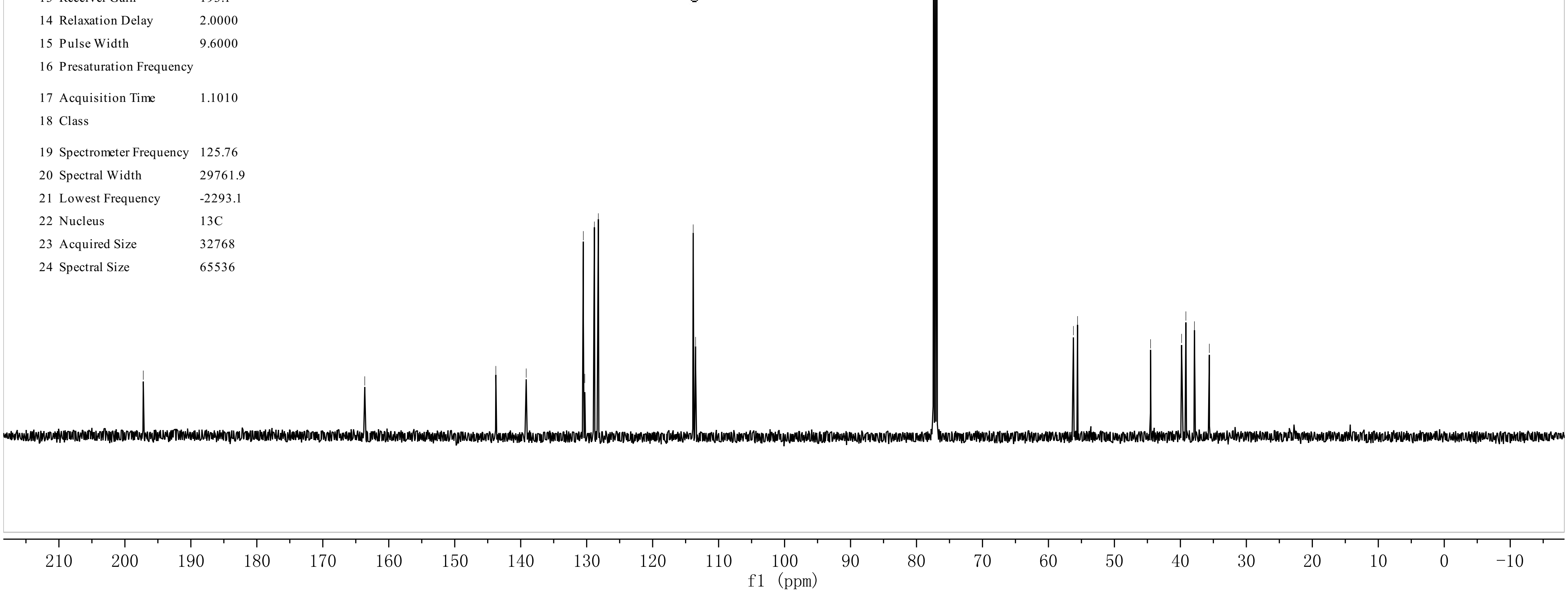

	Parameter	Value
1	Title	
2	Comment	
3	Origin	Bruker BioSpin GmbH
4	Owner	nmr
5	Site	
6	Instrument	spect
7	Author	
8	Solvent	CDCl3
9	Temperature	296.1
10	Pulse Sequence	zgpg30
11	Experiment	1D
12	Number of Scans	20
13	Receiver Gain	193.1
14	Relaxation Delay	2.0000
15	Pulse Width	9.6000
16	Presaturation Frequency	
17	Acquisition Time	1.1010
18	Class	
19	Spectrometer Frequency	125.76
20	Spectral Width	29761.9
21	Lowest Frequency	-2293.1
22	Nucleus	13C
23	Acquired Size	32768
24	Spectral Size	65536

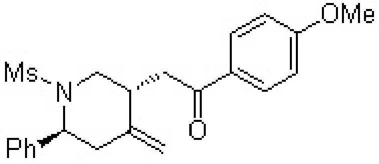

relative configuration

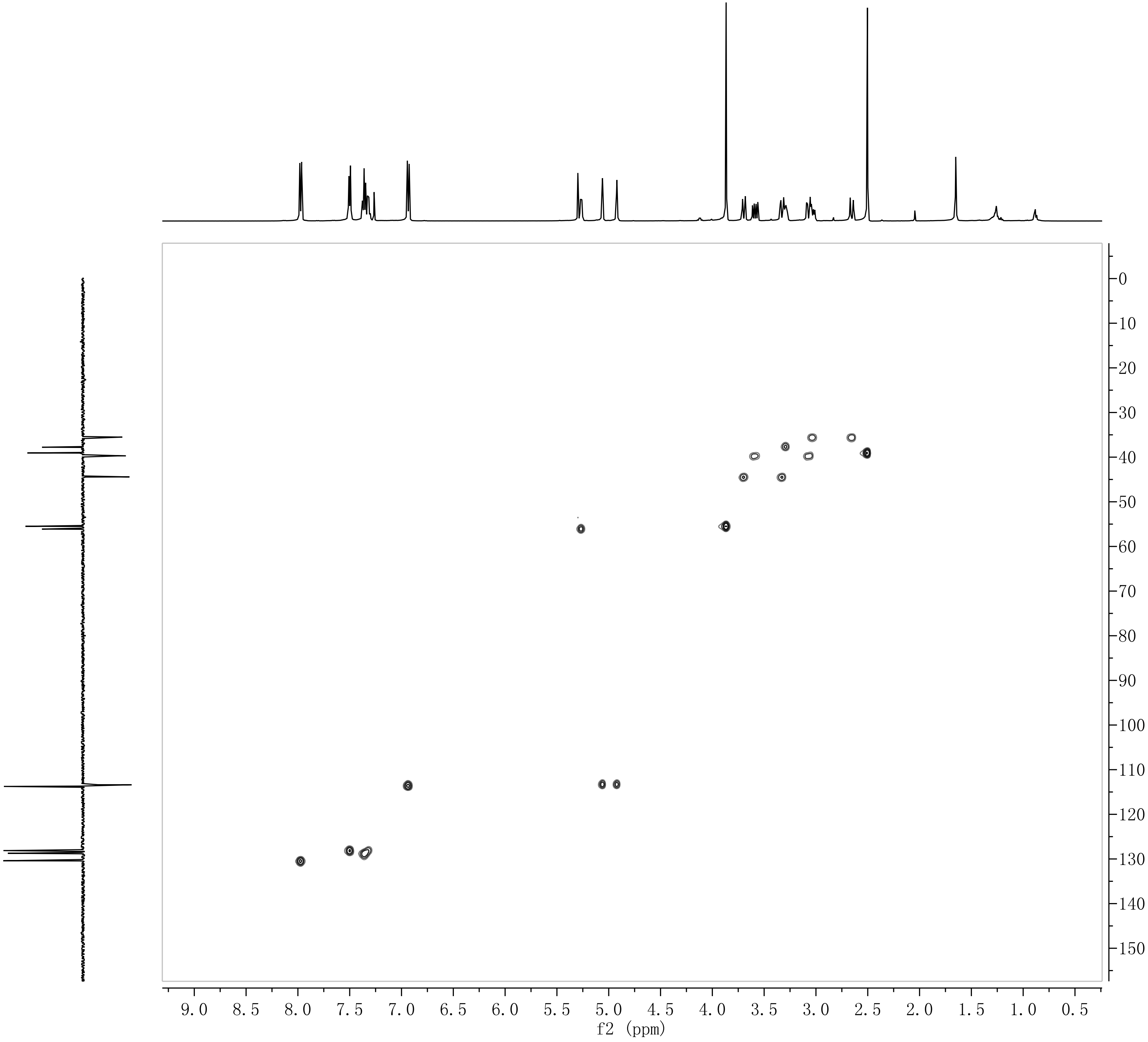

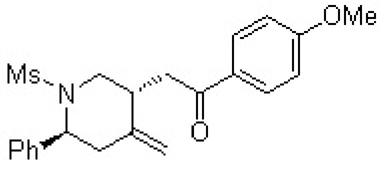

relative configuration

Parameter	Value
Data File Name	E:/ NMR/ 2017/ 2017-6-(15-21)/ xfy-0618-1/ 7/ ser
Comment	
Origin	Bruker BioSpin GmbH
Owner	nmr
Site	
Instrument	spect
Solvent	CDCl3
Temperature	296.1
Pulse Sequence	hsqcetgp
Experiment	HSQC
Number of Scans	2
Receiver Gain	193.1
Relaxation Delay	1.4662
Pulse Width	11.2900
Presaturation Frequency	
Acquisition Time	0.1126
Class	
Spectrometer Frequency	(500.13, 125.77)
Spectral Width	(4545.5, 20833.3)
Lowest Frequency	(110.1, -1037.0)
Nucleus	(1H, 13C)
Acquired Size	(512, 256)
Spectral Size	(512, 512)

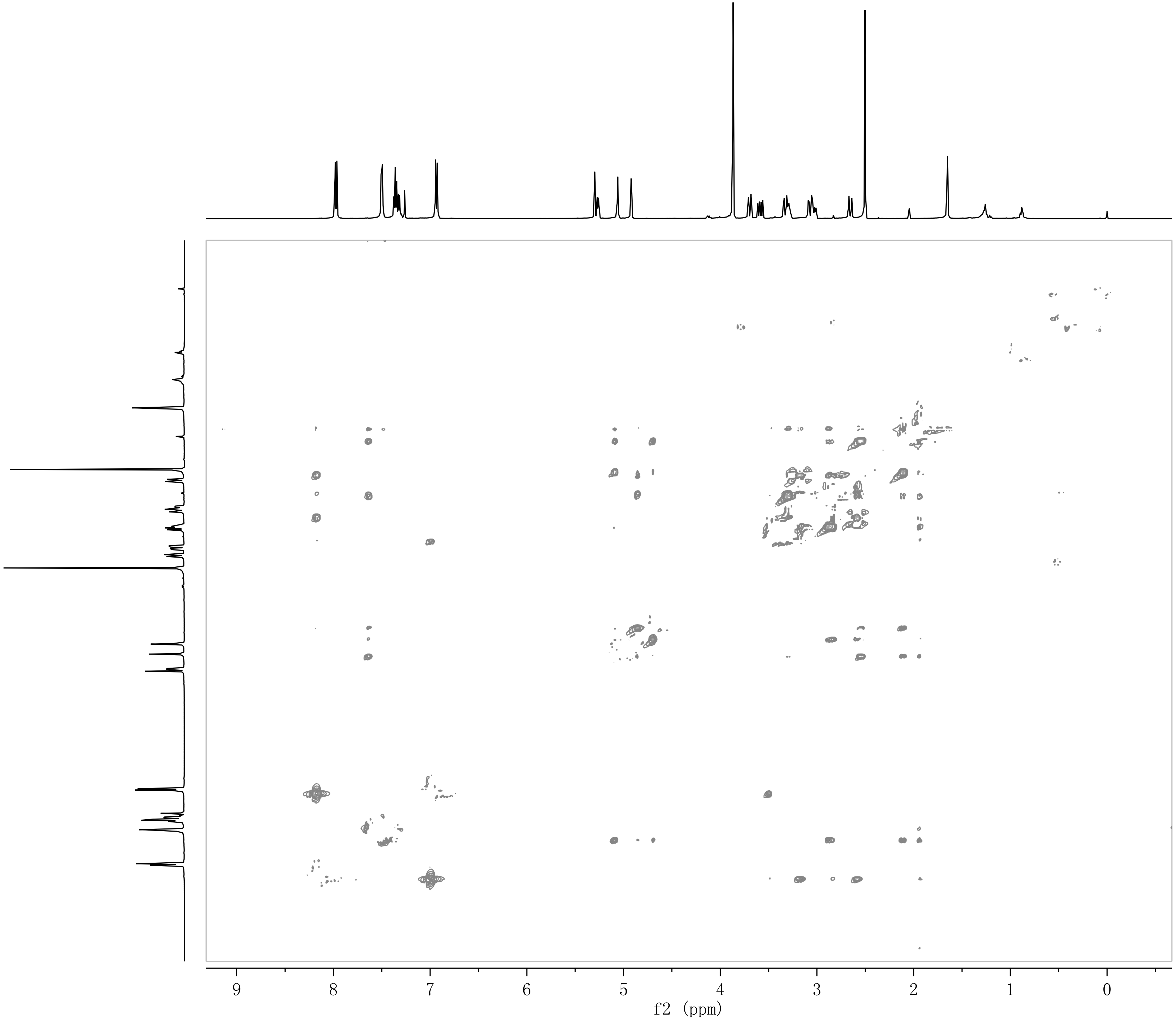

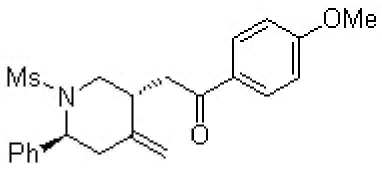

relative configuration

	Parameter	Value
1	Title	
2	Comment	
3	Origin	Bruker BioSpin GmbH
4	Owner	nmr
5	Site	
6	Instrument	spect
7	Author	
8	Solvent	CDCl3
9	Temperature	296.2
10	Pulse Sequence	noesygpphpp
11	Experiment	NOESY
12	Number of Scans	4
13	Receiver Gain	48.5
14	Relaxation Delay	1.9713
15	Pulse Width	11.2900
16	Presaturation Frequency	
17	Acquisition Time	0.2335
18	Class	
19	Spectrometer Frequency	(500.13, 500.13)
20	Spectral Width	(5000.0, 5000.0)
21	Lowest Frequency	(-340.4, -340.4)
22	Nucleus	(1H, 1H)
23	Acquired Size	(1024, 256)
24	Spectral Size	(1024, 1024)

Parameter	Value
Title	xfy-190629-2.2.1.1r
Comment	
Origin	Bruker BioSpin GmbH
Owner	nmr
Site	
Instrument	spect
Solvent	CDCl3
Temperature	297.5
Pulse Sequence	zg30
Experiment	1D
Number of Scans	8
Receiver Gain	87.6
Relaxation Delay	1.0000
Pulse Width	8.7300
Presaturation Frequency	
Acquisition Time	1.9999
Acquisition Date	2019-06-29T19:01:32
Modification Date	2019-06-29T19:25:02
Spectrometer Frequency	400.13
Spectral Width	8012.8
Lowest Frequency	-1535.4
Nucleus	1H
Acquired Size	16025
Spectral Size	65536

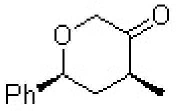

relative configuration

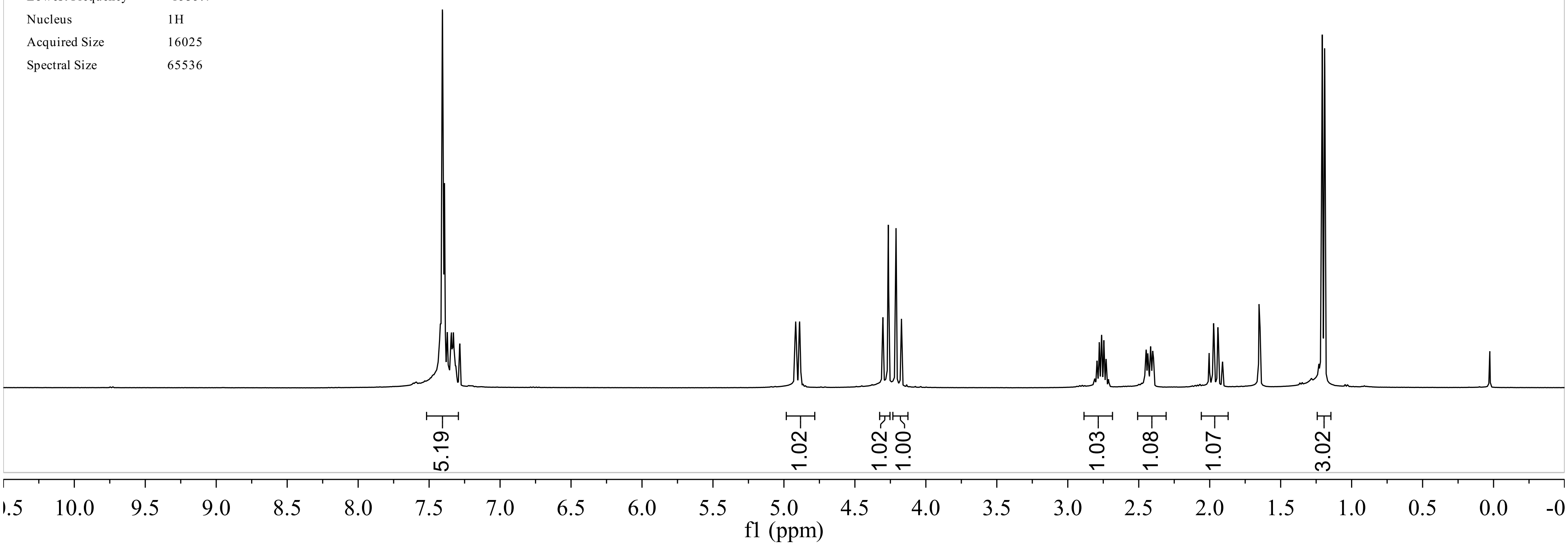

Parameter	Value
Title	xfy-190629-2.3.1.1r
Comment	
Origin	Bruker BioSpin GmbH
Owner	nmr
Site	
Instrument	spect
Solvent	CDCl3
Temperature	297.7
Pulse Sequence	zgpg30
Experiment	1D
Number of Scans	24
Receiver Gain	196.4
Relaxation Delay	2.0000
Pulse Width	10.0000
Presaturation Frequency	
Acquisition Time	1.3631
Acquisition Date	2019-06-29T19:03:32
Modification Date	2019-06-29T19:25:02
Spectrometer Frequency	100.61
Spectral Width	24038.5
Lowest Frequency	-1958.0
Nucleus	13C
Acquired Size	32768
Spectral Size	32768

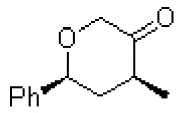

relative configuration

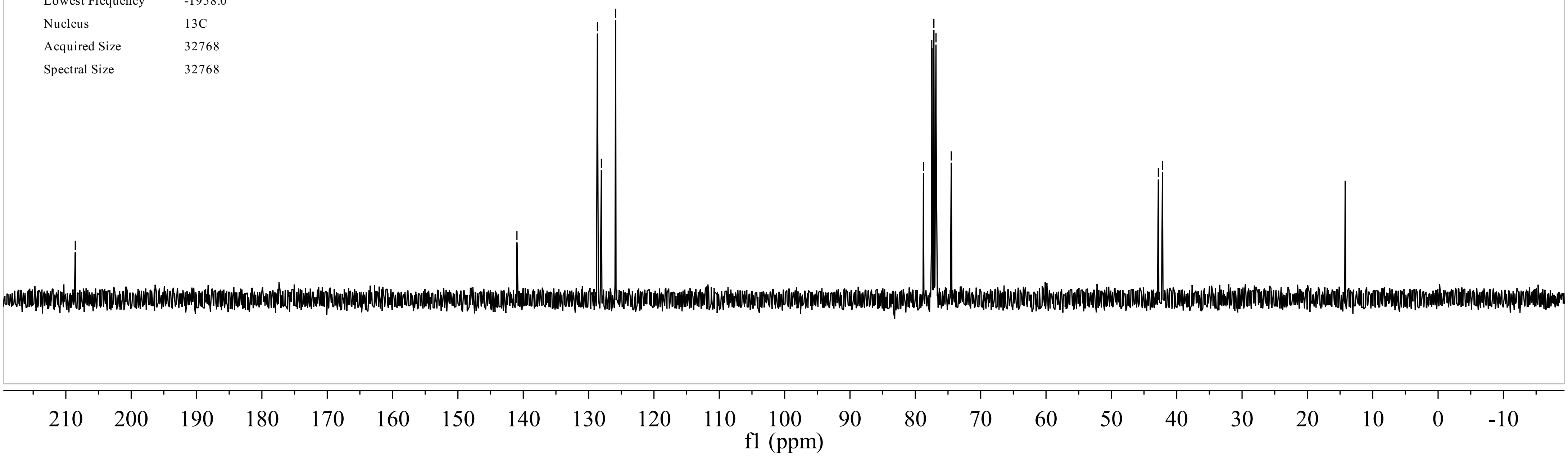

Parameter	Value
标题	xfy-190717-4-s1.1.1.1r
Comment	
Origin	Bruker BioSpin GmbH
Owner	nmr
Site	
Instrument	spect
Author	
Solvent	CDCl3
Temperature	296.2
Pulse Sequence	zg30
Experiment	1D
Number of Scans	6
Receiver Gain	77.6
Relaxation Delay	1.0000
Pulse Width	10.7100
Presaturation Frequency	
Acquisition Time	3.2768
Class	
Spectrometer Frequency	500.13
Spectral Width	10000.0
Lowest Frequency	-1924.3
Nucleus	1H
Acquired Size	32768
Spectral Size	65536

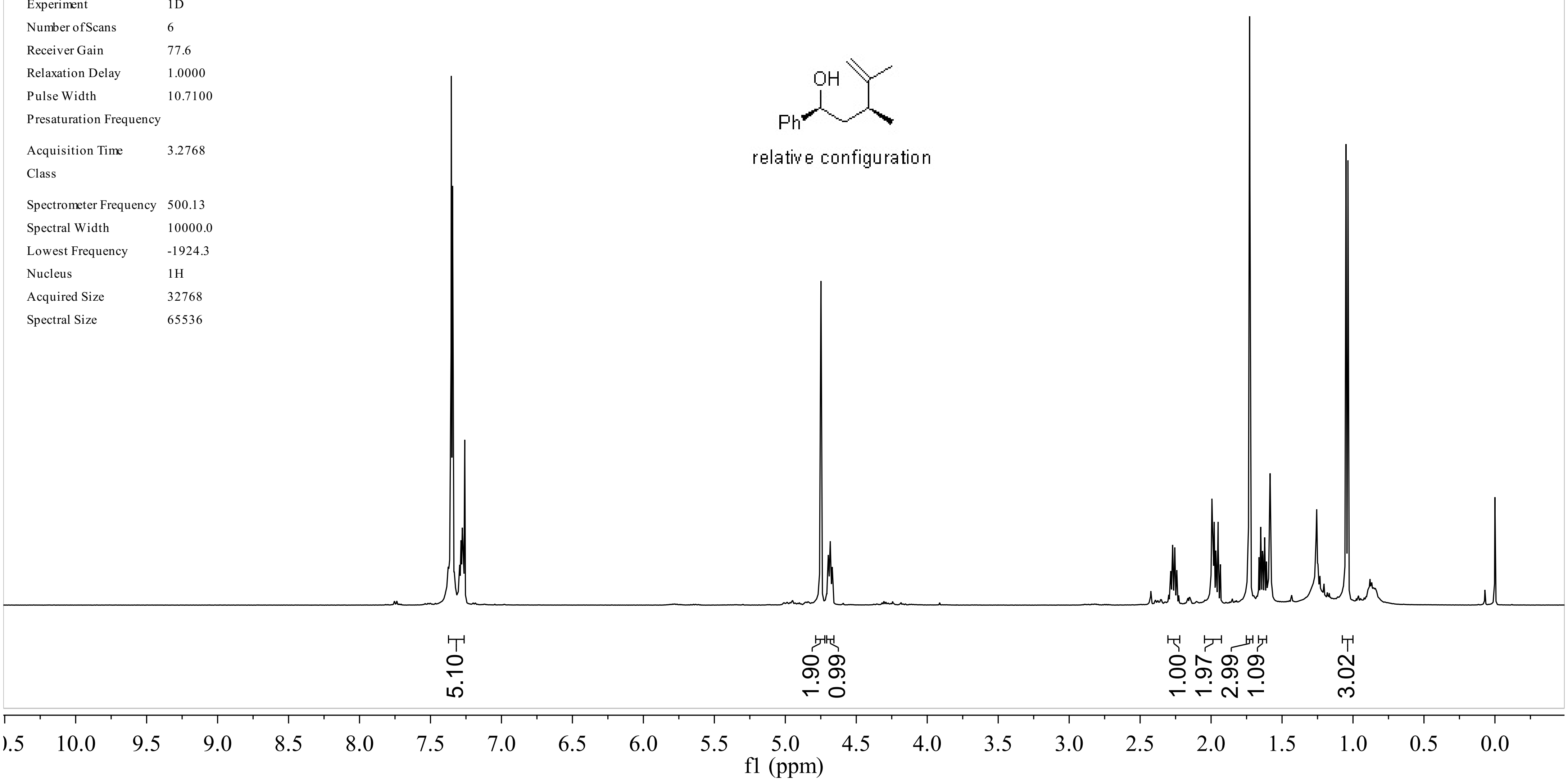

Parameter	Value
标题	xfy-190717-4-s1.2.1.1r
Comment	
Origin	Bruker BioSpin GmbH
Owner	nmr
Site	
Instrument	spect
Author	
Solvent	CDCl3
Temperature	296.2
Pulse Sequence	zgpg30
Experiment	1D
Number of Scans	40
Receiver Gain	193.1
Relaxation Delay	2.0000
Pulse Width	9.6000
Presaturation Frequency	
Acquisition Time	1.1010
Class	
Spectrometer Frequency	125.76
Spectral Width	29761.9
Lowest Frequency	-2290.2
Nucleus	13C
Acquired Size	32768
Spectral Size	32768

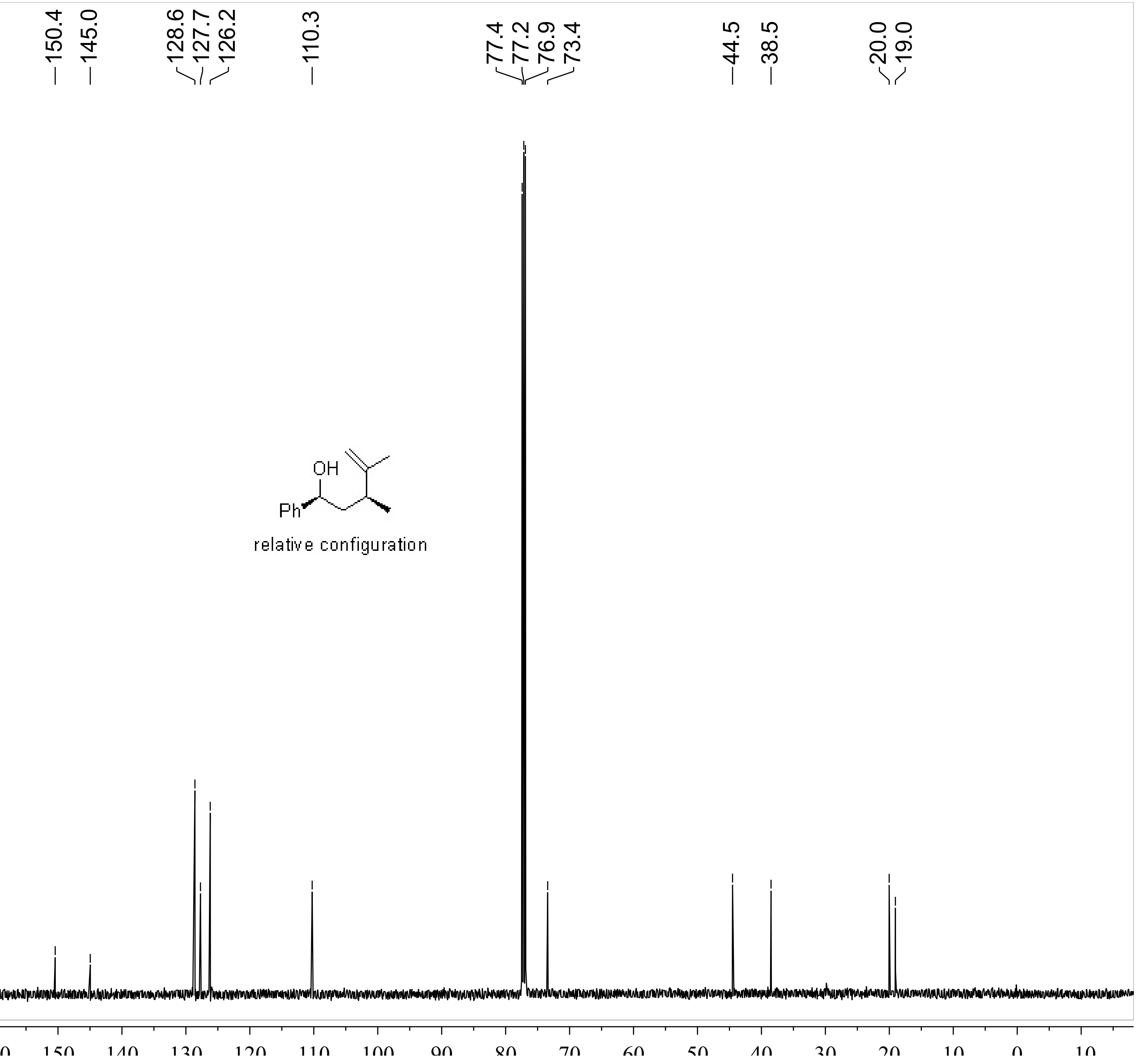

Supplement: Supplementary file 3 — Supplementary Data 1 [file 42004_2020_299_MOESM3_ESM.pdf]
